# Sense-antisense pairs in mammals: functional and evolutionary considerations

**DOI:** 10.1186/gb-2007-8-3-r40

**Published:** 2007-03-19

**Authors:** Pedro AF Galante, Daniel O Vidal, Jorge E de Souza, Anamaria A Camargo, Sandro J de Souza

**Affiliations:** 1 Ludwig Institute for Cancer Research, São Paulo Branch, Hospital Alemão Oswaldo Cruz, Rua João Juliao 245, 1 andar, São Paulo, SP 01323-903, Brazil; 2 Department Of Biochemistry, University of São Paulo, Av. Prof. Lineu Prestes, 748 - sala 351, São Paulo, SP 05508-900, Brazil

## Abstract

**Background:**

A significant number of genes in mammalian genomes are being found to have natural antisense transcripts (NATs). These sense-antisense (S-AS) pairs are believed to be involved in several cellular phenomena.

**Results:**

Here, we generated a catalog of S-AS pairs occurring in the human and mouse genomes by analyzing different sources of expressed sequences available in the public domain plus 122 massively parallel signature sequencing (MPSS) libraries from a variety of human and mouse tissues. Using this dataset of almost 20,000 S-AS pairs in both genomes we investigated, in a computational and experimental way, several putative roles that have been assigned to NATs, including gene expression regulation. Furthermore, these global analyses allowed us to better dissect and propose new roles for NATs. Surprisingly, we found that a significant fraction of NATs are artifacts produced by genomic priming during cDNA library construction.

**Conclusion:**

We propose an evolutionary and functional model in which alternative polyadenylation and retroposition account for the origin of a significant number of functional S-AS pairs in mammalian genomes.

## Abstract 2

Analysis of a catalog of S-AS pairs in the human and mouse genomes revealed several putative roles for natural antisense transcripts and showed that some are artifacts of cDNA library construction.

## Background

Natural antisense RNAs (or natural antisense transcripts (NATs)) are endogenous transcripts with sequence complementarity to other transcripts. There are two types of NATs in eukaryotic genomes: *cis*-encoded antisense NATs, which are transcribed from the opposite strand of the same genomic locus as the sense RNA and have a long (or perfect) overlap with the sense transcripts; and *trans*-encoded antisense NATs, which are transcribed from a different genomic locus of the sense RNA and have a short (or imperfect) overlap with the sense transcripts. *Cis*-NATs are usually related in a one-to-one fashion to the sense transcript, whereas a single *trans*-NAT may target several sense transcripts [1-3]. In this manuscript, we describe analyses in which only cis-NATs were considered. From now on, we refer to these loci as sense-antisense (S-AS) pairs.

When evaluated globally, several features related to the distribution of NATs strongly suggest they have a prominent role in antisense regulation in gene expression [4-7]. For instance, expression of S-AS transcripts tends to be positively or negatively correlated and is more evolutionarily conserved than expected by chance [4,5,7]. Although experimental validation of a putative regulatory role has been achieved for a few models [8-10], it is still unknown whether antisense regulation is a rule or an exception in the human genome. NATs have been implicated in RNA and translational interference [11], genomic imprinting [12], transcriptional interference [13], X-inactivation [14], alternative splicing [10,15] and RNA editing [16]. Moreover, an accumulating body of evidence suggests that NATs might have a pivotal role in a range of human diseases [2].

NATs were initially identified in studies looking at individual genes. However, with the accumulation of whole genome and expressed sequences (mRNA and ESTs) in public databases, a significant number of NATs has been identified using computational analysis [17-22]. These studies showed a widespread occurrence of these transcripts in mammalian genomes. The first evidence that antisense transcription is a common feature of mammalian genomes came from analysis of reverse complementarity between all available mRNA sequences [17]. Subsequent studies, using larger collections of mRNA sequences, ESTs and genomic sequences, confirmed and extended these initial observations [18-22]. More recently, other sources of expression data, such as serial analysis of gene expression (SAGE) tags, were used to expand the catalog of NATs present in mammalian genomes [23,24]. At present, it is estimated that at least 15% and 20% of mouse and human transcripts, respectively, might form S-AS pairs [18,22], although a recent analysis [25] reported that 47% of human transcriptional units are involved in S-AS pairing (24.7% and 22.7% corresponding to S-AS pairs with exon and non-exon overlapping, respectively).

The major obstacle in using expressed sequence data for NAT identification is how to determine the correct orientation of the sequences, especially ESTs. Many ESTs were not directionally cloned and even well-known mRNA sequences were registered from both strands of cloned cDNAs or are incorrectly annotated. As done by others [18,22,23], we here established a set of stringent criteria, including the orientation of splicing sites, the presence of poly-A signal and tail as well as sequence annotation, to determine the correct orientation of each transcript relative to the genomic sequence and made a deep survey of NAT distribution in the human and mouse genomes. Using a set of computational and experimental procedures, we extensively explored expressed sequences and massively parallel signature sequencing (MPSS) data mapped onto the human and mouse genomes. Besides generating a catalog of known and new S-AS pairs, our analyses shed some light on functional and evolutionary aspects of S-AS pairs in mammalian genomes.

## Results and discussion

### Overall distribution of S-AS pairs in human and mouse genomes

To identify transcripts that derive from opposite strands of the same locus, we used a modified version of an in-house knowledgebase previously described for humans [26-28]. This knowledgebase contains more than 6 million expressed sequences mapped onto the human genome sequence and clustered in approximately 111,000 groups. Furthermore, SAGE [29] and MPSS [30] tags were also annotated with all associated information, such as tag frequency, library source and tag-to-gene-assignment (using a strategy developed by us for SAGE Genie [31]). An equivalent knowledgebase was built for the mouse genome (for more details see Materials and methods).

We first designed software that searched the human and mouse genomes extracting gene information from transcripts mapped onto opposite strands of the same locus. Several parameters were used by the software to identify S-AS pairs, such as: sequence orientation given by the respective GenBank entry; presence and orientation of splice site consensus; and presence of a poly-A tail (for more details see Materials and methods). We found 3,113 and 2,599 S-AS pairs in human and mouse genomes, respectively, containing at least one full-insert cDNA (sequences annotated as 'mRNA' in GenBank and referred to here as such) in each orientation (Table 1). Furthermore, we also made use of EST data from both species. A critical issue when using ESTs is the orientation of the sequence, a feature not always available in the respective GenBank entries. We overcame this problem by simply using those ESTs that had a poly-A tail or spanned an intron and, therefore, disclosed their strand of origin by the orientation of a splicing consensus sequence (GT...AG rule). We found 6,964 and 5,492 additional S-AS pairs when EST data were incorporated into the analysis, totaling 10,077 and 8,091 pairs for human and mouse genomes, respectively (Table 1). All of these pairs contained at least one mRNA since we did not analyze EST/EST pairs. It is important to note that we haven't considered in the present analysis non-polyadenylated transcripts and *trans*-NATs. Thus, the total number of NATs is likely to be even higher in both genomes. Data presented in Table 1 are split in cases where a single S-AS pair is present in a given locus (single bidirectional transcription) and in cases where more than one pair is present per locus (multiple bidirectional transcription). Additional data file 1 lists two representative GenBank entries for all S-AS pairs split by chromosome mapping in the two species. As previously observed [17], S-AS pairs are under-represented in the sex chromosomes of both species (Additional data file 2).

**Table 1:** Overall distribution of S-AS pairs in the human and mouse genomes

cDNA type	Single bidirectional transcription		Multiple bidirectional transcription	
		
	Human	Mouse	Human	Mouse
mRNA-mRNA	2,109	1,879	1,004	720
mRNAs-ESTs	3,299	3,265	3,665	2,227
Total	5,408	5,144	4,669	2,947

Single bidirectional transcription corresponds to those loci in which only one S-AS pair is present. Multiple bidirectional transcription corresponds to those loci in which more than one S-AS pairs is present (at least one gene belongs to more than one S-AS pair).

The above numbers confirm that S-AS pairs are much more frequent in mammalian genomes than originally estimated [4,17,18]. Our analyses suggest that at least 21,000 human and 16,000 mouse genes are involved in S-AS pairing. These numbers are more in agreement with those from [32] in their analysis using tiling microarrays to evaluate gene expression of a fraction of the human genome. For the mouse genome, our numbers are in agreement with those reported by Katayama *et al*. [8]. A more recent analysis [25] also gives a similar estimate of S-AS pairs in both human and mouse genomes.

Could this high number of S-AS pairs be due to the stringency of our clustering strategy? If the same transcriptional unit is fragmented in close contigs due to 3' untranslated region (UTR) heterogeneity, the total number of clusters would be inflated, leading to an erroneous count of S-AS pairs. To evaluate this possibility, we relaxed our clustering parameters, requiring a minimum of 1 base-pair (bp) same strand overlap for clustering. Furthermore, we collapsed into a single cluster all pairs of clusters located in the same strand and less than 30 bp away from each other. Additional data file 3 shows the total number of clusters and S-AS pairs after this new clustering strategy was employed. As expected, both the total number of clusters and S-AS pairs decreased with the new clustering methodology. The total number of clusters decreased by 2% and 1% for human and mouse, respectively, while the total number of S-AS pairs decreased by 0.3% for both human and mouse. Thus, the small difference observed does not affect the conclusions on the genomic organization of S-AS pairs. For all further analyses, we decided to use the original dataset obtained with a more stringent clustering methodology.

We further explored the genomic organization of S-AS pairs using the subset of 3,113 human and 2,599 mouse pairs that contained mRNAs in both sense and antisense orientations. The genomic organization of S-AS pairs can be further divided into three subtypes based on their overlapping patterns: head-head (5'5'), tail-tail (3'3') or embedded (one gene contained entirely within the other) pairs (Table 2). For a schematic view of the genomic organization of S-AS pairs, see Additional data file 4. Embedded pairs are more frequent in both species, corresponding to 47.8% and 42.5% of all pairs in human and mouse, respectively. If we take into account the intron/exon organization of both genes, we observe that the most frequent overlap involves at least one exon-intron border. In spite of this, a significant amount of NATs maps completely within introns from the sense gene in both human and mouse (category 'Fully intronic' in Table 2). Interestingly, more than three-quarters of all S-AS pairs categorized as 'Fully intronic' fall within the embedded category for human and mouse. How unique is this distribution? Monte Carlo simulations, in which we randomly replaced NATs in relation to sense genes while keeping their 5'5'/embedded/3'3' orientation, show that the distribution of S-AS pairs is quite unique. All three categories of S-AS pairs deviate from a random distribution (chi-square = 11.5, df (degrees of freedom) = 2, *p *= 0.003 for embedded pairs; chi-square = 49, df = 2, *p *= 2.3 × 10^-11 ^for 5'5' pairs; chi-square = 132, df = 2, *p *= 2.1 × 10^-29 ^for 3'3' pairs). This peculiar distribution will be further discussed in the light of the expression analyses. Since these intronic NATs have been shown to be over-expressed in prostate tumors [33], our dataset should be further explored regarding differential expression in cancer. Due to their genomic distribution, any putative regulatory role of these intronic NATs would have to be restricted to the nucleus. Interestingly, Kiyosawa *et al*. [34] observed that a significant amount of NATs in mouse is poly-A negative and nuclear localized.

**Table 2:** Distribution of NATs in relation to the genomic structure of the sense transcript

	Human			Mouse		
		
	5'5'	Embedded	3'3'	5'5'	Embedded	3'3'
Fully exonic	112 (20%)	32 (3%)	213 (40%)	156 (27%)	14 (2%)	227 (45%)
Exonic/intronic	362 (64%)	372 (37%)	259 (48%)	360 (62%)	338 (42%)	242 (48%)
Fully intronic	92 (16%)	606 (60%)	61 (12%)	61 (11%)	448 (56%)	33 (7%)
Total	566	1,010	533	577	800	502

5'5', head-head orientation; 3'3', tail-tail orientation.

Another interesting observation is the higher frequency of intronless genes within the set of S-AS pairs (Table 3). About half (47%) of all mRNA/mRNA S-AS pairs in humans contains at least one intronless gene. This number is slightly lower for mouse (44%) (Table 3). Interestingly, intronless genes are significantly enriched within the set of embedded pairs (chi-square = 95.9, *p *< 1.2 × 10^-22 ^for human and chi-square = 3.98 and *p *< 0.045 for mouse). For humans, 66% of all S-AS pairs containing at least one intronless gene are within the 'embedded' category; Sun *et al*. [5] found 43.4% of their S-AS pairs as 'embedded'. Furthermore, they found 35% of 3'3' pairs while we found only 25%. These differences are probably due to the fact that Sun *et al*. [5] included in their analyses pairs containing only ESTs.

**Table 3:** Classification of S-AS pairs in reference to their orientation and the presence of introns at the genome level for both genes in a pair

NAT pair	Human			Mouse		
		
	5'5'	Embedded	3'3'	5'5'	Embedded	3'3'
Both with intron	342 (61%)	351 (35%)	417 (78%)	259 (45%)	394 (49%)	390 (78%)
Intron-intronless	206 (36%)	645 (64%)	103 (19%)	285 (49%)	398 (50%)	96 (19%)
Both intronless	18 (3%)	14 (1%)	13 (3%)	33 (6%)	8 (1%)	16 (3%)
Total	566	1,010	533	577	800	502

5'5', head-head orientation; 3'3', tail-tail orientation.

All these results clearly show that subsets of S-AS pairs have distinct genomic organization, suggesting that they may play different biological roles in mammalian genomes. Below we will discuss these data in a functional/evolutionary context.

### Conservation of S-AS pairs between human and mouse

Using our set of human and mouse S-AS pairs, we measured the degree of conservation between S-AS pairs from human and mouse. Since the numbers reported so far are discrepant, ranging from a few hundred [5,6] to almost a thousand [25], we decided to use different strategies. We first used a strategy based on HomoloGene [35]. The number of S-AS pairs with both genes mapped to HomoloGene is 854 for human and 579 for mouse. Among these, 190 S-AS pairs are conserved between human and mouse. One problem with this type of analysis lies in its dependence on HomoloGene, which, for example, does not take into consideration genes that do not code for proteins. Therefore, we decided to implement a different strategy, in which we identified those pairs that had at least one conserved gene mapped by HomoloGene and tested each known gene's NAT for sequence level conservation. Using this strategy, we found an additional 546 cases, giving a total of 736 (190 + 546) conserved S-AS pairs between human and mouse. Finally, we also applied to our dataset the same strategy used by Engstrom *et al*. [25], in which they counted the number of human and mouse S-AS pairs that had exon overlap in corresponding positions in a BLASTZ alignment of the two genomes. We applied the same strategy to our dataset and found 1,136 and 1,144 corresponding S-AS pairs in human and mouse, respectively. As observed by Engstrom *et al*. [25] the numbers from human and mouse slightly differ because a small proportion of mouse pairs corresponded to several human pairs and *vice versa*. Additional data file 5 lists all S-AS pairs found by the three methodologies discussed above.

There is a predominance of 3'3' pairs in all sets of conserved S-AS pairs. For the first strategy solely based on HomoloGene, 67% of all pairs are 3'3' compared to 19% embedded and 14% 5'5'. For the dataset obtained using the strategy from Engstrom *et al*. [25], there is also a prevalence of 3'3'pairs (48%) compared to embedded (14%) and 5'5 (38%) pairs. We have also modified the method of Engstrom *et al*. [25] to take into account all S-AS pairs and not only those presenting exon-exon overlap. These data are shown in Additional Data File 6. We observed that S-AS pairs whose overlap is classified as 'Fully intronic' are less represented in the set of conserved S-AS pairs (18% in this set compared to 29% in the whole dataset of S-AS pairs). The same is true for S-AS pairs containing at least one intronless gene (26% in the set of conserved S-AS pairs compared to 47% in the whole dataset). These last results are in accordance with our previous observation that conserved S-AS pairs are enriched with 3'3' pairs. As seen in Tables 2 and 3, 3'3' pairs are poorly represented in the categories 'Fully intronic' (Table 2) and 'Intron/intronless' (Table 3).

### Discovery of new S-AS pairs in human and mouse genomes using MPSS data

Large-scale expression profiling tools have been used to discover and analyze the co-expression of S-AS pairs [5,23,34]. Quéré *et al*. [23], for instance, recently explored the SAGE repositories to detect NATs. These authors searched for tags mapped on the reverse complement of known transcripts and analyzed their expression pattern on different SAGE libraries. However, no attempt was made to experimentally validate the existence of such NATs. Here, we made use of MPSS data available in public repositories [36,37] to search for new NATs in both human and mouse genomes. Since MPSS tags are longer than conventional SAGE tags, we can use the genome sequence for tag mapping. Furthermore, MPSS offers a much deeper coverage of the transcriptome since at least a million tags are generated from each sample.

We made use of 122 MPSS libraries derived from a variety of human and mouse tissues (81 libraries for mouse, 41 for human; see the list in Additional data file 7). Our strategy was based on the generation of virtual tags from each genome by simply searching the respective genome sequence for *Dpn*II sites. Since these sites are palindromes, we extract, for each one, two virtual tags (13 and 16 nucleotide long tags for human and mouse, respectively), both immediately downstream of the restriction site but in opposite orientations (see Materials and methods for more details). In this way, we could evaluate the expression of transcriptional units present in both strands of DNA. We obtained 5,580,158 and 8,645,994 virtual tags for the human and mouse genomes, respectively. This set of virtual tags was then compared to a list of tags observed in the MPSS libraries. As true for any study using mapped tags, our analysis misses those cases in which a tag maps exactly at an exon/exon border at the cDNA level.

We first evaluated the number of cDNA-based S-AS pairs (shown in Table 1) that were further confirmed by the presence of an MPSS tag. Data for this analysis are presented as Additional data file 8. Roughly, 84% and 51% of all cDNA-based S-AS pairs were confirmed by MPSS data for human and mouse, respectively.

Since we were interested in finding new antisense transcripts, we searched for tags found in the MPSS libraries that were mapped on the opposite strand of both introns and exons of known genes. For this analysis we excluded those genes that were already part of S-AS pairs as described above. For humans, 4,308 genes have at least one MPSS tag derived from the antisense strand (Table 4). For 1,221 human genes there were two or more distinct MPSS tags in the antisense orientation. Another interesting observation is the larger number of MPSS tags antisense to exonic regions of the sense genes. Unexpectedly, we found a much smaller number of antisense tags for mouse (Table 4). Although the number of mouse libraries is larger (81 mouse and 41 human libraries), the number of unique tags is significantly smaller (56,061 for mouse and 340,820 for human). The assignment of these unique tags to known genes shows a smaller representation of known genes in the mouse dataset (51% against 66% for human). It is unlikely, however, that these differences can explain the dramatic difference shown in Table 4. Further analyses are needed to solve this apparent discrepancy.

**Table 4:** Distribution of MPSS tags in an antisense orientation in human and mouse genomes

	Number of clusters	
	
	Human	Mouse
One exonic tag	2,212 (51.3%)	124 (57.3%)
One intronic tag	875 (20.3%)	90 (41.7%)
Exonic and intronic tag	707 (16.4%)	2 (1%)
Multiple exonic tags	318 (7.4%)	0
Multiple intronic tags	196 (4.6%)	0
Total	4,308	216

Exonic and intronic refer to the genome organization of the sense gene. For instance, the category 'One exonic tag' corresponds to those genes with only one antisense tag complementary to its exonic region. All identified tags are found at a frequency ≥3 tags per million (see Materials and methods).

**Figure 1:**
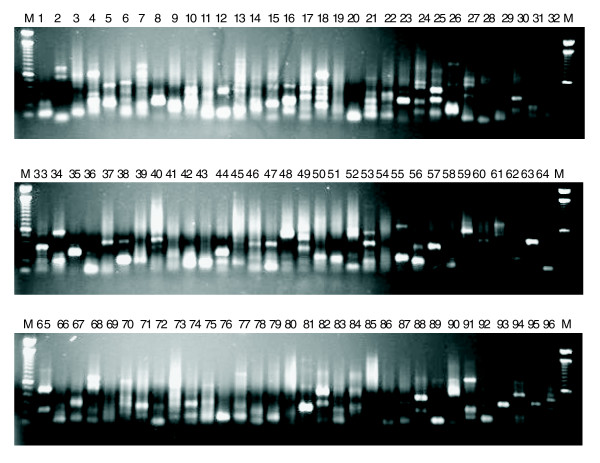
GLGI-MPSS amplification. GLGI amplifications for 96 MPSS antisense tags were analyzed on agarose gels stained with ethidium bromide. Note that some lanes show only a single amplified band whereas others have more than one band and sometimes a smear. A 100 bp ladder (M) was used as molecular weight marker.

To experimentally validate the existence of these novel human NAT candidates we used the GLGI (Generation of Longer cDNA fragments from SAGE for Gene Identification)-MPSS technique [38] to convert 96 antisense MPSS tags into their corresponding 3' cDNA fragments. A sense primer corresponding to the antisense MPSS tag was used for GLGI-MPSS amplification as described in Materials and methods. A predominant band was obtained for most of the GLGI-MPSS reactions (Figure 1). Amplified fragments were purified, cloned, sequenced and aligned to the human genome sequence. We were able to generate a specific 3' cDNA fragment for 46 (50.5%) out of 91 novel antisense candidates. Of these 46, the poly-A tail of 19 aligned with stretches of As in the human genome sequence (this finding will be discussed further). The existence of three of these antisense transcripts, out of three that were tested, was further confirmed by orientation-specific RT-PCR (data not shown).

Among the 49.5% (91 - 46 = 45) of candidates that were not considered to be validated, we found 25 that were amplified in the GLGI-MPSS experiment but whose exon-intron organization was identical to the sense gene. Although antisense sequences like these have already been observed [39], we did not consider them as validated antisense transcripts. Orientation-specific RT-PCR confirmed the existence of one transcript, out of two that were tested.

### Alternative polyadenylation as a major factor in defining S-AS pairs

Dahary *et al*. [6] observed that S-AS overlap usually involves transcripts generated by alternative polyadenylation. This observation had already been reported by us and others [40]. We decided to test if these preliminary observations would survive a more quantitative analysis. We found that the S-AS overlap is predominantly due to alternative polyadenylation variants. Roughly, 51% of all S-AS pairs (274 out of 533 3'3' pairs) overlap due to the existence of at least one variant. This number is certainly underestimated since many variants are still not represented in the sequence databases. The above observation raises the exciting possibility that antisense regulation is associated with the regulation of alternative polyadenylation. It is expected that the presence of overlapping genes imposes constraints on their evolution since any mutation will be evaluated by natural selection according to its effect in both genes. Thus, in principle, overlapping genes should impose a negative effect on the fitness of a subject. Alternative polyadenylation has the potential to relax such negative selection since the overlapping is dependent on a post-transcriptional modification.

If alternative polyadenylation is a significant factor in defining S-AS pairs, we would expect a lower rate of alternative polyadenylation in chromosome X, which has the smallest density of S-AS pairs. Indeed, only 20% of all messages from the X chromosome show at least two polyadenylation variants, compared to 27.5%, on average, for the autosomes (chi-square = 34.91, df = 1, *p *< 0.0001).

### A fraction of S-AS pairs is generated through internal priming and retroposition events

During the validation of new NATs identified using the MPSS data, we noticed that a significant fraction of GLGI amplicons (19 out of 46 validated fragments) had their 3' ends aligning to stretches of As in the human genome. This motivated us to search for similar cases in the set of cDNA-based S-AS pairs identified in this study. We found that 18% and 26% of all S-AS pairs have at least one gene with its 3' end aligning with a stretch of A's in the human and mouse genomes, respectively. This number is certainly inflated by ESTs since it decreases to 11.7% for human and 12.6% for mouse when only mRNA/mRNA S-AS pairs are considered. Two possibilities could account for this observation. First, a fraction of all antisense transcripts would be artifacts due to genomic priming with contaminant genomic DNA during cDNA library construction. An alternative is the possibility that antisense genes were constructed during evolution by retroposition events. Both possibilities are in agreement with the observation that antisense genes are depleted of introns.

**Figure 2:**
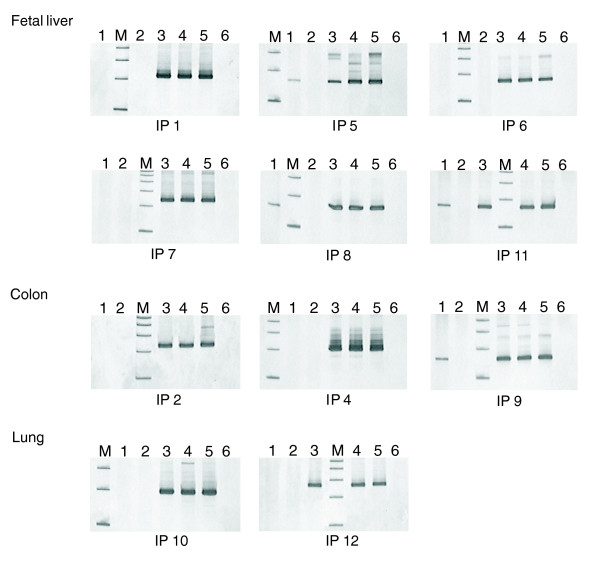
RT-PCR analysis for the internal priming (IP) candidates in fetal liver, colon and lung total RNA. RT-PCR was conducted in DNA-free RNA previously treated with *DNAse *(lanes 1 and 2) and in untreated RNA, which was, therefore, contaminated with genomic DNA (gDNA; lanes 3 and 4) for each candidate in the corresponding tissue. As a control, RT-PCR was conducted in the presence (lanes 1 and 3) and absence (lanes 2 and 4) of reverse transcriptase. gDNA was used as a positive control of the PCR reaction (lane 5) and no template as a negative control (lane 6). For fetal liver, in 3 IP candidates (5, 8 and 11) the PCR products (152 bp, 153 bp and 160 bp, respectively) were observed in the treated RNA when RT was added (lane 1) or in untreated RNA independent of the RT (lanes 3 and 4). For colon, in 1 IP candidate (9) the PCR product (158 bp) was observed in the treated RNA when RT was added (lane 1) or in untreated RNA independent of the RT (lanes 3 and 4). For the remaining IP candidates (1, 2, 4, 6, 7, 10 and 12), the PCR products (214 bp, 229 bp, 207 bp, 156 bp, 227 bp, 205 bp and 234 bp, respectively) were observed only in untreated RNA independent of the RT (lanes 3 and 4). The PCR products were analyzed on 8% polyacrylamide gels with silver staining. A 100 bp ladder (M) was used as molecular weight marker. In each gel the lower fragment in lane M correspond to 100 bp.

An experimental strategy was developed to evaluate the likelihood of genomic priming as a factor generating artifactual antisense cDNAs. A total of 11 mRNA candidates derived from cDNA libraries from fetal liver, colon and lung with a high proportion of sequences that had their 3' ends aligning to stretches of As in the human genome were selected for experimental validation by RT-PCR. cDNA samples used in these experiments were reverse transcribed from fetal liver, colon and lung total RNA treated or not with DNAse. As can be seen in Figure 2, specific amplifications could not be achieved for 7 (63.6%) out of the 11 selected candidates when cDNA samples used as templates for PCR amplification were prepared from DNA-free RNA. On the other hand, when untreated RNA was used for cDNA synthesis, all candidates could be amplified, suggesting that a significant proportion of these internal priming sequences were indeed generated from contaminant genomic DNA.

Some other features support the artifactual origin of these antisense transcripts. First, cDNAs containing a stretch of As at their 3' genomic end have much less polyadenylation signals than genes in general (17% compared to 85%). Furthermore, these genes have a much narrower and rarer expression pattern when analyzed by SAGE and MPSS than genes in general (data not shown). These observations suggest that a significant fraction of all antisense genes are actually artifacts, due to genomic priming during library construction.

Retroposition generates intronless copies of existing genes through reverse transcription of mature mRNAs followed by integration of the resulting cDNA into the genome (for a review, see Long *et al*. [41]). Eventually, the cDNA copy can be involved in homologous recombination with the original source gene as has been suggested for yeast [42]. Retroposition was thought to generate non-functional copies of functional genes. However, several groups have shown that retroposition has generated a significant amount of new functional genes in several species [43-45]. Recently, Marques *et al*. [43] found almost 4,000 retrocopies of functional genes in the human genome. More recently, the same group reported that more than 1,000 of these retrocopies are transcribed, of which at least 120 have evolved as *bona fide *genes [46].

Retrocopies usually have a poly-A tail at their 3' end because of the insertion of this post-transcriptional modification together with the remaining cDNA. Thus, retroposition can explain the high incidence of antisense transcripts with a poly-A tail at their 3' end. To evaluate the contribution of retrocopies to the formation of S-AS pairs we compared the loci identified by Marques *et al*. [43] as retrocopies with the list of S-AS pairs identified in this study. Out of 413 retrocopies represented in the cDNA databases, 138 were involved in S-AS pairs (70 mRNA/mRNA and 68 mRNA/EST pairs). For the 70 mRNA/mRNA pairs, 78% were classified as embedded. This is in agreement with our previous observation that embedded pairs are enriched with intronless genes. Thus, retroposition seems to significantly contribute to the origin of embedded S-AS pairs.

### Expression patterns within S-AS pairs

A critical issue to effectively evaluate the role of antisense transcripts in regulating distinct cellular phenomena is related to the expression pattern of both sense and antisense transcripts belonging to the same S-AS pair. Several reports have been published based on large-scale gene-expression analyses [5,19,23,47,48]. Similar to Wang *et al*. [48], we here used MPSS libraries available for human to explore this issue. Tag to gene assignment was performed as previously described [31,49]. To ensure the MPSS sequences were unambiguously matched to the assigned transcript, we removed tags mapped to more than one locus. Frequencies for all tags assigned to genes in an S-AS pair were collected from all MPSS libraries.

**Figure 3:**
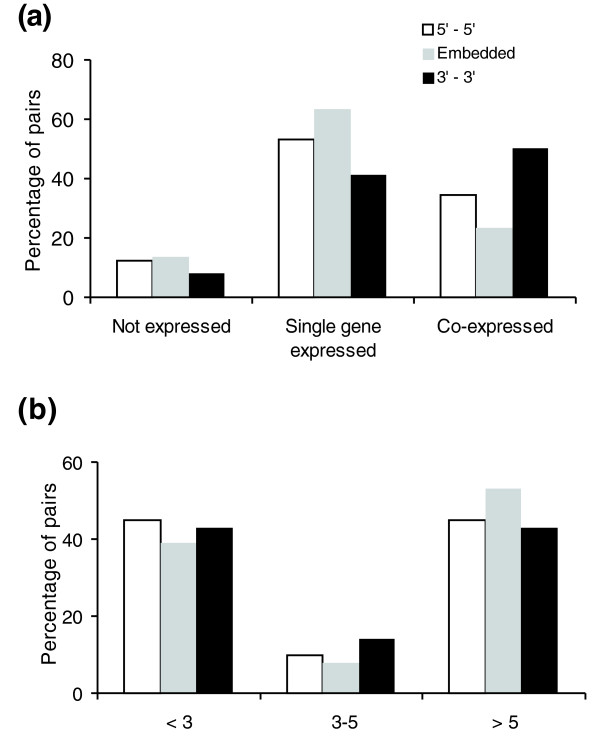
Expression pattern (in a set of 31 tissues covered by MPSS) of genes belonging to all three types of S-AS pairs (3'3', 5'5'and embedded). **(a) **Categories are as follows: 'no expression', for S-AS pairs whose expression was not detected (see Materials and methods for details); 'single-gene expression', for S-AS pairs in which expression is observed for only one gene in the pair; 'co-expression', for pairs in which expression is seen for both genes in the pair. **(b) **Rate of differential expression for the set of co-expressed S-AS pairs. Ratio of sense/antisense genes in the pair is shown on the x-axis.

Figure 3 shows the expression pattern of S-AS pairs for all MPSS libraries for human. We divided the dataset into the following categories as before: 3'3', 5'5' or embedded. Several features are evident. The rate of co-expression in our dataset was 35.1% compared to 44.9% observed by Chen *et al*. [4]. The differences are probably due to experiment design in both reports (for example, differences in the dataset and in the way the rate was calculated). Second, the rate of co-expression is significantly higher for 3'3' pairs when compared to the frequency of the embedded pairs (50.3%, chi-square = 134, df = 1, *p *= 5.4 × 10^-31^). This supports a previous conclusion from Sun *et al*. [5] that 3'3' S-AS pairs are significantly more co-expressed than other pairs and, therefore, are more prone to be involved in antisense regulation. It is important to mention that 5'5' pairs are also enriched in co-expressed pairs when compared to embedded pairs (chi-square = 23.5, df = 1, *p *= 1.2 × 10^-6^). We observed no statistical difference among the three categories regarding differential expression of both genes in a pair.

### Influence of antisense transcripts in the splicing of sense transcripts

It is quite clear nowadays that a significant fraction of all human genes undergo regulated alternative splicing, producing more than one mature mRNA from a gene (Galante *et al*. [27] and references therein). Although several regulatory elements in *cis *and *trans *have been identified (for a review see Pagani and Baralle [50]), it is reasonable to say that we are far from a complete understanding of how constitutive and alternative splicing are regulated. One possible regulatory mechanism involves antisense sequences. Since the late 1980s, it is known that antisense RNA can inhibit splicing of a pre-mRNA *in vitro *[15]. A few years later, Munroe and Lazar [51] observed that NATs could inhibit the splicing of a message derived from the other DNA strand, more specifically the *ErbAα *gene. More recently, Yan *et al*. [52] characterized a new human gene, called *SAF*, which is transcribed from the opposite strand of the *FAS *gene. Over-expression of *SAF *altered the splicing pattern of *FAS *in a regulated way, suggesting that *SAF *controls the splicing of *FAS*. With the growing amount of genomic loci presenting both sense and antisense transcripts, a general role for S-AS pairing in splicing regulation has been proposed [47]. However, no systematic large-scale analysis has been reported so far investigating this issue for mammals. We made use of the human dataset described in this report to tackle this problem.

We first tested whether the rate of alternative splicing in the sense gene would be affected by the existence of an antisense transcript. It is expected that the effect of S-AS pairing on splicing would be restricted to those exon-intron borders located in the region involved in pairing. We therefore restricted the analysis to those exon-intron borders spanning the region involved in an S-AS pairing. Our strategy was to compare the number of splicing variants for those borders against all other exon-intron borders (those without an antisense transcript) in the same genes. To make the analysis more informative we split the borders into four categories (terminal donor, internal donor, internal acceptor and terminal acceptor). For both internal donor and acceptor sites, the presence of an antisense transcript slightly increased the rate of alternative splicing (Table 5; 4% and 3% increases, respectively). For the terminal sites, the presence of a NAT had the opposite effect (5% and 6% decrease for donor and acceptor, respectively). Table 5 also shows that these differences are predominantly due to intron retention. On the other hand, NATs located within the introns and exons (but not spanning the border) have no major effect on the splicing of the respective borders. The observed differences between borders with or without NATs is statistically significant (chi-square = 31.2, df = 1, *p *= 2.3 × 10^-8 ^for donor sites; and chi-square = 23, df = 1, *p *= 1.6 × 10^-6 ^for acceptor sites).

**Table 5:** Frequency of different types of alternative splicing in exon-intron borders with or without an antisense transcript

	Total	Alternative borders	Intron retention	Exon skipping	Alternative 3'/5' site
**Borders with antisense**					
Terminal donor	2,578	553	130	7	416
Internal donor	7,632	3,100	535	1,616	949
Terminal acceptor	7,749	3,145	493	1,642	1,010
Internal acceptor	2,763	688	208	7	473
					
**Borders without antisense**					
Terminal donor	2,200	579	101	32	446
Internal donor	23,414	8,674	1,080	4,997	2,597
Terminal acceptor	23,447	8,787	1,022	5,007	2,758
Internal acceptor	1,732	545	154	16	375

Recently, Wiemann *et al*. [53] reported a new variant of IL4L1 that contains the first two exons of an upstream gene, NUP62. This chimeric transcript was expressed in a tissue and cell-specific manner. The authors speculated that cell type specific alternative splicing was involved in the generation of this chimeric transcript. We speculate that NATs could be involved in the generation of this type of chimeric cDNA. The same antisense message pairing with both sense messages would form a double-stranded RNA that could induce the spliceosome to skip the paired region and join the two sense messages, a process very similar to the one proposed for *trans*-splicing in mammals [54]. Interestingly, we found five examples in our dataset of S-AS pairs in which the genomic organization of both sense and antisense genes suggest a process like this. Additional data file 9 illustrates one of these cases. It can be seen that two transcripts represented by cDNAs AK095876 and AK000438 join messages from genes *SERF2 *and *HYPK*. The antisense transcript is represented by cDNA AK097682. Additional data file 10 lists all other putative cases of chimeric transcripts. The fact that both sense genes share a common antisense transcript raises the possibility that antisense transcripts can mediate *trans*-splicing of the sense genes, thereby generating the chimeric transcript.

### On the evolution of S-AS pairs: functional implications

It is reasonable to assume that a fraction of all S-AS pairs reached this genome organization solely by chance. However, evidence presented here and elsewhere suggest that this fraction is probably small [6,55,56]. For example, Dahary *et al*. [6] concluded that antisense transcription had a significant effect on vertebrate genome evolution since the genomic organization of S-AS pairs is much more conserved than the organization of genes in general. However, how did this organization come to be? In principle, S-AS genomic organization should carry a negative effect on the overall fitness of a subject. For each gene in an S-AS pair, its evolution is constrained not only by features of its own sequence but also by functional features encoded by the other gene in the pair. The fact that we observed a significant amount of S-AS pairs in mammalian genomes suggests that there are advantages inherent to this organization to counter-balance the negative effects. The proposed role of NATs in gene regulation is certainly advantageous. We propose here two evolutionary scenarios, not mutually exclusive, that would speed up the generation of S-AS pairs. In one scenario, alternative polyadenylation has a fundamental role. Sun *et al*. [5] observed a preferential targeting of 3' UTRs for NATs. Our observation that 51% of 3'3' S-AS pairs overlap because of polyadenylation variants suggests that selection has favored cases where overlapping occurs only in a time and spatially regulated manner.

In a second scenario, retroposition generates NATs, which lack introns and may even show a polyadenylation tail integrated into the genome. We observe here that retroposition contributed significantly to the origin of S-AS pairs, especially those classified as embedded. What would be the selective advantages of retrocopies as NATs? Chen *et al*. [56] observed that antisense genes have shorter introns when compared to genes in general. They speculated that this feature was advantageous during evolution since NATs need to be "rapid responsers" to execute their regulatory activities. Although transcription is a slow process in eukaryotes, another bottleneck in the expression of a gene is splicing. Furthermore, Nott *et al*. [57] observed that the presence of introns in a gene affects gene expression by enhancing mRNA accumulation. Thus, the argument from Chen *et al*. [56] gets stronger with the data reported here and by Nott *et al*. [57] since intronless antisense genes would be transcribed even faster; their transcripts would simply skip splicing and the half-life of the respective messages would be shorter. All key features for genes involved in regulatory activities.

An important issue is the conservation of S-AS pairs between human and mouse. Although we found more than a thousand conserved pairs, this number is still small compared to the whole set of S-AS pairs in both species. Several factors, however, suggest that the number reported here is an underestimate. First, as discussed by Engstrom *et al*. [25], sequence conservation might not be of primary importance for antisense regulation. Furthermore, it is likely that many truly conserved pairs were not detected because transcript sequences have not been discovered yet. This is more critical in the face of our findings that a significant proportion of 3'3' S-AS pairs depend on alternative polyadenylation for an overlap. It is also quite likely that some S-AS pairs are lineage-specific. For instance, our finding that retroposition contributes to the origin of many S-AS pairs could explain the appearance of lineage-specific S-AS pairs, assuming that the retroposition event occurred after the divergence between human and mouse.

These two evolutionary scenarios (alternative polyadenylation and retroposition) might produce S-AS pairs with different functional implications. The expression and evolutionary conservation analyses presented here, together with evidence from others [5,19,23,47,48] suggest that 3'3' overlap achieved by polyadenylation variants was used throughout evolution to regulate gene expression. Those pairs generated through retroposition may be involved in some other types of regulation, such as alternative splicing.

## Conclusion

This is the deepest survey so far of S-AS pairs in the human and mouse genomes. We made use of all cDNAs available in the public domain together with 122 MPSS libraries for human and mouse. The major findings of the present report include: as many as 10,077 and 8,091 S-AS pairs were identified for human and mouse respectively; using MPSS data, we found 4,308 and 216 new putative S-AS loci in human and mouse, respectively; a small fraction of all S-AS pairs are artifacts caused by genomic priming during cDNA library construction; a significant amount of S-AS pairs is due to retroposition events of one of the genes in the pair; quantitative analyses suggest that the presence of an antisense gene, complementary to an exon-intron border of the sense gene, increases the rate of retention of the respective intron. Furthermore, we propose an evolutionary model in which alternative polyadenylation and retroposition are important forces in the generation of S-AS pairs.

Taken together, these results offer, up to now, the vastest catalog of S-AS pairs in human and mouse genomes.

## Materials and methods

### Mapping cDNAs and MPSS tags onto the human and mouse genomes

We used a modified protocol similar to the one described previously to identify transcription clusters in the human and mouse genomes [27,28]. Briefly, genome sequence (NCBI build no. 35 for human and NCBI build no. 33 for mouse), EST collections (5,992,459 sequences for human and 4,246,824 sequences for mouse) and mRNA sequences (186,358 for human and 120,058 for mouse) were downloaded from UCSC [58]. All cDNAs were mapped to the respective genome sequence using BLAT (default parameters) [59]. The best hit for each cDNA in the genome was identified, followed by a pairwise alignment using Sim4 [60]. Only transcripts presenting identity ≥94%, coverage ≥50% and all splice sites in the same orientations were used.

Correct orientation of ESTs was determined by the presence of a poly-A tail (a stretch of 8 As at the 3' end) and/or a splicing donor (GT) and acceptor (AG) sites. All mRNAs were considered in the 'sense' orientation (oriented from 5' end to 3' end). All cDNAs mapped and reliably orientated were assembled into clusters. One cluster contains cDNAs presenting the same orientation and sharing at least one exon-intron boundary or a minimum of 30 nucleotides of overlap (only for those sequences without a common exon/intron organization).

For the mapping of MPSS data, we first extracted 'virtual' tags for both human and mouse genomes by simply finding all *Dpn*II sites and extracting a 13 (human) or 16 (mouse) nucleotide long sequence immediately downstream of the restriction site in both orientations. These 'virtual' tags present only once in the respective genomes were further used and matched against the 'real' tags found in 41 and 81 MPSS libraries for human and mouse, respectively. Only MPSS tags classified as 'reliable' (present in more than one sequencing run) and 'significant' (tags per million >3) were considered as trusted signatures.

### Identification of S-AS pairs

S-AS pairs were identified as those cases in which two clusters, in opposite orientations, overlap at the genome level. For the correct orientation of all mapped cDNAs, we took into consideration several parameters, including: sequence annotation as available in the respective GenBank entry; splice junctions; and poly-A tails and poly-T heads. We excluded from our analyses all cDNAs that presented conflicting orientations as defined by the three criteria above. If only two clusters overlap in the opposite orientation, they were classified as a single bidirectional S-AS pair. If a given cluster overlaps with more than one antisense cluster, they were classified as multiple bidirectional S-AS pairs. S-AS pairs were also classified according to their genomic pattern. Parameters evaluated included: pattern of S-AS overlap (exonic, intronic and exonic/intronic); spanning of introns by the components of a pair as defined by their alignment onto the genome; and chromosome localization and relative orientation within the S-AS pairs (tail-tail, head-head and embedded).

### Conservation between human and mouse S-AS pairs

We used three strategies to evaluate the degree of conservation between human and mouse S-AS pairs. First, all pairs were searched against the dataset from HomoloGene [35] and those pairs conserved in both species were counted. In our second strategy, we selected those S-AS pairs in which at least one gene was conserved according to HomoloGene. We then used Needle, an alignment algorithm [61], to test sequence conservation between the respective antisense genes. We classified as conserved those global alignments with identity >30%. Finally, we also used the strategy from Engstrom *et al*. [25]. We used the net alignment between human and mouse genomes (retrieved from the UCSC Genome Browser database) to define the corresponding (synthenic) regions. We considered a human S-AS pair to be conserved in mouse if it had an exon region aligning (>20 bp) to an exon region from a mouse pair.

### Investigation of the expression pattern of S-AS transcripts

We evaluated the expression pattern of S-AS pairs at the whole genome level based on their expression profiles obtained from MPSS libraries (available at [36]). The procedure was previously described by us for SAGE and MPSS [27,31,49]. The tag to gene assignment was done by scanning and extracting virtual tags (13 nucleotide-long sequences present downstream to the 3'-most *Dpn*II restriction sites of each mRNA sequence). To accurately represent the 3' end of a transcript, only mRNA sequences containing a poly-A tail were used. All tags mapped to two or more different genes were excluded and the frequencies of different tags for the same gene (mainly alternative polyadenylation variants) were summed. MPSS tags were normalized to counts-per-million and the expression data were cross-linked to genomic positions by the extraction of virtual tags for both the human and mouse genomes. Only tags showing 100% identity with a genomic locus were used in the analyses.

The classification of the expression pattern of S-AS pairs was done using those tags with ≥3 tags per million across all MPSS libraries. To evaluate the co-expression of all S-AS pairs, both genes in a pair had to be co-expressed in at least 04 libraries. If both genes in a pair were co-expressed in less than four libraries or they were independently expressed in different libraries, the pair was classified as 'single-gene expression'. The remaining S-AS pairs were classified as 'no-expression'.

### Identification of antisense MPSS tags

All *Dpn*II sites in the human and mouse genomes were identified and for each site two 'virtual' MPSS tags were extracted from both DNA strands in the correct orientation. All 'virtual' MPSS tags mapped in the opposite strand of known mRNAs in both genomes were identified. Those mRNAs belonging to an S-AS pair previously identified were excluded. Those antisense MPSS tags mapped just once in the respective genome and present in at least one MPSS library were identified and submitted to experimental validation.

### Simulations on the genomic organization of S-AS pairs

A random distribution of S-AS pairs was obtained by re-indexing the coordinates of one gene in all the pairs 1,000 times. This was done by randomly selecting a genomic coordinate for the start of mapping of a given gene. All the remaining exon-intron borders were then re-indexed based on this initial coordinate. The relative organization of both genes in all random S-AS pairs was stored and frequencies for each category were calculated. Those frequencies were used as the expectation for chi-square tests of the null hypothesis.

### Identification of splicing variants

Using the database mentioned earlier and described elsewhere [26-28] we identified all exon-intron borders complementary to a NAT. We then compared the rate of alternative splicing in these borders against the borders from the same genes without a NAT. We established a set of stringent criteria to identify alternative borders. These criteria are detailed elsewhere [26-28].

### Experimental validation of MPSS antisense tags

MPSS tags corresponding to antisense transcripts were converted into their corresponding 3' cDNA fragments using GLGI-MPSS [37]. Antisense tags were selected from a MPSS library derived from the normal breast luminal epithelial cell line HB4a and the same RNA source was used for GLGI amplification. For the GLGI-MPSS amplification, we used a sense primer including 17 bases of the MPSS tag sequence and 6 additional bases (CAGGGA), giving a total of 23 bases for each primer (5'-CAGGGAGATCXXXXXXXXXXXXX-3'). We also used an antisense primer (ACTATCTAGAGCGGCCGCTT) present in the 3' end of all cDNA molecules that was incorporated from reverse transcription primers in cDNA synthesis. The reaction mixture was prepared in a final volume of 30 μl, including 1× PCR buffer, 2.0 mM MgCl_2_, 83 μM dNTPs, 2.3 ng/μl antisense primer, 2.3 ng/μl sense primer, 1.5 U of Taq Platinum DNA polymerase (Invitrogen, San Diego, CA, USA) and 0.5-0.8 μl of the same cDNA source used for MPSS library construction. PCR conditions used for amplification were 94°C for 2 minutes, followed by 30 cycles at 94°C for 30 s, 64°C for 30 s, and 72°C for 35 s. Reactions were kept at 72°C for 5 minutes after the last cycle. The amplified products were ethanol precipitated and cloned into the pGEM^®^-T Easy vector (Promega, Madison, WI, USA). Twelve colonies for each GLGI-MPSS fragment were screened by PCR using pGEM universal primers and positive colonies were sequenced using Big-Dye Terminator (Applied Biosystems, Foster City, CA, USA) and an ABI3100 sequencer (Applied Biosystems).

### Experimental validation of genomic primed sequences

Total RNA derived from fetal liver, colon and lung was purchased from Clontech laboratories (Palo Alto, CA, USA). For cDNA synthesis, 2 μg of total RNA were treated (or not) with 100 units of *DNAse *I (FPLC-pure, Amersham, Piscataway, NJ, USA) and were reverse transcribed using oligo(dT)12-18, random primers and *SuperScript *II (Invitrogen), following the manufacturers' instructions. After synthesis, the resulting cDNA was subjected to *RNase *H treatment. The absence of genomic DNA contamination was evaluated for each preparation. DNA-free total RNA was subjected to PCR amplification using primers within intronic sequences flanking exon 12 of the hMLH-1 gene (forward, 5' TGGTGTCTCTAGTTCTGG3'; reverse 5' CATTGTTGTAGTAGCTCTGC 3'). All PCR amplifications were carried out using 2 μl of cDNA as a template to the final volume of 25 μl and 1× buffer, 1.5 mM MgCl_2_, 0.2 mM dNTP, 0.2 μM of each specific primer and 0.025 U/μl of Taq DNA polymerase (Life Technologies, San Diego, CA, USA). The following cycling protocol was used: initial denaturation of 94°C for 4 minutes; 94°C for 30 s; 55°C for 45 s; 72°C for 1 minute for 35 cycles; along with a final extension at 72°C for 7 minutes. All PCR products were resolved on 8% polyacrylamide gels and sequenced as described above to verify amplification specificity.

### Strand-specific RT-PCR

In the strand-specific RT-PCR, orientation of the transcript is accessed by restricting which gene-specific primer is present during first-strand cDNA synthesis. For each candidate, 1 μg of total RNA was treated with Promega RQ1 RNAse-free DNAse and tested for remaining DNA contamination as described above. First-strand cDNA synthesis was carried out at 50°C for 2 h using 200 U of *SuperScript *II (Invitrogen) and 0.9 μM of a primer complementary to the antisense transcript. PCR amplifications were performed using 1 μl of the first-strand cDNA as a template in a final volume of 25 μl and 1× buffer, 1.5 mM MgCl_2_, 0.1 mM dNTP, 0.4 μM of gene specific primers and 1 U of Platinum Taq DNA polymerase (Invitrogen). The following cycling conditions were used for amplification: initial denaturation of 95°C for 2 minutes; 94°C for 40 s; reaction-specific annealing temperature for 40 s and 72°C for 1 minute for 35 cycles; followed by a final extension step at 72°C for 7 minutes. All PCR products were resolved on 8% polyacrylamide gels. Controls for the absence of self-priming during cDNA synthesis were done with reverse transcriptase in the absence of primers, and controls for the absence of DNA were done by incubation with primers but with no reverse transcriptase.

### Availability

To make our dataset fully accessible to the community we have set up a worldwide web portal [62] containing all raw data generated in this study and a series of tools to explore the data.

## Additional data files

The following additional data are available with the online version of this paper. Additional data file 1 is a list of representative GenBank entries for all S-AS pairs in both human and mouse. Additional data file 2 is a table showing the total number of S-AS pairs by chromosome for both human and mouse. Additional data file 3 shows the number of clusters and S-AS pairs when a less stringent clustering methodology is applied. Additional data file 4 shows a schematic view of all possible genomic organizations of S-AS pairs. Additional data file 5 lists all S-AS pairs conserved between human and mouse using the three strategies described in the text. Additional data file 6 shows the fraction of S-AS pairs conserved between human and mouse that are classified as 'Fully intronic' and the fraction of conserved S-AS pairs that contain at least one intronless gene. Additional data file 7 is a list of all MPSS libraries used in this study. Additional data file 8 presents the number of cDNA-based pairs that were further confirmed by the MPSS data. Additional data file 9 is a figure illustrating chimeric transcripts joining two adjacent genes (*SERF2 *and *HYPK*) with a NAT located between them. Additional file 10 lists all cases of chimeric transcripts identified in our dataset.

## Supplementary Material

Additional data file 1 Representative GenBank entries for all S-AS pairs in both human and mouse. Click here for file

Additional data file 2 Total number of S-AS pairs by chromosome for both human and mouse. Click here for file

Additional data file 3 Number of clusters and S-AS pairs when a less stringent clustering methodology is applied. Click here for file

Additional data file 4 All possible genomic organizations of S-AS pairs. Click here for file

Additional data file 5 All S-AS pairs conserved between human and mouse using the three strategies described in the text. Click here for file

Additional data file 6 The fraction of S-AS pairs conserved between human and mouse that are classified as 'Fully intronic' and the fraction of conserved S-AS pairs that contain at least one intronless gene. Click here for file

Additional data file 7 All MPSS libraries used in this study. Click here for file

Additional data file 8 The number of cDNA-based pairs that were further confirmed by the MPSS data. Click here for file

Additional data file 9 Chimeric transcripts joining two adjacent genes (*SERF2 *and *HYPK*) with a NAT located between them. Click here for file

Additional data file 10 All cases of chimeric transcripts identified in our dataset. Click here for file
