# Supplementary material for: Sense-antisense pairs in mammals: functional and evolutionary considerations
Source: Genome Biol. 2007 Mar 19;8(3):R40. doi: 10.1186/gb-2007-8-3-r40 (PMC1868933; doi:10.1186/gb-2007-8-3-r40)
Supplement: Additional data file 2 — Total number of S-AS pairs by chromosome for both human and mouse. [file gb-2007-8-3-r40-S2.doc]

**Additional data file 2**. Distribution of S-AS pairs per chromosome for both human and mouse.

|  | **Human** | | **Mouse** | |
| --- | --- | --- | --- | --- |
| **Chromosome** | **Number of loci** | **S-AS loci / total loci** | **Number of loci** | **S-AS loci / total loci** |
| **1** | 1018 | 45.3% | 529 | 37.9% |
| **2** | 766 | 45.9% | 693 | 40.5% |
| **3** | 613 | 46.3% | 403 | 34.7% |
| **4** | 428 | 44.0% | 505 | 37.0% |
| **5** | 495 | 42.1% | 537 | 39.0% |
| **6** | 538 | 44.3% | 466 | 36.8% |
| **7** | 485 | 41.8% | 499 | 32.9% |
| **8** | 416 | 46.6% | 439 | 38.0% |
| **9** | 415 | 44.9% | 415 | 36.3% |
| **10** | 417 | 44.8% | 379 | 34.4% |
| **11** | 540 | 42.3% | 658 | 39.3% |
| **12** | 505 | 44.2% | 295 | 35.5% |
| **13** | 217 | 45.5% | 304 | 34.4% |
| **14** | 318 | 44.0% | 332 | 39.6% |
| **15** | 365 | 47.9% | 328 | 37.0% |
| **16** | 394 | 43.6% | 270 | 36.7% |
| **17** | 556 | 43.4% | 337 | 35.7% |
| **18** | 184 | 47.1% | 247 | 40.4% |
| **19** | 506 | 35.9% | 290 | 40.0% |
| **20** | 282 | 44.7% | - | - |
| **21** | 125 | 44.8% | - | - |
| **22** | 247 | 47.4% | - | - |
| **X** | 320 | 35.8% | 223 | 26.0% |
| **Y** | 11 | 12.5% | 4 | 12.5% |
