# Supplementary material for: Sense-antisense pairs in mammals: functional and evolutionary considerations
Source: Genome Biol. 2007 Mar 19;8(3):R40. doi: 10.1186/gb-2007-8-3-r40 (PMC1868933; doi:10.1186/gb-2007-8-3-r40)
Supplement: Additional data file 3 — Number of clusters and S-AS pairs when a less stringent clustering methodology is applied. [file gb-2007-8-3-r40-S3.rtf]

Additional data file 3. Number of clusters and S-AS pairs obtained after different clustering strategies.  “Original” refers to the more stringent strategy originally described in the main text. “Strategy #1” clusters same strand sequences overlapping 1 nt and collapses all clusters (same strand) distant from each other less than 30nt. “Strategy #2” clusters same strand sequences overlapping 1 nt and collapses all clusters (same strand) distant from each other less than 60nt. “Total (mRNAs/ESTs)” refers to all clusters in the database while “Total (mRNA)”refers to those clusters containing at least one full-length mRNA.
  
Clustering	Human	Mouse	
	Total
(mRNAs ESTs) 	Total
(mRNA)	S-AS pairs 	Total (mRNAs+ESTs)	Total (mRNAs)	S-AS pairs 	
Original 	111442	35,925	10,077	65497	34,921	8091	
Stringency #1	109,124 (~2%)	35,610 (~1%)	10,046 (0.3%)	 64,890 (~1%)	34,256 (2%)	8064 (0.3%)	
Stringency #2	107,372 (~3%)	35,534 (1.1%)	10,004 (0.7%)	64,620 (1.3%)	34,211 (~2%)	8040 (0.6%)	
               
