# Supplementary figures and images for: Sense-antisense pairs in mammals: functional and evolutionary considerations
Source: Genome Biol. 2007 Mar 19;8(3):R40. doi: 10.1186/gb-2007-8-3-r40 (PMC1868933; doi:10.1186/gb-2007-8-3-r40)

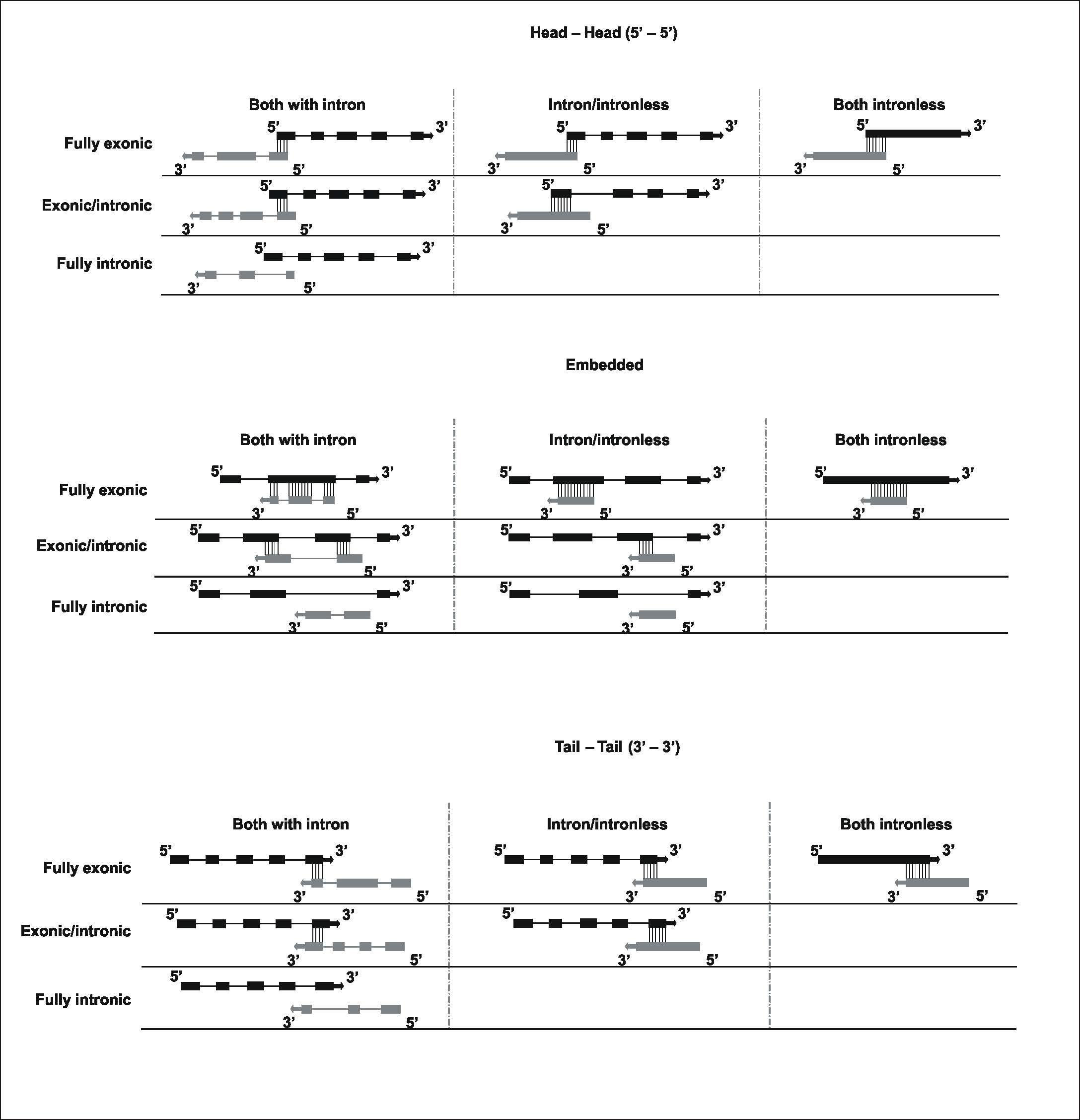

Supplement: Additional data file 4 — All possible genomic organizations of S-AS pairs. [file gb-2007-8-3-r40-S4.tiff]

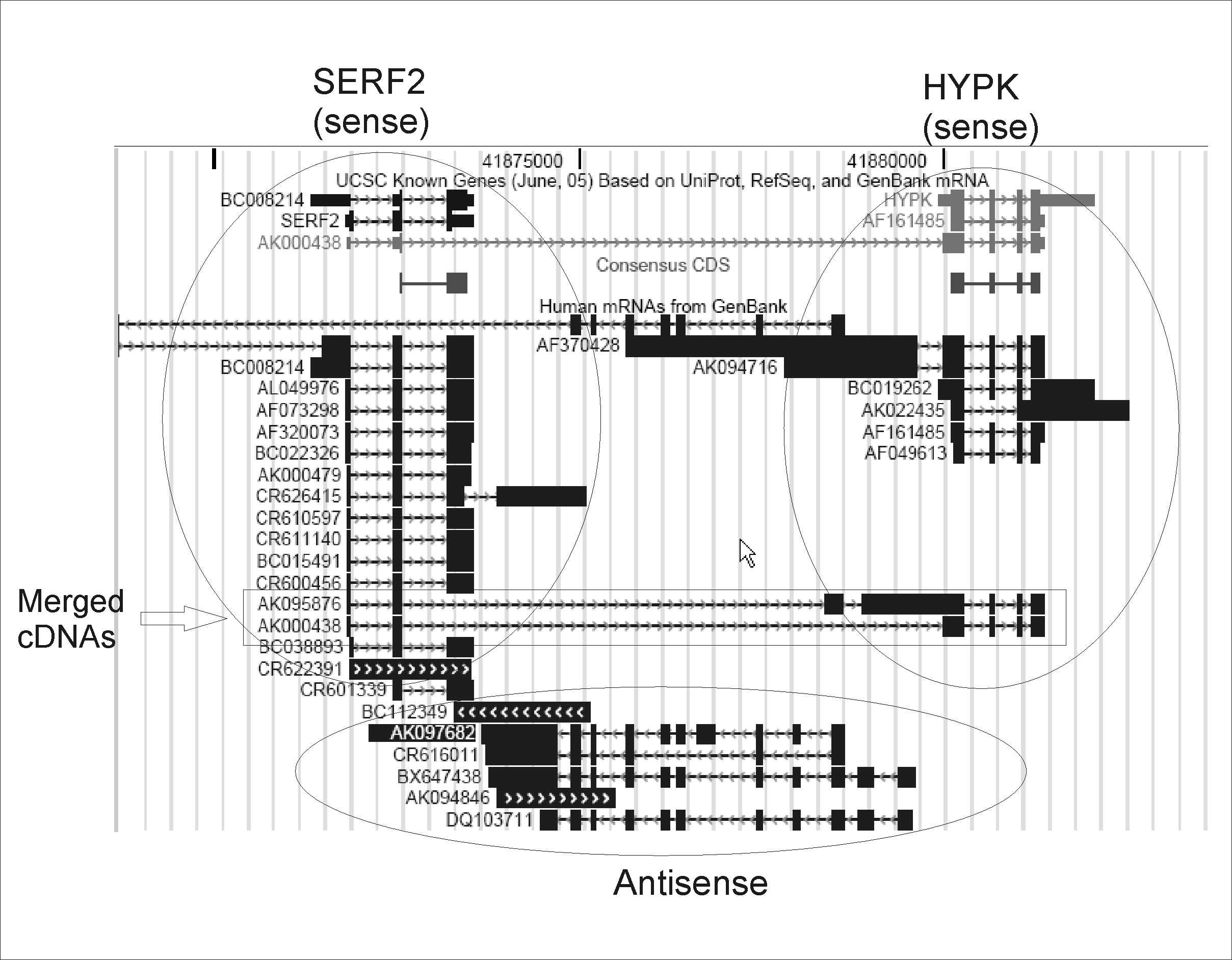

Supplement: Additional data file 9 — Chimeric transcripts joining two adjacent genes (SERF2 and HYPK) with a NAT located between them. [file gb-2007-8-3-r40-S9.tiff]
