# Supplementary material for: Sense-antisense pairs in mammals: functional and evolutionary considerations
Source: Genome Biol. 2007 Mar 19;8(3):R40. doi: 10.1186/gb-2007-8-3-r40 (PMC1868933; doi:10.1186/gb-2007-8-3-r40)
Supplement: Additional data file 6 — The fraction of S-AS pairs conserved between human and mouse that are classified as 'Fully intronic' and the fraction of conserved S-AS pairs that contain at least one intronless gene. [file gb-2007-8-3-r40-S6.doc]

**Additional data file 6**: Classification of S-AS pairs conserved between human and mouse in reference to their exon/intron overlap (A) and presence of an intronless gene in the pair (B).

**A**

|  | Human | Mouse |
| --- | --- | --- |
| **Fully exonic** | 37% | 32% |
| **Exonic / intronic** | 57% | 50% |
| **Fully intronic** | 6% | 18% |

**B**

|  | Human | Mouse |
| --- | --- | --- |
| **Both with intron** | 70% | 74% |
| **Intron-Intronless** | 25% | 24% |
| **Both intronless** | 5% | 2% |
