# Supplementary material for: Sense-antisense pairs in mammals: functional and evolutionary considerations
Source: Genome Biol. 2007 Mar 19;8(3):R40. doi: 10.1186/gb-2007-8-3-r40 (PMC1868933; doi:10.1186/gb-2007-8-3-r40)
Supplement: Additional data file 7 — All MPSS libraries used in this study. [file gb-2007-8-3-r40-S7.pdf]

Additional data file 6 - 41 MPSS libraries derived from human

libid	libname	tissue_or_Cell_type
1	HuAG.basl	adrenal gland - normal
2	HuBl.basl	bladder - normal
3	HuBM.basl	bone marrow - normal
4	HuBr.amyg	brain, amygdala - normal
5	HuBr.CaNu	brain, caudate nucleus - norma
6	HuBr.cere	brain, cerebellum - normal
7	HuBr.CoCa	brain, corpus callosum - norm
8	HuBr.hypo	brain, hypothalamus - normal
9	HuBr.thal	brain, thalamus - normal
10	HuFB.basl	fetal brain, whole - normal
11	HuHe.basl	heart - normal
12	HuKi.basl	kidney - normal
13	HuLu.basl	lung - normal
14	HuMG.basl	mammary gland - normal
15	HuPG.basl	pituitary gland - normal
16	HuPl.basl	placenta - normal
17	HuPn.basl	pancreas - normal
18	HuPr.basl	prostate - normal
19	HuRe.basl	retina - normal
20	HuSC.basl	spinal cord - normal
21	HuSG.basl	salivary gland - normal
22	HuSI.basl	small intestine - normal
23	HuSp.basl	spleen - normal
24	HuSt.basl	stomach - normal
25	HuTe.basl	testis - normal
26	HuTm.basl	thymus - normal
27	HuTr.basl	trachea - normal
28	HuTy.basl	thyroid - normal
29	HuUt.basl	uterus - normal
30	HuCo.basl	colon transversum - normal
49	HuMo.basl	Monocytes
55	HuPB.ctrl	peripheral blood lymphocytes
56	HuLC.basl_sig03	HB4A Normal sample
62	HuPl.norm_sig01	Human placenta
63	HuTe.norm_sig01	Human testis
64	HuLE.basl_sig01	Human normal epithelial cell
65	HuMc.LiPg_sig02	human melanocyte lightly pigme
68	colon.3	colon
37	tmp.liver	liver
38	tmp.skeletal.muscle	skeletal muscle
39	tmp.brain.whole	brain whole

Additional data file 6 - 81 MPSS libraries derived from mouse

libid	libname	tissue_or_Cell_type
1	MoBl.Male.sig21	Bladder
2	MoEs.Male.sig21	Esophagus
3	MoHe.FeVS.sig21	Heart:ventricles and septum
4	MoKi.FeMd.sig21	Kidney:medulla
5	MoKi.MaCr.sig21	Kidney:contex
6	MoKi.MaMd.sig21	Kidney:medulla
7	MoWF.FeAb.sig21	White fat
8	MoBr.FeTh.sig22	Brain:Thalamus
9	MoBr.MaCb.sig21	Brain:Cerebellum
10	MoBr.MaHi.sig22	Brain:Hippocampus
11	MoBr.MaMb.sig21	Brain:Midbrain
12	MoLi.MaLL.sig21	Liver:left lobe
13	MoSk.FeHB.sig22	Skin:hariy, from back
14	MoWF.MaAb.sig22	White fat
15	MoAd.Feml.sig21	Adrenal
16	MoBl.Feml.sig21	Bladder
17	MoBn.MaFm.sig21	Bone:Femur
18	MoBr.MHyP.sig21	Brain:Hypothalamus/preoptic ar
19	MoBr.MaAm.sig21	Brain:Amygdala
20	MoKi.FeCr.sig23	Kidney:cortex
21	MoOv.Feml.sig21	Ovary
22	MoSt.Feml.sig23	Stomach
23	MoUt.FP18.sig21	Uterus:Pregnant E18
24	MoUt.Feml.sig21	Uterus
25	MoEs.Feml.sig22	Esophagus
26	MoMG.Feml.sig23	Mammary gland
27	MoSC.FeEn.sig21	Spinal cord:entire
28	MoBn.FeFm.sig21	Bone:Femur
29	MoBr.FeOB.sig22	Brain:Olfactory Bulb
30	MoBr.MCPA.sig21	Brain:Caudate,Putamen,Accumben
31	MoEy.Male.sig21	Eye
32	MoSk.MaHB.sig21	Skin:hairy, from back
33	MoSp.Feml.sig22	Spleen
34	MoTp.Feml.sig21	Thyroid/parathyroid
35	MoBr.FTCS.sig22	Brain:OlfactoryTubercle,Prefro
36	MoHe.MaVS.sig21	Heart: ventricles and septum
37	MoLI.Male.sig21	Large intestine
38	MoLi.MaRL.sig21	Liver:right lobe
39	MoTh.Male.sig21	Thymus
40	MoAo.Male.sig21	Heart:Aorta
41	MoBr.FHyP.sig23	Brain:Hypothalamus/preoptic ar
42	MoBr.MaTh.sig21	Brain:Thalamus
43	MoCg.MaXi.sig21	Cartilage:Xiphoid
44	MoEy.Feml.sig21	Eye
45	MoLN.MaMs.sig22	Lymph nodes: mesenteric
46	MoPi.Feml.sig21	Pituitary
47	MoPi.Male.sig22	Pituitary
48	MoSC.MaEn.sig21	Spinal cord:entire

49	MoTh.Feml.sig21	Thymus
50	MoTp.Male.sig21	Thyroid/parathyroid
51	MoBr.MTCS.sig22	Brain:OlfactoryTubercle,Prefro
52	MoCV.Feml.sig23	Cervix and vagina
53	MoCg.FeXi.sig21	Cartilage:Xiphoid
54	MoEm.MF18.sig21	Embryo E18
55	MoHe.FeAt.sig21	Heart: atria
56	MoHe.MaAt.sig21	Heart:Atria
57	MoLI.Feml.sig21	Large intestine
58	MoLu.Feml.sig21	Lung
59	MoLu.Male.sig21	Lung
60	MoSI.Feml.sig21	Small intestine
61	MoTe.Male.sig22	Testis
62	MoAd.Male.sig21	Adrenal
63	MoAo.Feml.sig21	Heart:Aorta
64	MoBF.Feml.sig21	Brown Fat
65	MoBF.Male.sig21	Brown Fat
66	MoBr.FCPA.sig22	Brain:Caudate,Putamen,Accumben
67	MoLN.FeMs.sig21	Lymph nodes: mesenteric
68	MoPl.FE18.sig21	Placenta - E18
69	MoBr.FeCM.sig22	Brain:Cortical mantle
70	MoBr.FeMb.sig21	Brain:Midbrain
71	MoBr.MaCM.sig22	Brain:Cortical mantle
72	MoBr.MaOB.sig22	Brain:Olfactory Bulb
73	MoBr.FeAm.sig23	Brain:Amygdala
74	MoBr.FeCb.sig21	Brain:Cerebellum
75	MoBr.FeHi.sig21	Brain:Hippocampus
76	MoLi.FeRL.sig23	Liver:right lobe
77	MoPr.Male.sig21	Prostate
78	MoSI.Male.sig22	Small intestine
79	MoSM.FeTh.sig21	SkeletalMuscle:Thigh
80	MoSM.MaTh.sig21	SkeletalMuscle:Thigh
81	MoSp.Male.sig22	Spleen
