# Supplementary material for: Sense-antisense pairs in mammals: functional and evolutionary considerations
Source: Genome Biol. 2007 Mar 19;8(3):R40. doi: 10.1186/gb-2007-8-3-r40 (PMC1868933; doi:10.1186/gb-2007-8-3-r40)
Supplement: Additional data file 8 — The number of cDNA-based pairs that were further confirmed by the MPSS data. [file gb-2007-8-3-r40-S8.doc]

**Additional data file 8**: Number of cDNA-based S-AS pairs supported by MPSS data. The percentage in parenthesis corresponds to the fraction of cDNA-based S-AS pairs supported by MPSS. Please refer to Table I in the manuscript to obtain the total number of pairs in each category.

| cDNA type | Single bidirectional transcription | | Multiple bidirectional transcription | |
| --- | --- | --- | --- | --- |
|  | **Human** | **Mouse** | **Human** | **Mouse** |
| **mRNA-mRNA** | 1804  (86%) | 778  (41%) | 948  (94%) | 519  (72%) |
| **mRNA-ESTs** | 2349  (71%) | 972  (30%) | 3359  (92%) | 1267  (57%) |
| **Total** | **4153**  **(77%)** | **1750**  **(34%)** | **4307**  **(92%)** | **1786**  **(61%)** |
