# Supplementary material for: Sense-antisense pairs in mammals: functional and evolutionary considerations
Source: Genome Biol. 2007 Mar 19;8(3):R40. doi: 10.1186/gb-2007-8-3-r40 (PMC1868933; doi:10.1186/gb-2007-8-3-r40)
Supplement: Additional data file 10 — All cases of chimeric transcripts identified in our dataset. [file gb-2007-8-3-r40-S10.doc]

Additional data file 10: All 5 cases of chimeric transcripts identified in our set of S-AS pairs (see main text for more details).

| Sense #1 | Sense #2 | Antisense | cDNA joining both  sense genes |
| --- | --- | --- | --- |
| SERF2 | HYPK | AK093233 | AK095876  AK000438 |
| STAG3 | MGC2463 | AK095056 | AL834328 |
| CHMP4 | MGC5987 | AK094345 | BX161512 |
| PTPM1 | NDUFS3 | AK001312 | AK127967 |
| CR749349 | BC073916 | CR627203 | AK127470 |
