# Supplementary material for: Sense-antisense pairs in mammals: functional and evolutionary considerations
Source: Genome Biol. 2007 Mar 19;8(3):R40. doi: 10.1186/gb-2007-8-3-r40 (PMC1868933; doi:10.1186/gb-2007-8-3-r40)
Supplement: Additional data file 5 — All S-AS pairs conserved between human and mouse using the three strategies described in the text. [file gb-2007-8-3-r40-S5.zip › Additional_data_file5_190_pairs.doc]

```
Additional data file 5: 190 S-AS pairs conserved between human and mouse.
+-----------------------+------------------------------+-------------------------------+
|   Conserved pairs     |      Human S-AS pairs        |       Mouse S-AS pairs        |
+-----------------------+------------------------------+-------------------------------+
|      #    1           | *Seq. mapped in strand Plus* | *Seq. mapped in strand Plus*  |
|                       |           CR593482           |            U07425             |
|                       |           BC035028           |          BC034543             |
|                       |           CR456573           |          AY008722             |
|                       |             M12849           |          AK007065             |
|                       |             X03498           |          AK039203             |
|                       |                              |          AK005042             |
|                       |                              |          BC030066             |
|                       | *Seq. mapped in strand Minus*| *Seq. mapped in strand Minus* |
|                       |           BC046202           |          BC055479             |
|                       |           AK124692           |          BC022999             |
|                       |           BC053654           |          BC075629             |
|                       |             L36151           |          BC049252             |
|                       |           BC018120           |                               |
|                       |           AF012872           |                               |
|                       |           AK095811           |                               |
|                       |           CR620194           |                               |
|                       |           AK131431           |                               |
+-----------------------+------------------------------+-------------------------------+
|      #    2           | *Seq. mapped in strand Plus* | *Seq. mapped in strand Plus*  |
|                       |           BX648521           |          AK016459             |
|                       |           AK021414           |          BC061039             |
|                       |           AK026594           |                               |
|                       |           AK026609           |                               |
|                       |           BC029529           |                               |
|                       |           AK000560           |                               |
|                       |           AK026167           |                               |
|                       |           AK098686           |                               |
|                       |           BC019829           |                               |
|                       |           BC071888           |                               |
|                       |           BC004188           |                               |
|                       |           BC012835           |                               |
|                       |           AF064849           |                               |
|                       |           BC002783           |                               |
|                       |           BC039175           |                               |
|                       |           BC002885           |                               |
|                       |           BC019359           |                               |
|                       |           BC007889           |                               |
|                       |           BC024038           |                               |
|                       |           BC071889           |                               |
|                       |           BC001911           |                               |
|                       |           AL832497           |                               |
|                       |           AY167990           |                               |
|                       |           BC008006           |                               |
|                       | *Seq. mapped in strand Minus*| *Seq. mapped in strand Minus* |
|                       |           AK097419           |          BC022919             |
|                       |           AY604177           |          BC005547             |
+-----------------------+------------------------------+-------------------------------+
|      #    3           | *Seq. mapped in strand Plus* | *Seq. mapped in strand Plus*  |
|                       |           AF151850           |          AK050459             |
|                       |           CR601285           |          AK039676             |
|                       |           CR607746           |          AK039422             |
|                       |           AK025376           |          AK043574             |
|                       |           CR594347           |          BC039570             |
|                       |           AK126646           |                               |
|                       |           BC011895           |                               |
|                       |           CR602433           |                               |
|                       |           CR616941           |                               |
|                       |           AK128853           |                               |
|                       |           BC036603           |                               |
|                       |           AK095083           |                               |
|                       | *Seq. mapped in strand Minus*| *Seq. mapped in strand Minus* |
|                       |           AJ420462           |          AK087102             |
|                       |           AK001956           |          AK081579             |
|                       |           AF131848           |          BC024075             |
|                       |           CR616104           |          BC015285             |
|                       |           CR622527           |          AK090004             |
|                       |           CR601040           |          AK050587             |
|                       |           BC001457           |          AK043949             |
|                       |           CR618393           |          AK050583             |
+-----------------------+------------------------------+-------------------------------+
|      #    4           | *Seq. mapped in strand Plus* | *Seq. mapped in strand Plus*  |
|                       |           BX640789           |          BC061161             |
|                       |           BC028604           |          AK077275             |
|                       |             D29641           |            D84376             |
|                       |           BC065258           |          AY247795             |
|                       |           BC014669           |          AY247796             |
|                       |           BC031779           |                               |
|                       |           AK123505           |                               |
|                       | *Seq. mapped in strand Minus*| *Seq. mapped in strand Minus* |
|                       |           AL117656           |          BC014810             |
|                       |           AF014402           |          AK088426             |
|                       |           AF014403           |          BC029230             |
|                       |           CR593294           |          AK012158             |
|                       |           CR597320           |          AK078091             |
|                       |           CR618167           |                               |
|                       |           CR615521           |                               |
|                       |           CR602046           |                               |
|                       |           CR601918           |                               |
|                       |           CR619523           |                               |
|                       |           BC039847           |                               |
|                       |           CR617594           |                               |
|                       |           CR617429           |                               |
|                       |           AB000888           |                               |
|                       |           BC008787           |                               |
+-----------------------+------------------------------+-------------------------------+
|      #    5           | *Seq. mapped in strand Plus* | *Seq. mapped in strand Plus*  |
|                       |           BC036181           |            U12236             |
|                       |           AB039834           |          AK040983             |
|                       |           AF289865           |                               |
|                       |           BC016626           |                               |
|                       |           BC047457           |                               |
|                       |           CR615999           |                               |
|                       |           AK056691           |                               |
|                       |           BC050662           |                               |
|                       | *Seq. mapped in strand Minus*| *Seq. mapped in strand Minus* |
|                       |           AF289864           |            D87326             |
|                       |             L25851           |                               |
|                       |           AK097567           |                               |
+-----------------------+------------------------------+-------------------------------+
|      #    6           | *Seq. mapped in strand Plus* | *Seq. mapped in strand Plus*  |
|                       |           CR594393           |          BC027061             |
|                       |           AL832441           |          BC022914             |
|                       |           AK025681           |          AK035123             |
|                       |           BC021662           |                               |
|                       |           AK125461           |                               |
|                       |           AK095813           |                               |
|                       | *Seq. mapped in strand Minus*| *Seq. mapped in strand Minus* |
|                       |             D37984           |          AB017105             |
|                       |             L36140           |          AK046660             |
|                       |           AY157499           |          BC014735             |
|                       |           BC001052           |          AB017104             |
|                       |           BT007119           |                               |
+-----------------------+------------------------------+-------------------------------+
|      #    7           | *Seq. mapped in strand Plus* | *Seq. mapped in strand Plus*  |
|                       |           BC039014           |          AK049889             |
|                       |           AK023212           |          AK034418             |
|                       |           BC031070           |          BC021895             |
|                       |           CR613797           |                               |
|                       | *Seq. mapped in strand Minus*| *Seq. mapped in strand Minus* |
|                       |           CR624143           |          AK034160             |
|                       |             U73704           |          BC003446             |
|                       |           BC001257           |          AJ566083             |
|                       |           AJ347709           |          AK020948             |
|                       |           CR610554           |          AK018066             |
|                       |           AJ302735           |          AK042124             |
+-----------------------+------------------------------+-------------------------------+
|      #    8           | *Seq. mapped in strand Plus* | *Seq. mapped in strand Plus*  |
|                       |           BC013202           |          BC010816             |
|                       |             Z94154           |                               |
|                       |             Y12546           |                               |
|                       |           BC031653           |                               |
|                       |           BC039595           |                               |
|                       |           AK126849           |                               |
|                       |             Z94155           |                               |
|                       | *Seq. mapped in strand Minus*| *Seq. mapped in strand Minus* |
|                       |           CR592921           |          BC070439             |
|                       |           AK123014           |          AY255543             |
|                       |           CR613400           |                               |
|                       |           AL512716           |                               |
|                       |           BC008977           |                               |
|                       |           BC001370           |                               |
|                       |           AK093692           |                               |
|                       |           AF527769           |                               |
|                       |           AF527768           |                               |
|                       |           AF527770           |                               |
|                       |           AK091652           |                               |
|                       |           AF527767           |                               |
|                       |           AK094954           |                               |
|                       |           AF527764           |                               |
|                       |           AK055363           |                               |
|                       |           AK022470           |                               |
|                       |           AK000906           |                               |
|                       |           BC065816           |                               |
|                       |           AF527765           |                               |
|                       |           AK095790           |                               |
|                       |           AF527766           |                               |
|                       |           CR621396           |                               |
|                       |           CR620349           |                               |
|                       |           CR620493           |                               |
|                       |           CR610908           |                               |
|                       |           CR626426           |                               |
|                       |           CR620664           |                               |
|                       |           CR598773           |                               |
|                       |           AF484961           |                               |
+-----------------------+------------------------------+-------------------------------+
|      #    9           | *Seq. mapped in strand Plus* | *Seq. mapped in strand Plus*  |
|                       |             D14705           |          AK036752             |
|                       |             D13866           |            X59990             |
|                       |           AK094151           |          BC060174             |
|                       |           BC000385           |          BC068316             |
|                       |             L23805           |            D90362             |
|                       |             U03100           |          BC048163             |
|                       |             L22080           |          AK004623             |
|                       |           BC031262           |          AK046513             |
|                       |           CR591756           |                               |
|                       |           AK055751           |                               |
|                       | *Seq. mapped in strand Minus*| *Seq. mapped in strand Minus* |
|                       |           AB007876           |          AK122278             |
|                       |           CR600503           |          AK049920             |
|                       |           AY182026           |          AK046818             |
|                       |                              |          AK044705             |
|                       |                              |          AK036155             |
|                       |                              |          AK043173             |
|                       |                              |          AK046774             |
|                       |                              |          AK043784             |
+-----------------------+------------------------------+-------------------------------+
|      #   10           | *Seq. mapped in strand Plus* | *Seq. mapped in strand Plus*  |
|                       |             D12625           |            L10370             |
|                       |             M82814           |            D30730             |
|                       |             M89914           |            D30731             |
|                       |             D42072           |            X54924             |
|                       |           BX537850           |          AK033306             |
|                       |             M38106           |          AK050818             |
|                       |             M38107           |                               |
|                       |             M60915           |                               |
|                       |             M60496           |                               |
|                       | *Seq. mapped in strand Minus*| *Seq. mapped in strand Minus* |
|                       |             X51694           |          AK082179             |
|                       |           BC018050           |          AK039141             |
|                       |           BC035572           |          BC024757             |
|                       |           CR590095           |          AK034440             |
|                       |           BC005926           |          BC017548             |
|                       |           CR616854           |          AK046407             |
+-----------------------+------------------------------+-------------------------------+
|      #   11           | *Seq. mapped in strand Plus* | *Seq. mapped in strand Plus*  |
|                       |           CR597645           |          AK002559             |
|                       |           AF109873           |          AK012453             |
|                       |           AF092135           |          BC010712             |
|                       |           AF349444           |          AK077783             |
|                       |           CR596911           |          AK017543             |
|                       |           CR591214           |          AK005258             |
|                       |           CR613655           |          AL357453             |
|                       |           CR610869           |          AK078402             |
|                       |           CR597592           |          AK078409             |
|                       |           CR590606           |          AK014440             |
|                       |           CR600484           |          BC069897             |
|                       |           CR609929           |          AK078826             |
|                       |           CR596029           |          AL357452             |
|                       |           CR609265           |                               |
|                       |           CR594809           |                               |
|                       |           CR614421           |                               |
|                       |           CR598629           |                               |
|                       |           CR599918           |                               |
|                       |           AL109701           |                               |
|                       |           CR623365           |                               |
|                       |           BC024151           |                               |
|                       |           CR607243           |                               |
|                       |           CR621532           |                               |
|                       |           CR605769           |                               |
|                       |           CR609938           |                               |
|                       |           AL357456           |                               |
|                       |           CR611063           |                               |
|                       |           CR597731           |                               |
|                       |           CR612975           |                               |
|                       |           CR599202           |                               |
|                       |           BC007944           |                               |
|                       |           CR598752           |                               |
|                       |           CR624399           |                               |
|                       |           AF274951           |                               |
|                       |           AF274947           |                               |
|                       |           AL137570           |                               |
|                       |           AK098383           |                               |
|                       | *Seq. mapped in strand Minus*| *Seq. mapped in strand Minus* |
|                       |           AK124916           |          BC060643             |
|                       |           AK125762           |          AY464564             |
|                       |           AY358250           |          AK036946             |
|                       |           BC033907           |                               |
|                       |           AY464565           |                               |
+-----------------------+------------------------------+-------------------------------+
|      #   12           | *Seq. mapped in strand Plus* | *Seq. mapped in strand Plus*  |
|                       |           AK097657           |          AK013489             |
|                       |           AK054600           |          BC024461             |
|                       |           CR616453           |          AK085984             |
|                       |           CR610440           |                               |
|                       |           CR610544           |                               |
|                       |           CR620427           |                               |
|                       |           CR591328           |                               |
|                       |           CR619752           |                               |
|                       |           BC004561           |                               |
|                       |           CR617383           |                               |
|                       |           BC009359           |                               |
|                       |           CR607074           |                               |
|                       |           CR606019           |                               |
|                       |           CR590133           |                               |
|                       |           CR610666           |                               |
|                       |           BC001616           |                               |
|                       |           BC002625           |                               |
|                       |           BC015556           |                               |
|                       |           CR608100           |                               |
|                       |           CR619932           |                               |
|                       |           CR623272           |                               |
|                       |           CR602810           |                               |
|                       |           CR609806           |                               |
|                       |           BC036708           |                               |
|                       |           CR619967           |                               |
|                       |             M65028           |                               |
|                       |             U76713           |                               |
|                       |             Z36844           |                               |
|                       | *Seq. mapped in strand Minus*| *Seq. mapped in strand Minus* |
|                       |           AK023470           |          AK013709             |
|                       |           BC067745           |          AK013384             |
|                       |           BC037238           |          BC043069             |
|                       |           BC008009           |          AK076132             |
|                       |           BC037567           |            D90151             |
|                       |           CR611632           |          AY137376             |
|                       |           BC039012           |          AK047205             |
|                       |           BC028002           |          AK019387             |
|                       |           CR619518           |                               |
|                       |           AK093737           |                               |
+-----------------------+------------------------------+-------------------------------+
|      #   13           | *Seq. mapped in strand Plus* | *Seq. mapped in strand Plus*  |
|                       |           AK024552           |          BC010190             |
|                       |           AL136640           |          AK005462             |
|                       |           BC001716           |          AK009909             |
|                       |           CR592408           |          AK008252             |
|                       |           CR611769           |          AK012839             |
|                       |           CR626771           |                               |
|                       |           AF151052           |                               |
|                       |           CR600873           |                               |
|                       |           CR593605           |                               |
|                       |           BC007764           |                               |
|                       |           AF317675           |                               |
|                       |           BC048106           |                               |
|                       |           BC062718           |                               |
|                       |           BC039337           |                               |
|                       |           CR533489           |                               |
|                       | *Seq. mapped in strand Minus*| *Seq. mapped in strand Minus* |
|                       |           AK092655           |          AK085499             |
|                       |           AF098277           |          BC013528             |
|                       |           BC050261           |          AF058318             |
|                       |           BC019225           |                               |
|                       |           AF170911           |                               |
|                       |           AF058317           |                               |
|                       |           AJ269477           |                               |
|                       |           AJ250807           |                               |
+-----------------------+------------------------------+-------------------------------+
|      #   14           | *Seq. mapped in strand Plus* | *Seq. mapped in strand Plus*  |
|                       |           AK026969           |          AK036141             |
|                       |           BC065037           |          AK034371             |
|                       |           BC007325           |          AK083168             |
|                       |           AF312865           |          AK080106             |
|                       |           BC014351           |          BC046322             |
|                       |                              |          AK038860             |
|                       |                              |          AK048496             |
|                       |                              |          AK043028             |
|                       | *Seq. mapped in strand Minus*| *Seq. mapped in strand Minus* |
|                       |           AF288390           |          AK029710             |
|                       |           BC022507           |          AK048027             |
|                       |             Y15060           |          BC031127             |
|                       |                              |          BC027756             |
|                       |                              |          AK080861             |
|                       |                              |                               |
|                       |                              |                               |
|                       |                              |                               |
+-----------------------+------------------------------+-------------------------------+
|      #   15           | *Seq. mapped in strand Plus* | *Seq. mapped in strand Plus*  |
|                       |           AK027859           |          AK122298             |
|                       |           CR592453           |          BC057113             |
|                       |           CR599751           |          BC057316             |
|                       |           BC002870           |          AF458089             |
|                       |           CR617150           |          AF253540             |
|                       |           BC047034           |          BC067010             |
|                       |           AK128334           |          AK010531             |
|                       | *Seq. mapped in strand Minus*| *Seq. mapped in strand Minus* |
|                       |           AK075424           |          AK013198             |
|                       |           AK095173           |          AK029226             |
|                       |           BC020148           |          AK032545             |
|                       |           AB011083           |          BC023148             |
|                       |           AK027857           |          BC036278             |
|                       |           AK122926           |          AK036089             |
|                       |           AF033861           |          BC034399             |
|                       |           BC073803           |          AK018444             |
+-----------------------+------------------------------+-------------------------------+
|      #   16           | *Seq. mapped in strand Plus* | *Seq. mapped in strand Plus*  |
|                       |             M95549           |          AK128900             |
|                       |           AK054830           |          AY033886             |
|                       |                              |          BC022226             |
|                       | *Seq. mapped in strand Minus*| *Seq. mapped in strand Minus* |
|                       |           BC059952           |          BC024080             |
|                       |           BC000822           |          BC017158             |
|                       |           AK092580           |                               |
|                       |           AK000679           |                               |
|                       |           BC009308           |                               |
|                       |           AK023930           |                               |
|                       |           CR593188           |                               |
+-----------------------+------------------------------+-------------------------------+
|      #   17           | *Seq. mapped in strand Plus* | *Seq. mapped in strand Plus*  |
|                       |           CR600041           |          BC049637             |
|                       |           CR596120           |          AK002276             |
|                       |           CR591565           |          BC057097             |
|                       |           AF077037           |                               |
|                       |           BC011006           |                               |
|                       |           CR602915           |                               |
|                       |           AY358968           |                               |
|                       |           AF144055           |                               |
|                       |           AF275744           |                               |
|                       |           BC002846           |                               |
|                       |           BC035850           |                               |
|                       |           CR607502           |                               |
|                       |           BC021237           |                               |
|                       |           CR604043           |                               |
|                       |           CR602545           |                               |
|                       |           CR607127           |                               |
|                       | *Seq. mapped in strand Minus*| *Seq. mapped in strand Minus* |
|                       |           AL096737           |          AK088682             |
|                       |           BC012806           |          BC065061             |
|                       |           BC015631           |          AY572835             |
|                       |           AK027855           |                               |
|                       |           AK075078           |                               |
|                       |           AF081571           |                               |
|                       |           AF069307           |                               |
|                       |           AF442150           |                               |
|                       |           AF442149           |                               |
|                       |           AF288781           |                               |
+-----------------------+------------------------------+-------------------------------+
|      #   18           | *Seq. mapped in strand Plus* | *Seq. mapped in strand Plus*  |
|                       |           AK027296           |          AF378759             |
|                       |           AB040964           |          AK018412             |
|                       |           AY090637           |          AK020642             |
|                       |           AF378755           |                               |
|                       |           AY181242           |                               |
|                       |           AK074099           |                               |
|                       |           AL832959           |                               |
|                       |           AL832524           |                               |
|                       |           AL110244           |                               |
|                       |           AL137610           |                               |
|                       |           CR603687           |                               |
|                       | *Seq. mapped in strand Minus*| *Seq. mapped in strand Minus* |
|                       |           CR604537           |          AK017767             |
|                       |           AF130058           |          AK083122             |
|                       |           BC010648           |          AK012680             |
|                       |           AK001914           |          AK028228             |
|                       |           AF206673           |          AK012459             |
|                       |           CR616155           |                               |
|                       |           CR614939           |                               |
|                       |           CR613791           |                               |
|                       |           CR607467           |                               |
|                       |           CR591369           |                               |
|                       |           CR602291           |                               |
|                       |           AF298153           |                               |
+-----------------------+------------------------------+-------------------------------+
|      #   19           | *Seq. mapped in strand Plus* | *Seq. mapped in strand Plus*  |
|                       |           BC005184           |          AK010662             |
|                       |           BC012537           |          AB063106             |
|                       |           AB063105           |          BC031764             |
|                       |           AF151805           |          AK011764             |
|                       |           AK124137           |                               |
|                       |           CR622655           |                               |
|                       |           AF130117           |                               |
|                       | *Seq. mapped in strand Minus*| *Seq. mapped in strand Minus* |
|                       |           CR627060           |          BC046967             |
|                       |           BX647351           |          AK050557             |
|                       |           BC059171           |          BC033458             |
|                       |           BC067811           |          AF229032             |
|                       |           AK123077           |          BC069905             |
|                       |           AF117230           |          AK032836             |
|                       |           AK058191           |          AK076144             |
|                       |           AK027507           |          AK077707             |
|                       |           BC017419           |          AK075959             |
|                       |           CR608669           |                               |
|                       |           CR617747           |                               |
+-----------------------+------------------------------+-------------------------------+
|      #   20           | *Seq. mapped in strand Plus* | *Seq. mapped in strand Plus*  |
|                       |             Y14385           |            X75014             |
|                       |             L36818           |                               |
|                       |           CR591731           |                               |
|                       |             L24444           |                               |
|                       | *Seq. mapped in strand Minus*| *Seq. mapped in strand Minus* |
|                       |           CR590341           |          BC049961             |
|                       |           BC041564           |            U92477             |
|                       |           CR607218           |          BC063080             |
|                       |           CR593042           |          BC039207             |
|                       |           CR600761           |          AF162781             |
|                       |           CR607262           |                               |
|                       |           CR596068           |                               |
|                       |           CR621077           |                               |
|                       |           CR604478           |                               |
|                       |           CR595525           |                               |
+-----------------------+------------------------------+-------------------------------+
|      #   21           | *Seq. mapped in strand Plus* | *Seq. mapped in strand Plus*  |
|                       |           CR596181           |          BC004645             |
|                       |           BC026196           |          AK084023             |
|                       |           BC014092           |                               |
|                       |             U80040           |                               |
|                       |           CR621307           |                               |
|                       |           CR595461           |                               |
|                       |           CR456365           |                               |
|                       |           CR596278           |                               |
|                       |           CR619036           |                               |
|                       |           CR536568           |                               |
|                       |           CR592345           |                               |
|                       |           CR596641           |                               |
|                       |           CR613743           |                               |
|                       | *Seq. mapped in strand Minus*| *Seq. mapped in strand Minus* |
|                       |           AK026524           |          AK032967             |
|                       |           AK000365           |          AK019868             |
|                       |           AB051452           |          BC010793             |
|                       |           BC017248           |                               |
|                       |           CR606203           |                               |
|                       |           CR597879           |                               |
|                       |           CR621091           |                               |
|                       |           AY092087           |                               |
|                       |           CR456459           |                               |
|                       |           AK129663           |                               |
+-----------------------+------------------------------+-------------------------------+
|      #   22           | *Seq. mapped in strand Plus* | *Seq. mapped in strand Plus*  |
|                       |           BC069118           |          AK047827             |
|                       |           AF436849           |          AK077996             |
|                       |             M86932           |          AK048507             |
|                       |           BC074951           |          AK048162             |
|                       |             S67147           |          AK079651             |
|                       | *Seq. mapped in strand Minus*| *Seq. mapped in strand Minus* |
|                       |           BC035785           |          BC059090             |
|                       |           AF177663           |            D31768             |
|                       |           AF022212           |            D31769             |
|                       |           AF117067           |          AK029358             |
|                       |           AF177665           |            M10095             |
|                       |           AF012272           |                               |
+-----------------------+------------------------------+-------------------------------+
|      #   23           | *Seq. mapped in strand Plus* | *Seq. mapped in strand Plus*  |
|                       |           BC071866           |            L09192             |
|                       |           BC015581           |            M97957             |
|                       |           BC007718           |          AK077665             |
|                       |           BC027475           |                               |
|                       |           CR615886           |                               |
|                       |           BC014040           |                               |
|                       |           BC000207           |                               |
|                       |           CR601830           |                               |
|                       | *Seq. mapped in strand Minus*| *Seq. mapped in strand Minus* |
|                       |           CR618813           |          BC023036             |
|                       |             S72370           |          BC023156             |
|                       |             U30891           |          AK081560             |
|                       |           CR620429           |                               |
|                       |             K02282           |                               |
|                       |             M26122           |                               |
+-----------------------+------------------------------+-------------------------------+
|      #   24           | *Seq. mapped in strand Plus* | *Seq. mapped in strand Plus*  |
|                       |           AF451989           |          AK039618             |
|                       |           AL832247           |                               |
|                       |           BC020844           |                               |
|                       |           BX647957           |                               |
|                       |           CR624785           |                               |
|                       |           CR618271           |                               |
|                       |           CR597464           |                               |
|                       |           AK074733           |                               |
|                       |           AF289571           |                               |
|                       |           CR601006           |                               |
|                       | *Seq. mapped in strand Minus*| *Seq. mapped in strand Minus* |
|                       |           AF038170           |          BC014757             |
|                       |           AF110331           |          AK014623             |
|                       |           BC025661           |          AK082745             |
|                       |           BC036929           |          AK080726             |
|                       |           AK000467           |                               |
|                       |           AF223944           |                               |
|                       |           BC048344           |                               |
|                       |           AF110330           |                               |
|                       |           CR624702           |                               |
+-----------------------+------------------------------+-------------------------------+
|      #   25           | *Seq. mapped in strand Plus* | *Seq. mapped in strand Plus*  |
|                       |           AK054889           |          AK044097             |
|                       |           BC027919           |          BC038500             |
|                       |           CR600554           |                               |
|                       |           AY358163           |                               |
|                       |           CR615264           |                               |
|                       |           BC063469           |                               |
|                       |           BC009560           |                               |
|                       |           AK075064           |                               |
|                       | *Seq. mapped in strand Minus*| *Seq. mapped in strand Minus* |
|                       |           AK126203           |          AK014510             |
|                       |           AK000007           |          BC057367             |
|                       |           AY217346           |          AK079461             |
|                       |           AY358478           |          BC052886             |
|                       |           BC047016           |                               |
|                       |           AF086518           |                               |
+-----------------------+------------------------------+-------------------------------+
|      #   26           | *Seq. mapped in strand Plus* | *Seq. mapped in strand Plus*  |
|                       |           AB002349           |          AK037265             |
|                       |           BC033708           |          AF125176             |
|                       |           BC019329           |                               |
|                       |           BC032372           |                               |
|                       |           AF221098           |                               |
|                       |           BX648170           |                               |
|                       |           AK023463           |                               |
|                       | *Seq. mapped in strand Minus*| *Seq. mapped in strand Minus* |
|                       |           AK054861           |          BC056182             |
|                       |           AF007150           |          BC037725             |
|                       |           AK075026           |          BC072656             |
|                       |           AK074726           |          AK129123             |
|                       |           BC012368           |          AK076767             |
|                       |           AY358274           |                               |
|                       |           AF125175           |                               |
+-----------------------+------------------------------+-------------------------------+
|      #   27           | *Seq. mapped in strand Plus* | *Seq. mapped in strand Plus*  |
|                       |           AJ505989           |          AK076096             |
|                       |           AJ517294           |          BC005622             |
|                       |           BX649106           |          AK010253             |
|                       |           CR595328           |          AF152841             |
|                       |           CR617928           |          BC052156             |
|                       |           CR621291           |          AF152842             |
|                       |           CR596830           |          AK011636             |
|                       |           CR621174           |                               |
|                       |           CR600057           |                               |
|                       |           BC012371           |                               |
|                       |             M58460           |                               |
|                       |             U09215           |                               |
|                       | *Seq. mapped in strand Minus*| *Seq. mapped in strand Minus* |
|                       |           CR619143           |          AK044924             |
|                       |           CR592551           |          BC052730             |
|                       |           CR597266           |            X75483             |
|                       |             X51688           |                               |
|                       |           CR604810           |                               |
|                       |           CR407692           |                               |
+-----------------------+------------------------------+-------------------------------+
|      #   28           | *Seq. mapped in strand Plus* | *Seq. mapped in strand Plus*  |
|                       |           AK094464           |          AF096867             |
|                       |           AK091286           |          BC053427             |
|                       |                              |          BC066004             |
|                       |                              |          AK029181             |
|                       |                              |          AK013810             |
|                       | *Seq. mapped in strand Minus*| *Seq. mapped in strand Minus* |
|                       |             U76456           |          BC064046             |
|                       |           BC010553           |          AK038820             |
|                       |           AK094210           |          AK079347             |
|                       |                              |          AF282730             |
|                       |                              |          AY622856             |
+-----------------------+------------------------------+-------------------------------+
|      #   29           | *Seq. mapped in strand Plus* | *Seq. mapped in strand Plus*  |
|                       |           CR598555           |            Y09632             |
|                       |           AK025790           |          AK083412             |
|                       |           AF070672           |          AK051995             |
|                       |           AF153329           |          AK084732             |
|                       |           BC012999           |          AK083697             |
|                       |           AK024804           |          AK051201             |
|                       |                              |          AK053280             |
|                       |                              |          BC060608             |
|                       | *Seq. mapped in strand Minus*| *Seq. mapped in strand Minus* |
|                       |           BC017713           |          AK047789             |
|                       |           AF053977           |          AK052567             |
|                       |           AB011472           |          AK081461             |
|                       |           AF191341           |          AK038523             |
|                       |           BT009810           |          AK048246             |
|                       |           BC005258           |          AK031459             |
|                       |           BC010944           |          BC059013             |
|                       |                              |          AK079681             |
+-----------------------+------------------------------+-------------------------------+
|      #   30           | *Seq. mapped in strand Plus* | *Seq. mapped in strand Plus*  |
|                       |           AF020796           |          AK031638             |
|                       |           CR590642           |          BC011174             |
|                       |           CR611943           |          AF242858             |
|                       |           CR621800           |                               |
|                       |             Y08387           |                               |
|                       |           CR595500           |                               |
|                       |           BC018705           |                               |
|                       |           AF155158           |                               |
|                       |           BC012023           |                               |
|                       |           CR625474           |                               |
|                       |           BX640759           |                               |
|                       |           AK055686           |                               |
|                       |           CR625655           |                               |
|                       | *Seq. mapped in strand Minus*| *Seq. mapped in strand Minus* |
|                       |           BC018115           |          BC058583             |
|                       |           CR627390           |            D49439             |
|                       |             U31659           |          AK047944             |
|                       |           AK097781           |                               |
|                       |             L25444           |                               |
|                       |           CR624352           |                               |
+-----------------------+------------------------------+-------------------------------+
|      #   31           | *Seq. mapped in strand Plus* | *Seq. mapped in strand Plus*  |
|                       |           AK000002           |          AB011019             |
|                       |           AY032599           |                               |
|                       |           AK024446           |                               |
|                       |           AL133613           |                               |
|                       |           CR598420           |                               |
|                       |           BC024103           |                               |
|                       |           AL122095           |                               |
|                       | *Seq. mapped in strand Minus*| *Seq. mapped in strand Minus* |
|                       |           AY358126           |          BC060644             |
|                       |           BC000230           |          AF406642             |
|                       |           BC006425           |          AF417121             |
|                       |           AK055380           |                               |
|                       |           CR605295           |                               |
+-----------------------+------------------------------+-------------------------------+
|      #   32           | *Seq. mapped in strand Plus* | *Seq. mapped in strand Plus*  |
|                       |           CR592523           |          AK014038             |
|                       |           BC033796           |          AK043694             |
|                       |           BC036747           |          AK018548             |
|                       |           AK054841           |          BC052830             |
|                       |           BC004353           |          BC020468             |
|                       |           BC015008           |          AK003464             |
|                       |           AK092097           |                               |
|                       |           AK128869           |                               |
|                       |           AK024476           |                               |
|                       |           BC006437           |                               |
|                       |           BC018184           |                               |
|                       |           BC006278           |                               |
|                       |           CR596832           |                               |
|                       | *Seq. mapped in strand Minus*| *Seq. mapped in strand Minus* |
|                       |           AK095675           |          AK047517             |
|                       |           AK094812           |          AK012748             |
|                       |           AK056467           |          AK021220             |
|                       |           BC030248           |          AK034798             |
|                       |           AK126151           |          BC030466             |
|                       |           AK025861           |          AK005274             |
|                       |           AY129231           |                               |
|                       |           AK025641           |                               |
|                       |           CR595518           |                               |
|                       |           AF527735           |                               |
|                       |           CR595179           |                               |
|                       |           BC012951           |                               |
|                       |           BC036279           |                               |
|                       |           BC011783           |                               |
|                       |           CR604984           |                               |
|                       |           CR611036           |                               |
|                       |           CR593558           |                               |
|                       |           CR610455           |                               |
|                       |           CR612793           |                               |
|                       |           CR605414           |                               |
|                       |           CR599759           |                               |
|                       |           CR610459           |                               |
|                       |           CR625931           |                               |
|                       |           CR623234           |                               |
|                       |           AY029486           |                               |
|                       |           AB030207           |                               |
|                       |           AF493880           |                               |
|                       |           AK022743           |                               |
|                       |           AK125201           |                               |
|                       |           AK093050           |                               |
|                       |           AK025955           |                               |
|                       |           AB075873           |                               |
|                       |           BC010738           |                               |
|                       |           BC014196           |                               |
|                       |           AL832177           |                               |
|                       |           BC081535           |                               |
|                       |           CR620877           |                               |
|                       |           BC070105           |                               |
|                       |           CR616309           |                               |
|                       |           CR620551           |                               |
|                       |           AK056154           |                               |
|                       |           AK125605           |                               |
+-----------------------+------------------------------+-------------------------------+
|      #   33           | *Seq. mapped in strand Plus* | *Seq. mapped in strand Plus*  |
|                       |           CR625345           |          BC030905             |
|                       |           CR598398           |          AK007519             |
|                       |           BC009530           |                               |
|                       |           CR615306           |                               |
|                       |           AK091047           |                               |
|                       |           CR607060           |                               |
|                       |           CR618642           |                               |
|                       |           AK023981           |                               |
|                       | *Seq. mapped in strand Minus*| *Seq. mapped in strand Minus* |
|                       |           AF132952           |          AK009172             |
|                       |           AY013290           |          AK012280             |
|                       |           AK093971           |          AK088376             |
|                       |           AK124364           |          BC025117             |
|                       |           AK096409           |                               |
|                       |           AK094170           |                               |
|                       |           AK023436           |                               |
|                       |           CR619850           |                               |
|                       |           CR604423           |                               |
|                       |           CR619307           |                               |
|                       |           BC012291           |                               |
|                       |           CR624504           |                               |
|                       |           CR602037           |                               |
|                       |           AL359339           |                               |
+-----------------------+------------------------------+-------------------------------+
|      #   34           | *Seq. mapped in strand Plus* | *Seq. mapped in strand Plus*  |
|                       |           CR609785           |          AK004465             |
|                       |           CR598374           |          AK008741             |
|                       |           BC001771           |          AK032624             |
|                       |             X16901           |          BC028748             |
|                       |             X59745           |          AK032314             |
|                       | *Seq. mapped in strand Minus*| *Seq. mapped in strand Minus* |
|                       |           BC018063           |          AK003999             |
|                       |                              |          BC027173             |
|                       |                              |          AK083404             |
|                       |                              |                               |
|                       |                              |                               |
+-----------------------+------------------------------+-------------------------------+
|      #   35           | *Seq. mapped in strand Plus* | *Seq. mapped in strand Plus*  |
|                       |           BC008881           |          AK031309             |
|                       |             L04733           |          AF055665             |
|                       |           AF527736           |          BC055744             |
|                       |           AY180164           |          AK014256             |
|                       |           CR607419           |          BC031426             |
|                       |           CR613465           |                               |
|                       |           CR619184           |                               |
|                       |           AY180163           |                               |
|                       |           AY244715           |                               |
|                       |           AY180166           |                               |
|                       |           AY180170           |                               |
|                       |           AY180167           |                               |
|                       |           AY180169           |                               |
|                       |           AY180168           |                               |
|                       |           AY180165           |                               |
|                       |           CR608303           |                               |
|                       |           CR618577           |                               |
|                       |           AL133587           |                               |
|                       | *Seq. mapped in strand Minus*| *Seq. mapped in strand Minus* |
|                       |           AK022829           |          BC043073             |
|                       |           BC002949           |          AK014491             |
|                       |           AK126706           |                               |
|                       |           AK124498           |                               |
|                       |           AF035586           |                               |
|                       |           BC011725           |                               |
|                       |           BC001036           |                               |
|                       |           AK023646           |                               |
|                       |           CR622268           |                               |
|                       |           BX161398           |                               |
|                       |           CR603680           |                               |
|                       |           CR605233           |                               |
|                       |           CR613005           |                               |
|                       |           BT007417           |                               |
|                       |           CR610241           |                               |
|                       |           BC073820           |                               |
+-----------------------+------------------------------+-------------------------------+
|      #   36           | *Seq. mapped in strand Plus* | *Seq. mapped in strand Plus*  |
|                       |           BX647913           |          BC003262             |
|                       |           BC039074           |          AK077532             |
|                       |           BC046117           |                               |
|                       |           AK126963           |                               |
|                       | *Seq. mapped in strand Minus*| *Seq. mapped in strand Minus* |
|                       |           AK130697           |          AK077109             |
|                       |             L05425           |          AK006256             |
|                       |           BC000107           |                               |
|                       |           BC009250           |                               |
+-----------------------+------------------------------+-------------------------------+
|      #   37           | *Seq. mapped in strand Plus* | *Seq. mapped in strand Plus*  |
|                       |           CR625625           |          AK011766             |
|                       |           CR598952           |          AK002344             |
|                       |           CR615121           |            U94479             |
|                       |           CR601713           |          BC003737             |
|                       |           CR611887           |                               |
|                       |           CR608758           |                               |
|                       |             U40282           |                               |
|                       |           CR604127           |                               |
|                       |           CR625972           |                               |
|                       |           CR599632           |                               |
|                       |           BC001554           |                               |
|                       |           CR749220           |                               |
|                       |           CR624967           |                               |
|                       |           CR407673           |                               |
|                       |           CR622983           |                               |
|                       |           CR619697           |                               |
|                       | *Seq. mapped in strand Minus*| *Seq. mapped in strand Minus* |
|                       |           AL833496           |          BC031418             |
|                       |             U13991           |                               |
|                       |           CR597361           |                               |
|                       |           CR598728           |                               |
|                       |           BC012088           |                               |
|                       |           CR541943           |                               |
|                       |           CR597841           |                               |
+-----------------------+------------------------------+-------------------------------+
|      #   38           | *Seq. mapped in strand Plus* | *Seq. mapped in strand Plus*  |
|                       |           BC030702           |          AK083572             |
|                       |           BC056195           |          AK042026             |
|                       |           CR593857           |          BC056924             |
|                       |           AK022909           |          AY070216             |
|                       |           AK095865           |                               |
|                       |           AK057995           |                               |
|                       |           AK023946           |                               |
|                       | *Seq. mapped in strand Minus*| *Seq. mapped in strand Minus* |
|                       |           AK075219           |          AK040746             |
|                       |           AF004327           |          AF004326             |
|                       |           AF218015           |          BC027216             |
|                       |           BC022490           |          AK048622             |
|                       |           CR620685           |          AK019860             |
|                       |           AF187858           |                               |
|                       |           AB009865           |                               |
|                       |           CR611007           |                               |
|                       |           AJ289781           |                               |
|                       |           AK057771           |                               |
|                       |           CR599065           |                               |
+-----------------------+------------------------------+-------------------------------+
|      #   39           | *Seq. mapped in strand Plus* | *Seq. mapped in strand Plus*  |
|                       |           BC034619           |          AF182033             |
|                       |           BC044581           |          AK030120             |
|                       |           BC038714           |                               |
|                       |           AF182034           |                               |
|                       |           AF118125           |                               |
|                       | *Seq. mapped in strand Minus*| *Seq. mapped in strand Minus* |
|                       |           AF157316           |          BC029030             |
|                       |           AF251038           |          BC031465             |
|                       |           AL833699           |                               |
|                       |           CR611121           |                               |
|                       |           BC056887           |                               |
|                       |           BC041335           |                               |
|                       |           BC048965           |                               |
|                       |           AK130245           |                               |
+-----------------------+------------------------------+-------------------------------+
|      #   40           | *Seq. mapped in strand Plus* | *Seq. mapped in strand Plus*  |
|                       |           AL833242           |          AF117887             |
|                       |           AF055027           |          BC071267             |
|                       |           BC036886           |          BC036974             |
|                       |           CR604934           |          BC003964             |
|                       |                              |          BC008263             |
|                       |                              |          BC026427             |
|                       | *Seq. mapped in strand Minus*| *Seq. mapped in strand Minus* |
|                       |           CR591972           |          BC002282             |
|                       |           BC013014           |          BC003289             |
|                       |           BC000056           |          AK004959             |
|                       |           CR601786           |          AK033705             |
+-----------------------+------------------------------+-------------------------------+
|      #   41           | *Seq. mapped in strand Plus* | *Seq. mapped in strand Plus*  |
|                       |           BC021727           |          AK006174             |
|                       |           AY557347           |          AK005809             |
|                       |                              |          AK051087             |
|                       | *Seq. mapped in strand Minus*| *Seq. mapped in strand Minus* |
|                       |           AK122604           |          AK051258             |
|                       |           AF330002           |          AY065653             |
|                       |           AF315683           |          AY071828             |
|                       |           AY358987           |          AY071829             |
|                       |           BC002631           |          AK046112             |
|                       |           AF219624           |                               |
|                       |           CR590246           |                               |
|                       |           BC011774           |                               |
+-----------------------+------------------------------+-------------------------------+
|      #   42           | *Seq. mapped in strand Plus* | *Seq. mapped in strand Plus*  |
|                       |           CR618564           |          AK088329             |
|                       |           AF069987           |          AK006814             |
|                       |           CR622990           |          AK054496             |
|                       |           CR606194           |          BC054445             |
|                       |           BC046149           |          AK009728             |
|                       |           CR609891           |          BC023668             |
|                       |           CR593362           |          AF100342             |
|                       |           CR626659           |          AJ011386             |
|                       |           CR602066           |                               |
|                       |           CR541846           |                               |
|                       |           CR541814           |                               |
|                       |           CR616950           |                               |
|                       | *Seq. mapped in strand Minus*| *Seq. mapped in strand Minus* |
|                       |           AK001497           |          AK032189             |
|                       |           CR612611           |          BC021634             |
|                       |           AK022531           |          AK002269             |
|                       |           BC013910           |          AF069988             |
|                       |           BC016724           |                               |
|                       |           CR619646           |                               |
|                       |           CR625264           |                               |
|                       |           CR596035           |                               |
|                       |           CR597847           |                               |
|                       |           AF043733           |                               |
|                       |           AF064605           |                               |
|                       |           AF083236           |                               |
|                       |           AF100341           |                               |
|                       |           AJ010973           |                               |
|                       |           CR536556           |                               |
+-----------------------+------------------------------+-------------------------------+
|      #   43           | *Seq. mapped in strand Plus* | *Seq. mapped in strand Plus*  |
|                       |           AF348468           |          AB085629             |
|                       |           BC073944           |          AY236527             |
|                       | *Seq. mapped in strand Minus*| *Seq. mapped in strand Minus* |
|                       |           BX537526           |          AK078411             |
|                       |           AK021664           |          AK084235             |
|                       |           AK027316           |          AK047637             |
|                       |           BX647197           |          AK048404             |
|                       |           AB037762           |          AK129336             |
|                       |           CR596492           |          AK012133             |
|                       |           AK002075           |            U13262             |
|                       |           AF190159           |          AF483504             |
|                       |           BC014533           |          AF483505             |
|                       |           AK023133           |          AK034990             |
|                       |           AF106685           |                               |
|                       |           BC068523           |                               |
+-----------------------+------------------------------+-------------------------------+
|      #   44           | *Seq. mapped in strand Plus* | *Seq. mapped in strand Plus*  |
|                       |           BC036100           |          BC038475             |
|                       |           BC036443           |          AK013035             |
|                       |           AL161976           |          AK012171             |
|                       |           AF030196           |                               |
|                       |           CR542138           |                               |
|                       |           CR542127           |                               |
|                       |           BC005837           |                               |
|                       |           AK025795           |                               |
|                       | *Seq. mapped in strand Minus*| *Seq. mapped in strand Minus* |
|                       |           BC013727           |          BC019564             |
|                       |           AF131780           |          BC029643             |
|                       |           AK027464           |          BC020363             |
|                       |           AK027646           |          AK013044             |
|                       |           BC002856           |          AK029166             |
|                       |           BX648407           |                               |
|                       |           CR457152           |                               |
|                       |             D16955           |                               |
|                       |           BC018635           |                               |
+-----------------------+------------------------------+-------------------------------+
|      #   45           | *Seq. mapped in strand Plus* | *Seq. mapped in strand Plus*  |
|                       |           AF217968           |          AK011308             |
|                       |           CR625822           |          BC010800             |
|                       |           CR605807           |                               |
|                       |           CR599229           |                               |
|                       |           AF432221           |                               |
|                       |           CR610644           |                               |
|                       |           CR624881           |                               |
|                       |           CR614839           |                               |
|                       |           BC007545           |                               |
|                       |           BC022788           |                               |
|                       |           AF129085           |                               |
|                       |           BC017178           |                               |
|                       |           CR603664           |                               |
|                       |           AF039689           |                               |
|                       |           BC063617           |                               |
|                       |           CR608722           |                               |
|                       |           CR595251           |                               |
|                       |           CR593928           |                               |
|                       | *Seq. mapped in strand Minus*| *Seq. mapped in strand Minus* |
|                       |           AF370420           |          AK045776             |
|                       |           BC073785           |          AF129086             |
|                       |           CR592573           |          AK004464             |
|                       |           CR625506           |          BC038939             |
|                       |           CR608736           |          AK002752             |
|                       |           CR607606           |          BC027427             |
|                       |           CR621444           |                               |
|                       |           CR613585           |                               |
|                       |           CR624596           |                               |
+-----------------------+------------------------------+-------------------------------+
|      #   46           | *Seq. mapped in strand Plus* | *Seq. mapped in strand Plus*  |
|                       |             U86751           |          AK088271             |
|                       |           BC054044           |          BC011224             |
|                       |           BC046141           |            X07414             |
|                       |           BC009705           |                               |
|                       |           BC028109           |                               |
|                       |           BC065251           |                               |
|                       |           BC038992           |                               |
|                       |           AF017633           |                               |
|                       |             D16366           |                               |
|                       |             L49169           |                               |
|                       |           BC036724           |                               |
|                       |           BC063042           |                               |
|                       |           CR542101           |                               |
|                       |           BX538117           |                               |
|                       |           BC040197           |                               |
|                       |           BC051767           |                               |
|                       |           AK097324           |                               |
|                       | *Seq. mapped in strand Minus*| *Seq. mapped in strand Minus* |
|                       |           BC020589           |          AK075701             |
|                       |           AF078037           |          BC029206             |
|                       |           BC001475           |          AB091121             |
|                       |           BC014499           |          BC071199             |
|                       |           BC020259           |          BC016115             |
|                       |           BC032298           |          AK011811             |
|                       |           BC064913           |                               |
|                       |           AK130326           |                               |
|                       |           AK092039           |                               |
|                       |           BC008930           |                               |
|                       |           CR616265           |                               |
|                       |           AB069681           |                               |
|                       |           AF001925           |                               |
|                       |             M13194           |                               |
|                       |             M28650           |                               |
|                       |           CR590512           |                               |
|                       |           BC052813           |                               |
|                       |           AF433652           |                               |
+-----------------------+------------------------------+-------------------------------+
|      #   47           | *Seq. mapped in strand Plus* | *Seq. mapped in strand Plus*  |
|                       |           CR596103           |          AK043515             |
|                       |           CR622201           |          AK085222             |
|                       |           BC000006           |          AK044962             |
|                       |             U16799           |          BC038021             |
|                       |           CR407616           |          BC049225             |
|                       |           BT009787           |          AF202048             |
|                       | *Seq. mapped in strand Minus*| *Seq. mapped in strand Minus* |
|                       |           AF075062           |            X61433             |
|                       |           AF153191           |          AK005071             |
|                       |           BC006983           |          BC027319             |
|                       |           AK094513           |          AK010677             |
|                       |           CR605539           |            X16646             |
|                       |           CR599535           |                               |
+-----------------------+------------------------------+-------------------------------+
|      #   48           | *Seq. mapped in strand Plus* | *Seq. mapped in strand Plus*  |
|                       |           AF395707           |          AK045445             |
|                       |           CR621685           |          BC011146             |
|                       |           AF173375           |          AK053314             |
|                       |           AF161509           |          AK045345             |
|                       |           AB064961           |          AK083878             |
|                       |           AF395706           |          AK032681             |
|                       |           AF395705           |          AK017830             |
|                       |           BC012543           |          AK082579             |
|                       |                              |          AK046606             |
|                       | *Seq. mapped in strand Minus*| *Seq. mapped in strand Minus* |
|                       |           AY188447           |          AK002583             |
|                       |           BC080655           |          AK012681             |
|                       |           AK055945           |          AK007548             |
|                       |           AK123025           |          AK002824             |
|                       |           AF351784           |          BC002146             |
|                       |           AF141342           |          AK013455             |
+-----------------------+------------------------------+-------------------------------+
|      #   49           | *Seq. mapped in strand Plus* | *Seq. mapped in strand Plus*  |
|                       |           AK091112           |          BC026682             |
|                       |           BC001517           |          BC024426             |
|                       |           AK023937           |          BC024430             |
|                       |           AB014607           |          AY168619             |
|                       |           AF416922           |                               |
|                       |           AF416921           |                               |
|                       |           AL713723           |                               |
|                       | *Seq. mapped in strand Minus*| *Seq. mapped in strand Minus* |
|                       |           AY358629           |          AK122350             |
|                       |           BC073921           |          AK003873             |
|                       |           BC020874           |          BC042492             |
|                       |           BC015993           |          AK031588             |
+-----------------------+------------------------------+-------------------------------+
|      #   50           | *Seq. mapped in strand Plus* | *Seq. mapped in strand Plus*  |
|                       |           BC028106           |          AF121080             |
|                       |           AK000546           |          BC051940             |
|                       |           BC032059           |          AK051941             |
|                       |           AL359557           |                               |
|                       |           AK023577           |                               |
|                       |           CR613429           |                               |
|                       | *Seq. mapped in strand Minus*| *Seq. mapped in strand Minus* |
|                       |           CR607140           |          BC006896             |
|                       |           BC037974           |          AK032956             |
|                       |           BC025710           |          AK048556             |
|                       |           AB020598           |          AK036651             |
|                       |           AK127216           |                               |
|                       |           CR615238           |                               |
|                       |           AK130141           |                               |
|                       |           CR623241           |                               |
+-----------------------+------------------------------+-------------------------------+
|      #   51           | *Seq. mapped in strand Plus* | *Seq. mapped in strand Plus*  |
|                       |           AK093447           |          AF189764             |
|                       |           BC039576           |          BC013505             |
|                       |           BC056403           |                               |
|                       |           AY033290           |                               |
|                       | *Seq. mapped in strand Minus*| *Seq. mapped in strand Minus* |
|                       |           AF203687           |          AK029838             |
|                       |           AF226684           |          AK036866             |
|                       |           BC008658           |          BC017527             |
|                       |           BC012890           |          AK013330             |
|                       |           AK001976           |          AK003323             |
|                       |           BC036185           |          AF150808             |
|                       |           BC002765           |          AF193017             |
|                       |           BC016906           |                               |
|                       |           BC016472           |                               |
|                       |           AK023064           |                               |
|                       |           BC041032           |                               |
|                       |           CR612201           |                               |
|                       |           CR599904           |                               |
|                       |           CR622322           |                               |
|                       |           CR625761           |                               |
|                       |           CR610494           |                               |
|                       |           CR620242           |                               |
|                       |           CR610473           |                               |
+-----------------------+------------------------------+-------------------------------+
|      #   52           | *Seq. mapped in strand Plus* | *Seq. mapped in strand Plus*  |
|                       |           AL137661           |          AY050249             |
|                       |           BC036021           |          AK046752             |
|                       |           AK075278           |                               |
|                       |           AL137275           |                               |
|                       |           CR608097           |                               |
|                       |           BX641162           |                               |
|                       |           AB015331           |                               |
|                       |           AK021725           |                               |
|                       | *Seq. mapped in strand Minus*| *Seq. mapped in strand Minus* |
|                       |           BX537832           |          AY424292             |
|                       |           BC029604           |          BC050248             |
|                       |           BC031256           |          BC051173             |
|                       |           AK126273           |                               |
|                       |           BC047510           |                               |
|                       |           AL080110           |                               |
|                       |           BX648346           |                               |
|                       |           CR604581           |                               |
|                       |           CR606971           |                               |
|                       |           AK055774           |                               |
|                       |           AY424281           |                               |
+-----------------------+------------------------------+-------------------------------+
|      #   53           | *Seq. mapped in strand Plus* | *Seq. mapped in strand Plus*  |
|                       |           BC021019           |            L19661             |
|                       |           BC071979           |            X75947             |
|                       |           CR533459           |            X97982             |
|                       |           CR603166           |          AK012297             |
|                       |           BC004512           |          BC020478             |
|                       |           CR619133           |          AK006450             |
|                       |           CR592789           |                               |
|                       |           CR608819           |                               |
|                       |           BC071942           |                               |
|                       |           CR603638           |                               |
|                       |           CR620547           |                               |
|                       |           CR602496           |                               |
|                       |           BC001155           |                               |
|                       |           CR590150           |                               |
|                       |             X78136           |                               |
|                       |           CR606382           |                               |
|                       |           AK130583           |                               |
|                       |           AK094109           |                               |
|                       | *Seq. mapped in strand Minus*| *Seq. mapped in strand Minus* |
|                       |           BC050050           |          AK029882             |
|                       |             U07358           |          AK081623             |
|                       |           AK094195           |          AK034374             |
|                       |                              |            U23789             |
|                       |                              |          BC047158             |
|                       |                              |            U14636             |
+-----------------------+------------------------------+-------------------------------+
|      #   54           | *Seq. mapped in strand Plus* | *Seq. mapped in strand Plus*  |
|                       |           BC040540           |          AK032901             |
|                       |           BC039060           |          AK011967             |
|                       |             M19701           |          BC047931             |
|                       |             M28419           |          AK008952             |
|                       |             M15400           |          BC057175             |
|                       |             L41870           |          BC069991             |
|                       |             M33647           |          AK041799             |
|                       |           AF043224           |                               |
|                       |           AY429568           |                               |
|                       |           AK097860           |                               |
|                       | *Seq. mapped in strand Minus*| *Seq. mapped in strand Minus* |
|                       |           BX647109           |            M26391             |
|                       |           AK123059           |          AK011246             |
|                       |           BX537392           |                               |
|                       |           BC040850           |                               |
|                       |           BX537445           |                               |
|                       |           BX537438           |                               |
|                       |           AK122856           |                               |
|                       |           BC045651           |                               |
|                       |           BC070295           |                               |
|                       |           AF000546           |                               |
|                       |           CR597194           |                               |
|                       |           BC054346           |                               |
+-----------------------+------------------------------+-------------------------------+
|      #   55           | *Seq. mapped in strand Plus* | *Seq. mapped in strand Plus*  |
|                       |           BC031093           |          BC023414             |
|                       |           BC062993           |          AK020701             |
|                       |           BC015439           |          AK076337             |
|                       |           AL713789           |          AK009770             |
|                       | *Seq. mapped in strand Minus*| *Seq. mapped in strand Minus* |
|                       |           BC034696           |          AK020859             |
|                       |                              |          AK016794             |
|                       |                              |          AK021270             |
|                       |                              |          AK051159             |
+-----------------------+------------------------------+-------------------------------+
|      #   56           | *Seq. mapped in strand Plus* | *Seq. mapped in strand Plus*  |
|                       |           BC028037           |          BC055399             |
|                       |           BC028214           |            D16141             |
|                       |             X86371           |          BC050913             |
|                       |             D50550           |          AK084386             |
|                       |           BC051466           |          BC006017             |
|                       |                              |          BC043458             |
|                       |                              |          AK083672             |
|                       |                              |          BC029987             |
|                       | *Seq. mapped in strand Minus*| *Seq. mapped in strand Minus* |
|                       |             U01184           |          BC032282             |
|                       |           BC025300           |          BC027744             |
|                       |           BC021885           |          AF287264             |
|                       |           BC017030           |          AK014400             |
|                       |                              |          AK049365             |
|                       |                              |                               |
|                       |                              |                               |
|                       |                              |                               |
+-----------------------+------------------------------+-------------------------------+
|      #   57           | *Seq. mapped in strand Plus* | *Seq. mapped in strand Plus*  |
|                       |           AF176088           |          BC057919             |
|                       |           BC004556           |          AK051965             |
|                       |           AK022138           |          BC066141             |
|                       |                              |          BC066826             |
|                       |                              |          BC006874             |
|                       | *Seq. mapped in strand Minus*| *Seq. mapped in strand Minus* |
|                       |           AY358485           |          AK032108             |
|                       |           BC012191           |          AK053867             |
|                       |           AF171091           |          BC047209             |
|                       |           BC022849           |                               |
|                       |           CR613875           |                               |
+-----------------------+------------------------------+-------------------------------+
|      #   58           | *Seq. mapped in strand Plus* | *Seq. mapped in strand Plus*  |
|                       |           AK055855           |          AK020131             |
|                       |           BC010920           |          BC021770             |
|                       |             U89916           |          BC029019             |
|                       |           CR456845           |          AF124425             |
|                       |           AF339794           |          BC012238             |
|                       | *Seq. mapped in strand Minus*| *Seq. mapped in strand Minus* |
|                       |           AL049931           |          AK085147             |
|                       |           AK094042           |          BC060051             |
|                       |           AB023213           |          BC051931             |
|                       |           BC041804           |          AK032834             |
|                       |           CR606837           |          AK129256             |
|                       |           AF272347           |          AK013136             |
|                       |           BC018744           |          BC037016             |
|                       |           CR621496           |          AK011093             |
|                       |           BC011450           |          AK010997             |
|                       |           BC024972           |                               |
|                       |           AK130513           |                               |
|                       |           CR613825           |                               |
|                       |           AF339791           |                               |
|                       |           AK092363           |                               |
+-----------------------+------------------------------+-------------------------------+
|      #   59           | *Seq. mapped in strand Plus* | *Seq. mapped in strand Plus*  |
|                       |           BC022386           |          AK052623             |
|                       |           AB033549           |            D88533             |
|                       |           AK091932           |          BC013441             |
|                       |             M95548           |                               |
|                       |             L11696           |                               |
|                       |           AK092000           |                               |
|                       | *Seq. mapped in strand Minus*| *Seq. mapped in strand Minus* |
|                       |           AB007896           |          AK013363             |
|                       |           CR594385           |          BC004612             |
|                       |           AK025649           |          AK083070             |
+-----------------------+------------------------------+-------------------------------+
|      #   60           | *Seq. mapped in strand Plus* | *Seq. mapped in strand Plus*  |
|                       |           BC003159           |            D83999             |
|                       |           CR599625           |          BC002023             |
|                       |           CR620330           |                               |
|                       |             J05448           |                               |
|                       |           BC028157           |                               |
|                       |           AJ224144           |                               |
|                       |           AJ224143           |                               |
|                       |           CR600374           |                               |
|                       |           CR615542           |                               |
|                       |           CR600258           |                               |
|                       |           CR606923           |                               |
|                       |           BC000409           |                               |
|                       |           CR620622           |                               |
|                       |           CR542041           |                               |
|                       |           AF008443           |                               |
|                       |           CR594917           |                               |
|                       |           CR615834           |                               |
|                       |           CR626527           |                               |
|                       | *Seq. mapped in strand Minus*| *Seq. mapped in strand Minus* |
|                       |           CR623999           |          AK077612             |
|                       |           CR610258           |          BC004705             |
|                       |           BC001540           |          AF418207             |
|                       |           BC003541           |          AK032352             |
|                       |           CR608867           |                               |
|                       |           CR619149           |                               |
|                       |           AK001350           |                               |
|                       |           AF466369           |                               |
+-----------------------+------------------------------+-------------------------------+
|      #   61           | *Seq. mapped in strand Plus* | *Seq. mapped in strand Plus*  |
|                       |           AL834339           |          AK021133             |
|                       |           CR749429           |          AK041147             |
|                       |           CR593164           |          AK004797             |
|                       |           BC011268           |          BC003231             |
|                       |           AK054956           |          BC066835             |
|                       |           CR611792           |                               |
|                       |           AL365474           |                               |
|                       |           AY358162           |                               |
|                       |           AF085843           |                               |
|                       | *Seq. mapped in strand Minus*| *Seq. mapped in strand Minus* |
|                       |           BC036453           |          AK029522             |
|                       |           BC064431           |          AK034448             |
|                       |             U79304           |          BC027081             |
|                       |                              |          AK032166             |
|                       |                              |          AK014946             |
+-----------------------+------------------------------+-------------------------------+
|      #   62           | *Seq. mapped in strand Plus* | *Seq. mapped in strand Plus*  |
|                       |           AJ278577           |          AJ278576             |
|                       |           AJ278578           |          BC068124             |
|                       |           AK074529           |          AB041664             |
|                       |           AK075022           |          AK075586             |
|                       |           BC000195           |          BC023188             |
|                       |           AK075237           |                               |
|                       |           AF497245           |                               |
|                       |           AF151839           |                               |
|                       |           CR615766           |                               |
|                       |           AK025565           |                               |
|                       |           BX647201           |                               |
|                       | *Seq. mapped in strand Minus*| *Seq. mapped in strand Minus* |
|                       |           BC017844           |          AK079126             |
|                       |           AJ223183           |          AK008836             |
|                       |                              |          AJ278575             |
|                       |                              |          AK040569             |
|                       |                              |          BC057951             |
+-----------------------+------------------------------+-------------------------------+
|      #   63           | *Seq. mapped in strand Plus* | *Seq. mapped in strand Plus*  |
|                       |           AK130002           |          AK044371             |
|                       |           AK122992           |          AK028450             |
|                       |           CR594662           |          AK035819             |
|                       |           CR613644           |          BC027341             |
|                       |           CR602879           |          BC057062             |
|                       |           CR605235           |          AK052633             |
|                       |           CR605813           |          AK044750             |
|                       |           CR622691           |                               |
|                       |           CR605818           |                               |
|                       |           CR605885           |                               |
|                       |           AF378118           |                               |
|                       |           CR595990           |                               |
|                       |           CR596988           |                               |
|                       |             M13519           |                               |
|                       |           CR593680           |                               |
|                       |           CR601840           |                               |
|                       |           CR626674           |                               |
|                       |           CR626531           |                               |
|                       |           CR591164           |                               |
|                       |           CR626778           |                               |
|                       |           CR618468           |                               |
|                       |           CR606849           |                               |
|                       |           AK130375           |                               |
|                       |           BC017378           |                               |
|                       |           CR612759           |                               |
|                       |           CR620268           |                               |
|                       |           CR608425           |                               |
|                       |           CR615381           |                               |
|                       |           CR605256           |                               |
|                       |           CR611028           |                               |
|                       |           CR613188           |                               |
|                       |           CR609405           |                               |
|                       |           CR624982           |                               |
|                       |           BT009919           |                               |
|                       |           CR625484           |                               |
|                       |           CR606930           |                               |
|                       |             M19735           |                               |
|                       |           CR611913           |                               |
|                       |           CR602387           |                               |
|                       |           CR598936           |                               |
|                       |           CR624261           |                               |
|                       |             M34906           |                               |
|                       |             D10518           |                               |
|                       |             D10520           |                               |
|                       |           CR614304           |                               |
|                       |           CR610802           |                               |
|                       |           CR601804           |                               |
|                       | *Seq. mapped in strand Minus*| *Seq. mapped in strand Minus* |
|                       |           AF111808           |            Y00964             |
|                       |           BC015712           |            U07633             |
|                       |           BC030612           |                               |
|                       |           AF367997           |                               |
|                       |           CR607096           |                               |
|                       |           CR592399           |                               |
|                       |           AK025314           |                               |
+-----------------------+------------------------------+-------------------------------+
|      #   64           | *Seq. mapped in strand Plus* | *Seq. mapped in strand Plus*  |
|                       |           BC000381           |          AJ549317             |
|                       |           AF136570           |          AK028970             |
|                       |           CR593032           |          AK031659             |
|                       |           AB020881           |          AK035972             |
|                       |           BC017559           |          AK028621             |
|                       |           CR596561           |                               |
|                       | *Seq. mapped in strand Minus*| *Seq. mapped in strand Minus* |
|                       |           AL833602           |          AK078547             |
|                       |           BX648889           |          AK049975             |
|                       |           AK056554           |          BC033926             |
|                       |           AY046419           |          AB017697             |
|                       |           AK122628           |          AK005604             |
|                       |           BC070149           |                               |
+-----------------------+------------------------------+-------------------------------+
|      #   65           | *Seq. mapped in strand Plus* | *Seq. mapped in strand Plus*  |
|                       |           AB040882           |          BC050106             |
|                       |           BC026353           |          AK031244             |
|                       |           AK025513           |          AK018396             |
|                       |           BC037168           |          BC062967             |
|                       |           AF468833           |          AK043970             |
|                       |           AL832926           |          AK035273             |
|                       |           AK074278           |          AK012599             |
|                       |           CR595795           |          AK030041             |
|                       |           AL162064           |          AK033631             |
|                       |           BX649170           |          AK128953             |
|                       |           BC012861           |          BC048155             |
|                       |           AJ487015           |          BC006679             |
|                       |           BC062621           |          BC040795             |
|                       |           AK022483           |                               |
|                       |           BC039691           |                               |
|                       |           AK093313           |                               |
|                       |           AK098528           |                               |
|                       |           BX647142           |                               |
|                       | *Seq. mapped in strand Minus*| *Seq. mapped in strand Minus* |
|                       |           AK001574           |          AK017293             |
|                       |           AJ409349           |          BC012251             |
|                       |           AK124755           |                               |
|                       |           AK091168           |                               |
|                       |           BC064624           |                               |
|                       |           BC008928           |                               |
|                       |           AK057043           |                               |
|                       |           CR603340           |                               |
|                       |           BC075854           |                               |
+-----------------------+------------------------------+-------------------------------+
|      #   66           | *Seq. mapped in strand Plus* | *Seq. mapped in strand Plus*  |
|                       |           CR610281           |          AK019657             |
|                       |           CR615534           |          BC024437             |
|                       |           CR602193           |          BC042214             |
|                       |           CR594502           |            Z30970             |
|                       |             U14394           |            L27424             |
|                       |           CR616306           |          AK128943             |
|                       |           CR615393           |          AK083905             |
|                       |           CR620436           |          AB041611             |
|                       |           CR595938           |          BC014713             |
|                       |           CR625842           |            L19622             |
|                       |           CR625951           |          AY622855             |
|                       |           CR596287           |          AK048207             |
|                       |           CR625847           |            Z31358             |
|                       |           CR593019           |          AK084069             |
|                       |           CR617989           |                               |
|                       |           CR604843           |                               |
|                       |           CR594958           |                               |
|                       |           CR597142           |                               |
|                       |           CR613766           |                               |
|                       |           CR603101           |                               |
|                       |           CR621505           |                               |
|                       |             U02571           |                               |
|                       |           CR605518           |                               |
|                       |           CR595883           |                               |
|                       |           CR593651           |                               |
|                       |           CR607327           |                               |
|                       |             S78453           |                               |
|                       |             U67195           |                               |
|                       |           CR623084           |                               |
|                       |           BC014277           |                               |
|                       |             X76227           |                               |
|                       |           CR456593           |                               |
|                       |           CR609852           |                               |
|                       |           CR593372           |                               |
|                       |           BT006848           |                               |
|                       |             L15078           |                               |
|                       |           CR608290           |                               |
|                       |             D45917           |                               |
|                       |             X80791           |                               |
|                       |           CR619079           |                               |
|                       |           CR624342           |                               |
|                       |           CR620838           |                               |
|                       |           AK098517           |                               |
|                       |           CR613128           |                               |
|                       |           CR613064           |                               |
|                       |           CR613233           |                               |
|                       |           AF035289           |                               |
|                       |           CR590317           |                               |
|                       |           CR617133           |                               |
|                       |           CR591532           |                               |
|                       |           CR609959           |                               |
|                       | *Seq. mapped in strand Minus*| *Seq. mapped in strand Minus* |
|                       |           AF046873           |          AK080557             |
|                       |           CR456589           |          AF192754             |
|                       |           BC075065           |          AF498252             |
|                       |           BC075066           |          AF498253             |
|                       |                              |          AK047590             |
|                       |                              |                               |
|                       |                              |                               |
|                       |                              |                               |
|                       |                              |                               |
|                       |                              |                               |
|                       |                              |                               |
|                       |                              |                               |
|                       |                              |                               |
|                       |                              |                               |
+-----------------------+------------------------------+-------------------------------+
|      #   67           | *Seq. mapped in strand Plus* | *Seq. mapped in strand Plus*  |
|                       |           BX648657           |          AK019633             |
|                       |           AL833327           |          BC050751             |
|                       |           AL833328           |          AK006656             |
|                       |           AL833336           |                               |
|                       |           AK057238           |                               |
|                       |           BC024009           |                               |
|                       |           AK058059           |                               |
|                       |           AL137334           |                               |
|                       |           AK131102           |                               |
|                       |           AK093046           |                               |
|                       |           BC033491           |                               |
|                       |           AF447586           |                               |
|                       |           AK093523           |                               |
|                       | *Seq. mapped in strand Minus*| *Seq. mapped in strand Minus* |
|                       |             L39059           |          BC058383             |
|                       |           BC028131           |          BC051944             |
|                       |           CR605319           |          AK087534             |
|                       |           AK097031           |            Y09974             |
|                       |           AK095331           |          AK030522             |
|                       |           AK123582           |                               |
+-----------------------+------------------------------+-------------------------------+
|      #   68           | *Seq. mapped in strand Plus* | *Seq. mapped in strand Plus*  |
|                       |           CR603000           |          AK077339             |
|                       |           AK122792           |          AK037364             |
|                       |           BX648450           |          AK047686             |
|                       |           AL049667           |          BC055955             |
|                       |           AK125601           |                               |
|                       |           CR609673           |                               |
|                       |           AK126139           |                               |
|                       |           AK092905           |                               |
|                       | *Seq. mapped in strand Minus*| *Seq. mapped in strand Minus* |
|                       |           AK022798           |          BC036561             |
|                       |           AK057807           |          AK031400             |
|                       |           BC039719           |          AY383616             |
|                       |           CR599983           |          AK041994             |
|                       |           CR598637           |                               |
+-----------------------+------------------------------+-------------------------------+
|      #   69           | *Seq. mapped in strand Plus* | *Seq. mapped in strand Plus*  |
|                       |           BC011570           |          AK079734             |
|                       |           BC032581           |          AK033393             |
|                       |           BC065284           |          AK032383             |
|                       |           AY358617           |          AK030479             |
|                       |           AF312024           |          AK014600             |
|                       |           AY010111           |          BC003967             |
|                       |           AK074160           |          AK131118             |
|                       |           AB058715           |                               |
|                       |           AL122081           |                               |
|                       |           AK093818           |                               |
|                       |           AB053445           |                               |
|                       | *Seq. mapped in strand Minus*| *Seq. mapped in strand Minus* |
|                       |           BC071608           |          AK039126             |
|                       |           AK074172           |          AF308939             |
|                       |           AK123442           |          AY026062             |
|                       |           AK024449           |          AK016015             |
|                       |           AF193048           |                               |
|                       |           BC020568           |                               |
|                       |           AY265805           |                               |
|                       |           CR611971           |                               |
|                       |           AY358379           |                               |
|                       |           CR598635           |                               |
|                       |           CR595858           |                               |
|                       |           CR604197           |                               |
|                       |           CR621313           |                               |
|                       |           CR610409           |                               |
|                       |           CR626183           |                               |
|                       |           CR599757           |                               |
|                       |           CR612511           |                               |
|                       |           CR600781           |                               |
|                       |           CR614300           |                               |
|                       |           CR614217           |                               |
|                       |           CR624628           |                               |
+-----------------------+------------------------------+-------------------------------+
|      #   70           | *Seq. mapped in strand Plus* | *Seq. mapped in strand Plus*  |
|                       |           BC057234           |          BC031531             |
|                       |           AK023169           |          AK129350             |
|                       |           BC074728           |                               |
|                       |           AF169974           |                               |
|                       |           AY034081           |                               |
|                       |           CR457300           |                               |
|                       |           AL834378           |                               |
|                       | *Seq. mapped in strand Minus*| *Seq. mapped in strand Minus* |
|                       |           AK026565           |          AK079343             |
|                       |           BC019090           |          AK043738             |
+-----------------------+------------------------------+-------------------------------+
|      #   71           | *Seq. mapped in strand Plus* | *Seq. mapped in strand Plus*  |
|                       |           BX647755           |          AY282793             |
|                       |           BC035031           |            M74515             |
|                       |             D13318           |          BC052448             |
|                       |             U13044           |          AY282794             |
|                       |                              |          AY282795             |
|                       |                              |          BC013562             |
|                       |                              |          AY282796             |
|                       |                              |          AY282797             |
|                       |                              |          AY282798             |
|                       |                              |          AY282799             |
|                       | *Seq. mapped in strand Minus*| *Seq. mapped in strand Minus* |
|                       |             M73031           |            U77128             |
|                       |             M37104           |          BC010766             |
|                       |           AL110183           |          AK078484             |
|                       |           BC066310           |                               |
|                       |           BC001178           |                               |
|                       |           CR456950           |                               |
|                       |           BT007244           |                               |
+-----------------------+------------------------------+-------------------------------+
|      #   72           | *Seq. mapped in strand Plus* | *Seq. mapped in strand Plus*  |
|                       |           AK172808           |          AK019861             |
|                       |           CR611107           |            J05261             |
|                       |           CR596957           |          AK032197             |
|                       |           CR618673           |          BC018534             |
|                       |           CR621669           |                               |
|                       |           CR607706           |                               |
|                       |           BC000597           |                               |
|                       |           CR595889           |                               |
|                       |             M22960           |                               |
|                       |           CR600724           |                               |
|                       |           CR624913           |                               |
|                       |           CR626192           |                               |
|                       |           CR609128           |                               |
|                       |           AK097786           |                               |
|                       |           CR609734           |                               |
|                       |           CR626418           |                               |
|                       |           CR605948           |                               |
|                       |           CR590686           |                               |
|                       |           CR608418           |                               |
|                       |           AK055177           |                               |
|                       |           CR625597           |                               |
|                       |           AF086236           |                               |
|                       | *Seq. mapped in strand Minus*| *Seq. mapped in strand Minus* |
|                       |           AJ295985           |            U37226             |
|                       |           AK054821           |          BC003782             |
|                       |           BC074737           |            U28960             |
|                       |           CR597431           |                               |
|                       |           BC019898           |                               |
|                       |           AF052157           |                               |
|                       |           AB076694           |                               |
|                       |           CR611165           |                               |
|                       |           CR591380           |                               |
|                       |           BC005045           |                               |
|                       |           BC019847           |                               |
|                       |           AK092397           |                               |
|                       |             L26232           |                               |
|                       |           CR621173           |                               |
|                       |           AK097009           |                               |
|                       |           CR599330           |                               |
|                       |           CR608163           |                               |
|                       |           CR621817           |                               |
|                       |           CR600678           |                               |
|                       |           CR613728           |                               |
|                       |           CR597556           |                               |
|                       |           CR593911           |                               |
|                       |           CR611353           |                               |
|                       |           CR616299           |                               |
|                       |           CR590475           |                               |
|                       |           CR625284           |                               |
|                       |           CR593296           |                               |
|                       |           CR611922           |                               |
|                       |           CR606867           |                               |
|                       |           CR601132           |                               |
|                       |           CR604838           |                               |
|                       |           CR614849           |                               |
|                       |           CR604806           |                               |
|                       |           CR610375           |                               |
|                       |           CR620390           |                               |
|                       |           CR614247           |                               |
|                       |           CR609091           |                               |
|                       |           CR590500           |                               |
|                       |           CR599059           |                               |
|                       |           CR601380           |                               |
|                       |           CR618175           |                               |
|                       |           CR593086           |                               |
|                       |           CR599835           |                               |
|                       |           CR617131           |                               |
|                       |           CR618641           |                               |
|                       |           CR600343           |                               |
|                       |           CR615147           |                               |
|                       |           CR608376           |                               |
|                       |           CR617304           |                               |
|                       |           CR603115           |                               |
|                       |           CR618178           |                               |
|                       |           CR595598           |                               |
|                       |           CR592030           |                               |
|                       |           CR603856           |                               |
|                       |           CR614141           |                               |
|                       |           CR595084           |                               |
|                       |           CR608777           |                               |
|                       |           CR617010           |                               |
|                       |           CR603810           |                               |
|                       |           CR598231           |                               |
|                       |           CR612436           |                               |
|                       |           CR613720           |                               |
|                       |           CR612193           |                               |
|                       |           CR596763           |                               |
|                       |           CR604318           |                               |
|                       |           CR617251           |                               |
|                       |           CR609895           |                               |
|                       |           AK092523           |                               |
|                       |           CR610029           |                               |
+-----------------------+------------------------------+-------------------------------+
|      #   73           | *Seq. mapped in strand Plus* | *Seq. mapped in strand Plus*  |
|                       |           AB058723           |          BC049785             |
|                       |           AY172136           |            D29801             |
|                       |           CR590729           |          AK129449             |
|                       |           BC021209           |          AK013909             |
|                       |           AJ271790           |                               |
|                       |           AL834468           |                               |
|                       |           AL834486           |                               |
|                       |           AL133649           |                               |
|                       | *Seq. mapped in strand Minus*| *Seq. mapped in strand Minus* |
|                       |           AF467442           |          AK029606             |
|                       |             S66168           |          AK089395             |
|                       |                              |          BC056922             |
|                       |                              |          AK052628             |
+-----------------------+------------------------------+-------------------------------+
|      #   74           | *Seq. mapped in strand Plus* | *Seq. mapped in strand Plus*  |
|                       |           AK096995           |          AK053299             |
|                       |           CR620783           |          AK035483             |
|                       |           AK055665           |          AK031216             |
|                       |           AF262032           |                               |
|                       |           BC009983           |                               |
|                       |           AF370007           |                               |
|                       | *Seq. mapped in strand Minus*| *Seq. mapped in strand Minus* |
|                       |           AF467287           |          AK035118             |
|                       |             M83822           |          AK080563             |
|                       |           BX537552           |          AK082589             |
+-----------------------+------------------------------+-------------------------------+
|      #   75           | *Seq. mapped in strand Plus* | *Seq. mapped in strand Plus*  |
|                       |           AK122634           |          AB027142             |
|                       |           CR591803           |          AK075614             |
|                       |           BC005094           |          AK007803             |
|                       |           AK056816           |          BC021547             |
|                       |                              |          AK080538             |
|                       |                              |          AK002728             |
|                       |                              |          AB027141             |
|                       | *Seq. mapped in strand Minus*| *Seq. mapped in strand Minus* |
|                       |           AF086351           |          AK009760             |
|                       |           CR597258           |          AK010144             |
|                       |           AY358450           |          BC003309             |
|                       |           BC011575           |                               |
|                       |           BC036340           |                               |
|                       |           CR618333           |                               |
|                       |           AK075349           |                               |
|                       |           BC018757           |                               |
|                       |           CR615363           |                               |
|                       |           CR600990           |                               |
|                       |           CR607336           |                               |
|                       |           CR625850           |                               |
|                       |           CR624009           |                               |
|                       |           CR604091           |                               |
|                       |           CR597923           |                               |
|                       |           CR626023           |                               |
|                       |           CR625843           |                               |
|                       |           CR600472           |                               |
|                       |           CR606079           |                               |
|                       |           CR596478           |                               |
|                       |           CR595391           |                               |
|                       |           CR625752           |                               |
|                       |           CR608841           |                               |
|                       |           CR609055           |                               |
|                       |           CR590480           |                               |
|                       |           CR613020           |                               |
|                       |           CR591234           |                               |
|                       |           CR592268           |                               |
|                       |           CR613276           |                               |
|                       |           CR597089           |                               |
+-----------------------+------------------------------+-------------------------------+
|      #   76           | *Seq. mapped in strand Plus* | *Seq. mapped in strand Plus*  |
|                       |           AK090624           |          AK014376             |
|                       |           AL136921           |          AK129327             |
|                       |           AY569455           |          BC060720             |
|                       |           BC024267           |          BC059916             |
|                       |           CR595735           |          AK049987             |
|                       |                              |          AK048449             |
|                       |                              |          AK011460             |
|                       | *Seq. mapped in strand Minus*| *Seq. mapped in strand Minus* |
|                       |           AB037727           |          BC005649             |
|                       |           AF451977           |          BC008598             |
|                       |                              |          AK088431             |
|                       |                              |          AK080607             |
|                       |                              |          AK043912             |
|                       |                              |                               |
|                       |                              |                               |
+-----------------------+------------------------------+-------------------------------+
|      #   77           | *Seq. mapped in strand Plus* | *Seq. mapped in strand Plus*  |
|                       |           CR590826           |          BC011472             |
|                       |           CR594712           |          BC024420             |
|                       |           CR594497           |                               |
|                       |           AL035082           |                               |
|                       |           CR612711           |                               |
|                       |           BC001277           |                               |
|                       |           AK129888           |                               |
|                       |           AL035081           |                               |
|                       |           CR600149           |                               |
|                       |           CR456509           |                               |
|                       |           CR614427           |                               |
|                       |           CR613642           |                               |
|                       | *Seq. mapped in strand Minus*| *Seq. mapped in strand Minus* |
|                       |           CR594526           |          AK079442             |
|                       |           CR620697           |          BC012249             |
|                       |           AL080113           |          BC031802             |
|                       |           CR625919           |          BC027758             |
|                       |           BC045747           |          BC038378             |
|                       |           BC029553           |          AK011338             |
|                       |           CR608668           |          AK079294             |
|                       |           CR591397           |          AK027954             |
|                       |           BC000595           |                               |
|                       |           AK024985           |                               |
|                       |           AK074224           |                               |
|                       |           CR456432           |                               |
|                       |             U59321           |                               |
+-----------------------+------------------------------+-------------------------------+
|      #   78           | *Seq. mapped in strand Plus* | *Seq. mapped in strand Plus*  |
|                       |           CR624824           |            X63013             |
|                       |           CR612669           |                               |
|                       |           CR623024           |                               |
|                       |           BC015138           |                               |
|                       |           AK090918           |                               |
|                       |           AY203935           |                               |
|                       | *Seq. mapped in strand Minus*| *Seq. mapped in strand Minus* |
|                       |             S68531           |          BC045529             |
+-----------------------+------------------------------+-------------------------------+
|      #   79           | *Seq. mapped in strand Plus* | *Seq. mapped in strand Plus*  |
|                       |           BC068575           |          AK083684             |
|                       |           AY358299           |          AK012169             |
|                       |           AY166584           |          AJ458938             |
|                       |           BC013767           |                               |
|                       | *Seq. mapped in strand Minus*| *Seq. mapped in strand Minus* |
|                       |           AK094596           |          AK040408             |
|                       |           AK025674           |          AK018739             |
|                       |           AK126176           |          BC061006             |
+-----------------------+------------------------------+-------------------------------+
|      #   80           | *Seq. mapped in strand Plus* | *Seq. mapped in strand Plus*  |
|                       |           AK075373           |          BC011281             |
|                       |           BC031095           |                               |
|                       |           CR597195           |                               |
|                       |           AB019038           |                               |
|                       |           BC004402           |                               |
|                       |           AY359073           |                               |
|                       |           CR624559           |                               |
|                       |           CR592042           |                               |
|                       |           CR619322           |                               |
|                       | *Seq. mapped in strand Minus*| *Seq. mapped in strand Minus* |
|                       |           BC010084           |          BC051078             |
+-----------------------+------------------------------+-------------------------------+
|      #   81           | *Seq. mapped in strand Plus* | *Seq. mapped in strand Plus*  |
|                       |           BC047391           |          AK012888             |
|                       |           AK001106           |          AK027978             |
|                       |           AK098470           |                               |
|                       |           BC045691           |                               |
|                       | *Seq. mapped in strand Minus*| *Seq. mapped in strand Minus* |
|                       |           AL137687           |          AK029993             |
|                       |           AK096082           |          AK036803             |
|                       |           AF086266           |          AK019543             |
|                       |           AY358278           |          AK033549             |
|                       |           BC047670           |          BC052663             |
|                       |           BC050640           |          BC043132             |
|                       |           CR621065           |          AK007570             |
|                       |           AB056476           |          AF312924             |
|                       |           AF107253           |          AK029049             |
+-----------------------+------------------------------+-------------------------------+
|      #   82           | *Seq. mapped in strand Plus* | *Seq. mapped in strand Plus*  |
|                       |           CR619346           |          AK076201             |
|                       |           BC014991           |          BC014754             |
|                       |           CR612592           |          AK018694             |
|                       |           CR598824           |            U10420             |
|                       |           CR606356           |          AK088878             |
|                       |             L10752           |                               |
|                       |             M74905           |                               |
|                       |             S51033           |                               |
|                       |             M71215           |                               |
|                       |             X56528           |                               |
|                       |           CR600098           |                               |
|                       |             M99626           |                               |
|                       | *Seq. mapped in strand Minus*| *Seq. mapped in strand Minus* |
|                       |             X90857           |          AK090290             |
|                       |           AK125420           |          AK086378             |
|                       |           AK026990           |          AK036405             |
|                       |           BC004185           |          AK081902             |
|                       |           BC012825           |          AK036633             |
+-----------------------+------------------------------+-------------------------------+
|      #   83           | *Seq. mapped in strand Plus* | *Seq. mapped in strand Plus*  |
|                       |           BC067130           |          AB003494             |
|                       |           BC038448           |                               |
|                       |           BC011799           |                               |
|                       |           BC037561           |                               |
|                       |           CR623884           |                               |
|                       |           AF507907           |                               |
|                       |           AF521047           |                               |
|                       |           AF507909           |                               |
|                       |           AF507908           |                               |
|                       |           AF521049           |                               |
|                       |           AF452589           |                               |
|                       |           AF479317           |                               |
|                       |           AF521046           |                               |
|                       |           AF521045           |                               |
|                       | *Seq. mapped in strand Minus*| *Seq. mapped in strand Minus* |
|                       |             M54992           |            J04170             |
|                       |           BC011734           |          BC003824             |
|                       |           BC030227           |          AF543213             |
|                       |           AF283777           |                               |
|                       |           CR589985           |                               |
+-----------------------+------------------------------+-------------------------------+
|      #   84           | *Seq. mapped in strand Plus* | *Seq. mapped in strand Plus*  |
|                       |           BC021999           |          AK044738             |
|                       |           AL834188           |          AF454947             |
|                       |           AB053309           |                               |
|                       |           AB053310           |                               |
|                       |           AY032876           |                               |
|                       |           BC033777           |                               |
|                       |           AF454946           |                               |
|                       |           AK025232           |                               |
|                       | *Seq. mapped in strand Minus*| *Seq. mapped in strand Minus* |
|                       |           AF242546           |          BC052386             |
|                       |           BC008082           |          AK016611             |
+-----------------------+------------------------------+-------------------------------+
|      #   85           | *Seq. mapped in strand Plus* | *Seq. mapped in strand Plus*  |
|                       |             X05615           |            U76389             |
|                       |             X02154           |          AF076186             |
|                       |             S40807           |          AF076187             |
|                       |             S80793           |                               |
|                       |           AK023948           |                               |
|                       |           AK023852           |                               |
|                       | *Seq. mapped in strand Minus*| *Seq. mapped in strand Minus* |
|                       |           CR617946           |          AJ131777             |
|                       |             D89077           |          BC032922             |
|                       |           BX648382           |          AK036167             |
|                       |           CR601997           |          AK037901             |
|                       |           CR594017           |          AK041565             |
|                       |           BX647569           |            U29056             |
|                       |             U44403           |                               |
|                       |           BC007042           |                               |
|                       |           CR617843           |                               |
|                       |             U30473           |                               |
|                       |           CR536537           |                               |
|                       |           AJ238591           |                               |
|                       |           AK024366           |                               |
+-----------------------+------------------------------+-------------------------------+
|      #   86           | *Seq. mapped in strand Plus* | *Seq. mapped in strand Plus*  |
|                       |           CR616059           |          BC046510             |
|                       |           BC013035           |          BC035532             |
|                       |           CR619782           |          AK087507             |
|                       |                              |          AF196476             |
|                       | *Seq. mapped in strand Minus*| *Seq. mapped in strand Minus* |
|                       |           BC028600           |          BC048089             |
|                       |             L20852           |                               |
|                       |           CR600416           |                               |
+-----------------------+------------------------------+-------------------------------+
|      #   87           | *Seq. mapped in strand Plus* | *Seq. mapped in strand Plus*  |
|                       |           AB161944           |          AK010929             |
|                       |           AK091278           |          AK037791             |
|                       |           BX538203           |          AK031116             |
|                       |           AK001403           |          AF091432             |
|                       |           BC064915           |          BC053727             |
|                       |           BC050536           |          AF106691             |
|                       |           AK000537           |          AK011883             |
|                       | *Seq. mapped in strand Minus*| *Seq. mapped in strand Minus* |
|                       |           AF091433           |          AK049513             |
|                       |           BC007015           |          AK039325             |
|                       |           AF102778           |          BC024524             |
|                       |           AF106690           |          BC060149             |
|                       |           AF112857           |          AK041151             |
|                       |           BC020729           |          BC050192             |
|                       |                              |          BC049092             |
+-----------------------+------------------------------+-------------------------------+
|      #   88           | *Seq. mapped in strand Plus* | *Seq. mapped in strand Plus*  |
|                       |           AY273894           |          BC007126             |
|                       |             U22055           |          AB021491             |
|                       |           BC017180           |          AK088028             |
|                       |           AK096583           |                               |
|                       |           BT009785           |                               |
|                       |           CR614603           |                               |
|                       |           CR614491           |                               |
|                       |           BC020423           |                               |
|                       | *Seq. mapped in strand Minus*| *Seq. mapped in strand Minus* |
|                       |           BC036685           |          AF290542             |
|                       |           AF196976           |          AK046781             |
|                       |           AK172751           |          BC012209             |
|                       |           AY358307           |          AF300458             |
|                       |           AJ297858           |                               |
+-----------------------+------------------------------+-------------------------------+
|      #   89           | *Seq. mapped in strand Plus* | *Seq. mapped in strand Plus*  |
|                       |           AK131078           |          AK007114             |
|                       |           BX640820           |                               |
|                       |           AK097276           |                               |
|                       |           BX538312           |                               |
|                       |           AK123054           |                               |
|                       |           BC035364           |                               |
|                       |           AK096856           |                               |
|                       |           BC040614           |                               |
|                       |           AK057128           |                               |
|                       |           BC030715           |                               |
|                       |           AK091226           |                               |
|                       |           CR614431           |                               |
|                       |             L27586           |                               |
|                       |           BC051670           |                               |
|                       | *Seq. mapped in strand Minus*| *Seq. mapped in strand Minus* |
|                       |           BC033386           |          AK020425             |
|                       |           CR613504           |          AK131114             |
|                       |           CR602083           |          AK042220             |
|                       |           AK024433           |          AK013196             |
|                       |           BC003590           |          BC022953             |
|                       |           CR607348           |          AK004037             |
|                       |           AK024702           |                               |
+-----------------------+------------------------------+-------------------------------+
|      #   90           | *Seq. mapped in strand Plus* | *Seq. mapped in strand Plus*  |
|                       |           AK091123           |          AK086176             |
|                       |           AK002138           |          BC029102             |
|                       |           AF322642           |          AF363457             |
|                       |           BC018142           |          BC004692             |
|                       |           AY032927           |          AK028888             |
|                       |           BC001326           |                               |
|                       |           AK054723           |                               |
|                       | *Seq. mapped in strand Minus*| *Seq. mapped in strand Minus* |
|                       |           AL832796           |          AK034220             |
|                       |           AL833696           |          BC028782             |
|                       |           AK095969           |          AK076267             |
|                       |           BC047318           |          AK052266             |
|                       |             U30894           |          AK128926             |
+-----------------------+------------------------------+-------------------------------+
|      #   91           | *Seq. mapped in strand Plus* | *Seq. mapped in strand Plus*  |
|                       |           AK024794           |          AK016319             |
|                       |           CR622831           |          AK030845             |
|                       |           CR620909           |          AB041657             |
|                       |           AF061731           |          AK007261             |
|                       |           BC056255           |          BC011283             |
|                       |           BC061637           |                               |
|                       |           CR611802           |                               |
|                       |           AK000678           |                               |
|                       |           CR607655           |                               |
|                       |           BC009645           |                               |
|                       | *Seq. mapped in strand Minus*| *Seq. mapped in strand Minus* |
|                       |           AL050382           |          AK122342             |
|                       |           AF124727           |          BC052755             |
|                       |           AF124728           |          AF124725             |
|                       |           AL833228           |          AF124729             |
|                       |           BC032770           |          AF168782             |
+-----------------------+------------------------------+-------------------------------+
|      #   92           | *Seq. mapped in strand Plus* | *Seq. mapped in strand Plus*  |
|                       |           BC058898           |          AK010105             |
|                       |           CR597196           |          AK014120             |
|                       |           BC034253           |          AK019438             |
|                       |           AK024759           |          AK009813             |
|                       |           BC058847           |          BC011312             |
|                       |           BC075793           |          AK029940             |
|                       |                              |          AK031032             |
|                       | *Seq. mapped in strand Minus*| *Seq. mapped in strand Minus* |
|                       |           AK074564           |          AK048316             |
|                       |           BC029503           |          AK006596             |
|                       |                              |          AK077003             |
|                       |                              |          AK077188             |
|                       |                              |          AK035291             |
|                       |                              |          AK077957             |
|                       |                              |          BC010483             |
+-----------------------+------------------------------+-------------------------------+
|      #   93           | *Seq. mapped in strand Plus* | *Seq. mapped in strand Plus*  |
|                       |           CR600382           |          BC057917             |
|                       |           AK129986           |          BC042199             |
|                       |           AK129795           |          AK004688             |
|                       |           CR625371           |          BC046829             |
|                       |           CR598452           |                               |
|                       |             U39400           |                               |
|                       |           BC004378           |                               |
|                       |           AB062395           |                               |
|                       |           CR593043           |                               |
|                       |           AK123252           |                               |
|                       |           CR602808           |                               |
|                       | *Seq. mapped in strand Minus*| *Seq. mapped in strand Minus* |
|                       |           BC013860           |          AK014723             |
|                       |           BC035473           |          BC028956             |
|                       |           BC030530           |          AK077397             |
|                       |           AL834262           |          AK049794             |
+-----------------------+------------------------------+-------------------------------+
|      #   94           | *Seq. mapped in strand Plus* | *Seq. mapped in strand Plus*  |
|                       |           AK127073           |          AK046260             |
|                       |           BC040547           |          AK046222             |
|                       |           AK054846           |          AK045995             |
|                       |           CR601310           |          AK040216             |
|                       |           AK098068           |          BC050757             |
|                       |           BC016615           |          AK039716             |
|                       |           AK057069           |          AK050483             |
|                       |                              |          AF233582             |
|                       | *Seq. mapped in strand Minus*| *Seq. mapped in strand Minus* |
|                       |           AF251706           |          BC057864             |
|                       |           AY358545           |          AF251703             |
|                       |           AK092757           |          AY214460             |
|                       |           BC028199           |          AY457047             |
|                       |           AF375480           |          AK089313             |
|                       |           AF375481           |          AK089465             |
|                       |           AY303545           |                               |
+-----------------------+------------------------------+-------------------------------+
|      #   95           | *Seq. mapped in strand Plus* | *Seq. mapped in strand Plus*  |
|                       |           CR599104           |          AK008766             |
|                       |           CR609872           |          AK019134             |
|                       |           AK093481           |          BC024944             |
|                       |           AK094065           |          AK007340             |
|                       |           AK125337           |                               |
|                       |           BC009911           |                               |
|                       |           CR616716           |                               |
|                       |           CR598018           |                               |
|                       |           CR607362           |                               |
|                       |           AL713670           |                               |
|                       |           AY359092           |                               |
|                       |           AK122692           |                               |
|                       |           AK025971           |                               |
|                       |           AL137381           |                               |
|                       |           AK093622           |                               |
|                       | *Seq. mapped in strand Minus*| *Seq. mapped in strand Minus* |
|                       |           CR612055           |          BC016075             |
|                       |           AF401217           |          AK075599             |
|                       |           AK000486           |          AK005344             |
|                       |           BC000009           |                               |
|                       |           AF161404           |                               |
|                       |           AJ293309           |                               |
|                       |           BC006387           |                               |
|                       |           AF401219           |                               |
|                       |           CR457238           |                               |
+-----------------------+------------------------------+-------------------------------+
|      #   96           | *Seq. mapped in strand Plus* | *Seq. mapped in strand Plus*  |
|                       |           BC012114           |          AY112937             |
|                       |           AY114157           |          BC021431             |
|                       |           AF074382           |          AF513109             |
|                       |           BC050612           |          AK037020             |
|                       |           CR600010           |          AF069542             |
|                       |           CR604149           |                               |
|                       |           CR603029           |                               |
|                       |           BC000299           |                               |
|                       |           AK000593           |                               |
|                       |           BC046922           |                               |
|                       |           AF062089           |                               |
|                       |           AF261086           |                               |
|                       |           AF091453           |                               |
|                       |           AY429548           |                               |
|                       |           AY429547           |                               |
|                       |           AY429546           |                               |
|                       | *Seq. mapped in strand Minus*| *Seq. mapped in strand Minus* |
|                       |           BC000337           |            Z11911             |
|                       |             X03674           |          AK088135             |
|                       |             M21248           |          BC075663             |
|                       |           CR606589           |                               |
|                       |           CR624380           |                               |
|                       |             S58359           |                               |
|                       |             M27940           |                               |
+-----------------------+------------------------------+-------------------------------+
|      #   97           | *Seq. mapped in strand Plus* | *Seq. mapped in strand Plus*  |
|                       |           BC007404           |          AK003537             |
|                       |           BC007992           |          BC022666             |
|                       |             U29725           |          AK075659             |
|                       |             U29726           |                               |
|                       |           BC030134           |                               |
|                       |           BC009963           |                               |
|                       |             U25278           |                               |
|                       |           AY534741           |                               |
|                       |           CR604533           |                               |
|                       |           CR592256           |                               |
|                       | *Seq. mapped in strand Minus*| *Seq. mapped in strand Minus* |
|                       |           CR606056           |          AB019373             |
|                       |           AB030506           |          AY534740             |
|                       |           BC002944           |          BC033598             |
|                       |           BC035560           |          AF126159             |
|                       |           CR598399           |          AF126160             |
|                       |           CR617970           |          AF126161             |
|                       |           CR613174           |          BC013745             |
|                       |           CR599644           |          BC051020             |
|                       |           CR597747           |          BC058096             |
|                       |           CR604850           |                               |
|                       |           CR599684           |                               |
|                       |           CR599896           |                               |
|                       |             L38486           |                               |
|                       |           CR605610           |                               |
|                       |           CR625505           |                               |
|                       |           CR620840           |                               |
|                       |           CR604299           |                               |
|                       |           CR620310           |                               |
|                       |           CR595863           |                               |
|                       |           CR610875           |                               |
|                       |           CR621699           |                               |
|                       |           CR611587           |                               |
|                       |           CR592740           |                               |
|                       |           BC062415           |                               |
|                       |           BC047725           |                               |
|                       |           CR594997           |                               |
+-----------------------+------------------------------+-------------------------------+
|      #   98           | *Seq. mapped in strand Plus* | *Seq. mapped in strand Plus*  |
|                       |             S83182           |          AK128915             |
|                       |             D49742           |          BC031775             |
|                       |           BC031412           |                               |
|                       | *Seq. mapped in strand Minus*| *Seq. mapped in strand Minus* |
|                       |           BX641052           |            U76618             |
|                       |           AF542553           |          AY177622             |
|                       |           AY081943           |          AY177623             |
|                       |           AL832457           |                               |
|                       |           BX640682           |                               |
|                       |           AK056605           |                               |
|                       |           AL831980           |                               |
|                       |           AY177620           |                               |
|                       |           AY177621           |                               |
|                       |           AK096886           |                               |
|                       |           AK096875           |                               |
|                       |           AL832025           |                               |
|                       |           AL832330           |                               |
|                       |           BX647658           |                               |
|                       |           BX640730           |                               |
|                       |           AK056969           |                               |
|                       |           BC005933           |                               |
|                       |           BC017866           |                               |
|                       |           BC058825           |                               |
|                       |           BC070130           |                               |
+-----------------------+------------------------------+-------------------------------+
|      #   99           | *Seq. mapped in strand Plus* | *Seq. mapped in strand Plus*  |
|                       |           AL831995           |          AK015716             |
|                       |           BC013437           |          AK033681             |
|                       |             X68504           |          AK039294             |
|                       |             X63381           |          BC057459             |
|                       |             Y16312           |                               |
|                       |             X68505           |                               |
|                       |           BC053871           |                               |
|                       |             X68503           |                               |
|                       | *Seq. mapped in strand Minus*| *Seq. mapped in strand Minus* |
|                       |           AK056718           |          AK040263             |
|                       |           BX640928           |          AK084623             |
|                       |           AK057570           |          AK043067             |
|                       |           AK124852           |          AK047233             |
+-----------------------+------------------------------+-------------------------------+
|      #  100           | *Seq. mapped in strand Plus* | *Seq. mapped in strand Plus*  |
|                       |           BC001777           |          BC019563             |
|                       |             D16593           |          AK008935             |
|                       |                              |          AK007525             |
|                       |                              |          AK009830             |
|                       | *Seq. mapped in strand Minus*| *Seq. mapped in strand Minus* |
|                       |           CR596164           |          BC058588             |
|                       |           BC001418           |          BC049607             |
|                       |           BC013953           |          AF326552             |
|                       |           BC023260           |          AF326551             |
|                       |           AY027543           |                               |
|                       |           AB012692           |                               |
|                       |           AY359883           |                               |
+-----------------------+------------------------------+-------------------------------+
|      #  101           | *Seq. mapped in strand Plus* | *Seq. mapped in strand Plus*  |
|                       |           AK021536           |          AK079437             |
|                       |           AK098762           |                               |
|                       |           AY223837           |                               |
|                       |           CR591683           |                               |
|                       |           CR625821           |                               |
|                       |           CR613800           |                               |
|                       |           BC052292           |                               |
|                       |           CR599764           |                               |
|                       |           CR604093           |                               |
|                       |           CR596651           |                               |
|                       |           CR621798           |                               |
|                       |           BC001313           |                               |
|                       |           BC020499           |                               |
|                       |           AK022227           |                               |
|                       |           CR610557           |                               |
|                       |           CR596334           |                               |
|                       |           CR596002           |                               |
|                       |           CR618215           |                               |
|                       |           CR613836           |                               |
|                       |           CR605693           |                               |
|                       |           CR610748           |                               |
|                       |           BC017119           |                               |
|                       |           CR605614           |                               |
|                       |           CR599293           |                               |
|                       |           CR617593           |                               |
|                       |           CR602887           |                               |
|                       |           CR610797           |                               |
|                       |           AF195883           |                               |
|                       |           CR603025           |                               |
|                       |           AK123195           |                               |
|                       | *Seq. mapped in strand Minus*| *Seq. mapped in strand Minus* |
|                       |           BC039154           |          BC024545             |
+-----------------------+------------------------------+-------------------------------+
|      #  102           | *Seq. mapped in strand Plus* | *Seq. mapped in strand Plus*  |
|                       |           AF213666           |          AK011478             |
|                       |           AF213667           |          BC031380             |
|                       |           AF213668           |          BC038004             |
|                       |           BC010689           |            U43607             |
|                       |           AK000150           |          AF213670             |
|                       |           CR590342           |          AF213671             |
|                       |           AF203978           |          AF213672             |
|                       |           BT009812           |            U43548             |
|                       |           CR606467           |          AK013973             |
|                       | *Seq. mapped in strand Minus*| *Seq. mapped in strand Minus* |
|                       |             U18921           |          BC030169             |
|                       |           AK126369           |          AB000121             |
|                       |           AB030304           |                               |
|                       |           BC008792           |                               |
|                       |           CR594795           |                               |
|                       |           CR625144           |                               |
|                       |           AF440240           |                               |
+-----------------------+------------------------------+-------------------------------+
|      #  103           | *Seq. mapped in strand Plus* | *Seq. mapped in strand Plus*  |
|                       |           BC005064           |          BC053519             |
|                       |           BC028146           |          AK005307             |
|                       |           AF320622           |          AK005362             |
|                       |           AF416712           |                               |
|                       |           AK094091           |                               |
|                       |           CR623978           |                               |
|                       | *Seq. mapped in strand Minus*| *Seq. mapped in strand Minus* |
|                       |           BC011782           |          AY013699             |
|                       |           AF254795           |          BC051227             |
|                       |             M28210           |                               |
|                       |           AF498931           |                               |
+-----------------------+------------------------------+-------------------------------+
|      #  104           | *Seq. mapped in strand Plus* | *Seq. mapped in strand Plus*  |
|                       |           AF297709           |            L40459             |
|                       |           BC069233           |          BC040761             |
|                       |           AB047077           |          BC054420             |
|                       |           AB051427           |          AK007774             |
|                       |           AB051428           |                               |
|                       |           CR610529           |                               |
|                       |           AF225424           |                               |
|                       |           BC009967           |                               |
|                       | *Seq. mapped in strand Minus*| *Seq. mapped in strand Minus* |
|                       |           AF318354           |          AK019240             |
|                       |           AF135960           |          BC034519             |
|                       |           AK126126           |          AF276514             |
|                       |           AL117551           |                               |
|                       |           AK097212           |                               |
|                       |           AK090750           |                               |
|                       |           AK124524           |                               |
|                       |           BC008761           |                               |
|                       |           AK024477           |                               |
|                       |           AF135961           |                               |
|                       |           AK127484           |                               |
|                       |           AF011407           |                               |
+-----------------------+------------------------------+-------------------------------+
|      #  105           | *Seq. mapped in strand Plus* | *Seq. mapped in strand Plus*  |
|                       |           AK091318           |          BC025547             |
|                       |           BC015715           |          AK077926             |
|                       |           CR626820           |          AK011295             |
|                       |           CR610136           |          AK009515             |
|                       |           AF302084           |          AF277171             |
|                       |           BC001799           |          AK085558             |
|                       |           AL834512           |          AK052941             |
|                       |           AK124449           |                               |
|                       | *Seq. mapped in strand Minus*| *Seq. mapped in strand Minus* |
|                       |           CR609564           |          AK018272             |
|                       |           CR617502           |          AB052739             |
|                       |             X06409           |          AB057663             |
|                       |             X03484           |          BC015273             |
|                       |           BC018119           |          BC056439             |
|                       |           CR608506           |          AB057655             |
|                       |           CR598160           |          AK036317             |
|                       |           CR616511           |          AB077145             |
|                       |           BX648063           |                               |
+-----------------------+------------------------------+-------------------------------+
|      #  106           | *Seq. mapped in strand Plus* | *Seq. mapped in strand Plus*  |
|                       |           BC034490           |          BC020528             |
|                       |             X76061           |            U47333             |
|                       |             S67171           |            U50850             |
|                       |             X74594           |            U36799             |
|                       |           BX537767           |            Z68184             |
|                       |           AK129969           |                               |
|                       |           CR608692           |                               |
|                       | *Seq. mapped in strand Minus*| *Seq. mapped in strand Minus* |
|                       |           AK123139           |          BC008130             |
|                       |           AK123086           |            Z67963             |
|                       |           CR602186           |            X71978             |
|                       |           AK098222           |          BC034861             |
|                       |           AL133106           |                               |
|                       |           AK023320           |                               |
|                       |           BC001134           |                               |
|                       |           CR457308           |                               |
+-----------------------+------------------------------+-------------------------------+
|      #  107           | *Seq. mapped in strand Plus* | *Seq. mapped in strand Plus*  |
|                       |             X91911           |          AK045526             |
|                       |           BC005227           |          AK012108             |
|                       |           BC012510           |          AK036447             |
|                       |           AF400440           |          AK041854             |
|                       |                              |          AK030560             |
|                       |                              |          AK032472             |
|                       |                              |          AK011972             |
|                       |                              |          AK078627             |
|                       | *Seq. mapped in strand Minus*| *Seq. mapped in strand Minus* |
|                       |           AK024153           |          BC025083             |
|                       |           AK093825           |          AK010768             |
|                       |           CR613297           |                               |
|                       |           BC033887           |                               |
|                       |           CR604914           |                               |
|                       |           BC026107           |                               |
|                       |           BC016778           |                               |
|                       |           CR593218           |                               |
|                       |           BC014034           |                               |
|                       |             U55766           |                               |
|                       |           BC005225           |                               |
+-----------------------+------------------------------+-------------------------------+
|      #  108           | *Seq. mapped in strand Plus* | *Seq. mapped in strand Plus*  |
|                       |           CR622248           |          BC011462             |
|                       |           BC011825           |            U22325             |
|                       |           BC007699           |          BC004838             |
|                       |           CR599852           |                               |
|                       |           CR619596           |                               |
|                       |             U92980           |                               |
|                       |           CR594539           |                               |
|                       | *Seq. mapped in strand Minus*| *Seq. mapped in strand Minus* |
|                       |           BC034530           |          AK044504             |
|                       |             U11690           |          AK009202             |
|                       |           CR617413           |          BC037444             |
+-----------------------+------------------------------+-------------------------------+
|      #  109           | *Seq. mapped in strand Plus* | *Seq. mapped in strand Plus*  |
|                       |           AJ009985           |          AK015696             |
|                       |           AF230929           |          AK036838             |
|                       |           BC005830           |          AK087859             |
|                       |           CR536481           |          AB075603             |
|                       |                              |          BC051048             |
|                       |                              |          BC039762             |
|                       |                              |          BC057901             |
|                       |                              |          BC069953             |
|                       |                              |          BC008112             |
|                       |                              |          AK007342             |
|                       |                              |          AK036588             |
|                       | *Seq. mapped in strand Minus*| *Seq. mapped in strand Minus* |
|                       |           AB037811           |          BC058635             |
|                       |           BC032321           |          BC057546             |
|                       |           BC014447           |          AK081637             |
|                       |           AK125959           |          AF051908             |
|                       |           AK125493           |          AK053118             |
|                       |           AK002142           |          AK082182             |
|                       |           BC075812           |          AJ401160             |
|                       |           CR594376           |          BC062140             |
|                       |                              |          AK010128             |
|                       |                              |          AK003395             |
|                       |                              |                               |
+-----------------------+------------------------------+-------------------------------+
|      #  110           | *Seq. mapped in strand Plus* | *Seq. mapped in strand Plus*  |
|                       |           AY064249           |          AK003666             |
|                       |           AY064248           |          AK010684             |
|                       |           BC009771           |          BC048465             |
|                       |           AB040451           |          AK041267             |
|                       |           AK055691           |          AK017071             |
|                       |           AB040450           |          AK005694             |
|                       |           CR616411           |          BC046278             |
|                       |           AL834458           |                               |
|                       |           CR592263           |                               |
|                       |           CR590039           |                               |
|                       |           AY092061           |                               |
|                       |           CR626197           |                               |
|                       |           CR595451           |                               |
|                       |           AK092054           |                               |
|                       | *Seq. mapped in strand Minus*| *Seq. mapped in strand Minus* |
|                       |           BC037925           |          BC022920             |
|                       |           AL162051           |          AK033624             |
|                       |           BC002473           |          BC024885             |
|                       |           AK001556           |          BC006911             |
|                       |           AY064250           |          AK079184             |
|                       |           AK001751           |          AY065980             |
|                       |           AF427340           |          AK028910             |
|                       |           AK024869           |          BC038701             |
|                       |           BC068471           |                               |
|                       |           AF427341           |                               |
+-----------------------+------------------------------+-------------------------------+
|      #  111           | *Seq. mapped in strand Plus* | *Seq. mapped in strand Plus*  |
|                       |           AK131064           |          AK029862             |
|                       |           AK001768           |                               |
|                       |           CR593695           |                               |
|                       |           BC051292           |                               |
|                       |           BC011937           |                               |
|                       |           BC019103           |                               |
|                       |           CR450300           |                               |
|                       |           CR598759           |                               |
|                       |           CR608161           |                               |
|                       |           CR614249           |                               |
|                       |           BC020458           |                               |
|                       |           AF277183           |                               |
|                       |           AK123310           |                               |
|                       | *Seq. mapped in strand Minus*| *Seq. mapped in strand Minus* |
|                       |           AK128159           |          AK082176             |
+-----------------------+------------------------------+-------------------------------+
|      #  112           | *Seq. mapped in strand Plus* | *Seq. mapped in strand Plus*  |
|                       |           CR610886           |          BC046487             |
|                       |           CR616943           |          AF136399             |
|                       |             M58525           |          AF412308             |
|                       |           CR613786           |          AB027566             |
|                       |           CR625831           |          BC013688             |
|                       |           AK130031           |          BC052157             |
|                       |           CR612917           |          BC052758             |
|                       |           CR624969           |          AK075828             |
|                       |           BC000419           |          BC029344             |
|                       |           BC005867           |          AF171053             |
|                       |           CR612170           |                               |
|                       |           CR603360           |                               |
|                       |           CR609952           |                               |
|                       |           BC011935           |                               |
|                       |             M65212           |                               |
|                       |           CR603785           |                               |
|                       |           CR456422           |                               |
|                       |           BT007125           |                               |
|                       |           CR456997           |                               |
|                       |           CR595297           |                               |
|                       |           CR590298           |                               |
|                       |           CR610047           |                               |
|                       |           CR602287           |                               |
|                       | *Seq. mapped in strand Minus*| *Seq. mapped in strand Minus* |
|                       |             U84507           |          AK084547             |
|                       |             U84511           |          AF076156             |
|                       |           AL390148           |          BC010402             |
|                       |           AK092664           |                               |
|                       |             U51269           |                               |
|                       |           CR590273           |                               |
|                       |           CR623754           |                               |
|                       |           BC036893           |                               |
|                       |           AB019695           |                               |
|                       |           AB019694           |                               |
|                       |           AF166126           |                               |
|                       |           AF166127           |                               |
|                       |           AK097708           |                               |
|                       |           CR602507           |                               |
|                       |           CR590304           |                               |
|                       |           CR608079           |                               |
|                       |           AF106697           |                               |
|                       |           AK094698           |                               |
|                       |           BC032029           |                               |
|                       |           BC007489           |                               |
|                       |           BC038708           |                               |
|                       |           AF171054           |                               |
|                       |           AF044212           |                               |
|                       |           CR603526           |                               |
|                       |           CR590262           |                               |
|                       |           CR601782           |                               |
|                       |           CR594969           |                               |
|                       |           CR593863           |                               |
|                       |           CR591901           |                               |
|                       |           BX957216           |                               |
|                       |           CR749428           |                               |
+-----------------------+------------------------------+-------------------------------+
|      #  113           | *Seq. mapped in strand Plus* | *Seq. mapped in strand Plus*  |
|                       |           BC047465           |          BC049541             |
|                       |           AK098224           |          AK034636             |
|                       |           BC021985           |          AF109377             |
|                       |           AB037802           |          BC063056             |
|                       |           AK025633           |          AK031510             |
|                       |           CR603633           |          AK019727             |
|                       |           AL359611           |                               |
|                       | *Seq. mapped in strand Minus*| *Seq. mapped in strand Minus* |
|                       |           BC011054           |          BC025149             |
|                       |           BC025238           |          BC056429             |
|                       |           AK027681           |          AK009763             |
|                       |           AK074755           |          AK087540             |
|                       |           BX537858           |          BC060965             |
|                       |           BX648968           |          AK017659             |
|                       |           CR624411           |          AK050675             |
|                       |             U92989           |          AK028386             |
+-----------------------+------------------------------+-------------------------------+
|      #  114           | *Seq. mapped in strand Plus* | *Seq. mapped in strand Plus*  |
|                       |           AK027704           |          AF009328             |
|                       |           AK022832           |          BC037645             |
|                       |           BX537951           |          AF009327             |
|                       |           BC014946           |                               |
|                       | *Seq. mapped in strand Minus*| *Seq. mapped in strand Minus* |
|                       |           BC069626           |          BC058978             |
|                       |           BC069651           |          AK028388             |
|                       |           AY572806           |          AK012264             |
|                       |           AY572807           |          AK029485             |
|                       |           AY572808           |                               |
|                       |           AY572809           |                               |
|                       |           AY572810           |                               |
|                       |           AY572811           |                               |
|                       |           AY572812           |                               |
|                       |           AY572813           |                               |
|                       |           AY572814           |                               |
|                       |           AY572815           |                               |
|                       |           AY572816           |                               |
|                       |           AY572817           |                               |
|                       |           AY572818           |                               |
|                       |           AY572819           |                               |
|                       |           AY572820           |                               |
|                       |           AY572821           |                               |
|                       |           AY572822           |                               |
|                       |           AY572823           |                               |
|                       |           AY572824           |                               |
|                       |           AY572825           |                               |
|                       |           AY572826           |                               |
|                       |           AY572827           |                               |
|                       |           BC030972           |                               |
+-----------------------+------------------------------+-------------------------------+
|      #  115           | *Seq. mapped in strand Plus* | *Seq. mapped in strand Plus*  |
|                       |           CR591869           |          AK076325             |
|                       |           BC017711           |          BC043049             |
|                       |             D42138           |          AK007973             |
|                       |                              |          AK050898             |
|                       | *Seq. mapped in strand Minus*| *Seq. mapped in strand Minus* |
|                       |           CR590505           |          BC052658             |
|                       |           BC042826           |            D84436             |
|                       |           AB033080           |                               |
|                       |           AK022459           |                               |
|                       |           AF011794           |                               |
|                       |           AF212228           |                               |
|                       |           BC039871           |                               |
|                       |           BC034914           |                               |
|                       |           BC027621           |                               |
|                       |           BC029398           |                               |
|                       |           BC015203           |                               |
+-----------------------+------------------------------+-------------------------------+
|      #  116           | *Seq. mapped in strand Plus* | *Seq. mapped in strand Plus*  |
|                       |           BC042525           |          BC031195             |
|                       |           AF353674           |          AK033757             |
|                       |           BC006414           |                               |
|                       |           AF086420           |                               |
|                       | *Seq. mapped in strand Minus*| *Seq. mapped in strand Minus* |
|                       |             U75276           |          AK010890             |
|                       |           AK126499           |          BC058112             |
|                       |           AK125024           |          BC037224             |
|                       |           AK124665           |                               |
|                       |           BC071637           |                               |
|                       |           AJ297407           |                               |
|                       |           AJ297406           |                               |
|                       |           AK096471           |                               |
|                       |           CR607892           |                               |
|                       |           BC016743           |                               |
|                       |           AJ297408           |                               |
+-----------------------+------------------------------+-------------------------------+
|      #  117           | *Seq. mapped in strand Plus* | *Seq. mapped in strand Plus*  |
|                       |             M60315           |            X80992             |
|                       |           BC028580           |            J04566             |
|                       |                              |          AK014797             |
|                       | *Seq. mapped in strand Minus*| *Seq. mapped in strand Minus* |
|                       |           AY326464           |          AK016326             |
|                       |           AY358646           |          BC036155             |
|                       |           CR592427           |          BC046789             |
|                       |           BC003529           |          AY243534             |
|                       |           AL834423           |          BC016252             |
|                       |           BC001199           |          BC024505             |
|                       |           AL833008           |          AY548112             |
|                       |           AK075291           |                               |
|                       |           AK098089           |                               |
|                       |           BC052310           |                               |
|                       |           CR606756           |                               |
|                       |           CR612583           |                               |
|                       |           CR593245           |                               |
|                       |           AJ440721           |                               |
|                       |           AK025442           |                               |
|                       |           CR749569           |                               |
|                       |           BX647336           |                               |
|                       |           AF426434           |                               |
|                       |           BC032438           |                               |
+-----------------------+------------------------------+-------------------------------+
|      #  118           | *Seq. mapped in strand Plus* | *Seq. mapped in strand Plus*  |
|                       |           AY225123           |          BC070466             |
|                       |           AK125086           |          BC010312             |
|                       |           BC060509           |          AK050032             |
|                       |           AL833598           |          BC003281             |
|                       |           BC042191           |                               |
|                       |           AF484416           |                               |
|                       |           BX648645           |                               |
|                       | *Seq. mapped in strand Minus*| *Seq. mapped in strand Minus* |
|                       |           AK130147           |          AK090152             |
|                       |           BC039580           |                               |
|                       |           AF307338           |                               |
|                       |           AF307339           |                               |
|                       |           AL713679           |                               |
|                       |           AK123412           |                               |
|                       |           AL832929           |                               |
|                       |           BX648869           |                               |
|                       |           CR599565           |                               |
|                       |           BC017463           |                               |
|                       |           CR596753           |                               |
+-----------------------+------------------------------+-------------------------------+
|      #  119           | *Seq. mapped in strand Plus* | *Seq. mapped in strand Plus*  |
|                       |           BC003094           |          AK050199             |
|                       |           AB023205           |          AK054343             |
|                       |           BC006364           |          AK041464             |
|                       |           AK091959           |          BC059843             |
|                       |           AJ006417           |          AB093276             |
|                       |           AL133562           |          AK020695             |
|                       |           BC039654           |          AK075908             |
|                       |           AK057975           |          BC024992             |
|                       |           AL096745           |          AK082430             |
|                       |           BC024167           |                               |
|                       |           CR607508           |                               |
|                       |           BC012824           |                               |
|                       |           CR593345           |                               |
|                       |           AF193042           |                               |
|                       |           AK129619           |                               |
|                       | *Seq. mapped in strand Minus*| *Seq. mapped in strand Minus* |
|                       |           AK023903           |          AK037185             |
|                       |           AK054757           |          AK030690             |
|                       |           AY634364           |          AK037240             |
|                       |           BC065543           |          AK028879             |
|                       |             U79265           |          AK076268             |
|                       |           AK126018           |          BC062106             |
|                       |           BC041588           |          BC022739             |
|                       |           BC019623           |          BC051647             |
|                       |           BC035891           |          AK031361             |
+-----------------------+------------------------------+-------------------------------+
|      #  120           | *Seq. mapped in strand Plus* | *Seq. mapped in strand Plus*  |
|                       |           AY093951           |          AK038830             |
|                       |           AY093952           |          BC027575             |
|                       |           AK093719           |                               |
|                       |           BC004347           |                               |
|                       |           BC013366           |                               |
|                       |           BC015584           |                               |
|                       |           CR621529           |                               |
|                       |           CR613949           |                               |
|                       | *Seq. mapped in strand Minus*| *Seq. mapped in strand Minus* |
|                       |           BC005133           |          BC066803             |
|                       |           AK026364           |          AK090332             |
|                       |           CR591349           |                               |
|                       |           AY211494           |                               |
|                       |           CR599436           |                               |
+-----------------------+------------------------------+-------------------------------+
|      #  121           | *Seq. mapped in strand Plus* | *Seq. mapped in strand Plus*  |
|                       |             L13220           |          AK052854             |
|                       |             X65869           |          AK084595             |
|                       |                              |          AK085381             |
|                       |                              |          BC003257             |
|                       |                              |          AK032018             |
|                       |                              |            U49385             |
|                       | *Seq. mapped in strand Minus*| *Seq. mapped in strand Minus* |
|                       |           AK025654           |          AF293948             |
|                       |           AK125332           |          AF293949             |
|                       |           BC034986           |          BC010751             |
|                       |           BX647712           |          AF136283             |
|                       |           AK024070           |          AF028071             |
|                       |           BC006256           |            U88544             |
|                       |           AK125348           |                               |
|                       |           AF226667           |                               |
|                       |           AK023549           |                               |
+-----------------------+------------------------------+-------------------------------+
|      #  122           | *Seq. mapped in strand Plus* | *Seq. mapped in strand Plus*  |
|                       |           AK025974           |          AB109092             |
|                       |           CR590195           |          AK041947             |
|                       |           CR611947           |                               |
|                       |           CR623418           |                               |
|                       |           CR622897           |                               |
|                       |           CR613603           |                               |
|                       |           CR596012           |                               |
|                       |           CR624452           |                               |
|                       |           BC001378           |                               |
|                       |           CR625528           |                               |
|                       |           CR624036           |                               |
|                       |           CR592065           |                               |
|                       |           CR599766           |                               |
|                       |           CR602235           |                               |
|                       |           CR620265           |                               |
|                       | *Seq. mapped in strand Minus*| *Seq. mapped in strand Minus* |
|                       |           AF144308           |          AK036812             |
|                       |           AB008535           |          AK078407             |
+-----------------------+------------------------------+-------------------------------+
|      #  123           | *Seq. mapped in strand Plus* | *Seq. mapped in strand Plus*  |
|                       |           AJ580932           |          AK015299             |
|                       |           AK096876           |          AK015954             |
|                       |           CR601150           |          BC037142             |
|                       |           CR606146           |          BC019170             |
|                       |           CR619193           |          BC019156             |
|                       |           CR620013           |          BC049899             |
|                       |           BC003399           |          BC037648             |
|                       |           CR592739           |          AK017037             |
|                       |           CR616539           |                               |
|                       |           CR621283           |                               |
|                       |           BC012852           |                               |
|                       |           CR749718           |                               |
|                       | *Seq. mapped in strand Minus*| *Seq. mapped in strand Minus* |
|                       |           BC071561           |            D78572             |
|                       |           CR626564           |                               |
|                       |           AF381545           |                               |
|                       |           BC014276           |                               |
|                       |           AK126745           |                               |
|                       |           AB050468           |                               |
+-----------------------+------------------------------+-------------------------------+
|      #  124           | *Seq. mapped in strand Plus* | *Seq. mapped in strand Plus*  |
|                       |           AB094093           |          BC002088             |
|                       |                              |          BC027208             |
|                       |                              |          AK077450             |
|                       | *Seq. mapped in strand Minus*| *Seq. mapped in strand Minus* |
|                       |           CR620392           |          BC055929             |
|                       |           CR608319           |          AK052757             |
|                       |           CR622778           |                               |
|                       |           BC004986           |                               |
|                       |             M64716           |                               |
|                       |           BC017930           |                               |
|                       |           BC060042           |                               |
+-----------------------+------------------------------+-------------------------------+
|      #  125           | *Seq. mapped in strand Plus* | *Seq. mapped in strand Plus*  |
|                       |           AK055943           |          AK079680             |
|                       |                              |          AK089062             |
|                       |                              |          AK005277             |
|                       | *Seq. mapped in strand Minus*| *Seq. mapped in strand Minus* |
|                       |           BC042548           |          BC066021             |
|                       |           BC063391           |          AK085511             |
|                       |           AL122109           |                               |
|                       |           CR598994           |                               |
|                       |           BC073881           |                               |
+-----------------------+------------------------------+-------------------------------+
|      #  126           | *Seq. mapped in strand Plus* | *Seq. mapped in strand Plus*  |
|                       |           BC063294           |          AK077896             |
|                       |             M93718           |            U53142             |
|                       |             M95296           |          BC052636             |
|                       |           BC069465           |                               |
|                       |           AF400594           |                               |
|                       |             S80791           |                               |
|                       |           CR596138           |                               |
|                       | *Seq. mapped in strand Minus*| *Seq. mapped in strand Minus* |
|                       |           AY316116           |          AK010032             |
|                       |           AY515311           |                               |
|                       |           AK027791           |                               |
|                       |           AK096734           |                               |
|                       |           CR607530           |                               |
|                       |           CR613491           |                               |
+-----------------------+------------------------------+-------------------------------+
|      #  127           | *Seq. mapped in strand Plus* | *Seq. mapped in strand Plus*  |
|                       |           CR594882           |          AK041285             |
|                       |           CR606909           |          AK033112             |
|                       |           CR592460           |          AK080498             |
|                       |           CR615909           |          AK082317             |
|                       |           CR600882           |          AK082230             |
|                       |           CR597887           |          AK046175             |
|                       |           CR626280           |          AK049626             |
|                       |           BC004927           |          AK014464             |
|                       |             D17409           |          BC048189             |
|                       |           CR612359           |          AF200346             |
|                       |           BC065829           |          AF190726             |
|                       |           CR541681           |          AK014390             |
|                       |             M83106           |          AK028131             |
|                       |           CR536484           |          BC056456             |
|                       |           BX640723           |          BC007322             |
|                       |                              |            U48830             |
|                       |                              |          BC006730             |
|                       |                              |          AK050638             |
|                       | *Seq. mapped in strand Minus*| *Seq. mapped in strand Minus* |
|                       |           AK127262           |          BC037121             |
|                       |           AK097207           |          AK046997             |
|                       |             U40623           |          AK032376             |
|                       |           BC006357           |          AK051527             |
|                       |           BC010696           |          AK033867             |
|                       |             U33849           |          AK028768             |
|                       |           BT006870           |          BC064100             |
|                       |                              |          AK078706             |
|                       |                              |          BC023073             |
|                       |                              |          BC049909             |
|                       |                              |          AK030208             |
|                       |                              |          AK046190             |
|                       |                              |          AK077235             |
|                       |                              |          BC003795             |
|                       |                              |            L41154             |
|                       |                              |          AK002880             |
|                       |                              |            U36588             |
|                       |                              |                               |
+-----------------------+------------------------------+-------------------------------+
|      #  128           | *Seq. mapped in strand Plus* | *Seq. mapped in strand Plus*  |
|                       |           AY358308           |          AK052998             |
|                       |           BC018370           |          BC008653             |
|                       |           AF169675           |                               |
|                       |           AF007139           |                               |
|                       | *Seq. mapped in strand Minus*| *Seq. mapped in strand Minus* |
|                       |           BC003188           |          AK017535             |
|                       |           AF202922           |          AK052737             |
|                       |           BC000270           |          AY495668             |
|                       |           BC007297           |          BC070403             |
|                       |           BC008316           |          AY495667             |
|                       |           AF086206           |                               |
+-----------------------+------------------------------+-------------------------------+
|      #  129           | *Seq. mapped in strand Plus* | *Seq. mapped in strand Plus*  |
|                       |           BC036031           |          AK049933             |
|                       |             U71300           |          AK016168             |
|                       |             U66413           |          AK031924             |
|                       |           BC014985           |          AK031870             |
|                       |           CR624760           |          AK010932             |
|                       |           AK026895           |          AK007563             |
|                       |                              |          BC048713             |
|                       |                              |          AK019863             |
|                       |                              |          AK053153             |
|                       |                              |          AK048571             |
|                       |                              |          AK042735             |
|                       |                              |          AK020824             |
|                       |                              |          AK038357             |
|                       | *Seq. mapped in strand Minus*| *Seq. mapped in strand Minus* |
|                       |             U94319           |          BC002260             |
|                       |           AF063020           |          AJ308965             |
|                       |           CR611837           |          BC043079             |
|                       |           AF432220           |          AF339082             |
|                       |           BX649155           |          BC038820             |
|                       |           AF098483           |          BC052177             |
|                       |           AK096405           |          AF339083             |
|                       |           AF098482           |          AJ308966             |
|                       |           BC064135           |          BC027215             |
|                       |           BC044568           |          BC002104             |
|                       |           BC040032           |          AK011572             |
|                       |           BC022971           |          AK042466             |
|                       |           BC013160           |                               |
|                       |           BC033817           |                               |
+-----------------------+------------------------------+-------------------------------+
|      #  130           | *Seq. mapped in strand Plus* | *Seq. mapped in strand Plus*  |
|                       |           BX647402           |          BC046336             |
|                       |           BC010377           |          BC052694             |
|                       |             U01160           |          AK011871             |
|                       |           CR616737           |          AK006918             |
|                       |           CR592832           |          AK012064             |
|                       |           CR623983           |          AK003804             |
|                       |           CR407647           |          AK019380             |
|                       |                              |          AK078440             |
|                       |                              |          AK013400             |
|                       |                              |            L01640             |
|                       |                              |            X57238             |
|                       |                              |            X65069             |
|                       |                              |          AK012202             |
|                       | *Seq. mapped in strand Minus*| *Seq. mapped in strand Minus* |
|                       |           BC003644           |          AK012567             |
|                       |           CR602761           |          AK012853             |
|                       |           CR622282           |          AK012457             |
|                       |           CR602927           |          AK075962             |
|                       |           BC010153           |          BC023306             |
|                       |             M14505           |          AK014183             |
|                       |             U79269           |          AK013523             |
|                       |             Z48970           |                               |
|                       |           BC015669           |                               |
|                       |           BC005864           |                               |
|                       |           CR598582           |                               |
|                       |           CR590586           |                               |
|                       |           CR597992           |                               |
|                       |           CR609991           |                               |
|                       |           CR591116           |                               |
|                       |           CR616342           |                               |
|                       |           CR594194           |                               |
|                       |           CR608247           |                               |
|                       |           CR542247           |                               |
|                       |           CR407668           |                               |
+-----------------------+------------------------------+-------------------------------+
|      #  131           | *Seq. mapped in strand Plus* | *Seq. mapped in strand Plus*  |
|                       |             L38487           |          BC016191             |
|                       |           BC063795           |          AK002791             |
|                       |           BC073956           |          AK003292             |
|                       |           CR624219           |          BC019418             |
|                       |           BC007915           |                               |
|                       |           BC033701           |                               |
|                       |           BC011528           |                               |
|                       | *Seq. mapped in strand Minus*| *Seq. mapped in strand Minus* |
|                       |           AF110774           |          AB041573             |
|                       |           BC029482           |          AF124994             |
|                       |           BC017172           |          BC014682             |
|                       |           AF161501           |          AK002383             |
+-----------------------+------------------------------+-------------------------------+
|      #  132           | *Seq. mapped in strand Plus* | *Seq. mapped in strand Plus*  |
|                       |           BC029050           |            U90886             |
|                       |           CR625558           |          BC023349             |
|                       |           AY074489           |          AF032466             |
|                       |           BC008464           |                               |
|                       |           CR598558           |                               |
|                       |             U75667           |                               |
|                       |             D86724           |                               |
|                       |           BC001350           |                               |
|                       |             U82256           |                               |
|                       |           CR536550           |                               |
|                       |           CR595481           |                               |
|                       |           CR595402           |                               |
|                       |           CR599903           |                               |
|                       | *Seq. mapped in strand Minus*| *Seq. mapped in strand Minus* |
|                       |           BC042390           |          AK002289             |
|                       |           CR604112           |          BC002290             |
|                       |           CR610010           |          BC009810             |
|                       |           CR606212           |          AF035208             |
|                       |           BC003142           |          AF035821             |
|                       |           AF060902           |                               |
|                       |           CR603439           |                               |
|                       |           CR612585           |                               |
|                       |           AF035824           |                               |
|                       |           CR456757           |                               |
|                       |           CR542095           |                               |
+-----------------------+------------------------------+-------------------------------+
|      #  133           | *Seq. mapped in strand Plus* | *Seq. mapped in strand Plus*  |
|                       |           AF320072           |          AK076063             |
|                       |           CR605739           |          AF190624             |
|                       |             U37219           |          BC049737             |
|                       |           BC000022           |          AK006858             |
|                       |           AK127259           |          AK005955             |
|                       |           BC028385           |          AK019819             |
|                       |           CR456548           |          AK018910             |
|                       |             U37221           |          AK006505             |
|                       |             U37220           |          AK014931             |
|                       |           AK097249           |          AK015752             |
|                       |           CR611119           |                               |
|                       | *Seq. mapped in strand Minus*| *Seq. mapped in strand Minus* |
|                       |           BC015229           |          AK087378             |
|                       |           CR616192           |          BC028899             |
|                       |           AK096530           |          AK012472             |
|                       |                              |          AK081792             |
|                       |                              |          AK090052             |
|                       |                              |          AK009460             |
|                       |                              |                               |
|                       |                              |                               |
|                       |                              |                               |
|                       |                              |                               |
+-----------------------+------------------------------+-------------------------------+
|      #  134           | *Seq. mapped in strand Plus* | *Seq. mapped in strand Plus*  |
|                       |           AF230336           |          BC040776             |
|                       |           AF340183           |          BC042619             |
|                       |           BC038666           |          BC042870             |
|                       |           BC013582           |                               |
|                       |           AK022613           |                               |
|                       |           AL832427           |                               |
|                       |           AK074406           |                               |
|                       | *Seq. mapped in strand Minus*| *Seq. mapped in strand Minus* |
|                       |           BC028122           |          AK016461             |
|                       |           AF230334           |          BC049660             |
|                       |           AY248899           |          BC042706             |
+-----------------------+------------------------------+-------------------------------+
|      #  135           | *Seq. mapped in strand Plus* | *Seq. mapped in strand Plus*  |
|                       |           CR594447           |          AF175892             |
|                       |           CR623107           |          BC025061             |
|                       |           AK054564           |                               |
|                       |           AK092173           |                               |
|                       |           BC033703           |                               |
|                       |           AL832527           |                               |
|                       |           AY424282           |                               |
|                       |           CR609513           |                               |
|                       |           CR622431           |                               |
|                       |           CR609445           |                               |
|                       |           AF086057           |                               |
|                       | *Seq. mapped in strand Minus*| *Seq. mapped in strand Minus* |
|                       |           CR597027           |          BC011185             |
|                       |             U56816           |          AK005142             |
|                       |           AK098452           |          AY424293             |
|                       |           CR617420           |          BC026921             |
|                       |           AK097642           |          AK044092             |
|                       |           CR595647           |          AK033625             |
|                       |           CR601395           |          AB041564             |
|                       |           CR610333           |          AK044486             |
|                       |           CR608067           |                               |
|                       |           CR598569           |                               |
|                       |           CR622913           |                               |
|                       |           CR603670           |                               |
+-----------------------+------------------------------+-------------------------------+
|      #  136           | *Seq. mapped in strand Plus* | *Seq. mapped in strand Plus*  |
|                       |           AY103469           |          AK077528             |
|                       |           AF503290           |          BC024462             |
|                       |           AY358684           |                               |
|                       |           BC018409           |                               |
|                       |           AK000653           |                               |
|                       |           BC002652           |                               |
|                       | *Seq. mapped in strand Minus*| *Seq. mapped in strand Minus* |
|                       |           AK055820           |          AK040537             |
|                       |           BX647304           |          BC035208             |
|                       |           AK026410           |          AK014482             |
|                       |           AL713770           |                               |
|                       |           AL834293           |                               |
|                       |           BC033437           |                               |
+-----------------------+------------------------------+-------------------------------+
|      #  137           | *Seq. mapped in strand Plus* | *Seq. mapped in strand Plus*  |
|                       |           CR624597           |          BC056363             |
|                       |           AK095329           |          BC048813             |
|                       |           BC056154           |          BC057451             |
|                       |           BC070485           |                               |
|                       |           BC065291           |                               |
|                       |           AF498976           |                               |
|                       |             M31470           |                               |
|                       |           CR594469           |                               |
|                       | *Seq. mapped in strand Minus*| *Seq. mapped in strand Minus* |
|                       |           BC039392           |          BC028862             |
|                       |           BC048424           |            D50264             |
|                       |           BC029408           |                               |
|                       |           BC021725           |                               |
|                       |             D13435           |                               |
|                       |           CR604721           |                               |
|                       |           CR457000           |                               |
+-----------------------+------------------------------+-------------------------------+
|      #  138           | *Seq. mapped in strand Plus* | *Seq. mapped in strand Plus*  |
|                       |           AB095941           |          AK050912             |
|                       |           BC025332           |          AK011570             |
|                       |           BX647311           |          AK037831             |
|                       |           BX248018           |          AK032873             |
|                       |           CR593232           |          BC018523             |
|                       |           CR619293           |          AK083861             |
|                       |           AK123717           |          AK015585             |
|                       |           AK025512           |                               |
|                       |           BC015481           |                               |
|                       |           CR590406           |                               |
|                       |           AF277230           |                               |
|                       |           AB029892           |                               |
|                       |           AB044402           |                               |
|                       |           AF308571           |                               |
|                       |             D89079           |                               |
|                       |             U41070           |                               |
|                       |           BC038437           |                               |
|                       | *Seq. mapped in strand Minus*| *Seq. mapped in strand Minus* |
|                       |           AF218586           |          AF041377             |
|                       |           AF544398           |          BC046340             |
|                       |           BC000982           |          BC012664             |
|                       |           AF190901           |          AK003968             |
|                       |           AK000387           |                               |
|                       |           AK122828           |                               |
|                       |           CR611009           |                               |
|                       |           BC035970           |                               |
+-----------------------+------------------------------+-------------------------------+
|      #  139           | *Seq. mapped in strand Plus* | *Seq. mapped in strand Plus*  |
|                       |           CR622317           |          BC035549             |
|                       |             L07633           |          AK019390             |
|                       |           BC000352           |            D87909             |
|                       |           BC007503           |          AK008514             |
|                       |           CR594470           |            U60328             |
|                       |           CR593193           |                               |
|                       |           CR541658           |                               |
|                       |           CR456780           |                               |
|                       |           BX248293           |                               |
|                       |           CR614004           |                               |
|                       | *Seq. mapped in strand Minus*| *Seq. mapped in strand Minus* |
|                       |           AF151870           |          AB054001             |
|                       |           BC002491           |          BC024704             |
|                       |           AY368908           |          AK005154             |
+-----------------------+------------------------------+-------------------------------+
|      #  140           | *Seq. mapped in strand Plus* | *Seq. mapped in strand Plus*  |
|                       |           AY358940           |          AK049097             |
|                       |           AK095746           |          AY037786             |
|                       |           BC031399           |                               |
|                       |           CR591685           |                               |
|                       |           AB049586           |                               |
|                       |           AL834452           |                               |
|                       | *Seq. mapped in strand Minus*| *Seq. mapped in strand Minus* |
|                       |           AK057778           |          AK002887             |
|                       |           CR612340           |          AK082903             |
|                       |           CR598498           |          BC054109             |
|                       |           AK001399           |          AK077198             |
|                       |           BC004417           |          AF203914             |
|                       |           AF262240           |          AK012760             |
|                       |           BC046209           |          BC024780             |
|                       |           AK024768           |                               |
|                       |           BC011909           |                               |
|                       |           CR598154           |                               |
|                       |           CR621745           |                               |
|                       |           CR596276           |                               |
|                       |           AF298770           |                               |
|                       |           AY313210           |                               |
+-----------------------+------------------------------+-------------------------------+
|      #  141           | *Seq. mapped in strand Plus* | *Seq. mapped in strand Plus*  |
|                       |           CR619773           |          AK086028             |
|                       |           BC014241           |          AK087317             |
|                       |           AY255535           |          AK046512             |
|                       |           AF015257           |          AK089232             |
|                       |             U63917           |          AK050034             |
|                       |           BC067849           |          AK087100             |
|                       |             X98510           |          BC003323             |
|                       |           BC011634           |          AK048344             |
|                       |           CR541655           |          AK086625             |
|                       |           CR541741           |          AK030375             |
|                       |             Y08162           |          AK018203             |
|                       |           AY265807           |                               |
|                       |           CR619529           |                               |
|                       |           CR605880           |                               |
|                       |           AK095012           |                               |
|                       | *Seq. mapped in strand Minus*| *Seq. mapped in strand Minus* |
|                       |           CR600810           |          AK014271             |
|                       |           BC006224           |          BC044867             |
|                       |           AF495721           |          BC058580             |
|                       |           CR610454           |                               |
|                       |           CR622684           |                               |
|                       |           CR602461           |                               |
|                       |           CR622011           |                               |
|                       |           CR596689           |                               |
|                       |           BC025971           |                               |
+-----------------------+------------------------------+-------------------------------+
|      #  142           | *Seq. mapped in strand Plus* | *Seq. mapped in strand Plus*  |
|                       |             L46810           |          BC061119             |
|                       |             L35853           |          BC050966             |
|                       |             L34355           |          AK005718             |
|                       |           BC025702           |          AB022320             |
|                       |             U08895           |          AY286319             |
|                       |                              |          AB045724             |
|                       | *Seq. mapped in strand Minus*| *Seq. mapped in strand Minus* |
|                       |           BC048250           |          AK009015             |
|                       |           BC033456           |          BC060287             |
|                       |           AY286318           |          AK075915             |
|                       |                              |          AB024920             |
|                       |                              |          AF019564             |
|                       |                              |                               |
+-----------------------+------------------------------+-------------------------------+
|      #  143           | *Seq. mapped in strand Plus* | *Seq. mapped in strand Plus*  |
|                       |           AF353721           |          BC042793             |
|                       |           AY358387           |          AK015058             |
|                       |           BC047334           |                               |
|                       | *Seq. mapped in strand Minus*| *Seq. mapped in strand Minus* |
|                       |           AK000207           |          BC048608             |
|                       |                              |          AK005760             |
+-----------------------+------------------------------+-------------------------------+
|      #  144           | *Seq. mapped in strand Plus* | *Seq. mapped in strand Plus*  |
|                       |           AF168714           |          AK007453             |
|                       |           AF151070           |          AK035739             |
|                       |           AF265205           |          BC019996             |
|                       |           AF265206           |          BC004569             |
|                       |           CR749387           |                               |
|                       |           BC012552           |                               |
|                       |           BC006486           |                               |
|                       |           CR457206           |                               |
|                       | *Seq. mapped in strand Minus*| *Seq. mapped in strand Minus* |
|                       |           AK097536           |          BC038470             |
|                       |           BC041597           |          AF243512             |
|                       |           BC052233           |          BC018290             |
|                       |           AY498866           |          AK010288             |
+-----------------------+------------------------------+-------------------------------+
|      #  145           | *Seq. mapped in strand Plus* | *Seq. mapped in strand Plus*  |
|                       |           AB024964           |          AK052449             |
|                       |           AB026048           |            Y10926             |
|                       |           AB027710           |                               |
|                       | *Seq. mapped in strand Minus*| *Seq. mapped in strand Minus* |
|                       |           AK027323           |          AK041073             |
|                       |           AK074595           |          BC036558             |
|                       |           AK074597           |          AJ006140             |
|                       |           AK074495           |                               |
|                       |           AK074593           |                               |
|                       |           AJ007581           |                               |
+-----------------------+------------------------------+-------------------------------+
|      #  146           | *Seq. mapped in strand Plus* | *Seq. mapped in strand Plus*  |
|                       |           AK055926           |          AK053450             |
|                       |           CR625324           |          BC006877             |
|                       |           BC016470           |          BC066172             |
|                       |           AK021607           |                               |
|                       | *Seq. mapped in strand Minus*| *Seq. mapped in strand Minus* |
|                       |           AF119857           |          AK078516             |
|                       |           BC052279           |                               |
|                       |           AK025328           |                               |
|                       |           CR614409           |                               |
|                       |           AK130572           |                               |
|                       |           BC017168           |                               |
|                       |           CR624076           |                               |
|                       |           BC033695           |                               |
+-----------------------+------------------------------+-------------------------------+
|      #  147           | *Seq. mapped in strand Plus* | *Seq. mapped in strand Plus*  |
|                       |           AY358660           |          BC012672             |
|                       |           CR617763           |          AK090285             |
|                       |           BC014123           |                               |
|                       |           BC012053           |                               |
|                       |           AK024573           |                               |
|                       |             D17056           |                               |
|                       |             D17227           |                               |
|                       | *Seq. mapped in strand Minus*| *Seq. mapped in strand Minus* |
|                       |           BC036360           |          BC018371             |
|                       |           BC073974           |          BC063269             |
|                       |           AF371328           |          AK034209             |
+-----------------------+------------------------------+-------------------------------+
|      #  148           | *Seq. mapped in strand Plus* | *Seq. mapped in strand Plus*  |
|                       |           BC011705           |          AK030350             |
|                       |             L41162           |          AF349718             |
|                       |           BC007574           |          BC030945             |
|                       |           CR619945           |          AF237721             |
|                       |           AK075240           |            X91012             |
|                       |           BC004412           |          AK003258             |
|                       |           CR611329           |                               |
|                       |             X91013           |                               |
|                       | *Seq. mapped in strand Minus*| *Seq. mapped in strand Minus* |
|                       |           AF070992           |          AY234363             |
|                       |           AB012124           |                               |
|                       |           CR621094           |                               |
|                       |           BC046933           |                               |
|                       |           BC015689           |                               |
|                       |           BC065520           |                               |
+-----------------------+------------------------------+-------------------------------+
|      #  149           | *Seq. mapped in strand Plus* | *Seq. mapped in strand Plus*  |
|                       |             M74715           |          AK031414             |
|                       |           AK125223           |          AK045330             |
|                       |           BC029959           |          BC057935             |
|                       |           AK098805           |            L34111             |
|                       | *Seq. mapped in strand Minus*| *Seq. mapped in strand Minus* |
|                       |           BC015517           |          AY093420             |
|                       |           AY124771           |          AK128888             |
|                       |           AF297659           |          BC025824             |
|                       |                              |          BC022130             |
+-----------------------+------------------------------+-------------------------------+
|      #  150           | *Seq. mapped in strand Plus* | *Seq. mapped in strand Plus*  |
|                       |           AK023175           |          AF309072             |
|                       |           BX640701           |          AK053515             |
|                       |           AK122787           |          BC030631             |
|                       |           BC036900           |                               |
|                       |           AK000898           |                               |
|                       |           AK027468           |                               |
|                       | *Seq. mapped in strand Minus*| *Seq. mapped in strand Minus* |
|                       |           AK090598           |          BC027435             |
|                       |           AY358729           |          AK050917             |
|                       |           CR625092           |          AK009559             |
|                       |           CR616839           |          AK019825             |
|                       |           CR624474           |          AK012004             |
|                       |           BC009888           |          AK082802             |
|                       |           CR619963           |                               |
|                       |           CR591571           |                               |
|                       |           BC010151           |                               |
|                       |           BC001365           |                               |
|                       |           BC018840           |                               |
|                       |           CR626593           |                               |
|                       |           CR615745           |                               |
|                       |           CR603167           |                               |
|                       |           CR619821           |                               |
|                       |           CR590389           |                               |
|                       |             L20868           |                               |
|                       |           CR603701           |                               |
|                       |           CR592509           |                               |
|                       |           CR617069           |                               |
|                       |           CR607843           |                               |
|                       |           CR615743           |                               |
|                       |           CR619311           |                               |
|                       |           CR593486           |                               |
|                       |           CR623015           |                               |
|                       |           BC007996           |                               |
|                       |           BC007748           |                               |
|                       |           AK125772           |                               |
|                       |           CR609054           |                               |
|                       |           BC030000           |                               |
|                       |           CR592894           |                               |
|                       |           BC014653           |                               |
|                       |           BC066925           |                               |
|                       |           CR594115           |                               |
|                       |           CR626518           |                               |
|                       |           CR618330           |                               |
|                       |           CR600252           |                               |
|                       |           CR598782           |                               |
|                       |           CR600001           |                               |
|                       |           CR597698           |                               |
|                       |           BC005817           |                               |
|                       |           CR622536           |                               |
|                       |           CR608556           |                               |
|                       |           BC040610           |                               |
|                       |           CR619787           |                               |
|                       |           AK130784           |                               |
|                       |             D23660           |                               |
|                       |           CR593700           |                               |
|                       |           CR592916           |                               |
|                       |           CR611824           |                               |
|                       |           CR612622           |                               |
|                       |           CR611893           |                               |
|                       |           CR624000           |                               |
|                       |           CR600520           |                               |
|                       |           CR600624           |                               |
|                       |           CR613835           |                               |
|                       |           CR617296           |                               |
|                       |           CR590024           |                               |
|                       |           CR621294           |                               |
|                       |           CR619736           |                               |
|                       |           CR615366           |                               |
|                       |           CR605435           |                               |
|                       |           CR600936           |                               |
|                       |           CR608966           |                               |
|                       |           CR623016           |                               |
|                       |           CR612355           |                               |
|                       |           CR615206           |                               |
|                       |           CR607454           |                               |
|                       |           CR603779           |                               |
|                       |           CR612284           |                               |
|                       |           CR604722           |                               |
|                       |           CR604273           |                               |
|                       |           CR621229           |                               |
|                       |           CR596506           |                               |
|                       |           CR617258           |                               |
|                       |           CR599223           |                               |
|                       |           CR590199           |                               |
|                       |           CR598597           |                               |
|                       |           CR596594           |                               |
|                       |           CR611716           |                               |
|                       |           CR598818           |                               |
|                       |           CR598274           |                               |
|                       |           CR618503           |                               |
|                       |           CR626053           |                               |
|                       |           CR609391           |                               |
|                       |           CR603778           |                               |
|                       |           CR593926           |                               |
|                       |           CR594668           |                               |
|                       |           CR606421           |                               |
|                       |           CR590785           |                               |
|                       |           CR593403           |                               |
|                       |           CR592711           |                               |
|                       |           CR619644           |                               |
|                       |           CR598321           |                               |
|                       |           CR604370           |                               |
|                       |           CR603827           |                               |
|                       |           CR606932           |                               |
|                       |           CR624303           |                               |
|                       |           CR609569           |                               |
|                       |           CR626519           |                               |
|                       |           CR615143           |                               |
|                       |           CR624900           |                               |
|                       |           CR621297           |                               |
|                       |           CR623277           |                               |
|                       |           CR626450           |                               |
|                       |           CR596867           |                               |
|                       |           CR612494           |                               |
|                       |           CR605656           |                               |
+-----------------------+------------------------------+-------------------------------+
|      #  151           | *Seq. mapped in strand Plus* | *Seq. mapped in strand Plus*  |
|                       |           BC026187           |          AK006754             |
|                       |           AK097456           |          AK049116             |
|                       |           AL833052           |          BC006866             |
|                       |                              |          AK007245             |
|                       |                              |          AK014884             |
|                       | *Seq. mapped in strand Minus*| *Seq. mapped in strand Minus* |
|                       |           BC010276           |          BC005770             |
|                       |           BX647788           |          AK083800             |
|                       |           AF139131           |          AK050329             |
|                       |             L38932           |          AF077302             |
|                       |           AK130396           |          BC027738             |
|                       |           AF077301           |                               |
+-----------------------+------------------------------+-------------------------------+
|      #  152           | *Seq. mapped in strand Plus* | *Seq. mapped in strand Plus*  |
|                       |           BC038960           |          BC042574             |
|                       |             D25215           |          AK017531             |
|                       |                              |          BC022719             |
|                       | *Seq. mapped in strand Minus*| *Seq. mapped in strand Minus* |
|                       |           AL834329           |          AB041556             |
|                       |           AK054689           |          AK003875             |
|                       |           BC022544           |          BC046332             |
|                       |           BC039416           |                               |
|                       |           CR591413           |                               |
+-----------------------+------------------------------+-------------------------------+
|      #  153           | *Seq. mapped in strand Plus* | *Seq. mapped in strand Plus*  |
|                       |           AK094319           |          BC012217             |
|                       |           BC064523           |          AK011621             |
|                       |           BC047778           |          AK020084             |
|                       |           BC034962           |          AK082130             |
|                       |           CR611665           |                               |
|                       |           CR612638           |                               |
|                       | *Seq. mapped in strand Minus*| *Seq. mapped in strand Minus* |
|                       |             U30825           |          AK047064             |
|                       |           CR598070           |          AK034326             |
|                       |           CR593564           |          BC021775             |
|                       |           CR603096           |          AK008080             |
|                       |           CR617389           |                               |
|                       |           CR615364           |                               |
|                       |           CR607246           |                               |
|                       |           CR606248           |                               |
|                       |           CR616096           |                               |
+-----------------------+------------------------------+-------------------------------+
|      #  154           | *Seq. mapped in strand Plus* | *Seq. mapped in strand Plus*  |
|                       |           AK057192           |          AK010309             |
|                       |           CR592203           |          BC021846             |
|                       |           BC008305           |          AF003352             |
|                       |           BC002950           |          BC044861             |
|                       |           CR626373           |                               |
|                       |           AK027106           |                               |
|                       | *Seq. mapped in strand Minus*| *Seq. mapped in strand Minus* |
|                       |           AF002020           |          AK003935             |
|                       |           BC063302           |          AF003348             |
|                       |           CR600440           |          BC052437             |
|                       |                              |          BC054539             |
+-----------------------+------------------------------+-------------------------------+
|      #  155           | *Seq. mapped in strand Plus* | *Seq. mapped in strand Plus*  |
|                       |           AK074888           |          AY007238             |
|                       |           AF095136           |          AK053326             |
|                       |           AK074874           |          AK053889             |
|                       |           BC065268           |          BC027325             |
|                       |           BC022877           |                               |
|                       |           AK094558           |                               |
|                       |           AK000475           |                               |
|                       | *Seq. mapped in strand Minus*| *Seq. mapped in strand Minus* |
|                       |           BC015547           |            L31783             |
|                       |           AK022317           |          BC025146             |
|                       |           AK057848           |                               |
|                       |           CR611362           |                               |
|                       |           AF125106           |                               |
|                       |           AF237290           |                               |
|                       |           AF254133           |                               |
|                       |           CR457281           |                               |
+-----------------------+------------------------------+-------------------------------+
|      #  156           | *Seq. mapped in strand Plus* | *Seq. mapped in strand Plus*  |
|                       |           BX647367           |          BC015287             |
|                       |           BX537784           |          AK089938             |
|                       |           BX648544           |          BC004088             |
|                       |           BX537389           |                               |
|                       |           BX647365           |                               |
|                       |           BX647366           |                               |
|                       |           BX649072           |                               |
|                       |           BC037354           |                               |
|                       |           AK128456           |                               |
|                       |           BX647368           |                               |
|                       |           AK092691           |                               |
|                       | *Seq. mapped in strand Minus*| *Seq. mapped in strand Minus* |
|                       |           BC046118           |          AK011370             |
|                       |           AK026935           |          BC036292             |
|                       |           BC058900           |          AY081946             |
|                       |           AK092299           |                               |
|                       |           BC027584           |                               |
+-----------------------+------------------------------+-------------------------------+
|      #  157           | *Seq. mapped in strand Plus* | *Seq. mapped in strand Plus*  |
|                       |           AK055787           |          AK049872             |
|                       |           CR608865           |          AB056721             |
|                       |           AF308801           |          BC025626             |
|                       |           CR608979           |          AK032558             |
|                       |           AL834401           |          BC003944             |
|                       |           BC073959           |                               |
|                       |           CR626842           |                               |
|                       |           BC012422           |                               |
|                       |           BC021291           |                               |
|                       |             M34668           |                               |
|                       |           BC064406           |                               |
|                       |           BC044662           |                               |
|                       |           BC030571           |                               |
|                       |           BX571753           |                               |
|                       |           BC027308           |                               |
|                       |             X54890           |                               |
|                       |             X53364           |                               |
|                       |             X54130           |                               |
|                       |           AK090743           |                               |
|                       |           CR623898           |                               |
|                       |           CR595941           |                               |
|                       | *Seq. mapped in strand Minus*| *Seq. mapped in strand Minus* |
|                       |           BC051816           |          AK081584             |
|                       |           AK124917           |          AK028090             |
|                       |           AK056353           |          AK085613             |
|                       |           AK126535           |          AK044590             |
|                       |           BC014247           |          AK032762             |
|                       |           BC037240           |          AK045248             |
|                       |           AK026029           |          AK032354             |
|                       |           CR604790           |          BC064802             |
|                       |           CR618243           |          AK031469             |
|                       |           AY211913           |          AK079521             |
+-----------------------+------------------------------+-------------------------------+
|      #  158           | *Seq. mapped in strand Plus* | *Seq. mapped in strand Plus*  |
|                       |           AK097659           |            X56044             |
|                       |             D38076           |          BC046976             |
|                       |           CR590513           |          BC027431             |
|                       |             X83617           |          AK080170             |
|                       |           BC071998           |          BC038486             |
|                       |           CR456556           |                               |
|                       |           AK094410           |                               |
|                       | *Seq. mapped in strand Minus*| *Seq. mapped in strand Minus* |
|                       |           BC017184           |          AK002989             |
|                       |           AK025106           |          BC061140             |
|                       |           BC013352           |          AK088781             |
|                       |           AK057029           |            X56045             |
|                       |           CR595584           |            X56046             |
+-----------------------+------------------------------+-------------------------------+
|      #  159           | *Seq. mapped in strand Plus* | *Seq. mapped in strand Plus*  |
|                       |           BC063135           |          BC019126             |
|                       |           BC016764           |          AK029088             |
|                       |           AK056028           |          AK007735             |
|                       |           CR625804           |          AK088152             |
|                       |           AK093658           |          AK017774             |
|                       |           AK096199           |          BC006953             |
|                       |           AL833120           |          AK052126             |
|                       |           BC072401           |          AK013450             |
|                       |           CR621657           |          AK033645             |
|                       |           BC005148           |                               |
|                       |           AJ224326           |                               |
|                       |           AK093026           |                               |
|                       |           AK055724           |                               |
|                       |             D17200           |                               |
|                       | *Seq. mapped in strand Minus*| *Seq. mapped in strand Minus* |
|                       |           AK074441           |          AK082897             |
|                       |           AK056911           |          AK028035             |
|                       |           BC032837           |          AK081413             |
|                       |           BC070141           |                               |
+-----------------------+------------------------------+-------------------------------+
|      #  160           | *Seq. mapped in strand Plus* | *Seq. mapped in strand Plus*  |
|                       |           BC035137           |          AK078233             |
|                       |           CR596083           |          BC046795             |
|                       |             X55005           |            X51983             |
|                       |           BC000261           |            X07750             |
|                       |           BC002728           |            X07751             |
|                       |             M24899           |            X07752             |
|                       |             Y00479           |                               |
|                       |             J03239           |                               |
|                       |           CR541915           |                               |
|                       |           AF522368           |                               |
|                       |           BC062421           |                               |
|                       | *Seq. mapped in strand Minus*| *Seq. mapped in strand Minus* |
|                       |             M24900           |          BC008989             |
|                       |             M24898           |          AK041047             |
|                       |           BC047875           |          AF291821             |
|                       |           BC056148           |            X86010             |
|                       |             X72631           |                               |
|                       |           CR598991           |                               |
|                       |           CR608219           |                               |
|                       |           AY429554           |                               |
+-----------------------+------------------------------+-------------------------------+
|      #  161           | *Seq. mapped in strand Plus* | *Seq. mapped in strand Plus*  |
|                       |           BC023503           |          AK039766             |
|                       |             X63563           |          BC016415             |
|                       |           BC046499           |          BC038472             |
|                       |           AF055028           |                               |
|                       |           CR591583           |                               |
|                       |           CR609388           |                               |
|                       |           CR592056           |                               |
|                       |           AK098271           |                               |
|                       |           BC039519           |                               |
|                       | *Seq. mapped in strand Minus*| *Seq. mapped in strand Minus* |
|                       |           BC066339           |            L75822             |
|                       |             L19182           |          BC047202             |
|                       |           BC017201           |          AB012886             |
|                       |             S75725           |                               |
|                       |           CR619713           |                               |
|                       |           CR626227           |                               |
|                       |           CR599263           |                               |
|                       |           CR615138           |                               |
|                       |           CR619194           |                               |
|                       |           CR607474           |                               |
|                       |           CR612946           |                               |
|                       |           BT006654           |                               |
|                       |           AF540057           |                               |
|                       |           AK024195           |                               |
+-----------------------+------------------------------+-------------------------------+
|      #  162           | *Seq. mapped in strand Plus* | *Seq. mapped in strand Plus*  |
|                       |           AF053952           |          AK020639             |
|                       |                              |          BC029309             |
|                       |                              |          AK075916             |
|                       |                              |          BC051540             |
|                       | *Seq. mapped in strand Minus*| *Seq. mapped in strand Minus* |
|                       |           AF064804           |          AK016217             |
|                       |           AK092340           |          BC048763             |
|                       |           BC050384           |            D14637             |
|                       |           BC035816           |          AK089291             |
+-----------------------+------------------------------+-------------------------------+
|      #  163           | *Seq. mapped in strand Plus* | *Seq. mapped in strand Plus*  |
|                       |           AF464088           |          AK081999             |
|                       |           BC018288           |          AK076093             |
|                       |           BC001267           |          BC004842             |
|                       |             M28215           |          AK010495             |
|                       |           AF498936           |          AK002631             |
|                       |           CR536492           |          BC034370             |
|                       |           CR625175           |          AK008198             |
|                       |           CR597533           |                               |
|                       | *Seq. mapped in strand Minus*| *Seq. mapped in strand Minus* |
|                       |           BC021717           |          AK076579             |
|                       |           AK058178           |                               |
+-----------------------+------------------------------+-------------------------------+
|      #  164           | *Seq. mapped in strand Plus* | *Seq. mapped in strand Plus*  |
|                       |           BC029360           |          AF387731             |
|                       |           CR607132           |          AK037126             |
|                       |           CR600204           |          AK052369             |
|                       |           AK027067           |          BC030680             |
|                       |           BC007754           |          BC030672             |
|                       |           CR590158           |                               |
|                       |           CR602124           |                               |
|                       |           CR457372           |                               |
|                       |           AL834488           |                               |
|                       |           CR615952           |                               |
|                       | *Seq. mapped in strand Minus*| *Seq. mapped in strand Minus* |
|                       |           BC022254           |          AK083615             |
|                       |           BC000863           |          AK083457             |
|                       |           AK093757           |          BC032960             |
|                       |           AK022922           |          AK015728             |
|                       |           AF395747           |          AF149205             |
|                       |           AF395748           |                               |
|                       |           AF395749           |                               |
|                       |           AF395750           |                               |
|                       |           AF395751           |                               |
|                       |           AF395752           |                               |
|                       |           AJ296101           |                               |
|                       |           BC009185           |                               |
|                       |           BX648858           |                               |
|                       |           AF088055           |                               |
+-----------------------+------------------------------+-------------------------------+
|      #  165           | *Seq. mapped in strand Plus* | *Seq. mapped in strand Plus*  |
|                       |           CR601375           |            U10484             |
|                       |           BC029391           |          AK079932             |
|                       |             U10485           |          BC052909             |
|                       |                              |          AK039595             |
|                       | *Seq. mapped in strand Minus*| *Seq. mapped in strand Minus* |
|                       |           BC047415           |          BC072568             |
|                       |           AK001783           |          AY423542             |
|                       |           BC028415           |          AK034148             |
|                       |           AK093102           |          AK082677             |
|                       |           BC031047           |          AK039005             |
|                       |           BC057795           |          AK029682             |
|                       |           AY423543           |                               |
+-----------------------+------------------------------+-------------------------------+
|      #  166           | *Seq. mapped in strand Plus* | *Seq. mapped in strand Plus*  |
|                       |             U43408           |          AK075841             |
|                       |           BC035782           |          AK011716             |
|                       |           CR623896           |          AK029154             |
|                       |                              |          AF159850             |
|                       |                              |          BC020143             |
|                       | *Seq. mapped in strand Minus*| *Seq. mapped in strand Minus* |
|                       |           BC011735           |          AK076220             |
|                       |           AK124006           |          BC055303             |
|                       |           AK098229           |          BC029623             |
|                       |           AK055822           |          AK033440             |
|                       |           CR618924           |          AF332512             |
|                       |           AF289602           |          AF307746             |
|                       |           BC028080           |                               |
|                       |           AK075188           |                               |
|                       |           AF159442           |                               |
|                       |           CR600673           |                               |
|                       |           CR597400           |                               |
|                       |           CR604817           |                               |
|                       |           CR600989           |                               |
|                       |           CR621218           |                               |
|                       |           CR622721           |                               |
|                       |           BC030270           |                               |
+-----------------------+------------------------------+-------------------------------+
|      #  167           | *Seq. mapped in strand Plus* | *Seq. mapped in strand Plus*  |
|                       |           AK023707           |          BC005798             |
|                       |           BC023634           |          AK014770             |
|                       |           AY040325           |          AK011690             |
|                       |           CR617782           |          AK053369             |
|                       |           BC056875           |          BC023760             |
|                       |           CR621331           |          BC049156             |
|                       |                              |          AK006492             |
|                       | *Seq. mapped in strand Minus*| *Seq. mapped in strand Minus* |
|                       |           AK025070           |          BC029715             |
|                       |           AK024955           |          AK033594             |
|                       |           BC056252           |          BC061228             |
+-----------------------+------------------------------+-------------------------------+
|      #  168           | *Seq. mapped in strand Plus* | *Seq. mapped in strand Plus*  |
|                       |           BC065931           |            L47650             |
|                       |             U48361           |          AF481808             |
|                       |           BC007756           |          AF481809             |
|                       |           CR622165           |          BC029318             |
|                       |             X70991           |          BC029853             |
|                       |           CR603613           |          BC040202             |
|                       | *Seq. mapped in strand Minus*| *Seq. mapped in strand Minus* |
|                       |           BC075852           |          BC045139             |
|                       |           BC004973           |            U47543             |
|                       |           BC005823           |          AK034709             |
|                       |           CR606877           |                               |
|                       |             U16031           |                               |
|                       |           BT007380           |                               |
+-----------------------+------------------------------+-------------------------------+
|      #  169           | *Seq. mapped in strand Plus* | *Seq. mapped in strand Plus*  |
|                       |             D63478           |          AK077215             |
|                       |           AJ243668           |          AK017833             |
|                       |           AK124294           |          AK034749             |
|                       |           BC003170           |          BC018438             |
|                       |           AJ243670           |          AK051238             |
|                       |           CR598011           |          BC027190             |
|                       |           AJ243669           |          AY533135             |
|                       |           CR617355           |          BC022598             |
|                       |                              |          AK009362             |
|                       | *Seq. mapped in strand Minus*| *Seq. mapped in strand Minus* |
|                       |           BC001377           |          AK016538             |
|                       |           AF077036           |          BC023906             |
|                       |           AJ243665           |          BC029075             |
|                       |           AJ243664           |          AK048286             |
|                       |           BC014391           |          AK050956             |
|                       |           CR597953           |          BC050910             |
|                       |           CR606555           |          AK036526             |
|                       |           CR626375           |          AK031934             |
|                       |           CR606853           |          AK122210             |
|                       |           AJ243663           |          AK079895             |
|                       |           AL117463           |          AK088702             |
|                       |           BC006514           |          AF276967             |
|                       |           BC000152           |          AK086314             |
|                       |           CR607212           |          AK030218             |
|                       |           CR590700           |          AK051093             |
|                       |           CR594201           |          AK082804             |
|                       |           CR605126           |          AK014274             |
|                       |           CR617350           |          AK040551             |
|                       |           CR616404           |                               |
|                       |           CR597803           |                               |
|                       |           CR610380           |                               |
|                       |           CR599474           |                               |
|                       |           CR606449           |                               |
|                       |           CR615706           |                               |
|                       |           CR605838           |                               |
|                       |           CR608586           |                               |
|                       |           CR606320           |                               |
|                       |           CR614553           |                               |
|                       |           CR609329           |                               |
|                       |           CR611597           |                               |
|                       |           CR598243           |                               |
|                       |           CR601087           |                               |
|                       |           CR624910           |                               |
|                       |           AF529365           |                               |
|                       |           CR457131           |                               |
|                       |           CR613431           |                               |
|                       |           BC008306           |                               |
+-----------------------+------------------------------+-------------------------------+
|      #  170           | *Seq. mapped in strand Plus* | *Seq. mapped in strand Plus*  |
|                       |           AK057730           |          AF412300             |
|                       |           CR616971           |          AF412298             |
|                       |           BC071769           |          AK075835             |
|                       |           CR601350           |          AK015530             |
|                       |           CR593194           |          AF412299             |
|                       |           AK095164           |          AK046179             |
|                       |                              |          BC021522             |
|                       | *Seq. mapped in strand Minus*| *Seq. mapped in strand Minus* |
|                       |           BC010502           |          BC014700             |
|                       |           CR625034           |          BC031623             |
+-----------------------+------------------------------+-------------------------------+
|      #  171           | *Seq. mapped in strand Plus* | *Seq. mapped in strand Plus*  |
|                       |           AK000780           |          BC013093             |
|                       |           AF367998           |          BC031772             |
|                       |           AF309777           |          AF315511             |
|                       |           BC049210           |          AK018125             |
|                       |           CR617202           |                               |
|                       |           AK092293           |                               |
|                       |           CR623235           |                               |
|                       | *Seq. mapped in strand Minus*| *Seq. mapped in strand Minus* |
|                       |           AF282594           |            D88769             |
|                       |           BC008438           |          AK032170             |
|                       |           AF087851           |                               |
|                       |           AF282626           |                               |
|                       |           BC005346           |                               |
|                       |           CR605027           |                               |
|                       |           CR622283           |                               |
+-----------------------+------------------------------+-------------------------------+
|      #  172           | *Seq. mapped in strand Plus* | *Seq. mapped in strand Plus*  |
|                       |           BC019239           |          AK028489             |
|                       |           BC025376           |          AK038086             |
|                       |           BC053896           |          AK038897             |
|                       |           BC017941           |          AK010315             |
|                       |           AK097260           |          BC023086             |
|                       |                              |          AF155354             |
|                       | *Seq. mapped in strand Minus*| *Seq. mapped in strand Minus* |
|                       |           BC009569           |          BC025100             |
|                       |           BC006488           |          AK010914             |
|                       |           AK056069           |          AK007629             |
|                       |           AK000985           |          AK078993             |
|                       |           AK001283           |          AK021391             |
|                       |           CR590535           |                               |
|                       |           AF156778           |                               |
+-----------------------+------------------------------+-------------------------------+
|      #  173           | *Seq. mapped in strand Plus* | *Seq. mapped in strand Plus*  |
|                       |           BC016351           |          AB012108             |
|                       |             X79444           |          BC030177             |
|                       |           CR608292           |            X99394             |
|                       |           BC004922           |                               |
|                       |           CR603245           |                               |
|                       |           AK130973           |                               |
|                       |           CR603254           |                               |
|                       | *Seq. mapped in strand Minus*| *Seq. mapped in strand Minus* |
|                       |           AL110193           |          AK044983             |
|                       |           BC010579           |          AK036945             |
|                       |           BC046133           |          AK046148             |
|                       |           BC063644           |                               |
|                       |           BC033677           |                               |
|                       |           BC056890           |                               |
|                       |           BC039590           |                               |
|                       |           CR606465           |                               |
|                       |           CR599269           |                               |
|                       |           CR612717           |                               |
|                       |           CR616507           |                               |
|                       |           CR600459           |                               |
|                       |           CR623306           |                               |
|                       |           AF161459           |                               |
+-----------------------+------------------------------+-------------------------------+
|      #  174           | *Seq. mapped in strand Plus* | *Seq. mapped in strand Plus*  |
|                       |           AF442495           |          AK052640             |
|                       |           BC028074           |          AB055902             |
|                       |           BC008672           |          AK048146             |
|                       |           BC036123           |          BC056368             |
|                       |           AK023221           |          BC058082             |
|                       |           AY055003           |                               |
|                       |           AY055004           |                               |
|                       |           AK124239           |                               |
|                       |           CR624449           |                               |
|                       |           AL833906           |                               |
|                       |           AK074340           |                               |
|                       | *Seq. mapped in strand Minus*| *Seq. mapped in strand Minus* |
|                       |           AB075843           |          BC006946             |
|                       |           AK055248           |          AK014966             |
|                       |           AY070019           |                               |
+-----------------------+------------------------------+-------------------------------+
|      #  175           | *Seq. mapped in strand Plus* | *Seq. mapped in strand Plus*  |
|                       |           BC003092           |          AK081983             |
|                       |             X74262           |          AJ251641             |
|                       |             X71810           |                               |
|                       |           BC053904           |                               |
|                       |           BC075836           |                               |
|                       |           BT007309           |                               |
|                       |           BC015123           |                               |
|                       |           CR600272           |                               |
|                       |           CR611583           |                               |
|                       |           AK054859           |                               |
|                       |           AK056550           |                               |
|                       |           AK056356           |                               |
|                       |           AK125399           |                               |
|                       | *Seq. mapped in strand Minus*| *Seq. mapped in strand Minus* |
|                       |           AK024707           |          AK082419             |
|                       |                              |            U35141             |
+-----------------------+------------------------------+-------------------------------+
|      #  176           | *Seq. mapped in strand Plus* | *Seq. mapped in strand Plus*  |
|                       |           BC036434           |          AF513619             |
|                       |           BC027854           |          AK011520             |
|                       |           AB000450           |          AK008605             |
|                       |           AJ512204           |          AK020981             |
|                       |           AY228367           |          AK076017             |
|                       |           AY228368           |          AK018018             |
|                       |           AY228369           |          AK011752             |
|                       |           AY228370           |          BC066181             |
|                       |           AY228371           |          BC032876             |
|                       |           AY228372           |                               |
|                       |           AK058199           |                               |
|                       |           BC021663           |                               |
|                       |           CR622767           |                               |
|                       |           CR605412           |                               |
|                       |           CR624256           |                               |
|                       | *Seq. mapped in strand Minus*| *Seq. mapped in strand Minus* |
|                       |           BC037570           |          AK016196             |
|                       |           AK001197           |          AK008982             |
|                       |           BC009042           |          BC013520             |
|                       |           BC054517           |          AK012664             |
|                       |           CR594145           |          AF513620             |
|                       |           CR597729           |                               |
|                       |           BC036673           |                               |
+-----------------------+------------------------------+-------------------------------+
|      #  177           | *Seq. mapped in strand Plus* | *Seq. mapped in strand Plus*  |
|                       |           AK057447           |          AK045103             |
|                       |           AK097822           |          AF347694             |
|                       |           AL832764           |          BC034104             |
|                       |           BC003154           |          AF230385             |
|                       |             U18543           |          AK007783             |
|                       |           CR610656           |          AK028316             |
|                       |           CR605953           |                               |
|                       |           CR597365           |                               |
|                       |           AK097239           |                               |
|                       |           CR609070           |                               |
|                       |           AK021992           |                               |
|                       | *Seq. mapped in strand Minus*| *Seq. mapped in strand Minus* |
|                       |           BC029272           |          AF116573             |
|                       |           AK024064           |                               |
|                       |           AK127350           |                               |
|                       |           AF116574           |                               |
|                       |           AB014534           |                               |
|                       |           BC018759           |                               |
|                       |           CR618241           |                               |
+-----------------------+------------------------------+-------------------------------+
|      #  178           | *Seq. mapped in strand Plus* | *Seq. mapped in strand Plus*  |
|                       |           AY033141           |          BC062957             |
|                       |           AK055245           |          AK014835             |
|                       |           BC040359           |          AK043248             |
|                       |           CR600710           |            U49430             |
|                       |           BC022062           |          AK033842             |
|                       |           AK056575           |          AK080701             |
|                       |           AL832735           |          AK086999             |
|                       |           CR622327           |                               |
|                       |           AL833878           |                               |
|                       |           BC016901           |                               |
|                       |           AK026357           |                               |
|                       | *Seq. mapped in strand Minus*| *Seq. mapped in strand Minus* |
|                       |           BX647327           |          BC058235             |
|                       |           BC039239           |          AK034377             |
|                       |             M13536           |          BC049350             |
|                       |             X69706           |          BC020105             |
|                       |           AB073614           |          AK036663             |
|                       |             M13699           |          AF393747             |
|                       |           AK095290           |          BC038087             |
|                       |           BC061702           |          AF393780             |
|                       |             X04138           |          AK079896             |
|                       |             X04137           |          AK049204             |
|                       |           BC070304           |                               |
+-----------------------+------------------------------+-------------------------------+
|      #  179           | *Seq. mapped in strand Plus* | *Seq. mapped in strand Plus*  |
|                       |           AK125161           |          AK050339             |
|                       |           AL833591           |          AK046919             |
|                       |           AY032844           |          BC050784             |
|                       |           CR599776           |          BC057068             |
|                       |           BC010708           |          AK015327             |
|                       |           AF444143           |          AK083918             |
|                       |           AF131801           |          AK077004             |
|                       |           CR457153           |          BC027570             |
|                       | *Seq. mapped in strand Minus*| *Seq. mapped in strand Minus* |
|                       |           CR618486           |          AJ292968             |
|                       |           BC036013           |          AK034983             |
|                       |             U77129           |          AK006655             |
|                       |           BC013159           |          BC019377             |
|                       |             Z25426           |          AK089407             |
|                       |           AL833378           |                               |
|                       |           AK023071           |                               |
|                       |           AF088000           |                               |
|                       |           AJ292969           |                               |
|                       |           BC006385           |                               |
|                       |           AK095903           |                               |
|                       |           BC020537           |                               |
|                       |           AK021500           |                               |
|                       |           CR457297           |                               |
+-----------------------+------------------------------+-------------------------------+
|      #  180           | *Seq. mapped in strand Plus* | *Seq. mapped in strand Plus*  |
|                       |           BC030618           |          BC021950             |
|                       |           CR625723           |          BC021605             |
|                       |                              |          BC011259             |
|                       | *Seq. mapped in strand Minus*| *Seq. mapped in strand Minus* |
|                       |           BC020750           |          AK042089             |
|                       |           CR609930           |          AF217003             |
|                       |             J05037           |          AK043608             |
+-----------------------+------------------------------+-------------------------------+
|      #  181           | *Seq. mapped in strand Plus* | *Seq. mapped in strand Plus*  |
|                       |           BC023327           |          BC034126             |
|                       |           BC032794           |                               |
|                       |           BC041581           |                               |
|                       |           BC062558           |                               |
|                       |           AB019692           |                               |
|                       |           CR614288           |                               |
|                       | *Seq. mapped in strand Minus*| *Seq. mapped in strand Minus* |
|                       |           BC009280           |          AK004475             |
|                       |           BC048251           |          BC021432             |
|                       |           AK027430           |          AK087676             |
|                       |           AK075332           |          AK013074             |
+-----------------------+------------------------------+-------------------------------+
|      #  182           | *Seq. mapped in strand Plus* | *Seq. mapped in strand Plus*  |
|                       |             L76702           |          AK010863             |
|                       |           BC001175           |          AK008305             |
|                       |           CR590993           |          AK008194             |
|                       |             D78360           |          AK010962             |
|                       |           BC010692           |          AK012617             |
|                       |           BC001095           |          AK010904             |
|                       |           AK122701           |          AK005369             |
|                       |           AB000635           |          AK005106             |
|                       |           AB000634           |          AK011189             |
|                       |           CR625285           |            L10401             |
|                       |                              |            M27938             |
|                       |                              |            D17341             |
|                       |                              |          BC013344             |
|                       | *Seq. mapped in strand Minus*| *Seq. mapped in strand Minus* |
|                       |           CR606344           |          AY338234             |
|                       |             L10400           |            X81059             |
|                       |             M27937           |          BC010716             |
|                       |           BC001754           |          AB053464             |
|                       |           BT009831           |          BC009163             |
|                       |                              |          AF316616             |
|                       |                              |                               |
|                       |                              |                               |
|                       |                              |                               |
|                       |                              |                               |
|                       |                              |                               |
|                       |                              |                               |
|                       |                              |                               |
+-----------------------+------------------------------+-------------------------------+
|      #  183           | *Seq. mapped in strand Plus* | *Seq. mapped in strand Plus*  |
|                       |           CR622228           |          AK038369             |
|                       |           CR595766           |          AK078171             |
|                       |           CR593472           |          AK050789             |
|                       |           BC030144           |                               |
|                       |           CR605824           |                               |
|                       |           CR607194           |                               |
|                       |           BC021051           |                               |
|                       |             D14660           |                               |
|                       |           AL157503           |                               |
|                       |           AK024792           |                               |
|                       | *Seq. mapped in strand Minus*| *Seq. mapped in strand Minus* |
|                       |           BC064559           |          AK030793             |
|                       |           AB026911           |          BC061064             |
|                       |           AK097564           |          BC020315             |
+-----------------------+------------------------------+-------------------------------+
|      #  184           | *Seq. mapped in strand Plus* | *Seq. mapped in strand Plus*  |
|                       |           BC036549           |            Z36885             |
|                       |           AK091896           |          BC004798             |
|                       |           AK090831           |          BC041679             |
|                       |           AY358107           |          AK041964             |
|                       |           AL713676           |          AK009299             |
|                       |           AK057733           |                               |
|                       |           CR599236           |                               |
|                       |           AK127321           |                               |
|                       |           AL110125           |                               |
|                       |           BC016377           |                               |
|                       | *Seq. mapped in strand Minus*| *Seq. mapped in strand Minus* |
|                       |           BC040145           |          AK086589             |
|                       |             M85165           |          BC058790             |
|                       |             M85164           |          AK044667             |
|                       |           BC063676           |          AK048692             |
|                       |           CR623173           |          AK038882             |
+-----------------------+------------------------------+-------------------------------+
|      #  185           | *Seq. mapped in strand Plus* | *Seq. mapped in strand Plus*  |
|                       |           AY039759           |          AK005989             |
|                       |           BC012142           |          AK076558             |
|                       |           AK127836           |          BC006865             |
|                       | *Seq. mapped in strand Minus*| *Seq. mapped in strand Minus* |
|                       |           AK127433           |          BC003810             |
|                       |           CR601036           |          AB088357             |
|                       |           AK125449           |          AK004157             |
|                       |           CR614510           |          BC064071             |
|                       |           BC001312           |          BC056636             |
|                       |           AK131234           |                               |
|                       |           CR624923           |                               |
|                       |           CR620116           |                               |
|                       |             U79278           |                               |
|                       |           CR590867           |                               |
|                       |           CR595109           |                               |
|                       |           CR608056           |                               |
|                       |           CR606263           |                               |
|                       |           CR595577           |                               |
|                       |           CR622384           |                               |
|                       |           CR595714           |                               |
|                       |           CR607388           |                               |
|                       |           CR606166           |                               |
|                       |           CR617062           |                               |
|                       |           CR599723           |                               |
|                       |           CR608933           |                               |
|                       |           CR593821           |                               |
|                       |           CR601561           |                               |
|                       |           CR594255           |                               |
|                       |           CR618905           |                               |
|                       |           CR602748           |                               |
|                       |           CR602648           |                               |
+-----------------------+------------------------------+-------------------------------+
|      #  186           | *Seq. mapped in strand Plus* | *Seq. mapped in strand Plus*  |
|                       |             X97795           |          BC022976             |
|                       |           CR594300           |          BC024940             |
|                       |           CR591799           |          AK052762             |
|                       |           AK097978           |          BC071258             |
|                       |                              |          BC005765             |
|                       |                              |          BC007169             |
|                       |                              |          AK028000             |
|                       | *Seq. mapped in strand Minus*| *Seq. mapped in strand Minus* |
|                       |             X86018           |          AK030753             |
|                       |           BC004953           |            X97796             |
|                       |           BX537366           |          BC021643             |
|                       |           AK125827           |          AK088876             |
|                       |           AF370430           |                               |
|                       |           AK024051           |                               |
|                       |           BC042988           |                               |
+-----------------------+------------------------------+-------------------------------+
|      #  187           | *Seq. mapped in strand Plus* | *Seq. mapped in strand Plus*  |
|                       |           BC025779           |          AK029866             |
|                       |           BC032755           |          AK017063             |
|                       |           AB022192           |          BC025555             |
|                       |           AF048755           |          AK087766             |
|                       |           BC067090           |          AK012737             |
|                       |           BC020547           |          AK088562             |
|                       |           BC040953           |          AK028368             |
|                       |           CR609626           |                               |
|                       |           BC035584           |                               |
|                       |             U71374           |                               |
|                       | *Seq. mapped in strand Minus*| *Seq. mapped in strand Minus* |
|                       |           AL832208           |          AK031710             |
|                       |           AK056874           |          AK031975             |
|                       |           BC065197           |          AK029336             |
|                       |                              |          AK032650             |
|                       |                              |          AK029099             |
|                       |                              |          AJ304506             |
|                       |                              |          BC023683             |
+-----------------------+------------------------------+-------------------------------+
|      #  188           | *Seq. mapped in strand Plus* | *Seq. mapped in strand Plus*  |
|                       |           BC027732           |          BC003247             |
|                       |             U54999           |          AK090092             |
|                       |           CR456786           |                               |
|                       |           AY136740           |                               |
|                       |           CR602234           |                               |
|                       |           AK000053           |                               |
|                       | *Seq. mapped in strand Minus*| *Seq. mapped in strand Minus* |
|                       |           AB018304           |          AY081187             |
|                       |           AK090563           |          AK018084             |
|                       |           CR622878           |          BC021308             |
|                       |           BC002939           |          AK040996             |
|                       |           CR606058           |                               |
|                       |           AB052915           |                               |
|                       |           AB052916           |                               |
|                       |           AB052917           |                               |
|                       |           AB052918           |                               |
+-----------------------+------------------------------+-------------------------------+
|      #  189           | *Seq. mapped in strand Plus* | *Seq. mapped in strand Plus*  |
|                       |           BC064332           |          BC030394             |
|                       |           AJ298844           |            D83038             |
|                       |           BX648257           |          BC014718             |
|                       |             M55983           |                               |
|                       |           BC029437           |                               |
|                       |           AK126850           |                               |
|                       |           BX648480           |                               |
|                       |           AK095412           |                               |
|                       |           AK098732           |                               |
|                       | *Seq. mapped in strand Minus*| *Seq. mapped in strand Minus* |
|                       |           AF043254           |          BC022912             |
|                       |           AK057620           |          AK010341             |
|                       |           BC000406           |          BC006685             |
|                       |           BC002994           |          BC006864             |
|                       |           AF154108           |          AK002409             |
|                       |           BC023585           |          AK088471             |
|                       |           AK093344           |                               |
|                       |             U12595           |                               |
|                       |           BC001455           |                               |
|                       |           BC018950           |                               |
|                       |           CR612283           |                               |
|                       |           CR623319           |                               |
|                       |           BC035759           |                               |
+-----------------------+------------------------------+-------------------------------+
|      #  190           | *Seq. mapped in strand Plus* | *Seq. mapped in strand Plus*  |
|                       |           CR621164           |            D14849             |
|                       |           BC028119           |          AK013048             |
|                       |                              |          BC046624             |
|                       |                              |          AK083178             |
|                       | *Seq. mapped in strand Minus*| *Seq. mapped in strand Minus* |
|                       |           AK094529           |          AK032490             |
|                       |           AK057542           |          BC006667             |
|                       |           BC034991           |          AB000619             |
|                       |           AK002084           |          BC031504             |
|                       |           BC031046           |          AK008505             |
|                       |           BC012554           |          AK012189             |
+-----------------------+------------------------------+-------------------------------+
```
