# Supplementary material for: Sense-antisense pairs in mammals: functional and evolutionary considerations
Source: Genome Biol. 2007 Mar 19;8(3):R40. doi: 10.1186/gb-2007-8-3-r40 (PMC1868933; doi:10.1186/gb-2007-8-3-r40)
Supplement: Additional data file 5 — All S-AS pairs conserved between human and mouse using the three strategies described in the text. [file gb-2007-8-3-r40-S5.zip › Additional_data_file5_1136_pairs.doc]

```
Additional data file 5: 1136 S-AS pairs conserved between human and mouse.
+-----------------------+------------------------------+-------------------------------+
|   Conserved pairs     |      Human S-AS pairs        |       Mouse S-AS pairs        |
+-----------------------+------------------------------+-------------------------------+
|      #    1           | *Seq. mapped in strand Plus* | *Seq. mapped in strand Plus*  |
|                       |           AV728284           |          BB822573             |
|                       |           BM983949           |          AW227535             |
|                       |                              |          BB822138             |
|                       |                              |          BY376085             |
|                       |                              |          BB828067             |
|                       |                              |          CD774074             |
|                       |                              |          BF459286             |
|                       |                              |          BF459238             |
|                       |                              |          AA798359             |
|                       | *Seq. mapped in strand Minus*| *Seq. mapped in strand Minus* |
|                       |           CA449010           |          BF458202             |
|                       |           AI375381           |          AW124244             |
|                       |           BM971330           |          BU758260             |
|                       |           BM984237           |          BC006928             |
|                       |           BM144283           |          AK004913             |
|                       |           BM144782           |          BQ920054             |
|                       |           AI948856           |          BQ899565             |
|                       |           AA806814           |          BF533887             |
|                       |           AA883294           |          BU614125             |
|                       |           AI434454           |          BQ958299             |
|                       |           AA824496           |          CA320599             |
|                       |           AW519264           |          AA530553             |
|                       |           AI287385           |          BE533227             |
|                       |           BF431125           |          BF168150             |
|                       |           AW139327           |          AA087591             |
|                       |           AI200974           |          BG861572             |
|                       |           AA806458           |          CB840148             |
|                       |           AI584001           |          BI155323             |
|                       |           AI452808           |          CD354335             |
|                       |           BM830482           |          BQ443382             |
|                       |           BE044627           |          CN668390             |
|                       |           AI862690           |          CA315220             |
|                       |           BU681834           |          CD353720             |
|                       |           CA306253           |          CA317507             |
|                       |           CA309286           |          AI020881             |
|                       |           CA425583           |          AW240211             |
|                       |           CA502787           |          CF730285             |
|                       |           AI097638           |          BY115367             |
|                       |           CA444340           |          BY114717             |
|                       |           CA503247           |          BY116050             |
|                       |           BQ015548           |          CF906385             |
|                       |           BQ024917           |          CN666877             |
|                       |           BU625613           |          CK393118             |
|                       |           BU726448           |                               |
|                       |           CA307402           |                               |
|                       |           BM995867           |                               |
|                       |           BM989142           |                               |
|                       |           BU607308           |                               |
|                       |           CA437087           |                               |
|                       |           BU145485           |                               |
|                       |           AI807146           |                               |
|                       |           BC015026           |                               |
|                       |             D31884           |                               |
|                       |           AI088037           |                               |
|                       |           CR596979           |                               |
|                       |           BX407228           |                               |
|                       |           AU131631           |                               |
|                       |           BX409521           |                               |
|                       |           BX456263           |                               |
|                       |           BM800315           |                               |
|                       |           BP350913           |                               |
|                       |           AL044250           |                               |
|                       |           BM987223           |                               |
|                       |           BG822958           |                               |
|                       |           BE907104           |                               |
|                       |           CB120740           |                               |
|                       |           AW204963           |                               |
|                       |           CA314885           |                               |
|                       |           CA309956           |                               |
|                       |           AA664246           |                               |
|                       |           BG231093           |                               |
|                       |           BP248966           |                               |
|                       |           BP236420           |                               |
|                       |           CR456473           |                               |
|                       |           BQ231977           |                               |
|                       |           BQ929893           |                               |
|                       |           BP314723           |                               |
|                       |           CV023373           |                               |
|                       |           CR456923           |                               |
|                       |           BX409519           |                               |
|                       |           BU192424           |                               |
|                       |           AL039248           |                               |
|                       |           BP200238           |                               |
|                       |           BI256586           |                               |
|                       |           BG708486           |                               |
|                       |           BP257066           |                               |
|                       |           BE786723           |                               |
|                       |           BP378249           |                               |
|                       |           BP242008           |                               |
|                       |           CN307062           |                               |
|                       |           CN307071           |                               |
|                       |           BX419805           |                               |
|                       |           CN307068           |                               |
|                       |           CN307058           |                               |
|                       |           BU542301           |                               |
|                       |           BP232411           |                               |
|                       |           CN307069           |                               |
|                       |           BP256683           |                               |
|                       |           CN307065           |                               |
|                       |           BM147123           |                               |
|                       |           CN307063           |                               |
+-----------------------+------------------------------+-------------------------------+
|      #    2           | *Seq. mapped in strand Plus* | *Seq. mapped in strand Plus*  |
|                       |           BC063292           |          AK044537             |
|                       |           AF250310           |          AK033358             |
|                       |           AF205437           |          AK040738             |
|                       |           AI827927           |          AK041212             |
|                       |           AL832388           |          AK028626             |
|                       |           CK826341           |          AK080228             |
|                       |           BU787759           |          BC006800             |
|                       |           AU135970           |          AF358866             |
|                       |             W24914           |                               |
|                       |           BI005380           |                               |
|                       |           BE871030           |                               |
|                       |           BF770856           |                               |
|                       |           AJ000480           |                               |
|                       |           CN258650           |                               |
|                       |           AW378352           |                               |
|                       |           AU125722           |                               |
|                       |           BU521465           |                               |
|                       |           BF171203           |                               |
|                       |           AI867255           |                               |
|                       |           BF062780           |                               |
|                       |           BC012441           |                               |
|                       |           AA503709           |                               |
|                       |           AK001319           |                               |
|                       |           AK022787           |                               |
|                       |           CR608019           |                               |
|                       |           CA431969           |                               |
|                       |           BM677047           |                               |
|                       |           BM678075           |                               |
|                       |           BM682979           |                               |
|                       |           BU682834           |                               |
|                       |           CA423009           |                               |
|                       |           BU787484           |                               |
|                       |           BQ001480           |                               |
|                       |           AW082309           |                               |
|                       |           CA440767           |                               |
|                       |           AW629523           |                               |
|                       |           AI340243           |                               |
|                       |           AI275546           |                               |
|                       |           AI765186           |                               |
|                       |           AA824445           |                               |
|                       |           AW770719           |                               |
|                       |           AA975535           |                               |
|                       |           AA807615           |                               |
|                       |           AI373497           |                               |
|                       |           AV709849           |                               |
|                       |           AI253126           |                               |
|                       |           AI766962           |                               |
|                       |           AW193471           |                               |
|                       |           BF059749           |                               |
|                       |           AI926918           |                               |
|                       |           AI093994           |                               |
|                       |           BU743267           |                               |
|                       |           AI521175           |                               |
|                       |           AI926386           |                               |
|                       |           AI612991           |                               |
|                       |           AW779202           |                               |
|                       | *Seq. mapped in strand Minus*| *Seq. mapped in strand Minus* |
|                       |           CA312928           |          AK053706             |
|                       |           BM990654           |                               |
+-----------------------+------------------------------+-------------------------------+
|      #    3           | *Seq. mapped in strand Plus* | *Seq. mapped in strand Plus*  |
|                       |           BG722486           |          BB867031             |
|                       |           BP225635           |          BF789828             |
|                       |           BP229856           |          BC026802             |
|                       |           BG719698           |          BQ571832             |
|                       |           AK075256           |          CF531688             |
|                       |           CD624865           |          BQ944453             |
|                       |           BQ705880           |          BU514080             |
|                       |           CR456493           |          CA316390             |
|                       |           BC010917           |          BY231571             |
|                       |           BI553971           |          BY196239             |
|                       |           BG703792           |          BY177678             |
|                       |           BI560528           |          BY188924             |
|                       |           BF673917           |          BY231818             |
|                       |           BG485334           |          BY180476             |
|                       |           BC029388           |          BY174363             |
|                       |           BM833916           |          BY016667             |
|                       |           BP211796           |          BY750326             |
|                       |           BM834034           |          BY188873             |
|                       |           AU280190           |          BY174519             |
|                       |           BP362889           |          BY175545             |
|                       |           BI549689           |          BY177692             |
|                       |           BX421475           |          BY202012             |
|                       |           BX407098           |          BY188204             |
|                       |           BX346447           |          BB869792             |
|                       |           BX332277           |          BY010250             |
|                       |           CR609702           |          BY187729             |
|                       |           BQ424789           |          BY062502             |
|                       |           BQ929556           |          BY022544             |
|                       |           BP212186           |          BY029311             |
|                       |           BP305372           |          BY031747             |
|                       |           BI552572           |          BY188718             |
|                       |           CD624868           |          BB848829             |
|                       |           CD624869           |          BB857748             |
|                       |           CA392649           |          BG871029             |
|                       |           BI754917           |          BC054422             |
|                       |           BC044629           |          BY179524             |
|                       |           BM927459           |          BY071794             |
|                       |           AF190862           |          BY204158             |
|                       |           CD624876           |          BY245587             |
|                       |           CD624877           |          BY343319             |
|                       |           BG760236           |          BB868315             |
|                       |           CN403708           |          BB872157             |
|                       |           CN403715           |          BY182466             |
|                       |           BI761194           |          BY743077             |
|                       |           BI764099           |          BY184659             |
|                       |           BX387350           |          BY206942             |
|                       |           BX355320           |          BY275200             |
|                       |           BM463420           |          BY241798             |
|                       |           BI547502           |          BY012603             |
|                       |           BI764718           |          BY177681             |
|                       |           AF233521           |          BY287389             |
|                       |           CD624866           |          BY347224             |
|                       |           CD624867           |          BY327080             |
|                       |           CD624870           |          BY352065             |
|                       |           CD624871           |          BY100352             |
|                       |           BG029591           |          BY325194             |
|                       |           AF218584           |          BY349140             |
|                       |           AL038950           |          BY349887             |
|                       |           AL038926           |          BY352693             |
|                       |           BI839039           |          BY782204             |
|                       |           BU155634           |          BY351013             |
|                       |           BX363775           |          BY348285             |
|                       |           CD624875           |          BY207624             |
|                       |           CN297537           |          BY196820             |
|                       |           BX345253           |          BY781353             |
|                       |           BX334189           |          BY349685             |
|                       |           BX371274           |          BY197982             |
|                       |           CD624872           |          BY102166             |
|                       |           CD624874           |          BY311498             |
|                       |           CA487594           |          BY080678             |
|                       |           BG334784           |          BY179195             |
|                       |           BI559673           |          BY204143             |
|                       |           BI560180           |          BY748810             |
|                       |           BP310779           |          BY748363             |
|                       |           CB988136           |          BY342004             |
|                       |           CD109809           |          BY181729             |
|                       |           AK092417           |          BY177819             |
|                       |           AK122898           |          BY289487             |
|                       |           BI831683           |          BY196007             |
|                       |           CN268396           |          BY194716             |
|                       |           BQ345740           |          BY085257             |
|                       |           BG762444           |          BY198458             |
|                       |           BG767666           |          BY087921             |
|                       |           CO246629           |          BY004641             |
|                       |           BE410966           |          BY252746             |
|                       |           BU172729           |          BY263317             |
|                       |           BQ673334           |          BY252865             |
|                       |           BU167069           |          BY094057             |
|                       |           BE276611           |          BB872794             |
|                       |           BG762161           |          BY095744             |
|                       |           AW408001           |          BY035004             |
|                       |           AL110219           |          BY352406             |
|                       |           AW391591           |          BY194272             |
|                       |           AW665787           |          BY104036             |
|                       |           BG251016           |          BY210526             |
|                       |           BE044434           |          BY207616             |
|                       |           CN403712           |          BY232245             |
|                       |           CD673213           |          BY223775             |
|                       |           CN403707           |          BY182338             |
|                       |           CD624880           |          BY229901             |
|                       |           CD624881           |          BY229808             |
|                       |           CN403705           |          BB848878             |
|                       |           BX428381           |          BY154934             |
|                       |           BQ368684           |          BB849881             |
|                       |           CN403709           |          BY754183             |
|                       |           AI902182           |          BB645396             |
|                       |           AI902187           |          AK080881             |
|                       |           BI113938           |          BY148127             |
|                       |           BM474932           |          BY146236             |
|                       |           AI907800           |          BY300223             |
|                       |           BX353034           |          BY195667             |
|                       |           CR596724           |          BB870049             |
|                       |           BX430461           |          BY278200             |
|                       |           BM909345           |          BY274968             |
|                       |           BU182195           |          BB858521             |
|                       |           BQ710624           |          BB844662             |
|                       |           CD579771           |          AI225490             |
|                       |           BM007654           |          BE290996             |
|                       |           CD244215           |          CF725654             |
|                       |           AK095427           |          BQ444367             |
|                       |           AV689644           |          BY355714             |
|                       |           BG479149           |          AW762029             |
|                       |           CB149195           |          BI555618             |
|                       |           BM006432           |          CF540178             |
|                       |           CD624878           |          CD354307             |
|                       |           CD624879           |          BU709297             |
|                       |           BX409630           |          BB863058             |
|                       |           BX409232           |          CA751607             |
|                       |           BE732148           |          BQ179604             |
|                       |           BE796475           |          BI156029             |
|                       |           BX428494           |          BM950115             |
|                       |           BQ049594           |          BQ573463             |
|                       |           BE145593           |          BQ573310             |
|                       |           BX328256           |          BG976968             |
|                       |           BX388285           |          BY548706             |
|                       |           AI902338           |          BF783129             |
|                       |           BX367473           |          BU053683             |
|                       |           BQ066863           |          BU053666             |
|                       |           BX325513           |          BY599393             |
|                       |           BX326834           |          BI873169             |
|                       |           BG341777           |          BY540633             |
|                       |           BG340719           |          BY549455             |
|                       |           BG994494           |          BF535718             |
|                       |           BF769209           |          AV136947             |
|                       |           BQ327948           |          BY589778             |
|                       |           CD624884           |          BF385951             |
|                       |           CD624885           |          CK129259             |
|                       |           BM014237           |          BE303418             |
|                       |           BX347718           |          AW213146             |
|                       |           AL039460           |          BG965041             |
|                       |           CB152208           |          BG972515             |
|                       |           BC000538           |          BQ950347             |
|                       |           BI771475           |          BF117363             |
|                       |           BX347819           |          BG872781             |
|                       |           AL043025           |          BF788687             |
|                       |           CN403716           |          CB205744             |
|                       |           BI768767           |          BQ954839             |
|                       |           BG178806           |          AW229606             |
|                       |           BM830064           |          BG083371             |
|                       |           CN403713           |          BI695083             |
|                       |           BQ722504           |          BF119024             |
|                       |           AA985250           |          AA509812             |
|                       |           CN403710           |          CK618272             |
|                       |           BF878125           |            W35960             |
|                       |           BG256725           |            W35963             |
|                       |           AW606321           |          BI692974             |
|                       |           BG956127           |          BI649598             |
|                       |           BG030268           |          BE859821             |
|                       |           BG009581           |          BQ938556             |
|                       |           BE796160           |                               |
|                       |           BG011057           |                               |
|                       |           BI023559           |                               |
|                       |           BX325563           |                               |
|                       |           BM543641           |                               |
|                       |           BX389049           |                               |
|                       |           CD300460           |                               |
|                       |           CD359159           |                               |
|                       |           CD358757           |                               |
|                       |           CD300459           |                               |
|                       |           BF154663           |                               |
|                       |           BX388333           |                               |
|                       |           BP370488           |                               |
|                       |           CF146435           |                               |
|                       |           BX428554           |                               |
|                       |           BX325484           |                               |
|                       |           CB156443           |                               |
|                       |           CA454207           |                               |
|                       |           BI562704           |                               |
|                       |           BI562608           |                               |
|                       |           BX407436           |                               |
|                       |           BX409015           |                               |
|                       |           CN403711           |                               |
|                       |           BG177241           |                               |
|                       |             R09705           |                               |
|                       |           BI826660           |                               |
|                       |           BP299144           |                               |
|                       |           BM703359           |                               |
|                       |           BG912407           |                               |
|                       |           CD624882           |                               |
|                       |           CD624883           |                               |
|                       |           AA405939           |                               |
|                       |           BG696604           |                               |
|                       |           AW630864           |                               |
|                       |           BX457693           |                               |
|                       |           BG576858           |                               |
|                       |           BM796233           |                               |
|                       |           BX331582           |                               |
|                       |           BM853604           |                               |
|                       | *Seq. mapped in strand Minus*| *Seq. mapped in strand Minus* |
|                       |           BF026208           |          AW125311             |
|                       |           AI150569           |          BE980517             |
|                       |           BF516079           |                               |
|                       |           BF516281           |                               |
+-----------------------+------------------------------+-------------------------------+
|      #    4           | *Seq. mapped in strand Plus* | *Seq. mapped in strand Plus*  |
|                       |           CB991653           |          AK050732             |
|                       |           BC060859           |          BC062822             |
|                       |           BU146014           |                               |
|                       |           BI518789           |                               |
|                       |           CR616618           |                               |
|                       |           BM762591           |                               |
|                       |           BM820723           |                               |
|                       |           BM771506           |                               |
|                       |           BM842763           |                               |
|                       |           BM846269           |                               |
|                       |           AL539189           |                               |
|                       |           CR613339           |                               |
|                       |           BX367213           |                               |
|                       |           CR599986           |                               |
|                       |           BI194898           |                               |
|                       |           BC035511           |                               |
|                       |           AI582849           |                               |
|                       |           BQ684942           |                               |
|                       |           BF309200           |                               |
|                       |           BF126004           |                               |
|                       |           CN345252           |                               |
|                       |           AA278503           |                               |
|                       |           CF126547           |                               |
|                       |           BF083150           |                               |
|                       |           BG255481           |                               |
|                       |           CR596963           |                               |
|                       |           BU738572           |                               |
|                       |           AI337032           |                               |
|                       |           BU683174           |                               |
|                       |           BQ955499           |                               |
|                       |           CR590966           |                               |
|                       |           BU688185           |                               |
|                       |           CA427151           |                               |
|                       |           AI760616           |                               |
|                       |           CF529936           |                               |
|                       |           BM976734           |                               |
|                       |           BM670214           |                               |
|                       |           BU682539           |                               |
|                       |           CA415305           |                               |
|                       |           BU624459           |                               |
|                       |           BM671870           |                               |
|                       |           BQ447136           |                               |
|                       |           AI498586           |                               |
|                       |           BM974588           |                               |
|                       |           CA306628           |                               |
|                       |           BM970747           |                               |
|                       |           CA431592           |                               |
|                       |           BI868634           |                               |
|                       |           CA749037           |                               |
|                       |           BM665983           |                               |
|                       |           CA308035           |                               |
|                       |           BQ129366           |                               |
|                       |           CK823617           |                               |
|                       |           AA810692           |                               |
|                       |           AA807430           |                               |
|                       |             W74402           |                               |
|                       |           AI056736           |                               |
|                       |           AI088820           |                               |
|                       |           AA731524           |                               |
|                       |             W72878           |                               |
|                       |           AA768840           |                               |
|                       |           AA884042           |                               |
|                       |           CA433381           |                               |
|                       |           AA804415           |                               |
|                       |           AA643055           |                               |
|                       |           AA279056           |                               |
|                       | *Seq. mapped in strand Minus*| *Seq. mapped in strand Minus* |
|                       |           AI990551           |          BC052554             |
|                       |           BM710426           |                               |
+-----------------------+------------------------------+-------------------------------+
|      #    5           | *Seq. mapped in strand Plus* | *Seq. mapped in strand Plus*  |
|                       |           BU183491           |          CB595016             |
|                       |           BG719512           |          AA212810             |
|                       |           BM007430           |          BB840116             |
|                       |           CR605227           |          BY289444             |
|                       |           BF979733           |          BY013850             |
|                       |           BG720285           |          BY283310             |
|                       |           CB990732           |          AI528319             |
|                       |           CB997757           |          BY027982             |
|                       |           CD704081           |          BY704779             |
|                       |           BX405274           |          AK004555             |
|                       |           AI903894           |          AI593879             |
|                       |           BM149702           |          BI652872             |
|                       |           BQ219605           |          BM461314             |
|                       |           CD697554           |          AA982160             |
|                       |             Z41981           |          BI733379             |
|                       |           AF151047           |          BI662226             |
|                       |           AF161445           |          BF142974             |
|                       |           AF212245           |          AA014917             |
|                       |             R13337           |          BE860172             |
|                       |           BC010359           |          CD808344             |
|                       |           CD690782           |          BU510126             |
|                       |           CD689007           |          AA024162             |
|                       |           BF691687           |          AI747791             |
|                       |           CB988698           |          AA656963             |
|                       |           BG528075           |          BY048554             |
|                       |           BI227210           |          CB195983             |
|                       |           CD709369           |          BY262117             |
|                       |           BG720426           |          BB867004             |
|                       |             H16532           |          CB570593             |
|                       |             H17763           |          BY350501             |
|                       |           BP197247           |          BY349836             |
|                       |           BP206149           |          BB851769             |
|                       |           BP344891           |          BY356012             |
|                       |           CD103616           |          AI785086             |
|                       |           BU928810           |          BU505730             |
|                       |           BP224990           |            W10873             |
|                       |           CD692521           |          BG518639             |
|                       |           BX508924           |          BC002238             |
|                       |           CD709921           |          AA472448             |
|                       |           BG426502           |          BE303129             |
|                       |           BG565668           |          BI695591             |
|                       |           BG623875           |          AI037609             |
|                       |           CD710680           |          CD807512             |
|                       |             R21494           |          BF137395             |
|                       |           BP193000           |          CD740900             |
|                       |           CD706284           |          AA390122             |
|                       |           CD701189           |          AA607719             |
|                       |           CD697764           |          CB203215             |
|                       |           CD699396           |          BE376835             |
|                       |           AK027591           |          BB769846             |
|                       |           BC005198           |          BG276437             |
|                       |           AL038768           |          BB765363             |
|                       |           AV717005           |          BB763625             |
|                       |           BM053010           |          AI256468             |
|                       |           BX417702           |            W41367             |
|                       |           BX449840           |          BU755323             |
|                       |           CR615396           |          BB785276             |
|                       |           BP255561           |          BB727855             |
|                       |           BQ716044           |          BY390847             |
|                       |           AL548991           |          AI835831             |
|                       |           CR596184           |          BE198562             |
|                       |           BQ434917           |          AI303795             |
|                       |           BQ051314           |          AI835067             |
|                       |           BG623933           |          BY377797             |
|                       |           BM750950           |          AI256182             |
|                       |           BG699094           |          AI841271             |
|                       |           BF575231           |          BY644878             |
|                       |           BU192171           |          BY667750             |
|                       |           BU186908           |          AI835694             |
|                       |           BF246715           |          AI462447             |
|                       |           BI519981           |          CB575597             |
|                       |           BP377897           |          AA543165             |
|                       |           AV728273           |          BY712466             |
|                       |           BE738971           |          AK013502             |
|                       |           AI750581           |          AW046391             |
|                       |           BE568917           |          BB458513             |
|                       |           CD000965           |          AV118746             |
|                       |           CN298007           |          CF982365             |
|                       |           AU118684           |          AI505535             |
|                       |           AK021683           |          AV073532             |
|                       |           BE785069           |                               |
|                       |           CD709759           |                               |
|                       |           AL035739           |                               |
|                       |           BM717215           |                               |
|                       |           AL136696           |                               |
|                       |           BG434405           |                               |
|                       |           BQ716428           |                               |
|                       |           BP258688           |                               |
|                       |           AA084554           |                               |
|                       |           BI222305           |                               |
|                       |           BX382880           |                               |
|                       |           BX328825           |                               |
|                       |           BX344390           |                               |
|                       |           CR594163           |                               |
|                       |           BX161501           |                               |
|                       |           BX384052           |                               |
|                       |           BI439014           |                               |
|                       |           BX442740           |                               |
|                       |           CR612457           |                               |
|                       |           AK056145           |                               |
|                       |           CA948800           |                               |
|                       |           AA322574           |                               |
|                       |           BX326327           |                               |
|                       |           BX367881           |                               |
|                       |           CN298008           |                               |
|                       |           BF033632           |                               |
|                       |           CV027992           |                               |
|                       |           CR533510           |                               |
|                       |           CA843379           |                               |
|                       |           CR457163           |                               |
|                       |           BF904068           |                               |
|                       |           BG104800           |                               |
|                       |           AF529304           |                               |
|                       |           BF983256           |                               |
|                       |           CB115591           |                               |
|                       |           BP338883           |                               |
|                       |           BU150773           |                               |
|                       |           BX326019           |                               |
|                       |           BI761402           |                               |
|                       |           BP370726           |                               |
|                       |           BX408494           |                               |
|                       |           BF970684           |                               |
|                       |           BM844025           |                               |
|                       |           BE877473           |                               |
|                       |           BQ573904           |                               |
|                       |             Z46107           |                               |
|                       |           CB148658           |                               |
|                       |           BG502545           |                               |
|                       |           BP265223           |                               |
|                       |           CB141631           |                               |
|                       |           BM782906           |                               |
|                       |           BM785251           |                               |
|                       |           BM785220           |                               |
|                       |           BF790498           |                               |
|                       |           BF035136           |                               |
|                       |           BE087028           |                               |
|                       |           BX344389           |                               |
|                       |           AU151320           |                               |
|                       |           AL573352           |                               |
|                       |           AA813354           |                               |
|                       |           BX328824           |                               |
|                       |             N45076           |                               |
|                       |           BX449839           |                               |
|                       |           BE708531           |                               |
|                       |           BX450367           |                               |
|                       |           CK817727           |                               |
|                       |             Z43102           |                               |
|                       |           BU192033           |                               |
|                       |           BM739935           |                               |
|                       |           BM754131           |                               |
|                       |           BM754172           |                               |
|                       |           BM760156           |                               |
|                       |           BM833313           |                               |
|                       |           BE172329           |                               |
|                       |           BX429289           |                               |
|                       |           BI254882           |                               |
|                       |           AK128628           |                               |
|                       |           BU742186           |                               |
|                       |           BQ002125           |                               |
|                       |           AA813928           |                               |
|                       |           AA826930           |                               |
|                       |             R43277           |                               |
|                       |             R42583           |                               |
|                       |           BM982428           |                               |
|                       |           BM684336           |                               |
|                       |           AL038769           |                               |
|                       |           BX091685           |                               |
|                       |           AA767043           |                               |
|                       |           AI080668           |                               |
|                       |             R37862           |                               |
|                       |             R48950           |                               |
|                       | *Seq. mapped in strand Minus*| *Seq. mapped in strand Minus* |
|                       |           AI133102           |          AV256239             |
|                       |           BM969469           |          AI596413             |
|                       |           BG910368           |          AV274391             |
|                       |                              |          AV277361             |
|                       |                              |          BB017831             |
|                       |                              |          AV279891             |
|                       |                              |          AV281909             |
|                       |                              |          AV264428             |
|                       |                              |          AV048064             |
|                       |                              |                               |
|                       |                              |                               |
|                       |                              |                               |
|                       |                              |                               |
|                       |                              |                               |
|                       |                              |                               |
|                       |                              |                               |
|                       |                              |                               |
|                       |                              |                               |
|                       |                              |                               |
|                       |                              |                               |
|                       |                              |                               |
|                       |                              |                               |
|                       |                              |                               |
|                       |                              |                               |
|                       |                              |                               |
|                       |                              |                               |
|                       |                              |                               |
|                       |                              |                               |
|                       |                              |                               |
|                       |                              |                               |
|                       |                              |                               |
|                       |                              |                               |
|                       |                              |                               |
|                       |                              |                               |
|                       |                              |                               |
|                       |                              |                               |
|                       |                              |                               |
|                       |                              |                               |
|                       |                              |                               |
|                       |                              |                               |
|                       |                              |                               |
|                       |                              |                               |
|                       |                              |                               |
|                       |                              |                               |
|                       |                              |                               |
|                       |                              |                               |
|                       |                              |                               |
|                       |                              |                               |
|                       |                              |                               |
|                       |                              |                               |
|                       |                              |                               |
|                       |                              |                               |
|                       |                              |                               |
|                       |                              |                               |
|                       |                              |                               |
|                       |                              |                               |
|                       |                              |                               |
|                       |                              |                               |
|                       |                              |                               |
|                       |                              |                               |
|                       |                              |                               |
|                       |                              |                               |
|                       |                              |                               |
|                       |                              |                               |
|                       |                              |                               |
|                       |                              |                               |
|                       |                              |                               |
|                       |                              |                               |
|                       |                              |                               |
|                       |                              |                               |
|                       |                              |                               |
|                       |                              |                               |
|                       |                              |                               |
|                       |                              |                               |
|                       |                              |                               |
|                       |                              |                               |
|                       |                              |                               |
|                       |                              |                               |
|                       |                              |                               |
|                       |                              |                               |
+-----------------------+------------------------------+-------------------------------+
|      #    6           | *Seq. mapped in strand Plus* | *Seq. mapped in strand Plus*  |
|                       |           BM907817           |          BB630349             |
|                       |           BG772800           |          AK037222             |
|                       |           BF966457           |          BQ715229             |
|                       |           BI667703           |                               |
|                       |           BG717358           |                               |
|                       |           BI459958           |                               |
|                       |           BG714494           |                               |
|                       |           BG721897           |                               |
|                       |           BG707582           |                               |
|                       |           BI553376           |                               |
|                       |           BG771481           |                               |
|                       |           AU141254           |                               |
|                       |           CB959039           |                               |
|                       |           BF966357           |                               |
|                       |           BG571127           |                               |
|                       |           BG705756           |                               |
|                       |           AL702950           |                               |
|                       |           BG717243           |                               |
|                       |           BP199181           |                               |
|                       |           CD698100           |                               |
|                       |           BP330462           |                               |
|                       |           CD704052           |                               |
|                       |           BX955197           |                               |
|                       |           BX473103           |                               |
|                       |           CD699618           |                               |
|                       |           CD697779           |                               |
|                       |           AU139666           |                               |
|                       |           AU140024           |                               |
|                       |           BG427200           |                               |
|                       |           AU134922           |                               |
|                       |           AU121958           |                               |
|                       |           AU122108           |                               |
|                       |           BP280033           |                               |
|                       |           BM837852           |                               |
|                       |           BM750453           |                               |
|                       |           BP340709           |                               |
|                       |           BP230295           |                               |
|                       |           AU134147           |                               |
|                       |           AU136390           |                               |
|                       |           BG620218           |                               |
|                       |           AU135289           |                               |
|                       |           AU139231           |                               |
|                       |           AU136276           |                               |
|                       |           AL697864           |                               |
|                       |           AU121846           |                               |
|                       |           BP289785           |                               |
|                       |           AU141463           |                               |
|                       |           AU139667           |                               |
|                       |           AU136420           |                               |
|                       |           AU121991           |                               |
|                       |           AU121649           |                               |
|                       |           AU121088           |                               |
|                       |           BP383122           |                               |
|                       |           BP261840           |                               |
|                       |           BX537824           |                               |
|                       |           AU122044           |                               |
|                       |           AW163519           |                               |
|                       |           CB990639           |                               |
|                       |           CF993825           |                               |
|                       |           AU118400           |                               |
|                       |           AU135329           |                               |
|                       |           BI562736           |                               |
|                       |           AL709357           |                               |
|                       |           CF994525           |                               |
|                       |           CF994873           |                               |
|                       |           AU252570           |                               |
|                       |           AU135965           |                               |
|                       |           BP343557           |                               |
|                       |           BI553804           |                               |
|                       |           BP345691           |                               |
|                       |           BP270571           |                               |
|                       |           BP378221           |                               |
|                       |           BP198048           |                               |
|                       |           BM838114           |                               |
|                       |           AU121919           |                               |
|                       |           AL692003           |                               |
|                       |           BU506822           |                               |
|                       |           BP232017           |                               |
|                       |           CN421301           |                               |
|                       |           CD513673           |                               |
|                       |           BX425576           |                               |
|                       |           CA392437           |                               |
|                       |           CD622744           |                               |
|                       |           AA347367           |                               |
|                       |           CD622750           |                               |
|                       |           CD622745           |                               |
|                       |           CD622742           |                               |
|                       |           BM477693           |                               |
|                       |           CD622740           |                               |
|                       |           AL519140           |                               |
|                       |           AL545259           |                               |
|                       |           BX439116           |                               |
|                       |           AL564749           |                               |
|                       |           AL548538           |                               |
|                       |           AL550745           |                               |
|                       |           AL554568           |                               |
|                       |           AL543364           |                               |
|                       |           BX425892           |                               |
|                       |           BX495935           |                               |
|                       |           CB216774           |                               |
|                       |           AU122126           |                               |
|                       |           BG818771           |                               |
|                       |           BU150216           |                               |
|                       |           BU156230           |                               |
|                       |           CF551925           |                               |
|                       |           AL702953           |                               |
|                       |           CF552845           |                               |
|                       |           AL547623           |                               |
|                       |           BG898543           |                               |
|                       |           CA868066           |                               |
|                       |           CD622739           |                               |
|                       |           BM905447           |                               |
|                       |           CN421281           |                               |
|                       |           BX436530           |                               |
|                       |           BU508828           |                               |
|                       |           CN421305           |                               |
|                       |           CD622735           |                               |
|                       |           BQ923605           |                               |
|                       |           BM907786           |                               |
|                       |           CN421307           |                               |
|                       |           BG828899           |                               |
|                       |           CN421306           |                               |
|                       |           CD654982           |                               |
|                       |           BM905874           |                               |
|                       |           BG897009           |                               |
|                       |           CF785989           |                               |
|                       |           BG896178           |                               |
|                       |           CB216787           |                               |
|                       |           CD622755           |                               |
|                       |           CB069386           |                               |
|                       |           CN421291           |                               |
|                       |           CD622754           |                               |
|                       |           BX437175           |                               |
|                       |           BQ876376           |                               |
|                       |           BX463024           |                               |
|                       |           CF552819           |                               |
|                       |           BX449109           |                               |
|                       |           BX415812           |                               |
|                       |           AL543348           |                               |
|                       |           BM922191           |                               |
|                       |           BG698474           |                               |
|                       |           BQ433419           |                               |
|                       |           BM724144           |                               |
|                       |           CD245428           |                               |
|                       |           AL516436           |                               |
|                       |           AL551263           |                               |
|                       |           AV706250           |                               |
|                       |           BI223607           |                               |
|                       |           CD622746           |                               |
|                       |           BU157212           |                               |
|                       |           CD622752           |                               |
|                       |           BQ417183           |                               |
|                       |           BI914514           |                               |
|                       |           BF309182           |                               |
|                       |           BU145473           |                               |
|                       |           BP367218           |                               |
|                       |           CN421317           |                               |
|                       |           BQ884079           |                               |
|                       |           CA396210           |                               |
|                       |           BE885677           |                               |
|                       |           CN421284           |                               |
|                       |           BE615777           |                               |
|                       |           CN421311           |                               |
|                       |           AL552482           |                               |
|                       |           BF685606           |                               |
|                       |           BQ946947           |                               |
|                       |           BX342730           |                               |
|                       |           AL541744           |                               |
|                       |           BQ109319           |                               |
|                       |           BI256778           |                               |
|                       |           CB266143           |                               |
|                       |           CN421308           |                               |
|                       |           CF124964           |                               |
|                       |           AA206523           |                               |
|                       |           AA206534           |                               |
|                       |           CF125823           |                               |
|                       |           CN421309           |                               |
|                       |           CF125952           |                               |
|                       |           CD622741           |                               |
|                       |           CD622737           |                               |
|                       |           CD622743           |                               |
|                       |           BQ719500           |                               |
|                       |           BQ631060           |                               |
|                       |           CN421314           |                               |
|                       |           AL598612           |                               |
|                       |           BE543622           |                               |
|                       |           BQ924263           |                               |
|                       |           BE544749           |                               |
|                       |           CA841172           |                               |
|                       |           CN421319           |                               |
|                       |           BQ437879           |                               |
|                       |           BI438999           |                               |
|                       |           AA203131           |                               |
|                       |           CD558120           |                               |
|                       |           BG120506           |                               |
|                       |           BU150867           |                               |
|                       |           CD622751           |                               |
|                       |           CN421290           |                               |
|                       |           BI603668           |                               |
|                       |           BU149731           |                               |
|                       |           BQ889518           |                               |
|                       |           AU122187           |                               |
|                       |           BP374448           |                               |
|                       |           BP377035           |                               |
|                       |             S73591           |                               |
|                       |           BM458251           |                               |
|                       |           BP342903           |                               |
|                       |           BP343326           |                               |
|                       |           AL046109           |                               |
|                       |           BG427772           |                               |
|                       |           BX482773           |                               |
|                       |           CO398512           |                               |
|                       |           CD622749           |                               |
|                       |           CO247352           |                               |
|                       |           AU139227           |                               |
|                       |           BP381972           |                               |
|                       |           BP220237           |                               |
|                       |           BP294020           |                               |
|                       |           BP293800           |                               |
|                       |           AU137341           |                               |
|                       |           AA044194           |                               |
|                       |           CB132951           |                               |
|                       |           BP221021           |                               |
|                       |           BE270107           |                               |
|                       |           BU679460           |                               |
|                       |           BU685297           |                               |
|                       |           AA716269           |                               |
|                       |           AW770607           |                               |
|                       |           BG928608           |                               |
|                       |           AW603682           |                               |
|                       |           BP378266           |                               |
|                       |           BF305877           |                               |
|                       |           CA868314           |                               |
|                       |           BP366804           |                               |
|                       |           CB987763           |                               |
|                       |           BE883605           |                               |
|                       |           AU141243           |                               |
|                       |           BM786538           |                               |
|                       |           CA840916           |                               |
|                       |           BU679438           |                               |
|                       |           BQ025561           |                               |
|                       |           CN421288           |                               |
|                       |           CD622759           |                               |
|                       |           BP219661           |                               |
|                       |           BF672280           |                               |
|                       |           BG117586           |                               |
|                       |           AA812232           |                               |
|                       |           AI338583           |                               |
|                       |             W88520           |                               |
|                       |           BE674371           |                               |
|                       |           AV726839           |                               |
|                       |           BQ641438           |                               |
|                       |             T39118           |                               |
|                       |           BE504273           |                               |
|                       |           BQ050710           |                               |
|                       |           AA777362           |                               |
|                       |           BP378589           |                               |
|                       |           BQ721517           |                               |
|                       |           AI685871           |                               |
|                       |           BQ022944           |                               |
|                       |           AA131966           |                               |
|                       |           BI028203           |                               |
|                       |             N93308           |                               |
|                       |           AA398576           |                               |
|                       |           BM688645           |                               |
|                       |           BI024395           |                               |
|                       |           AA044019           |                               |
|                       |           AA036885           |                               |
|                       |             H73763           |                               |
|                       |           BM460616           |                               |
|                       |             H62236           |                               |
|                       |           BG998996           |                               |
|                       |           CD556790           |                               |
|                       |           BQ888988           |                               |
|                       |           BG900974           |                               |
|                       |           AY594328           |                               |
|                       |           BG900284           |                               |
|                       |           AI910747           |                               |
|                       |           BI222690           |                               |
|                       |           CN421280           |                               |
|                       |           BI044932           |                               |
|                       |           AA448631           |                               |
|                       |           AI357598           |                               |
|                       |             W90420           |                               |
|                       |           CF595836           |                               |
|                       |           AI364820           |                               |
|                       |           CD515717           |                               |
|                       |             H48221           |                               |
|                       |           BP320215           |                               |
|                       |           CB853824           |                               |
|                       |           BU659836           |                               |
|                       |           BE166542           |                               |
|                       |           CD689123           |                               |
|                       |           CD684276           |                               |
|                       |           BU662372           |                               |
|                       |           BU535970           |                               |
|                       |           BF964702           |                               |
|                       |           AV761260           |                               |
|                       |           BF688107           |                               |
|                       |           BF671618           |                               |
|                       |           AV762615           |                               |
|                       |           AV762806           |                               |
|                       |           AV760419           |                               |
|                       |           BG548800           |                               |
|                       |           BI030539           |                               |
|                       |           BP377294           |                               |
|                       |           BG483414           |                               |
|                       |           AV759000           |                               |
|                       |           BP339630           |                               |
|                       |           BC024305           |                               |
|                       |             H42475           |                               |
|                       |           BP209602           |                               |
|                       |             H57515           |                               |
|                       |           AW362992           |                               |
|                       |           AW370578           |                               |
|                       |           BP379336           |                               |
|                       |           BP220616           |                               |
|                       |           AA281102           |                               |
|                       |           AW370606           |                               |
|                       |             R93140           |                               |
|                       |           AI700941           |                               |
|                       |           AA136227           |                               |
|                       |           BG285627           |                               |
|                       |           CB216968           |                               |
|                       |           CF552691           |                               |
|                       |           BP379010           |                               |
|                       |           BP377730           |                               |
|                       |           BP224311           |                               |
|                       |           BQ893618           |                               |
|                       |           BM008225           |                               |
|                       |           BG761488           |                               |
|                       |           BU657659           |                               |
|                       |           CA843786           |                               |
|                       |           CD703711           |                               |
|                       |           BQ224817           |                               |
|                       |           BP255933           |                               |
|                       |           BP380252           |                               |
|                       |           CB216351           |                               |
|                       |           CA843030           |                               |
|                       |           CO249740           |                               |
|                       |           CD622738           |                               |
|                       |           BG765561           |                               |
|                       |           CD622736           |                               |
|                       |             N55997           |                               |
|                       |           CD512648           |                               |
|                       |           CD622734           |                               |
|                       |           BG928573           |                               |
|                       |           BG760269           |                               |
|                       |           AV746426           |                               |
|                       |           AV745286           |                               |
|                       |           AV745194           |                               |
|                       |           AV745192           |                               |
|                       |           AV745279           |                               |
|                       |           AV746386           |                               |
|                       |           AV746333           |                               |
|                       |           AV746281           |                               |
|                       |           AV746269           |                               |
|                       |           AV745287           |                               |
|                       |           BP327658           |                               |
|                       |           CA976144           |                               |
|                       |           BG929239           |                               |
|                       |           AW390699           |                               |
|                       |           AA132038           |                               |
|                       |           BF846136           |                               |
|                       |           BG025636           |                               |
|                       |           AW379783           |                               |
|                       |           AA250911           |                               |
|                       |           BI260761           |                               |
|                       |             R72421           |                               |
|                       |             N90596           |                               |
|                       |           BP346449           |                               |
|                       |           BG899698           |                               |
|                       |             N79616           |                               |
|                       |           AU135227           |                               |
|                       |           BP377120           |                               |
|                       |           BI022067           |                               |
|                       |           CB216176           |                               |
|                       |           CD622758           |                               |
|                       |           BF149402           |                               |
|                       |           BG432972           |                               |
|                       |           BP328733           |                               |
|                       |           BG987905           |                               |
|                       |             F05153           |                               |
|                       |             Z43463           |                               |
|                       |             T32909           |                               |
|                       |           BG900554           |                               |
|                       |           BG928729           |                               |
|                       |           BQ670862           |                               |
|                       |           BQ890251           |                               |
|                       |           CN421277           |                               |
|                       |           BG568161           |                               |
|                       |           BI824744           |                               |
|                       |           CB268676           |                               |
|                       |           AV762380           |                               |
|                       |           BG035289           |                               |
|                       |           BG818121           |                               |
|                       |           BU617094           |                               |
|                       |           BQ632614           |                               |
|                       |           CB157562           |                               |
|                       |           CA393812           |                               |
|                       |           CF595460           |                               |
|                       |           CF553016           |                               |
|                       |           AA780287           |                               |
|                       |           BE884349           |                               |
|                       |           BP376632           |                               |
|                       |           CD622766           |                               |
|                       |           BP340530           |                               |
|                       |           AA350576           |                               |
|                       |           CD622768           |                               |
|                       |           BM752295           |                               |
|                       |           AW370657           |                               |
|                       |           CD580086           |                               |
|                       |           AW370604           |                               |
|                       |           AW897549           |                               |
|                       |           BP344087           |                               |
|                       |           BG483300           |                               |
|                       |           AW392988           |                               |
|                       |           CN421312           |                               |
|                       |           BU661530           |                               |
|                       |           BP375681           |                               |
|                       |           BM464443           |                               |
|                       |           BG924416           |                               |
|                       |           CD622769           |                               |
|                       |           BF790985           |                               |
|                       |           CN421287           |                               |
|                       |           BP344604           |                               |
|                       |           BG898345           |                               |
|                       |           CD518625           |                               |
|                       |           BP247556           |                               |
|                       |           BP248383           |                               |
|                       |           BP246508           |                               |
|                       |           BP249515           |                               |
|                       |           BP249581           |                               |
|                       |           BP247003           |                               |
|                       |           BP345969           |                               |
|                       |           BE544856           |                               |
|                       |           BI257468           |                               |
|                       |           AA330287           |                               |
|                       |           BG991739           |                               |
|                       |           BQ301526           |                               |
|                       |           CF141544           |                               |
|                       |           AA194713           |                               |
|                       |           BQ301460           |                               |
|                       |           CF139983           |                               |
|                       |           CF139982           |                               |
|                       |           BG989589           |                               |
|                       |           CB998514           |                               |
|                       |           BP344941           |                               |
|                       |           BG740744           |                               |
|                       |           BE736718           |                               |
|                       |           BP317580           |                               |
|                       |           AL040809           |                               |
|                       |             R93577           |                               |
|                       |           CN421297           |                               |
|                       |           BP337950           |                               |
|                       |           AW601825           |                               |
|                       |           AW601845           |                               |
|                       |           BG547856           |                               |
|                       |           CN421302           |                               |
|                       |           BP293837           |                               |
|                       |           BP293994           |                               |
|                       |           BP294516           |                               |
|                       |           AW364795           |                               |
|                       |           AW601832           |                               |
|                       |           AW601823           |                               |
|                       |           AW601820           |                               |
|                       |           AW361875           |                               |
|                       |           CB269010           |                               |
|                       |           BI023833           |                               |
|                       |           AW843646           |                               |
|                       |           AW843655           |                               |
|                       |           AW601881           |                               |
|                       |           BP292931           |                               |
|                       |           AL528852           |                               |
|                       |           AL550255           |                               |
|                       |           CR624503           |                               |
|                       |           CR625339           |                               |
|                       |           BG386844           |                               |
|                       |           BG545743           |                               |
|                       |           BG546033           |                               |
|                       |           BP291908           |                               |
|                       |           BP378790           |                               |
|                       |           BP367535           |                               |
|                       |           AI949744           |                               |
|                       |           AW888937           |                               |
|                       |           AV743459           |                               |
|                       |           BG538186           |                               |
|                       |           AA569077           |                               |
|                       |           BP196965           |                               |
|                       |           BP293552           |                               |
|                       |           BE881183           |                               |
|                       |           BP346079           |                               |
|                       |           CN421304           |                               |
|                       |           BP379417           |                               |
|                       |           BU658063           |                               |
|                       |           BP344940           |                               |
|                       |           BG928999           |                               |
|                       |           BG118765           |                               |
|                       |           BP344804           |                               |
|                       |           BP292916           |                               |
|                       |           BP293084           |                               |
|                       |           BE544934           |                               |
|                       |           CF618819           |                               |
|                       |           AL036317           |                               |
|                       |           CR608500           |                               |
|                       |           AW608105           |                               |
|                       |           AW608150           |                               |
|                       |           AA370384           |                               |
|                       |           BP274455           |                               |
|                       |           CD514173           |                               |
|                       |           CD622770           |                               |
|                       |           CD622771           |                               |
|                       |           BG485454           |                               |
|                       |             R63491           |                               |
|                       |           BF759434           |                               |
|                       |           BF695224           |                               |
|                       |           AA361126           |                               |
|                       |           BP293187           |                               |
|                       |           AA359853           |                               |
|                       |           BG546509           |                               |
|                       |           BP290376           |                               |
|                       |           CO245706           |                               |
|                       |           AW856064           |                               |
|                       |           AW856282           |                               |
|                       |           AW815570           |                               |
|                       |           BE149711           |                               |
|                       |           AA134381           |                               |
|                       |           AA134393           |                               |
|                       |           BP291621           |                               |
|                       |           AW856268           |                               |
|                       |           BE877130           |                               |
|                       |           AW582287           |                               |
|                       |           BG983287           |                               |
|                       |           CA396788           |                               |
|                       |           BG926857           |                               |
|                       |           AW815375           |                               |
|                       |           BG928497           |                               |
|                       |           BP220219           |                               |
|                       |           AW582300           |                               |
|                       |           BG011964           |                               |
|                       |           BG951473           |                               |
|                       |           AA658580           |                               |
|                       |           BP258404           |                               |
|                       |           BP258406           |                               |
|                       |           BG431665           |                               |
|                       |           CF596434           |                               |
|                       |           AW881438           |                               |
|                       |           AL038617           |                               |
|                       |           BQ271973           |                               |
|                       |           BQ271901           |                               |
|                       |           BI023741           |                               |
|                       |           BM507322           |                               |
|                       |           BP366678           |                               |
|                       |           AU134971           |                               |
|                       |           BF694449           |                               |
|                       |           BP264880           |                               |
|                       |           AA135352           |                               |
|                       |           AA135368           |                               |
|                       |           BG951320           |                               |
|                       |           AA337581           |                               |
|                       |           BM798281           |                               |
|                       |           BG539160           |                               |
|                       |           BG036146           |                               |
|                       |           AU122150           |                               |
|                       |           BU072911           |                               |
|                       |           BX437224           |                               |
|                       |             R82538           |                               |
|                       |           CB268470           |                               |
|                       |           BG927524           |                               |
|                       |           CA975878           |                               |
|                       |           AA329795           |                               |
|                       |           BP376571           |                               |
|                       |           BG927128           |                               |
|                       |           BG675282           |                               |
|                       |           BG952200           |                               |
|                       |           BQ720359           |                               |
|                       |           BE697146           |                               |
|                       |             N42003           |                               |
|                       |           AA443005           |                               |
|                       |           BE565682           |                               |
|                       |           AU139225           |                               |
|                       |           CD172426           |                               |
|                       |           AW362592           |                               |
|                       |           BF839166           |                               |
|                       |           BQ933013           |                               |
|                       |           AA180919           |                               |
|                       |           BQ896659           |                               |
|                       |           CB216890           |                               |
|                       |           AL542346           |                               |
|                       |           CD244703           |                               |
|                       |           BF675730           |                               |
|                       |           BG400263           |                               |
|                       |           BP376337           |                               |
|                       |           AA463448           |                               |
|                       |             R36801           |                               |
|                       |           BQ326977           |                               |
|                       |           BE709681           |                               |
|                       |           BG978379           |                               |
|                       |           BI042115           |                               |
|                       |           BI038604           |                               |
|                       |           BP368666           |                               |
|                       |           CD686145           |                               |
|                       |           BG118153           |                               |
|                       |           BG826982           |                               |
|                       |           BG826098           |                               |
|                       |           CD701433           |                               |
|                       |           BI255297           |                               |
|                       |             H43021           |                               |
|                       |           BU076724           |                               |
|                       |           CN421310           |                               |
|                       |           BI857883           |                               |
|                       |           AA341556           |                               |
|                       |           BP340222           |                               |
|                       |           BE162184           |                               |
|                       |           BF966585           |                               |
|                       |           BX500773           |                               |
|                       |           BF675064           |                               |
|                       |           BQ009126           |                               |
|                       |           BQ631559           |                               |
|                       |           CA392560           |                               |
|                       |           BM837079           |                               |
|                       |             D31314           |                               |
|                       |             D31386           |                               |
|                       |           BG545754           |                               |
|                       |           BG683648           |                               |
|                       |           BF678786           |                               |
|                       |           BG928606           |                               |
|                       |           BP376094           |                               |
|                       |           BF847181           |                               |
|                       |           BP197206           |                               |
|                       |           AJ714743           |                               |
|                       |           BF171556           |                               |
|                       |           BF904055           |                               |
|                       |           CD706614           |                               |
|                       |           BG896865           |                               |
|                       |           AA361831           |                               |
|                       |           AI718560           |                               |
|                       |           CB998974           |                               |
|                       |           AL041248           |                               |
|                       |           BG952273           |                               |
|                       |           BP305303           |                               |
|                       |           BG952190           |                               |
|                       |           CN421299           |                               |
|                       |           BF902129           |                               |
|                       |           BG951326           |                               |
|                       |           AV707143           |                               |
|                       |           CN421293           |                               |
|                       |           BI031027           |                               |
|                       |           BQ335398           |                               |
|                       |           BI055699           |                               |
|                       |           BE513180           |                               |
|                       |           BP196023           |                               |
|                       |           BF792134           |                               |
|                       |           BQ898049           |                               |
|                       |           BG705448           |                               |
|                       |           BG900449           |                               |
|                       |           BQ720785           |                               |
|                       |           AA090955           |                               |
|                       |           AU118391           |                               |
|                       |           BG434799           |                               |
|                       |           BM680318           |                               |
|                       |           BM999302           |                               |
|                       |           BM312691           |                               |
|                       |           BU686039           |                               |
|                       |           CD244965           |                               |
|                       |           AW860387           |                               |
|                       |           BG951336           |                               |
|                       |           BG998314           |                               |
|                       |           BF904061           |                               |
|                       |           BQ307271           |                               |
|                       |           AI828493           |                               |
|                       |           CA777933           |                               |
|                       |           BQ008528           |                               |
|                       |           CA308518           |                               |
|                       |           CA423191           |                               |
|                       |           BE876612           |                               |
|                       |           CK822160           |                               |
|                       |           CA778204           |                               |
|                       |           BI056038           |                               |
|                       |           CD704058           |                               |
|                       |           AI924102           |                               |
|                       |           BU950462           |                               |
|                       |           BI602673           |                               |
|                       |           BP369668           |                               |
|                       |           CN421279           |                               |
|                       |             R19654           |                               |
|                       |           AV705996           |                               |
|                       |           BG896867           |                               |
|                       |           AL564680           |                               |
|                       |           BP270775           |                               |
|                       |           AA903737           |                               |
|                       |             R74286           |                               |
|                       |           BG925784           |                               |
|                       |             H54110           |                               |
|                       |             H53925           |                               |
|                       |           BU664708           |                               |
|                       |           BG924703           |                               |
|                       |           AI563990           |                               |
|                       |           BP375426           |                               |
|                       |           BI260537           |                               |
|                       |           BM989869           |                               |
|                       |           CN421278           |                               |
|                       |           CD516231           |                               |
|                       |             N87907           |                               |
|                       |           BX427000           |                               |
|                       |           CR598379           |                               |
|                       |           BG538124           |                               |
|                       |           BQ632323           |                               |
|                       |           AI888113           |                               |
|                       |           BM980480           |                               |
|                       |           BM565582           |                               |
|                       |           BQ631282           |                               |
|                       |           BM312379           |                               |
|                       |           BQ613811           |                               |
|                       |           BE858792           |                               |
|                       |           CA416371           |                               |
|                       |           AL036213           |                               |
|                       |           BG286672           |                               |
|                       |           AI860299           |                               |
|                       |           BU603027           |                               |
|                       |           CB131009           |                               |
|                       |           CN421295           |                               |
|                       |           BE735841           |                               |
|                       |           BI829060           |                               |
|                       |             H54547           |                               |
|                       |           AA599780           |                               |
|                       |           CD517747           |                               |
|                       |           AA376801           |                               |
|                       |           BG111785           |                               |
|                       |           AI742042           |                               |
|                       |           AA496852           |                               |
|                       |           CA407745           |                               |
|                       |           CA776462           |                               |
|                       |           BF244362           |                               |
|                       |           BM312982           |                               |
|                       |           BP260685           |                               |
|                       |           BG952275           |                               |
|                       |           CD688718           |                               |
|                       |           BM312952           |                               |
|                       |           AW379845           |                               |
|                       |           AW380098           |                               |
|                       |           AW380125           |                               |
|                       |           CA943728           |                               |
|                       |           BM744769           |                               |
|                       |           BM747531           |                               |
|                       |           BM746056           |                               |
|                       |           BM744736           |                               |
|                       |           BM743241           |                               |
|                       |           BM746363           |                               |
|                       |           BM746397           |                               |
|                       |           BM746316           |                               |
|                       |           BM992524           |                               |
|                       |           CD103737           |                               |
|                       |           BM705285           |                               |
|                       |           BG897296           |                               |
|                       |           AA192405           |                               |
|                       |           BI335488           |                               |
|                       |           CR613891           |                               |
|                       |           CR620402           |                               |
|                       |           BM311010           |                               |
|                       |           AY358325           |                               |
|                       |           CK821579           |                               |
|                       |           AF112345           |                               |
|                       |           BP285155           |                               |
|                       |           BX504665           |                               |
|                       |           BX474527           |                               |
|                       |           BX474009           |                               |
|                       |           BX474428           |                               |
|                       |           BX474556           |                               |
|                       |           BX474109           |                               |
|                       |           BX474100           |                               |
|                       |           BP277771           |                               |
|                       |           AF074015           |                               |
|                       |           BG896789           |                               |
|                       |           AL708313           |                               |
|                       |           CK000752           |                               |
|                       |           BU624984           |                               |
|                       |           BP345328           |                               |
|                       |           AL710595           |                               |
|                       |           BG938743           |                               |
|                       |           BQ683664           |                               |
|                       |           BQ677070           |                               |
|                       |             N72734           |                               |
|                       |           AA461265           |                               |
|                       |           BM704891           |                               |
|                       |           BU732047           |                               |
|                       |           BU684458           |                               |
|                       |           AA521075           |                               |
|                       |           BU736848           |                               |
|                       |           CR613859           |                               |
|                       |           BU784547           |                               |
|                       |             H15529           |                               |
|                       |           BU070326           |                               |
|                       |           CD369510           |                               |
|                       |           AI591157           |                               |
|                       |           BU732282           |                               |
|                       |           BM904028           |                               |
|                       |           AI814799           |                               |
|                       |           AW339344           |                               |
|                       |           CR612183           |                               |
|                       |           BX404012           |                               |
|                       |           BM662725           |                               |
|                       |           AA659096           |                               |
|                       |           CR593638           |                               |
|                       |           CR614030           |                               |
|                       |           AL536223           |                               |
|                       |           BX393652           |                               |
|                       |           BQ774098           |                               |
|                       |           CA442948           |                               |
|                       |           BQ018063           |                               |
|                       |           BM981478           |                               |
|                       |           CA447427           |                               |
|                       |           BM671990           |                               |
|                       |           CA447270           |                               |
|                       |           CA446125           |                               |
|                       |           CA448613           |                               |
|                       |           BQ004325           |                               |
|                       |           BU621251           |                               |
|                       |           BU688083           |                               |
|                       |           BQ774210           |                               |
|                       |           CA418132           |                               |
|                       |           BM971670           |                               |
|                       |           BQ574194           |                               |
|                       |           BM996421           |                               |
|                       |           BQ004364           |                               |
|                       |           BQ001703           |                               |
|                       |           CA420924           |                               |
|                       |           BM969876           |                               |
|                       |           CA420164           |                               |
|                       |           CA445995           |                               |
|                       |           BU678134           |                               |
|                       |           BQ011253           |                               |
|                       |           CA420173           |                               |
|                       |           BX344069           |                               |
|                       |           BU736401           |                               |
|                       |           BU857059           |                               |
|                       |           BQ045328           |                               |
|                       |           BU674586           |                               |
|                       |           CA413523           |                               |
|                       |           CA416108           |                               |
|                       |           BQ016534           |                               |
|                       |           CA422267           |                               |
|                       |           BQ005033           |                               |
|                       |           BU678412           |                               |
|                       |           CA415508           |                               |
|                       |           BQ446764           |                               |
|                       |           BQ008890           |                               |
|                       |           AL535662           |                               |
|                       |           BI491141           |                               |
|                       |           BM984406           |                               |
|                       |           BX495936           |                               |
|                       |           AA088405           |                               |
|                       |           AA913613           |                               |
|                       |           CB850947           |                               |
|                       |           BQ020347           |                               |
|                       |           BM510507           |                               |
|                       |           BU729311           |                               |
|                       |           CA416599           |                               |
|                       |           BM990088           |                               |
|                       |           CK822161           |                               |
|                       |           BM999167           |                               |
|                       |           CR609336           |                               |
|                       |           AL552805           |                               |
|                       |           BM145346           |                               |
|                       |           AI755133           |                               |
|                       |           AL038618           |                               |
|                       |           BM876322           |                               |
|                       |           BI495860           |                               |
|                       |           BQ000225           |                               |
|                       |           CN479795           |                               |
|                       |           AA069616           |                               |
|                       |           AI754729           |                               |
|                       |           CA309064           |                               |
|                       |           BQ000611           |                               |
|                       |             W37539           |                               |
|                       |           AA086001           |                               |
|                       |           BU685214           |                               |
|                       |           BQ772402           |                               |
|                       |           BQ004203           |                               |
|                       |           BQ772735           |                               |
|                       |           BQ775385           |                               |
|                       |           CA417120           |                               |
|                       |           CA448648           |                               |
|                       |           CA428422           |                               |
|                       |           BX101312           |                               |
|                       |           CR743317           |                               |
|                       |           BM817921           |                               |
|                       |           BU735361           |                               |
|                       |           BI492285           |                               |
|                       |           CA444018           |                               |
|                       |           AI707810           |                               |
|                       |           CA432943           |                               |
|                       |           AA598890           |                               |
|                       |           AA772104           |                               |
|                       |           AI624009           |                               |
|                       |           AI830789           |                               |
|                       |           AI753462           |                               |
|                       |           AI753533           |                               |
|                       |           AW340839           |                               |
|                       |           AI418624           |                               |
|                       |             R55126           |                               |
|                       |           BM149638           |                               |
|                       |           AI539299           |                               |
|                       |           AA639015           |                               |
|                       |           AA897567           |                               |
|                       |           AW027818           |                               |
|                       |           AA159622           |                               |
|                       |           BF432181           |                               |
|                       |           AA148935           |                               |
|                       |           BM988374           |                               |
|                       |           BM968916           |                               |
|                       |           AW130139           |                               |
|                       |           AI077800           |                               |
|                       |           AW419094           |                               |
|                       |           AW590975           |                               |
|                       |           AI031542           |                               |
|                       |           AA169502           |                               |
|                       |           CA448362           |                               |
|                       |             W61273           |                               |
|                       |           BQ030003           |                               |
|                       |           AV744669           |                               |
|                       |           AA761515           |                               |
|                       |           AA807312           |                               |
|                       |           AI565450           |                               |
|                       |           AA658444           |                               |
|                       |           AI224400           |                               |
|                       |           BF475607           |                               |
|                       |           AW027656           |                               |
|                       |           AA810300           |                               |
|                       |           AA770634           |                               |
|                       |           AA640089           |                               |
|                       |           AI973226           |                               |
|                       |           AI817222           |                               |
|                       |           BM510571           |                               |
|                       |           BM193056           |                               |
|                       |           AA953197           |                               |
|                       |           AA805348           |                               |
|                       |           AA872738           |                               |
|                       |           BU733064           |                               |
|                       |           BM673345           |                               |
|                       |           AI027874           |                               |
|                       |           AW662018           |                               |
|                       |           AW193297           |                               |
|                       |           AI049863           |                               |
|                       |           AW166068           |                               |
|                       |           AI983172           |                               |
|                       |           AW304944           |                               |
|                       |           AI537057           |                               |
|                       |           BM969749           |                               |
|                       |           BM983672           |                               |
|                       |           AI079678           |                               |
|                       |           AI537659           |                               |
|                       |           AI805497           |                               |
|                       |           AA781016           |                               |
|                       |           AJ346307           |                               |
|                       |           AI951504           |                               |
|                       |           BG057164           |                               |
|                       |           AI031543           |                               |
|                       |           AW572631           |                               |
|                       |           AA872739           |                               |
|                       |           AW050744           |                               |
|                       |           BE046133           |                               |
|                       |           BM145044           |                               |
|                       |           BX335517           |                               |
|                       |           AI924052           |                               |
|                       |           AI126479           |                               |
|                       |           AW136747           |                               |
|                       |           BQ029953           |                               |
|                       |           BQ030390           |                               |
|                       |           BX387594           |                               |
|                       |           CA441187           |                               |
|                       |             T29999           |                               |
|                       |             R40061           |                               |
|                       |           AI289915           |                               |
|                       |           AA732670           |                               |
|                       |           BX333073           |                               |
|                       |           AA599642           |                               |
|                       |           AW166578           |                               |
|                       |             F24381           |                               |
|                       |           AI580718           |                               |
|                       |           AI865030           |                               |
|                       |           AA854698           |                               |
|                       |           AK128050           |                               |
|                       |           AK094282           |                               |
|                       |           CB134361           |                               |
|                       |           AK128733           |                               |
|                       |           BM546917           |                               |
|                       |           CR747392           |                               |
|                       |           BC035727           |                               |
|                       |           BU162078           |                               |
|                       |           CN281620           |                               |
|                       |           BU431497           |                               |
|                       |           BM475664           |                               |
|                       |           AI810144           |                               |
|                       |           AI805840           |                               |
|                       |           BQ224265           |                               |
|                       |           CR627381           |                               |
|                       |           AA234297           |                               |
|                       |           BF970451           |                               |
|                       |           AA669495           |                               |
|                       |           BG910251           |                               |
|                       |           BX096645           |                               |
|                       |           BM712963           |                               |
|                       |           BM715475           |                               |
|                       |           BM723497           |                               |
|                       |           BX117134           |                               |
|                       |           AK123675           |                               |
|                       |           AA744924           |                               |
|                       |           BQ230978           |                               |
|                       |           BF475795           |                               |
|                       |           BM673948           |                               |
|                       | *Seq. mapped in strand Minus*| *Seq. mapped in strand Minus* |
|                       |           BP383347           |          AW476622             |
|                       |           BP379658           |                               |
|                       |           CN421320           |                               |
|                       |           AW138469           |                               |
|                       |           BM147009           |                               |
|                       |           BM989951           |                               |
|                       |           CA442576           |                               |
|                       |           BM999219           |                               |
|                       |           BQ017507           |                               |
|                       |           BM978844           |                               |
|                       |           BM990056           |                               |
|                       |           CA440351           |                               |
|                       |           BQ017586           |                               |
|                       |           AA492448           |                               |
|                       |             F26761           |                               |
|                       |           AV739444           |                               |
|                       |           AI612975           |                               |
|                       |           AI522248           |                               |
|                       |           CB321873           |                               |
|                       |           AA228747           |                               |
|                       |           BX118360           |                               |
|                       |           AI359032           |                               |
|                       |           AI301864           |                               |
|                       |           BM510032           |                               |
|                       |           BI492981           |                               |
|                       |           CB242242           |                               |
|                       |           CA450633           |                               |
|                       |           BX479547           |                               |
|                       |           BF432427           |                               |
|                       |           BQ285949           |                               |
|                       |           CB045954           |                               |
|                       |           BU526638           |                               |
|                       |           BQ006437           |                               |
|                       |           BU627948           |                               |
|                       |           CA308046           |                               |
|                       |           AK092209           |                               |
|                       |           AK056984           |                               |
|                       |           BC004355           |                               |
|                       |           BC050418           |                               |
|                       |           AI761509           |                               |
|                       |           BG942997           |                               |
|                       |           BG324388           |                               |
|                       |           BG685801           |                               |
|                       |           AV735986           |                               |
|                       |           AV737684           |                               |
|                       |           BE826475           |                               |
|                       |           BQ430111           |                               |
|                       |           BM472510           |                               |
|                       |           BF026134           |                               |
|                       |           BF223369           |                               |
|                       |           BQ898011           |                               |
|                       |           BE737046           |                               |
|                       |           BU521519           |                               |
|                       |           BG682361           |                               |
|                       |           BG756154           |                               |
|                       |           BF974342           |                               |
|                       |           BG389318           |                               |
|                       |           BG941244           |                               |
|                       |           BG111339           |                               |
|                       |           BE791528           |                               |
|                       |           BQ053116           |                               |
|                       |           BI821970           |                               |
|                       |           CV025657           |                               |
|                       |           BM018782           |                               |
|                       |           BU183652           |                               |
|                       |           BU600134           |                               |
|                       |           BM909088           |                               |
|                       |           CN261801           |                               |
|                       |           BM545023           |                               |
|                       |           BF969270           |                               |
|                       |           BI914801           |                               |
|                       |           BG759871           |                               |
|                       |           BE829989           |                               |
|                       |           BF698828           |                               |
|                       |           AW512447           |                               |
|                       |           BM020508           |                               |
|                       |           BF218013           |                               |
|                       |             D25938           |                               |
|                       |           AI583632           |                               |
|                       |           AI792542           |                               |
|                       |           BM503891           |                               |
|                       |           BE564067           |                               |
|                       |           BG397864           |                               |
|                       |           AI591245           |                               |
|                       |           CA951048           |                               |
|                       |           CK825721           |                               |
|                       |           AI054159           |                               |
|                       |           AI792481           |                               |
|                       |           CA950796           |                               |
|                       |           AI053595           |                               |
|                       |           BE139383           |                               |
|                       |           CA424513           |                               |
|                       |           BQ269306           |                               |
|                       |           BG472849           |                               |
|                       |           BM044773           |                               |
|                       |           BE258420           |                               |
|                       |           BP235483           |                               |
|                       |           BP217146           |                               |
|                       |           CN261804           |                               |
|                       |           BE895813           |                               |
|                       |           BF129936           |                               |
|                       |           BG910663           |                               |
|                       |           BU590181           |                               |
|                       |             T67591           |                               |
|                       |           BX488881           |                               |
|                       |           AA317494           |                               |
|                       |           AA337939           |                               |
|                       |           CB148097           |                               |
|                       |           CN287023           |                               |
|                       |           AI217777           |                               |
|                       |           BX107041           |                               |
|                       |           AW602962           |                               |
|                       |           BE076691           |                               |
|                       |           AA781443           |                               |
|                       |           AW662835           |                               |
|                       |           BU567863           |                               |
|                       |           AW182502           |                               |
|                       |           BU569132           |                               |
|                       |           AW137431           |                               |
|                       |           BQ775531           |                               |
|                       |           AA858044           |                               |
|                       |           AW892445           |                               |
|                       |           AW903084           |                               |
|                       |           BE935637           |                               |
|                       |           AW014190           |                               |
|                       |           CA312973           |                               |
|                       |           CB250453           |                               |
|                       |           AY233132           |                               |
|                       |           BG195314           |                               |
|                       |           BG207187           |                               |
+-----------------------+------------------------------+-------------------------------+
|      #    7           | *Seq. mapped in strand Plus* | *Seq. mapped in strand Plus*  |
|                       |           BU674725           |          AK015009             |
|                       |           CR740768           |          BE981585             |
|                       | *Seq. mapped in strand Minus*| *Seq. mapped in strand Minus* |
|                       |           BM681785           |          BF319608             |
|                       |           BQ019148           |                               |
|                       |           AK023525           |                               |
|                       |           CR617733           |                               |
|                       |           BU674110           |                               |
|                       |           AI640215           |                               |
|                       |           AA761296           |                               |
|                       |           AA760779           |                               |
|                       |           AA743697           |                               |
|                       |           AK123753           |                               |
|                       |           AK098289           |                               |
|                       |           BC017179           |                               |
|                       |           BC040682           |                               |
|                       |           BU622713           |                               |
|                       |           AK093377           |                               |
|                       |           AW105441           |                               |
|                       |           AK122788           |                               |
|                       |           BC043612           |                               |
|                       |           AK093186           |                               |
|                       |           AF525399           |                               |
|                       |           BE906325           |                               |
|                       |           BP296911           |                               |
|                       |           BC062551           |                               |
|                       |           BE257221           |                               |
|                       |           BE254998           |                               |
|                       |           BF962565           |                               |
|                       |           BP352038           |                               |
|                       |           BQ230084           |                               |
+-----------------------+------------------------------+-------------------------------+
|      #    8           | *Seq. mapped in strand Plus* | *Seq. mapped in strand Plus*  |
|                       |           BC053557           |          BY722983             |
|                       |           AK091946           |          BY734199             |
|                       |                              |          BY071252             |
|                       |                              |          BY124592             |
|                       |                              |          CB244443             |
|                       |                              |          CD348393             |
|                       |                              |          BY789435             |
|                       |                              |          BB867000             |
|                       |                              |          BU702045             |
|                       |                              |          CD350228             |
|                       |                              |          BY232895             |
|                       |                              |          BY020505             |
|                       |                              |          BQ180575             |
|                       |                              |          CB247724             |
|                       |                              |          CA328686             |
|                       |                              |          CB525431             |
|                       |                              |          BQ771143             |
|                       |                              |          BF319949             |
|                       |                              |          BE986479             |
|                       |                              |          AI851301             |
|                       |                              |          CF895287             |
|                       |                              |          CB248039             |
|                       |                              |          AI854009             |
|                       |                              |          BY261632             |
|                       |                              |          CB248122             |
|                       |                              |          BQ712950             |
|                       |                              |          CF536932             |
|                       |                              |          AA050136             |
|                       |                              |          BC066011             |
|                       |                              |          CB183781             |
|                       |                              |          AA051715             |
|                       |                              |          CN527615             |
|                       |                              |          CF739912             |
|                       |                              |          AI158096             |
|                       |                              |          CN676354             |
|                       |                              |          CD544921             |
|                       |                              |          CD350239             |
|                       |                              |          BC030410             |
|                       |                              |          BI736348             |
|                       |                              |          CF892732             |
|                       |                              |          BE571101             |
|                       |                              |          BU708329             |
|                       |                              |          BI555904             |
|                       |                              |          CF736101             |
|                       |                              |          BE572731             |
|                       |                              |          CN703638             |
|                       |                              |          BU613782             |
|                       | *Seq. mapped in strand Minus*| *Seq. mapped in strand Minus* |
|                       |           AB044561           |          AW120896             |
|                       |           AF116545           |          AW413140             |
|                       |           AF116548           |          AW700937             |
|                       |           AF116547           |          BM226244             |
|                       |           AK122681           |          AI043109             |
|                       |           BC052249           |          BF782362             |
|                       |                              |          AI605786             |
|                       |                              |          AU018875             |
|                       |                              |          AW764187             |
|                       |                              |          BF228005             |
|                       |                              |          CD353332             |
|                       |                              |          AY033912             |
|                       |                              |          AI929965             |
|                       |                              |          AI891511             |
|                       |                              |          AA261365             |
|                       |                              |          BI218641             |
|                       |                              |          AA546528             |
|                       |                              |          BG969589             |
|                       |                              |          BI685628             |
|                       |                              |          BQ944745             |
|                       |                              |          BI143674             |
|                       |                              |          AA244956             |
|                       |                              |          AW610920             |
|                       |                              |          AA388582             |
|                       |                              |          AA199449             |
|                       |                              |          CN696550             |
|                       |                              |          BI556022             |
|                       |                              |          BI526278             |
|                       |                              |          BF780967             |
|                       |                              |          BF142970             |
|                       |                              |          BF786332             |
|                       |                              |          AW909655             |
|                       |                              |          BF536812             |
|                       |                              |          BU614063             |
|                       |                              |          BF780137             |
|                       |                              |          BQ827890             |
|                       |                              |          BE911099             |
|                       |                              |          BE648987             |
|                       |                              |          BI107619             |
|                       |                              |          BQ831920             |
|                       |                              |          CB948113             |
|                       |                              |          BI687645             |
|                       |                              |          CB950665             |
|                       |                              |          BI330614             |
|                       |                              |          BI556264             |
|                       |                              |          BI101433             |
|                       |                              |          CD241644             |
+-----------------------+------------------------------+-------------------------------+
|      #    9           | *Seq. mapped in strand Plus* | *Seq. mapped in strand Plus*  |
|                       |           BF898132           |          BE944710             |
|                       |           BX094916           |          BE946894             |
|                       |             R41184           |          BM875830             |
|                       |           BX091794           |                               |
|                       | *Seq. mapped in strand Minus*| *Seq. mapped in strand Minus* |
|                       |           BM970618           |          BQ043348             |
|                       |           BM674566           |          BE953764             |
|                       |           BF221879           |          AK011896             |
|                       |           AA830630           |          BC029231             |
|                       |           AA808219           |          CF737898             |
|                       |           BM995757           |          CN530254             |
|                       |           BX091831           |          CA315894             |
|                       |           AK023511           |          CN682960             |
|                       |           AL136651           |          CN697474             |
|                       |           AI951517           |          BQ886266             |
|                       |           BC028586           |          CN527814             |
|                       |           CR591202           |          AK013314             |
|                       |           AI377424           |          AK020974             |
|                       |           AI026765           |          BY727035             |
|                       |           AK098640           |          BY740037             |
|                       |           CN300602           |          BQ565825             |
|                       |           CV022995           |          BQ942253             |
|                       |           BU431612           |          BI986775             |
|                       |           AJ243230           |          CF897769             |
|                       |           CD656011           |          BG088933             |
|                       |           BI550796           |          BY253677             |
|                       |           BI562500           |          BY268987             |
|                       |           CN300598           |          BY072360             |
|                       |           CN300600           |          BY082332             |
|                       |           CF137638           |          BY063490             |
|                       |           CD108555           |          BY128377             |
|                       |           BI460637           |          BY130043             |
|                       |           BM474679           |                               |
|                       |           AW503438           |                               |
|                       |           BG772588           |                               |
|                       |           CN300603           |                               |
|                       |           BP314069           |                               |
|                       |           BI559589           |                               |
|                       |           BP311370           |                               |
|                       |           CN300599           |                               |
|                       |           BP199687           |                               |
|                       |           BP370691           |                               |
|                       |           CD688842           |                               |
|                       |           BG772918           |                               |
|                       |           CF137515           |                               |
+-----------------------+------------------------------+-------------------------------+
|      #   10           | *Seq. mapped in strand Plus* | *Seq. mapped in strand Plus*  |
|                       |           CB959793           |          BY794533             |
|                       |           CB988401           |          BY792068             |
|                       |           AL534902           |          BY771773             |
|                       |           BP274101           |          BY206199             |
|                       |           AJ311123           |          BY208097             |
|                       |           BC017725           |          BY739614             |
|                       |           CB996286           |          CB193348             |
|                       |           CF994599           |          BY091467             |
|                       |           BP275877           |          BY074360             |
|                       |           CD109563           |          BY342121             |
|                       |           BG773534           |          BY342426             |
|                       |           BF978071           |          BY337144             |
|                       |           BF978258           |          BY339572             |
|                       |           CF593384           |          BY066851             |
|                       |           CF593383           |          BY279478             |
|                       |           AK075565           |          BC016210             |
|                       |           BM924756           |          BY099652             |
|                       |           BE879420           |          BG870098             |
|                       |           BX458517           |          BC057642             |
|                       |           BQ879066           |          BY067276             |
|                       |           CD171733           |          BY339976             |
|                       |           BU071677           |          BY741147             |
|                       |           BM462765           |          BI655305             |
|                       |           AI761399           |          BY126079             |
|                       |           BQ548930           |          BB658380             |
|                       |           BU929095           |          CK623010             |
|                       |           BF204095           |          BY741338             |
|                       |           BG826887           |          AK084415             |
|                       |           BF576922           |          BY342125             |
|                       |           CD244964           |          BI693526             |
|                       |           BI862479           |          AW012403             |
|                       |           BM685148           |          AK076087             |
|                       |           AW295579           |          BI731576             |
|                       |             L17329           |          AK008190             |
|                       |           AL133555           |          BY081866             |
|                       |           BM990513           |          BB611103             |
|                       |           BU679182           |          BY023608             |
|                       |           BM972829           |          BY103016             |
|                       |           BU740658           |          BY079527             |
|                       |           CK818302           |          BY080458             |
|                       |           CF529697           |          BY097781             |
|                       |           AA149767           |          BY742629             |
|                       |           BU782813           |          BY708145             |
|                       |           BU073224           |          BY741564             |
|                       |           BM674318           |          BY196598             |
|                       |           AA102347           |          AA048994             |
|                       |           AA722132           |          AI510103             |
|                       |           AA718965           |          BY783289             |
|                       |           AV708449           |          BE289447             |
|                       |           BM676344           |          BF472206             |
|                       |           AA918713           |          BE287084             |
|                       |           CA944941           |          CK792631             |
|                       |           AA888241           |          BE689503             |
|                       |           BU735722           |          BB831536             |
|                       |           BU684856           |          BI332720             |
|                       |           BU752488           |                               |
|                       |           BU752425           |                               |
|                       |           BM971945           |                               |
|                       |           AA737032           |                               |
|                       |           AI192161           |                               |
|                       |           AA837021           |                               |
|                       |           BQ268345           |                               |
|                       |           BQ270290           |                               |
|                       |           BM675243           |                               |
|                       |           BM681536           |                               |
|                       |           AA662475           |                               |
|                       |           AI669071           |                               |
|                       |           BM473087           |                               |
|                       |           CB178497           |                               |
|                       | *Seq. mapped in strand Minus*| *Seq. mapped in strand Minus* |
|                       |           BQ016573           |          AV134050             |
|                       |           AI133137           |          BG869257             |
|                       |           CB241762           |                               |
|                       |           CN478649           |                               |
|                       |           CA502827           |                               |
+-----------------------+------------------------------+-------------------------------+
|      #   11           | *Seq. mapped in strand Plus* | *Seq. mapped in strand Plus*  |
|                       |           BG706542           |          AK088294             |
|                       |           BX424745           |          AK080447             |
|                       |           BX459730           |          AK037175             |
|                       |           BI559694           |          AK083981             |
|                       |           BX345397           |          AK085584             |
|                       |           BU163166           |          BC003900             |
|                       |           CR590330           |          AK002487             |
|                       |           AL037485           |          AK004160             |
|                       |           AW410827           |          AK086661             |
|                       |           BM542787           |          AK086441             |
|                       |           CD656692           |                               |
|                       |           CN310148           |                               |
|                       |           BU165361           |                               |
|                       |           BQ425162           |                               |
|                       |           BX366255           |                               |
|                       |           BG911303           |                               |
|                       |           BG109890           |                               |
|                       |           BX387311           |                               |
|                       |           BX461649           |                               |
|                       |           BX393856           |                               |
|                       |           CR609849           |                               |
|                       |           CR601437           |                               |
|                       |           BM724104           |                               |
|                       |           BM543777           |                               |
|                       |           BG830664           |                               |
|                       |           BM541422           |                               |
|                       |           BG761388           |                               |
|                       |           BQ059056           |                               |
|                       |           BM459595           |                               |
|                       |           BI756906           |                               |
|                       |           BM915928           |                               |
|                       |           BQ214004           |                               |
|                       |           BM009758           |                               |
|                       |           BI115774           |                               |
|                       |           BM010722           |                               |
|                       |           BM009267           |                               |
|                       |             F11333           |                               |
|                       |           BX095591           |                               |
|                       |           BG421665           |                               |
|                       |           BI831714           |                               |
|                       |           BG765937           |                               |
|                       |           BC000473           |                               |
|                       |           BC001509           |                               |
|                       |           BC001833           |                               |
|                       |           BQ219375           |                               |
|                       |             Z44689           |                               |
|                       |           BM696144           |                               |
|                       |           CN310147           |                               |
|                       |           BF528838           |                               |
|                       |           BI766713           |                               |
|                       |           BI908520           |                               |
|                       |           BM016955           |                               |
|                       |           BM017508           |                               |
|                       |           BM473389           |                               |
|                       |           BM922088           |                               |
|                       |           BQ068063           |                               |
|                       |           BM457974           |                               |
|                       |           BC001298           |                               |
|                       |             H14385           |                               |
|                       |           CD673339           |                               |
|                       |           BM560461           |                               |
|                       |           BU173150           |                               |
|                       |           BI754934           |                               |
|                       |           BX447113           |                               |
|                       |           BI757178           |                               |
|                       |           BX407000           |                               |
|                       |           BX374427           |                               |
|                       |           BQ070638           |                               |
|                       |           BX425876           |                               |
|                       |           BX332684           |                               |
|                       |           CR605045           |                               |
|                       |           BE018820           |                               |
|                       |           BM744156           |                               |
|                       |           BM752704           |                               |
|                       |           BM772704           |                               |
|                       |           BI918950           |                               |
|                       |           BQ933486           |                               |
|                       |           BM848341           |                               |
|                       |           BQ067336           |                               |
|                       |           AL021682           |                               |
|                       |           CN310136           |                               |
|                       |           CF132297           |                               |
|                       |           BE262833           |                               |
|                       |           BM707566           |                               |
|                       |           AW248159           |                               |
|                       |           BM725906           |                               |
|                       |           BQ652521           |                               |
|                       |           CN310149           |                               |
|                       |           CN310142           |                               |
|                       |           BF316595           |                               |
|                       |           BG395523           |                               |
|                       |           BG421207           |                               |
|                       |           BG823197           |                               |
|                       |           BI836303           |                               |
|                       |           BM467746           |                               |
|                       |             R25092           |                               |
|                       |           BX363861           |                               |
|                       |           BX462291           |                               |
|                       |           BX354265           |                               |
|                       |           CR626379           |                               |
|                       |           CR599763           |                               |
|                       |           BI116147           |                               |
|                       |           BI194547           |                               |
|                       |           BF685913           |                               |
|                       |           BM005950           |                               |
|                       |           BX385221           |                               |
|                       |           BM563583           |                               |
|                       |           BG325960           |                               |
|                       |           BI822218           |                               |
|                       |           BI913455           |                               |
|                       |           BI824197           |                               |
|                       |           BI756779           |                               |
|                       |           BC001937           |                               |
|                       |           BM691426           |                               |
|                       |           BE735093           |                               |
|                       |           BC009441           |                               |
|                       |           BG677996           |                               |
|                       |           BM013382           |                               |
|                       |           BM011394           |                               |
|                       |           BX381425           |                               |
|                       |           CR614331           |                               |
|                       |           BF219950           |                               |
|                       |           BI826824           |                               |
|                       |           BU174990           |                               |
|                       |           BE389047           |                               |
|                       |           CN310139           |                               |
|                       |           BM019925           |                               |
|                       |           BF304592           |                               |
|                       |           BE890148           |                               |
|                       |           CN310141           |                               |
|                       |           BX386587           |                               |
|                       |           BX383303           |                               |
|                       |           BX424730           |                               |
|                       |           BX440562           |                               |
|                       |           CR626715           |                               |
|                       |           BX343087           |                               |
|                       |           CR601745           |                               |
|                       |           BQ651658           |                               |
|                       |           BM808925           |                               |
|                       |           BQ647600           |                               |
|                       |           CB269616           |                               |
|                       |           CD300079           |                               |
|                       |           BE797782           |                               |
|                       |           AW295782           |                               |
|                       |           CA406955           |                               |
|                       |           BM006103           |                               |
|                       |           BM006194           |                               |
|                       |           BM006035           |                               |
|                       |           BQ648502           |                               |
|                       |           BQ645783           |                               |
|                       |           BQ648007           |                               |
|                       |           BQ896683           |                               |
|                       |           BQ648917           |                               |
|                       |           BQ668355           |                               |
|                       |           BE273616           |                               |
|                       |           BE273234           |                               |
|                       |           BE258236           |                               |
|                       |           BG763552           |                               |
|                       |           CN310145           |                               |
|                       |           BE731456           |                               |
|                       |           BI824460           |                               |
|                       |           BI917664           |                               |
|                       |           BX333278           |                               |
|                       |           BX376258           |                               |
|                       |           CR591701           |                               |
|                       |           CR590436           |                               |
|                       |           BE264707           |                               |
|                       |           BF314709           |                               |
|                       |           BM806140           |                               |
|                       |           BE890142           |                               |
|                       |           AA809621           |                               |
|                       |           CN310137           |                               |
|                       |           BE260785           |                               |
|                       |           BG681819           |                               |
|                       |           BE296915           |                               |
|                       |           CR456604           |                               |
|                       |           AW340187           |                               |
|                       |           CN310138           |                               |
|                       |           AA766653           |                               |
|                       |           BE797073           |                               |
|                       |           BM752709           |                               |
|                       |           BX364255           |                               |
|                       |           BM010228           |                               |
|                       |           BG390294           |                               |
|                       |           BM559522           |                               |
|                       |           BM808342           |                               |
|                       |           BX375238           |                               |
|                       |           BX388245           |                               |
|                       |           CR590552           |                               |
|                       |           BQ435934           |                               |
|                       |           BM559515           |                               |
|                       |           BI256741           |                               |
|                       |           BX401616           |                               |
|                       |           CR604635           |                               |
|                       |           BQ948977           |                               |
|                       |           BQ071689           |                               |
|                       |           BE898100           |                               |
|                       |           BM806542           |                               |
|                       |           BM742488           |                               |
|                       |           CR590681           |                               |
|                       |           BX362589           |                               |
|                       |           CN310146           |                               |
|                       |           BG391842           |                               |
|                       |           BM722148           |                               |
|                       |           BG767051           |                               |
|                       |           BG769208           |                               |
|                       |           BG770108           |                               |
|                       |           BG765692           |                               |
|                       |           BG761180           |                               |
|                       |           CD674624           |                               |
|                       |           BX347570           |                               |
|                       |           AI916664           |                               |
|                       |           CV026002           |                               |
|                       |           CV029959           |                               |
|                       |           CV028125           |                               |
|                       |           CV025127           |                               |
|                       |           BM806860           |                               |
|                       |           BF724300           |                               |
|                       |           BM846624           |                               |
|                       |           BG471660           |                               |
|                       |           BQ212920           |                               |
|                       |           BC014939           |                               |
|                       |           BG748376           |                               |
|                       |           BX389102           |                               |
|                       |           BQ717128           |                               |
|                       |           BX439436           |                               |
|                       |           BX347564           |                               |
|                       |           BX389041           |                               |
|                       |           BI824246           |                               |
|                       |           BX428627           |                               |
|                       |           BG003046           |                               |
|                       |           BX326609           |                               |
|                       |           AL044448           |                               |
|                       |           BX372403           |                               |
|                       |           BQ642029           |                               |
|                       |           BM726534           |                               |
|                       |           BX326714           |                               |
|                       |           BG111592           |                               |
|                       |           BX407359           |                               |
|                       |           BX388324           |                               |
|                       |           BE894495           |                               |
|                       |           BG911228           |                               |
|                       |           AI347059           |                               |
|                       |           BX348150           |                               |
|                       |           CV023385           |                               |
|                       |           AA682212           |                               |
|                       |           BQ653655           |                               |
|                       |           BM928548           |                               |
|                       |           BQ647754           |                               |
|                       |           BQ312281           |                               |
|                       |           BX449629           |                               |
|                       |           BX430379           |                               |
|                       |           BX368387           |                               |
|                       |           BX326573           |                               |
|                       |           BG913145           |                               |
|                       |           BU630073           |                               |
|                       |           BG470596           |                               |
|                       |           BF220026           |                               |
|                       |           BX348155           |                               |
|                       |           BX349347           |                               |
|                       |           AA337520           |                               |
|                       |           CN310144           |                               |
|                       |           BX368613           |                               |
|                       |           BX408986           |                               |
|                       |           BQ680927           |                               |
|                       |             H17144           |                               |
|                       |           AW380093           |                               |
|                       |           BU632273           |                               |
|                       |           BQ083396           |                               |
|                       |           BM830370           |                               |
|                       |           BI060719           |                               |
|                       |           BI060809           |                               |
|                       |           BU633419           |                               |
|                       |           CA306909           |                               |
|                       |           AI377953           |                               |
|                       |           BX363860           |                               |
|                       |           AI590865           |                               |
|                       |           BM678017           |                               |
|                       |           BX401615           |                               |
|                       |           BI915635           |                               |
|                       |           BX329548           |                               |
|                       |           AI653491           |                               |
|                       |           BM007141           |                               |
|                       |           BM679089           |                               |
|                       |           BU162463           |                               |
|                       |           BQ878113           |                               |
|                       |           BM760514           |                               |
|                       |           AW246336           |                               |
|                       |           BM681191           |                               |
|                       |           BG222452           |                               |
|                       |           AI890766           |                               |
|                       |           BQ027748           |                               |
|                       |           AA632685           |                               |
|                       |           BM664427           |                               |
|                       |           CB158732           |                               |
|                       |           AW247119           |                               |
|                       |           AA632841           |                               |
|                       |           CD300679           |                               |
|                       |           BG120338           |                               |
|                       |           AA455393           |                               |
|                       |           AI685103           |                               |
|                       |           AI536781           |                               |
|                       |           BU733138           |                               |
|                       |           AI499728           |                               |
|                       |           CF132583           |                               |
|                       |           AI334091           |                               |
|                       |             H18591           |                               |
|                       |           BQ670250           |                               |
|                       |           BQ672174           |                               |
|                       |           BQ675089           |                               |
|                       |           BE885944           |                               |
|                       |           BE302380           |                               |
|                       |           CB120671           |                               |
|                       |           BQ006100           |                               |
|                       |           BM662488           |                               |
|                       |           BM683582           |                               |
|                       |           BM929629           |                               |
|                       |           BQ013670           |                               |
|                       |           AI263364           |                               |
|                       |           AA661847           |                               |
|                       |           AA633816           |                               |
|                       |           AI494262           |                               |
|                       |           BF808449           |                               |
|                       |           BQ439195           |                               |
|                       |           BU739579           |                               |
|                       |           BM713550           |                               |
|                       |             T85179           |                               |
|                       |             T90560           |                               |
|                       |           BI254787           |                               |
|                       |           BG056781           |                               |
|                       |           AI198799           |                               |
|                       |           BM564805           |                               |
|                       |           AA774155           |                               |
|                       |           BE884493           |                               |
|                       |           AA630051           |                               |
|                       |             R15808           |                               |
|                       |           AA306248           |                               |
|                       |           BU742444           |                               |
|                       |           BU742448           |                               |
|                       |           AA603948           |                               |
|                       |           AA661752           |                               |
|                       |           AA456032           |                               |
|                       |           AF136382           |                               |
|                       |           BC047527           |                               |
|                       |           BC037271           |                               |
|                       |           BC009940           |                               |
|                       |           BI597004           |                               |
|                       |           AF218778           |                               |
|                       |           AI971885           |                               |
|                       |           BE857385           |                               |
|                       |           BM805228           |                               |
|                       |           AI884984           |                               |
|                       |           CK904634           |                               |
|                       |           CK904635           |                               |
|                       |           BM068796           |                               |
|                       |           BM054759           |                               |
|                       |           AL021708           |                               |
|                       |           BM543553           |                               |
|                       |           BM545339           |                               |
|                       |             T32933           |                               |
|                       |           AW949565           |                               |
|                       |           BM709297           |                               |
|                       |           CF265005           |                               |
|                       |             Z43824           |                               |
|                       |           BF316823           |                               |
|                       |           BF304283           |                               |
|                       |             T78842           |                               |
|                       |             U79261           |                               |
|                       |           AA324906           |                               |
|                       |           BI550457           |                               |
|                       |           AW888859           |                               |
|                       |           BE703646           |                               |
|                       |           BE703587           |                               |
|                       |           BE703885           |                               |
|                       |           BE703647           |                               |
|                       |           BE703575           |                               |
|                       |           BE703798           |                               |
|                       |           AI372492           |                               |
|                       |           AA323678           |                               |
|                       |           BF964404           |                               |
|                       |           BE263071           |                               |
|                       |           BG002407           |                               |
|                       |             R87578           |                               |
|                       |             H30298           |                               |
|                       |             H38480           |                               |
|                       |           AA505001           |                               |
|                       |             N53101           |                               |
|                       |           AA323084           |                               |
|                       |           AA323104           |                               |
|                       |           AA322904           |                               |
|                       |           AA351451           |                               |
|                       |           AA323870           |                               |
|                       |           AW955180           |                               |
|                       |           BI093547           |                               |
|                       |           BE621145           |                               |
|                       |           BU732443           |                               |
|                       |           BQ270598           |                               |
|                       |           AI369459           |                               |
|                       |           BM664062           |                               |
|                       |           BM672272           |                               |
|                       |           CA433394           |                               |
|                       |           BM671684           |                               |
|                       |           AW003531           |                               |
|                       |           AL044449           |                               |
|                       |           AW246550           |                               |
|                       |           AA490655           |                               |
|                       |           AW960860           |                               |
|                       |           AI376538           |                               |
|                       |           AI085813           |                               |
|                       |           AA948279           |                               |
|                       |           AW135529           |                               |
|                       |           BF511306           |                               |
|                       |           AI383030           |                               |
|                       |           CD557448           |                               |
|                       | *Seq. mapped in strand Minus*| *Seq. mapped in strand Minus* |
|                       |           BU734727           |          AK002952             |
|                       |           AJ572919           |          BC019554             |
|                       |           AA568231           |          AK013765             |
|                       |           BU623699           |          AB060274             |
|                       |           AA857878           |                               |
|                       |           AA580265           |                               |
|                       |           AA595365           |                               |
|                       |           BM987034           |                               |
|                       |           BM988705           |                               |
|                       |           AI340075           |                               |
|                       |           AI560048           |                               |
|                       |           AI417178           |                               |
|                       |           AI673517           |                               |
|                       |           AA613650           |                               |
|                       |           AI553796           |                               |
|                       |           BQ015692           |                               |
|                       |           BM682370           |                               |
|                       |           BM991883           |                               |
|                       |           AW024407           |                               |
|                       |           BQ015730           |                               |
|                       |           CA424249           |                               |
|                       |           CA313422           |                               |
|                       |           CD742601           |                               |
|                       |           BQ772022           |                               |
|                       |           BU675175           |                               |
|                       |           CA307141           |                               |
|                       |           BU687462           |                               |
|                       |           CA310030           |                               |
|                       |           CB305725           |                               |
|                       |           CD370397           |                               |
|                       |           BQ181734           |                               |
|                       |           AW082921           |                               |
|                       |           AW081534           |                               |
|                       |           AL021683           |                               |
|                       |           CR603653           |                               |
|                       |           CR456569           |                               |
|                       |           BE886380           |                               |
|                       |           BG762508           |                               |
|                       |           BQ686256           |                               |
|                       |           BQ878572           |                               |
|                       |           BX391429           |                               |
|                       |           BG765768           |                               |
|                       |           BG248226           |                               |
|                       |           BG250696           |                               |
|                       |           BF207082           |                               |
|                       |           BQ688027           |                               |
|                       |           BE784939           |                               |
|                       |           BM010018           |                               |
|                       |           BI224347           |                               |
|                       |           BU528870           |                               |
|                       |           BQ686179           |                               |
|                       |           BQ689453           |                               |
|                       |           CN409692           |                               |
|                       |           BG337936           |                               |
|                       |           BF036691           |                               |
|                       |           BG680556           |                               |
|                       |           BG392981           |                               |
|                       |           BM787182           |                               |
|                       |           AW248341           |                               |
|                       |           BM844928           |                               |
|                       |           BM787227           |                               |
|                       |           BF244493           |                               |
|                       |           CR736385           |                               |
|                       |           AW407144           |                               |
|                       |           BX389150           |                               |
|                       |           BF338223           |                               |
|                       |           AI970251           |                               |
|                       |           AI571084           |                               |
|                       |           AI733579           |                               |
|                       |           AI201771           |                               |
|                       |           AI281806           |                               |
|                       |           BF594321           |                               |
|                       |           BF732947           |                               |
|                       |           BX391268           |                               |
|                       |           BX409664           |                               |
|                       |           BE502258           |                               |
|                       |           AW006326           |                               |
|                       |           BX409663           |                               |
|                       |           BE857125           |                               |
|                       |           BF196599           |                               |
|                       |           AI638344           |                               |
|                       |           AI285633           |                               |
|                       |           CB216979           |                               |
|                       |           BQ447931           |                               |
|                       |           AW290972           |                               |
|                       |           AI247014           |                               |
|                       |           AI420801           |                               |
|                       |           BQ073080           |                               |
|                       |           AI672004           |                               |
|                       |           BM744178           |                               |
|                       |           AI672802           |                               |
|                       |           CA433599           |                               |
|                       |           BU616165           |                               |
|                       |           BM145054           |                               |
|                       |           AI569158           |                               |
|                       |           BG774839           |                               |
|                       |           BQ446907           |                               |
|                       |           CB046996           |                               |
|                       |           BF591104           |                               |
|                       |           BI789182           |                               |
|                       |           AI521603           |                               |
|                       |           BE045924           |                               |
|                       |           CD366369           |                               |
|                       |           BM972653           |                               |
|                       |           CA309026           |                               |
|                       |           BX407122           |                               |
|                       |           BU681093           |                               |
|                       |           CA307144           |                               |
|                       |           CA308386           |                               |
|                       |           CB850910           |                               |
|                       |           CD370937           |                               |
|                       |           AI401034           |                               |
|                       |           BU730919           |                               |
|                       |           CA306381           |                               |
|                       |           CD743035           |                               |
|                       |           CD368368           |                               |
|                       |           BU740712           |                               |
|                       |           CA313581           |                               |
|                       |           CA308094           |                               |
|                       |           CB529925           |                               |
|                       |           CB851017           |                               |
|                       |           CD368513           |                               |
|                       |           CD370644           |                               |
|                       |           CA307090           |                               |
|                       |           BM980584           |                               |
|                       |           CA307649           |                               |
|                       |           CD367524           |                               |
|                       |           BM977405           |                               |
|                       |           CR610293           |                               |
|                       |             M63193           |                               |
|                       |           BC018160           |                               |
|                       |           BC052211           |                               |
|                       |           AI916027           |                               |
|                       |           BX373739           |                               |
|                       |           BX351430           |                               |
|                       |           BX454513           |                               |
|                       |           BX453941           |                               |
|                       |           CR610057           |                               |
|                       |           AI867456           |                               |
|                       |           CR598122           |                               |
|                       |           AI655096           |                               |
|                       |           AI767382           |                               |
|                       |           BX328748           |                               |
|                       |           CR608523           |                               |
|                       |           BX328765           |                               |
|                       |           BF438612           |                               |
|                       |           BM126771           |                               |
|                       |           BF594332           |                               |
|                       |           BX391357           |                               |
|                       |           CR615106           |                               |
|                       |           CR604852           |                               |
|                       |           BX344179           |                               |
|                       |           CR600249           |                               |
|                       |           BX391269           |                               |
|                       |           CR601424           |                               |
|                       |           CR623877           |                               |
|                       |           CR613512           |                               |
|                       |           CR622550           |                               |
|                       |           BX339651           |                               |
|                       |           CR608324           |                               |
|                       |           BM053014           |                               |
|                       |           BX377184           |                               |
|                       |           BX355755           |                               |
|                       |           CR619786           |                               |
|                       |           AI886225           |                               |
|                       |           AW194360           |                               |
|                       |           AI920823           |                               |
|                       |           AI680300           |                               |
|                       |           AI539116           |                               |
|                       |           AW193431           |                               |
|                       |           AW083891           |                               |
|                       |           AW469163           |                               |
|                       |           AI887701           |                               |
|                       |           BG775441           |                               |
|                       |           BX357175           |                               |
|                       |           BG745076           |                               |
|                       |           BX396989           |                               |
|                       |           CR601687           |                               |
|                       |           BX422174           |                               |
|                       |           BX439236           |                               |
|                       |           CR610417           |                               |
|                       |           BX340133           |                               |
|                       |           CR616233           |                               |
|                       |           BX397831           |                               |
|                       |           CR613294           |                               |
|                       |           BF568988           |                               |
|                       |           BX326915           |                               |
|                       |           BX361251           |                               |
|                       |           BX336371           |                               |
|                       |           CR602986           |                               |
|                       |           BX368766           |                               |
|                       |           AI792181           |                               |
|                       |           BG678454           |                               |
|                       |           BX449571           |                               |
|                       |           BG398097           |                               |
|                       |           BX409269           |                               |
|                       |           BF568681           |                               |
|                       |           BF568589           |                               |
|                       |           BG675214           |                               |
|                       |           BX407313           |                               |
|                       |           CR611811           |                               |
|                       |           BM727471           |                               |
|                       |           BG698965           |                               |
|                       |           BM127080           |                               |
|                       |           BG330416           |                               |
|                       |           BX368740           |                               |
|                       |           BI519577           |                               |
|                       |           BX407183           |                               |
|                       |           BX368799           |                               |
|                       |           BX347989           |                               |
|                       |           BX348052           |                               |
|                       |           BG676192           |                               |
|                       |           BX361520           |                               |
|                       |           CB046997           |                               |
|                       |           BX425149           |                               |
|                       |           BG330140           |                               |
|                       |           BM701776           |                               |
|                       |           BX104808           |                               |
|                       |           BG529990           |                               |
|                       |           BX451138           |                               |
|                       |           BM709018           |                               |
|                       |           BX407211           |                               |
|                       |           BG120390           |                               |
|                       |           BX449686           |                               |
|                       |           BX422175           |                               |
|                       |           BX368650           |                               |
|                       |           BE619353           |                               |
|                       |           BX356007           |                               |
|                       |           BX357176           |                               |
|                       |           BX361252           |                               |
|                       |           BX463244           |                               |
|                       |           BX328749           |                               |
|                       |           BX377185           |                               |
|                       |           BX396990           |                               |
|                       |           BX428583           |                               |
|                       |           BI518582           |                               |
|                       |           BX338527           |                               |
|                       |           BX389069           |                               |
|                       |           BM967142           |                               |
|                       |           BX382387           |                               |
|                       |           BX379147           |                               |
|                       |           BX340134           |                               |
|                       |           BF877451           |                               |
|                       |           BX391270           |                               |
|                       |           BF809758           |                               |
|                       |           CB151384           |                               |
|                       |           BX396386           |                               |
|                       |           BX439305           |                               |
|                       |           BX439237           |                               |
|                       |           BX336372           |                               |
|                       |           BX398673           |                               |
|                       |           BI837150           |                               |
|                       |           BX453435           |                               |
|                       |           BX344180           |                               |
|                       |           BX328766           |                               |
|                       |           BI760678           |                               |
|                       |           BI761742           |                               |
|                       |           BQ353103           |                               |
|                       |           BI521398           |                               |
|                       |           BX428332           |                               |
|                       |           BI962661           |                               |
|                       |           BX339652           |                               |
|                       |           BX368516           |                               |
|                       |           BG674897           |                               |
|                       |           CV409725           |                               |
|                       |           BX458372           |                               |
|                       |           BI917805           |                               |
|                       |           BI521594           |                               |
|                       |           BQ315383           |                               |
|                       |           BI518489           |                               |
|                       |           BQ330733           |                               |
|                       |           BE785287           |                               |
|                       |           BX348084           |                               |
|                       |           BE789895           |                               |
|                       |           BQ225451           |                               |
|                       |           BG331310           |                               |
|                       |           BQ354576           |                               |
|                       |           CV023679           |                               |
|                       |           BX355756           |                               |
|                       |           BX397832           |                               |
|                       |           BQ934956           |                               |
|                       |           BQ312503           |                               |
|                       |           CA487910           |                               |
|                       |           BF674772           |                               |
|                       |           BX366117           |                               |
|                       |           BG331393           |                               |
|                       |           BU186633           |                               |
|                       |           BI909247           |                               |
|                       |           BQ690049           |                               |
|                       |           BG764296           |                               |
|                       |           BX391358           |                               |
|                       |           BG332824           |                               |
|                       |           BF342481           |                               |
|                       |           BX382063           |                               |
|                       |           BM919986           |                               |
|                       |           BQ352223           |                               |
|                       |           BM922641           |                               |
|                       |           BX360221           |                               |
|                       |           BI521637           |                               |
|                       |           AI910048           |                               |
|                       |           CK821234           |                               |
|                       |           BI822371           |                               |
|                       |           BG483721           |                               |
|                       |           AK057214           |                               |
|                       |           BM561973           |                               |
|                       |           BI836252           |                               |
|                       |           BI824473           |                               |
|                       |           BG333238           |                               |
|                       |           BG684315           |                               |
|                       |           CB150480           |                               |
|                       |           BP305645           |                               |
|                       |           BP382062           |                               |
|                       |           BG437342           |                               |
|                       |           BM127130           |                               |
|                       |           BX348024           |                               |
|                       |           BI910457           |                               |
|                       |           BP367768           |                               |
|                       |           BP304545           |                               |
|                       |           CB997854           |                               |
|                       |           BP227170           |                               |
|                       |           CB128864           |                               |
|                       |           BQ942890           |                               |
|                       |           BM680993           |                               |
|                       |           BI771034           |                               |
|                       |           CB131786           |                               |
|                       |           BP367757           |                               |
|                       |           BI838588           |                               |
|                       |           BP227766           |                               |
|                       |           CB152453           |                               |
|                       |           BP227406           |                               |
|                       |           BI907175           |                               |
|                       |           BP305478           |                               |
|                       |           BU161350           |                               |
|                       |           BM837138           |                               |
|                       |           CB150277           |                               |
|                       |           BP366845           |                               |
|                       |           BP382190           |                               |
|                       |           BM146136           |                               |
|                       |           AW629833           |                               |
|                       |           CB150803           |                               |
|                       |           CB151757           |                               |
|                       |           CB130601           |                               |
|                       |           BX361521           |                               |
|                       |           CB116921           |                               |
|                       |           CB113528           |                               |
|                       |             S72487           |                               |
|                       |           BI836393           |                               |
|                       |           AI927002           |                               |
|                       |           CA944860           |                               |
|                       |           BI908735           |                               |
|                       |           CB305873           |                               |
|                       |           AA908323           |                               |
|                       |           AI863369           |                               |
|                       |           AI962367           |                               |
|                       |           BU607448           |                               |
|                       |           CA309320           |                               |
|                       |           CD743074           |                               |
|                       |           BM973820           |                               |
|                       |           CB850961           |                               |
|                       |           BM976451           |                               |
|                       |           AA435920           |                               |
|                       |           BF854615           |                               |
|                       |           AA159016           |                               |
|                       |           BI907276           |                               |
|                       |           BE048068           |                               |
|                       |           BX280388           |                               |
|                       |           AW135645           |                               |
|                       |           AI632959           |                               |
|                       |           AI830779           |                               |
|                       |           AW014906           |                               |
|                       |           BG149596           |                               |
|                       |           AI308857           |                               |
|                       |           AI268481           |                               |
|                       |           AI268720           |                               |
|                       |           AI457142           |                               |
|                       |           AW975809           |                               |
|                       |           BG150240           |                               |
|                       |           CD674272           |                               |
|                       |           AA158597           |                               |
|                       |           AI301738           |                               |
|                       |           AW291097           |                               |
|                       |           AI952155           |                               |
|                       |           BF514393           |                               |
|                       |           AA953200           |                               |
|                       |           BQ027928           |                               |
|                       |           AA428460           |                               |
|                       |           BX100292           |                               |
|                       |           AA988647           |                               |
|                       |           AI806953           |                               |
|                       |           AA832175           |                               |
|                       |           CR593162           |                               |
|                       |           CB240960           |                               |
|                       |           CK300524           |                               |
+-----------------------+------------------------------+-------------------------------+
|      #   12           | *Seq. mapped in strand Plus* | *Seq. mapped in strand Plus*  |
|                       |           AB113647           |          BB646562             |
|                       |           AL832446           |          BE655192             |
|                       |           AK090421           |          CF580431             |
|                       |           BC043154           |          BI153360             |
|                       |                              |          CB056823             |
|                       |                              |          CO044515             |
|                       |                              |          CA326172             |
|                       |                              |          BP427344             |
|                       |                              |          BE685813             |
|                       |                              |          AI850250             |
|                       |                              |          AW048648             |
|                       |                              |          CD774395             |
|                       |                              |          BE456512             |
|                       |                              |          BE988965             |
|                       |                              |          BG967754             |
|                       |                              |          BU700942             |
|                       |                              |          BB647260             |
|                       |                              |          AK019153             |
|                       |                              |          BY710657             |
|                       |                              |          BB849507             |
|                       |                              |          BU695632             |
|                       |                              |          BU694870             |
|                       |                              |          BU697785             |
|                       |                              |          BU695813             |
|                       |                              |          BY233481             |
|                       |                              |          BY078522             |
|                       |                              |          BY066420             |
|                       |                              |          BY061408             |
|                       |                              |          BU939458             |
|                       |                              |          AA003698             |
|                       |                              |          AA061229             |
|                       |                              |          BU841858             |
|                       |                              |          BC061114             |
|                       |                              |          BY280124             |
|                       |                              |          CA464154             |
|                       |                              |          BY280439             |
|                       |                              |          BY255265             |
|                       |                              |          BC049593             |
|                       |                              |          BY106294             |
|                       |                              |          AK003030             |
|                       |                              |          AF070970             |
|                       |                              |          AA016558             |
|                       |                              |          BG806503             |
|                       |                              |          CO040313             |
|                       |                              |          CO040458             |
|                       |                              |          CO040168             |
|                       |                              |          BF466814             |
|                       |                              |          BG298742             |
|                       |                              |          BE947488             |
|                       |                              |          AI842283             |
|                       | *Seq. mapped in strand Minus*| *Seq. mapped in strand Minus* |
|                       |           AF070535           |          BY609236             |
|                       |           CR615919           |          BY613720             |
|                       |           AK023726           |          AA414548             |
|                       |           BC016904           |          AV248714             |
|                       |           CR605529           |          BB502609             |
|                       |           CR610904           |          BB226415             |
|                       |           CR616320           |          BB502927             |
|                       |           CR603519           |          BF322975             |
|                       |           CR592709           |          AW322619             |
|                       |           CR599071           |          BE993292             |
|                       |           CR609290           |          BE983787             |
|                       |           AY502940           |          AV001748             |
|                       |                              |          BB302602             |
|                       |                              |          AV337369             |
|                       |                              |          BB410639             |
|                       |                              |          BB227626             |
|                       |                              |          AV085538             |
|                       |                              |          BB207054             |
|                       |                              |          BB276672             |
|                       |                              |          BB339129             |
|                       |                              |          BB363551             |
|                       |                              |          BB448694             |
|                       |                              |          BB506203             |
|                       |                              |          BB349600             |
|                       |                              |          BB025370             |
|                       |                              |          BB474986             |
|                       |                              |          AV087457             |
|                       |                              |          BB134705             |
|                       |                              |          BB206964             |
|                       |                              |          BB256196             |
|                       |                              |          BB266446             |
|                       |                              |          BB339216             |
|                       |                              |          BB388853             |
|                       |                              |          BB433647             |
|                       |                              |          BE987403             |
|                       |                              |          BB268560             |
|                       |                              |          BB727897             |
|                       |                              |          BB263812             |
|                       |                              |          BB450081             |
|                       |                              |          BB346536             |
|                       |                              |          BF461067             |
|                       |                              |          AI845912             |
|                       |                              |          BB394638             |
|                       |                              |          BB271805             |
|                       |                              |          AI842686             |
|                       |                              |          AI843899             |
|                       |                              |          BY407076             |
|                       |                              |          BY428065             |
|                       |                              |          BY399966             |
|                       |                              |          AA432907             |
+-----------------------+------------------------------+-------------------------------+
|      #   13           | *Seq. mapped in strand Plus* | *Seq. mapped in strand Plus*  |
|                       |           AF068227           |          BY730884             |
|                       |           BF977311           |          AK085741             |
|                       |           BQ719074           |          BB641564             |
|                       |           BG820113           |          BY024060             |
|                       |           BX342662           |          BY170585             |
|                       |           BP306524           |          BY310912             |
|                       |           BX331615           |          BY012998             |
|                       |           BI090566           |          AK043247             |
|                       |           AK075109           |          BY185382             |
|                       |           BP326902           |          BY033908             |
|                       |           CN394777           |          BB640650             |
|                       |           AU280382           |          BY169128             |
|                       |           BP217113           |          AI930426             |
|                       |           BP232529           |          BI111605             |
|                       |           BM849262           |          BY089700             |
|                       |           BF982158           |          BY090490             |
|                       |           BI223533           |          BY096424             |
|                       |           BQ417448           |          BY179445             |
|                       |           CA488543           |          BY161654             |
|                       |           AL546472           |          BY167679             |
|                       |           CR620773           |          CK793632             |
|                       |           AJ705216           |          BY020613             |
|                       |           AA300650           |          BY117840             |
|                       |           BP279017           |          BI077452             |
|                       |           CB215456           |          BY018266             |
|                       |           BF739857           |          BY734042             |
|                       |           AW594284           |          BY168756             |
|                       |           AV723592           |          BY167631             |
|                       |           BM757757           |          BY315466             |
|                       |           BE873363           |          BE632989             |
|                       |           BM833184           |          BI734549             |
|                       |           AI025493           |          BQ926170             |
|                       |           AA635882           |          BY093378             |
|                       |           AA429936           |          BY328681             |
|                       |           CA431029           |          BY011024             |
|                       |           AA625936           |          BB848400             |
|                       |           BU621797           |          BB839746             |
|                       |           AI280843           |          CK627556             |
|                       |           BX370641           |          BY023934             |
|                       |           AA256064           |          BB859747             |
|                       |           BP343308           |          CF724176             |
|                       |           BU633296           |          AW475730             |
|                       |           AI308832           |          BY099145             |
|                       |           AA037599           |          AI746877             |
|                       |           AI452678           |          CD561319             |
|                       |           BF446969           |          BE685938             |
|                       |           AA768586           |          BC025487             |
|                       |           BQ021733           |          CK128221             |
|                       |           BQ446724           |          BI082103             |
|                       |           BU740597           |          BF022302             |
|                       |           BU742417           |          BI456284             |
|                       |           BM683232           |          BI156421             |
|                       |           AW025333           |          BU923466             |
|                       |           CA945081           |          AK032293             |
|                       |           BM978657           |          BB622627             |
|                       |           BM683390           |          BY006534             |
|                       |           BQ018805           |          BY044597             |
|                       |           AA243714           |          BQ443453             |
|                       |           AI765000           |          BI080303             |
|                       |           AI817191           |          AI509780             |
|                       |           AI810141           |          AA866985             |
|                       |           AI289848           |          AK030852             |
|                       |           AW292484           |          BF472345             |
|                       |           AW293114           |          BE947918             |
|                       |           BU753439           |          AW488122             |
|                       |           AA906581           |          BU757884             |
|                       |           AA043351           |          BQ033978             |
|                       |           AI654635           |          BU756040             |
|                       |           AI688173           |                               |
|                       |           BG054542           |                               |
|                       |           AI640506           |                               |
|                       |           AV735818           |                               |
|                       | *Seq. mapped in strand Minus*| *Seq. mapped in strand Minus* |
|                       |           AA844243           |          BE955593             |
|                       |           AW087729           |          AI838956             |
|                       |           AW470840           |                               |
|                       |           AI765345           |                               |
|                       |           CF594359           |                               |
|                       |           BG771402           |                               |
|                       |           BM679862           |                               |
|                       |           BC072448           |                               |
|                       |           AI458796           |                               |
|                       |           AI934213           |                               |
|                       |           BE349514           |                               |
|                       |           CA412572           |                               |
|                       |           AK025484           |                               |
|                       |           AA805942           |                               |
|                       |             R20664           |                               |
|                       |             R37265           |                               |
|                       |           AI141362           |                               |
|                       |           AI017436           |                               |
|                       |           AA418406           |                               |
|                       |           AI640148           |                               |
|                       |           BE326666           |                               |
|                       |           BM984144           |                               |
|                       |           CA314874           |                               |
|                       |           AF126028           |                               |
|                       |           AL833187           |                               |
|                       |           AA488844           |                               |
|                       |           AI675092           |                               |
|                       |           BM677336           |                               |
|                       |           AI092696           |                               |
|                       |             W31374           |                               |
|                       |           BU789511           |                               |
|                       |           CA312518           |                               |
|                       |           CB241486           |                               |
|                       |           BM677409           |                               |
|                       |           CB306395           |                               |
|                       |           CB305591           |                               |
|                       |           BU687656           |                               |
|                       |           BC063796           |                               |
|                       |           AA905463           |                               |
|                       |           CB529595           |                               |
|                       |           AF129532           |                               |
|                       |           BE613543           |                               |
|                       |           BM450028           |                               |
|                       |           CA393470           |                               |
|                       |           AA283163           |                               |
|                       |           BP237914           |                               |
|                       |           BE169359           |                               |
|                       |           BX281018           |                               |
|                       |           BP337248           |                               |
|                       |           CF594069           |                               |
|                       |             Z43904           |                               |
|                       |           BM696524           |                               |
|                       |           CF594324           |                               |
|                       |           BF951187           |                               |
|                       |           BU951829           |                               |
|                       |           CA407367           |                               |
|                       |           BF884971           |                               |
|                       |           BM546850           |                               |
|                       |           BQ424398           |                               |
|                       |           BI604171           |                               |
|                       |           AA852108           |                               |
|                       |           BG705844           |                               |
|                       |           BQ050755           |                               |
|                       |           AI133484           |                               |
|                       |           BX500292           |                               |
|                       |           CN371997           |                               |
|                       |           BI603364           |                               |
|                       |           BQ424278           |                               |
|                       |           CN371995           |                               |
|                       |           AI819336           |                               |
|                       |           CA388641           |                               |
|                       |           CF137797           |                               |
|                       |           BU431633           |                               |
|                       |           CN371996           |                               |
|                       |           AI862084           |                               |
|                       |           BU738920           |                               |
|                       |           BM676730           |                               |
|                       |           CA429008           |                               |
|                       |           AW136774           |                               |
+-----------------------+------------------------------+-------------------------------+
|      #   14           | *Seq. mapped in strand Plus* | *Seq. mapped in strand Plus*  |
|                       |           AL133657           |          BC046319             |
|                       |           BI601151           |            U73941             |
|                       |           BI549532           |          BC022745             |
|                       |           BP208555           |          AK002856             |
|                       |           BP307408           |                               |
|                       |           BG910377           |                               |
|                       |           BG910444           |                               |
|                       |           BI821545           |                               |
|                       |           BE206840           |                               |
|                       |           BI913595           |                               |
|                       |           BP308596           |                               |
|                       |           BP211517           |                               |
|                       |           BQ424069           |                               |
|                       |           BI522472           |                               |
|                       |           BP202435           |                               |
|                       |           BX353645           |                               |
|                       |           AL522962           |                               |
|                       |           AL520447           |                               |
|                       |           BQ109540           |                               |
|                       |           AL533546           |                               |
|                       |           BP211788           |                               |
|                       |           BP212282           |                               |
|                       |           BP213679           |                               |
|                       |           BP213953           |                               |
|                       |           BI596948           |                               |
|                       |           BM922170           |                               |
|                       |           BI919245           |                               |
|                       |           AL527759           |                               |
|                       |           BX393574           |                               |
|                       |           BX445437           |                               |
|                       |           AL533525           |                               |
|                       |           BI522630           |                               |
|                       |           BG820322           |                               |
|                       |           BI757278           |                               |
|                       |             F08403           |                               |
|                       |           BX441366           |                               |
|                       |             Z45123           |                               |
|                       |           BI552408           |                               |
|                       |           CV026502           |                               |
|                       |             R87923           |                               |
|                       |             H38507           |                               |
|                       |           BM466946           |                               |
|                       |           BG913382           |                               |
|                       |           BF347206           |                               |
|                       |             T30979           |                               |
|                       |           BF530718           |                               |
|                       |             F12534           |                               |
|                       |             T17041           |                               |
|                       |           BF528272           |                               |
|                       |           AA323808           |                               |
|                       |           AW955173           |                               |
|                       |           BG818547           |                               |
|                       |             H14157           |                               |
|                       |             R86861           |                               |
|                       |           BF960524           |                               |
|                       |             Z45828           |                               |
|                       |           BI196263           |                               |
|                       |           BC006194           |                               |
|                       |           BX537443           |                               |
|                       |           CR622819           |                               |
|                       |           AF055026           |                               |
|                       |           CR456748           |                               |
|                       |             U93871           |                               |
|                       |           AK123330           |                               |
|                       |           AV728115           |                               |
|                       |           BM544637           |                               |
|                       |           BI820972           |                               |
|                       |           BF963577           |                               |
|                       |           BI034604           |                               |
|                       |           CD106206           |                               |
|                       |           BI767993           |                               |
|                       |           BF958868           |                               |
|                       |           BX452683           |                               |
|                       |           BI754035           |                               |
|                       |           CD251401           |                               |
|                       |           BG912303           |                               |
|                       |           CA488531           |                               |
|                       |           BI523300           |                               |
|                       |           BX453770           |                               |
|                       |           BG740621           |                               |
|                       |           AL520446           |                               |
|                       |           AL522961           |                               |
|                       |           BF528631           |                               |
|                       |           BF528961           |                               |
|                       |           BC013240           |                               |
|                       |           BM675216           |                               |
|                       |           BM714421           |                               |
|                       |           AA323161           |                               |
|                       |           AL527758           |                               |
|                       |           BX413221           |                               |
|                       |           CN345244           |                               |
|                       |           BF931026           |                               |
|                       |           BF931025           |                               |
|                       |             H23638           |                               |
|                       |           BF346805           |                               |
|                       |             H22425           |                               |
|                       |           BF528741           |                               |
|                       |           AI352391           |                               |
|                       |           AA770038           |                               |
|                       |           AI197870           |                               |
|                       |           AW590597           |                               |
|                       |           AW137933           |                               |
|                       |           BG060153           |                               |
|                       |           BM663832           |                               |
|                       |           AW250954           |                               |
|                       |           BM988279           |                               |
|                       |             R51076           |                               |
|                       |           CA942250           |                               |
|                       | *Seq. mapped in strand Minus*| *Seq. mapped in strand Minus* |
|                       |           BC051705           |          AK007019             |
|                       |           AI963398           |                               |
|                       |           BE220336           |                               |
|                       |           BF976738           |                               |
|                       |           BM835539           |                               |
+-----------------------+------------------------------+-------------------------------+
|      #   15           | *Seq. mapped in strand Plus* | *Seq. mapped in strand Plus*  |
|                       |           CA952702           |          BG922284             |
|                       |           CB241743           |          BE987202             |
|                       |           BG220968           |                               |
|                       |           BG189376           |                               |
|                       |           BG206403           |                               |
|                       |           AW451739           |                               |
|                       | *Seq. mapped in strand Minus*| *Seq. mapped in strand Minus* |
|                       |           AI699165           |          BG261699             |
|                       |           AW080169           |          CF729042             |
|                       |           AW083854           |          CN461415             |
|                       |           BQ007202           |          BU788330             |
|                       |           AI089148           |          CF580302             |
|                       |           AW021511           |          AK050376             |
|                       |           AI630726           |          BC057165             |
|                       |           AW615036           |          AK080250             |
|                       |           AA836336           |          BE982970             |
|                       |           AA878977           |          BI739065             |
|                       |           BI491265           |          BF163516             |
|                       |           AI003745           |          CF728092             |
|                       |           AA725421           |          CA541496             |
|                       |             H06657           |          CD771240             |
|                       |             R45946           |            W40847             |
|                       |           BU615294           |          BF662012             |
|                       |           AI280513           |          BU924716             |
|                       |           BM968455           |          CN528004             |
|                       |           AA725415           |          BM508414             |
|                       |           AI752935           |          BM508715             |
|                       |           BQ286109           |          AA036111             |
|                       |             R60310           |          CF734100             |
|                       |           AW130500           |          CF533271             |
|                       |           BU752502           |          CF735432             |
|                       |           BQ416641           |          CF727036             |
|                       |           CA446714           |          CN459776             |
|                       |           BQ573839           |          CF746859             |
|                       |           BQ010340           |          CF746795             |
|                       |           CA312269           |          CF728243             |
|                       |           BM679555           |          BX635627             |
|                       |           BQ776263           |            D19319             |
|                       |           CA450170           |          AI326273             |
|                       |           BU607838           |          BY684629             |
|                       |           AA639160           |          BF538945             |
|                       |           AK025773           |          AV284428             |
|                       |           AI027058           |          BB418100             |
|                       |           AI860031           |          AV301847             |
|                       |             W46950           |          BY504885             |
|                       |           AA452855           |          BY511019             |
|                       |           AA452845           |          BY508739             |
|                       |           BM145738           |          BY513455             |
|                       |           AI811499           |          BY505720             |
|                       |           AI074164           |          BY512095             |
|                       |           AA888540           |          BY498989             |
|                       |           BQ020103           |          BI221046             |
|                       |           AA503336           |          BI348733             |
|                       |           BQ001418           |          AV312246             |
|                       |           CR599221           |          CB246967             |
|                       |           AW139491           |          AK011495             |
|                       |           BU675308           |          BF730421             |
|                       |           BM984126           |          BQ031191             |
|                       |             X71661           |          CN526026             |
|                       |             U09716           |          BY513365             |
|                       |           CN292410           |          CN526523             |
|                       |           CD655336           |          CN456263             |
|                       |           BG024114           |          CK638556             |
|                       |           AV661839           |          BF454347             |
|                       |           BG024052           |          BI683850             |
|                       |           CB154313           |          BF163708             |
|                       |           BF984043           |          CF748021             |
|                       |           BF036666           |          BE952352             |
|                       |           BF982730           |          AW908083             |
|                       |           BU170873           |          AA914683             |
|                       |           CN292421           |          CF733309             |
|                       |           BX399730           |          CF738714             |
|                       |           CR625542           |          CF904599             |
|                       |           AA428522           |          BF099507             |
|                       |           AI151372           |          BF100581             |
|                       |           BF508001           |          AA799160             |
|                       |           BQ001057           |          BF454309             |
|                       |           BQ012074           |          CK639914             |
|                       |           BM999943           |          BG976917             |
|                       |           BQ775759           |          AA061857             |
|                       |           BC032330           |          CK636085             |
|                       |           AA627720           |          AK044539             |
|                       |           AI161429           |          BG869385             |
|                       |           BF002522           |          CF535845             |
|                       |           CR592087           |          BU923511             |
|                       |           AL530614           |          CF531028             |
|                       |           CR618370           |          CA537844             |
|                       |             N41381           |          BQ938630             |
|                       |           CK725133           |          CD550251             |
|                       |           BX399442           |          CD562086             |
|                       |           AI363776           |          CN530506             |
|                       |           AA719977           |          CD562476             |
|                       |           AA057813           |          CF742956             |
|                       |           BF590808           |          CF747419             |
|                       |           BM681360           |          CF534985             |
|                       |           BX345668           |          BB642529             |
|                       |           BU626954           |          AA265127             |
|                       |           BX363972           |          BQ950825             |
|                       |           CR600055           |          CF739800             |
|                       |           BU620084           |          BQ890990             |
|                       |           BE620037           |          CA540158             |
|                       |           BX342918           |          CK634477             |
|                       |           CR609154           |          AA856397             |
|                       |           AA329495           |          CN529516             |
|                       |           BP350710           |          CB526735             |
|                       |           BE156568           |          BI793503             |
|                       |           CN484164           |          AV292111             |
|                       |           AW628072           |          BB295578             |
|                       |           AI188842           |          AV295765             |
|                       |           AI190010           |          CF585557             |
|                       |           BG292562           |          AI481823             |
|                       |           BE156486           |          AV173443             |
|                       |           CB143575           |          AV118603             |
|                       |           CN357404           |          AV054536             |
|                       |           AW371668           |          BF120160             |
|                       |           AW371722           |          AI788798             |
|                       |           CN292408           |          BI412403             |
|                       |           CN292409           |          BQ899596             |
|                       |           CR741557           |          AI787074             |
|                       |           BP335525           |          BI409858             |
|                       |           AA657880           |          CB195486             |
|                       |           AW975039           |          CF727801             |
|                       |           BE619379           |          CB202407             |
|                       |           CN292406           |          CF733508             |
|                       |           CN292415           |          CF727746             |
|                       |           AW610082           |          BI647314             |
|                       |           BX399731           |          CF733052             |
|                       |           BM146936           |          BQ934634             |
|                       |           AI821252           |          CF735161             |
|                       |           BM728183           |          CF733132             |
|                       |           BX399443           |          BE916281             |
|                       |           AL530615           |          AI613602             |
|                       |           BX394194           |          BI248466             |
|                       |           AI183476           |          CF726770             |
|                       |           AA333157           |          CN531290             |
|                       |           AI075150           |          BI739616             |
|                       |           AI654098           |          CF733950             |
|                       |           BQ478189           |          CF733582             |
|                       |           BE349551           |          BG918750             |
|                       |           BM716901           |          BY745385             |
|                       |           BU738487           |          AA501046             |
|                       |           BC017858           |          BI349000             |
|                       |           AA600257           |          BU708751             |
|                       |           AA554560           |          CK624550             |
|                       |           BC008401           |          AA467103             |
|                       |             H74190           |          CN663611             |
|                       |           CN292416           |          CK620889             |
|                       |           BM713032           |          BM730310             |
|                       |           CD557368           |          CA527412             |
|                       |           BU541530           |          BF540556             |
|                       |           BM041560           |          BY710759             |
|                       |           BQ478437           |          BI792676             |
|                       |           AV685092           |          CN664934             |
|                       |           AV685096           |          BU952738             |
|                       |           BX345669           |          CK635023             |
|                       |           BI489501           |          CN665519             |
|                       |           AI970441           |          CN668391             |
|                       |           CN292422           |          AI789351             |
|                       |           AI523946           |          CK391204             |
|                       |           BG388639           |          CN536838             |
|                       |           BE675859           |          BX520690             |
|                       |           AI467855           |          AA939554             |
|                       |           BP380962           |          CF158138             |
|                       |           BU930357           |          CF159443             |
|                       |           CN292412           |          AW258961             |
|                       |             N30854           |          BB610026             |
|                       |           CN292405           |          BY744404             |
|                       |           BI458259           |          CN687706             |
|                       |           BF434384           |          AI876160             |
|                       |           CN292407           |          BB863112             |
|                       |           CN292420           |          CD551429             |
|                       |           CN292414           |          BU551731             |
|                       |           BF679584           |          BB864527             |
|                       |           AA059416           |          BB854101             |
|                       |           CF593732           |          BY157065             |
|                       |           AA446103           |          BY228786             |
|                       |           AA994382           |          BY227058             |
|                       |           BG429448           |          BY095043             |
|                       |           BF195064           |          BY228620             |
|                       |           BF061705           |          BY308909             |
|                       |           BP336685           |          AA289749             |
|                       |           BF105076           |          BY165824             |
|                       |           AA809351           |          BY132734             |
|                       |           BF432301           |          BY767994             |
|                       |           BG900669           |          BY172071             |
|                       |           BI827013           |          BY775672             |
|                       |           AI350185           |          BY012636             |
|                       |           BG900659           |          BY168742             |
|                       |           BP352903           |          BY134281             |
|                       |           AA987781           |          BY163082             |
|                       |           BP354384           |          BY788824             |
|                       |           BP335492           |          BY788996             |
|                       |           BP207575           |          BY150716             |
|                       |           BP338393           |          BY162762             |
|                       |           BP353151           |          BY000293             |
|                       |           BP319198           |          BY781059             |
|                       |           BP351168           |          BY766745             |
|                       |           BP352388           |          BY171199             |
|                       |           BF732760           |          BY779800             |
|                       |           BP354670           |          BY166136             |
|                       |           AW608006           |          BY306022             |
|                       |           CB994131           |          BY148506             |
|                       |           BX643032           |                               |
|                       |           BE169939           |                               |
|                       |           BE813744           |                               |
|                       |           BX643102           |                               |
|                       |           BX484920           |                               |
|                       |           BX643101           |                               |
|                       |           AA223421           |                               |
|                       |           BE327460           |                               |
|                       |           BF317418           |                               |
|                       |           AU098546           |                               |
|                       |           BF667524           |                               |
+-----------------------+------------------------------+-------------------------------+
|      #   16           | *Seq. mapped in strand Plus* | *Seq. mapped in strand Plus*  |
|                       |           AL133091           |          AW123154             |
|                       |                              |          AW124022             |
|                       |                              |          BE954546             |
|                       |                              |          BM932408             |
|                       |                              |          AW123310             |
|                       |                              |          AW494902             |
|                       |                              |          AI591984             |
|                       |                              |          AA189988             |
|                       |                              |          AI662543             |
|                       | *Seq. mapped in strand Minus*| *Seq. mapped in strand Minus* |
|                       |           BC028015           |          BG229542             |
|                       |           AK131251           |          BC025038             |
|                       |           AL049978           |          BC057360             |
|                       |                              |          AK029330             |
|                       |                              |          AK029967             |
|                       |                              |          CA752178             |
|                       |                              |          BG922607             |
|                       |                              |          AA103377             |
|                       |                              |          CD540923             |
+-----------------------+------------------------------+-------------------------------+
|      #   17           | *Seq. mapped in strand Plus* | *Seq. mapped in strand Plus*  |
|                       |           BC032097           |          BY282476             |
|                       |           AJ420457           |          BY726960             |
|                       |           AF318355           |          BB644427             |
|                       |                              |          AK045880             |
|                       |                              |          AB030186             |
|                       |                              |          AF332189             |
|                       |                              |          BB623290             |
|                       |                              |          BB624996             |
|                       |                              |          BB654227             |
|                       |                              |          AK050584             |
|                       |                              |          BF462151             |
|                       |                              |          BE995515             |
|                       |                              |          BF461512             |
|                       |                              |          BF462465             |
|                       |                              |          BI133307             |
|                       |                              |          BF455659             |
|                       |                              |          BF463090             |
|                       |                              |          BF464428             |
|                       |                              |          BF464449             |
|                       |                              |          BF464553             |
|                       |                              |          BF464812             |
|                       |                              |          BG093479             |
|                       |                              |          BE996437             |
|                       |                              |          AI836800             |
|                       |                              |          AI850577             |
|                       |                              |          BE982103             |
|                       |                              |          AW493960             |
|                       |                              |          BE984779             |
|                       |                              |          BE982273             |
|                       |                              |          BE982308             |
|                       |                              |          AI846870             |
|                       |                              |          BE984308             |
|                       |                              |          BE949135             |
|                       |                              |          BE947915             |
|                       |                              |          BE988620             |
|                       |                              |          AI838394             |
|                       |                              |          BE987170             |
|                       |                              |          BE987052             |
|                       |                              |          BE943563             |
|                       |                              |          AW987526             |
|                       |                              |          BY357432             |
|                       |                              |          BB567985             |
|                       |                              |          BQ571212             |
|                       |                              |          BC051954             |
|                       |                              |          BC060081             |
|                       |                              |          BE192520             |
|                       |                              |          BQ179038             |
|                       |                              |          AL362448             |
|                       |                              |          AA015528             |
|                       |                              |          BM946001             |
|                       |                              |          AA471902             |
|                       |                              |          BI415614             |
|                       |                              |          CO041659             |
|                       |                              |          CO041806             |
|                       |                              |          CK780749             |
|                       |                              |          BQ031103             |
|                       |                              |          AI841484             |
|                       |                              |          BE945794             |
|                       |                              |          BE989615             |
|                       |                              |          AI840756             |
|                       |                              |          AA028426             |
|                       | *Seq. mapped in strand Minus*| *Seq. mapped in strand Minus* |
|                       |           AK054908           |            W90822             |
|                       |           AL832531           |                               |
|                       |           CR599011           |                               |
|                       |           CR599965           |                               |
|                       |           CR603416           |                               |
|                       |           BC059956           |                               |
|                       |           CR616236           |                               |
|                       |           BC061888           |                               |
|                       |           CR625351           |                               |
|                       |           CR611143           |                               |
|                       |           CR614579           |                               |
|                       |           CR625763           |                               |
|                       |           BC055011           |                               |
|                       |           CR617249           |                               |
|                       |           AK090585           |                               |
+-----------------------+------------------------------+-------------------------------+
|      #   18           | *Seq. mapped in strand Plus* | *Seq. mapped in strand Plus*  |
|                       |           AV725600           |          BQ175125             |
|                       |           BI906422           |          CK626586             |
|                       |           CD368939           |                               |
|                       |           CA419013           |                               |
|                       |           AV710655           |                               |
|                       |           AV698175           |                               |
|                       | *Seq. mapped in strand Minus*| *Seq. mapped in strand Minus* |
|                       |           AB007969           |          AW494443             |
|                       |           AW444959           |          BC027232             |
|                       |           AW009194           |          BC024665             |
|                       |           BQ045090           |          BQ173980             |
|                       |           AK023828           |          AW120531             |
|                       |           AW207348           |          AK034416             |
|                       |           CN360653           |          AW122819             |
|                       |           AI701781           |          BE957204             |
|                       |           AA923247           |          BE980727             |
|                       |           AI809710           |          CD802554             |
|                       |           AW025200           |          BE954987             |
|                       |           AA912835           |                               |
|                       |           AA759217           |                               |
|                       |           BU684839           |                               |
|                       |           AA557555           |                               |
|                       |           AA662476           |                               |
|                       |           AI363047           |                               |
|                       |           CK299897           |                               |
|                       |           AU148105           |                               |
|                       |           AA573737           |                               |
|                       |           BU608597           |                               |
|                       |           BM016134           |                               |
|                       |           BM016549           |                               |
|                       |           BC014554           |                               |
|                       |           BC025251           |                               |
|                       |           AB047979           |                               |
|                       |           BC068482           |                               |
|                       |           AL134951           |                               |
|                       |           BC049365           |                               |
|                       |           BQ186779           |                               |
|                       |           BE837914           |                               |
|                       |           BE837963           |                               |
|                       |           BF966915           |                               |
|                       |           BI857057           |                               |
|                       |             R22517           |                               |
|                       |           AA431473           |                               |
|                       |           CR738565           |                               |
|                       |           AA431676           |                               |
|                       |           AA635080           |                               |
|                       |           BE697016           |                               |
|                       |           BF091253           |                               |
|                       |           BE843314           |                               |
|                       |           BP198579           |                               |
|                       |           AA689539           |                               |
|                       |           AW902154           |                               |
|                       |           CN265404           |                               |
|                       |           AW974794           |                               |
|                       |           AL117526           |                               |
|                       |           AI200916           |                               |
|                       |           AI371150           |                               |
|                       |           AU253195           |                               |
|                       |           AI138241           |                               |
|                       |           AI444982           |                               |
|                       |           AW272361           |                               |
|                       |           AI610337           |                               |
|                       |           AW081942           |                               |
|                       |             R68650           |                               |
|                       |           AI660907           |                               |
|                       |           BM127923           |                               |
|                       |           BQ718616           |                               |
|                       |           AU154835           |                               |
|                       |           BM700638           |                               |
|                       |           AI655219           |                               |
|                       |           AI394664           |                               |
|                       |           AU147564           |                               |
|                       |           CA444991           |                               |
|                       |           BU733952           |                               |
|                       |           BX097022           |                               |
|                       |           AB033014           |                               |
|                       |           AA689480           |                               |
|                       |           BC040108           |                               |
|                       |           BC032699           |                               |
|                       |           AK022279           |                               |
|                       |           AK025226           |                               |
|                       |           AA775028           |                               |
|                       |           AI873004           |                               |
|                       |             T96597           |                               |
|                       |           BG392563           |                               |
|                       |           BM672104           |                               |
|                       |           BG747463           |                               |
|                       |           CD515371           |                               |
|                       |           BM453354           |                               |
|                       |           BI858994           |                               |
|                       |           AU121821           |                               |
|                       |           BU179353           |                               |
|                       |           BU188988           |                               |
|                       |           BQ683076           |                               |
|                       |           BP345299           |                               |
|                       |           BP305488           |                               |
|                       |           CB996179           |                               |
|                       |           BE736258           |                               |
|                       |           CF780775           |                               |
|                       |           BI113815           |                               |
|                       |           BE250329           |                               |
|                       |           BM128232           |                               |
|                       |           BP256640           |                               |
|                       |           BP313310           |                               |
|                       |           BP274598           |                               |
|                       |           BG328980           |                               |
|                       |           BG036693           |                               |
|                       |           CB987988           |                               |
|                       |           CK819237           |                               |
|                       |           BP200319           |                               |
|                       |           AU253194           |                               |
|                       |           BP221031           |                               |
|                       |           CB989403           |                               |
|                       |           BP275910           |                               |
|                       |           CB155696           |                               |
+-----------------------+------------------------------+-------------------------------+
|      #   19           | *Seq. mapped in strand Plus* | *Seq. mapped in strand Plus*  |
|                       |           BX464372           |          BU525991             |
|                       |           CR599551           |          AK045911             |
|                       |           AL533084           |          BI104905             |
|                       |           BI546531           |          AK016491             |
|                       |           BC035476           |          BC019531             |
|                       |           BU844837           |          BQ951778             |
|                       |           BU849635           |          AW012274             |
|                       |           CA487350           |          AI592398             |
|                       |           AL545414           |          BY084430             |
|                       |           CR620373           |          BY095541             |
|                       |           BX399814           |          CN832945             |
|                       |           BX417423           |          CF104721             |
|                       |           BG704681           |          BY105131             |
|                       |           BG707999           |          BY078448             |
|                       |           BI548144           |          AA755523             |
|                       |           BI600392           |          CF197822             |
|                       |           BG708540           |          CK137373             |
|                       |           BI669979           |          BI988914             |
|                       |           BG708106           |          BY069586             |
|                       |           BI596966           |          BG082536             |
|                       |           AU139545           |                               |
|                       |           AK023674           |                               |
|                       |           BG699727           |                               |
|                       |           BG700206           |                               |
|                       |           BG702116           |                               |
|                       |           CB996556           |                               |
|                       |           BP211992           |                               |
|                       |           BI115218           |                               |
|                       |           BF304475           |                               |
|                       |           BG394370           |                               |
|                       |           AL551444           |                               |
|                       |           BX427985           |                               |
|                       |           AL544055           |                               |
|                       |           CR603402           |                               |
|                       |           AW410900           |                               |
|                       |           BC002449           |                               |
|                       |           BG705925           |                               |
|                       |           BM718744           |                               |
|                       |           BI597567           |                               |
|                       |           BG829306           |                               |
|                       |           BI553529           |                               |
|                       |           BF205499           |                               |
|                       |           BG896911           |                               |
|                       |           BM924255           |                               |
|                       |           BM560630           |                               |
|                       |           AL551936           |                               |
|                       |           CR597707           |                               |
|                       |           AL545843           |                               |
|                       |           BM923206           |                               |
|                       |           AL546572           |                               |
|                       |           CR599516           |                               |
|                       |           AF218006           |                               |
|                       |           AW411072           |                               |
|                       |           BI598284           |                               |
|                       |           BC004128           |                               |
|                       |           BG826674           |                               |
|                       |           BF309494           |                               |
|                       |           BI825072           |                               |
|                       |           BE251881           |                               |
|                       |           BU179913           |                               |
|                       |           BQ683872           |                               |
|                       |           BQ929825           |                               |
|                       |           BE793016           |                               |
|                       |           BE514790           |                               |
|                       |           AL548946           |                               |
|                       |           CR624361           |                               |
|                       |           BX453804           |                               |
|                       |           BG898800           |                               |
|                       |           BG830630           |                               |
|                       |           BI460146           |                               |
|                       |             C17719           |                               |
|                       |           BI159766           |                               |
|                       |           BE264542           |                               |
|                       |           BF203295           |                               |
|                       |           BE299401           |                               |
|                       |           BF309230           |                               |
|                       |           BE298457           |                               |
|                       |           BG037157           |                               |
|                       |             C16961           |                               |
|                       |           BF310120           |                               |
|                       |           AF176923           |                               |
|                       |           CD519669           |                               |
|                       |           CR627385           |                               |
|                       |           AL544229           |                               |
|                       |           CR611215           |                               |
|                       |           AL540364           |                               |
|                       |           CR616869           |                               |
|                       |           AF193044           |                               |
|                       |           BP257027           |                               |
|                       |           BP195948           |                               |
|                       |           BE259279           |                               |
|                       |           BE791414           |                               |
|                       |           BE740283           |                               |
|                       |           AW749956           |                               |
|                       |           BI058546           |                               |
|                       |           BI835585           |                               |
|                       |           BF769649           |                               |
|                       |           AL119516           |                               |
|                       |           BM721285           |                               |
|                       |           CN430841           |                               |
|                       |           CA448500           |                               |
|                       |           BM677018           |                               |
|                       |           CR601176           |                               |
|                       |           BM970173           |                               |
|                       |           CB243239           |                               |
|                       |           BQ447984           |                               |
|                       |           AI281972           |                               |
|                       |           AW136728           |                               |
|                       |           BU727458           |                               |
|                       |             R51352           |                               |
|                       |           CA444982           |                               |
|                       |           AW271276           |                               |
|                       |             R41777           |                               |
|                       |           AL120047           |                               |
|                       |           BM682955           |                               |
|                       |           AW148440           |                               |
|                       | *Seq. mapped in strand Minus*| *Seq. mapped in strand Minus* |
|                       |           BE315311           |          BE629324             |
|                       |           BU731354           |                               |
|                       |           CB243515           |                               |
+-----------------------+------------------------------+-------------------------------+
|      #   20           | *Seq. mapped in strand Plus* | *Seq. mapped in strand Plus*  |
|                       |           CF994733           |          BC012235             |
|                       |           BX400212           |          BI852703             |
|                       |           CR612713           |          BY739686             |
|                       |           CN481865           |          BI150677             |
|                       |           BX443084           |          AK089059             |
|                       |             L35263           |          BY747468             |
|                       |           CF594056           |          BF785034             |
|                       |           CB960462           |          CB228060             |
|                       |           BI597409           |          BU918070             |
|                       |           BI553057           |          BU918780             |
|                       |           BE281156           |          CK620052             |
|                       |           AW503710           |          AF128892             |
|                       |           CF138213           |            U10871             |
|                       |           CK429870           |          BC018227             |
|                       |           BC000092           |          BE915732             |
|                       |           BI822094           |            D83073             |
|                       |           AL528818           |          BY238390             |
|                       |           BC031574           |          BY238948             |
|                       |           CN423752           |          BY251239             |
|                       |           BU846425           |          BY245187             |
|                       |           BX425414           |          BI220793             |
|                       |           AL518249           |          AI504169             |
|                       |           CN423751           |          AA690983             |
|                       |             L35264           |          BY162652             |
|                       |           AF100544           |          BY159070             |
|                       |           BT006933           |          BY167281             |
|                       |           CR536505           |          BY161846             |
|                       |           AB074150           |          BY166906             |
|                       |           BG118518           |          BY159314             |
|                       |           BI027151           |          BY155792             |
|                       |           BI025355           |          BY165316             |
|                       |           BI029924           |          BY157045             |
|                       |           BU180674           |          BY744200             |
|                       |           BX646706           |          BU562238             |
|                       |           BI032193           |          BI256076             |
|                       |             T20238           |          BU610752             |
|                       |           BX348434           |          BI646674             |
|                       |           CB155348           |          BI079023             |
|                       |           BE763902           |          AF195850             |
|                       |             Z25432           |          BI855149             |
|                       |           BQ921685           |          BQ445232             |
|                       |           CF135355           |          BU583496             |
|                       |           BG257788           |            W33729             |
|                       |           BG105989           |            W78296             |
|                       |           CD636615           |            W98996             |
|                       |           AW408700           |          CA317783             |
|                       |           CD636616           |          CF739132             |
|                       |           CF272392           |          BI851973             |
|                       |           BI868255           |          CB055489             |
|                       |           AW848613           |          CN528861             |
|                       |           AW973841           |          BI101758             |
|                       |           CN296961           |            W33432             |
|                       |           AA569377           |          AA002328             |
|                       |           BX393061           |          AI893128             |
|                       |           BU934721           |          BQ478856             |
|                       |             H12036           |          BG976752             |
|                       |           BX496542           |          CB056579             |
|                       |           AI621134           |          BY316821             |
|                       |           BG251020           |          BY317823             |
|                       |           BF980616           |          BF162758             |
|                       |           BM739590           |          BU054931             |
|                       |           BM758641           |          CN724148             |
|                       |           BQ368632           |          CN717530             |
|                       |           BM740435           |          CN692121             |
|                       |           CB269157           |          CF162864             |
|                       |           BG743937           |          CF162943             |
|                       |           CN423754           |          CN689150             |
|                       |           BG954856           |          CN716905             |
|                       |           CN423755           |          CN695079             |
|                       |           AA614446           |          CN721739             |
|                       |           BF183021           |          CF905235             |
|                       |           BE311425           |          BG061663             |
|                       |           AA857845           |          BG061678             |
|                       |           BQ773229           |          CN685162             |
|                       |           CA417668           |          BU058736             |
|                       |           CB529553           |          BF161257             |
|                       |             H11773           |          CN724681             |
|                       |           BQ014266           |          CF894611             |
|                       |           BM981077           |          AA770847             |
|                       |           BU622697           |          BQ573356             |
|                       |           BU621968           |          CD562771             |
|                       |           CA308571           |          CN689305             |
|                       |           BM979114           |                               |
|                       |           BU742996           |                               |
|                       |           BU631727           |                               |
|                       |           CA441540           |                               |
|                       |           AV714129           |                               |
|                       |           BU675555           |                               |
|                       |           BQ028527           |                               |
|                       |           BQ015964           |                               |
|                       |           BM667304           |                               |
|                       |           AA836502           |                               |
|                       |           AI890318           |                               |
|                       |           CR598726           |                               |
|                       |           AI193209           |                               |
|                       |           CN423749           |                               |
|                       |           AW316847           |                               |
|                       |           AI424478           |                               |
|                       |           CA429031           |                               |
|                       |           AI363421           |                               |
|                       |           AI361129           |                               |
|                       |           AI434494           |                               |
|                       |             T47824           |                               |
|                       |           CA436670           |                               |
|                       |           AI803456           |                               |
|                       |           AA479655           |                               |
|                       |           BM840510           |                               |
|                       |           BQ446377           |                               |
|                       |           BU630854           |                               |
|                       |           AI082645           |                               |
|                       |           AW451278           |                               |
|                       |           CD366384           |                               |
|                       |           AI868075           |                               |
|                       |           AW021148           |                               |
|                       | *Seq. mapped in strand Minus*| *Seq. mapped in strand Minus* |
|                       |           BM968780           |          CF927933             |
|                       |           AL518248           |          CF928231             |
|                       |           BM991111           |          BE983942             |
|                       |           BQ447072           |                               |
+-----------------------+------------------------------+-------------------------------+
|      #   21           | *Seq. mapped in strand Plus* | *Seq. mapped in strand Plus*  |
|                       |           BP381619           |          BC027165             |
|                       |           AK074196           |                               |
|                       |           AA256750           |                               |
|                       |           AJ242782           |                               |
|                       |           AJ238994           |                               |
|                       |           BP382204           |                               |
|                       |           AK074204           |                               |
|                       |           AJ272195           |                               |
|                       |           BP338580           |                               |
|                       |           BI909388           |                               |
|                       |           BC033758           |                               |
|                       |           BM561847           |                               |
|                       |           BM922463           |                               |
|                       |           BX338207           |                               |
|                       |           BP304969           |                               |
|                       |           BP382668           |                               |
|                       |           BP295990           |                               |
|                       |           BM906228           |                               |
|                       |           CV030702           |                               |
|                       |           BP340263           |                               |
|                       |           BI040577           |                               |
|                       |           CB133965           |                               |
|                       |           AA625433           |                               |
|                       |           BX390430           |                               |
|                       |           CB148001           |                               |
|                       |           BX327865           |                               |
|                       |           BI545941           |                               |
|                       |           BX102751           |                               |
|                       |           BI756215           |                               |
|                       |             R67241           |                               |
|                       |           CA307252           |                               |
|                       |           AI761520           |                               |
|                       |           AI824043           |                               |
|                       |           AA262556           |                               |
|                       |           AI355999           |                               |
|                       |           AI051397           |                               |
|                       |           AW452188           |                               |
|                       | *Seq. mapped in strand Minus*| *Seq. mapped in strand Minus* |
|                       |           BX493510           |          AK053136             |
+-----------------------+------------------------------+-------------------------------+
|      #   22           | *Seq. mapped in strand Plus* | *Seq. mapped in strand Plus*  |
|                       |           CR618001           |          AW108299             |
|                       |           AF060798           |          BF583108             |
|                       |           BC002618           |                               |
|                       |           CR598797           |                               |
|                       |           AF203910           |                               |
|                       |           AK096954           |                               |
|                       |           CR622571           |                               |
|                       |           CR618122           |                               |
|                       |           BC053998           |                               |
|                       |           CR623144           |                               |
|                       |           AJ005791           |                               |
|                       |           CR603103           |                               |
|                       |           AB020739           |                               |
|                       |           AJ010872           |                               |
|                       |           CR407675           |                               |
|                       |           CR607103           |                               |
|                       |           BC019915           |                               |
|                       |           BC007381           |                               |
|                       |           AK024002           |                               |
|                       | *Seq. mapped in strand Minus*| *Seq. mapped in strand Minus* |
|                       |           AK054731           |          AI843935             |
|                       |           BC018854           |          BC019959             |
+-----------------------+------------------------------+-------------------------------+
|      #   23           | *Seq. mapped in strand Plus* | *Seq. mapped in strand Plus*  |
|                       |           AJ488947           |          BY449707             |
|                       |           AJ488946           |          BY449681             |
|                       |                              |          BQ566782             |
|                       | *Seq. mapped in strand Minus*| *Seq. mapped in strand Minus* |
|                       |           AK024764           |          AK013230             |
|                       |           CR610182           |          BG228999             |
|                       |           CR614952           |          AI849714             |
+-----------------------+------------------------------+-------------------------------+
|      #   24           | *Seq. mapped in strand Plus* | *Seq. mapped in strand Plus*  |
|                       |           AK056324           |          BU698613             |
|                       |           BI161009           |          CF169410             |
|                       |           BI457147           |          BG246967             |
|                       |           BX357502           |          BY108628             |
|                       |           BG118168           |          AA451318             |
|                       |           BG770255           |          BB856896             |
|                       |           AF479813           |          BI685190             |
|                       |           BX398157           |          AA035870             |
|                       |           BM563711           |          BB664706             |
|                       |           BX385943           |          BU522515             |
|                       |           BX335807           |          AY241870             |
|                       |           CR597108           |          BY083306             |
|                       |           BC047701           |          BY150990             |
|                       |           BI835432           |          BB658106             |
|                       |           AL514858           |          AK079165             |
|                       |           CB959820           |          BY084432             |
|                       |           BX365163           |          BY097109             |
|                       |           AL550478           |          BY096263             |
|                       |           CR611794           |          BY094321             |
|                       |           BI827612           |          BY735192             |
|                       |           BC036042           |          BB627459             |
|                       |           CN403992           |          BY772170             |
|                       |           BC040227           |          BB843290             |
|                       |           CF593713           |          BB861010             |
|                       |           BX353643           |          BY074796             |
|                       |           BM560395           |          BY791323             |
|                       |           BM926441           |          BY106285             |
|                       |           BI826251           |          BY785021             |
|                       |           AL526558           |          AA051408             |
|                       |           CR621105           |          BY013422             |
|                       |           BX365301           |          BY014119             |
|                       |           CR625182           |          BY134711             |
|                       |           BM453093           |          BC003230             |
|                       |           BI562351           |            W91292             |
|                       |           CB996581           |          AA222056             |
|                       |           BX324981           |          BF144696             |
|                       |           CR598427           |          BI456214             |
|                       |           BX376202           |          BY215248             |
|                       |           AY166714           |          BI081778             |
|                       |           CR615620           |          CB181490             |
|                       |           BM545926           |          BE310917             |
|                       |           BG421676           |          AA518317             |
|                       |           BG761479           |          BY236792             |
|                       |           BG766427           |          BB563730             |
|                       |           BI759674           |          BY206587             |
|                       |           BU542240           |          BY079195             |
|                       |           BQ919974           |          BY092769             |
|                       |           AL544210           |          AA027700             |
|                       |           BX364819           |          BU149068             |
|                       |           CR619122           |          CN664584             |
|                       |           BQ956538           |          BG245830             |
|                       |           BU543936           |          BG245740             |
|                       |           BX424909           |          CK624083             |
|                       |           BX383396           |          BQ961236             |
|                       |           CR618108           |          BY704139             |
|                       |           BX387716           |          AK003882             |
|                       |           BM906504           |          BG321883             |
|                       |           BX394208           |          AA668001             |
|                       |           BX380438           |          BY012535             |
|                       |           CR616563           |          BU699288             |
|                       |           CR613081           |          CK129161             |
|                       |           CR606566           |          BY176403             |
|                       |           CR594798           |          CB182089             |
|                       |           CR608574           |          BY185333             |
|                       |           BE903609           |          BY118996             |
|                       |           AL551269           |          BY117659             |
|                       |           CR621723           |          BG062355             |
|                       |           BX375105           |          BE370943             |
|                       |           BX356518           |          BG806642             |
|                       |           AL542357           |            W82426             |
|                       |           CR599238           |            W91293             |
|                       |           CR596167           |          CB057574             |
|                       |           BI822650           |          CN456296             |
|                       |           BI759175           |          CN455592             |
|                       |           CB994359           |          BI852841             |
|                       |           BC023509           |          BG916614             |
|                       |           BM920223           |          CB321028             |
|                       |           BG748053           |          BU562330             |
|                       |           BX280358           |          CF581136             |
|                       |           BQ068255           |          BI792707             |
|                       |           CN403993           |          BE370777             |
|                       |           BC023591           |          AW647586             |
|                       |           BG828846           |          BQ548045             |
|                       |           CN403991           |          BQ746150             |
|                       |           BI518832           |          CF734924             |
|                       |           BX394842           |                               |
|                       |           CR593528           |                               |
|                       |           BE747130           |                               |
|                       |           AL527977           |                               |
|                       |           CR606357           |                               |
|                       |           BQ924873           |                               |
|                       |           BI087043           |                               |
|                       |           BM563036           |                               |
|                       |           BU150300           |                               |
|                       |           BX354638           |                               |
|                       |           CR589961           |                               |
|                       |           BU150171           |                               |
|                       |           CD514412           |                               |
|                       |           BU621695           |                               |
|                       |           BX952540           |                               |
|                       |           AW327517           |                               |
|                       |           BM906164           |                               |
|                       |           BP291141           |                               |
|                       |           BG251098           |                               |
|                       |           AK075075           |                               |
|                       |           AL042115           |                               |
|                       |           BU855029           |                               |
|                       |           BP337309           |                               |
|                       |           BP367481           |                               |
|                       |           BF813945           |                               |
|                       |           BU599202           |                               |
|                       |             R14227           |                               |
|                       |             H15332           |                               |
|                       |           BX340224           |                               |
|                       |             Z46193           |                               |
|                       |           BF813939           |                               |
|                       |           CD559142           |                               |
|                       |           CD518077           |                               |
|                       |           CN403998           |                               |
|                       |           BG323505           |                               |
|                       |           BG697860           |                               |
|                       |           BE907226           |                               |
|                       |           BX335806           |                               |
|                       |           BF981832           |                               |
|                       |           BI199176           |                               |
|                       |           BG744169           |                               |
|                       |           AW957722           |                               |
|                       |           AA351880           |                               |
|                       |           BM918233           |                               |
|                       |           BQ054182           |                               |
|                       |           CA309177           |                               |
|                       |           CA308811           |                               |
|                       |           BQ014772           |                               |
|                       |           BU622946           |                               |
|                       |           BQ008437           |                               |
|                       |           BU682824           |                               |
|                       |           BQ004224           |                               |
|                       |           BM476701           |                               |
|                       |           BU682418           |                               |
|                       |           CA421948           |                               |
|                       |           BU686202           |                               |
|                       |           BQ446404           |                               |
|                       |           BM971810           |                               |
|                       |           BM145699           |                               |
|                       |           CB118643           |                               |
|                       |           AV735997           |                               |
|                       |           AI554669           |                               |
|                       |           BF035607           |                               |
|                       |           AI769369           |                               |
|                       |           CB123487           |                               |
|                       |           AI199561           |                               |
|                       |           CB119462           |                               |
|                       |           BU074011           |                               |
|                       |           AW006534           |                               |
|                       |           AI990389           |                               |
|                       |           AI631814           |                               |
|                       |           AW016914           |                               |
|                       |           BU742788           |                               |
|                       |           BM970780           |                               |
|                       |           AL047918           |                               |
|                       |           CA436568           |                               |
|                       |           BM144908           |                               |
|                       |           AW327573           |                               |
|                       |           AI685615           |                               |
|                       |           AI492163           |                               |
|                       |           BQ021434           |                               |
|                       |           BU618779           |                               |
|                       |           CA429909           |                               |
|                       |           BQ018899           |                               |
|                       |           AI127476           |                               |
|                       |             R40646           |                               |
|                       |           BF446192           |                               |
|                       |           AA609306           |                               |
|                       |           AI203197           |                               |
|                       |           AW235727           |                               |
|                       | *Seq. mapped in strand Minus*| *Seq. mapped in strand Minus* |
|                       |           BU737297           |          CF356959             |
+-----------------------+------------------------------+-------------------------------+
|      #   25           | *Seq. mapped in strand Plus* | *Seq. mapped in strand Plus*  |
|                       |           CD705322           |          AK008840             |
|                       |           BG749354           |          AW822108             |
|                       |           CD691874           |          AW489390             |
|                       |           BI666950           |          BG142689             |
|                       |           BI668768           |          AI195773             |
|                       |           CD690289           |          BX517909             |
|                       |           AL703545           |          BG797561             |
|                       |           BX482659           |                               |
|                       |           CD683922           |                               |
|                       |           BG699229           |                               |
|                       |           BF797383           |                               |
|                       |           AK172845           |                               |
|                       |           BC013173           |                               |
|                       |           CR747381           |                               |
|                       |           CA392843           |                               |
|                       |           AK027549           |                               |
|                       |           AB075852           |                               |
|                       |           BQ439291           |                               |
|                       |           BU500574           |                               |
|                       |           CA392052           |                               |
|                       |           CA392054           |                               |
|                       |           BE882864           |                               |
|                       |           BI768690           |                               |
|                       |           BU072529           |                               |
|                       |           AU253442           |                               |
|                       |           BM010902           |                               |
|                       |           CK902426           |                               |
|                       |           BU161287           |                               |
|                       |           CD109369           |                               |
|                       |           CN263211           |                               |
|                       |           BG165215           |                               |
|                       |           BG772050           |                               |
|                       |           BG744401           |                               |
|                       |           BG620045           |                               |
|                       |           BF332134           |                               |
|                       |           CN263205           |                               |
|                       |           BU196560           |                               |
|                       |           BQ680025           |                               |
|                       |           BQ677455           |                               |
|                       |           AA352611           |                               |
|                       |           AA088309           |                               |
|                       |             Z43391           |                               |
|                       |           CN263208           |                               |
|                       |             T30428           |                               |
|                       |           AW958044           |                               |
|                       |           CB140943           |                               |
|                       |           CN263209           |                               |
|                       |           CN263212           |                               |
|                       |           AW276830           |                               |
|                       |           BG745046           |                               |
|                       |           BG315180           |                               |
|                       |           BI018452           |                               |
|                       |           AU253443           |                               |
|                       |           CA430171           |                               |
|                       |           BG983496           |                               |
|                       |           BU072257           |                               |
|                       |           BM972597           |                               |
|                       |           BG181454           |                               |
|                       |           AI206557           |                               |
|                       |           BM682578           |                               |
|                       |           BM726151           |                               |
|                       |           CK902427           |                               |
|                       |           AI750662           |                               |
|                       |           AI459716           |                               |
|                       |           AI128839           |                               |
|                       |           AA165677           |                               |
|                       |           AA159690           |                               |
|                       |           BG028856           |                               |
|                       |           AI471732           |                               |
|                       |           BU177039           |                               |
|                       |           AI246400           |                               |
|                       |             Z39464           |                               |
|                       |           AI758780           |                               |
|                       |           BG122939           |                               |
|                       |           AI417879           |                               |
|                       |           CF121349           |                               |
|                       |           AL834402           |                               |
|                       |           BM477487           |                               |
|                       |           BU675829           |                               |
|                       |           CD580076           |                               |
|                       |             H47748           |                               |
|                       |             H47749           |                               |
|                       |           BX094585           |                               |
|                       |           BF515098           |                               |
|                       |           AI911927           |                               |
|                       | *Seq. mapped in strand Minus*| *Seq. mapped in strand Minus* |
|                       |           BU686051           |          BE987060             |
+-----------------------+------------------------------+-------------------------------+
|      #   26           | *Seq. mapped in strand Plus* | *Seq. mapped in strand Plus*  |
|                       |           AF385926           |          AY046947             |
|                       |           BC000140           |          BC006915             |
|                       |             X14608           |          BC049802             |
|                       |           AK055982           |          AK049052             |
|                       |             Z36837           |                               |
|                       |             M13572           |                               |
|                       |             S79219           |                               |
|                       |           AK027256           |                               |
|                       |           AF339804           |                               |
|                       |             M26121           |                               |
|                       |           AK055420           |                               |
|                       | *Seq. mapped in strand Minus*| *Seq. mapped in strand Minus* |
|                       |           AK093228           |          AK050251             |
|                       |           AK091974           |                               |
|                       |           AK095850           |                               |
|                       |           BC001077           |                               |
|                       |           BC004360           |                               |
|                       |           BC034289           |                               |
+-----------------------+------------------------------+-------------------------------+
|      #   27           | *Seq. mapped in strand Plus* | *Seq. mapped in strand Plus*  |
|                       |           BX118576           |          AK080379             |
|                       |             R44998           |          AK049190             |
|                       |                              |          AK042060             |
|                       |                              |          AK043733             |
|                       |                              |          AK088624             |
|                       |                              |          BC024691             |
|                       |                              |          AK005044             |
|                       |                              |          BC056481             |
|                       | *Seq. mapped in strand Minus*| *Seq. mapped in strand Minus* |
|                       |           CN359134           |          AK085526             |
|                       |           BE907609           |          AK049389             |
|                       |           BQ001026           |          BC022730             |
|                       |           AI510707           |          AK003900             |
|                       |           AI140946           |                               |
|                       |           AF161383           |                               |
|                       |           AA534724           |                               |
|                       |           AI199608           |                               |
|                       |           BU728457           |                               |
|                       |           AI697063           |                               |
|                       |           AW137082           |                               |
|                       |           BU600881           |                               |
|                       |           BU601943           |                               |
|                       |           AW273586           |                               |
|                       |           AI863060           |                               |
|                       |           AI051794           |                               |
|                       |           AI079384           |                               |
|                       |             W78114           |                               |
|                       |           AI889226           |                               |
|                       |           AI377555           |                               |
|                       |           AI745221           |                               |
|                       |           AI741076           |                               |
|                       |           CA418587           |                               |
|                       |             N80942           |                               |
|                       |           BQ421248           |                               |
|                       |           BE794699           |                               |
|                       |           BU597257           |                               |
|                       |           BF526092           |                               |
|                       |           BG612415           |                               |
|                       |           BM665681           |                               |
|                       |           BG527854           |                               |
|                       |           AA557225           |                               |
|                       |           AI129043           |                               |
|                       |           AI494241           |                               |
|                       |           AI422896           |                               |
|                       |           BU564571           |                               |
|                       |           AI743635           |                               |
|                       |           BU171518           |                               |
|                       |           BM663227           |                               |
|                       |           BG166392           |                               |
|                       |           BQ021000           |                               |
|                       |           BU682330           |                               |
|                       |           BU740313           |                               |
|                       |           BU738087           |                               |
|                       |           BU535331           |                               |
|                       |           BU600761           |                               |
|                       |           AW250853           |                               |
|                       |           BC003515           |                               |
|                       |           AV710403           |                               |
|                       |           BU855159           |                               |
|                       |           BM972693           |                               |
|                       |           BU753473           |                               |
|                       |           AW971003           |                               |
|                       |           AV726616           |                               |
|                       |           BM690376           |                               |
|                       |           BM711870           |                               |
|                       |           BF217530           |                               |
|                       |           BG777412           |                               |
|                       |           AA876784           |                               |
|                       |           BE906570           |                               |
|                       |             F36541           |                               |
|                       |             F31111           |                               |
|                       |           BU602815           |                               |
|                       |           AJ707717           |                               |
|                       |           BM691960           |                               |
|                       |           AA027916           |                               |
|                       |           BG497607           |                               |
|                       |           BU602527           |                               |
|                       |           BE018630           |                               |
|                       |             W79821           |                               |
|                       |           AV756016           |                               |
|                       |           BM700506           |                               |
|                       |           AW250099           |                               |
|                       |             T63010           |                               |
|                       |           BM824014           |                               |
|                       |           AA531042           |                               |
|                       |           CB106262           |                               |
|                       |           BP432146           |                               |
|                       |           CB994023           |                               |
|                       |           AV743943           |                               |
+-----------------------+------------------------------+-------------------------------+
|      #   28           | *Seq. mapped in strand Plus* | *Seq. mapped in strand Plus*  |
|                       |           BP275460           |          BY293615             |
|                       |           BP195403           |          BY099251             |
|                       |           BI465019           |          CA556143             |
|                       |           BP199515           |          BQ930215             |
|                       |           BI770664           |          BU557575             |
|                       |           BI770588           |          BY166490             |
|                       |           BP329307           |          BY164335             |
|                       |           CB993965           |          BY167844             |
|                       |           BG702261           |          BY161486             |
|                       |           AL553640           |          BY155810             |
|                       |           CR622707           |          BY744211             |
|                       |           BI549730           |          BF578054             |
|                       |           BI667061           |          BY304028             |
|                       |           BI545037           |          BU559401             |
|                       |           BG717682           |          BY250569             |
|                       |           BG713999           |          BY184364             |
|                       |           BP214101           |          BY196660             |
|                       |           CB959632           |          BY196662             |
|                       |           CB990225           |          BY335136             |
|                       |           CB988691           |          BY195225             |
|                       |           CD627458           |          AU079079             |
|                       |           BU073197           |          BY207735             |
|                       |           CD696751           |          BY257812             |
|                       |           BG530411           |          BY321675             |
|                       |           CB994797           |          BY318438             |
|                       |           BG535065           |          BY738065             |
|                       |           BM838083           |          BY259742             |
|                       |           BP326054           |          BY102399             |
|                       |           CD693720           |          BY283697             |
|                       |           CD686164           |          BY084893             |
|                       |           AF172940           |          AA771160             |
|                       |           CD708842           |          CB601512             |
|                       |           CD697249           |          BY187647             |
|                       |           BM562331           |          BY069305             |
|                       |           AL552786           |          BY186427             |
|                       |           CR617677           |          BY193020             |
|                       |           AA346660           |          BY253480             |
|                       |           AF151885           |          BY253378             |
|                       |           CB961179           |          BY740735             |
|                       |           BG772099           |          BY120547             |
|                       |           BU930328           |          BY116997             |
|                       |           BG717898           |          BY117854             |
|                       |           BF184877           |          BY118200             |
|                       |           BI546719           |          BY084085             |
|                       |           BQ639083           |          BY119837             |
|                       |           CD695888           |          AK049096             |
|                       |           BF211545           |          BY232757             |
|                       |           AV761220           |          BY777745             |
|                       |           BI599635           |          BY180818             |
|                       |           AA683291           |          BB644879             |
|                       |           BP193830           |          BB652638             |
|                       |           CD699496           |          BY734137             |
|                       |           BP368671           |          BB650415             |
|                       |           BP346875           |          BY748955             |
|                       |           BP230019           |          AK049644             |
|                       |           AL710985           |          AK046281             |
|                       |           BP229608           |          CA490638             |
|                       |           AL602959           |          CA494986             |
|                       |           BM828932           |          BY010387             |
|                       |             W04863           |          AL361945             |
|                       |           AU280316           |          BI157597             |
|                       |           CD688108           |          BY321770             |
|                       |           BP431114           |          BY319944             |
|                       |           BP363326           |          CA558903             |
|                       |           BP259511           |          BF539467             |
|                       |           BF030937           |          BY035474             |
|                       |           BU195542           |          BY285710             |
|                       |           AV751996           |          CK022584             |
|                       |           CN297203           |          BI652558             |
|                       |           AL530903           |          CN686460             |
|                       |           BX325336           |          CK019946             |
|                       |           AL541242           |          BE692702             |
|                       |           AL549105           |          BE849485             |
|                       |           AL554943           |          BE849607             |
|                       |           CR612144           |          BY175005             |
|                       |           CR590716           |          BY267113             |
|                       |           CD522800           |          BY199235             |
|                       |           BP209332           |          BY722632             |
|                       |           BQ067843           |          AI120668             |
|                       |           BP430969           |          BY709702             |
|                       |           BG474659           |          BF682889             |
|                       |           AL543109           |          AK010201             |
|                       |           CR623255           |          BB580085             |
|                       |           AF135161           |          BY230688             |
|                       |           AV698320           |          BU562805             |
|                       |           AV684273           |          BY100827             |
|                       |           BI559428           |          AA980830             |
|                       |             C17218           |          BY081190             |
|                       |           BI860696           |          BF144208             |
|                       |           BP290884           |          CN705932             |
|                       |           CA394366           |          CN709474             |
|                       |           BI824436           |          BI250745             |
|                       |           AA402577           |          BG918533             |
|                       |           AL544001           |          AA104232             |
|                       |           CR612194           |          BE859449             |
|                       |             N55788           |          BF715347             |
|                       |             R96313           |          BU946378             |
|                       |           BG260733           |          BY297842             |
|                       |           BM716887           |          BY200813             |
|                       |           AA373216           |          BY186681             |
|                       |           CD518057           |          BY192167             |
|                       |           CD559122           |          BY188635             |
|                       |           CD691496           |          BY179717             |
|                       |           CN297204           |          BY290364             |
|                       |           BG723345           |          BY278050             |
|                       |           BG121323           |          BY280729             |
|                       |           AA321934           |          BB863353             |
|                       |           AA148436           |          BY265709             |
|                       |           BM272778           |          CF902858             |
|                       |           BI462432           |          BY156371             |
|                       |           CD638878           |          BY196438             |
|                       |           BE378300           |          AI181225             |
|                       |           BM146774           |          BB849246             |
|                       |           CK001079           |          BY039452             |
|                       |           BX388217           |          BY051971             |
|                       |           BG502926           |          BY050728             |
|                       |           BG502454           |          BY050654             |
|                       |           BG617809           |          BY059240             |
|                       |           BG502888           |          CK021714             |
|                       |           BU738473           |          CA491602             |
|                       |           BP275199           |          BY084377             |
|                       |           BP342419           |          BG088701             |
|                       |           BE972332           |          AI152624             |
|                       |           BF216016           |          BG089449             |
|                       |           BM835110           |          BF122600             |
|                       |           BU663276           |          BQ176545             |
|                       |           BI755289           |          AI120607             |
|                       |           CD522805           |          BC025125             |
|                       |           CK002290           |          AI836887             |
|                       |           BP288883           |          AW124977             |
|                       |           CD512415           |          AA821347             |
|                       |           CB984773           |                               |
|                       |           CD522688           |                               |
|                       |           BG537131           |                               |
|                       |           BE243862           |                               |
|                       |           BE246322           |                               |
|                       |           BE244420           |                               |
|                       |           BE244882           |                               |
|                       |           BU660427           |                               |
|                       |           BP243479           |                               |
|                       |           BP293480           |                               |
|                       |           BG547165           |                               |
|                       |           AK025730           |                               |
|                       |           CD700647           |                               |
|                       |           BM710119           |                               |
|                       |           BG034785           |                               |
|                       |           BQ067643           |                               |
|                       |           BF081529           |                               |
|                       |           BQ050980           |                               |
|                       |           BF171121           |                               |
|                       |             N29206           |                               |
|                       |           AA452695           |                               |
|                       |           BG740849           |                               |
|                       |           BF131934           |                               |
|                       |           CN297206           |                               |
|                       |           BF795697           |                               |
|                       |           BC000836           |                               |
|                       |             N28458           |                               |
|                       |           CN297207           |                               |
|                       |           AL546164           |                               |
|                       |           BX361082           |                               |
|                       |           CR617186           |                               |
|                       |           CR620890           |                               |
|                       |           AA325890           |                               |
|                       |           AA083939           |                               |
|                       |           BU662554           |                               |
|                       |             H87410           |                               |
|                       |             W60562           |                               |
|                       |             W32702           |                               |
|                       |           AA319796           |                               |
|                       |             W95796           |                               |
|                       |           BE787658           |                               |
|                       |           BQ216254           |                               |
|                       |           AV748401           |                               |
|                       |           BM172205           |                               |
|                       |           BG704923           |                               |
|                       |           BI463690           |                               |
|                       |           AA360583           |                               |
|                       |           AA382795           |                               |
|                       |           AU099011           |                               |
|                       |           AW402599           |                               |
|                       |           AA402458           |                               |
|                       |           CA397005           |                               |
|                       |           BG165908           |                               |
|                       |           CA407187           |                               |
|                       |           BG698732           |                               |
|                       |           BP327667           |                               |
|                       |           BG571402           |                               |
|                       |           BX386775           |                               |
|                       |           CB177259           |                               |
|                       |             N40125           |                               |
|                       |           CN297202           |                               |
|                       |           CB177263           |                               |
|                       |           AL576579           |                               |
|                       |           CK005448           |                               |
|                       |             W05840           |                               |
|                       |           AA115050           |                               |
|                       |           AA027923           |                               |
|                       |           AA150348           |                               |
|                       |             H49227           |                               |
|                       |           BG142207           |                               |
|                       |           AL575970           |                               |
|                       |           BF175645           |                               |
|                       |           AA210879           |                               |
|                       |           BP378349           |                               |
|                       |             N35260           |                               |
|                       |           AI902181           |                               |
|                       |           AA411860           |                               |
|                       |           CA306709           |                               |
|                       |           CA748512           |                               |
|                       |           BU622025           |                               |
|                       |           CB243757           |                               |
|                       |           CD743052           |                               |
|                       |           BU619839           |                               |
|                       |           BQ009319           |                               |
|                       |           BU607944           |                               |
|                       |           BM671293           |                               |
|                       |           CA308541           |                               |
|                       |           BQ020381           |                               |
|                       |           CA429244           |                               |
|                       |           CN479498           |                               |
|                       |           BM676627           |                               |
|                       |           CA445608           |                               |
|                       |           AI263830           |                               |
|                       |           CB243573           |                               |
|                       |           BU739044           |                               |
|                       |           AW103450           |                               |
|                       |           AW575257           |                               |
|                       |           BQ021193           |                               |
|                       |           BM750220           |                               |
|                       |           BU620057           |                               |
|                       |           BQ020977           |                               |
|                       |           CD369723           |                               |
|                       |           AI342144           |                               |
|                       |           CA448243           |                               |
|                       |           AI066682           |                               |
|                       |           AI936317           |                               |
|                       |           AI150177           |                               |
|                       |           AA453622           |                               |
|                       |           AA777078           |                               |
|                       |           AA152318           |                               |
|                       |           CF528944           |                               |
|                       |           AI927370           |                               |
|                       |           BF507584           |                               |
|                       |           AA741027           |                               |
|                       |           AA411995           |                               |
|                       |           AI627657           |                               |
|                       |           AA621431           |                               |
|                       |           AI272131           |                               |
|                       |             F35729           |                               |
|                       |           CA433248           |                               |
|                       |           BM969612           |                               |
|                       |           AA825373           |                               |
|                       |           AA682763           |                               |
|                       |           CR615384           |                               |
|                       |           AI351989           |                               |
|                       |           CA428948           |                               |
|                       |           AA027857           |                               |
|                       |           BQ446715           |                               |
|                       |           AW468405           |                               |
|                       |           CA414762           |                               |
|                       |           AI149536           |                               |
|                       |           BQ773130           |                               |
|                       |           BU684077           |                               |
|                       |           BM995172           |                               |
|                       |           BM998111           |                               |
|                       |           AV734814           |                               |
|                       |           AW150662           |                               |
|                       |           BM664463           |                               |
|                       |           AW043868           |                               |
|                       |           AW105020           |                               |
|                       |           BQ024964           |                               |
|                       |           BQ025433           |                               |
|                       |             C05924           |                               |
|                       |           BM145632           |                               |
|                       |           BM856482           |                               |
|                       |           AA883267           |                               |
|                       |           BI438633           |                               |
|                       |           AI963269           |                               |
|                       |           AI804705           |                               |
|                       |           AI961457           |                               |
|                       |           BX109807           |                               |
|                       |           CA443615           |                               |
|                       |             W32538           |                               |
|                       |             W44808           |                               |
|                       |           AA703773           |                               |
|                       |           AA446817           |                               |
|                       |           AA083837           |                               |
|                       |           BU950322           |                               |
|                       |           AI983483           |                               |
|                       |           CA945064           |                               |
|                       |           AI950667           |                               |
|                       |           AI865137           |                               |
|                       |           BM787082           |                               |
|                       |           AI380799           |                               |
|                       |             W44802           |                               |
|                       |           BM681642           |                               |
|                       |           BM675610           |                               |
|                       |             H24218           |                               |
|                       |           AI950580           |                               |
|                       |           AW194993           |                               |
|                       |           CB149862           |                               |
|                       |           AI926352           |                               |
|                       |             R38842           |                               |
|                       |           AI569424           |                               |
|                       |           AI281717           |                               |
|                       | *Seq. mapped in strand Minus*| *Seq. mapped in strand Minus* |
|                       |           BG182761           |          AI842696             |
|                       |           BM713647           |                               |
|                       |           BM684284           |                               |
+-----------------------+------------------------------+-------------------------------+
|      #   29           | *Seq. mapped in strand Plus* | *Seq. mapped in strand Plus*  |
|                       |           CR618252           |          AF104411             |
|                       |           BX407020           |          BY284056             |
|                       |           BX465056           |          BY263084             |
|                       |           BX418684           |          BY095840             |
|                       |           CR604795           |          BY101890             |
|                       |           BX460711           |          BY100494             |
|                       |           CR616689           |          BY105951             |
|                       |           AL833942           |          BY094840             |
|                       |           BE891766           |          BU706797             |
|                       |           CN429605           |          BC055738             |
|                       |           CR456572           |          BQ550645             |
|                       |           AF285107           |          BY122079             |
|                       |           AF285108           |          BY784134             |
|                       |           AF285109           |          CA978839             |
|                       |           BQ340411           |          BY713785             |
|                       |           BQ340389           |          BB645723             |
|                       |           BQ932341           |          AK046722             |
|                       |           CN429601           |          BY251192             |
|                       |           BF962138           |          BB611398             |
|                       |           BG008389           |          BY079525             |
|                       |           BG995413           |          BB618240             |
|                       |           BX369018           |          AK030668             |
|                       |           BX465055           |          CD354836             |
|                       |           BX463642           |          BY102550             |
|                       |           CR611488           |          BM951620             |
|                       |           BX367457           |          BY123737             |
|                       |           CK002423           |          BQ572434             |
|                       |           AW965670           |          CD806187             |
|                       |           AA351087           |          BM950485             |
|                       |           BQ070234           |          BQ179874             |
|                       |           BX449499           |          CA978377             |
|                       |           BX346544           |          CF538058             |
|                       |           AW898404           |          BQ442099             |
|                       |           BX409426           |          BG806762             |
|                       |           AA339704           |          BM950964             |
|                       |                              |          BM951505             |
|                       |                              |          BU613069             |
|                       |                              |          CB290099             |
|                       |                              |          BQ570886             |
|                       |                              |          CB173185             |
|                       |                              |          BM948489             |
|                       |                              |          BU615057             |
|                       |                              |          CD806809             |
|                       |                              |          BQ443051             |
|                       |                              |          BG277322             |
|                       |                              |          BU614855             |
|                       |                              |          CB518778             |
|                       |                              |          CN525767             |
|                       |                              |          BU525275             |
|                       |                              |          CF533767             |
|                       |                              |          CB523293             |
|                       |                              |          CB247288             |
|                       |                              |          CB841822             |
|                       |                              |          AA036534             |
|                       |                              |          BF449247             |
|                       |                              |          BU058241             |
|                       |                              |          AA003408             |
|                       |                              |            W64898             |
|                       |                              |          AA002534             |
|                       |                              |          BB328802             |
|                       |                              |          CB182772             |
|                       |                              |          CO042211             |
|                       |                              |          AK019418             |
|                       |                              |          BY713063             |
|                       |                              |          BQ176645             |
|                       |                              |          AI465285             |
|                       | *Seq. mapped in strand Minus*| *Seq. mapped in strand Minus* |
|                       |           AA454075           |          AI837057             |
|                       |           BX088896           |          CD775433             |
|                       |           AA907042           |                               |
|                       |           AI379824           |                               |
|                       |           AI393153           |                               |
+-----------------------+------------------------------+-------------------------------+
|      #   30           | *Seq. mapped in strand Plus* | *Seq. mapped in strand Plus*  |
|                       |           BG482470           |          BQ174847             |
|                       |           BE901757           |          AV375436             |
|                       |           BU849493           |          BB615316             |
|                       |           BI562476           |                               |
|                       |           BQ687857           |                               |
|                       |           BU150128           |                               |
|                       |           CF995161           |                               |
|                       |           BG419038           |                               |
|                       |           AW732587           |                               |
|                       |           CF137974           |                               |
|                       |           CF137975           |                               |
|                       |           BG394446           |                               |
|                       |           AW503907           |                               |
|                       |             L41067           |                               |
|                       |           AI380644           |                               |
|                       |           BE244436           |                               |
|                       |           BI832317           |                               |
|                       |           CF138064           |                               |
|                       |           CN355955           |                               |
|                       |           BE273734           |                               |
|                       |           BC001050           |                               |
|                       |             U85430           |                               |
|                       |             U14510           |                               |
|                       |             U85428           |                               |
|                       |             U85429           |                               |
|                       |           AA297980           |                               |
|                       |           BQ423593           |                               |
|                       |           BQ070507           |                               |
|                       |           BQ307399           |                               |
|                       |           BQ307520           |                               |
|                       |           BQ307401           |                               |
|                       |           BQ307368           |                               |
|                       |           BQ307397           |                               |
|                       |           BQ307486           |                               |
|                       |           BQ307525           |                               |
|                       |           BQ307403           |                               |
|                       |           AU129466           |                               |
|                       |           AU123215           |                               |
|                       |           AL120778           |                               |
|                       |           AA770124           |                               |
|                       |           BG746287           |                               |
|                       |           AI953073           |                               |
|                       |           BQ054685           |                               |
|                       |           BQ712506           |                               |
|                       |           BE719642           |                               |
|                       |           BG822905           |                               |
|                       |           BE257384           |                               |
|                       |           BE251786           |                               |
|                       |           BF308204           |                               |
|                       |           BF307534           |                               |
|                       |           BE297605           |                               |
|                       |           BU162991           |                               |
|                       |           AW503657           |                               |
|                       |           BU428450           |                               |
|                       |           BU431781           |                               |
|                       |           AU127767           |                               |
|                       |           AW630226           |                               |
|                       |           AW500500           |                               |
|                       |           BQ949102           |                               |
|                       |           BE394319           |                               |
|                       |           BG684246           |                               |
|                       |           AW499757           |                               |
|                       |           BE893770           |                               |
|                       |           BG686912           |                               |
|                       |           BM910159           |                               |
|                       |           BM908365           |                               |
|                       |           BQ068604           |                               |
|                       |           BG472899           |                               |
|                       |           AW362028           |                               |
|                       |           BQ344422           |                               |
|                       |           BF920544           |                               |
|                       |             W24544           |                               |
|                       |           BQ320260           |                               |
|                       |           BF920547           |                               |
|                       |           CA453543           |                               |
|                       |           BQ888242           |                               |
|                       |           AW937781           |                               |
|                       |           AL045568           |                               |
|                       |           BG760091           |                               |
|                       |           BU145430           |                               |
|                       |           BG758541           |                               |
|                       |           BQ070288           |                               |
|                       |           BG754125           |                               |
|                       |           BG334540           |                               |
|                       |           BG281437           |                               |
|                       |           BQ685361           |                               |
|                       |           BQ686294           |                               |
|                       |           BU174435           |                               |
|                       |           BE728324           |                               |
|                       |           BG389781           |                               |
|                       |           BM479989           |                               |
|                       |           BE391992           |                               |
|                       |           BM564507           |                               |
|                       |           BU156335           |                               |
|                       |           BG767285           |                               |
|                       |           BG769511           |                               |
|                       |           BG769273           |                               |
|                       |           BE207242           |                               |
|                       |           BQ653595           |                               |
|                       |           BI252343           |                               |
|                       |           BM006234           |                               |
|                       |           BQ653970           |                               |
|                       |           BQ645444           |                               |
|                       |           BQ644919           |                               |
|                       |           BQ652039           |                               |
|                       |           BQ647947           |                               |
|                       |           BQ652747           |                               |
|                       |           BQ654244           |                               |
|                       |           CR741943           |                               |
|                       |           AA293819           |                               |
|                       |           BU951040           |                               |
|                       |           BI253014           |                               |
|                       |           BE394875           |                               |
|                       |           BQ271367           |                               |
|                       |           CD675617           |                               |
|                       |           BF685217           |                               |
|                       |             R07070           |                               |
|                       |           BQ673139           |                               |
|                       |             H59047           |                               |
|                       |           AI795944           |                               |
|                       |           BM984664           |                               |
|                       |           BE042881           |                               |
|                       |           BM976712           |                               |
|                       |           BM979650           |                               |
|                       |           BU956419           |                               |
|                       |           BU951323           |                               |
|                       |           CN478846           |                               |
|                       |           CA419522           |                               |
|                       |           CA446273           |                               |
|                       |           BX096093           |                               |
|                       |           AA282602           |                               |
|                       |             R51333           |                               |
|                       |           AW593557           |                               |
|                       |           AW025968           |                               |
|                       |           BX117124           |                               |
|                       |           CA489880           |                               |
|                       |           AK074828           |                               |
|                       |           BX378632           |                               |
|                       |           BM193482           |                               |
|                       |           BM153701           |                               |
|                       |           BP201720           |                               |
|                       |           BI668926           |                               |
|                       |           AL110209           |                               |
|                       |           AU280043           |                               |
|                       |           BX419547           |                               |
|                       |           BP215999           |                               |
|                       |           BP272717           |                               |
|                       |           BP306306           |                               |
|                       |           BP272718           |                               |
|                       |           BP304076           |                               |
|                       |           BP295361           |                               |
|                       |           BM837536           |                               |
|                       |           BX339229           |                               |
|                       |           BX342683           |                               |
|                       |           AU098814           |                               |
|                       |           CR598578           |                               |
|                       |           BI517449           |                               |
|                       |           BC062605           |                               |
|                       |           BG832280           |                               |
|                       |           BP361001           |                               |
|                       |           AU133126           |                               |
|                       |           AK001705           |                               |
|                       |           BP306903           |                               |
|                       |           BP224324           |                               |
|                       |           BP223793           |                               |
|                       |           BP366295           |                               |
|                       |           BP353390           |                               |
|                       |           BP353359           |                               |
|                       |           BX381837           |                               |
|                       |           AB017494           |                               |
|                       |           BX401485           |                               |
|                       |           BP305918           |                               |
|                       |           CO247284           |                               |
|                       |           BP356526           |                               |
|                       |           CD251939           |                               |
|                       |           AY358425           |                               |
|                       |           CO249839           |                               |
|                       |           AL524311           |                               |
|                       |           BP237849           |                               |
|                       |           BG829912           |                               |
|                       |           AK127898           |                               |
|                       |           AW732293           |                               |
|                       |           BM770745           |                               |
|                       |           BM771227           |                               |
|                       |           BM771817           |                               |
|                       |           BM770078           |                               |
|                       |           BM771852           |                               |
|                       |           BM771795           |                               |
|                       |           BM771194           |                               |
|                       |           BM771402           |                               |
|                       |           BM770153           |                               |
|                       |           BM770144           |                               |
|                       |           BM752013           |                               |
|                       |           CA454721           |                               |
|                       |           BM751870           |                               |
|                       |           BF310537           |                               |
|                       |           BC011640           |                               |
|                       |           BQ711543           |                               |
|                       |           BX386964           |                               |
|                       |           CV027858           |                               |
|                       |           BP346051           |                               |
|                       |           BP304184           |                               |
|                       |           BP297988           |                               |
|                       |           CF552194           |                               |
|                       |           AL389957           |                               |
|                       |           BG422420           |                               |
|                       |           CR609474           |                               |
|                       |           CR597665           |                               |
|                       |           BU625897           |                               |
|                       |           BQ010130           |                               |
|                       |           CD742834           |                               |
|                       |           CA442359           |                               |
|                       |           CB528782           |                               |
|                       |           BU681799           |                               |
|                       |           CD366449           |                               |
|                       |           CN480744           |                               |
|                       |           CA429119           |                               |
|                       |           BM671627           |                               |
|                       |           CN480826           |                               |
|                       |           CN430063           |                               |
|                       |           BM663443           |                               |
|                       |           BQ013207           |                               |
|                       |           AI247022           |                               |
|                       |           AA035572           |                               |
|                       |           AA825979           |                               |
|                       |           AI868745           |                               |
|                       | *Seq. mapped in strand Minus*| *Seq. mapped in strand Minus* |
|                       |           AA857871           |          CO040721             |
|                       |           BX645678           |          BC034194             |
|                       |           AA247855           |          AK076416             |
+-----------------------+------------------------------+-------------------------------+
|      #   31           | *Seq. mapped in strand Plus* | *Seq. mapped in strand Plus*  |
|                       |           BC002660           |          BY283312             |
|                       |             M77016           |          BY251151             |
|                       |           AK095748           |          BY127972             |
|                       |           AK096156           |          AU079921             |
|                       |                              |          BY125041             |
|                       |                              |          BB864536             |
|                       |                              |          BY239314             |
|                       |                              |          BY774902             |
|                       |                              |          BY767647             |
|                       |                              |          BY126516             |
|                       |                              |          BY784907             |
|                       |                              |          BY784932             |
|                       |                              |          BY260191             |
|                       |                              |          BY294092             |
|                       |                              |          BY091921             |
|                       |                              |          BY297128             |
|                       |                              |          BY074322             |
|                       |                              |          BY102643             |
|                       |                              |          BY103667             |
|                       |                              |          BY072642             |
|                       |                              |          BY064746             |
|                       |                              |          BY083898             |
|                       |                              |          BY092356             |
|                       |                              |          BY296190             |
|                       |                              |          BB855911             |
|                       |                              |          BY792326             |
|                       |                              |          BY794271             |
|                       |                              |          BY767321             |
|                       |                              |          BY256441             |
|                       |                              |          BY071327             |
|                       |                              |          AI787629             |
|                       |                              |          AI788092             |
|                       |                              |          AI006541             |
|                       |                              |          AA673831             |
|                       |                              |          CD806869             |
|                       |                              |          CN837926             |
|                       |                              |          BB615338             |
|                       |                              |          BY773017             |
|                       |                              |          BY098760             |
|                       |                              |          BY100388             |
|                       |                              |          AA791118             |
|                       |                              |          AA031252             |
|                       |                              |          AI322547             |
|                       |                              |          AA793976             |
|                       |                              |          BM936399             |
|                       |                              |          BB014362             |
|                       |                              |            W54078             |
|                       |                              |            W61419             |
|                       |                              |          BY078627             |
|                       |                              |          BY089062             |
|                       |                              |          AA682111             |
|                       |                              |          AI324231             |
|                       |                              |          AI413158             |
|                       |                              |          AW061277             |
|                       | *Seq. mapped in strand Minus*| *Seq. mapped in strand Minus* |
|                       |           AF131836           |          BC057893             |
|                       |           BC022958           |          BE996665             |
|                       |           AK094043           |          AW488635             |
|                       |           BX538308           |          AK028258             |
|                       |           AK056752           |          AA517224             |
|                       |           AK056453           |          CF950265             |
|                       |           BX648493           |          CA871434             |
|                       |           BC035604           |          AI613715             |
|                       |           AK128615           |          CA878400             |
|                       |           AK122809           |          CN698104             |
|                       |           AF258575           |          CB527010             |
|                       |                              |          BY149539             |
|                       |                              |          CD549731             |
|                       |                              |          CB840211             |
|                       |                              |          CF893140             |
|                       |                              |          CF737301             |
|                       |                              |          BU610792             |
|                       |                              |          BE332897             |
|                       |                              |          BG975187             |
|                       |                              |          BY732641             |
|                       |                              |          CD553582             |
|                       |                              |          BB610035             |
|                       |                              |          CN692393             |
|                       |                              |          CN704745             |
|                       |                              |          CF171912             |
|                       |                              |          CN664404             |
|                       |                              |          CD553564             |
|                       |                              |          BE457697             |
|                       |                              |          CN694307             |
|                       |                              |          CA569428             |
|                       |                              |          BY276526             |
|                       |                              |          BY324890             |
|                       |                              |          BE953713             |
|                       |                              |          BB869245             |
|                       |                              |          BY065575             |
|                       |                              |          BY191090             |
|                       |                              |          BY188415             |
|                       |                              |          BY773297             |
|                       |                              |          BY068521             |
|                       |                              |          BY013797             |
|                       |                              |          BY782074             |
|                       |                              |          BY782059             |
|                       |                              |          BY333003             |
|                       |                              |          CB245616             |
|                       |                              |          BY028893             |
|                       |                              |          BY793493             |
|                       |                              |                               |
|                       |                              |                               |
|                       |                              |                               |
|                       |                              |                               |
|                       |                              |                               |
|                       |                              |                               |
|                       |                              |                               |
|                       |                              |                               |
+-----------------------+------------------------------+-------------------------------+
|      #   32           | *Seq. mapped in strand Plus* | *Seq. mapped in strand Plus*  |
|                       |           AK055656           |          AK036046             |
|                       | *Seq. mapped in strand Minus*| *Seq. mapped in strand Minus* |
|                       |           AK127130           |          BE948094             |
+-----------------------+------------------------------+-------------------------------+
|      #   33           | *Seq. mapped in strand Plus* | *Seq. mapped in strand Plus*  |
|                       |           AK123796           |          BU938133             |
|                       |           BC032064           |          BY716362             |
|                       |           BC041612           |          AK016673             |
|                       |           BC064498           |          AI614158             |
|                       |           BC082249           |                               |
|                       | *Seq. mapped in strand Minus*| *Seq. mapped in strand Minus* |
|                       |           BC008284           |          BB688456             |
|                       |           AY358834           |          BB690952             |
|                       |           AL136786           |          BB683054             |
|                       |           CR614373           |          BY682726             |
+-----------------------+------------------------------+-------------------------------+
|      #   34           | *Seq. mapped in strand Plus* | *Seq. mapped in strand Plus*  |
|                       |             D45371           |          AK041214             |
|                       |                              |          AK003138             |
|                       |                              |          BC028770             |
|                       |                              |            U49915             |
|                       |                              |            U37222             |
|                       | *Seq. mapped in strand Minus*| *Seq. mapped in strand Minus* |
|                       |           BC036509           |          AK018896             |
+-----------------------+------------------------------+-------------------------------+
|      #   35           | *Seq. mapped in strand Plus* | *Seq. mapped in strand Plus*  |
|                       |           CA311186           |          AW456925             |
|                       |           BU608532           |                               |
|                       |           BE878429           |                               |
|                       |           BP203594           |                               |
|                       |           BG563928           |                               |
|                       |           BG564610           |                               |
|                       |           BG287815           |                               |
|                       |           AW969334           |                               |
|                       |           BG111573           |                               |
|                       |           BI860831           |                               |
|                       |           BP288815           |                               |
|                       |           BG283394           |                               |
|                       |           AI580006           |                               |
|                       |           BP305309           |                               |
|                       | *Seq. mapped in strand Minus*| *Seq. mapped in strand Minus* |
|                       |           AW874140           |          AK012185             |
|                       |           AA657712           |          AI035290             |
|                       |           AA156441           |          AK013243             |
|                       |           AI753230           |          BC003881             |
|                       |           AA828059           |          CA555152             |
|                       |           AI436234           |          CB571080             |
|                       |           AW192143           |          CA550355             |
|                       |           AI872218           |          CA548351             |
|                       |           AI191607           |          CK022686             |
|                       |           AI796295           |          AW913231             |
|                       |           AA043292           |          CF907533             |
|                       |           AI368841           |          CK791572             |
|                       |           AA890501           |          AK018623             |
|                       |           CB854348           |          BB015226             |
|                       |           AA167328           |          AW105899             |
|                       |           BM876318           |          BF120186             |
|                       |           BQ025107           |          CA555112             |
|                       |           BU686503           |          CA567489             |
|                       |           CA312393           |          BI219324             |
|                       |           CA446243           |          BB609909             |
|                       |           BU609163           |          BY711371             |
|                       |           BC018447           |          BU694808             |
|                       |           AF130104           |          BF139539             |
|                       |           AK027632           |          BI731783             |
|                       |           BC060842           |          BG919669             |
|                       |           AV738264           |          BG862402             |
|                       |           BC016342           |          CN683177             |
|                       |           CR599925           |          AI226759             |
|                       |           AA171802           |          BG973026             |
|                       |           AI925653           |          AW106484             |
|                       |           BU726173           |          CA329013             |
|                       |           BM857668           |          CK792618             |
|                       |           BC041014           |          BI655150             |
|                       |           AY358691           |          BY720979             |
|                       |           AK075394           |          BG142551             |
|                       |           AL136636           |          AW611091             |
|                       |           AA377503           |          AI789324             |
|                       |           AI498655           |          CF896483             |
|                       |           AV707825           |          CA876748             |
|                       |           BP288791           |          AI746805             |
|                       |           BM876579           |          AI746668             |
|                       |           BF241086           |          AI036676             |
|                       |           BP203967           |          CB235381             |
|                       |           AA046836           |          AI037135             |
|                       |           BF694224           |          BX526188             |
|                       |           AA188308           |          AI036684             |
|                       |           BG654457           |          AW226893             |
|                       |           BU072446           |          CA896023             |
|                       |           BQ416644           |          CF896523             |
|                       |           BF206746           |          AI931457             |
|                       |           BP241502           |          BY016926             |
|                       |           CK818013           |          BE911913             |
|                       |           BQ416947           |          BY244023             |
|                       |           CN262234           |          BE286372             |
|                       |           BP344320           |          CD563203             |
|                       |           BP266953           |          BY211265             |
|                       |           BP266944           |          CA885183             |
|                       |           BP266633           |          CD543179             |
|                       |           BP327768           |          AI448366             |
|                       |           BG655076           |          CD542016             |
|                       |           AW238561           |          CD564006             |
|                       |           CN262228           |          BY062443             |
|                       |           AA936736           |          BB860042             |
|                       |           BE747812           |          BY007817             |
|                       |           AA297716           |          BY022543             |
|                       |           CN262227           |          AW259373             |
|                       |           BG481300           |          BY099635             |
|                       |           AA297717           |          BY011100             |
|                       |           BI462070           |          BY303316             |
|                       |           CB997368           |          BY243268             |
|                       |           BU929293           |          BY237084             |
|                       |           CB993442           |          BY154266             |
|                       |           CB988754           |          BY787633             |
|                       |           BG563100           |          BY132203             |
|                       |           BF031678           |          BY306562             |
|                       |           BG618794           |          BY052782             |
|                       |           CB997136           |          BY064142             |
|                       |           BE788481           |          BY302493             |
|                       |           BU072738           |          BY014788             |
|                       |           CB996144           |          BY015150             |
|                       |           CB129391           |          BY321189             |
|                       |           BM763184           |          BY138432             |
|                       |           CB987676           |          BY300999             |
|                       |           BG485304           |          BY145941             |
|                       |           BU571073           |          BY324521             |
|                       |           BX499058           |          BY325250             |
|                       |           BG473625           |          BY057367             |
|                       |           BQ477289           |          BY335814             |
|                       |           BF694342           |          BY019881             |
|                       |           BU951590           |          BY013436             |
|                       |           BX471448           |          BY199089             |
|                       |           CB995741           |          BY323541             |
|                       |           BG431236           |          BY142415             |
|                       |           BF676542           |          BY326148             |
|                       |           AW499954           |          BY069057             |
|                       |           BU663946           |          BY237455             |
|                       |           BU658193           |          AW213620             |
|                       |           CN262230           |          BY012935             |
|                       |           BG426701           |          BY329338             |
|                       |           BF031888           |          BY778158             |
|                       |           CD703222           |          CA885091             |
|                       |           BQ432752           |          BY300308             |
|                       |           BI868494           |          BY144855             |
|                       |           AL601695           |          BY778774             |
|                       |           BP244020           |          BY054455             |
|                       |           BX482932           |          BY793377             |
|                       |           BP313340           |          BB666911             |
|                       |           BP225090           |                               |
|                       |           BX472267           |                               |
|                       |           BP274473           |                               |
|                       |           BP290189           |                               |
|                       |           BP328167           |                               |
|                       |           BP276554           |                               |
|                       |           BP315565           |                               |
|                       |           BX475055           |                               |
|                       |           BP313992           |                               |
|                       |           BP241055           |                               |
|                       |           BF541209           |                               |
|                       |           BF680271           |                               |
|                       |           CF596640           |                               |
|                       |           BP288868           |                               |
|                       |           AU280691           |                               |
|                       |           BP325834           |                               |
|                       |           BP244088           |                               |
|                       |           BG400107           |                               |
|                       |           BX956360           |                               |
|                       |           AW732317           |                               |
|                       |           AL693492           |                               |
|                       |           BF382863           |                               |
|                       |           BX475092           |                               |
|                       |           BX477152           |                               |
|                       |           AL697895           |                               |
|                       |           CD689276           |                               |
|                       |           BP257920           |                               |
|                       |           BP327783           |                               |
|                       |           BX474051           |                               |
|                       |           BX503066           |                               |
|                       |           BC050657           |                               |
|                       |           BC063037           |                               |
|                       |           BF792317           |                               |
|                       |           BF792121           |                               |
|                       |           BX641873           |                               |
|                       |           AA206940           |                               |
|                       |           BG941682           |                               |
|                       |             N87112           |                               |
|                       |           BU659730           |                               |
|                       |           CN262229           |                               |
|                       |           CD700435           |                               |
|                       |           CD698438           |                               |
|                       |           CN262231           |                               |
|                       |           AA186993           |                               |
|                       |           BG896464           |                               |
|                       |           BM839571           |                               |
|                       |           BG531067           |                               |
|                       |           CD709561           |                               |
|                       |           AA094982           |                               |
+-----------------------+------------------------------+-------------------------------+
|      #   36           | *Seq. mapped in strand Plus* | *Seq. mapped in strand Plus*  |
|                       |           BI460583           |          AW048852             |
|                       |           AI126112           |          AK078990             |
|                       |           CN428487           |          AI849121             |
|                       |           BM762405           |          AI429620             |
|                       |           AK124139           |          BG798057             |
|                       |           BI559573           |          BQ084777             |
|                       |           AK092696           |          BQ268546             |
|                       |           CN428484           |          BQ085085             |
|                       |           CN428488           |          BQ416429             |
|                       |           BP243167           |          BU757538             |
|                       |           BU167627           |          BQ268538             |
|                       |           BX283688           |          BF462214             |
|                       |           BM928459           |                               |
|                       |           BQ650203           |                               |
|                       |           CN428489           |                               |
|                       |           BU428742           |                               |
|                       |           CN428482           |                               |
|                       |           BU507483           |                               |
|                       |           BG765896           |                               |
|                       |           CN428483           |                               |
|                       |           CN428486           |                               |
|                       |           BE885013           |                               |
|                       |           BQ940963           |                               |
|                       |           BM469804           |                               |
|                       |           BM542651           |                               |
|                       |           BE792545           |                               |
|                       |           BI837244           |                               |
|                       |           CN428490           |                               |
|                       |           AL596772           |                               |
|                       |           BQ417027           |                               |
|                       |           BU950669           |                               |
|                       |           BM273296           |                               |
|                       |           AW385770           |                               |
|                       |           BG033340           |                               |
|                       |           CN428491           |                               |
|                       |           CN428485           |                               |
|                       |           AA025476           |                               |
|                       |             W81042           |                               |
|                       |           CF138106           |                               |
|                       |           AL705144           |                               |
|                       |           AI625008           |                               |
|                       |           BQ416721           |                               |
|                       |           AI913667           |                               |
|                       |           BU950367           |                               |
|                       |           BF588723           |                               |
|                       |           AI654706           |                               |
|                       |           CF138122           |                               |
|                       |           AK096189           |                               |
|                       |           BE671140           |                               |
|                       |           AI697491           |                               |
|                       |           BF591654           |                               |
|                       |           AW470513           |                               |
|                       |           AW135136           |                               |
|                       |           AK093029           |                               |
|                       |           AW392596           |                               |
|                       |           AW964967           |                               |
|                       |           AI161322           |                               |
|                       |           BM719442           |                               |
|                       |           AW205155           |                               |
|                       |           AA341635           |                               |
|                       |           BE672255           |                               |
|                       |           AI380903           |                               |
|                       |           AA194051           |                               |
|                       |           AA194209           |                               |
|                       |           AK025656           |                               |
|                       |           BC011830           |                               |
|                       |           BC001413           |                               |
|                       |           BC007880           |                               |
|                       |           CA443432           |                               |
|                       |           BQ182649           |                               |
|                       |           BU675052           |                               |
|                       |           BM679123           |                               |
|                       |           CA311334           |                               |
|                       |           CA428855           |                               |
|                       |           BQ015800           |                               |
|                       |           AI092889           |                               |
|                       |           BM669763           |                               |
|                       |           AW197137           |                               |
|                       |           BU072396           |                               |
|                       |           AI373812           |                               |
|                       |           AI379435           |                               |
|                       |           AW190745           |                               |
|                       |           CN295251           |                               |
|                       |           AI278070           |                               |
|                       |           AA262472           |                               |
|                       |           AA737070           |                               |
|                       |           AA844052           |                               |
|                       |           AW615343           |                               |
|                       |             T65152           |                               |
|                       |           AI027104           |                               |
|                       |           BU069238           |                               |
|                       |             R37372           |                               |
|                       |           AV740292           |                               |
|                       |           AA804442           |                               |
|                       |           AA854521           |                               |
|                       |           AA805529           |                               |
|                       | *Seq. mapped in strand Minus*| *Seq. mapped in strand Minus* |
|                       |           AA460782           |          BF467066             |
|                       |           BM676613           |          BE985222             |
|                       |           BU739037           |                               |
|                       |           BU676147           |                               |
|                       |           AA827825           |                               |
|                       |           BF062144           |                               |
|                       |           AW136607           |                               |
|                       |           AI889740           |                               |
|                       |           AI889750           |                               |
|                       |           BM979004           |                               |
|                       |           BX648129           |                               |
|                       |           AL832489           |                               |
|                       |           AI743245           |                               |
|                       |           BQ005729           |                               |
|                       |           CA306283           |                               |
|                       |           AW135080           |                               |
|                       |           AA766258           |                               |
|                       |           BC020592           |                               |
|                       |           AK095998           |                               |
|                       |           AB075850           |                               |
|                       |           CA438332           |                               |
|                       |           AI924769           |                               |
|                       |           BU632616           |                               |
|                       |           AI942309           |                               |
|                       |           AI287631           |                               |
|                       |           AW044167           |                               |
|                       |           AI376887           |                               |
|                       |           AW070443           |                               |
|                       |           BU618832           |                               |
|                       |           AA190906           |                               |
|                       |           CA422766           |                               |
|                       |           CA427430           |                               |
|                       |           AF088053           |                               |
|                       |           AW188110           |                               |
|                       |           BU191866           |                               |
|                       |           BU687088           |                               |
|                       |           CR613598           |                               |
|                       |           AW965080           |                               |
|                       |           BG399583           |                               |
|                       |           BX350427           |                               |
|                       |           BG177671           |                               |
|                       |           BG335246           |                               |
|                       |           BX369643           |                               |
|                       |           CD639353           |                               |
|                       |           BX369642           |                               |
|                       |           BM046302           |                               |
|                       |           AW248374           |                               |
|                       |           BM049267           |                               |
|                       |           BC040013           |                               |
|                       |           BQ651425           |                               |
|                       |           BE897809           |                               |
|                       |           BG024673           |                               |
|                       |           CF272645           |                               |
|                       |           BE794758           |                               |
|                       |           BQ069720           |                               |
|                       |           BQ649682           |                               |
|                       |           BG818514           |                               |
|                       |           BG337213           |                               |
|                       |           BM721662           |                               |
|                       |           BG036429           |                               |
|                       |           AL520724           |                               |
|                       |           BX419646           |                               |
|                       |           BX400815           |                               |
|                       |           BX376241           |                               |
|                       |           BX383622           |                               |
|                       |           BQ889878           |                               |
|                       |           BX341354           |                               |
|                       |           BQ067087           |                               |
|                       |           BQ070700           |                               |
|                       |           BG913918           |                               |
|                       |           BM548249           |                               |
|                       |           AL559488           |                               |
|                       |           BI490029           |                               |
|                       |           BQ689430           |                               |
|                       |           BU844630           |                               |
|                       |           BQ069992           |                               |
|                       |           BF982439           |                               |
|                       |           CN334379           |                               |
|                       |           BE778357           |                               |
|                       |           BE879536           |                               |
|                       |           BE612832           |                               |
|                       |           BQ644209           |                               |
|                       |             Z24927           |                               |
|                       |             F00197           |                               |
|                       |           BE894554           |                               |
|                       |           BU177351           |                               |
|                       |           BQ651814           |                               |
|                       |           CN334378           |                               |
|                       |           BG478735           |                               |
|                       |           CD675477           |                               |
|                       |           CB156316           |                               |
|                       |           BG333924           |                               |
|                       |           AI929390           |                               |
|                       |           CN334377           |                               |
|                       |           CO247631           |                               |
|                       |           BE409414           |                               |
|                       |           BE242636           |                               |
|                       |           AA491290           |                               |
|                       |             Z44584           |                               |
+-----------------------+------------------------------+-------------------------------+
|      #   37           | *Seq. mapped in strand Plus* | *Seq. mapped in strand Plus*  |
|                       |           CA311625           |          AK051213             |
|                       |           BP309052           |          AK090029             |
|                       |           BP195272           |          BC003830             |
|                       |           BP198158           |          BC011493             |
|                       |           BP209119           |            X13171             |
|                       |           BG747688           |          AK077473             |
|                       |           BM750839           |            X72894             |
|                       |           BP307970           |                               |
|                       |           BP308488           |                               |
|                       |           BP368605           |                               |
|                       |           AU099328           |                               |
|                       |           BP199433           |                               |
|                       |           BP349133           |                               |
|                       |           CB137500           |                               |
|                       |           BP255625           |                               |
|                       |           BP322849           |                               |
|                       |           BP347583           |                               |
|                       |           AK123190           |                               |
|                       |           BP324122           |                               |
|                       |           BP330572           |                               |
|                       |           BG762288           |                               |
|                       |           BC014457           |                               |
|                       |           BQ680679           |                               |
|                       |           BQ677439           |                               |
|                       |           BU145158           |                               |
|                       |           BU157004           |                               |
|                       |           BQ882470           |                               |
|                       |           BG720482           |                               |
|                       |           BU190954           |                               |
|                       |           BQ676580           |                               |
|                       |           BI551010           |                               |
|                       |           BQ897192           |                               |
|                       |           BI552272           |                               |
|                       |           BI550225           |                               |
|                       |           BU597551           |                               |
|                       |           CB110041           |                               |
|                       |           CN347397           |                               |
|                       |           BG717895           |                               |
|                       |           AF077740           |                               |
|                       |           BP370730           |                               |
|                       |           BP369347           |                               |
|                       |           CV028958           |                               |
|                       |           BU526482           |                               |
|                       |           CN347400           |                               |
|                       |           AA252373           |                               |
|                       |           AI935359           |                               |
|                       |           AW001123           |                               |
|                       |           AI814342           |                               |
|                       |           AI924201           |                               |
|                       |           BX325548           |                               |
|                       |           BI763768           |                               |
|                       |           AA888820           |                               |
|                       |           AI198343           |                               |
|                       |           AA665651           |                               |
|                       |           AA670436           |                               |
|                       |           BU950262           |                               |
|                       |           CA776921           |                               |
|                       |           BG119877           |                               |
|                       |           CN347396           |                               |
|                       |           CA778095           |                               |
|                       |           CA778022           |                               |
|                       |           AA766826           |                               |
|                       |           BG928003           |                               |
|                       |           BG752866           |                               |
|                       |           BI198223           |                               |
|                       |           BX325788           |                               |
|                       |           BP328473           |                               |
|                       |           BI251895           |                               |
|                       |           CN347398           |                               |
|                       |             W21033           |                               |
|                       |           BF973814           |                               |
|                       |           BX388274           |                               |
|                       |           BM769327           |                               |
|                       |           BQ082775           |                               |
|                       |           BM819092           |                               |
|                       |           BM823613           |                               |
|                       |           BM769328           |                               |
|                       |           CN347399           |                               |
|                       |           BI857953           |                               |
|                       |           BX331419           |                               |
|                       |           BM016700           |                               |
|                       |           BE779966           |                               |
|                       |           BX331420           |                               |
|                       |           BI252535           |                               |
|                       |           BM018723           |                               |
|                       |           AI970413           |                               |
|                       |           AI492094           |                               |
|                       |           BM715489           |                               |
|                       |           BX101962           |                               |
|                       |           BE881560           |                               |
|                       |           BQ771646           |                               |
|                       |           BU625865           |                               |
|                       |           BU689454           |                               |
|                       |           AI468008           |                               |
|                       |           BX400397           |                               |
|                       |           CR603831           |                               |
|                       |           AW772362           |                               |
|                       |           BF001480           |                               |
|                       |           AI521923           |                               |
|                       |           CA777204           |                               |
|                       |           BU950554           |                               |
|                       |           AI028459           |                               |
|                       |             R56959           |                               |
|                       |           AI040406           |                               |
|                       |           CN484965           |                               |
|                       |           BE465816           |                               |
|                       |           AA877725           |                               |
|                       |           AI333579           |                               |
|                       |           AA252428           |                               |
|                       |           AA703357           |                               |
|                       |           AI682438           |                               |
|                       |           BQ941420           |                               |
|                       |           BU739455           |                               |
|                       |           AI656960           |                               |
|                       |           AI624888           |                               |
|                       |           AI964003           |                               |
|                       |           AW972947           |                               |
|                       |           AI972753           |                               |
|                       |           CA778315           |                               |
|                       |           AI669759           |                               |
|                       |           AI804669           |                               |
|                       |           AW196667           |                               |
|                       |           AI638764           |                               |
|                       |           BF062892           |                               |
|                       |           AA541628           |                               |
|                       |           AI076149           |                               |
|                       |           AI610987           |                               |
|                       |           AI832204           |                               |
|                       |           BG337843           |                               |
|                       |             N90234           |                               |
|                       |           BG993672           |                               |
|                       |           BG997105           |                               |
|                       |           BG997091           |                               |
|                       |           AI424567           |                               |
|                       |           AV743057           |                               |
|                       |           AK091372           |                               |
|                       |           CR624468           |                               |
|                       |           CR610156           |                               |
|                       |           CR623479           |                               |
|                       |           CR623269           |                               |
|                       |           CR598601           |                               |
|                       |           CR609515           |                               |
|                       |           CR604812           |                               |
|                       |           CR613392           |                               |
|                       |           CR591666           |                               |
|                       |           CR617321           |                               |
|                       |           CR619372           |                               |
|                       |           CR606842           |                               |
|                       |           CR615993           |                               |
|                       |           CR601737           |                               |
|                       |           CR615517           |                               |
|                       |           CR591712           |                               |
|                       |           CR598888           |                               |
|                       |           BC000145           |                               |
|                       |           BC029046           |                               |
|                       |           CR596843           |                               |
|                       |           CR604452           |                               |
|                       |           CR606708           |                               |
|                       |           CR620114           |                               |
|                       |           BU617870           |                               |
|                       |           CA425534           |                               |
|                       |           CR456502           |                               |
|                       |             N54406           |                               |
|                       |           CR597945           |                               |
|                       |           CR542220           |                               |
|                       |           CD364437           |                               |
|                       |           CR594003           |                               |
|                       |           AW295162           |                               |
|                       |           CR623443           |                               |
|                       |           CR606855           |                               |
|                       |           CA431097           |                               |
|                       |           BU689052           |                               |
|                       |           BU629851           |                               |
|                       |           BM679144           |                               |
|                       |           CB528909           |                               |
|                       |           BU626125           |                               |
|                       |           BU689338           |                               |
|                       |           BU624947           |                               |
|                       |           CA314972           |                               |
|                       |           BQ574277           |                               |
|                       |           BU633577           |                               |
|                       |           BU633911           |                               |
|                       |           CA424788           |                               |
|                       |           BM993392           |                               |
|                       |           CA442298           |                               |
|                       |           BM973459           |                               |
|                       |           BU677865           |                               |
|                       |           AW453068           |                               |
|                       |           AI660975           |                               |
|                       |           BM971984           |                               |
|                       |           AI184996           |                               |
|                       |           BM679228           |                               |
|                       |           BU679833           |                               |
|                       |           BM674414           |                               |
|                       |           BE139576           |                               |
|                       |           AI500234           |                               |
|                       |           AW449016           |                               |
|                       |           AA171412           |                               |
|                       |             W15291           |                               |
|                       |           AA477273           |                               |
|                       |           AI568595           |                               |
|                       | *Seq. mapped in strand Minus*| *Seq. mapped in strand Minus* |
|                       |           AI133666           |          AK052663             |
|                       |           BQ021610           |                               |
|                       |             H65259           |                               |
|                       |           AA045489           |                               |
|                       |           AA593288           |                               |
|                       |           AI300359           |                               |
|                       |           AA327095           |                               |
+-----------------------+------------------------------+-------------------------------+
|      #   38           | *Seq. mapped in strand Plus* | *Seq. mapped in strand Plus*  |
|                       |           AK095760           |          AK044440             |
|                       |           CN353838           |          BC031541             |
|                       |           BC044636           |          AK087500             |
|                       |           BF799050           |          AB033168             |
|                       |           BE939253           |          CF182463             |
|                       |           AK090435           |          CF532137             |
|                       |           AK091050           |          BQ885616             |
|                       |           AL046823           |          BU708248             |
|                       |           AW381641           |          CN534460             |
|                       |           CN334771           |          AI390443             |
|                       |           BF987132           |          AA217835             |
|                       |           BG432222           |          BE368836             |
|                       |             L40400           |          CB558023             |
|                       |           BM472446           |          BF149440             |
|                       |           BU184444           |          CF744209             |
|                       |           CD652930           |          BU147813             |
|                       |           BQ961416           |          BM195566             |
|                       |           CD651883           |          AL364981             |
|                       |           CD691340           |          CF742131             |
|                       |           AU142662           |          AW125464             |
|                       |           AU122434           |          BE951025             |
|                       |           BF313104           |          BE955842             |
|                       |           BI049567           |          BM934766             |
|                       |           BI049566           |          BE980039             |
|                       |           BI051257           |          CB057698             |
|                       |           BI051289           |          AA060255             |
|                       |           BF954989           |          CF893440             |
|                       |           BF948913           |          CF893494             |
|                       |           BF962641           |          BE334400             |
|                       |           AW274870           |          AA822975             |
|                       |           BG014436           |          CF746719             |
|                       |           CB143149           |          CF747026             |
|                       |           BX488275           |          AW553212             |
|                       |           BF243471           |          BF731120             |
|                       |           BG258569           |          BE368845             |
|                       |           BE876618           |          BM507923             |
|                       |             L40403           |          BM508291             |
|                       |           BE567087           |          BF452025             |
|                       |           BE389234           |          BY720210             |
|                       |           BE277518           |          CF740936             |
|                       |           BE390876           |          CD770318             |
|                       |           BX282016           |          BE991793             |
|                       |           BI524021           |          BM936127             |
|                       |           BM699252           |          AV261870             |
|                       |           AI681056           |          BB613027             |
|                       |           BG248095           |          AK028731             |
|                       |           CA442200           |          BU520729             |
|                       |           BE835643           |          CF579586             |
|                       |           BE068268           |          BI466527             |
|                       |           BF432441           |          BB283637             |
|                       |           AW862106           |          BM947342             |
|                       |           AW859249           |          BB284610             |
|                       |           AW859102           |          CF951046             |
|                       |           AW859240           |          CA980613             |
|                       |           AW859248           |          BB836439             |
|                       |           BM669467           |          CB849690             |
|                       |           AW859174           |          BE862787             |
|                       |           BE158946           |          CA328677             |
|                       |           AW859176           |          AA591935             |
|                       |           BI257696           |          CD559962             |
|                       |           BE271700           |          AA270118             |
|                       |           AL710386           |          CA750448             |
|                       |           BQ023001           |          AA097516             |
|                       |           CF144302           |          AA125527             |
|                       |           CF144384           |          AA109545             |
|                       |           AW590187           |          CB526505             |
|                       |           AU126079           |          AA144202             |
|                       |           AI337107           |          BQ043732             |
|                       |           AI968354           |          CB182430             |
|                       |           AI653024           |          BE628338             |
|                       |           BG423237           |            W08889             |
|                       |           BI762910           |          AA760361             |
|                       |           AW885476           |          BG916955             |
|                       |           BU928317           |          BC055465             |
|                       |           AI637902           |          AI183251             |
|                       |             W26622           |          AA105380             |
|                       |           BC007792           |          BI466302             |
|                       |           AI352606           |          BG065229             |
|                       |           BF125437           |          AW537032             |
|                       |           CF140855           |          CA877154             |
|                       |           BE696335           |          BF464857             |
|                       |           BF953167           |          CB203608             |
|                       |           AL601230           |          AW547630             |
|                       |           AA248801           |          CD538338             |
|                       |           BG500635           |          BF227502             |
|                       |           AA090742           |          BQ032161             |
|                       |           BF953255           |          BF227495             |
|                       |           CB133225           |          BE331796             |
|                       |           BU676463           |          BM938264             |
|                       |           AU159925           |          BB810733             |
|                       |           AW949632           |          BF463466             |
|                       |           AA355127           |          BE990395             |
|                       |           AI147227           |                               |
|                       |           AA633087           |                               |
|                       |           CA503149           |                               |
|                       |           BI829562           |                               |
|                       |           BM796571           |                               |
|                       |           CB116754           |                               |
|                       |           BE795132           |                               |
|                       |           BG289918           |                               |
|                       |           AW377966           |                               |
|                       |           BF349715           |                               |
|                       |           AA182445           |                               |
|                       |           BC023570           |                               |
|                       |           AL043233           |                               |
|                       |           CF145942           |                               |
|                       |           BE746216           |                               |
|                       |           BE743026           |                               |
|                       |           AA464106           |                               |
|                       |           BF573676           |                               |
|                       |           AW602513           |                               |
|                       |           AW602525           |                               |
|                       |           AW602516           |                               |
|                       |           AW602527           |                               |
|                       |           BG675850           |                               |
|                       |           AW602511           |                               |
|                       |           BG164559           |                               |
|                       |           BU430228           |                               |
|                       |           CB153087           |                               |
|                       |             R58811           |                               |
|                       |           BG431377           |                               |
|                       |           BE176466           |                               |
|                       |           BM792594           |                               |
|                       |           AA292607           |                               |
|                       |           AA420866           |                               |
|                       |           AA420467           |                               |
|                       |           AA191147           |                               |
|                       |           BF753624           |                               |
|                       |           BQ008617           |                               |
|                       |           BE176456           |                               |
|                       |           AV713690           |                               |
|                       |           BM982466           |                               |
|                       |             T19925           |                               |
|                       |           BM977559           |                               |
|                       |           BM970162           |                               |
|                       |           BM973535           |                               |
|                       |           BQ025434           |                               |
|                       |           BM987348           |                               |
|                       |           BU682281           |                               |
|                       |           CA436367           |                               |
|                       |             T30829           |                               |
|                       |           AW960839           |                               |
|                       |           CV361421           |                               |
|                       |             L40396           |                               |
|                       |           AA779008           |                               |
|                       |           CA441112           |                               |
|                       |           BQ446857           |                               |
|                       |           CB067382           |                               |
|                       |           CB067306           |                               |
|                       |           CB067681           |                               |
|                       |           CA414230           |                               |
|                       |           BF000435           |                               |
|                       |           CK902938           |                               |
|                       |           AI952986           |                               |
|                       |           AW510667           |                               |
|                       |           BE830103           |                               |
|                       |           AA190600           |                               |
|                       |           AU148014           |                               |
|                       |           BE048052           |                               |
|                       |           BU154092           |                               |
|                       |           AA418666           |                               |
|                       |           AA363880           |                               |
|                       |           AI651807           |                               |
|                       |           AI763020           |                               |
|                       |           BE245657           |                               |
|                       |           AW594307           |                               |
|                       |           AI082626           |                               |
|                       |           AA608540           |                               |
|                       |           BQ005188           |                               |
|                       |           CA444442           |                               |
|                       |           AI085521           |                               |
|                       |             R17373           |                               |
|                       |           AI275882           |                               |
|                       |           AI333208           |                               |
|                       |           AI204210           |                               |
|                       |           BM716250           |                               |
|                       |           BQ010483           |                               |
|                       |           AI970186           |                               |
|                       |           AA804592           |                               |
|                       |           AI167440           |                               |
|                       |           AA181552           |                               |
|                       |           AI948685           |                               |
|                       |           AI262924           |                               |
|                       |           AI479962           |                               |
|                       |           BQ018818           |                               |
|                       |           AA862386           |                               |
|                       |             H23405           |                               |
|                       |           AA536065           |                               |
|                       |           AI669788           |                               |
|                       |           AI458061           |                               |
|                       |           BE045971           |                               |
|                       |           AI572386           |                               |
|                       |           BQ017488           |                               |
|                       |           BM998250           |                               |
|                       |           BX102033           |                               |
|                       |           AL691708           |                               |
|                       |           BX090800           |                               |
|                       |           BG057803           |                               |
|                       |           AL691709           |                               |
|                       | *Seq. mapped in strand Minus*| *Seq. mapped in strand Minus* |
|                       |           CA439542           |          AI854589             |
|                       |           AW291256           |          BE991825             |
|                       |           AK094068           |                               |
+-----------------------+------------------------------+-------------------------------+
|      #   39           | *Seq. mapped in strand Plus* | *Seq. mapped in strand Plus*  |
|                       |           BG821869           |          AA645869             |
|                       |           BI524655           |          BY664040             |
|                       |           BE314416           |          BQ175745             |
|                       |           BM550848           |          BM118187             |
|                       |           BM916877           |          BB697384             |
|                       |           BP231480           |          BB695730             |
|                       |           BG474256           |          BB836841             |
|                       |           BG395125           |          BY387251             |
|                       |           BM915551           |          BB773792             |
|                       |           BQ644503           |          BF461965             |
|                       |           BM850097           |          AW125041             |
|                       |           BG499446           |          BE954279             |
|                       |           BG478013           |          AI842489             |
|                       |           BI252523           |          BE951520             |
|                       |           CD611948           |          AI839621             |
|                       |           CK003449           |          BE333511             |
|                       |           BP322138           |          AI839060             |
|                       |           BQ651856           |          BY199234             |
|                       |           BM912150           |          BC068293             |
|                       |           BF527037           |                               |
|                       |           BI192675           |                               |
|                       |           BG752084           |                               |
|                       |           BI818837           |                               |
|                       |           BI838124           |                               |
|                       |           BM050424           |                               |
|                       |           BM842043           |                               |
|                       |           BM842136           |                               |
|                       |           BG827664           |                               |
|                       |           BE885489           |                               |
|                       |           AA404298           |                               |
|                       |           BG034505           |                               |
|                       |           BQ670915           |                               |
|                       |           BQ672288           |                               |
|                       |           BG325139           |                               |
|                       |           CB123512           |                               |
|                       |           BP226566           |                               |
|                       |           BQ888381           |                               |
|                       |           BM840712           |                               |
|                       |           BM843716           |                               |
|                       |           CD389364           |                               |
|                       |           BG340434           |                               |
|                       |           BI116130           |                               |
|                       |           BG325437           |                               |
|                       |           BG422495           |                               |
|                       |           BM686251           |                               |
|                       |           BG574593           |                               |
|                       |           BG255209           |                               |
|                       |           BQ685773           |                               |
|                       |           BG030814           |                               |
|                       |           BI850613           |                               |
|                       |           BG402874           |                               |
|                       |           BF343830           |                               |
|                       |           BU501231           |                               |
|                       |           BE741215           |                               |
|                       |           CK904675           |                               |
|                       |           BI193043           |                               |
|                       |           BM801612           |                               |
|                       |           BQ423119           |                               |
|                       |           BQ777997           |                               |
|                       |           BI861963           |                               |
|                       |           BI834528           |                               |
|                       |             U90906           |                               |
|                       |           BM549031           |                               |
|                       |           CB111239           |                               |
|                       |           BQ962089           |                               |
|                       |           BG477051           |                               |
|                       |           BE390799           |                               |
|                       |           BE385256           |                               |
|                       |           BE785118           |                               |
|                       |           BM791568           |                               |
|                       |           BQ933381           |                               |
|                       |           BC000510           |                               |
|                       |           BM825369           |                               |
|                       |           CF130633           |                               |
|                       |           BE514885           |                               |
|                       |           BM749049           |                               |
|                       |           BP205125           |                               |
|                       |           BE898826           |                               |
|                       |           BE746113           |                               |
|                       |           CD048929           |                               |
|                       |           BP286396           |                               |
|                       |           CB124585           |                               |
|                       |           BP290108           |                               |
|                       |           BP328236           |                               |
|                       |           BP206003           |                               |
|                       |           BM743003           |                               |
|                       |           BM475222           |                               |
|                       |           BM844014           |                               |
|                       |           BM840655           |                               |
|                       |           BM759348           |                               |
|                       |           BM840762           |                               |
|                       |           BP316718           |                               |
|                       |           BM845606           |                               |
|                       |           BM818453           |                               |
|                       |           BM781447           |                               |
|                       |           BI824458           |                               |
|                       |           CD389709           |                               |
|                       |           CB137441           |                               |
|                       |           BM761806           |                               |
|                       |           BM830269           |                               |
|                       |           BP214459           |                               |
|                       |           BE254464           |                               |
|                       |           CB119232           |                               |
|                       |           AL534829           |                               |
|                       |           AL534830           |                               |
|                       |           CR607252           |                               |
|                       |           AA479028           |                               |
|                       |           BP367775           |                               |
|                       |           BE385971           |                               |
|                       |           BE563588           |                               |
|                       |           BG574904           |                               |
|                       |           CB053901           |                               |
|                       |           BM313965           |                               |
|                       |           BU539281           |                               |
|                       |           BM459607           |                               |
|                       |           CF129824           |                               |
|                       |           BI836465           |                               |
|                       |           BG324851           |                               |
|                       |           BF220142           |                               |
|                       |           BQ671593           |                               |
|                       |           CB053999           |                               |
|                       |           BF525621           |                               |
|                       |           BG422569           |                               |
|                       |           BE734512           |                               |
|                       |           BM552426           |                               |
|                       |           BI764109           |                               |
|                       |           BI836927           |                               |
|                       |           AL544146           |                               |
|                       |           BF314375           |                               |
|                       |           BG259034           |                               |
|                       |           AL548665           |                               |
|                       |           BF312159           |                               |
|                       |           AL544171           |                               |
|                       |           CR605148           |                               |
|                       |           AL548683           |                               |
|                       |           CR603713           |                               |
|                       |           BF313234           |                               |
|                       |           BG425358           |                               |
|                       |           BU955875           |                               |
|                       |           BM479704           |                               |
|                       |           BM467931           |                               |
|                       |           BQ421188           |                               |
|                       |           BG114932           |                               |
|                       |           BU189491           |                               |
|                       |           BQ440413           |                               |
|                       |           BU508617           |                               |
|                       |           BG291100           |                               |
|                       |           BG574105           |                               |
|                       |           BC012292           |                               |
|                       |           BM556331           |                               |
|                       |           BF971497           |                               |
|                       |           BM810858           |                               |
|                       |           BI193578           |                               |
|                       |           BF971665           |                               |
|                       |           BM476750           |                               |
|                       |           BM794079           |                               |
|                       |           BQ931855           |                               |
|                       |           BG697838           |                               |
|                       |           CN295524           |                               |
|                       |           BU839684           |                               |
|                       |           BI252208           |                               |
|                       |             W65381           |                               |
|                       |           BI752488           |                               |
|                       |           AF086135           |                               |
|                       |           BM765089           |                               |
|                       |           AA465010           |                               |
|                       |           BM746692           |                               |
|                       |           AA292175           |                               |
|                       |           BF972795           |                               |
|                       |           BQ651753           |                               |
|                       |           BQ961964           |                               |
|                       |           BG679937           |                               |
|                       |           BI764153           |                               |
|                       |           BM789788           |                               |
|                       |             W56885           |                               |
|                       |           BG254455           |                               |
|                       |           BQ888341           |                               |
|                       |           BE616713           |                               |
|                       |           BQ227812           |                               |
|                       |           BG327929           |                               |
|                       |           BF237886           |                               |
|                       |           BI821030           |                               |
|                       |           BQ923242           |                               |
|                       |           BQ639756           |                               |
|                       |           BF342946           |                               |
|                       |           BG326164           |                               |
|                       |           BG324514           |                               |
|                       |           BQ441696           |                               |
|                       |           BM563413           |                               |
|                       |           BM697989           |                               |
|                       |           BE743084           |                               |
|                       |           BE261076           |                               |
|                       |           AW963387           |                               |
|                       |           BE263618           |                               |
|                       |           BU859646           |                               |
|                       |           BE787641           |                               |
|                       |           BI252950           |                               |
|                       |           AA122295           |                               |
|                       |           BE264080           |                               |
|                       |           BU527558           |                               |
|                       |           BU155243           |                               |
|                       |           BQ276503           |                               |
|                       |           BE273899           |                               |
|                       |           BG827321           |                               |
|                       |           BG474452           |                               |
|                       |           BG327537           |                               |
|                       |           BE879721           |                               |
|                       |           BE312459           |                               |
|                       |           BE256768           |                               |
|                       |           BM806002           |                               |
|                       |           BU527950           |                               |
|                       |           BE799378           |                               |
|                       |           CA389962           |                               |
|                       |           BI258490           |                               |
|                       |             X54079           |                               |
|                       |           BE888037           |                               |
|                       |           BE875458           |                               |
|                       |           CB995562           |                               |
|                       |           BU601991           |                               |
|                       |           BU596585           |                               |
|                       |           BU602344           |                               |
|                       |           BI552841           |                               |
|                       |           BI544821           |                               |
|                       |           BE615032           |                               |
|                       |           CF995213           |                               |
|                       |           BP382738           |                               |
|                       |           BG619109           |                               |
|                       |           BF036186           |                               |
|                       |           BP329438           |                               |
|                       |           BP382737           |                               |
|                       |           BG616243           |                               |
|                       |           BG614544           |                               |
|                       |           BG503584           |                               |
|                       |           BG613111           |                               |
|                       |           BG612793           |                               |
|                       |           CB150348           |                               |
|                       |           BM788557           |                               |
|                       |           BF670837           |                               |
|                       |           BM755135           |                               |
|                       |           BM761566           |                               |
|                       |           BG548098           |                               |
|                       |           BM839865           |                               |
|                       |           BM840859           |                               |
|                       |           BM845795           |                               |
|                       |           CB147038           |                               |
|                       |           BM818076           |                               |
|                       |           BP368555           |                               |
|                       |           BP317817           |                               |
|                       |           BP196791           |                               |
|                       |           BP270376           |                               |
|                       |           BP319931           |                               |
|                       |           BP323363           |                               |
|                       |           BP328405           |                               |
|                       |           BP331766           |                               |
|                       |           BP316950           |                               |
|                       |           BP318274           |                               |
|                       |           BP318990           |                               |
|                       |           BP329276           |                               |
|                       |           BP318231           |                               |
|                       |           BP319862           |                               |
|                       |           BG548374           |                               |
|                       |           BM833002           |                               |
|                       |           BM827714           |                               |
|                       |           BM788571           |                               |
|                       |           BG612721           |                               |
|                       |           BM764599           |                               |
|                       |           BM788165           |                               |
|                       |           BM834047           |                               |
|                       |           BM788568           |                               |
|                       |           BG171649           |                               |
|                       |           BM747288           |                               |
|                       |           BM762153           |                               |
|                       |           BM788168           |                               |
|                       |           BM838270           |                               |
|                       |           CD047884           |                               |
|                       |           CD050530           |                               |
|                       |           CD051091           |                               |
|                       |           CD050431           |                               |
|                       |           CD177381           |                               |
|                       |           CD389323           |                               |
|                       |           AU120726           |                               |
|                       |           CB107041           |                               |
|                       |           CD050804           |                               |
|                       |           CD388600           |                               |
|                       |           BM822066           |                               |
|                       |           CB107883           |                               |
|                       |           CB108030           |                               |
|                       |           CB136547           |                               |
|                       |           CB139332           |                               |
|                       |           CB107207           |                               |
|                       |           CB110262           |                               |
|                       |           CB137909           |                               |
|                       |           BM839321           |                               |
|                       |           CB124249           |                               |
|                       |           BP205605           |                               |
|                       |           BP261483           |                               |
|                       |           BP321836           |                               |
|                       |           BP321598           |                               |
|                       |           BP331603           |                               |
|                       |           BP262694           |                               |
|                       |           BP317008           |                               |
|                       |           BP210548           |                               |
|                       |           BP287417           |                               |
|                       |           CB134226           |                               |
|                       |           BP194061           |                               |
|                       |           BP317157           |                               |
|                       |           BP317276           |                               |
|                       |           BP317632           |                               |
|                       |           BP318838           |                               |
|                       |           BM819269           |                               |
|                       |           BP198193           |                               |
|                       |           BP316099           |                               |
|                       |           BP316793           |                               |
|                       |           BP318698           |                               |
|                       |           BP329250           |                               |
|                       |           BP205275           |                               |
|                       |           BP232204           |                               |
|                       |           BP260930           |                               |
|                       |           BP322427           |                               |
|                       |           BP317314           |                               |
|                       |           BP317906           |                               |
|                       |           BP321531           |                               |
|                       |           BM832521           |                               |
|                       |           BM827986           |                               |
|                       |           BM834405           |                               |
|                       |           BP316121           |                               |
|                       |           BP318527           |                               |
|                       |           BM787291           |                               |
|                       |           BM787855           |                               |
|                       |           BP340721           |                               |
|                       |           BM787412           |                               |
|                       |           BM834125           |                               |
|                       |           BM838285           |                               |
|                       |           BM837981           |                               |
|                       |           AU134929           |                               |
|                       |           CD386546           |                               |
|                       |           CD384953           |                               |
|                       |           BI457356           |                               |
|                       |           CD050046           |                               |
|                       |           CD385120           |                               |
|                       |           CD250162           |                               |
|                       |           CD048965           |                               |
|                       |           CD051223           |                               |
|                       |           CD556083           |                               |
|                       |           BI193092           |                               |
|                       |           CD176466           |                               |
|                       |           CD388165           |                               |
|                       |           CD385956           |                               |
|                       |           CD050450           |                               |
|                       |           CD387528           |                               |
|                       |           BI457056           |                               |
|                       |           BP223350           |                               |
|                       |           BP342788           |                               |
|                       |           BP319508           |                               |
|                       |           BM760302           |                               |
|                       |           CD389251           |                               |
|                       |           BM840814           |                               |
|                       |           BM746112           |                               |
|                       |           BM840241           |                               |
|                       |           BM843894           |                               |
|                       |           BM744553           |                               |
|                       |           BM841449           |                               |
|                       |           BM738715           |                               |
|                       |           BM841412           |                               |
|                       |           BM760242           |                               |
|                       |           BM840217           |                               |
|                       |           BM750014           |                               |
|                       |           BM782654           |                               |
|                       |           BM741660           |                               |
|                       |           AU136555           |                               |
|                       |           BM789185           |                               |
|                       |           BM765405           |                               |
|                       |           BM903681           |                               |
|                       |           CD048165           |                               |
|                       |           CD050229           |                               |
|                       |           CD051008           |                               |
|                       |           CB139496           |                               |
|                       |           CB138071           |                               |
|                       |           BP319914           |                               |
|                       |           BM747607           |                               |
|                       |           BM741327           |                               |
|                       |           BE884593           |                               |
|                       |           BM686599           |                               |
|                       |           CD558373           |                               |
|                       |           BM757401           |                               |
|                       |           AW161515           |                               |
|                       |           BM741374           |                               |
|                       |           BF219909           |                               |
|                       |           AA423916           |                               |
|                       |           BM686255           |                               |
|                       |           CN295526           |                               |
|                       |           BQ640743           |                               |
|                       |           BM687711           |                               |
|                       |           BM686269           |                               |
|                       |           BE267490           |                               |
|                       |           AW249070           |                               |
|                       |           BI762323           |                               |
|                       |           BG489702           |                               |
|                       |           BI195118           |                               |
|                       |           BE735830           |                               |
|                       |           BI195955           |                               |
|                       |           BM810604           |                               |
|                       |           BE798283           |                               |
|                       |           CN295511           |                               |
|                       |           BP279997           |                               |
|                       |           AA476366           |                               |
|                       |           BF724999           |                               |
|                       |           BE410680           |                               |
|                       |           BM045175           |                               |
|                       |           BG752568           |                               |
|                       |           BG422171           |                               |
|                       |           BM049598           |                               |
|                       |           BQ649821           |                               |
|                       |           BM706911           |                               |
|                       |           BU187177           |                               |
|                       |           BG751309           |                               |
|                       |           BG753653           |                               |
|                       |           BG677849           |                               |
|                       |           BG424260           |                               |
|                       |           BG331823           |                               |
|                       |           BG419196           |                               |
|                       |           BG327500           |                               |
|                       |           BG325604           |                               |
|                       |           AJ710399           |                               |
|                       |           BF726836           |                               |
|                       |           BM687756           |                               |
|                       |           CN295527           |                               |
|                       |           CD675585           |                               |
|                       |           BG164123           |                               |
|                       |           BI821263           |                               |
|                       |           BU152931           |                               |
|                       |           BM706933           |                               |
|                       |           BF970052           |                               |
|                       |           BI764296           |                               |
|                       |           CR611496           |                               |
|                       |           BM918922           |                               |
|                       |           BM926592           |                               |
|                       |           BM543708           |                               |
|                       |           BM808371           |                               |
|                       |           BI824387           |                               |
|                       |           BM924201           |                               |
|                       |           BM700056           |                               |
|                       |           AW245194           |                               |
|                       |           BF338948           |                               |
|                       |           BI857799           |                               |
|                       |           BU539232           |                               |
|                       |           BU856809           |                               |
|                       |           BU177947           |                               |
|                       |           AA148464           |                               |
|                       |           AJ711285           |                               |
|                       |           AA476529           |                               |
|                       |           BF337611           |                               |
|                       |           BG338882           |                               |
|                       |           BI835632           |                               |
|                       |           BF340205           |                               |
|                       |           BG338134           |                               |
|                       |           BG419587           |                               |
|                       |           BI518178           |                               |
|                       |           BM460530           |                               |
|                       |           AW160938           |                               |
|                       |           BI838531           |                               |
|                       |           BI194743           |                               |
|                       |           BG491544           |                               |
|                       |           BF685059           |                               |
|                       |           BM009116           |                               |
|                       |           BQ924295           |                               |
|                       |           BG104323           |                               |
|                       |           BF974084           |                               |
|                       |           BG104297           |                               |
|                       |           BE736003           |                               |
|                       |           BG748950           |                               |
|                       |           BF688189           |                               |
|                       |           BG281173           |                               |
|                       |           BI116294           |                               |
|                       |           BG419761           |                               |
|                       |           BM044759           |                               |
|                       |           BI761251           |                               |
|                       |           BG752448           |                               |
|                       |           BF973962           |                               |
|                       |           BG491633           |                               |
|                       |           BM010353           |                               |
|                       |           BG340095           |                               |
|                       |           BM700747           |                               |
|                       |           CA393724           |                               |
|                       |           CN295520           |                               |
|                       |           BG748221           |                               |
|                       |           BM793136           |                               |
|                       |           CN295525           |                               |
|                       |           AL079934           |                               |
|                       |           BI193651           |                               |
|                       |           CD675553           |                               |
|                       |           CN484600           |                               |
|                       |           BG258149           |                               |
|                       |           BI755891           |                               |
|                       |           BM706124           |                               |
|                       |           CD676345           |                               |
|                       |           BG698280           |                               |
|                       |           BI116845           |                               |
|                       |           BF526807           |                               |
|                       |           BI085110           |                               |
|                       |           BF791700           |                               |
|                       |           BG391849           |                               |
|                       |           BQ108684           |                               |
|                       |           BI835934           |                               |
|                       |           BI521115           |                               |
|                       |           BG253698           |                               |
|                       |           BI117720           |                               |
|                       |           BI836513           |                               |
|                       |           BI756156           |                               |
|                       |           BI759929           |                               |
|                       |           BG491943           |                               |
|                       |           BF685932           |                               |
|                       |           BI521618           |                               |
|                       |           BI856310           |                               |
|                       |           BQ048900           |                               |
|                       |           BG392527           |                               |
|                       |           BQ224827           |                               |
|                       |           BI522372           |                               |
|                       |           BG491872           |                               |
|                       |           BM800678           |                               |
|                       |           BF689134           |                               |
|                       |           BI517899           |                               |
|                       |           BQ718908           |                               |
|                       |           BM554902           |                               |
|                       |           BG327048           |                               |
|                       |           BG421042           |                               |
|                       |           BM802630           |                               |
|                       |           BQ216258           |                               |
|                       |           BU508181           |                               |
|                       |           BM541800           |                               |
|                       |           AB020027           |                               |
|                       |           BM560982           |                               |
|                       |           BQ432200           |                               |
|                       |           BC012768           |                               |
|                       |           BQ223663           |                               |
|                       |           BQ950588           |                               |
|                       |           BQ879760           |                               |
|                       |           BI836990           |                               |
|                       |           BI488827           |                               |
|                       |           BE302647           |                               |
|                       |           AW672756           |                               |
|                       |           BM811391           |                               |
|                       |           BE410800           |                               |
|                       |           BI489340           |                               |
|                       |           AL551506           |                               |
|                       |           BQ880960           |                               |
|                       |           BX403396           |                               |
|                       |           BM476244           |                               |
|                       |           CR609210           |                               |
|                       |           AL552709           |                               |
|                       |           BG163980           |                               |
|                       |           CR602417           |                               |
|                       |           AL551542           |                               |
|                       |           BM464159           |                               |
|                       |           CR591310           |                               |
|                       |           BM473828           |                               |
|                       |           BQ883975           |                               |
|                       |           BM912776           |                               |
|                       |           BM914102           |                               |
|                       |           BQ933966           |                               |
|                       |           BQ945319           |                               |
|                       |           BU856664           |                               |
|                       |           BM919872           |                               |
|                       |           BQ070317           |                               |
|                       |           BM909888           |                               |
|                       |           BM911973           |                               |
|                       |           AL552746           |                               |
|                       |           BG115554           |                               |
|                       |           BM926409           |                               |
|                       |           BX338390           |                               |
|                       |           CR606208           |                               |
|                       |           AV686832           |                               |
|                       |           BM689200           |                               |
|                       |           BM706084           |                               |
|                       |           AJ711189           |                               |
|                       |           BM700549           |                               |
|                       |           CD674472           |                               |
|                       |           CD674734           |                               |
|                       |           CD673792           |                               |
|                       |           BM272882           |                               |
|                       |           BU169990           |                               |
|                       |           BI085749           |                               |
|                       |           BU528785           |                               |
|                       |           BC073768           |                               |
|                       |           BQ213151           |                               |
|                       |           BM551275           |                               |
|                       |           BM924842           |                               |
|                       |           BQ213274           |                               |
|                       |           BM918574           |                               |
|                       |           BQ228916           |                               |
|                       |           AW630069           |                               |
|                       |           BI518007           |                               |
|                       |           BQ212614           |                               |
|                       |           BU168131           |                               |
|                       |           BQ921034           |                               |
|                       |           BM823712           |                               |
|                       |           AA461362           |                               |
|                       |           BM009172           |                               |
|                       |           BG334734           |                               |
|                       |           BM423925           |                               |
|                       |           BM423486           |                               |
|                       |           BG325837           |                               |
|                       |           AA057234           |                               |
|                       |           CN482268           |                               |
|                       |           AW629792           |                               |
|                       |           BU857467           |                               |
|                       |           BU537999           |                               |
|                       |           BQ637497           |                               |
|                       |           BU860466           |                               |
|                       |           BI489487           |                               |
|                       |           BI833496           |                               |
|                       |           BU194362           |                               |
|                       |           BU957493           |                               |
|                       |           BG477363           |                               |
|                       |           BU557735           |                               |
|                       |           BI117583           |                               |
|                       |           BU846414           |                               |
|                       |           BG829460           |                               |
|                       |           BQ950962           |                               |
|                       |           BU535435           |                               |
|                       |           BU857636           |                               |
|                       |           BX446337           |                               |
|                       |           BU944541           |                               |
|                       |           BI488604           |                               |
|                       |           BM799165           |                               |
|                       |           BQ102520           |                               |
|                       |           BM705945           |                               |
|                       |           BM692914           |                               |
|                       |           BQ086147           |                               |
|                       |           BI518214           |                               |
|                       |           BI517789           |                               |
|                       |           BU685108           |                               |
|                       |           BI819052           |                               |
|                       |             W46658           |                               |
|                       |           AW068180           |                               |
|                       |           AA427710           |                               |
|                       |           BG166933           |                               |
|                       |           AI791936           |                               |
|                       |           BG392920           |                               |
|                       |           BU783580           |                               |
|                       |           BQ631041           |                               |
|                       |           CD674863           |                               |
|                       |             W81664           |                               |
|                       |           AA479827           |                               |
|                       |           CN295509           |                               |
|                       |           BM509590           |                               |
|                       |           BM730051           |                               |
|                       |           BQ549902           |                               |
|                       |           CN295522           |                               |
|                       |           BG674009           |                               |
|                       |           BQ231957           |                               |
|                       |           BE409655           |                               |
|                       |           BI756135           |                               |
|                       |           BI820918           |                               |
|                       |           BM542281           |                               |
|                       |           BI759958           |                               |
|                       |           AV724332           |                               |
|                       |           BM685456           |                               |
|                       |           CD676117           |                               |
|                       |           BE883978           |                               |
|                       |           BM016561           |                               |
|                       |           BU955961           |                               |
|                       |           BU944119           |                               |
|                       |           BU944116           |                               |
|                       |           BU538683           |                               |
|                       |           AI096895           |                               |
|                       |           AW026252           |                               |
|                       |           AI827884           |                               |
|                       |           AL050380           |                               |
|                       |           AA886189           |                               |
|                       |           AI127185           |                               |
|                       |           BQ222180           |                               |
|                       |           AI347925           |                               |
|                       |           BQ222552           |                               |
|                       |           BM822477           |                               |
|                       |           BM142317           |                               |
|                       |           BM354070           |                               |
|                       |           AA464424           |                               |
|                       |           BF316092           |                               |
|                       |           BG747659           |                               |
|                       |           BE265160           |                               |
|                       |           AI692868           |                               |
|                       |           CD365558           |                               |
|                       |           BE265595           |                               |
|                       |           BE295392           |                               |
|                       |           CN295504           |                               |
|                       |           BF339792           |                               |
|                       |           BQ951393           |                               |
|                       |           BG476463           |                               |
|                       |           BM913450           |                               |
|                       |           BU526884           |                               |
|                       |           AI346199           |                               |
|                       |           BE266569           |                               |
|                       |           BM690630           |                               |
|                       |           BE746085           |                               |
|                       |           BE563363           |                               |
|                       |           BX281939           |                               |
|                       |           BE795033           |                               |
|                       |             W05483           |                               |
|                       |           BM510343           |                               |
|                       |           BM512219           |                               |
|                       |           CD671352           |                               |
|                       |           BU073017           |                               |
|                       |           BE273258           |                               |
|                       |           BE387365           |                               |
|                       |           BE254504           |                               |
|                       |           BI259766           |                               |
|                       |           BE790910           |                               |
|                       |           BE742313           |                               |
|                       |           BM069833           |                               |
|                       |           BE612505           |                               |
|                       |           BI760221           |                               |
|                       |           BE613092           |                               |
|                       |           BE794131           |                               |
|                       |           BE791363           |                               |
|                       |           BE875291           |                               |
|                       |           BE782359           |                               |
|                       |           BF037228           |                               |
|                       |           BE384161           |                               |
|                       |           BI761150           |                               |
|                       |           BE895965           |                               |
|                       |           BE276513           |                               |
|                       |           BE407882           |                               |
|                       |           BE267312           |                               |
|                       |           BE795267           |                               |
|                       |           BE293062           |                               |
|                       |           BE792198           |                               |
|                       |           BE264603           |                               |
|                       |           BE265634           |                               |
|                       |           CD674141           |                               |
|                       |           BE621368           |                               |
|                       |           BI196124           |                               |
|                       |           BQ935990           |                               |
|                       |           BM923737           |                               |
|                       |           BE274881           |                               |
|                       |           BE877630           |                               |
|                       |           BE789347           |                               |
|                       |           BE254928           |                               |
|                       |           BE728650           |                               |
|                       |           BE271336           |                               |
|                       |           BE875724           |                               |
|                       |           BE311989           |                               |
|                       |           BM685651           |                               |
|                       |           BE792340           |                               |
|                       |           BE741695           |                               |
|                       |           BE562970           |                               |
|                       |           BE264262           |                               |
|                       |           CR407614           |                               |
|                       |           CR536489           |                               |
|                       |           BQ223233           |                               |
|                       |           BE728553           |                               |
|                       |           BM556505           |                               |
|                       |           BE790799           |                               |
|                       |           BE389247           |                               |
|                       |           BE390577           |                               |
|                       |           CN295507           |                               |
|                       |           BE409070           |                               |
|                       |           BE878724           |                               |
|                       |           BE735672           |                               |
|                       |           BM716388           |                               |
|                       |           BE391501           |                               |
|                       |           BE514598           |                               |
|                       |           BE395183           |                               |
|                       |           BE275347           |                               |
|                       |           BI763019           |                               |
|                       |           BE894638           |                               |
|                       |           BE906911           |                               |
|                       |           BE408814           |                               |
|                       |           BE275697           |                               |
|                       |           BE736023           |                               |
|                       |           BE382907           |                               |
|                       |           BE877964           |                               |
|                       |           BE794351           |                               |
|                       |           BE794888           |                               |
|                       |           BE788047           |                               |
|                       |           BF025661           |                               |
|                       |           BE728962           |                               |
|                       |           BF685075           |                               |
|                       |           BU956001           |                               |
|                       |           CN295519           |                               |
|                       |           CN295515           |                               |
|                       |           CN295521           |                               |
|                       |           BE621622           |                               |
|                       |           AI357450           |                               |
|                       |           BQ636347           |                               |
|                       |             W49517           |                               |
|                       |           BP317689           |                               |
|                       |           BI253040           |                               |
|                       |           BE018857           |                               |
|                       |           BU599701           |                               |
|                       |           CK902507           |                               |
|                       |           BQ222538           |                               |
|                       |           BE546393           |                               |
|                       |           BQ009971           |                               |
|                       |           CA773830           |                               |
|                       |           AI954166           |                               |
|                       |           BC014920           |                               |
|                       |           CA777059           |                               |
|                       |           BQ879235           |                               |
|                       |           BE905191           |                               |
|                       |           AI806173           |                               |
|                       |           BQ009711           |                               |
|                       |           CA427421           |                               |
|                       |           AW170224           |                               |
|                       |           AI829534           |                               |
|                       |           AI075331           |                               |
|                       |           AI817076           |                               |
|                       |           BU071656           |                               |
|                       |           BM511434           |                               |
|                       |           AI361929           |                               |
|                       |           AI358883           |                               |
|                       |           BE882232           |                               |
|                       |           BQ183635           |                               |
|                       |           BM977447           |                               |
|                       |           BU687444           |                               |
|                       |             R65974           |                               |
|                       |           CD368060           |                               |
|                       |           CA420868           |                               |
|                       |           BM982271           |                               |
|                       |           AI074392           |                               |
|                       |           AI887243           |                               |
|                       |           BF432462           |                               |
|                       |           BG679844           |                               |
|                       |           BG169102           |                               |
|                       |           BG577089           |                               |
|                       |           AI246737           |                               |
|                       |           BF939964           |                               |
|                       |           BG421784           |                               |
|                       |           BU194962           |                               |
|                       |           BU071419           |                               |
|                       |           BE615436           |                               |
|                       |           BF940087           |                               |
|                       |           AI890118           |                               |
|                       |           BU071003           |                               |
|                       |           BF568856           |                               |
|                       |           BI194190           |                               |
|                       |           BF568558           |                               |
|                       |           AI080646           |                               |
|                       |           CD675221           |                               |
|                       |           BM700168           |                               |
|                       |           CA428391           |                               |
|                       |           BF038218           |                               |
|                       |           BM995058           |                               |
|                       |           CA428692           |                               |
|                       |           AI191036           |                               |
|                       |           AW949789           |                               |
|                       |           AA603124           |                               |
|                       |           BQ674149           |                               |
|                       |           BU948782           |                               |
|                       |           AW270146           |                               |
|                       |           AI814286           |                               |
|                       |           BU730383           |                               |
|                       |           CA412987           |                               |
|                       |           BI085900           |                               |
|                       |           AI814921           |                               |
|                       |           AW162543           |                               |
|                       |           BQ575654           |                               |
|                       |           BM973897           |                               |
|                       |           CA841197           |                               |
|                       |           BU949036           |                               |
|                       |           BM708851           |                               |
|                       |           BQ027161           |                               |
|                       |           CD370142           |                               |
|                       |           CD368584           |                               |
|                       |           BM990407           |                               |
|                       |           BQ182850           |                               |
|                       |           BU734332           |                               |
|                       |           BM313697           |                               |
|                       |           BF432520           |                               |
|                       |           BM512409           |                               |
|                       |           BM995823           |                               |
|                       |           BE742903           |                               |
|                       |           BU617309           |                               |
|                       |           BQ576084           |                               |
|                       |           BU616607           |                               |
|                       |           BU630653           |                               |
|                       |           BM996259           |                               |
|                       |           BG388990           |                               |
|                       |           AI936741           |                               |
|                       |           BF439830           |                               |
|                       |           BE906607           |                               |
|                       |           BM670932           |                               |
|                       |           CD369867           |                               |
|                       |           CA306698           |                               |
|                       |           AI982929           |                               |
|                       |           AW515302           |                               |
|                       |           BU626694           |                               |
|                       |           CA423912           |                               |
|                       |           AA888854           |                               |
|                       |           AA614670           |                               |
|                       |           AA161290           |                               |
|                       |           AI219271           |                               |
|                       |           CA307711           |                               |
|                       |           AI800198           |                               |
|                       |           BE888374           |                               |
|                       |           BM510603           |                               |
|                       |           BU148826           |                               |
|                       |           BM746309           |                               |
|                       |           AW088336           |                               |
|                       |           BU731588           |                               |
|                       |           BM990004           |                               |
|                       |           BQ613789           |                               |
|                       |           AI688882           |                               |
|                       |           BG178236           |                               |
|                       |           BM508162           |                               |
|                       |             R24227           |                               |
|                       |           BM688545           |                               |
|                       |           AW087342           |                               |
|                       |           BM511705           |                               |
|                       |           AA142930           |                               |
|                       |           AI569680           |                               |
|                       |           AW873514           |                               |
|                       |           AI589057           |                               |
|                       |           AA411194           |                               |
|                       |           AW102992           |                               |
|                       |           BE799997           |                               |
|                       |             H00499           |                               |
|                       |           BM668230           |                               |
|                       |           CA774132           |                               |
|                       |           AI609809           |                               |
|                       |           BE856415           |                               |
|                       |           AI801350           |                               |
|                       |           BQ777693           |                               |
|                       |           BQ920554           |                               |
|                       |           BM706551           |                               |
|                       |           BM557864           |                               |
|                       |           BM272633           |                               |
|                       |           AI829544           |                               |
|                       |           AI089434           |                               |
|                       |           AI039703           |                               |
|                       |           BM511979           |                               |
|                       |           AA912203           |                               |
|                       |             R21648           |                               |
|                       |           AW084753           |                               |
|                       |           AA937550           |                               |
|                       |           BQ267247           |                               |
|                       |           BF593626           |                               |
|                       |           BM505196           |                               |
|                       |           AJ711606           |                               |
|                       |           BE855706           |                               |
|                       |           BM069590           |                               |
|                       |           AA576790           |                               |
|                       |           AA161231           |                               |
|                       |           AW073403           |                               |
|                       |           AA464425           |                               |
|                       |           BM273123           |                               |
|                       |           AA384426           |                               |
|                       |             W47296           |                               |
|                       |           AI887837           |                               |
|                       |           BE858184           |                               |
|                       |           AI423035           |                               |
|                       |           BM128394           |                               |
|                       |           AA156766           |                               |
|                       |           BM311747           |                               |
|                       |           AW190677           |                               |
|                       |           BU601382           |                               |
|                       |           BE855683           |                               |
|                       |           BE858412           |                               |
|                       |           AA522817           |                               |
|                       |           AA127926           |                               |
|                       |           BM971096           |                               |
|                       |           BQ102262           |                               |
|                       |           BM142451           |                               |
|                       |           BU632920           |                               |
|                       |           CA424316           |                               |
|                       |           AA180863           |                               |
|                       |           AI423374           |                               |
|                       |           AA617812           |                               |
|                       |           BQ932518           |                               |
|                       |           AA411093           |                               |
|                       |           AA158873           |                               |
|                       |           AI283860           |                               |
|                       |           AI202865           |                               |
|                       |           BM665488           |                               |
|                       |           CB053900           |                               |
|                       |           AA127918           |                               |
|                       |           BU683196           |                               |
|                       |           AI222848           |                               |
|                       |           AI215160           |                               |
|                       |           BE855413           |                               |
|                       |           AA602578           |                               |
|                       |           AA613268           |                               |
|                       |           BF877236           |                               |
|                       |           AA292509           |                               |
|                       |           BG222388           |                               |
|                       |           CN295506           |                               |
|                       |           BI756136           |                               |
|                       |             R80330           |                               |
|                       |           AI366040           |                               |
|                       |           AA807827           |                               |
|                       |           AA631894           |                               |
|                       |           AA505424           |                               |
|                       |           AA642702           |                               |
|                       |           AI423985           |                               |
|                       |           AI094834           |                               |
|                       |           AW103073           |                               |
|                       |           AA708655           |                               |
|                       |           AA936744           |                               |
|                       |           BM997361           |                               |
|                       |           AI281809           |                               |
|                       |           AI283868           |                               |
|                       |           BE207489           |                               |
|                       |           AA404680           |                               |
|                       |           AA100896           |                               |
|                       |           AA614583           |                               |
|                       |           AI191301           |                               |
|                       |           AW963812           |                               |
|                       |           AA939325           |                               |
|                       |           AI222749           |                               |
|                       |           AI418473           |                               |
|                       |           AI380085           |                               |
|                       |           AA657968           |                               |
|                       |           AI347924           |                               |
|                       |           AI282210           |                               |
|                       |           AI312061           |                               |
|                       |           AI745137           |                               |
|                       |           AI037909           |                               |
|                       |           BX392926           |                               |
|                       |           AA293167           |                               |
|                       |           CR608836           |                               |
|                       |           AA631099           |                               |
|                       |           AA926717           |                               |
|                       |           AI271891           |                               |
|                       |           AI090389           |                               |
|                       |           BX360592           |                               |
|                       |           AA059410           |                               |
|                       |           AA416728           |                               |
|                       |           AA115776           |                               |
|                       |           AI419365           |                               |
|                       |           AA640950           |                               |
|                       |           AW663449           |                               |
|                       |           BE855383           |                               |
|                       |           AA928367           |                               |
|                       |           AI088773           |                               |
|                       |           AA575940           |                               |
|                       |           AA059367           |                               |
|                       |           AA829846           |                               |
|                       |           AI222892           |                               |
|                       |           AI827882           |                               |
|                       |           AA628623           |                               |
|                       |           AI079741           |                               |
|                       |           BQ183344           |                               |
|                       |           AA461186           |                               |
|                       |           AA770517           |                               |
|                       |           BE207703           |                               |
|                       |           AJ711992           |                               |
|                       |           AA865946           |                               |
|                       |           AA918227           |                               |
|                       |           AA741341           |                               |
|                       |           CA952887           |                               |
|                       |             H01371           |                               |
|                       |           AA826379           |                               |
|                       |           AA160804           |                               |
|                       |           AI277833           |                               |
|                       |           CA439823           |                               |
|                       |           AI222797           |                               |
|                       |           BU558067           |                               |
|                       |           BU737772           |                               |
|                       |           BM887777           |                               |
|                       |           AA479705           |                               |
|                       |           AA161222           |                               |
|                       |           AJ708301           |                               |
|                       |           AA115444           |                               |
|                       |           AI040081           |                               |
|                       |           AW087985           |                               |
|                       |           BU175386           |                               |
|                       |           AI027578           |                               |
|                       |           AA481902           |                               |
|                       |           AW070597           |                               |
|                       |           AW157007           |                               |
|                       |           AA476425           |                               |
|                       |           AI189943           |                               |
|                       |           BU737858           |                               |
|                       |             F25214           |                               |
|                       |           AA564745           |                               |
|                       |           AW362872           |                               |
|                       |           AI222791           |                               |
|                       |           AA983607           |                               |
|                       |           AI284106           |                               |
|                       |           AW248632           |                               |
|                       |             W84551           |                               |
|                       |           AA708909           |                               |
|                       |           AA151549           |                               |
|                       |           AI200309           |                               |
|                       |           AI004440           |                               |
|                       |           BM663109           |                               |
|                       |           BM672677           |                               |
|                       |           AI382180           |                               |
|                       |           AI073681           |                               |
|                       |           CD611946           |                               |
|                       |           CD611947           |                               |
|                       |           CD674729           |                               |
|                       |             F24668           |                               |
|                       |           AI139428           |                               |
|                       |           AA584260           |                               |
|                       |           AI188480           |                               |
|                       |           AI917142           |                               |
|                       |           AA554387           |                               |
|                       |           AI056342           |                               |
|                       |           AI188472           |                               |
|                       |           AI148130           |                               |
|                       |           BQ102251           |                               |
|                       |           AI582841           |                               |
|                       |           AI669110           |                               |
|                       |           AI024912           |                               |
|                       |           BF359268           |                               |
|                       |           AA341600           |                               |
|                       |           AA922873           |                               |
|                       |           BF985120           |                               |
|                       |           AA643275           |                               |
|                       |           AI690442           |                               |
|                       |           BM667156           |                               |
|                       |           CA435488           |                               |
|                       |           AA708502           |                               |
|                       |           AI811588           |                               |
|                       |           BM662995           |                               |
|                       |           BM663420           |                               |
|                       |           BU728236           |                               |
|                       |           BU728252           |                               |
|                       |             N54949           |                               |
|                       |           AA995757           |                               |
|                       |           BU626686           |                               |
|                       |           CA435951           |                               |
|                       |           CA425201           |                               |
|                       |           AW249652           |                               |
|                       |           AA583767           |                               |
|                       |           AA843475           |                               |
|                       |           CK904674           |                               |
|                       |           AA622555           |                               |
|                       |           AI352134           |                               |
|                       |           AI149133           |                               |
|                       |           AI245963           |                               |
|                       |           AI126940           |                               |
|                       |           AJ708268           |                               |
|                       |           BU786238           |                               |
|                       |           AI634310           |                               |
|                       |             F25167           |                               |
|                       |           AI183610           |                               |
|                       |           BE857281           |                               |
|                       |           AI336000           |                               |
|                       |           AI184758           |                               |
|                       |           AA643344           |                               |
|                       |           AI186879           |                               |
|                       |           AI359455           |                               |
|                       |           AI292055           |                               |
|                       |           AA464459           |                               |
|                       |           AI348045           |                               |
|                       |           AI373148           |                               |
|                       |           AI285508           |                               |
|                       |           AA455290           |                               |
|                       |           BF914746           |                               |
|                       |           AI358913           |                               |
|                       |           AI797266           |                               |
|                       |           BQ645233           |                               |
|                       |           BU839445           |                               |
|                       |           AI086004           |                               |
|                       |           AI204552           |                               |
|                       |           AI372921           |                               |
|                       |           AI336231           |                               |
|                       |             R67775           |                               |
|                       |           AI096415           |                               |
|                       |           AA937578           |                               |
|                       |           AI090065           |                               |
|                       |           AA612628           |                               |
|                       |             F25415           |                               |
|                       |           AA480050           |                               |
|                       |           AA885883           |                               |
|                       |           AI160809           |                               |
|                       |           BM782442           |                               |
|                       |           BM782622           |                               |
|                       |           AA496335           |                               |
|                       |           AI291291           |                               |
|                       |           AA834110           |                               |
|                       |           AI272735           |                               |
|                       |             F21894           |                               |
|                       |           AA444076           |                               |
|                       |           AA496384           |                               |
|                       |           AJ711512           |                               |
|                       |           AI161264           |                               |
|                       |           BM996435           |                               |
|                       |           AI281311           |                               |
|                       |           AI373455           |                               |
|                       |           AI374961           |                               |
|                       |           AW589579           |                               |
|                       |           AI346855           |                               |
|                       |           AI273344           |                               |
|                       |           AA992140           |                               |
|                       |           BE856796           |                               |
|                       |           BM993213           |                               |
|                       |             F35182           |                               |
|                       |           AJ709135           |                               |
|                       |           AI151011           |                               |
|                       |             F26924           |                               |
|                       |           AA371058           |                               |
|                       |           AA370765           |                               |
|                       |           AA371332           |                               |
|                       |           AA371249           |                               |
|                       |           AA370831           |                               |
|                       |           AA037607           |                               |
|                       |             W69311           |                               |
|                       |           AW613839           |                               |
|                       |           AI312751           |                               |
|                       |             W73909           |                               |
|                       |           BF438565           |                               |
|                       |           AW082440           |                               |
|                       |           AI351620           |                               |
|                       |           BU727974           |                               |
|                       |           AI040178           |                               |
|                       |           AI423230           |                               |
|                       |           BM826162           |                               |
|                       |           AA723695           |                               |
|                       |           AI635888           |                               |
|                       |           AI743670           |                               |
|                       |           AI339679           |                               |
|                       |           AI373849           |                               |
|                       |           AI811095           |                               |
|                       |           AI367137           |                               |
|                       |           AA505253           |                               |
|                       |           BQ023600           |                               |
|                       |           AI418952           |                               |
|                       |           AI089488           |                               |
|                       |           AA083960           |                               |
|                       |           AA444059           |                               |
|                       |           AA044229           |                               |
|                       |           BE146342           |                               |
|                       |           BE706143           |                               |
|                       |             F20213           |                               |
|                       |           BF974087           |                               |
|                       |           BM992923           |                               |
|                       |           BM667745           |                               |
|                       |           AI192765           |                               |
|                       |           BI013873           |                               |
|                       |           AA371765           |                               |
|                       |           AI360073           |                               |
|                       |           AI342193           |                               |
|                       |           AI346387           |                               |
|                       |           AI356118           |                               |
|                       |           AA845969           |                               |
|                       |           AI310744           |                               |
|                       |           AA304683           |                               |
|                       |           AA373062           |                               |
|                       |             R31988           |                               |
|                       |           AJ346064           |                               |
|                       |           AI278725           |                               |
|                       |           AI358904           |                               |
|                       |           AW074341           |                               |
|                       |           AI339708           |                               |
|                       |           AA369406           |                               |
|                       |           AW949765           |                               |
|                       |           BQ614257           |                               |
|                       |           CN295503           |                               |
|                       |             F28110           |                               |
|                       |           AJ573578           |                               |
|                       |           AA903154           |                               |
|                       |           BU782590           |                               |
|                       |           AA309314           |                               |
|                       |           AA303490           |                               |
|                       |           AI273423           |                               |
|                       |           AI923792           |                               |
|                       |             F18920           |                               |
|                       |           AI356233           |                               |
|                       |           AA916408           |                               |
|                       |           AA989499           |                               |
|                       |           AI015891           |                               |
|                       |           AA304275           |                               |
|                       |           AA069796           |                               |
|                       |           AA457105           |                               |
|                       |           AA583741           |                               |
|                       |           BQ030043           |                               |
|                       |           AA632048           |                               |
|                       |             W84480           |                               |
|                       |           AA586959           |                               |
|                       |           AA876480           |                               |
|                       |           BQ130324           |                               |
|                       |           AA938124           |                               |
|                       |           AI304770           |                               |
|                       |           AI347126           |                               |
|                       |           AI038603           |                               |
|                       |           AA373913           |                               |
|                       |           AA176083           |                               |
|                       |           AA143446           |                               |
|                       |           AI360863           |                               |
|                       |           AA040623           |                               |
|                       |           AI356119           |                               |
|                       |           AI271981           |                               |
|                       |           AA904369           |                               |
|                       |           AA284624           |                               |
|                       |           AA464172           |                               |
|                       |           AA936214           |                               |
|                       |           AI079808           |                               |
|                       |           AV729094           |                               |
|                       |           AA807481           |                               |
|                       |           AI367784           |                               |
|                       |             F25918           |                               |
|                       |           AJ573965           |                               |
|                       |           BQ130074           |                               |
|                       |           BF842729           |                               |
|                       |           BE146059           |                               |
|                       |           BQ614043           |                               |
|                       |           AI056493           |                               |
|                       |           AJ572303           |                               |
|                       |             N35984           |                               |
|                       |           AA155637           |                               |
|                       |           AI424194           |                               |
|                       |           AJ573597           |                               |
|                       |           AI346857           |                               |
|                       |           AI272891           |                               |
|                       |           AI361430           |                               |
|                       |           AI356685           |                               |
|                       |           BE146362           |                               |
|                       |           AI343888           |                               |
|                       |           AW173249           |                               |
|                       |           AI278534           |                               |
|                       |           AI348004           |                               |
|                       |           AI377864           |                               |
|                       |           AI274401           |                               |
|                       |           AA113282           |                               |
|                       |           AI361408           |                               |
|                       |           AI371898           |                               |
|                       |           AI285107           |                               |
|                       |             H97363           |                               |
|                       |           AI745126           |                               |
|                       |             R77032           |                               |
|                       |           BM980290           |                               |
|                       |           AA668537           |                               |
|                       |           AI829911           |                               |
|                       |           AI246761           |                               |
|                       |           AA383760           |                               |
|                       |             F27777           |                               |
|                       |           AA989067           |                               |
|                       |           AA961779           |                               |
|                       |             H97364           |                               |
|                       |           AI983393           |                               |
|                       |           AJ711839           |                               |
|                       |           AW250199           |                               |
|                       |           AI096525           |                               |
|                       |           AI588879           |                               |
|                       |           AA304596           |                               |
|                       |           BF439368           |                               |
|                       |           AJ708243           |                               |
|                       |           AA150583           |                               |
|                       |           AA987884           |                               |
|                       |           AA746809           |                               |
|                       |             N22710           |                               |
|                       |             F21110           |                               |
|                       |           AA903063           |                               |
|                       |             W60421           |                               |
|                       |           BE075380           |                               |
|                       |           AA505882           |                               |
|                       |           AI277276           |                               |
|                       |           AW084318           |                               |
|                       |           AI422700           |                               |
|                       |           AI262187           |                               |
|                       |             W02691           |                               |
|                       |           AA157278           |                               |
|                       |           AA961608           |                               |
|                       |           BU728443           |                               |
|                       |           BM054811           |                               |
|                       |           BM793382           |                               |
|                       |           AA668538           |                               |
|                       |           AA505998           |                               |
|                       |           AI273222           |                               |
|                       |             N98516           |                               |
|                       |           AI962003           |                               |
|                       |           AI268889           |                               |
|                       |           AJ711617           |                               |
|                       |           BM993231           |                               |
|                       |           AA079832           |                               |
|                       |           AI346153           |                               |
|                       |           AI754202           |                               |
|                       |           BF743603           |                               |
|                       |           AA622214           |                               |
|                       |           CA840939           |                               |
|                       |           AA159008           |                               |
|                       |           AA564162           |                               |
|                       |           AJ706920           |                               |
|                       |           AA725379           |                               |
|                       |           BI005956           |                               |
|                       |           AA923400           |                               |
|                       |           AA156408           |                               |
|                       |           AA112568           |                               |
|                       |           AI368472           |                               |
|                       |           AI383746           |                               |
|                       |           AI953434           |                               |
|                       |           AI383745           |                               |
|                       |           BU733319           |                               |
|                       |             W45175           |                               |
|                       |           AJ707726           |                               |
|                       |           AI304683           |                               |
|                       |           BF762783           |                               |
|                       |           BF761643           |                               |
|                       |           AW369722           |                               |
|                       |           AI188723           |                               |
|                       |           AA665943           |                               |
|                       |             F28567           |                               |
|                       |           BF842540           |                               |
|                       |           AA640076           |                               |
|                       |           AA136837           |                               |
|                       |           AA045787           |                               |
|                       |           AA055484           |                               |
|                       |           AW367092           |                               |
|                       |             R63847           |                               |
|                       |           AA113176           |                               |
|                       |             F17707           |                               |
|                       |           AA157559           |                               |
|                       |             W01874           |                               |
|                       |             H01324           |                               |
|                       |           AI359057           |                               |
|                       |             X16477           |                               |
|                       |           AA291350           |                               |
|                       |             N33124           |                               |
|                       |           CD108448           |                               |
|                       |           BF940353           |                               |
|                       |           AA427552           |                               |
|                       |             F22659           |                               |
|                       |           AJ572954           |                               |
|                       |           BU526475           |                               |
|                       |             N33502           |                               |
|                       |           AA064612           |                               |
|                       |           AI424204           |                               |
|                       |             F25221           |                               |
|                       |             F19424           |                               |
|                       |           AA069125           |                               |
|                       |             H61763           |                               |
|                       |           AI753637           |                               |
|                       |             W61120           |                               |
|                       |           AA181915           |                               |
|                       |           AJ573693           |                               |
|                       |           AA976146           |                               |
|                       |           BE093117           |                               |
|                       |             F36087           |                               |
|                       |             R65975           |                               |
|                       |           AA112159           |                               |
|                       |           AW269579           |                               |
|                       |             H57331           |                               |
|                       |             F20695           |                               |
|                       |           AA157563           |                               |
|                       |           AI625504           |                               |
|                       |           BF341225           |                               |
|                       |           BM664348           |                               |
|                       |           AA455835           |                               |
|                       |           AA022819           |                               |
|                       |           AL574808           |                               |
|                       |           AL569053           |                               |
|                       |           AL576265           |                               |
|                       |           BM785076           |                               |
|                       |           AU129587           |                               |
|                       |           AW327453           |                               |
|                       |           AU151674           |                               |
|                       |           BI257459           |                               |
|                       |             H41858           |                               |
|                       |             H18172           |                               |
|                       |           BU521180           |                               |
|                       |             H39197           |                               |
|                       |           BU960876           |                               |
|                       |           BE904672           |                               |
|                       |           BE175125           |                               |
|                       |           CB108032           |                               |
|                       |           CB107674           |                               |
|                       |           AA301834           |                               |
|                       |           AI684898           |                               |
|                       |           CN295510           |                               |
|                       |           CN295517           |                               |
|                       |           AI922190           |                               |
|                       |           AI087058           |                               |
|                       |           BE146065           |                               |
|                       |           BU941930           |                               |
|                       |             H18173           |                               |
|                       |             N29960           |                               |
|                       |           CD176072           |                               |
|                       |             H23804           |                               |
|                       |           BF696100           |                               |
|                       |           BI850270           |                               |
|                       |           CD248839           |                               |
|                       |           CN295518           |                               |
|                       |           AW327454           |                               |
|                       |           CD174784           |                               |
|                       |           BU536618           |                               |
|                       |           BU957947           |                               |
|                       |           BU927965           |                               |
|                       |             F24348           |                               |
|                       |           CD176419           |                               |
|                       |           AI304431           |                               |
|                       |             F30815           |                               |
|                       |             F26486           |                               |
|                       |             F36080           |                               |
|                       |           AA304608           |                               |
|                       |           BX444208           |                               |
|                       |           BF838927           |                               |
|                       |           AW796853           |                               |
|                       |           AA319837           |                               |
|                       |           BF823451           |                               |
|                       |           BG014513           |                               |
|                       |           AA042877           |                               |
|                       |           AA492197           |                               |
|                       |             F19027           |                               |
|                       |           BF838941           |                               |
|                       |             F30102           |                               |
|                       |             F34114           |                               |
|                       |             F35262           |                               |
|                       |           AJ573545           |                               |
|                       |           AJ346698           |                               |
|                       |           AI263942           |                               |
|                       |           AJ712117           |                               |
|                       |           AW975342           |                               |
|                       |             F25727           |                               |
|                       |           AJ572941           |                               |
|                       |           AJ573090           |                               |
|                       |           AA058679           |                               |
|                       |           AA160792           |                               |
|                       |           AI355151           |                               |
|                       |           AJ572279           |                               |
|                       |             F27840           |                               |
|                       |             H99647           |                               |
|                       |             W46623           |                               |
|                       |           AJ573738           |                               |
|                       |           AA156511           |                               |
|                       |           AI358936           |                               |
|                       |           AJ572805           |                               |
|                       |           BU538716           |                               |
|                       |           BU956405           |                               |
|                       |           BU956833           |                               |
|                       |           AJ707367           |                               |
|                       |             N26789           |                               |
|                       |             N34440           |                               |
|                       |           AA115445           |                               |
|                       |           AJ573994           |                               |
|                       |           AJ708135           |                               |
|                       |           BQ300586           |                               |
|                       |           AU122906           |                               |
|                       |             F20743           |                               |
|                       |             F35930           |                               |
|                       |             F30535           |                               |
|                       |           BU752372           |                               |
|                       |           AA293274           |                               |
|                       |           AA954353           |                               |
|                       |             F32293           |                               |
|                       |           BI014344           |                               |
|                       |             H73956           |                               |
|                       |             F29355           |                               |
|                       |           AA194639           |                               |
|                       |             R76533           |                               |
|                       |             S74571           |                               |
|                       |           AI031925           |                               |
|                       |           AA052937           |                               |
|                       |           AA410890           |                               |
|                       |           CF528418           |                               |
|                       |           AI264454           |                               |
|                       |           CN295514           |                               |
|                       |           AI754679           |                               |
|                       |           AA401937           |                               |
|                       |           AI241677           |                               |
|                       |           AI074914           |                               |
|                       |           AI262188           |                               |
|                       |           AA070636           |                               |
|                       |           BQ717173           |                               |
|                       |           AA826863           |                               |
|                       |             H23801           |                               |
|                       |           AW602287           |                               |
|                       |           BE693879           |                               |
|                       |           AI190057           |                               |
|                       |           AJ573719           |                               |
|                       |           AA953272           |                               |
|                       |           AI159971           |                               |
|                       |           AA587173           |                               |
|                       |             R24286           |                               |
|                       |           BQ021969           |                               |
|                       |           BQ023583           |                               |
|                       |             F22735           |                               |
|                       |           AA384494           |                               |
|                       |           AA775726           |                               |
|                       |           BG992791           |                               |
|                       |           BM672656           |                               |
|                       |           BI054959           |                               |
|                       |           BI053748           |                               |
|                       |             F19535           |                               |
|                       |           AA357444           |                               |
|                       |           BG988561           |                               |
|                       |             F35341           |                               |
|                       |             F31868           |                               |
|                       |           AA410891           |                               |
|                       |           AA443613           |                               |
|                       |             F30571           |                               |
|                       |           AA552474           |                               |
|                       |           BF842542           |                               |
|                       |             F30974           |                               |
|                       |           BG283583           |                               |
|                       |           BG992782           |                               |
|                       |           BQ300481           |                               |
|                       |           BF748142           |                               |
|                       |           BQ300370           |                               |
|                       |           BQ300437           |                               |
|                       |           BI009017           |                               |
|                       |           BQ300368           |                               |
|                       |           BQ300380           |                               |
|                       |           BQ300382           |                               |
|                       |           BQ300385           |                               |
|                       |           BQ300435           |                               |
|                       |           BQ300436           |                               |
|                       |           BQ300445           |                               |
|                       |           BQ300447           |                               |
|                       |           BQ300453           |                               |
|                       |           BQ300566           |                               |
|                       |             F35550           |                               |
|                       |           BG011794           |                               |
|                       |             F27709           |                               |
|                       |           AA465024           |                               |
|                       |           AI141412           |                               |
|                       |             N72155           |                               |
|                       |           AA551982           |                               |
|                       |           BG993111           |                               |
|                       |           AJ572750           |                               |
|                       |             F25623           |                               |
|                       |           AA422130           |                               |
|                       |             N84443           |                               |
|                       |           BQ306987           |                               |
|                       |           AA455028           |                               |
|                       |           AJ571940           |                               |
|                       |           BU857845           |                               |
|                       |           AI423206           |                               |
|                       |           AJ572493           |                               |
|                       |           AI347062           |                               |
|                       |             F29097           |                               |
|                       |             F26454           |                               |
|                       |             R34104           |                               |
|                       |           AI352618           |                               |
|                       |           BF841488           |                               |
|                       |             T49405           |                               |
|                       |             F24174           |                               |
|                       |           BF737382           |                               |
|                       |           BF737381           |                               |
|                       |           BF737407           |                               |
|                       |           BG988568           |                               |
|                       |           BF845407           |                               |
|                       |             F26721           |                               |
|                       |           AJ574069           |                               |
|                       |           AW370237           |                               |
|                       |           BF823465           |                               |
|                       |           BQ299961           |                               |
|                       |           BF747276           |                               |
|                       |             F25659           |                               |
|                       |             F28660           |                               |
|                       |           AJ573276           |                               |
|                       |           AJ346769           |                               |
|                       |           AW081825           |                               |
|                       |           AJ572080           |                               |
|                       |             T98668           |                               |
|                       |             F30962           |                               |
|                       |           AA688231           |                               |
|                       |           AJ574103           |                               |
|                       |           AA875864           |                               |
|                       |           AA478150           |                               |
|                       |           AW068090           |                               |
|                       |           AW873735           |                               |
|                       |           AA022727           |                               |
|                       |           AJ573541           |                               |
|                       |             F31827           |                               |
|                       |             F31054           |                               |
|                       |             F29208           |                               |
|                       |             F36089           |                               |
|                       |           AA328897           |                               |
|                       |             T59720           |                               |
|                       |           AI824362           |                               |
|                       |           AA425421           |                               |
|                       |           AI868326           |                               |
|                       |           AA374781           |                               |
|                       |           AA295701           |                               |
|                       |           BE620824           |                               |
|                       |           BG012399           |                               |
|                       |           BE620290           |                               |
|                       |           AA366006           |                               |
|                       |           BQ029931           |                               |
|                       |             F33332           |                               |
|                       |             F19400           |                               |
|                       |           AI701087           |                               |
|                       |           CA412143           |                               |
|                       |           BU632805           |                               |
|                       |           BM672674           |                               |
|                       |           CN295528           |                               |
|                       |           CN295513           |                               |
|                       |           AI984865           |                               |
|                       |           BU732342           |                               |
|                       |           AW082755           |                               |
|                       |           AA505728           |                               |
|                       |           AW085904           |                               |
|                       |           AA455306           |                               |
|                       |           AI266012           |                               |
|                       |           CN295505           |                               |
|                       |           AI302958           |                               |
|                       |             F29965           |                               |
|                       |           BU727709           |                               |
|                       |           AW148353           |                               |
|                       |           BU730242           |                               |
|                       |           BQ305253           |                               |
|                       |           AA507581           |                               |
|                       |           BU734636           |                               |
|                       |           CD369201           |                               |
|                       |           AA587156           |                               |
|                       |           BM670175           |                               |
|                       |           BM842178           |                               |
|                       |           AI308781           |                               |
|                       |           AI478462           |                               |
|                       |           AI680947           |                               |
|                       |           AV726582           |                               |
|                       |           CA335501           |                               |
|                       |             M85895           |                               |
|                       |           BM991365           |                               |
|                       |           AW293605           |                               |
|                       |           BF947687           |                               |
|                       |           BQ024351           |                               |
|                       |           CN360728           |                               |
|                       |           BF357005           |                               |
|                       | *Seq. mapped in strand Minus*| *Seq. mapped in strand Minus* |
|                       |           AI801539           |          AA733731             |
|                       |           CB243046           |          AA592386             |
|                       |           BM670118           |          CD645161             |
|                       |           BI495132           |          CF380251             |
|                       |             T95736           |          CF380562             |
|                       |           BF222159           |          CF926225             |
|                       |           AA548251           |          CF928562             |
|                       |           AI040457           |          CF928700             |
|                       |           BM663475           |          CK382098             |
|                       |           CK904976           |          CF620583             |
|                       |           BU634095           |          CF804609             |
|                       |           AI978599           |          CF970299             |
|                       |           BU742279           |          CK371986             |
|                       |           AA136287           |          CK380282             |
|                       |           CB241694           |          CK380818             |
|                       |           CA944563           |          CK428589             |
|                       |           AJ713900           |          AI847320             |
|                       |           CB113013           |          AI841021             |
|                       |           AA137224           |          AI836946             |
+-----------------------+------------------------------+-------------------------------+
|      #   40           | *Seq. mapped in strand Plus* | *Seq. mapped in strand Plus*  |
|                       |           BM990037           |          BF466105             |
|                       |           CA442210           |          BB697656             |
|                       |           BF512350           |          BX527391             |
|                       |           BG193156           |          AA271279             |
|                       |           BG203404           |          AI662694             |
|                       |           BG208105           |          BY354970             |
|                       |           BG214955           |                               |
|                       |           BG192108           |                               |
|                       |           BG212809           |                               |
|                       |           BG202893           |                               |
|                       |           BG190086           |                               |
|                       |           BG217629           |                               |
|                       |           BG185828           |                               |
|                       |           BG192109           |                               |
|                       |           BG200919           |                               |
|                       |           BG212810           |                               |
|                       |           BG216142           |                               |
|                       |           BG211226           |                               |
|                       |           BG206040           |                               |
|                       |           BG197204           |                               |
|                       |           BG204917           |                               |
|                       |           BG206534           |                               |
|                       |           BF515048           |                               |
|                       |           BF057591           |                               |
|                       |           AW297032           |                               |
|                       |           AW474214           |                               |
|                       |           BF512839           |                               |
|                       | *Seq. mapped in strand Minus*| *Seq. mapped in strand Minus* |
|                       |           AW263265           |          AI848036             |
|                       |           BM994681           |          AK013538             |
|                       |           BM976586           |          BQ175813             |
|                       |           BU736438           |          BF466495             |
|                       |           CB305894           |          BI408308             |
|                       |           BM684539           |          BC034609             |
+-----------------------+------------------------------+-------------------------------+
|      #   41           | *Seq. mapped in strand Plus* | *Seq. mapped in strand Plus*  |
|                       |           BC036100           |          BC038475             |
|                       |           BC036443           |          AK013035             |
|                       |           AL161976           |          AK012171             |
|                       |           AF030196           |                               |
|                       |           CR542138           |                               |
|                       |           CR542127           |                               |
|                       |           BC005837           |                               |
|                       |           AK025795           |                               |
|                       | *Seq. mapped in strand Minus*| *Seq. mapped in strand Minus* |
|                       |           BC013727           |          BC019564             |
|                       |           AF131780           |          BC029643             |
|                       |           AK027464           |          BC020363             |
|                       |           AK027646           |          AK013044             |
|                       |           BC002856           |          AK029166             |
|                       |           BX648407           |                               |
|                       |           CR457152           |                               |
|                       |             D16955           |                               |
|                       |           BC018635           |                               |
+-----------------------+------------------------------+-------------------------------+
|      #   42           | *Seq. mapped in strand Plus* | *Seq. mapped in strand Plus*  |
|                       |           BP356683           |          AK002633             |
|                       |           BP315154           |          BC055035             |
|                       |           AU140918           |          AF411213             |
|                       |           BC069633           |          BC042575             |
|                       |           BP313140           |          BC004783             |
|                       |           BX950928           |          BC041795             |
|                       |           BI057280           |                               |
|                       |           BI057282           |                               |
|                       |           BX505717           |                               |
|                       |           AI872702           |                               |
|                       |           BF370440           |                               |
|                       |           AW473832           |                               |
|                       |           AI918478           |                               |
|                       |           AU137827           |                               |
|                       |           AK002034           |                               |
|                       | *Seq. mapped in strand Minus*| *Seq. mapped in strand Minus* |
|                       |           BQ012533           |          AK017454             |
|                       |           AI769212           |          AK005410             |
|                       |           BM979613           |                               |
|                       |           BX105796           |                               |
+-----------------------+------------------------------+-------------------------------+
|      #   43           | *Seq. mapped in strand Plus* | *Seq. mapped in strand Plus*  |
|                       |           BC037849           |          BB639924             |
|                       |                              |          CD553501             |
|                       |                              |          AW048064             |
|                       |                              |          AB091828             |
|                       |                              |          BY271035             |
|                       |                              |          BY277231             |
|                       |                              |          BY282067             |
|                       |                              |          BF464561             |
|                       |                              |          BF467645             |
|                       |                              |          BY239553             |
|                       |                              |          BC053748             |
|                       |                              |          BB617696             |
|                       |                              |          AK030399             |
|                       |                              |          AY308065             |
|                       |                              |          BB615501             |
|                       |                              |          AK029737             |
|                       |                              |          BB748939             |
|                       |                              |          BB326617             |
|                       |                              |          BC048729             |
|                       |                              |          BU514202             |
|                       |                              |          BB605747             |
|                       |                              |          AK122472             |
|                       |                              |          BE859473             |
|                       |                              |          AV266576             |
|                       |                              |          BQ950734             |
|                       |                              |          CD548931             |
|                       |                              |          BU563388             |
|                       |                              |          CF744482             |
|                       |                              |          CF746025             |
|                       | *Seq. mapped in strand Minus*| *Seq. mapped in strand Minus* |
|                       |           AK094892           |          BE993450             |
+-----------------------+------------------------------+-------------------------------+
|      #   44           | *Seq. mapped in strand Plus* | *Seq. mapped in strand Plus*  |
|                       |           BP372920           |          BG083611             |
|                       |           BX418162           |          AK011900             |
|                       |           BM547121           |          AI852439             |
|                       |           BG718557           |          CB519985             |
|                       |           AU098965           |          BE953692             |
|                       |           BP315838           |          AK085234             |
|                       |           BC003684           |                               |
|                       |           BP239273           |                               |
|                       |           BP241115           |                               |
|                       |           BP238588           |                               |
|                       |           BM750447           |                               |
|                       |           BP243944           |                               |
|                       |           BP239172           |                               |
|                       |           BP242838           |                               |
|                       |           BP243151           |                               |
|                       |           BP242100           |                               |
|                       |           BP280595           |                               |
|                       |           AU131437           |                               |
|                       |           BX376692           |                               |
|                       |           BE545085           |                               |
|                       |           AL521066           |                               |
|                       |           BC010536           |                               |
|                       |           AL514786           |                               |
|                       |             Y07593           |                               |
|                       |           BE543957           |                               |
|                       |             C16023           |                               |
|                       |           BP259806           |                               |
|                       |           BP240032           |                               |
|                       |           BP241054           |                               |
|                       |           CD708568           |                               |
|                       |           CF597534           |                               |
|                       |           BP342597           |                               |
|                       |           BP237540           |                               |
|                       |           CV023420           |                               |
|                       |           AY072910           |                               |
|                       |           AY072911           |                               |
|                       |           AY072912           |                               |
|                       |             U90716           |                               |
|                       |           BI754365           |                               |
|                       |           AL709335           |                               |
|                       |           AF124598           |                               |
|                       |             N31467           |                               |
|                       |           AL534436           |                               |
|                       |           AL555497           |                               |
|                       |           CR617256           |                               |
|                       |           BP343393           |                               |
|                       |             W39780           |                               |
|                       |           CN369264           |                               |
|                       |           AA249468           |                               |
|                       |           AA299472           |                               |
|                       |           CB132874           |                               |
|                       |           BP221156           |                               |
|                       |           BU192852           |                               |
|                       |           AL577981           |                               |
|                       |           CN274390           |                               |
|                       |           BG570867           |                               |
|                       |           BP293870           |                               |
|                       |           CD238805           |                               |
|                       |           CR603583           |                               |
|                       |           CR601061           |                               |
|                       |           BI492573           |                               |
|                       |           CA314161           |                               |
|                       |           BU677040           |                               |
|                       |           BU677281           |                               |
|                       |           AW206290           |                               |
|                       |           BX097283           |                               |
|                       |           AI142558           |                               |
|                       |           AI925064           |                               |
|                       |           AA622661           |                               |
|                       |           AA933888           |                               |
|                       |           AA244134           |                               |
|                       |           BU730087           |                               |
|                       |           AI955070           |                               |
|                       | *Seq. mapped in strand Minus*| *Seq. mapped in strand Minus* |
|                       |           AW205793           |          BB623918             |
|                       |           CR593770           |          BE457041             |
|                       |           BM807346           |            Z72000             |
|                       |           AW169377           |            D83745             |
|                       |           CN411027           |          BC012705             |
|                       |           AI367479           |          BU755803             |
|                       |           BM662333           |          CA451775             |
|                       |           BU784991           |          CA979391             |
|                       |           BM679300           |          BU841072             |
|                       |           BF061534           |          BF167325             |
|                       |           AI688680           |          BI655002             |
|                       |           BM876335           |          CB573525             |
|                       |           AI767978           |          AA254058             |
|                       |           BM992747           |          BI657368             |
|                       |           CA418138           |          BF720821             |
|                       |           CB241265           |          CA463631             |
|                       |           AW161922           |          AI226301             |
|                       |           CB850690           |          AW823883             |
|                       |           CB853277           |          CF617407             |
|                       |           BU631658           |          CK794935             |
|                       |           BQ774591           |          BF101870             |
|                       |           CD742605           |          BI697568             |
|                       |           CA443136           |          CF892747             |
|                       |           BU619844           |            W48364             |
|                       |           AI671860           |          CF895319             |
|                       |           AW118461           |          CK795952             |
|                       |           BM669031           |          BF152185             |
|                       |           CK818627           |          BB867895             |
|                       |           CA841831           |          CK623612             |
|                       |           BQ015229           |          BE626245             |
|                       |           CR602342           |          BE848412             |
|                       |           CA418383           |          CA773118             |
|                       |           BC011957           |          BE333612             |
|                       |           CR598696           |          BF467448             |
|                       |           CA868329           |                               |
|                       |           CR620760           |                               |
|                       |           AL578317           |                               |
|                       |           AL544244           |                               |
|                       |           AL571451           |                               |
|                       |           AL575496           |                               |
|                       |           CR608349           |                               |
|                       |           CR600061           |                               |
|                       |           CR607894           |                               |
|                       |           AL526948           |                               |
|                       |           CR604425           |                               |
|                       |           BX393147           |                               |
|                       |           BX427913           |                               |
|                       |           CR618065           |                               |
|                       |           CR590787           |                               |
|                       |           AL574613           |                               |
|                       |           CR618737           |                               |
|                       |           AL525459           |                               |
|                       |             D64110           |                               |
|                       |           CB051507           |                               |
|                       |             N95516           |                               |
|                       |             W58339           |                               |
|                       |           CD709054           |                               |
|                       |           CB046846           |                               |
|                       |             N24293           |                               |
|                       |           BM691712           |                               |
|                       |           BE535748           |                               |
|                       |           AL526987           |                               |
|                       |           AL550864           |                               |
|                       |           BI544358           |                               |
|                       |           BX105675           |                               |
|                       |           BG743348           |                               |
|                       |           AL523247           |                               |
|                       |           AL555952           |                               |
|                       |           BG529835           |                               |
|                       |           CA393252           |                               |
|                       |           CB146802           |                               |
|                       |           BX380433           |                               |
|                       |           BM559707           |                               |
|                       |           BM546833           |                               |
|                       |           BX427915           |                               |
|                       |           BM705292           |                               |
|                       |           BX383585           |                               |
|                       |           CR457061           |                               |
|                       |           BT007276           |                               |
|                       |           BG110736           |                               |
|                       |           BG675997           |                               |
|                       |           BM559915           |                               |
|                       |           BE168810           |                               |
|                       |           BU852582           |                               |
|                       |           BU526704           |                               |
|                       |           BI917161           |                               |
|                       |           BI086894           |                               |
|                       |           CA454434           |                               |
|                       |           CD105186           |                               |
|                       |           AL552097           |                               |
|                       |           CB115543           |                               |
|                       |           AL544262           |                               |
|                       |           BX427914           |                               |
|                       |           BQ233579           |                               |
|                       |           BG742973           |                               |
|                       |           BU177872           |                               |
|                       |           BM838970           |                               |
|                       |           BM839026           |                               |
|                       |           BU155086           |                               |
|                       |           BI668105           |                               |
|                       |           BF083478           |                               |
|                       |           BQ291951           |                               |
|                       |           BF088322           |                               |
|                       |           BF155521           |                               |
|                       |           BM836239           |                               |
|                       |           BG622455           |                               |
|                       |             N35197           |                               |
|                       |             N74741           |                               |
|                       |           BF805371           |                               |
|                       |           CN280487           |                               |
|                       |           BE896159           |                               |
|                       |           CB991573           |                               |
|                       |           BG035566           |                               |
|                       |           CD109522           |                               |
|                       |           BF244456           |                               |
|                       |           CB145776           |                               |
|                       |           BI833139           |                               |
|                       |           BF034231           |                               |
|                       |           BI465354           |                               |
|                       |           BM720581           |                               |
|                       |           BU600093           |                               |
|                       |           BM876597           |                               |
|                       |           AW238814           |                               |
|                       |           BM842873           |                               |
|                       |           CN280486           |                               |
|                       |             H91423           |                               |
|                       |           BG699651           |                               |
|                       |           AI609557           |                               |
|                       |           CB995109           |                               |
|                       |           BM842452           |                               |
|                       |           BP289906           |                               |
|                       |           CB138860           |                               |
|                       |           BM842360           |                               |
|                       |           CA842125           |                               |
|                       |           BP367364           |                               |
|                       |           AW629632           |                               |
|                       |           BU785252           |                               |
|                       |           BX458254           |                               |
|                       |           BM752227           |                               |
|                       |           AW161580           |                               |
|                       |           BE906747           |                               |
|                       |           BG656180           |                               |
|                       |           BM763197           |                               |
|                       |           BP870752           |                               |
|                       |           BP870769           |                               |
|                       |           BU564223           |                               |
+-----------------------+------------------------------+-------------------------------+
|      #   45           | *Seq. mapped in strand Plus* | *Seq. mapped in strand Plus*  |
|                       |           AF130056           |          CF356444             |
|                       |                              |          BI793604             |
|                       | *Seq. mapped in strand Minus*| *Seq. mapped in strand Minus* |
|                       |           AK127880           |          BE943956             |
|                       |           BX648613           |          BE986786             |
+-----------------------+------------------------------+-------------------------------+
|      #   46           | *Seq. mapped in strand Plus* | *Seq. mapped in strand Plus*  |
|                       |           BC062440           |          CF622094             |
|                       |           BF677900           |          BY719817             |
|                       |           AV738635           |          CF172957             |
|                       |           BE245294           |          AA536940             |
|                       |           AK026107           |          AU080092             |
|                       |           BG547420           |          BQ257710             |
|                       |           BC033664           |          BY169048             |
|                       |           BP872323           |          BY144968             |
|                       |           CN370325           |          BY145656             |
|                       |           BM829441           |          BY323844             |
|                       |           BQ272109           |          BY032293             |
|                       |           BI087635           |          BY246481             |
|                       |           CN370327           |          AU036066             |
|                       |           BQ940181           |          BY280621             |
|                       |           BF530119           |          BY710686             |
|                       |           BM927324           |          AK011403             |
|                       |           AA353267           |          BE283324             |
|                       |           CD697244           |          BG919846             |
|                       |           BI822731           |          BE134788             |
|                       |           CN370324           |          BG245615             |
|                       |           BF680207           |          BF136825             |
|                       |           BQ686118           |          BM933791             |
|                       |           BX647116           |          BM939469             |
|                       |           CB142088           |          BQ257828             |
|                       |           CN370329           |          BY076037             |
|                       |           BM541387           |          BY061431             |
|                       |           AL713542           |          BY066314             |
|                       |           AA007519           |          BU518713             |
|                       |             H40373           |          AA137349             |
|                       |           CV379212           |          BY032023             |
|                       |           AA804520           |          BB611788             |
|                       |           CN370323           |          BY082899             |
|                       |           CN370322           |          BY293514             |
|                       |           CN370328           |          BY066670             |
|                       |           BI032980           |          BY065929             |
|                       |           BI032932           |          BY075762             |
|                       |           BI032937           |          BI904984             |
|                       |           AA312855           |          AA107954             |
|                       |           AA312557           |          AI956686             |
|                       |           AW958690           |          BY165174             |
|                       |             R26428           |          BY165101             |
|                       |           AW376216           |          CB571494             |
|                       |           AK094697           |          BY744629             |
|                       |           BM803417           |          BE572816             |
|                       |           AI114573           |          BQ258007             |
|                       |           BI049200           |          CF537159             |
|                       |           AA155958           |          AA108622             |
|                       |           CV389573           |          CD554176             |
|                       |           BF902177           |          CN686352             |
|                       |           BI019882           |          BI988805             |
|                       |           CD674722           |          BI111530             |
|                       |           BE646481           |          BG806380             |
|                       |           BG260998           |          BY307050             |
|                       |           BX506968           |          BU706913             |
|                       |           AI400786           |          CB272453             |
|                       |           BI862108           |          BF658281             |
|                       |           AA393814           |          BB657666             |
|                       |           BF985865           |          CD553079             |
|                       |           AI700569           |          BM935493             |
|                       |           BE466128           |          BE283523             |
|                       |           BF365430           |          CK346637             |
|                       |           AW594290           |          CF907627             |
|                       |           BE326684           |          CN667161             |
|                       |           AI356314           |          BY100600             |
|                       |           AI669806           |          CD562203             |
|                       |           BI496499           |          BF582048             |
|                       |           AI659257           |          BC010792             |
|                       |           BI496498           |          BE948915             |
|                       |           BG998370           |          AA796196             |
|                       |           AI247672           |          BC023749             |
|                       |           CV379224           |          BI687928             |
|                       |           AA155855           |          CA749682             |
|                       |           AI971146           |          BF120931             |
|                       |           AA007520           |          AW494381             |
|                       |           AA677603           |          AW491931             |
|                       |           AW014065           |          AW491918             |
|                       |           BX437746           |          CN671680             |
|                       |           BU955600           |          CF533579             |
|                       |             T07209           |          BF660288             |
|                       |           BX476296           |          AW490161             |
|                       |           AA677761           |          AW543125             |
|                       |           AW952724           |          BE135807             |
|                       |           BF844493           |          BB680440             |
|                       |           AA732593           |          BB686890             |
|                       |           AL707999           |          AW539001             |
|                       |           BG114047           |          BB662163             |
|                       |           CN284127           |          BY019177             |
|                       |           CV385694           |          AW457574             |
|                       |           AK125513           |          AA549720             |
|                       |           BF950322           |          BF018306             |
|                       |           BF804806           |          BY126356             |
|                       |           AW274331           |          AA413229             |
|                       |           AI053507           |          BY282926             |
|                       |           BG942295           |          AA414279             |
|                       |           AI053899           |          AV137186             |
|                       |           BG941348           |          BY385623             |
|                       |           BG940146           |          BG078048             |
|                       |           AW749350           |          BB697251             |
|                       |           AW949361           |          BY389818             |
|                       |           AW008457           |          BB704411             |
|                       |             C75219           |          CN718790             |
|                       |           AI282736           |          CN723939             |
|                       |           CF128640           |          CN716032             |
|                       |           BQ437829           |          CN716960             |
|                       |           AW023249           |          CN718428             |
|                       |           AW183617           |          CN724956             |
|                       |           AL833497           |          BY641947             |
|                       |           AI038854           |          AW260186             |
|                       |           AA621423           |          BY434164             |
|                       |           CN350709           |          AA672191             |
|                       |           BG940597           |          BY501662             |
|                       |           CN353240           |          AW492195             |
|                       |           AU139098           |          CK617104             |
|                       |           AI888048           |          BB824631             |
|                       |           BQ318860           |          BY498486             |
|                       |           AA513522           |          BY431341             |
|                       |           BX509819           |          BB025627             |
|                       |             N24122           |          BY495735             |
|                       |           AA826001           |          BY510023             |
|                       |           AA829191           |          BY441419             |
|                       |           AA361495           |          BB023035             |
|                       |           AA334984           |          AV120620             |
|                       |           AW867047           |          BB462073             |
|                       |           AW867051           |          BB024045             |
|                       |           BF215025           |          BY416193             |
|                       |           BU860265           |          BB022310             |
|                       |           AI538124           |          BI133611             |
|                       |           AV650187           |          CK336148             |
|                       |           BU676251           |          BB724311             |
|                       |           CB240775           |          BQ922028             |
|                       |             L40392           |          BY518483             |
|                       |           CN349398           |          BB071197             |
|                       |           AA355425           |          BP427496             |
|                       |           BF081349           |          AK038317             |
|                       |           AV746485           |          CN690110             |
|                       |           BQ427800           |          CF169402             |
|                       |           AU136749           |          CF897028             |
|                       |           AA356393           |          BG083505             |
|                       |           AA086093           |          CF166293             |
|                       |           AA358727           |          CA570244             |
|                       |           AW949672           |          CA572221             |
|                       |           BG613245           |          CA571898             |
|                       |           CN349397           |          CA573229             |
|                       |           AW818369           |          CN661745             |
|                       |           BM148785           |          CA570875             |
|                       |           BG542702           |          CK343096             |
|                       |           BX444126           |          CN661967             |
|                       |           AA910441           |          CN663382             |
|                       |           BU182519           |          CA545890             |
|                       |           BF763717           |          BB704692             |
|                       |           BE168338           |          BB701800             |
|                       |           CN350719           |          AA666700             |
|                       |           CN353241           |          BB672974             |
|                       |           AA361135           |          CK031097             |
|                       |           BF896130           |          AA530666             |
|                       |           BE835313           |          BX516712             |
|                       |           BQ277077           |          BB705952             |
|                       |           AL134475           |          CD547551             |
|                       |           AA345273           |          BC066150             |
|                       |             H21867           |          BC067400             |
|                       |           AI582588           |          CD560831             |
|                       |           AW195551           |          CF901829             |
|                       |           BE768554           |          AA726045             |
|                       |           BE702825           |          AA274725             |
|                       |           BE768665           |          BY710511             |
|                       |           BE175626           |          AK011184             |
|                       |           AA256785           |          AA624208             |
|                       |           AW003219           |          CK639153             |
|                       |           BF444977           |          BY745855             |
|                       |           BE881668           |          AK087936             |
|                       |           AI915534           |          AV359070             |
|                       |           AW271631           |          CB182571             |
|                       |           AW770747           |          CF913321             |
|                       |           CB242119           |          CN686543             |
|                       |           AU253206           |          BB614485             |
|                       |           AI193790           |          AK076553             |
|                       |           AI823371           |          CF907044             |
|                       |           BF448130           |          BQ951700             |
|                       |           CN349399           |          AA921137             |
|                       |           AI625554           |          BE653736             |
|                       |           CN349401           |          AA103179             |
|                       |           AW237192           |          CD363641             |
|                       |           AI803406           |          AA673539             |
|                       |           AW770442           |          CB317878             |
|                       |           BM784541           |          CD560258             |
|                       |           BE768656           |          BI693882             |
|                       |           CN353242           |          BU057786             |
|                       |           AA947974           |          AA638041             |
|                       |           AI917737           |          AA545551             |
|                       |           BF433914           |          AU255127             |
|                       |           AA307878           |          BG082591             |
|                       |           BU680031           |          CD354695             |
|                       |           BE768542           |          AA509969             |
|                       |           BX402400           |          AA396911             |
|                       |           AL546433           |          AU019831             |
|                       |           CR608971           |          BG069577             |
|                       |                              |          BX518479             |
|                       |                              |          CA527823             |
|                       |                              |          BG277800             |
|                       |                              |          BQ177712             |
|                       |                              |          BI872644             |
|                       |                              |          AI848222             |
|                       |                              |          AU019484             |
|                       |                              |          BE534952             |
|                       |                              |          BE632728             |
|                       |                              |          BG228787             |
|                       |                              |          CO043427             |
|                       |                              |          AA286536             |
|                       |                              |          BI690736             |
|                       |                              |          AI663432             |
|                       |                              |          BG070676             |
|                       |                              |          CD560346             |
|                       |                              |          CB317418             |
|                       |                              |          BQ032623             |
|                       |                              |          CD771914             |
|                       |                              |          AA250262             |
|                       |                              |          BI151471             |
|                       |                              |          AI325919             |
|                       |                              |          BE633122             |
|                       |                              |          AA389276             |
|                       |                              |          AA561326             |
|                       |                              |          AA596517             |
|                       | *Seq. mapped in strand Minus*| *Seq. mapped in strand Minus* |
|                       |           AI269019           |          AW121577             |
|                       |           BU741154           |          AI851193             |
|                       |           CR739627           |          BE333456             |
|                       |           AA458949           |          AA422700             |
|                       |           BF514639           |          CF356041             |
|                       |           CB854244           |          CF805379             |
|                       |           AL571895           |          AK090334             |
|                       |                              |          CF928436             |
|                       |                              |          CF380824             |
|                       |                              |          BX524860             |
|                       |                              |          BC022607             |
|                       |                              |          CO043975             |
|                       |                              |          BC050015             |
|                       |                              |          BE655502             |
|                       |                              |          BX634855             |
|                       |                              |          AI789529             |
|                       |                              |          BM214341             |
|                       |                              |          BY685787             |
|                       |                              |          BE944485             |
|                       |                              |          CF616574             |
|                       |                              |          BY092105             |
|                       |                              |          CD808098             |
|                       |                              |          AW743376             |
|                       |                              |          BU614202             |
|                       |                              |          BQ828952             |
|                       |                              |          BU937347             |
|                       |                              |          CA316630             |
|                       |                              |          CF725445             |
|                       |                              |          AA144972             |
|                       |                              |          BI248750             |
|                       |                              |          CF734251             |
|                       |                              |          AA497799             |
|                       |                              |          BU707015             |
|                       |                              |          CF162944             |
|                       |                              |          BB621855             |
|                       |                              |          CF154036             |
|                       |                              |          CA554100             |
|                       |                              |          CN459954             |
|                       |                              |          CA554287             |
|                       |                              |          CA551838             |
|                       |                              |          CF160661             |
|                       |                              |          CA551818             |
|                       |                              |          CA554208             |
|                       |                              |          CF160368             |
|                       |                              |          CF155529             |
|                       |                              |          CF159260             |
|                       |                              |          CF160757             |
|                       |                              |          BQ559875             |
|                       |                              |          BY017696             |
|                       |                              |          CF159919             |
|                       |                              |          CA551174             |
|                       |                              |          CF156283             |
|                       |                              |          CF159596             |
|                       |                              |          CF159487             |
|                       |                              |          CA552385             |
|                       |                              |          CK333310             |
|                       |                              |          BQ571543             |
|                       |                              |          CB526501             |
|                       |                              |          CA328773             |
|                       |                              |          BU613875             |
|                       |                              |          BM939983             |
|                       |                              |          CD348263             |
|                       |                              |          BU702124             |
|                       |                              |          BB640722             |
|                       |                              |          BY725919             |
|                       |                              |          CF902713             |
|                       |                              |          CF167930             |
|                       |                              |          CF165343             |
|                       |                              |          CA877464             |
|                       |                              |          BB659426             |
|                       |                              |          BY004781             |
|                       |                              |          BY256667             |
|                       |                              |          BY733589             |
|                       |                              |          CN692621             |
|                       |                              |          CF165708             |
|                       |                              |          BB853628             |
|                       |                              |          CN693577             |
|                       |                              |          BY267123             |
|                       |                              |          BY277097             |
|                       |                              |          BY256105             |
|                       |                              |          BY017713             |
|                       |                              |          BY024639             |
|                       |                              |          BY012911             |
|                       |                              |          BB871707             |
|                       |                              |          BY770925             |
|                       |                              |          BY325310             |
|                       |                              |          BY135559             |
|                       |                              |          BY185660             |
|                       |                              |          BY325474             |
|                       |                              |          BY008346             |
|                       |                              |          BY073566             |
|                       |                              |          CF902241             |
|                       |                              |          CN691996             |
|                       |                              |                               |
|                       |                              |                               |
|                       |                              |                               |
|                       |                              |                               |
|                       |                              |                               |
|                       |                              |                               |
|                       |                              |                               |
|                       |                              |                               |
|                       |                              |                               |
|                       |                              |                               |
|                       |                              |                               |
|                       |                              |                               |
|                       |                              |                               |
|                       |                              |                               |
|                       |                              |                               |
|                       |                              |                               |
|                       |                              |                               |
|                       |                              |                               |
|                       |                              |                               |
|                       |                              |                               |
|                       |                              |                               |
|                       |                              |                               |
|                       |                              |                               |
|                       |                              |                               |
|                       |                              |                               |
|                       |                              |                               |
|                       |                              |                               |
|                       |                              |                               |
|                       |                              |                               |
|                       |                              |                               |
|                       |                              |                               |
|                       |                              |                               |
|                       |                              |                               |
|                       |                              |                               |
|                       |                              |                               |
|                       |                              |                               |
|                       |                              |                               |
|                       |                              |                               |
|                       |                              |                               |
|                       |                              |                               |
|                       |                              |                               |
|                       |                              |                               |
|                       |                              |                               |
|                       |                              |                               |
|                       |                              |                               |
|                       |                              |                               |
|                       |                              |                               |
|                       |                              |                               |
|                       |                              |                               |
|                       |                              |                               |
|                       |                              |                               |
|                       |                              |                               |
|                       |                              |                               |
|                       |                              |                               |
|                       |                              |                               |
|                       |                              |                               |
|                       |                              |                               |
|                       |                              |                               |
|                       |                              |                               |
|                       |                              |                               |
|                       |                              |                               |
|                       |                              |                               |
|                       |                              |                               |
|                       |                              |                               |
|                       |                              |                               |
|                       |                              |                               |
|                       |                              |                               |
|                       |                              |                               |
|                       |                              |                               |
|                       |                              |                               |
|                       |                              |                               |
|                       |                              |                               |
|                       |                              |                               |
|                       |                              |                               |
|                       |                              |                               |
|                       |                              |                               |
|                       |                              |                               |
|                       |                              |                               |
|                       |                              |                               |
|                       |                              |                               |
|                       |                              |                               |
|                       |                              |                               |
|                       |                              |                               |
|                       |                              |                               |
|                       |                              |                               |
|                       |                              |                               |
|                       |                              |                               |
|                       |                              |                               |
|                       |                              |                               |
|                       |                              |                               |
|                       |                              |                               |
|                       |                              |                               |
|                       |                              |                               |
|                       |                              |                               |
|                       |                              |                               |
|                       |                              |                               |
|                       |                              |                               |
|                       |                              |                               |
|                       |                              |                               |
|                       |                              |                               |
|                       |                              |                               |
|                       |                              |                               |
|                       |                              |                               |
|                       |                              |                               |
|                       |                              |                               |
|                       |                              |                               |
|                       |                              |                               |
|                       |                              |                               |
|                       |                              |                               |
|                       |                              |                               |
|                       |                              |                               |
|                       |                              |                               |
|                       |                              |                               |
|                       |                              |                               |
|                       |                              |                               |
|                       |                              |                               |
|                       |                              |                               |
|                       |                              |                               |
|                       |                              |                               |
|                       |                              |                               |
|                       |                              |                               |
|                       |                              |                               |
|                       |                              |                               |
|                       |                              |                               |
|                       |                              |                               |
|                       |                              |                               |
|                       |                              |                               |
|                       |                              |                               |
|                       |                              |                               |
+-----------------------+------------------------------+-------------------------------+
|      #   47           | *Seq. mapped in strand Plus* | *Seq. mapped in strand Plus*  |
|                       |           BM968097           |          BC025426             |
|                       |           AI582604           |                               |
|                       |           BM724317           |                               |
|                       | *Seq. mapped in strand Minus*| *Seq. mapped in strand Minus* |
|                       |           BM978733           |          AB059357             |
|                       |           AI758416           |          AB059358             |
|                       |           AA828013           |          BC061694             |
|                       |           BU609115           |          BC058776             |
|                       |           AA804214           |          BC029664             |
|                       |           AA814077           |          BC046992             |
|                       |           BU680244           |          AK044682             |
|                       |           BQ020841           |          AK044600             |
|                       |           BQ021165           |          BC046828             |
|                       |           BM991676           |          AK014320             |
|                       |           BU741596           |          AK029858             |
|                       |           BU681518           |          BC031479             |
|                       |           BU615421           |                               |
|                       |           AI379546           |                               |
|                       |           AB046783           |                               |
|                       |           AB053305           |                               |
|                       |           AF391100           |                               |
|                       |           AA621390           |                               |
|                       |           BU187012           |                               |
|                       |           AK056413           |                               |
|                       |           BM544824           |                               |
|                       |           BG539215           |                               |
|                       |           BP210670           |                               |
|                       |           BP362192           |                               |
|                       |           CF552638           |                               |
|                       |             N39805           |                               |
|                       |           BQ219856           |                               |
|                       |           BU164048           |                               |
|                       |           BF327498           |                               |
|                       |           BX643522           |                               |
|                       |           AW905587           |                               |
|                       |           BE003282           |                               |
|                       |           BC071576           |                               |
|                       |           BE535882           |                               |
|                       |           CN359894           |                               |
|                       |           BQ437730           |                               |
|                       |             H68935           |                               |
|                       |           AW867853           |                               |
|                       |             N20939           |                               |
|                       |           AI337242           |                               |
|                       |           AI224489           |                               |
|                       |             F27732           |                               |
|                       |             F37358           |                               |
|                       |             N27808           |                               |
|                       |           BC029174           |                               |
|                       |           BG426436           |                               |
|                       |           BM453202           |                               |
|                       |           BE147975           |                               |
|                       |           BQ328901           |                               |
|                       |           CN359891           |                               |
|                       |           BE082284           |                               |
|                       |           BM971232           |                               |
|                       |           BM983458           |                               |
|                       |           AI273643           |                               |
|                       |           BU623061           |                               |
|                       |           AK023024           |                               |
|                       |           AB053306           |                               |
|                       |           BQ425128           |                               |
|                       |           AU125528           |                               |
|                       |           CD558034           |                               |
|                       |           BE006110           |                               |
|                       |           CD101681           |                               |
|                       |           BE006111           |                               |
|                       |           CV023135           |                               |
|                       |           AL706095           |                               |
|                       |           BM560426           |                               |
|                       |           AL603120           |                               |
|                       |           AU129510           |                               |
|                       |           BP349944           |                               |
|                       |           BP245399           |                               |
|                       |           CN359893           |                               |
|                       |           CD700870           |                               |
|                       |           BI760406           |                               |
|                       |           BP350941           |                               |
|                       |           BP348546           |                               |
|                       |           BU659454           |                               |
|                       |           AL710614           |                               |
+-----------------------+------------------------------+-------------------------------+
|      #   48           | *Seq. mapped in strand Plus* | *Seq. mapped in strand Plus*  |
|                       |           CR592336           |          AK027956             |
|                       |           BC080193           |                               |
|                       |             X03363           |                               |
|                       |             M11730           |                               |
|                       |           AF177761           |                               |
|                       |             S57296           |                               |
|                       |           CR591377           |                               |
|                       | *Seq. mapped in strand Minus*| *Seq. mapped in strand Minus* |
|                       |           BC063395           |          AK041314             |
|                       |           BC006006           |          BC021589             |
|                       |           CR613484           |          AK009922             |
|                       |           AY508814           |          AK007795             |
|                       |           AF490253           |          AK002959             |
+-----------------------+------------------------------+-------------------------------+
|      #   49           | *Seq. mapped in strand Plus* | *Seq. mapped in strand Plus*  |
|                       |           AK055855           |          AK020131             |
|                       |           BC010920           |          BC021770             |
|                       |             U89916           |          BC029019             |
|                       |           CR456845           |          AF124425             |
|                       |           AF339794           |          BC012238             |
|                       | *Seq. mapped in strand Minus*| *Seq. mapped in strand Minus* |
|                       |           AL049931           |          AK085147             |
|                       |           AK094042           |          BC060051             |
|                       |           AB023213           |          BC051931             |
|                       |           BC041804           |          AK032834             |
|                       |           CR606837           |          AK129256             |
|                       |           AF272347           |          AK013136             |
|                       |           BC018744           |          BC037016             |
|                       |           CR621496           |          AK011093             |
|                       |           BC011450           |          AK010997             |
|                       |           BC024972           |                               |
|                       |           AK130513           |                               |
|                       |           CR613825           |                               |
|                       |           AF339791           |                               |
|                       |           AK092363           |                               |
+-----------------------+------------------------------+-------------------------------+
|      #   50           | *Seq. mapped in strand Plus* | *Seq. mapped in strand Plus*  |
|                       |           BC052266           |          BC055727             |
|                       |           BM970097           |          AK048420             |
|                       |           AI983696           |          AF206321             |
|                       |           CA432236           |          AF139183             |
|                       |           BU681711           |          AF206322             |
|                       |           BU624031           |          AF363723             |
|                       |           BQ021138           |          BQ174975             |
|                       |           BM666204           |          AW123618             |
|                       |           CA429523           |          AW123440             |
|                       |           BF512236           |          BF017527             |
|                       |           AW245391           |          CF584096             |
|                       |           AW247931           |          BE952291             |
|                       |           AI917950           |          AA217545             |
|                       |                              |          BI437964             |
|                       |                              |          BI794236             |
|                       |                              |          CB847667             |
|                       |                              |          CB847934             |
|                       |                              |          BC049774             |
|                       | *Seq. mapped in strand Minus*| *Seq. mapped in strand Minus* |
|                       |           CN484180           |          BF468006             |
|                       |                              |          BE949435             |
|                       |                              |                               |
|                       |                              |                               |
|                       |                              |                               |
|                       |                              |                               |
|                       |                              |                               |
|                       |                              |                               |
|                       |                              |                               |
|                       |                              |                               |
|                       |                              |                               |
|                       |                              |                               |
|                       |                              |                               |
|                       |                              |                               |
|                       |                              |                               |
|                       |                              |                               |
|                       |                              |                               |
|                       |                              |                               |
+-----------------------+------------------------------+-------------------------------+
|      #   51           | *Seq. mapped in strand Plus* | *Seq. mapped in strand Plus*  |
|                       |           AA829745           |          AW490472             |
|                       |           BM968807           |          BE995329             |
|                       |           BU676411           |          AW124799             |
|                       |                              |          BE996533             |
|                       | *Seq. mapped in strand Minus*| *Seq. mapped in strand Minus* |
|                       |           AI983444           |          AK029740             |
|                       |           AW874515           |          AK017687             |
|                       |           BM978864           |          BE691656             |
|                       |           BQ773806           |          BB637332             |
|                       |           BM021051           |          AK017645             |
|                       |           BM021275           |          BE946352             |
|                       |           CK904194           |                               |
|                       |           CK904193           |                               |
|                       |           AI334711           |                               |
|                       |           BX103988           |                               |
|                       |           AI640674           |                               |
|                       |           AW316559           |                               |
|                       |           AW316575           |                               |
|                       |           AW769262           |                               |
|                       |           AW296050           |                               |
|                       |           AL832015           |                               |
|                       |           AA864721           |                               |
|                       |           AI818265           |                               |
|                       |           BU679680           |                               |
|                       |           AL596840           |                               |
|                       |           AK027020           |                               |
|                       |           AY359880           |                               |
|                       |           AL832458           |                               |
|                       |           BM969179           |                               |
|                       |           CN334449           |                               |
|                       |           BQ013543           |                               |
|                       |           AK001670           |                               |
|                       |           BM454564           |                               |
|                       |           BI756711           |                               |
|                       |           BI755801           |                               |
|                       |           AA525173           |                               |
|                       |           AK094164           |                               |
|                       |           AA490969           |                               |
|                       |           AA449298           |                               |
|                       |           AW165998           |                               |
|                       |           AW449800           |                               |
|                       |           BU754302           |                               |
|                       |           AI342190           |                               |
|                       |           BE883104           |                               |
|                       |           BP379285           |                               |
|                       |           AA000974           |                               |
|                       |           AW368197           |                               |
|                       |           AA035249           |                               |
|                       |           AB014773           |                               |
|                       |           BE218143           |                               |
|                       |           AU185690           |                               |
|                       |           BP241573           |                               |
|                       |           BP312540           |                               |
|                       |           AI693282           |                               |
|                       |           BI025471           |                               |
|                       |           AA280252           |                               |
|                       |           BI222554           |                               |
|                       |           CN334450           |                               |
|                       |           AU132918           |                               |
|                       |           CN334444           |                               |
|                       |           BP260001           |                               |
|                       |           CF138132           |                               |
|                       |           AI936442           |                               |
|                       |           BF832810           |                               |
|                       |           BQ012469           |                               |
|                       |           BX390701           |                               |
|                       |           BM460152           |                               |
|                       |           AA491162           |                               |
|                       |           CN334445           |                               |
|                       |           CN295249           |                               |
|                       |           CN295250           |                               |
|                       |           BC031637           |                               |
|                       |           CR624797           |                               |
|                       |           AK094969           |                               |
|                       |           AI908934           |                               |
|                       |           BX384287           |                               |
|                       |           CD643592           |                               |
|                       |           CF994156           |                               |
|                       |           CN334451           |                               |
|                       |           BI829411           |                               |
|                       |           BX481775           |                               |
|                       |           AL704425           |                               |
|                       |           CN334447           |                               |
|                       |           BX490004           |                               |
|                       |           BP284278           |                               |
|                       |           AL710245           |                               |
|                       |           AL596839           |                               |
|                       |           BP348515           |                               |
|                       |           AL699427           |                               |
|                       |           AL710107           |                               |
|                       |           CV030220           |                               |
|                       |           AL699392           |                               |
|                       |           CN334448           |                               |
|                       |           BX474365           |                               |
|                       |           BX954138           |                               |
|                       |           BE005393           |                               |
|                       |           BF335012           |                               |
|                       |           AL710087           |                               |
|                       |             D61636           |                               |
|                       |           CD629068           |                               |
|                       |           CD629070           |                               |
|                       |           CD629072           |                               |
|                       |           CD629073           |                               |
|                       |           CD629075           |                               |
|                       |           CD629069           |                               |
|                       |           CD629071           |                               |
|                       |           CD629074           |                               |
+-----------------------+------------------------------+-------------------------------+
|      #   52           | *Seq. mapped in strand Plus* | *Seq. mapped in strand Plus*  |
|                       |           BU191740           |          BX515175             |
|                       |           BX363351           |          AI117064             |
|                       |           AU132423           |                               |
|                       |           AF054996           |                               |
|                       |           BQ689108           |                               |
|                       |           CD673192           |                               |
|                       |           AW405608           |                               |
|                       |           BM808781           |                               |
|                       |           CB142892           |                               |
|                       |           BE397486           |                               |
|                       |           BE562415           |                               |
|                       |           BE397405           |                               |
|                       |           BI227388           |                               |
|                       |           BU597553           |                               |
|                       |           BP325651           |                               |
|                       |           BE397547           |                               |
|                       |           BE397741           |                               |
|                       |           BG342008           |                               |
|                       |           BG912768           |                               |
|                       |           BG755681           |                               |
|                       |           BG754365           |                               |
|                       |           BG342185           |                               |
|                       |           BG340516           |                               |
|                       |           BG759316           |                               |
|                       |           BG753952           |                               |
|                       |           BG718039           |                               |
|                       |           CN344968           |                               |
|                       |           BG720143           |                               |
|                       |           BM747882           |                               |
|                       |           BM758126           |                               |
|                       |           BM758629           |                               |
|                       |           BM739604           |                               |
|                       |           BG390654           |                               |
|                       |           BM479134           |                               |
|                       |           CB994401           |                               |
|                       |           CB991102           |                               |
|                       |           BG674604           |                               |
|                       |           AL560484           |                               |
|                       |           BG035475           |                               |
|                       |           BF129163           |                               |
|                       |           CD673515           |                               |
|                       |           BE791672           |                               |
|                       |           BU595409           |                               |
|                       |           BX335021           |                               |
|                       |           CR593468           |                               |
|                       |           CN344964           |                               |
|                       |           BE278488           |                               |
|                       |           BI113766           |                               |
|                       |           BF309685           |                               |
|                       |           BF348003           |                               |
|                       |           BP373430           |                               |
|                       |           BG027095           |                               |
|                       |           BG757064           |                               |
|                       |           BG757680           |                               |
|                       |           BG392665           |                               |
|                       |           BI830473           |                               |
|                       |           BX406830           |                               |
|                       |           BU147396           |                               |
|                       |           BM834494           |                               |
|                       |           BP247421           |                               |
|                       |           BP247486           |                               |
|                       |           BM787472           |                               |
|                       |           CB267787           |                               |
|                       |           BE548923           |                               |
|                       |           CN344970           |                               |
|                       |           BG403641           |                               |
|                       |           BG422156           |                               |
|                       |           CA488164           |                               |
|                       |           AL701546           |                               |
|                       |           BQ651813           |                               |
|                       |           BG401371           |                               |
|                       |           CN344965           |                               |
|                       |           BF314766           |                               |
|                       |           BE241759           |                               |
|                       |           BE258636           |                               |
|                       |           BX479063           |                               |
|                       |           BE253791           |                               |
|                       |           BF984021           |                               |
|                       |           BM688862           |                               |
|                       |           BM835077           |                               |
|                       |           BG755174           |                               |
|                       |           BE251788           |                               |
|                       |           BF337951           |                               |
|                       |           BI857539           |                               |
|                       |           BE252819           |                               |
|                       |           BF525851           |                               |
|                       |           BI600709           |                               |
|                       |           BG708732           |                               |
|                       |           CN344966           |                               |
|                       |           BX483786           |                               |
|                       |           BQ219314           |                               |
|                       |           CN344969           |                               |
|                       |           BM809178           |                               |
|                       |           BM809304           |                               |
|                       |           BP348032           |                               |
|                       |           BX280090           |                               |
|                       |           AA132814           |                               |
|                       |           BM469249           |                               |
|                       |           BM927429           |                               |
|                       |           BC010042           |                               |
|                       |           BE257588           |                               |
|                       |           BX341446           |                               |
|                       |           BP311376           |                               |
|                       |           BP198934           |                               |
|                       |           BP208149           |                               |
|                       |           BG436654           |                               |
|                       |           BM714597           |                               |
|                       |           BG479929           |                               |
|                       |           BG438060           |                               |
|                       |           BG774267           |                               |
|                       |           BM804238           |                               |
|                       |           BI832858           |                               |
|                       |           BQ689899           |                               |
|                       |           BQ888500           |                               |
|                       |           BU145963           |                               |
|                       |           BI761694           |                               |
|                       |           BM853479           |                               |
|                       |           BQ440045           |                               |
|                       |           CB156743           |                               |
|                       |           BG818229           |                               |
|                       |           BG471111           |                               |
|                       |           BU150523           |                               |
|                       |           BG281614           |                               |
|                       |           BG767879           |                               |
|                       |           BU860161           |                               |
|                       |           BU846227           |                               |
|                       |           BG165747           |                               |
|                       |           BU854939           |                               |
|                       |           BQ052779           |                               |
|                       |           BG764996           |                               |
|                       |           BG424638           |                               |
|                       |           BG419908           |                               |
|                       |           BI115134           |                               |
|                       |           BM702393           |                               |
|                       |           BM048674           |                               |
|                       |           BG679217           |                               |
|                       |           BX444433           |                               |
|                       |           CR619192           |                               |
|                       |           BU541061           |                               |
|                       |           CN344962           |                               |
|                       |           BM826038           |                               |
|                       |           AA232604           |                               |
|                       |           BG031396           |                               |
|                       |           BF304787           |                               |
|                       |           BG419085           |                               |
|                       |           BU501998           |                               |
|                       |           AW407811           |                               |
|                       |           BE259883           |                               |
|                       |           BE296014           |                               |
|                       |           BQ889461           |                               |
|                       |           BQ957386           |                               |
|                       |           BQ706523           |                               |
|                       |           BE271537           |                               |
|                       |           BI194578           |                               |
|                       |           BE409847           |                               |
|                       |           BE275343           |                               |
|                       |           CF128524           |                               |
|                       |           BQ889555           |                               |
|                       |           AL525049           |                               |
|                       |           BG832008           |                               |
|                       |           BQ422049           |                               |
|                       |           BG479246           |                               |
|                       |           AU125480           |                               |
|                       |           BX335020           |                               |
|                       |           BE792269           |                               |
|                       |           BE258591           |                               |
|                       |           BE396959           |                               |
|                       |           BE265937           |                               |
|                       |           BE791828           |                               |
|                       |           BE254936           |                               |
|                       |           AI205177           |                               |
|                       |           BE271250           |                               |
|                       |           BF212671           |                               |
|                       |           BE271263           |                               |
|                       |           BG421498           |                               |
|                       |           BI825647           |                               |
|                       |           BX453752           |                               |
|                       |           CN344971           |                               |
|                       |           AA459148           |                               |
|                       |             W44466           |                               |
|                       |           BI546367           |                               |
|                       |           BI546366           |                               |
|                       |           AA282150           |                               |
|                       |           AA207136           |                               |
|                       |           BG823474           |                               |
|                       |           BM044766           |                               |
|                       |           BM913611           |                               |
|                       |             H61261           |                               |
|                       |           BI859783           |                               |
|                       |           BI046062           |                               |
|                       |           BE173810           |                               |
|                       |           BI045799           |                               |
|                       |           BI046348           |                               |
|                       |           AA338109           |                               |
|                       |           BQ707565           |                               |
|                       |           BQ708702           |                               |
|                       |           BG955063           |                               |
|                       |           AW374430           |                               |
|                       |           BG121034           |                               |
|                       |           BG468428           |                               |
|                       |           BG823852           |                               |
|                       |           BQ945890           |                               |
|                       |           AA307895           |                               |
|                       |           CN398887           |                               |
|                       |           BP378627           |                               |
|                       |           BM675361           |                               |
|                       |           BU629484           |                               |
|                       |           BU631175           |                               |
|                       |           BM975099           |                               |
|                       |           BE908885           |                               |
|                       |           BF529134           |                               |
|                       |           BG112236           |                               |
|                       |           CF264883           |                               |
|                       |           CF272534           |                               |
|                       |           BG392569           |                               |
|                       |           AW971441           |                               |
|                       |             W01368           |                               |
|                       |           AI345947           |                               |
|                       |           CD370186           |                               |
|                       |           AI149522           |                               |
|                       |           AW605059           |                               |
|                       |           AW580608           |                               |
|                       |           CF552770           |                               |
|                       |           BE819649           |                               |
|                       |           BE819651           |                               |
|                       |           BE819653           |                               |
|                       |           BE018754           |                               |
|                       |           BU683153           |                               |
|                       |           BM742331           |                               |
|                       |           BM742800           |                               |
|                       |           BM744454           |                               |
|                       |           BM746675           |                               |
|                       |           BU597707           |                               |
|                       |           AW834735           |                               |
|                       |           BQ021802           |                               |
|                       |           BX385862           |                               |
|                       |           BX396042           |                               |
|                       |           CR621399           |                               |
|                       |           BU729172           |                               |
|                       |           BG677213           |                               |
|                       |           BE833048           |                               |
|                       |           BP213447           |                               |
|                       |           AW575367           |                               |
|                       |           AW362883           |                               |
|                       |           AA777461           |                               |
|                       |           AW362879           |                               |
|                       |           AI566310           |                               |
|                       |           AI284212           |                               |
|                       |           BG120719           |                               |
|                       |           AA458932           |                               |
|                       |           BG747960           |                               |
|                       |           BG254937           |                               |
|                       |           BU941409           |                               |
|                       |           AI708753           |                               |
|                       |           BU161645           |                               |
|                       |           BM802623           |                               |
|                       |           AA995863           |                               |
|                       |           BU727329           |                               |
|                       |           AW630966           |                               |
|                       |           AI523406           |                               |
|                       |           AW468726           |                               |
|                       |           AA149891           |                               |
|                       |             H61265           |                               |
|                       |             W44427           |                               |
|                       |             N78623           |                               |
|                       |           AA857236           |                               |
|                       |           BM794988           |                               |
|                       |           AA132703           |                               |
|                       |           AI339634           |                               |
|                       |           BM452129           |                               |
|                       |           BM452149           |                               |
|                       |             F36688           |                               |
|                       |           AA995162           |                               |
|                       |           AA282151           |                               |
|                       |           AA743533           |                               |
|                       |           AA767301           |                               |
|                       |           AA632328           |                               |
|                       |           BG170568           |                               |
|                       |           BG180074           |                               |
|                       |             F35800           |                               |
|                       |           BI013029           |                               |
|                       |             F26803           |                               |
|                       | *Seq. mapped in strand Minus*| *Seq. mapped in strand Minus* |
|                       |           AA831631           |          AI853654             |
|                       |           BQ575029           |          BQ031615             |
|                       |           BE048574           |          BG230083             |
|                       |           AI083990           |          AK010002             |
|                       |           CK301040           |          BC019430             |
|                       |           AA148512           |          BG243363             |
|                       |           CB529756           |          BE863324             |
|                       |           BC006429           |          AA718297             |
|                       |           AK054693           |          BI112530             |
|                       |           CA420264           |          BG518616             |
|                       |           CA748482           |          BE284125             |
|                       |           AW243192           |                               |
|                       |           AA838429           |                               |
|                       |           BM678257           |                               |
|                       |           BQ772465           |                               |
|                       |           CD365943           |                               |
|                       |           CD368555           |                               |
|                       |           AK092196           |                               |
|                       |           AK124405           |                               |
|                       |           BM998748           |                               |
|                       |           CR591728           |                               |
|                       |           BX382040           |                               |
|                       |           CR626420           |                               |
|                       |           CR618993           |                               |
|                       |           BU622818           |                               |
|                       |           BU727025           |                               |
|                       |           AW207675           |                               |
|                       |           AW162612           |                               |
|                       |           CB852646           |                               |
|                       |           BM973036           |                               |
|                       |           AW583996           |                               |
|                       |           AI926821           |                               |
|                       |           AI624659           |                               |
|                       |           AW190370           |                               |
|                       |           AI431528           |                               |
|                       |           AI934360           |                               |
|                       |           AL571677           |                               |
|                       |           CR603465           |                               |
|                       |           BX379485           |                               |
|                       |           CR621252           |                               |
|                       |           BG743358           |                               |
|                       |           BG685360           |                               |
|                       |           CR600871           |                               |
|                       |           BM015598           |                               |
|                       |           BX382041           |                               |
|                       |           BI603080           |                               |
|                       |           BG117899           |                               |
|                       |           BM906263           |                               |
|                       |           BM547699           |                               |
|                       |           AL546280           |                               |
|                       |           BE385697           |                               |
|                       |           BG473304           |                               |
|                       |           BG329299           |                               |
|                       |           BM451734           |                               |
|                       |           BE736069           |                               |
|                       |           BM550253           |                               |
|                       |           BQ065852           |                               |
|                       |           BQ060845           |                               |
|                       |           BQ063054           |                               |
|                       |           BQ070633           |                               |
|                       |           BG329419           |                               |
|                       |           BM542307           |                               |
|                       |           BG332706           |                               |
|                       |           BM017754           |                               |
|                       |           BI855842           |                               |
|                       |           BQ064393           |                               |
|                       |           BI521276           |                               |
|                       |           BU183165           |                               |
|                       |           BI909636           |                               |
|                       |           BU180767           |                               |
|                       |           BU557724           |                               |
|                       |           AL557449           |                               |
|                       |           BG472993           |                               |
|                       |           BQ056584           |                               |
|                       |           BI114214           |                               |
|                       |           BE737014           |                               |
|                       |           BU526984           |                               |
|                       |           BI915411           |                               |
|                       |           BQ653552           |                               |
|                       |           BG539139           |                               |
|                       |           BQ678148           |                               |
|                       |           BF974284           |                               |
|                       |           BG697222           |                               |
|                       |           BQ887087           |                               |
|                       |           BM920060           |                               |
|                       |           BX379486           |                               |
|                       |           BQ059524           |                               |
|                       |           BG740756           |                               |
|                       |           BG437306           |                               |
|                       |           BQ059150           |                               |
|                       |           BF967521           |                               |
|                       |           BI910807           |                               |
|                       |           BG823638           |                               |
|                       |           BQ641918           |                               |
|                       |           BG329121           |                               |
|                       |           BQ928504           |                               |
|                       |           BQ692021           |                               |
|                       |           BE894629           |                               |
|                       |           BX338896           |                               |
|                       |           BE251985           |                               |
|                       |           BG700889           |                               |
|                       |           BG179982           |                               |
|                       |           BI553860           |                               |
|                       |           BI823189           |                               |
|                       |           BQ684268           |                               |
|                       |           BG401574           |                               |
|                       |           CN261635           |                               |
|                       |           BI668694           |                               |
|                       |           BI907738           |                               |
|                       |           AL558958           |                               |
|                       |           BG429563           |                               |
|                       |           CN261637           |                               |
|                       |           BE895053           |                               |
|                       |           BE255874           |                               |
|                       |           CB989952           |                               |
|                       |           BI770977           |                               |
|                       |           BU149458           |                               |
|                       |           BG398513           |                               |
|                       |           BI766381           |                               |
|                       |           BE255658           |                               |
|                       |           BI912234           |                               |
|                       |           BE735533           |                               |
|                       |           BF212966           |                               |
|                       |           BQ897148           |                               |
|                       |           BP308526           |                               |
|                       |           BG035636           |                               |
|                       |           AW160427           |                               |
|                       |           BX386575           |                               |
|                       |           BP309078           |                               |
|                       |           BQ639247           |                               |
|                       |           CN261636           |                               |
|                       |           BI837379           |                               |
|                       |           BE254306           |                               |
|                       |           BP297818           |                               |
|                       |           BP340651           |                               |
|                       |           BP297331           |                               |
|                       |           BP301843           |                               |
|                       |           BP371572           |                               |
|                       |           BP299468           |                               |
|                       |           BP297541           |                               |
|                       |             H10707           |                               |
|                       |           CV026934           |                               |
|                       |           BP213735           |                               |
|                       |           BI765748           |                               |
|                       |           BI914506           |                               |
|                       |           AI879351           |                               |
|                       |             W38853           |                               |
|                       |           CD674957           |                               |
|                       |           BI600053           |                               |
|                       |             Z44536           |                               |
|                       |           BE827371           |                               |
|                       |           BI919206           |                               |
|                       |           BM352938           |                               |
|                       |           BP212225           |                               |
|                       |           AW161045           |                               |
|                       |           AW582996           |                               |
|                       |           BI913214           |                               |
|                       |           CB134484           |                               |
|                       |           BI910277           |                               |
|                       |           BX473378           |                               |
|                       |           BP366642           |                               |
|                       |           BF882590           |                               |
|                       |           BF897735           |                               |
|                       |           AW630237           |                               |
|                       |           BF928500           |                               |
|                       |           BI915687           |                               |
|                       |           AL702513           |                               |
|                       |           CB270212           |                               |
|                       |           BM688639           |                               |
|                       |             R34406           |                               |
|                       |           BQ676446           |                               |
|                       |           BU558162           |                               |
|                       |           BQ640293           |                               |
|                       |             Z44243           |                               |
|                       |           AA323634           |                               |
|                       |           AA341658           |                               |
|                       |           AL702680           |                               |
+-----------------------+------------------------------+-------------------------------+
|      #   53           | *Seq. mapped in strand Plus* | *Seq. mapped in strand Plus*  |
|                       |           AF132747           |          BY329098             |
|                       |           AF159063           |          BY266045             |
|                       |           AF255952           |          BY274522             |
|                       |           BC047932           |          BY281408             |
|                       |           AF112215           |          BF123025             |
|                       |           AF155740           |          BY753296             |
|                       |           AK000200           |          CK617189             |
|                       |           AL133634           |          BB864372             |
|                       |           AK025850           |          BY285459             |
|                       |           BC013021           |          BY093906             |
|                       |           CR603988           |          BY263818             |
|                       |           BC035121           |          BY264940             |
|                       |                              |          BY241125             |
|                       |                              |          BY252028             |
|                       |                              |          BY240443             |
|                       |                              |          BY240214             |
|                       |                              |          BB843479             |
|                       |                              |          AK047821             |
|                       |                              |          BY114844             |
|                       |                              |          BY122709             |
|                       |                              |          BY771380             |
|                       |                              |          BY113197             |
|                       |                              |          BY116530             |
|                       |                              |          BY324571             |
|                       |                              |          BY193770             |
|                       |                              |          BY252875             |
|                       |                              |          BY246952             |
|                       |                              |          BY268414             |
|                       |                              |          BY248859             |
|                       |                              |          BB595403             |
|                       |                              |          BQ573468             |
|                       |                              |          BI690199             |
|                       |                              |          BC018368             |
|                       |                              |          BI987546             |
|                       |                              |          CF169625             |
|                       |                              |          BY006077             |
|                       |                              |          BY261067             |
|                       |                              |          BF124060             |
|                       |                              |          BY775501             |
|                       |                              |          BY347056             |
|                       |                              |          BY102317             |
|                       |                              |          BY775497             |
|                       |                              |          BY317075             |
|                       |                              |          BY768014             |
|                       |                              |          BY344743             |
|                       |                              |          BY096392             |
|                       |                              |          BY096604             |
|                       |                              |          BY093043             |
|                       |                              |          BY135580             |
|                       |                              |          BY294738             |
|                       |                              |          BY296134             |
|                       |                              |          BY290496             |
|                       |                              |          BY246970             |
|                       |                              |          BY263521             |
|                       |                              |          BY174855             |
|                       |                              |          BF020760             |
|                       |                              |          BG245022             |
|                       |                              |          BY723991             |
|                       |                              |          BE912638             |
|                       |                              |          CA945409             |
|                       |                              |          BG519277             |
|                       |                              |          CA979399             |
|                       |                              |          BU705796             |
|                       |                              |          BU053951             |
|                       |                              |          BG914637             |
|                       |                              |          CF106368             |
|                       |                              |          BU559479             |
|                       |                              |          BY326131             |
|                       |                              |          BY271424             |
|                       |                              |          BG086849             |
|                       |                              |          CF725880             |
|                       |                              |          CN837424             |
|                       |                              |          BE303217             |
|                       |                              |          CN661749             |
|                       |                              |          BI684211             |
|                       |                              |          CB232780             |
|                       |                              |          AA104811             |
|                       |                              |          BI409744             |
|                       |                              |          CD806561             |
|                       |                              |          CD807769             |
|                       |                              |          BE380938             |
|                       |                              |          BF151964             |
|                       |                              |          BI647137             |
|                       |                              |          BF152036             |
|                       |                              |          BE380646             |
|                       |                              |          AA982538             |
|                       |                              |          AA544981             |
|                       |                              |          AA004031             |
|                       |                              |          AW763412             |
|                       |                              |          BF785109             |
|                       |                              |          CB947797             |
|                       |                              |          BF151300             |
|                       |                              |          BE306826             |
|                       |                              |          CB232207             |
|                       |                              |          CB202855             |
|                       |                              |          BY114364             |
|                       |                              |          BY114340             |
|                       |                              |          BY115806             |
|                       |                              |          BY116178             |
|                       |                              |          BY114410             |
|                       |                              |          BY114477             |
|                       |                              |          BY114453             |
|                       |                              |          BY112920             |
|                       |                              |          BY112952             |
|                       |                              |          BY116339             |
|                       |                              |          CD772889             |
|                       |                              |          CD363851             |
|                       |                              |          BY231671             |
|                       |                              |          BB849007             |
|                       |                              |          CF198074             |
|                       |                              |          BE447948             |
|                       |                              |          CB843052             |
|                       |                              |          AA674187             |
|                       |                              |          BI106402             |
|                       |                              |          BF470146             |
|                       |                              |          AK019833             |
|                       |                              |          BY716019             |
|                       |                              |          BX514197             |
|                       |                              |          AA138500             |
|                       |                              |          AI386396             |
|                       |                              |          CB723488             |
|                       |                              |          BI555244             |
|                       |                              |          CB319822             |
|                       |                              |          BU610346             |
|                       |                              |          BB754672             |
|                       |                              |          BI690576             |
|                       |                              |          AW495018             |
|                       |                              |          BF224572             |
|                       | *Seq. mapped in strand Minus*| *Seq. mapped in strand Minus* |
|                       |           AK092372           |          BU755704             |
|                       |                              |          AI847493             |
|                       |                              |          BE457518             |
|                       |                              |          BM570786             |
|                       |                              |          CB233320             |
|                       |                              |          BC023861             |
|                       |                              |          AI447856             |
|                       |                              |          BE625014             |
|                       |                              |          BE943996             |
|                       |                              |          BY712791             |
|                       |                              |          BC025453             |
|                       |                              |          AI840219             |
|                       |                              |          AK019363             |
|                       |                              |          AK008820             |
|                       |                              |          AK011473             |
|                       |                              |          BE303602             |
|                       |                              |          CB594156             |
|                       |                              |          BE980463             |
|                       |                              |          AV099506             |
|                       |                              |          AK075957             |
|                       |                              |          BE848578             |
|                       |                              |          AV237614             |
|                       |                              |          AA162512             |
|                       |                              |          BI690382             |
|                       |                              |          CA979140             |
|                       |                              |          BE847238             |
|                       |                              |          BF658152             |
|                       |                              |          CK790618             |
|                       |                              |          BY732945             |
|                       |                              |          AA438042             |
|                       |                              |          BY084157             |
|                       |                              |          BY739146             |
|                       |                              |          BY090877             |
|                       |                              |          BG807864             |
|                       |                              |          BB658019             |
|                       |                              |          BG085488             |
|                       |                              |          BM939643             |
|                       |                              |          CA316321             |
|                       |                              |          BY756310             |
|                       |                              |          BU756942             |
|                       |                              |          BQ032749             |
|                       |                              |          AI837382             |
|                       |                              |          BB829363             |
|                       |                              |          BB430448             |
|                       |                              |          AA756656             |
|                       |                              |          BB449707             |
|                       |                              |          BY756877             |
|                       |                              |          AK083734             |
|                       |                              |          AK049957             |
|                       |                              |          BY598787             |
|                       |                              |          AB041610             |
|                       |                              |          BF140324             |
|                       |                              |            C87832             |
|                       |                              |          AA254011             |
|                       |                              |          AA472535             |
|                       |                              |          BI415025             |
|                       |                              |          BE850499             |
|                       |                              |          CD562185             |
|                       |                              |          BY558427             |
|                       |                              |          AA475089             |
|                       |                              |          BG067882             |
|                       |                              |          AA789371             |
|                       |                              |          CN717465             |
|                       |                              |          CK137711             |
|                       |                              |          CN723480             |
|                       |                              |          BU524037             |
|                       |                              |          BU059133             |
|                       |                              |          BI082719             |
|                       |                              |          BI652664             |
|                       |                              |          CN709497             |
|                       |                              |          CN711251             |
|                       |                              |          CN705894             |
|                       |                              |          AA867878             |
|                       |                              |          BG080940             |
|                       |                              |          BE847780             |
|                       |                              |          BQ443008             |
|                       |                              |          CA532976             |
|                       |                              |          AU080474             |
|                       |                              |          BU709010             |
|                       |                              |          CF738889             |
|                       |                              |          CA750114             |
|                       |                              |          CF750465             |
|                       |                              |          CD348615             |
|                       |                              |          BY736504             |
|                       |                              |          CA324258             |
|                       |                              |          BY735935             |
|                       |                              |          BY753953             |
|                       |                              |          AA155333             |
|                       |                              |          BE687956             |
|                       |                              |          CN707684             |
|                       |                              |          BF100473             |
|                       |                              |          BY733394             |
|                       |                              |          BU935682             |
|                       |                              |          BY728991             |
|                       |                              |          BI107571             |
|                       |                              |          BM503659             |
|                       |                              |          BY745571             |
|                       |                              |            W54453             |
|                       |                              |          BB651836             |
|                       |                              |          BG146068             |
|                       |                              |          BB654867             |
|                       |                              |          BB612836             |
|                       |                              |          CN525123             |
|                       |                              |          AA002497             |
|                       |                              |          BB628892             |
|                       |                              |          AA003624             |
|                       |                              |          BY253033             |
|                       |                              |                               |
|                       |                              |                               |
|                       |                              |                               |
|                       |                              |                               |
|                       |                              |                               |
|                       |                              |                               |
|                       |                              |                               |
|                       |                              |                               |
|                       |                              |                               |
|                       |                              |                               |
|                       |                              |                               |
|                       |                              |                               |
|                       |                              |                               |
|                       |                              |                               |
|                       |                              |                               |
|                       |                              |                               |
|                       |                              |                               |
|                       |                              |                               |
|                       |                              |                               |
|                       |                              |                               |
|                       |                              |                               |
+-----------------------+------------------------------+-------------------------------+
|      #   54           | *Seq. mapped in strand Plus* | *Seq. mapped in strand Plus*  |
|                       |           AW452962           |          AV100847             |
|                       |           BU726218           |                               |
|                       |           AA767626           |                               |
|                       |           BG198523           |                               |
|                       |           CD364547           |                               |
|                       |           BM021070           |                               |
|                       |           AL042322           |                               |
|                       | *Seq. mapped in strand Minus*| *Seq. mapped in strand Minus* |
|                       |           BI334838           |          BU053473             |
|                       |           AI735305           |          AW538045             |
|                       |           BU600626           |          CA466609             |
|                       |           AI935227           |          CK782249             |
|                       |           BE875127           |          BG966426             |
|                       |           AW190609           |          CF617405             |
|                       |           AI869973           |          AA124706             |
|                       |           AA745969           |          BQ747587             |
|                       |           AA932544           |          AA959868             |
|                       |           BF687531           |          AI461820             |
|                       |           CA446268           |          BF682796             |
|                       |           BC015958           |          BY471862             |
|                       |           BG288743           |          AA616265             |
|                       |           AW079326           |          BQ126701             |
|                       |           BU734576           |          AA260350             |
|                       |           AI434040           |          BI438141             |
|                       |           BU734658           |          CB934195             |
|                       |           AI241645           |          CF926592             |
|                       |           BI496774           |          CK373221             |
|                       |           AA040080           |          BX525234             |
|                       |           AA894515           |          AA960245             |
|                       |           AA888746           |          AA895638             |
|                       |           AA936204           |          BF018261             |
|                       |           AA602621           |          BG871945             |
|                       |           BM758131           |          BY388989             |
|                       |           AI587558           |          BY665531             |
|                       |             N63136           |          BM201469             |
|                       |           BG035032           |          CB573964             |
|                       |           BQ018435           |          BY468085             |
|                       |           AA737764           |          BY469219             |
|                       |           AW305027           |          CA879027             |
|                       |           AW167427           |          AW538095             |
|                       |           AI745237           |          CK336869             |
|                       |           BI091095           |          BY471744             |
|                       |           BI090089           |          AW540776             |
|                       |           BU617321           |          BY466615             |
|                       |           AI566492           |          AW913340             |
|                       |           AA774628           |          AW545624             |
|                       |           BM792100           |          AI323809             |
|                       |           CD520816           |          BB759319             |
|                       |           AL048392           |          AW540530             |
|                       |           BM975987           |          AU041466             |
|                       |           BM998860           |          AW537417             |
|                       |           BU187351           |          BM241338             |
|                       |           BF432473           |          AA959719             |
|                       |           BM998825           |          BM218786             |
|                       |           CA417046           |          AW544465             |
|                       |           CA445377           |          BQ125950             |
|                       |           BG033778           |          CB204775             |
|                       |           CA447926           |          BM125443             |
|                       |           CA442924           |          AU041731             |
|                       |           CA447357           |          BM877089             |
|                       |           BQ045137           |          CF622707             |
|                       |           BU683070           |          CD741433             |
|                       |           AI554384           |          BF538251             |
|                       |           BQ007917           |          BM230743             |
|                       |           BU689244           |          BG072173             |
|                       |           CA417501           |          AW540945             |
|                       |           BU682679           |          AW545790             |
|                       |           BM975953           |          BM236094             |
|                       |           BQ007669           |          CK329902             |
|                       |           CA310031           |          BM233789             |
|                       |           CA306803           |          BM245360             |
|                       |           BQ437579           |          CB193458             |
|                       |           BM799963           |          BM230817             |
|                       |           CA446071           |          BM239516             |
|                       |           BU616251           |          BM241523             |
|                       |           BM989624           |          BM231575             |
|                       |           CA420871           |          AW539895             |
|                       |           AW026340           |          AW553446             |
|                       |           CA444469           |          BM242622             |
|                       |           BQ008670           |          AW546283             |
|                       |           CB852959           |          AW558561             |
|                       |           AI609601           |          AW990557             |
|                       |           CA431508           |          AW546398             |
|                       |             Y00345           |          CF551275             |
|                       |           AA888519           |          BU938913             |
|                       |           AI360879           |          CF751348             |
|                       |           AA730824           |          BU755393             |
|                       |           BM755866           |          BY556706             |
|                       |             R44670           |          BB760630             |
|                       |           AI952130           |          BY468910             |
|                       |           BU595504           |          BB756273             |
|                       |           BG777876           |          BB756290             |
|                       |           BM466220           |          BB771864             |
|                       |           BM799679           |          BB752628             |
|                       |           BQ772939           |          BB762748             |
|                       |           AV742394           |          BB754584             |
|                       |           BE613011           |          CO040903             |
|                       |           BM015772           |          BY754835             |
|                       |           AA887155           |          BC046233             |
|                       |           AL048405           |          BF457150             |
|                       |           BG179686           |          BU755391             |
|                       |           BX414090           |          BY591120             |
|                       |           BM479486           |          BB754348             |
|                       |           CN337469           |          BY466166             |
|                       |           AI619970           |          BB751784             |
|                       |           BI094379           |          BB769408             |
|                       |           AL566793           |          BY480995             |
|                       |           AW026347           |          BY469677             |
|                       |           AL516310           |          BY470171             |
|                       |           BX423680           |          BB754578             |
|                       |           BG679416           |          BY479463             |
|                       |           BG291978           |            C76466             |
|                       |           BG531038           |          CK623072             |
|                       |           BG036201           |          BY759236             |
|                       |           CD641213           |          AI788811             |
|                       |           BG744897           |          BF661514             |
|                       |           BE971236           |          AA563137             |
|                       |           BU077257           |          BQ258113             |
|                       |           AL516265           |          AA590504             |
|                       |           BF241914           |          BF607674             |
|                       |           BG030633           |          BF682084             |
|                       |           BE613173           |          CK130118             |
|                       |           BG033004           |          AA170349             |
|                       |           BG500155           |          BU939357             |
|                       |           BM746759           |          AA607312             |
|                       |           BG027025           |          BE625520             |
|                       |           CR744296           |          BE628919             |
|                       |           AF074617           |          BE851043             |
|                       |           AI110710           |          BI106289             |
|                       |           BG393709           |          CD564458             |
|                       |           BQ648753           |          CF951726             |
|                       |           BM314630           |          AA271429             |
|                       |           BM744470           |          AA242468             |
|                       |           CD521378           |          CF951195             |
|                       |           BE881044           |          CB847748             |
|                       |           BQ439759           |          AW209995             |
|                       |           AJ713499           |          BE627600             |
|                       |           BQ218561           |          CF751829             |
|                       |           CN337461           |          AA426846             |
|                       |           BM765868           |          BF118059             |
|                       |           BE877427           |          CK618109             |
|                       |           BU942419           |          BM232194             |
|                       |           AA664970           |          AW764038             |
|                       |           AV715804           |          BG229702             |
|                       |           BX405003           |          AL118347             |
|                       |           AV650986           |          AI183017             |
|                       |           BQ429518           |          AA543129             |
|                       |           AV685975           |          BE334348             |
|                       |           AV689920           |          CF379465             |
|                       |           CD639185           |          BF319067             |
|                       |           AV683195           |          AA106783             |
|                       |           CB135663           |          BE335207             |
|                       |           CN337404           |          BG228840             |
|                       |           BE932391           |          AW987286             |
|                       |           CD108489           |          AW990662             |
|                       |           CN337448           |          BF318579             |
|                       |           BE873994           |          BF450382             |
|                       |           AW973096           |          AW229039             |
|                       |           CN337462           |          AV284332             |
|                       |           BF803126           |          BF319045             |
|                       |           BQ435845           |          BB828423             |
|                       |           BI857897           |          AW213790             |
|                       |           BF970693           |          AA409986             |
|                       |           AV714541           |          BB811125             |
|                       |           AW974400           |          BB809873             |
|                       |           BI253883           |          AW322236             |
|                       |           AW074338           |          BB810280             |
|                       |           BG743147           |          BY392249             |
|                       |           AW662297           |          AA407163             |
|                       |           AW613908           |          BM211782             |
|                       |           AW628372           |          BB820567             |
|                       |           AI492720           |          BY387950             |
|                       |           AI074390           |          AW909511             |
|                       |           AW664292           |          BM216712             |
|                       |           AI969677           |          BM221517             |
|                       |           AW264271           |          BM233689             |
|                       |           AW074021           |          AW701761             |
|                       |           BF666099           |          BF721903             |
|                       |           AU117796           |          BM222717             |
|                       |           BG497670           |          BM224131             |
|                       |           CN337470           |          AW764182             |
|                       |           AI926265           |          BM211685             |
|                       |           AW073512           |          BM212045             |
|                       |           AI300390           |          BF148729             |
|                       |           AI300397           |          BM083801             |
|                       |           BF058183           |          BF721954             |
|                       |           AA130979           |          BF021319             |
|                       |           BG501005           |          AW209084             |
|                       |             H78327           |          AK089747             |
|                       |           AI520855           |          AA272487             |
|                       |             R12685           |          AA050116             |
|                       |           AW338911           |          BF123669             |
|                       |           BF211239           |          BY573658             |
|                       |           AA046124           |          BB761932             |
|                       |           AA318568           |          BB757112             |
|                       |           AI872766           |          BB755068             |
|                       |           AI917961           |          BB757430             |
|                       |           AI358216           |          BF160216             |
|                       |           AI888636           |          BF721465             |
|                       |           AW117627           |          BY564966             |
|                       |           AA903933           |          AV351797             |
|                       |           AU151581           |          AV228402             |
|                       |           AW770015           |          AV225487             |
|                       |           AW514601           |          AV303371             |
|                       |           AW302608           |          AV306839             |
|                       |           AU160424           |          BB297794             |
|                       |           AI214253           |          BM199023             |
|                       |           AU159839           |          BB676512             |
|                       |           BE221345           |          BB733657             |
|                       |           AW769021           |          BY367773             |
|                       |           AW613220           |          BB689206             |
|                       |           AW440858           |          BB679787             |
|                       |           AI969501           |          BB676204             |
|                       |           AW662576           |          BB687045             |
|                       |           AI734929           |          BB675917             |
|                       |           AI884378           |          BB674546             |
|                       |           BM825258           |          BB677414             |
|                       |           AI924132           |          BB676683             |
|                       |           BI261318           |          BM208757             |
|                       |           AK130523           |          AA270581             |
|                       |           BQ637206           |          AA538443             |
|                       |           CN337374           |          CA871858             |
|                       |           BG615133           |          BI656505             |
|                       |           AI708517           |          AV146688             |
|                       |           AW662970           |          BE456729             |
|                       |           BE784785           |          AA409993             |
|                       |           BM849817           |          AI848812             |
|                       |           BM142604           |          BQ936288             |
|                       |           BG569191           |          BG921951             |
|                       |           CA310557           |          AA407008             |
|                       |           AI830972           |          BB728791             |
|                       |           BU735637           |          AU020487             |
|                       |           CD641134           |          AI227404             |
|                       |           BQ228925           |          AI852094             |
|                       |           AA034019           |          AW123202             |
|                       |             H67720           |          BF780391             |
|                       |           AI805467           |          BG965895             |
|                       |           BM856785           |          AU021070             |
|                       |           BU533501           |          AW545293             |
|                       |           BM458943           |          BE225737             |
|                       |             T07631           |          AW701899             |
|                       |           BM695875           |          BM210826             |
|                       |           AA307693           |          AV222177             |
|                       |           BG392738           |          BI696116             |
|                       |           BQ029896           |          BY765192             |
|                       |           BE245515           |          CO043611             |
|                       |           AW193448           |          BC023145             |
|                       |             H68995           |            C80705             |
|                       |           AA804295           |            C78518             |
|                       |             N31457           |          BI851591             |
|                       |           AW316676           |          BF133895             |
|                       |           AI459831           |          AW989030             |
|                       |           CA419396           |          AA267316             |
|                       |           BU737942           |          BQ257570             |
|                       |           BQ083233           |          AA497794             |
|                       |           BQ447256           |          AA967240             |
|                       |           AA121394           |          AA273285             |
|                       |           AU153570           |          BF581993             |
|                       |           AW337475           |          AA438185             |
|                       |           BG031343           |          AW681369             |
|                       |           BG392794           |          BG095392             |
|                       |           AW613460           |          AV248281             |
|                       |           CN480838           |          BB046257             |
|                       |           BM782382           |          AW826110             |
|                       |           BE138725           |          AL364621             |
|                       |           CB243838           |          BG093728             |
|                       |           BQ425945           |          CN837286             |
|                       |           AI619440           |          BF161814             |
|                       |           AI889678           |          BI330138             |
|                       |           BM982351           |          BF017567             |
|                       |           CN337364           |          AA561346             |
|                       |           BM015060           |          BI690011             |
|                       |           BM989734           |          BM053555             |
|                       |           BM990588           |          BM069381             |
|                       |           BU689310           |          BM125728             |
|                       |           BU730800           |          CN689290             |
|                       |           BU156697           |          AW740788             |
|                       |             T90886           |          BM053461             |
|                       |           AA888449           |          BY674970             |
|                       |           AI268870           |          AW228870             |
|                       |           BU532832           |          BI688477             |
|                       |           AU185892           |          CA984336             |
|                       |           BM747586           |          BM053192             |
|                       |             H41953           |          BM053265             |
|                       |           CD641365           |          BM069080             |
|                       |           BG770996           |          BM125496             |
|                       |           BM700450           |          AW240753             |
|                       |           CD692253           |          CF622569             |
|                       |             R06885           |          AW322100             |
|                       |           CN337446           |          AA517208             |
|                       |           AA553905           |          BF451434             |
|                       |           AA583301           |          BF319909             |
|                       |           AA582592           |          AW322177             |
|                       |           AA565223           |          BF451344             |
|                       |           BE250648           |          BE335188             |
|                       |           BF689285           |          AW228118             |
|                       |           BU154214           |          BF148881             |
|                       |           BF212193           |          AW558312             |
|                       |           CA866617           |          BB823080             |
|                       |           BG779594           |          AW823185             |
|                       |           BF896322           |          AW555286             |
|                       |           AW996358           |          BY466485             |
|                       |           AW796092           |          BM232687             |
|                       |           BQ893707           |          BM217880             |
|                       |           CD517455           |          BM232659             |
|                       |           AW796161           |          AW542604             |
|                       |           BE925007           |          BF225531             |
|                       |           BE925017           |          BI689763             |
|                       |           BE925001           |          BG063771             |
|                       |           BE925005           |          BG063770             |
|                       |           BM696843           |          BE283847             |
|                       |           BF896278           |          BE306499             |
|                       |           BF807433           |          AV238713             |
|                       |           AW796069           |          AV262845             |
|                       |           BF805410           |          AV227486             |
|                       |           CB135174           |          AV254379             |
|                       |           BF805418           |          BB676643             |
|                       |           BF805613           |          BB673870             |
|                       |           BF744838           |          AW321045             |
|                       |           BF911974           |          AA607770             |
|                       |             C75375           |          BF321728             |
|                       |           AI683737           |          AA863935             |
|                       |             N42228           |          BF227358             |
|                       |           AV737203           |          AW540818             |
|                       |           BG897514           |          BF320443             |
|                       |           BG499316           |          BF321445             |
|                       |           AA007137           |          AA166066             |
|                       |           BF170078           |          BI104796             |
|                       |           AW607801           |          BY763395             |
|                       |           BF909631           |          BU534312             |
|                       |             T96661           |          BY367855             |
|                       |           AI798335           |          BB709368             |
|                       |           AI129805           |          BB710597             |
|                       |           BF793162           |          BB687233             |
|                       |             T89873           |          BB713394             |
|                       |           AA158457           |          BE912032             |
|                       |           AU185743           |          BF136275             |
|                       |             W45489           |          AA596775             |
|                       |           AW070656           |          AI326947             |
|                       |           AI361734           |          AA499168             |
|                       |           AA203481           |          AW258312             |
|                       |           AI610369           |          BG915449             |
|                       |           CN337463           |          BB474214             |
|                       |           AA083487           |          AV262644             |
|                       |           BF796137           |          CF622570             |
|                       |           AA188648           |          BI658283             |
|                       |           AW272654           |          BB033473             |
|                       |           AA186453           |          BG243131             |
|                       |             W68053           |          BB156279             |
|                       |           AA069195           |          BB407803             |
|                       |             T58647           |          BB365964             |
|                       |           AA171785           |          BG171980             |
|                       |             W60312           |          BI692725             |
|                       |           AW005539           |          AV117003             |
|                       |             H73919           |          BI105328             |
|                       |           AA223578           |          BB116160             |
|                       |           AI074074           |          BI330032             |
|                       |           AA417222           |          BI654440             |
|                       |           AI339829           |          BI149362             |
|                       |           AI263976           |          BY676021             |
|                       |           AA622310           |          BB817899             |
|                       |           AA858200           |          BB674898             |
|                       |           BM671007           |          BB677074             |
|                       |           AW071867           |          BQ265809             |
|                       |           BU665018           |          BB686892             |
|                       |           AI130895           |          BB712520             |
|                       |           AU185913           |          BB684227             |
|                       |           AI962964           |          BY387442             |
|                       |           BG168196           |          BY389572             |
|                       |             N87739           |          BG868291             |
|                       |           BM721274           |          BI851153             |
|                       |           BU532173           |          BY466155             |
|                       |           CF135083           |          BG919635             |
|                       |           AW514079           |          BM223052             |
|                       |           AA532725           |          BI645538             |
|                       |           AI686708           |          AW215867             |
|                       |           BE780332           |          BE200086             |
|                       |           BG395578           |          AI593680             |
|                       |           BE614476           |          BQ266455             |
|                       |           AI370402           |          AW538411             |
|                       |           AI699721           |          AW539487             |
|                       |           AW779497           |          BC004587             |
|                       |           AW511950           |          BC003870             |
|                       |           AI274578           |          BC011207             |
|                       |           AI886491           |          BF098965             |
|                       |           AW090363           |          BQ443438             |
|                       |           AW517738           |          BI106007             |
|                       |           AW198169           |          CB596064             |
|                       |           AW511855           |          BE374735             |
|                       |           AW026627           |          CA464589             |
|                       |           AW169591           |          BM216428             |
|                       |           AW572217           |          AA561656             |
|                       |           AW472890           |          AA591993             |
|                       |           AA506606           |            W97241             |
|                       |           AW002754           |          AA575633             |
|                       |           AW518764           |          AI789853             |
|                       |           AW591759           |          AA690541             |
|                       |             H96657           |          BE197581             |
|                       |           AW169410           |          AA688511             |
|                       |           AW510382           |          AA117196             |
|                       |           AW337901           |          AA117197             |
|                       |           AI284499           |          AA222621             |
|                       |           AW337448           |          AA589023             |
|                       |           AW517420           |          AA139140             |
|                       |           AI284857           |          BI157176             |
|                       |           AW073572           |          AA117944             |
|                       |           BE301772           |          BF581504             |
|                       |           AI570971           |            X65553             |
|                       |           AW471141           |          BM200231             |
|                       |           AI972153           |          BI851965             |
|                       |           AW151280           |          BB017550             |
|                       |           AW474486           |          BB049320             |
|                       |           AW070830           |          BI159036             |
|                       |           AW468093           |          BB189705             |
|                       |           AI620288           |          BF226484             |
|                       |           AW438588           |          BB099177             |
|                       |           AW439412           |            W57260             |
|                       |           AW131248           |          BY361005             |
|                       |           AI888569           |          AA616897             |
|                       |           AI859202           |          BB817015             |
|                       |           AW167426           |          BE197506             |
|                       |           AI445811           |          BB727137             |
|                       |           AW467955           |          BF450684             |
|                       |           AW474527           |          BB710474             |
|                       |           AW172926           |          BI676800             |
|                       |           AW195134           |          AI226413             |
|                       |           AI633005           |          BI319411             |
|                       |           AI678891           |          BE377838             |
|                       |           AI870217           |          AW413441             |
|                       |           AI889312           |          AA759821             |
|                       |           AW130522           |          BE553673             |
|                       |           AI049611           |          AA797916             |
|                       |           AI564157           |          AA434838             |
|                       |           BM558071           |          BF319652             |
|                       |           AA033548           |          BU937956             |
|                       |           AI567184           |          BB676306             |
|                       |             H99995           |          CB574444             |
|                       |           AW612868           |          BI525644             |
|                       |           AU159962           |          AV167717             |
|                       |           AI633098           |            W15034             |
|                       |           AI720906           |          BB377400             |
|                       |           AA146575           |          BB680938             |
|                       |             T12082           |          AL034859             |
|                       |           AA664267           |          AI596439             |
|                       |           AI869469           |          CB945701             |
|                       |           AI698418           |          BF142678             |
|                       |           AI698425           |          BI465720             |
|                       |             R85274           |          CA539767             |
|                       |           BE890808           |          BI411767             |
|                       |             H88507           |          BE554771             |
|                       |             N68555           |          BE572604             |
|                       |           AI469491           |          AA008270             |
|                       |           AI627166           |          BF301044             |
|                       |           AI886104           |          BB140335             |
|                       |           AW169636           |          BB296726             |
|                       |           AW771790           |          AA560598             |
|                       |           AI669407           |          BI077804             |
|                       |           BE046324           |          CB846901             |
|                       |           AI686846           |          BF150596             |
|                       |           AI921763           |          AA216911             |
|                       |           AW512703           |          BB737617             |
|                       |           AI499246           |          BI696311             |
|                       |           AI961477           |            W66988             |
|                       |           BG502772           |          BB744784             |
|                       |           AI564368           |          BI793662             |
|                       |           BM463571           |          CA857452             |
|                       |           AI700145           |          AA596527             |
|                       |           AI634445           |          AA118144             |
|                       |           BE089004           |            W66681             |
|                       |           AI559805           |          BI466395             |
|                       |           AI491843           |          BE334642             |
|                       |           BI028505           |          AA474199             |
|                       |           AW440007           |            W89434             |
|                       |           AI470685           |            W84301             |
|                       |           AW079136           |          BY699793             |
|                       |             T90559           |          CB272562             |
|                       |             H73530           |          AA794417             |
|                       |           AI362176           |          BB291600             |
|                       |           AW169515           |          AK044829             |
|                       |           AU148916           |          BI651688             |
|                       |           AI174553           |          BE306625             |
|                       |           AI269716           |          CN673650             |
|                       |           AI539122           |            W70725             |
|                       |           BF438473           |          CB317058             |
|                       |           AI624764           |          BF136891             |
|                       |           AI889407           |          AA032346             |
|                       |           AI924791           |          BU961141             |
|                       |           AW073818           |          CK137047             |
|                       |           AW087404           |          BG148269             |
|                       |           AW090787           |          BG277852             |
|                       |           BE622870           |          AL360527             |
|                       |           BM469185           |          BI438144             |
|                       |           AI949233           |          BM877565             |
|                       |           AW593609           |          BF301504             |
|                       |           AI811164           |          AI007313             |
|                       |           AI963124           |          AA529695             |
|                       |           BU537743           |          BE334770             |
|                       |           AW131203           |          BI665369             |
|                       |           AA610464           |          BI689730             |
|                       |           BQ276609           |          BI653852             |
|                       |           AW270760           |          AA655917             |
|                       |           BG777246           |          AA536898             |
|                       |           BG610721           |            W65629             |
|                       |             W68194           |          AW213239             |
|                       |           BU596326           |          BQ827936             |
|                       |           BG611716           |          CA537777             |
|                       |           BE779849           |          CA980149             |
|                       |           BG286902           |            D76926             |
|                       |           BE538767           |          BI556936             |
|                       |           BU600019           |          BG100394             |
|                       |           BE966627           |          BI159306             |
|                       |           BE963569           |          BU551865             |
|                       |           BQ421682           |          AI049037             |
|                       |           BM915967           |          CD352924             |
|                       |           BF968529           |          AA560843             |
|                       |           BM479598           |          AW681308             |
|                       |             W86657           |          AA033382             |
|                       |           BQ228655           |          AW413436             |
|                       |           BU789209           |          AA462159             |
|                       |           AA055037           |          AA212189             |
|                       |           BM148697           |          BE288201             |
|                       |           BM468752           |          BF540152             |
|                       |           AW022309           |          BF143351             |
|                       |           BQ436938           |          BF012238             |
|                       |           BM462819           |          BP427063             |
|                       |           BG996294           |          BE913462             |
|                       |           BQ423577           |          BG917085             |
|                       |           BM466899           |          BE333877             |
|                       |           BF345627           |          AI385840             |
|                       |           CD677629           |          BE913296             |
|                       |           BQ436610           |          BF139117             |
|                       |           BG390566           |          BG866909             |
|                       |           BM450717           |          BQ942395             |
|                       |           BQ432115           |          BQ840368             |
|                       |           BG759000           |          CK138000             |
|                       |           BM145765           |          CF981773             |
|                       |           BU169615           |          BU583021             |
|                       |           AW327416           |          BU582834             |
|                       |           AI537019           |          BU605069             |
|                       |           AW084182           |          AA546165             |
|                       |             H23275           |          CF982682             |
|                       |           CA444097           |          AA571368             |
|                       |           AI754355           |          AI158095             |
|                       |           AI759005           |          BG919340             |
|                       |           BU737971           |          BQ923931             |
|                       |           BG389914           |          BF180914             |
|                       |           AA728994           |          CF173239             |
|                       |           BM475752           |          CF913636             |
|                       |           BM457227           |          CF913689             |
|                       |           AI491754           |          CF914707             |
|                       |           BQ231518           |          CF915386             |
|                       |           BG926136           |          CF915849             |
|                       |           BG926463           |          CF173372             |
|                       |           AA715721           |          BY012900             |
|                       |           CB045852           |          BG276180             |
|                       |           AU153542           |          AI157526             |
|                       |           AU155466           |          BI696690             |
|                       |           AA583166           |          CF916540             |
|                       |           AU153335           |          CF915516             |
|                       |           BI860864           |          BQ947366             |
|                       |           BQ221304           |          BU898985             |
|                       |           AI653876           |          CF915646             |
|                       |           BQ232302           |          AA007903             |
|                       |           BQ019427           |          AA562218             |
|                       |           AI284725           |          AA549093             |
|                       |           AW117816           |          AA171356             |
|                       |           AW771080           |          BF167429             |
|                       |           BG258023           |          BF160232             |
|                       |           AA604528           |          BF450852             |
|                       |           BM998176           |          AA530161             |
|                       |           BM681346           |          CF171983             |
|                       |           BM972400           |          BI904835             |
|                       |           BQ575314           |          CK333693             |
|                       |           BQ217350           |          BG144780             |
|                       |           AI986279           |          BF164212             |
|                       |           BQ027927           |          CF916587             |
|                       |           CB044040           |          CF198202             |
|                       |           AW512272           |          BQ564244             |
|                       |           BQ020520           |          CF916074             |
|                       |           BQ775197           |          BG863890             |
|                       |           BQ441750           |          CF173043             |
|                       |           CD370553           |          CF172583             |
|                       |           BU730976           |          AA560088             |
|                       |           BU618377           |          BI149578             |
|                       |           BU633954           |            W71429             |
|                       |           AI610401           |          BE285657             |
|                       |           AI744957           |          CA559571             |
|                       |           BU153013           |          BG966564             |
|                       |           BM995269           |          BQ749012             |
|                       |           BM990323           |          BQ947604             |
|                       |           BU631245           |          AA655662             |
|                       |           CA309174           |          BF158939             |
|                       |           BQ421561           |          BE286626             |
|                       |           BU182575           |          CF912715             |
|                       |           AI718601           |          AA073281             |
|                       |           BU618647           |          BI110951             |
|                       |           BQ438502           |          CA555646             |
|                       |           BU633505           |          CF174480             |
|                       |           BM907675           |          AI790027             |
|                       |           BC023217           |          CA559848             |
|                       |           BC023520           |          CA560416             |
|                       |             T81928           |          BE376401             |
|                       |           AA578812           |          CA557347             |
|                       |           BE777922           |          CB183708             |
|                       |           BF089698           |          BQ840495             |
|                       |           BM768681           |          AA561778             |
|                       |           BF093405           |          AW210431             |
|                       |           BG165933           |          CF732032             |
|                       |           BU788953           |          BI456760             |
|                       |           BM838691           |          BQ840429             |
|                       |           BE788417           |          AA120549             |
|                       |           BF035784           |          BI158758             |
|                       |           AA729392           |          CB946717             |
|                       |             W60261           |          AA596648             |
|                       |           BE885618           |          CK137165             |
|                       |           BF103650           |          AA656362             |
|                       |           BE779012           |          AA117394             |
|                       |           AW008298           |          AL034973             |
|                       |           BG390274           |          CA561870             |
|                       |           BE716189           |          CA560634             |
|                       |           AA216386           |          CF981646             |
|                       |           BE177477           |          CK137037             |
|                       |           BM744712           |          CA464674             |
|                       |           BG687650           |          CA555955             |
|                       |           BI253953           |          BF536843             |
|                       |           BU194124           |          BQ086734             |
|                       |           AV686586           |          AA086731             |
|                       |           AI521406           |          CN530244             |
|                       |           AI917949           |          AA615571             |
|                       |             R08050           |          AW214795             |
|                       |           BG537500           |          AA666489             |
|                       |           AW316533           |          BF165691             |
|                       |           AI869977           |          BF180328             |
|                       |           BG499473           |          CB209480             |
|                       |           BE043941           |          BE136961             |
|                       |           BG260837           |          AA542413             |
|                       |           AI864773           |          BQ085405             |
|                       |           BM469384           |          BI685437             |
|                       |             R85429           |          BF135332             |
|                       |           AI872108           |          AA072090             |
|                       |             T67460           |          AA561404             |
|                       |             R00260           |            W12960             |
|                       |           BQ018856           |            W08986             |
|                       |           BU589348           |          CB946415             |
|                       |           AA857509           |          BI694622             |
|                       |           AI587340           |          BI855169             |
|                       |           AU150465           |          BX633767             |
|                       |           AU153604           |          AA646311             |
|                       |           BG778483           |          CF915146             |
|                       |           AW572660           |          CN833344             |
|                       |           AU148437           |          CB573624             |
|                       |           BG178223           |          CA978700             |
|                       |           BM467229           |          BE380574             |
|                       |           CD368451           |          BE370646             |
|                       |           AI983902           |          CA555944             |
|                       |           CA441174           |          CA557496             |
|                       |           AW167471           |          CA555984             |
|                       |           AI678793           |          AA798415             |
|                       |           BM679962           |          AW209262             |
|                       |           AI499363           |          AL118047             |
|                       |           CB528485           |          AA537238             |
|                       |           BU172277           |          BE226966             |
|                       |           BI862346           |          BG870630             |
|                       |           AW515033           |          BI455248             |
|                       |           AA491878           |          BI558139             |
|                       |           AA613657           |          AA864112             |
|                       |           AA737223           |          CF894494             |
|                       |           BE042571           |          AW320563             |
|                       |           AW365693           |          AA031045             |
|                       |           BG674041           |          CF948728             |
|                       |           AW373821           |          CA557804             |
|                       |           AW608024           |          CA562072             |
|                       |           BI825758           |          BI411548             |
|                       |             H25582           |          BI110910             |
|                       |           BG180663           |          AA822239             |
|                       |           AW469980           |          AW910943             |
|                       |           BG291994           |          CF900602             |
|                       |           AA149971           |          BE369092             |
|                       |           BQ232645           |          AA546044             |
|                       |           AI678570           |          BQ564699             |
|                       |             R91801           |          BF722297             |
|                       |           BG289563           |          AA607843             |
|                       |           BG528659           |          AA617584             |
|                       |           AI245666           |          BY114091             |
|                       |           AA581860           |          BX519290             |
|                       |           AA604478           |          BQ564432             |
|                       |           BF688313           |          CF900349             |
|                       |           BG470644           |          CA944168             |
|                       |             R02778           |          CK334273             |
|                       |           BE076333           |          CA546481             |
|                       |           BI199233           |          CK334272             |
|                       |           CN337473           |          AA000207             |
|                       |           AA164629           |          AA231604             |
|                       |           BG113570           |          AA124214             |
|                       |           BG745075           |          BE854198             |
|                       |           AI961621           |            W77710             |
|                       |           BM782584           |          AA104162             |
|                       |           AV717636           |          BF122754             |
|                       |           BE620858           |          BG867225             |
|                       |           BE874686           |          BI683785             |
|                       |           AA301744           |          BU961751             |
|                       |           AU151492           |          CA531801             |
|                       |           BU955077           |          AA562224             |
|                       |           BG391864           |          AA061069             |
|                       |             R81314           |          BE309131             |
|                       |           CN337428           |          AA116365             |
|                       |           CB999078           |          BI112965             |
|                       |           AA857510           |          AA562884             |
|                       |           AA565994           |          CN711129             |
|                       |             H78542           |          CB847594             |
|                       |           BQ429681           |          CB181616             |
|                       |           BM710755           |          BG961900             |
|                       |           AW364023           |          BU961625             |
|                       |           BI860340           |            W10701             |
|                       |           BF570109           |          AA445059             |
|                       |           BF689104           |          CD772058             |
|                       |           AA715010           |          BF538548             |
|                       |           BE181914           |          CK788931             |
|                       |             W87316           |          CN707612             |
|                       |           BM983566           |            W56921             |
|                       |           BG771040           |          BE281768             |
|                       |           BM782611           |          AA473517             |
|                       |           CR741332           |          BE655497             |
|                       |           AW373889           |          AA645902             |
|                       |           AW373926           |          AA222575             |
|                       |           AA978233           |          AA717349             |
|                       |           BE092648           |          BE368689             |
|                       |           AW373826           |          AA546821             |
|                       |           BF091057           |          AA125575             |
|                       |           BI086033           |          BU583347             |
|                       |             R89343           |          BI852234             |
|                       |           BF802577           |          AA681784             |
|                       |           AA662628           |          CA563240             |
|                       |           BE767029           |          BI151601             |
|                       |           BE181981           |          CB271151             |
|                       |           BE748726           |          BI852614             |
|                       |           BE075711           |          AA399911             |
|                       |           BG995961           |          AA438162             |
|                       |           AW821323           |          BG868021             |
|                       |           AA665483           |          AI172909             |
|                       |           BE092640           |          BQ895055             |
|                       |           BG995959           |          BI693159             |
|                       |           AW867929           |          AA154026             |
|                       |           AW821318           |          BQ715869             |
|                       |           AA055154           |          CN681165             |
|                       |           AA932305           |          BY052765             |
|                       |           BM790254           |          CD565263             |
|                       |           BG388696           |          BG968254             |
|                       |           BF857421           |          BE373250             |
|                       |           BF688879           |          BY170796             |
|                       |           BE206451           |          CN679150             |
|                       |           BE207378           |          BE306788             |
|                       |           BE300919           |          CF895534             |
|                       |           BG254609           |          BY171307             |
|                       |           BU742077           |          AA562058             |
|                       |           BU620046           |          CB573604             |
|                       |           AW360804           |          BF142223             |
|                       |           AW360839           |          BF119815             |
|                       |           BE182068           |          BI112580             |
|                       |           BM740900           |          CB183014             |
|                       |           BI049715           |          CA577695             |
|                       |           AW801117           |          BE554304             |
|                       |           AA678465           |          AL118182             |
|                       |             D55392           |            W70720             |
|                       |           AW602171           |          AA647192             |
|                       |           BM824061           |          CA564495             |
|                       |           BE568472           |          AW825745             |
|                       |           AW602061           |          AW228470             |
|                       |           CD522522           |          BF150369             |
|                       |           CD640463           |          BF320193             |
|                       |           CD639509           |          BI664748             |
|                       |           BG035245           |          CN664459             |
|                       |           BG167537           |          BE284496             |
|                       |           CD000056           |          BE334067             |
|                       |           BE300198           |          AA798579             |
|                       |           BQ429372           |          BI151546             |
|                       |           BG697873           |          CF172010             |
|                       |           BG615515           |          BQ885755             |
|                       |           BU153312           |          CF731912             |
|                       |           BI223230           |          BQ566780             |
|                       |           BU188942           |          AA645194             |
|                       |           BM012450           |          BU924069             |
|                       |           BM554286           |          CF732155             |
|                       |           BF807435           |          BE288913             |
|                       |           BF699151           |          BE305473             |
|                       |           BG027172           |          BI077662             |
|                       |           BE881684           |          BF537197             |
|                       |           BG755414           |          BF166500             |
|                       |           BE814061           |          BG923425             |
|                       |           BF094580           |          CN708556             |
|                       |           BE934227           |          CA979659             |
|                       |             T58697           |          AA089243             |
|                       |             N57395           |          AA537244             |
|                       |           AA186452           |          CN840595             |
|                       |           BF131898           |          BI653429             |
|                       |           BE300284           |          AA717160             |
|                       |           BU166429           |          BI152449             |
|                       |           BQ220892           |          CA562460             |
|                       |           BU941668           |          AA498941             |
|                       |           AA626429           |          BF100303             |
|                       |           BF971209           |          AA921604             |
|                       |           BG291180           |          BQ877826             |
|                       |             Z48501           |          AA529434             |
|                       |           AA092856           |          BF722956             |
|                       |           BE769451           |          AA718342             |
|                       |           BE786510           |          BE308565             |
|                       |           BF526901           |          BG247032             |
|                       |           AI783491           |          BG872635             |
|                       |           AI268897           |          BI688124             |
|                       |           AI277053           |          BI083537             |
|                       |           AA598879           |          BQ109076             |
|                       |           AI174409           |          CA564461             |
|                       |           AI973296           |          BF536608             |
|                       |           AA962698           |          BE306232             |
|                       |           BE092649           |          BI527053             |
|                       |           AA228675           |          AA473398             |
|                       |           AI758179           |            W77696             |
|                       |           AI445946           |          BE288448             |
|                       |           AI952803           |          CB233091             |
|                       |           AI884317           |            D77035             |
|                       |           BG574485           |          AA560874             |
|                       |           AI061469           |          BU938003             |
|                       |           BM454879           |          BF136004             |
|                       |           BF212804           |          AA672755             |
|                       |           BE568073           |          BI689537             |
|                       |           BG031687           |          BI455425             |
|                       |           BG282821           |          AA041692             |
|                       |           BE735928           |          BI151490             |
|                       |           BM841320           |          CA569221             |
|                       |           CB107907           |          BI660961             |
|                       |           BU182712           |          AA591443             |
|                       |             H81953           |          BI650589             |
|                       |           BI010480           |          AA763426             |
|                       |           BM740762           |          AI120821             |
|                       |           BE182064           |          BG806209             |
|                       |           BG484807           |          BF450254             |
|                       |           BE769458           |          BM877751             |
|                       |           BF242855           |          BF017819             |
|                       |           BG169641           |          CF724570             |
|                       |           BE770412           |          BE371695             |
|                       |           BF698571           |          AW647619             |
|                       |           BU954656           |          CD298064             |
|                       |           BE872905           |          BI661655             |
|                       |           AA354960           |          AA546448             |
|                       |           BM263029           |          AA929663             |
|                       |           BM700242           |          AA666785             |
|                       |           BE073646           |            W75448             |
|                       |           CN337508           |          BE914014             |
|                       |           CN337419           |          BI851562             |
|                       |           BU854093           |          AA098209             |
|                       |           BG290624           |          AA068210             |
|                       |           BI869910           |          BI143789             |
|                       |           BF762601           |          CK789929             |
|                       |           BQ683429           |          BI454447             |
|                       |           BM781623           |          BF723461             |
|                       |           BF028695           |          CF750187             |
|                       |           BG114061           |          BE916175             |
|                       |           BE934225           |          AA529650             |
|                       |           BE179020           |          CF982244             |
|                       |           AA720553           |            W65980             |
|                       |           BP383100           |          BI466681             |
|                       |           BG493872           |          AA638251             |
|                       |           BE778432           |          CF749811             |
|                       |             D54884           |          BU707621             |
|                       |           BU153338           |          BI555312             |
|                       |           AL045289           |          BQ892197             |
|                       |           BG960561           |          AA674118             |
|                       |           CD694875           |          BI154842             |
|                       |           BI860174           |          BE381173             |
|                       |           BI861286           |          BE371854             |
|                       |           BE177478           |          BF168629             |
|                       |           CN337453           |          BE375420             |
|                       |           BM849625           |          BF163055             |
|                       |           BE177475           |          BF124450             |
|                       |           BF744753           |            W62769             |
|                       |           BM851063           |          AW213137             |
|                       |           BE825329           |          AA117203             |
|                       |           BQ084301           |          AA667343             |
|                       |           BF902447           |          BU054537             |
|                       |           BG575414           |          CD562755             |
|                       |           BF895655           |          BI650924             |
|                       |           AA730686           |          AW681636             |
|                       |           BG950171           |          CK793007             |
|                       |           BE082717           |          CK620131             |
|                       |           CD523105           |          BF152001             |
|                       |           BE876908           |          BF142267             |
|                       |           BG477737           |          BE380948             |
|                       |           BE881014           |          BQ950334             |
|                       |           BG030557           |          BF539370             |
|                       |             T97414           |          AA144201             |
|                       |           BQ370958           |          AA589633             |
|                       |           BF244115           |          BF137159             |
|                       |           BU561449           |          BI079928             |
|                       |           BQ677801           |          AA794553             |
|                       |           BG680494           |          BF535375             |
|                       |           BG756831           |          BQ930649             |
|                       |           AW836794           |          AA072830             |
|                       |           AW836785           |          BF180805             |
|                       |           AW836796           |          BE368783             |
|                       |           BE716315           |          BQ895211             |
|                       |           BE018112           |          BF462055             |
|                       |           BG437318           |          BQ178917             |
|                       |           BG036487           |          BI694987             |
|                       |           BE904467           |          AA543742             |
|                       |           BE908911           |          BY353887             |
|                       |           BM463569           |          BU517887             |
|                       |           BG677672           |          BF168165             |
|                       |           AA451770           |          AI119754             |
|                       |           AW937640           |          BU924507             |
|                       |           BF696249           |          BF536479             |
|                       |           BE769034           |          BQ937220             |
|                       |           BG179997           |          BE284878             |
|                       |           AW364849           |          BQ944976             |
|                       |           AW972657           |          BF730472             |
|                       |           AA526328           |          BQ936397             |
|                       |           BG574970           |          AA855593             |
|                       |           AA639905           |          BG100984             |
|                       |           CB157491           |          AA183483             |
|                       |           BF796205           |          BF098651             |
|                       |           CF618810           |          BG915176             |
|                       |           BE768991           |          BF134971             |
|                       |           AW404258           |          BF138300             |
|                       |           BF242490           |          BE376636             |
|                       |           BG542074           |          BF166814             |
|                       |           BG986263           |          CK791045             |
|                       |           BG497621           |          CA976738             |
|                       |           BU941912           |          AA109586             |
|                       |           BG034680           |          BQ955659             |
|                       |           BU164796           |          BE372013             |
|                       |           CA952811           |          BF731484             |
|                       |           BG117571           |          BU517402             |
|                       |           BG392696           |          CN702941             |
|                       |           AW605274           |          CN687637             |
|                       |           AW378229           |          CF750726             |
|                       |           BG614686           |          AK005009             |
|                       |           BM830643           |          CN660955             |
|                       |           CB163813           |          BE370922             |
|                       |           CB163820           |          BQ952818             |
|                       |           BM792171           |          BI985858             |
|                       |           BM700216           |          BU517403             |
|                       |           AW370154           |          AA162612             |
|                       |           AW370158           |          CF165162             |
|                       |           AW378195           |          AW230834             |
|                       |           CN337506           |          CA976249             |
|                       |           CN337458           |          CA563930             |
|                       |           CN337454           |          CA541790             |
|                       |           CF122489           |          BQ180059             |
|                       |           BQ324208           |          BU519390             |
|                       |           BM765234           |          BM963071             |
|                       |           CN337360           |          CN689169             |
|                       |             H87678           |          CK342407             |
|                       |           CF121370           |          CK391073             |
|                       |           BX446614           |          BE308562             |
|                       |           AW803149           |          CF161750             |
|                       |           BM822810           |          AA547472             |
|                       |           BU781871           |          BI155316             |
|                       |           BE897613           |          BU510866             |
|                       |           BM465021           |          CA984523             |
|                       |           AW937581           |          CN723027             |
|                       |             D52674           |          AW244645             |
|                       |           BE770509           |          BF022583             |
|                       |           CN337371           |          CD562539             |
|                       |           BG534984           |          CA983814             |
|                       |           AJ713560           |          BF150597             |
|                       |           BU940080           |          CB273844             |
|                       |           BU073114           |          AW322904             |
|                       |           AA355172           |          CK391033             |
|                       |             W32370           |          BQ963514             |
|                       |             C06054           |          BF147119             |
|                       |           BM148331           |          BY271013             |
|                       |           BM153525           |          BY732143             |
|                       |           BE247165           |          BU522396             |
|                       |           BM193017           |          BQ921335             |
|                       |           BM193496           |          BU510291             |
|                       |           BE247783           |          BU513361             |
|                       |           BM150796           |          BU510871             |
|                       |           AA248326           |          BU520426             |
|                       |             H88545           |          BQ956481             |
|                       |           BU782785           |          BQ951203             |
|                       |           BU074145           |          BF022422             |
|                       |           BU071108           |          CD553277             |
|                       |           BE092540           |          CF163939             |
|                       |           BU786829           |          CF165467             |
|                       |           BM875457           |          CD561538             |
|                       |           BU784427           |          CD562725             |
|                       |           BM510645           |          CF162835             |
|                       |           BM511956           |          CF748996             |
|                       |           BI711395           |          BU939155             |
|                       |           BU783075           |          CA321217             |
|                       |           BM505558           |          BU709068             |
|                       |           BU164548           |          BQ444029             |
|                       |           BM509235           |          CA319404             |
|                       |           BF362382           |          BQ951314             |
|                       |           BE935928           |          BY291553             |
|                       |           BU902345           |          BQ928100             |
|                       |           BG753999           |          CB228104             |
|                       |           BM786815           |          CA321148             |
|                       |           BM423842           |            W35997             |
|                       |           CF147262           |            W99071             |
|                       |           BG250217           |          CK391086             |
|                       |           BF679968           |          BY107578             |
|                       |           BQ128385           |          BY110596             |
|                       |           BM510535           |          BQ922746             |
|                       |           BQ478288           |          BY078639             |
|                       |           AV655070           |          CA318470             |
|                       |           AI693138           |          BQ946993             |
|                       |           BG108172           |          CK794900             |
|                       |           BE770480           |          CF739173             |
|                       |           BG290681           |          BY780960             |
|                       |           BE770650           |          BU925237             |
|                       |           BE770654           |          BU743831             |
|                       |           BE817153           |          CF748114             |
|                       |           BE770625           |          CA320208             |
|                       |           BE817207           |          BG802965             |
|                       |           BG996149           |          CA879028             |
|                       |           BF964710           |          CA319410             |
|                       |           BM794448           |          BU702431             |
|                       |           BU168887           |          CF736086             |
|                       |           AU123742           |          BQ571418             |
|                       |           BM844957           |          AI592982             |
|                       |           BE716739           |          BQ571451             |
|                       |           BM083641           |          AA727531             |
|                       |           BE817115           |          AA727586             |
|                       |           BQ128124           |          CF169294             |
|                       |           AW582223           |          BU923755             |
|                       |           BM831422           |          BY738934             |
|                       |           CB156808           |          CF104617             |
|                       |           CB141005           |          BI985308             |
|                       |           CB124890           |          BY744757             |
|                       |           BI087892           |          BC003928             |
|                       |             N84965           |          CD354202             |
|                       |           CN337478           |          CA316864             |
|                       |           BQ722495           |          BM936224             |
|                       |           BM824047           |          BM507793             |
|                       |           CF146778           |          BU743932             |
|                       |           AW370173           |                               |
|                       |           CB119241           |                               |
|                       |           BG194961           |                               |
|                       |             D54883           |                               |
|                       |           BF155905           |                               |
|                       |           BM503383           |                               |
|                       |           BI520052           |                               |
|                       |           BU940757           |                               |
|                       |           AA055802           |                               |
|                       |           AA149735           |                               |
|                       |           AA169413           |                               |
|                       |           AA101952           |                               |
|                       |           AA169408           |                               |
|                       |           AI460110           |                               |
|                       |           AA670111           |                               |
|                       |           AW877233           |                               |
|                       |           AA634269           |                               |
|                       |           AA143799           |                               |
|                       |           AA773337           |                               |
|                       |           CB116920           |                               |
|                       |           BF770814           |                               |
|                       |           BU658567           |                               |
|                       |           BG389554           |                               |
|                       |           CN337441           |                               |
|                       |           CB118242           |                               |
|                       |           BM742549           |                               |
|                       |           BP206094           |                               |
|                       |           BI491794           |                               |
|                       |           BF382832           |                               |
|                       |           BP205583           |                               |
|                       |           BI858634           |                               |
|                       |           BG778247           |                               |
|                       |           BI855967           |                               |
|                       |           BU185030           |                               |
|                       |           BG575334           |                               |
|                       |           AW238638           |                               |
|                       |           BG960559           |                               |
|                       |           BU781694           |                               |
|                       |           BG675169           |                               |
|                       |           BF244821           |                               |
|                       |           AA146755           |                               |
|                       |           BI195429           |                               |
|                       |           AW370176           |                               |
|                       |           BM728168           |                               |
|                       |             W05161           |                               |
|                       |           BE407658           |                               |
|                       |           BQ425111           |                               |
|                       |           BF796552           |                               |
|                       |           BU169673           |                               |
|                       |           AW629885           |                               |
|                       |           BF676394           |                               |
|                       |           BU154047           |                               |
|                       |           BM559028           |                               |
|                       |           BU942204           |                               |
|                       |           BE716527           |                               |
|                       |           BG960544           |                               |
|                       |           BF184070           |                               |
|                       |           BQ324242           |                               |
|                       |             W21143           |                               |
|                       |           AW877236           |                               |
|                       |           AU135997           |                               |
|                       |           AA456362           |                               |
|                       |           BF240962           |                               |
|                       |           BF214254           |                               |
|                       |           AW362963           |                               |
|                       |           BF692082           |                               |
|                       |           BU603114           |                               |
|                       |           BE894831           |                               |
|                       |           BM723974           |                               |
|                       |           BF796195           |                               |
|                       |           BG542296           |                               |
|                       |           BM454263           |                               |
|                       |           BG178774           |                               |
|                       |           BQ424926           |                               |
|                       |           BG741382           |                               |
|                       |           BG574785           |                               |
|                       |           BM558391           |                               |
|                       |             H66293           |                               |
|                       |           BU858961           |                               |
|                       |           BM467934           |                               |
|                       |           BU507863           |                               |
|                       |           AW877248           |                               |
|                       |           BU183563           |                               |
|                       |           BM472356           |                               |
|                       |           AA130974           |                               |
|                       |           BG256323           |                               |
|                       |           AA355003           |                               |
|                       |           CN337488           |                               |
|                       |           AA384516           |                               |
|                       |           AJ706287           |                               |
|                       |           BG680411           |                               |
|                       |           CD685157           |                               |
|                       |           CD522223           |                               |
|                       |           CN337522           |                               |
|                       |           BE385466           |                               |
|                       |           AW404148           |                               |
|                       |           BE070457           |                               |
|                       |           BF737731           |                               |
|                       |             R76347           |                               |
|                       |           BP357249           |                               |
|                       |           BM844980           |                               |
|                       |           BP357216           |                               |
|                       |           CD640069           |                               |
|                       |           AA736555           |                               |
|                       |           BG178203           |                               |
|                       |           BG765344           |                               |
|                       |           BQ425595           |                               |
|                       |           BF808771           |                               |
|                       |           BM723769           |                               |
|                       |           CD615088           |                               |
|                       |           CD615089           |                               |
|                       |             R84494           |                               |
|                       |           BG429557           |                               |
|                       |           BM820345           |                               |
|                       |           AW389418           |                               |
|                       |           BI223789           |                               |
|                       |           BE693410           |                               |
|                       |           AW361316           |                               |
|                       |           BM083650           |                               |
|                       |           BG167474           |                               |
|                       |           AA641280           |                               |
|                       |           BM844970           |                               |
|                       |           AA047638           |                               |
|                       |           BP226392           |                               |
|                       |           BF804474           |                               |
|                       |           BP205681           |                               |
|                       |             H47968           |                               |
|                       |           BG950076           |                               |
|                       |           AJ706531           |                               |
|                       |           BX114073           |                               |
|                       |           BP307062           |                               |
|                       |           BU178419           |                               |
|                       |           BM741003           |                               |
|                       |           CN337457           |                               |
|                       |           BG950078           |                               |
|                       |           AV701319           |                               |
|                       |           BG256791           |                               |
|                       |           BG500925           |                               |
|                       |           BG425744           |                               |
|                       |           BG495238           |                               |
|                       |           CF618811           |                               |
|                       |           BQ226722           |                               |
|                       |           BP340012           |                               |
|                       |           BG287739           |                               |
|                       |           AW364143           |                               |
|                       |           AW880851           |                               |
|                       |           BG254634           |                               |
|                       |           CF141467           |                               |
|                       |           BE838573           |                               |
|                       |           CK004746           |                               |
|                       |           BM015205           |                               |
|                       |           BM696444           |                               |
|                       |           BF724515           |                               |
|                       |           AA357170           |                               |
|                       |           CF141007           |                               |
|                       |           BM765340           |                               |
|                       |           AI904588           |                               |
|                       |           AI904596           |                               |
|                       |           AA305144           |                               |
|                       |           BM829376           |                               |
|                       |           BQ635882           |                               |
|                       |           CF145721           |                               |
|                       |           BU657074           |                               |
|                       |           CB162911           |                               |
|                       |           CB119464           |                               |
|                       |           CB128860           |                               |
|                       |           CB161972           |                               |
|                       |           BM837948           |                               |
|                       |           CB135079           |                               |
|                       |           BM851524           |                               |
|                       |           AW245499           |                               |
|                       |           CB157598           |                               |
|                       |           BM823460           |                               |
|                       |           BE908828           |                               |
|                       |           BE873310           |                               |
|                       |           BG292242           |                               |
|                       |           BF089555           |                               |
|                       |           AW630470           |                               |
|                       |           CF597232           |                               |
|                       |           BM558788           |                               |
|                       |           BI335734           |                               |
|                       |           CN337471           |                               |
|                       |           AW882842           |                               |
|                       |           AW630652           |                               |
|                       |           CF139428           |                               |
|                       |           BM511480           |                               |
|                       |           CB135082           |                               |
|                       |           BE885159           |                               |
|                       |           BG422433           |                               |
|                       |           BF791774           |                               |
|                       |           AU136827           |                               |
|                       |           BQ220571           |                               |
|                       |           BG403786           |                               |
|                       |           BQ683490           |                               |
|                       |           BU173313           |                               |
|                       |           BM831852           |                               |
|                       |           AW408826           |                               |
|                       |           BF571800           |                               |
|                       |           CN337477           |                               |
|                       |           BG029087           |                               |
|                       |           BG178543           |                               |
|                       |           BM824227           |                               |
|                       |           BF217186           |                               |
|                       |           BQ050940           |                               |
|                       |             N83528           |                               |
|                       |             R94309           |                               |
|                       |           AA128091           |                               |
|                       |           CD357519           |                               |
|                       |           AW853987           |                               |
|                       |           AW675213           |                               |
|                       |           CB986249           |                               |
|                       |           BG026178           |                               |
|                       |           BQ896756           |                               |
|                       |           BI858575           |                               |
|                       |           BI058470           |                               |
|                       |           BU196834           |                               |
|                       |           CN337434           |                               |
|                       |           CN337422           |                               |
|                       |           BI871494           |                               |
|                       |           BM477817           |                               |
|                       |           AW386935           |                               |
|                       |           BM875706           |                               |
|                       |           BF089541           |                               |
|                       |           BU788096           |                               |
|                       |           AW327415           |                               |
|                       |           BQ434898           |                               |
|                       |           BU167577           |                               |
|                       |           BG535394           |                               |
|                       |           CD656778           |                               |
|                       |           CN337489           |                               |
|                       |           BG389121           |                               |
|                       |           BE005610           |                               |
|                       |           AW950026           |                               |
|                       |           BG752051           |                               |
|                       |           BG176638           |                               |
|                       |           BG750352           |                               |
|                       |           AW853992           |                               |
|                       |           BE715201           |                               |
|                       |           BF089751           |                               |
|                       |           BM694749           |                               |
|                       |           CF139464           |                               |
|                       |           AW364729           |                               |
|                       |           BG613662           |                               |
|                       |           BQ927103           |                               |
|                       |           BG260992           |                               |
|                       |           CD559218           |                               |
|                       |           BE905857           |                               |
|                       |           BF810935           |                               |
|                       |           AW382362           |                               |
|                       |           BM722633           |                               |
|                       |           BM834667           |                               |
|                       |           CD520305           |                               |
|                       |             R97387           |                               |
|                       |           BM742535           |                               |
|                       |           BQ433531           |                               |
|                       |           AW877249           |                               |
|                       |           BI047658           |                               |
|                       |           BG287086           |                               |
|                       |           AW877232           |                               |
|                       |           AW997148           |                               |
|                       |           AW364794           |                               |
|                       |           BE392870           |                               |
|                       |           BG287563           |                               |
|                       |           AW879750           |                               |
|                       |           BG028361           |                               |
|                       |           BQ223987           |                               |
|                       |           BE904439           |                               |
|                       |           BG623577           |                               |
|                       |           CB956759           |                               |
|                       |           BM016023           |                               |
|                       |           BG249473           |                               |
|                       |           CB119901           |                               |
|                       |           BG108284           |                               |
|                       |           AW991865           |                               |
|                       |           AA383348           |                               |
|                       |             H58283           |                               |
|                       |           BM794810           |                               |
|                       |           BG177948           |                               |
|                       |           AW392186           |                               |
|                       |           BQ373339           |                               |
|                       |           AA354542           |                               |
|                       |           BG032517           |                               |
|                       |           BM457563           |                               |
|                       |           BP383612           |                               |
|                       |           CN337431           |                               |
|                       |           CD242841           |                               |
|                       |           BU934301           |                               |
|                       |           AW877244           |                               |
|                       |           AA307640           |                               |
|                       |           BM765504           |                               |
|                       |           CB119549           |                               |
|                       |           BM708494           |                               |
|                       |           CB114299           |                               |
|                       |           BM787830           |                               |
|                       |           AA040127           |                               |
|                       |           BM824836           |                               |
|                       |           CB120256           |                               |
|                       |           CB136177           |                               |
|                       |           BI862827           |                               |
|                       |           AW403908           |                               |
|                       |           BQ935103           |                               |
|                       |           BU656469           |                               |
|                       |           AW991872           |                               |
|                       |           AW368360           |                               |
|                       |           CD639606           |                               |
|                       |           BU183632           |                               |
|                       |           BF815028           |                               |
|                       |           BG504873           |                               |
|                       |           BE536138           |                               |
|                       |           BG114212           |                               |
|                       |           BF988685           |                               |
|                       |           AW609643           |                               |
|                       |           AW391487           |                               |
|                       |           BF760630           |                               |
|                       |           BU164498           |                               |
|                       |           BG255279           |                               |
|                       |           CA388622           |                               |
|                       |           BQ322195           |                               |
|                       |           BG749453           |                               |
|                       |           CB127377           |                               |
|                       |           AA385679           |                               |
|                       |           AW937764           |                               |
|                       |           AW577431           |                               |
|                       |           AW951460           |                               |
|                       |           BG674360           |                               |
|                       |           BF091376           |                               |
|                       |           BF214410           |                               |
|                       |           BF760091           |                               |
|                       |           BF760086           |                               |
|                       |           CN337361           |                               |
|                       |           BE958550           |                               |
|                       |           AW379512           |                               |
|                       |           BI014989           |                               |
|                       |           BU945248           |                               |
|                       |           BM724563           |                               |
|                       |             H29711           |                               |
|                       |           BM724524           |                               |
|                       |           BQ355030           |                               |
|                       |           CF145802           |                               |
|                       |           BF766583           |                               |
|                       |           AW366834           |                               |
|                       |           AW369837           |                               |
|                       |           BG255828           |                               |
|                       |           AA354017           |                               |
|                       |           BF155128           |                               |
|                       |           BQ320610           |                               |
|                       |           BQ320627           |                               |
|                       |           BF856599           |                               |
|                       |           AW373895           |                               |
|                       |           AW373932           |                               |
|                       |           BM765917           |                               |
|                       |           BE300137           |                               |
|                       |           BI862205           |                               |
|                       |           CF146243           |                               |
|                       |           AV742500           |                               |
|                       |           CN337399           |                               |
|                       |           AA488549           |                               |
|                       |           BQ320623           |                               |
|                       |           AA373184           |                               |
|                       |           BF797318           |                               |
|                       |           BQ320354           |                               |
|                       |           BG391263           |                               |
|                       |           BF326505           |                               |
|                       |           BE748053           |                               |
|                       |           BE907307           |                               |
|                       |           BF757370           |                               |
|                       |           BF815447           |                               |
|                       |           BE828845           |                               |
|                       |           AW994351           |                               |
|                       |             R57142           |                               |
|                       |           AA172010           |                               |
|                       |           BF218431           |                               |
|                       |           AW994438           |                               |
|                       |           BI253273           |                               |
|                       |           BF104094           |                               |
|                       |           BF817249           |                               |
|                       |           BF814829           |                               |
|                       |           BF810092           |                               |
|                       |           BF814837           |                               |
|                       |           BF814847           |                               |
|                       |           BF815448           |                               |
|                       |           BF817337           |                               |
|                       |           BF817346           |                               |
|                       |           BE152160           |                               |
|                       |           AW994063           |                               |
|                       |           AA096267           |                               |
|                       |           BM798128           |                               |
|                       |             F00028           |                               |
|                       |           BF819194           |                               |
|                       |           AW381866           |                               |
|                       |           AW381890           |                               |
|                       |           BF817250           |                               |
|                       |           BF815444           |                               |
|                       |           BF814844           |                               |
|                       |           BF813596           |                               |
|                       |           BF815424           |                               |
|                       |           BF817316           |                               |
|                       |           BF899213           |                               |
|                       |           BM691362           |                               |
|                       |           AW391111           |                               |
|                       |           AW390926           |                               |
|                       |           AW390928           |                               |
|                       |           AW391043           |                               |
|                       |           AW391064           |                               |
|                       |           AW391102           |                               |
|                       |           AW391107           |                               |
|                       |           AW391114           |                               |
|                       |           AW609409           |                               |
|                       |           AW609534           |                               |
|                       |           AW609371           |                               |
|                       |           BE074557           |                               |
|                       |           BQ478536           |                               |
|                       |           AW373888           |                               |
|                       |           AW373925           |                               |
|                       |           AW994292           |                               |
|                       |           AW577450           |                               |
|                       |             T12081           |                               |
|                       |           BE931585           |                               |
|                       |           BF087179           |                               |
|                       |           BF087126           |                               |
|                       |           BM764359           |                               |
|                       |           BM697208           |                               |
|                       |           CF145528           |                               |
|                       |           BF983285           |                               |
|                       |           CN337414           |                               |
|                       |           BU845270           |                               |
|                       |             R16426           |                               |
|                       |             H48615           |                               |
|                       |           BF084895           |                               |
|                       |           BF326502           |                               |
|                       |           AW991763           |                               |
|                       |           BE928824           |                               |
|                       |           BE693413           |                               |
|                       |           BF084903           |                               |
|                       |           BE828611           |                               |
|                       |           BE828747           |                               |
|                       |           CN337443           |                               |
|                       |           BP429059           |                               |
|                       |           BF028968           |                               |
|                       |           AW886530           |                               |
|                       |           BG502970           |                               |
|                       |           BG170473           |                               |
|                       |           BE612818           |                               |
|                       |           BG497264           |                               |
|                       |           BU508576           |                               |
|                       |             T85891           |                               |
|                       |             T82182           |                               |
|                       |           AA360635           |                               |
|                       |             T83096           |                               |
|                       |             R52568           |                               |
|                       |           BG024968           |                               |
|                       |           CD559319           |                               |
|                       |           BM460320           |                               |
|                       |             R00372           |                               |
|                       |             R84632           |                               |
|                       |           AW609400           |                               |
|                       |             T19116           |                               |
|                       |           BF092964           |                               |
|                       |           AA353721           |                               |
|                       |             T78260           |                               |
|                       |           BG959684           |                               |
|                       |           AA055853           |                               |
|                       |           BE928826           |                               |
|                       |           BF184278           |                               |
|                       |           BU198486           |                               |
|                       |           CB160578           |                               |
|                       |           BG527977           |                               |
|                       |           BU177684           |                               |
|                       |           BG329257           |                               |
|                       |           BG610845           |                               |
|                       |           BG612694           |                               |
|                       |           BU941561           |                               |
|                       |           BU557828           |                               |
|                       |           BQ424443           |                               |
|                       |           BG740954           |                               |
|                       |           BF336689           |                               |
|                       |           BI015688           |                               |
|                       |             R02777           |                               |
|                       |           AW582191           |                               |
|                       |           BF570576           |                               |
|                       |           AW368986           |                               |
|                       |           CD640693           |                               |
|                       |           BG113866           |                               |
|                       |           BE735542           |                               |
|                       |           CF780735           |                               |
|                       |           BU845230           |                               |
|                       |           CD521521           |                               |
|                       |           BM458271           |                               |
|                       |           CN337384           |                               |
|                       |             H70509           |                               |
|                       |           AA007138           |                               |
|                       |           BF337482           |                               |
|                       |           BF911660           |                               |
|                       |             T26965           |                               |
|                       |           BE744031           |                               |
|                       |           BF326560           |                               |
|                       |           BF796286           |                               |
|                       |           BG751627           |                               |
|                       |           CD642159           |                               |
|                       |           AW994049           |                               |
|                       |           BG024652           |                               |
|                       |           BG256875           |                               |
|                       |           AW577432           |                               |
|                       |           CN337416           |                               |
|                       |           BM475577           |                               |
|                       |           BQ439961           |                               |
|                       |           BU502360           |                               |
|                       |           BG489170           |                               |
|                       |           BQ930862           |                               |
|                       |           BF081633           |                               |
|                       |           AA094409           |                               |
|                       |           BF684191           |                               |
|                       |           AW361305           |                               |
|                       |           BG468435           |                               |
|                       |           AW352430           |                               |
|                       |           BG163414           |                               |
|                       |           BU553470           |                               |
|                       |           AA343261           |                               |
|                       |           BG999692           |                               |
|                       |           BI861340           |                               |
|                       |           BF089789           |                               |
|                       |           BI710943           |                               |
|                       |           BF084891           |                               |
|                       |           BG995727           |                               |
|                       |           BF807586           |                               |
|                       |           AW391025           |                               |
|                       |           BF089766           |                               |
|                       |           AW387313           |                               |
|                       |           BF828082           |                               |
|                       |           BF829091           |                               |
|                       |           BM838356           |                               |
|                       |           AW403925           |                               |
|                       |           AW387306           |                               |
|                       |           CN337472           |                               |
|                       |           BF761206           |                               |
|                       |           AW351629           |                               |
|                       |           AW351589           |                               |
|                       |           AW375254           |                               |
|                       |           BQ213154           |                               |
|                       |           BM853735           |                               |
|                       |           BE841908           |                               |
|                       |           BG989508           |                               |
|                       |           BG989175           |                               |
|                       |           AW375313           |                               |
|                       |           BE161947           |                               |
|                       |           AW375311           |                               |
|                       |           CF142454           |                               |
|                       |           BG999651           |                               |
|                       |           BI037423           |                               |
|                       |           AW351611           |                               |
|                       |           AW351630           |                               |
|                       |           AW375294           |                               |
|                       |           AW375308           |                               |
|                       |           AW351628           |                               |
|                       |           AW351621           |                               |
|                       |           AW351627           |                               |
|                       |           AW351641           |                               |
|                       |           AW375230           |                               |
|                       |           BE841987           |                               |
|                       |           AW351587           |                               |
|                       |           AW375282           |                               |
|                       |           AW352420           |                               |
|                       |           AW375240           |                               |
|                       |           AW375261           |                               |
|                       |           AW375276           |                               |
|                       |           AW387295           |                               |
|                       |           AW375223           |                               |
|                       |           BE183475           |                               |
|                       |           AW375290           |                               |
|                       |           BI012908           |                               |
|                       |           BG990481           |                               |
|                       |           BG989176           |                               |
|                       |           BG989505           |                               |
|                       |           BG990322           |                               |
|                       |           BG990325           |                               |
|                       |           BI054267           |                               |
|                       |           BE182563           |                               |
|                       |           BE895938           |                               |
|                       |             F06465           |                               |
|                       |             W19520           |                               |
|                       |           CD641591           |                               |
|                       |           AW375259           |                               |
|                       |           BU181688           |                               |
|                       |           BE841902           |                               |
|                       |           BG494305           |                               |
|                       |           AW365259           |                               |
|                       |           AW375258           |                               |
|                       |           AW375281           |                               |
|                       |           BM756414           |                               |
|                       |           CD520400           |                               |
|                       |           AW365267           |                               |
|                       |           AW367371           |                               |
|                       |           AW365258           |                               |
|                       |           AW375275           |                               |
|                       |           AW375283           |                               |
|                       |           BE841918           |                               |
|                       |           AW179182           |                               |
|                       |           AW351612           |                               |
|                       |           AW351602           |                               |
|                       |           BE841897           |                               |
|                       |           AW351600           |                               |
|                       |           AW375260           |                               |
|                       |           AW375278           |                               |
|                       |           BE841901           |                               |
|                       |           BE841988           |                               |
|                       |           AW375286           |                               |
|                       |           AW375228           |                               |
|                       |           AA639154           |                               |
|                       |           AW365255           |                               |
|                       |           BE813878           |                               |
|                       |           BE865997           |                               |
|                       |           AW351632           |                               |
|                       |           AW375241           |                               |
|                       |           AW178254           |                               |
|                       |           BF374382           |                               |
|                       |           AW179193           |                               |
|                       |           AW179100           |                               |
|                       |           AW365270           |                               |
|                       |           AW366041           |                               |
|                       |           BE841904           |                               |
|                       |           AW351595           |                               |
|                       |           AW387303           |                               |
|                       |           AW351623           |                               |
|                       |           AW176995           |                               |
|                       |           AW375233           |                               |
|                       |           BE841885           |                               |
|                       |           AW365224           |                               |
|                       |           AW365236           |                               |
|                       |           AW365265           |                               |
|                       |           AW365260           |                               |
|                       |           AW176985           |                               |
|                       |           AW179096           |                               |
|                       |           AW179098           |                               |
|                       |           AW179099           |                               |
|                       |           AW179101           |                               |
|                       |           AW179183           |                               |
|                       |           AW179185           |                               |
|                       |           AW179188           |                               |
|                       |           AW179189           |                               |
|                       |           AW352429           |                               |
|                       |           AW365253           |                               |
|                       |           AW375256           |                               |
|                       |           AW375268           |                               |
|                       |           AW387237           |                               |
|                       |           AW387242           |                               |
|                       |           AW387245           |                               |
|                       |           AW179184           |                               |
|                       |           AW365266           |                               |
|                       |           BE841887           |                               |
|                       |           AW375247           |                               |
|                       |           AW179092           |                               |
|                       |           AW179095           |                               |
|                       |           AW365244           |                               |
|                       |           AW387239           |                               |
|                       |           AW375243           |                               |
|                       |           AW365222           |                               |
|                       |           AW375218           |                               |
|                       |           AW375231           |                               |
|                       |           AW375265           |                               |
|                       |           AW375285           |                               |
|                       |           AW365254           |                               |
|                       |           AW375253           |                               |
|                       |           BE841915           |                               |
|                       |           BI012912           |                               |
|                       |           CB114534           |                               |
|                       |           CB122511           |                               |
|                       |           CB153836           |                               |
|                       |           CB267413           |                               |
|                       |           AW375255           |                               |
|                       |           BI091847           |                               |
|                       |           BE073567           |                               |
|                       |           CN337366           |                               |
|                       |           BG531712           |                               |
|                       |           BE719951           |                               |
|                       |             R08103           |                               |
|                       |           BF817591           |                               |
|                       |           BE769473           |                               |
|                       |           BG978441           |                               |
|                       |           BG257745           |                               |
|                       |           BG990728           |                               |
|                       |           AA360985           |                               |
|                       |           CF146479           |                               |
|                       |           BM750976           |                               |
|                       |           BU783320           |                               |
|                       |           CD692346           |                               |
|                       |           BF724560           |                               |
|                       |           BE790196           |                               |
|                       |           BE927245           |                               |
|                       |           BI223374           |                               |
|                       |             N90367           |                               |
|                       |           AW351588           |                               |
|                       |           BE086049           |                               |
|                       |           BE834087           |                               |
|                       |           BF806938           |                               |
|                       |           BG767331           |                               |
|                       |           BG757224           |                               |
|                       |           BE251640           |                               |
|                       |           AW835294           |                               |
|                       |           BU182269           |                               |
|                       |           BM511364           |                               |
|                       |           BF087118           |                               |
|                       |           BF856529           |                               |
|                       |           BF857593           |                               |
|                       |           CN337481           |                               |
|                       |           BE841894           |                               |
|                       |           AA328751           |                               |
|                       |           BE841906           |                               |
|                       |           BG951256           |                               |
|                       |             N80634           |                               |
|                       |             H22813           |                               |
|                       |           AW375229           |                               |
|                       |             N99628           |                               |
|                       |           BI010170           |                               |
|                       |           CN337474           |                               |
|                       |             H94776           |                               |
|                       |           BF027746           |                               |
|                       |           BF571744           |                               |
|                       |           BU188196           |                               |
|                       |           BE781074           |                               |
|                       |           CD639334           |                               |
|                       |           BG426976           |                               |
|                       |           BF795921           |                               |
|                       |           BU933717           |                               |
|                       |           BE879399           |                               |
|                       |           BI869120           |                               |
|                       |           BU855325           |                               |
|                       |           BE250179           |                               |
|                       |           BU175341           |                               |
|                       |           BG113996           |                               |
|                       |           BG114107           |                               |
|                       |           BG696583           |                               |
|                       |           BU158992           |                               |
|                       |           BQ437796           |                               |
|                       |           BU151333           |                               |
|                       |           BM909446           |                               |
|                       |             R18798           |                               |
|                       |             T99490           |                               |
|                       |             H74111           |                               |
|                       |           BG289402           |                               |
|                       |           BQ882619           |                               |
|                       |           BE870323           |                               |
|                       |           BF858821           |                               |
|                       |           BF819230           |                               |
|                       |           BG983658           |                               |
|                       |           BG031694           |                               |
|                       |           BQ939241           |                               |
|                       |           BF761511           |                               |
|                       |           BF761513           |                               |
|                       |           AW792797           |                               |
|                       |           AW994514           |                               |
|                       |           BE694103           |                               |
|                       |           BU558198           |                               |
|                       |           BP428496           |                               |
|                       |           AW351975           |                               |
|                       |           BE089927           |                               |
|                       |           BE089943           |                               |
|                       |           CD107780           |                               |
|                       |           BF879912           |                               |
|                       |           AW366010           |                               |
|                       |           AW366045           |                               |
|                       |           AL516266           |                               |
|                       |           BM512194           |                               |
|                       |           AW178255           |                               |
|                       |           BF761521           |                               |
|                       |           BG385958           |                               |
|                       |           AW351649           |                               |
|                       |           AW378900           |                               |
|                       |           BM790289           |                               |
|                       |           BU175448           |                               |
|                       |           BE546253           |                               |
|                       |           AA360252           |                               |
|                       |           CB115090           |                               |
|                       |           CB130952           |                               |
|                       |           BF808772           |                               |
|                       |           AA354673           |                               |
|                       |           BF754386           |                               |
|                       |           BM479602           |                               |
|                       |           CF145103           |                               |
|                       |           CF145183           |                               |
|                       |           AW351637           |                               |
|                       |           AW365262           |                               |
|                       |           BG959685           |                               |
|                       |           BF378071           |                               |
|                       |           AW991761           |                               |
|                       |           BE547225           |                               |
|                       |           CN337528           |                               |
|                       |           BG289799           |                               |
|                       |           AW351603           |                               |
|                       |             R58732           |                               |
|                       |           BG503381           |                               |
|                       |           BG610766           |                               |
|                       |           BG420266           |                               |
|                       |           BI868500           |                               |
|                       |           AW889409           |                               |
|                       |           BG827066           |                               |
|                       |           BQ308446           |                               |
|                       |           BF754247           |                               |
|                       |             R91800           |                               |
|                       |           CB119619           |                               |
|                       |           AW366042           |                               |
|                       |           CF145402           |                               |
|                       |           BE841916           |                               |
|                       |           BE277030           |                               |
|                       |           BF798487           |                               |
|                       |           AW351644           |                               |
|                       |           AW351618           |                               |
|                       |           BM833318           |                               |
|                       |           AU120398           |                               |
|                       |           BG758280           |                               |
|                       |           BE841880           |                               |
|                       |           BE018293           |                               |
|                       |           BE007981           |                               |
|                       |           AW366038           |                               |
|                       |           BG273007           |                               |
|                       |           BF763410           |                               |
|                       |           BF735548           |                               |
|                       |           BF753627           |                               |
|                       |           BG251713           |                               |
|                       |           BE546703           |                               |
|                       |           AW363068           |                               |
|                       |           AW380130           |                               |
|                       |           AW380127           |                               |
|                       |           AA056400           |                               |
|                       |           BM722385           |                               |
|                       |           BE007979           |                               |
|                       |           AA379760           |                               |
|                       |             H25628           |                               |
|                       |             H78922           |                               |
|                       |             H94770           |                               |
|                       |           AW853017           |                               |
|                       |           CD521498           |                               |
|                       |           CD638354           |                               |
|                       |           BF307393           |                               |
|                       |           BG106559           |                               |
|                       |           BG179936           |                               |
|                       |           AW994058           |                               |
|                       |           BF669183           |                               |
|                       |           CD522999           |                               |
|                       |           BG180063           |                               |
|                       |           BG164373           |                               |
|                       |           BG290663           |                               |
|                       |           BM914474           |                               |
|                       |           BQ439660           |                               |
|                       |           CB120615           |                               |
|                       |           BG389792           |                               |
|                       |           AA303852           |                               |
|                       |           BF804464           |                               |
|                       |           AW629892           |                               |
|                       |           BU158980           |                               |
|                       |           BG168524           |                               |
|                       |           BM556922           |                               |
|                       |           CD521244           |                               |
|                       |           BE545099           |                               |
|                       |           BE870115           |                               |
|                       |           BG167190           |                               |
|                       |           BQ685765           |                               |
|                       |           BG674726           |                               |
|                       |           BG502246           |                               |
|                       |           BE936821           |                               |
|                       |           BU164605           |                               |
|                       |           BM465826           |                               |
|                       |           BE074633           |                               |
|                       |           BE074701           |                               |
|                       |           BE074818           |                               |
|                       |           BG954305           |                               |
|                       |           BF247892           |                               |
|                       |           CD641741           |                               |
|                       |           BE729040           |                               |
|                       |             N56760           |                               |
|                       |           BQ581122           |                               |
|                       |           BI829651           |                               |
|                       |           BM015939           |                               |
|                       |           BM469785           |                               |
|                       |           CD520157           |                               |
|                       |           AW247021           |                               |
|                       |             C03993           |                               |
|                       |           BF091764           |                               |
|                       |             R52413           |                               |
|                       |             H94769           |                               |
|                       |             R56744           |                               |
|                       |           BU945540           |                               |
|                       |           CD639193           |                               |
|                       |           AW970202           |                               |
|                       |           CD243502           |                               |
|                       |           CA453240           |                               |
|                       |           CB265514           |                               |
|                       |           CB142422           |                               |
|                       |           BF087530           |                               |
|                       |           BE409925           |                               |
|                       |           BF906514           |                               |
|                       |           BF906505           |                               |
|                       |           BF906512           |                               |
|                       |           BF965855           |                               |
|                       |           BQ957236           |                               |
|                       |           BF900967           |                               |
|                       |           BG999876           |                               |
|                       |           BF901580           |                               |
|                       |             W02399           |                               |
|                       |           AU133537           |                               |
|                       |           CN337502           |                               |
|                       |           BQ212828           |                               |
|                       |           CF140901           |                               |
|                       |           CN337516           |                               |
|                       |           BG283387           |                               |
|                       |           BX452488           |                               |
|                       |           AW880773           |                               |
|                       |           BI870510           |                               |
|                       |           BE536914           |                               |
|                       |           BG289959           |                               |
|                       |           BQ888776           |                               |
|                       |           BM783473           |                               |
|                       |             H23386           |                               |
|                       |           BM761612           |                               |
|                       |           BG178257           |                               |
|                       |           BG117277           |                               |
|                       |           CF134784           |                               |
|                       |           AW965251           |                               |
|                       |           AA774116           |                               |
|                       |           BF895412           |                               |
|                       |           BG111597           |                               |
|                       |           BE905506           |                               |
|                       |           BG778629           |                               |
|                       |           BG259700           |                               |
|                       |           BE870928           |                               |
|                       |           BF528900           |                               |
|                       |           BG114005           |                               |
|                       |           BM043292           |                               |
|                       |           BM844941           |                               |
|                       |           BF856530           |                               |
|                       |           BF857594           |                               |
|                       |           BQ232511           |                               |
|                       |           AW860558           |                               |
|                       |           BM014059           |                               |
|                       |           BG576912           |                               |
|                       |           BQ230330           |                               |
|                       |           BF326516           |                               |
|                       |           BE073502           |                               |
|                       |           AW991755           |                               |
|                       |           AW991702           |                               |
|                       |           AW991866           |                               |
|                       |           CB143434           |                               |
|                       |           CD640093           |                               |
|                       |           BE295262           |                               |
|                       |           AW991874           |                               |
|                       |           AW991677           |                               |
|                       |           AW991754           |                               |
|                       |           AW991757           |                               |
|                       |           BM830059           |                               |
|                       |           AW991873           |                               |
|                       |           BG254488           |                               |
|                       |           BI253351           |                               |
|                       |           CN337437           |                               |
|                       |           BE788022           |                               |
|                       |           BF326504           |                               |
|                       |           AW991758           |                               |
|                       |           AW991759           |                               |
|                       |           AW991818           |                               |
|                       |           AW991820           |                               |
|                       |           AW991812           |                               |
|                       |           AW991756           |                               |
|                       |           BF326537           |                               |
|                       |           BF961684           |                               |
|                       |           AW991692           |                               |
|                       |           AW991698           |                               |
|                       |           AW991695           |                               |
|                       |           AW991864           |                               |
|                       |           AW991762           |                               |
|                       |           BQ323680           |                               |
|                       |           AW366857           |                               |
|                       |           BG987065           |                               |
|                       |           AL039301           |                               |
|                       |           BU182368           |                               |
|                       |           BQ324126           |                               |
|                       |           CN337385           |                               |
|                       |           BM772017           |                               |
|                       |           CD641324           |                               |
|                       |           BQ340456           |                               |
|                       |           CD522671           |                               |
|                       |             T26991           |                               |
|                       |           AW991694           |                               |
|                       |           CD698900           |                               |
|                       |           AU142547           |                               |
|                       |           AW991703           |                               |
|                       |           BM754441           |                               |
|                       |           BG391486           |                               |
|                       |           BP353759           |                               |
|                       |             F07481           |                               |
|                       |           BM843789           |                               |
|                       |           AW629674           |                               |
|                       |           AA376845           |                               |
|                       |           AU126405           |                               |
|                       |           BM802230           |                               |
|                       |           BE007977           |                               |
|                       |           BE297659           |                               |
|                       |           BG286918           |                               |
|                       |           BF836762           |                               |
|                       |           BM845665           |                               |
|                       |           BE789087           |                               |
|                       |           AA354644           |                               |
|                       |           AU133548           |                               |
|                       |           AW991696           |                               |
|                       |           CD243989           |                               |
|                       |           BG403516           |                               |
|                       |           BM014931           |                               |
|                       |           BE276928           |                               |
|                       |           AW994287           |                               |
|                       |           BM463251           |                               |
|                       |           BG911934           |                               |
|                       |           BI224142           |                               |
|                       |           AW603235           |                               |
|                       |           CD641304           |                               |
|                       |           BU179090           |                               |
|                       |           BG253239           |                               |
|                       |           BF754014           |                               |
|                       |           AW577898           |                               |
|                       |           BF794515           |                               |
|                       |           BG386439           |                               |
|                       |           BU156277           |                               |
|                       |           AA382206           |                               |
|                       |           AW860531           |                               |
|                       |           BG284026           |                               |
|                       |           CD615086           |                               |
|                       |           CD615087           |                               |
|                       |           CD615085           |                               |
|                       |           BE007803           |                               |
|                       |           BM820219           |                               |
|                       |           BM904457           |                               |
|                       |             W39453           |                               |
|                       |           AW991690           |                               |
|                       |           AW994498           |                               |
|                       |           BE694086           |                               |
|                       |           CD615083           |                               |
|                       |           CK430635           |                               |
|                       |           BG402796           |                               |
|                       |           BF360546           |                               |
|                       |           AW991706           |                               |
|                       |           BI861581           |                               |
|                       |           BG818425           |                               |
|                       |           BU194034           |                               |
|                       |             D56258           |                               |
|                       |           BI049946           |                               |
|                       |           BF900978           |                               |
|                       |           BE007975           |                               |
|                       |           BM312678           |                               |
|                       |           AW881152           |                               |
|                       |           AU123929           |                               |
|                       |           AW577884           |                               |
|                       |           BG260503           |                               |
|                       |           AW840946           |                               |
|                       |           BF674792           |                               |
|                       |           BE729717           |                               |
|                       |           BG255676           |                               |
|                       |           AW881178           |                               |
|                       |           AA314666           |                               |
|                       |           BE073561           |                               |
|                       |           BE182478           |                               |
|                       |           BE182475           |                               |
|                       |           AW889079           |                               |
|                       |             H73529           |                               |
|                       |           AA347167           |                               |
|                       |           BF923312           |                               |
|                       |           BF932319           |                               |
|                       |           AW880952           |                               |
|                       |           BG283151           |                               |
|                       |           BG390750           |                               |
|                       |           BG680608           |                               |
|                       |           BQ422537           |                               |
|                       |           CD695896           |                               |
|                       |           BU189268           |                               |
|                       |           BM458927           |                               |
|                       |             R12837           |                               |
|                       |           BE763142           |                               |
|                       |           BM759521           |                               |
|                       |           CN337480           |                               |
|                       |             T81927           |                               |
|                       |           BG292450           |                               |
|                       |           BE938231           |                               |
|                       |           BE779572           |                               |
|                       |           AU138952           |                               |
|                       |           AW796484           |                               |
|                       |           BM842356           |                               |
|                       |           CN337410           |                               |
|                       |           BU940958           |                               |
|                       |           BF063918           |                               |
|                       |           BM698253           |                               |
|                       |           BE182934           |                               |
|                       |           BQ421409           |                               |
|                       |           BM752964           |                               |
|                       |           BE298624           |                               |
|                       |           BM745790           |                               |
|                       |           BE388558           |                               |
|                       |           BQ224445           |                               |
|                       |           BU149994           |                               |
|                       |           CN337530           |                               |
|                       |           BG027492           |                               |
|                       |           AW607662           |                               |
|                       |           BI003679           |                               |
|                       |           BF856516           |                               |
|                       |           BE091927           |                               |
|                       |           AA366534           |                               |
|                       |           AA165091           |                               |
|                       |           BE889224           |                               |
|                       |           CD246021           |                               |
|                       |           AW966746           |                               |
|                       |           BG286251           |                               |
|                       |           AL042203           |                               |
|                       |           AW991870           |                               |
|                       |           AA149671           |                               |
|                       |           AA313472           |                               |
|                       |           AW994190           |                               |
|                       |           CD640393           |                               |
|                       |           BF036341           |                               |
|                       |           BI222754           |                               |
|                       |           BM742143           |                               |
|                       |             T91469           |                               |
|                       |           AA318252           |                               |
|                       |           CN337486           |                               |
|                       |           AA180522           |                               |
|                       |           CN337401           |                               |
|                       |           BI859580           |                               |
|                       |           BU601371           |                               |
|                       |           BF857577           |                               |
|                       |           BP873503           |                               |
|                       |           BE410495           |                               |
|                       |           BP872672           |                               |
|                       |           BG282516           |                               |
|                       |           BE709599           |                               |
|                       |           BE709619           |                               |
|                       |           BM508927           |                               |
|                       |           BE827277           |                               |
|                       |           AA114086           |                               |
|                       |           CN337468           |                               |
|                       |           BG034566           |                               |
|                       |           CN337427           |                               |
|                       |           BG256828           |                               |
|                       |           CN337395           |                               |
|                       |           BM048042           |                               |
|                       |           BF336690           |                               |
|                       |           AU127022           |                               |
|                       |           BF926658           |                               |
|                       |           CD615084           |                               |
|                       |           AW991815           |                               |
|                       |           CF139406           |                               |
|                       |           CN337418           |                               |
|                       |           BG779258           |                               |
|                       |           BM763046           |                               |
|                       |           CB115779           |                               |
|                       |           BP339426           |                               |
|                       |           BF992485           |                               |
|                       |           CN337400           |                               |
|                       |           BQ710887           |                               |
|                       |           AA368385           |                               |
|                       |           AW373829           |                               |
|                       |           AW662957           |                               |
|                       |           BM842513           |                               |
|                       |           AU142721           |                               |
|                       |           BM014135           |                               |
|                       |           BG178667           |                               |
|                       |           BM694949           |                               |
|                       |           BM844012           |                               |
|                       |           BG111408           |                               |
|                       |           BG477830           |                               |
|                       |           BM745274           |                               |
|                       |           BM843685           |                               |
|                       |           BM847102           |                               |
|                       |           BE257930           |                               |
|                       |           BM790345           |                               |
|                       |           CD723783           |                               |
|                       |           BI031407           |                               |
|                       |           CF132640           |                               |
|                       |           CN337439           |                               |
|                       |           CB119420           |                               |
|                       |           CN337417           |                               |
|                       |           CN337467           |                               |
|                       |           BM712335           |                               |
|                       |           BF795484           |                               |
|                       |           BQ721667           |                               |
|                       |           BM800957           |                               |
|                       |           BQ719624           |                               |
|                       |           BU161743           |                               |
|                       |           BE001285           |                               |
|                       |           AA351478           |                               |
|                       |             H65411           |                               |
|                       |           CN337479           |                               |
|                       |           BM755071           |                               |
|                       |           AW951688           |                               |
|                       |           AW630762           |                               |
|                       |           CB161919           |                               |
|                       |           CN337450           |                               |
|                       |           BE275841           |                               |
|                       |           BM818992           |                               |
|                       |           BF663952           |                               |
|                       |           BE890002           |                               |
|                       |           CD640565           |                               |
|                       |           BG506161           |                               |
|                       |           BM843013           |                               |
|                       |           BU941219           |                               |
|                       |           CD615082           |                               |
|                       |           BQ431484           |                               |
|                       |           AL037098           |                               |
|                       |           BF527247           |                               |
|                       |           AW937830           |                               |
|                       |           BG828476           |                               |
|                       |           BF973633           |                               |
|                       |           BG324618           |                               |
|                       |           BP343506           |                               |
|                       |           BF027398           |                               |
|                       |           AU128158           |                               |
|                       |           BG034158           |                               |
|                       |           BE813300           |                               |
|                       |           AA313454           |                               |
|                       |           BF310798           |                               |
|                       |           BI856143           |                               |
|                       |           BE018798           |                               |
|                       |           BM708647           |                               |
|                       |           BM831646           |                               |
|                       |           AA148183           |                               |
|                       |           BE825235           |                               |
|                       |           BG256380           |                               |
|                       |           BM451808           |                               |
|                       |           BG325995           |                               |
|                       |           AW239021           |                               |
|                       |           AA356958           |                               |
|                       |           AL046512           |                               |
|                       |           BM745305           |                               |
|                       |           BE149210           |                               |
|                       |           BU600466           |                               |
|                       |           BM843167           |                               |
|                       |           BG954971           |                               |
|                       |           BG255955           |                               |
|                       |           CD643042           |                               |
|                       |           CA392964           |                               |
|                       |           AA101951           |                               |
|                       |           BE929965           |                               |
|                       |           CD692014           |                               |
|                       |           BE867379           |                               |
|                       |           BG422030           |                               |
|                       |           BF772080           |                               |
|                       |           BG951263           |                               |
|                       |             R85578           |                               |
|                       |           AA326658           |                               |
|                       |           BE782806           |                               |
|                       |           AW505477           |                               |
|                       |           AV656479           |                               |
|                       |           BE007807           |                               |
|                       |           BF570600           |                               |
|                       |           BF326501           |                               |
|                       |           BE742031           |                               |
|                       |           BG754946           |                               |
|                       |           CD104054           |                               |
|                       |           BF083891           |                               |
|                       |           BE019283           |                               |
|                       |           BG163597           |                               |
|                       |             W40155           |                               |
|                       |             H22721           |                               |
|                       |             R87216           |                               |
|                       |             T69963           |                               |
|                       |           BQ221269           |                               |
|                       |           BE175660           |                               |
|                       |           AW390295           |                               |
|                       |           BQ215320           |                               |
|                       |           BQ223048           |                               |
|                       |           BG284427           |                               |
|                       |           BF974158           |                               |
|                       |           BE207641           |                               |
|                       |           BG105292           |                               |
|                       |           BM845400           |                               |
|                       |           CN337398           |                               |
|                       |           BM818983           |                               |
|                       |           BQ944875           |                               |
|                       |           BF828084           |                               |
|                       |           BE277013           |                               |
|                       |           BF753189           |                               |
|                       |           BG280909           |                               |
|                       |           BG757929           |                               |
|                       |           BM917086           |                               |
|                       |           BE881158           |                               |
|                       |           CN337518           |                               |
|                       |           AW410874           |                               |
|                       |           AA351480           |                               |
|                       |           BE087823           |                               |
|                       |           BE293130           |                               |
|                       |           BM008803           |                               |
|                       |           AA306256           |                               |
|                       |           AW991867           |                               |
|                       |           BM457243           |                               |
|                       |           AA360664           |                               |
|                       |           BM541312           |                               |
|                       |           BE543004           |                               |
|                       |           BF663796           |                               |
|                       |           BQ941608           |                               |
|                       |           BF904896           |                               |
|                       |           BE707561           |                               |
|                       |           BM746198           |                               |
|                       |           AW884536           |                               |
|                       |           AW991869           |                               |
|                       |           BE085978           |                               |
|                       |           AW367838           |                               |
|                       |           BM844143           |                               |
|                       |             W74246           |                               |
|                       |           BM843892           |                               |
|                       |           AW630408           |                               |
|                       |           AW402083           |                               |
|                       |           BM905052           |                               |
|                       |           AA385613           |                               |
|                       |           BP335785           |                               |
|                       |           BP240734           |                               |
|                       |           BP278020           |                               |
|                       |           CN337494           |                               |
|                       |           BP241617           |                               |
|                       |           CB146453           |                               |
|                       |           AW673311           |                               |
|                       |           BU594831           |                               |
|                       |           AW401662           |                               |
|                       |           AA285253           |                               |
|                       |           BU187238           |                               |
|                       |           AV760515           |                               |
|                       |           BM706126           |                               |
|                       |           BM697436           |                               |
|                       |           BF569466           |                               |
|                       |           CN337476           |                               |
|                       |           BG754868           |                               |
|                       |           BG686870           |                               |
|                       |           BF326522           |                               |
|                       |           BP266547           |                               |
|                       |           BG291064           |                               |
|                       |           AA331402           |                               |
|                       |           AA367226           |                               |
|                       |             R19420           |                               |
|                       |           BF899829           |                               |
|                       |           BF834832           |                               |
|                       |           BF835535           |                               |
|                       |           BG341710           |                               |
|                       |           BF906415           |                               |
|                       |           BP306284           |                               |
|                       |           CD722321           |                               |
|                       |           BQ953080           |                               |
|                       |           CN337393           |                               |
|                       |           BF326534           |                               |
|                       |           BP352308           |                               |
|                       |           AA361741           |                               |
|                       |           AA152064           |                               |
|                       |           BG759033           |                               |
|                       |           BG389708           |                               |
|                       |           CD688361           |                               |
|                       |           BQ439062           |                               |
|                       |           BG753601           |                               |
|                       |           BG035806           |                               |
|                       |           BG701588           |                               |
|                       |           BF326521           |                               |
|                       |           BP232877           |                               |
|                       |           BP336165           |                               |
|                       |           CA393901           |                               |
|                       |           CN337372           |                               |
|                       |           AW991819           |                               |
|                       |           AI906314           |                               |
|                       |           BP205117           |                               |
|                       |           AW991821           |                               |
|                       |           AW991825           |                               |
|                       |           BG338777           |                               |
|                       |           BG032114           |                               |
|                       |           BF326523           |                               |
|                       |           BM755085           |                               |
|                       |           AA312133           |                               |
|                       |           BE872186           |                               |
|                       |           BQ056465           |                               |
|                       |           AA171870           |                               |
|                       |           AA188750           |                               |
|                       |           BE296600           |                               |
|                       |           BP336430           |                               |
|                       |           AW504728           |                               |
|                       |           BP335217           |                               |
|                       |           BP336605           |                               |
|                       |           BP276788           |                               |
|                       |           BE873137           |                               |
|                       |           BG027420           |                               |
|                       |           BG751891           |                               |
|                       |           BF308216           |                               |
|                       |           AA056307           |                               |
|                       |           AA377926           |                               |
|                       |           BE543637           |                               |
|                       |           AV760524           |                               |
|                       |           AW630107           |                               |
|                       |           CD696127           |                               |
|                       |           BP336655           |                               |
|                       |           BF817729           |                               |
|                       |           AW991822           |                               |
|                       |           BF748388           |                               |
|                       |           BF748393           |                               |
|                       |           CB143959           |                               |
|                       |             H65031           |                               |
|                       |           BG746378           |                               |
|                       |           CN337523           |                               |
|                       |           AW991697           |                               |
|                       |           AW368104           |                               |
|                       |           AW368108           |                               |
|                       |           AW991814           |                               |
|                       |           AW991705           |                               |
|                       |           BP336877           |                               |
|                       |           BP333015           |                               |
|                       |           AW991701           |                               |
|                       |           BP279568           |                               |
|                       |           BP333012           |                               |
|                       |           BP336391           |                               |
|                       |           BP336096           |                               |
|                       |           BP283548           |                               |
|                       |           BP221271           |                               |
|                       |           BP336291           |                               |
|                       |           CD171735           |                               |
|                       |           BF229881           |                               |
|                       |           BP344303           |                               |
|                       |           BF856561           |                               |
|                       |           AW991693           |                               |
|                       |           BF794881           |                               |
|                       |           CN337496           |                               |
|                       |           BQ212218           |                               |
|                       |           BQ672283           |                               |
|                       |           CD244169           |                               |
|                       |           BQ933553           |                               |
|                       |           BQ213232           |                               |
|                       |           BF361469           |                               |
|                       |           BE293124           |                               |
|                       |           BG419003           |                               |
|                       |           BF887740           |                               |
|                       |           BG824981           |                               |
|                       |           CN337465           |                               |
|                       |           AW991764           |                               |
|                       |           BX409530           |                               |
|                       |           AW991744           |                               |
|                       |           AW327694           |                               |
|                       |           AW991750           |                               |
|                       |           CD704491           |                               |
|                       |           BU195904           |                               |
|                       |           BQ938580           |                               |
|                       |           AW991760           |                               |
|                       |           CN483373           |                               |
|                       |             T62784           |                               |
|                       |           BX439647           |                               |
|                       |           AW991765           |                               |
|                       |           BG257047           |                               |
|                       |           CN337526           |                               |
|                       |           BU568749           |                               |
|                       |           BP283808           |                               |
|                       |           BI200044           |                               |
|                       |           BF899836           |                               |
|                       |           BM798420           |                               |
|                       |           AA486626           |                               |
|                       |           BE817209           |                               |
|                       |           BE770644           |                               |
|                       |           BE817119           |                               |
|                       |           BE716730           |                               |
|                       |           BE817157           |                               |
|                       |           BE817208           |                               |
|                       |           BE817210           |                               |
|                       |           BP284241           |                               |
|                       |           BE770638           |                               |
|                       |           BP336567           |                               |
|                       |           BE765177           |                               |
|                       |           BP243684           |                               |
|                       |           BE087883           |                               |
|                       |           CN337529           |                               |
|                       |           AL041925           |                               |
|                       |           BE709552           |                               |
|                       |           BF747871           |                               |
|                       |           CB158918           |                               |
|                       |           BG979098           |                               |
|                       |           AW389788           |                               |
|                       |           BF082452           |                               |
|                       |           BF760221           |                               |
|                       |           BG722385           |                               |
|                       |           CN337459           |                               |
|                       |           BF760208           |                               |
|                       |           BU190270           |                               |
|                       |           BF740416           |                               |
|                       |           CN337423           |                               |
|                       |           BP236187           |                               |
|                       |           CD557283           |                               |
|                       |           BM456769           |                               |
|                       |           BM469638           |                               |
|                       |           BM479856           |                               |
|                       |           BU190177           |                               |
|                       |           BE764994           |                               |
|                       |           BM468845           |                               |
|                       |           BQ228068           |                               |
|                       |           BQ431555           |                               |
|                       |           AL514512           |                               |
|                       |           BM045350           |                               |
|                       |           AU127351           |                               |
|                       |           BX462268           |                               |
|                       |           AW392233           |                               |
|                       |           BP337886           |                               |
|                       |           AA380413           |                               |
|                       |           BP247559           |                               |
|                       |           BP239289           |                               |
|                       |           BX425759           |                               |
|                       |           BP359268           |                               |
|                       |           BF760658           |                               |
|                       |           BU186706           |                               |
|                       |           BP235593           |                               |
|                       |           BP234488           |                               |
|                       |           CN337381           |                               |
|                       |           AW835277           |                               |
|                       |           CN482807           |                               |
|                       |           BE772339           |                               |
|                       |           BE772358           |                               |
|                       |           BU152814           |                               |
|                       |           AL534225           |                               |
|                       |           AW630219           |                               |
|                       |           BX441450           |                               |
|                       |           BP334988           |                               |
|                       |           CN337413           |                               |
|                       |           BM466295           |                               |
|                       |           BX426375           |                               |
|                       |           AA384795           |                               |
|                       |           BM802601           |                               |
|                       |           AU131963           |                               |
|                       |           AJ705243           |                               |
|                       |           BG979783           |                               |
|                       |           BQ441761           |                               |
|                       |           CN337517           |                               |
|                       |           AA422010           |                               |
|                       |           BU156428           |                               |
|                       |           BM016949           |                               |
|                       |           BM478549           |                               |
|                       |           BQ961536           |                               |
|                       |           CD557195           |                               |
|                       |           BX421439           |                               |
|                       |           BM469146           |                               |
|                       |           BQ071816           |                               |
|                       |           BQ881432           |                               |
|                       |           BM479394           |                               |
|                       |           BP234248           |                               |
|                       |           BU838890           |                               |
|                       |           BM473629           |                               |
|                       |           BQ231287           |                               |
|                       |           BU176181           |                               |
|                       |           BM479829           |                               |
|                       |           BQ949101           |                               |
|                       |           BU194059           |                               |
|                       |           BM560587           |                               |
|                       |           BI463279           |                               |
|                       |           BQ225994           |                               |
|                       |           CD245095           |                               |
|                       |           BQ880553           |                               |
|                       |           BQ723562           |                               |
|                       |           CN337521           |                               |
|                       |           AW408794           |                               |
|                       |           BQ430741           |                               |
|                       |           BU177130           |                               |
|                       |           BQ921108           |                               |
|                       |           BM464198           |                               |
|                       |           BU194122           |                               |
|                       |           BQ233214           |                               |
|                       |           BQ421200           |                               |
|                       |           CN337425           |                               |
|                       |           BQ430439           |                               |
|                       |           BQ438222           |                               |
|                       |           BX443310           |                               |
|                       |           BU542834           |                               |
|                       |           BX405087           |                               |
|                       |           BM480246           |                               |
|                       |           BU521356           |                               |
|                       |           BQ437317           |                               |
|                       |           BX442179           |                               |
|                       |           CN337493           |                               |
|                       |           CN337370           |                               |
|                       |           AA361898           |                               |
|                       |           BM456188           |                               |
|                       |           BU160394           |                               |
|                       |           CN337514           |                               |
|                       |           BQ423183           |                               |
|                       |           BQ228568           |                               |
|                       |           AW029381           |                               |
|                       |           BM683833           |                               |
|                       |           BQ439040           |                               |
|                       |           BM452975           |                               |
|                       |           BU192732           |                               |
|                       |           BU170089           |                               |
|                       |           AL537400           |                               |
|                       |           BQ437127           |                               |
|                       |           CD251820           |                               |
|                       |           BP220691           |                               |
|                       |           BQ220198           |                               |
|                       |           BU165479           |                               |
|                       |           AU124613           |                               |
|                       |           BG979102           |                               |
|                       |           CN337415           |                               |
|                       |           BQ423238           |                               |
|                       |           BU155928           |                               |
|                       |           AL516311           |                               |
|                       |           AA318103           |                               |
|                       |           BF767703           |                               |
|                       |           CN337379           |                               |
|                       |           BM542590           |                               |
|                       |           AW363310           |                               |
|                       |           CN337440           |                               |
|                       |           CN337475           |                               |
|                       |           BQ954187           |                               |
|                       |           CN337452           |                               |
|                       |           BM467038           |                               |
|                       |           BG025847           |                               |
|                       |           AU131877           |                               |
|                       |           BP274503           |                               |
|                       |           BF808662           |                               |
|                       |           CN337487           |                               |
|                       |           AW629981           |                               |
|                       |           BP265381           |                               |
|                       |           BE832875           |                               |
|                       |           CN337388           |                               |
|                       |           BM810659           |                               |
|                       |           BP333212           |                               |
|                       |           CN337430           |                               |
|                       |           CN337420           |                               |
|                       |           BM460537           |                               |
|                       |           BP327517           |                               |
|                       |           AW408097           |                               |
|                       |           CN337356           |                               |
|                       |           CN337358           |                               |
|                       |           CN337456           |                               |
|                       |           BX477530           |                               |
|                       |           CN337503           |                               |
|                       |           AU141619           |                               |
|                       |           CN337464           |                               |
|                       |           CN337402           |                               |
|                       |           CN337512           |                               |
|                       |           BQ639981           |                               |
|                       |           BU753115           |                               |
|                       |           BG720554           |                               |
|                       |           BI560325           |                               |
|                       |           BI559930           |                               |
|                       |           AW296579           |                               |
+-----------------------+------------------------------+-------------------------------+
|      #   55           | *Seq. mapped in strand Plus* | *Seq. mapped in strand Plus*  |
|                       |           AK023624           |          AY090614             |
|                       |           AB021179           |          BC022111             |
|                       |           BC006460           |          CO042276             |
|                       |           CR617663           |          CO042261             |
|                       |           CR614564           |          CO042287             |
|                       |           CN407456           |          AW457372             |
|                       |           CA417191           |          AA755213             |
|                       |           CA444673           |          CF926723             |
|                       |           CN479496           |          CK381334             |
|                       |           BU620746           |          CD679867             |
|                       |           BU752404           |          BU504607             |
|                       |           BU752472           |          AI841702             |
|                       |           CB853296           |          AK078644             |
|                       |           BU627749           |          BQ176374             |
|                       |           BU739439           |          BQ031488             |
|                       |           AA809264           |          BE947346             |
|                       |           AI394163           |          CF381678             |
|                       |           AI023500           |          AW047241             |
|                       |           AW296979           |                               |
|                       | *Seq. mapped in strand Minus*| *Seq. mapped in strand Minus* |
|                       |           AW204524           |          BQ175907             |
|                       |           AI811680           |          CB848887             |
|                       |           CA430432           |          AK052212             |
|                       |           BF446806           |                               |
|                       |           AI620761           |                               |
|                       |           AI219944           |                               |
+-----------------------+------------------------------+-------------------------------+
|      #   56           | *Seq. mapped in strand Plus* | *Seq. mapped in strand Plus*  |
|                       |           BC003159           |            D83999             |
|                       |           CR599625           |          BC002023             |
|                       |           CR620330           |                               |
|                       |             J05448           |                               |
|                       |           BC028157           |                               |
|                       |           AJ224144           |                               |
|                       |           AJ224143           |                               |
|                       |           CR600374           |                               |
|                       |           CR615542           |                               |
|                       |           CR600258           |                               |
|                       |           CR606923           |                               |
|                       |           BC000409           |                               |
|                       |           CR620622           |                               |
|                       |           CR542041           |                               |
|                       |           AF008443           |                               |
|                       |           CR594917           |                               |
|                       |           CR615834           |                               |
|                       |           CR626527           |                               |
|                       | *Seq. mapped in strand Minus*| *Seq. mapped in strand Minus* |
|                       |           CR623999           |          AK077612             |
|                       |           CR610258           |          BC004705             |
|                       |           BC001540           |          AF418207             |
|                       |           BC003541           |          AK032352             |
|                       |           CR608867           |                               |
|                       |           CR619149           |                               |
|                       |           AK001350           |                               |
|                       |           AF466369           |                               |
+-----------------------+------------------------------+-------------------------------+
|      #   57           | *Seq. mapped in strand Plus* | *Seq. mapped in strand Plus*  |
|                       |           BP228536           |          BB852612             |
|                       |           AK075382           |          BY792270             |
|                       |           CF993870           |          BY784908             |
|                       |           BP227463           |          BY788757             |
|                       |           BI669398           |          BB849305             |
|                       |           BI550728           |          BY289559             |
|                       |           BM146497           |          BY264610             |
|                       |           CB996629           |          CB231320             |
|                       |           CB996881           |          BY042997             |
|                       |           CB991979           |          BB586499             |
|                       |           BG529889           |          BY110415             |
|                       |           BP380008           |          BY792499             |
|                       |           BP212507           |          CB233275             |
|                       |           BI598550           |          AU050986             |
|                       |           BG701716           |          AA815658             |
|                       |           BM788036           |          BE948392             |
|                       |           BM172186           |          CA888322             |
|                       |           BG714144           |          CB236484             |
|                       |           BG709010           |          CB232195             |
|                       |           BG703581           |          CB231925             |
|                       |           BG748332           |          BB842995             |
|                       |           BP237793           |          AW912002             |
|                       |           BP378864           |          CB236372             |
|                       |           BX956245           |          AI316897             |
|                       |           BG757745           |          BY309990             |
|                       |           BG759989           |          BY146497             |
|                       |           BG756910           |          BY334251             |
|                       |           BI602693           |          BY786542             |
|                       |           BI597260           |          BY081472             |
|                       |           BI553110           |          BY312663             |
|                       |           BG426572           |          BY033349             |
|                       |           BM146142           |          BY095738             |
|                       |           BI670104           |          BY097797             |
|                       |           BG714762           |          BY266883             |
|                       |           CB961051           |          BY156446             |
|                       |           CB988816           |          BY279095             |
|                       |           BG548534           |          BY290451             |
|                       |           BP231424           |          BY260179             |
|                       |           BM839658           |          BY286334             |
|                       |           BG714399           |          BY268473             |
|                       |           BG702354           |          BY031642             |
|                       |           BG717413           |          BY738328             |
|                       |           AA194554           |          AA823046             |
|                       |           AF248966           |          AU035395             |
|                       |           AY038990           |          AA822963             |
|                       |           BP334987           |          AU080227             |
|                       |           BP354526           |          BG976388             |
|                       |           BI553543           |          AU079816             |
|                       |           BG720064           |          AW107107             |
|                       |           BG705507           |          BY274469             |
|                       |           BG702177           |          BI645981             |
|                       |           BI551171           |          BG863776             |
|                       |           BI547816           |          BY287015             |
|                       |           BI667962           |          CB596677             |
|                       |           BI552612           |          CK793277             |
|                       |           BI544956           |          BY790259             |
|                       |           BG719929           |          BY141846             |
|                       |           BG699243           |          BY787345             |
|                       |           BI461369           |          BY784257             |
|                       |           BI561588           |          AI876886             |
|                       |           BI545593           |          CB236843             |
|                       |           BF185484           |          BG298560             |
|                       |           BF666506           |          BY131103             |
|                       |           CB994132           |          BY302228             |
|                       |           BP276620           |          CB234478             |
|                       |           BP241040           |          CA545323             |
|                       |           BP227840           |          CB209302             |
|                       |           AU280668           |          BG916154             |
|                       |           BP276763           |          BU571357             |
|                       |           BP213329           |          AU080111             |
|                       |           BP276132           |          BU503818             |
|                       |           BP297536           |          AA755546             |
|                       |           BP210367           |          AK076495             |
|                       |           CB996364           |          BI733996             |
|                       |           BI667550           |          BI665810             |
|                       |           BI597465           |          BY320674             |
|                       |           BP280503           |          BB614185             |
|                       |           CB990533           |          BY351002             |
|                       |           CB996406           |          BB847462             |
|                       |           BI667418           |          CB237147             |
|                       |           CD385906           |          CF168973             |
|                       |           CN291360           |          BY123495             |
|                       |           BE312617           |          CB231825             |
|                       |           BM193854           |          CN708065             |
|                       |           BG703676           |            C88814             |
|                       |           AF291814           |          BU706946             |
|                       |           BI669246           |          BY160985             |
|                       |           BI549117           |          BI732052             |
|                       |           BG713982           |          BF784590             |
|                       |           BG714337           |          BI657661             |
|                       |           BG708218           |          BB859528             |
|                       |           BG716215           |          BB864663             |
|                       |           BI599716           |          BB864169             |
|                       |           BI554007           |          BB860377             |
|                       |           BI596873           |          BF178365             |
|                       |           BI551951           |          CF905294             |
|                       |           BI600190           |          CF904358             |
|                       |           BI600429           |          CF906049             |
|                       |           BI551798           |          CK617883             |
|                       |           BI596508           |          BY717274             |
|                       |           BP227620           |          CB234293             |
|                       |           BP227167           |          AK017482             |
|                       |           BP290790           |          BY126470             |
|                       |           BP372630           |          BY129000             |
|                       |           BP196483           |          BY123138             |
|                       |           BP270704           |          BY316835             |
|                       |           BP341537           |          BY320053             |
|                       |           BM751198           |          BY352323             |
|                       |           BG759126           |          BB841857             |
|                       |           BI552663           |          BY317415             |
|                       |           BU543595           |          BY260465             |
|                       |           BI457903           |          BI078312             |
|                       |           BI598368           |          BY173837             |
|                       |           BG705052           |          BY171700             |
|                       |           BM839240           |          BY080585             |
|                       |           BI667259           |          BY161959             |
|                       |           BI547777           |          BB630870             |
|                       |           BP211693           |          BY277374             |
|                       |           BI547699           |          CA493165             |
|                       |           BG699888           |          AU080392             |
|                       |           BI667797           |          AI226594             |
|                       |           BI553732           |          AA867880             |
|                       |           BI560770           |          AI931504             |
|                       |           BQ956599           |          AI527733             |
|                       |           BU556894           |          AW106795             |
|                       |           BI599385           |          BI685790             |
|                       |           BG703191           |          BE573270             |
|                       |           CB960730           |          BE533490             |
|                       |           CB960864           |          BE569483             |
|                       |           AA297471           |          AW988918             |
|                       |           AA298119           |          AU035148             |
|                       |           BI603333           |          CB237754             |
|                       |           BG773211           |          BY250731             |
|                       |           BF697773           |          AA645356             |
|                       |           BG705983           |          AW318610             |
|                       |           BI545697           |          AW318828             |
|                       |           BP353046           |          BF143123             |
|                       |           BP292300           |          BI250326             |
|                       |           AI092581           |          BF123569             |
|                       |           AI333667           |          AA277184             |
|                       |           BI755821           |          CB234637             |
|                       |           BP267890           |          BG172247             |
|                       |           AI880032           |          BG294880             |
|                       |           AI349539           |          BI329731             |
|                       |           BF431079           |          BQ715584             |
|                       |           AI366997           |          AW989004             |
|                       |           AI040265           |          BQ551095             |
|                       |           AW392222           |          BE853800             |
|                       |           AW003884           |          BI729740             |
|                       |           AY429341           |          AW259031             |
|                       |           AI589478           |          AW260017             |
|                       |           AI567231           |          BY267460             |
|                       |           AI682889           |          BY269941             |
|                       |           CB066889           |          BG966028             |
|                       |           AW438835           |          BG965853             |
|                       |           AA584287           |          CB233258             |
|                       |           CA388902           |          BE687794             |
|                       |           BP328025           |          BE851264             |
|                       |           BI468447           |          BG972466             |
|                       |           CA773546           |          BG519469             |
|                       |           BI764649           |          BQ928017             |
|                       |           BI603997           |          BF101430             |
|                       |             H07998           |          BI988188             |
|                       |           AA782669           |          BE569816             |
|                       |           AI804330           |          BG965935             |
|                       |           AI244079           |          BF719294             |
|                       |           AI244083           |          CD805868             |
|                       |           AI349540           |          BF011672             |
|                       |           AA025751           |          CF911760             |
|                       |           AI244926           |          BF468315             |
|                       |           AI084246           |          BU707267             |
|                       |             H86728           |          BY122811             |
|                       |           AA053862           |          BE303448             |
|                       |           AI352243           |          AK029405             |
|                       |           AI948455           |          BF119697             |
|                       |           AI349546           |          AA171248             |
|                       |           AI214761           |          AW741947             |
|                       |           AI034047           |          CD771692             |
|                       |           AA854083           |          CN539891             |
|                       |             R63080           |          BI078606             |
|                       |           BF058045           |          AA839653             |
|                       |           AA489232           |          BG173896             |
|                       |           AA878389           |          BU920947             |
|                       |             H12551           |          BE851218             |
|                       |             R44801           |          AI789021             |
|                       |             R66609           |          AI930319             |
|                       |           AA890540           |          BM943075             |
|                       |             W15286           |          BC014706             |
|                       |             H96859           |          BU709540             |
|                       |             R63665           |          BF100333             |
|                       |           AA846586           |          BE291891             |
|                       |           AI095352           |          AW413298             |
|                       |           AI022504           |          CK619748             |
|                       |           AA251015           |          AW744313             |
|                       |           AA844543           |          CA947798             |
|                       |             R79321           |          CF352899             |
|                       |           AI128976           |          CF908663             |
|                       |             N24900           |          BF786430             |
|                       |             N27425           |          AW985491             |
|                       |             R79812           |          BI104299             |
|                       |           AI366775           |          AA647140             |
|                       |           AI159809           |          CK624500             |
|                       |           AI075460           |          CF749485             |
|                       |           AA903999           |          BG298183             |
|                       |           AI242587           |          BE309388             |
|                       |           AI023956           |          CD773738             |
|                       |           BF732377           |          BG919050             |
|                       |           AA947478           |          CN707920             |
|                       |           AA988744           |          BB176707             |
|                       |           AA713478           |          BF149989             |
|                       |             W37530           |          BE374581             |
|                       |           AW166105           |          AW908431             |
|                       |           BF196461           |          BF166848             |
|                       |           AI817629           |          BI145899             |
|                       |           AA397898           |          BG244389             |
|                       |           AW024459           |          BE198768             |
|                       |           BF060652           |          BF020733             |
|                       |           AI493440           |          BY579357             |
|                       |           AA528178           |          CF105663             |
|                       |             R34849           |          BF788565             |
|                       |           AA890442           |          AI173768             |
|                       |           AI571215           |          AI789946             |
|                       |           AI028502           |          BQ177855             |
|                       |           AA298829           |          BG101010             |
|                       |           AA313317           |          BF100402             |
|                       |             W52608           |          AA763415             |
|                       |           AL049929           |                               |
|                       |           AA025912           |                               |
|                       |           BP341864           |                               |
|                       |           AA255919           |                               |
|                       |             W37670           |                               |
|                       |           CN291362           |                               |
|                       |             R17981           |                               |
|                       |             H65558           |                               |
|                       |             H10236           |                               |
|                       |           AA636009           |                               |
|                       |           BQ777336           |                               |
|                       |             N76218           |                               |
|                       |           AA393599           |                               |
|                       |           BP265486           |                               |
|                       |           BF087152           |                               |
|                       |           AV702380           |                               |
|                       |           BG530418           |                               |
|                       |           AA298825           |                               |
|                       |           AW958900           |                               |
|                       |             R77590           |                               |
|                       |           BE927639           |                               |
|                       |           CK301135           |                               |
|                       |             H59176           |                               |
|                       |           AV721438           |                               |
|                       |             H56745           |                               |
|                       |           CD251988           |                               |
|                       |           CB998477           |                               |
|                       |           BP368053           |                               |
|                       |           BG696765           |                               |
|                       |           BG403007           |                               |
|                       |           BP289916           |                               |
|                       |           BF214591           |                               |
|                       |           BP294801           |                               |
|                       |           AA416560           |                               |
|                       |             N41633           |                               |
|                       |           AV726680           |                               |
|                       |             F12487           |                               |
|                       |             R51426           |                               |
|                       |           BE967755           |                               |
|                       |             T74223           |                               |
|                       |           CN291365           |                               |
|                       |           BP367103           |                               |
|                       |           AA313617           |                               |
|                       |           AW951698           |                               |
|                       |           BX118820           |                               |
|                       |           CB123145           |                               |
|                       |           BC010395           |                               |
|                       |           AA084217           |                               |
|                       |           BI850231           |                               |
|                       |           BF665336           |                               |
|                       |           CN291363           |                               |
|                       |           BG288867           |                               |
|                       |             N35735           |                               |
|                       |           AA298831           |                               |
|                       |           BQ219111           |                               |
|                       |             H85240           |                               |
|                       |           BP228514           |                               |
|                       |           BE897106           |                               |
|                       |           BU679094           |                               |
|                       |           CB994486           |                               |
|                       |           BI334714           |                               |
|                       |             N48016           |                               |
|                       |           BI906526           |                               |
|                       |           BQ335419           |                               |
|                       |           CA432635           |                               |
|                       |           BU193939           |                               |
|                       |           CK904572           |                               |
|                       |           BG501143           |                               |
|                       |           AA298846           |                               |
|                       |             Y17975           |                               |
|                       |           BU607997           |                               |
|                       |           BM069679           |                               |
|                       |           BM054885           |                               |
|                       |           CR739577           |                               |
|                       |             N30541           |                               |
|                       |           CA776340           |                               |
|                       |           CA773328           |                               |
|                       |             N57460           |                               |
|                       |           BI468446           |                               |
|                       |           BG679828           |                               |
|                       |           AA164656           |                               |
|                       |           AA437350           |                               |
|                       |           BE709243           |                               |
|                       |           BG109874           |                               |
|                       |           AA298870           |                               |
|                       |           BG622445           |                               |
|                       |           BF570757           |                               |
|                       |           CK904571           |                               |
|                       |           BE707078           |                               |
|                       |           BE169352           |                               |
|                       |           BE707045           |                               |
|                       |           BQ788022           |                               |
|                       |           BG778430           |                               |
|                       |             W58241           |                               |
|                       |           BG714109           |                               |
|                       |           BG538574           |                               |
|                       |           AW392267           |                               |
|                       |             W42526           |                               |
|                       |           BF212134           |                               |
|                       |             N41531           |                               |
|                       |           BM054691           |                               |
|                       |           AA576267           |                               |
|                       |           AA255918           |                               |
|                       |           BM069457           |                               |
|                       |           AA417296           |                               |
|                       |           CD678490           |                               |
|                       |             N27239           |                               |
|                       |           BE827568           |                               |
|                       |             H96444           |                               |
|                       |           AA928947           |                               |
|                       |           BG104855           |                               |
|                       |           AA148013           |                               |
|                       |           AA298852           |                               |
|                       |           CV030058           |                               |
|                       |             W94541           |                               |
|                       |             R78826           |                               |
|                       |           BE068801           |                               |
|                       |           BP382958           |                               |
|                       |             T61972           |                               |
|                       |           AA773339           |                               |
|                       |           CA393493           |                               |
|                       |           AI983274           |                               |
|                       |           BI869290           |                               |
|                       |           AI797520           |                               |
|                       |           BE538275           |                               |
|                       |           AA825307           |                               |
|                       |           AA281406           |                               |
|                       |           AI200275           |                               |
|                       |           BG219537           |                               |
|                       |             N73409           |                               |
|                       |             R34946           |                               |
|                       | *Seq. mapped in strand Minus*| *Seq. mapped in strand Minus* |
|                       |           BU685665           |          AI844668             |
+-----------------------+------------------------------+-------------------------------+
|      #   58           | *Seq. mapped in strand Plus* | *Seq. mapped in strand Plus*  |
|                       |           BX248000           |          CF105039             |
|                       |           CR618016           |          BF168593             |
|                       |           CR626826           |          BY790421             |
|                       |           AK130167           |          AI226836             |
|                       |           BC014181           |          BY347100             |
|                       |           BC014483           |          CF540770             |
|                       |           CR609198           |          CB058076             |
|                       |           CR622209           |          CF983348             |
|                       |           CR610307           |          BY077979             |
|                       |           AF132944           |          BB860039             |
|                       |                              |          BB863092             |
|                       |                              |          BY093100             |
|                       |                              |          BY094414             |
|                       |                              |          BY247186             |
|                       |                              |          BY301251             |
|                       |                              |          BY086444             |
|                       |                              |          BY077800             |
|                       |                              |          BY274384             |
|                       |                              |          BY736899             |
|                       |                              |          BY258728             |
|                       |                              |          BY769240             |
|                       |                              |          BY089428             |
|                       |                              |          BY078648             |
|                       |                              |          AI788330             |
|                       |                              |          BY202211             |
|                       |                              |          BY188266             |
|                       |                              |          BY181198             |
|                       |                              |          BY767738             |
|                       |                              |          BB620496             |
|                       |                              |          AK077863             |
|                       |                              |          BI104433             |
|                       |                              |          BC051055             |
|                       |                              |          BU695022             |
|                       |                              |          AA103405             |
|                       |                              |          BI903996             |
|                       |                              |          BI105998             |
|                       |                              |          BI415067             |
|                       |                              |          BG967055             |
|                       |                              |          BC024135             |
|                       |                              |          AA413978             |
|                       |                              |          BU899148             |
|                       |                              |          BF141921             |
|                       |                              |          CB181119             |
|                       |                              |          CB274035             |
|                       |                              |          CA983747             |
|                       |                              |          AA000638             |
|                       |                              |          AA511385             |
|                       |                              |          BM947573             |
|                       |                              |          BI102040             |
|                       |                              |          BC057914             |
|                       |                              |          BI526421             |
|                       |                              |          BE624587             |
|                       |                              |          AA183980             |
|                       |                              |          BY360585             |
|                       |                              |          AA222106             |
|                       |                              |          CB321583             |
|                       |                              |          AA500094             |
|                       |                              |          CN722111             |
|                       |                              |          AI425218             |
|                       |                              |          AA760320             |
|                       |                              |          CB196723             |
|                       |                              |          BB037468             |
|                       |                              |          BQ031181             |
|                       |                              |          BY757260             |
|                       |                              |          AA755521             |
|                       |                              |          AA476002             |
|                       |                              |          BE627868             |
|                       |                              |          CB946868             |
|                       |                              |          BE986518             |
|                       |                              |          BE985906             |
|                       |                              |          BM224048             |
|                       |                              |          CA547305             |
|                       |                              |          CN706528             |
|                       |                              |          BQ553300             |
|                       |                              |          BE986493             |
|                       |                              |          BM900098             |
|                       |                              |          BQ553299             |
|                       |                              |          BE199191             |
|                       |                              |          AA434970             |
|                       |                              |          AW743032             |
|                       |                              |          BE982013             |
|                       |                              |          BB776909             |
|                       |                              |          AI666796             |
|                       |                              |          BE981949             |
|                       |                              |          AI848179             |
|                       |                              |            W75832             |
|                       |                              |          AI893280             |
|                       |                              |          BY446439             |
|                       |                              |          BY546453             |
|                       |                              |          BY460732             |
|                       |                              |          BE985591             |
|                       |                              |          BY534173             |
|                       |                              |          BY697426             |
|                       |                              |          BY094316             |
|                       |                              |          BY436933             |
|                       |                              |          BY525277             |
|                       |                              |          AW124800             |
|                       |                              |          BY457655             |
|                       |                              |          AI644551             |
|                       |                              |          BY576639             |
|                       |                              |          BY578243             |
|                       |                              |          BY636602             |
|                       |                              |          BB793916             |
|                       |                              |          BY575688             |
|                       |                              |          AI604359             |
|                       |                              |          BY423242             |
|                       |                              |          BF463725             |
|                       |                              |          BY467254             |
|                       |                              |          BX640303             |
|                       |                              |          AV165786             |
|                       |                              |          AV137265             |
|                       |                              |          BB447229             |
|                       | *Seq. mapped in strand Minus*| *Seq. mapped in strand Minus* |
|                       |           BC053909           |          BI731596             |
|                       |           AK056259           |          BE951312             |
|                       |                              |          BC062132             |
|                       |                              |          AK036740             |
|                       |                              |          AK034254             |
|                       |                              |          BY468151             |
|                       |                              |          BG069487             |
|                       |                              |          AK046195             |
|                       |                              |          BB314805             |
|                       |                              |          BU054789             |
|                       |                              |          BB527169             |
|                       |                              |          BB038409             |
|                       |                              |          AK031252             |
|                       |                              |          AI616102             |
|                       |                              |          CB525545             |
|                       |                              |          BQ963722             |
|                       |                              |          CA320696             |
|                       |                              |          BB620580             |
|                       |                              |          AU079993             |
|                       |                              |          CN531881             |
|                       |                              |          BM937201             |
|                       |                              |          BB659480             |
|                       |                              |          BB663296             |
|                       |                              |          BB626250             |
|                       |                              |          BI986342             |
|                       |                              |          BY718347             |
|                       |                              |          BB629724             |
|                       |                              |          BB611023             |
|                       |                              |          BB644765             |
|                       |                              |          BY241257             |
|                       |                              |          BY238659             |
|                       |                              |          BY255965             |
|                       |                              |          BY307573             |
|                       |                              |          BG082427             |
|                       |                              |          BY784745             |
|                       |                              |          BY781086             |
|                       |                              |          AW701160             |
|                       |                              |                               |
|                       |                              |                               |
|                       |                              |                               |
|                       |                              |                               |
|                       |                              |                               |
|                       |                              |                               |
|                       |                              |                               |
|                       |                              |                               |
|                       |                              |                               |
|                       |                              |                               |
|                       |                              |                               |
|                       |                              |                               |
|                       |                              |                               |
|                       |                              |                               |
|                       |                              |                               |
|                       |                              |                               |
|                       |                              |                               |
|                       |                              |                               |
|                       |                              |                               |
|                       |                              |                               |
|                       |                              |                               |
|                       |                              |                               |
|                       |                              |                               |
|                       |                              |                               |
|                       |                              |                               |
|                       |                              |                               |
|                       |                              |                               |
|                       |                              |                               |
|                       |                              |                               |
|                       |                              |                               |
|                       |                              |                               |
|                       |                              |                               |
|                       |                              |                               |
|                       |                              |                               |
|                       |                              |                               |
|                       |                              |                               |
|                       |                              |                               |
|                       |                              |                               |
|                       |                              |                               |
|                       |                              |                               |
|                       |                              |                               |
|                       |                              |                               |
|                       |                              |                               |
|                       |                              |                               |
|                       |                              |                               |
|                       |                              |                               |
|                       |                              |                               |
|                       |                              |                               |
|                       |                              |                               |
|                       |                              |                               |
|                       |                              |                               |
|                       |                              |                               |
|                       |                              |                               |
|                       |                              |                               |
|                       |                              |                               |
|                       |                              |                               |
|                       |                              |                               |
|                       |                              |                               |
|                       |                              |                               |
|                       |                              |                               |
|                       |                              |                               |
|                       |                              |                               |
|                       |                              |                               |
|                       |                              |                               |
|                       |                              |                               |
|                       |                              |                               |
|                       |                              |                               |
|                       |                              |                               |
|                       |                              |                               |
|                       |                              |                               |
|                       |                              |                               |
|                       |                              |                               |
|                       |                              |                               |
|                       |                              |                               |
|                       |                              |                               |
+-----------------------+------------------------------+-------------------------------+
|      #   59           | *Seq. mapped in strand Plus* | *Seq. mapped in strand Plus*  |
|                       |           BU678853           |          AK012174             |
|                       |           AA215856           |          BY096488             |
|                       |           BM744650           |          BU512489             |
|                       |           BU951079           |          BC037099             |
|                       |           BX090192           |          AK014354             |
|                       |           AI808588           |          CA980429             |
|                       |           BX118007           |          BU147793             |
|                       |           AA889171           |          BU516096             |
|                       |                              |          BQ890927             |
|                       |                              |          BQ179683             |
|                       |                              |          BC043044             |
|                       |                              |          CF553209             |
|                       |                              |          AA120605             |
|                       |                              |          CD353760             |
|                       |                              |          BU152092             |
|                       |                              |          BG965865             |
|                       |                              |          BI683873             |
|                       |                              |          BC057997             |
|                       |                              |          AA619302             |
|                       |                              |          BE285447             |
|                       |                              |          BQ926062             |
|                       |                              |          CN695014             |
|                       |                              |          BE572537             |
|                       |                              |          CD353987             |
|                       |                              |          CO046452             |
|                       |                              |          AW682293             |
|                       |                              |          BM946347             |
|                       |                              |          BB565230             |
|                       |                              |          AK008996             |
|                       |                              |          AI050603             |
|                       |                              |            W82115             |
|                       |                              |          CA537283             |
|                       |                              |          CF894445             |
|                       |                              |          BC025134             |
|                       |                              |          AW412310             |
|                       |                              |          BF782311             |
|                       |                              |          BB570528             |
|                       |                              |          BE532336             |
|                       |                              |          CK391249             |
|                       |                              |          BM945284             |
|                       |                              |          BI067993             |
|                       |                              |          CA539215             |
|                       |                              |          AA881948             |
|                       |                              |          BB868186             |
|                       |                              |          BI715743             |
|                       |                              |          BI685262             |
|                       |                              |          BQ564145             |
|                       |                              |          BF226009             |
|                       |                              |          BE226732             |
|                       |                              |          BI730210             |
|                       |                              |          AW208944             |
|                       |                              |          AW681595             |
|                       |                              |          AW681764             |
|                       |                              |          BI694230             |
|                       |                              |          BI104644             |
|                       |                              |          AA104341             |
|                       |                              |          BM203223             |
|                       |                              |          BE849731             |
|                       |                              |          AI527361             |
|                       |                              |          BG797271             |
|                       |                              |          CF950012             |
|                       |                              |          CD772694             |
|                       |                              |          BQ257906             |
|                       |                              |          AA546778             |
|                       |                              |          BY448916             |
|                       |                              |          CN678346             |
|                       |                              |          BY390554             |
|                       |                              |          CB317983             |
|                       |                              |          BB822483             |
|                       |                              |          AI195963             |
|                       |                              |          BB790631             |
|                       |                              |          BM204921             |
|                       |                              |          BB686715             |
|                       |                              |          BY459321             |
|                       |                              |          BY429876             |
|                       |                              |          BY604467             |
|                       |                              |          BY641683             |
|                       |                              |          AW412749             |
|                       |                              |          AI835383             |
|                       |                              |          BY637328             |
|                       |                              |          BM124596             |
|                       |                              |          BM124852             |
|                       |                              |          BE573801             |
|                       |                              |          BY395333             |
|                       |                              |          BY399474             |
|                       |                              |          CB599882             |
|                       |                              |          BM241130             |
|                       |                              |          BM230941             |
|                       |                              |          CA887589             |
|                       |                              |          BM124560             |
|                       |                              |          BU851680             |
|                       |                              |          CA888788             |
|                       |                              |          AI839828             |
|                       |                              |          AV216542             |
|                       |                              |          BY407389             |
|                       |                              |          BI715090             |
|                       |                              |          AV101519             |
|                       |                              |          BX639519             |
|                       |                              |          CF751514             |
|                       |                              |          AV118723             |
|                       |                              |          AI836423             |
|                       |                              |          BQ748838             |
|                       |                              |          AV281658             |
|                       |                              |          BM124276             |
|                       |                              |          BM114647             |
|                       |                              |          AA255303             |
|                       |                              |          BM569888             |
|                       |                              |          AA211997             |
|                       |                              |          BM221226             |
|                       |                              |          AA221351             |
|                       |                              |          BE983502             |
|                       |                              |          BE995155             |
|                       |                              |          AI839270             |
|                       |                              |          CO040375             |
|                       |                              |          CO040610             |
|                       |                              |          CO040230             |
|                       |                              |          CO040669             |
|                       |                              |          AI451058             |
|                       |                              |          AI098058             |
|                       |                              |          AI593218             |
|                       |                              |          BE950918             |
|                       |                              |          BE988083             |
|                       |                              |          BY019577             |
|                       |                              |          BB616858             |
|                       |                              |          AK030122             |
|                       |                              |          BY726267             |
|                       |                              |          BY734335             |
|                       |                              |          AK046970             |
|                       |                              |          BY030159             |
|                       |                              |          BE655047             |
|                       |                              |          AK042741             |
|                       |                              |          BB639933             |
|                       |                              |          BY719708             |
|                       |                              |            U34973             |
|                       |                              |          AI894062             |
|                       |                              |          BC038608             |
|                       |                              |          BB621095             |
|                       |                              |          BY230159             |
|                       |                              |          BY783456             |
|                       |                              |          BQ569216             |
|                       |                              |          AW825532             |
|                       |                              |          BB618318             |
|                       |                              |          BY783961             |
|                       |                              |          BF451631             |
|                       |                              |          BG519039             |
|                       |                              |          BE336472             |
|                       |                              |          BF124629             |
|                       |                              |            L26737             |
|                       |                              |          AI323304             |
|                       |                              |          BG298204             |
|                       |                              |          CN688960             |
|                       |                              |          BI731543             |
|                       |                              |          AW825856             |
|                       |                              |          BI665746             |
|                       |                              |          AI854414             |
|                       |                              |          BY389116             |
|                       |                              |          BY683085             |
|                       |                              |          BU147830             |
|                       |                              |          BQ032151             |
|                       |                              |          AW681460             |
|                       |                              |          BY731886             |
|                       | *Seq. mapped in strand Minus*| *Seq. mapped in strand Minus* |
|                       |           CA415281           |          BI465766             |
|                       |           BU784411           |          BM502089             |
|                       |           AA804857           |          BM502359             |
|                       |           BX646430           |          AI852572             |
|                       |           AI130938           |          BF467041             |
|                       |           BQ019600           |          BC033436             |
|                       |           BU619353           |          BE945612             |
|                       |           AK126426           |          BQ478581             |
|                       |           AK091532           |          BE198078             |
|                       |           BC011125           |          BE626023             |
|                       |           AW827188           |          CA951555             |
|                       |           AW275504           |          BE994235             |
|                       |           AW069523           |          BC034168             |
|                       |           AA135725           |          AA960555             |
|                       |           AI246733           |          AI605034             |
|                       |           AI435408           |          BU788309             |
|                       |           BF589217           |          BU152359             |
|                       |           AI754802           |          BF234521             |
|                       |           CK903853           |          BY755967             |
|                       |           AI127809           |          CK021730             |
|                       |           AA088218           |          BF142070             |
|                       |           AL599010           |          CF584736             |
|                       |           BM666160           |          AI553057             |
|                       |           AW069712           |          BI715817             |
|                       |           BQ574357           |          AA422192             |
|                       |           CA418305           |          AI415190             |
|                       |           BM992834           |          BE630436             |
|                       |           BM999636           |          BG094763             |
|                       |           BM979313           |          BQ033232             |
|                       |           BQ006070           |          AU021641             |
|                       |           BQ014071           |          BB784379             |
|                       |             Z24725           |          BB779353             |
|                       |           BU951900           |          AW540591             |
|                       |           BU625477           |          BQ265402             |
|                       |           AI244250           |          BG070082             |
|                       |           AF443279           |          BF124045             |
|                       |           CR624174           |          CB947124             |
|                       |           CD250668           |          AI120740             |
|                       |           BX388752           |          AI121434             |
|                       |           BG622633           |          CN716799             |
|                       |           BX389747           |          BE624639             |
|                       |           CD672754           |          CD807767             |
|                       |           CK003260           |          CB599589             |
|                       |           CB265794           |          BI112358             |
|                       |           BF964986           |          BG867422             |
|                       |           BX389748           |          BQ900633             |
|                       |           BX346981           |          BI852793             |
|                       |           BX326417           |          BI102388             |
|                       |             H04831           |          CA587872             |
|                       |           CB266244           |          BE625068             |
|                       |           BF984771           |          BG082935             |
|                       |           BQ890012           |          CD773009             |
|                       |           AI948572           |          BG077127             |
|                       |           AI217208           |          AI226192             |
|                       |           BQ011082           |          AI391018             |
|                       |           BM987268           |          AW047139             |
|                       |           BG210972           |          BI653313             |
|                       |           AI804028           |          AW911276             |
|                       |           BE327003           |          BF124237             |
|                       |             R52691           |          BF160022             |
|                       |             H29253           |          BU614046             |
|                       |           BX119558           |          CD354118             |
|                       |           AI927205           |            W29755             |
|                       |           BC017327           |          BG970455             |
|                       |           AA906083           |          BQ257854             |
|                       |             W57659           |          AW742174             |
|                       |           AA370935           |          BQ257799             |
|                       |           CF597095           |          CK020032             |
|                       |           BG435558           |          BE284543             |
|                       |             R14517           |          BE912159             |
|                       |           BG398600           |          BI454953             |
|                       |           BE539720           |          BI111386             |
|                       |           CN429908           |          CD550909             |
|                       |           CK002425           |          BU612930             |
|                       |           BU689981           |          CK334667             |
|                       |           BX161467           |          BI663946             |
|                       |           BX330544           |          BF642821             |
|                       |           BF247076           |          BQ566048             |
|                       |           BI464761           |          BF118705             |
|                       |           BF832559           |          BU518387             |
|                       |           BQ355801           |          BF141508             |
|                       |           AA190738           |          BF143541             |
|                       |           BP340270           |          BI692347             |
|                       |           BI056094           |          BU925369             |
|                       |           BQ694999           |          CB248133             |
|                       |           AA319153           |          BQ265403             |
|                       |           BF000983           |          BI332020             |
|                       |           BG426272           |          CO045311             |
|                       |           AA372664           |          CF899522             |
|                       |           BX360023           |          CN677074             |
|                       |           BX325716           |          BU922259             |
|                       |           AA368442           |          BF383912             |
|                       |           BX499316           |          AA799025             |
|                       |           AL598960           |          CN703010             |
|                       |           BF087144           |          CD561873             |
|                       |             D53452           |          BU920748             |
|                       |           BX450622           |          AI663650             |
|                       |           AL600973           |          CK023125             |
|                       |           BG577226           |          BE916773             |
|                       |             N88349           |          AA067156             |
|                       |           BX389767           |          AA667212             |
|                       |             Z26992           |          BG519019             |
|                       |             T80292           |          BQ179963             |
|                       |             H29252           |          BG922053             |
|                       |             T11330           |          BY720345             |
|                       |           CN429911           |          BY735580             |
|                       |           BI088121           |          CN705240             |
|                       |           CN429909           |          CN703287             |
|                       |           BX388609           |          CF728348             |
|                       |           BU788079           |          BB643156             |
|                       |           CN429910           |          CN668782             |
|                       |           BX450231           |          CN704120             |
|                       |             H71436           |          BY734819             |
|                       |           CN429915           |          BB625431             |
|                       |             N91727           |          BE533708             |
|                       |           BX389766           |          BB633275             |
|                       |           BX371837           |          BY644253             |
|                       |           AW992056           |          BQ553705             |
|                       |           BX458611           |          AK079588             |
|                       |           BX407626           |          AI414847             |
|                       |           BX398816           |          BY588645             |
|                       |           BX358063           |          BQ032871             |
|                       |           BX346718           |          BQ569195             |
|                       |           BF951621           |          BB186133             |
|                       |           BI562552           |          BB820627             |
|                       |           CN429918           |          AK087720             |
|                       |           BX464883           |          CB589133             |
|                       |           BF752698           |          CD542344             |
|                       |           CN429916           |          CA564254             |
|                       |           CN429919           |          BG277676             |
|                       |           CV029143           |          AA637131             |
|                       |           CN429921           |          BY302840             |
|                       |           BU188003           |          BY329895             |
|                       |           BI771814           |          AI197301             |
|                       |           BG704215           |          BY229532             |
|                       |           BG006458           |          CN699653             |
|                       |           BG723282           |          BY241982             |
|                       |           AL044112           |          CA563557             |
|                       |           CN429913           |          BY223460             |
|                       |           BI553714           |          BY333252             |
|                       |             H80095           |          AA087584             |
|                       |           AI017075           |          CF166849             |
|                       |           AI355335           |          BY220166             |
|                       |           AI991489           |          BY325082             |
|                       |           AI808833           |          BY301704             |
|                       |           AA634107           |          BY342535             |
|                       |           AW024526           |          BY328853             |
|                       |           BE501442           |          BB840765             |
|                       |           AI970938           |          BY332102             |
|                       |           AI768705           |          BY788120             |
|                       |           AW271443           |          BX518085             |
|                       |           AI627283           |          BX638637             |
|                       |           BE501451           |          AK036369             |
|                       |           AI928241           |          AI852269             |
|                       |           AI804082           |          BC018552             |
|                       |           CD388939           |          AK048690             |
|                       |           CN429917           |          BC043475             |
|                       |           BP872459           |          BQ032742             |
|                       |           AA256424           |          AI449269             |
|                       |           AI224965           |          BM936569             |
|                       |           BQ437790           |          BB018971             |
+-----------------------+------------------------------+-------------------------------+
|      #   60           | *Seq. mapped in strand Plus* | *Seq. mapped in strand Plus*  |
|                       |           BG684824           |          BY260985             |
|                       |           CD109914           |          BE554244             |
|                       |           BG685926           |          BQ957877             |
|                       |           BI911321           |          BQ952689             |
|                       |           BI224297           |          BB639068             |
|                       |           BG116424           |          BU706745             |
|                       |           AF419857           |          BY014209             |
|                       |           CN359125           |          BY710835             |
|                       |           AL530463           |          BY195398             |
|                       |           CR596995           |          BY069477             |
|                       |           AF542552           |          BY092994             |
|                       |           BE786633           |          BY190871             |
|                       |           BI670080           |          AA214908             |
|                       |           BX399452           |          BY229777             |
|                       |           CR620298           |          BY005193             |
|                       |           BU428772           |          BY027436             |
|                       |           BE778421           |          BB840227             |
|                       |           BF686158           |          BY754185             |
|                       |           BF237759           |          BY231985             |
|                       |           BQ431230           |          BY014658             |
|                       |           BE877123           |          BY040314             |
|                       |           BU180855           |          CA891763             |
|                       |           BU155268           |          CA891723             |
|                       |           BC054049           |          CA890220             |
|                       |           BC064903           |          BE306526             |
|                       |           BU784023           |          BI103893             |
|                       |           CK902610           |          BY009671             |
|                       |           BQ421607           |          BY231853             |
|                       |           CK902609           |          BE860819             |
|                       |           BU783753           |          BQ257419             |
|                       |           CN359124           |          BY216783             |
|                       |           BG025088           |          BB856750             |
|                       |           AA373370           |          BY791503             |
|                       |           AW966499           |          BY783835             |
|                       |           BX381719           |          BY768796             |
|                       |           CR597781           |          BY133816             |
|                       |           CD694157           |          BB583061             |
|                       |           CF128540           |          BY013465             |
|                       |           CN359120           |          BY163108             |
|                       |           AA247930           |          BY171542             |
|                       |           AV736218           |          AA254862             |
|                       |           CB137162           |          AA268678             |
|                       |           CB137847           |          AA510398             |
|                       |           AV752633           |          AA919532             |
|                       |           BU197185           |          BY297596             |
|                       |           BG033847           |          AI152918             |
|                       |           CF596161           |                               |
|                       |           BG200338           |                               |
|                       |           BF670872           |                               |
|                       |           BF672098           |                               |
|                       |           AJ711811           |                               |
|                       |           AL079314           |                               |
|                       |           AL530462           |                               |
|                       |           BF081558           |                               |
|                       |           CN359122           |                               |
|                       |           CN359121           |                               |
|                       |           BG335828           |                               |
|                       |           AA035129           |                               |
|                       |           BX399451           |                               |
|                       |           AA380083           |                               |
|                       |           CV319604           |                               |
|                       |             W46196           |                               |
|                       |           BG501549           |                               |
|                       |           BE763121           |                               |
|                       |           BE763159           |                               |
|                       |           BX381718           |                               |
|                       |           AW889191           |                               |
|                       |           AW889253           |                               |
|                       |           BF350434           |                               |
|                       |           CA412612           |                               |
|                       |           AA035130           |                               |
|                       |           AV729592           |                               |
|                       |           AA306564           |                               |
|                       |           AW955261           |                               |
|                       |           CN359123           |                               |
|                       |           CA418380           |                               |
|                       |           BQ225386           |                               |
|                       |           AI478152           |                               |
|                       |             R08959           |                               |
|                       |           AI565984           |                               |
|                       |           CA440626           |                               |
|                       |           CK300403           |                               |
|                       |           AL079312           |                               |
|                       |           BX113844           |                               |
|                       |           AI754535           |                               |
|                       |           AA602110           |                               |
|                       |           BQ023059           |                               |
|                       |           BM677189           |                               |
|                       | *Seq. mapped in strand Minus*| *Seq. mapped in strand Minus* |
|                       |           AA761751           |          CN461762             |
|                       |           AI742176           |          AK016716             |
|                       |           AL577292           |          BB759791             |
|                       |           CR605615           |          AA119976             |
|                       |           AL580503           |          BG795168             |
|                       |           AI336964           |          AA051102             |
|                       |           BC002586           |          AW911501             |
|                       |           AI554123           |          CK334259             |
|                       |           BX433648           |          CF950822             |
|                       |           AL038597           |          AA124999             |
|                       |           AI742660           |          BX516853             |
|                       |           BG199646           |          AI509403             |
|                       |           BE855989           |          BI655056             |
|                       |           BX334410           |          AA104448             |
|                       |           AI357793           |          CF910988             |
|                       |           AI146380           |          CF913383             |
|                       |           AW242741           |          CF911141             |
|                       |           BM978381           |          CF912837             |
|                       |           AW248882           |          BY154936             |
|                       |           BC004424           |          BY170052             |
|                       |           BU677391           |          CN719208             |
|                       |           AW572455           |          BU054958             |
|                       |           BG938528           |          CA573619             |
|                       |           AI077472           |          CF894339             |
|                       |           AI861837           |          BI453276             |
|                       |           BX329828           |          CN721239             |
|                       |           BM980652           |          BE915480             |
|                       |           AI949984           |          BU514996             |
|                       |             U93867           |          BQ949052             |
|                       |           AI934957           |          AW106272             |
|                       |           AY091463           |          CA750191             |
|                       |           AA826817           |          CK030837             |
|                       |           AL525784           |          CA750783             |
|                       |           CR592330           |          CA749914             |
|                       |             T95486           |          AI642041             |
|                       |           BX324555           |          BC026793             |
|                       |           CR608817           |          AI841776             |
|                       |           CR613712           |          BE993627             |
|                       |           AL527348           |          AI840272             |
|                       |           BF690368           |          AV048153             |
|                       |           BF432439           |          BE859792             |
|                       |           BF792401           |          CB194190             |
|                       |           BP371462           |          BX527321             |
|                       |           AW392495           |          BG967176             |
|                       |           AW392489           |          BI653529             |
|                       |           BG216971           |          CF904489             |
|                       |           AI909122           |          CF905281             |
|                       |           BM794403           |          BU709522             |
|                       |           AW392484           |          CD771252             |
|                       |           AL544738           |          CF916001             |
|                       |           AW392490           |          CD540322             |
|                       |             T84671           |          CD542781             |
|                       |           AW392491           |          BU924027             |
|                       |           AW392493           |          CD541337             |
|                       |           BG249142           |          BG806687             |
|                       |             R11431           |          CF913780             |
|                       |           AW376192           |          BB640540             |
|                       |           AW376191           |          BB640666             |
|                       |           AW376195           |          BY727424             |
|                       |             F00152           |          CD538465             |
|                       |           BX369570           |          CA567699             |
|                       |           CD516931           |          BG974756             |
|                       |           BX093964           |          CA538586             |
|                       |           BF376590           |          CA542864             |
|                       |             Z24802           |          CD540215             |
|                       |           AW392366           |          AV165644             |
|                       |           CD677882           |          CF894373             |
|                       |           BI020096           |          BY080930             |
|                       |           AW386870           |          BY226470             |
|                       |           AW960559           |          BB561921             |
|                       |           AW960562           |          BY224512             |
|                       |             T95802           |          BY062125             |
|                       |           CD622730           |          CA545139             |
|                       |           CD622731           |          CA547749             |
|                       |           BI027305           |          BY078886             |
|                       |             R11894           |          BY084033             |
|                       |             T95485           |          BY139037             |
|                       |           CB143033           |          BY782333             |
|                       |           BQ368452           |          AW681352             |
|                       |           BE080794           |          AW258769             |
|                       |           BQ307762           |          BY100680             |
|                       |           BQ306840           |                               |
|                       |           BE260943           |                               |
|                       |           CN334785           |                               |
|                       |           BE264476           |                               |
|                       |           CD520671           |                               |
|                       |           BG401227           |                               |
|                       |           BM545912           |                               |
|                       |           BQ073021           |                               |
|                       |           BM916377           |                               |
|                       |             T95937           |                               |
|                       |           BE780252           |                               |
|                       |           BX350392           |                               |
|                       |           BI025851           |                               |
|                       |           BI025852           |                               |
|                       |           BX331758           |                               |
|                       |           BX395308           |                               |
|                       |           AL554519           |                               |
|                       |           BI757925           |                               |
|                       |           BE877448           |                               |
|                       |           AL527741           |                               |
|                       |           BM556814           |                               |
|                       |           AL525823           |                               |
|                       |           BX361851           |                               |
|                       |           BM921784           |                               |
|                       |           BI755100           |                               |
|                       |           CD244339           |                               |
|                       |           BX446566           |                               |
|                       |           AL558755           |                               |
|                       |           BU154045           |                               |
|                       |           BE814036           |                               |
|                       |           BI909419           |                               |
|                       |           AL527389           |                               |
|                       |           BI769891           |                               |
|                       |           BP326835           |                               |
|                       |           BM806769           |                               |
|                       |           BI918836           |                               |
|                       |           BQ688306           |                               |
|                       |           BM919758           |                               |
|                       |           CD101763           |                               |
|                       |           BI909081           |                               |
|                       |           BE867780           |                               |
|                       |           CV026008           |                               |
|                       |           BE315117           |                               |
|                       |           BE740402           |                               |
|                       |           CN334784           |                               |
|                       |           BF794687           |                               |
|                       |           CN334787           |                               |
|                       |           BM919695           |                               |
|                       |           BQ308380           |                               |
|                       |           BQ309259           |                               |
|                       |           AA280982           |                               |
|                       |           CV029227           |                               |
|                       |           BG403605           |                               |
|                       |           BQ307760           |                               |
|                       |           BQ308379           |                               |
|                       |           BQ307145           |                               |
|                       |           BM010506           |                               |
|                       |           BI860118           |                               |
|                       |           BI831011           |                               |
|                       |           BE389326           |                               |
|                       |           BE267338           |                               |
|                       |           BG328119           |                               |
|                       |           CN334786           |                               |
|                       |           BI907327           |                               |
|                       |           BI092766           |                               |
|                       |           BP206356           |                               |
|                       |           AW500271           |                               |
|                       |           BQ225244           |                               |
|                       |           BQ308378           |                               |
|                       |           AA413398           |                               |
|                       |           BP349494           |                               |
|                       |           BG116240           |                               |
|                       |           BP200212           |                               |
|                       |           BE257375           |                               |
|                       |           AA134612           |                               |
|                       |           CB993946           |                               |
|                       |           CN334783           |                               |
|                       |             H19022           |                               |
|                       |             H82192           |                               |
|                       |           AW249301           |                               |
|                       |           AA146700           |                               |
|                       |             W16650           |                               |
|                       |           BG900581           |                               |
|                       |           AA385341           |                               |
|                       |           BM151844           |                               |
|                       |           AL038596           |                               |
|                       |           AA146668           |                               |
+-----------------------+------------------------------+-------------------------------+
|      #   61           | *Seq. mapped in strand Plus* | *Seq. mapped in strand Plus*  |
|                       |           AI831837           |          BF148719             |
|                       | *Seq. mapped in strand Minus*| *Seq. mapped in strand Minus* |
|                       |           AI391505           |          BF658916             |
|                       |           AW449397           |          AW411575             |
|                       |           BG057197           |          BE996262             |
|                       |           CA443458           |          CF354869             |
|                       |             U94831           |          CF382344             |
|                       |             R44778           |          CF423821             |
|                       |             N93753           |          CF805118             |
|                       |           BQ001853           |          CF925892             |
|                       |           AW296244           |          CK385095             |
|                       |           CA424205           |          AI893436             |
|                       |           AI690830           |          AK004754             |
|                       |           AI167659           |          CF423902             |
|                       |           BE503565           |          BG920687             |
|                       |           AW589479           |          CF354532             |
|                       |           BG057709           |          CF805059             |
|                       |           BQ001680           |          CF927031             |
|                       |           BP330035           |          BE691068             |
|                       |           AA861929           |          AW488028             |
|                       |           BU683191           |          AI851160             |
|                       |           AI982533           |          BC007187             |
|                       |           BU631911           |          AF269152             |
|                       |           BU630261           |          BC017617             |
|                       |           CA449038           |          BG917179             |
|                       |           CA446452           |          BY755221             |
|                       |           CB241575           |          AK090065             |
|                       |           AI609706           |          BE571685             |
|                       |           BC010856           |          CB193028             |
|                       |           BM682525           |          BU846871             |
|                       |           CR604869           |          CK793465             |
|                       |           BX448280           |          BE532519             |
|                       |           CN407358           |          AI322383             |
|                       |           BX381585           |          BX518851             |
|                       |           CR606704           |          AA940158             |
|                       |           CR618221           |            W83187             |
|                       |           CR614922           |          BE862236             |
|                       |           BX161390           |          BE289524             |
|                       |           BF529529           |          BG976961             |
|                       |           BF984481           |          CN704207             |
|                       |           BX347118           |          CN704616             |
|                       |           BX377557           |          CB571089             |
|                       |           AA127785           |          CB203660             |
|                       |           BF946032           |          CB947583             |
|                       |           BM727764           |          BU148726             |
|                       |           BG777562           |          BE288335             |
|                       |           BE881525           |          BI661984             |
|                       |             R70430           |          CB587568             |
|                       |           BX450618           |          BF532894             |
|                       |           BX367828           |          BU058972             |
|                       |             N26442           |          CF741632             |
|                       |           BF679313           |          BF015623             |
|                       |           CB988286           |          BE914042             |
|                       |           CB990147           |          BI653871             |
|                       |             W44908           |          CB231738             |
|                       |           BX347058           |          BF101257             |
|                       |           BM887877           |          BE285738             |
|                       |           BE003856           |          BE285478             |
|                       |           BP431963           |          CB237491             |
|                       |           BE786730           |          BF607178             |
|                       |           CA489196           |          BQ936109             |
|                       |           BM556175           |          BI078592             |
|                       |           BX326236           |          BB610966             |
|                       |           BX450245           |          BI555458             |
|                       |           BE717116           |          BF714768             |
|                       |           BM717185           |          CA533707             |
|                       |           BM726091           |          CA894425             |
|                       |           BX429470           |          BY745059             |
|                       |           BX368291           |          BY745108             |
|                       |           AA303616           |          BF658615             |
|                       |           CF596293           |          CA893371             |
|                       |           CB216297           |          BG807685             |
|                       |           BX373120           |          BI328070             |
|                       |           BX352487           |          CD563005             |
|                       |           CR621520           |          BU922823             |
|                       |           BX346656           |          BY316465             |
|                       |           BX161494           |          BY249827             |
|                       |           BX362269           |          AA268737             |
|                       |           AW001226           |          BY704952             |
|                       |           BX347271           |          AI607769             |
|                       |           BX347395           |          BY166567             |
|                       |           BX388657           |          BY171413             |
|                       |           BX450617           |          BY159647             |
|                       |           BX326211           |          BY165532             |
|                       |           BX368249           |          BY074153             |
|                       |           CB989490           |          BY160722             |
|                       |           BX347006           |                               |
|                       |           CD243164           |                               |
|                       |           BX347049           |                               |
|                       |           BX390767           |                               |
|                       |           BX347250           |                               |
|                       |           BX368102           |                               |
|                       |           BX329303           |                               |
|                       |           BX408040           |                               |
|                       |           BG925497           |                               |
|                       |           BF094556           |                               |
|                       |           BF094552           |                               |
|                       |           BP261939           |                               |
|                       |           BP262267           |                               |
|                       |           BM701393           |                               |
|                       |           BX347165           |                               |
|                       |           BX417101           |                               |
|                       |             W52643           |                               |
|                       |           BX368124           |                               |
|                       |           BE872435           |                               |
|                       |           BF945858           |                               |
|                       |           CB147545           |                               |
|                       |           AI601220           |                               |
|                       |           BX331391           |                               |
|                       |           CR614007           |                               |
|                       |           BU527086           |                               |
|                       |           AI086839           |                               |
|                       |           BM353671           |                               |
|                       |           BG398091           |                               |
|                       |           BX331392           |                               |
|                       |           BF316720           |                               |
|                       |           BQ897875           |                               |
|                       |           BX335531           |                               |
|                       |           AU118420           |                               |
|                       |           BQ221987           |                               |
|                       |           BG179330           |                               |
|                       |           BP300148           |                               |
|                       |           BX367924           |                               |
|                       |           BX429145           |                               |
|                       |           CF138781           |                               |
|                       |           BX367971           |                               |
|                       |           BX347480           |                               |
|                       |           BX347479           |                               |
|                       |           BI823379           |                               |
|                       |           AI752329           |                               |
|                       |           AI752330           |                               |
|                       |           BF742191           |                               |
|                       |           BU162304           |                               |
|                       |           BF765542           |                               |
|                       |           BQ436677           |                               |
|                       |           BG824193           |                               |
|                       |           CN484799           |                               |
|                       |           BX377558           |                               |
|                       |           AA436865           |                               |
|                       |           AI638805           |                               |
|                       |           BX339718           |                               |
|                       |           BQ682946           |                               |
|                       |           BG427372           |                               |
|                       |           BX446577           |                               |
|                       |           BX393958           |                               |
|                       |           BX346657           |                               |
|                       |           BX396160           |                               |
|                       |           BX381586           |                               |
|                       |           AW673452           |                               |
|                       |           BX448281           |                               |
|                       |           BX388485           |                               |
|                       |           BX332624           |                               |
|                       |           CO247342           |                               |
|                       |           BE899402           |                               |
|                       |           BP292570           |                               |
|                       |           BX362270           |                               |
|                       |           CA487757           |                               |
|                       |           BP292566           |                               |
|                       |           BI916120           |                               |
|                       |           BI826650           |                               |
|                       |           AI766919           |                               |
|                       |           AA857204           |                               |
|                       |           AI264423           |                               |
|                       |           AI288175           |                               |
|                       |           AI262410           |                               |
|                       |           AI346254           |                               |
|                       |           BE669959           |                               |
|                       |           AW205944           |                               |
|                       |           AI281278           |                               |
|                       |           AI167155           |                               |
|                       |           AI861789           |                               |
|                       |           CK299561           |                               |
|                       |           AI401405           |                               |
|                       |           BU624905           |                               |
|                       |           CD368817           |                               |
|                       |           AI765810           |                               |
|                       |           BX395173           |                               |
|                       |           AU140435           |                               |
|                       |           BE898318           |                               |
|                       |           BQ189428           |                               |
|                       |           BU195363           |                               |
|                       |           BI517770           |                               |
|                       |           BI561945           |                               |
|                       |           BE907977           |                               |
|                       |           BX398908           |                               |
|                       |           BM887909           |                               |
|                       |           CF122418           |                               |
|                       |           BI770689           |                               |
|                       |           BM353954           |                               |
|                       |           BI518795           |                               |
|                       |           BM048425           |                               |
|                       |           BU164600           |                               |
|                       |           CN407359           |                               |
|                       |           BM827837           |                               |
|                       |           AI084548           |                               |
|                       |           BI601991           |                               |
|                       |           BQ439592           |                               |
|                       |           CB988769           |                               |
|                       |           BP237944           |                               |
|                       |           CB960778           |                               |
|                       |           AI557365           |                               |
|                       |           CV023228           |                               |
|                       |           BQ437298           |                               |
|                       |           CN407363           |                               |
|                       |           BE091331           |                               |
|                       |           CN407364           |                               |
|                       |           CF141100           |                               |
|                       |           BI820652           |                               |
|                       |           BI765299           |                               |
|                       |           BP335584           |                               |
|                       |           CN407361           |                               |
|                       |           BM046168           |                               |
|                       |           BP213450           |                               |
|                       |           BM922952           |                               |
|                       |             T10746           |                               |
|                       |           BX103990           |                               |
|                       |           BI457830           |                               |
|                       |             H72494           |                               |
|                       |           BP197795           |                               |
|                       |           BP257067           |                               |
|                       |           BI769800           |                               |
|                       |           BP256682           |                               |
|                       |           BP243067           |                               |
|                       |           BM919828           |                               |
|                       |           CN407360           |                               |
|                       |           BP343056           |                               |
|                       |           BM926944           |                               |
|                       |           CB164645           |                               |
|                       |           CB997961           |                               |
|                       |           CB128737           |                               |
|                       |           CB960948           |                               |
|                       |           BG327948           |                               |
|                       |           BI916668           |                               |
|                       |           CD109375           |                               |
|                       |           CB164732           |                               |
|                       |           BP265173           |                               |
|                       |           BI829089           |                               |
|                       |           BG913854           |                               |
|                       |           BP253539           |                               |
|                       |           BM755434           |                               |
|                       |           AL037237           |                               |
|                       |           CF121149           |                               |
|                       |           AU098818           |                               |
|                       |           BP326003           |                               |
|                       |           CN407362           |                               |
|                       |           AA442622           |                               |
|                       |             T75428           |                               |
|                       |           AU076647           |                               |
+-----------------------+------------------------------+-------------------------------+
|      #   62           | *Seq. mapped in strand Plus* | *Seq. mapped in strand Plus*  |
|                       |           CK903690           |          BI133607             |
|                       |           BQ067513           |                               |
|                       |           BC047904           |                               |
|                       |           BQ067498           |                               |
|                       |           AK095921           |                               |
|                       |           BI963986           |                               |
|                       |           CR620273           |                               |
|                       |           AY037159           |                               |
|                       |           BI257920           |                               |
|                       |           BI834861           |                               |
|                       |           BX350491           |                               |
|                       |           BI822294           |                               |
|                       |           BQ920805           |                               |
|                       |           BI917202           |                               |
|                       |           CF552169           |                               |
|                       |           BX248286           |                               |
|                       |           CA391340           |                               |
|                       |           AL551568           |                               |
|                       |           AL557872           |                               |
|                       |           CR617087           |                               |
|                       |           BM560637           |                               |
|                       |           BM563307           |                               |
|                       |           BG724341           |                               |
|                       |           BG721071           |                               |
|                       |           BC032039           |                               |
|                       |           AK096720           |                               |
|                       |           CF551872           |                               |
|                       |           AK096124           |                               |
|                       |           BI259558           |                               |
|                       |           AA321069           |                               |
|                       |           BG476624           |                               |
|                       |           BG927023           |                               |
|                       |           BX370472           |                               |
|                       |           AL575096           |                               |
|                       |           CB852505           |                               |
|                       |           CF552805           |                               |
|                       |           BM758367           |                               |
|                       |           BX384293           |                               |
|                       |           BM687537           |                               |
|                       |           CV315459           |                               |
|                       |           BG122083           |                               |
|                       |           BQ946027           |                               |
|                       |           BI830577           |                               |
|                       |           BI222716           |                               |
|                       |           BP319052           |                               |
|                       |             R24601           |                               |
|                       |             R23657           |                               |
|                       |           BX103335           |                               |
|                       |           AA194467           |                               |
|                       |           BQ337768           |                               |
|                       |           BM700714           |                               |
|                       |           BF002870           |                               |
|                       |           BM975978           |                               |
|                       |           CA447355           |                               |
|                       |           AI948584           |                               |
|                       |             W19706           |                               |
|                       |           BF593252           |                               |
|                       |           AI678666           |                               |
|                       |           BM665319           |                               |
|                       |           AJ708504           |                               |
|                       |           AA507760           |                               |
|                       |           CV342580           |                               |
|                       |           CA428563           |                               |
|                       |           AK054648           |                               |
|                       |           BM807505           |                               |
|                       |           AJ707886           |                               |
|                       |           AA194383           |                               |
|                       |           BI963722           |                               |
|                       |             H83611           |                               |
|                       |           AI765219           |                               |
|                       |           BF434962           |                               |
|                       |           AA436128           |                               |
|                       |           AA702993           |                               |
|                       |           BE463806           |                               |
|                       |           AI990303           |                               |
|                       |           BE671938           |                               |
|                       |           AA620965           |                               |
|                       |           AI290470           |                               |
|                       |           AI624882           |                               |
|                       |           AI003925           |                               |
|                       |           AI338870           |                               |
|                       |           AI004689           |                               |
|                       |           AI004690           |                               |
|                       |           AI127228           |                               |
|                       |           BE856290           |                               |
|                       |           AI934786           |                               |
|                       |           AI770075           |                               |
|                       |           AA812597           |                               |
|                       |           AW203978           |                               |
|                       |           AA436020           |                               |
|                       |           BU727813           |                               |
|                       |             H82735           |                               |
|                       |           AI813349           |                               |
|                       |           AI142908           |                               |
|                       |             C04894           |                               |
|                       |           BE671242           |                               |
|                       |           AI208243           |                               |
|                       |           AW205754           |                               |
|                       |           BU728427           |                               |
|                       |           AI865963           |                               |
|                       |           BF115200           |                               |
|                       |           AJ345984           |                               |
|                       |           AJ573538           |                               |
|                       |           AJ573490           |                               |
|                       |           AJ346456           |                               |
|                       |           AJ346565           |                               |
|                       |             R24502           |                               |
|                       |           AI870165           |                               |
|                       |           BU195069           |                               |
|                       |           BM789636           |                               |
|                       |           CB961642           |                               |
|                       |           CK001877           |                               |
|                       |           BM091202           |                               |
|                       |           BP368863           |                               |
|                       |           BU595714           |                               |
|                       |           BQ943607           |                               |
|                       |           BG702083           |                               |
|                       |           BI544292           |                               |
|                       |           BI544528           |                               |
|                       |           BM783650           |                               |
|                       |           CD249332           |                               |
|                       |           BU077293           |                               |
|                       |           CA943112           |                               |
|                       |           AW250652           |                               |
|                       |           CB990826           |                               |
|                       |             W05516           |                               |
|                       |           BQ776968           |                               |
|                       |           BX097440           |                               |
|                       |           CB994609           |                               |
|                       |           AA434523           |                               |
|                       |           AF033111           |                               |
|                       |           BF310229           |                               |
|                       |           BC034562           |                               |
|                       |           BG291176           |                               |
|                       |           BQ214897           |                               |
|                       |           CN310604           |                               |
|                       |           BG754466           |                               |
|                       |           BG831031           |                               |
|                       |           AV695188           |                               |
|                       |           AV697392           |                               |
|                       |           AV691386           |                               |
|                       |           AV695195           |                               |
|                       |           AV688234           |                               |
|                       |           AV686484           |                               |
|                       |           AV688716           |                               |
|                       |           BE873130           |                               |
|                       |           CA776624           |                               |
|                       |           BQ269971           |                               |
|                       |           BG424282           |                               |
|                       |           BG423464           |                               |
|                       |           BI458493           |                               |
|                       |           BQ278040           |                               |
|                       |           BG107866           |                               |
|                       |           BQ670026           |                               |
|                       |           CN310600           |                               |
|                       |           AA167823           |                               |
|                       |           BE396087           |                               |
|                       |           BI858063           |                               |
|                       |           BM051769           |                               |
|                       |           BI227048           |                               |
|                       |           CD171773           |                               |
|                       |           BI117274           |                               |
|                       |           AA324710           |                               |
|                       |           BF312543           |                               |
|                       |           AW964842           |                               |
|                       |           BE397187           |                               |
|                       |           BE513524           |                               |
|                       |           BE560509           |                               |
|                       |           BE901431           |                               |
|                       |           CB132782           |                               |
|                       |           CB163199           |                               |
|                       |           BQ941035           |                               |
|                       |           BU601685           |                               |
|                       |           BU902538           |                               |
|                       |           BG683742           |                               |
|                       |           BG683095           |                               |
|                       |           BF309757           |                               |
|                       |           BI114523           |                               |
|                       |           BE901185           |                               |
|                       |           BX475685           |                               |
|                       |           BF128779           |                               |
|                       |           BF038663           |                               |
|                       |           BE792641           |                               |
|                       |           CD247417           |                               |
|                       |           BU957163           |                               |
|                       |           BX406335           |                               |
|                       |           CA432072           |                               |
|                       |           BX406336           |                               |
|                       |           CR593834           |                               |
|                       |           CK824336           |                               |
|                       |           BU190644           |                               |
|                       |           BE295506           |                               |
|                       |           BE259521           |                               |
|                       |           BE900624           |                               |
|                       |           CF552512           |                               |
|                       |           BE790053           |                               |
|                       |           BM919839           |                               |
|                       |           BF207246           |                               |
|                       |           BQ057974           |                               |
|                       |           CN310609           |                               |
|                       |           BG685020           |                               |
|                       |           BE871923           |                               |
|                       |           BF974919           |                               |
|                       |           BU681838           |                               |
|                       |           BU180121           |                               |
|                       |           BM046655           |                               |
|                       |           BG774414           |                               |
|                       |           BQ681321           |                               |
|                       |           BM044533           |                               |
|                       |           BM043890           |                               |
|                       |           CN310606           |                               |
|                       |           BF125227           |                               |
|                       |           BM310878           |                               |
|                       |           BQ689600           |                               |
|                       |           BE277371           |                               |
|                       |           BQ687384           |                               |
|                       |           BU162880           |                               |
|                       |           CN310601           |                               |
|                       |           BE727060           |                               |
|                       |           BP202072           |                               |
|                       |           BM795222           |                               |
|                       |           CV024971           |                               |
|                       |           AK128704           |                               |
|                       |           BQ958650           |                               |
|                       |           CB529698           |                               |
|                       |           BQ935365           |                               |
|                       |           BU502553           |                               |
|                       |           CN310605           |                               |
|                       |           AW025365           |                               |
|                       |           BU845323           |                               |
|                       |           BU846436           |                               |
|                       |           CN310603           |                               |
|                       |           CD622641           |                               |
|                       |           CD622639           |                               |
|                       |           BU183200           |                               |
|                       |           BE734682           |                               |
|                       |           BM798664           |                               |
|                       |           AL542488           |                               |
|                       |           CN310607           |                               |
|                       |           AI553864           |                               |
|                       |           CD622640           |                               |
|                       |           BE262281           |                               |
|                       |           CD622638           |                               |
|                       |           BE260471           |                               |
|                       |           BF037110           |                               |
|                       |           BE900662           |                               |
|                       |           BQ278755           |                               |
|                       |           CN310608           |                               |
|                       |           BP431679           |                               |
|                       |           BG475607           |                               |
|                       |           BE732714           |                               |
|                       |           CD370667           |                               |
|                       |           BU076950           |                               |
|                       |           CN310602           |                               |
|                       |           AW516126           |                               |
|                       |           BU507517           |                               |
|                       |           BQ028499           |                               |
|                       |           AW264500           |                               |
|                       |           BE394704           |                               |
|                       |           BQ286628           |                               |
|                       |           CA776389           |                               |
|                       |           BQ778150           |                               |
|                       |           BQ669864           |                               |
|                       |           BM692244           |                               |
|                       |           BI192580           |                               |
|                       |           AI564035           |                               |
|                       |           BM310580           |                               |
|                       |           BU679342           |                               |
|                       |           AA167728           |                               |
|                       |           AI570533           |                               |
|                       |           AI453071           |                               |
|                       |           BF893510           |                               |
|                       |           AI873574           |                               |
|                       |           AI421732           |                               |
|                       |           AI209022           |                               |
|                       |           AI435487           |                               |
|                       |           AI242463           |                               |
|                       |           AA034497           |                               |
|                       |           BQ013613           |                               |
|                       |           BM673514           |                               |
|                       |           BQ011046           |                               |
|                       |           BF036977           |                               |
|                       |           AI312028           |                               |
|                       |           BM091536           |                               |
|                       |           BQ013092           |                               |
|                       |           AI277626           |                               |
|                       |           BQ326493           |                               |
|                       |           BQ433694           |                               |
|                       |           AA541578           |                               |
|                       |           AI127962           |                               |
|                       |           BQ012606           |                               |
|                       |           AI005667           |                               |
|                       |           AI015207           |                               |
|                       |           AA828519           |                               |
|                       |           AI199524           |                               |
|                       |           AI159758           |                               |
|                       |           AI190769           |                               |
|                       |           BI033548           |                               |
|                       |           AW407760           |                               |
|                       |           BQ013306           |                               |
|                       |           BU588698           |                               |
|                       |           AI802676           |                               |
|                       |           AI262906           |                               |
|                       |           BP431192           |                               |
|                       |           AI338319           |                               |
|                       |           BQ013307           |                               |
|                       |           AI369273           |                               |
|                       |           AA129605           |                               |
|                       |           AI278879           |                               |
|                       |           BM673683           |                               |
|                       |           AW957029           |                               |
|                       |           BM091506           |                               |
|                       |             N70634           |                               |
|                       |           AI802002           |                               |
|                       |           AI350158           |                               |
|                       |           BQ013489           |                               |
|                       |             F31352           |                               |
|                       |             F26136           |                               |
|                       |           AA866059           |                               |
|                       |           CF528839           |                               |
|                       |           AA808436           |                               |
|                       |           AA961200           |                               |
|                       |           AA878447           |                               |
|                       |           AA887764           |                               |
|                       |           AI936751           |                               |
|                       |           BQ012240           |                               |
|                       |             N66007           |                               |
|                       |           AW246789           |                               |
|                       |           AA812815           |                               |
|                       |           BM686869           |                               |
|                       |           BF477864           |                               |
|                       |           BQ011024           |                               |
|                       |           AI695622           |                               |
|                       |           AA129606           |                               |
|                       |           CF529731           |                               |
|                       |           AA953323           |                               |
|                       |           BF438921           |                               |
|                       |           AW575647           |                               |
|                       |           AA402226           |                               |
|                       |             T77755           |                               |
|                       |           AA628332           |                               |
|                       |           AA507416           |                               |
|                       |           AA938516           |                               |
|                       |           AI312709           |                               |
|                       |           BF875528           |                               |
|                       |           AA594532           |                               |
|                       |           AA427821           |                               |
|                       |           AK095949           |                               |
|                       |           AK124078           |                               |
|                       |           BQ447873           |                               |
|                       |           BU633256           |                               |
|                       |           BM971668           |                               |
|                       |           AA609448           |                               |
|                       |           AA467871           |                               |
|                       |           BQ012177           |                               |
|                       |             W33136           |                               |
|                       |           AF401214           |                               |
|                       |           BU683039           |                               |
|                       |           BM989887           |                               |
|                       |           AK125003           |                               |
|                       |           CR602005           |                               |
|                       |           CA439410           |                               |
|                       |           AW070522           |                               |
|                       |           AW070534           |                               |
|                       |           AW733129           |                               |
|                       |           BE208083           |                               |
|                       |           AA781393           |                               |
|                       |           AI421976           |                               |
|                       |           AW292113           |                               |
|                       |           BF221887           |                               |
|                       | *Seq. mapped in strand Minus*| *Seq. mapped in strand Minus* |
|                       |           BM810341           |          AI843381             |
|                       |           AI480199           |            X65687             |
|                       |           BQ001789           |          BG864637             |
|                       |           BX476342           |          AW741535             |
|                       |           CB157453           |          AI844204             |
|                       |           CN289636           |          BG229711             |
|                       |           BM144775           |          AA003121             |
|                       |           AA029628           |          AW988210             |
|                       |           BQ001552           |          BU755069             |
|                       |           BU727641           |          BF163539             |
|                       |           AA494485           |          AI646868             |
|                       |           BM673084           |          BG918554             |
|                       |           AI865530           |          CO043065             |
|                       |           AA504155           |          BC066018             |
|                       |           AI089552           |          BG973537             |
|                       |           AI291929           |          BG918911             |
|                       |           AI300996           |          BU517894             |
|                       |           BE859036           |          BI078486             |
|                       |           BU737610           |          BI646191             |
|                       |           BU155664           |          BQ178186             |
|                       |           BU157486           |          BG922332             |
|                       |           BU162580           |          CB194486             |
|                       |           BM145160           |          BF180653             |
|                       |           CB161837           |          BG974354             |
|                       |           AA449097           |          BF119582             |
|                       |           AW874242           |          BI250368             |
|                       |           BM975724           |          AA048881             |
|                       |           BQ267476           |          BG518829             |
|                       |           BQ005258           |          BQ922635             |
|                       |           BQ961822           |          CK780583             |
|                       |           BQ962166           |          AI117741             |
|                       |           BM670067           |          BE281688             |
|                       |           CA444566           |          CA569603             |
|                       |           AI612771           |          BF161288             |
|                       |           CA425940           |          CB949835             |
|                       |           BQ002831           |          BG803864             |
|                       |           AW874674           |          BI645452             |
|                       |           CA450424           |          BI690290             |
|                       |           AW651711           |          BB214847             |
|                       |           BU738291           |          AK040758             |
|                       |           AW731935           |          BF715673             |
|                       |           BG402982           |          BF303482             |
|                       |           BU168075           |          BE336280             |
|                       |           BM680824           |          BU148032             |
|                       |           BM994967           |          BQ746371             |
|                       |           BQ183869           |          AA237366             |
|                       |           BQ446425           |          BG969199             |
|                       |           BU618960           |          BE376556             |
|                       |           BU682985           |          BI903737             |
|                       |           BU620367           |          AA240990             |
|                       |           BQ637809           |          BI250736             |
|                       |           BM973939           |          BF164887             |
|                       |           BM972569           |          CA976260             |
|                       |           BM972140           |          BI250349             |
|                       |           AI888939           |          BI904537             |
|                       |           BM982856           |            M94335             |
|                       |           BQ008580           |          BE653245             |
|                       |           BQ008663           |          CN671586             |
|                       |           BQ447163           |          BF451449             |
|                       |           CA310710           |          BM946946             |
|                       |           CA430654           |          BU703347             |
|                       |           BQ772547           |          CA553823             |
|                       |           AI564461           |          CF157326             |
|                       |           BM973016           |          BF682811             |
|                       |           BM977553           |          AA544493             |
|                       |           BQ008572           |          BQ919987             |
|                       |           BM982044           |          BF788714             |
|                       |           BU615579           |          CF156845             |
|                       |           BQ008554           |          CF749054             |
|                       |           CA428895           |          BI905628             |
|                       |           BQ772125           |          CA976883             |
|                       |           CA944766           |          BG920131             |
|                       |           CD369186           |          BG920406             |
|                       |           CB243110           |          BQ930899             |
|                       |           BM969100           |          BM949637             |
|                       |           CF243000           |          BF178041             |
|                       |           BU190388           |          BQ573506             |
|                       |           AI608649           |          BM947808             |
|                       |           AA063612           |          CF724011             |
|                       |           BU528351           |          BI148406             |
|                       |           AK094287           |          BG245046             |
|                       |           AK131465           |          BU057933             |
|                       |           BQ674055           |          CF723736             |
|                       |           BQ893916           |          BU053948             |
|                       |           BI084241           |          CB181825             |
|                       |           BC001737           |          BQ043073             |
|                       |           AK127193           |          BU707366             |
|                       |           BX648205           |          AW909920             |
|                       |           AK122894           |          BM950858             |
|                       |           BC000479           |          CA979281             |
|                       |           BQ021294           |          BG873038             |
|                       |             M63167           |          BE533315             |
|                       |           CR611982           |          BU614577             |
|                       |           BQ669021           |          BU054372             |
|                       |           BI084736           |          BM951651             |
|                       |           BM553228           |          CF730306             |
|                       |           BQ671889           |          CF725993             |
|                       |           BM424011           |          BF577579             |
|                       |           BM913552           |          BQ931088             |
|                       |           BU509545           |          BU053617             |
|                       |           BM913471           |          BM949704             |
|                       |           BM557125           |          BU612749             |
|                       |           BU538410           |          CK788852             |
|                       |           BM915608           |          BU054044             |
|                       |           BU169226           |          CD559822             |
|                       |           BM915484           |          CA567240             |
|                       |           AL536141           |          BM950969             |
|                       |           BX464003           |          BM947490             |
|                       |           BM914663           |          BM950888             |
|                       |           BQ688892           |          CA749163             |
|                       |           BM916829           |          BQ573613             |
|                       |           BM556640           |          BU614502             |
|                       |           BU190937           |          CF892089             |
|                       |           BU539837           |          CF914928             |
|                       |           BE799356           |          BQ442457             |
|                       |           BM912329           |          BF162742             |
|                       |           BG766418           |          BI663809             |
|                       |           BG757757           |          BE306624             |
|                       |           BG912721           |          CD562289             |
|                       |           BM924882           |          CA881147             |
|                       |           BQ224140           |          BU706194             |
|                       |           BM009667           |          AV118310             |
|                       |           BG753751           |          BY356657             |
|                       |           BG425414           |          BI152870             |
|                       |           BI918580           |          AA142710             |
|                       |           BG119777           |          BF178484             |
|                       |           BM794345           |          BE309600             |
|                       |           CD516456           |          CA566250             |
|                       |           BQ685848           |          CD805774             |
|                       |           BG686974           |          CA751239             |
|                       |           BM554307           |          BG077899             |
|                       |           BE379371           |          BQ964684             |
|                       |           BE545983           |          BY092168             |
|                       |           BM010216           |          BQ572588             |
|                       |           BU539015           |          BQ442321             |
|                       |           BQ675413           |          BF137744             |
|                       |           BF304156           |          BB636685             |
|                       |           BE887257           |          BY748556             |
|                       |           BE531158           |          AA469733             |
|                       |           BU944013           |          CA752139             |
|                       |           BG324361           |          BQ443119             |
|                       |           BQ921995           |          CA984661             |
|                       |           BM726207           |          BQ443358             |
|                       |           BQ883071           |          BY193045             |
|                       |           BQ943858           |          CK788732             |
|                       |           BG680870           |          BF015846             |
|                       |           BQ420473           |          AW323524             |
|                       |           BQ650062           |          BY188367             |
|                       |           BG422390           |          CA749833             |
|                       |           BU839543           |          BE226516             |
|                       |           BE902297           |          BB870442             |
|                       |           BE378926           |                               |
|                       |           BQ671801           |                               |
|                       |           BQ056945           |                               |
|                       |           BU537908           |                               |
|                       |           BE747353           |                               |
|                       |           BQ645497           |                               |
|                       |           BQ892906           |                               |
|                       |           BE867629           |                               |
|                       |           BE562983           |                               |
|                       |           BX371893           |                               |
|                       |           BI252049           |                               |
|                       |           BQ652325           |                               |
|                       |           BM792661           |                               |
|                       |           BE207117           |                               |
|                       |           BE531031           |                               |
|                       |           CN289644           |                               |
|                       |           BQ650038           |                               |
|                       |           BQ644377           |                               |
|                       |           AW411396           |                               |
|                       |           BE294049           |                               |
|                       |           BQ653144           |                               |
|                       |           CN289635           |                               |
|                       |           BQ059884           |                               |
|                       |           BQ651798           |                               |
|                       |           BQ647389           |                               |
|                       |           BE410711           |                               |
|                       |           BQ651112           |                               |
|                       |           BE296103           |                               |
|                       |           BM928704           |                               |
|                       |           BQ653318           |                               |
|                       |           BG324688           |                               |
|                       |           BG327305           |                               |
|                       |           BE562930           |                               |
|                       |           BE901515           |                               |
|                       |           BU540732           |                               |
|                       |           BF839498           |                               |
|                       |           BF839535           |                               |
|                       |           BU558030           |                               |
|                       |           BQ900346           |                               |
|                       |           AI356208           |                               |
|                       |           BQ644954           |                               |
|                       |           BQ882543           |                               |
|                       |             H20593           |                               |
|                       |           BQ931710           |                               |
|                       |           BQ879611           |                               |
|                       |           BE735947           |                               |
|                       |           BQ959043           |                               |
|                       |           BQ053423           |                               |
|                       |           BI770349           |                               |
|                       |           BI916527           |                               |
|                       |           BM563782           |                               |
|                       |           BE264427           |                               |
|                       |           BE278916           |                               |
|                       |           BQ434776           |                               |
|                       |           AW249361           |                               |
|                       |           BU529023           |                               |
|                       |           BQ278172           |                               |
|                       |           BM554053           |                               |
|                       |           BQ653320           |                               |
|                       |           BE925238           |                               |
|                       |           BE407373           |                               |
|                       |           BG034703           |                               |
|                       |           BQ649961           |                               |
|                       |           CN289638           |                               |
|                       |           BU558051           |                               |
|                       |           BQ927886           |                               |
|                       |           BQ672737           |                               |
|                       |           CF139680           |                               |
|                       |           BQ649462           |                               |
|                       |           BQ961715           |                               |
|                       |           BM045203           |                               |
|                       |           BG675825           |                               |
|                       |           BE315179           |                               |
|                       |           BE278001           |                               |
|                       |           BF724201           |                               |
|                       |           BQ055844           |                               |
|                       |           BU553150           |                               |
|                       |           BQ645112           |                               |
|                       |           BE379221           |                               |
|                       |           BE253906           |                               |
|                       |           CA487318           |                               |
|                       |           BQ920706           |                               |
|                       |           BQ645075           |                               |
|                       |           BM913066           |                               |
|                       |           BG775230           |                               |
|                       |           BE206796           |                               |
|                       |           BF026763           |                               |
|                       |           BG747347           |                               |
|                       |           BG831962           |                               |
|                       |           BQ680446           |                               |
|                       |           BE727833           |                               |
|                       |           BQ669098           |                               |
|                       |           BQ675519           |                               |
|                       |           BG118176           |                               |
|                       |           BG478629           |                               |
|                       |           BE378800           |                               |
|                       |           BG822833           |                               |
|                       |           CD516503           |                               |
|                       |           BE245919           |                               |
|                       |           BM146016           |                               |
|                       |           BM193040           |                               |
|                       |           CN289648           |                               |
|                       |           AI880088           |                               |
|                       |           AI207939           |                               |
|                       |           BM908506           |                               |
|                       |           BE273341           |                               |
|                       |           BM794133           |                               |
|                       |           BU197307           |                               |
|                       |           BG419945           |                               |
|                       |           BU838146           |                               |
|                       |           BQ646612           |                               |
|                       |           BG472686           |                               |
|                       |           BG747009           |                               |
|                       |           BE379531           |                               |
|                       |           BG421649           |                               |
|                       |           BE730039           |                               |
|                       |           BM826791           |                               |
|                       |           BI192718           |                               |
|                       |           AA504424           |                               |
|                       |           AA551706           |                               |
|                       |           BF027450           |                               |
|                       |           BE263985           |                               |
|                       |           BF838153           |                               |
|                       |           BE395750           |                               |
|                       |           BM045016           |                               |
|                       |           BE258340           |                               |
|                       |           BF339545           |                               |
|                       |           BG330744           |                               |
|                       |           BG396099           |                               |
|                       |           BM050128           |                               |
|                       |           BE259159           |                               |
|                       |           BI457131           |                               |
|                       |           BM549896           |                               |
|                       |           BE728731           |                               |
|                       |           BI160112           |                               |
|                       |           BQ108715           |                               |
|                       |           BG746629           |                               |
|                       |           BF838143           |                               |
|                       |           BQ933878           |                               |
|                       |           BF726149           |                               |
|                       |           BG954606           |                               |
|                       |           CN289642           |                               |
|                       |           CN289645           |                               |
|                       |           CN289651           |                               |
|                       |           BU931036           |                               |
|                       |           BM559169           |                               |
|                       |           BP330150           |                               |
|                       |           BQ937145           |                               |
|                       |           BE393829           |                               |
|                       |           AL548951           |                               |
|                       |           BE408512           |                               |
|                       |           BQ673953           |                               |
|                       |           BQ880608           |                               |
|                       |           BQ944765           |                               |
|                       |             X61037           |                               |
|                       |           BE387182           |                               |
|                       |           BM083376           |                               |
|                       |           AV645642           |                               |
|                       |           BE742153           |                               |
|                       |           BP269557           |                               |
|                       |           BF526630           |                               |
|                       |           BE396078           |                               |
|                       |           CN289646           |                               |
|                       |           BE275825           |                               |
|                       |           BG747298           |                               |
|                       |           BG329794           |                               |
|                       |           BU541679           |                               |
|                       |           BE378536           |                               |
|                       |           CF141089           |                               |
|                       |           BF916648           |                               |
|                       |           BG436898           |                               |
|                       |           BG489859           |                               |
|                       |           BU188847           |                               |
|                       |           BU552487           |                               |
|                       |           BE926392           |                               |
|                       |           AA325725           |                               |
|                       |           BE818959           |                               |
|                       |           BE818954           |                               |
|                       |           BU502645           |                               |
|                       |           BU502654           |                               |
|                       |           BU543038           |                               |
|                       |           BG775183           |                               |
|                       |           BQ960714           |                               |
|                       |           BQ062097           |                               |
|                       |           BE388487           |                               |
|                       |           AA319181           |                               |
|                       |           BG323416           |                               |
|                       |           BE840080           |                               |
|                       |           BE840043           |                               |
|                       |             H38502           |                               |
|                       |           BG874829           |                               |
|                       |           BI964126           |                               |
|                       |           BE840038           |                               |
|                       |           BM017594           |                               |
|                       |           CK903763           |                               |
|                       |           BF751168           |                               |
|                       |           CF146674           |                               |
|                       |           CF551901           |                               |
|                       |           BU542814           |                               |
|                       |           BU178620           |                               |
|                       |           BI224520           |                               |
|                       |           AL545564           |                               |
|                       |           BI906769           |                               |
|                       |           AA234994           |                               |
|                       |           BU738539           |                               |
|                       |           BX647722           |                               |
|                       |           BI918732           |                               |
|                       |           BG289868           |                               |
|                       |           BU177895           |                               |
|                       |           CD110018           |                               |
|                       |           BQ223862           |                               |
|                       |           BE390966           |                               |
|                       |           BE617252           |                               |
|                       |           BQ961156           |                               |
|                       |           BG913158           |                               |
|                       |           BU857383           |                               |
|                       |           BE900914           |                               |
|                       |           BF955734           |                               |
|                       |           BM699870           |                               |
|                       |             W77811           |                               |
|                       |           CD517703           |                               |
|                       |           BU163703           |                               |
|                       |           BE388551           |                               |
|                       |           BM044140           |                               |
|                       |           CV339502           |                               |
|                       |           CV339034           |                               |
|                       |           BE279562           |                               |
|                       |           BG424114           |                               |
|                       |           CD558884           |                               |
|                       |           BG821633           |                               |
|                       |           BQ673312           |                               |
|                       |           AL534146           |                               |
|                       |           BQ306237           |                               |
|                       |           BQ649377           |                               |
|                       |           BQ652621           |                               |
|                       |           BQ057212           |                               |
|                       |           BQ919596           |                               |
|                       |           BQ308692           |                               |
|                       |           AL040775           |                               |
|                       |           BE514899           |                               |
|                       |           BQ062455           |                               |
|                       |           BM010723           |                               |
|                       |           BG748551           |                               |
|                       |           BG750090           |                               |
|                       |           BP267761           |                               |
|                       |           BE298845           |                               |
|                       |           BU543130           |                               |
|                       |           BG291519           |                               |
|                       |             H63703           |                               |
|                       |           BI820277           |                               |
|                       |           BE392368           |                               |
|                       |           BE395139           |                               |
|                       |           CV339450           |                               |
|                       |           BE880543           |                               |
|                       |           BU543009           |                               |
|                       |           BI868504           |                               |
|                       |           BG678197           |                               |
|                       |           BU542028           |                               |
|                       |           CD672541           |                               |
|                       |           BQ066077           |                               |
|                       |           BI193992           |                               |
|                       |           AA322663           |                               |
|                       |           BQ303373           |                               |
|                       |           BM817552           |                               |
|                       |           BE904226           |                               |
|                       |           BE254984           |                               |
|                       |           BM561502           |                               |
|                       |           BI160059           |                               |
|                       |           BG744528           |                               |
|                       |           BP202682           |                               |
|                       |           BG331446           |                               |
|                       |           BE797515           |                               |
|                       |           AA334732           |                               |
|                       |           BQ222387           |                               |
|                       |           BG749524           |                               |
|                       |           BP306678           |                               |
|                       |           BG825964           |                               |
|                       |           BG822083           |                               |
|                       |           BG748350           |                               |
|                       |           BG747687           |                               |
|                       |           BE745959           |                               |
|                       |           BM853559           |                               |
|                       |           BM047935           |                               |
|                       |           BG323587           |                               |
|                       |           CB265879           |                               |
|                       |           BQ306709           |                               |
|                       |           BG979099           |                               |
|                       |           BG333953           |                               |
|                       |           BE563589           |                               |
|                       |           BQ073758           |                               |
|                       |           BE390437           |                               |
|                       |           BG825333           |                               |
|                       |           BU543254           |                               |
|                       |           BU543253           |                               |
|                       |           BE736507           |                               |
|                       |           BG420723           |                               |
|                       |           BG757556           |                               |
|                       |           BG748177           |                               |
|                       |           BU543010           |                               |
|                       |           BM552046           |                               |
|                       |           BG686874           |                               |
|                       |           BU543336           |                               |
|                       |           BP363336           |                               |
|                       |           BM917487           |                               |
|                       |           BG335906           |                               |
|                       |           BG677291           |                               |
|                       |           BE901331           |                               |
|                       |           BG397337           |                               |
|                       |           BF974205           |                               |
|                       |           BE735049           |                               |
|                       |           BF307576           |                               |
|                       |           BQ691617           |                               |
|                       |           AW498827           |                               |
|                       |           CN289647           |                               |
|                       |           AW006645           |                               |
|                       |           BE933139           |                               |
|                       |           BF340053           |                               |
|                       |           BF309126           |                               |
|                       |           BP242730           |                               |
|                       |           BF314042           |                               |
|                       |           BE720305           |                               |
|                       |           BQ189272           |                               |
|                       |           BE278104           |                               |
|                       |           CV341540           |                               |
|                       |           BE868558           |                               |
|                       |           BM742922           |                               |
|                       |           BE933141           |                               |
|                       |           BQ376702           |                               |
|                       |           BF036391           |                               |
|                       |           BE146382           |                               |
|                       |           AI909623           |                               |
|                       |           BQ958744           |                               |
|                       |           BE265132           |                               |
|                       |           BG489971           |                               |
|                       |           BG421689           |                               |
|                       |           BM928115           |                               |
|                       |           BE311665           |                               |
|                       |           BI913318           |                               |
|                       |           BI913667           |                               |
|                       |           BG110670           |                               |
|                       |           BP293968           |                               |
|                       |           BF203633           |                               |
|                       |           BM773284           |                               |
|                       |           BF203892           |                               |
|                       |           BM910161           |                               |
|                       |           AI079186           |                               |
|                       |           BU950273           |                               |
|                       |             C75024           |                               |
|                       |           BG340823           |                               |
|                       |           BE742501           |                               |
|                       |           AL134271           |                               |
|                       |           BG424273           |                               |
|                       |           BF827226           |                               |
|                       |           BG472132           |                               |
|                       |           BG472130           |                               |
|                       |           BE735675           |                               |
|                       |           BE772919           |                               |
|                       |           BP269293           |                               |
|                       |           BE270136           |                               |
|                       |           BQ674693           |                               |
|                       |           BE736125           |                               |
|                       |           BQ645774           |                               |
|                       |           BQ652616           |                               |
|                       |           BE772946           |                               |
|                       |           AW673172           |                               |
|                       |           BE772918           |                               |
|                       |             W72696           |                               |
|                       |           AI951141           |                               |
|                       |           BG757720           |                               |
|                       |           AW575721           |                               |
|                       |           AA910085           |                               |
|                       |           BI029435           |                               |
|                       |           AA573336           |                               |
|                       |           AA444055           |                               |
|                       |           AI092106           |                               |
|                       |           AA883137           |                               |
|                       |           BG326005           |                               |
|                       |           BQ933482           |                               |
|                       |           AI283656           |                               |
|                       |           BE315081           |                               |
|                       |           AA768153           |                               |
|                       |           AA716185           |                               |
|                       |           AA460547           |                               |
|                       |           AA746277           |                               |
|                       |           BE772940           |                               |
|                       |           BE772929           |                               |
|                       |           BE700934           |                               |
|                       |           CV027158           |                               |
|                       |           AI084659           |                               |
|                       |           BQ328274           |                               |
|                       |           BQ922042           |                               |
|                       |           BF976442           |                               |
|                       |           AI140219           |                               |
|                       |           BG419334           |                               |
|                       |           AI277694           |                               |
|                       |           BM005829           |                               |
|                       |           BQ064036           |                               |
|                       |           BQ062624           |                               |
|                       |           BQ642449           |                               |
|                       |           AI359126           |                               |
|                       |           AW015062           |                               |
|                       |           BU502667           |                               |
|                       |           BQ672937           |                               |
|                       |           BQ937357           |                               |
|                       |           AW271325           |                               |
|                       |           BG774197           |                               |
|                       |           AW592115           |                               |
|                       |           AA349777           |                               |
|                       |           BU543267           |                               |
|                       |           BG423001           |                               |
|                       |           BE769123           |                               |
|                       |           BX488421           |                               |
|                       |           BF953482           |                               |
|                       |           BU501435           |                               |
|                       |           BF953474           |                               |
|                       |           BU150779           |                               |
|                       |           BQ957324           |                               |
|                       |           BU501536           |                               |
|                       |           BU838162           |                               |
|                       |           BF953536           |                               |
|                       |           BU855904           |                               |
|                       |           BE645459           |                               |
|                       |           CN289643           |                               |
|                       |           BU554049           |                               |
|                       |           BU542599           |                               |
|                       |           BI601404           |                               |
|                       |             H25749           |                               |
|                       |           BM149333           |                               |
|                       |           BM148200           |                               |
|                       |           AA305957           |                               |
|                       |           BM194195           |                               |
|                       |           AW411138           |                               |
|                       |           AW080998           |                               |
|                       |           AI597912           |                               |
|                       |           AI202846           |                               |
|                       |           AI950060           |                               |
|                       |           AW300180           |                               |
|                       |           AI220456           |                               |
|                       |           AI696231           |                               |
|                       |             H41913           |                               |
|                       |             R72064           |                               |
|                       |           AA927922           |                               |
+-----------------------+------------------------------+-------------------------------+
|      #   63           | *Seq. mapped in strand Plus* | *Seq. mapped in strand Plus*  |
|                       |           AK055539           |          AK037109             |
|                       |           AY358554           |          AI662211             |
|                       |           CR590889           |          AK046791             |
|                       |           BC025407           |          CO044448             |
|                       |           AK055654           |          BQ478547             |
|                       |           CR608344           |          BE944246             |
|                       |           CR612367           |          AI448888             |
|                       |                              |          AW124414             |
|                       |                              |          AK047119             |
|                       | *Seq. mapped in strand Minus*| *Seq. mapped in strand Minus* |
|                       |           AK096925           |          BG080894             |
|                       |                              |          BB646620             |
|                       |                              |          BB646601             |
|                       |                              |                               |
|                       |                              |                               |
|                       |                              |                               |
|                       |                              |                               |
|                       |                              |                               |
|                       |                              |                               |
+-----------------------+------------------------------+-------------------------------+
|      #   64           | *Seq. mapped in strand Plus* | *Seq. mapped in strand Plus*  |
|                       |           BC053410           |          AW488468             |
|                       | *Seq. mapped in strand Minus*| *Seq. mapped in strand Minus* |
|                       |           AF480435           |          AK032110             |
+-----------------------+------------------------------+-------------------------------+
|      #   65           | *Seq. mapped in strand Plus* | *Seq. mapped in strand Plus*  |
|                       |           AA478714           |            W14950             |
|                       |           AA594093           |                               |
|                       |           AI122800           |                               |
|                       |           CF993913           |                               |
|                       | *Seq. mapped in strand Minus*| *Seq. mapped in strand Minus* |
|                       |           BC029953           |          BC058986             |
|                       |           AI247282           |          AI837037             |
|                       |           AW451719           |          BC028832             |
|                       |           AW207542           |          BI966366             |
|                       |           AI952512           |          BF582314             |
|                       |           BX109850           |          BC048850             |
|                       |           AW136603           |          AA575227             |
|                       |           AW206758           |          AA002315             |
|                       |           BF511463           |          AI006023             |
|                       |           BX642086           |          BF098977             |
|                       |           AW204138           |          AA388251             |
|                       |             W46415           |          AA387674             |
|                       |           CD366628           |          BI248011             |
|                       |           BM669452           |          BF237238             |
|                       |           AW138545           |          BF236603             |
|                       |           BM977040           |          BY735706             |
|                       |           AI675364           |          AA387584             |
|                       |           AA551778           |          BE570158             |
|                       |             N94432           |          AA387763             |
|                       |           CB150979           |          BF303331             |
|                       |           AK096227           |          AA145104             |
|                       |           CD366824           |          BE192798             |
|                       |           AA478580           |          BI332010             |
|                       |           AA495786           |          BB858965             |
|                       |           BM670333           |          BY030058             |
|                       |           CD365124           |          BQ179549             |
|                       |           CF541165           |                               |
|                       |             X75342           |                               |
|                       |           BM913833           |                               |
|                       |           BM712054           |                               |
|                       |           BG751150           |                               |
|                       |           AA495721           |                               |
|                       |           BF913030           |                               |
|                       |           BG420490           |                               |
|                       |           BM555562           |                               |
|                       |           BM697539           |                               |
|                       |           AW362013           |                               |
|                       |           AW963518           |                               |
|                       |           AV751809           |                               |
|                       |           BG002301           |                               |
|                       |           BF058505           |                               |
|                       |           BE795043           |                               |
|                       |           BG054672           |                               |
|                       |           AI814254           |                               |
|                       |           AA434156           |                               |
|                       |           AA402320           |                               |
|                       |           AA428797           |                               |
|                       |           AA437346           |                               |
|                       |           AI924774           |                               |
|                       |           AI380607           |                               |
|                       |           AI814249           |                               |
|                       |           AI925114           |                               |
|                       |           BF115944           |                               |
|                       |             T29732           |                               |
|                       |           AA379624           |                               |
|                       |           AI566242           |                               |
|                       |           AI131199           |                               |
|                       |           AI885533           |                               |
|                       |           AA411304           |                               |
|                       |           AA434274           |                               |
|                       |           AA427595           |                               |
|                       |           AA291472           |                               |
|                       |           AA425928           |                               |
|                       |           AW299487           |                               |
|                       |           BX502427           |                               |
|                       |           AW297991           |                               |
|                       |           BE385933           |                               |
|                       |           AA429266           |                               |
|                       |           BG326702           |                               |
|                       |           BE562483           |                               |
|                       |           AK001225           |                               |
+-----------------------+------------------------------+-------------------------------+
|      #   66           | *Seq. mapped in strand Plus* | *Seq. mapped in strand Plus*  |
|                       |           AL530930           |          BU524604             |
|                       |           CR612694           |          BY326063             |
|                       |           BC008083           |          BY331701             |
|                       |           BG323468           |          BY124165             |
|                       |           BP229581           |          BY327660             |
|                       |           BE277703           |          BY310372             |
|                       |           BF025744           |          BY775166             |
|                       |           BP275841           |          BY326506             |
|                       |           BP236749           |          BY309661             |
|                       |           BP375224           |          BY074780             |
|                       |           BP215632           |          BY316739             |
|                       |           BP287642           |          BY770431             |
|                       |           BG472181           |          BY147347             |
|                       |           BC064934           |          BY014761             |
|                       |           BU188288           |          BY182593             |
|                       |           BE251280           |          BY132962             |
|                       |           BP263939           |          BY314087             |
|                       |           BP234486           |          BY265665             |
|                       |           CB960906           |          BY255188             |
|                       |           CB989153           |          BY754363             |
|                       |           BP349575           |          BY749885             |
|                       |           BP270078           |          BY102223             |
|                       |           AU133156           |          BY323535             |
|                       |           AK001711           |          BY325864             |
|                       |           BP310497           |          BQ256396             |
|                       |           BP310470           |          BU058444             |
|                       |           CN426719           |          BU704119             |
|                       |           BP232507           |          CK341794             |
|                       |           BE797571           |          BU055736             |
|                       |           BP332172           |          CD355447             |
|                       |           BG339275           |          CF537776             |
|                       |           AL110300           |          BU054345             |
|                       |           BP337471           |          CB520672             |
|                       |           BP257707           |          CA324727             |
|                       |           BP270486           |          CB520283             |
|                       |           BE797102           |          BI251831             |
|                       |           BE793939           |          BC062206             |
|                       |           BU595600           |          BE859884             |
|                       |           BP285722           |          BI853251             |
|                       |           CN426705           |          BU058434             |
|                       |           BP241436           |          BU055834             |
|                       |           BC063615           |          BU706386             |
|                       |           BF091954           |          CA528876             |
|                       |           BF760139           |          BU058398             |
|                       |           BE868461           |          CA327821             |
|                       |           CN426708           |          BC064466             |
|                       |           BX472569           |          BY286737             |
|                       |           CB132488           |          BY254873             |
|                       |             M86008           |          BI660194             |
|                       |           CN426697           |          BY276594             |
|                       |           BX371465           |          BY264919             |
|                       |           BF684601           |          BY273708             |
|                       |           AW963807           |          BB853517             |
|                       |           BP218635           |          BY108820             |
|                       |           BQ478446           |          BY106477             |
|                       |           BI868343           |          BY108692             |
|                       |           BE243122           |          BY107003             |
|                       |           BM146230           |          BY299425             |
|                       |           BF676384           |          BY278875             |
|                       |           AW992367           |          BY291468             |
|                       |           AW992313           |          BY238102             |
|                       |           AW992311           |          BY266457             |
|                       |           AU117523           |          BY248060             |
|                       |           AK027279           |          BY233431             |
|                       |           CA421773           |          BY267160             |
|                       |           CN426712           |          BY260258             |
|                       |           CB157898           |          BY002543             |
|                       |           AI640298           |          AI604050             |
|                       |           CB268521           |          BY125046             |
|                       |           BU500857           |          BY331580             |
|                       |           BQ478199           |          BY256648             |
|                       |           AU130951           |          BY257677             |
|                       |           AK027633           |          BY266177             |
|                       |           AI492589           |          BY268371             |
|                       |           BU627475           |          BY257178             |
|                       |           AI984640           |          CN704878             |
|                       |           BM794348           |          CN700669             |
|                       |           CF134883           |          AK028475             |
|                       |           CN426715           |          BY292244             |
|                       |           AA985228           |          BY106975             |
|                       |           BI760798           |          BY263956             |
|                       |           BI761136           |          BY264469             |
|                       |           BG257808           |          BY271188             |
|                       |           AA037170           |          BY251498             |
|                       |           BG616046           |          BY263322             |
|                       |             R59151           |          BY241336             |
|                       |           BX115651           |          BY256147             |
|                       |           AA354520           |          AA636820             |
|                       |           AL530929           |          BY243682             |
|                       |           CN426695           |          BY256775             |
|                       |           AI927459           |          BY293866             |
|                       |           CK005295           |          BY277594             |
|                       |           CN426692           |          BY261984             |
|                       |           CN426687           |          CA979913             |
|                       |           BQ641900           |          CA876572             |
|                       |           BM794305           |          CK639791             |
|                       |           BQ340948           |          CF531078             |
|                       |           BF439080           |          CF729846             |
|                       |           AI539361           |          CN535103             |
|                       |           BX643289           |          CK781281             |
|                       |           BM834375           |          CN537709             |
|                       |           CB148061           |          CN685277             |
|                       |           AW900483           |          CN534464             |
|                       |           AW070463           |          CN538773             |
|                       |           BG620004           |          CN536728             |
|                       |           CN426694           |          BC031456             |
|                       |           BX475042           |          CF907586             |
|                       |           BQ881606           |          CN525771             |
|                       |           BQ270710           |          CN677765             |
|                       |           BQ787314           |          CA328509             |
|                       |           BX495829           |          CB519525             |
|                       |           AA853067           |          CN525563             |
|                       |           BQ015390           |          CN457915             |
|                       |           CA419142           |          BY177649             |
|                       |           BU070312           |          BY176078             |
|                       |           AA151003           |          BY748874             |
|                       |           BM989346           |          CB521885             |
|                       |             W81208           |          CN530399             |
|                       |           BP220380           |          CN461661             |
|                       |           AU144332           |          CB205661             |
|                       |           BX957105           |          CD350398             |
|                       |           BU542280           |          BU058347             |
|                       |           AA853066           |          CN667322             |
|                       |           BQ070246           |          CK634556             |
|                       |           CB047176           |          CK781702             |
|                       |           AA374936           |          BY720000             |
|                       |           BE501876           |          CA562586             |
|                       |           AK124592           |          CK389244             |
|                       |           CR627457           |          CA325037             |
|                       |           AI765122           |          BU056571             |
|                       |           BM981600           |          CD348916             |
|                       |           AI479259           |          CF733085             |
|                       |           BQ001699           |          CN534333             |
|                       |           AW242641           |          CD564308             |
|                       |           BQ001626           |          CN527124             |
|                       |           BQ019525           |          CN527201             |
|                       |           CA418765           |          BU057384             |
|                       |           AI675599           |          CD348106             |
|                       |           BU679302           |          BQ570501             |
|                       |           BM664526           |          CN535629             |
|                       |           AI863723           |          CF535844             |
|                       |           AW452207           |          CB521937             |
|                       |           AI753833           |          CK639889             |
|                       |           CB051356           |          BI657644             |
|                       |           AI185583           |          CD349271             |
|                       |           BG707038           |          CK636254             |
|                       |             H04810           |          CN458947             |
|                       |           CR592186           |          CB247047             |
|                       |           AA150683           |          CN457348             |
|                       |           CN426714           |          CN459381             |
|                       |           AI037848           |          CD551811             |
|                       |           CA437796           |          CA752261             |
|                       |           AI752978           |          CA327596             |
|                       |           AI625781           |          CK624314             |
|                       |           CR625731           |          BQ180578             |
|                       |           BU616580           |          CN535896             |
|                       |           CA446261           |          CD349039             |
|                       |           BQ017993           |          CA328720             |
|                       |           CA416684           |          CF733282             |
|                       |           CA422047           |          CA326993             |
|                       |           BM144999           |          CF742957             |
|                       |           AI479445           |          CN534569             |
|                       |             N64134           |          CK639998             |
|                       |           BF109558           |          CK639017             |
|                       |           AI741944           |          CN526580             |
|                       |           CA414463           |          CN456999             |
|                       |           BQ024470           |          CK639494             |
|                       |           AI742961           |          CN535588             |
|                       |           BQ029393           |          CK390231             |
|                       |           BM683852           |          CF535358             |
|                       |           BQ024550           |          CB525274             |
|                       |           AA680010           |          CA569138             |
|                       |           BU753370           |          BU708614             |
|                       |           AA742626           |          BQ745973             |
|                       |           AI141593           |          CN456202             |
|                       |           AI803964           |          BQ771480             |
|                       |           AI832259           |          CN461322             |
|                       |           AW768571           |          CN531638             |
|                       |           CN426700           |          BQ567747             |
|                       |           AI754318           |          CA567424             |
|                       |           CA448922           |          CN457048             |
|                       |           AW014355           |          BQ769298             |
|                       |           AI203560           |          CD348587             |
|                       |           AI697606           |          CN538160             |
|                       |           AI401428           |          CB521464             |
|                       |           AI580485           |          CK639613             |
|                       |           AA035422           |          CD348693             |
|                       |           AA743540           |          CF742462             |
|                       |           BG943971           |          CN669152             |
|                       |           BU678663           |          CN534937             |
|                       |           AA832208           |          CN454840             |
|                       |           AI038658           |          CF728366             |
|                       |           AL353942           |          BU055505             |
|                       |                              |          CD578642             |
|                       |                              |          CA891780             |
|                       |                              |          CA889163             |
|                       |                              |          CN457363             |
|                       |                              |          BY096014             |
|                       |                              |          CN536367             |
|                       |                              |          CN525493             |
|                       |                              |          CN459307             |
|                       |                              |          CA563715             |
|                       |                              |          CA563013             |
|                       |                              |          BF578450             |
|                       |                              |          CN665027             |
|                       |                              |          CF532505             |
|                       |                              |          CN525020             |
|                       |                              |          AA000408             |
|                       |                              |          CK635314             |
|                       |                              |          CD551773             |
|                       |                              |          CA540553             |
|                       |                              |          CF892106             |
|                       |                              |          CD349080             |
|                       |                              |          BY720116             |
|                       |                              |          CN526849             |
|                       |                              |          CK640117             |
|                       |                              |          CD773721             |
|                       |                              |          CK633720             |
|                       |                              |          CF735333             |
|                       |                              |          CN461597             |
|                       |                              |          BM963438             |
|                       |                              |          CN454966             |
|                       |                              |          BI854039             |
|                       |                              |          CK639073             |
|                       |                              |          CK634512             |
|                       |                              |          CN457852             |
|                       |                              |          CB246917             |
|                       |                              |          CK633708             |
|                       |                              |          CN525811             |
|                       |                              |          CF746991             |
|                       |                              |          CN525799             |
|                       |                              |          CK635064             |
|                       |                              |          CN456065             |
|                       |                              |          CK635289             |
|                       |                              |          BQ769411             |
|                       |                              |          CN663577             |
|                       |                              |          CK634372             |
|                       |                              |          CK635105             |
|                       |                              |          CF612568             |
|                       |                              |          CF903099             |
|                       |                              |          CB518369             |
|                       |                              |          CN525552             |
|                       |                              |          CN459587             |
|                       |                              |          CN458368             |
|                       |                              |          CN457036             |
|                       |                              |          BU757264             |
|                       |                              |          CN457946             |
|                       |                              |          CK635364             |
|                       |                              |          CN673365             |
|                       |                              |          BI664370             |
|                       |                              |          CK639960             |
|                       |                              |          CA568643             |
|                       |                              |          BU702351             |
|                       |                              |          CN671856             |
|                       |                              |          CN689454             |
|                       |                              |          BY599643             |
|                       |                              |          CF734222             |
|                       |                              |            W45877             |
|                       |                              |          BX518974             |
|                       |                              |          CF586816             |
|                       |                              |          BF224922             |
|                       |                              |          BI788539             |
|                       |                              |          BQ084813             |
|                       |                              |          BG798075             |
|                       |                              |          BF454823             |
|                       |                              |          CK383825             |
|                       |                              |          AK031522             |
|                       |                              |          AK033173             |
|                       |                              |          AA517452             |
|                       |                              |          AK018083             |
|                       |                              |          AK075989             |
|                       |                              |          AK020155             |
|                       |                              |          BM940457             |
|                       |                              |          CO042161             |
|                       |                              |          CF949547             |
|                       |                              |          CF949901             |
|                       |                              |          AI853361             |
|                       |                              |          BG797685             |
|                       |                              |          BI793775             |
|                       |                              |          CA944254             |
|                       |                              |          BI714077             |
|                       |                              |          BU951237             |
|                       |                              |          CA951231             |
|                       |                              |          CA774391             |
|                       |                              |          BU700280             |
|                       |                              |          CA947305             |
|                       |                              |          BI790485             |
|                       |                              |          CB177719             |
|                       |                              |          CB177659             |
|                       |                              |          BG798169             |
|                       |                              |          BU788261             |
|                       |                              |          BI465752             |
|                       |                              |          BM877945             |
|                       |                              |          CA947640             |
|                       |                              |          BQ417708             |
|                       |                              |          CF533709             |
|                       |                              |          BI499103             |
|                       |                              |          CB848347             |
|                       |                              |          BI465665             |
|                       |                              |          BG797358             |
|                       |                              |          AA238809             |
|                       |                              |          AA145253             |
|                       |                              |          CF747028             |
|                       |                              |          CA951244             |
|                       |                              |          BM052307             |
|                       |                              |          CA951197             |
|                       | *Seq. mapped in strand Minus*| *Seq. mapped in strand Minus* |
|                       |           CA415574           |          AW493840             |
|                       |           AI654462           |          BE992267             |
|                       |           BX101590           |          BE987591             |
|                       |           AI654452           |          BU788262             |
|                       |           AI184571           |          BE947684             |
|                       |           AW898888           |          AI836723             |
|                       |           AI750798           |          AW047883             |
|                       |                              |          BB655312             |
|                       |                              |          BI499845             |
|                       |                              |          CK639030             |
|                       |                              |                               |
|                       |                              |                               |
|                       |                              |                               |
|                       |                              |                               |
|                       |                              |                               |
|                       |                              |                               |
|                       |                              |                               |
|                       |                              |                               |
|                       |                              |                               |
|                       |                              |                               |
|                       |                              |                               |
|                       |                              |                               |
|                       |                              |                               |
|                       |                              |                               |
|                       |                              |                               |
|                       |                              |                               |
|                       |                              |                               |
|                       |                              |                               |
|                       |                              |                               |
|                       |                              |                               |
|                       |                              |                               |
|                       |                              |                               |
|                       |                              |                               |
|                       |                              |                               |
|                       |                              |                               |
|                       |                              |                               |
|                       |                              |                               |
|                       |                              |                               |
|                       |                              |                               |
|                       |                              |                               |
|                       |                              |                               |
|                       |                              |                               |
|                       |                              |                               |
|                       |                              |                               |
|                       |                              |                               |
|                       |                              |                               |
|                       |                              |                               |
|                       |                              |                               |
|                       |                              |                               |
|                       |                              |                               |
|                       |                              |                               |
|                       |                              |                               |
|                       |                              |                               |
|                       |                              |                               |
|                       |                              |                               |
|                       |                              |                               |
|                       |                              |                               |
|                       |                              |                               |
|                       |                              |                               |
|                       |                              |                               |
|                       |                              |                               |
|                       |                              |                               |
|                       |                              |                               |
|                       |                              |                               |
|                       |                              |                               |
|                       |                              |                               |
|                       |                              |                               |
|                       |                              |                               |
|                       |                              |                               |
|                       |                              |                               |
|                       |                              |                               |
|                       |                              |                               |
|                       |                              |                               |
|                       |                              |                               |
|                       |                              |                               |
|                       |                              |                               |
|                       |                              |                               |
|                       |                              |                               |
|                       |                              |                               |
|                       |                              |                               |
|                       |                              |                               |
|                       |                              |                               |
|                       |                              |                               |
|                       |                              |                               |
|                       |                              |                               |
|                       |                              |                               |
|                       |                              |                               |
|                       |                              |                               |
|                       |                              |                               |
|                       |                              |                               |
|                       |                              |                               |
|                       |                              |                               |
|                       |                              |                               |
|                       |                              |                               |
|                       |                              |                               |
|                       |                              |                               |
|                       |                              |                               |
|                       |                              |                               |
|                       |                              |                               |
|                       |                              |                               |
|                       |                              |                               |
|                       |                              |                               |
|                       |                              |                               |
|                       |                              |                               |
|                       |                              |                               |
|                       |                              |                               |
|                       |                              |                               |
|                       |                              |                               |
|                       |                              |                               |
|                       |                              |                               |
|                       |                              |                               |
|                       |                              |                               |
|                       |                              |                               |
|                       |                              |                               |
|                       |                              |                               |
|                       |                              |                               |
|                       |                              |                               |
|                       |                              |                               |
|                       |                              |                               |
|                       |                              |                               |
|                       |                              |                               |
|                       |                              |                               |
|                       |                              |                               |
|                       |                              |                               |
|                       |                              |                               |
|                       |                              |                               |
|                       |                              |                               |
|                       |                              |                               |
|                       |                              |                               |
|                       |                              |                               |
|                       |                              |                               |
|                       |                              |                               |
|                       |                              |                               |
|                       |                              |                               |
|                       |                              |                               |
|                       |                              |                               |
|                       |                              |                               |
|                       |                              |                               |
|                       |                              |                               |
|                       |                              |                               |
|                       |                              |                               |
|                       |                              |                               |
|                       |                              |                               |
|                       |                              |                               |
|                       |                              |                               |
|                       |                              |                               |
|                       |                              |                               |
|                       |                              |                               |
|                       |                              |                               |
|                       |                              |                               |
|                       |                              |                               |
|                       |                              |                               |
|                       |                              |                               |
|                       |                              |                               |
|                       |                              |                               |
|                       |                              |                               |
|                       |                              |                               |
|                       |                              |                               |
|                       |                              |                               |
|                       |                              |                               |
|                       |                              |                               |
|                       |                              |                               |
|                       |                              |                               |
|                       |                              |                               |
|                       |                              |                               |
|                       |                              |                               |
|                       |                              |                               |
|                       |                              |                               |
|                       |                              |                               |
|                       |                              |                               |
|                       |                              |                               |
|                       |                              |                               |
|                       |                              |                               |
|                       |                              |                               |
|                       |                              |                               |
|                       |                              |                               |
|                       |                              |                               |
|                       |                              |                               |
|                       |                              |                               |
|                       |                              |                               |
|                       |                              |                               |
|                       |                              |                               |
|                       |                              |                               |
|                       |                              |                               |
|                       |                              |                               |
|                       |                              |                               |
|                       |                              |                               |
|                       |                              |                               |
|                       |                              |                               |
|                       |                              |                               |
|                       |                              |                               |
|                       |                              |                               |
|                       |                              |                               |
|                       |                              |                               |
|                       |                              |                               |
|                       |                              |                               |
|                       |                              |                               |
|                       |                              |                               |
|                       |                              |                               |
|                       |                              |                               |
|                       |                              |                               |
|                       |                              |                               |
|                       |                              |                               |
|                       |                              |                               |
|                       |                              |                               |
|                       |                              |                               |
|                       |                              |                               |
|                       |                              |                               |
|                       |                              |                               |
|                       |                              |                               |
|                       |                              |                               |
|                       |                              |                               |
|                       |                              |                               |
|                       |                              |                               |
|                       |                              |                               |
|                       |                              |                               |
|                       |                              |                               |
|                       |                              |                               |
|                       |                              |                               |
|                       |                              |                               |
|                       |                              |                               |
|                       |                              |                               |
|                       |                              |                               |
|                       |                              |                               |
|                       |                              |                               |
|                       |                              |                               |
|                       |                              |                               |
|                       |                              |                               |
|                       |                              |                               |
|                       |                              |                               |
|                       |                              |                               |
|                       |                              |                               |
|                       |                              |                               |
|                       |                              |                               |
|                       |                              |                               |
|                       |                              |                               |
|                       |                              |                               |
|                       |                              |                               |
|                       |                              |                               |
|                       |                              |                               |
|                       |                              |                               |
|                       |                              |                               |
|                       |                              |                               |
|                       |                              |                               |
|                       |                              |                               |
|                       |                              |                               |
|                       |                              |                               |
|                       |                              |                               |
|                       |                              |                               |
|                       |                              |                               |
|                       |                              |                               |
|                       |                              |                               |
|                       |                              |                               |
|                       |                              |                               |
|                       |                              |                               |
|                       |                              |                               |
|                       |                              |                               |
|                       |                              |                               |
|                       |                              |                               |
|                       |                              |                               |
|                       |                              |                               |
|                       |                              |                               |
|                       |                              |                               |
|                       |                              |                               |
|                       |                              |                               |
|                       |                              |                               |
|                       |                              |                               |
|                       |                              |                               |
|                       |                              |                               |
|                       |                              |                               |
|                       |                              |                               |
|                       |                              |                               |
|                       |                              |                               |
|                       |                              |                               |
|                       |                              |                               |
|                       |                              |                               |
|                       |                              |                               |
|                       |                              |                               |
|                       |                              |                               |
|                       |                              |                               |
|                       |                              |                               |
|                       |                              |                               |
|                       |                              |                               |
|                       |                              |                               |
|                       |                              |                               |
|                       |                              |                               |
|                       |                              |                               |
|                       |                              |                               |
|                       |                              |                               |
|                       |                              |                               |
|                       |                              |                               |
|                       |                              |                               |
|                       |                              |                               |
|                       |                              |                               |
|                       |                              |                               |
|                       |                              |                               |
|                       |                              |                               |
|                       |                              |                               |
|                       |                              |                               |
|                       |                              |                               |
|                       |                              |                               |
|                       |                              |                               |
|                       |                              |                               |
|                       |                              |                               |
|                       |                              |                               |
|                       |                              |                               |
+-----------------------+------------------------------+-------------------------------+
|      #   67           | *Seq. mapped in strand Plus* | *Seq. mapped in strand Plus*  |
|                       |           BC029918           |          AK016890             |
|                       |           AK131042           |          BY716569             |
|                       |           AY461663           |          AA139105             |
|                       |           AF542390           |          AA139126             |
|                       |                              |          CF105719             |
|                       |                              |          AK006144             |
|                       |                              |          BY706250             |
|                       |                              |          AY461664             |
|                       | *Seq. mapped in strand Minus*| *Seq. mapped in strand Minus* |
|                       |           AK056676           |            W81898             |
+-----------------------+------------------------------+-------------------------------+
|      #   68           | *Seq. mapped in strand Plus* | *Seq. mapped in strand Plus*  |
|                       |           AK127284           |          AK011114             |
|                       |           BC038439           |          BY018506             |
|                       |                              |          AK042884             |
|                       |                              |          BB640108             |
|                       |                              |          BY238248             |
|                       |                              |          BY715671             |
|                       |                              |          AK016073             |
|                       |                              |          BI985607             |
|                       |                              |          BY293087             |
|                       |                              |          BU938944             |
|                       |                              |          BB572606             |
|                       |                              |          CA328451             |
|                       |                              |          BB249737             |
|                       |                              |          AA183671             |
|                       |                              |          AI643091             |
|                       |                              |          BB823587             |
|                       |                              |          AV108785             |
|                       | *Seq. mapped in strand Minus*| *Seq. mapped in strand Minus* |
|                       |           AY358949           |          BI554642             |
|                       |           BC071616           |          BI100630             |
|                       |           BC064948           |          BE648009             |
|                       |           CR623218           |          BF786957             |
|                       |           CR615959           |          CK788466             |
|                       |                              |          AI607349             |
|                       |                              |          BE953790             |
|                       |                              |          AA065945             |
|                       |                              |          BI331369             |
|                       |                              |          CA468336             |
|                       |                              |          CB949107             |
|                       |                              |          BF533815             |
|                       |                              |          CF755020             |
|                       |                              |          AI835706             |
|                       |                              |          BF455504             |
|                       |                              |          AA717610             |
|                       |                              |          BF232678             |
+-----------------------+------------------------------+-------------------------------+
|      #   69           | *Seq. mapped in strand Plus* | *Seq. mapped in strand Plus*  |
|                       |           BC041961           |          BB649073             |
|                       |           AK094650           |          AK081622             |
|                       |           AL834454           |          AK034391             |
|                       |           AB056652           |          BB623023             |
|                       |           AK094191           |          BB641342             |
|                       |                              |          AK043673             |
|                       |                              |          AK039555             |
|                       |                              |          BB190361             |
|                       |                              |          BB633558             |
|                       |                              |          BB628810             |
|                       |                              |          BY731699             |
|                       |                              |          BY238389             |
|                       |                              |          BF471454             |
|                       |                              |          BY080299             |
|                       |                              |          BY728140             |
|                       |                              |          AK081222             |
|                       | *Seq. mapped in strand Minus*| *Seq. mapped in strand Minus* |
|                       |           AK092549           |          BB641842             |
|                       |           BC041480           |          BB580431             |
+-----------------------+------------------------------+-------------------------------+
|      #   70           | *Seq. mapped in strand Plus* | *Seq. mapped in strand Plus*  |
|                       |           BI831916           |          BB654623             |
|                       |           AA913230           |          CN721671             |
|                       |           AA953067           |          BY728972             |
|                       |           BX648012           |          BY110076             |
|                       |           BG703413           |          CA492603             |
|                       |           CB994717           |          CA787256             |
|                       |           CD110177           |          CN718971             |
|                       |           BP346358           |          CK031033             |
|                       |           BP321263           |          BQ891106             |
|                       |           BP355590           |          BC021156             |
|                       |           AU098838           |          CF983367             |
|                       |           BI462269           |          AB001927             |
|                       |           BU517281           |          AI049055             |
|                       |           AL597318           |          BU609234             |
|                       |           CF994325           |          BE864690             |
|                       |           CR614415           |          CK633633             |
|                       |           CR625819           |          AA896537             |
|                       |           BI829167           |          CF732512             |
|                       |           BG717731           |          BG800988             |
|                       |           BI560656           |          BG862477             |
|                       |           BI559946           |          BG404141             |
|                       |           BG699038           |          AW045189             |
|                       |           BI551565           |          BG242657             |
|                       |           BI551510           |          AA198650             |
|                       |           BI462261           |          BB786129             |
|                       |           BU553346           |          AA591359             |
|                       |           BU188602           |                               |
|                       |           BM194138           |                               |
|                       |           BU598865           |                               |
|                       |           BI226188           |                               |
|                       |           BM194158           |                               |
|                       |           BP277053           |                               |
|                       |           BP226766           |                               |
|                       |           AL705511           |                               |
|                       |           BM152879           |                               |
|                       |           BP243948           |                               |
|                       |           BX950969           |                               |
|                       |           BP242829           |                               |
|                       |           BP351208           |                               |
|                       |           BP356042           |                               |
|                       |           BP242641           |                               |
|                       |           BP370136           |                               |
|                       |           BP363399           |                               |
|                       |           BP231413           |                               |
|                       |           BP344007           |                               |
|                       |           BP238190           |                               |
|                       |           CD641833           |                               |
|                       |           BF184813           |                               |
|                       |           AL598643           |                               |
|                       |           BG570940           |                               |
|                       |           BM787512           |                               |
|                       |           AU130099           |                               |
|                       |           AU130058           |                               |
|                       |           BG503151           |                               |
|                       |           BX647869           |                               |
|                       |           BC006997           |                               |
|                       |           AV736208           |                               |
|                       |           BP311109           |                               |
|                       |           BM819229           |                               |
|                       |           BP262572           |                               |
|                       |           AL711023           |                               |
|                       |           BM833958           |                               |
|                       |           CB144497           |                               |
|                       |           BP221618           |                               |
|                       |           BP276993           |                               |
|                       |           BP345504           |                               |
|                       |           BM835695           |                               |
|                       |           BM828017           |                               |
|                       |           BM834057           |                               |
|                       |           AU279485           |                               |
|                       |           BP258253           |                               |
|                       |           BP335146           |                               |
|                       |           BP365845           |                               |
|                       |           AU253065           |                               |
|                       |           BP268234           |                               |
|                       |           BP363209           |                               |
|                       |           BP259951           |                               |
|                       |           BP275142           |                               |
|                       |           BP354162           |                               |
|                       |           BP355375           |                               |
|                       |           BP364065           |                               |
|                       |           BP234738           |                               |
|                       |           BP311291           |                               |
|                       |           BP314461           |                               |
|                       |           BP335175           |                               |
|                       |           BP353317           |                               |
|                       |           BP356442           |                               |
|                       |           BP357592           |                               |
|                       |           BP205497           |                               |
|                       |           BP267579           |                               |
|                       |           BP284324           |                               |
|                       |           BP315531           |                               |
|                       |           BP315818           |                               |
|                       |           BP339592           |                               |
|                       |           BP357714           |                               |
|                       |           BP358216           |                               |
|                       |           BP205375           |                               |
|                       |           BP239646           |                               |
|                       |           BP342251           |                               |
|                       |           BP245721           |                               |
|                       |           BP358281           |                               |
|                       |           BP229488           |                               |
|                       |           BP202538           |                               |
|                       |           BP231194           |                               |
|                       |           BP233048           |                               |
|                       |           BP202421           |                               |
|                       |           BP341955           |                               |
|                       |           CB994602           |                               |
|                       |           AU121202           |                               |
|                       |           AU130584           |                               |
|                       |           AU133266           |                               |
|                       |           AU131829           |                               |
|                       |           AU132832           |                               |
|                       |           BQ212150           |                               |
|                       |           AU131851           |                               |
|                       |           BP380870           |                               |
|                       |           BG503474           |                               |
|                       |           CD704789           |                               |
|                       |           BG502766           |                               |
|                       |           BP249787           |                               |
|                       |           CD702718           |                               |
|                       |           BG501657           |                               |
|                       |           BG504601           |                               |
|                       |           BG501653           |                               |
|                       |           BG503567           |                               |
|                       |           BG502544           |                               |
|                       |           BG503454           |                               |
|                       |           BG503696           |                               |
|                       |           BG502408           |                               |
|                       |           BG502330           |                               |
|                       |           BE798406           |                               |
|                       |           BE798995           |                               |
|                       |           AL521824           |                               |
|                       |           AU127036           |                               |
|                       |           BM749452           |                               |
|                       |           BP221721           |                               |
|                       |           BF797342           |                               |
|                       |           BG392597           |                               |
|                       |           BI837486           |                               |
|                       |           BM803648           |                               |
|                       |             U32519           |                               |
|                       |           CN365973           |                               |
|                       |           BG256675           |                               |
|                       |           BG328113           |                               |
|                       |           AA373174           |                               |
|                       |           BG177810           |                               |
|                       |           BX414816           |                               |
|                       |           CN365974           |                               |
|                       |           BX462396           |                               |
|                       |           AL557747           |                               |
|                       |           BU192715           |                               |
|                       |           BU508123           |                               |
|                       |           BU155147           |                               |
|                       |           BM469642           |                               |
|                       |           CB265656           |                               |
|                       |           BM463594           |                               |
|                       |           AL703127           |                               |
|                       |           CD643631           |                               |
|                       |           BE974164           |                               |
|                       |           BX361821           |                               |
|                       |           BF131473           |                               |
|                       |           BG769012           |                               |
|                       |           BU170727           |                               |
|                       |           BG762868           |                               |
|                       |           BQ954390           |                               |
|                       |           BG177070           |                               |
|                       |           AL558855           |                               |
|                       |           BX114487           |                               |
|                       |           BG505610           |                               |
|                       |             F11765           |                               |
|                       |             N88282           |                               |
|                       |           CD657256           |                               |
|                       |           BU181099           |                               |
|                       |           CN365968           |                               |
|                       |           BG767230           |                               |
|                       |           BM314028           |                               |
|                       |           BE896142           |                               |
|                       |           BE613642           |                               |
|                       |           BE744160           |                               |
|                       |           AL520564           |                               |
|                       |           BM924849           |                               |
|                       |           BX426364           |                               |
|                       |           CR601908           |                               |
|                       |           BE897549           |                               |
|                       |           BM467418           |                               |
|                       |           BI771616           |                               |
|                       |           BQ049227           |                               |
|                       |           BG772759           |                               |
|                       |           BM194039           |                               |
|                       |           BF981972           |                               |
|                       |           BU182539           |                               |
|                       |           CF272301           |                               |
|                       |             T74514           |                               |
|                       |           CN365978           |                               |
|                       |           BP356548           |                               |
|                       |           CV027390           |                               |
|                       |           CR536535           |                               |
|                       |           AA190765           |                               |
|                       |           BE970956           |                               |
|                       |           BG479966           |                               |
|                       |           BE005184           |                               |
|                       |           BM828579           |                               |
|                       |           BX436401           |                               |
|                       |           BU929414           |                               |
|                       |           AA354164           |                               |
|                       |           BQ230139           |                               |
|                       |           AL515767           |                               |
|                       |           BG252042           |                               |
|                       |           BE208294           |                               |
|                       |           BE164741           |                               |
|                       |           CN365969           |                               |
|                       |           AI927513           |                               |
|                       |           BG252290           |                               |
|                       |           BQ215164           |                               |
|                       |           BP356535           |                               |
|                       |           CB130680           |                               |
|                       |           BP203279           |                               |
|                       |           BM910289           |                               |
|                       |           BG331886           |                               |
|                       |           BX405314           |                               |
|                       |           BM750507           |                               |
|                       |           CN365970           |                               |
|                       |           BP290832           |                               |
|                       |           BU156468           |                               |
|                       |           BE884247           |                               |
|                       |           AA256344           |                               |
|                       |           BP294026           |                               |
|                       |           BP293740           |                               |
|                       |           BX426363           |                               |
|                       |           CN483430           |                               |
|                       |           AL580581           |                               |
|                       |           BM797359           |                               |
|                       |           BE786599           |                               |
|                       |             F06641           |                               |
|                       |             W94797           |                               |
|                       |           AA429405           |                               |
|                       |           BX095359           |                               |
|                       |             N84107           |                               |
|                       |             Z42330           |                               |
|                       |             T32864           |                               |
|                       |           BE166477           |                               |
|                       |           BE166484           |                               |
|                       |           BE166509           |                               |
|                       |           BX414096           |                               |
|                       |           BE166498           |                               |
|                       |           BX348581           |                               |
|                       |           BG392993           |                               |
|                       |           BE711206           |                               |
|                       |           BX348580           |                               |
|                       |           BM145518           |                               |
|                       |           BG120591           |                               |
|                       |           BM542577           |                               |
|                       |           AA336513           |                               |
|                       |           BF095600           |                               |
|                       |           BF095709           |                               |
|                       |           BF095750           |                               |
|                       |           AA576563           |                               |
|                       |           AI826104           |                               |
|                       |           BG260356           |                               |
|                       |           BE386226           |                               |
|                       |           BG334056           |                               |
|                       |             N56452           |                               |
|                       |           AA191357           |                               |
|                       |           BF748718           |                               |
|                       |           CA495363           |                               |
|                       |           BE384809           |                               |
|                       |           BE385263           |                               |
|                       |           BE729625           |                               |
|                       |           BU620479           |                               |
|                       |           CF146886           |                               |
|                       |           CA310286           |                               |
|                       |           BP378434           |                               |
|                       |           BE018486           |                               |
|                       |           BE891572           |                               |
|                       |           BE503288           |                               |
|                       |           AL133845           |                               |
|                       |           BQ270000           |                               |
|                       |           BQ188483           |                               |
|                       |           AA129537           |                               |
|                       |           AW044287           |                               |
|                       |           BU195195           |                               |
|                       |           AA779316           |                               |
|                       |           AW590343           |                               |
|                       |           CN365971           |                               |
|                       |           BF115352           |                               |
|                       |           AA360761           |                               |
|                       |           BF115716           |                               |
|                       |           AL120926           |                               |
|                       |           AU117760           |                               |
|                       |           AA885672           |                               |
|                       |             H97493           |                               |
|                       |           AI658515           |                               |
|                       |           AA425151           |                               |
|                       |           AA360892           |                               |
|                       |           AA814268           |                               |
|                       |           BM794605           |                               |
|                       |           CB161094           |                               |
|                       |           BQ308677           |                               |
|                       |           BI855603           |                               |
|                       |           AA975288           |                               |
|                       |           BP368553           |                               |
|                       |           BP379497           |                               |
|                       |           BF196887           |                               |
|                       |           CN365967           |                               |
|                       |           BP340235           |                               |
|                       |           BF171458           |                               |
|                       |           BG776892           |                               |
|                       |           BM193588           |                               |
|                       |           BM971146           |                               |
|                       |           BU685594           |                               |
|                       |           BM997120           |                               |
|                       |           BU742951           |                               |
|                       |           BM971311           |                               |
|                       |           BM984224           |                               |
|                       |           BU684304           |                               |
|                       |           BQ025473           |                               |
|                       |           AW225338           |                               |
|                       |           CK825868           |                               |
|                       |           BU679849           |                               |
|                       |           BU679857           |                               |
|                       |           AI394318           |                               |
|                       |           AI805614           |                               |
|                       |           BQ286658           |                               |
|                       |           BM463785           |                               |
|                       |           AI679147           |                               |
|                       |           AI679719           |                               |
|                       |           AA603140           |                               |
|                       |           AW079949           |                               |
|                       |           CB143400           |                               |
|                       |           AI985382           |                               |
|                       |           BF341840           |                               |
|                       |           AA425738           |                               |
|                       |           AA829243           |                               |
|                       |           AV739747           |                               |
|                       |           AV741211           |                               |
|                       |           AI537017           |                               |
|                       |           AI950533           |                               |
|                       |           AL038718           |                               |
|                       |           AL038717           |                               |
|                       |           AA136500           |                               |
|                       |           AW152382           |                               |
|                       |           BM543340           |                               |
|                       |           AA432032           |                               |
|                       |           AA448480           |                               |
|                       |           BX095646           |                               |
|                       | *Seq. mapped in strand Minus*| *Seq. mapped in strand Minus* |
|                       |           BM799145           |          BX523572             |
|                       |           BM908061           |          BE332978             |
|                       |           BM451180           |                               |
|                       |           CN293193           |                               |
|                       |           AW014494           |                               |
|                       |           AI400755           |                               |
|                       |           BU679618           |                               |
|                       |           BG029270           |                               |
|                       |           CB241102           |                               |
|                       |           CA312758           |                               |
|                       |           AU137714           |                               |
|                       |           BI752534           |                               |
|                       |           BI916716           |                               |
|                       |           BP308473           |                               |
|                       |           AK128210           |                               |
|                       |           BE093978           |                               |
|                       |           BE694736           |                               |
|                       |           BE094002           |                               |
|                       |           AW868594           |                               |
|                       |           AV740560           |                               |
|                       |           BI599233           |                               |
|                       |           BE694725           |                               |
|                       |           AA502946           |                               |
|                       |           AI167691           |                               |
|                       |           AW340592           |                               |
|                       |           BI494976           |                               |
|                       |           AA973295           |                               |
|                       |           AW904692           |                               |
|                       |           AV683994           |                               |
|                       |           AV698054           |                               |
|                       |           AW953080           |                               |
|                       |           BI598370           |                               |
|                       |           CB105180           |                               |
|                       |           BE094087           |                               |
|                       |           AA761006           |                               |
|                       |           AI208273           |                               |
|                       |           AA889654           |                               |
|                       |           BU075951           |                               |
|                       |           BU952600           |                               |
|                       |           BQ286801           |                               |
|                       |           BQ276497           |                               |
|                       |           AA534750           |                               |
|                       |           BE009478           |                               |
|                       |           BE694790           |                               |
|                       |           AV686011           |                               |
|                       |           CD176775           |                               |
|                       |           AA835452           |                               |
|                       |           BM970910           |                               |
|                       |           BX092626           |                               |
|                       |           AA524415           |                               |
|                       |           AV754019           |                               |
|                       |           AI084781           |                               |
|                       |           AA890081           |                               |
|                       |           AI498154           |                               |
|                       |             W35105           |                               |
|                       |           AV689591           |                               |
|                       |           AA284853           |                               |
|                       |           AA493964           |                               |
|                       |             W32450           |                               |
|                       |           AV733302           |                               |
|                       |           BG170911           |                               |
|                       |           AV740615           |                               |
|                       |           CN280771           |                               |
|                       |           CN280769           |                               |
|                       |           BU935353           |                               |
|                       |             D80702           |                               |
|                       |             D81001           |                               |
|                       |           AI492819           |                               |
|                       |             D80829           |                               |
|                       |             N27759           |                               |
|                       |           BE694748           |                               |
|                       |             D80601           |                               |
|                       |           AL706870           |                               |
|                       |           AA719075           |                               |
|                       |           AI583993           |                               |
|                       |           AI375822           |                               |
|                       |           AI018626           |                               |
|                       |           AA347386           |                               |
|                       |           AA907001           |                               |
|                       |           AA878613           |                               |
|                       |           AA418694           |                               |
|                       |           AI803960           |                               |
|                       |           AA972377           |                               |
|                       |           AI685336           |                               |
|                       |           BU732996           |                               |
|                       |           AI198772           |                               |
|                       |           BU071944           |                               |
|                       |             W02135           |                               |
|                       |             W23672           |                               |
|                       |           BU607812           |                               |
|                       |           AW104934           |                               |
|                       |           AV689394           |                               |
|                       |             D81002           |                               |
|                       |           AV689125           |                               |
|                       |           AA600867           |                               |
|                       |           AV703250           |                               |
|                       |           AW026495           |                               |
|                       |           AA284806           |                               |
|                       |           AI022692           |                               |
|                       |           BM769150           |                               |
|                       |           AA703181           |                               |
|                       |           AI076381           |                               |
|                       |           BQ270138           |                               |
|                       |           BU190042           |                               |
|                       |           BU951164           |                               |
|                       |             D80602           |                               |
|                       |             D80703           |                               |
|                       |             D81594           |                               |
|                       |           AI276280           |                               |
|                       |           BU534088           |                               |
|                       |           BP306056           |                               |
|                       |           CD388030           |                               |
|                       |           BQ650173           |                               |
|                       |           AJ707369           |                               |
|                       |           AV763091           |                               |
|                       |           CD388236           |                               |
|                       |           AI433791           |                               |
|                       |           AV717108           |                               |
|                       |           CF123678           |                               |
|                       |           BQ276812           |                               |
|                       |           BU590154           |                               |
|                       |           BU860859           |                               |
|                       |           AV762648           |                               |
|                       |           BU533814           |                               |
|                       |           BU533850           |                               |
|                       |           BU958248           |                               |
|                       |           AA417750           |                               |
|                       |           BU590155           |                               |
|                       |           BE906727           |                               |
|                       |           AV732997           |                               |
|                       |           BU537553           |                               |
|                       |           AA315474           |                               |
|                       |           AI381827           |                               |
|                       |           BF978392           |                               |
|                       |           BG204339           |                               |
|                       |           BE047712           |                               |
|                       |           BG206469           |                               |
|                       |           BG214872           |                               |
|                       |           BF173490           |                               |
|                       |           AA418755           |                               |
|                       |           BE093996           |                               |
|                       |           AW796247           |                               |
|                       |           BG196142           |                               |
|                       |           AV722523           |                               |
|                       |           AI268339           |                               |
|                       |           BU858873           |                               |
|                       |           CF124096           |                               |
|                       |           AA304326           |                               |
|                       |             T39377           |                               |
|                       |           AI878955           |                               |
|                       |             C15535           |                               |
|                       |           CD697787           |                               |
|                       |           BM833826           |                               |
|                       |           AA379880           |                               |
|                       |           BX474049           |                               |
|                       |           AV712435           |                               |
|                       |           BU172675           |                               |
|                       |             F27122           |                               |
|                       |           CN280770           |                               |
|                       |           BT009786           |                               |
|                       |           CR456901           |                               |
|                       |           AW517447           |                               |
|                       |           AA947434           |                               |
|                       |           AA379924           |                               |
+-----------------------+------------------------------+-------------------------------+
|      #   71           | *Seq. mapped in strand Plus* | *Seq. mapped in strand Plus*  |
|                       |           AW292907           |          BE944250             |
|                       | *Seq. mapped in strand Minus*| *Seq. mapped in strand Minus* |
|                       |           AA629830           |          BE625179             |
+-----------------------+------------------------------+-------------------------------+
|      #   72           | *Seq. mapped in strand Plus* | *Seq. mapped in strand Plus*  |
|                       |           BU170658           |          BB639884             |
|                       |           BM458175           |          BB845836             |
|                       |           BF685271           |          BB855395             |
|                       |           BC008834           |          AK076365             |
|                       |           CA398218           |          BI695639             |
|                       |           BG771443           |          BG295058             |
|                       |           CF594351           |          BI664637             |
|                       |           AU279556           |          BU503193             |
|                       |           AU126069           |          BI155381             |
|                       |           BU148782           |          CA460704             |
|                       |           CF131472           |          BI157861             |
|                       |           CF132106           |          BY263365             |
|                       |           BQ109557           |          AB011550             |
|                       |           BU553024           |          BY208032             |
|                       |           BU848756           |          CF174120             |
|                       |           BQ706756           |          CA556291             |
|                       |           BM468653           |          CF914273             |
|                       |           BQ438019           |          BQ917704             |
|                       |           BI196993           |          BB856478             |
|                       |           AF029678           |          BY209829             |
|                       |           AF052205           |          BY199125             |
|                       |           AU100553           |          BY273410             |
|                       |           CV026911           |          BY294835             |
|                       |           BI193286           |          BY303721             |
|                       |           BF822413           |          BB617352             |
|                       |           BF819929           |          BY291892             |
|                       |           BF342325           |          CF915482             |
|                       |           BQ706046           |            U81490             |
|                       |           BG754158           |          AA096940             |
|                       |           BF918717           |          BG976569             |
|                       |             T08026           |          BI557794             |
|                       |           BX477785           |          BC015077             |
|                       |           BQ639084           |          BF134629             |
|                       |           BG250444           |          BI692377             |
|                       |           AU130802           |          BF022685             |
|                       |           CN258598           |          AA414787             |
|                       |           CB994466           |          AW475809             |
|                       |           AA324583           |          BF579158             |
|                       |           AA326673           |          BF534772             |
|                       |           AW964733           |          BI738110             |
|                       |           BE547724           |          BI645728             |
|                       |           BF568552           |          BG968663             |
|                       |           CD673044           |          BF582779             |
|                       |           BU173276           |          BF320588             |
|                       |           BX329083           |          BQ955565             |
|                       |           CF140746           |          BG088342             |
|                       |           BG469991           |          CN456685             |
|                       |           BM828173           |            W56975             |
|                       |           BG926169           |          AA681729             |
|                       |           BI756981           |          AA547405             |
|                       |           BG678055           |          BI218746             |
|                       |           BX418806           |          BG089502             |
|                       |             R13825           |          AA203880             |
|                       |           BF345753           |          BB713592             |
|                       |           CR740073           |          BB247097             |
|                       |           AA504321           |          BB005796             |
|                       |           BF343239           |          BE225801             |
|                       |           BF856282           |          AI415259             |
|                       |             F11687           |          AW475786             |
|                       |             F12609           |                               |
|                       |           AA504550           |                               |
|                       |             Z45101           |                               |
|                       |             T74088           |                               |
|                       |           BF855453           |                               |
|                       |           AA085184           |                               |
|                       |           BG679908           |                               |
|                       |           BF920225           |                               |
|                       |           BG769720           |                               |
|                       |           BG680166           |                               |
|                       |           BM822962           |                               |
|                       |           CN258600           |                               |
|                       |           BF895800           |                               |
|                       |           BQ923483           |                               |
|                       |           BF034016           |                               |
|                       |           BG697376           |                               |
|                       |           BG008458           |                               |
|                       |           BI522868           |                               |
|                       |           BI769537           |                               |
|                       |           BF127794           |                               |
|                       |           BM906658           |                               |
|                       |           BQ071978           |                               |
|                       |           CN258596           |                               |
|                       |           BI870112           |                               |
|                       |           BF868621           |                               |
|                       |           CA487176           |                               |
|                       |           BG003869           |                               |
|                       |           BX339233           |                               |
|                       |           BF812896           |                               |
|                       |           BI053754           |                               |
|                       |           BI759891           |                               |
|                       |           BF923556           |                               |
|                       |           BQ375862           |                               |
|                       |           BU153858           |                               |
|                       |           BQ315154           |                               |
|                       |           BX451923           |                               |
|                       |           BF038197           |                               |
|                       |           BM546315           |                               |
|                       |           BE706288           |                               |
|                       |           CN258594           |                               |
|                       |           AW365089           |                               |
|                       |           BQ268159           |                               |
|                       |           BU077812           |                               |
|                       |           CB850549           |                               |
|                       |           BU684873           |                               |
|                       |           CK818976           |                               |
|                       |           CN258597           |                               |
|                       |           BU625461           |                               |
|                       |           BQ008130           |                               |
|                       |           CA421466           |                               |
|                       |           BM126380           |                               |
|                       |           BU754112           |                               |
|                       |           BM673833           |                               |
|                       |           AW104159           |                               |
|                       |           CA426200           |                               |
|                       |           BU077604           |                               |
|                       |           BM126093           |                               |
|                       |           CK818975           |                               |
|                       |           AW134555           |                               |
|                       |           BE677304           |                               |
|                       |           BF475261           |                               |
|                       |           CA435858           |                               |
|                       |           AW131316           |                               |
|                       |           AI917410           |                               |
|                       |           BM992585           |                               |
|                       |           CA429721           |                               |
|                       |           CB528697           |                               |
|                       |           AW137168           |                               |
|                       |           BQ015502           |                               |
|                       |           AI684778           |                               |
|                       |           BM471043           |                               |
|                       |           CD369614           |                               |
|                       |           BE327250           |                               |
|                       |           AW297285           |                               |
|                       |           AW197397           |                               |
|                       |           BM975417           |                               |
|                       |           AI568834           |                               |
|                       |           AA504473           |                               |
|                       |           AI150083           |                               |
|                       |           AA489241           |                               |
|                       |             R37685           |                               |
|                       |           BG056782           |                               |
|                       |           BG236833           |                               |
|                       |           AI139870           |                               |
|                       |           CB242267           |                               |
|                       | *Seq. mapped in strand Minus*| *Seq. mapped in strand Minus* |
|                       |           AW009519           |          BY735161             |
|                       |           AI373814           |          CB590281             |
|                       |             N32014           |          AI426011             |
|                       |           BU187678           |          BE136638             |
|                       |           BF718638           |          AI413968             |
|                       |           CB158988           |          BF682970             |
|                       |           AI582796           |          AW540847             |
|                       |           AI340198           |          BU058517             |
|                       |           CA841419           |          CK128390             |
|                       |           AI220370           |          BU846928             |
|                       |           AI567726           |          CB320756             |
|                       |           AI340323           |          AA530043             |
|                       |           BQ654310           |          AI326454             |
|                       |           BI825177           |          CB202637             |
|                       |           AI087362           |          CF553724             |
|                       |           AF106943           |          CK624897             |
|                       |           CA307203           |          BU558586             |
|                       |           AI928603           |          BY707627             |
|                       |           AA446227           |          BU562295             |
|                       |           AI950144           |          AK012608             |
|                       |           AI168128           |          AV007284             |
|                       |           AI073391           |          AV015455             |
|                       |           BM451402           |          AA221564             |
|                       |           BF308876           |          AA207710             |
|                       |           BG113951           |          AV166223             |
|                       |             W04572           |          AV013501             |
|                       |           AI027132           |          BB742663             |
|                       |           AA302516           |          BY398976             |
|                       |           AI476771           |          BY409118             |
|                       |             N89798           |          AW124257             |
|                       |           AA890024           |          BY398411             |
|                       |           AI274762           |          BY406370             |
|                       |           AA725127           |          BY687802             |
|                       |           AA931729           |          BY405715             |
|                       |           AW872894           |          BB754657             |
|                       |           BU069220           |          AI839790             |
|                       |           BI821481           |          BY641985             |
|                       |           BX331728           |          AW553119             |
|                       |           CR595524           |          BE979423             |
|                       |           BU680887           |          BY447495             |
|                       |           BU536629           |          BY439277             |
|                       |           BE858757           |          BB680171             |
|                       |           BF683354           |          BQ031520             |
|                       |           BG339442           |          BY645076             |
|                       |           BI766834           |          BY456673             |
|                       |           BQ683293           |          BY477811             |
|                       |           BM547316           |          BI714924             |
|                       |           CD387518           |          BI903391             |
|                       |           AI057287           |          BC037500             |
|                       |           AA909129           |          BC021463             |
|                       |           AI197772           |          BG072935             |
|                       |           AA843316           |          BG695531             |
|                       |           AA934024           |          BQ922798             |
|                       |           BU785543           |          CF585792             |
|                       |           BE792377           |          AW547479             |
|                       |           BU158743           |          CK023018             |
|                       |           BQ683635           |          CA468621             |
|                       |           BQ052555           |          AK007580             |
|                       |           BQ676568           |          CB320976             |
|                       |           BQ679689           |          BC024422             |
|                       |           BU196660           |          BC028531             |
|                       |           CD173974           |          BI078882             |
|                       |           BX378877           |          BY703638             |
|                       |           CR590722           |          BY704440             |
|                       |           BX334868           |          AK003381             |
|                       |           CR613274           |          AK003647             |
|                       |           BE840497           |          AK004197             |
|                       |           BE840574           |          CA323205             |
|                       |           AI313425           |          BB197887             |
|                       |           AW183502           |          AV131680             |
|                       |           AA707816           |          BY701896             |
|                       |           AA708233           |          BY678415             |
|                       |           BQ434412           |          BB688385             |
|                       |           AA707865           |          BY379353             |
|                       |             N80206           |          BB772720             |
|                       |           AW327350           |          BB834113             |
|                       |           BU728232           |          BB756881             |
|                       |           BF526627           |          CK789636             |
|                       |           BE253218           |          BY703910             |
|                       |           BG332952           |          BX630071             |
|                       |           AA514452           |          BY507439             |
|                       |           AA181976           |          BY391801             |
|                       |           AA430686           |          BY510430             |
|                       |           AA971499           |          BB683507             |
|                       |           AA429256           |          CA468410             |
|                       |           AA917922           |          BY703330             |
|                       |           AA642573           |          AK002828             |
|                       |           AA743813           |          BY753676             |
|                       |           AW468667           |          BF539759             |
|                       |             N23640           |          AA692610             |
|                       |           AW629813           |          BU557698             |
|                       |           BE843315           |          CK621460             |
|                       |             N71594           |          CF424104             |
|                       |           AI244831           |          BY481027             |
|                       |           AL038996           |          AA230476             |
|                       |           AW327658           |          BB770065             |
|                       |             W93462           |          CA467973             |
|                       |           BM988425           |          AA967247             |
|                       |           AA766372           |          CK625974             |
|                       |           AI129092           |          AK007912             |
|                       |           AA703144           |          AI316685             |
|                       |           AA766039           |          CK790763             |
|                       |           AA703121           |            W83391             |
|                       |           AA703132           |          BU841969             |
|                       |           BQ027512           |          BF682427             |
|                       |             T73812           |          BF011747             |
|                       |           AA069440           |          CK793945             |
|                       |           AA708477           |          BG147883             |
|                       |           CD641400           |          BI715532             |
|                       |           AA643503           |          BE626683             |
|                       |           AI027189           |          AA562042             |
|                       |           CB852657           |          AA624388             |
|                       |           AA846105           |          BE133586             |
|                       |           AI031526           |          AW012056             |
|                       |           AI200340           |          CD768777             |
|                       |           AI299117           |          BM936166             |
|                       |           AI369725           |          AA692021             |
|                       |           AI524460           |          AA980151             |
|                       |           AI311658           |          AA218432             |
|                       |           AI342536           |          AA473770             |
|                       |           AJ572030           |          BE137699             |
|                       |           AI766601           |          AI536356             |
|                       |             N70920           |          AA990209             |
|                       |           AI762297           |          AA756020             |
|                       |           AA468809           |            W36684             |
|                       |           AA759289           |          AA638358             |
|                       |           AI096824           |          AI386290             |
|                       |           AI245618           |          AA230663             |
|                       |             N34313           |          AA260528             |
|                       |           BF109534           |            W64873             |
|                       |           AW439911           |            W30649             |
|                       |           AA843230           |          BI147613             |
|                       |           AW194108           |            W57111             |
|                       |           AW270607           |            W78592             |
|                       |           AV725793           |          AI606971             |
|                       |           AV727167           |          AI119757             |
|                       |           BF434045           |          BU560807             |
|                       |           BG250294           |          AA657066             |
|                       |           AV722417           |          CA481963             |
|                       |           AV722555           |          CF585791             |
|                       |           CA441996           |          BE335717             |
|                       |           BE674697           |          BY711753             |
|                       |           BG472676           |          BF147515             |
|                       |           BU621884           |            W78271             |
|                       |           CN481193           |          AI154940             |
|                       |           BU623333           |          CB849210             |
|                       |           AF230924           |          BF720191             |
|                       |           CA438792           |          BY053795             |
|                       |           CA748683           |          AA197830             |
|                       |           BG775156           |          AA003213             |
|                       |           BQ933147           |          AA003250             |
|                       |           BG576752           |            W53244             |
|                       |           BG751382           |          AI882459             |
|                       |           BM014285           |          BE192938             |
|                       |           BG821025           |          BY028994             |
|                       |           BC005890           |          BY033979             |
|                       |           BG104911           |          BY079967             |
|                       |           BM980380           |          BY173842             |
|                       |           BM562837           |          BY027110             |
|                       |           BM471879           |          BG085719             |
|                       |           BQ018726           |          BY050535             |
|                       |           BU176177           |          BY042395             |
|                       |           BU535396           |          BY345738             |
|                       |           BI829298           |          BY027932             |
|                       |           BI860143           |          AA049252             |
|                       |           BQ232018           |          BY092459             |
|                       |           BU956611           |          BY057392             |
|                       |           BG030194           |            W12095             |
|                       |           BE271709           |          CA490247             |
|                       |           BE743258           |            W80238             |
|                       |           BM983048           |          AA002475             |
|                       |           BG765675           |          AA509652             |
|                       |           BI758070           |          BY327987             |
|                       |           BE871860           |                               |
|                       |           BE906260           |                               |
|                       |           BP272614           |                               |
|                       |           BG255556           |                               |
|                       |           BQ230602           |                               |
|                       |           BF340837           |                               |
|                       |           BU622948           |                               |
|                       |           BG437125           |                               |
|                       |           BG249284           |                               |
|                       |           BU857567           |                               |
|                       |           AA503486           |                               |
|                       |             W70287           |                               |
|                       |           AI267936           |                               |
|                       |             D52353           |                               |
|                       |           AA887934           |                               |
|                       |           BI496932           |                               |
|                       |           AA622278           |                               |
|                       |           CN363648           |                               |
|                       |           BX396632           |                               |
|                       |           CR620346           |                               |
|                       |           BF791427           |                               |
|                       |           BM783897           |                               |
|                       |           AA009784           |                               |
|                       |           AW341580           |                               |
|                       |           BE563945           |                               |
|                       |           BU527146           |                               |
|                       |           BE746761           |                               |
|                       |           AA937820           |                               |
|                       |           BP431524           |                               |
|                       |           BM794546           |                               |
|                       |           CN363645           |                               |
|                       |           BQ052034           |                               |
|                       |           CD246982           |                               |
|                       |           AV745541           |                               |
|                       |           BE274896           |                               |
|                       |           BF025812           |                               |
|                       |           CN363644           |                               |
|                       |           BG713977           |                               |
|                       |           BF678882           |                               |
|                       |           BG747365           |                               |
|                       |           BE266473           |                               |
|                       |           BI861375           |                               |
|                       |           BE252524           |                               |
|                       |           BE253406           |                               |
|                       |           CD250243           |                               |
|                       |           BI856317           |                               |
|                       |           BE778905           |                               |
|                       |           BG260728           |                               |
|                       |           AW953950           |                               |
|                       |           BE313869           |                               |
|                       |           BG484354           |                               |
|                       |           AA446226           |                               |
|                       |           BF306376           |                               |
|                       |           BI521159           |                               |
|                       |           BI053484           |                               |
|                       |           CR606293           |                               |
|                       |           BE748439           |                               |
|                       |           AA620804           |                               |
|                       |           AA379516           |                               |
|                       |           BG823271           |                               |
|                       |             R72650           |                               |
|                       |           BE384729           |                               |
|                       |           BF341600           |                               |
|                       |           BG824890           |                               |
|                       |           AV745368           |                               |
|                       |           BX331727           |                               |
|                       |           BI216996           |                               |
|                       |           BF314550           |                               |
|                       |           BE280087           |                               |
|                       |           AL533787           |                               |
|                       |           CR597845           |                               |
|                       |             T49589           |                               |
|                       |           BX334867           |                               |
|                       |           BE386713           |                               |
|                       |           BU596336           |                               |
|                       |           BU785859           |                               |
|                       |           CN363650           |                               |
|                       |           BP214134           |                               |
|                       |           BG253121           |                               |
|                       |           BQ315325           |                               |
|                       |           CN363654           |                               |
|                       |           CD684023           |                               |
|                       |           AA807160           |                               |
|                       |           AA937386           |                               |
|                       |           BU662026           |                               |
|                       |           CA773947           |                               |
|                       |           BP209518           |                               |
|                       |           AA056366           |                               |
|                       |           CD700336           |                               |
|                       |           BU170954           |                               |
|                       |           BG743659           |                               |
|                       |           BE897286           |                               |
|                       |           BI764161           |                               |
|                       |           BG819281           |                               |
|                       |           BE748737           |                               |
|                       |           CB053567           |                               |
|                       |           AI792323           |                               |
|                       |           BG548242           |                               |
|                       |           BQ787417           |                               |
|                       |           BU789826           |                               |
|                       |           AW963827           |                               |
|                       |           BM706906           |                               |
|                       |           AV721412           |                               |
|                       |           CD239673           |                               |
|                       |           CB215075           |                               |
|                       |           BM782388           |                               |
|                       |           BE832326           |                               |
|                       |           AJ708756           |                               |
|                       |           BF982606           |                               |
|                       |             R24597           |                               |
|                       |           BG394911           |                               |
|                       |           BI860483           |                               |
|                       |           CN363646           |                               |
|                       |           AI929640           |                               |
|                       |           BM782722           |                               |
|                       |           BU603422           |                               |
|                       |             W39055           |                               |
|                       |           BE386188           |                               |
|                       |           CV026594           |                               |
|                       |           CD676219           |                               |
|                       |           CA842515           |                               |
|                       |           BU070409           |                               |
|                       |           AV752593           |                               |
|                       |           BE348323           |                               |
|                       |           AA384803           |                               |
|                       |           AI310946           |                               |
|                       |           CD688210           |                               |
|                       |           CD689750           |                               |
|                       |           BE395049           |                               |
|                       |           BU941978           |                               |
|                       |           AI095306           |                               |
|                       |           BG615267           |                               |
|                       |           BQ441560           |                               |
|                       |           BQ227341           |                               |
|                       |           BM706475           |                               |
|                       |           BM746526           |                               |
|                       |           AW957431           |                               |
|                       |           BE440147           |                               |
|                       |           BM839807           |                               |
|                       |           BI517518           |                               |
|                       |           AA187766           |                               |
|                       |           AA580173           |                               |
|                       |             R77008           |                               |
|                       |           CN363653           |                               |
|                       |           CV421209           |                               |
|                       |           AA343463           |                               |
|                       |           AL038995           |                               |
|                       |             W75983           |                               |
|                       |           AJ709777           |                               |
|                       |             H25235           |                               |
|                       |           AW583144           |                               |
|                       |           AA357305           |                               |
|                       |           BG770706           |                               |
|                       |           CN363652           |                               |
|                       |           BM856483           |                               |
|                       |             N94792           |                               |
|                       |           AA090206           |                               |
|                       |           BF337782           |                               |
|                       |           BM821665           |                               |
|                       |           AW612720           |                               |
|                       |           BM834498           |                               |
|                       |           CB138373           |                               |
|                       |           CD675208           |                               |
|                       |             R81592           |                               |
|                       |           AA364911           |                               |
|                       |             T49588           |                               |
|                       |           BF444927           |                               |
|                       |           CN363643           |                               |
|                       |           AI929571           |                               |
|                       |           CN363642           |                               |
|                       |           AW163654           |                               |
|                       |           CA393395           |                               |
|                       |           BF678695           |                               |
|                       |           CN363639           |                               |
|                       |           CB106834           |                               |
|                       |           BM010680           |                               |
|                       |           CN363655           |                               |
|                       |           CN363647           |                               |
|                       |           BE178772           |                               |
|                       |           BM887789           |                               |
|                       |           AL533788           |                               |
|                       |           BE264748           |                               |
|                       |             N48534           |                               |
|                       |           BG769271           |                               |
|                       |           BU540844           |                               |
|                       |           AV660698           |                               |
|                       |             D52710           |                               |
|                       |           CD386532           |                               |
|                       |           AA329903           |                               |
|                       |           AA377858           |                               |
|                       |             H52756           |                               |
|                       |           AA375529           |                               |
|                       |           CB111622           |                               |
|                       |             N44205           |                               |
|                       |           CD707681           |                               |
|                       |           AA009893           |                               |
|                       |           BE265853           |                               |
|                       |           AA471384           |                               |
|                       |           CB136487           |                               |
|                       |           BX282503           |                               |
|                       |           BG472577           |                               |
|                       |           AV694174           |                               |
|                       |             H49758           |                               |
|                       |           AV688946           |                               |
|                       |           AV688955           |                               |
|                       |           AV693096           |                               |
|                       |           CD695550           |                               |
|                       |             H85714           |                               |
|                       |           AV711510           |                               |
|                       |           CN363641           |                               |
|                       |           AV683424           |                               |
|                       |           AV691035           |                               |
|                       |           AV696419           |                               |
|                       |             T73900           |                               |
|                       |           AA364890           |                               |
|                       |             R64509           |                               |
|                       |             R78970           |                               |
|                       |           CN363651           |                               |
|                       |             W31492           |                               |
|                       |           CD674980           |                               |
|                       |           BF742817           |                               |
|                       |           CD694950           |                               |
|                       |           BM688600           |                               |
|                       |           BG942218           |                               |
|                       |             C03961           |                               |
|                       |             R72729           |                               |
|                       |           BF172370           |                               |
|                       |           AA069467           |                               |
|                       |             R50583           |                               |
|                       |           CD701578           |                               |
|                       |           AA143653           |                               |
+-----------------------+------------------------------+-------------------------------+
|      #   73           | *Seq. mapped in strand Plus* | *Seq. mapped in strand Plus*  |
|                       |           AJ000517           |          AK020781             |
|                       |           AF032104           |                               |
|                       |           AF032105           |                               |
|                       |           AI337452           |                               |
|                       |           AA581769           |                               |
|                       |           AI263200           |                               |
|                       |           BF507568           |                               |
|                       |           AW207737           |                               |
|                       |           BF940502           |                               |
|                       |           AW591252           |                               |
|                       |           AW172284           |                               |
|                       |           AL120878           |                               |
|                       |           CN271735           |                               |
|                       |           AF032103           |                               |
|                       |           AA099235           |                               |
|                       |           CB988415           |                               |
|                       |           BX499680           |                               |
|                       |           BP220889           |                               |
|                       |           AW516774           |                               |
|                       |           CN271734           |                               |
|                       |           AA489134           |                               |
|                       |           AI660730           |                               |
|                       |           BQ308478           |                               |
|                       |           CN271733           |                               |
|                       |           BX643945           |                               |
|                       |           AW592396           |                               |
|                       |           BP332319           |                               |
|                       |           BF993947           |                               |
|                       |           AW997332           |                               |
|                       |           AW997253           |                               |
|                       |           AW997259           |                               |
|                       |           BI559626           |                               |
|                       |           BG745250           |                               |
|                       |           BP314402           |                               |
|                       |           AL704665           |                               |
|                       |           AL704346           |                               |
|                       |           AK125125           |                               |
|                       |           BU683615           |                               |
|                       |           BM983995           |                               |
|                       |           BG390306           |                               |
|                       |           BQ004400           |                               |
|                       |           BU742846           |                               |
|                       |           CA440076           |                               |
|                       |           AL043992           |                               |
|                       |           CA433000           |                               |
|                       |           AA745252           |                               |
|                       |           AI933392           |                               |
|                       |           AI687211           |                               |
|                       |           AA765098           |                               |
|                       |           AI265801           |                               |
|                       |           AI741617           |                               |
|                       | *Seq. mapped in strand Minus*| *Seq. mapped in strand Minus* |
|                       |           CN267586           |          AA426937             |
|                       |           AI335541           |                               |
|                       |           CN413086           |                               |
+-----------------------+------------------------------+-------------------------------+
|      #   74           | *Seq. mapped in strand Plus* | *Seq. mapped in strand Plus*  |
|                       |           AK026208           |          AK079116             |
|                       |           BC017959           |          AK007831             |
|                       |           CR622948           |          BC071241             |
|                       |           CR591130           |          BC024945             |
|                       |           AL832964           |                               |
|                       | *Seq. mapped in strand Minus*| *Seq. mapped in strand Minus* |
|                       |           BX647677           |          BC058590             |
|                       |           AK095272           |          BC023868             |
|                       |           BC025758           |                               |
|                       |           BC063502           |                               |
|                       |           BX648265           |                               |
+-----------------------+------------------------------+-------------------------------+
|      #   75           | *Seq. mapped in strand Plus* | *Seq. mapped in strand Plus*  |
|                       |           AA039370           |          AK045406             |
|                       |           AI199443           |          AK035191             |
|                       |           AI349374           |          BC027158             |
|                       |           AL833289           |          BC049153             |
|                       |           AL035973           |          BQ950094             |
|                       |           BE790759           |          AK011524             |
|                       |           AA039369           |          BQ032893             |
|                       |           CN371728           |          BF461154             |
|                       |           AA253395           |          AW457217             |
|                       |           AI276560           |          BM502670             |
|                       |             W07219           |                               |
|                       |           BM799501           |                               |
|                       |           CN371731           |                               |
|                       |           AA846471           |                               |
|                       |           BG655942           |                               |
|                       |           BM314618           |                               |
|                       |           BX094657           |                               |
|                       |           CB070092           |                               |
|                       |             H97716           |                               |
|                       |           BF027760           |                               |
|                       |           CA429315           |                               |
|                       |           AK075236           |                               |
|                       |           AL133574           |                               |
|                       |           AK075176           |                               |
|                       |           AW296369           |                               |
|                       |           CR617889           |                               |
|                       |           AL117450           |                               |
|                       |           AK027342           |                               |
|                       |           CF529292           |                               |
|                       |           AK075070           |                               |
|                       |           BC026959           |                               |
|                       |             N21270           |                               |
|                       |           BX350543           |                               |
|                       |           AW085003           |                               |
|                       |           BU632651           |                               |
|                       |           CA442900           |                               |
|                       |           AW295982           |                               |
|                       |           BQ021081           |                               |
|                       |           BQ001020           |                               |
|                       |           CA420177           |                               |
|                       |           CB242670           |                               |
|                       |           BU622369           |                               |
|                       |           CA428613           |                               |
|                       |           BQ689887           |                               |
|                       |           BQ879344           |                               |
|                       |           BU191028           |                               |
|                       |           BU191403           |                               |
|                       |           BM994269           |                               |
|                       |           BQ018769           |                               |
|                       |           BQ020436           |                               |
|                       |           BQ004133           |                               |
|                       |           CA440717           |                               |
|                       |           AI076779           |                               |
|                       |           AW173124           |                               |
|                       |           AI051897           |                               |
|                       |           AW302140           |                               |
|                       |           AI073784           |                               |
|                       |           AI168123           |                               |
|                       |           AI189919           |                               |
|                       |           AI214632           |                               |
|                       |           AI290329           |                               |
|                       |           AI160547           |                               |
|                       |           BE882820           |                               |
|                       |           BF516356           |                               |
|                       | *Seq. mapped in strand Minus*| *Seq. mapped in strand Minus* |
|                       |           BG978234           |          BE987573             |
|                       |           BF732854           |          BE983113             |
|                       |           AI198606           |          BM935099             |
|                       |           CK299137           |                               |
|                       |           BQ184492           |                               |
|                       |           BX102403           |                               |
|                       |           BG494796           |                               |
|                       |           AK001432           |                               |
|                       |           CR594735           |                               |
|                       |           AI935292           |                               |
|                       |           AL542716           |                               |
|                       |           BU156813           |                               |
|                       |           BQ929741           |                               |
|                       |           BG478581           |                               |
|                       |           BE274958           |                               |
|                       |           AU128274           |                               |
|                       |           CB152436           |                               |
|                       |           BF210979           |                               |
|                       |           CB158587           |                               |
|                       |           CB142568           |                               |
|                       |           BX119708           |                               |
|                       |           AA416847           |                               |
|                       |           BE897918           |                               |
|                       |           BG210659           |                               |
|                       |           AW016664           |                               |
|                       |           BU680284           |                               |
|                       |           BQ020263           |                               |
+-----------------------+------------------------------+-------------------------------+
|      #   76           | *Seq. mapped in strand Plus* | *Seq. mapped in strand Plus*  |
|                       |           BC069624           |          CN461044             |
|                       |           BC069628           |          BB616025             |
|                       |           BC069636           |          BX526746             |
|                       |           BC069645           |          AI451186             |
|                       |           BC067524           |          BB625968             |
|                       |           BC067525           |                               |
|                       |           BC069997           |                               |
|                       |           AF410457           |                               |
|                       |           AK126044           |                               |
|                       |           AK094958           |                               |
|                       |           AL137431           |                               |
|                       |           AK022143           |                               |
|                       |           CR749675           |                               |
|                       | *Seq. mapped in strand Minus*| *Seq. mapped in strand Minus* |
|                       |           AK096991           |          BM940465             |
|                       |                              |          BQ175022             |
|                       |                              |          CO042240             |
|                       |                              |          CA450890             |
|                       |                              |            D63520             |
+-----------------------+------------------------------+-------------------------------+
|      #   77           | *Seq. mapped in strand Plus* | *Seq. mapped in strand Plus*  |
|                       |           AI796937           |          BE985538             |
|                       |                              |          BI525885             |
|                       |                              |          BF021848             |
|                       |                              |          BF319428             |
|                       | *Seq. mapped in strand Minus*| *Seq. mapped in strand Minus* |
|                       |           AW292973           |          BQ175854             |
|                       |           AF275805           |          BC019148             |
|                       |           AA844125           |          AK003979             |
|                       |           BF983587           |          CN539557             |
|                       |           AI524764           |          CN719793             |
|                       |           BM710480           |          CF173918             |
|                       |           BU734268           |          CN721570             |
|                       |             R37401           |          BI149334             |
|                       |           BM153342           |          CA320845             |
|                       |           BQ646447           |          CB247826             |
|                       |           CA416487           |          CF531564             |
|                       |           AK025609           |          BI412650             |
|                       |           AB018302           |          BQ770918             |
|                       |           AL137268           |          CN693021             |
|                       |           BC028714           |          CN668568             |
|                       |           BM665305           |          BI148796             |
|                       |           BM144828           |          BY005123             |
|                       |           AA906848           |          BB840422             |
|                       |           AA908989           |          BY005085             |
|                       |           AW183947           |          BB872693             |
|                       |           AI024703           |          BY194341             |
|                       |           AL529284           |                               |
|                       |           BX479762           |                               |
|                       |           BP305671           |                               |
|                       |           BF815876           |                               |
|                       |           BF739278           |                               |
|                       |           BF739276           |                               |
|                       |           CN301091           |                               |
|                       |           CD644175           |                               |
|                       |           BM551691           |                               |
|                       |           BM549750           |                               |
|                       |           BQ056913           |                               |
|                       |           BF819270           |                               |
|                       |           BM018581           |                               |
|                       |           BX425676           |                               |
|                       |           BM459921           |                               |
|                       |           BM477041           |                               |
|                       |           BI826356           |                               |
|                       |           AA325626           |                               |
|                       |           BG721981           |                               |
|                       |           CB960800           |                               |
|                       |           CF994975           |                               |
|                       |           CB990627           |                               |
|                       |           BG722383           |                               |
|                       |           BI460837           |                               |
|                       |           CN301090           |                               |
|                       |           BP368805           |                               |
|                       |           CN301089           |                               |
+-----------------------+------------------------------+-------------------------------+
|      #   78           | *Seq. mapped in strand Plus* | *Seq. mapped in strand Plus*  |
|                       |           BQ576121           |          AW492748             |
|                       |           AL036429           |          CA944221             |
|                       |           AI741122           |                               |
|                       |           CB113048           |                               |
|                       |           CK300833           |                               |
|                       |           BU686605           |                               |
|                       |           BF509483           |                               |
|                       |           BF514660           |                               |
|                       |           AA360563           |                               |
|                       |           BX117231           |                               |
|                       | *Seq. mapped in strand Minus*| *Seq. mapped in strand Minus* |
|                       |           AK024294           |          AW475774             |
|                       |           AI753829           |          BX519759             |
|                       |           BU729248           |          AK016687             |
|                       |           BU728612           |          AF326229             |
|                       |           BF206389           |          CB337309             |
|                       |           CK821710           |          AW321841             |
|                       |           CA431053           |          AI850546             |
|                       |           BU742582           |          AK013341             |
|                       |           BU683354           |          AK010884             |
|                       |           AA878345           |          AK013910             |
|                       |             C05956           |          AI036322             |
|                       |           AK025753           |          BM894896             |
|                       |           AA764981           |          BC011255             |
|                       |           AA035500           |          AI747701             |
|                       |           BE872182           |          BE687695             |
|                       |           BQ028113           |          AK031538             |
|                       |           BM768635           |          AK088181             |
|                       |             W44987           |          AI843249             |
|                       |           AI755274           |          AW060712             |
|                       |           AW613653           |          AW488922             |
|                       |           AI338086           |          AV299026             |
|                       |           AA605212           |          BI557502             |
|                       |           AI282494           |          CB588130             |
|                       |           AA923534           |          CF538007             |
|                       |           AI476307           |          CD772624             |
|                       |           AA564792           |          BI988133             |
|                       |           AI620686           |          CF908448             |
|                       |           AI446129           |          CK639447             |
|                       |           AI192136           |          BQ567784             |
|                       |           BU753102           |          AW322921             |
|                       |           AW054968           |          CN716541             |
|                       |           AI075645           |          CA566524             |
|                       |           AI697278           |          BI696087             |
|                       |           BM984958           |          CF749585             |
|                       |           BE675471           |          AA879709             |
|                       |           BQ006093           |          CF579982             |
|                       |           BE670609           |          BI319679             |
|                       |           BM976862           |          AI326255             |
|                       |           BU741683           |          BX631458             |
|                       |           AI201565           |          BG801097             |
|                       |           AI890075           |          BI790551             |
|                       |           AK075562           |          BB817457             |
|                       |           AA232291           |          BY145176             |
|                       |           AF156965           |          CN531315             |
|                       |           AI418340           |          AF395811             |
|                       |           BQ433444           |          BI790841             |
|                       |           AA233456           |          BF138742             |
|                       |           AW662058           |          BG921843             |
|                       |           BM678575           |          BI696021             |
|                       |           BU683342           |          CF749314             |
|                       |           BG942163           |          CN694649             |
|                       |           BI494218           |          CF169712             |
|                       |           CR599599           |          CF171180             |
|                       |           CA445701           |          AW909729             |
|                       |           BU170136           |          BY710290             |
|                       |           BF516118           |          BU513934             |
|                       |           BE041630           |          BE533618             |
|                       |           BE550212           |          CF170835             |
|                       |           AI656977           |          BQ899409             |
|                       |           CR610481           |          BU613899             |
|                       |           BE878148           |          AA389369             |
|                       |           CD693498           |          AI322614             |
|                       |           BP240800           |          BM569934             |
|                       |           BF057570           |          BI112226             |
|                       |           BE868255           |          AA036265             |
|                       |           BM751296           |          CF748851             |
|                       |           BP243034           |          BI558127             |
|                       |           BM478479           |          BI246335             |
|                       |           BE463871           |          BI666212             |
|                       |           AW771493           |          BQ886936             |
|                       |           BE646149           |          CB182037             |
|                       |           AI654344           |          CN529890             |
|                       |           AI089587           |          CF892887             |
|                       |           AI129444           |          CF750329             |
|                       |           BE302164           |          BI082424             |
|                       |           AA935181           |          BI078273             |
|                       |           BQ018355           |          BI453232             |
|                       |           AI688616           |          BI559273             |
|                       |           BM984781           |          BG976363             |
|                       |           CN407506           |          BF536638             |
|                       |           BQ575956           |          CB575975             |
|                       |           BP203900           |          AA260438             |
|                       |           BQ014297           |          BQ942493             |
|                       |           BQ014005           |          BI661944             |
|                       |           BU165953           |          BG917247             |
|                       |           BE018574           |          CK787793             |
|                       |           BE502007           |          BF164895             |
|                       |           AI962535           |          BF161067             |
|                       |           AI738746           |          CN538376             |
|                       |             N36017           |          CK787903             |
|                       |           AL039176           |          BE570600             |
|                       |           BC007710           |          CA568705             |
|                       |           BU686113           |          CN703312             |
|                       |           BE169472           |          AI115057             |
|                       |           BP240392           |          BY166780             |
|                       |           CN407494           |          BE309381             |
|                       |           BE709227           |          BF168686             |
|                       |           AI016620           |          BF532334             |
|                       |           BM672111           |          BI159412             |
|                       |           BF509942           |          CB589515             |
|                       |           BM700647           |          BY745150             |
|                       |           AA993117           |          BY173060             |
|                       |           CB113094           |          BY173503             |
|                       |           AA713807           |          CK342579             |
|                       |           AA807751           |          BG862576             |
|                       |           AI952451           |          BY734736             |
|                       |           BF694794           |          AI317599             |
|                       |           BQ276566           |          BG870881             |
|                       |           AL550910           |          BF321692             |
|                       |           AI204189           |          CA569586             |
|                       |           AI382074           |          BB652265             |
|                       |           BE076988           |          CA564901             |
|                       |           BM461637           |          CA564911             |
|                       |             W47394           |          CA528866             |
|                       |           BU929754           |          CN671431             |
|                       |           AA737175           |          BF140861             |
|                       |           CB529097           |          CN699326             |
|                       |           BU689315           |          CN663655             |
|                       |           CR591675           |          CN665487             |
|                       |           BF002233           |          CF896540             |
|                       |           BM970727           |          BI441142             |
|                       |           BE927726           |          BF140850             |
|                       |           BF216055           |          BI965716             |
|                       |           AL527054           |          BI455732             |
|                       |           AW852106           |          AI037295             |
|                       |           CD652531           |          CF168440             |
|                       |           AW999151           |          BG144695             |
|                       |           BP240128           |          CN697433             |
|                       |           AL551750           |          BF122813             |
|                       |           BP236183           |          BG800035             |
|                       |             Z12830           |          BI109940             |
|                       |           AA357470           |          BF135547             |
|                       |           AL527645           |          AW741782             |
|                       |           BQ307819           |          BB621048             |
|                       |           BM720948           |          CN664903             |
|                       |           BP262185           |          AW320518             |
|                       |           BQ216448           |          CA569476             |
|                       |           BP299800           |          CF165733             |
|                       |           BM802075           |          CA535506             |
|                       |           BQ278835           |          CA568217             |
|                       |           AA312214           |          AI787631             |
|                       |           BG434202           |          AU079007             |
|                       |           BQ309459           |          BF022327             |
|                       |           CB136884           |          AI317078             |
|                       |           BU429753           |          CA565825             |
|                       |           AA449004           |          BQ561640             |
|                       |           CR456961           |          CD540190             |
|                       |           BT007387           |          BB611317             |
|                       |           BQ720457           |          CN664859             |
|                       |           AW470938           |          BB856924             |
|                       |           BF002316           |          CN675260             |
|                       |           BU957535           |          CN692660             |
|                       |           BG194407           |          BB619494             |
|                       |           BP242440           |          BE309510             |
|                       |           BU663966           |          AA270017             |
|                       |           BQ431898           |          CF167331             |
|                       |           BU159188           |          AA755605             |
|                       |           BU178789           |          BE308303             |
|                       |           BU538850           |          BE629587             |
|                       |           CN407496           |          CF167248             |
|                       |           BF058294           |          CD553747             |
|                       |           BP204244           |          CA891275             |
|                       |           BI549845           |          BE629506             |
|                       |           CN407491           |          BB851861             |
|                       |           BF246728           |          AA109700             |
|                       |           AV658420           |          BG793199             |
|                       |           CN407507           |          CF904754             |
|                       |           BU156413           |          BB862425             |
|                       |           BG496172           |          AA471861             |
|                       |           BQ309455           |          AI787668             |
|                       |           BF756608           |          BY061383             |
|                       |           AL543679           |          BY062111             |
|                       |           CN407499           |          BE333123             |
|                       |           BG698143           |          BY154288             |
|                       |           BG564419           |          BB859687             |
|                       |           AL522337           |          BY155635             |
|                       |           BG496494           |          BY007013             |
|                       |           AV658414           |          BB862626             |
|                       |           BG281602           |          BB861480             |
|                       |           AV710820           |          BY227684             |
|                       |           BG612629           |          AA793936             |
|                       |           BQ227103           |          BB861770             |
|                       |           CB159275           |          CA540486             |
|                       |           BP242231           |          BY167685             |
|                       |           AA281421           |          BY079009             |
|                       |           BI551663           |          BY300048             |
|                       |           AW374041           |          BY109260             |
|                       |           AW374053           |          BY302939             |
|                       |           BF541100           |          BY769732             |
|                       |             N28568           |          BY303795             |
|                       |           BX505173           |          BY055036             |
|                       |           AV718196           |          BY091786             |
|                       |           BX106909           |          BY316230             |
|                       |           CF135870           |          BY027877             |
|                       |           CN407500           |          BY133087             |
|                       |           BG532416           |          BY311764             |
|                       |           BX485741           |          BY044416             |
|                       |           BF204663           |          BY766995             |
|                       |           CB992234           |          BY043644             |
|                       |           BQ277402           |          BY163389             |
|                       |           BF128602           |          BY037773             |
|                       |           BU933699           |          BY042754             |
|                       |           BG429229           |          BY034408             |
|                       |           BI553058           |          BY043843             |
|                       |           CN407495           |          BY161774             |
|                       |           BG535934           |          BM570108             |
|                       |           BX496742           |          BY149414             |
|                       |           BG568439           |          BY782222             |
|                       |           BG506591           |          BY054356             |
|                       |           CN407493           |          BY775886             |
|                       |             W52700           |          BY308151             |
|                       |           CN407489           |          BY150302             |
|                       |           BU950129           |          BY146918             |
|                       |           CN407505           |          BY049228             |
|                       |           AL553541           |          BY009910             |
|                       |           CB107576           |          BY309000             |
|                       |           BX472396           |          BY096486             |
|                       |           CN482431           |          BY016166             |
|                       |           CN484214           |          BY148289             |
|                       |           BG531465           |          CN684912             |
|                       |           BP381572           |          BY301353             |
|                       |           BP239212           |          BY148590             |
|                       |           CB995203           |          BY121102             |
|                       |           BP237688           |          CN698366             |
|                       |           CN407498           |                               |
|                       |           BE745768           |                               |
|                       |           BI562039           |                               |
|                       |           CB989248           |                               |
|                       |           BE958536           |                               |
|                       |           CD695844           |                               |
|                       |           BX496731           |                               |
|                       |           BP238743           |                               |
|                       |           CF596570           |                               |
|                       |           AA130160           |                               |
|                       |           AL708255           |                               |
|                       |           BF212270           |                               |
|                       |           BM827414           |                               |
|                       |           BP243842           |                               |
|                       |           CB996398           |                               |
|                       |           BG399805           |                               |
|                       |           CD695859           |                               |
|                       |           BE960890           |                               |
|                       |           BG612094           |                               |
|                       |           AL599969           |                               |
|                       |           BX504283           |                               |
|                       |           BP238731           |                               |
|                       |           BP238028           |                               |
|                       |           BP239489           |                               |
|                       |           BP314942           |                               |
|                       |           BP360542           |                               |
|                       |           BP195687           |                               |
|                       |           BP243515           |                               |
|                       |           BP241989           |                               |
|                       |           BP242799           |                               |
|                       |           AA099515           |                               |
|                       |           BG547432           |                               |
|                       |           CD700297           |                               |
|                       |           CD689854           |                               |
|                       |           CD701005           |                               |
|                       |           BP241726           |                               |
|                       |           BP240553           |                               |
|                       |           CD686847           |                               |
|                       |           BE005863           |                               |
|                       |           BP243114           |                               |
|                       |           BX497368           |                               |
|                       |           AA989029           |                               |
|                       |           AL710668           |                               |
|                       |           AL708111           |                               |
|                       |             W47424           |                               |
|                       |           AL597238           |                               |
|                       |             W26155           |                               |
|                       |           BP351968           |                               |
|                       |           CD686581           |                               |
|                       |           CB994092           |                               |
|                       |           CB143157           |                               |
|                       |           AA313551           |                               |
|                       |           AA297530           |                               |
|                       |           AA187502           |                               |
|                       |           BX950976           |                               |
|                       |           CD704177           |                               |
|                       |           CB143412           |                               |
|                       |           CN407492           |                               |
|                       |           BX486231           |                               |
|                       |           CD698085           |                               |
|                       |           BX486820           |                               |
|                       |           BX952417           |                               |
|                       |             N45965           |                               |
|                       |           BX490259           |                               |
|                       |             H69981           |                               |
|                       |           AL599831           |                               |
|                       |           AL702457           |                               |
|                       |           AL596985           |                               |
|                       |           AA311731           |                               |
|                       |           AA297455           |                               |
|                       |           BF697013           |                               |
|                       |           AL712950           |                               |
|                       |           BU429122           |                               |
|                       |           BX477053           |                               |
|                       |           AL708632           |                               |
|                       |           CD685638           |                               |
|                       |           AA157616           |                               |
|                       |           AV690352           |                               |
|                       |           BX488414           |                               |
|                       |           BX478881           |                               |
|                       |           AV716378           |                               |
|                       |           BX478748           |                               |
|                       |           CN407502           |                               |
|                       |           BX479358           |                               |
|                       |           AL710646           |                               |
|                       |           BX473865           |                               |
|                       |           AA304983           |                               |
|                       |           BU429123           |                               |
|                       |           BU428451           |                               |
|                       |           AL708600           |                               |
|                       |           AA233496           |                               |
|                       |           BX498277           |                               |
|                       |           BF170042           |                               |
|                       |           AW663246           |                               |
|                       |           BE247455           |                               |
|                       |           AA297333           |                               |
|                       |           AU098822           |                               |
|                       |             T97417           |                               |
|                       |           AA298678           |                               |
|                       |           CD689769           |                               |
|                       |           CD698229           |                               |
|                       |           CD710013           |                               |
|                       |           CA424805           |                               |
|                       |           AI360664           |                               |
|                       |           AI810853           |                               |
|                       |           CA311548           |                               |
|                       |           CK818393           |                               |
|                       |           BM685131           |                               |
|                       |           CA422797           |                               |
|                       |           BU633566           |                               |
|                       |           BM677577           |                               |
|                       |           AJ420492           |                               |
|                       |             H07925           |                               |
|                       |           AI797317           |                               |
|                       |           AI311916           |                               |
|                       |           AW296065           |                               |
|                       |           BQ017170           |                               |
|                       |           AK026687           |                               |
|                       |           AI041588           |                               |
|                       |           AI290315           |                               |
+-----------------------+------------------------------+-------------------------------+
|      #   79           | *Seq. mapped in strand Plus* | *Seq. mapped in strand Plus*  |
|                       |           AW952364           |          AI843136             |
|                       |             T31193           |                               |
|                       |           BF510619           |                               |
|                       |           BX496658           |                               |
|                       | *Seq. mapped in strand Minus*| *Seq. mapped in strand Minus* |
|                       |           AI685455           |          AI837300             |
|                       |             T09010           |          BI134281             |
|                       |           AK130029           |          AI835298             |
|                       |           AF304051           |          AI845126             |
|                       |           BE645169           |          AI118417             |
|                       |             T59417           |          BQ568263             |
|                       |           BE883778           |          AI850009             |
|                       |             N90531           |          AI841948             |
|                       |           BF055692           |          BE946603             |
|                       |           BQ027699           |          AI174021             |
|                       |           BF055809           |          BE991481             |
|                       |             F24866           |          BE992186             |
|                       |           BM975949           |          BE992250             |
|                       |           BU688102           |          AI837140             |
|                       |           BM977812           |          AI173434             |
|                       |           BU902460           |          AI255739             |
|                       |           AI193426           |          AI838116             |
|                       |           BC013209           |          AI839977             |
|                       |           BX647091           |          CA318585             |
|                       |           CR749252           |          CD774201             |
|                       |           BX647748           |          CD775113             |
|                       |           AK024521           |          CD774248             |
|                       |           AK090657           |          CD742469             |
|                       |           AK057843           |          CD775613             |
|                       |           AK057847           |          CD802518             |
|                       |           AF159092           |          AB033921             |
|                       |           AB033074           |          AK004051             |
|                       |           BC010458           |          AI845292             |
|                       |           BC011240           |          CB205284             |
|                       |           BX485853           |          AK003364             |
|                       |           AL136574           |          BC012963             |
|                       |           BF054869           |          BG797663             |
|                       |           AV738520           |          AK076514             |
|                       |           AI802620           |          CF580187             |
|                       |           BX423300           |          CD741789             |
|                       |           CR598346           |          CD297889             |
|                       |           CR612684           |          BY703621             |
|                       |           CR596981           |          BF578375             |
|                       |           CR601715           |          BI146562             |
|                       |           CR603144           |          CB947632             |
|                       |           CR611368           |          CB948701             |
|                       |           CR597040           |          BF584319             |
|                       |           CR607901           |          CD352777             |
|                       |           BX248031           |          BI220270             |
|                       |           CR624613           |          CB948078             |
|                       |           BX423197           |          CB949832             |
|                       |           BX247987           |          AI529140             |
|                       |           BX450413           |          CB593906             |
|                       |           CR620953           |          CB950989             |
|                       |           AW291437           |          BI220487             |
|                       |           BI764528           |          BE652290             |
|                       |           CF453638           |          CB952188             |
|                       |           BF526188           |          AA000155             |
|                       |           BI907667           |          BY267957             |
|                       |           CD245743           |          CB946918             |
|                       |           BX367766           |          BQ109195             |
|                       |           BG697121           |          BI217286             |
|                       |           CR596015           |            W82113             |
|                       |           BI768310           |          AW988057             |
|                       |           BF528195           |          BG084093             |
|                       |           BX388555           |          AI006167             |
|                       |           CF453605           |          BQ896309             |
|                       |           BX367783           |          AI527115             |
|                       |           BG697993           |          CB318143             |
|                       |           CF455489           |          AA386803             |
|                       |           BX368181           |          AA386811             |
|                       |           BX408350           |          AI099173             |
|                       |           BX429354           |          AI131856             |
|                       |           BX450407           |          BI145804             |
|                       |           BX408834           |          BQ445134             |
|                       |           BF347782           |          BF449541             |
|                       |           BX429315           |          CA890944             |
|                       |           CF455412           |          AA245246             |
|                       |           BM920007           |          CA786748             |
|                       |           CR609913           |          CK619973             |
|                       |           BP264855           |            W16173             |
|                       |           BX429887           |          BI332294             |
|                       |           BX450539           |          AU080691             |
|                       |           BQ421897           |          CN539169             |
|                       |           BX368058           |          BG866779             |
|                       |           BX450835           |          CB950406             |
|                       |           BX450836           |          BQ879939             |
|                       |           BP317564           |          CA786882             |
|                       |           BX450414           |          AA575354             |
|                       |           BX326070           |          AI180966             |
|                       |           BQ883648           |          AA245244             |
|                       |           BQ889296           |          AA245245             |
|                       |           BX423056           |          BF585007             |
|                       |           BI599063           |          BY714290             |
|                       |           BP342464           |          BI146258             |
|                       |           BP264987           |          CB590487             |
|                       |           CB135199           |          BI083574             |
|                       |           CB135593           |          BQ715789             |
|                       |             R52608           |          AU080984             |
|                       |           BX408186           |          AU080741             |
|                       |           BX407973           |          BF015422             |
|                       |           CD627672           |          BI657489             |
|                       |             N80357           |          BI332469             |
|                       |           BF526255           |          BI147144             |
|                       |           BF345109           |          BF533588             |
|                       |           CB146607           |          BI328300             |
|                       |           CB134852           |          BI217255             |
|                       |           BX346922           |          BI219598             |
|                       |             H48869           |          BM949922             |
|                       |           BP347455           |          BG971301             |
|                       |           BX367983           |          AI529399             |
|                       |           BF967523           |          CA751629             |
|                       |           BQ327325           |          BI331549             |
|                       |           BX422589           |          BF384235             |
|                       |           BG676881           |          BI146992             |
|                       |           BP348017           |          BI148823             |
|                       |           CB133219           |          BI329479             |
|                       |           CB121413           |          AU080884             |
|                       |           BM766206           |          CA458164             |
|                       |             W27282           |          CA459043             |
|                       |           BX429118           |          CA495094             |
|                       |           CD722591           |          AU079627             |
|                       |           BX284008           |          AU079517             |
|                       |           CB135757           |          CA460106             |
|                       |           CB139087           |          BU560884             |
|                       |             R84386           |          CA459541             |
|                       |           CB121436           |          CN539335             |
|                       |           CB130129           |          CB598038             |
|                       |           CB134914           |          BF533568             |
|                       |           CB135748           |          BI082932             |
|                       |           CB146684           |          BI217160             |
|                       |           CB133193           |          BF782095             |
|                       |           CB135754           |          BF237310             |
|                       |           CB148857           |          BM951498             |
|                       |           CB135779           |          BI145624             |
|                       |           CB144218           |          BI246337             |
|                       |           CB146644           |          BF236465             |
|                       |           CB138205           |          BU057240             |
|                       |           CB135201           |          BM949513             |
|                       |           CB138527           |          BY739384             |
|                       |           CB146615           |          BY738082             |
|                       |             R93674           |          CB593702             |
|                       |             T35800           |          BF234667             |
|                       |           CF455110           |          CD807720             |
|                       |           CF455146           |          BI331087             |
|                       |             T08543           |          CK625071             |
|                       |           CB138264           |          CA460112             |
|                       |           CB146938           |          AU079771             |
|                       |           CB148818           |          AI227118             |
|                       |           BX450881           |          BF300791             |
|                       |           CB146695           |          CK626478             |
|                       |             Z24921           |          BI218834             |
|                       |             R11417           |          BF464999             |
|                       |           BM820419           |          AI182025             |
|                       |           BX429613           |          BF235717             |
|                       |           CF142434           |          BY727223             |
|                       |           BG896619           |          CF580186             |
|                       |           CB137778           |          CA457896             |
|                       |           CB137685           |          CB593784             |
|                       |           CB146971           |          BU847615             |
|                       |           BU146482           |          AA288909             |
|                       |           CB147307           |          BF234020             |
|                       |           BF337285           |          BI221740             |
|                       |           BX463563           |          CA459280             |
|                       |           BP338459           |          CB845130             |
|                       |           BG914296           |          AI226895             |
|                       |           BQ331975           |          BG793132             |
|                       |           CK023798           |          CK623485             |
|                       |           BI816786           |          BY238971             |
|                       |           BI771628           |          CB593672             |
|                       |             R18563           |          CA459303             |
|                       |           BX423198           |          CA480942             |
|                       |           BX354120           |          AU035709             |
|                       |           CF552383           |          AA930950             |
|                       |           BX376848           |          CA458574             |
|                       |           AA025827           |          BF234888             |
|                       |           CB138499           |          CA892399             |
|                       |           BF342500           |          AW475398             |
|                       |             T79124           |          CK618894             |
|                       |           BX423301           |            W82391             |
|                       |           BQ338885           |          BE652228             |
|                       |             R35374           |          AA840183             |
|                       |           BF090316           |          AI315745             |
|                       |             H61679           |          AI315435             |
|                       |           BF347262           |          AI788004             |
|                       |             R95716           |          AA138509             |
|                       |           CD627669           |          CA885227             |
|                       |           CD627670           |          CA887438             |
|                       |           BP309009           |          BG797739             |
|                       |           AW895728           |          CA887015             |
|                       |           BE698989           |          BY244691             |
|                       |           CB138255           |          BY258166             |
|                       |           AW895991           |          BY257693             |
|                       |           CF139453           |          AU051224             |
|                       |           AW895865           |          BY242833             |
|                       |           BU957368           |          BY242920             |
|                       |           CB992924           |          AI785145             |
|                       |           BP333517           |          CA947914             |
|                       |           CK004088           |          BY255229             |
|                       |             W20020           |          AA511731             |
|                       |           CF142432           |          CA461120             |
|                       |           BF922502           |          BY245080             |
|                       |           AI272253           |          BY247516             |
|                       |           BP333737           |          BY245042             |
|                       |           BX460944           |          BB862889             |
|                       |           BM923957           |          BY242680             |
|                       |           BX418699           |          AI315793             |
|                       |           BQ722634           |          BY256525             |
|                       |           BF930964           |          BY255752             |
|                       |           BF950272           |          BY242425             |
|                       |           BM745809           |          BY238969             |
|                       |           BQ636638           |          BY262909             |
|                       |           CB154812           |          BY241685             |
|                       |           BI517902           |          BY252898             |
|                       |           BP239338           |          BY245256             |
|                       |           BQ898777           |          BY241862             |
|                       |             Z41957           |          BY249844             |
|                       |           BP333966           |          BY251944             |
|                       |             T09222           |          BY259741             |
|                       |           BP333728           |          BY256888             |
|                       |           BP209902           |          BY258043             |
|                       |           BP382581           |          BY249300             |
|                       |           BM753173           |          BY239834             |
|                       |           BM764462           |          BY282306             |
|                       |           BM772108           |          BY255156             |
|                       |           BX376977           |          BY246646             |
|                       |           BM810623           |          BY262202             |
|                       |           BX428905           |          BY238366             |
|                       |           BX441606           |          AI527014             |
|                       |           BQ881927           |          AI876022             |
|                       |           BQ878052           |          BY287863             |
|                       |             T33821           |          BY258933             |
|                       |           BX408753           |          BY245642             |
|                       |           BF920220           |          BY252135             |
|                       |             W72768           |          BY262732             |
|                       |           BI912756           |          BY289208             |
|                       |           BX450289           |          BY249732             |
|                       |           BI012911           |          BY286711             |
|                       |           BX444404           |          BY289381             |
|                       |           BX449856           |          CA888630             |
|                       |           BM742827           |          BY288963             |
|                       |           BX428207           |          BY268439             |
|                       |           BI458116           |          AI286944             |
|                       |           BU145803           |          BY257565             |
|                       |           BU147095           |          BY276244             |
|                       |           BI771051           |          BY259953             |
|                       |           CD515320           |          BY290641             |
|                       |           BU149695           |          BY247810             |
|                       |           BI764557           |          BY295341             |
|                       |           BI548131           |          BY240938             |
|                       |           BX428854           |          BY241180             |
|                       |           BI762386           |          BY241848             |
|                       |           BQ888484           |          BY249871             |
|                       |           BP346949           |          BY285060             |
|                       |           BM753107           |          BY257929             |
|                       |           BP346929           |          AW412937             |
|                       |           BM743836           |          BY267982             |
|                       |           BP214657           |          BY285267             |
|                       |           BP362049           |          BF385740             |
|                       |           BP362089           |          BY250463             |
|                       |           BX441749           |          BY285772             |
|                       |           BP362257           |          BY272451             |
|                       |           BI551688           |          BY268301             |
|                       |           BP258926           |          BY249721             |
|                       |           BF839088           |          BY241306             |
|                       |           BI548507           |          BY251059             |
|                       |           BE764120           |          BY285683             |
|                       |             R88676           |          BY294292             |
|                       |           CB139115           |          BY280379             |
|                       |           CF141086           |          BY094349             |
|                       |           BM741291           |          BY239422             |
|                       |           BI545888           |          BY295933             |
|                       |           BI049099           |          BY094774             |
|                       |           BE714967           |          BY279143             |
|                       |           BE715030           |          BY273707             |
|                       |           BU145610           |          BY250659             |
|                       |           BQ889771           |          BY283141             |
|                       |           BF932212           |          BY289968             |
|                       |           BX440238           |          BY254690             |
|                       |           BP283275           |          BY276693             |
|                       |           CB153061           |          BY287292             |
|                       |             D56053           |          BY288419             |
|                       |           BI517492           |          BY266599             |
|                       |           CB137686           |          BY285188             |
|                       |           BG819252           |          BY263953             |
|                       |           BF087210           |          BY249820             |
|                       |           BP334368           |          BY787637             |
|                       |           BF969554           |          BY249807             |
|                       |           BE707327           |          BY294798             |
|                       |           BI552277           |          BY250355             |
|                       |           BI836765           |          BY130462             |
|                       |           CB130094           |          BY241292             |
|                       |           CB135602           |          BY071822             |
|                       |             T64238           |          BY297298             |
|                       |           AI498532           |          BY293911             |
|                       |           BM753061           |          BY253715             |
|                       |           CB139060           |          BY288953             |
|                       |           CA389642           |          BY238979             |
|                       |           BI762986           |          BY273979             |
|                       |           AL546540           |          BY291937             |
|                       |           BI759006           |          BY079616             |
|                       |           BG706712           |          BY271208             |
|                       |           BI550571           |          BY256838             |
|                       |           BQ893123           |          BY298122             |
|                       |           BG701465           |          BY123473             |
|                       |           BX463346           |          BY784990             |
|                       |             Z42395           |          BY238753             |
|                       |           BI862200           |          BY259984             |
|                       |           BX461883           |          AA754738             |
|                       |           BF530969           |          BY079282             |
|                       |           CB154071           |          BY264823             |
|                       |           BG702614           |          BY089732             |
|                       |           BI041885           |          BY121189             |
|                       |           BI036566           |          BY130943             |
|                       |           BI036943           |          BY074330             |
|                       |           BI036568           |          BY091241             |
|                       |           BI041374           |          BY122226             |
|                       |           BI039912           |          BY343166             |
|                       |           BI036542           |          BY111850             |
|                       |           BF923741           |          BY237488             |
|                       |           BQ345023           |          BY784901             |
|                       |           AI201607           |          BY085007             |
|                       |           BI761672           |          BY125153             |
|                       |           BI910299           |          BY343260             |
|                       |           BI911514           |          BY182218             |
|                       |           BI548830           |          AI227248             |
|                       |           BI544930           |          BY340734             |
|                       |           BG818977           |          BY117668             |
|                       |             R49896           |          BY288825             |
|                       |           BF935247           |          BY250701             |
|                       |           BF924860           |          BY338782             |
|                       |           BF927277           |          BF322181             |
|                       |           BG715871           |          BY266838             |
|                       |           CB152330           |          BY281409             |
|                       |           CB153512           |          BY771440             |
|                       |           BI038722           |          BY344937             |
|                       |           BF793156           |          BY103900             |
|                       |           BI035980           |          BY125358             |
|                       |           BP209456           |          BY091508             |
|                       |           BF923742           |          AA666533             |
|                       |           CV025284           |          BY137782             |
|                       |           BM753217           |          BY149014             |
|                       |             R84717           |          BY085825             |
|                       |           CB137732           |          BY148583             |
|                       |           BF346349           |          BB848819             |
|                       |             M62142           |          BY103832             |
|                       |           BI603625           |          BY076136             |
|                       |           BM764461           |          BY081617             |
|                       |           BF923745           |          BY345861             |
|                       |           BG707283           |          BY147642             |
|                       |           BM753839           |          BY122845             |
|                       |           BM753545           |          BY131195             |
|                       |           BF529454           |          BY197054             |
|                       |           BQ331523           |          BY134011             |
|                       |           CV023465           |          BY237916             |
|                       |           BG819908           |          BY272609             |
|                       |           CB265573           |          BX630770             |
|                       |           AI380537           |          BY149370             |
|                       |           BP333523           |          BY247599             |
|                       |           AA706731           |                               |
|                       |           CB134372           |                               |
|                       |           CB270498           |                               |
|                       |           CB152673           |                               |
|                       |           CB153091           |                               |
|                       |           CB152001           |                               |
|                       |           CB153626           |                               |
|                       |           CB139135           |                               |
|                       |           BQ109509           |                               |
|                       |           BI544873           |                               |
|                       |           BI521171           |                               |
|                       |           BI667158           |                               |
|                       |           BP309464           |                               |
|                       |           BP216453           |                               |
|                       |           BP208017           |                               |
|                       |           BG911592           |                               |
|                       |           BG742135           |                               |
|                       |           BG820149           |                               |
|                       |           BG699259           |                               |
|                       |           BP348549           |                               |
|                       |           BP193711           |                               |
|                       |           BP227456           |                               |
|                       |           BP333923           |                               |
|                       |           BF967527           |                               |
|                       |           BP330119           |                               |
|                       |           BP349205           |                               |
|                       |           BP378385           |                               |
|                       |           BP208364           |                               |
|                       |           BP218305           |                               |
|                       |           CB152075           |                               |
|                       |           BI549398           |                               |
|                       |           BI045466           |                               |
|                       |           BG913706           |                               |
|                       |           BF922544           |                               |
|                       |           CB154039           |                               |
|                       |           BE762881           |                               |
|                       |           BU598545           |                               |
|                       |           BE762858           |                               |
|                       |           BI551528           |                               |
|                       |             H50177           |                               |
|                       |           BP218456           |                               |
|                       |             W74452           |                               |
|                       |           BP257243           |                               |
|                       |           BF575097           |                               |
|                       |           BG703902           |                               |
|                       |           BP214098           |                               |
|                       |           BU930040           |                               |
|                       |           BI833252           |                               |
|                       |           BP193389           |                               |
|                       |           BP201408           |                               |
|                       |           BP309004           |                               |
|                       |           CB153265           |                               |
|                       |           BF926854           |                               |
|                       |           BP333097           |                               |
|                       |           BP257312           |                               |
|                       |           BP352663           |                               |
|                       |           CB153559           |                               |
|                       |           BP347006           |                               |
|                       |           BP307330           |                               |
|                       |           BI670119           |                               |
|                       |           BP310157           |                               |
|                       |           CB152021           |                               |
|                       |           BP320189           |                               |
|                       |           BP250022           |                               |
|                       |           BP196525           |                               |
|                       |           BP352630           |                               |
|                       |           AL709085           |                               |
|                       |           BI554116           |                               |
|                       |           CB153013           |                               |
|                       |           CD707740           |                               |
|                       |           CB153955           |                               |
|                       |           BP209828           |                               |
|                       |           CB117994           |                               |
|                       |           BP328742           |                               |
|                       |           CB128421           |                               |
|                       |           CB152315           |                               |
|                       |           CB154820           |                               |
|                       |           CB156530           |                               |
|                       |           CB153684           |                               |
|                       |           CB152663           |                               |
|                       |           BP308824           |                               |
|                       |           CB153553           |                               |
|                       |           CB153129           |                               |
|                       |           CB151992           |                               |
|                       |           CB153966           |                               |
|                       |           AI358939           |                               |
|                       |           BP196681           |                               |
|                       |           BP208679           |                               |
|                       |           BP311356           |                               |
|                       |           BP214163           |                               |
|                       |           AA019649           |                               |
|                       |           BF529160           |                               |
|                       |           BP197661           |                               |
|                       |           BP210748           |                               |
|                       |           BP381337           |                               |
|                       |           BP348318           |                               |
|                       |           BP211653           |                               |
|                       |           CB153122           |                               |
|                       |           BP331414           |                               |
|                       |           BP275089           |                               |
|                       |           BP332991           |                               |
|                       |           AA017448           |                               |
|                       |           BP376074           |                               |
|                       |           BP216678           |                               |
|                       |           BP332915           |                               |
|                       |           BP194152           |                               |
|                       |           BP194205           |                               |
|                       |           BP211121           |                               |
|                       |           BP342735           |                               |
|                       |           AI878845           |                               |
|                       |           BP196338           |                               |
|                       |           AI422201           |                               |
|                       |           BP315937           |                               |
|                       |           AI422239           |                               |
|                       |           BP208000           |                               |
|                       |           BP216013           |                               |
|                       |           BX488433           |                               |
|                       |           BP333268           |                               |
|                       |           AI879304           |                               |
|                       |           CB153283           |                               |
|                       |           CB153431           |                               |
|                       |             H40692           |                               |
|                       |           CB155800           |                               |
|                       |           BP193967           |                               |
|                       |           BP347120           |                               |
|                       |           CB153189           |                               |
|                       |           BE825860           |                               |
|                       |           CD627671           |                               |
|                       |           CN310534           |                               |
|                       |           BP192773           |                               |
|                       |           BP210551           |                               |
|                       |           BP259055           |                               |
|                       |           BP334288           |                               |
|                       |           BX488499           |                               |
|                       |           BP334789           |                               |
|                       |           BP197473           |                               |
|                       |           BP213383           |                               |
|                       |           CB144301           |                               |
|                       |           CB152762           |                               |
|                       |           CB153950           |                               |
|                       |           BP332820           |                               |
|                       |           BP333359           |                               |
|                       |           BP213377           |                               |
|                       |           BP333365           |                               |
|                       |           BI669467           |                               |
|                       |           BX487318           |                               |
|                       |           AI880155           |                               |
|                       |           BP341004           |                               |
|                       |           BP193633           |                               |
|                       |           AA025741           |                               |
|                       |           AI923364           |                               |
|                       |           BP378889           |                               |
|                       |           BP350307           |                               |
|                       |           BX471958           |                               |
|                       |           BQ694640           |                               |
|                       |           AW163815           |                               |
|                       |           BP333678           |                               |
|                       |           BP333522           |                               |
|                       |           BP333193           |                               |
|                       |           BP334341           |                               |
|                       |             H49926           |                               |
|                       |           BX508340           |                               |
|                       |           AW451024           |                               |
|                       |           BP334813           |                               |
|                       |             H30752           |                               |
|                       |             T31153           |                               |
|                       |           CB156483           |                               |
|                       |           BF345888           |                               |
|                       |           AA724499           |                               |
|                       |           BE937930           |                               |
|                       |           BP202598           |                               |
|                       |           CB155079           |                               |
|                       |             R87477           |                               |
|                       |             H85846           |                               |
|                       |           BP211459           |                               |
|                       |           CB117933           |                               |
|                       |           AA973196           |                               |
|                       |           BX488313           |                               |
|                       |           AI422192           |                               |
|                       |           AL599026           |                               |
|                       |           BP258361           |                               |
|                       |           CD514346           |                               |
|                       |           BP333808           |                               |
|                       |           CB148193           |                               |
|                       |           BP212895           |                               |
|                       |           AA909921           |                               |
|                       |             H51601           |                               |
|                       |           BP333426           |                               |
|                       |           BX487947           |                               |
|                       |           AA917485           |                               |
|                       |           AI016675           |                               |
|                       |           AA917691           |                               |
|                       |             R85145           |                               |
|                       |           AI865955           |                               |
|                       |           AI419113           |                               |
|                       |             M78975           |                               |
|                       |           BX407710           |                               |
|                       |             Z20535           |                               |
|                       |           BI550962           |                               |
|                       |           AA013239           |                               |
|                       |           BX478111           |                               |
|                       |           BX418967           |                               |
|                       |           CA393018           |                               |
|                       |           AW351521           |                               |
|                       |           BE826989           |                               |
|                       |           BE826801           |                               |
|                       |             Z28942           |                               |
+-----------------------+------------------------------+-------------------------------+
|      #   80           | *Seq. mapped in strand Plus* | *Seq. mapped in strand Plus*  |
|                       |           CB306692           |          AI835074             |
|                       |                              |          BE448611             |
|                       |                              |          BE985005             |
|                       | *Seq. mapped in strand Minus*| *Seq. mapped in strand Minus* |
|                       |           AA044336           |          BQ566089             |
|                       |           BU686109           |          AI661898             |
|                       |           AA832321           |          BE851773             |
|                       |           AL048074           |          BI465852             |
|                       |           AI241045           |          AW491589             |
|                       |           BM128471           |          BQ031369             |
|                       |           AA458832           |          CK792716             |
|                       |           AI702929           |          AK077410             |
|                       |           AI624199           |          BF461577             |
|                       |           AA165192           |          BF465013             |
|                       |           BM668998           |          BE950951             |
|                       |           AI913112           |          BF465060             |
|                       |           BU684719           |          AW824234             |
|                       |           AI823494           |          BC015081             |
|                       |           AW204115           |          CA321086             |
|                       |           BU683216           |          BU511642             |
|                       |           CA448942           |          BM937312             |
|                       |           BI468527           |          CB203296             |
|                       |           BU686101           |          AW209336             |
|                       |           BM666363           |          BQ938831             |
|                       |           BM996251           |            U82324             |
|                       |           BU631913           |          BM940560             |
|                       |           BU673955           |          BY414059             |
|                       |           BU628554           |          BY397574             |
|                       |           BU753364           |          BY405154             |
|                       |           CA415150           |          BY406587             |
|                       |           CA442893           |          BY403836             |
|                       |           BM999918           |          BY395222             |
|                       |           BM980980           |          BY758681             |
|                       |           AI770056           |          BY758640             |
|                       |           AW016753           |          CK622437             |
|                       |           AI479242           |          AW911252             |
|                       |           AK127868           |          AY267188             |
|                       |           BC022491           |          BI964420             |
|                       |           BC022344           |          AW910485             |
|                       |             U02680           |          CF579703             |
|                       |           BC068548           |          CF578599             |
|                       |           BC043148           |          CF949025             |
|                       |           BU680354           |          BI466126             |
|                       |           BU949103           |          CB570929             |
|                       |           CB243329           |          AA673335             |
|                       |           BQ129268           |          BF658256             |
|                       |           BM069562           |          BG863286             |
|                       |           BQ961512           |          CN530855             |
|                       |           BX483713           |          AW044719             |
|                       |           BG287596           |          BY741218             |
|                       |           BP206431           |          BI554676             |
|                       |           AU138579           |          BY738332             |
|                       |           BU165710           |          CK622032             |
|                       |           BQ948796           |          BY738374             |
|                       |           BP289508           |          BB618387             |
|                       |           BP206538           |          BI964630             |
|                       |           BX497235           |          BI964666             |
|                       |             W30889           |          BI965883             |
|                       |           BM829406           |          AI666631             |
|                       |           BG167824           |          BX525882             |
|                       |           BP264373           |          BY080366             |
|                       |           BI089952           |          BY007401             |
|                       |           CD615824           |          BY158437             |
|                       |           CD615825           |          BY273868             |
|                       |           CD615826           |          BY047604             |
|                       |           CD615827           |          BY321725             |
|                       |           AL036939           |          BY068348             |
|                       |           BX480056           |          BY048437             |
|                       |           AW951801           |          BY131314             |
|                       |           BG163606           |          BY010386             |
|                       |           AU137871           |          BY055714             |
|                       |           AA165260           |          AA178331             |
|                       |             C18301           |                               |
|                       |             W52729           |                               |
|                       |           AL036933           |                               |
|                       |           AW362714           |                               |
|                       |           CR541736           |                               |
|                       |           CR541761           |                               |
|                       |           AW362717           |                               |
|                       |           AW362730           |                               |
|                       |           BI856085           |                               |
|                       |           BQ954616           |                               |
|                       |           BG104675           |                               |
|                       |           AA307991           |                               |
|                       |           BI868516           |                               |
|                       |           AU137513           |                               |
|                       |           AU130002           |                               |
|                       |           BI091213           |                               |
|                       |           AW362657           |                               |
|                       |           AW815718           |                               |
|                       |             R28543           |                               |
|                       |           BU601556           |                               |
|                       |           CN413472           |                               |
|                       |           BE895158           |                               |
|                       |           BI257346           |                               |
|                       |           BG702178           |                               |
|                       |           AU131819           |                               |
|                       |           BP223529           |                               |
|                       |           BQ319058           |                               |
|                       |           CB990367           |                               |
|                       |           BI459100           |                               |
|                       |           AA505193           |                               |
|                       |             N29014           |                               |
|                       |             W68189           |                               |
|                       |             R54149           |                               |
|                       |           AW589804           |                               |
|                       |           AL048603           |                               |
|                       |           BI668004           |                               |
|                       |           CD701640           |                               |
|                       |           BF993161           |                               |
|                       |           BI545517           |                               |
|                       |           BI560457           |                               |
|                       |           AA514388           |                               |
|                       |           AI290846           |                               |
|                       |             N31768           |                               |
|                       |           BP355581           |                               |
|                       |           BQ189031           |                               |
|                       |           AI279167           |                               |
|                       |           BM787901           |                               |
|                       |           BI468528           |                               |
|                       |           BP236919           |                               |
|                       |           AA884234           |                               |
|                       |           AU280521           |                               |
|                       |           AI377969           |                               |
|                       |           BE245149           |                               |
|                       |           AI368020           |                               |
|                       |           BQ326783           |                               |
|                       |           AW104569           |                               |
|                       |           AI915802           |                               |
|                       |           CB988108           |                               |
|                       |             N24838           |                               |
|                       |           AA936071           |                               |
|                       |           AI094215           |                               |
|                       |           AA946621           |                               |
|                       |             N20642           |                               |
|                       |           AA434438           |                               |
|                       |           CN413476           |                               |
|                       |           AW005949           |                               |
|                       |           AA936069           |                               |
|                       |           AI032265           |                               |
|                       |           AI752022           |                               |
|                       |           BF438965           |                               |
|                       |           BF590151           |                               |
|                       |           AI185344           |                               |
|                       |           AA804910           |                               |
|                       |           BE244831           |                               |
|                       |           AA465698           |                               |
|                       |           AA019459           |                               |
|                       |           BF109100           |                               |
|                       |           BP199367           |                               |
|                       |             R28684           |                               |
|                       |           AA313962           |                               |
|                       |           AA904023           |                               |
|                       |             H30570           |                               |
|                       |           AU099707           |                               |
|                       |           BX103998           |                               |
+-----------------------+------------------------------+-------------------------------+
|      #   81           | *Seq. mapped in strand Plus* | *Seq. mapped in strand Plus*  |
|                       |           AB046802           |          BY752858             |
|                       |           AL834429           |          BG088148             |
|                       |           AK098609           |          BY189540             |
|                       |           BC045631           |          BY199443             |
|                       |           AK056421           |          BY212437             |
|                       |           AK092762           |          BY216473             |
|                       |           AK000022           |          BY175511             |
|                       |           AY099358           |          BY181244             |
|                       |           AY099359           |          BY176795             |
|                       |           BC028076           |          BY202015             |
|                       |           AK090478           |          BY217883             |
|                       |           AY236500           |          BY223308             |
|                       |           AY057380           |          BY190247             |
|                       |           AK128327           |          BY181561             |
|                       |           AK125672           |          BY214744             |
|                       |           AL834508           |          BY186986             |
|                       |                              |          BY178219             |
|                       |                              |          BY216371             |
|                       |                              |          BY212136             |
|                       |                              |          BY750767             |
|                       |                              |          BB636210             |
|                       |                              |          BY213475             |
|                       |                              |          BY732538             |
|                       |                              |          BY212683             |
|                       |                              |          BB849643             |
|                       |                              |          BY751621             |
|                       |                              |          AW824833             |
|                       |                              |          AY236501             |
|                       |                              |          BY732701             |
|                       |                              |          AY263160             |
|                       |                              |          BY720447             |
|                       |                              |          AA285518             |
|                       |                              |          BI411058             |
|                       |                              |          BY213366             |
|                       |                              |          AA137911             |
|                       |                              |          BY592871             |
|                       | *Seq. mapped in strand Minus*| *Seq. mapped in strand Minus* |
|                       |           AK074065           |          AW822373             |
|                       |           BC023597           |          AW763742             |
|                       |           BC035648           |          BM507848             |
|                       |           AB002405           |          AW763968             |
|                       |           BC018346           |          BY582088             |
|                       |           AY099356           |          BY576261             |
|                       |           AY099357           |          BB740130             |
|                       |           CR596410           |          BY578772             |
|                       |           AY236496           |          BY579993             |
|                       |           AY057379           |          BB732765             |
|                       |           BC007766           |          BF020680             |
|                       |           AK096076           |          BY541524             |
|                       |           AK021738           |          BM118624             |
|                       |                              |          BG066295             |
|                       |                              |          BY368137             |
|                       |                              |          BY561168             |
|                       |                              |          BB825157             |
|                       |                              |          BB731025             |
|                       |                              |          BI648299             |
|                       |                              |          BU757288             |
|                       |                              |          BY473600             |
|                       |                              |          BY481044             |
|                       |                              |          BY479902             |
|                       |                              |          BI645962             |
|                       |                              |          BY467700             |
|                       |                              |          BC013502             |
|                       |                              |          BC006956             |
|                       |                              |          BC058195             |
|                       |                              |          AK090179             |
|                       |                              |          BB814367             |
|                       |                              |          BB674996             |
|                       |                              |          AY236497             |
|                       |                              |          BB798133             |
|                       |                              |          BB800851             |
|                       |                              |          BB797805             |
|                       |                              |          AU042073             |
+-----------------------+------------------------------+-------------------------------+
|      #   82           | *Seq. mapped in strand Plus* | *Seq. mapped in strand Plus*  |
|                       |           BI762714           |          BQ175529             |
|                       |           CD697341           |          AK016219             |
|                       |           BU860965           |                               |
|                       |           CD619408           |                               |
|                       |           CN286202           |                               |
|                       |           BE889761           |                               |
|                       |           BI093897           |                               |
|                       |           BU194961           |                               |
|                       |           BM545350           |                               |
|                       |           BI762636           |                               |
|                       |           BI909259           |                               |
|                       |           BQ216640           |                               |
|                       |           BI917206           |                               |
|                       |           BG477568           |                               |
|                       |           BM721629           |                               |
|                       |           BC001364           |                               |
|                       |           AL602308           |                               |
|                       |           BE891299           |                               |
|                       |           AA306105           |                               |
|                       |           BE888831           |                               |
|                       |           BQ428101           |                               |
|                       |           CN286203           |                               |
|                       |           BF724863           |                               |
|                       |           BE897974           |                               |
|                       |           BE275338           |                               |
|                       |           BE728614           |                               |
|                       |           BE276993           |                               |
|                       |           BU161394           |                               |
|                       |           BM152505           |                               |
|                       |           BE541580           |                               |
|                       |           CN286204           |                               |
|                       |           BM727837           |                               |
|                       |           BE891858           |                               |
|                       |           AV739328           |                               |
|                       |           BI910309           |                               |
|                       |           BM922769           |                               |
|                       |           BG389910           |                               |
|                       |           AF047442           |                               |
|                       |           BI771489           |                               |
|                       |           BI754339           |                               |
|                       |           BG257912           |                               |
|                       |           BM807249           |                               |
|                       |           BE891507           |                               |
|                       |           BX282692           |                               |
|                       |           BQ433298           |                               |
|                       |           CN286201           |                               |
|                       |           AV755676           |                               |
|                       |           CV028291           |                               |
|                       |           AA304969           |                               |
|                       |           AW961186           |                               |
|                       |           AW360937           |                               |
|                       |           AW361006           |                               |
|                       |           BX334197           |                               |
|                       |           AW360911           |                               |
|                       |           BX399360           |                               |
|                       |           BX399788           |                               |
|                       |           BX363510           |                               |
|                       |           CR624978           |                               |
|                       |           BX339506           |                               |
|                       |           BU945567           |                               |
|                       |           BX384476           |                               |
|                       |           AW993268           |                               |
|                       |           BP264657           |                               |
|                       |           AW993471           |                               |
|                       |           AW993481           |                               |
|                       |           AW993283           |                               |
|                       |           BF816488           |                               |
|                       |           AW993201           |                               |
|                       |           AW992919           |                               |
|                       |           AW993467           |                               |
|                       |           AW992921           |                               |
|                       |           AW992861           |                               |
|                       |           BP260918           |                               |
|                       |           BP198899           |                               |
|                       |           AW993220           |                               |
|                       |           AW992980           |                               |
|                       |           BP350772           |                               |
|                       |           BF229136           |                               |
|                       |           AW579514           |                               |
|                       |           BP332191           |                               |
|                       | *Seq. mapped in strand Minus*| *Seq. mapped in strand Minus* |
|                       |           AI003990           |          BE988556             |
|                       |           AA890010           |                               |
|                       |           AA455917           |                               |
|                       |           CN479119           |                               |
|                       |           BM978624           |                               |
|                       |           AA694237           |                               |
+-----------------------+------------------------------+-------------------------------+
|      #   83           | *Seq. mapped in strand Plus* | *Seq. mapped in strand Plus*  |
|                       |           BP281542           |          AK037164             |
|                       |           BP198356           |          BC055393             |
|                       |           BP364852           |          AK037432             |
|                       |           BP350131           |                               |
|                       |           BX485839           |                               |
|                       |           CB155734           |                               |
|                       |           BU590420           |                               |
|                       |           BU930854           |                               |
|                       |           BP363169           |                               |
|                       |           BG502563           |                               |
|                       |           BF701401           |                               |
|                       |           BF207666           |                               |
|                       |           BF665125           |                               |
|                       |           BF694268           |                               |
|                       |           BG493060           |                               |
|                       |           BF212911           |                               |
|                       |           BF691767           |                               |
|                       |           CD691844           |                               |
|                       |           BP194389           |                               |
|                       |           BP236280           |                               |
|                       |           BP341315           |                               |
|                       |           BP311768           |                               |
|                       |           BF696329           |                               |
|                       |           BP357460           |                               |
|                       |           BM455573           |                               |
|                       |           BF576542           |                               |
|                       |           BG566276           |                               |
|                       |           BG542322           |                               |
|                       |           CB107479           |                               |
|                       |           CD177215           |                               |
|                       |           BQ638249           |                               |
|                       |           CB141867           |                               |
|                       |           BP319122           |                               |
|                       |           BP248556           |                               |
|                       |           BP310513           |                               |
|                       |           BP349628           |                               |
|                       |           CB142856           |                               |
|                       |           BF790424           |                               |
|                       |           BP350872           |                               |
|                       |           BU603012           |                               |
|                       |           BF248123           |                               |
|                       |           BE865648           |                               |
|                       |           BP354843           |                               |
|                       |           BF382645           |                               |
|                       |           AB018284           |                               |
|                       |           BF131818           |                               |
|                       |           AL598188           |                               |
|                       |           BP315857           |                               |
|                       |           BP248062           |                               |
|                       |           BE889077           |                               |
|                       |           BP349935           |                               |
|                       |           AJ006776           |                               |
|                       |             F24780           |                               |
|                       |           CD697718           |                               |
|                       |           BF664163           |                               |
|                       |           BP429381           |                               |
|                       |           BU596654           |                               |
|                       |           BP245672           |                               |
|                       |           BX503402           |                               |
|                       |           BP319951           |                               |
|                       |           CN364545           |                               |
|                       |           BU535368           |                               |
|                       |           BF107238           |                               |
|                       |           AI392718           |                               |
|                       |           AW614267           |                               |
|                       |           BG257316           |                               |
|                       |           CN364539           |                               |
|                       |           BG031914           |                               |
|                       |           AW237263           |                               |
|                       |           AW237324           |                               |
|                       |           AF078035           |                               |
|                       |           BU589678           |                               |
|                       |           BG108980           |                               |
|                       |           CN364542           |                               |
|                       |           BI033191           |                               |
|                       |           AW470353           |                               |
|                       |           BC032639           |                               |
|                       |           BF063922           |                               |
|                       |             F21081           |                               |
|                       |           AI190460           |                               |
|                       |           BG776340           |                               |
|                       |           BF195451           |                               |
|                       |           BM456168           |                               |
|                       |           BF002080           |                               |
|                       |           BF062466           |                               |
|                       |           CN364541           |                               |
|                       |           AI651406           |                               |
|                       |           AW470948           |                               |
|                       |           AI796874           |                               |
|                       |           BU945128           |                               |
|                       |           BF977196           |                               |
|                       |           BG531105           |                               |
|                       |           BG777759           |                               |
|                       |           BF575388           |                               |
|                       |           BC022260           |                               |
|                       |           CD516162           |                               |
|                       |           AW271064           |                               |
|                       |           AI345003           |                               |
|                       |           AW303266           |                               |
|                       |           BE564535           |                               |
|                       |           AW887308           |                               |
|                       |           AW887309           |                               |
|                       |           AW887336           |                               |
|                       |           BC006970           |                               |
|                       |           BG942445           |                               |
|                       |           BF210207           |                               |
|                       |           BF718777           |                               |
|                       |           BG942361           |                               |
|                       |           BG940538           |                               |
|                       |           BG940156           |                               |
|                       |           BF130177           |                               |
|                       |           CV030818           |                               |
|                       |           AW052166           |                               |
|                       |           BU677330           |                               |
|                       |           BE002169           |                               |
|                       |           AW799611           |                               |
|                       |           AW276111           |                               |
|                       |           BE843675           |                               |
|                       |           BF875311           |                               |
|                       |           BG940157           |                               |
|                       |           AW235788           |                               |
|                       |           CN364534           |                               |
|                       |           AW376295           |                               |
|                       |           BG031641           |                               |
|                       |           AI522228           |                               |
|                       |           BF844519           |                               |
|                       |           AA988620           |                               |
|                       |           CA314850           |                               |
|                       |           AL712965           |                               |
|                       |           BQ435703           |                               |
|                       |           BU676157           |                               |
|                       |           BF965606           |                               |
|                       |           AI202718           |                               |
|                       |           CA312294           |                               |
|                       |           AI699584           |                               |
|                       |           CN364543           |                               |
|                       |           CB115119           |                               |
|                       |           BG427916           |                               |
|                       |           BF700567           |                               |
|                       |           AL133563           |                               |
|                       |           BG940728           |                               |
|                       |           BG941416           |                               |
|                       |           BF002966           |                               |
|                       |           AL561396           |                               |
|                       |           BX386803           |                               |
|                       |           CR604527           |                               |
|                       |           BG940449           |                               |
|                       |           CB250657           |                               |
|                       |           BG261322           |                               |
|                       |           BE646124           |                               |
|                       |           BU076872           |                               |
|                       |           BE070152           |                               |
|                       |           AW608367           |                               |
|                       |           AI254188           |                               |
|                       |           AI252724           |                               |
|                       |           AW271037           |                               |
|                       |           AW271133           |                               |
|                       |           AI054081           |                               |
|                       |           AI053431           |                               |
|                       |           AI241985           |                               |
|                       |           AI252496           |                               |
|                       |           AI241981           |                               |
|                       |           AI254073           |                               |
|                       |           BG940969           |                               |
|                       |           BU678614           |                               |
|                       |           AI053970           |                               |
|                       |           AI254164           |                               |
|                       |           AL037741           |                               |
|                       |           AA903064           |                               |
|                       |           BG940539           |                               |
|                       |           BG940253           |                               |
|                       |           BG941697           |                               |
|                       |           BG941740           |                               |
|                       |           BG942628           |                               |
|                       |           BG943079           |                               |
|                       |           BG943738           |                               |
|                       |           BG943842           |                               |
|                       |           BG942087           |                               |
|                       |           BG943718           |                               |
|                       |           BG941420           |                               |
|                       |           BG941736           |                               |
|                       |           BG942363           |                               |
|                       |           BG944487           |                               |
|                       |           BG940968           |                               |
|                       |           BG944926           |                               |
|                       |           BG941637           |                               |
|                       |           AI053572           |                               |
|                       |           BG940276           |                               |
|                       |           BM681630           |                               |
|                       |           BE673432           |                               |
|                       |           AI053883           |                               |
|                       |           AV738982           |                               |
|                       |           AA470514           |                               |
|                       |           CA427312           |                               |
|                       |           AA165212           |                               |
|                       |           AI252375           |                               |
|                       |           BE158313           |                               |
|                       |           AI053929           |                               |
|                       |           AI246090           |                               |
|                       |           BE158316           |                               |
|                       |           BQ330338           |                               |
|                       |           BQ338650           |                               |
|                       |           BG940159           |                               |
|                       |           AA312346           |                               |
|                       |           CD697228           |                               |
|                       |           BE164649           |                               |
|                       |           BI860749           |                               |
|                       |           BQ378573           |                               |
|                       |           CB151274           |                               |
|                       |           AU127252           |                               |
|                       |           AK001386           |                               |
|                       |           BP363043           |                               |
|                       |           BU186301           |                               |
|                       |           BG941919           |                               |
|                       |           BQ317512           |                               |
|                       |           BF798060           |                               |
|                       |           BQ316395           |                               |
|                       |           BG875964           |                               |
|                       |           AW502722           |                               |
|                       |           BM043504           |                               |
|                       |           BQ316123           |                               |
|                       |           BQ316042           |                               |
|                       |           BQ316292           |                               |
|                       |           BQ315974           |                               |
|                       |           BQ315977           |                               |
|                       |           BQ315978           |                               |
|                       |           BQ316620           |                               |
|                       |           BQ317511           |                               |
|                       |           AA192479           |                               |
|                       |           AU131545           |                               |
|                       |           BU177286           |                               |
|                       |           CD557907           |                               |
|                       |           BQ317504           |                               |
|                       |           CK000880           |                               |
|                       |           CB113147           |                               |
|                       |           BP283838           |                               |
|                       |           BQ316043           |                               |
|                       |           BP238603           |                               |
|                       |           BP243369           |                               |
|                       |           BG875954           |                               |
|                       |           BG747979           |                               |
|                       |           BP383045           |                               |
|                       |           AU135291           |                               |
|                       |           AW391058           |                               |
|                       |           BQ350347           |                               |
|                       |           BP327055           |                               |
|                       |           BP327077           |                               |
|                       |           BI857404           |                               |
|                       |           BQ317910           |                               |
|                       |           BM746594           |                               |
|                       |           BI090733           |                               |
|                       |           BM800469           |                               |
|                       |           BG257730           |                               |
|                       |           BQ337793           |                               |
|                       |           BQ337782           |                               |
|                       |           BQ337784           |                               |
|                       |           BQ337783           |                               |
|                       |           BQ337785           |                               |
|                       |           BM742625           |                               |
|                       |           BG187857           |                               |
|                       |           BF796516           |                               |
|                       |           CD298931           |                               |
|                       |           BG875932           |                               |
|                       |           CN364535           |                               |
|                       |           AA192409           |                               |
|                       |           AA166798           |                               |
|                       |           AA166797           |                               |
|                       |           CN364547           |                               |
|                       |           BQ362395           |                               |
|                       |           BG251210           |                               |
|                       |           BQ423892           |                               |
|                       |           AJ006412           |                               |
|                       |           BE087480           |                               |
|                       |           BE087605           |                               |
|                       |           BM905284           |                               |
|                       |           BU194723           |                               |
|                       |           BQ362396           |                               |
|                       |           BQ350339           |                               |
|                       |           BQ350340           |                               |
|                       |           BQ350341           |                               |
|                       |           BQ350338           |                               |
|                       |           BQ001231           |                               |
|                       |           CV350686           |                               |
|                       |           CV350698           |                               |
|                       |           CN364540           |                               |
|                       |           BG182902           |                               |
|                       |           CF454992           |                               |
|                       |           AL582401           |                               |
|                       |           CR617158           |                               |
|                       |           BI091981           |                               |
|                       |           AU125253           |                               |
|                       |           BM790377           |                               |
|                       |           AI242705           |                               |
|                       |           AA188621           |                               |
|                       |           BM992273           |                               |
|                       |           BM722700           |                               |
|                       |           CB128127           |                               |
|                       |           BX118039           |                               |
|                       |           BQ050590           |                               |
|                       |             N47171           |                               |
|                       |           BM462000           |                               |
|                       |           BF000422           |                               |
|                       |           BM903810           |                               |
|                       |           AA311063           |                               |
|                       |           AA030047           |                               |
|                       |           AA211340           |                               |
|                       |           BF094826           |                               |
|                       |           BF675081           |                               |
|                       |           AL528092           |                               |
|                       |           CR613918           |                               |
|                       |           BF242649           |                               |
|                       |           AI916168           |                               |
|                       |           BM698174           |                               |
|                       |           AL582383           |                               |
|                       |           CB110167           |                               |
|                       |           BF790037           |                               |
|                       |           BG292022           |                               |
|                       |           BQ350728           |                               |
|                       |           BU198843           |                               |
|                       |           BM799265           |                               |
|                       |           CN485327           |                               |
|                       |           BE874452           |                               |
|                       |             R83170           |                               |
|                       |           CB111477           |                               |
|                       |           AI761569           |                               |
|                       |           CB243493           |                               |
|                       |           BM792373           |                               |
|                       |           BU625504           |                               |
|                       |           CA418276           |                               |
|                       |           CA419727           |                               |
|                       |           BQ014387           |                               |
|                       |           BE173293           |                               |
|                       |           BF096133           |                               |
|                       |           BU625741           |                               |
|                       |           BE936025           |                               |
|                       |           CB129258           |                               |
|                       |           BE699228           |                               |
|                       |           BF954749           |                               |
|                       |           AA587572           |                               |
|                       |           BF876469           |                               |
|                       |           BF876462           |                               |
|                       |           BF954744           |                               |
|                       |           BI093443           |                               |
|                       |           AW020238           |                               |
|                       |           BE935980           |                               |
|                       |           AK091864           |                               |
|                       |           BG196250           |                               |
|                       |           BG185326           |                               |
|                       |           AJ713193           |                               |
|                       |           CD698775           |                               |
|                       |           BF002028           |                               |
|                       |           AW073539           |                               |
|                       |           AV724668           |                               |
|                       |           AI814709           |                               |
|                       |           BX476458           |                               |
|                       |           AI376566           |                               |
|                       |           AA090843           |                               |
|                       |           BI491291           |                               |
|                       |           BU735971           |                               |
|                       |           CA314755           |                               |
|                       |           AI624318           |                               |
|                       |           AW043629           |                               |
|                       |           BF931019           |                               |
|                       |           AW856275           |                               |
|                       |           AW274520           |                               |
|                       |           AA524068           |                               |
|                       |           CR738781           |                               |
|                       |           BF431930           |                               |
|                       |           AI022628           |                               |
|                       |           AI381943           |                               |
|                       |           BQ013542           |                               |
|                       |           BU675105           |                               |
|                       |           AU155029           |                               |
|                       |           AI493253           |                               |
|                       |           AA993296           |                               |
|                       |           AI123070           |                               |
|                       |           AA977315           |                               |
|                       |           AI868044           |                               |
|                       |           AA977243           |                               |
|                       |           AU151620           |                               |
|                       |           AI352453           |                               |
|                       |           BE698976           |                               |
|                       |           BQ018929           |                               |
|                       |           AI290422           |                               |
|                       |           AI590303           |                               |
|                       |           AI761747           |                               |
|                       |           AI167243           |                               |
|                       |           AA554283           |                               |
|                       |           AA587549           |                               |
|                       |           AA029935           |                               |
|                       |           AW134931           |                               |
|                       |           BU677664           |                               |
|                       |           AW291741           |                               |
|                       |           BQ003673           |                               |
|                       |           CA434102           |                               |
|                       |           AA946677           |                               |
|                       |           AA481405           |                               |
|                       |           BU675470           |                               |
|                       |           AI869347           |                               |
|                       | *Seq. mapped in strand Minus*| *Seq. mapped in strand Minus* |
|                       |           AU138299           |          BC058093             |
|                       |           BM683065           |          AF179302             |
|                       |           CR618059           |          AB057418             |
|                       |           AL568929           |          AK048838             |
|                       |           CF453729           |                               |
|                       |           BF224185           |                               |
|                       |           BE463598           |                               |
|                       |           CA436680           |                               |
|                       |           CR740419           |                               |
|                       |           CA416394           |                               |
|                       |           AI075796           |                               |
|                       |           AW023641           |                               |
|                       |           AA747532           |                               |
|                       |           AI657128           |                               |
|                       |           AJ131720           |                               |
|                       |           AF151538           |                               |
|                       |           AW976074           |                               |
|                       |           CB851608           |                               |
|                       |           BQ435354           |                               |
|                       |           BQ186990           |                               |
|                       |           CK300059           |                               |
|                       |           BM983874           |                               |
|                       |           BM969588           |                               |
|                       |           BM840902           |                               |
|                       |           CA944436           |                               |
|                       |           AF357886           |                               |
|                       |           AF206019           |                               |
|                       |           BC037734           |                               |
|                       |           BX479996           |                               |
|                       |           AB047646           |                               |
|                       |           AL541483           |                               |
|                       |           BQ427650           |                               |
|                       |           BI768271           |                               |
|                       |           BQ429164           |                               |
|                       |           AW382371           |                               |
|                       |           AW195938           |                               |
|                       |           AW129383           |                               |
|                       |           CA951106           |                               |
|                       |           AW501064           |                               |
|                       |           BM839593           |                               |
|                       |           CB958310           |                               |
|                       |           AW772658           |                               |
|                       |           BI857649           |                               |
|                       |           AA026971           |                               |
|                       |           CN298817           |                               |
|                       |           BQ361708           |                               |
|                       |           AA358235           |                               |
|                       |           CR736537           |                               |
|                       |           CR744766           |                               |
|                       |             W87783           |                               |
|                       |           CN298818           |                               |
|                       |           BM564698           |                               |
|                       |           BQ894198           |                               |
|                       |           AI372847           |                               |
|                       |           BM991672           |                               |
|                       |           AW005291           |                               |
|                       |           AI240677           |                               |
|                       |           AA705362           |                               |
|                       |           AA451939           |                               |
|                       |           BU682259           |                               |
|                       |           CD369118           |                               |
|                       |           AI683047           |                               |
|                       |           AA195405           |                               |
|                       |           AI500570           |                               |
|                       |           AI248924           |                               |
|                       |           AI452513           |                               |
|                       |           BQ009819           |                               |
|                       |           CB851062           |                               |
|                       |           AK025176           |                               |
|                       |           AI216804           |                               |
|                       |           AI301157           |                               |
|                       |           AI432201           |                               |
|                       |           BM833559           |                               |
|                       |           AA677409           |                               |
|                       |           BG993645           |                               |
|                       |           BF127653           |                               |
|                       |           BG288771           |                               |
|                       |           AA195457           |                               |
|                       |           AA746273           |                               |
|                       |           BG025670           |                               |
|                       |           CB123061           |                               |
|                       |           AA713663           |                               |
|                       |           BP320037           |                               |
|                       |           BX479503           |                               |
|                       |           BP197815           |                               |
|                       |           AA393888           |                               |
|                       |             H89829           |                               |
|                       |           AW376861           |                               |
|                       |           AA029147           |                               |
|                       |           BF981993           |                               |
|                       |           BP243952           |                               |
|                       |           BF671964           |                               |
|                       |           AI147937           |                               |
|                       |           BM477338           |                               |
|                       |             T08134           |                               |
|                       |           CN298816           |                               |
|                       |           BP218030           |                               |
|                       |           BP337385           |                               |
|                       |           BP336964           |                               |
|                       |           BM468065           |                               |
|                       |           CB149551           |                               |
|                       |           BF002563           |                               |
|                       |           BQ232138           |                               |
|                       |           BM719099           |                               |
|                       |           BP332286           |                               |
|                       |           AI903743           |                               |
|                       |           BP383000           |                               |
|                       |           BP383001           |                               |
|                       |           BP289282           |                               |
|                       |           BP268678           |                               |
|                       |           BP269179           |                               |
|                       |           BX953482           |                               |
|                       |           BU902033           |                               |
|                       |           AK002087           |                               |
|                       |           CD655507           |                               |
|                       |           CN389245           |                               |
|                       |           CN389246           |                               |
|                       |           BG720381           |                               |
+-----------------------+------------------------------+-------------------------------+
|      #   84           | *Seq. mapped in strand Plus* | *Seq. mapped in strand Plus*  |
|                       |           BQ184645           |          BG145062             |
|                       | *Seq. mapped in strand Minus*| *Seq. mapped in strand Minus* |
|                       |           AI050023           |            W29611             |
|                       |           AW022189           |          AB000803             |
|                       |           AW075850           |            D87747             |
|                       |           AI582682           |          BC031665             |
|                       |             T29035           |            X99582             |
|                       |             H29001           |            U59760             |
|                       |           AW193726           |            Z80112             |
|                       |           AW594481           |          BU523025             |
|                       |           AI683322           |          BG173867             |
|                       |           BQ025781           |          BF101953             |
|                       |           AI358446           |          BG915636             |
|                       |           AI675038           |          BG174412             |
|                       |           AI866909           |          AW227957             |
|                       |           AI920946           |            Z80111             |
|                       |           CA436565           |          BX513842             |
|                       |           BQ024573           |          BY041153             |
|                       |           AI300807           |          BB872868             |
|                       |           AI344724           |          BQ265435             |
|                       |           AA426566           |            W88094             |
|                       |           AA426644           |                               |
|                       |           AI159856           |                               |
|                       |           AA760743           |                               |
|                       |           AI583169           |                               |
|                       |           AI434652           |                               |
|                       |           AI249788           |                               |
|                       |           AA824607           |                               |
|                       |           AA804282           |                               |
|                       |           AA731086           |                               |
|                       |           AA743645           |                               |
|                       |           AA747545           |                               |
|                       |           AA411265           |                               |
|                       |           BU682354           |                               |
|                       |           BM971140           |                               |
|                       |           BM978372           |                               |
|                       |           BQ446320           |                               |
|                       |           CA436903           |                               |
|                       |           BQ003324           |                               |
|                       |           AI493618           |                               |
|                       |           CR598681           |                               |
|                       |           CR604071           |                               |
|                       |             D10924           |                               |
|                       |           AA284569           |                               |
|                       |           AF147204           |                               |
|                       |           BC020968           |                               |
|                       |             X71635           |                               |
|                       |             L06797           |                               |
|                       |           AK129916           |                               |
|                       |           CR614663           |                               |
|                       |           AW594595           |                               |
|                       |           CR596547           |                               |
|                       |           CR619476           |                               |
|                       |           CR605131           |                               |
|                       |           CR594428           |                               |
|                       |           CR623838           |                               |
|                       |             M99293           |                               |
|                       |           CR610268           |                               |
|                       |           CR598713           |                               |
|                       |           CR614594           |                               |
|                       |           CR614199           |                               |
|                       |           CR594588           |                               |
|                       |           CR601301           |                               |
|                       |             L01639           |                               |
|                       |           AF025375           |                               |
|                       |           AY242129           |                               |
|                       |           AF348491           |                               |
|                       |           BT006660           |                               |
|                       |           BX396365           |                               |
|                       |           BM545259           |                               |
|                       |           BX397685           |                               |
|                       |           BX418530           |                               |
|                       |           BX421511           |                               |
|                       |           BX462627           |                               |
|                       |           BX353253           |                               |
|                       |           BX421287           |                               |
|                       |           BX358346           |                               |
|                       |           BM920800           |                               |
|                       |           BX462182           |                               |
|                       |           BI824663           |                               |
|                       |           BI754094           |                               |
|                       |           BM051973           |                               |
|                       |           BI762229           |                               |
|                       |           BQ718617           |                               |
|                       |           CD389217           |                               |
|                       |           BX362477           |                               |
|                       |           CA454255           |                               |
|                       |           BI756157           |                               |
|                       |           BP260405           |                               |
|                       |           BI819797           |                               |
|                       |           BX443258           |                               |
|                       |           BX443334           |                               |
|                       |           BX443228           |                               |
|                       |           BI761664           |                               |
|                       |           BU146456           |                               |
|                       |           BI771103           |                               |
|                       |           BU945383           |                               |
|                       |           BX424593           |                               |
|                       |           BG685901           |                               |
|                       |           CO960366           |                               |
|                       |           BI917014           |                               |
|                       |           CB988971           |                               |
|                       |           BI597875           |                               |
|                       |           BI546999           |                               |
|                       |           BF797340           |                               |
|                       |           AU117058           |                               |
|                       |           CF595894           |                               |
|                       |           BQ109515           |                               |
|                       |           BI761118           |                               |
|                       |           BE617402           |                               |
|                       |           BI765768           |                               |
|                       |           BX421959           |                               |
|                       |           CB987769           |                               |
|                       |           CD640668           |                               |
|                       |           BI835125           |                               |
|                       |           CB215245           |                               |
|                       |           BG025793           |                               |
|                       |           CB122606           |                               |
|                       |           BF591285           |                               |
|                       |           CD251240           |                               |
|                       |           BP300248           |                               |
|                       |           BP224898           |                               |
|                       |           BP236598           |                               |
|                       |           BP338360           |                               |
|                       |           BP197054           |                               |
|                       |           BP198585           |                               |
|                       |           BP225804           |                               |
|                       |           BI668010           |                               |
|                       |           BP197755           |                               |
|                       |           BP197789           |                               |
|                       |           BP198110           |                               |
|                       |           BP208818           |                               |
|                       |           BP225182           |                               |
|                       |           BP338386           |                               |
|                       |           BP367587           |                               |
|                       |           BP331003           |                               |
|                       |           BP215948           |                               |
|                       |           BP339598           |                               |
|                       |           BP366593           |                               |
|                       |           BP224524           |                               |
|                       |           BP264409           |                               |
|                       |           BP304515           |                               |
|                       |           AW403941           |                               |
|                       |           AA354393           |                               |
|                       |           CD695230           |                               |
|                       |           BE245301           |                               |
|                       |           AA248174           |                               |
|                       |           AA354040           |                               |
|                       |           BE244758           |                               |
|                       |           BM700824           |                               |
|                       |           AA354044           |                               |
|                       |           BF792200           |                               |
|                       |           BP260206           |                               |
|                       |           AA357257           |                               |
|                       |           AA378777           |                               |
|                       |           AU099377           |                               |
|                       |           AA339027           |                               |
|                       |           AA091989           |                               |
|                       |           AA356857           |                               |
|                       |           BF173003           |                               |
|                       |           BF175266           |                               |
|                       |           AA355098           |                               |
|                       |           AA357215           |                               |
+-----------------------+------------------------------+-------------------------------+
|      #   85           | *Seq. mapped in strand Plus* | *Seq. mapped in strand Plus*  |
|                       |           BM994830           |          BY299572             |
|                       |           CB242678           |          BY078345             |
|                       |           AV687517           |          BY273805             |
|                       |           BM988573           |          BB652892             |
|                       |           CA437793           |          AK049963             |
|                       |           AA886338           |          AI786906             |
|                       |           BM994441           |          BY107867             |
|                       |           AI085577           |          BY106315             |
|                       |           AI291133           |          BY737163             |
|                       |           AI348651           |          BB871930             |
|                       |           BQ772099           |          BB871281             |
|                       |           BM693710           |          BB871053             |
|                       |           AA044450           |          BI248627             |
|                       |           BQ233868           |          CF550891             |
|                       |                              |          BY769198             |
|                       |                              |          BY130562             |
|                       |                              |          AU035105             |
|                       |                              |          BI248173             |
|                       |                              |          CA494617             |
|                       |                              |          BY098450             |
|                       |                              |          BY065793             |
|                       |                              |          BY060869             |
|                       |                              |          BB859318             |
|                       |                              |          BU524549             |
|                       |                              |          CB953943             |
|                       |                              |          BY227424             |
|                       |                              |          CB952061             |
|                       |                              |          BY128667             |
|                       |                              |          BY123593             |
|                       |                              |          BY331972             |
|                       |                              |          BY121561             |
|                       |                              |          BY073596             |
|                       |                              |          BY074696             |
|                       |                              |          BY328598             |
|                       |                              |          BY150877             |
|                       |                              |          BY106417             |
|                       |                              |          BY163610             |
|                       |                              |          BY068736             |
|                       |                              |          BY054035             |
|                       |                              |          BY306743             |
|                       |                              |          BY315003             |
|                       |                              |          BY027825             |
|                       |                              |          BY067458             |
|                       |                              |          BY050534             |
|                       |                              |          BY069468             |
|                       |                              |          BY044880             |
|                       |                              |          BY063248             |
|                       |                              |          BY093131             |
|                       |                              |          BY026844             |
|                       |                              |          BY086284             |
|                       |                              |          BY029645             |
|                       |                              |          BY299088             |
|                       |                              |          BB845577             |
|                       |                              |          BY277435             |
|                       |                              |          BY258877             |
|                       |                              |          BY228311             |
|                       |                              |          BY282047             |
|                       |                              |          BY059490             |
|                       |                              |          BY729631             |
|                       |                              |          BY736621             |
|                       |                              |          BY736498             |
|                       |                              |          AK083046             |
|                       |                              |          AI156879             |
|                       |                              |          BY070157             |
|                       |                              |          BY103176             |
|                       |                              |          BY166079             |
|                       |                              |          BY150259             |
|                       |                              |          BY123400             |
|                       |                              |          BY057250             |
|                       |                              |          BY151847             |
|                       |                              |          BY043502             |
|                       |                              |          BY344680             |
|                       |                              |          BY306556             |
|                       |                              |          BY159828             |
|                       |                              |          AI663172             |
|                       |                              |          CA469401             |
|                       |                              |          BY090428             |
|                       |                              |          BY092552             |
|                       |                              |          AA989822             |
|                       |                              |          AI891680             |
|                       |                              |          AI119455             |
|                       |                              |          AI874524             |
|                       |                              |          AI746772             |
|                       |                              |          AW106148             |
|                       |                              |          CA493194             |
|                       |                              |          AI098541             |
|                       |                              |          AI182550             |
|                       |                              |          AI891803             |
|                       |                              |          AI529213             |
|                       |                              |          AI573980             |
|                       |                              |          CA573950             |
|                       |                              |          CB566525             |
|                       |                              |          AI035765             |
|                       |                              |          AW226784             |
|                       |                              |          CD353967             |
|                       |                              |          CF895746             |
|                       |                              |          BY304085             |
|                       |                              |          BY111485             |
|                       |                              |          BY303290             |
|                       |                              |          BY108895             |
|                       |                              |          CF723723             |
|                       |                              |          CA871244             |
|                       |                              |          CA870571             |
|                       |                              |          CA875544             |
|                       |                              |          CN717991             |
|                       |                              |          BG963709             |
|                       |                              |          CD553513             |
|                       |                              |          BI330771             |
|                       |                              |          AW107061             |
|                       |                              |          BY007159             |
|                       |                              |          CD549783             |
|                       |                              |          CA552350             |
|                       |                              |          BF179008             |
|                       |                              |          BG914839             |
|                       |                              |          BG973524             |
|                       |                              |          AI573959             |
|                       |                              |          BG974517             |
|                       |                              |          CF172882             |
|                       |                              |          CN671153             |
|                       |                              |          BF169120             |
|                       |                              |          BI656737             |
|                       |                              |          CB182092             |
|                       |                              |          BQ963129             |
|                       |                              |          BU516150             |
|                       |                              |          CA543585             |
|                       |                              |          BM171756             |
|                       |                              |          BQ084910             |
|                       |                              |          BG974723             |
|                       |                              |          BI651979             |
|                       |                              |          BG920120             |
|                       |                              |          BE226945             |
|                       |                              |          CA540202             |
|                       |                              |          BG173885             |
|                       |                              |          CA535283             |
|                       |                              |          CA535376             |
|                       |                              |          CA542257             |
|                       |                              |          BI156075             |
|                       |                              |          BF138957             |
|                       |                              |          AA408088             |
|                       |                              |          CA540005             |
|                       |                              |          CN712977             |
|                       |                              |          BU511828             |
|                       |                              |          BU514416             |
|                       |                              |          CA978910             |
|                       |                              |          BQ887621             |
|                       |                              |          BU519460             |
|                       |                              |          AF055664             |
|                       |                              |          CF901732             |
|                       |                              |          CK021673             |
|                       |                              |          CA544107             |
|                       |                              |          BF181102             |
|                       |                              |          BI686207             |
|                       |                              |          BE303547             |
|                       |                              |          BC057876             |
|                       |                              |          BF180254             |
|                       |                              |          AI746952             |
|                       |                              |          BQ887244             |
|                       |                              |          CA576290             |
|                       |                              |          CF165367             |
|                       |                              |          CN711495             |
|                       |                              |          BI454020             |
|                       |                              |            D28630             |
|                       |                              |          CA549041             |
|                       |                              |          BQ884916             |
|                       |                              |          CF911956             |
|                       |                              |          CF162956             |
|                       |                              |          CD562766             |
|                       |                              |          CA568246             |
|                       |                              |          BE370125             |
|                       |                              |          CN714575             |
|                       |                              |          CN708351             |
|                       |                              |          CN690709             |
|                       |                              |          CN712517             |
|                       |                              |          CN710922             |
|                       |                              |          AW412068             |
|                       |                              |          BG078307             |
|                       |                              |          BG972171             |
|                       |                              |          CN710684             |
|                       |                              |          BU057703             |
|                       |                              |          CK625434             |
|                       |                              |          BI685192             |
|                       |                              |          BG261875             |
|                       |                              |          CF726097             |
|                       |                              |          BG973943             |
|                       |                              |          BG864996             |
|                       |                              |          CK788510             |
|                       |                              |          CD353579             |
|                       |                              |          CB204200             |
|                       |                              |          CN709361             |
|                       |                              |          CN709000             |
|                       |                              |          CN705527             |
|                       |                              |          BQ570303             |
|                       |                              |          BF141730             |
|                       |                              |          BF168282             |
|                       |                              |          AI573757             |
|                       |                              |          BM502631             |
|                       |                              |          CN713678             |
|                       |                              |          BF141834             |
|                       |                              |          BE569547             |
|                       |                              |          CA573974             |
|                       |                              |          CF912788             |
|                       |                              |          BG915083             |
|                       |                              |          CN673028             |
|                       |                              |          CA551029             |
|                       |                              |          CD561031             |
|                       |                              |          BE284647             |
|                       |                              |          CF902004             |
|                       |                              |          BE533960             |
|                       |                              |          AW742153             |
|                       |                              |          BM729850             |
|                       |                              |          AW226669             |
|                       |                              |          AW260588             |
|                       |                              |          BY070772             |
|                       |                              |          BI151700             |
|                       |                              |          BE369460             |
|                       |                              |          BQ563483             |
|                       |                              |          CA575264             |
|                       |                              |          BE860140             |
|                       |                              |          CF160157             |
|                       |                              |          CF156121             |
|                       |                              |          CF156136             |
|                       |                              |          BY245852             |
|                       |                              |          CK617936             |
|                       |                              |          AI157370             |
|                       |                              |          CN669546             |
|                       |                              |          CA976912             |
|                       |                              |          CF530990             |
|                       |                              |          BC060653             |
|                       |                              |          BY307251             |
|                       |                              |          CN673640             |
|                       |                              |          BQ258216             |
|                       |                              |          BG245172             |
|                       |                              |          BM947859             |
|                       |                              |          BB853280             |
|                       |                              |          BY110617             |
|                       |                              |          BF022164             |
|                       |                              |          AI786852             |
|                       |                              |          AI049306             |
|                       |                              |          CN708654             |
|                       |                              |          CN712088             |
|                       |                              |          BG972092             |
|                       |                              |          BI659326             |
|                       |                              |          CF158143             |
|                       |                              |          CA553721             |
|                       |                              |          CA548612             |
|                       |                              |          CF159984             |
|                       |                              |          CA549921             |
|                       |                              |          CA549752             |
|                       |                              |          CF158965             |
|                       |                              |          CA548438             |
|                       |                              |          CA550383             |
|                       |                              |          CA539322             |
|                       |                              |          BQ563482             |
|                       |                              |          CA551742             |
|                       |                              |          BI112506             |
|                       |                              |          CN709427             |
|                       |                              |          AA616657             |
|                       |                              |          CA554747             |
|                       |                              |          CF902233             |
|                       |                              |          BM171697             |
|                       |                              |          BG972102             |
|                       |                              |          BB704917             |
|                       |                              |          CF734913             |
|                       |                              |          BQ042982             |
|                       |                              |          CD543486             |
|                       |                              |          CA555148             |
|                       |                              |          BM947964             |
|                       |                              |          CN714239             |
|                       |                              |          CN714919             |
|                       |                              |          BY367714             |
|                       |                              |          BI660531             |
|                       |                              |          BI691103             |
|                       |                              |          AA414938             |
|                       |                              |          AA422896             |
|                       |                              |          BQ264795             |
|                       |                              |          CN706700             |
|                       |                              |          BF163844             |
|                       |                              |          CN712971             |
|                       |                              |          CF748267             |
|                       |                              |          BM949995             |
|                       |                              |          BB577686             |
|                       |                              |          CF735886             |
|                       |                              |          BG920908             |
|                       |                              |          CN539736             |
|                       |                              |          AA209757             |
|                       |                              |          CB181313             |
|                       |                              |          CD805022             |
|                       |                              |          BI149628             |
|                       |                              |          AA726121             |
|                       |                              |          CF726068             |
|                       |                              |          CB195905             |
|                       |                              |            D10917             |
|                       |                              |          BG976443             |
|                       |                              |          BF179509             |
|                       |                              |          BQ218680             |
|                       |                              |          BI103289             |
|                       |                              |          BI108165             |
|                       |                              |          BF141208             |
|                       |                              |          AA388024             |
|                       |                              |          AA388010             |
|                       |                              |          AA387802             |
|                       |                              |          AA387981             |
|                       |                              |          CK633618             |
|                       |                              |          CB173830             |
|                       |                              |          CK130026             |
|                       |                              |          BI559132             |
|                       |                              |          AA152884             |
|                       |                              |          AW988431             |
|                       |                              |          CB840962             |
|                       |                              |          CF161371             |
|                       |                              |          CF161308             |
|                       |                              |          CA553421             |
|                       |                              |          CF156664             |
|                       |                              |          CA550463             |
|                       |                              |          CA550356             |
|                       |                              |          CA548228             |
|                       |                              |          CA553885             |
|                       |                              |          AL022866             |
|                       |                              |          AI172954             |
|                       |                              |          CN708679             |
|                       |                              |          AI098200             |
|                       |                              |          AA791267             |
|                       |                              |            X61872             |
|                       |                              |          AW536145             |
|                       |                              |          BU512092             |
|                       |                              |          CF581024             |
|                       |                              |          CF581025             |
|                       |                              |          AA497510             |
|                       |                              |          BE448156             |
|                       |                              |          BI348940             |
|                       |                              |          CA748274             |
|                       |                              |          BG088755             |
|                       |                              |          AA798535             |
|                       |                              |          AA673489             |
|                       |                              |          AW551675             |
|                       |                              |          BM210824             |
|                       |                              |          AW260005             |
|                       |                              |          AA163365             |
|                       |                              |          BM729580             |
|                       |                              |          CA452015             |
|                       |                              |          CD353118             |
|                       |                              |          BI466907             |
|                       |                              |          AA560344             |
|                       |                              |          AW548152             |
|                       |                              |          BQ264794             |
|                       |                              |          AU043135             |
|                       |                              |          BM293766             |
|                       |                              |          AW114039             |
|                       |                              |          BF681968             |
|                       |                              |          BE851016             |
|                       |                              |          BM214804             |
|                       |                              |          BM240851             |
|                       |                              |          BM502316             |
|                       |                              |          AA467431             |
|                       |                              |          AW548618             |
|                       |                              |          AW546380             |
|                       |                              |          AI043018             |
|                       |                              |          AW111684             |
|                       |                              |          AI448912             |
|                       |                              |          BM242149             |
|                       |                              |          BY412741             |
|                       |                              |          BB762494             |
|                       |                              |          AI036147             |
|                       |                              |          AI646945             |
|                       |                              |          BI738174             |
|                       |                              |          AI120368             |
|                       |                              |          BM202515             |
|                       |                              |          AA170895             |
|                       |                              |          BE692768             |
|                       |                              |          BM211858             |
|                       |                              |          AW557265             |
|                       |                              |          BM213551             |
|                       |                              |          CB953593             |
|                       |                              |          BM204129             |
|                       |                              |          CK336815             |
|                       |                              |          AW544976             |
|                       |                              |          BU613122             |
|                       |                              |          BF225796             |
|                       |                              |          AI607997             |
|                       |                              |          BM898977             |
|                       |                              |          AI787348             |
|                       |                              |          BY388762             |
|                       |                              |          BE134185             |
|                       |                              |          BG091263             |
|                       |                              |          BF117646             |
|                       |                              |          BF117656             |
|                       |                              |          CD363338             |
|                       |                              |          BU846771             |
|                       |                              |          AW230179             |
|                       |                              |          AU018932             |
|                       |                              |          BY514821             |
|                       |                              |          BY515911             |
|                       |                              |          CB058074             |
|                       |                              |          BY435887             |
|                       |                              |          BY435218             |
|                       |                              |          BY585243             |
|                       |                              |          BY585923             |
|                       |                              |          BY579927             |
|                       |                              |          BY409425             |
|                       |                              |          BY698345             |
|                       |                              |          BI330163             |
|                       |                              |          BY509438             |
|                       |                              |          CF533869             |
|                       |                              |          AI852330             |
|                       |                              |          BF461299             |
|                       | *Seq. mapped in strand Minus*| *Seq. mapped in strand Minus* |
|                       |           CR605922           |          BE656271             |
|                       |           BM670991           |          BI135158             |
|                       |           BC045773           |          BU786701             |
|                       |           BM712324           |          AI842015             |
|                       |           AW016571           |          AI845086             |
|                       |           BU624177           |          CO039211             |
|                       |           BM971747           |          AK019174             |
|                       |           CA430286           |          AI131685             |
|                       |           BM679793           |          AI849632             |
|                       |           BU624063           |          BY665128             |
|                       |           AW768808           |          BY432056             |
|                       |           BM676100           |          BY411681             |
|                       |           BU741406           |          BY580124             |
|                       |           AW575684           |          BQ829921             |
|                       |           AI146328           |          BY407351             |
|                       |           BF064229           |          BY668080             |
|                       |           AW513502           |          AU020852             |
|                       |           AI275209           |          CD548275             |
|                       |           AI308977           |          BM212549             |
|                       |           AI636177           |          BM216906             |
|                       |           AA419016           |          CA772835             |
|                       |           AI334314           |          AU021404             |
|                       |           AA401928           |          BM215731             |
|                       |           BG939276           |          AW823592             |
|                       |           BU686494           |          CK337222             |
|                       |           AI983214           |          BI109614             |
|                       |           AI559257           |          BY396525             |
|                       |           BU500837           |          BY397869             |
|                       |           BU742352           |          BY400554             |
|                       |           BU686505           |          BY612952             |
|                       |           CA447483           |          BY689347             |
|                       |           CB851737           |          AW049877             |
|                       |           BU688610           |          BU614962             |
|                       |           AI827430           |          BY665054             |
|                       |           BM969891           |          BY407924             |
|                       |           BU684627           |          BY415341             |
|                       |           CA418725           |          BY620836             |
|                       |           CB529344           |          BB836408             |
|                       |           BM988674           |          BY643613             |
|                       |           BG939277           |          AA071820             |
|                       |           BM988550           |          BY435722             |
|                       |           CA313262           |          BY384240             |
|                       |           CA425496           |          BB830675             |
|                       |           CB241604           |          BB821714             |
|                       |           CB242760           |          BY434698             |
|                       |           BU607403           |          BB834365             |
|                       |           BU609107           |          BB829672             |
|                       |           BU676424           |          BI658559             |
|                       |           CB306799           |          AW557073             |
|                       |           BU616328           |          AW539079             |
|                       |           BC034244           |          AW544312             |
|                       |           BC035442           |          CD776915             |
|                       |             D29805           |          AW536129             |
|                       |           AI761631           |          AW537266             |
|                       |           BM151223           |          BX517346             |
|                       |           BM153496           |          BF147873             |
|                       |           BU857100           |          AW324232             |
|                       |           BU626102           |          BY391411             |
|                       |           BU196325           |          BE956928             |
|                       |           AI057031           |          BF021388             |
|                       |           CA314385           |          BY393327             |
|                       |           CA437619           |          BM244543             |
|                       |           BQ002021           |          AI854567             |
|                       |           CA444682           |          BB793557             |
|                       |           BM984131           |          BY407964             |
|                       |           BX648438           |          AW123805             |
|                       |           AI311915           |          BB734388             |
|                       |           BG939552           |          BY424714             |
|                       |           BU679188           |          BY429484             |
|                       |           BU620021           |          BY581629             |
|                       |           BQ706434           |          BY419051             |
|                       |           BU674360           |          BY491322             |
|                       |           AA917825           |          BY438636             |
|                       |           AA917814           |          AU044073             |
|                       |           BM994221           |          BY444682             |
|                       |           BU633559           |          AU043330             |
|                       |           BU685506           |          BB788202             |
|                       |             X13223           |          BY384017             |
|                       |             U10472           |          AI449510             |
|                       |             W49627           |          BY432311             |
|                       |           BX105627           |          AW545538             |
|                       |           BU184731           |          BB777075             |
|                       |             W46207           |          BY581252             |
|                       |           BP355154           |          BB796035             |
|                       |           AA043668           |          BB783087             |
|                       |           BX474631           |          CN723540             |
|                       |           BU431538           |          BG071657             |
|                       |             M13701           |          AU044886             |
|                       |             X14085           |          BM207912             |
|                       |             M22921           |          BQ831056             |
|                       |           BG012758           |          BE630798             |
|                       |           BP281022           |          BM222222             |
|                       |           AW362033           |          BM244019             |
|                       |           BU430256           |          BM245258             |
|                       |           AA233236           |          BB763666             |
|                       |           AI192425           |          AU043154             |
|                       |             H74175           |          BM244167             |
|                       |             H43226           |          BM231914             |
|                       |           BX490190           |          BB697392             |
|                       |             W31512           |          BM244378             |
|                       |           CA390750           |          AW764142             |
|                       |             R67590           |          BM233464             |
|                       |           AW467118           |          BM247017             |
|                       |             U10473           |          BQ265444             |
|                       |             X55415           |          BM502099             |
|                       |             R66709           |          AW553408             |
|                       |           BQ334185           |          BY760070             |
|                       |           BF891120           |          BC020034             |
|                       |           BF891373           |          BC057446             |
|                       |             R30947           |          AK003493             |
|                       |           AI572046           |          BU782437             |
|                       |           AA476930           |          BB735157             |
|                       |           BM750694           |          BB687813             |
|                       |           BF434295           |          BF133911             |
|                       |           AI028425           |          BB706530             |
|                       |           BX471802           |          BI654301             |
|                       |           AA233160           |          BU898944             |
|                       |           AW293833           |          AB044414             |
|                       |           BF590081           |          AI006011             |
|                       |           BM930878           |          AA939752             |
|                       |           BG272371           |          AA154991             |
|                       |           AA889902           |          AA981869             |
|                       |           AA731231           |          BE627716             |
|                       |           AI149377           |          AA866898             |
|                       |           AI149203           |          BG084502             |
|                       |             U10474           |          AA153899             |
|                       |           AI275447           |          CN456791             |
|                       |             H43230           |          AA881228             |
|                       |             R76006           |          AA726950             |
|                       |             T29439           |          CB846279             |
|                       |           AA890427           |          AA620042             |
|                       |           BP336085           |          AA245719             |
|                       |           AA282090           |          BY358025             |
|                       |             W04613           |          BF117960             |
|                       |           AI827408           |          BE198732             |
|                       |           AI565973           |          BF117986             |
|                       |             H73253           |          AA272288             |
|                       |           AW392078           |            W14212             |
|                       |           AI220042           |          BF012415             |
|                       |           AW592059           |          BG794804             |
|                       |           BF993456           |          BG795634             |
|                       |             R76007           |            W87221             |
|                       |           AA463694           |          AA124056             |
|                       |           AI354774           |          BU505262             |
|                       |           AI872346           |          AA033044             |
|                       |           AA243611           |          AA000663             |
|                       |           BP279554           |          AA682140             |
|                       |           AI910739           |          AA414463             |
|                       |             H43190           |          AW913180             |
|                       |           BF431414           |          BQ265445             |
|                       |           BP357160           |          AA105013             |
|                       |           CB133370           |          CD771532             |
|                       |           BG335498           |          AA239984             |
|                       |           CB960625           |          BF150312             |
|                       |           BP278895           |          CN456324             |
|                       |           BP278023           |          CD772238             |
|                       |           BP358101           |          AA265062             |
|                       |           BP281233           |          CF734007             |
|                       |           BG772745           |          CN708864             |
|                       |           BP249507           |          AA117265             |
|                       |           AW140000           |          AA273793             |
|                       |           AA284292           |          AA104495             |
|                       |           BF115171           |          CD770068             |
|                       |           AI091561           |          CF950681             |
|                       |             W02073           |          BU847258             |
|                       |           AI206485           |          AA589077             |
|                       |           BF116182           |          CA544447             |
|                       |           AW236746           |          CB057496             |
|                       |           AI275059           |            W34308             |
|                       |           BF115891           |          CB057573             |
|                       |           BU076050           |            W62565             |
|                       |           AI889080           |          CN711589             |
|                       |           AW024326           |          BY052295             |
|                       |           AI492393           |          BQ891428             |
|                       |             H85120           |          AA265321             |
|                       |           BP315394           |          BQ899856             |
|                       |             R69021           |          BM946833             |
|                       |                              |          BI081844             |
|                       |                              |          AA212262             |
|                       |                              |          BG919217             |
|                       |                              |          CB233561             |
|                       |                              |          BM949216             |
|                       |                              |          BI735425             |
|                       |                              |          CN673188             |
|                       |                              |          CF732083             |
|                       |                              |          BG917593             |
|                       |                              |          AA561260             |
|                       |                              |          AI316265             |
|                       |                              |          CD773124             |
|                       |                              |          BE573767             |
|                       |                              |          CB231284             |
|                       |                              |          BU782238             |
|                       |                              |          AA498017             |
|                       |                              |          CA577516             |
|                       |                              |          BG087976             |
|                       |                              |          BI455853             |
|                       |                              |          BF102142             |
|                       |                              |          BG094267             |
|                       |                              |          BG147991             |
|                       |                              |          CB058102             |
|                       |                              |          BG922893             |
|                       |                              |          CK794436             |
|                       |                              |          BY711087             |
|                       |                              |          BG861538             |
|                       |                              |          BY703754             |
|                       |                              |          CB232685             |
|                       |                              |          CB571427             |
|                       |                              |          AI527847             |
|                       |                              |          BE310440             |
|                       |                              |          CK628181             |
|                       |                              |          CF914395             |
|                       |                              |          AW209890             |
|                       |                              |          BY739310             |
|                       |                              |          CB237675             |
|                       |                              |          CF900061             |
|                       |                              |          AA123077             |
|                       |                              |          BY754028             |
|                       |                              |          CN722863             |
|                       |                              |          BF162442             |
|                       |                              |          CF894093             |
|                       |                              |          BM502370             |
|                       |                              |          CA527932             |
|                       |                              |          BG805660             |
|                       |                              |          CF894693             |
|                       |                              |          CN718197             |
|                       |                              |          CF915087             |
|                       |                              |          BY739754             |
|                       |                              |          BY737229             |
|                       |                              |          BI986862             |
|                       |                              |          BI986978             |
|                       |                              |          CA545622             |
|                       |                              |          CK625865             |
|                       |                              |          CN672060             |
|                       |                              |          CN681684             |
|                       |                              |          CA577674             |
|                       |                              |          CN676592             |
|                       |                              |          CN712217             |
|                       |                              |          BG801631             |
|                       |                              |          CN712250             |
|                       |                              |          CN715138             |
|                       |                              |          AI317719             |
|                       |                              |          BI988885             |
|                       |                              |          CA565360             |
|                       |                              |          CA554534             |
|                       |                              |          CF154974             |
|                       |                              |          CF155087             |
|                       |                              |          CF157715             |
|                       |                              |          CF159383             |
|                       |                              |          CA884355             |
|                       |                              |          CA563626             |
|                       |                              |          CA537731             |
|                       |                              |          CA553252             |
|                       |                              |          CF154098             |
|                       |                              |          AA869360             |
|                       |                              |          BY265610             |
|                       |                              |          AI115801             |
|                       |                              |          CF162297             |
|                       |                              |          CA549822             |
|                       |                              |          BX521945             |
|                       |                              |            W59383             |
|                       |                              |          CA871024             |
|                       |                              |          CF158944             |
|                       |                              |          AA798333             |
|                       |                              |          CF154751             |
|                       |                              |          CD560541             |
|                       |                              |          AI116428             |
|                       |                              |          BE655460             |
|                       |                              |          CF914040             |
|                       |                              |          CA577159             |
|                       |                              |          CA577303             |
|                       |                              |          CA580036             |
|                       |                              |          CA542497             |
|                       |                              |          CN706013             |
|                       |                              |          CF156443             |
|                       |                              |          BB867373             |
|                       |                              |          CA870992             |
|                       |                              |          CA578324             |
|                       |                              |          AA289706             |
|                       |                              |          BY039179             |
|                       |                              |          CD538582             |
|                       |                              |          CF156712             |
|                       |                              |          BE655123             |
|                       |                              |          CD548276             |
|                       |                              |          BB866845             |
|                       |                              |          AW823318             |
|                       |                              |          CA530668             |
|                       |                              |          CK337554             |
|                       |                              |          CN709923             |
|                       |                              |          CF162542             |
|                       |                              |          CF166422             |
|                       |                              |          BB863306             |
|                       |                              |          BG100451             |
|                       |                              |            D21782             |
|                       |                              |          BY260688             |
|                       |                              |          BE192857             |
|                       |                              |          BY226161             |
|                       |                              |          BY033321             |
|                       |                              |          BU073565             |
|                       |                              |          AL360880             |
|                       |                              |          BY247250             |
|                       |                              |          BY136172             |
|                       |                              |            W34454             |
|                       |                              |          BY090015             |
|                       |                              |          BY039085             |
|                       |                              |          BY097303             |
|                       |                              |          BY307151             |
|                       |                              |          BY226900             |
|                       |                              |          AA870877             |
|                       |                              |          BY025621             |
|                       |                              |          BY275010             |
|                       |                              |          BY089030             |
|                       |                              |          BY313989             |
|                       |                              |          BY070941             |
|                       |                              |          BY093425             |
|                       |                              |          BY237409             |
|                       |                              |          BY022690             |
|                       |                              |          BY095875             |
|                       |                              |          BY768247             |
|                       |                              |          BY040688             |
|                       |                              |          BY092410             |
|                       |                              |          BY051685             |
|                       |                              |          BY089089             |
|                       |                              |          BB841489             |
|                       |                              |          BY025364             |
|                       |                              |          CB236091             |
|                       |                              |          BY158547             |
|                       |                              |          BY044021             |
|                       |                              |          BY321358             |
|                       |                              |          BY135564             |
|                       |                              |          AL363187             |
|                       |                              |          BY029664             |
|                       |                              |          BY788896             |
|                       |                              |          BY070329             |
|                       |                              |          BY041130             |
|                       |                              |          BY282175             |
|                       |                              |          BY073037             |
|                       |                              |          BY097193             |
|                       |                              |          BY065382             |
|                       |                              |          BY777232             |
|                       |                              |          BY293240             |
|                       |                              |          BY035562             |
|                       |                              |          BY770526             |
|                       |                              |          BY150622             |
|                       |                              |          BY065940             |
|                       |                              |          BY129170             |
|                       |                              |          BY096738             |
|                       |                              |          BY144246             |
|                       |                              |          BY144733             |
|                       |                              |          BY328787             |
|                       |                              |          AW227463             |
|                       |                              |          BY036103             |
|                       |                              |          BY351069             |
|                       |                              |          BY778502             |
|                       |                              |          BY350670             |
|                       |                              |          AA052332             |
|                       |                              |          BY324793             |
|                       |                              |          BY065280             |
|                       |                              |          BY140964             |
|                       |                              |          BY052252             |
|                       |                              |          BY323020             |
|                       |                              |          AA125119             |
|                       |                              |          BB566727             |
|                       |                              |          CF174702             |
|                       |                              |          CF160655             |
|                       |                              |          CF154088             |
|                       |                              |          BE995500             |
|                       |                              |          BE995391             |
|                       |                              |          BE981183             |
|                       |                              |          BM052277             |
|                       |                              |          BY335334             |
|                       |                              |          BF463079             |
|                       |                              |          BC026824             |
|                       |                              |            M36289             |
|                       |                              |            L16840             |
|                       |                              |          BB703809             |
|                       |                              |            W35439             |
|                       |                              |          BG792233             |
|                       |                              |          BQ031093             |
|                       |                              |          BY524147             |
|                       |                              |          BY502477             |
|                       |                              |          BY512843             |
|                       |                              |          BY533035             |
|                       |                              |          CB523077             |
|                       |                              |          AK013611             |
|                       |                              |          BC003722             |
|                       |                              |          AU014762             |
|                       |                              |          BG069990             |
|                       |                              |          BF146572             |
|                       |                              |          BF319765             |
|                       |                              |          AI836867             |
|                       |                              |          BE983800             |
|                       |                              |          BY496481             |
|                       |                              |          AI450074             |
|                       |                              |          AA240998             |
|                       |                              |          AW741296             |
|                       |                              |          AW741307             |
|                       |                              |          BF138747             |
|                       |                              |          BG246726             |
|                       |                              |          BE995884             |
|                       |                              |          AA516684             |
|                       |                              |          BF180750             |
|                       |                              |          BF226714             |
|                       |                              |          BQ043484             |
|                       |                              |          AW763889             |
|                       |                              |          AA189839             |
+-----------------------+------------------------------+-------------------------------+
|      #   86           | *Seq. mapped in strand Plus* | *Seq. mapped in strand Plus*  |
|                       |           BM449558           |          CK387126             |
|                       |           CB242304           |          CN528964             |
|                       |           AW884580           |          AF328907             |
|                       |           AW884497           |          BY026011             |
|                       |           AF328905           |          BY028080             |
|                       |           CA392621           |          BE851651             |
|                       |           AK096846           |          CN532491             |
|                       |           AB040915           |          BB613175             |
|                       |           BI559936           |          BQ891017             |
|                       |           AL136577           |          AW106055             |
|                       |           BX370810           |          BF118044             |
|                       |           CN429214           |          BI155476             |
|                       |             W40569           |          BE624617             |
|                       |           BP311787           |          AW910374             |
|                       |           BE158914           |          BB638512             |
|                       |           BE158910           |          AK041804             |
|                       |           BP201177           |          BB635107             |
|                       |             D63226           |          BI103881             |
|                       |           AW025860           |          BE652414             |
|                       |           BG749672           |          BF135960             |
|                       |           BG748892           |          BI112822             |
|                       |           BC015659           |          BG246395             |
|                       |           AW583088           |          BC043455             |
|                       |           BP310883           |          CN699379             |
|                       |           CN429215           |          CN697962             |
|                       |           AU134169           |          CN700236             |
|                       |           AK023369           |          BF120930             |
|                       |           AA334307           |          CN697971             |
|                       |           BC057231           |          BF463756             |
|                       |           BU597495           |          CA871945             |
|                       |           BF850441           |          CA880636             |
|                       |           BF993984           |          CD768808             |
|                       |           BI002319           |          BI685971             |
|                       |           BM975376           |          AA088943             |
|                       |           CA314744           |          AI643092             |
|                       |           AW753212           |                               |
|                       |           BG548581           |                               |
|                       |           BP352973           |                               |
|                       |           BQ184101           |                               |
|                       |           AI613267           |                               |
|                       |           BM926093           |                               |
|                       |           AI366036           |                               |
|                       |           BC043604           |                               |
|                       |           BM977475           |                               |
|                       |           AA489216           |                               |
|                       |           AW016246           |                               |
|                       |           AA642400           |                               |
|                       |           AA805332           |                               |
|                       |           AA808025           |                               |
|                       |           BQ002514           |                               |
|                       |           CB306429           |                               |
|                       |           BU741823           |                               |
|                       |           CA431369           |                               |
|                       |           CK824817           |                               |
|                       |           AA810184           |                               |
|                       |           BM264458           |                               |
|                       |           AA807436           |                               |
|                       |           AI359295           |                               |
|                       |           BQ018016           |                               |
|                       |           AI359748           |                               |
|                       |           AA832258           |                               |
|                       |           AA805451           |                               |
|                       |           BM684750           |                               |
|                       |           BQ185236           |                               |
|                       |           BU743230           |                               |
|                       |             H23291           |                               |
|                       |           AI811796           |                               |
|                       |           AI281951           |                               |
|                       |             W40570           |                               |
|                       |           BE048395           |                               |
|                       | *Seq. mapped in strand Minus*| *Seq. mapped in strand Minus* |
|                       |           BU951472           |          AW495248             |
|                       |           BU951742           |                               |
|                       |           AI203685           |                               |
|                       |           AA886911           |                               |
|                       |           CA311079           |                               |
+-----------------------+------------------------------+-------------------------------+
|      #   87           | *Seq. mapped in strand Plus* | *Seq. mapped in strand Plus*  |
|                       |           BC019332           |          AK090003             |
|                       |           BE269104           |          BC004014             |
|                       |           BC008908           |          BC052899             |
|                       |           BQ224534           |          AK014143             |
|                       |           BX416885           |          AY263385             |
|                       |           BP278566           |          AK004021             |
|                       |           BM805146           |          BC010778             |
|                       |           BI669759           |                               |
|                       |           BI602048           |                               |
|                       |           BI667698           |                               |
|                       |           BI544827           |                               |
|                       |           BG701123           |                               |
|                       |           BI458570           |                               |
|                       |           BI599229           |                               |
|                       |           BQ067591           |                               |
|                       |           BP314757           |                               |
|                       |           BP308710           |                               |
|                       |           BM766409           |                               |
|                       |           BM756992           |                               |
|                       |           BM757035           |                               |
|                       |           BM756929           |                               |
|                       |           BM757063           |                               |
|                       |           CB153126           |                               |
|                       |           BM756868           |                               |
|                       |           BM756860           |                               |
|                       |           BM756939           |                               |
|                       |           BM756922           |                               |
|                       |           BM771104           |                               |
|                       |           BP213627           |                               |
|                       |           BM757089           |                               |
|                       |           BM744336           |                               |
|                       |           BP312083           |                               |
|                       |           BP216032           |                               |
|                       |           BP216103           |                               |
|                       |           BM743861           |                               |
|                       |           BM756936           |                               |
|                       |           BM771118           |                               |
|                       |           BM741430           |                               |
|                       |           BM770535           |                               |
|                       |           BM795156           |                               |
|                       |           BP214248           |                               |
|                       |           BM742576           |                               |
|                       |           BM741165           |                               |
|                       |           BM770602           |                               |
|                       |           BM768114           |                               |
|                       |           BM767231           |                               |
|                       |           BX375081           |                               |
|                       |           BM926373           |                               |
|                       |           AL561216           |                               |
|                       |           AL534191           |                               |
|                       |           AL554530           |                               |
|                       |           CR610591           |                               |
|                       |           BI912713           |                               |
|                       |           BM744323           |                               |
|                       |           BP222560           |                               |
|                       |           BI764683           |                               |
|                       |           BI759449           |                               |
|                       |           BP225119           |                               |
|                       |           BM741877           |                               |
|                       |           BP214439           |                               |
|                       |           BP214825           |                               |
|                       |           BP216305           |                               |
|                       |           BP351285           |                               |
|                       |           BG683333           |                               |
|                       |           BG820313           |                               |
|                       |           BM765368           |                               |
|                       |           BP219184           |                               |
|                       |           BP216822           |                               |
|                       |           BM848251           |                               |
|                       |           AU279976           |                               |
|                       |           BM764184           |                               |
|                       |           BP214764           |                               |
|                       |           BP215390           |                               |
|                       |           BP219423           |                               |
|                       |           BP362582           |                               |
|                       |           BP216125           |                               |
|                       |           BP216352           |                               |
|                       |           BP217116           |                               |
|                       |           BP212058           |                               |
|                       |           BP360652           |                               |
|                       |           BX397435           |                               |
|                       |           AL552994           |                               |
|                       |           BG698011           |                               |
|                       |           BI818340           |                               |
|                       |           BU183974           |                               |
|                       |           BQ073416           |                               |
|                       |           CD244255           |                               |
|                       |           BM463880           |                               |
|                       |           BI858690           |                               |
|                       |           BM554552           |                               |
|                       |           AL528062           |                               |
|                       |           CN308824           |                               |
|                       |           BX384921           |                               |
|                       |           AL533284           |                               |
|                       |           CR621138           |                               |
|                       |           BI601885           |                               |
|                       |           BG715245           |                               |
|                       |           BI604010           |                               |
|                       |           BG708855           |                               |
|                       |           BX393816           |                               |
|                       |           BX361611           |                               |
|                       |           AW029110           |                               |
|                       |           CF265030           |                               |
|                       |           CA489615           |                               |
|                       |           BQ941697           |                               |
|                       |           BF309562           |                               |
|                       |           BM479531           |                               |
|                       |           BM923078           |                               |
|                       |           BQ879391           |                               |
|                       |           AJ272057           |                               |
|                       |           BE294818           |                               |
|                       |           BE294879           |                               |
|                       |             M78740           |                               |
|                       |           AU124407           |                               |
|                       |           AK001248           |                               |
|                       |           BP315135           |                               |
|                       |           BP217133           |                               |
|                       |           BM741924           |                               |
|                       |           BM152073           |                               |
|                       |           BM829157           |                               |
|                       |           BP208958           |                               |
|                       |           BX346247           |                               |
|                       |           BX386753           |                               |
|                       |           CR595671           |                               |
|                       |           BM549771           |                               |
|                       |           BX462614           |                               |
|                       |           BX416347           |                               |
|                       |           BI825634           |                               |
|                       |           BI258928           |                               |
|                       |           AL559808           |                               |
|                       |           BX393548           |                               |
|                       |           CR594371           |                               |
|                       |           CR598612           |                               |
|                       |           BU174562           |                               |
|                       |           BU858229           |                               |
|                       |           AA099795           |                               |
|                       |           BU856417           |                               |
|                       |           BI830303           |                               |
|                       |           BU177217           |                               |
|                       |           BI489972           |                               |
|                       |           BX439367           |                               |
|                       |           AL552913           |                               |
|                       |           CR623767           |                               |
|                       |           BM770381           |                               |
|                       |           CD557316           |                               |
|                       |           CN308821           |                               |
|                       |           BM799167           |                               |
|                       |           CN308822           |                               |
|                       |           CR597771           |                               |
|                       |           CR592650           |                               |
|                       |           CR457220           |                               |
|                       |           AF050158           |                               |
|                       |           CB306158           |                               |
|                       |           CK725002           |                               |
|                       |           BM992072           |                               |
|                       |           CD366533           |                               |
|                       |           BQ017484           |                               |
|                       |           BM683214           |                               |
|                       |           CA311170           |                               |
|                       |           AI885438           |                               |
|                       |           BM973091           |                               |
|                       |           CA944869           |                               |
|                       |           BM684078           |                               |
|                       |           BU733541           |                               |
|                       |           BX119269           |                               |
|                       |           CA421174           |                               |
|                       |           CA438817           |                               |
|                       |           AI369797           |                               |
|                       |           AA731775           |                               |
|                       |           AW016331           |                               |
|                       |           CB241495           |                               |
|                       |           AI223089           |                               |
|                       |           AW327585           |                               |
|                       |           BF516388           |                               |
|                       |           BU736168           |                               |
|                       |           BU624313           |                               |
|                       |           AW086438           |                               |
|                       |           AI360339           |                               |
|                       |           CD365487           |                               |
|                       |           AA102265           |                               |
|                       | *Seq. mapped in strand Minus*| *Seq. mapped in strand Minus* |
|                       |           AW470852           |          AK047551             |
|                       |           AI738880           |                               |
|                       |           CA314832           |                               |
|                       |           CB241883           |                               |
|                       |           BM781588           |                               |
|                       |           BM990981           |                               |
|                       |           CA313468           |                               |
+-----------------------+------------------------------+-------------------------------+
|      #   88           | *Seq. mapped in strand Plus* | *Seq. mapped in strand Plus*  |
|                       |           BI667392           |          AK087258             |
|                       |           BG715413           |          AK036571             |
|                       |           AK056210           |          AK081002             |
|                       |           BU844674           |          AK036282             |
|                       |           BC068992           |          AK081605             |
|                       |           BF206152           |          BC030861             |
|                       |           BF689824           |          BC069851             |
|                       |           BC007639           |                               |
|                       |           BI559616           |                               |
|                       |           BM006622           |                               |
|                       |           BQ897806           |                               |
|                       |           AI830881           |                               |
|                       |           AW024569           |                               |
|                       |           AW024282           |                               |
|                       |           AW170307           |                               |
|                       |           AW192605           |                               |
|                       |           AJ312332           |                               |
|                       |           AI971639           |                               |
|                       |           BE737036           |                               |
|                       |           BM560604           |                               |
|                       |           BE790704           |                               |
|                       |           BI117023           |                               |
|                       |           BE392066           |                               |
|                       |           CA489840           |                               |
|                       |           BQ674063           |                               |
|                       |           BQ879986           |                               |
|                       |           BQ674330           |                               |
|                       |           BC016845           |                               |
|                       |           BE791979           |                               |
|                       |           BE887885           |                               |
|                       |           BM710114           |                               |
|                       |           BQ638019           |                               |
|                       |           BE563369           |                               |
|                       |           BX283408           |                               |
|                       |           BG762094           |                               |
|                       |           BQ893956           |                               |
|                       |           BM017835           |                               |
|                       |           BM019867           |                               |
|                       |           AA416624           |                               |
|                       |           BM719083           |                               |
|                       |           BQ691888           |                               |
|                       |           BQ685882           |                               |
|                       |           BU157350           |                               |
|                       |           BQ897613           |                               |
|                       |           BQ685568           |                               |
|                       |           BQ687308           |                               |
|                       |           BQ691009           |                               |
|                       |           BQ880521           |                               |
|                       |           AW246095           |                               |
|                       |           BM695036           |                               |
|                       |           BU553580           |                               |
|                       |           BM713152           |                               |
|                       |           CV029724           |                               |
|                       |           CB161138           |                               |
|                       |           BF690210           |                               |
|                       |           AW183194           |                               |
|                       |           BM675021           |                               |
|                       |           BM691966           |                               |
|                       |           BU681506           |                               |
|                       |           BM045055           |                               |
|                       |             W25662           |                               |
|                       |           CF132111           |                               |
|                       |           BF791801           |                               |
|                       |           BF791732           |                               |
|                       |           BM666820           |                               |
|                       |           BM683053           |                               |
|                       |           BU728461           |                               |
|                       |           BU615868           |                               |
|                       |           BF515667           |                               |
|                       |           CB851811           |                               |
|                       |           BM671285           |                               |
|                       |           AI824949           |                               |
|                       |           AW249556           |                               |
|                       |             F30370           |                               |
|                       |           CN366752           |                               |
|                       |             R43862           |                               |
|                       |           AI271998           |                               |
|                       |           BF593091           |                               |
|                       |           AI554573           |                               |
|                       | *Seq. mapped in strand Minus*| *Seq. mapped in strand Minus* |
|                       |           AA764729           |          BC021438             |
|                       |           BU739852           |          AK051997             |
|                       |           AW016135           |                               |
|                       |           BM676050           |                               |
|                       |           BM969710           |                               |
|                       |           AW469972           |                               |
|                       |           BU736470           |                               |
|                       |             W31199           |                               |
|                       |           BF513460           |                               |
|                       |           BF514423           |                               |
|                       |           BQ636190           |                               |
|                       |           BQ028941           |                               |
|                       |           BU608739           |                               |
|                       |           BC009876           |                               |
|                       |           BM683858           |                               |
|                       |           BU155562           |                               |
|                       |           BU157452           |                               |
|                       |           BU161157           |                               |
|                       |           BU634208           |                               |
|                       |           BQ050538           |                               |
|                       |           CD242745           |                               |
|                       |           AK025907           |                               |
|                       |           BC002890           |                               |
|                       |           AK023011           |                               |
|                       |           BG685960           |                               |
|                       |           BG755033           |                               |
|                       |           BG697987           |                               |
|                       |           CB269500           |                               |
|                       |           BG757872           |                               |
|                       |           BQ051780           |                               |
|                       |           BG481293           |                               |
|                       |           BG482167           |                               |
|                       |           BE258947           |                               |
|                       |           CF128264           |                               |
|                       |           BE281057           |                               |
|                       |           BE279266           |                               |
|                       |           BE278993           |                               |
|                       |           BG005126           |                               |
|                       |           CB123919           |                               |
|                       |           BG025766           |                               |
|                       |           BQ957365           |                               |
|                       |           BE792136           |                               |
|                       |           BM021009           |                               |
|                       |           BF878933           |                               |
|                       |           BM044638           |                               |
|                       |           BX283794           |                               |
|                       |           BF818349           |                               |
|                       |           BM044704           |                               |
|                       |           CN371991           |                               |
|                       |           BG324431           |                               |
|                       |           BU194530           |                               |
|                       |           CN371990           |                               |
|                       |           AA421738           |                               |
|                       |           BQ695143           |                               |
|                       |           BP253095           |                               |
|                       |           BG982331           |                               |
|                       |           BM008237           |                               |
|                       |           BQ683166           |                               |
|                       |           BU168623           |                               |
|                       |           BQ052530           |                               |
|                       |           BE747480           |                               |
|                       |           BP376425           |                               |
|                       |           BG680140           |                               |
|                       |           CO247791           |                               |
|                       |           CD631718           |                               |
|                       |           BE743915           |                               |
|                       |           CN371986           |                               |
|                       |           BG748605           |                               |
|                       |           BE799910           |                               |
|                       |           AA320472           |                               |
|                       |           BG489002           |                               |
|                       |           BG766777           |                               |
|                       |           BG770104           |                               |
|                       |           BG766386           |                               |
|                       |           AU124084           |                               |
|                       |           BG768148           |                               |
|                       |           BE378414           |                               |
|                       |           BQ950163           |                               |
|                       |           BQ918981           |                               |
|                       |           BX343824           |                               |
|                       |           BU528569           |                               |
|                       |           BU538709           |                               |
|                       |           BM738605           |                               |
|                       |           BI258525           |                               |
|                       |           BG764225           |                               |
|                       |           BU557995           |                               |
|                       |           BG403277           |                               |
|                       |           BU944168           |                               |
|                       |           BM452480           |                               |
|                       |           BE408531           |                               |
|                       |           BQ922121           |                               |
|                       |           CN371992           |                               |
|                       |           CN371988           |                               |
|                       |           AA355671           |                               |
|                       |           BF892166           |                               |
|                       |           BM557090           |                               |
|                       |           AL546178           |                               |
|                       |           BG761239           |                               |
|                       |           BM470633           |                               |
|                       |           BE262225           |                               |
|                       |           BE206823           |                               |
|                       |           CN371993           |                               |
|                       |           AU129335           |                               |
|                       |           BG761502           |                               |
|                       |           CN371994           |                               |
|                       |           BE276512           |                               |
|                       |           BE894766           |                               |
|                       |           BE541125           |                               |
|                       |           BP357691           |                               |
|                       |           BF336729           |                               |
|                       |           BF326261           |                               |
|                       |           CD631706           |                               |
|                       |           CD631709           |                               |
|                       |           CD631710           |                               |
|                       |           CD631707           |                               |
|                       |           CD631711           |                               |
|                       |           BM150288           |                               |
|                       |           BM747925           |                               |
|                       |           BG468603           |                               |
|                       |           CD631708           |                               |
|                       |           CO248962           |                               |
|                       |           BM833746           |                               |
|                       |           CN371989           |                               |
|                       |           BP372346           |                               |
|                       |           BP281551           |                               |
|                       |           BI116870           |                               |
|                       |           BM149791           |                               |
|                       |           AL039569           |                               |
|                       |           AW672783           |                               |
|                       |           BF352657           |                               |
|                       |           BG489997           |                               |
|                       |           AU280109           |                               |
|                       |           AW859926           |                               |
|                       |           BG765393           |                               |
|                       |           BQ318244           |                               |
|                       |           BM834915           |                               |
|                       |           CD631716           |                               |
|                       |           CD631713           |                               |
|                       |           CD631714           |                               |
|                       |           CD631712           |                               |
|                       |           CD631715           |                               |
|                       |           CN371987           |                               |
+-----------------------+------------------------------+-------------------------------+
|      #   89           | *Seq. mapped in strand Plus* | *Seq. mapped in strand Plus*  |
|                       |           BC042096           |          BE988933             |
|                       |                              |          BY262720             |
|                       |                              |          BY718467             |
|                       | *Seq. mapped in strand Minus*| *Seq. mapped in strand Minus* |
|                       |           AK124181           |          BE980222             |
|                       |           BX648085           |          CF751225             |
|                       |           AY043282           |          CF550712             |
+-----------------------+------------------------------+-------------------------------+
|      #   90           | *Seq. mapped in strand Plus* | *Seq. mapped in strand Plus*  |
|                       |           AJ417067           |          AY008297             |
|                       |           BU568229           |          BC016092             |
|                       |           BI826832           |          AK015701             |
|                       |           BC039089           |          AK039097             |
|                       |           AF260257           |                               |
|                       |           CV030334           |                               |
|                       |           AF265666           |                               |
|                       |           AF227257           |                               |
|                       |           AF265667           |                               |
|                       |           BX571740           |                               |
|                       |           BI825694           |                               |
|                       |           BX491264           |                               |
|                       |             R93221           |                               |
|                       |           BU729630           |                               |
|                       |           BI829146           |                               |
|                       |             W26173           |                               |
|                       |           AA928161           |                               |
|                       |           AI015922           |                               |
|                       |           AA782963           |                               |
|                       |           AI964059           |                               |
|                       | *Seq. mapped in strand Minus*| *Seq. mapped in strand Minus* |
|                       |           BU678755           |          AF323667             |
|                       |           CB241108           |          BC012433             |
|                       |           BQ015193           |                               |
+-----------------------+------------------------------+-------------------------------+
|      #   91           | *Seq. mapped in strand Plus* | *Seq. mapped in strand Plus*  |
|                       |           CK299187           |          BY602067             |
|                       |                              |          BF468345             |
|                       |                              |          BY321174             |
|                       |                              |          BY253615             |
|                       | *Seq. mapped in strand Minus*| *Seq. mapped in strand Minus* |
|                       |           AJ420478           |          BE992844             |
|                       |           AA622369           |          AW048427             |
|                       |           BF509060           |          BX520627             |
|                       |           BX092448           |          AF277093             |
+-----------------------+------------------------------+-------------------------------+
|      #   92           | *Seq. mapped in strand Plus* | *Seq. mapped in strand Plus*  |
|                       |           BU507402           |          AK031925             |
|                       |           AV751543           |          AI852314             |
|                       |           BF946189           |          BU755777             |
|                       |           AA411938           |          CK374078             |
|                       |           BX281435           |          CK373944             |
|                       |           AA330314           |          BQ176488             |
|                       |           AA330507           |          CD776209             |
|                       |           AW961560           |          BE630023             |
|                       |           BE086819           |          BE943655             |
|                       |           AW949042           |          AW488424             |
|                       |           BG150381           |          BE630213             |
|                       |           BE672446           |          BQ417581             |
|                       |           AI400489           |          AW493885             |
|                       |           AW451923           |          AW493759             |
|                       |           BF089682           |                               |
|                       |           AI580981           |                               |
|                       |           AI040914           |                               |
|                       |           BX484532           |                               |
|                       |           BX509386           |                               |
|                       |           CB988831           |                               |
|                       |           CB150870           |                               |
|                       |           AL708770           |                               |
|                       |           BX954267           |                               |
|                       |           AL709337           |                               |
|                       |           BM921339           |                               |
|                       |           BM172426           |                               |
|                       |           BU902171           |                               |
|                       |           CN428607           |                               |
|                       |           BU509290           |                               |
|                       |           CA396007           |                               |
|                       |           BQ068988           |                               |
|                       |           AL582999           |                               |
|                       |           CR606592           |                               |
|                       |           BE278384           |                               |
|                       |           CN428606           |                               |
|                       |           AA305920           |                               |
|                       |           AW962946           |                               |
|                       |           AF327345           |                               |
|                       |           BQ435226           |                               |
|                       |           CN428608           |                               |
|                       |           BU932136           |                               |
|                       |           BF376309           |                               |
|                       |           BF376303           |                               |
|                       |           BF376304           |                               |
|                       |           BE883592           |                               |
|                       |           CB146219           |                               |
|                       |           CN276252           |                               |
|                       |           BE694627           |                               |
|                       |           BG696039           |                               |
|                       |           AW205637           |                               |
|                       |             W87465           |                               |
|                       |           AW205114           |                               |
|                       |           BX370685           |                               |
|                       |           AA315855           |                               |
|                       |           AW961152           |                               |
|                       |           BM470719           |                               |
|                       |           BE705965           |                               |
|                       |           BU536352           |                               |
|                       |           BX426463           |                               |
|                       |           CN352647           |                               |
|                       |           BM471450           |                               |
|                       |           AI580780           |                               |
|                       |             D17076           |                               |
|                       |           BM973968           |                               |
|                       |           CA441179           |                               |
|                       |           BU686714           |                               |
|                       |           BU683224           |                               |
|                       |           CB150188           |                               |
|                       |           BM989216           |                               |
|                       |           AW169079           |                               |
|                       |           AI741276           |                               |
|                       |           AI276993           |                               |
|                       |           AA765128           |                               |
|                       |             D17249           |                               |
|                       |           AW294057           |                               |
|                       | *Seq. mapped in strand Minus*| *Seq. mapped in strand Minus* |
|                       |           BE674437           |          AW456580             |
|                       |           BE069888           |                               |
|                       |           CB242320           |                               |
+-----------------------+------------------------------+-------------------------------+
|      #   93           | *Seq. mapped in strand Plus* | *Seq. mapped in strand Plus*  |
|                       |             U87864           |          BC058386             |
|                       |           BG700260           |          AK046382             |
|                       |           BC026336           |          AF400063             |
|                       |           CB998011           |          AF401228             |
|                       |           AF029729           |          AK078529             |
|                       |             R90763           |                               |
|                       |           CA843851           |                               |
|                       |           CB992271           |                               |
|                       |           BQ286290           |                               |
|                       |           CB992205           |                               |
|                       |           CB991009           |                               |
|                       |           CB992681           |                               |
|                       |           BX098217           |                               |
|                       |           BG656185           |                               |
|                       |           CA772780           |                               |
|                       |           CA948745           |                               |
|                       |           BI712632           |                               |
|                       |           CA941769           |                               |
|                       |           CA771909           |                               |
|                       |           CB068968           |                               |
|                       |             H82634           |                               |
|                       |           BU950983           |                               |
|                       |           CA774789           |                               |
|                       |           BQ269642           |                               |
|                       |           BI712399           |                               |
|                       |           CA941491           |                               |
|                       |           CA948476           |                               |
|                       |           BU952430           |                               |
|                       |           BG656496           |                               |
|                       |           CA775047           |                               |
|                       |           AI830365           |                               |
|                       |           AA782125           |                               |
|                       |           AA772093           |                               |
|                       |           BF591302           |                               |
|                       |           AW771627           |                               |
|                       |           BM724707           |                               |
|                       |           BM930417           |                               |
|                       |           AI220894           |                               |
|                       |             H82635           |                               |
|                       |           BF002588           |                               |
|                       |           BM505387           |                               |
|                       |           AW016567           |                               |
|                       |           AI219235           |                               |
|                       |           AI149602           |                               |
|                       |           AW449663           |                               |
|                       |           BM855108           |                               |
|                       |           CB127566           |                               |
|                       |           BU570619           |                               |
|                       |           AI096380           |                               |
|                       |           AA612873           |                               |
|                       |           AI025302           |                               |
|                       |           AI739022           |                               |
|                       |           AA887940           |                               |
|                       |           AI139814           |                               |
|                       |           BQ638204           |                               |
|                       |           AI660543           |                               |
|                       |             N89880           |                               |
|                       |           BQ287987           |                               |
|                       |           CD618184           |                               |
|                       |           CD618181           |                               |
|                       |           CD618182           |                               |
|                       |           CD618183           |                               |
|                       |           AW804455           |                               |
|                       |           BM712445           |                               |
|                       | *Seq. mapped in strand Minus*| *Seq. mapped in strand Minus* |
|                       |           AA412181           |          AK052522             |
|                       |           AA399019           |          AK044464             |
|                       |           AA621058           |          AK032915             |
|                       |           BX095052           |                               |
|                       |           AA758841           |                               |
|                       |           CA312912           |                               |
|                       |           BU617262           |                               |
|                       |           CA428118           |                               |
|                       |           BM678909           |                               |
|                       |           BU739257           |                               |
+-----------------------+------------------------------+-------------------------------+
|      #   94           | *Seq. mapped in strand Plus* | *Seq. mapped in strand Plus*  |
|                       |           BC047465           |          BC049541             |
|                       |           AK098224           |          AK034636             |
|                       |           BC021985           |          AF109377             |
|                       |           AB037802           |          BC063056             |
|                       |           AK025633           |          AK031510             |
|                       |           CR603633           |          AK019727             |
|                       |           AL359611           |                               |
|                       | *Seq. mapped in strand Minus*| *Seq. mapped in strand Minus* |
|                       |           BC011054           |          BC025149             |
|                       |           BC025238           |          BC056429             |
|                       |           AK027681           |          AK009763             |
|                       |           AK074755           |          AK087540             |
|                       |           BX537858           |          BC060965             |
|                       |           BX648968           |          AK017659             |
|                       |           CR624411           |          AK050675             |
|                       |             U92989           |          AK028386             |
+-----------------------+------------------------------+-------------------------------+
|      #   95           | *Seq. mapped in strand Plus* | *Seq. mapped in strand Plus*  |
|                       |           AK097052           |          BY130665             |
|                       |           AL136564           |          BY127695             |
|                       |           AK056443           |          BI788846             |
|                       |           BC017191           |          CF579036             |
|                       |           AJ305226           |          BB856386             |
|                       |           AJ305227           |          BB864919             |
|                       |           CR456482           |          BB867866             |
|                       |           CR594826           |          BY274834             |
|                       |                              |          CD549356             |
|                       |                              |          BY219861             |
|                       |                              |          BY212001             |
|                       |                              |          BY286949             |
|                       |                              |          BY739237             |
|                       |                              |          CA316496             |
|                       |                              |          AL362769             |
|                       |                              |          BY287717             |
|                       |                              |          BB629422             |
|                       |                              |          BB611104             |
|                       |                              |          AK036510             |
|                       |                              |          CD551024             |
|                       |                              |          CA877513             |
|                       |                              |          BB613576             |
|                       |                              |          AK029115             |
|                       |                              |          BY267076             |
|                       |                              |          BY271561             |
|                       |                              |          BY272098             |
|                       |                              |          BY211468             |
|                       |                              |          BY273322             |
|                       |                              |          CF168139             |
|                       |                              |          BG292756             |
|                       |                              |          BY242415             |
|                       |                              |          BB856620             |
|                       |                              |          BY348849             |
|                       |                              |          BI556750             |
|                       |                              |          BB630434             |
|                       |                              |          BY736042             |
|                       |                              |          CN700386             |
|                       |                              |          BY076421             |
|                       |                              |          CF897257             |
|                       |                              |          CF168137             |
|                       |                              |          AY237002             |
|                       |                              |          AY237000             |
|                       |                              |          AY237001             |
|                       |                              |          CA317280             |
|                       |                              |          CA320869             |
|                       |                              |          CF166931             |
|                       |                              |          CB249375             |
|                       |                              |          CN690619             |
|                       |                              |          CN690107             |
|                       |                              |          CN689292             |
|                       |                              |          BI666326             |
|                       |                              |          BC023933             |
|                       |                              |          CK391164             |
|                       |                              |          CK392243             |
|                       |                              |          CF750782             |
|                       |                              |          BY283070             |
|                       |                              |          BY038792             |
|                       |                              |          BY292013             |
|                       |                              |          BY274377             |
|                       |                              |          BY295567             |
|                       |                              |          BY271059             |
|                       |                              |          BY288988             |
|                       |                              |          CA555603             |
|                       |                              |          CN684960             |
|                       |                              |          CN685195             |
|                       |                              |          BC030864             |
|                       |                              |          CF898812             |
|                       |                              |          BF532427             |
|                       |                              |          BB656235             |
|                       |                              |          CD544398             |
|                       |                              |          CN703310             |
|                       |                              |          CN682861             |
|                       |                              |          CN682295             |
|                       |                              |          BI737248             |
|                       |                              |          BQ944124             |
|                       |                              |          BB858168             |
|                       |                              |          BG100661             |
|                       |                              |          BB854157             |
|                       |                              |          CK390069             |
|                       |                              |          CK392953             |
|                       |                              |          CD564294             |
|                       |                              |          BM934789             |
|                       |                              |          BG808475             |
|                       |                              |          CK337560             |
|                       |                              |          CF895084             |
|                       |                              |          CF894034             |
|                       |                              |          CF735404             |
|                       |                              |          CF893861             |
|                       |                              |          BE649981             |
|                       |                              |          CA564726             |
|                       |                              |          CN693607             |
|                       |                              |          BY272476             |
|                       |                              |          CN722356             |
|                       |                              |          CN718280             |
|                       |                              |          BQ572663             |
|                       |                              |          BQ573608             |
|                       |                              |          CF165579             |
|                       |                              |          CF162283             |
|                       |                              |          CD544427             |
|                       |                              |          CF748561             |
|                       |                              |          CD560323             |
|                       |                              |          BY756418             |
|                       |                              |          BY357184             |
|                       |                              |          CA750138             |
|                       |                              |          BQ887252             |
|                       |                              |          BM949359             |
|                       |                              |          BI333009             |
|                       |                              |          BC023676             |
|                       |                              |          BI691191             |
|                       |                              |          BI688092             |
|                       |                              |          BB730761             |
|                       |                              |          BU922102             |
|                       |                              |          BY395971             |
|                       |                              |          AW489725             |
|                       |                              |          AA066436             |
|                       |                              |          AI552858             |
|                       |                              |            D76919             |
|                       |                              |          BM947392             |
|                       |                              |          AV162961             |
|                       |                              |          BY353967             |
|                       |                              |          BQ950287             |
|                       |                              |          BY759543             |
|                       |                              |          BM948756             |
|                       |                              |          CN679832             |
|                       |                              |          BE862793             |
|                       |                              |          BU504687             |
|                       | *Seq. mapped in strand Minus*| *Seq. mapped in strand Minus* |
|                       |           AK057177           |          CK788908             |
|                       |           BC048421           |          CF551388             |
|                       |           BC012882           |          BB524020             |
|                       |           BC019839           |          AW121089             |
|                       |           BC040188           |          BY559159             |
|                       |           BC068590           |          BF462492             |
|                       |                              |          AI413672             |
|                       |                              |          BQ033455             |
|                       |                              |          AI430098             |
|                       |                              |            W58996             |
|                       |                              |          BB642130             |
|                       |                              |          BB662762             |
|                       |                              |                               |
|                       |                              |                               |
|                       |                              |                               |
|                       |                              |                               |
|                       |                              |                               |
|                       |                              |                               |
|                       |                              |                               |
|                       |                              |                               |
|                       |                              |                               |
|                       |                              |                               |
|                       |                              |                               |
|                       |                              |                               |
|                       |                              |                               |
|                       |                              |                               |
|                       |                              |                               |
|                       |                              |                               |
|                       |                              |                               |
|                       |                              |                               |
|                       |                              |                               |
|                       |                              |                               |
|                       |                              |                               |
|                       |                              |                               |
|                       |                              |                               |
|                       |                              |                               |
|                       |                              |                               |
|                       |                              |                               |
|                       |                              |                               |
|                       |                              |                               |
|                       |                              |                               |
|                       |                              |                               |
|                       |                              |                               |
|                       |                              |                               |
|                       |                              |                               |
|                       |                              |                               |
|                       |                              |                               |
|                       |                              |                               |
|                       |                              |                               |
|                       |                              |                               |
|                       |                              |                               |
|                       |                              |                               |
|                       |                              |                               |
|                       |                              |                               |
|                       |                              |                               |
|                       |                              |                               |
|                       |                              |                               |
|                       |                              |                               |
|                       |                              |                               |
|                       |                              |                               |
|                       |                              |                               |
|                       |                              |                               |
|                       |                              |                               |
|                       |                              |                               |
|                       |                              |                               |
|                       |                              |                               |
|                       |                              |                               |
|                       |                              |                               |
|                       |                              |                               |
|                       |                              |                               |
|                       |                              |                               |
|                       |                              |                               |
|                       |                              |                               |
|                       |                              |                               |
|                       |                              |                               |
|                       |                              |                               |
|                       |                              |                               |
|                       |                              |                               |
|                       |                              |                               |
|                       |                              |                               |
|                       |                              |                               |
|                       |                              |                               |
|                       |                              |                               |
|                       |                              |                               |
|                       |                              |                               |
|                       |                              |                               |
|                       |                              |                               |
|                       |                              |                               |
|                       |                              |                               |
|                       |                              |                               |
|                       |                              |                               |
|                       |                              |                               |
|                       |                              |                               |
|                       |                              |                               |
|                       |                              |                               |
|                       |                              |                               |
|                       |                              |                               |
|                       |                              |                               |
|                       |                              |                               |
|                       |                              |                               |
|                       |                              |                               |
|                       |                              |                               |
|                       |                              |                               |
|                       |                              |                               |
|                       |                              |                               |
|                       |                              |                               |
|                       |                              |                               |
|                       |                              |                               |
|                       |                              |                               |
|                       |                              |                               |
|                       |                              |                               |
|                       |                              |                               |
|                       |                              |                               |
|                       |                              |                               |
|                       |                              |                               |
|                       |                              |                               |
|                       |                              |                               |
|                       |                              |                               |
|                       |                              |                               |
|                       |                              |                               |
|                       |                              |                               |
|                       |                              |                               |
|                       |                              |                               |
|                       |                              |                               |
|                       |                              |                               |
|                       |                              |                               |
+-----------------------+------------------------------+-------------------------------+
|      #   96           | *Seq. mapped in strand Plus* | *Seq. mapped in strand Plus*  |
|                       |           AL133022           |          BY715517             |
|                       |                              |          BB615572             |
|                       |                              |          AV268082             |
|                       |                              |          BE987944             |
|                       | *Seq. mapped in strand Minus*| *Seq. mapped in strand Minus* |
|                       |           BC034928           |          AI846593             |
|                       |                              |          AK031056             |
|                       |                              |          BY525128             |
|                       |                              |          BY763843             |
+-----------------------+------------------------------+-------------------------------+
|      #   97           | *Seq. mapped in strand Plus* | *Seq. mapped in strand Plus*  |
|                       |           BQ438482           |          BG922182             |
|                       |           BU852261           |                               |
|                       |           AK090537           |                               |
|                       |           CN411869           |                               |
|                       |           BC067085           |                               |
|                       |           AB014555           |                               |
|                       |           CB177217           |                               |
|                       |           BM706487           |                               |
|                       |           AI910277           |                               |
|                       |           AW504715           |                               |
|                       |           BF853683           |                               |
|                       |           AB013384           |                               |
|                       |           BG569566           |                               |
|                       |           BF347655           |                               |
|                       |           AW504218           |                               |
|                       |           BQ440520           |                               |
|                       |           BF527206           |                               |
|                       |           BF530760           |                               |
|                       |           BG819121           |                               |
|                       |           BI772606           |                               |
|                       |           BE700123           |                               |
|                       |           BG818838           |                               |
|                       |           BM673310           |                               |
|                       |           AL120508           |                               |
|                       |           AL120410           |                               |
|                       |           CD580482           |                               |
|                       |           BQ719921           |                               |
|                       |           BG819953           |                               |
|                       |           CA488778           |                               |
|                       |           BI919025           |                               |
|                       |           BM926946           |                               |
|                       |           CD580312           |                               |
|                       |           BE244254           |                               |
|                       |           BG757099           |                               |
|                       |           AW402592           |                               |
|                       |           BX487084           |                               |
|                       |           BI013886           |                               |
|                       |           AU143644           |                               |
|                       |           AK024062           |                               |
|                       |           BF345440           |                               |
|                       |           BM152565           |                               |
|                       |           BF822632           |                               |
|                       |           BG910792           |                               |
|                       |           BF347401           |                               |
|                       |           BI160653           |                               |
|                       |           CA487289           |                               |
|                       |           BG913434           |                               |
|                       |           AW247539           |                               |
|                       |           CD580490           |                               |
|                       |           CB216375           |                               |
|                       |           BF855445           |                               |
|                       |           BF530165           |                               |
|                       |           BG754124           |                               |
|                       |           BG753892           |                               |
|                       |           BF128690           |                               |
|                       |           BF348082           |                               |
|                       |           BI333976           |                               |
|                       |           AI272228           |                               |
|                       |           BI917453           |                               |
|                       |           CD672992           |                               |
|                       |             T33837           |                               |
|                       |             T08283           |                               |
|                       |           BU539868           |                               |
|                       |           BG820246           |                               |
|                       |           BF337218           |                               |
|                       |           BE696603           |                               |
|                       |           CB269212           |                               |
|                       |           CF457389           |                               |
|                       |           BU558073           |                               |
|                       |           AA351038           |                               |
|                       |           BM714747           |                               |
|                       |           BQ711096           |                               |
|                       |           BF929980           |                               |
|                       |           BI913204           |                               |
|                       |           AV745738           |                               |
|                       |           AA627184           |                               |
|                       |             N31509           |                               |
|                       |           BG187735           |                               |
|                       | *Seq. mapped in strand Minus*| *Seq. mapped in strand Minus* |
|                       |           AW450311           |          CF425342             |
+-----------------------+------------------------------+-------------------------------+
|      #   98           | *Seq. mapped in strand Plus* | *Seq. mapped in strand Plus*  |
|                       |           BM726878           |          AK053602             |
|                       |           BU741166           |                               |
|                       |           BM991338           |                               |
|                       | *Seq. mapped in strand Minus*| *Seq. mapped in strand Minus* |
|                       |           AF086440           |          AK049304             |
|                       |           BF980072           |          AK041758             |
|                       |           BF979992           |          AK046055             |
|                       |           AA447552           |          AK013790             |
|                       |           AK128795           |                               |
|                       |           BM552659           |                               |
|                       |           AA490830           |                               |
|                       |           BU632310           |                               |
|                       |           BM684547           |                               |
|                       |           AL040358           |                               |
|                       |           BU740446           |                               |
|                       |           BQ009083           |                               |
|                       |           AK096696           |                               |
|                       |           AF097645           |                               |
|                       |           BC039829           |                               |
|                       |           AL833524           |                               |
|                       |           BU581509           |                               |
|                       |           BU561494           |                               |
|                       |           BF031180           |                               |
|                       |           AA521423           |                               |
|                       |           BF512198           |                               |
|                       |           AK074946           |                               |
|                       |           CK002848           |                               |
|                       |           CD678653           |                               |
|                       |           BM931082           |                               |
|                       |           BF681409           |                               |
|                       |           BU854515           |                               |
|                       |           AI015911           |                               |
|                       |           AI921056           |                               |
|                       |           AL117626           |                               |
|                       |           BC040581           |                               |
|                       |           AF141326           |                               |
|                       |           BQ441304           |                               |
|                       |           BP369596           |                               |
|                       |           BX280278           |                               |
|                       |           AK094036           |                               |
|                       |           CN432235           |                               |
|                       |           BU621361           |                               |
|                       |           BM721372           |                               |
|                       |           BE089791           |                               |
|                       |           AV657377           |                               |
|                       |           AV657435           |                               |
|                       |           AV657469           |                               |
|                       |           AA448534           |                               |
|                       |           AW959308           |                               |
|                       |           BU852106           |                               |
|                       |           CN432233           |                               |
|                       |           BF574440           |                               |
|                       |           CB134637           |                               |
|                       |           BI827749           |                               |
|                       |           BC013358           |                               |
|                       |           AA405143           |                               |
|                       |           BU567564           |                               |
|                       |           BG435317           |                               |
|                       |           BF247255           |                               |
|                       |           AI129894           |                               |
|                       |           CA407016           |                               |
|                       |           BF858238           |                               |
|                       |           CN432241           |                               |
|                       |           BP367173           |                               |
|                       |           AA282364           |                               |
|                       |           CF780804           |                               |
|                       |           CF142948           |                               |
|                       |           CF143754           |                               |
|                       |           BE830619           |                               |
|                       |           BM544948           |                               |
|                       |           BE928115           |                               |
|                       |           BU754286           |                               |
|                       |           AA133485           |                               |
|                       |           BG723467           |                               |
|                       |           CD359043           |                               |
|                       |           BE178651           |                               |
|                       |           BI561247           |                               |
|                       |           BE694328           |                               |
|                       |           AL596988           |                               |
|                       |           BM787810           |                               |
|                       |           BQ421103           |                               |
|                       |           BE159231           |                               |
|                       |           BP320536           |                               |
|                       |           BI462405           |                               |
|                       |           BF979707           |                               |
|                       |           BF979768           |                               |
|                       |           AL528205           |                               |
|                       |           BP356369           |                               |
|                       |           CA389942           |                               |
|                       |           BX644282           |                               |
|                       |           AI806186           |                               |
|                       |           BE972640           |                               |
|                       |           AL706410           |                               |
|                       |           AL514686           |                               |
|                       |           BE159232           |                               |
|                       |           AL706557           |                               |
|                       |           BG723973           |                               |
|                       |           AW129631           |                               |
|                       |           AW129661           |                               |
|                       |           BC032386           |                               |
|                       |           BC018725           |                               |
|                       |           BX110629           |                               |
|                       |           CA436828           |                               |
|                       |           CR607963           |                               |
|                       |           AW248502           |                               |
|                       |             D61831           |                               |
|                       |           BX381204           |                               |
|                       |           AW502252           |                               |
|                       |           CN432240           |                               |
|                       |           BI022032           |                               |
|                       |           BI461163           |                               |
|                       |           AW504624           |                               |
+-----------------------+------------------------------+-------------------------------+
|      #   99           | *Seq. mapped in strand Plus* | *Seq. mapped in strand Plus*  |
|                       |             H44183           |          AK038780             |
|                       |           BP332025           |          AK012070             |
|                       |           BP332679           |          AK029764             |
|                       |           BP275109           |          AK014879             |
|                       |           BP332724           |          AK029752             |
|                       |           BP274898           |          BC013512             |
|                       |           BG400756           |          AF474247             |
|                       |           BG400857           |          AF220100             |
|                       |           BM083516           |                               |
|                       |           BP274483           |                               |
|                       |           BX494973           |                               |
|                       |           BG431149           |                               |
|                       |           AU100469           |                               |
|                       |           BG425738           |                               |
|                       |           BG428997           |                               |
|                       |           BG428476           |                               |
|                       |           BF105220           |                               |
|                       |           BP276683           |                               |
|                       |           BG429147           |                               |
|                       |           BG425819           |                               |
|                       |           BG422947           |                               |
|                       |           AF012281           |                               |
|                       |           BF105001           |                               |
|                       |           BC006496           |                               |
|                       |           BC006518           |                               |
|                       |           BE249846           |                               |
|                       |           BG325054           |                               |
|                       |           CV026505           |                               |
|                       |           BQ307311           |                               |
|                       |           BQ307261           |                               |
|                       |           BG424780           |                               |
|                       |           BG740256           |                               |
|                       |           BG431510           |                               |
|                       |           BG428193           |                               |
|                       |           BG433671           |                               |
|                       |           BG431128           |                               |
|                       |           BG428754           |                               |
|                       |           BE090292           |                               |
|                       |           BX096667           |                               |
|                       |           BG402026           |                               |
|                       |             T84140           |                               |
|                       |           BG697921           |                               |
|                       |           BG433341           |                               |
|                       |             T87024           |                               |
|                       |           BG398665           |                               |
|                       |           CN268210           |                               |
|                       |           AW003503           |                               |
|                       |           AV683662           |                               |
|                       |           AV685910           |                               |
|                       |           AV687017           |                               |
|                       |           AV688896           |                               |
|                       |           AV693509           |                               |
|                       |           AV697585           |                               |
|                       |           AV698954           |                               |
|                       |             T97830           |                               |
|                       |             R98715           |                               |
|                       |           AI953372           |                               |
|                       |           BG210907           |                               |
|                       |           BG217271           |                               |
|                       |           AV708817           |                               |
|                       |           AI765506           |                               |
|                       |           CK300599           |                               |
|                       |           BQ187272           |                               |
|                       |           BP275377           |                               |
|                       |           BX477679           |                               |
|                       |           BM790628           |                               |
|                       |           AW205432           |                               |
|                       |           AI225044           |                               |
|                       |           AA985444           |                               |
|                       |           AA887455           |                               |
|                       |           BX497037           |                               |
|                       | *Seq. mapped in strand Minus*| *Seq. mapped in strand Minus* |
|                       |           BI194571           |          AK016736             |
|                       |           BG202451           |          AK015364             |
|                       |           AA770013           |          AK006978             |
|                       |           AI579900           |          AK034931             |
|                       |           BM785388           |          AK016787             |
|                       |           AI659248           |          AK009734             |
|                       |           BG150319           |          BC010729             |
|                       |           AW078861           |          AK002937             |
|                       |           AA492346           |          AK020347             |
|                       |           AI702894           |                               |
|                       |           AA137242           |                               |
|                       |           AI761236           |                               |
|                       |           BI494234           |                               |
|                       |           AW207336           |                               |
|                       |           AI138467           |                               |
|                       |           AI138222           |                               |
|                       |             W73805           |                               |
|                       |           AF151035           |                               |
|                       |           AK001354           |                               |
|                       |           BQ278886           |                               |
|                       |           CR590313           |                               |
|                       |           AL565411           |                               |
|                       |           CR625868           |                               |
|                       |           CR620581           |                               |
|                       |           BX117855           |                               |
|                       |           BM825200           |                               |
|                       |           BX360846           |                               |
|                       |           BG519760           |                               |
|                       |           BF316302           |                               |
|                       |           CR742333           |                               |
|                       |           BG519674           |                               |
|                       |           BG675135           |                               |
|                       |           BE541691           |                               |
|                       |           CA488677           |                               |
|                       |           BM712276           |                               |
|                       |           BP255269           |                               |
|                       |           BI196101           |                               |
|                       |             R88612           |                               |
|                       |           BE672682           |                               |
|                       |             T86232           |                               |
|                       |           BF800738           |                               |
|                       |           BG939127           |                               |
|                       |           BP246340           |                               |
|                       |           CD674946           |                               |
|                       |             T93856           |                               |
|                       |           CD102528           |                               |
|                       |           BE833908           |                               |
|                       |           AA136462           |                               |
|                       |           BX424469           |                               |
|                       |           BG110085           |                               |
|                       |           BQ212944           |                               |
|                       |           BG427501           |                               |
|                       |           CB133881           |                               |
|                       |           AL514046           |                               |
|                       |           BP233777           |                               |
|                       |           BF979090           |                               |
|                       |           AA090641           |                               |
|                       |           BP291246           |                               |
|                       |           AW965382           |                               |
|                       |           AA333799           |                               |
|                       |           BE077977           |                               |
|                       |           BP343431           |                               |
|                       |           BP343602           |                               |
|                       |           AA722367           |                               |
+-----------------------+------------------------------+-------------------------------+
|      #  100           | *Seq. mapped in strand Plus* | *Seq. mapped in strand Plus*  |
|                       |           BG772246           |          BY740327             |
|                       |           BP251742           |          BY305352             |
|                       |           AL542017           |          BY152675             |
|                       |           BQ435549           |          BY152961             |
|                       |           BP289719           |          BY306047             |
|                       |           BP228660           |          BB854542             |
|                       |           BG721440           |          AK012088             |
|                       |           BX472576           |          BE334686             |
|                       |           BG772660           |          BF015616             |
|                       |           BI833416           |          BQ570086             |
|                       |           BC041692           |          BQ918084             |
|                       |           BP243897           |          CK788120             |
|                       |           BG700865           |          BU511980             |
|                       |           BI596596           |          BU841325             |
|                       |           BI460022           |          CK032636             |
|                       |           BF966111           |          AW911697             |
|                       |           AB061712           |          BQ256753             |
|                       |           BI822722           |          BU708232             |
|                       |           AL545222           |          BQ564379             |
|                       |           CN283526           |          BU511324             |
|                       |           CN283516           |          CK790368             |
|                       |           BQ549234           |          BY243199             |
|                       |           BQ950592           |          BY274710             |
|                       |           BQ437321           |          AY316161             |
|                       |           AL519262           |          BG977100             |
|                       |           CN283524           |          BB616382             |
|                       |           BP242634           |          AK029972             |
|                       |           CN283517           |          CN529747             |
|                       |           CN283519           |          CA893976             |
|                       |           AI125146           |          CN715575             |
|                       |           BX486559           |          CN716085             |
|                       |           AL710872           |          CN719664             |
|                       |           CD657197           |          CN720355             |
|                       |           BG108144           |          BC031529             |
|                       |             D89053           |          CK391342             |
|                       |           BX494841           |          BI737752             |
|                       |           AU131643           |          CA316610             |
|                       |           AK023191           |          CK638179             |
|                       |           BP268302           |          AA671278             |
|                       |           BP267819           |          CF749090             |
|                       |           BP328166           |          CA887235             |
|                       |           CD657223           |          BU559244             |
|                       |           BI772350           |          BG917800             |
|                       |           AU128895           |          CN530942             |
|                       |           BP288979           |          CB035342             |
|                       |           BQ644588           |          CK331558             |
|                       |           BI755150           |          BM461274             |
|                       |           BP209516           |          CN532262             |
|                       |           AU130030           |          BI691380             |
|                       |           BP242958           |          AI503321             |
|                       |           AA563870           |          CD353882             |
|                       |           CB144395           |          CF724716             |
|                       |           CB149552           |          CN696587             |
|                       |           AU130119           |          BQ568217             |
|                       |           AI989295           |          BE571900             |
|                       |           BF093911           |          CN531983             |
|                       |           BP872155           |          BQ929636             |
|                       |           BE383039           |          BF468826             |
|                       |           AL558879           |          BF100359             |
|                       |           CR608706           |          CF748099             |
|                       |           BG697302           |          BQ568502             |
|                       |           AL119851           |          CD808296             |
|                       |           BP302945           |          AA450958             |
|                       |           BE081110           |          BM197991             |
|                       |           AU129303           |          BF582498             |
|                       |           AK001471           |          BG245116             |
|                       |           BP238205           |          BE372240             |
|                       |           AU128429           |          BG919989             |
|                       |           BP243574           |          CB057974             |
|                       |           BG823789           |          BG242028             |
|                       |           CF145727           |          BF168898             |
|                       |           CN283518           |          AA612205             |
|                       |           BE250001           |          CA527753             |
|                       |           CB158273           |          BF162620             |
|                       |           BM472141           |          BF579387             |
|                       |           CA487833           |            W35438             |
|                       |           BE869032           |          AA691833             |
|                       |           BG724040           |          CA556946             |
|                       |           BP370565           |          BQ560187             |
|                       |           BF149140           |          BQ900688             |
|                       |           AU125259           |          CN531369             |
|                       |           BE168968           |          AI116037             |
|                       |           AW609208           |          CB844509             |
|                       |           BG290959           |          BG293523             |
|                       |           BE568021           |          BF722984             |
|                       |           CA487297           |          BF607091             |
|                       |           CD643360           |          BG863879             |
|                       |           BF240819           |          AA967769             |
|                       |           CN283521           |          AA414751             |
|                       |           CN283520           |          AA870092             |
|                       |           BF240612           |          AA636796             |
|                       |           AL135191           |          BX526286             |
|                       |           BG164738           |          AA529452             |
|                       |           CF139208           |          BM950943             |
|                       |           BM906711           |          BQ569986             |
|                       |           BP260093           |          BE687116             |
|                       |           CN283523           |          AA265214             |
|                       |           BU179791           |          BI104038             |
|                       |           AV726023           |          BY760630             |
|                       |           BG207998           |          AA616527             |
|                       |           BI821505           |          BG147425             |
|                       |           AA321690           |          AA184190             |
|                       |           BP289069           |          BF134387             |
|                       |           CA454816           |          BU782264             |
|                       |           BG676821           |          CA529004             |
|                       |           BX327493           |          BE956625             |
|                       |           BE866791           |          AW122661             |
|                       |           BG285442           |          AK046000             |
|                       |           AL542016           |          BE982917             |
|                       |           BF149141           |          AI843601             |
|                       |           AL519261           |          BF461629             |
|                       |           BI197947           |          AI845756             |
|                       |           AI133576           |          AI846905             |
|                       |           AF116690           |                               |
|                       |           CB116385           |                               |
|                       |           BX091864           |                               |
|                       |           BE568120           |                               |
|                       |           BP265410           |                               |
|                       |           CK001938           |                               |
|                       |           AA190984           |                               |
|                       |           BM014993           |                               |
|                       |           BM979616           |                               |
|                       |           BP378094           |                               |
|                       |           BE931721           |                               |
|                       |           BG330703           |                               |
|                       |           BG331623           |                               |
|                       |           BC012066           |                               |
|                       |           BC032144           |                               |
|                       |           BM754447           |                               |
|                       |           BG111778           |                               |
|                       |           AA148850           |                               |
|                       |           AU139651           |                               |
|                       |           BX095257           |                               |
|                       |           CA447387           |                               |
|                       |             W31074           |                               |
|                       |           AL580602           |                               |
|                       |           CN283522           |                               |
|                       |           BG618800           |                               |
|                       |           AI206454           |                               |
|                       |           BF211543           |                               |
|                       |           AV750767           |                               |
|                       |           AA191465           |                               |
|                       |           AW603192           |                               |
|                       |           AA812279           |                               |
|                       |           BG699720           |                               |
|                       |             H29214           |                               |
|                       |             W20143           |                               |
|                       |           AA352088           |                               |
|                       |           AW957267           |                               |
|                       |             R59747           |                               |
|                       |           BM469596           |                               |
|                       |           AL135277           |                               |
|                       |             R50924           |                               |
|                       |           BE222025           |                               |
|                       |           BP230980           |                               |
|                       |           BF095244           |                               |
|                       |           BM705776           |                               |
|                       |           BM925423           |                               |
|                       |           BG289344           |                               |
|                       |           BX362676           |                               |
|                       |           BE440111           |                               |
|                       |           AI989323           |                               |
|                       |           BF115175           |                               |
|                       |           BU684792           |                               |
|                       |           CB306976           |                               |
|                       |           CF529357           |                               |
|                       |           BU618432           |                               |
|                       |           CA423138           |                               |
|                       |           AV646654           |                               |
|                       |           AA781009           |                               |
|                       |           BF059620           |                               |
|                       |           CA430141           |                               |
|                       |           AI811296           |                               |
|                       |           AW675700           |                               |
|                       |           BU734976           |                               |
|                       |           BI496910           |                               |
|                       |             N98509           |                               |
|                       |             N91014           |                               |
|                       |           AI078690           |                               |
|                       |           AV646796           |                               |
|                       |           AV646829           |                               |
|                       |           AV651379           |                               |
|                       |           BQ185430           |                               |
|                       | *Seq. mapped in strand Minus*| *Seq. mapped in strand Minus* |
|                       |           AV646577           |          AW491913             |
|                       |                              |          BE948716             |
|                       |                              |          BF467554             |
|                       |                              |                               |
|                       |                              |                               |
|                       |                              |                               |
|                       |                              |                               |
|                       |                              |                               |
|                       |                              |                               |
|                       |                              |                               |
|                       |                              |                               |
|                       |                              |                               |
|                       |                              |                               |
|                       |                              |                               |
|                       |                              |                               |
|                       |                              |                               |
|                       |                              |                               |
|                       |                              |                               |
|                       |                              |                               |
|                       |                              |                               |
|                       |                              |                               |
|                       |                              |                               |
|                       |                              |                               |
|                       |                              |                               |
|                       |                              |                               |
|                       |                              |                               |
|                       |                              |                               |
|                       |                              |                               |
|                       |                              |                               |
|                       |                              |                               |
|                       |                              |                               |
|                       |                              |                               |
|                       |                              |                               |
|                       |                              |                               |
|                       |                              |                               |
|                       |                              |                               |
|                       |                              |                               |
|                       |                              |                               |
|                       |                              |                               |
|                       |                              |                               |
|                       |                              |                               |
|                       |                              |                               |
|                       |                              |                               |
|                       |                              |                               |
|                       |                              |                               |
|                       |                              |                               |
|                       |                              |                               |
|                       |                              |                               |
|                       |                              |                               |
|                       |                              |                               |
|                       |                              |                               |
|                       |                              |                               |
|                       |                              |                               |
|                       |                              |                               |
|                       |                              |                               |
|                       |                              |                               |
|                       |                              |                               |
|                       |                              |                               |
|                       |                              |                               |
|                       |                              |                               |
|                       |                              |                               |
|                       |                              |                               |
|                       |                              |                               |
|                       |                              |                               |
|                       |                              |                               |
|                       |                              |                               |
|                       |                              |                               |
|                       |                              |                               |
|                       |                              |                               |
|                       |                              |                               |
|                       |                              |                               |
|                       |                              |                               |
|                       |                              |                               |
|                       |                              |                               |
|                       |                              |                               |
|                       |                              |                               |
|                       |                              |                               |
|                       |                              |                               |
|                       |                              |                               |
|                       |                              |                               |
|                       |                              |                               |
|                       |                              |                               |
|                       |                              |                               |
|                       |                              |                               |
|                       |                              |                               |
|                       |                              |                               |
|                       |                              |                               |
|                       |                              |                               |
|                       |                              |                               |
|                       |                              |                               |
|                       |                              |                               |
|                       |                              |                               |
|                       |                              |                               |
|                       |                              |                               |
|                       |                              |                               |
|                       |                              |                               |
|                       |                              |                               |
|                       |                              |                               |
|                       |                              |                               |
|                       |                              |                               |
|                       |                              |                               |
|                       |                              |                               |
|                       |                              |                               |
|                       |                              |                               |
|                       |                              |                               |
|                       |                              |                               |
|                       |                              |                               |
|                       |                              |                               |
|                       |                              |                               |
|                       |                              |                               |
|                       |                              |                               |
|                       |                              |                               |
|                       |                              |                               |
+-----------------------+------------------------------+-------------------------------+
|      #  101           | *Seq. mapped in strand Plus* | *Seq. mapped in strand Plus*  |
|                       |           AK123832           |          AK030638             |
|                       |           BC051707           |          AK081764             |
|                       |           BC036772           |          BQ937882             |
|                       |           CN272132           |          CD349285             |
|                       |           BG118963           |          CB246723             |
|                       |           BU163826           |          BE646969             |
|                       |                              |          AW047429             |
|                       | *Seq. mapped in strand Minus*| *Seq. mapped in strand Minus* |
|                       |           BM717371           |          BE988992             |
|                       |           BM674737           |                               |
+-----------------------+------------------------------+-------------------------------+
|      #  102           | *Seq. mapped in strand Plus* | *Seq. mapped in strand Plus*  |
|                       |           AK057340           |          AK017918             |
|                       |           AY227113           |          CO044395             |
|                       |           BM787446           |          AI839897             |
|                       |           AL708248           |          AW047504             |
|                       |           BX537963           |                               |
|                       |           AY227111           |                               |
|                       |           AL708154           |                               |
|                       |           CN402199           |                               |
|                       |           AY162213           |                               |
|                       |           AF516142           |                               |
|                       |           AI140615           |                               |
|                       |           AY227112           |                               |
|                       |           AL043182           |                               |
|                       |           BM696484           |                               |
|                       |           AI953890           |                               |
|                       |           CN402200           |                               |
|                       |           AK025102           |                               |
|                       |           AL833703           |                               |
|                       |           AK000729           |                               |
|                       |           BQ772770           |                               |
|                       |           BM991766           |                               |
|                       |           CA432126           |                               |
|                       |           AA225281           |                               |
|                       |           AW576364           |                               |
|                       |           AW471248           |                               |
|                       |           AI457941           |                               |
|                       |           AI932971           |                               |
|                       |           AI128991           |                               |
|                       |           BG057947           |                               |
|                       | *Seq. mapped in strand Minus*| *Seq. mapped in strand Minus* |
|                       |           BX114440           |          BQ031404             |
|                       |                              |          BE992184             |
|                       |                              |          BB094700             |
|                       |                              |                               |
+-----------------------+------------------------------+-------------------------------+
|      #  103           | *Seq. mapped in strand Plus* | *Seq. mapped in strand Plus*  |
|                       |           CB129868           |          CF909335             |
|                       |           BP870483           |          AK014291             |
|                       |           BM792611           |          BU841555             |
|                       |           CN408182           |          BE331951             |
|                       |           BU603714           |          BY091951             |
|                       |           AU142518           |          BU519315             |
|                       |           BG530909           |          BY066806             |
|                       |           CN408173           |          BY041259             |
|                       |           CN408126           |          BY055146             |
|                       |           BF670260           |          BY044594             |
|                       |           CN408155           |          BY778426             |
|                       |           CN408139           |          BC018255             |
|                       |           CN408164           |          BY067914             |
|                       |           AA223121           |          BF608016             |
|                       |           BU553216           |          BQ950630             |
|                       |           CN408116           |          BU513724             |
|                       |           BU187484           |          BY122694             |
|                       |           CN408180           |          BI251068             |
|                       |           CN408174           |          BQ948372             |
|                       |           BF309832           |          BY031463             |
|                       |           BE563543           |          BY007074             |
|                       |           CN408128           |          BU510164             |
|                       |           CN408142           |          BY150799             |
|                       |           CN408172           |          BY777926             |
|                       |           AA206519           |          BY331803             |
|                       |           CN408149           |          BY145129             |
|                       |           CA392110           |          BY305105             |
|                       |           BP276173           |          BY305039             |
|                       |           BG529281           |          BY305278             |
|                       |           BE541861           |          BY784427             |
|                       |             D45198           |          BY789559             |
|                       |           BE538496           |          BY775508             |
|                       |           BG290695           |          BY335134             |
|                       |           BG503825           |          BY080151             |
|                       |           AU123512           |          BY082847             |
|                       |           BM839623           |          BY028208             |
|                       |           AU099526           |          BY300453             |
|                       |           AA313621           |          BY025367             |
|                       |           BP262679           |          BY139406             |
|                       |           AW957830           |          BY025210             |
|                       |           BI521031           |          BY002510             |
|                       |           BQ706971           |          BY766313             |
|                       |           CN408130           |          BB651888             |
|                       |           BG387155           |          BY142747             |
|                       |           BQ679174           |          BY767596             |
|                       |           BE563307           |          BY031524             |
|                       |             U47704           |            C89073             |
|                       |           AW373676           |          BQ256624             |
|                       |           BE397837           |          BQ256877             |
|                       |           AW749871           |          CN676826             |
|                       |           AW373693           |          CD565684             |
|                       |           BM749531           |          CN720010             |
|                       |           CN408151           |          CF900609             |
|                       |           CN408143           |          CN688002             |
|                       |           BM551876           |          CN674327             |
|                       |           BM563316           |          CF900880             |
|                       |           BM047580           |          CF951194             |
|                       |           BU542944           |          BQ257326             |
|                       |           BU543146           |          CA559071             |
|                       |           BX432253           |          CA559903             |
|                       |           BX458013           |          CA560517             |
|                       |           BX446846           |          CK341311             |
|                       |           BU508418           |          CA562282             |
|                       |           BX465176           |          CF173035             |
|                       |           BU180313           |          CF173508             |
|                       |           BC014567           |          CF172498             |
|                       |           CR602404           |          BQ963397             |
|                       |           BU541775           |          BF723223             |
|                       |           BU527660           |          CA534229             |
|                       |           BU526511           |          CA540767             |
|                       |           AU118784           |          CN723010             |
|                       |           BM557105           |          CF895277             |
|                       |           BM687690           |          CN683960             |
|                       |           BM555689           |          AB044937             |
|                       |           AA314914           |            D76877             |
|                       |           BQ708529           |          AA624202             |
|                       |           BQ923208           |          CB575875             |
|                       |           BM550349           |          CN686843             |
|                       |           BM550310           |          CN722376             |
|                       |           CN408166           |          CN689395             |
|                       |           BU158400           |          CD539489             |
|                       |           BU848740           |          CD542600             |
|                       |           BX410870           |          CN717039             |
|                       |           BX410869           |          BQ572524             |
|                       |           BX461679           |          CN678803             |
|                       |           BQ229126           |          CA543819             |
|                       |           CB131666           |          CN680525             |
|                       |           AA179445           |          BI439319             |
|                       |           BC032749           |          CN679456             |
|                       |           BQ708040           |          BI715815             |
|                       |           BM800752           |          CF732329             |
|                       |           BX458598           |          BI715766             |
|                       |           BX428791           |          BU511460             |
|                       |           BX429054           |          CA321082             |
|                       |           CR594714           |          CF895955             |
|                       |           BG750910           |          CN679216             |
|                       |           BX418894           |          CN688883             |
|                       |           BX428836           |          CD560776             |
|                       |           AA318099           |          CD564244             |
|                       |             X75091           |          BU514952             |
|                       |           AA341055           |          AL024232             |
|                       |             M93651           |          CD548182             |
|                       |           BQ226417           |          CD771874             |
|                       |           CV030859           |          CN696492             |
|                       |           CR542050           |          BB855745             |
|                       |           AY349172           |          BF788965             |
|                       |             U51924           |          BU147753             |
|                       |           CR536543           |          BI900409             |
|                       |           AW239416           |          BQ899734             |
|                       |           BU153603           |          BU151920             |
|                       |           BM803350           |          CB181322             |
|                       |           BE165897           |          BU516914             |
|                       |           BM721900           |          BY717666             |
|                       |           AA296390           |          BI986870             |
|                       |           BM825151           |          BQ964858             |
|                       |           CD243248           |          BE306439             |
|                       |           BF359324           |          BU513435             |
|                       |           CN408171           |          BI987539             |
|                       |           BX407656           |          BY739833             |
|                       |           BX460027           |          CD353929             |
|                       |           BF435091           |          BQ713711             |
|                       |           BF110123           |          CA751932             |
|                       |           AI768142           |          BQ942349             |
|                       |           CN408156           |          BF781308             |
|                       |           BE746145           |          BI790577             |
|                       |           BE740960           |          BI437773             |
|                       |           AW375987           |          BQ885350             |
|                       |           AW375988           |          CA978887             |
|                       |           BF736119           |          BY227460             |
|                       |           BF736123           |          BU519861             |
|                       |           BF736124           |          BY169134             |
|                       |           BF735948           |          AK019960             |
|                       |           BU500235           |          BB618781             |
|                       |           BM915046           |          BY717371             |
|                       |           AW900658           |          CF952055             |
|                       |           BQ380043           |          CB841978             |
|                       |           BQ380044           |          AB015613             |
|                       |           BQ380047           |          BY773054             |
|                       |           BQ380045           |          BF579671             |
|                       |           BI064295           |          BY354605             |
|                       |           BQ380046           |          BY358521             |
|                       |           BE179296           |          CF734962             |
|                       |           BX460648           |          BQ896455             |
|                       |           BF336052           |          BI655931             |
|                       |           AA377175           |          BQ840188             |
|                       |           AA377176           |          CF751751             |
|                       |           CB133792           |          AL118403             |
|                       |           BU502578           |          BF608820             |
|                       |           AW384700           |          CF904708             |
|                       |           BM017767           |          BQ445104             |
|                       |           AA206939           |          CF951256             |
|                       |           AA233558           |            D21513             |
|                       |           BU182627           |          CN455778             |
|                       |           BG427278           |          CF748639             |
|                       |           BG110788           |          BU515075             |
|                       |           AA356749           |          BQ042750             |
|                       |           BE080391           |            D28721             |
|                       |           CN408146           |          CF158312             |
|                       |           CN408170           |          BG519263             |
|                       |           AI267315           |          AL022685             |
|                       |           BE179295           |          BE303211             |
|                       |           AU128938           |          AW681843             |
|                       |           BG118056           |          BQ887366             |
|                       |           BF735952           |          AW682597             |
|                       |           BU500886           |          BQ933694             |
|                       |           AI907472           |          CD354852             |
|                       |           BQ674715           |          AA474356             |
|                       |           BF238362           |          CB317240             |
|                       |           BE886846           |          BG078372             |
|                       |           AW999018           |          BG078373             |
|                       |           BE814352           |          BQ964880             |
|                       |           BQ230566           |          CA537278             |
|                       |           AI656103           |          BM878249             |
|                       |           BE090089           |          CF749615             |
|                       |           BX408496           |          BU515026             |
|                       |           AA329702           |          CB589854             |
|                       |           BE177338           |          CN676853             |
|                       |           BF238885           |          BQ177396             |
|                       |           BE883200           |          CB201947             |
|                       |           CB160086           |          BM933514             |
|                       |           BE550621           |          BM942315             |
|                       |           BG740962           |          AA796821             |
|                       |           BU601487           |          BM945094             |
|                       |           AW629348           |          CB845318             |
|                       |           CA773797           |          CK334140             |
|                       |           CN408141           |          CB588022             |
|                       |           CB142949           |          CB193017             |
|                       |           CN408144           |          BI684441             |
|                       |           CN408176           |          CF582910             |
|                       |           BG820578           |          CA977239             |
|                       |           BX429314           |          AW537027             |
|                       |           BX407962           |          AA606606             |
|                       |           CR604929           |          BU755590             |
|                       |           BE090105           |          AA549660             |
|                       |           BG620458           |          AW537141             |
|                       |           BQ353829           |          AW543995             |
|                       |           CB156498           |          AW545647             |
|                       |           AW390844           |          AW549516             |
|                       |           AW390843           |          BM203217             |
|                       |           AA738062           |          AW539747             |
|                       |           BX429232           |          BG975747             |
|                       |           BE090141           |          AW545613             |
|                       |           BM470994           |          AL362332             |
|                       |           BQ330342           |          BC012508             |
|                       |           BQ330341           |          AA529339             |
|                       |           BF229160           |          BM229104             |
|                       |             D54409           |          BI794705             |
|                       |           BQ330343           |          AW543251             |
|                       |           BG461305           |          BI900101             |
|                       |           CB139826           |          AW537304             |
|                       |           BU564513           |          BI794663             |
|                       |           BU943163           |          BI790322             |
|                       |           AW804718           |          AV053787             |
|                       |           BX450389           |          AA606818             |
|                       |           BG028694           |          AA795180             |
|                       |           BX429377           |          BI438013             |
|                       |           CB131935           |          AW542369             |
|                       |           BG681988           |          BM218831             |
|                       |           BP382555           |          AW538604             |
|                       |           AU136320           |          BM206340             |
|                       |           CB963881           |          CB841734             |
|                       |           BG926568           |          CB524680             |
|                       |           BM475619           |          AW541942             |
|                       |           BE090149           |          CB523749             |
|                       |           BP242609           |          BU524625             |
|                       |           BQ334018           |          BY385143             |
|                       |           BM785813           |          CD539488             |
|                       |           AW993169           |          BB768653             |
|                       |           CN408165           |          BM199112             |
|                       |           CB134182           |          CD538137             |
|                       |           BE840909           |          CD542599             |
|                       |           BF110701           |                               |
|                       |           BE046796           |                               |
|                       |           BE644754           |                               |
|                       |           AI348548           |                               |
|                       |           CB159266           |                               |
|                       |           CN408138           |                               |
|                       |           BE840773           |                               |
|                       |           BF105140           |                               |
|                       |           BU180716           |                               |
|                       |           BI044860           |                               |
|                       |           BE812818           |                               |
|                       |           BE812813           |                               |
|                       |           BG259603           |                               |
|                       |           BE172728           |                               |
|                       |           BQ332248           |                               |
|                       |             T19570           |                               |
|                       |           BQ425480           |                               |
|                       |           BX429053           |                               |
|                       |           BE164754           |                               |
|                       |           BE814574           |                               |
|                       |           BE175229           |                               |
|                       |           CA429513           |                               |
|                       |           BU618631           |                               |
|                       |           BU618788           |                               |
|                       |           CA417995           |                               |
|                       |           BU622444           |                               |
|                       |           CA432407           |                               |
|                       |           BU159609           |                               |
|                       |           BU683368           |                               |
|                       |           BU681622           |                               |
|                       |           BQ439145           |                               |
|                       |           AW975885           |                               |
|                       |           BM793662           |                               |
|                       |           CA440095           |                               |
|                       |           BQ050587           |                               |
|                       |           BQ330337           |                               |
|                       |           BM974043           |                               |
|                       |           BQ020425           |                               |
|                       |           BU509389           |                               |
|                       |           BM678323           |                               |
|                       |           BU683140           |                               |
|                       |           BU687819           |                               |
|                       |           BU688587           |                               |
|                       |           BM970953           |                               |
|                       |           CA424471           |                               |
|                       |           AW293933           |                               |
|                       |           BM973796           |                               |
|                       |           AA356213           |                               |
|                       |           AA314939           |                               |
|                       |           CB135445           |                               |
|                       |           CB147903           |                               |
|                       |           CB135482           |                               |
|                       |           CF541354           |                               |
|                       |           AI924906           |                               |
|                       |           CA441111           |                               |
|                       |           BM969462           |                               |
|                       |             T94763           |                               |
|                       |           BX474494           |                               |
|                       |           CN408145           |                               |
|                       |           CN408177           |                               |
|                       |           AW245056           |                               |
|                       |           BM678412           |                               |
|                       |           CN408147           |                               |
|                       |           BU949251           |                               |
|                       |           AI753576           |                               |
|                       |           BI712579           |                               |
|                       |           CN408157           |                               |
|                       |           BM969562           |                               |
|                       |           BM979758           |                               |
|                       |           BU674614           |                               |
|                       |           BU679171           |                               |
|                       |           AL037448           |                               |
|                       |           BI677250           |                               |
|                       |           BU678793           |                               |
|                       |           BU678889           |                               |
|                       |           BM968317           |                               |
|                       |           CA311584           |                               |
|                       |           BM968466           |                               |
|                       |           BM979175           |                               |
|                       |           BQ018959           |                               |
|                       |           BU674726           |                               |
|                       |           BU686121           |                               |
|                       |           CA437819           |                               |
|                       |           BQ028524           |                               |
|                       |           CA440825           |                               |
|                       |           BU680160           |                               |
|                       |           CA434402           |                               |
|                       |           CA437737           |                               |
|                       |           BQ018350           |                               |
|                       |           BU619135           |                               |
|                       |           AA578980           |                               |
|                       |           AA848156           |                               |
|                       |           BM672633           |                               |
|                       |           BU198011           |                               |
|                       |           BU071260           |                               |
|                       |           BU071474           |                               |
|                       |           CB115277           |                               |
|                       |           CB119181           |                               |
|                       |           CB119814           |                               |
|                       |           BU688123           |                               |
|                       |           CA311515           |                               |
|                       |           BU676736           |                               |
|                       |           BU616590           |                               |
|                       |           BM990436           |                               |
|                       |           AI857783           |                               |
|                       |           BM990197           |                               |
|                       |           CR595023           |                               |
|                       |           AW023271           |                               |
|                       |           BU620085           |                               |
|                       |           AI357417           |                               |
|                       |           BU615362           |                               |
|                       |           BQ446495           |                               |
|                       |           BU618813           |                               |
|                       |           CA419483           |                               |
|                       |           BQ775874           |                               |
|                       |           AW591548           |                               |
|                       |           AI678341           |                               |
|                       |           AI284414           |                               |
|                       |           AI337731           |                               |
|                       |           BM665874           |                               |
|                       |           AA814515           |                               |
|                       |           AA814512           |                               |
|                       |           BU633683           |                               |
|                       |           CA418153           |                               |
|                       |           BQ775274           |                               |
|                       |           BG231864           |                               |
|                       |           BU622190           |                               |
|                       |           BF510869           |                               |
|                       |           CN408184           |                               |
|                       |           AA527621           |                               |
|                       |           AA528362           |                               |
|                       |           CN408136           |                               |
|                       |           AW022625           |                               |
|                       |           CN408183           |                               |
|                       |           CA416851           |                               |
|                       |           CA418376           |                               |
|                       |           BQ008016           |                               |
|                       |           CB851601           |                               |
|                       |           AI809049           |                               |
|                       |           BQ775408           |                               |
|                       |           BU631610           |                               |
|                       |           BM460771           |                               |
|                       |           CA418938           |                               |
|                       |           BQ775815           |                               |
|                       |           CA440849           |                               |
|                       |           BM968485           |                               |
|                       |           BU682673           |                               |
|                       |           AW005348           |                               |
|                       |           BU677586           |                               |
|                       |           BQ775736           |                               |
|                       |           CA441911           |                               |
|                       |           BQ774097           |                               |
|                       |           BQ026859           |                               |
|                       |           AI813582           |                               |
|                       |           AW474572           |                               |
|                       |           BQ004584           |                               |
|                       |           AA975070           |                               |
|                       |           AI246019           |                               |
|                       |           AA778451           |                               |
|                       |           AW028966           |                               |
|                       |           BQ021366           |                               |
|                       |           BQ019373           |                               |
|                       |           CA443099           |                               |
|                       |           CN408160           |                               |
|                       |           BU682320           |                               |
|                       |           AI186688           |                               |
|                       |           BE675268           |                               |
|                       |           AA858182           |                               |
|                       |           CA419540           |                               |
|                       |           AI266247           |                               |
|                       |           AA460676           |                               |
|                       |           AI096767           |                               |
|                       |           CR744496           |                               |
|                       |           BM993025           |                               |
|                       |           BQ004089           |                               |
|                       |           BU631282           |                               |
|                       |           CN408119           |                               |
|                       |           AA668579           |                               |
|                       |           AA552184           |                               |
|                       |           BG110679           |                               |
|                       |           AI753939           |                               |
|                       |             W57870           |                               |
|                       |           AA772588           |                               |
|                       |           AI128789           |                               |
|                       |           AI472202           |                               |
|                       |             N62367           |                               |
|                       |           AW043765           |                               |
|                       |           AW450621           |                               |
|                       |           AI370266           |                               |
|                       |           BM663756           |                               |
|                       |           CA444878           |                               |
|                       |           BF590339           |                               |
|                       |           BM678999           |                               |
|                       |           AA974937           |                               |
|                       |           BQ030311           |                               |
|                       |           BM828895           |                               |
|                       |           CB112493           |                               |
|                       |           AA978156           |                               |
|                       |           BI495799           |                               |
|                       |           BI495800           |                               |
|                       |           BI966149           |                               |
|                       | *Seq. mapped in strand Minus*| *Seq. mapped in strand Minus* |
|                       |           AI564211           |          BU757493             |
|                       |           BE717704           |          AI854686             |
|                       |           AI669128           |          BF462550             |
|                       |           AA781061           |          CD774492             |
|                       |           AW083629           |          BE953736             |
|                       |           BF089185           |          BI987081             |
|                       |           BE439849           |          BU924818             |
|                       |           AA917780           |          BF713905             |
|                       |           AI524784           |                               |
|                       |           AI053988           |                               |
|                       |           AI054111           |                               |
|                       |           BU674336           |                               |
|                       |           BM969135           |                               |
|                       |           AI054073           |                               |
|                       |           AA644321           |                               |
|                       |           AI053418           |                               |
|                       |           AA450108           |                               |
|                       |           CB113796           |                               |
|                       |           AI053736           |                               |
|                       |           AI053892           |                               |
|                       |           CB131862           |                               |
|                       |           AV707020           |                               |
|                       |           BE043407           |                               |
|                       |           AI053967           |                               |
|                       |           AI950006           |                               |
|                       |           AA062923           |                               |
|                       |           CN408178           |                               |
|                       |           CN408132           |                               |
|                       |           AA160127           |                               |
|                       |             N32850           |                               |
|                       |           CN408163           |                               |
|                       |           BF448410           |                               |
|                       |           BQ000499           |                               |
|                       |           AA564853           |                               |
|                       |           AA971838           |                               |
|                       |           AI581359           |                               |
|                       |           CA314130           |                               |
+-----------------------+------------------------------+-------------------------------+
|      #  104           | *Seq. mapped in strand Plus* | *Seq. mapped in strand Plus*  |
|                       |           AL039025           |          BY252576             |
|                       |           BI544285           |          BY149603             |
|                       |           BI458022           |          BY792316             |
|                       |           BG700039           |          BY141581             |
|                       |           BG719704           |          BY142903             |
|                       |           BI603062           |          BY779849             |
|                       |           BG716521           |          BY781228             |
|                       |           BI553463           |          BY143178             |
|                       |           BI600997           |          BY144638             |
|                       |           BI545362           |          BY785976             |
|                       |           BI552705           |          BY150691             |
|                       |           BG541036           |          BY150692             |
|                       |           BG434362           |          BY137738             |
|                       |           BG619996           |          BY778598             |
|                       |           AL035801           |          BY775286             |
|                       |           AL035808           |          BY794600             |
|                       |           AB032607           |          BY142034             |
|                       |           AW161496           |          BY773887             |
|                       |           CB146088           |          BY788702             |
|                       |           CB156762           |          BY134134             |
|                       |           AA557759           |          BY788489             |
|                       |           BM544142           |          BY789020             |
|                       |           BM919525           |          BY141884             |
|                       |           BM919491           |          BY791702             |
|                       |           AV706943           |          BY137081             |
|                       |           BI712978           |          BY145625             |
|                       |           BI160994           |          BY774913             |
|                       |           BI457289           |          BY770412             |
|                       |           BX462852           |          BY137695             |
|                       |           BX247998           |          BY301737             |
|                       |             R84442           |          BY139205             |
|                       |           BX338177           |          BY275457             |
|                       |           CN483371           |          BY238412             |
|                       |           AI133545           |          BY268810             |
|                       |           BX403373           |          BY244086             |
|                       |           BX332215           |          CA787187             |
|                       |           BX325060           |          BY788213             |
|                       |           BX459667           |          BY775553             |
|                       |           BX459919           |          CB232587             |
|                       |           BX325123           |          CB233487             |
|                       |           BX422422           |          BU559764             |
|                       |           BX445327           |          BY250138             |
|                       |           BX460093           |          BY003614             |
|                       |           BX422438           |          BY260721             |
|                       |           BX432727           |          BY252126             |
|                       |           BX418977           |          BY249963             |
|                       |           BX367139           |          BB596263             |
|                       |           BX397338           |          BY790358             |
|                       |           BX429130           |          BY292867             |
|                       |           BX444221           |          BY284831             |
|                       |           BM920614           |          BY298404             |
|                       |           BX405678           |          BY001092             |
|                       |           BX411177           |          AU066913             |
|                       |           BX442877           |          AU051469             |
|                       |           BX388426           |          AU051493             |
|                       |           BX441531           |          AU035679             |
|                       |           BX407662           |          AU051375             |
|                       |           BX346603           |          AU050870             |
|                       |           BX444351           |          AU080624             |
|                       |           BX444450           |          AU080543             |
|                       |           BX407756           |          AU051778             |
|                       |           BX449881           |          AU080290             |
|                       |           BX450006           |          BY781280             |
|                       |           BX407693           |          BY305199             |
|                       |           BX406370           |          BY785382             |
|                       |           BX440609           |          BY787581             |
|                       |           BX463384           |          BY098297             |
|                       |           CR607907           |          BY216376             |
|                       |           CR624712           |          BY781306             |
|                       |           BX161452           |          BY335521             |
|                       |           CR592849           |          BY214851             |
|                       |           CR603886           |          BY084021             |
|                       |           CR606010           |          BY212919             |
|                       |           CR610066           |          BY076380             |
|                       |           CR612108           |          BY222597             |
|                       |           CR620087           |          BY264670             |
|                       |           CR591136           |          BY266759             |
|                       |           CR603748           |          BY771790             |
|                       |           CR598389           |          BY218171             |
|                       |           CR615599           |          BY264085             |
|                       |           CR603291           |          BY304488             |
|                       |           AF119863           |          BY288745             |
|                       |           CR608797           |          BY261814             |
|                       |           CR621793           |          BY261893             |
|                       |           CR609941           |          BY269050             |
|                       |           CR626265           |          BY250481             |
|                       |             H59227           |          BY243589             |
|                       |           AV693188           |          BY211166             |
|                       |           AV689832           |          BY282732             |
|                       |           AV688678           |          BY252417             |
|                       |           AV684461           |          BY250066             |
|                       |           AV685380           |          BY245676             |
|                       |           AV684463           |          AW106420             |
|                       |           BQ581991           |          BY735778             |
|                       |           BX371175           |          BY103414             |
|                       |           BX399541           |          BY240543             |
|                       |           BX448226           |          BY147637             |
|                       |           BX462855           |          BY248184             |
|                       |           BX422612           |          BY238917             |
|                       |           CR620703           |          BY140319             |
|                       |           CR608449           |          BY780165             |
|                       |           BX248012           |          BY260116             |
|                       |           AV690806           |          BY098315             |
|                       |           AA297821           |          BY146024             |
|                       |           BX444313           |          BY328657             |
|                       |           BX407747           |          BY104565             |
|                       |           CR596535           |          BY146168             |
|                       |           AA297678           |          BY146147             |
|                       |           BQ719859           |          BY793741             |
|                       |           BQ716544           |          BY098652             |
|                       |             R87222           |          BY150467             |
|                       |           BQ718013           |          BY098662             |
|                       |           BM906142           |          BY140039             |
|                       |           BM805094           |          BY778219             |
|                       |           BF314353           |          BY310702             |
|                       |           AY314975           |          BY775565             |
|                       |           AV707488           |          BY303102             |
|                       |           AA297021           |          BY341126             |
|                       |           BQ718723           |          BY072692             |
|                       |           BM722506           |          BY327031             |
|                       |           BQ067825           |          BY794423             |
|                       |           BX367552           |          BY144339             |
|                       |           BX427898           |          BY794606             |
|                       |           BX353476           |          BY135684             |
|                       |           BX374820           |          BY794577             |
|                       |           CR602474           |          BY078996             |
|                       |           CR611497           |          BY792918             |
|                       |           CR618544           |          BY141148             |
|                       |           AA297100           |          BY792892             |
|                       |           CD251795           |          BY237560             |
|                       |           BX405426           |          BY778202             |
|                       |           BX346167           |          BY774207             |
|                       |           BX443856           |          BY279489             |
|                       |           BX429093           |          BY280671             |
|                       |           CR591736           |          BY275133             |
|                       |           CR615598           |          BY267849             |
|                       |           BX358341           |          BY786206             |
|                       |           AA297227           |          BY775163             |
|                       |           BG896653           |          BY770510             |
|                       |             H49427           |          BY265784             |
|                       |           BX336001           |          BY266758             |
|                       |           BX449785           |          BY151486             |
|                       |           BX438953           |          BY133681             |
|                       |           CR623957           |          BY774909             |
|                       |           AV692560           |          BY134401             |
|                       |           AA984335           |          BY281599             |
|                       |           BX161432           |          BY294404             |
|                       |           BE383106           |          BY268903             |
|                       |           AA297065           |          BY306479             |
|                       |             W67892           |          BY139016             |
|                       |           BE939217           |          BY767959             |
|                       |           BE939204           |          BY253871             |
|                       |           BQ717638           |          BY139042             |
|                       |           AV749581           |          BY293271             |
|                       |             R89121           |          BY132262             |
|                       |           BQ269256           |          BY133157             |
|                       |           AV748482           |          BY272324             |
|                       |           AA368167           |          BY264509             |
|                       |           AA297093           |          BY256800             |
|                       |           BG012902           |          BY330519             |
|                       |           BM722286           |          BY261907             |
|                       |             R94442           |          BY769765             |
|                       |           BG014671           |          BY135532             |
|                       |           BG014716           |          BY275057             |
|                       |           AA969743           |          BY272784             |
|                       |           BF741576           |          BY266908             |
|                       |           BX347294           |          BY132710             |
|                       |           AA296981           |          BY279974             |
|                       |           BG014281           |          BY278447             |
|                       |           BX408457           |          BY273549             |
|                       |           BX368070           |          BY247345             |
|                       |           AF052114           |          BY277917             |
|                       |           BX347464           |          BY263102             |
|                       |           BX347463           |          BY238808             |
|                       |           BF509584           |          BY286739             |
|                       |           AW291645           |          BY298226             |
|                       |           BQ787527           |          BY261399             |
|                       |           CB154821           |          BY289997             |
|                       |           BX444144           |          BY277260             |
|                       |           CR613369           |          BY268759             |
|                       |           AV688028           |          BY277624             |
|                       |           BX429449           |          BY238559             |
|                       |           BX429546           |          BY270505             |
|                       |           CR610233           |          BY247239             |
|                       |           BG015185           |          BY285092             |
|                       |           BX429954           |          BY289097             |
|                       |           BX463221           |          BY255144             |
|                       |           BX422421           |          BY268596             |
|                       |           BX407755           |          BY239069             |
|                       |           BX462854           |          BY276005             |
|                       |           BX444312           |          BY265945             |
|                       |           CR609760           |          BY270722             |
|                       |           BX407692           |          BY277104             |
|                       |           BX444143           |          BY281348             |
|                       |           BX422167           |          BY266015             |
|                       |           BX358335           |          BY287670             |
|                       |           BX368158           |          BY240173             |
|                       |           BX407661           |          BY271736             |
|                       |           BX444350           |          BY287783             |
|                       |           BX462851           |          BY246670             |
|                       |           BX449880           |          BY287529             |
|                       |           AI174720           |          BY250575             |
|                       |           BX346602           |          BY239617             |
|                       |           BX367746           |          BY266448             |
|                       |           BX448225           |          BY290918             |
|                       |           BX422437           |          BY251858             |
|                       |           BE046799           |          BY272379             |
|                       |           AI292216           |          BY263174             |
|                       |           BX336043           |          BY246190             |
|                       |           BU681590           |          BY256053             |
|                       |           BX346166           |          BY249100             |
|                       |           BX408189           |          BY242436             |
|                       |           BX388604           |          BY277210             |
|                       |           BX449784           |          BY242241             |
|                       |           AV753036           |          BY239846             |
|                       |             H87083           |          BY243423             |
|                       |           BX450276           |          BY254483             |
|                       |           BX368232           |          BY241062             |
|                       |           BX408226           |          BY248814             |
|                       |           BX445326           |          BY276892             |
|                       |           BX427897           |          BY244924             |
|                       |           BX408219           |          BY249207             |
|                       |           BX346951           |          BY245487             |
|                       |           BX406369           |          BY254527             |
|                       |           CA943189           |          BY255080             |
|                       |           BX412855           |          BY243611             |
|                       |             N76324           |          BY258363             |
|                       |           BX434722           |          BY265488             |
|                       |           BX325059           |          BY265496             |
|                       |           BX367138           |          BB610215             |
|                       |           BX405677           |          BY793588             |
|                       |           BU580862           |          BY781333             |
|                       |           AV747610           |          BY781390             |
|                       |             W07248           |          BY263731             |
|                       |             H91969           |          BY281094             |
|                       |           CB154926           |          BY279113             |
|                       |           AV748261           |          BY268632             |
|                       |           BX455272           |          BY272640             |
|                       |           BX408784           |          BY268447             |
|                       |             H71305           |            Y13832             |
|                       |           BX450687           |            W61993             |
|                       |           BU782673           |          CB058596             |
|                       |           AV748976           |          BU058739             |
|                       |           BF927790           |          BG655394             |
|                       |           BG703541           |          AA118356             |
|                       |           BX351913           |          BQ829250             |
|                       |             R99163           |          BQ832774             |
|                       |             H59650           |          BQ830039             |
|                       |           BM876329           |          BQ890995             |
|                       |           BX429896           |          BQ952094             |
|                       |           BX429869           |          BQ715916             |
|                       |           BX326323           |          BI734965             |
|                       |           BX367551           |          CD349783             |
|                       |           BX368151           |          BQ963957             |
|                       |           AV748189           |          BG655342             |
|                       |             R91627           |          BQ828223             |
|                       |           BX408696           |          BQ829165             |
|                       |           BX408697           |          BM946639             |
|                       |           BF932705           |            W50765             |
|                       |           AV723467           |            W50752             |
|                       |           BQ130786           |            W78636             |
|                       |           BX408790           |          AA929904             |
|                       |           BX435163           |            R75395             |
|                       |           BU075431           |          AA014845             |
|                       |           BQ130070           |          AA097607             |
|                       |           BX397337           |          CF538624             |
|                       |           BX429998           |          BQ829178             |
|                       |           BX353475           |          CB525494             |
|                       |           AK057522           |          AA543255             |
|                       |           BX394256           |          BM943384             |
|                       |           CR620657           |          CB520391             |
|                       |           BX405425           |          CD806074             |
|                       |             H62151           |          BQ964844             |
|                       |           AV707714           |          BM950027             |
|                       |           BX412723           |          BF181364             |
|                       |           AV708711           |          BU707313             |
|                       |           BF947175           |          AA389286             |
|                       |           BX453065           |          CK387301             |
|                       |           BX428871           |          CK387479             |
|                       |           BX428872           |          CK387913             |
|                       |           BX453064           |          AA172804             |
|                       |           BI913162           |          CB589323             |
|                       |           BQ009195           |          BQ833185             |
|                       |           BM679015           |          CA979451             |
|                       |           AA029246           |          CB589497             |
|                       |           BU783773           |          CB202248             |
|                       |           BX407746           |          BU709001             |
|                       |           BQ447062           |            W97303             |
|                       |           BM699430           |          AA427067             |
|                       |             N56264           |          BI731740             |
|                       |           BQ027065           |          BQ922246             |
|                       |           BU579722           |          AA051026             |
|                       |           BU787606           |          BM507392             |
|                       |           BI916294           |          BG142568             |
|                       |           BC036882           |          BG295794             |
|                       |           BU071298           |          BI739742             |
|                       |           BU784540           |          CA980715             |
|                       |           BM716949           |            R75263             |
|                       |           BX423733           |          BQ287553             |
|                       |           BU075598           |          BQ287673             |
|                       |           BE222690           |          AA052779             |
|                       |           BI524541           |            R74757             |
|                       |           BI524001           |            R74801             |
|                       |           BU678061           |          BU920898             |
|                       |           BQ270532           |          BG807002             |
|                       |           AL046857           |          BM964253             |
|                       |           BM505644           |            R75570             |
|                       |           BM894038           |          BG244440             |
|                       |           BU947702           |          BM054348             |
|                       |           BM508115           |          BY295945             |
|                       |           BM857617           |          BQ934578             |
|                       |           BQ130041           |          BX513488             |
|                       |           BM509128           |          BE916472             |
|                       |           BU071161           |          BE308125             |
|                       |           BM509171           |          BG370786             |
|                       |           BQ272478           |          BE573072             |
|                       |           BQ614085           |          BF124526             |
|                       |           BU073387           |          AW742662             |
|                       |           AK092504           |          BM894740             |
|                       |           BQ669975           |            W80169             |
|                       |           CA949980           |          AA002634             |
|                       |             W44755           |            W09773             |
|                       |             H91435           |          CN663660             |
|                       |             H59709           |          BE861872             |
|                       |             W02416           |          BG404711             |
|                       |           AK127864           |          BY240520             |
|                       |           AK055725           |          BM503593             |
|                       |           AJ413187           |          AA103620             |
|                       |           AJ413186           |          CD355328             |
|                       |           CR614788           |          CN664508             |
|                       |           AK092707           |          BC024818             |
|                       |           BQ181140           |          BI730909             |
|                       |           CR617022           |          BF471019             |
|                       |           CR620241           |          CB058206             |
|                       |           BI766509           |          CK030896             |
|                       |           BX335796           |          AW742476             |
|                       |           BC062783           |          AA000879             |
|                       |           BQ477876           |          AA004115             |
|                       |           BX398581           |          BF120932             |
|                       |           BX385938           |          CF746141             |
|                       |           BU789202           |          BC048191             |
|                       |           BX396700           |          CB524587             |
|                       |             C17126           |          CB524487             |
|                       |           AL042942           |          CF585122             |
|                       |           BM315103           |          BI441350             |
|                       |           BQ581130           |          BI965842             |
|                       |           BM510363           |          BM053951             |
|                       |           BQ548989           |          BM053819             |
|                       |           AV685582           |          BI965904             |
|                       |           BM687248           |          AK032021             |
|                       |           AV691186           |          BY718623             |
|                       |           BX367615           |          AV033589             |
|                       |           BM507210           |          CB590891             |
|                       |           BM509444           |          AA004059             |
|                       |           BM512260           |            W64614             |
|                       |           BM313904           |          BU058056             |
|                       |           BU947295           |          CK019912             |
|                       |           BQ270798           |          CF584777             |
|                       |           BU075874           |          CB202480             |
|                       |           AV694165           |          CF584712             |
|                       |           BX427196           |          CF618073             |
|                       |           BX354606           |          BC051667             |
|                       |           CR597219           |          CF578092             |
|                       |           AV695947           |          CF584711             |
|                       |           BX367745           |          BE855234             |
|                       |           BX330409           |          AL362596             |
|                       |           BX331908           |          CB059150             |
|                       |           BU952631           |          AA007998             |
|                       |             H58998           |          BF658505             |
|                       |           BX354605           |          AA183389             |
|                       |           CR612008           |          AA033444             |
|                       |           BX336855           |          BM894630             |
|                       |           AI133465           |          CO042160             |
|                       |           BM929634           |          CB194103             |
|                       |             R78857           |          BM729672             |
|                       |             W95825           |          BQ954402             |
|                       |           BX333200           |          CD776077             |
|                       |           CN479847           |          AA103529             |
|                       |           BQ548879           |          CF745386             |
|                       |           CD513101           |          CB194691             |
|                       |           BX333199           |          CB574930             |
|                       |           BU687787           |          BI965589             |
|                       |           AA054858           |          BM021724             |
|                       |           BI838293           |          BQ416304             |
|                       |           BM683586           |          BM508400             |
|                       |           BQ268259           |          BM729413             |
|                       |           BQ183148           |          AV117970             |
|                       |             H68787           |          AW230980             |
|                       |             H69132           |          CB844247             |
|                       |           AA432355           |          CF585204             |
|                       |           BU681765           |          BQ031556             |
|                       |           BU786542           |          CF585203             |
|                       |             R63593           |          BM508696             |
|                       |             R74372           |          BI965580             |
|                       |           BF999013           |          BG296920             |
|                       |             R53766           |          BI441291             |
|                       |           BU075693           |          BE913420             |
|                       |           BQ447322           |          BM565277             |
|                       |           BM679161           |          CB846932             |
|                       |           BU579805           |          AA207703             |
|                       |             H48704           |          BQ175478             |
|                       |           BU786085           |          BY639725             |
|                       |           BQ478473           |          AA270491             |
|                       |           BM507054           |          BY639887             |
|                       |           BM511324           |          BM021614             |
|                       |           BM875701           |          BY610166             |
|                       |           BM875450           |          CB588611             |
|                       |           BM055113           |          AI507101             |
|                       |           BQ638092           |          BG295660             |
|                       |             W69224           |          BF020991             |
|                       |           AI459255           |          BY612610             |
|                       |           BU079357           |          BY610851             |
|                       |           BQ771670           |            R75262             |
|                       |           BU070170           |          BE984512             |
|                       |           BU070003           |          BE983674             |
|                       |           BU070059           |          BY635019             |
|                       |           BQ549308           |          AW742881             |
|                       |             R81006           |          BY620305             |
|                       |           CR741469           |          BE954659             |
|                       |           BQ016490           |          BB611227             |
|                       |           BU069827           |          AW049517             |
|                       |           BQ549202           |          BE991496             |
|                       |           BU788946           |          BG261675             |
|                       |             W38427           |          BE993167             |
|                       |           AI018638           |          CA951586             |
|                       |           AW003239           |          BM114485             |
|                       |             T93821           |          BE995999             |
|                       |           AA318859           |          BE986259             |
|                       |             W80682           |          BF459314             |
|                       |           AV704361           |          BF458557             |
|                       |           BQ575489           |          BE992002             |
|                       |             H70692           |          BQ418018             |
|                       |           AA582558           |          AK086356             |
|                       |           BQ548748           |          BI134564             |
|                       |           BU579864           |          AI834913             |
|                       |           BU783352           |          AI842646             |
|                       |           BQ130239           |          BC028971             |
|                       |           BU579625           |          BE944436             |
|                       |           BQ129987           |          BE992903             |
|                       |           BU783107           |          BE996652             |
|                       |           BQ181864           |          AK018121             |
|                       |           AA984278           |          AK019916             |
|                       |           BE072890           |          CO041252             |
|                       |           AI681544           |          AI448278             |
|                       |           BE669505           |          BF464236             |
|                       |           BQ130198           |          BF464102             |
|                       |           BQ774315           |          BM899792             |
|                       |           AI870856           |          CD776779             |
|                       |             W25097           |          AK018142             |
|                       |             W93422           |          AK046138             |
|                       |           AA442765           |          BE990468             |
|                       |           AA319172           |          BE990715             |
|                       |           BF115526           |          BE950406             |
|                       |           BM512019           |          BQ175567             |
|                       |           AA620289           |          BE991047             |
|                       |           BM565480           |          BE991381             |
|                       |           BX459666           |          BE947211             |
|                       |           AI125171           |          AI843351             |
|                       |           AA779239           |                               |
|                       |           AI003540           |                               |
|                       |             H49428           |                               |
|                       |             H48532           |                               |
|                       |           BU784805           |                               |
|                       |           BU075124           |                               |
|                       |           BU950864           |                               |
|                       |           CA943793           |                               |
|                       |           BM508181           |                               |
|                       |           BQ268254           |                               |
|                       |           BU787882           |                               |
|                       |           CA774137           |                               |
|                       |           BU580404           |                               |
|                       |           AA988898           |                               |
|                       |           BU784045           |                               |
|                       |           AI874325           |                               |
|                       |           BQ549685           |                               |
|                       |           BU069931           |                               |
|                       |           BM313642           |                               |
|                       |           BM054960           |                               |
|                       |           BM509198           |                               |
|                       |           BQ270557           |                               |
|                       |           BU579968           |                               |
|                       |           AA918364           |                               |
|                       |           AA987892           |                               |
|                       |           BM807525           |                               |
|                       |           BM857657           |                               |
|                       |           AI138899           |                               |
|                       |           AI146279           |                               |
|                       |           AI401572           |                               |
|                       |           AW016235           |                               |
|                       |             N54358           |                               |
|                       |             N55284           |                               |
|                       |           AW090068           |                               |
|                       |             W69081           |                               |
|                       |           AA429656           |                               |
|                       |           AW156990           |                               |
|                       |             W95826           |                               |
|                       |           AA744996           |                               |
|                       |           BU738529           |                               |
|                       |             H70899           |                               |
|                       |           BE155774           |                               |
|                       |           AW817314           |                               |
|                       |             H73768           |                               |
|                       |           AI368233           |                               |
|                       |             R94676           |                               |
|                       |           BU070270           |                               |
|                       |           BM511346           |                               |
|                       |           BM512552           |                               |
|                       |             N78818           |                               |
|                       |             H87735           |                               |
|                       |           AI189406           |                               |
|                       |           AI801328           |                               |
|                       |           AI223081           |                               |
|                       |           AI143719           |                               |
|                       |           AW384297           |                               |
|                       |           AI621180           |                               |
|                       |           BM510517           |                               |
|                       |           BM512296           |                               |
|                       |           AA948316           |                               |
|                       |           AI188681           |                               |
|                       |           AW316731           |                               |
|                       |           AW371545           |                               |
|                       |           AW582515           |                               |
|                       |           AW817306           |                               |
|                       |           BE155780           |                               |
|                       |           AW381763           |                               |
|                       |           AW381768           |                               |
|                       |           AW609838           |                               |
|                       |           AW817275           |                               |
|                       |           BE155782           |                               |
|                       |           AW609837           |                               |
|                       |           BG194761           |                               |
|                       |           AL042644           |                               |
|                       |           BU947650           |                               |
|                       |           AI299753           |                               |
|                       |           BU751924           |                               |
|                       |           AW515017           |                               |
|                       |           AA628340           |                               |
|                       |           AI024488           |                               |
|                       |           AI493259           |                               |
|                       |           AV753072           |                               |
|                       |           AA722764           |                               |
|                       |           AA625740           |                               |
|                       |           BQ285898           |                               |
|                       |           BQ478227           |                               |
|                       |           AI346588           |                               |
|                       |           BQ477456           |                               |
|                       |           BQ581661           |                               |
|                       |           BM565623           |                               |
|                       |           BM510493           |                               |
|                       |           BQ129947           |                               |
|                       |           AI091315           |                               |
|                       |           BM506704           |                               |
|                       |           BU069445           |                               |
|                       |           AA904190           |                               |
|                       |           BU782628           |                               |
|                       |           BM894185           |                               |
|                       |             R44788           |                               |
|                       |           AI453538           |                               |
|                       |           AK123878           |                               |
|                       |           BX101225           |                               |
|                       |           AI638405           |                               |
|                       |           AI651103           |                               |
|                       |           AI969268           |                               |
|                       |           AI634109           |                               |
|                       |           AI632499           |                               |
|                       |           AI968579           |                               |
|                       |           AI374672           |                               |
|                       |           AI342082           |                               |
|                       |           BU727542           |                               |
|                       |           AI266648           |                               |
|                       |           AI637819           |                               |
|                       |           AW138857           |                               |
|                       |           AA610121           |                               |
|                       |           AK124580           |                               |
|                       |             R44918           |                               |
|                       |           AW207761           |                               |
|                       |           BQ184584           |                               |
|                       |           BU743238           |                               |
|                       |           CF541159           |                               |
|                       |           BE551364           |                               |
|                       |           BU075590           |                               |
|                       |           BQ270591           |                               |
|                       |             W73830           |                               |
|                       |           AA833951           |                               |
|                       |           BM314810           |                               |
|                       |           BM504225           |                               |
|                       |           BM504444           |                               |
|                       |           BQ267620           |                               |
|                       |           BQ267994           |                               |
|                       |           BQ272185           |                               |
|                       |             R42971           |                               |
|                       |           AA608939           |                               |
|                       |           AI122966           |                               |
|                       |           AI761892           |                               |
|                       |           AI205863           |                               |
|                       |           BQ023646           |                               |
|                       |           BM510633           |                               |
|                       |           BM511467           |                               |
|                       |           AW079014           |                               |
|                       |           CR593817           |                               |
|                       |           BM677973           |                               |
|                       |           AK098378           |                               |
|                       |           BX099173           |                               |
|                       |           BC036602           |                               |
|                       |           BU789123           |                               |
|                       |           BU787556           |                               |
|                       |           AI457451           |                               |
|                       |           BU789081           |                               |
|                       |           BM511785           |                               |
|                       |           CK823952           |                               |
|                       | *Seq. mapped in strand Minus*| *Seq. mapped in strand Minus* |
|                       |           AV709428           |          BQ177123             |
|                       |           BF988662           |          CD802617             |
|                       |             H89474           |          AI852838             |
|                       |           BE466794           |          BB662914             |
|                       |           AI990407           |          BE948040             |
|                       |           BE673029           |          AW494196             |
|                       |           AW274811           |          AI850416             |
|                       |           AI656033           |          BE994927             |
|                       |           AI203287           |          BE995003             |
|                       |           AI202901           |                               |
|                       |           AW590581           |                               |
|                       |           CA388711           |                               |
+-----------------------+------------------------------+-------------------------------+
|      #  105           | *Seq. mapped in strand Plus* | *Seq. mapped in strand Plus*  |
|                       |           AF207600           |          AK051039             |
|                       |           BC066907           |          AK044502             |
|                       |           BI463052           |          AK016135             |
|                       |           BG772650           |          AK033268             |
|                       |           BP217901           |          AK031560             |
|                       |           AA598956           |          AK049827             |
|                       |           AW474714           |          AK049691             |
|                       |           BX343032           |          BC023950             |
|                       |           BP273201           |          BC034743             |
|                       |           BC006111           |          BC048829             |
|                       |           AL137750           |          AK004338             |
|                       |           AA315012           |                               |
|                       |           CK822915           |                               |
|                       |           BQ086206           |                               |
|                       |           BP215533           |                               |
|                       |           AW957957           |                               |
|                       |           AI198673           |                               |
|                       |           AK074425           |                               |
|                       |           BM129168           |                               |
|                       |           BM129637           |                               |
|                       |           BE261194           |                               |
|                       |           AI984902           |                               |
|                       |           AA608809           |                               |
|                       |           AA884993           |                               |
|                       |           BI085247           |                               |
|                       |           CF125041           |                               |
|                       |           CA748569           |                               |
|                       |           AW631368           |                               |
|                       |           CV029976           |                               |
|                       |           BT007084           |                               |
|                       |           CR737701           |                               |
|                       |           BE041824           |                               |
|                       |           AW978434           |                               |
|                       |           AA830187           |                               |
|                       |             C75267           |                               |
|                       |           CN365101           |                               |
|                       |           BF811339           |                               |
|                       |             H08015           |                               |
|                       |           CB999170           |                               |
|                       |           BF084641           |                               |
|                       |           BU183653           |                               |
|                       |           BX391007           |                               |
|                       |           BG028959           |                               |
|                       |           AW628916           |                               |
|                       |           CA412716           |                               |
|                       |           BE006470           |                               |
|                       |           AW973662           |                               |
|                       |           BF589576           |                               |
|                       |             W56088           |                               |
|                       |           BG994783           |                               |
|                       |           BM129352           |                               |
|                       |           BM128895           |                               |
|                       |           AA770489           |                               |
|                       |           AI678118           |                               |
|                       |           BP336773           |                               |
|                       |           AW614910           |                               |
|                       |             W42580           |                               |
|                       |           BE865845           |                               |
|                       |           BF940157           |                               |
|                       |           AI655529           |                               |
|                       |           AW272903           |                               |
|                       |           BE465192           |                               |
|                       |           AI140596           |                               |
|                       |           AI239459           |                               |
|                       |           AL121525           |                               |
|                       |           BF573512           |                               |
|                       |           AA724564           |                               |
|                       |           BF002406           |                               |
|                       |           AI361619           |                               |
|                       |           AI914512           |                               |
|                       |           AA558957           |                               |
|                       |           BF106167           |                               |
|                       |           BG994384           |                               |
|                       | *Seq. mapped in strand Minus*| *Seq. mapped in strand Minus* |
|                       |           AA701375           |          AK007288             |
+-----------------------+------------------------------+-------------------------------+
|      #  106           | *Seq. mapped in strand Plus* | *Seq. mapped in strand Plus*  |
|                       |             H06870           |          BE952041             |
|                       |           AA598578           |          AI847701             |
|                       |           CB242066           |                               |
|                       |           BU607354           |                               |
|                       | *Seq. mapped in strand Minus*| *Seq. mapped in strand Minus* |
|                       |           AI358408           |          CB572704             |
|                       |           BU688763           |          CN539190             |
|                       |           BU681305           |          CD561110             |
|                       |           AI612733           |          AA475111             |
|                       |           CR598033           |          AA051210             |
|                       |           AI055927           |          BU706151             |
|                       |           AL039031           |          AA930436             |
|                       |           AA825777           |          CD805377             |
|                       |           CB240818           |          AA120731             |
|                       |           BE677339           |            W97108             |
|                       |           AI265899           |          BI695233             |
|                       |           AI217728           |          BX638166             |
|                       |           AW662350           |          AI226368             |
|                       |           BU739523           |          CF545572             |
|                       |           CA448915           |          CF621684             |
|                       |           BE645056           |          BM898630             |
|                       |           BM977850           |          CF755413             |
|                       |           AA393697           |          CK384261             |
|                       |           BM972652           |          CF545107             |
|                       |           AI684589           |          CF926624             |
|                       |           BM679662           |          BM199043             |
|                       |           BM973994           |          BG143406             |
|                       |           CA431179           |          BU529132             |
|                       |           BM978364           |          BU755727             |
|                       |             W94741           |          BB690678             |
|                       |           BM678231           |          BM121923             |
|                       |           BU184975           |          BI715029             |
|                       |             D89678           |          BI789934             |
|                       |           BQ028116           |          CF585479             |
|                       |           CR615922           |          BC021374             |
|                       |           AL528230           |          BB711893             |
|                       |           BX343537           |          CF982778             |
|                       |           AU118804           |          BQ747988             |
|                       |           AI268646           |          CA538217             |
|                       |           AL038153           |          CF383708             |
|                       |           BI254386           |          CF545082             |
|                       |           AU136388           |          CK380901             |
|                       |           BM543330           |          CF383909             |
|                       |           AA393930           |          CF927452             |
|                       |           CD366783           |          BE689559             |
|                       |           CB128402           |          BY582932             |
|                       |           BM920197           |          BE136196             |
|                       |             N54335           |          BF318729             |
|                       |             N71907           |          CA561000             |
|                       |             R36928           |          CK780847             |
|                       |             R43273           |          BY482136             |
|                       |             R44655           |          BB381151             |
|                       |           AI961386           |          CK379118             |
|                       |           BU617936           |          CF423758             |
|                       |           AI510813           |          CF424023             |
|                       |           BU727155           |          AA755194             |
|                       |           BI834643           |          AI155398             |
|                       |           BU679232           |          BY581233             |
|                       |           BF196374           |          BY386124             |
|                       |           BU688193           |          CA322977             |
|                       |           BG291950           |          AA536863             |
|                       |           CB242213           |          AV101245             |
|                       |           AI499671           |          CF544981             |
|                       |           AU157210           |          CF620569             |
|                       |           AA507891           |          AK011767             |
|                       |           AJ710923           |          AB017020             |
|                       |           AW515959           |          BY571135             |
|                       |           AW439834           |          BE851422             |
|                       |           BM986941           |          AA184643             |
|                       |           AA205115           |          AA404178             |
|                       |           AI140123           |          CK376849             |
|                       |           AI431275           |          AA183128             |
|                       |           BM145447           |          CF726132             |
|                       |           AI748788           |          CA558123             |
|                       |           CB242509           |          CB059034             |
|                       |           AI368939           |          CF948862             |
|                       |           AI061231           |          BU840068             |
|                       |           AW007898           |          CF165178             |
|                       |           AI677783           |          BI082584             |
|                       |           CN290360           |          CF161127             |
|                       |           CB119273           |          CA551359             |
|                       |           AA878470           |          CA980331             |
|                       |           AI695445           |          AA124546             |
|                       |           AI475016           |          CA550971             |
|                       |             R36909           |          BE135400             |
|                       |           AI934086           |          BF658535             |
|                       |           BQ027808           |          CA886609             |
|                       |           AI051089           |          AI049195             |
|                       |           AW467737           |          AA003324             |
|                       |           BQ028953           |          AI007232             |
|                       |           AA514037           |          CF161118             |
|                       |           AA598644           |          CD804966             |
|                       |           AW510308           |          CA555578             |
|                       |           AA886589           |          CF153556             |
|                       |           AI560157           |          CF153782             |
|                       |           AA742692           |          CA555412             |
|                       |           AI521367           |          CK391673             |
|                       |           AA504472           |          CD806906             |
|                       |           AI805343           |          CA552055             |
|                       |           AI085274           |          CD806184             |
|                       |           AW152269           |          CF732770             |
|                       |           BM791726           |          BU510172             |
|                       |           AW572171           |          BM949691             |
|                       |           AI274819           |          BI684381             |
|                       |           AI524016           |          CF156124             |
|                       |           CA414599           |          CF159791             |
|                       |           AI003516           |          CF161508             |
|                       |           AI217139           |          CD563584             |
|                       |           AA706245           |          BF168929             |
|                       |           BQ028836           |          BQ179049             |
|                       |           BU729675           |          BF720703             |
|                       |           CA773540           |          BG801229             |
|                       |           AI762552           |          CA548988             |
|                       |           BU616608           |          CF157438             |
|                       |           BU627427           |          CK392253             |
|                       |           BQ009996           |          AA203987             |
|                       |           BM996102           |          BM877840             |
|                       |           BQ007614           |          BU702599             |
|                       |           BQ009046           |          AV142567             |
|                       |           CB957812           |          BY671981             |
|                       |           AI190620           |          AA929394             |
|                       |           AA460092           |          BF714566             |
|                       |           AA639996           |          BU513743             |
|                       |           AI183651           |          BQ109001             |
|                       |           AW009597           |          BI082871             |
|                       |           AI743888           |          BI684330             |
|                       |           AI953547           |          BF657848             |
|                       |           BU734556           |          BG293597             |
|                       |           AA586856           |          BQ714849             |
|                       |           BE328478           |          BG311709             |
|                       |           AA211588           |          AI019790             |
|                       |           BQ270945           |          BU152164             |
|                       |           BF057552           |          BU512335             |
|                       |           BX448970           |          CF160975             |
|                       |           CR607924           |          CF158466             |
|                       |           AI816374           |          BF318730             |
|                       |           BQ231428           |          AI882506             |
|                       |           BG033347           |          BF608866             |
|                       |           AW082460           |          CK339802             |
|                       |           AV718483           |          CD364245             |
|                       |           AI492120           |          BI985225             |
|                       |             X75683           |          CD806743             |
|                       |             W56588           |          BU612710             |
|                       |           BG533104           |          CF725412             |
|                       |           AU158091           |          CN689067             |
|                       |           AA737862           |          CD807448             |
|                       |           AI524588           |          BQ443026             |
|                       |             T59234           |          CF724868             |
|                       |           BU565101           |          BQ964227             |
|                       |           AW271748           |          BU841080             |
|                       |           BU727645           |          AI153097             |
|                       |           AI568512           |          BU554385             |
|                       |           BF941655           |          CA979148             |
|                       |           AA857286           |          CB181426             |
|                       |           AA554550           |          BU147955             |
|                       |           BU615282           |          BQ884816             |
|                       |           AA164817           |          BU519908             |
|                       |           CA444211           |          BQ934160             |
|                       |           AA164821           |          CA562000             |
|                       |           CB113056           |          BU923090             |
|                       |             N59332           |          CA531854             |
|                       |           BM983224           |          CA782110             |
|                       |             N34659           |          BU708469             |
|                       |           AA876954           |          AL024268             |
|                       |           BM673266           |          CB193174             |
|                       |           BQ267854           |          BU925443             |
|                       |           BG113766           |          CK022037             |
|                       |           BM663633           |          BF167187             |
|                       |           AI799688           |            W54578             |
|                       |           CB156580           |          CN531680             |
|                       |           AA977427           |          BF164254             |
|                       |           AW438732           |          BG172865             |
|                       |           BQ775044           |          BI664210             |
|                       |           AA947156           |          BF162743             |
|                       |           AA976279           |          BF159786             |
|                       |           BF941423           |          BG296569             |
|                       |           AW067708           |          BI107513             |
|                       |           BG231687           |          BU707426             |
|                       |           AA046901           |          CF166996             |
|                       |           AI831039           |          CF167013             |
|                       |           BU736122           |          AU079733             |
|                       |           AW661894           |          BY710988             |
|                       |           BI860620           |          AA739298             |
|                       |           BM827233           |          BG297939             |
|                       |           AA974874           |          CK795459             |
|                       |           AI569571           |          BI691300             |
|                       |           BM973022           |          BB841569             |
|                       |           BE222272           |          AA390164             |
|                       |           BM509762           |          CB057417             |
|                       |           AW150168           |          BQ884579             |
|                       |           BU622007           |          CN722675             |
|                       |           BU737056           |          BB657951             |
|                       |           BM989702           |          BG142862             |
|                       |           CA423226           |          AU035956             |
|                       |           CA427685           |          AI549631             |
|                       |           AI955015           |          AI119499             |
|                       |           CB305675           |          CK620554             |
|                       |           BM984537           |          CD561937             |
|                       |           BQ017604           |          CA873736             |
|                       |           BU623448           |          CK628147             |
|                       |           BM979426           |          BI789677             |
|                       |           BM989139           |          BI715671             |
|                       |           BU688211           |          BI690898             |
|                       |           BQ962738           |          CA537472             |
|                       |           BU619214           |          CD565608             |
|                       |           BQ005521           |          AA413966             |
|                       |           BU679115           |          AA413649             |
|                       |           CB529436           |          AI746874             |
|                       |           CD368550           |          CA871578             |
|                       |           BM979163           |          CA543494             |
|                       |           BQ009788           |          CK392973             |
|                       |           CD364798           |          CN688572             |
[truncated: 17,772,040 more chars]
