# Supplementary material for: Sense-antisense pairs in mammals: functional and evolutionary considerations
Source: Genome Biol. 2007 Mar 19;8(3):R40. doi: 10.1186/gb-2007-8-3-r40 (PMC1868933; doi:10.1186/gb-2007-8-3-r40)
Supplement: Additional data file 5 — All S-AS pairs conserved between human and mouse using the three strategies described in the text. [file gb-2007-8-3-r40-S5.zip › Additional_data_file5_736_pairs.doc]

```
Additional data file 5: 736 S-AS pairs conserved between human and mouse.
+-----------------------+------------------------------+-------------------------------+
|   Conserved pairs     |      Human S-AS pairs        |       Mouse S-AS pairs        |
+-----------------------+------------------------------+-------------------------------+
|      #    1           | *Seq. mapped in strand Plus* | *Seq. mapped in strand Plus*  |
|                       |           BC014546           |          BY721383             |
|                       |           BX649080           |                               |
|                       |           AF006082           |                               |
|                       |           BC036253           |                               |
|                       | *Seq. mapped in strand Minus*| *Seq. mapped in strand Minus* |
|                       |           AL832712           |          BE991058             |
+-----------------------+------------------------------+-------------------------------+
|      #    2           | *Seq. mapped in strand Plus* | *Seq. mapped in strand Plus*  |
|                       |           AA356448           |          AK080079             |
|                       |           AF161080           |          BC071186             |
|                       |           BG536800           |                               |
|                       |           BC017812           |                               |
|                       |           BI906978           |                               |
|                       |           BG483194           |                               |
|                       |           BI909772           |                               |
|                       |           BI908633           |                               |
|                       |           BI907215           |                               |
|                       |           AJ400841           |                               |
|                       |           BX100688           |                               |
|                       |           AJ400842           |                               |
|                       |           AJ400843           |                               |
|                       |           BX418631           |                               |
|                       |           AL043122           |                               |
|                       |           CA396159           |                               |
|                       |           AW517488           |                               |
|                       |           AA579555           |                               |
|                       |           AK129528           |                               |
|                       |           BU687075           |                               |
|                       |           AW078572           |                               |
|                       |           BE693545           |                               |
|                       |           BE693551           |                               |
|                       |           BQ303850           |                               |
|                       |           AW834730           |                               |
|                       |           BE693544           |                               |
|                       |           BX430927           |                               |
|                       |           BE327585           |                               |
|                       | *Seq. mapped in strand Minus*| *Seq. mapped in strand Minus* |
|                       |           AA860942           |          AK041670             |
|                       |                              |          AJ400844             |
+-----------------------+------------------------------+-------------------------------+
|      #    3           | *Seq. mapped in strand Plus* | *Seq. mapped in strand Plus*  |
|                       |           AK055176           |          BC025462             |
|                       |           BC004277           |          BC027836             |
|                       |           AK001581           |          AK085572             |
|                       |           AK027564           |                               |
|                       |           AB058697           |                               |
|                       |           BC021859           |                               |
|                       |           CR602910           |                               |
|                       | *Seq. mapped in strand Minus*| *Seq. mapped in strand Minus* |
|                       |           AK130624           |          BC061472             |
|                       |             D84103           |            U53584             |
|                       |             U60325           |          BC053024             |
|                       |           BC050559           |          AB121698             |
|                       |           BC042571           |                               |
|                       |             X98093           |                               |
|                       |           AL110092           |                               |
+-----------------------+------------------------------+-------------------------------+
|      #    4           | *Seq. mapped in strand Plus* | *Seq. mapped in strand Plus*  |
|                       |           BP262047           |          AW048004             |
|                       |           CD707578           |                               |
|                       |           CD691293           |                               |
|                       |           CD689260           |                               |
|                       |           CD704348           |                               |
|                       |           CD684358           |                               |
|                       |           CD691760           |                               |
|                       |           BX486593           |                               |
|                       |           BX479674           |                               |
|                       |           AF186022           |                               |
|                       |           AF163254           |                               |
|                       |           BU198581           |                               |
|                       |           CD521610           |                               |
|                       |           CB159238           |                               |
|                       |           AF150266           |                               |
|                       |           AF161551           |                               |
|                       |           AV740547           |                               |
|                       |           BG341901           |                               |
|                       |           BG758034           |                               |
|                       |           BG685332           |                               |
|                       |           BI908402           |                               |
|                       |           BI224992           |                               |
|                       |           AF178987           |                               |
|                       |           BE244668           |                               |
|                       |           BE247471           |                               |
|                       |           BG288385           |                               |
|                       |           BC012924           |                               |
|                       |           BM922611           |                               |
|                       |           CD365738           |                               |
|                       |           AA251658           |                               |
|                       |             F08128           |                               |
|                       |             R14729           |                               |
|                       |           AA149488           |                               |
|                       |           BM976344           |                               |
|                       |           BX118264           |                               |
|                       |           AA806554           |                               |
|                       |           AA521016           |                               |
|                       |           AA811268           |                               |
|                       |           AA814333           |                               |
|                       |           AL713793           |                               |
|                       |           CN480794           |                               |
|                       |           AA459342           |                               |
|                       |             W58743           |                               |
|                       | *Seq. mapped in strand Minus*| *Seq. mapped in strand Minus* |
|                       |           AA805890           |          AF186023             |
+-----------------------+------------------------------+-------------------------------+
|      #    5           | *Seq. mapped in strand Plus* | *Seq. mapped in strand Plus*  |
|                       |           BX384765           |          BC042738             |
|                       |             Y10479           |                               |
|                       |           BU596688           |                               |
|                       |           CN312805           |                               |
|                       |             D38550           |                               |
|                       |           AI017221           |                               |
|                       |           AW663426           |                               |
|                       |           BC016847           |                               |
|                       |           BM973662           |                               |
|                       |           BG571646           |                               |
|                       |           BU633769           |                               |
|                       |           BM669957           |                               |
|                       |           AI148191           |                               |
|                       |           BU675686           |                               |
|                       |           BM972949           |                               |
|                       |           AA649029           |                               |
|                       |           AW518742           |                               |
|                       |           AI140061           |                               |
|                       | *Seq. mapped in strand Minus*| *Seq. mapped in strand Minus* |
|                       |           CA425435           |          AK129048             |
+-----------------------+------------------------------+-------------------------------+
|      #    6           | *Seq. mapped in strand Plus* | *Seq. mapped in strand Plus*  |
|                       |           BU740932           |          AK080457             |
|                       | *Seq. mapped in strand Minus*| *Seq. mapped in strand Minus* |
|                       |             Z26652           |            X59398             |
|                       |           BU681356           |            M64689             |
|                       |             U02687           |            L36163             |
|                       |           BM144393           |          AK045865             |
|                       |           BC036028           |                               |
|                       |           AI150354           |                               |
|                       |             L36162           |                               |
|                       |           BF176247           |                               |
|                       |           BF170536           |                               |
|                       |           BF170584           |                               |
|                       |           BI461248           |                               |
|                       |           BM147250           |                               |
+-----------------------+------------------------------+-------------------------------+
|      #    7           | *Seq. mapped in strand Plus* | *Seq. mapped in strand Plus*  |
|                       |             X12830           |          AK054125             |
|                       |             X58298           |                               |
|                       |           BC035327           |                               |
|                       |             S72848           |                               |
|                       | *Seq. mapped in strand Minus*| *Seq. mapped in strand Minus* |
|                       |           BC041469           |          AK082270             |
+-----------------------+------------------------------+-------------------------------+
|      #    8           | *Seq. mapped in strand Plus* | *Seq. mapped in strand Plus*  |
|                       |           CR619219           |          AK051140             |
|                       | *Seq. mapped in strand Minus*| *Seq. mapped in strand Minus* |
|                       |           BC078168           |          AK041236             |
|                       |           AF112218           |                               |
|                       |             D64159           |                               |
|                       |           AK024717           |                               |
|                       |           BC015539           |                               |
|                       |           BC060037           |                               |
|                       |           BC014905           |                               |
|                       |           AK025723           |                               |
+-----------------------+------------------------------+-------------------------------+
|      #    9           | *Seq. mapped in strand Plus* | *Seq. mapped in strand Plus*  |
|                       |           AV726649           |          BQ831473             |
|                       |                              |          BB856222             |
|                       |                              |          CN701134             |
|                       |                              |          CB274218             |
|                       |                              |          BY726031             |
|                       |                              |          BG101437             |
|                       |                              |          BY140766             |
|                       |                              |          BY238785             |
|                       |                              |          BY241224             |
|                       |                              |          BY244026             |
|                       |                              |          BY327467             |
|                       |                              |          BB853063             |
|                       |                              |          BI081032             |
|                       |                              |          BX524495             |
|                       |                              |          AA396858             |
|                       |                              |          BY073863             |
|                       |                              |          BB659032             |
|                       |                              |          BY038428             |
|                       |                              |          BB622439             |
|                       |                              |          AK032160             |
|                       |                              |          CA564764             |
|                       |                              |          BY091725             |
|                       |                              |          BY264844             |
|                       |                              |          BY042753             |
|                       |                              |          BY070716             |
|                       |                              |          BY065059             |
|                       |                              |          BY021162             |
|                       |                              |          BY136421             |
|                       |                              |          CB600129             |
|                       |                              |          CD808102             |
|                       |                              |          BI652390             |
|                       |                              |          CN682760             |
|                       |                              |          BB612868             |
|                       |                              |          BI648422             |
|                       |                              |          BY325066             |
|                       |                              |          BB846332             |
|                       |                              |          CA541548             |
|                       |                              |          AW762993             |
|                       |                              |          BF016960             |
|                       |                              |          CN678061             |
|                       |                              |          BI660300             |
|                       |                              |          CK383905             |
|                       |                              |          BF166579             |
|                       |                              |          BC019806             |
|                       |                              |          BG144606             |
|                       |                              |          AA711807             |
|                       |                              |          AK031978             |
|                       |                              |          AK075924             |
|                       |                              |          AW911933             |
|                       |                              |          BE692521             |
|                       |                              |          CB321134             |
|                       |                              |            W97532             |
|                       |                              |          BY357172             |
|                       |                              |          AA241251             |
|                       |                              |          BG172638             |
|                       |                              |          BF019852             |
|                       |                              |          BF729811             |
|                       |                              |          AA197656             |
|                       |                              |          BQ566049             |
|                       |                              |          CK793481             |
|                       | *Seq. mapped in strand Minus*| *Seq. mapped in strand Minus* |
|                       |             H15692           |          AW457254             |
|                       |             R37113           |          AW457541             |
|                       |           BU753667           |          BF463081             |
|                       |           BM662719           |          BE946173             |
|                       |           CN360275           |          BI134090             |
|                       |           CA441421           |          AK048097             |
|                       |           BU625885           |          BC039954             |
|                       |           CD370780           |          BF537633             |
|                       |           CA432647           |          AK017764             |
|                       |           AK022970           |          BU148598             |
|                       |             H18859           |          CA321474             |
|                       |           AI636623           |          CN683532             |
|                       |           AI278614           |          BB647990             |
|                       |           CD367505           |          BB610527             |
|                       |           CA309943           |          BQ033632             |
|                       |           BC073931           |          BG869825             |
|                       |           AK023203           |          BI901734             |
|                       |           AK092040           |          AW049532             |
|                       |           AA721309           |          BX637703             |
|                       |           AA709230           |          CB566485             |
|                       |           AA831174           |          CK788238             |
|                       |           BX393909           |          CA494634             |
|                       |           BU507109           |          CB572853             |
|                       |           AU128882           |          BY455863             |
|                       |           BE891354           |          BB780248             |
|                       |           BE622471           |          BY452766             |
|                       |           BF382742           |          BY461214             |
|                       |           BI754599           |          BG069365             |
|                       |           BU738657           |          BY450212             |
|                       |           AW772312           |          BB752040             |
|                       |           AA808270           |          AW541075             |
|                       |           AW117916           |          BI412504             |
|                       |           BU959664           |          CF617772             |
|                       |           AA777638           |          BM934308             |
|                       |           AI631780           |          BC029910             |
|                       |           BX115852           |          CO039554             |
|                       |           BG724291           |          AK011448             |
|                       |           AA865334           |          BY710720             |
|                       |           AW969395           |          CK793957             |
|                       |           AA888054           |          BI332084             |
|                       |           BG623256           |          CA323058             |
|                       |           AA889324           |          BE310761             |
|                       |           AI991211           |            C88691             |
|                       |           BF512498           |          CN694668             |
|                       |           BQ217553           |          CB058598             |
|                       |           AI341468           |          CF983447             |
|                       |           CA432478           |          CF104674             |
|                       |           BF063630           |          CK333800             |
|                       |           BG687467           |          AA239908             |
|                       |           BU655908           |            W89295             |
|                       |           CD679417           |          BI902015             |
|                       |           BF212223           |          CD565387             |
|                       |           BF126407           |          CB193104             |
|                       |           BU175672           |          AL024120             |
|                       |           CD697874           |          AA028797             |
|                       |           BX486526           |          AA199191             |
|                       |           BG492320           |          AA184808             |
|                       |           AL708904           |          AI181570             |
|                       |           CN349858           |          AA016825             |
|                       |           CN349857           |          AA276213             |
+-----------------------+------------------------------+-------------------------------+
|      #   10           | *Seq. mapped in strand Plus* | *Seq. mapped in strand Plus*  |
|                       |           AL119852           |          AF063229             |
|                       |           BG716859           |          AF063230             |
|                       |           BP230100           |          BC051992             |
|                       |           AK091339           |                               |
|                       |           BC032945           |                               |
|                       |           BP230574           |                               |
|                       |           BI547060           |                               |
|                       |           BI549664           |                               |
|                       |           BC037854           |                               |
|                       |           BC053542           |                               |
|                       |           BI549511           |                               |
|                       |           BP230808           |                               |
|                       |           BM547864           |                               |
|                       |           BI602192           |                               |
|                       |           BI597858           |                               |
|                       |           BI600034           |                               |
|                       |           BI667826           |                               |
|                       |           AF063228           |                               |
|                       |           BI597282           |                               |
|                       |           BX440109           |                               |
|                       |           BM172265           |                               |
|                       |           BG701794           |                               |
|                       |           AF123074           |                               |
|                       |           BC022540           |                               |
|                       |           BX460361           |                               |
|                       |           BC012504           |                               |
|                       |           BG702673           |                               |
|                       |           BM544547           |                               |
|                       |           CV024052           |                               |
|                       |           CV023000           |                               |
|                       |           CV024800           |                               |
|                       |           BP200234           |                               |
|                       |           BP210542           |                               |
|                       |           BX440108           |                               |
|                       |           BQ716072           |                               |
|                       |             R54541           |                               |
|                       |           AA220950           |                               |
|                       |           BX460610           |                               |
|                       |           CB155827           |                               |
|                       |             R26479           |                               |
|                       |             R26506           |                               |
|                       |           AL534296           |                               |
|                       |           BF210939           |                               |
|                       |           BX431094           |                               |
|                       |             T09431           |                               |
|                       |           CB156258           |                               |
|                       |           BG708730           |                               |
|                       |             R35092           |                               |
|                       |           CD618679           |                               |
|                       |           CD618680           |                               |
|                       |             F07633           |                               |
|                       |             R14950           |                               |
|                       |           BG216763           |                               |
|                       |           CK002364           |                               |
|                       |             D56343           |                               |
|                       |           CD673087           |                               |
|                       |           CD580231           |                               |
|                       |             H38479           |                               |
|                       |           BE702680           |                               |
|                       |           AW904921           |                               |
|                       |             F07475           |                               |
|                       |           BP307571           |                               |
|                       |           BX106392           |                               |
|                       |             H05552           |                               |
|                       |             H05560           |                               |
|                       |           AW160882           |                               |
|                       |           BG428905           |                               |
|                       |           BF977411           |                               |
|                       |             H96243           |                               |
|                       |           BU183912           |                               |
|                       |           BG696763           |                               |
|                       |           BQ431093           |                               |
|                       | *Seq. mapped in strand Minus*| *Seq. mapped in strand Minus* |
|                       |             R37690           |          AK006121             |
+-----------------------+------------------------------+-------------------------------+
|      #   11           | *Seq. mapped in strand Plus* | *Seq. mapped in strand Plus*  |
|                       |           BC053557           |          BY722983             |
|                       |           AK091946           |          BY734199             |
|                       |                              |          BY071252             |
|                       |                              |          BY124592             |
|                       |                              |          CB244443             |
|                       |                              |          CD348393             |
|                       |                              |          BY789435             |
|                       |                              |          BB867000             |
|                       |                              |          BU702045             |
|                       |                              |          CD350228             |
|                       |                              |          BY232895             |
|                       |                              |          BY020505             |
|                       |                              |          BQ180575             |
|                       |                              |          CB247724             |
|                       |                              |          CA328686             |
|                       |                              |          CB525431             |
|                       |                              |          BQ771143             |
|                       |                              |          BF319949             |
|                       |                              |          BE986479             |
|                       |                              |          AI851301             |
|                       |                              |          CF895287             |
|                       |                              |          CB248039             |
|                       |                              |          AI854009             |
|                       |                              |          BY261632             |
|                       |                              |          CB248122             |
|                       |                              |          BQ712950             |
|                       |                              |          CF536932             |
|                       |                              |          AA050136             |
|                       |                              |          BC066011             |
|                       |                              |          CB183781             |
|                       |                              |          AA051715             |
|                       |                              |          CN527615             |
|                       |                              |          CF739912             |
|                       |                              |          AI158096             |
|                       |                              |          CN676354             |
|                       |                              |          CD544921             |
|                       |                              |          CD350239             |
|                       |                              |          BC030410             |
|                       |                              |          BI736348             |
|                       |                              |          CF892732             |
|                       |                              |          BE571101             |
|                       |                              |          BU708329             |
|                       |                              |          BI555904             |
|                       |                              |          CF736101             |
|                       |                              |          BE572731             |
|                       |                              |          CN703638             |
|                       |                              |          BU613782             |
|                       | *Seq. mapped in strand Minus*| *Seq. mapped in strand Minus* |
|                       |           AB044561           |          AW120896             |
|                       |           AF116545           |          AW413140             |
|                       |           AF116548           |          AW700937             |
|                       |           AF116547           |          BM226244             |
|                       |           AK122681           |          AI043109             |
|                       |           BC052249           |          BF782362             |
|                       |                              |          AI605786             |
|                       |                              |          AU018875             |
|                       |                              |          AW764187             |
|                       |                              |          BF228005             |
|                       |                              |          CD353332             |
|                       |                              |          AY033912             |
|                       |                              |          AI929965             |
|                       |                              |          AI891511             |
|                       |                              |          AA261365             |
|                       |                              |          BI218641             |
|                       |                              |          AA546528             |
|                       |                              |          BG969589             |
|                       |                              |          BI685628             |
|                       |                              |          BQ944745             |
|                       |                              |          BI143674             |
|                       |                              |          AA244956             |
|                       |                              |          AW610920             |
|                       |                              |          AA388582             |
|                       |                              |          AA199449             |
|                       |                              |          CN696550             |
|                       |                              |          BI556022             |
|                       |                              |          BI526278             |
|                       |                              |          BF780967             |
|                       |                              |          BF142970             |
|                       |                              |          BF786332             |
|                       |                              |          AW909655             |
|                       |                              |          BF536812             |
|                       |                              |          BU614063             |
|                       |                              |          BF780137             |
|                       |                              |          BQ827890             |
|                       |                              |          BE911099             |
|                       |                              |          BE648987             |
|                       |                              |          BI107619             |
|                       |                              |          BQ831920             |
|                       |                              |          CB948113             |
|                       |                              |          BI687645             |
|                       |                              |          CB950665             |
|                       |                              |          BI330614             |
|                       |                              |          BI556264             |
|                       |                              |          BI101433             |
|                       |                              |          CD241644             |
+-----------------------+------------------------------+-------------------------------+
|      #   12           | *Seq. mapped in strand Plus* | *Seq. mapped in strand Plus*  |
|                       |           AV646526           |          AK038799             |
|                       |           AI290349           |                               |
|                       |           AV646490           |                               |
|                       | *Seq. mapped in strand Minus*| *Seq. mapped in strand Minus* |
|                       |           AA262770           |            M35525             |
|                       |           AW016456           |            M35526             |
|                       |           AI300278           |          BC003963             |
|                       |           BM979143           |          AK080166             |
|                       |           CB250401           |                               |
|                       |           BG618119           |                               |
|                       |           AI522067           |                               |
|                       |           AI768556           |                               |
|                       |           AW269380           |                               |
|                       |           AW052047           |                               |
|                       |             M65134           |                               |
|                       |             M57729           |                               |
|                       |           AA788924           |                               |
|                       |           BG533927           |                               |
|                       |           BX395430           |                               |
|                       |           AV721146           |                               |
|                       |           BU739576           |                               |
|                       |           AA299200           |                               |
|                       |           AA329952           |                               |
|                       |           CB162771           |                               |
|                       |           AA262851           |                               |
|                       |           BI015709           |                               |
|                       |           BX494534           |                               |
|                       |           AI685025           |                               |
|                       |           BX117573           |                               |
|                       |           BE827095           |                               |
|                       |           BE927944           |                               |
|                       |           BQ643579           |                               |
|                       |           BX391630           |                               |
|                       |           BI015591           |                               |
|                       |             W78787           |                               |
|                       |           BG176869           |                               |
|                       |           CB135782           |                               |
|                       |           CB135176           |                               |
|                       |           CB146590           |                               |
|                       |           CB121470           |                               |
|                       |           CB148868           |                               |
|                       |           CB135790           |                               |
|                       |           CB135798           |                               |
|                       |           CB137772           |                               |
|                       |           CB147203           |                               |
|                       |           CB121430           |                               |
|                       |           CB141219           |                               |
|                       |           BI015436           |                               |
|                       |           CB138494           |                               |
|                       |           CB139144           |                               |
|                       |           CB121420           |                               |
|                       |           CB141230           |                               |
|                       |           CB163560           |                               |
|                       |           CB133179           |                               |
|                       |           CB138210           |                               |
|                       |           CB138223           |                               |
|                       |             N50222           |                               |
|                       |           BP277518           |                               |
|                       |           BP237930           |                               |
|                       |           BP240661           |                               |
|                       |           BP243733           |                               |
|                       |             R29419           |                               |
|                       |           BP240525           |                               |
|                       |           AV648490           |                               |
|                       |           AV648298           |                               |
|                       |           AL702568           |                               |
|                       |           AV653516           |                               |
|                       |           BP241927           |                               |
|                       |           CN261398           |                               |
|                       |           BP238530           |                               |
|                       |           BP238080           |                               |
|                       |             T82068           |                               |
|                       |           AV691900           |                               |
|                       |           BG900837           |                               |
|                       |             N73030           |                               |
|                       |           BC022299           |                               |
|                       |             T87395           |                               |
|                       |           AV651321           |                               |
|                       |           BX495684           |                               |
|                       |           BX502778           |                               |
|                       |           BX503344           |                               |
|                       |           AL703711           |                               |
|                       |           BG569142           |                               |
|                       |           BX495412           |                               |
|                       |           AV660086           |                               |
|                       |           AV660268           |                               |
+-----------------------+------------------------------+-------------------------------+
|      #   13           | *Seq. mapped in strand Plus* | *Seq. mapped in strand Plus*  |
|                       |           BU101735           |          BY258044             |
|                       |                              |          BY264506             |
|                       |                              |          BY108619             |
|                       |                              |          BY108366             |
|                       |                              |          BB856057             |
|                       |                              |          CA328963             |
|                       |                              |          BC057338             |
|                       |                              |          CB246676             |
|                       |                              |          BB651759             |
|                       |                              |          AK049192             |
|                       |                              |          BU511265             |
|                       |                              |          BY126008             |
|                       |                              |          BY770441             |
|                       |                              |          BB840352             |
|                       |                              |          BY793567             |
|                       |                              |          CD347926             |
|                       |                              |          CD349905             |
|                       |                              |          CD347846             |
|                       |                              |          CB527320             |
|                       |                              |          CN531782             |
|                       |                              |          AF073788             |
|                       |                              |          CD348909             |
|                       |                              |          BQ771025             |
|                       |                              |          BB855553             |
|                       |                              |          BB855968             |
|                       |                              |          BB857853             |
|                       |                              |          CA325243             |
|                       |                              |          CA327416             |
|                       |                              |          CB247249             |
|                       |                              |          AF205889             |
|                       |                              |          CN531262             |
|                       |                              |          CB247359             |
|                       |                              |          BU702399             |
|                       |                              |          BU052872             |
|                       |                              |          CB249197             |
|                       |                              |          CA315703             |
|                       |                              |          BU515508             |
|                       |                              |          BQ770977             |
|                       |                              |          BU057755             |
|                       |                              |          BQ769357             |
|                       |                              |          BQ572550             |
|                       |                              |          BQ573015             |
|                       |                              |          AK084644             |
|                       |                              |          BB659169             |
|                       |                              |          BU840368             |
|                       |                              |          BF021386             |
|                       |                              |          CK022359             |
|                       | *Seq. mapped in strand Minus*| *Seq. mapped in strand Minus* |
|                       |           BM993271           |          AW762829             |
|                       |           AB052751           |          BX529883             |
|                       |           AA573711           |                               |
|                       |           AI672742           |                               |
|                       |           BE219635           |                               |
|                       |           AA772408           |                               |
|                       |           BE670767           |                               |
|                       |           AA917337           |                               |
|                       |           BM974846           |                               |
|                       |           CB306601           |                               |
|                       |           CK300336           |                               |
|                       |           BM974639           |                               |
|                       |           CA944713           |                               |
|                       |           BQ005585           |                               |
|                       |           AF078165           |                               |
|                       |           BM474589           |                               |
|                       |           BM473985           |                               |
|                       |           BM770270           |                               |
|                       |           AK025718           |                               |
|                       |           BM745159           |                               |
|                       |           BM771508           |                               |
|                       |           BP376354           |                               |
|                       |           BP293188           |                               |
|                       |           BP293526           |                               |
|                       |           AA446378           |                               |
|                       |           AF205888           |                               |
|                       |           BE882875           |                               |
|                       |           AW962356           |                               |
|                       |           CN388942           |                               |
|                       |           AA305361           |                               |
|                       |           AA502360           |                               |
|                       |           BF436343           |                               |
|                       |           AI383206           |                               |
|                       |           AA903395           |                               |
|                       |           AI912345           |                               |
|                       |           AI262837           |                               |
|                       |           CN302754           |                               |
|                       |           BM543373           |                               |
|                       |           AI336613           |                               |
|                       |           AI307112           |                               |
|                       |           AW207598           |                               |
|                       |           BX369419           |                               |
|                       |           BI489101           |                               |
|                       |           AI742962           |                               |
|                       |           AI660139           |                               |
|                       |           AI858567           |                               |
|                       |           AI272864           |                               |
|                       |           AI419942           |                               |
|                       |           AA884421           |                               |
|                       |           AA489644           |                               |
|                       |           AI363868           |                               |
|                       |           AI220032           |                               |
|                       |           AI818109           |                               |
|                       |           AA937585           |                               |
|                       |           CN302752           |                               |
|                       |           CN302753           |                               |
|                       |           AA489748           |                               |
|                       |           BF812711           |                               |
|                       |           BF757617           |                               |
|                       |           BE872155           |                               |
|                       |           BG984045           |                               |
+-----------------------+------------------------------+-------------------------------+
|      #   14           | *Seq. mapped in strand Plus* | *Seq. mapped in strand Plus*  |
|                       |           BC051830           |          BI986048             |
|                       |             Y08639           |                               |
|                       |             F05544           |                               |
|                       |             T26966           |                               |
|                       |           BX647070           |                               |
|                       |           CN354597           |                               |
|                       |           AL550103           |                               |
|                       |           BX350110           |                               |
|                       |           CR594193           |                               |
|                       |           BX348312           |                               |
|                       |           BP294007           |                               |
|                       |           AL550102           |                               |
|                       | *Seq. mapped in strand Minus*| *Seq. mapped in strand Minus* |
|                       |           BI520565           |          BC058269             |
+-----------------------+------------------------------+-------------------------------+
|      #   15           | *Seq. mapped in strand Plus* | *Seq. mapped in strand Plus*  |
|                       |           CA440446           |            U65986             |
|                       |                              |          AK044203             |
|                       |                              |          BC012875             |
|                       |                              |          AK086039             |
|                       | *Seq. mapped in strand Minus*| *Seq. mapped in strand Minus* |
|                       |           AA081227           |          AK038929             |
|                       |           AJ278463           |                               |
|                       |           AJ278464           |                               |
|                       |           AJ278465           |                               |
|                       |           CA436421           |                               |
|                       |           BU683828           |                               |
|                       |           AL357617           |                               |
|                       |           AI942427           |                               |
|                       |           AW444995           |                               |
|                       |           CD368461           |                               |
|                       |           AW445021           |                               |
|                       |           BM990733           |                               |
|                       |           CA398113           |                               |
|                       |           BI833338           |                               |
|                       |           AI749090           |                               |
|                       |           AL572277           |                               |
|                       |           CR604152           |                               |
|                       |           AW439661           |                               |
|                       |           AU148529           |                               |
|                       |           AI199108           |                               |
|                       |           AA534886           |                               |
|                       |           AA586694           |                               |
|                       |           AI093090           |                               |
|                       |           AL521741           |                               |
|                       |           BG168446           |                               |
|                       |           BU163306           |                               |
|                       |           BU168513           |                               |
|                       |           AI027231           |                               |
|                       |           BX373608           |                               |
|                       |           AL574465           |                               |
|                       |           BM828919           |                               |
|                       |           AL547276           |                               |
|                       |           AI275821           |                               |
|                       |           BQ021939           |                               |
|                       |           BQ021961           |                               |
|                       |           CD365212           |                               |
|                       |           BM150841           |                               |
|                       |             R20535           |                               |
|                       |           CF130571           |                               |
|                       |           AA972367           |                               |
|                       |           BM145243           |                               |
|                       |           AI923739           |                               |
|                       |           BE676542           |                               |
|                       |           BE676596           |                               |
|                       |           AA976860           |                               |
|                       |           BM994756           |                               |
|                       |           BU680928           |                               |
|                       |           AI888344           |                               |
|                       |           AK056107           |                               |
|                       |           AI051762           |                               |
|                       |           BF941419           |                               |
|                       |           AW338364           |                               |
|                       |           AI273872           |                               |
|                       |           BI493462           |                               |
|                       |             W46456           |                               |
|                       |           BG260917           |                               |
|                       |           CA447786           |                               |
|                       |           AJ572783           |                               |
|                       |           AA086312           |                               |
|                       |             W47210           |                               |
|                       |           AW149335           |                               |
|                       |           BM691491           |                               |
|                       |           AI094978           |                               |
|                       |           AI564321           |                               |
|                       |           AI936980           |                               |
|                       |           AA890522           |                               |
|                       |           AA617811           |                               |
|                       |           BG829145           |                               |
|                       |             H00663           |                               |
|                       |           AI818773           |                               |
|                       |           AI453197           |                               |
|                       |           CA434676           |                               |
|                       |           AW438873           |                               |
|                       |           BM673706           |                               |
|                       |           AI369447           |                               |
|                       |           AI631338           |                               |
|                       |           AW244065           |                               |
|                       |           BU733256           |                               |
|                       |           AI521234           |                               |
|                       |           AA464982           |                               |
|                       |           AW468434           |                               |
|                       |           AI082489           |                               |
|                       |           AI126927           |                               |
|                       |           AW473750           |                               |
|                       |           AA759330           |                               |
|                       |           AI890703           |                               |
|                       |             T64467           |                               |
|                       |           AI571558           |                               |
|                       |           BQ007541           |                               |
|                       |           AK000567           |                               |
|                       |           AI192223           |                               |
|                       |           AJ346460           |                               |
|                       |           AI827279           |                               |
|                       |           AI745430           |                               |
|                       |             H50866           |                               |
|                       |           BG288736           |                               |
|                       |           BE294577           |                               |
|                       |           BG287231           |                               |
|                       |           BG114636           |                               |
|                       |           AA878176           |                               |
|                       |           BQ006157           |                               |
|                       |           AA938678           |                               |
|                       |           AA740534           |                               |
|                       |           AI348621           |                               |
|                       |           AW438753           |                               |
|                       |           AW471295           |                               |
|                       |           AW172867           |                               |
|                       |           AA639403           |                               |
|                       |           BM662425           |                               |
|                       |           AI827022           |                               |
|                       |           BM996579           |                               |
|                       |           CD368660           |                               |
|                       |           CA434315           |                               |
|                       |           BU617214           |                               |
|                       |           CA307561           |                               |
|                       |           BU732715           |                               |
|                       |           AK126287           |                               |
|                       |           AI433142           |                               |
|                       |           AA079355           |                               |
|                       |           BE301251           |                               |
|                       |           AI094814           |                               |
|                       |           AI708310           |                               |
|                       |             R20781           |                               |
|                       |           AA197156           |                               |
|                       |           AA745666           |                               |
|                       |           AA664142           |                               |
|                       |           AI361942           |                               |
|                       |           AI863422           |                               |
|                       |           AA580351           |                               |
|                       |           AU159328           |                               |
|                       |           AA142953           |                               |
|                       |             H93114           |                               |
|                       |           AA554198           |                               |
|                       |           BM994744           |                               |
|                       |           AA528161           |                               |
|                       |           BQ017582           |                               |
|                       |           BM989681           |                               |
|                       |           AA542988           |                               |
|                       |           AA477504           |                               |
|                       |             F20763           |                               |
|                       |           AW966737           |                               |
|                       |           AI744815           |                               |
|                       |           BM988816           |                               |
|                       |             N93591           |                               |
|                       |           AA871988           |                               |
|                       |           AI140209           |                               |
|                       |           AI597954           |                               |
|                       |           AI935881           |                               |
|                       |           BM673474           |                               |
|                       |           AA401460           |                               |
|                       |           AI744824           |                               |
|                       |           BE546396           |                               |
|                       |           BE046529           |                               |
|                       |           BU532349           |                               |
|                       |           BF969328           |                               |
|                       |           AI081122           |                               |
|                       |           BG036844           |                               |
|                       |             N85103           |                               |
|                       |             L19605           |                               |
|                       |           BU734378           |                               |
|                       |           AI004322           |                               |
|                       |           BE501587           |                               |
|                       |           AW575040           |                               |
|                       |           AU157810           |                               |
|                       |           AI653429           |                               |
|                       |             W15501           |                               |
|                       |             T27638           |                               |
|                       |           BQ230394           |                               |
|                       |           AW263472           |                               |
|                       |             W72854           |                               |
|                       |             R64128           |                               |
|                       |           AA116066           |                               |
|                       |           AI214498           |                               |
|                       |           AI422396           |                               |
|                       |           AI400723           |                               |
|                       |           AI961433           |                               |
|                       |           CD366751           |                               |
|                       |           BG529682           |                               |
|                       |           BM988540           |                               |
|                       |           AA286872           |                               |
|                       |           AW673035           |                               |
|                       |           AW029334           |                               |
|                       |           AI749956           |                               |
|                       |           AU148449           |                               |
|                       |           AW070531           |                               |
|                       |           AW516890           |                               |
|                       |           BF983022           |                               |
|                       |           AA455526           |                               |
|                       |           AI864766           |                               |
|                       |           BU157213           |                               |
|                       |           BG231573           |                               |
|                       |           BM997244           |                               |
|                       |           BM789753           |                               |
|                       |           CA842903           |                               |
|                       |           BM994212           |                               |
|                       |           CA439728           |                               |
|                       |           BU689448           |                               |
|                       |           CA434409           |                               |
|                       |           BU619120           |                               |
|                       |           CB242543           |                               |
|                       |           BM974495           |                               |
|                       |           CA311967           |                               |
|                       |           AI354327           |                               |
|                       |           BM473216           |                               |
|                       |           BM921912           |                               |
|                       |           CB242237           |                               |
|                       |           CK724979           |                               |
|                       |           BQ945548           |                               |
|                       |           BC007564           |                               |
|                       |           AI354740           |                               |
|                       |           BG030879           |                               |
|                       |           BE302216           |                               |
|                       |           BE018638           |                               |
|                       |           BX435685           |                               |
|                       |           BX329472           |                               |
|                       |           BX374137           |                               |
|                       |             R80733           |                               |
|                       |             H89970           |                               |
|                       |             F27041           |                               |
|                       |           AA477080           |                               |
|                       |           BU943489           |                               |
|                       |             W46524           |                               |
|                       |           BF086559           |                               |
|                       |           CR592506           |                               |
|                       |             R71260           |                               |
|                       |             H00174           |                               |
|                       |             H52252           |                               |
|                       |           BF095459           |                               |
|                       |           BX373758           |                               |
|                       |           BF690132           |                               |
|                       |             H01202           |                               |
|                       |             H69886           |                               |
|                       |           BX399466           |                               |
|                       |           BX344195           |                               |
|                       |           BM808121           |                               |
|                       |           AL576782           |                               |
|                       |           AW351867           |                               |
|                       |           BG253713           |                               |
|                       |           AL529213           |                               |
|                       |           AL574362           |                               |
|                       |           AL582473           |                               |
|                       |           BX324625           |                               |
|                       |           CR591981           |                               |
|                       |             H67237           |                               |
|                       |           BF207899           |                               |
|                       |           AA576064           |                               |
|                       |           AA834319           |                               |
|                       |           AL572427           |                               |
|                       |           AW578026           |                               |
|                       |           AL564876           |                               |
|                       |           BE935065           |                               |
|                       |           BI859946           |                               |
|                       |           BG992873           |                               |
|                       |           BE546678           |                               |
|                       |           BF967813           |                               |
|                       |           AA393912           |                               |
|                       |           BF340085           |                               |
|                       |           CR606218           |                               |
|                       |           AL527256           |                               |
|                       |           AW881241           |                               |
|                       |           BG163238           |                               |
|                       |           CR621013           |                               |
|                       |           BE270931           |                               |
|                       |           BI028369           |                               |
|                       |           AL574805           |                               |
|                       |           BF349339           |                               |
|                       |             R28247           |                               |
|                       |           BG492060           |                               |
|                       |           BI520687           |                               |
|                       |           BM808327           |                               |
|                       |             H00758           |                               |
|                       |             H52349           |                               |
|                       |           AL575101           |                               |
|                       |           BE815125           |                               |
|                       |           BI252668           |                               |
|                       |           AL577555           |                               |
|                       |           AA658371           |                               |
|                       |           AA665601           |                               |
|                       |           AA748580           |                               |
|                       |           AA837092           |                               |
|                       |           BG009729           |                               |
|                       |           AW351858           |                               |
|                       |           BI253043           |                               |
|                       |             F18856           |                               |
|                       |           BE171174           |                               |
|                       |           AW273540           |                               |
|                       |           BG338182           |                               |
|                       |           BE815120           |                               |
|                       |           BG530433           |                               |
|                       |           AL524803           |                               |
|                       |           AA327170           |                               |
|                       |           AW750235           |                               |
|                       |           BE835683           |                               |
|                       |           BF089805           |                               |
|                       |           AA325274           |                               |
|                       |           AA328508           |                               |
|                       |           BG180804           |                               |
|                       |           AA878700           |                               |
|                       |           BG179902           |                               |
|                       |           BG255652           |                               |
|                       |           AW500315           |                               |
|                       |           BM794346           |                               |
|                       |           BE535455           |                               |
|                       |           BF340901           |                               |
|                       |           BE076390           |                               |
|                       |           BE077384           |                               |
|                       |           AA296105           |                               |
|                       |           BF084035           |                               |
|                       |           BE720268           |                               |
|                       |           BF084026           |                               |
|                       |           AA149714           |                               |
|                       |           AJ710540           |                               |
|                       |           BG747480           |                               |
|                       |           AA410704           |                               |
|                       |             H50865           |                               |
|                       |           AW877267           |                               |
|                       |           BM912338           |                               |
|                       |           BF037709           |                               |
|                       |           BG289055           |                               |
|                       |           BE835684           |                               |
|                       |           BM833229           |                               |
|                       |           BI011114           |                               |
|                       |           AW389238           |                               |
|                       |           CD637428           |                               |
|                       |           BF248326           |                               |
|                       |           BE868613           |                               |
|                       |           AW796851           |                               |
|                       |           BG391087           |                               |
|                       |           AA359806           |                               |
|                       |           AA313795           |                               |
|                       |           BM009816           |                               |
|                       |           BP431159           |                               |
|                       |           CN483314           |                               |
|                       |           AA384118           |                               |
|                       |           BP430306           |                               |
|                       |           AA477068           |                               |
|                       |           CR450323           |                               |
|                       |           AA421635           |                               |
|                       |           AA631633           |                               |
|                       |           AA116048           |                               |
|                       |           AI041949           |                               |
|                       |           AW675266           |                               |
|                       |           AA128475           |                               |
|                       |           AA301585           |                               |
|                       |           AA368807           |                               |
|                       |           AA428009           |                               |
|                       |           BM842443           |                               |
|                       |           AI478734           |                               |
|                       |           CF128279           |                               |
|                       |           BG122310           |                               |
|                       |           AA150663           |                               |
|                       |           BQ083135           |                               |
|                       |             R36432           |                               |
|                       |           CD701493           |                               |
|                       |           BM831861           |                               |
|                       |           CB160137           |                               |
|                       |           BM472364           |                               |
|                       |           BQ669810           |                               |
|                       |           BF349340           |                               |
|                       |           AA372170           |                               |
|                       |             R20888           |                               |
|                       |             H00221           |                               |
|                       |             R73283           |                               |
|                       |             N31404           |                               |
|                       |           BM709344           |                               |
|                       |           BQ213119           |                               |
|                       |           BM831906           |                               |
|                       |           BM789975           |                               |
|                       |             T88990           |                               |
|                       |           BM843870           |                               |
|                       |             W25441           |                               |
|                       |             R36192           |                               |
|                       |           BE180710           |                               |
|                       |             R71315           |                               |
|                       |           AW843160           |                               |
|                       |           BF822844           |                               |
|                       |           BF823161           |                               |
|                       |           AW802639           |                               |
|                       |             W47110           |                               |
|                       |           AA363111           |                               |
|                       |           BI860603           |                               |
|                       |             N87771           |                               |
|                       |             D58453           |                               |
|                       |             W39722           |                               |
|                       |             H93242           |                               |
|                       |             W05815           |                               |
|                       |           CB128676           |                               |
|                       |           AA340502           |                               |
|                       |           BM707219           |                               |
|                       |           CB119882           |                               |
|                       |           BM846595           |                               |
|                       |           CB118640           |                               |
|                       |           CB122502           |                               |
|                       |           CB160030           |                               |
|                       |           AA384611           |                               |
|                       |           BM707363           |                               |
|                       |           AW862523           |                               |
|                       |           CB163360           |                               |
|                       |           BM833370           |                               |
|                       |           BF997292           |                               |
|                       |             T48665           |                               |
|                       |           AA197140           |                               |
|                       |           BM832146           |                               |
|                       |           CB164540           |                               |
|                       |           AA356512           |                               |
|                       |           AA362413           |                               |
|                       |             R75682           |                               |
|                       |             T64607           |                               |
|                       |           BQ084058           |                               |
|                       |             W76041           |                               |
|                       |           CB163041           |                               |
|                       |           AA079594           |                               |
|                       |             H45539           |                               |
|                       |             H01300           |                               |
|                       |             H67420           |                               |
|                       |           BM707734           |                               |
|                       |           BE910260           |                               |
|                       |           AA034155           |                               |
|                       |             H81124           |                               |
|                       |           AA356960           |                               |
|                       |           BI910869           |                               |
|                       |           BG419428           |                               |
|                       |           BM045687           |                               |
|                       |           BM920087           |                               |
|                       |           AL550987           |                               |
|                       |           AL550718           |                               |
|                       |           BM918471           |                               |
|                       |           AA055714           |                               |
|                       |             Z21711           |                               |
|                       |             H69885           |                               |
|                       |           AL702126           |                               |
|                       |           BM811116           |                               |
|                       |           BX345589           |                               |
|                       |           BQ053996           |                               |
|                       |           AL550332           |                               |
|                       |           BX343260           |                               |
|                       |           AL547539           |                               |
|                       |           BG679353           |                               |
|                       |           CB155486           |                               |
|                       |           AL702125           |                               |
|                       |           AL529214           |                               |
|                       |           AA312231           |                               |
|                       |           BM047798           |                               |
|                       |           BX461987           |                               |
|                       |           AL551575           |                               |
|                       |           BX377798           |                               |
|                       |           BQ228831           |                               |
|                       |           BI333112           |                               |
|                       |           BM810797           |                               |
|                       |           BM910409           |                               |
|                       |           AL550520           |                               |
|                       |           BX344196           |                               |
|                       |           BU507881           |                               |
|                       |           BM921739           |                               |
|                       |           BQ430256           |                               |
|                       |           AL513642           |                               |
|                       |           AL521742           |                               |
|                       |           CF131506           |                               |
|                       |           BE513759           |                               |
|                       |           CV027071           |                               |
|                       |           BM915159           |                               |
|                       |           BE615668           |                               |
|                       |           BE562420           |                               |
|                       |           CD172071           |                               |
|                       |           BM918279           |                               |
|                       |           BI759496           |                               |
|                       |           BQ936596           |                               |
|                       |           BM544436           |                               |
|                       |           BX399678           |                               |
|                       |           BM548766           |                               |
|                       |           BU194524           |                               |
|                       |           BM552800           |                               |
|                       |           BM921072           |                               |
|                       |           BG252381           |                               |
|                       |           BI756941           |                               |
|                       |           BE737029           |                               |
|                       |           BI520480           |                               |
|                       |           BI821418           |                               |
|                       |           BU856284           |                               |
|                       |           BX395497           |                               |
|                       |           AU124731           |                               |
|                       |           BI761216           |                               |
|                       |           BQ690816           |                               |
|                       |           BQ064522           |                               |
|                       |           BX398722           |                               |
|                       |           BQ643792           |                               |
|                       |           BE530990           |                               |
|                       |           BX353515           |                               |
|                       |           BE397430           |                               |
|                       |           BE397401           |                               |
|                       |           BG037011           |                               |
|                       |           AU124622           |                               |
|                       |           AU122096           |                               |
|                       |           BI490028           |                               |
|                       |           BI518437           |                               |
|                       |           BM925104           |                               |
|                       |           BQ072986           |                               |
|                       |           CB133412           |                               |
|                       |           AU141454           |                               |
|                       |           BE901419           |                               |
|                       |           BQ898712           |                               |
|                       |           AU141879           |                               |
|                       |           BE397602           |                               |
|                       |           BQ962587           |                               |
|                       |           BE514361           |                               |
|                       |           BI862199           |                               |
|                       |           BG761842           |                               |
|                       |           BI756938           |                               |
|                       |           BI838273           |                               |
|                       |           BE898143           |                               |
|                       |           AU135673           |                               |
|                       |           BE886690           |                               |
|                       |           BM559242           |                               |
|                       |           BI560343           |                               |
|                       |           BI252213           |                               |
|                       |           BX331865           |                               |
|                       |           BG761753           |                               |
|                       |           BE883635           |                               |
|                       |           BM739066           |                               |
|                       |           BE728753           |                               |
|                       |           BU159518           |                               |
|                       |           BG116525           |                               |
|                       |           CN344621           |                               |
|                       |           BG707711           |                               |
|                       |           BG342201           |                               |
|                       |           CF131318           |                               |
|                       |           BQ651352           |                               |
|                       |           BE887180           |                               |
|                       |           BG386511           |                               |
|                       |           BI489502           |                               |
|                       |           BM771111           |                               |
|                       |           BI192666           |                               |
|                       |           BE279022           |                               |
|                       |           CB127843           |                               |
|                       |           BE870536           |                               |
|                       |           BI754886           |                               |
|                       |           CB331917           |                               |
|                       |           CB331918           |                               |
|                       |           AU136402           |                               |
|                       |           BM748834           |                               |
|                       |           CB155432           |                               |
|                       |           BI764243           |                               |
|                       |           BM710544           |                               |
|                       |             R12634           |                               |
|                       |           BP272263           |                               |
|                       |           CD110085           |                               |
|                       |           CB991769           |                               |
|                       |           BE278279           |                               |
|                       |           CA395922           |                               |
|                       |           BM787463           |                               |
|                       |           BM747962           |                               |
|                       |           BG479678           |                               |
|                       |           CN419816           |                               |
|                       |           AI750414           |                               |
|                       |           AA406017           |                               |
|                       |           BM750759           |                               |
|                       |           BM760904           |                               |
|                       |           CB997458           |                               |
|                       |             D58441           |                               |
|                       |           AW850073           |                               |
|                       |           BM744998           |                               |
|                       |           AW401873           |                               |
|                       |           BM194009           |                               |
|                       |           BM151753           |                               |
|                       |           AA464411           |                               |
|                       |           BM146266           |                               |
|                       |           BM146268           |                               |
+-----------------------+------------------------------+-------------------------------+
|      #   16           | *Seq. mapped in strand Plus* | *Seq. mapped in strand Plus*  |
|                       |           AK024552           |          BC010190             |
|                       |           AL136640           |          AK005462             |
|                       |           BC001716           |          AK009909             |
|                       |           CR592408           |          AK008252             |
|                       |           CR611769           |          AK012839             |
|                       |           CR626771           |                               |
|                       |           AF151052           |                               |
|                       |           CR600873           |                               |
|                       |           CR593605           |                               |
|                       |           BC007764           |                               |
|                       |           AF317675           |                               |
|                       |           BC048106           |                               |
|                       |           BC062718           |                               |
|                       |           BC039337           |                               |
|                       |           CR533489           |                               |
|                       | *Seq. mapped in strand Minus*| *Seq. mapped in strand Minus* |
|                       |           AK092655           |          AK085499             |
|                       |           AF098277           |          BC013528             |
|                       |           BC050261           |          AF058318             |
|                       |           BC019225           |                               |
|                       |           AF170911           |                               |
|                       |           AF058317           |                               |
|                       |           AJ269477           |                               |
|                       |           AJ250807           |                               |
+-----------------------+------------------------------+-------------------------------+
|      #   17           | *Seq. mapped in strand Plus* | *Seq. mapped in strand Plus*  |
|                       |           BC057396           |          BB859870             |
|                       |           AF147747           |          BB860070             |
|                       |           BX648877           |          CD539046             |
|                       |           BX647198           |          BY312557             |
|                       |                              |          BY312762             |
|                       |                              |          BY353173             |
|                       |                              |          BY299703             |
|                       |                              |          BY091070             |
|                       |                              |          BY094029             |
|                       |                              |          BY337582             |
|                       |                              |          BY336947             |
|                       |                              |          BY060679             |
|                       |                              |          BY251815             |
|                       |                              |          BB857526             |
|                       |                              |          AW320673             |
|                       |                              |          BB851516             |
|                       |                              |          CD550552             |
|                       |                              |          CD545016             |
|                       |                              |          BB853286             |
|                       |                              |          CD549420             |
|                       |                              |          BB638185             |
|                       |                              |          BB623039             |
|                       |                              |          BB619311             |
|                       |                              |          BB634519             |
|                       |                              |          CF906348             |
|                       |                              |          BB628928             |
|                       |                              |          BB613158             |
|                       |                              |          BF099125             |
|                       |                              |          BI554760             |
|                       |                              |          CA976951             |
|                       |                              |          AK028826             |
|                       |                              |          AK032634             |
|                       |                              |          AK041504             |
|                       |                              |          BC012284             |
|                       |                              |          CF914648             |
|                       |                              |          CF174277             |
|                       |                              |          BI854483             |
|                       |                              |          BG918233             |
|                       |                              |          BC034733             |
|                       |                              |          BF536299             |
|                       |                              |          BC028911             |
|                       |                              |          AK043259             |
|                       |                              |          BQ287524             |
|                       |                              |          BI111266             |
|                       |                              |          BF178211             |
|                       |                              |          BI685448             |
|                       |                              |          BI106133             |
|                       |                              |          BX525075             |
|                       |                              |          BG078369             |
|                       |                              |          BF722310             |
|                       |                              |          BB856087             |
|                       |                              |          BG270405             |
|                       |                              |          BI697722             |
|                       |                              |          BG974274             |
|                       |                              |          BG174758             |
|                       |                              |          BB004329             |
|                       |                              |          AV349008             |
|                       |                              |          BF320444             |
|                       |                              |          CF729151             |
|                       |                              |          BI650994             |
|                       |                              |          BM502589             |
|                       |                              |          BM502281             |
|                       | *Seq. mapped in strand Minus*| *Seq. mapped in strand Minus* |
|                       |           AK125250           |          BE947100             |
+-----------------------+------------------------------+-------------------------------+
|      #   18           | *Seq. mapped in strand Plus* | *Seq. mapped in strand Plus*  |
|                       |           CD356894           |          BC053970             |
|                       | *Seq. mapped in strand Minus*| *Seq. mapped in strand Minus* |
|                       |           BG705944           |          AK008788             |
|                       |           AA872218           |          AK011302             |
|                       |           AI161342           |          AK018688             |
|                       |           AI248971           |          AK003696             |
|                       |           BE270684           |          AK089103             |
|                       |           BE304374           |          AK018717             |
|                       |           BU595679           |          AK010307             |
|                       |           BG714831           |          AK009698             |
|                       |           AI128630           |          BC060951             |
|                       |           AV722168           |                               |
|                       |           BE561658           |                               |
|                       |           CA775776           |                               |
|                       |           BE304511           |                               |
|                       |           BE561676           |                               |
|                       |           BU600198           |                               |
|                       |           BE906832           |                               |
|                       |           BQ064876           |                               |
|                       |           AA508601           |                               |
|                       |           AI609646           |                               |
|                       |             N94436           |                               |
|                       |           AW073092           |                               |
|                       |           BM264428           |                               |
|                       |           BM312183           |                               |
|                       |           BQ288022           |                               |
|                       |           BQ778118           |                               |
|                       |           AA875886           |                               |
|                       |           AA883939           |                               |
|                       |           AI301635           |                               |
|                       |           AA993399           |                               |
|                       |           AI095590           |                               |
|                       |           BM310574           |                               |
|                       |           BE266480           |                               |
|                       |           BG821214           |                               |
|                       |           BE393447           |                               |
|                       |           CD174810           |                               |
|                       |           CN288640           |                               |
|                       |           BQ948791           |                               |
|                       |           BU189692           |                               |
|                       |           BI193542           |                               |
|                       |           AV686951           |                               |
|                       |           BI599776           |                               |
|                       |           CB991285           |                               |
|                       |           BI597009           |                               |
|                       |           CB989612           |                               |
|                       |           CB990156           |                               |
|                       |           BI561285           |                               |
|                       |           CB991145           |                               |
|                       |           CB995012           |                               |
|                       |           BI598131           |                               |
|                       |           BI551076           |                               |
|                       |           BI551165           |                               |
|                       |           BI552606           |                               |
|                       |           BI599401           |                               |
|                       |           BU565790           |                               |
|                       |           AA917976           |                               |
|                       |           BE261820           |                               |
|                       |           BF307873           |                               |
|                       |           BM999465           |                               |
|                       |           BQ932819           |                               |
|                       |           BM911499           |                               |
|                       |           BM916488           |                               |
|                       |           BM553256           |                               |
|                       |           BM562280           |                               |
|                       |           BG708377           |                               |
|                       |           BI547163           |                               |
|                       |           BI603530           |                               |
|                       |           BI667784           |                               |
|                       |           BG702388           |                               |
|                       |           BI603093           |                               |
|                       |           BF511690           |                               |
|                       |           CB119128           |                               |
|                       |           AA152103           |                               |
|                       |           BI254578           |                               |
|                       |             T78330           |                               |
|                       |           AA729741           |                               |
|                       |           AA729752           |                               |
|                       |           AA886275           |                               |
|                       |           BU949062           |                               |
|                       |           CB069612           |                               |
|                       |           AA689457           |                               |
|                       |           AI520938           |                               |
|                       |           AA833995           |                               |
|                       |           AA961682           |                               |
|                       |           BF057580           |                               |
|                       |           BF221629           |                               |
|                       |           BU790266           |                               |
|                       |           BE795372           |                               |
|                       |           BE732058           |                               |
|                       |           BG478753           |                               |
|                       |           BG340056           |                               |
|                       |           BE279199           |                               |
|                       |           BE791262           |                               |
|                       |           BX375955           |                               |
|                       |           CR609167           |                               |
|                       |           BE263826           |                               |
|                       |           BE792792           |                               |
|                       |           BE790676           |                               |
|                       |           BG481051           |                               |
|                       |           BE792195           |                               |
|                       |           BF972014           |                               |
|                       |           AF087660           |                               |
|                       |           BI560471           |                               |
|                       |           BU596003           |                               |
|                       |           AA508567           |                               |
|                       |           AA508645           |                               |
|                       |           AW518319           |                               |
|                       |             F18960           |                               |
|                       |           AI525049           |                               |
|                       |           AW592782           |                               |
|                       |           AA909264           |                               |
|                       |           AW519022           |                               |
|                       |           AA829435           |                               |
|                       |           AA868027           |                               |
|                       |           AI160021           |                               |
|                       |           AI017929           |                               |
|                       |           AW269526           |                               |
|                       |           BG755829           |                               |
|                       |           BE791526           |                               |
|                       |           BI562610           |                               |
|                       |           BG716086           |                               |
|                       |           AA321232           |                               |
|                       |           AI417261           |                               |
|                       |           AA046681           |                               |
|                       |           AW204886           |                               |
|                       |           AI686214           |                               |
|                       |           BE547177           |                               |
|                       |           AI352654           |                               |
|                       |           AA152181           |                               |
|                       |           AI281705           |                               |
|                       |           BE302312           |                               |
|                       |           AJ711867           |                               |
|                       |           AI863340           |                               |
|                       |           AW512267           |                               |
|                       |             N26481           |                               |
|                       |           BG207567           |                               |
|                       |           AA507444           |                               |
|                       |           AA909159           |                               |
|                       |           AA936142           |                               |
|                       |           AI016702           |                               |
|                       |           AI302612           |                               |
|                       |           AI873496           |                               |
|                       |           BM672864           |                               |
|                       |           AI220450           |                               |
|                       |           BQ029072           |                               |
|                       |           AI285263           |                               |
|                       |           AI720708           |                               |
|                       |           AW166336           |                               |
|                       |           AI083718           |                               |
|                       |           BG209181           |                               |
|                       |           BG195184           |                               |
|                       |           BG212816           |                               |
|                       |             N33459           |                               |
|                       |           AI598144           |                               |
|                       |           AI889052           |                               |
|                       |           AW026111           |                               |
|                       |           AA868719           |                               |
|                       |           AA873566           |                               |
|                       |           AW007505           |                               |
|                       |           AW665947           |                               |
|                       |           AW573186           |                               |
|                       |           CK824799           |                               |
|                       |           BE791443           |                               |
|                       |           CN288641           |                               |
|                       |           BG033006           |                               |
|                       |           AV702446           |                               |
|                       |           AV722035           |                               |
|                       |           BU601678           |                               |
|                       |           BM451307           |                               |
|                       |           BU597704           |                               |
|                       |           CD390120           |                               |
|                       |           BU602137           |                               |
|                       |           AA618021           |                               |
|                       |           BM351848           |                               |
|                       |           BE382669           |                               |
|                       |           BG337923           |                               |
|                       |           BE396990           |                               |
|                       |           BG260206           |                               |
|                       |           BE311985           |                               |
|                       |           BG108020           |                               |
|                       |           CB990872           |                               |
|                       |           AA587029           |                               |
|                       |           BE899438           |                               |
|                       |           BE615486           |                               |
|                       |           BE889745           |                               |
|                       |           BE259522           |                               |
|                       |           BE742782           |                               |
|                       |           BE909526           |                               |
|                       |           BE790873           |                               |
|                       |           CN288645           |                               |
|                       |           BI670449           |                               |
|                       |           CK005145           |                               |
|                       |           BU790232           |                               |
|                       |           CB961911           |                               |
|                       |           AA152029           |                               |
|                       |           BU595378           |                               |
|                       |           BE740528           |                               |
|                       |           AI708646           |                               |
|                       |           AI581953           |                               |
|                       |           AV682466           |                               |
|                       |           AW139387           |                               |
|                       |           BM929497           |                               |
|                       |           AI469742           |                               |
|                       |           BU742817           |                               |
|                       |             N35132           |                               |
|                       |           AA831482           |                               |
|                       |           AI032546           |                               |
|                       |           AI469794           |                               |
|                       |           AI581853           |                               |
|                       |           CA944787           |                               |
|                       |           AW328519           |                               |
|                       |           AW044220           |                               |
|                       |           AA935632           |                               |
|                       |           AW328518           |                               |
|                       |           BG231958           |                               |
|                       |           BQ185999           |                               |
|                       |           BQ184287           |                               |
|                       |           AA777631           |                               |
|                       |           BU627900           |                               |
|                       |           BQ184504           |                               |
|                       |           BU730586           |                               |
|                       |           BE394269           |                               |
|                       |           BG035243           |                               |
|                       |           CD249406           |                               |
|                       |           BM555997           |                               |
|                       |           BM913417           |                               |
|                       |           BU538794           |                               |
|                       |           BF981326           |                               |
|                       |           CR741598           |                               |
|                       |           BU501860           |                               |
|                       |           CD175863           |                               |
|                       |           BC058920           |                               |
|                       |           BG341997           |                               |
|                       |           BQ057195           |                               |
|                       |           BQ069118           |                               |
|                       |           BU538215           |                               |
|                       |           BF311473           |                               |
|                       |           BQ881670           |                               |
|                       |           BI869595           |                               |
|                       |           BQ071232           |                               |
|                       |           BQ935335           |                               |
|                       |           BQ435266           |                               |
|                       |           BQ961893           |                               |
|                       |           CK003370           |                               |
|                       |           BM467205           |                               |
|                       |           BQ212949           |                               |
|                       |           BM553127           |                               |
|                       |           BG829036           |                               |
|                       |           BF312803           |                               |
|                       |           BF317161           |                               |
|                       |           BG340594           |                               |
|                       |           BI116521           |                               |
|                       |           BG820150           |                               |
|                       |           BI160081           |                               |
|                       |           BG705698           |                               |
|                       |           CN288648           |                               |
|                       |           AA868795           |                               |
|                       |           BG682853           |                               |
|                       |           BG032425           |                               |
|                       |           BI598920           |                               |
|                       |           BQ925184           |                               |
|                       |           BE392645           |                               |
|                       |             R05838           |                               |
|                       |           BX375954           |                               |
|                       |           AA600754           |                               |
|                       |           BE261415           |                               |
|                       |           BG684630           |                               |
|                       |           CK824798           |                               |
|                       |           BM010444           |                               |
|                       |           BG468675           |                               |
|                       |           BM930541           |                               |
|                       |           BG762387           |                               |
|                       |           BG339817           |                               |
|                       |           BU790565           |                               |
|                       |           BU949300           |                               |
|                       |           BE264210           |                               |
|                       |           BM310873           |                               |
|                       |           BU596404           |                               |
|                       |           BG470231           |                               |
|                       |           CB995391           |                               |
|                       |           BU790529           |                               |
|                       |           BU073775           |                               |
|                       |           BF132234           |                               |
|                       |           BG341720           |                               |
|                       |           AW818985           |                               |
|                       |           BE937566           |                               |
|                       |           BQ776938           |                               |
|                       |           BM772385           |                               |
|                       |           AW248392           |                               |
|                       |           CB111825           |                               |
|                       |           BM706782           |                               |
|                       |           CN288643           |                               |
|                       |           AA310175           |                               |
|                       |           BP322189           |                               |
|                       |             F24032           |                               |
|                       |           BP370797           |                               |
|                       |           BQ270156           |                               |
|                       |           BI604006           |                               |
|                       |           BE018746           |                               |
|                       |           CB991185           |                               |
|                       |           BG683569           |                               |
|                       |           BU860156           |                               |
|                       |           BP317600           |                               |
|                       |           BP318135           |                               |
|                       |           BP319936           |                               |
|                       |           BP347242           |                               |
|                       |           BP318236           |                               |
|                       |           BP318001           |                               |
|                       |           BP317963           |                               |
|                       |           BP319234           |                               |
|                       |           BP276789           |                               |
|                       |           BP317487           |                               |
|                       |           BP341967           |                               |
|                       |           BP328940           |                               |
|                       |             F25329           |                               |
|                       |             F36770           |                               |
|                       |           BP334179           |                               |
|                       |           BU599968           |                               |
|                       |           BM312596           |                               |
|                       |           BP193552           |                               |
|                       |           BP244656           |                               |
|                       |           BP246123           |                               |
|                       |           BP280877           |                               |
|                       |           BP280918           |                               |
|                       |           BP202458           |                               |
|                       |           BP244285           |                               |
|                       |           BP244374           |                               |
|                       |           BP244558           |                               |
|                       |           BP246507           |                               |
|                       |           BP246914           |                               |
|                       |           BP247357           |                               |
|                       |           BP249247           |                               |
|                       |           BP279804           |                               |
|                       |           BP244902           |                               |
|                       |           BP245157           |                               |
|                       |           BP246050           |                               |
|                       |           BP246350           |                               |
|                       |           BP247312           |                               |
|                       |           BP247408           |                               |
|                       |           BP248977           |                               |
|                       |           BP249562           |                               |
|                       |           BP357632           |                               |
|                       |           BG756879           |                               |
|                       |           BP199898           |                               |
|                       |           BM272330           |                               |
|                       |           BP278422           |                               |
|                       |           CA948791           |                               |
|                       |           CB070097           |                               |
|                       |           BM352157           |                               |
|                       |           BM312265           |                               |
|                       |           BP370106           |                               |
|                       |           CB992157           |                               |
|                       |           BU949499           |                               |
|                       |           AJ709982           |                               |
|                       |           BM705809           |                               |
|                       |           AV753475           |                               |
|                       |           BP272255           |                               |
|                       |           BI159817           |                               |
|                       |           BP376881           |                               |
|                       |           BM271786           |                               |
|                       |           BE708835           |                               |
|                       |           BP203705           |                               |
|                       |           BE563972           |                               |
|                       |           AJ708582           |                               |
|                       |           BP201849           |                               |
|                       |           BP351614           |                               |
|                       |           BM841796           |                               |
|                       |           AA417135           |                               |
|                       |           BI666735           |                               |
|                       |           BX338399           |                               |
|                       |           BI908600           |                               |
|                       |           AW602860           |                               |
|                       |           AA313368           |                               |
|                       |             N42432           |                               |
|                       |           BQ302445           |                               |
|                       |           AI815674           |                               |
|                       |           BI094312           |                               |
|                       |             N35190           |                               |
|                       |             N35532           |                               |
|                       |           AA476531           |                               |
|                       |           CB992158           |                               |
|                       |           CB152870           |                               |
|                       |           CB994516           |                               |
|                       |             N86284           |                               |
|                       |           BQ323735           |                               |
|                       |           BU945530           |                               |
|                       |           BE048186           |                               |
|                       |           AA642285           |                               |
|                       |           CA777710           |                               |
|                       |           AA448553           |                               |
|                       |           AA459170           |                               |
|                       |           AA689510           |                               |
|                       |           AA906828           |                               |
|                       |           AA827419           |                               |
|                       |           AA416980           |                               |
|                       |           AA632370           |                               |
|                       |           AA639717           |                               |
|                       |           AA936986           |                               |
|                       |           AA885866           |                               |
|                       |           AA878640           |                               |
|                       |           CN288646           |                               |
|                       |           BM764353           |                               |
|                       |           AA150058           |                               |
|                       |           BM822084           |                               |
|                       |           BQ187601           |                               |
|                       |           CB108117           |                               |
|                       |           AV724353           |                               |
|                       |           BM792944           |                               |
|                       |           CN288644           |                               |
|                       |           AV724358           |                               |
|                       |           CN288647           |                               |
|                       |           AW732820           |                               |
|                       |           BP353529           |                               |
|                       |           BP353619           |                               |
|                       |           BP353805           |                               |
|                       |             D54530           |                               |
|                       |           AA994079           |                               |
|                       |           AA136946           |                               |
|                       |           AL710732           |                               |
|                       |           BP201130           |                               |
|                       |           CN288649           |                               |
|                       |           BM830267           |                               |
|                       |           BM705451           |                               |
|                       |           BP870577           |                               |
|                       |           CN288642           |                               |
|                       |           CB107386           |                               |
|                       |           CB139382           |                               |
|                       |             D80791           |                               |
|                       |           AU099784           |                               |
|                       |           AW379804           |                               |
|                       |           CB137961           |                               |
|                       |           BG654991           |                               |
|                       |           BE163158           |                               |
|                       |           AA602743           |                               |
+-----------------------+------------------------------+-------------------------------+
|      #   19           | *Seq. mapped in strand Plus* | *Seq. mapped in strand Plus*  |
|                       |           AL137716           |          AI666521             |
|                       |           BC065275           |                               |
|                       |           BU194843           |                               |
|                       |             U48408           |                               |
|                       | *Seq. mapped in strand Minus*| *Seq. mapped in strand Minus* |
|                       |           AA112383           |          BU755994             |
+-----------------------+------------------------------+-------------------------------+
|      #   20           | *Seq. mapped in strand Plus* | *Seq. mapped in strand Plus*  |
|                       |           BC016047           |          AK044335             |
|                       |             Z36835           |                               |
|                       |           BX100741           |                               |
|                       |           BF224066           |                               |
|                       |           AA769672           |                               |
|                       |           BF508555           |                               |
|                       | *Seq. mapped in strand Minus*| *Seq. mapped in strand Minus* |
|                       |           CA427190           |          AK037108             |
+-----------------------+------------------------------+-------------------------------+
|      #   21           | *Seq. mapped in strand Plus* | *Seq. mapped in strand Plus*  |
|                       |           AA233888           |          CA320380             |
|                       | *Seq. mapped in strand Minus*| *Seq. mapped in strand Minus* |
|                       |           CR611122           |          AI845946             |
|                       |           AI590420           |          AW049097             |
|                       |           BX099634           |          AW061135             |
|                       |           AI357434           |          BI219906             |
|                       |           AA731716           |          BF465436             |
|                       |           AA810686           |          CD805130             |
|                       |           AU133342           |          BU755614             |
|                       |           AA780104           |          AA986561             |
|                       |           AA582157           |          BF460994             |
|                       |           BQ029658           |          BE946438             |
|                       |           CB107902           |          BE992818             |
|                       |           AI250881           |          BE995420             |
|                       |           AA485151           |          BX520619             |
|                       |           BM837131           |          BY651629             |
|                       |           AI369932           |            D67016             |
|                       |           AA761750           |          BC018378             |
|                       |           AI123280           |            L40406             |
|                       |           AW015796           |            D67017             |
|                       |           AI753470           |          BE983750             |
|                       |           AA543054           |          BE984029             |
|                       |           BU680410           |          AA268648             |
|                       |           BM971989           |          AK075731             |
|                       |           BU677892           |          AI790491             |
|                       |           BU632747           |          BU514378             |
|                       |           CA421904           |          CA322299             |
|                       |           BM973263           |          CD298433             |
|                       |           BU630846           |          CA322836             |
|                       |           CA313924           |          CA323459             |
|                       |           BQ026450           |          BQ571249             |
|                       |           CB854035           |          CB208925             |
|                       |           BM983480           |          CF952118             |
|                       |           BE672159           |          BG921069             |
|                       |           BM971288           |          BY386136             |
|                       |           AI337175           |          AA572403             |
|                       |           AF039695           |          CB570861             |
|                       |           AB003333           |          BE136212             |
|                       |           BC037553           |          BY387104             |
|                       |           AB003334           |          BB756222             |
|                       |             D86956           |          BI499717             |
|                       |           BX648125           |          CF578827             |
|                       |           BM312391           |          BQ571063             |
|                       |           AW163535           |          BE135426             |
|                       |           AW117974           |          CB570807             |
|                       |           AI742981           |          AA624532             |
|                       |           BQ006975           |          BU963084             |
|                       |           AI312753           |          BG077915             |
|                       |           AI831339           |          CN454663             |
|                       |           CR748291           |          CD770036             |
|                       |           AL520323           |          AA571359             |
|                       |           BE739629           |          AI838486             |
|                       |           BX401265           |          BG066973             |
|                       |           AA417317           |            C81194             |
|                       |           BG482892           |          BU708228             |
|                       |           BG532824           |          CA324272             |
|                       |           BG680243           |          CB235456             |
|                       |           CB999661           |          BE533791             |
|                       |           BG165839           |          BI657075             |
|                       |           CB992238           |          BU512674             |
|                       |           BM783955           |          CD355782             |
|                       |           BC018124           |          BY058603             |
|                       |           AW771160           |          CN716791             |
|                       |           CA433222           |          CN539624             |
|                       |           BG166036           |          BY057495             |
|                       |           BX648623           |          CK031526             |
|                       |           BE739168           |          BI694510             |
|                       |           BG431448           |          BF117634             |
|                       |           BU192443           |          CF748717             |
|                       |           BG681493           |          CA563605             |
|                       |           CB991879           |          BG862916             |
|                       |           BE891235           |          BY266544             |
|                       |           CD640453           |          BY290031             |
|                       |           BG284876           |          BY286022             |
|                       |           AV744652           |          BI730490             |
|                       |           BG191701           |          BM949897             |
|                       |           AI658961           |          BF159120             |
|                       |           AA232636           |          BI697440             |
|                       |           AV722836           |          BU614230             |
|                       |           BM979486           |          BF232864             |
|                       |           AW470142           |          CD560230             |
|                       |           BM979082           |          BI100851             |
|                       |           BU632385           |          BU148541             |
|                       |           BM969894           |          CA551640             |
|                       |           AW571648           |          AA105012             |
|                       |           AW474070           |          BE283999             |
|                       |           CB243822           |          CF725704             |
|                       |           CN478671           |          CF156316             |
|                       |           AW572452           |          CB234215             |
|                       |           BU689167           |          CA539755             |
|                       |           AW385582           |          CN678458             |
|                       |           AW994721           |          AA867489             |
|                       |           AW608075           |          CF891752             |
|                       |           BE080587           |          BI816938             |
|                       |           AW970458           |          CN528949             |
|                       |           AU126851           |          BE192679             |
|                       |           AA166806           |          AW210124             |
|                       |           BM680423           |          CN532388             |
|                       |           BM979574           |          CF891742             |
|                       |           BQ722673           |          CF895566             |
|                       |           CN406516           |          CA540336             |
|                       |           AI751852           |          BU504190             |
|                       |           AA394027           |          CN678043             |
|                       |           AW628153           |          BU920230             |
|                       |           AA485036           |          CN529011             |
|                       |           AW604836           |          CN528943             |
|                       |           BX350546           |          CA894339             |
|                       |           AW390368           |          BU512995             |
|                       |           AW820299           |          BE652033             |
|                       |           AA953645           |          CD565259             |
|                       |           BP289686           |          CA569909             |
|                       |           BE934755           |          CA320677             |
|                       |           AW609781           |          BU610586             |
|                       |           BM800762           |          CN527794             |
|                       |           BF800806           |          CN714265             |
|                       |           BF185078           |          CA881351             |
|                       |             T63090           |          BU613890             |
|                       |           AV734168           |          CA319235             |
|                       |           BM667585           |          CD544640             |
|                       |             F07487           |          CA531928             |
|                       |             F08794           |          CB234168             |
|                       |           AW859943           |          CO046351             |
|                       |           AW859988           |          BI319882             |
|                       |           AW820234           |          CF739071             |
|                       |           BF895058           |          CD549030             |
|                       |           BF791809           |          CF950167             |
|                       |           BF334498           |          BQ571914             |
|                       |           BQ316999           |          CA876976             |
|                       |           BU561614           |          CD772435             |
|                       |           BM835045           |          CF750450             |
|                       |           AW820232           |          CN536802             |
|                       |           AW389335           |          CN724543             |
|                       |           CK001049           |          CN715918             |
|                       |           BF771389           |          BQ745061             |
|                       |           AA777031           |          CN715386             |
|                       |           BU167787           |          CA317835             |
|                       |           BQ717978           |          CA319091             |
|                       |           BG027770           |          CF739044             |
|                       |           BF772447           |          CF748195             |
|                       |           BQ720903           |          CD550123             |
|                       |           CO248707           |          CN528839             |
|                       |           BQ364023           |          CN528910             |
|                       |           BF772390           |          CN528759             |
|                       |           AL520324           |          CF738006             |
|                       |           BG008553           |          CF168743             |
|                       |           CD671804           |          BF785648             |
|                       |           BG008534           |          CD563838             |
|                       |           BG010046           |          AU066691             |
|                       |           BF910102           |          BE954141             |
|                       |           BE867822           |          CF736070             |
|                       |           AW362751           |          CA317635             |
|                       |           BP291873           |          BI257039             |
|                       |           BG025113           |          CD548653             |
|                       |           BF432835           |          BY726376             |
|                       |           AI702970           |          CN665191             |
|                       |           BU594779           |          BF582178             |
|                       |           AW206874           |          BF384283             |
|                       |           AI094015           |          BI732063             |
|                       |           AI758907           |          BQ180083             |
|                       |           BE673025           |          BI147222             |
|                       |           AA885873           |          CB238036             |
|                       |           BE550676           |          BF233180             |
|                       |             H63551           |          BF135576             |
|                       |             H64019           |          BY713766             |
|                       |           AA777564           |          CA569900             |
|                       |           AA580595           |          BM565242             |
|                       |             H91160           |          CN527465             |
|                       |             H54657           |          BI737673             |
|                       |           BM826367           |          BQ876119             |
|                       |           BG403660           |          BY739454             |
|                       |           CD107169           |          CN528112             |
|                       |           BE934756           |          BB650665             |
|                       |           AW391572           |          BF237075             |
|                       |           AW820231           |          AI787816             |
|                       |           AW362766           |          CD561602             |
|                       |           BG008532           |          AI746652             |
|                       |           BG010045           |          CK391627             |
|                       |           AA749004           |          CN532363             |
|                       |             W45471           |          BI144378             |
|                       |           AA811573           |          CN684534             |
|                       |           AI800379           |          AW105834             |
|                       |           BE693976           |          CN679032             |
|                       |           BM856776           |          BY713462             |
|                       |           BF093382           |          BY713873             |
|                       |           BG179356           |          BF786948             |
|                       |           AI290252           |          CN677870             |
|                       |           AW631423           |          CF164837             |
|                       |           AA805016           |          CD538864             |
|                       |           BQ300629           |          CA879220             |
|                       |           BP257273           |          CF166016             |
|                       |           CN406505           |          CA545564             |
|                       |             H63595           |          CF163396             |
|                       |             N84914           |          CF165036             |
|                       |           BE614160           |          CF164826             |
|                       |           BF681122           |          AW209689             |
|                       |             H54656           |          CF164049             |
|                       |           AW630933           |          CN680862             |
|                       |           AV755234           |          BY008127             |
|                       |           AA094644           |          CA881460             |
|                       |           AI002886           |          CN694999             |
|                       |           AU124176           |          CB247031             |
|                       |           AW577563           |          BY032514             |
|                       |             H91211           |          AI931227             |
|                       |           AA334479           |          BE227017             |
|                       |           AU127828           |          CN671598             |
|                       |           AU127938           |          BY006188             |
|                       |           BU177152           |          AW227137             |
|                       |           BU160342           |          BY324599             |
|                       |           BU431576           |          BY328663             |
|                       |           AL042714           |          BY067161             |
|                       |           BE613904           |          BY086597             |
|                       |           AA580712           |          BY134247             |
|                       |             H64073           |          BY323648             |
|                       |           CN406501           |          BY132232             |
|                       |           BI522723           |          BY022634             |
|                       |           BQ318430           |          BY047729             |
|                       |             W86141           |          BY769883             |
|                       |           CA397839           |          BY309357             |
|                       |           BX390811           |          BY020987             |
|                       |           AA714219           |          BY074927             |
|                       |           CN406512           |          BY231013             |
|                       |           BG949700           |          CN683077             |
|                       |           AA580845           |          BY123065             |
|                       |           BM456425           |          BY127523             |
|                       |           AI656127           |                               |
|                       |           AI203278           |                               |
|                       |           AI633338           |                               |
|                       |           AI267631           |                               |
|                       |           AA311379           |                               |
|                       |           BM803911           |                               |
|                       |           BP221745           |                               |
|                       |             Z21219           |                               |
|                       |             X85639           |                               |
|                       |           BP382925           |                               |
|                       |           CN406518           |                               |
|                       |           BQ337860           |                               |
|                       |           BQ338434           |                               |
|                       |           BQ338435           |                               |
|                       |           BQ338489           |                               |
|                       |           AW901837           |                               |
|                       |           BQ337861           |                               |
|                       |           BQ310861           |                               |
|                       |           BU162116           |                               |
|                       |           AA361653           |                               |
|                       |           AU127296           |                               |
|                       |           CN406507           |                               |
|                       |           BP355372           |                               |
|                       |           BM710774           |                               |
|                       |           BG622371           |                               |
|                       |           AU128016           |                               |
|                       |           CN406509           |                               |
|                       |           AW607389           |                               |
|                       |           BU156820           |                               |
|                       |           BP260451           |                               |
|                       |           BE867716           |                               |
|                       |           BQ230835           |                               |
|                       |           CB270546           |                               |
|                       |           AW818298           |                               |
|                       |           BX502933           |                               |
|                       |           BE898035           |                               |
|                       |           BM474963           |                               |
|                       |           CN406500           |                               |
|                       |           BP233297           |                               |
|                       |           AV718076           |                               |
|                       |             W40235           |                               |
|                       |           BG434699           |                               |
|                       |           AV756186           |                               |
|                       |           BQ233418           |                               |
|                       |           AL045611           |                               |
|                       |           BX505608           |                               |
|                       |           BF793111           |                               |
|                       |           BX342231           |                               |
|                       |           CA390632           |                               |
|                       |           BQ324448           |                               |
|                       |           BQ231774           |                               |
|                       |           BQ213990           |                               |
|                       |           CV030479           |                               |
|                       |           BG718319           |                               |
|                       |           BX482426           |                               |
|                       |           BQ324450           |                               |
|                       |           BG925262           |                               |
|                       |           BQ323965           |                               |
|                       |           BM450227           |                               |
|                       |           BU165744           |                               |
|                       |           AU131352           |                               |
|                       |           AV752985           |                               |
|                       |           BM469046           |                               |
|                       |           AA360776           |                               |
|                       |           CN406504           |                               |
|                       |           AA081692           |                               |
|                       |           BF810278           |                               |
|                       |           CN406508           |                               |
|                       |           CN406503           |                               |
|                       |           CN406511           |                               |
|                       |           AL705133           |                               |
|                       |           CN406502           |                               |
|                       |           BI551800           |                               |
|                       |           CN406515           |                               |
|                       |           AL043449           |                               |
|                       |           AL708258           |                               |
|                       |           BF308504           |                               |
|                       |           CD358375           |                               |
|                       |           BE887616           |                               |
|                       |           BP250530           |                               |
|                       |           BU170903           |                               |
|                       |           BG479813           |                               |
|                       |           BP218859           |                               |
|                       |           BP278015           |                               |
|                       |           BP219007           |                               |
|                       |           AV749381           |                               |
|                       |           BF305854           |                               |
|                       |           BG106283           |                               |
|                       |           CN406499           |                               |
|                       |           AL705523           |                               |
|                       |           BP251626           |                               |
|                       |           CD251908           |                               |
|                       |           AL044212           |                               |
|                       |           BQ231224           |                               |
|                       |           CN406506           |                               |
|                       |           CN406510           |                               |
|                       |           BP365269           |                               |
|                       |           BP343162           |                               |
|                       |           CN406517           |                               |
|                       |           BP270639           |                               |
|                       |           BP363752           |                               |
|                       |           CN406513           |                               |
|                       |           BP363011           |                               |
|                       |           BP217515           |                               |
|                       |           BP224648           |                               |
|                       |           BP279593           |                               |
|                       |           BP278528           |                               |
|                       |           BP364013           |                               |
|                       |           BP202334           |                               |
|                       |           BP224597           |                               |
|                       |           BP236448           |                               |
|                       |           BP222221           |                               |
|                       |           BP250488           |                               |
|                       |           BP250865           |                               |
|                       |           BP320767           |                               |
|                       |           BP353168           |                               |
|                       |           BP360893           |                               |
|                       |           BP363255           |                               |
|                       |           BP202389           |                               |
|                       |           BP278260           |                               |
|                       |           BP222316           |                               |
|                       |           BP279811           |                               |
|                       |           BP311997           |                               |
|                       |           BP202109           |                               |
|                       |           AU280629           |                               |
|                       |           AL701376           |                               |
|                       |           BP274745           |                               |
|                       |           BP200744           |                               |
|                       |           BP284614           |                               |
|                       |           BP320390           |                               |
|                       |           AW137489           |                               |
+-----------------------+------------------------------+-------------------------------+
|      #   22           | *Seq. mapped in strand Plus* | *Seq. mapped in strand Plus*  |
|                       |           BP381619           |          BC027165             |
|                       |           AK074196           |                               |
|                       |           AA256750           |                               |
|                       |           AJ242782           |                               |
|                       |           AJ238994           |                               |
|                       |           BP382204           |                               |
|                       |           AK074204           |                               |
|                       |           AJ272195           |                               |
|                       |           BP338580           |                               |
|                       |           BI909388           |                               |
|                       |           BC033758           |                               |
|                       |           BM561847           |                               |
|                       |           BM922463           |                               |
|                       |           BX338207           |                               |
|                       |           BP304969           |                               |
|                       |           BP382668           |                               |
|                       |           BP295990           |                               |
|                       |           BM906228           |                               |
|                       |           CV030702           |                               |
|                       |           BP340263           |                               |
|                       |           BI040577           |                               |
|                       |           CB133965           |                               |
|                       |           AA625433           |                               |
|                       |           BX390430           |                               |
|                       |           CB148001           |                               |
|                       |           BX327865           |                               |
|                       |           BI545941           |                               |
|                       |           BX102751           |                               |
|                       |           BI756215           |                               |
|                       |             R67241           |                               |
|                       |           CA307252           |                               |
|                       |           AI761520           |                               |
|                       |           AI824043           |                               |
|                       |           AA262556           |                               |
|                       |           AI355999           |                               |
|                       |           AI051397           |                               |
|                       |           AW452188           |                               |
|                       | *Seq. mapped in strand Minus*| *Seq. mapped in strand Minus* |
|                       |           BX493510           |          AK053136             |
+-----------------------+------------------------------+-------------------------------+
|      #   23           | *Seq. mapped in strand Plus* | *Seq. mapped in strand Plus*  |
|                       |           AK056324           |          BU698613             |
|                       |           BI161009           |          CF169410             |
|                       |           BI457147           |          BG246967             |
|                       |           BX357502           |          BY108628             |
|                       |           BG118168           |          AA451318             |
|                       |           BG770255           |          BB856896             |
|                       |           AF479813           |          BI685190             |
|                       |           BX398157           |          AA035870             |
|                       |           BM563711           |          BB664706             |
|                       |           BX385943           |          BU522515             |
|                       |           BX335807           |          AY241870             |
|                       |           CR597108           |          BY083306             |
|                       |           BC047701           |          BY150990             |
|                       |           BI835432           |          BB658106             |
|                       |           AL514858           |          AK079165             |
|                       |           CB959820           |          BY084432             |
|                       |           BX365163           |          BY097109             |
|                       |           AL550478           |          BY096263             |
|                       |           CR611794           |          BY094321             |
|                       |           BI827612           |          BY735192             |
|                       |           BC036042           |          BB627459             |
|                       |           CN403992           |          BY772170             |
|                       |           BC040227           |          BB843290             |
|                       |           CF593713           |          BB861010             |
|                       |           BX353643           |          BY074796             |
|                       |           BM560395           |          BY791323             |
|                       |           BM926441           |          BY106285             |
|                       |           BI826251           |          BY785021             |
|                       |           AL526558           |          AA051408             |
|                       |           CR621105           |          BY013422             |
|                       |           BX365301           |          BY014119             |
|                       |           CR625182           |          BY134711             |
|                       |           BM453093           |          BC003230             |
|                       |           BI562351           |            W91292             |
|                       |           CB996581           |          AA222056             |
|                       |           BX324981           |          BF144696             |
|                       |           CR598427           |          BI456214             |
|                       |           BX376202           |          BY215248             |
|                       |           AY166714           |          BI081778             |
|                       |           CR615620           |          CB181490             |
|                       |           BM545926           |          BE310917             |
|                       |           BG421676           |          AA518317             |
|                       |           BG761479           |          BY236792             |
|                       |           BG766427           |          BB563730             |
|                       |           BI759674           |          BY206587             |
|                       |           BU542240           |          BY079195             |
|                       |           BQ919974           |          BY092769             |
|                       |           AL544210           |          AA027700             |
|                       |           BX364819           |          BU149068             |
|                       |           CR619122           |          CN664584             |
|                       |           BQ956538           |          BG245830             |
|                       |           BU543936           |          BG245740             |
|                       |           BX424909           |          CK624083             |
|                       |           BX383396           |          BQ961236             |
|                       |           CR618108           |          BY704139             |
|                       |           BX387716           |          AK003882             |
|                       |           BM906504           |          BG321883             |
|                       |           BX394208           |          AA668001             |
|                       |           BX380438           |          BY012535             |
|                       |           CR616563           |          BU699288             |
|                       |           CR613081           |          CK129161             |
|                       |           CR606566           |          BY176403             |
|                       |           CR594798           |          CB182089             |
|                       |           CR608574           |          BY185333             |
|                       |           BE903609           |          BY118996             |
|                       |           AL551269           |          BY117659             |
|                       |           CR621723           |          BG062355             |
|                       |           BX375105           |          BE370943             |
|                       |           BX356518           |          BG806642             |
|                       |           AL542357           |            W82426             |
|                       |           CR599238           |            W91293             |
|                       |           CR596167           |          CB057574             |
|                       |           BI822650           |          CN456296             |
|                       |           BI759175           |          CN455592             |
|                       |           CB994359           |          BI852841             |
|                       |           BC023509           |          BG916614             |
|                       |           BM920223           |          CB321028             |
|                       |           BG748053           |          BU562330             |
|                       |           BX280358           |          CF581136             |
|                       |           BQ068255           |          BI792707             |
|                       |           CN403993           |          BE370777             |
|                       |           BC023591           |          AW647586             |
|                       |           BG828846           |          BQ548045             |
|                       |           CN403991           |          BQ746150             |
|                       |           BI518832           |          CF734924             |
|                       |           BX394842           |                               |
|                       |           CR593528           |                               |
|                       |           BE747130           |                               |
|                       |           AL527977           |                               |
|                       |           CR606357           |                               |
|                       |           BQ924873           |                               |
|                       |           BI087043           |                               |
|                       |           BM563036           |                               |
|                       |           BU150300           |                               |
|                       |           BX354638           |                               |
|                       |           CR589961           |                               |
|                       |           BU150171           |                               |
|                       |           CD514412           |                               |
|                       |           BU621695           |                               |
|                       |           BX952540           |                               |
|                       |           AW327517           |                               |
|                       |           BM906164           |                               |
|                       |           BP291141           |                               |
|                       |           BG251098           |                               |
|                       |           AK075075           |                               |
|                       |           AL042115           |                               |
|                       |           BU855029           |                               |
|                       |           BP337309           |                               |
|                       |           BP367481           |                               |
|                       |           BF813945           |                               |
|                       |           BU599202           |                               |
|                       |             R14227           |                               |
|                       |             H15332           |                               |
|                       |           BX340224           |                               |
|                       |             Z46193           |                               |
|                       |           BF813939           |                               |
|                       |           CD559142           |                               |
|                       |           CD518077           |                               |
|                       |           CN403998           |                               |
|                       |           BG323505           |                               |
|                       |           BG697860           |                               |
|                       |           BE907226           |                               |
|                       |           BX335806           |                               |
|                       |           BF981832           |                               |
|                       |           BI199176           |                               |
|                       |           BG744169           |                               |
|                       |           AW957722           |                               |
|                       |           AA351880           |                               |
|                       |           BM918233           |                               |
|                       |           BQ054182           |                               |
|                       |           CA309177           |                               |
|                       |           CA308811           |                               |
|                       |           BQ014772           |                               |
|                       |           BU622946           |                               |
|                       |           BQ008437           |                               |
|                       |           BU682824           |                               |
|                       |           BQ004224           |                               |
|                       |           BM476701           |                               |
|                       |           BU682418           |                               |
|                       |           CA421948           |                               |
|                       |           BU686202           |                               |
|                       |           BQ446404           |                               |
|                       |           BM971810           |                               |
|                       |           BM145699           |                               |
|                       |           CB118643           |                               |
|                       |           AV735997           |                               |
|                       |           AI554669           |                               |
|                       |           BF035607           |                               |
|                       |           AI769369           |                               |
|                       |           CB123487           |                               |
|                       |           AI199561           |                               |
|                       |           CB119462           |                               |
|                       |           BU074011           |                               |
|                       |           AW006534           |                               |
|                       |           AI990389           |                               |
|                       |           AI631814           |                               |
|                       |           AW016914           |                               |
|                       |           BU742788           |                               |
|                       |           BM970780           |                               |
|                       |           AL047918           |                               |
|                       |           CA436568           |                               |
|                       |           BM144908           |                               |
|                       |           AW327573           |                               |
|                       |           AI685615           |                               |
|                       |           AI492163           |                               |
|                       |           BQ021434           |                               |
|                       |           BU618779           |                               |
|                       |           CA429909           |                               |
|                       |           BQ018899           |                               |
|                       |           AI127476           |                               |
|                       |             R40646           |                               |
|                       |           BF446192           |                               |
|                       |           AA609306           |                               |
|                       |           AI203197           |                               |
|                       |           AW235727           |                               |
|                       | *Seq. mapped in strand Minus*| *Seq. mapped in strand Minus* |
|                       |           BU737297           |          CF356959             |
+-----------------------+------------------------------+-------------------------------+
|      #   24           | *Seq. mapped in strand Plus* | *Seq. mapped in strand Plus*  |
|                       |           AA469991           |          AK017335             |
|                       | *Seq. mapped in strand Minus*| *Seq. mapped in strand Minus* |
|                       |           AU280165           |          BC065068             |
|                       |           CD614369           |            U09383             |
|                       |           BP315199           |          AF465244             |
|                       |           BM128676           |            L16912             |
|                       |           AA512886           |          AF156674             |
|                       |           BM688852           |                               |
|                       |           CR739691           |                               |
|                       |           CR627384           |                               |
|                       |           AI367843           |                               |
|                       |           BF001467           |                               |
|                       |           AI263560           |                               |
|                       |           AA044244           |                               |
|                       |           BM893581           |                               |
|                       |           CK724888           |                               |
|                       |           BC009695           |                               |
|                       |           BC062659           |                               |
|                       |           BC024965           |                               |
|                       |           AI953949           |                               |
|                       |           BE856326           |                               |
|                       |           AW003118           |                               |
|                       |           BG108373           |                               |
|                       |             F36294           |                               |
+-----------------------+------------------------------+-------------------------------+
|      #   25           | *Seq. mapped in strand Plus* | *Seq. mapped in strand Plus*  |
|                       |           BI463943           |          BU701135             |
|                       |           BG771786           |          BF462941             |
|                       |           BP271324           |                               |
|                       |           BM834151           |                               |
|                       |           BG717410           |                               |
|                       |           BI560558           |                               |
|                       |           CN265648           |                               |
|                       |           BG699281           |                               |
|                       |             U70322           |                               |
|                       |           AU099077           |                               |
|                       |           CN265635           |                               |
|                       |           BX404124           |                               |
|                       |           CN265638           |                               |
|                       |           CN265636           |                               |
|                       |           CN265637           |                               |
|                       |           CN265640           |                               |
|                       |           BC040340           |                               |
|                       |           BF528821           |                               |
|                       |           AW161157           |                               |
|                       |           CN265641           |                               |
|                       |           CN265639           |                               |
|                       |           AU143202           |                               |
|                       |           AL704445           |                               |
|                       |           BX956256           |                               |
|                       |           AL704446           |                               |
|                       |           AW628560           |                               |
|                       |           CB990224           |                               |
|                       |           BP871829           |                               |
|                       |           BP871895           |                               |
|                       |           BP872494           |                               |
|                       |           CN265633           |                               |
|                       |           BX343058           |                               |
|                       |           CR592982           |                               |
|                       |           BM807028           |                               |
|                       |             U72395           |                               |
|                       |             U72069           |                               |
|                       |           CN265649           |                               |
|                       |           CN265650           |                               |
|                       |           BM471774           |                               |
|                       |           BG251704           |                               |
|                       |           BG392802           |                               |
|                       |           CN265642           |                               |
|                       |           AU132209           |                               |
|                       |           CN265647           |                               |
|                       |           BE895811           |                               |
|                       |           BE085860           |                               |
|                       |           BM845934           |                               |
|                       |           BX349267           |                               |
|                       |           BG620138           |                               |
|                       |           CN265644           |                               |
|                       |           BI088515           |                               |
|                       |           BE545370           |                               |
|                       |           CB114599           |                               |
|                       |           BE537988           |                               |
|                       |           AV709949           |                               |
|                       |           BE208428           |                               |
|                       |           BF752530           |                               |
|                       |           BM830536           |                               |
|                       |           BX328054           |                               |
|                       |           BU595986           |                               |
|                       |           AA853480           |                               |
|                       |           CA942252           |                               |
|                       |           CA866434           |                               |
|                       |           BM786410           |                               |
|                       |           CB144049           |                               |
|                       |           CN265634           |                               |
|                       |           BE547904           |                               |
|                       |           AU142986           |                               |
|                       |           AU119254           |                               |
|                       |           BM754091           |                               |
|                       |             R54232           |                               |
|                       |           BX372346           |                               |
|                       |           CB151412           |                               |
|                       |           AU119232           |                               |
|                       |           BP872225           |                               |
|                       |           CN265646           |                               |
|                       |           BM698680           |                               |
|                       |           AA100490           |                               |
|                       |           BF792344           |                               |
|                       |           BE560127           |                               |
|                       |           AA135102           |                               |
|                       |           BX280267           |                               |
|                       |           BX384276           |                               |
|                       |           AA249362           |                               |
|                       |           BG391069           |                               |
|                       |           CD673794           |                               |
|                       |           CN265645           |                               |
|                       |           BP871312           |                               |
|                       |           AA132155           |                               |
|                       |           AU123781           |                               |
|                       |           BG708207           |                               |
|                       |           AA195882           |                               |
|                       |           AL043497           |                               |
|                       |           BP873541           |                               |
|                       |           CB216416           |                               |
|                       |           BG576731           |                               |
|                       |           BQ636722           |                               |
|                       |           BG110575           |                               |
|                       |           BG109768           |                               |
|                       |           BX343057           |                               |
|                       |             F05852           |                               |
|                       |             Z42585           |                               |
|                       |           AW968706           |                               |
|                       |           AA481124           |                               |
|                       |           BE270233           |                               |
|                       |           BF205170           |                               |
|                       |           BF304462           |                               |
|                       |           BM824625           |                               |
|                       |           BQ670015           |                               |
|                       |           BI160706           |                               |
|                       |           AW968532           |                               |
|                       |           AA452943           |                               |
|                       |           BX390684           |                               |
|                       |           BE295290           |                               |
|                       |           AI307759           |                               |
|                       |           BX383436           |                               |
|                       |           AI962791           |                               |
|                       |           CN265651           |                               |
|                       |           BU634326           |                               |
|                       |           AV717799           |                               |
|                       |           BG674629           |                               |
|                       |           AA092435           |                               |
|                       |           BQ711380           |                               |
|                       |           CN265643           |                               |
|                       |           CA773754           |                               |
|                       |           AW976100           |                               |
|                       |           AA349077           |                               |
|                       |           AW949968           |                               |
|                       |           BM824473           |                               |
|                       |           BM977482           |                               |
|                       |           AV651208           |                               |
|                       |           BM453871           |                               |
|                       |           BG987016           |                               |
|                       |           BM796985           |                               |
|                       |           BG654352           |                               |
|                       |           BI005098           |                               |
|                       |           AW383482           |                               |
|                       |           CD238450           |                               |
|                       |           CA942863           |                               |
|                       |           BE350834           |                               |
|                       |           AI814278           |                               |
|                       |           AA604370           |                               |
|                       |           BX330884           |                               |
|                       |           BU072674           |                               |
|                       |           BG435121           |                               |
|                       |           AA700237           |                               |
|                       |           BF770445           |                               |
|                       |           CB136905           |                               |
|                       |           BM670770           |                               |
|                       |           BF001833           |                               |
|                       |           AW072792           |                               |
|                       |           AW572753           |                               |
|                       |           AI401138           |                               |
|                       |           AI701539           |                               |
|                       |           AI248990           |                               |
|                       |           AI968204           |                               |
|                       |           CA942620           |                               |
|                       |           AA454037           |                               |
|                       |           AA442609           |                               |
|                       |           AA481067           |                               |
|                       |           CA844041           |                               |
|                       |           AI631170           |                               |
|                       |           AI634428           |                               |
|                       |           AI651782           |                               |
|                       |           AW270739           |                               |
|                       |           BF063204           |                               |
|                       |           BG987011           |                               |
|                       |           AA055096           |                               |
|                       |           AW383650           |                               |
|                       |           AU160335           |                               |
|                       |           AU160160           |                               |
|                       |           AI693343           |                               |
|                       |           BF057045           |                               |
|                       |           AA946681           |                               |
|                       |           BE550299           |                               |
|                       |           AU153787           |                               |
|                       |           CB108114           |                               |
|                       |           BU753427           |                               |
|                       |           AI631357           |                               |
|                       |           AA789103           |                               |
|                       |           AI766014           |                               |
|                       |           AA834076           |                               |
|                       |           AW469946           |                               |
|                       |           CF143196           |                               |
|                       |           AA806246           |                               |
|                       |           AI147158           |                               |
|                       |           AI654889           |                               |
|                       |           AI630405           |                               |
|                       |           BF149183           |                               |
|                       |           AA595598           |                               |
|                       |           BU940765           |                               |
|                       |           AI475617           |                               |
|                       |           AI983108           |                               |
|                       |           AI699519           |                               |
|                       |           AW418623           |                               |
|                       |           BQ010967           |                               |
|                       |           BF592834           |                               |
|                       |           BE219048           |                               |
|                       |           AI632658           |                               |
|                       |           AI394292           |                               |
|                       |           AI352525           |                               |
|                       |           AA654813           |                               |
|                       |           BF002709           |                               |
|                       |           AA988740           |                               |
|                       |           AL043498           |                               |
|                       |           BU072390           |                               |
|                       |           AI867694           |                               |
|                       |           AU155898           |                               |
|                       |           AI867579           |                               |
|                       |           AA954040           |                               |
|                       |             N42555           |                               |
|                       |           CA844214           |                               |
|                       |           BF431437           |                               |
|                       |           AI653355           |                               |
|                       |           AA534902           |                               |
|                       |           AI148522           |                               |
|                       |           AW016208           |                               |
|                       |           AA853481           |                               |
|                       |           AW183387           |                               |
|                       |           BX484977           |                               |
|                       |           CB105182           |                               |
|                       |           AI765943           |                               |
|                       |           AI433435           |                               |
|                       |           AI569834           |                               |
|                       |           BM506742           |                               |
|                       |           CF529133           |                               |
|                       |           CR742927           |                               |
|                       |           BM750140           |                               |
|                       | *Seq. mapped in strand Minus*| *Seq. mapped in strand Minus* |
|                       |           AI524543           |          BF148677             |
|                       |                              |          AI506799             |
+-----------------------+------------------------------+-------------------------------+
|      #   26           | *Seq. mapped in strand Plus* | *Seq. mapped in strand Plus*  |
|                       |           CD705322           |          AK008840             |
|                       |           BG749354           |          AW822108             |
|                       |           CD691874           |          AW489390             |
|                       |           BI666950           |          BG142689             |
|                       |           BI668768           |          AI195773             |
|                       |           CD690289           |          BX517909             |
|                       |           AL703545           |          BG797561             |
|                       |           BX482659           |                               |
|                       |           CD683922           |                               |
|                       |           BG699229           |                               |
|                       |           BF797383           |                               |
|                       |           AK172845           |                               |
|                       |           BC013173           |                               |
|                       |           CR747381           |                               |
|                       |           CA392843           |                               |
|                       |           AK027549           |                               |
|                       |           AB075852           |                               |
|                       |           BQ439291           |                               |
|                       |           BU500574           |                               |
|                       |           CA392052           |                               |
|                       |           CA392054           |                               |
|                       |           BE882864           |                               |
|                       |           BI768690           |                               |
|                       |           BU072529           |                               |
|                       |           AU253442           |                               |
|                       |           BM010902           |                               |
|                       |           CK902426           |                               |
|                       |           BU161287           |                               |
|                       |           CD109369           |                               |
|                       |           CN263211           |                               |
|                       |           BG165215           |                               |
|                       |           BG772050           |                               |
|                       |           BG744401           |                               |
|                       |           BG620045           |                               |
|                       |           BF332134           |                               |
|                       |           CN263205           |                               |
|                       |           BU196560           |                               |
|                       |           BQ680025           |                               |
|                       |           BQ677455           |                               |
|                       |           AA352611           |                               |
|                       |           AA088309           |                               |
|                       |             Z43391           |                               |
|                       |           CN263208           |                               |
|                       |             T30428           |                               |
|                       |           AW958044           |                               |
|                       |           CB140943           |                               |
|                       |           CN263209           |                               |
|                       |           CN263212           |                               |
|                       |           AW276830           |                               |
|                       |           BG745046           |                               |
|                       |           BG315180           |                               |
|                       |           BI018452           |                               |
|                       |           AU253443           |                               |
|                       |           CA430171           |                               |
|                       |           BG983496           |                               |
|                       |           BU072257           |                               |
|                       |           BM972597           |                               |
|                       |           BG181454           |                               |
|                       |           AI206557           |                               |
|                       |           BM682578           |                               |
|                       |           BM726151           |                               |
|                       |           CK902427           |                               |
|                       |           AI750662           |                               |
|                       |           AI459716           |                               |
|                       |           AI128839           |                               |
|                       |           AA165677           |                               |
|                       |           AA159690           |                               |
|                       |           BG028856           |                               |
|                       |           AI471732           |                               |
|                       |           BU177039           |                               |
|                       |           AI246400           |                               |
|                       |             Z39464           |                               |
|                       |           AI758780           |                               |
|                       |           BG122939           |                               |
|                       |           AI417879           |                               |
|                       |           CF121349           |                               |
|                       |           AL834402           |                               |
|                       |           BM477487           |                               |
|                       |           BU675829           |                               |
|                       |           CD580076           |                               |
|                       |             H47748           |                               |
|                       |             H47749           |                               |
|                       |           BX094585           |                               |
|                       |           BF515098           |                               |
|                       |           AI911927           |                               |
|                       | *Seq. mapped in strand Minus*| *Seq. mapped in strand Minus* |
|                       |           BU686051           |          BE987060             |
+-----------------------+------------------------------+-------------------------------+
|      #   27           | *Seq. mapped in strand Plus* | *Seq. mapped in strand Plus*  |
|                       |           BM987661           |          BY732993             |
|                       | *Seq. mapped in strand Minus*| *Seq. mapped in strand Minus* |
|                       |           BI491091           |          AI467200             |
+-----------------------+------------------------------+-------------------------------+
|      #   28           | *Seq. mapped in strand Plus* | *Seq. mapped in strand Plus*  |
|                       |           AK131522           |          BM952069             |
|                       |           AJ236915           |          BY294597             |
|                       |           AF276893           |          BY061177             |
|                       |           BC035596           |          BY059666             |
|                       |                              |          BY291903             |
|                       |                              |          BY268930             |
|                       |                              |          BF151873             |
|                       |                              |          CA327774             |
|                       |                              |          BC059912             |
|                       |                              |          BY089711             |
|                       |                              |          BY126867             |
|                       |                              |          BY256971             |
|                       |                              |          BY035804             |
|                       |                              |          AK028788             |
|                       |                              |          BB613105             |
|                       |                              |          BY736148             |
|                       |                              |          BY150138             |
|                       |                              |          BY140856             |
|                       |                              |          BY255467             |
|                       |                              |          BM943281             |
|                       |                              |          BM950601             |
|                       |                              |          BI660662             |
|                       |                              |          BY590683             |
|                       |                              |          CD350296             |
|                       | *Seq. mapped in strand Minus*| *Seq. mapped in strand Minus* |
|                       |           BC061900           |          AI850458             |
|                       |           AK095915           |                               |
+-----------------------+------------------------------+-------------------------------+
|      #   29           | *Seq. mapped in strand Plus* | *Seq. mapped in strand Plus*  |
|                       |           BM987609           |          AK043153             |
|                       | *Seq. mapped in strand Minus*| *Seq. mapped in strand Minus* |
|                       |           BM663981           |          AK045362             |
|                       |           BF516270           |          AF077739             |
|                       |           CR456414           |                               |
|                       |           BC069612           |                               |
|                       |           AF096322           |                               |
|                       |           AI912567           |                               |
|                       |           AI680925           |                               |
|                       |           BE670632           |                               |
|                       |           BE222518           |                               |
+-----------------------+------------------------------+-------------------------------+
|      #   30           | *Seq. mapped in strand Plus* | *Seq. mapped in strand Plus*  |
|                       |           CA439891           |          AK046653             |
|                       | *Seq. mapped in strand Minus*| *Seq. mapped in strand Minus* |
|                       |           CA446358           |          BC006732             |
|                       |           AI004389           |          AK028780             |
|                       |           AA888338           |          AK014395             |
|                       |           AW006833           |            U16297             |
|                       |           AA759250           |                               |
|                       |           AA932839           |                               |
|                       |           AI056920           |                               |
|                       |           BF941976           |                               |
|                       |           AI168133           |                               |
|                       |           AI567682           |                               |
|                       |           AI769279           |                               |
|                       |           BM681527           |                               |
|                       |           AW275966           |                               |
|                       |           BM680884           |                               |
|                       |           AI201079           |                               |
|                       |           BM676051           |                               |
|                       |           BQ028822           |                               |
|                       |           BU622753           |                               |
|                       |           BU688673           |                               |
|                       |           BM983742           |                               |
|                       |           CA425580           |                               |
|                       |           CA425519           |                               |
|                       |           BU678740           |                               |
|                       |           BM979330           |                               |
|                       |           BQ773579           |                               |
|                       |           BM983027           |                               |
|                       |           BM981580           |                               |
|                       |           AK026598           |                               |
|                       |           AK095244           |                               |
|                       |           AI053716           |                               |
|                       |           BX648452           |                               |
|                       |           BE041774           |                               |
|                       |           AI349660           |                               |
|                       |           BF509538           |                               |
|                       |           CA941229           |                               |
|                       |           CR624200           |                               |
|                       |           BU579851           |                               |
|                       |           BU857600           |                               |
|                       |           CN390797           |                               |
|                       |           BC002976           |                               |
|                       |           BQ876342           |                               |
|                       |           BM672320           |                               |
|                       |           BE872420           |                               |
|                       |           BI088336           |                               |
|                       |           BU543466           |                               |
|                       |           BI199721           |                               |
|                       |           BM048167           |                               |
|                       |           BI861399           |                               |
|                       |           BU542033           |                               |
|                       |           BU527604           |                               |
|                       |           BI195108           |                               |
|                       |           BU150225           |                               |
|                       |           BQ669680           |                               |
|                       |           BU543377           |                               |
|                       |           BP269089           |                               |
|                       |           BI118036           |                               |
|                       |           BP269090           |                               |
|                       |           BP268993           |                               |
|                       |           BP268482           |                               |
|                       |           BF314171           |                               |
|                       |           CA454774           |                               |
|                       |           BC000021           |                               |
|                       |           BF314431           |                               |
|                       |           CN390798           |                               |
|                       |           BQ878575           |                               |
|                       |           AL515058           |                               |
|                       |           BX397678           |                               |
|                       |           BX457902           |                               |
|                       |           AL514258           |                               |
|                       |           BU543664           |                               |
|                       |           BQ919882           |                               |
|                       |           BP313044           |                               |
|                       |           AL547257           |                               |
|                       |           AL535358           |                               |
|                       |           AL514272           |                               |
|                       |           BG775569           |                               |
|                       |           BG774917           |                               |
|                       |           BI198818           |                               |
|                       |           BQ721584           |                               |
|                       |           BM547137           |                               |
|                       |           AL544241           |                               |
|                       |           BG775170           |                               |
|                       |           BT007096           |                               |
|                       |           BF311327           |                               |
|                       |           BI196509           |                               |
|                       |           BE382537           |                               |
|                       |           CB996702           |                               |
|                       |           BI334117           |                               |
|                       |           BF316933           |                               |
|                       |           BQ721220           |                               |
|                       |           BE312746           |                               |
|                       |           BF206346           |                               |
|                       |           BG519796           |                               |
|                       |           BM019222           |                               |
|                       |           BE383407           |                               |
|                       |           BI196214           |                               |
|                       |           BM049215           |                               |
|                       |           BF316348           |                               |
|                       |           BI195560           |                               |
|                       |           AL602419           |                               |
|                       |           AU124594           |                               |
|                       |           AU121901           |                               |
|                       |             T39581           |                               |
|                       |           BI200045           |                               |
|                       |           BQ716282           |                               |
|                       |           BF315914           |                               |
|                       |           BI597912           |                               |
|                       |           BF311586           |                               |
|                       |           BM049427           |                               |
|                       |           BF310593           |                               |
|                       |           CV025823           |                               |
|                       |           BF312266           |                               |
|                       |           BG325448           |                               |
|                       |           BF317118           |                               |
|                       |           BE262852           |                               |
|                       |           BE262637           |                               |
|                       |           AU121476           |                               |
|                       |           BE260621           |                               |
|                       |           BF313839           |                               |
|                       |           BP325899           |                               |
|                       |           BF314437           |                               |
|                       |           BF314069           |                               |
|                       |           BF304246           |                               |
|                       |           BI561168           |                               |
|                       |           BF311157           |                               |
|                       |           BG423901           |                               |
|                       |           BF316965           |                               |
|                       |           BE312329           |                               |
|                       |           BF311574           |                               |
|                       |           BE262892           |                               |
|                       |           BF206313           |                               |
|                       |           BF315205           |                               |
|                       |           BP352559           |                               |
|                       |           BE382638           |                               |
|                       |           BE314789           |                               |
|                       |           BE312214           |                               |
|                       |           BP277667           |                               |
|                       |           BP197894           |                               |
|                       |           BM827442           |                               |
|                       |           BP304175           |                               |
|                       |           BE314475           |                               |
|                       |           BP252165           |                               |
|                       |           BP314758           |                               |
|                       |           BP288383           |                               |
|                       |           BF313819           |                               |
|                       |           BP212487           |                               |
|                       |           BP382591           |                               |
|                       |           BE260749           |                               |
|                       |           CV025867           |                               |
|                       |           BE260101           |                               |
|                       |           BE260280           |                               |
|                       |           BE313554           |                               |
|                       |           BI195343           |                               |
|                       |           BM044466           |                               |
|                       |           BE260743           |                               |
|                       |           BF332630           |                               |
|                       |             W24970           |                               |
|                       |           AA428222           |                               |
|                       |           BE383278           |                               |
|                       |           BF316251           |                               |
|                       |           BE266345           |                               |
|                       |           BP374495           |                               |
|                       |           BP275398           |                               |
|                       |           AA464144           |                               |
+-----------------------+------------------------------+-------------------------------+
|      #   31           | *Seq. mapped in strand Plus* | *Seq. mapped in strand Plus*  |
|                       |           BQ478082           |          BU702109             |
|                       |           BU077637           |                               |
|                       |           BU947489           |                               |
|                       | *Seq. mapped in strand Minus*| *Seq. mapped in strand Minus* |
|                       |           BX281020           |          AI849273             |
+-----------------------+------------------------------+-------------------------------+
|      #   32           | *Seq. mapped in strand Plus* | *Seq. mapped in strand Plus*  |
|                       |             D84145           |          AA990052             |
|                       |           BI562535           |                               |
|                       |           BF967062           |                               |
|                       |           BI667174           |                               |
|                       |           AA345687           |                               |
|                       |           BG546108           |                               |
|                       |           AW953013           |                               |
|                       |           BF964605           |                               |
|                       |           BF681245           |                               |
|                       |           BF215970           |                               |
|                       |           BC013175           |                               |
|                       |           BU665528           |                               |
|                       |           BE739290           |                               |
|                       |           BF819200           |                               |
|                       |             W39113           |                               |
|                       |           BG426528           |                               |
|                       |           BI545604           |                               |
|                       |           CB987228           |                               |
|                       |           BU656447           |                               |
|                       |           BF207900           |                               |
|                       |           BP328874           |                               |
|                       |           BG530946           |                               |
|                       |           BM790714           |                               |
|                       |           AU136071           |                               |
|                       |           CB109198           |                               |
|                       |           BQ268264           |                               |
|                       |           BE898020           |                               |
|                       |           CN399494           |                               |
|                       |           CN399493           |                               |
|                       |           BM551461           |                               |
|                       |             H78562           |                               |
|                       |           BE885117           |                               |
|                       |           BU180994           |                               |
|                       |           BE866002           |                               |
|                       |           BE564235           |                               |
|                       |           BF107508           |                               |
|                       |           CB116785           |                               |
|                       |           CR456851           |                               |
|                       |           CV023829           |                               |
|                       |           CN399491           |                               |
|                       |           CB164258           |                               |
|                       |           CD674276           |                               |
|                       |           CN399495           |                               |
|                       |           BX116498           |                               |
|                       |           BI666998           |                               |
|                       |           AL545403           |                               |
|                       |           CA389529           |                               |
|                       |           AL537749           |                               |
|                       |           BI831080           |                               |
|                       |           BI769548           |                               |
|                       |           CR624991           |                               |
|                       |           AL537750           |                               |
|                       |           CR590871           |                               |
|                       |           AL559282           |                               |
|                       |           AL580927           |                               |
|                       |           CR593835           |                               |
|                       |           BQ130862           |                               |
|                       |           CK905887           |                               |
|                       |           BE856828           |                               |
|                       |           CN399496           |                               |
|                       |           BF032846           |                               |
|                       |           CA942967           |                               |
|                       |           BM990018           |                               |
|                       |           AA361577           |                               |
|                       |           AA451781           |                               |
|                       |           BE832821           |                               |
|                       |           BG425946           |                               |
|                       |           AL571139           |                               |
|                       |           CB126211           |                               |
|                       |           BQ083742           |                               |
|                       |           AA044431           |                               |
|                       |             N77038           |                               |
|                       |           CR741503           |                               |
|                       |           BM955959           |                               |
|                       |           BF699905           |                               |
|                       |           BM711013           |                               |
|                       |             W05852           |                               |
|                       |           BQ018786           |                               |
|                       |           AA424247           |                               |
|                       |           AW016838           |                               |
|                       |           AV748971           |                               |
|                       |           CA314695           |                               |
|                       |           AA665445           |                               |
|                       |           AA682486           |                               |
|                       |           AA249827           |                               |
|                       |           AI084013           |                               |
|                       | *Seq. mapped in strand Minus*| *Seq. mapped in strand Minus* |
|                       |           BF434546           |          BY703880             |
+-----------------------+------------------------------+-------------------------------+
|      #   33           | *Seq. mapped in strand Plus* | *Seq. mapped in strand Plus*  |
|                       |           AW297341           |          AK047540             |
|                       | *Seq. mapped in strand Minus*| *Seq. mapped in strand Minus* |
|                       |           AK023605           |          BC058103             |
|                       |           BE327866           |                               |
|                       |           BM984405           |                               |
|                       |           BQ000357           |                               |
|                       |           CF890766           |                               |
|                       |           AL137348           |                               |
|                       |           AB036063           |                               |
|                       |           AA495950           |                               |
|                       |           AI669595           |                               |
|                       |           AI378311           |                               |
|                       |           BU737569           |                               |
|                       |           BQ574024           |                               |
|                       |           BC042468           |                               |
|                       |           AI651905           |                               |
|                       |           AW874225           |                               |
|                       |           AI004057           |                               |
|                       |           AI478132           |                               |
|                       |           AI766981           |                               |
|                       |           AI022125           |                               |
|                       |           AW044466           |                               |
|                       |           CA413622           |                               |
|                       |           CA418636           |                               |
|                       |           AA766003           |                               |
|                       |           AA768847           |                               |
|                       |           BC042948           |                               |
|                       |           AA812196           |                               |
|                       |           BQ023243           |                               |
|                       |           CR627376           |                               |
|                       |           CR617553           |                               |
|                       |           AA765000           |                               |
|                       |           BX348782           |                               |
|                       |           AK001965           |                               |
|                       |           AA528169           |                               |
|                       |           BX509409           |                               |
|                       |           BG286192           |                               |
|                       |           BU590184           |                               |
|                       |           BQ011366           |                               |
|                       |           AB166669           |                               |
|                       |           AB166670           |                               |
|                       |           AB163437           |                               |
|                       |           AB163438           |                               |
|                       |           AB166671           |                               |
|                       |           AA730240           |                               |
|                       |           AL547501           |                               |
|                       |           CD656661           |                               |
|                       |           BQ694828           |                               |
|                       |           BE169367           |                               |
|                       |           BQ441857           |                               |
|                       |           BF673318           |                               |
|                       |           CF995079           |                               |
|                       |           BG995472           |                               |
|                       |           AL705595           |                               |
|                       |           BU170979           |                               |
|                       |           BM801298           |                               |
|                       |           BX951374           |                               |
|                       |           AU137050           |                               |
|                       |           CN358025           |                               |
|                       |           CN358022           |                               |
|                       |           BU429508           |                               |
|                       |           BX955728           |                               |
|                       |           BU429507           |                               |
|                       |           AL712577           |                               |
+-----------------------+------------------------------+-------------------------------+
|      #   34           | *Seq. mapped in strand Plus* | *Seq. mapped in strand Plus*  |
|                       |           AF086198           |          AK084864             |
|                       | *Seq. mapped in strand Minus*| *Seq. mapped in strand Minus* |
|                       |           AI381718           |          AK036845             |
+-----------------------+------------------------------+-------------------------------+
|      #   35           | *Seq. mapped in strand Plus* | *Seq. mapped in strand Plus*  |
|                       |           AK096314           |          BC051579             |
|                       | *Seq. mapped in strand Minus*| *Seq. mapped in strand Minus* |
|                       |           BC039836           |          BC057147             |
|                       |             X54870           |          AF054819             |
|                       |           CR607215           |          AF030313             |
|                       |           CR597595           |          AF039026             |
|                       |           AF461811           |                               |
|                       |           AF260135           |                               |
|                       |           AF260136           |                               |
|                       |           AF439512           |                               |
|                       |             U96845           |                               |
|                       |           AJ001683           |                               |
|                       |           BC017784           |                               |
|                       |           AF350018           |                               |
|                       |           AF350019           |                               |
+-----------------------+------------------------------+-------------------------------+
|      #   36           | *Seq. mapped in strand Plus* | *Seq. mapped in strand Plus*  |
|                       |           BF131942           |          BE334739             |
|                       |           BF575402           |          BE625593             |
|                       |           BG530997           |          BG086044             |
|                       |           BG613868           |          CB273246             |
|                       |           BP202637           |          CN673838             |
|                       |           BI772378           |          AA967795             |
|                       |           BP318154           |          AI155727             |
|                       |             W80644           |          AA562040             |
|                       |           AF212225           |          AA461787             |
|                       |           BG500266           |          AI573539             |
|                       |           CN428689           |          AA981748             |
|                       |           CB852272           |          BI466374             |
|                       |             R84420           |          AW540363             |
|                       |             W24410           |          AW549179             |
|                       |           BF242899           |          BB776938             |
|                       |           CN428686           |          BB781667             |
|                       |           CN428693           |          BY443033             |
|                       |           BG505953           |          BC028774             |
|                       |           CF597343           |          BY367564             |
|                       |           BG531794           |          AA553174             |
|                       |           BG532497           |          BX629911             |
|                       |           BU957957           |          BG073306             |
|                       |           CB963965           |          AI573467             |
|                       |           BF668226           |          AV099253             |
|                       |           BG501735           |          AV204558             |
|                       |           BC014356           |                               |
|                       |           BI850254           |                               |
|                       |           BP209588           |                               |
|                       |           CD250452           |                               |
|                       |           BF240849           |                               |
|                       |           BF695760           |                               |
|                       |           BF698837           |                               |
|                       |           BG613482           |                               |
|                       |           BF215511           |                               |
|                       |           BF700854           |                               |
|                       |           BG563952           |                               |
|                       |           BF217298           |                               |
|                       |           BF691898           |                               |
|                       |           BF207866           |                               |
|                       |           BF242630           |                               |
|                       |           CB999680           |                               |
|                       |           BU783238           |                               |
|                       |           BF243845           |                               |
|                       |           CB984979           |                               |
|                       |           BF576390           |                               |
|                       |           BF692017           |                               |
|                       |           BG532209           |                               |
|                       |           BG612657           |                               |
|                       |           BG566055           |                               |
|                       |           BG505628           |                               |
|                       |           BC017765           |                               |
|                       |           CD173373           |                               |
|                       |           BU958031           |                               |
|                       |           BP258229           |                               |
|                       |           BM832713           |                               |
|                       |           BP369186           |                               |
|                       |           CB144443           |                               |
|                       |           CB995016           |                               |
|                       |           BF244891           |                               |
|                       |           BF699424           |                               |
|                       |           BG568701           |                               |
|                       |           BG402347           |                               |
|                       |           BG430412           |                               |
|                       |           BG532133           |                               |
|                       |           BG615987           |                               |
|                       |           BG532810           |                               |
|                       |           BU928710           |                               |
|                       |           BC015109           |                               |
|                       |           BU928541           |                               |
|                       |           BF575765           |                               |
|                       |           BF695809           |                               |
|                       |           BF574848           |                               |
|                       |           BG502127           |                               |
|                       |           BF243025           |                               |
|                       |           BG615809           |                               |
|                       |           BG612113           |                               |
|                       |           CN428692           |                               |
|                       |           BF241662           |                               |
|                       |           BF242886           |                               |
|                       |           BF664839           |                               |
|                       |           CN428688           |                               |
|                       |           BG030694           |                               |
|                       |           CA454920           |                               |
|                       |             R22151           |                               |
|                       |           AV651968           |                               |
|                       |           BM772176           |                               |
|                       |           BM765570           |                               |
|                       |           BG389411           |                               |
|                       |           BE564907           |                               |
|                       |           BM806549           |                               |
|                       |           AA361418           |                               |
|                       |           BF103603           |                               |
|                       |           BU188603           |                               |
|                       |           BF028134           |                               |
|                       |           BC032595           |                               |
|                       |           AA374190           |                               |
|                       |           BM452903           |                               |
|                       |           AV694464           |                               |
|                       |           CN428684           |                               |
|                       |           BF028090           |                               |
|                       |           AA343749           |                               |
|                       |           BE789220           |                               |
|                       |           AW963049           |                               |
|                       |           BE564021           |                               |
|                       |           BF029342           |                               |
|                       |           BF668846           |                               |
|                       |           BE866240           |                               |
|                       |           CN428687           |                               |
|                       |           BP224684           |                               |
|                       |           CB123749           |                               |
|                       |             W38919           |                               |
|                       |             W56072           |                               |
|                       |           BF219092           |                               |
|                       |             H85258           |                               |
|                       |             D81727           |                               |
|                       |           BU199232           |                               |
|                       |           AB049474           |                               |
|                       |             R21005           |                               |
|                       |             R53138           |                               |
|                       |             T78314           |                               |
|                       |             R36313           |                               |
|                       |           AA001673           |                               |
|                       |           BE566469           |                               |
|                       |           BG178313           |                               |
|                       |           BF211095           |                               |
|                       |           CN428690           |                               |
|                       |           CV028782           |                               |
|                       |           CR457191           |                               |
|                       |           CV028806           |                               |
|                       |           BM699875           |                               |
|                       |           BG527605           |                               |
|                       |           BM765569           |                               |
|                       |           CA949723           |                               |
|                       |           BG182274           |                               |
|                       |           AL549290           |                               |
|                       |           CR606749           |                               |
|                       |           BX279876           |                               |
|                       |           BG107943           |                               |
|                       |           AK024791           |                               |
|                       |           CN428683           |                               |
|                       |             W05768           |                               |
|                       |           AA315327           |                               |
|                       |           AW960358           |                               |
|                       |           CN428691           |                               |
|                       |           AW363183           |                               |
|                       |           AI017758           |                               |
|                       |           AW363168           |                               |
|                       |           BG779759           |                               |
|                       |           AI240792           |                               |
|                       |             W04541           |                               |
|                       |             W32408           |                               |
|                       |             W24001           |                               |
|                       |           CN428685           |                               |
|                       |           BQ015157           |                               |
|                       |           AA001674           |                               |
|                       |           AW195365           |                               |
|                       |           BU679074           |                               |
|                       |           AW243044           |                               |
|                       |           BU675164           |                               |
|                       |           BE542570           |                               |
|                       |           BU737615           |                               |
|                       |             R53052           |                               |
|                       |           BG168335           |                               |
|                       |           BF476196           |                               |
|                       |             W80645           |                               |
|                       |             W32984           |                               |
|                       |           AV756986           |                               |
|                       |           AA676576           |                               |
|                       |           AA524142           |                               |
|                       |           BE220898           |                               |
|                       |           BE463444           |                               |
|                       |           BM970857           |                               |
|                       |           AI032880           |                               |
|                       |             W31210           |                               |
|                       |           AW083286           |                               |
|                       |             W39715           |                               |
|                       |             W56179           |                               |
|                       |           AI242863           |                               |
|                       |           AA424353           |                               |
|                       |           AI823378           |                               |
|                       |           AA424519           |                               |
|                       |           AI055903           |                               |
|                       |           AA988532           |                               |
|                       |           AI307330           |                               |
|                       |           BF589891           |                               |
|                       |           AW135177           |                               |
|                       |           AA811866           |                               |
|                       |             N94748           |                               |
|                       |           AA829513           |                               |
|                       |           AA535048           |                               |
|                       |           BG181369           |                               |
|                       |           BG183412           |                               |
|                       |           BG191781           |                               |
|                       |           BG201011           |                               |
|                       |           BG205671           |                               |
|                       |           BG205672           |                               |
|                       |           BG186041           |                               |
|                       |           BG218442           |                               |
|                       |           BG213022           |                               |
|                       |           BG195849           |                               |
|                       |           BG192835           |                               |
|                       |           BG181367           |                               |
|                       |           BG181370           |                               |
|                       |           BG192836           |                               |
|                       |           BG221291           |                               |
|                       |           BG198402           |                               |
|                       |           BG216791           |                               |
|                       |           BG202500           |                               |
|                       |           BG206236           |                               |
|                       |           BG217287           |                               |
|                       |             F21184           |                               |
|                       |           AV739964           |                               |
|                       |           BG192281           |                               |
|                       |           BG194993           |                               |
|                       |           BG198401           |                               |
|                       |           BG205640           |                               |
|                       |           BG215731           |                               |
|                       |           BG215733           |                               |
|                       |           BG213023           |                               |
|                       |           BG218443           |                               |
|                       |           BG195847           |                               |
|                       |           BG215732           |                               |
|                       |           BG221292           |                               |
|                       |           BG184976           |                               |
|                       |           BG186588           |                               |
|                       |           BG217289           |                               |
|                       |           BG205138           |                               |
|                       |           BG220808           |                               |
|                       |           BG198943           |                               |
|                       |             R84421           |                               |
|                       |           AI077950           |                               |
|                       |           BG198942           |                               |
|                       |             H98149           |                               |
|                       |           AA715844           |                               |
|                       |           BG216790           |                               |
|                       |           BG201374           |                               |
|                       |           BG189180           |                               |
|                       |           BG203546           |                               |
|                       |           BG183411           |                               |
|                       |           AA731109           |                               |
|                       |           BG211421           |                               |
|                       |           BG206237           |                               |
|                       |           BG181863           |                               |
|                       |           BG192282           |                               |
|                       |           AW236066           |                               |
|                       |           AA844292           |                               |
|                       |           AA775614           |                               |
|                       |           AA810648           |                               |
|                       |           AA991201           |                               |
|                       |           BG209870           |                               |
|                       |           BI859092           |                               |
|                       |           BG203064           |                               |
|                       |           BG200499           |                               |
|                       |           BG187080           |                               |
|                       |           BG194992           |                               |
|                       |           BG217288           |                               |
|                       |           BG209871           |                               |
|                       |           BG215734           |                               |
|                       |           BG192837           |                               |
|                       |           BG188671           |                               |
|                       |             R49245           |                               |
|                       |           BG183929           |                               |
|                       |           BG195848           |                               |
|                       |           BG187707           |                               |
|                       |           BG195378           |                               |
|                       |           BG202028           |                               |
|                       |           BG219846           |                               |
|                       |           BG216793           |                               |
|                       |           AA506582           |                               |
|                       |           AA490609           |                               |
|                       | *Seq. mapped in strand Minus*| *Seq. mapped in strand Minus* |
|                       |           BG201334           |          BE947779             |
|                       |                              |          CB057756             |
|                       |                              |                               |
|                       |                              |                               |
|                       |                              |                               |
|                       |                              |                               |
|                       |                              |                               |
|                       |                              |                               |
|                       |                              |                               |
|                       |                              |                               |
|                       |                              |                               |
|                       |                              |                               |
|                       |                              |                               |
|                       |                              |                               |
|                       |                              |                               |
|                       |                              |                               |
|                       |                              |                               |
|                       |                              |                               |
|                       |                              |                               |
|                       |                              |                               |
|                       |                              |                               |
|                       |                              |                               |
|                       |                              |                               |
|                       |                              |                               |
|                       |                              |                               |
+-----------------------+------------------------------+-------------------------------+
|      #   37           | *Seq. mapped in strand Plus* | *Seq. mapped in strand Plus*  |
|                       |           AI377414           |          AK003738             |
|                       |           AI078092           |                               |
|                       | *Seq. mapped in strand Minus*| *Seq. mapped in strand Minus* |
|                       |           AA564051           |          BE983098             |
|                       |           CA310236           |          BE946660             |
|                       |           BM668484           |          BE956303             |
|                       |           BQ446075           |          AI849101             |
|                       |           CD370084           |          BE985767             |
|                       |           CD743189           |          BE945165             |
|                       |           CD366695           |          BE985829             |
|                       |           BQ013461           |          BC005772             |
|                       |           BQ012983           |          AV253995             |
|                       |           AA993413           |          AK032051             |
|                       |           AA973512           |          AF332053             |
|                       |           AW134534           |          AF332054             |
|                       |           BE220139           |          BQ714342             |
|                       |           AI342924           |          BG261486             |
|                       |           AI095136           |          BI732092             |
|                       |           AW182910           |          BU707896             |
|                       |           AI421163           |          BM950965             |
|                       |           BE501149           |          BQ891850             |
|                       |           AA449437           |          BQ939982             |
|                       |           AW137924           |          BQ891828             |
|                       |           AI301806           |          BQ930590             |
|                       |           BE170837           |          CA324270             |
|                       |           AI424102           |          BI734172             |
|                       |           BF915090           |          BQ571580             |
|                       |           BF805279           |          BU504416             |
|                       |           CN340517           |          BE569361             |
|                       |           BU683858           |          BQ443445             |
|                       |           AL732114           |          BY724360             |
|                       |           AW614875           |          CD353959             |
|                       |           BF115740           |          CK620000             |
|                       |           AI472158           |          CK620617             |
|                       |           BE327400           |          BB632418             |
|                       |             H41723           |          BY718682             |
|                       |           BQ631713           |          BB614505             |
|                       |           BU072608           |          BY242934             |
|                       |           BM931535           |          BY254493             |
|                       |           CN344475           |          BY240037             |
|                       |           BC040030           |          BY256184             |
|                       |           BC033745           |          BY251317             |
|                       |           AI475595           |          BY253783             |
|                       |           AI808946           |          BY285974             |
|                       |           AI498140           |          BY246501             |
|                       |           AI423493           |          BY295715             |
|                       |             N89927           |          BY295499             |
|                       |           AI383147           |          BY270529             |
|                       |           AI215983           |          BY276064             |
|                       |           AI333990           |          BY287852             |
|                       |           AI818817           |          BY296301             |
|                       |           AI292097           |          BY003475             |
|                       |           AI138463           |          BY295043             |
|                       |           AI017876           |          BY263878             |
|                       |           AI870954           |          BY297573             |
|                       |           BU072328           |          BY286073             |
|                       |           CA841905           |          BY257920             |
|                       |           BQ787964           |          BB864791             |
|                       |           BQ632013           |          BY283690             |
|                       |           AL449860           |          BY294277             |
|                       |           AL732115           |          BY292969             |
|                       |           BF929945           |          BY340610             |
|                       |           CN340513           |          BY341398             |
|                       |           AW578042           |          BY342934             |
|                       |           AI204975           |          BY342808             |
|                       |           BF837337           |          BY127251             |
|                       |           AJ712016           |          BY126403             |
|                       |           AL449603           |                               |
|                       |           AL449604           |                               |
|                       |           AL449861           |                               |
|                       |           BF885472           |                               |
|                       |           CA843541           |                               |
|                       |           CN344479           |                               |
|                       |           BI823586           |                               |
|                       |           BM928134           |                               |
|                       |           CV325276           |                               |
|                       |           AK124788           |                               |
|                       |           BI753392           |                               |
|                       |           CN340516           |                               |
|                       |           AL732117           |                               |
|                       |           BX374819           |                               |
|                       |           BX353873           |                               |
|                       |           AA323764           |                               |
|                       |           BX420432           |                               |
|                       |           BX332630           |                               |
|                       |           AL523586           |                               |
|                       |           BX401449           |                               |
|                       |           AL732116           |                               |
|                       |           BX447678           |                               |
|                       |           BX403139           |                               |
|                       |           CN344477           |                               |
|                       |           CN340512           |                               |
|                       |           BI913275           |                               |
|                       |           BI908956           |                               |
|                       |           BI756500           |                               |
|                       |           BG701456           |                               |
|                       |           BI754383           |                               |
|                       |           BI758679           |                               |
|                       |           BI905838           |                               |
|                       |           BI755250           |                               |
|                       |           BM920697           |                               |
|                       |           BX337522           |                               |
|                       |           BI755192           |                               |
|                       |           BM547649           |                               |
|                       |           BI911052           |                               |
|                       |           BI547178           |                               |
|                       |           BQ067333           |                               |
|                       |           BX398058           |                               |
|                       |           BQ067650           |                               |
|                       |           BI821297           |                               |
|                       |           BX419864           |                               |
|                       |           CA454346           |                               |
|                       |           BM923437           |                               |
|                       |           BI917791           |                               |
|                       |           BI917175           |                               |
|                       |           CN344476           |                               |
|                       |           BI830298           |                               |
|                       |           BI752617           |                               |
|                       |           BI770525           |                               |
|                       |           AY138815           |                               |
|                       |           AW449545           |                               |
|                       |           BU501653           |                               |
|                       |           BM545166           |                               |
|                       |           BQ354524           |                               |
|                       |           BM906292           |                               |
|                       |           BM923294           |                               |
|                       |           BG818443           |                               |
|                       |           BM906394           |                               |
|                       |           BQ222584           |                               |
|                       |           BP258076           |                               |
|                       |           BF348344           |                               |
|                       |           CA489208           |                               |
|                       |           BI826836           |                               |
|                       |           BQ424353           |                               |
|                       |           BI919182           |                               |
|                       |           CN344478           |                               |
|                       |           BQ777269           |                               |
|                       |           CA842201           |                               |
|                       |           CN344474           |                               |
|                       |           CN344480           |                               |
|                       |           CO249591           |                               |
|                       |           BP218280           |                               |
|                       |           CD359393           |                               |
|                       |           BP332504           |                               |
|                       |           AW629677           |                               |
|                       |           BM008177           |                               |
|                       |           CA488140           |                               |
|                       |           BX424624           |                               |
|                       |           CA393207           |                               |
|                       |           CA393188           |                               |
|                       |           BG896616           |                               |
|                       |           CA393208           |                               |
+-----------------------+------------------------------+-------------------------------+
|      #   38           | *Seq. mapped in strand Plus* | *Seq. mapped in strand Plus*  |
|                       |           AK074664           |          BY707813             |
|                       |           BP280941           |          CN835298             |
|                       |           AF497623           |          BQ109113             |
|                       |           CO245095           |          BI985206             |
|                       |           BP351258           |          CK780824             |
|                       |           AY046396           |          BI713252             |
|                       |           AY297044           |                               |
|                       |           AY297045           |                               |
|                       |           AY297046           |                               |
|                       |           AJ575813           |                               |
|                       |           CN402550           |                               |
|                       |           AK000048           |                               |
|                       |           CN402549           |                               |
|                       |           CN402548           |                               |
|                       |           BI029563           |                               |
|                       |           BG984513           |                               |
|                       |           BF806583           |                               |
|                       |           CO248115           |                               |
|                       |           BI010525           |                               |
|                       |           BQ721564           |                               |
|                       |           CN402551           |                               |
|                       |           AK000235           |                               |
|                       |           BP223875           |                               |
|                       |           BE905591           |                               |
|                       |           BQ942718           |                               |
|                       |           BI198633           |                               |
|                       |           BI199159           |                               |
|                       |           BI198267           |                               |
|                       |           BF310664           |                               |
|                       |           BE313619           |                               |
|                       |           BF758132           |                               |
|                       |           BQ723742           |                               |
|                       |           BQ084154           |                               |
|                       |           CN335029           |                               |
|                       |           BP265210           |                               |
|                       |           AW816479           |                               |
|                       |           BI759746           |                               |
|                       |           BU858032           |                               |
|                       |           BM821224           |                               |
|                       |             H18835           |                               |
|                       |           BX474984           |                               |
|                       |           CA488162           |                               |
|                       |           BI910079           |                               |
|                       |           CB266373           |                               |
|                       |           CA488844           |                               |
|                       |           CD628373           |                               |
|                       |           CD628374           |                               |
|                       |           CD628371           |                               |
|                       |           CD628372           |                               |
|                       |           CD628375           |                               |
|                       |           CD628376           |                               |
|                       |           CD628377           |                               |
|                       |           CD628378           |                               |
|                       |           BM797899           |                               |
|                       |           BG341728           |                               |
|                       |           BG759687           |                               |
|                       |           CA312705           |                               |
|                       |           BU687299           |                               |
|                       |           CD628379           |                               |
|                       |           CD628380           |                               |
|                       |           AW840703           |                               |
|                       |           BX497249           |                               |
|                       |           BM982099           |                               |
|                       |           BI836103           |                               |
|                       |           CD628381           |                               |
|                       |           CD628382           |                               |
|                       |           CD628384           |                               |
|                       |           CD628383           |                               |
|                       |           CB162735           |                               |
|                       |           BQ574097           |                               |
|                       |             N31660           |                               |
|                       |           BM682277           |                               |
|                       |           AA454774           |                               |
|                       |           AA551759           |                               |
|                       |           AI417040           |                               |
|                       |           AA694490           |                               |
|                       |           AA633315           |                               |
|                       |           AI344661           |                               |
|                       |           CN335030           |                               |
|                       |           AA708532           |                               |
|                       |           BQ010997           |                               |
|                       |           AA878567           |                               |
|                       |           BM857470           |                               |
|                       |           BM857696           |                               |
|                       |           BQ478452           |                               |
|                       |           AI913465           |                               |
|                       |           AI092908           |                               |
|                       |           AA026974           |                               |
|                       |             H18836           |                               |
|                       |           AI274428           |                               |
|                       | *Seq. mapped in strand Minus*| *Seq. mapped in strand Minus* |
|                       |           AI460004           |          CO041115             |
|                       |                              |          BI320070             |
|                       |                              |          BY658273             |
|                       |                              |          BB732972             |
|                       |                              |          BB733014             |
|                       |                              |          BB732727             |
+-----------------------+------------------------------+-------------------------------+
|      #   39           | *Seq. mapped in strand Plus* | *Seq. mapped in strand Plus*  |
|                       |           AK123796           |          BU938133             |
|                       |           BC032064           |          BY716362             |
|                       |           BC041612           |          AK016673             |
|                       |           BC064498           |          AI614158             |
|                       |           BC082249           |                               |
|                       | *Seq. mapped in strand Minus*| *Seq. mapped in strand Minus* |
|                       |           BC008284           |          BB688456             |
|                       |           AY358834           |          BB690952             |
|                       |           AL136786           |          BB683054             |
|                       |           CR614373           |          BY682726             |
+-----------------------+------------------------------+-------------------------------+
|      #   40           | *Seq. mapped in strand Plus* | *Seq. mapped in strand Plus*  |
|                       |           BG205060           |          BB845420             |
|                       |           BG205612           |          BY139910             |
|                       |           BG188591           |          BY784693             |
|                       |           BG201547           |          BB860790             |
|                       |           BG182293           |          CN526893             |
|                       |                              |          BY064646             |
|                       |                              |          BY020755             |
|                       |                              |          BY021537             |
|                       |                              |          BY007006             |
|                       |                              |          CF536779             |
|                       |                              |          AK087992             |
|                       |                              |          BC067029             |
|                       |                              |          BY123036             |
|                       |                              |          BY771150             |
|                       |                              |          BY787006             |
|                       |                              |          BY773052             |
|                       |                              |          BY745927             |
|                       |                              |          BY026248             |
|                       |                              |          BY025195             |
|                       |                              |          BY333143             |
|                       |                              |          CN525238             |
|                       |                              |          BY134427             |
|                       |                              |          BU611759             |
|                       |                              |          BY720072             |
|                       |                              |          BY738138             |
|                       |                              |          BY738492             |
|                       |                              |          CN534920             |
|                       |                              |          CN457572             |
|                       |                              |          CN461494             |
|                       |                              |          BU055595             |
|                       |                              |          CB244635             |
|                       |                              |          CB519433             |
|                       |                              |          CN527500             |
|                       |                              |          AI317065             |
|                       |                              |          CD544355             |
|                       |                              |          CB528121             |
|                       |                              |          CN524964             |
|                       |                              |          BB871965             |
|                       |                              |          BY130538             |
|                       |                              |          BY125414             |
|                       |                              |          BE372182             |
|                       |                              |          BY150157             |
|                       |                              |          BY146016             |
|                       |                              |          BY018047             |
|                       |                              |          CD350700             |
|                       |                              |          BY786657             |
|                       |                              |          BY775533             |
|                       |                              |          BY130867             |
|                       |                              |          CN457864             |
|                       |                              |          AV167451             |
|                       |                              |          CN673918             |
|                       |                              |          CB523487             |
|                       |                              |          CF733414             |
|                       |                              |          CK636176             |
|                       |                              |          CB521633             |
|                       |                              |          CN459224             |
|                       |                              |          CK639216             |
|                       |                              |          BQ563475             |
|                       |                              |          CF535814             |
|                       |                              |          CA324054             |
|                       |                              |          CN526879             |
|                       |                              |          CK637579             |
|                       |                              |          BQ442823             |
|                       |                              |          BU708985             |
|                       |                              |          CN455967             |
|                       |                              |          CN459157             |
|                       |                              |          CD549095             |
|                       |                              |          CN459526             |
|                       |                              |          CB525793             |
|                       |                              |          CN455393             |
|                       |                              |          AW910386             |
|                       |                              |          CF535631             |
|                       |                              |          CK639038             |
|                       |                              |          CB525610             |
|                       |                              |          CD349006             |
|                       |                              |          CF531725             |
|                       |                              |          CK635805             |
|                       |                              |          CK640143             |
|                       |                              |          BU702336             |
|                       |                              |          CB246636             |
|                       |                              |          CD544356             |
|                       |                              |          CN456247             |
|                       |                              |          CF727962             |
|                       |                              |          CN458740             |
|                       |                              |          CD549096             |
|                       |                              |          CK635238             |
|                       |                              |          BU701950             |
|                       |                              |          CD553727             |
|                       |                              |          CF735222             |
|                       |                              |          CN458099             |
|                       |                              |          CK640316             |
|                       |                              |          CN456963             |
|                       |                              |          CB526545             |
|                       |                              |          CK639554             |
|                       |                              |          CD552396             |
|                       |                              |          BE573227             |
|                       |                              |          CB525023             |
|                       |                              |          CN530330             |
|                       |                              |          CF733426             |
|                       |                              |          BG864409             |
|                       |                              |          AI613717             |
|                       |                              |          AA517239             |
|                       |                              |          CN461581             |
|                       |                              |          CN459690             |
|                       |                              |          CK634820             |
|                       |                              |          CF727344             |
|                       |                              |          CN458218             |
|                       |                              |          CN457406             |
|                       |                              |          CK639852             |
|                       |                              |          CF727606             |
|                       |                              |          CN688421             |
|                       |                              |          CN679584             |
|                       |                              |          CN682085             |
|                       |                              |          CF906879             |
|                       |                              |          CN672008             |
|                       |                              |          CF909146             |
|                       |                              |          CF902912             |
|                       |                              |          CF900484             |
|                       |                              |          CA882137             |
|                       |                              |          CD542381             |
|                       |                              |          CF162202             |
|                       |                              |          CF164605             |
|                       |                              |          CA535456             |
|                       |                              |          CD554641             |
|                       |                              |          CA530651             |
|                       |                              |          CD540136             |
|                       |                              |          CD552252             |
|                       |                              |          CA536055             |
|                       |                              |          CA886858             |
|                       |                              |          CK392137             |
|                       |                              |          CA534789             |
|                       |                              |          CA534934             |
|                       |                              |          CA535391             |
|                       |                              |          CA874299             |
|                       |                              |          CA879523             |
|                       |                              |          CN694414             |
|                       |                              |          CF165502             |
|                       |                              |          CA562988             |
|                       |                              |          CF165458             |
|                       |                              |          CN672503             |
|                       |                              |          CA531325             |
|                       |                              |          CD551032             |
|                       |                              |          CD553500             |
|                       |                              |          CN665619             |
|                       |                              |          CN667646             |
|                       |                              |          CD563262             |
|                       |                              |          CN681723             |
|                       |                              |          CA877958             |
|                       |                              |          CD550162             |
|                       |                              |          CF167907             |
|                       |                              |          CN672193             |
|                       |                              |          CN683204             |
|                       |                              |          CD553425             |
|                       |                              |          CD553633             |
|                       |                              |          CF163211             |
|                       |                              |          CD549218             |
|                       |                              |          CA563272             |
|                       |                              |          CN673756             |
|                       |                              |          CN664725             |
|                       |                              |          CN688751             |
|                       |                              |          CN695906             |
|                       |                              |          CD563563             |
|                       |                              |          CF163136             |
|                       |                              |          CA879526             |
|                       |                              |          CA893494             |
|                       |                              |          CD543908             |
|                       |                              |          CN676824             |
|                       |                              |          CF164554             |
|                       |                              |          CD539646             |
|                       |                              |          CF164380             |
|                       |                              |          CD549428             |
|                       |                              |          CD562263             |
|                       |                              |          CA563974             |
|                       |                              |          CA885066             |
|                       |                              |          CD538428             |
|                       |                              |          CD539279             |
|                       |                              |          CK391331             |
|                       |                              |          CN691398             |
|                       |                              |          CD544608             |
|                       |                              |          CN691422             |
|                       |                              |          CA565937             |
|                       |                              |          CD548237             |
|                       |                              |          CK391644             |
|                       |                              |          CA873128             |
|                       |                              |          CN687694             |
|                       |                              |          CA890236             |
|                       |                              |          CA565969             |
|                       |                              |          CD550904             |
|                       |                              |          CN678737             |
|                       |                              |          CK392539             |
|                       |                              |          CN668379             |
|                       |                              |          CK390678             |
|                       |                              |          CF897031             |
|                       |                              |          CN663602             |
|                       |                              |          CF898591             |
|                       |                              |          CF892599             |
|                       |                              |          CF898247             |
|                       |                              |          CD545047             |
|                       |                              |          CD545032             |
|                       |                              |          CF729586             |
|                       |                              |          CN681763             |
|                       |                              |          CF164738             |
|                       |                              |          CN688286             |
|                       |                              |          CN679573             |
|                       |                              |          CN688369             |
|                       |                              |          CF909217             |
|                       |                              |          CF735915             |
|                       |                              |          CA886371             |
|                       |                              |          CD561152             |
|                       |                              |          CK391901             |
|                       |                              |          CA528457             |
|                       |                              |          CF168429             |
|                       |                              |          CD540587             |
|                       |                              |          CF750764             |
|                       |                              |          CA872463             |
|                       |                              |          BY082107             |
|                       |                              |          BC059884             |
|                       |                              |          CA559598             |
|                       |                              |          BU950737             |
|                       |                              |          AA591503             |
|                       |                              |          BQ952355             |
|                       |                              |          AA794135             |
|                       |                              |          CN525847             |
|                       |                              |          BX528519             |
|                       |                              |            W59044             |
|                       |                              |          BE981514             |
|                       |                              |          BM942546             |
|                       |                              |          BF142232             |
|                       |                              |          CK639863             |
|                       |                              |          CF730175             |
|                       |                              |          BG277478             |
|                       |                              |          BU922243             |
|                       |                              |          CF951573             |
|                       |                              |          AU255075             |
|                       |                              |          BI990493             |
|                       |                              |          AA880132             |
|                       |                              |          BG175697             |
|                       |                              |          AW913343             |
|                       |                              |          CA549264             |
|                       |                              |          CA550599             |
|                       |                              |          CA549576             |
|                       |                              |          CA551547             |
|                       |                              |          CF154419             |
|                       |                              |          CA549735             |
|                       |                              |          CF153742             |
|                       |                              |          CA548311             |
|                       |                              |          CA553499             |
|                       |                              |          CA555522             |
|                       |                              |          BI791332             |
|                       |                              |          AA118121             |
|                       |                              |          CO046416             |
|                       |                              |          AA522402             |
|                       |                              |          AA522231             |
|                       |                              |          CA527125             |
|                       |                              |          CA549415             |
|                       |                              |          BU611805             |
|                       |                              |          BG518579             |
|                       |                              |          BC002239             |
|                       |                              |          CK022499             |
|                       |                              |          CF735043             |
|                       |                              |          AA511890             |
|                       |                              |          AA237537             |
|                       |                              |          CF741649             |
|                       |                              |          CN690611             |
|                       |                              |          AA050144             |
|                       |                              |          BX528584             |
|                       |                              |          AA009281             |
|                       |                              |          AW986894             |
|                       |                              |          BE198634             |
|                       |                              |          BF165039             |
|                       |                              |          CN458250             |
|                       |                              |          AA794708             |
|                       |                              |          AL363077             |
|                       |                              |          CN459672             |
|                       |                              |          AA175000             |
|                       |                              |          AA199036             |
|                       |                              |          BQ928151             |
|                       |                              |          BG277528             |
|                       |                              |          CB840804             |
|                       |                              |          AA213188             |
|                       |                              |          CN681116             |
|                       |                              |          BY103271             |
|                       |                              |          BF147308             |
|                       |                              |          BF682684             |
|                       |                              |            W98266             |
|                       |                              |          BX529740             |
|                       |                              |          BF021360             |
|                       |                              |          AA473232             |
|                       |                              |          BY388118             |
|                       |                              |          BE850976             |
|                       |                              |          AA543256             |
|                       |                              |          AA174400             |
|                       |                              |          BX527090             |
|                       |                              |          AI529776             |
|                       |                              |          CB317874             |
|                       |                              |          AI529233             |
|                       |                              |          AA869894             |
|                       |                              |          CB202645             |
|                       |                              |          AI892506             |
|                       |                              |          BX522310             |
|                       |                              |          AI325812             |
|                       |                              |          AA116880             |
|                       |                              |          BE625232             |
|                       |                              |          AA466839             |
|                       |                              |          BF123409             |
|                       |                              |          BQ769594             |
|                       |                              |          BE915338             |
|                       |                              |          CO043120             |
|                       |                              |          BY758447             |
|                       |                              |          BE625858             |
|                       |                              |          AI227299             |
|                       |                              |          AI327140             |
|                       |                              |          BY758801             |
|                       |                              |          BM211313             |
|                       |                              |          BG148417             |
|                       |                              |          BQ563474             |
|                       |                              |          BQ033059             |
|                       |                              |          BQ031558             |
|                       |                              |          BG228507             |
|                       |                              |          AI848393             |
|                       |                              |          AI529578             |
|                       |                              |          AI196730             |
|                       |                              |          BI791028             |
|                       |                              |          BG089927             |
|                       |                              |          BC027841             |
|                       |                              |          BU951203             |
|                       |                              |          AI482029             |
|                       |                              |          BI696003             |
|                       |                              |          AI480854             |
|                       | *Seq. mapped in strand Minus*| *Seq. mapped in strand Minus* |
|                       |           CB045334           |          CK376478             |
|                       |           AA731323           |                               |
|                       |           BG212226           |                               |
|                       |           BQ027966           |                               |
|                       |           AI860345           |                               |
|                       |           AI640541           |                               |
|                       |           AI346413           |                               |
|                       |           AA830697           |                               |
|                       |           BF591722           |                               |
|                       |           AI769328           |                               |
|                       |           AI753169           |                               |
|                       |           AA830359           |                               |
|                       |           BM740162           |                               |
|                       |           BM471098           |                               |
|                       |           BG171489           |                               |
|                       |           AA506565           |                               |
|                       |             H05116           |                               |
|                       |           AW517290           |                               |
|                       |           CA448273           |                               |
|                       |           BU737225           |                               |
|                       |           AI077968           |                               |
|                       |           BX117258           |                               |
|                       |           AW069818           |                               |
|                       |           BE301097           |                               |
|                       |           AA845520           |                               |
|                       |           CA445640           |                               |
|                       |           BG213300           |                               |
|                       |           AW516032           |                               |
|                       |           BG195128           |                               |
|                       |           BG207505           |                               |
|                       |           BG184192           |                               |
|                       |           BG236105           |                               |
|                       |           BX472592           |                               |
|                       |           CA417556           |                               |
|                       |           BG186320           |                               |
|                       |           BG210670           |                               |
|                       |           AI434946           |                               |
|                       |           AI765677           |                               |
|                       |           AA861764           |                               |
|                       |           AI700547           |                               |
|                       |           BX393120           |                               |
|                       |           BX340934           |                               |
|                       |           CR608663           |                               |
|                       |           BX537417           |                               |
|                       |           BM314787           |                               |
|                       |           BF305995           |                               |
|                       |           AI028258           |                               |
|                       |           AI952853           |                               |
|                       |           BG169064           |                               |
|                       |           AI640552           |                               |
|                       |           AK123909           |                               |
|                       |           AK127087           |                               |
|                       |           BQ002085           |                               |
|                       |           BG939383           |                               |
|                       |           BQ004266           |                               |
|                       |           BM990212           |                               |
|                       |           CA416332           |                               |
|                       |           BQ020612           |                               |
|                       |           CA424006           |                               |
|                       |           CA420386           |                               |
|                       |           BQ183851           |                               |
|                       |           CA441555           |                               |
|                       |           BF792637           |                               |
|                       |           BU675225           |                               |
|                       |           CA424743           |                               |
|                       |           BM973665           |                               |
|                       |           CA311899           |                               |
|                       |           CA423692           |                               |
|                       |           BQ772905           |                               |
|                       |           BQ009290           |                               |
|                       |           AI560909           |                               |
|                       |           AA707568           |                               |
|                       |           BQ775737           |                               |
|                       |           BI492448           |                               |
|                       |           BM684491           |                               |
|                       |           BM971682           |                               |
|                       |           CA446730           |                               |
|                       |           BM975320           |                               |
|                       |           BU682593           |                               |
|                       |           CA314747           |                               |
|                       |           BU685494           |                               |
|                       |           AI346348           |                               |
|                       |           BQ026930           |                               |
|                       |           CA419321           |                               |
|                       |           BI086428           |                               |
|                       |           BC008318           |                               |
|                       |           BC065552           |                               |
|                       |             D28473           |                               |
|                       |           BX647236           |                               |
|                       |           BX647430           |                               |
|                       |             U04953           |                               |
|                       |           BX647071           |                               |
|                       |           BX648567           |                               |
|                       |           BG219095           |                               |
|                       |           BI714755           |                               |
|                       |           BX538109           |                               |
|                       |           CA419504           |                               |
|                       |           BE138780           |                               |
|                       |           AF113680           |                               |
|                       |           BX537429           |                               |
|                       |           BE781806           |                               |
|                       |           BG201770           |                               |
|                       |           AI749572           |                               |
|                       |           BG197671           |                               |
|                       |           AL515956           |                               |
|                       |           BG220076           |                               |
|                       |           AL045614           |                               |
|                       |           BI223847           |                               |
|                       |           BM907790           |                               |
|                       |           BU568116           |                               |
|                       |           BG201771           |                               |
|                       |           AW578752           |                               |
|                       |           AW578805           |                               |
|                       |           AW578806           |                               |
|                       |           BU854400           |                               |
|                       |           BF819046           |                               |
|                       |           CA453701           |                               |
|                       |           BG200850           |                               |
|                       |           BG403461           |                               |
|                       |           BG253086           |                               |
|                       |           BF666837           |                               |
|                       |           AV715394           |                               |
|                       |           BM770622           |                               |
|                       |           BF665724           |                               |
|                       |           AL046585           |                               |
|                       |           BU161772           |                               |
|                       |           BM758596           |                               |
|                       |           BM758954           |                               |
|                       |           BM766515           |                               |
|                       |           BM757695           |                               |
|                       |           BM758783           |                               |
|                       |           BM758080           |                               |
|                       |           AA001862           |                               |
|                       |           AI525564           |                               |
|                       |           AA609679           |                               |
|                       |           BI039961           |                               |
|                       |           BE536197           |                               |
|                       |           BE538196           |                               |
|                       |           AW392939           |                               |
|                       |           BM771462           |                               |
|                       |           BM749767           |                               |
|                       |           BM758791           |                               |
|                       |           BM758092           |                               |
|                       |           BM757992           |                               |
|                       |           BM758035           |                               |
|                       |           BM758484           |                               |
|                       |           BM758748           |                               |
|                       |           BU902657           |                               |
|                       |           BF369652           |                               |
|                       |           BF750494           |                               |
|                       |           BM749271           |                               |
|                       |           BM747187           |                               |
|                       |           BF798329           |                               |
|                       |           BM758742           |                               |
|                       |           BF351296           |                               |
|                       |           BM748422           |                               |
|                       |           BE165120           |                               |
|                       |           BM758885           |                               |
|                       |           BM757679           |                               |
|                       |           CD102483           |                               |
|                       |           BE165302           |                               |
|                       |           BE165125           |                               |
|                       |           BE165311           |                               |
|                       |           BE165320           |                               |
|                       |           BE165317           |                               |
|                       |           BE165316           |                               |
|                       |           BE165321           |                               |
|                       |           BE165129           |                               |
|                       |           BM748475           |                               |
|                       |           BE165118           |                               |
|                       |           BE165121           |                               |
|                       |           BE165126           |                               |
|                       |           BE165128           |                               |
|                       |           BE165122           |                               |
|                       |           AI678660           |                               |
|                       |           BE165123           |                               |
|                       |           CB142581           |                               |
|                       |           BE165127           |                               |
|                       |           BM748630           |                               |
|                       |           AW392965           |                               |
|                       |           CB133870           |                               |
|                       |           CB143389           |                               |
|                       |           BM743305           |                               |
|                       |           BM759373           |                               |
|                       |             T95421           |                               |
|                       |           BM757683           |                               |
|                       |           CB159860           |                               |
|                       |           BM741396           |                               |
|                       |           BM560654           |                               |
|                       |           BM749240           |                               |
|                       |           BM748877           |                               |
|                       |           BM749842           |                               |
|                       |           BM748468           |                               |
|                       |           BG538437           |                               |
|                       |           BG741449           |                               |
|                       |           BM748462           |                               |
|                       |           BM758697           |                               |
|                       |           BM758334           |                               |
|                       |           CN364276           |                               |
|                       |           CK818659           |                               |
|                       |           BM758283           |                               |
|                       |           BM758307           |                               |
|                       |           CF138813           |                               |
|                       |           BG497043           |                               |
|                       |           BI715313           |                               |
|                       |             R09575           |                               |
|                       |           BG675757           |                               |
|                       |           BG960053           |                               |
|                       |           CB148031           |                               |
|                       |           BG260687           |                               |
|                       |           CB123961           |                               |
|                       |             D12018           |                               |
|                       |           AW021490           |                               |
|                       |           BF911699           |                               |
|                       |           BF794847           |                               |
|                       |           BG178229           |                               |
|                       |           BF210604           |                               |
|                       |           BG254964           |                               |
|                       |           BE832850           |                               |
|                       |           BE165097           |                               |
|                       |           CB321950           |                               |
|                       |           BE165093           |                               |
|                       |           BI257825           |                               |
|                       |           BE165105           |                               |
|                       |           BQ720453           |                               |
|                       |           BE165107           |                               |
|                       |           BE165100           |                               |
|                       |           BE165081           |                               |
|                       |           BE165124           |                               |
|                       |           BI093846           |                               |
|                       |           BE165096           |                               |
|                       |           BE165301           |                               |
|                       |           CA773162           |                               |
|                       |           BM503418           |                               |
|                       |           BE165309           |                               |
|                       |           BE165315           |                               |
|                       |           BE165305           |                               |
|                       |           BE165318           |                               |
|                       |           BE720245           |                               |
|                       |           BE165322           |                               |
|                       |           BG028620           |                               |
|                       |           BE165308           |                               |
|                       |           CB135385           |                               |
|                       |           BE165323           |                               |
|                       |           BE165319           |                               |
|                       |           AW965767           |                               |
|                       |           BE165324           |                               |
|                       |           BE165299           |                               |
|                       |           BE165325           |                               |
|                       |           BE165300           |                               |
|                       |           BE165326           |                               |
|                       |           BM749222           |                               |
|                       |           BM749732           |                               |
|                       |           BE165304           |                               |
|                       |           BE165312           |                               |
|                       |           BE833524           |                               |
|                       |           BM748539           |                               |
|                       |           BE165075           |                               |
|                       |           BE165076           |                               |
|                       |           AL046075           |                               |
|                       |           CB131950           |                               |
|                       |           BG121433           |                               |
|                       |             H77537           |                               |
|                       |           BM758782           |                               |
|                       |           BE165314           |                               |
|                       |           BE165111           |                               |
|                       |           CF597337           |                               |
|                       |           BF211086           |                               |
|                       |             H48605           |                               |
|                       |           BM758333           |                               |
|                       |           BE165313           |                               |
|                       |           CK430448           |                               |
|                       |           BE165550           |                               |
|                       |           BF989458           |                               |
|                       |           BQ895987           |                               |
|                       |           BM749779           |                               |
|                       |           BG900597           |                               |
|                       |             H90174           |                               |
|                       |           CN364273           |                               |
|                       |           BP872780           |                               |
|                       |           BM817481           |                               |
|                       |           BE710072           |                               |
|                       |           CB141477           |                               |
|                       |           CB128188           |                               |
|                       |           BG771386           |                               |
|                       |             D58525           |                               |
|                       |           BQ225707           |                               |
|                       |           BE541706           |                               |
|                       |           BM790726           |                               |
|                       |           BP428705           |                               |
|                       |           CD677196           |                               |
|                       |           AA237050           |                               |
|                       |           BQ431564           |                               |
|                       |           BM454757           |                               |
|                       |             R20123           |                               |
|                       |           AV721536           |                               |
|                       |             D59216           |                               |
|                       |           AA129707           |                               |
|                       |           BF214837           |                               |
|                       |           CF145520           |                               |
|                       |           CF139786           |                               |
|                       |           AA345973           |                               |
|                       |           AA281522           |                               |
|                       |           BE739144           |                               |
|                       |           BG171226           |                               |
|                       |           BF764588           |                               |
|                       |           AA363546           |                               |
|                       |           CF145456           |                               |
|                       |           BM698765           |                               |
|                       |           CF123062           |                               |
|                       |           AL043922           |                               |
|                       |           BG177430           |                               |
|                       |           AA345592           |                               |
|                       |           CN364275           |                               |
|                       |           BE892636           |                               |
|                       |             F05893           |                               |
|                       |           AA352146           |                               |
|                       |           CD522049           |                               |
|                       |           BM463470           |                               |
|                       |             H16418           |                               |
|                       |           CD000754           |                               |
|                       |           BF965768           |                               |
|                       |           CN364272           |                               |
|                       |           CB146555           |                               |
|                       |           CF145014           |                               |
|                       |           CF146528           |                               |
|                       |           BM931004           |                               |
|                       |           BQ230269           |                               |
|                       |           AW063637           |                               |
|                       |           AU120155           |                               |
|                       |           CF145313           |                               |
|                       |           BI255226           |                               |
|                       |             H05581           |                               |
|                       |           AW383059           |                               |
|                       |           AW383001           |                               |
|                       |           CF140155           |                               |
|                       |           BU164327           |                               |
|                       |             F12077           |                               |
|                       |           BQ952123           |                               |
|                       |           AW383045           |                               |
|                       |             H75273           |                               |
|                       |             F06599           |                               |
|                       |             H96438           |                               |
|                       |           BG259431           |                               |
|                       |             F01044           |                               |
|                       |           BM786969           |                               |
|                       |           BE564982           |                               |
|                       |           AU124199           |                               |
|                       |           AU124321           |                               |
|                       |           BU155228           |                               |
|                       |           BM750057           |                               |
|                       |           BG396225           |                               |
|                       |           BG677553           |                               |
|                       |           BM806948           |                               |
|                       |           BF090227           |                               |
|                       |           BM755798           |                               |
|                       |             H53244           |                               |
|                       |             H54474           |                               |
|                       |             D83822           |                               |
|                       |           AU138963           |                               |
|                       |           AU125308           |                               |
|                       |           BM750294           |                               |
|                       |           AU125957           |                               |
|                       |           BE542795           |                               |
|                       |           AW383796           |                               |
|                       |           AA112747           |                               |
|                       |           AU119368           |                               |
|                       |             T27373           |                               |
|                       |           BM749511           |                               |
|                       |           BE780803           |                               |
|                       |           BP871991           |                               |
|                       |           CN364290           |                               |
|                       |             R17466           |                               |
|                       |           BI755773           |                               |
|                       |           BG032650           |                               |
|                       |           AA461261           |                               |
|                       |           AW411264           |                               |
|                       |           AU125785           |                               |
|                       |           AU124444           |                               |
|                       |           BI857965           |                               |
|                       |           BG389725           |                               |
|                       |             T66378           |                               |
|                       |           BG168085           |                               |
|                       |           BU173536           |                               |
|                       |           BG393053           |                               |
|                       |             T94824           |                               |
|                       |             R19993           |                               |
|                       |           BF968240           |                               |
|                       |           BM559235           |                               |
|                       |           BM810283           |                               |
|                       |           AA113328           |                               |
|                       |           AV750373           |                               |
|                       |           BQ356063           |                               |
|                       |           BG876599           |                               |
|                       |           BE765222           |                               |
|                       |           BF308161           |                               |
|                       |           AA336080           |                               |
|                       |           BE747245           |                               |
|                       |           BQ303415           |                               |
|                       |           BE299407           |                               |
|                       |           CB046468           |                               |
|                       |           CB114678           |                               |
|                       |           BP871587           |                               |
|                       |           CN364280           |                               |
|                       |           CD358444           |                               |
|                       |           BU159239           |                               |
|                       |           BP872699           |                               |
|                       |             F00580           |                               |
|                       |           AU127800           |                               |
|                       |           BG257625           |                               |
|                       |             F07718           |                               |
|                       |           BP383367           |                               |
|                       |           AU124252           |                               |
|                       |           BP283493           |                               |
|                       |           BQ716521           |                               |
|                       |           AA205764           |                               |
|                       |           BP281141           |                               |
|                       |           BG749058           |                               |
|                       |           BM752119           |                               |
|                       |           BP245784           |                               |
|                       |           BQ226894           |                               |
|                       |           AW410133           |                               |
|                       |           AU133214           |                               |
|                       |           AA361527           |                               |
|                       |           BM011584           |                               |
|                       |           BQ232220           |                               |
|                       |           AU129443           |                               |
|                       |           CB046469           |                               |
|                       |           BP281739           |                               |
|                       |           AA188853           |                               |
|                       |           BG253258           |                               |
|                       |           BE886215           |                               |
|                       |           AL515957           |                               |
|                       |             N86474           |                               |
|                       |           BE902478           |                               |
|                       |           BQ228704           |                               |
|                       |           BQ337924           |                               |
|                       |           BM456630           |                               |
|                       |           AU131459           |                               |
|                       |           BP873177           |                               |
|                       |           BE164957           |                               |
|                       |           AL134270           |                               |
|                       |           BF204994           |                               |
|                       |           AA984611           |                               |
|                       |           AA984618           |                               |
|                       |           AW385991           |                               |
|                       |           CN364277           |                               |
|                       |             W52666           |                               |
|                       |             W52498           |                               |
|                       |           BM312232           |                               |
|                       |           BU077954           |                               |
|                       |           AI926550           |                               |
|                       |           BM272286           |                               |
|                       |           BQ614392           |                               |
|                       |           BM312314           |                               |
|                       |             H04695           |                               |
|                       |           BM272513           |                               |
|                       |           BQ614610           |                               |
|                       |           BE269408           |                               |
|                       |           BE560061           |                               |
|                       |           BE560487           |                               |
|                       |           BE513909           |                               |
|                       |           BE559985           |                               |
|                       |           CN364283           |                               |
|                       |           BE269991           |                               |
|                       |           AW504455           |                               |
|                       |           BM472132           |                               |
|                       |           BG824592           |                               |
|                       |           BX457604           |                               |
|                       |           BQ422886           |                               |
|                       |           CN364285           |                               |
|                       |           BQ337644           |                               |
|                       |           CN364289           |                               |
|                       |           CF136297           |                               |
|                       |           CV337648           |                               |
|                       |           BI092042           |                               |
|                       |           BG483364           |                               |
|                       |           BE269983           |                               |
|                       |           CD625021           |                               |
|                       |           CD625014           |                               |
|                       |           CD625015           |                               |
|                       |           CD625019           |                               |
|                       |           CD625020           |                               |
|                       |           CD625022           |                               |
|                       |           CD625023           |                               |
|                       |           CD625017           |                               |
|                       |           CD625018           |                               |
|                       |           BI116650           |                               |
|                       |           AU128305           |                               |
|                       |           CD625016           |                               |
|                       |           CN364286           |                               |
|                       |           AI630503           |                               |
|                       |           AI630332           |                               |
|                       |           BQ433464           |                               |
|                       |           BG027927           |                               |
|                       |           BE790397           |                               |
|                       |           AA313205           |                               |
|                       |           CN364292           |                               |
|                       |           BQ351562           |                               |
|                       |           BG257838           |                               |
|                       |           BQ215248           |                               |
|                       |           CN364287           |                               |
|                       |           AW951652           |                               |
|                       |           BX462266           |                               |
|                       |           CV339759           |                               |
|                       |           AW954726           |                               |
|                       |           AA307923           |                               |
|                       |           AW500590           |                               |
|                       |           AU131492           |                               |
|                       |           AA112742           |                               |
|                       |           CF785846           |                               |
|                       |           CF272285           |                               |
|                       |           AW504963           |                               |
|                       |           BM016805           |                               |
|                       |           AU117116           |                               |
|                       |           BE867434           |                               |
|                       |           AL138303           |                               |
|                       |           BQ316394           |                               |
|                       |           BQ363431           |                               |
|                       |           CN364278           |                               |
|                       |           BQ310744           |                               |
|                       |           CN364279           |                               |
|                       |           BG874496           |                               |
|                       |           CN364281           |                               |
|                       |           CN364288           |                               |
|                       |           BU161511           |                               |
|                       |           BG388111           |                               |
|                       |           BG258684           |                               |
|                       |           BM462071           |                               |
|                       |           CF137819           |                               |
|                       |           BE207333           |                               |
|                       |           BU428459           |                               |
|                       |           BQ221831           |                               |
|                       |           CB155330           |                               |
|                       |           BM465263           |                               |
|                       |           AU133459           |                               |
|                       |           BM835050           |                               |
|                       |           CD654989           |                               |
|                       |           AA316254           |                               |
|                       |           AA506539           |                               |
|                       |           BU430552           |                               |
|                       |           CF136869           |                               |
|                       |           BU172546           |                               |
|                       |           BU431464           |                               |
|                       |           BQ343790           |                               |
|                       |           BQ292285           |                               |
|                       |           CN364291           |                               |
|                       |           BE781660           |                               |
|                       |           BQ292296           |                               |
|                       |           BQ292255           |                               |
|                       |           AW504752           |                               |
|                       |           CV347101           |                               |
|                       |           BP872882           |                               |
|                       |           BU429330           |                               |
|                       |           CB045335           |                               |
|                       |           BE777930           |                               |
|                       |           AA332042           |                               |
|                       |           BM465848           |                               |
|                       |           BM477152           |                               |
|                       |           BM905436           |                               |
|                       |           AL704957           |                               |
|                       |           BP871916           |                               |
|                       |           BU431263           |                               |
|                       |           BQ292270           |                               |
|                       |           BQ215612           |                               |
|                       |           BQ339005           |                               |
|                       |           CV334892           |                               |
|                       |           BQ213599           |                               |
|                       |           AU130080           |                               |
|                       |           CD657050           |                               |
|                       |           BU194849           |                               |
|                       |           CD652539           |                               |
|                       |           AU133063           |                               |
|                       |           CD244163           |                               |
|                       |           BU149422           |                               |
|                       |           AU142845           |                               |
|                       |           AA331593           |                               |
|                       |           CD653740           |                               |
|                       |           BQ362516           |                               |
|                       |           BQ362514           |                               |
|                       |           BQ317879           |                               |
|                       |           BQ339003           |                               |
|                       |           CN364284           |                               |
|                       |           CD655178           |                               |
|                       |           AU140501           |                               |
|                       |           AW501966           |                               |
|                       |           BX457874           |                               |
|                       |           BU429855           |                               |
|                       |           CF594050           |                               |
|                       |           BF968418           |                               |
|                       |           BG176735           |                               |
|                       |           BI560697           |                               |
|                       |           CD643171           |                               |
|                       |           BQ217223           |                               |
|                       |           BG106039           |                               |
|                       |           CF594234           |                               |
|                       |           BQ425587           |                               |
|                       |           BG258391           |                               |
|                       |           CB988341           |                               |
|                       |           CD109512           |                               |
|                       |           BM788719           |                               |
|                       |           BP871577           |                               |
|                       |           AU134475           |                               |
|                       |           BP871899           |                               |
|                       |           BP872275           |                               |
|                       |           BP872642           |                               |
|                       |           BP872615           |                               |
|                       |           AL602153           |                               |
|                       |           BP872200           |                               |
|                       |           CN364282           |                               |
|                       |           AL517345           |                               |
|                       |           BP872877           |                               |
|                       |           AL602230           |                               |
|                       |           CD643184           |                               |
|                       |           BM832525           |                               |
|                       |           CD653021           |                               |
|                       |           BP871569           |                               |
|                       |           BP872322           |                               |
|                       |           BP872865           |                               |
|                       |           BP872600           |                               |
|                       |           AU143186           |                               |
|                       |           BX470413           |                               |
|                       |           BP872328           |                               |
|                       |           BG717044           |                               |
|                       |           BP873524           |                               |
|                       |           BP871570           |                               |
|                       |           BP871856           |                               |
|                       |           BP871894           |                               |
|                       |           BP871876           |                               |
|                       |           BP872492           |                               |
|                       |           BP872815           |                               |
|                       |           BP872946           |                               |
|                       |           BP872317           |                               |
|                       |           BP872749           |                               |
|                       |           BP872687           |                               |
|                       |           BP871594           |                               |
|                       |           BP871747           |                               |
|                       |           BP872089           |                               |
|                       |           BP872329           |                               |
|                       |           BP872526           |                               |
|                       |           BP872788           |                               |
|                       |           BP872798           |                               |
|                       |           BP872513           |                               |
|                       |           BP872637           |                               |
|                       |           BP872790           |                               |
|                       |           BP872948           |                               |
|                       |           BP872752           |                               |
|                       |           BP871580           |                               |
|                       |           BP872566           |                               |
|                       |           BP872147           |                               |
|                       |           BP873564           |                               |
|                       |           BP873434           |                               |
|                       |           BP872116           |                               |
|                       |           BP873365           |                               |
|                       |           CF137562           |                               |
|                       |           BP872567           |                               |
|                       |           BP871743           |                               |
|                       |           BI914604           |                               |
|                       |           BX644253           |                               |
|                       |           BX642640           |                               |
|                       |           BP871717           |                               |
|                       |           BP872659           |                               |
|                       |           BP872661           |                               |
|                       |           BP873199           |                               |
|                       |           BG257439           |                               |
|                       |           BM828429           |                               |
|                       |           BI335512           |                               |
|                       |           BX955350           |                               |
|                       |           CF137495           |                               |
|                       |           BP873315           |                               |
|                       |           BP872810           |                               |
|                       |           BP873486           |                               |
|                       |           BP872475           |                               |
|                       |           BP872478           |                               |
|                       |           AA312424           |                               |
|                       |           BP872665           |                               |
|                       |           BP872546           |                               |
|                       |           BP872771           |                               |
|                       |           BP872215           |                               |
|                       |           BP872514           |                               |
|                       |           BP873376           |                               |
|                       |           BP872367           |                               |
|                       |           BP872548           |                               |
|                       |           AA452216           |                               |
|                       |           BG720308           |                               |
|                       |           BP872438           |                               |
|                       |           BP873473           |                               |
|                       |           CN364294           |                               |
|                       |           BX475190           |                               |
|                       |           BP872033           |                               |
|                       |           AW501142           |                               |
|                       |           BX474191           |                               |
|                       |           BP871841           |                               |
|                       |           BP873415           |                               |
|                       |           BP872400           |                               |
|                       |           BP872799           |                               |
|                       |           BX472799           |                               |
|                       |           BP350613           |                               |
|                       |           BU428617           |                               |
|                       |           BU428892           |                               |
|                       |           AA355956           |                               |
|                       |           BX474575           |                               |
|                       |           CD655428           |                               |
|                       |           BX503103           |                               |
|                       |           BX485144           |                               |
|                       |           CF137332           |                               |
|                       |           AA452990           |                               |
|                       |           BX950908           |                               |
|                       |           AL705460           |                               |
|                       |           CN364274           |                               |
|                       |           AL600648           |                               |
|                       |           CD708060           |                               |
+-----------------------+------------------------------+-------------------------------+
|      #   41           | *Seq. mapped in strand Plus* | *Seq. mapped in strand Plus*  |
|                       |           BF445351           |          AK013297             |
|                       |           BE670532           |          AK078448             |
|                       |           BF446547           |          AK087998             |
|                       |           BF475312           |          BC056962             |
|                       |           BE671389           |                               |
|                       |           BE670630           |                               |
|                       |           BE673887           |                               |
|                       |           BE669481           |                               |
|                       |           BF223766           |                               |
|                       |           BE674326           |                               |
|                       |           BF115801           |                               |
|                       |           BF224160           |                               |
|                       |           BF110922           |                               |
|                       |           BF223518           |                               |
|                       |           BF432990           |                               |
|                       |           AI699091           |                               |
|                       |           BE326386           |                               |
|                       |           AW664749           |                               |
|                       |           BE219041           |                               |
|                       |           AI273218           |                               |
|                       |           BE327056           |                               |
|                       |           BE328193           |                               |
|                       |           BE222424           |                               |
|                       |           AI091177           |                               |
|                       |           AW770780           |                               |
|                       |           BE468026           |                               |
|                       |           BE220356           |                               |
|                       |           AI762301           |                               |
|                       |           BE676250           |                               |
|                       |           BE672517           |                               |
|                       |           BE550774           |                               |
|                       |           AI435280           |                               |
|                       |           BE549963           |                               |
|                       |           BE326258           |                               |
|                       |           BE550220           |                               |
|                       |           AW134958           |                               |
|                       |           BE503873           |                               |
|                       |           BE348664           |                               |
|                       |           BE672161           |                               |
|                       |           BE855483           |                               |
|                       |           BF589209           |                               |
|                       |           AV751998           |                               |
|                       |           AI809054           |                               |
|                       |           AI970661           |                               |
|                       |           AI475586           |                               |
|                       |           BF194958           |                               |
|                       |           BF590212           |                               |
|                       |           AA935688           |                               |
|                       |           AA936448           |                               |
|                       |           AI298799           |                               |
|                       |           BE327270           |                               |
|                       |           AI083816           |                               |
|                       |           AI494353           |                               |
|                       |           AI362811           |                               |
|                       |           AI290724           |                               |
|                       |           AI074026           |                               |
|                       |           AI436612           |                               |
|                       |           AI470242           |                               |
|                       |           AI457864           |                               |
|                       |           AI693129           |                               |
|                       |           AI564071           |                               |
|                       |           AI868272           |                               |
|                       |           AI262485           |                               |
|                       |           AI281263           |                               |
|                       |           AI304909           |                               |
|                       |           AI368162           |                               |
|                       |           AW104564           |                               |
|                       |           AI262473           |                               |
|                       |           AI215919           |                               |
|                       |           AI350155           |                               |
|                       |           AI367117           |                               |
|                       |           AI760846           |                               |
|                       |           AI284960           |                               |
|                       |           AI356541           |                               |
|                       |           AI568381           |                               |
|                       |           AI285026           |                               |
|                       |           AI056473           |                               |
|                       |           AI288998           |                               |
|                       |           AI400599           |                               |
|                       |           AI494031           |                               |
|                       |           AI689921           |                               |
|                       |           AI281094           |                               |
|                       |           AI130925           |                               |
|                       |           AI095860           |                               |
|                       |           AI083690           |                               |
|                       |           AI281964           |                               |
|                       |           AW243114           |                               |
|                       |           AI475844           |                               |
|                       |           AI524075           |                               |
|                       |           AI681803           |                               |
|                       |           AI459009           |                               |
|                       |           AI634561           |                               |
|                       |           AI692652           |                               |
|                       |           AI703324           |                               |
|                       |           AI268428           |                               |
|                       |           AI693620           |                               |
|                       |           AI740984           |                               |
|                       |           AI361755           |                               |
|                       |           AI700754           |                               |
|                       |           AI700970           |                               |
|                       |           AI741170           |                               |
|                       |           AI912106           |                               |
|                       |           AI346725           |                               |
|                       |           AI694284           |                               |
|                       |           AI761746           |                               |
|                       |           AI912101           |                               |
|                       |           AI290984           |                               |
|                       |           BC040035           |                               |
|                       |           BI856718           |                               |
|                       |           BF108629           |                               |
|                       |           BQ653726           |                               |
|                       |           BI198838           |                               |
|                       |           CA334787           |                               |
|                       |           AI760706           |                               |
|                       |           CA952560           |                               |
|                       |           BI544817           |                               |
|                       |           BQ883789           |                               |
|                       |           AA988466           |                               |
|                       |           BX325061           |                               |
|                       |           BX325062           |                               |
|                       |           CR595159           |                               |
|                       |           BU737881           |                               |
|                       |           BQ631529           |                               |
|                       |           BQ644139           |                               |
|                       |           BE387384           |                               |
|                       |           AI693266           |                               |
|                       |           BE730547           |                               |
|                       |           AA911851           |                               |
|                       |             N34298           |                               |
|                       |           BM700080           |                               |
|                       |           BF433235           |                               |
|                       |           BF339381           |                               |
|                       |           BQ652524           |                               |
|                       |           BM561742           |                               |
|                       |           BE908283           |                               |
|                       |           BE882251           |                               |
|                       |           BF437902           |                               |
|                       |           BI761478           |                               |
|                       |             W69612           |                               |
|                       |           BQ685348           |                               |
|                       |           BF438160           |                               |
|                       |           CK431227           |                               |
|                       |           BQ959727           |                               |
|                       |           BQ422154           |                               |
|                       |           BG492118           |                               |
|                       |           BE798837           |                               |
|                       |           BU730494           |                               |
|                       |             H87749           |                               |
|                       |           AA911899           |                               |
|                       |           AA985589           |                               |
|                       |           BM918325           |                               |
|                       |           BM810811           |                               |
|                       |           AA971730           |                               |
|                       |           CF130726           |                               |
|                       |           BQ086163           |                               |
|                       |           CD672640           |                               |
|                       |           BM707716           |                               |
|                       |           CA952556           |                               |
|                       |           BM694350           |                               |
|                       |           AV752062           |                               |
|                       |           BE791637           |                               |
|                       |           BF446614           |                               |
|                       |           BI195445           |                               |
|                       |           BI859112           |                               |
|                       |           BI550262           |                               |
|                       |           BI550421           |                               |
|                       |           CB047107           |                               |
|                       |           AY232291           |                               |
|                       |           CB047106           |                               |
|                       |           AA431605           |                               |
|                       |           CN371234           |                               |
|                       |           CK823281           |                               |
|                       |           BQ631253           |                               |
|                       |           BE274155           |                               |
|                       |           BQ631971           |                               |
|                       |           BM966803           |                               |
|                       |           CK823282           |                               |
|                       |           BM750611           |                               |
|                       |           BM748564           |                               |
|                       |           BM757596           |                               |
|                       |           AA831221           |                               |
|                       |           BM758299           |                               |
|                       |           BM759009           |                               |
|                       |           BM748914           |                               |
|                       |           BM127592           |                               |
|                       |           BM748320           |                               |
|                       |           BM757578           |                               |
|                       |           BM758044           |                               |
|                       |           BM758364           |                               |
|                       |           BM748676           |                               |
|                       |           BM757694           |                               |
|                       |           BM757712           |                               |
|                       |           BM758300           |                               |
|                       |           BM743339           |                               |
|                       |           BM749242           |                               |
|                       |           BM758605           |                               |
|                       |           BM758754           |                               |
|                       |           BM758700           |                               |
|                       |           BM750567           |                               |
|                       |           BM749795           |                               |
|                       |           CN371233           |                               |
|                       | *Seq. mapped in strand Minus*| *Seq. mapped in strand Minus* |
|                       |           AV707205           |          AK032370             |
|                       |                              |          AK011837             |
|                       |                              |          BC034728             |
|                       |                              |          AK003340             |
+-----------------------+------------------------------+-------------------------------+
|      #   42           | *Seq. mapped in strand Plus* | *Seq. mapped in strand Plus*  |
|                       |             U33635           |          AK043765             |
|                       |           BC071557           |                               |
|                       |           BC002377           |                               |
|                       |           BC014626           |                               |
|                       |           AF531868           |                               |
|                       |           AF531869           |                               |
|                       |           AF531870           |                               |
|                       |           AF531871           |                               |
|                       |           AF531872           |                               |
|                       |             U40271           |                               |
|                       |           BC046109           |                               |
|                       |           AL157486           |                               |
|                       |           AK131487           |                               |
|                       |           AK055648           |                               |
|                       |           CR625968           |                               |
|                       |           CR611115           |                               |
|                       | *Seq. mapped in strand Minus*| *Seq. mapped in strand Minus* |
|                       |           AK124108           |          AK087891             |
+-----------------------+------------------------------+-------------------------------+
|      #   43           | *Seq. mapped in strand Plus* | *Seq. mapped in strand Plus*  |
|                       |           BM984107           |          BC014741             |
|                       |           BU726446           |          BI133127             |
|                       |                              |          BF463956             |
|                       |                              |          CD773832             |
|                       |                              |          BX528986             |
|                       |                              |          BE944487             |
|                       |                              |          BF455800             |
|                       |                              |          BE981044             |
|                       |                              |          BX524515             |
|                       | *Seq. mapped in strand Minus*| *Seq. mapped in strand Minus* |
|                       |           BU632531           |          AW489938             |
|                       |           AV748340           |                               |
|                       |           BM969787           |                               |
|                       |           CA429910           |                               |
|                       |           AF084479           |                               |
|                       |           BQ026168           |                               |
|                       |           AA485132           |                               |
|                       |           AI125656           |                               |
|                       |           BQ022241           |                               |
|                       |           BQ003593           |                               |
|                       |           CR743450           |                               |
|                       |           CA447578           |                               |
|                       |           BM995745           |                               |
|                       |           AI028370           |                               |
|                       |           AA831200           |                               |
|                       |           AI911252           |                               |
|                       |           CB306515           |                               |
|                       |           CD367605           |                               |
|                       |           BU677853           |                               |
|                       |           CA417988           |                               |
|                       |           CA426460           |                               |
|                       |           BU615447           |                               |
|                       |           CA416625           |                               |
|                       |             R40695           |                               |
|                       |           AI624308           |                               |
|                       |           CB159356           |                               |
|                       |           BQ009000           |                               |
|                       |           CA307761           |                               |
|                       |           BC041561           |                               |
|                       |           BC080544           |                               |
|                       |           AW170431           |                               |
|                       |           AI080642           |                               |
|                       |           AF072810           |                               |
|                       |           AI656420           |                               |
|                       |           BI963102           |                               |
|                       |           AI354614           |                               |
|                       |           AA130833           |                               |
|                       |           BM673311           |                               |
|                       |           AI401792           |                               |
|                       |           BU626031           |                               |
|                       |           CA309249           |                               |
|                       |           BM978842           |                               |
|                       |           BQ044992           |                               |
|                       |           BU684983           |                               |
|                       |           BU624535           |                               |
|                       |           AI310357           |                               |
|                       |           AI760308           |                               |
|                       |           AK123274           |                               |
|                       |           AI201315           |                               |
|                       |           BC050599           |                               |
|                       |           BQ004311           |                               |
|                       |           CR623422           |                               |
|                       |           BQ006217           |                               |
|                       |           BQ688396           |                               |
|                       |           BQ687061           |                               |
|                       |           CK300878           |                               |
|                       |           BU675309           |                               |
|                       |           CK300378           |                               |
|                       |           BE394141           |                               |
|                       |           BE933066           |                               |
|                       |           CN302251           |                               |
|                       |           AV655874           |                               |
|                       |           AA013017           |                               |
|                       |           BM796615           |                               |
|                       |           CN420049           |                               |
|                       |           BF912735           |                               |
|                       |           BF913464           |                               |
|                       |           BF913468           |                               |
|                       |           BF914733           |                               |
|                       |           BG721617           |                               |
|                       |           BE000378           |                               |
|                       |           AL118602           |                               |
|                       |           AW835499           |                               |
|                       |           BG000711           |                               |
|                       |           BG940134           |                               |
|                       |           AW853012           |                               |
|                       |           BX483632           |                               |
|                       |           BG940133           |                               |
|                       |           AA749222           |                               |
|                       |           AA720577           |                               |
|                       |           CB243591           |                               |
|                       |           AA713677           |                               |
|                       |           BC065029           |                               |
|                       |           BQ014882           |                               |
|                       |           BG281920           |                               |
|                       |           BG777021           |                               |
|                       |           BE078201           |                               |
|                       |           BE276175           |                               |
|                       |           BE787329           |                               |
|                       |           BE258324           |                               |
|                       |           CD357506           |                               |
|                       |           BF989141           |                               |
|                       |           BF989120           |                               |
|                       |           AA577412           |                               |
|                       |           BG827005           |                               |
|                       |           BE812140           |                               |
|                       |           BE086830           |                               |
|                       |           BE086831           |                               |
|                       |           BE086837           |                               |
|                       |           BE086816           |                               |
|                       |           BE086822           |                               |
|                       |           BQ303584           |                               |
|                       |           BE086832           |                               |
|                       |           BG424355           |                               |
|                       |           BQ303585           |                               |
|                       |           BE086776           |                               |
|                       |           BI053660           |                               |
|                       |           AA259089           |                               |
|                       |           AA255960           |                               |
|                       |           AI498634           |                               |
|                       |           AW968064           |                               |
|                       |           CN302252           |                               |
|                       |           BE086833           |                               |
|                       |           BU854726           |                               |
|                       |           BE702433           |                               |
|                       |           BU855156           |                               |
|                       |           BE836106           |                               |
|                       |           AU280242           |                               |
|                       |           AL704593           |                               |
|                       |           AW117583           |                               |
|                       |           AA744237           |                               |
|                       |           BF221513           |                               |
|                       |           AW183887           |                               |
|                       |           AI808617           |                               |
|                       |           AI554653           |                               |
|                       |           AI421573           |                               |
|                       |           BF059006           |                               |
|                       |           AW976871           |                               |
|                       |           BF514241           |                               |
|                       |           CB990739           |                               |
+-----------------------+------------------------------+-------------------------------+
|      #   44           | *Seq. mapped in strand Plus* | *Seq. mapped in strand Plus*  |
|                       |           BC000954           |          AK035626             |
|                       |           BX648807           |                               |
|                       |           CR596733           |                               |
|                       |           BX647444           |                               |
|                       |           CR623845           |                               |
|                       |           AF136630           |                               |
|                       |           CR617945           |                               |
|                       |           AF086270           |                               |
|                       |             U26312           |                               |
|                       |           AB030905           |                               |
|                       |           BC014380           |                               |
|                       | *Seq. mapped in strand Minus*| *Seq. mapped in strand Minus* |
|                       |           BX538099           |          AK078965             |
|                       |           AK026373           |          AK089012             |
|                       |           BX537494           |          AK049735             |
|                       |             M29065           |          BC059107             |
|                       |           BX641084           |          AF073993             |
|                       |           BC000506           |          AF406651             |
|                       |             M29064           |          AK049771             |
|                       |           AK129839           |                               |
|                       |           CR613897           |                               |
+-----------------------+------------------------------+-------------------------------+
|      #   45           | *Seq. mapped in strand Plus* | *Seq. mapped in strand Plus*  |
|                       |           BU743198           |          AK054477             |
|                       |                              |          AK081639             |
|                       | *Seq. mapped in strand Minus*| *Seq. mapped in strand Minus* |
|                       |           BX640794           |          AK031631             |
|                       |           AI274286           |          BC024871             |
|                       |             N90014           |          BC058191             |
|                       |           AI520754           |          AK005194             |
|                       |           AA564981           |          AK004864             |
|                       |           AI920804           |          AK053644             |
|                       |           BX647047           |          BC016432             |
|                       |           CB243447           |          BC019174             |
|                       |           AF100785           |          AK089027             |
|                       |           AK001553           |          AK008681             |
|                       |           BM782921           |          AB020203             |
|                       |           AA649290           |                               |
|                       |           AA838729           |                               |
|                       |           AI052134           |                               |
|                       |           CR745636           |                               |
|                       |           BU632276           |                               |
|                       |           BX111262           |                               |
|                       |           BQ003092           |                               |
|                       |           BU742092           |                               |
|                       |           BI966867           |                               |
|                       |           CK004524           |                               |
|                       |           AI539628           |                               |
|                       |           AA854457           |                               |
|                       |           AA435925           |                               |
|                       |           AA651840           |                               |
|                       |           AI439836           |                               |
|                       |           AA676504           |                               |
|                       |           AA860500           |                               |
|                       |           AI567507           |                               |
|                       |           BM666044           |                               |
|                       |           AA890333           |                               |
|                       |           BF508198           |                               |
|                       |           AA890563           |                               |
|                       |           BU687193           |                               |
|                       |           BU678837           |                               |
|                       |           CA311046           |                               |
|                       |           CB243541           |                               |
|                       |             W72009           |                               |
|                       |           AK027534           |                               |
|                       |           BM506713           |                               |
|                       |           BM272607           |                               |
|                       |           BQ270605           |                               |
|                       |           BM978906           |                               |
|                       |           BQ023459           |                               |
|                       |           AW069288           |                               |
|                       |           CA944515           |                               |
|                       |           BM978671           |                               |
|                       |           BM680392           |                               |
|                       |           AA810662           |                               |
|                       |           AI694970           |                               |
|                       |           AI653447           |                               |
|                       |           AW662551           |                               |
|                       |           AI139528           |                               |
|                       |           AA810651           |                               |
|                       |           AK124824           |                               |
|                       |           AK098205           |                               |
|                       |           BC013771           |                               |
|                       |           AK001951           |                               |
|                       |           AF183419           |                               |
|                       |           BQ024760           |                               |
|                       |           AA731459           |                               |
|                       |           BX342986           |                               |
|                       |           BX461238           |                               |
|                       |           BG545982           |                               |
|                       |           BF184452           |                               |
|                       |           AF086913           |                               |
|                       |           CN357864           |                               |
|                       |           CD643791           |                               |
|                       |           BP259543           |                               |
|                       |           CB265871           |                               |
|                       |           CD654125           |                               |
|                       |           BM798749           |                               |
|                       |           BP342636           |                               |
|                       |           BG619950           |                               |
|                       |           CD677271           |                               |
|                       |           AL138385           |                               |
|                       |           BX335776           |                               |
|                       |           BX283105           |                               |
|                       |           BM923355           |                               |
|                       |           BG687423           |                               |
|                       |           CN357862           |                               |
|                       |           BQ435142           |                               |
|                       |           BQ650443           |                               |
|                       |           AA007279           |                               |
|                       |           BX458669           |                               |
|                       |             F01095           |                               |
|                       |           BX387711           |                               |
|                       |           AV704895           |                               |
|                       |           BP289452           |                               |
|                       |           BP258223           |                               |
|                       |           BQ050240           |                               |
|                       |           AA194688           |                               |
|                       |             H65871           |                               |
|                       |           AA476639           |                               |
|                       |           CK141547           |                               |
|                       |           BI550730           |                               |
|                       |           AU130170           |                               |
|                       |           BQ367213           |                               |
|                       |           BG705248           |                               |
|                       |           AB021870           |                               |
|                       |           AA165658           |                               |
|                       |           BI553912           |                               |
|                       |           BM825799           |                               |
|                       |             R70844           |                               |
|                       |           BG106150           |                               |
|                       |           BF171850           |                               |
|                       |           BQ694111           |                               |
|                       |           AU126983           |                               |
|                       |           CN357870           |                               |
|                       |           CV324279           |                               |
|                       |           BP344553           |                               |
|                       |           BE700251           |                               |
|                       |           BQ338087           |                               |
|                       |           BQ337765           |                               |
|                       |           BG698420           |                               |
|                       |           CK825361           |                               |
|                       |           BI858992           |                               |
|                       |           AV695155           |                               |
|                       |           BI257720           |                               |
|                       |           BI561819           |                               |
|                       |           AU136907           |                               |
|                       |           BQ270849           |                               |
|                       |           BP233557           |                               |
|                       |           CN357860           |                               |
|                       |           AV698046           |                               |
|                       |           CV023918           |                               |
|                       |           BI601245           |                               |
|                       |           BI601992           |                               |
|                       |           BG682861           |                               |
|                       |           BP328169           |                               |
|                       |           CB990837           |                               |
|                       |           BF514669           |                               |
|                       |           BF513711           |                               |
|                       |           BP289946           |                               |
|                       |           CN357867           |                               |
|                       |           BM478239           |                               |
|                       |           BP273342           |                               |
|                       |           BM837013           |                               |
|                       |           CD627749           |                               |
|                       |           CD627748           |                               |
|                       |           BX279600           |                               |
|                       |           CB992077           |                               |
|                       |           BP222048           |                               |
|                       |           BP227524           |                               |
|                       |           BP367755           |                               |
|                       |           CD627742           |                               |
|                       |           CD627743           |                               |
|                       |           CD627746           |                               |
|                       |           CD627747           |                               |
|                       |           CD627745           |                               |
|                       |           BP276364           |                               |
|                       |           BP291231           |                               |
|                       |           CD627744           |                               |
|                       |           BM272857           |                               |
|                       |           BP212176           |                               |
|                       |           BP237621           |                               |
|                       |           BP257258           |                               |
|                       |           AA312979           |                               |
|                       |           BP240725           |                               |
|                       |           BP326805           |                               |
|                       |           CD627740           |                               |
|                       |           CD627741           |                               |
|                       |           CB150710           |                               |
|                       |           BP226914           |                               |
|                       |           CD706155           |                               |
|                       |           BM314033           |                               |
|                       |           CN357869           |                               |
|                       |           CB155221           |                               |
|                       |             F01168           |                               |
|                       |           CA393732           |                               |
|                       |           AW600241           |                               |
|                       |           BF814140           |                               |
|                       |           CN357868           |                               |
|                       |           CF242986           |                               |
|                       |           AV691157           |                               |
|                       |           BI597405           |                               |
|                       |           BP361137           |                               |
|                       |           CN413029           |                               |
+-----------------------+------------------------------+-------------------------------+
|      #   46           | *Seq. mapped in strand Plus* | *Seq. mapped in strand Plus*  |
|                       |           AK027764           |          AK122371             |
|                       |                              |          CN535294             |
|                       |                              |          BU705515             |
|                       |                              |          CF532976             |
|                       |                              |          BY129113             |
|                       |                              |          BI664252             |
|                       |                              |          CD349550             |
|                       |                              |          BB660017             |
|                       |                              |          CB246243             |
|                       |                              |          CO045779             |
|                       |                              |          BU055007             |
|                       |                              |          BI734814             |
|                       |                              |          CD348908             |
|                       |                              |          BC024692             |
|                       |                              |          BE914240             |
|                       |                              |          CN534578             |
|                       |                              |          BI662777             |
|                       |                              |          BG174347             |
|                       |                              |          BB609998             |
|                       |                              |          BU703951             |
|                       |                              |          CF172431             |
|                       |                              |          CF898688             |
|                       |                              |          BQ769803             |
|                       |                              |          AV119630             |
|                       |                              |          BY108660             |
|                       |                              |          BY107621             |
|                       |                              |          AA671002             |
|                       |                              |          AA215226             |
|                       |                              |          BG245618             |
|                       |                              |          AA880010             |
|                       |                              |          BU609433             |
|                       |                              |          BE534551             |
|                       |                              |          CD350629             |
|                       |                              |          BI134766             |
|                       |                              |          BB862156             |
|                       |                              |            U55210             |
|                       |                              |          AI158792             |
|                       |                              |          BG917307             |
|                       |                              |          CF981639             |
|                       |                              |          BY315754             |
|                       |                              |          BG914669             |
|                       |                              |          BY333451             |
|                       |                              |          BY335307             |
|                       |                              |          AA168830             |
|                       |                              |          CN528442             |
|                       |                              |          BY310348             |
|                       |                              |          BI695086             |
|                       |                              |          BI646569             |
|                       |                              |          BU610977             |
|                       |                              |          AA546447             |
|                       |                              |          BI851216             |
|                       |                              |          CF164865             |
|                       |                              |          CA885827             |
|                       |                              |          CF167616             |
|                       |                              |          AA178806             |
|                       |                              |          AI603939             |
|                       |                              |          AI661990             |
|                       |                              |          CN689757             |
|                       |                              |          BF536234             |
|                       |                              |          CF724093             |
|                       |                              |          BM935316             |
|                       |                              |          CA945727             |
|                       |                              |          BB621399             |
|                       |                              |          BE334504             |
|                       |                              |          BG793789             |
|                       |                              |          BB859149             |
|                       |                              |          CF172822             |
|                       |                              |          CF895093             |
|                       |                              |          CA559605             |
|                       |                              |          CA556217             |
|                       |                              |          CA556227             |
|                       |                              |          CK387917             |
|                       |                              |          CK387351             |
|                       |                              |          CA555971             |
|                       |                              |          CA555995             |
|                       |                              |          CA557047             |
|                       |                              |          CA563820             |
|                       |                              |          CK335803             |
|                       |                              |          CA887917             |
|                       |                              |          CA886780             |
|                       |                              |          CN692267             |
|                       |                              |          CA562621             |
|                       |                              |          CA561937             |
|                       |                              |          CA873056             |
|                       |                              |          CF174849             |
|                       |                              |          CF166207             |
|                       |                              |          CA557421             |
|                       |                              |          CA564900             |
|                       |                              |          CF167173             |
|                       |                              |          CA562899             |
|                       |                              |          CA563677             |
|                       |                              |          CA563731             |
|                       |                              |          CA885411             |
|                       |                              |          CK335980             |
|                       |                              |          CN693275             |
|                       |                              |          CA888958             |
|                       |                              |          CA557398             |
|                       |                              |          CA558623             |
|                       |                              |          CD551588             |
|                       |                              |          CA559672             |
|                       |                              |          CF894037             |
|                       |                              |          CA560746             |
|                       |                              |          CF173665             |
|                       |                              |          CK387170             |
|                       |                              |          CK388573             |
|                       |                              |          CA560760             |
|                       |                              |          CA890838             |
|                       |                              |          CN723287             |
|                       |                              |          CA559888             |
|                       |                              |          CA891370             |
|                       |                              |          CA559749             |
|                       |                              |          CN691198             |
|                       |                              |          CN700930             |
|                       |                              |          CN716025             |
|                       |                              |          CN722539             |
|                       |                              |          CA883232             |
|                       |                              |          CN693523             |
|                       |                              |          CA883295             |
|                       |                              |          CF173162             |
|                       |                              |          CF914394             |
|                       |                              |          CF904473             |
|                       |                              |          CF174816             |
|                       |                              |          CF914536             |
|                       |                              |          CF915530             |
|                       |                              |          CF916185             |
|                       |                              |          CF172945             |
|                       |                              |          CF914890             |
|                       |                              |          CF174321             |
|                       |                              |          CF905541             |
|                       |                              |          CF914886             |
|                       |                              |          CF913591             |
|                       |                              |          CF913580             |
|                       |                              |          CN721272             |
|                       |                              |          CF172272             |
|                       |                              |          CF172961             |
|                       |                              |          CF914856             |
|                       |                              |          CF174614             |
|                       |                              |          CF895259             |
|                       |                              |          CN674671             |
|                       |                              |          CF173230             |
|                       |                              |          CA557857             |
|                       |                              |          AW537606             |
|                       |                              |          BG078731             |
|                       |                              |          CD564881             |
|                       |                              |          BB398586             |
|                       |                              |          BM225265             |
|                       |                              |          BM225257             |
|                       |                              |          BM226826             |
|                       |                              |          BM232016             |
|                       |                              |          BM232092             |
|                       |                              |          BM226357             |
|                       |                              |          BM230961             |
|                       |                              |          BM233042             |
|                       |                              |          BM225045             |
|                       |                              |          BM225064             |
|                       |                              |          BM226377             |
|                       |                              |          BM227678             |
|                       |                              |          BM231206             |
|                       |                              |          AW763349             |
|                       |                              |          BM228472             |
|                       |                              |          BM228621             |
|                       |                              |          CF748818             |
|                       |                              |          BM229306             |
|                       |                              |          BM226047             |
|                       |                              |          BM228420             |
|                       |                              |          BM118893             |
|                       |                              |          BM229322             |
|                       |                              |          BM230130             |
|                       |                              |          CA887916             |
|                       |                              |          CK346788             |
|                       |                              |          BM230356             |
|                       |                              |          BB705481             |
|                       |                              |          BM119231             |
|                       |                              |          BB704816             |
|                       |                              |          CA883231             |
|                       |                              |          BB698617             |
|                       |                              |          BM118515             |
|                       |                              |          CA890837             |
|                       |                              |          BM231961             |
|                       |                              |          BB706081             |
|                       |                              |          BM118589             |
|                       |                              |          CN701469             |
|                       |                              |          BG065453             |
|                       |                              |          AA681980             |
|                       |                              |          AI050620             |
|                       |                              |            W41330             |
|                       |                              |          AU040513             |
|                       |                              |          BY315672             |
|                       |                              |          BY753639             |
|                       |                              |          CA885410             |
|                       |                              |          AW487999             |
|                       |                              |          BC037457             |
|                       |                              |          BQ032054             |
|                       | *Seq. mapped in strand Minus*| *Seq. mapped in strand Minus* |
|                       |           AK074717           |          AW046236             |
|                       |           AK001501           |                               |
|                       |           AF247457           |                               |
|                       |           AF234532           |                               |
|                       |           AB018342           |                               |
|                       |           AL832428           |                               |
|                       |           AF132022           |                               |
|                       |           AK125246           |                               |
|                       |           AK024719           |                               |
|                       |           BC014956           |                               |
|                       |           AF184153           |                               |
|                       |           BC071965           |                               |
|                       |           BC032669           |                               |
|                       |           BC050682           |                               |
|                       |           BC041694           |                               |
+-----------------------+------------------------------+-------------------------------+
|      #   47           | *Seq. mapped in strand Plus* | *Seq. mapped in strand Plus*  |
|                       |           AF010233           |          CD773955             |
|                       |           AF511533           |          BY702861             |
|                       |           AF512951           |          BQ175414             |
|                       |           AK057127           |          CA318635             |
|                       |                              |          CF738249             |
|                       |                              |          CA450867             |
|                       |                              |          BQ176543             |
|                       |                              |          AI846991             |
|                       |                              |          BF227837             |
|                       |                              |          BE983219             |
|                       |                              |          BE137015             |
|                       |                              |          BI790672             |
|                       | *Seq. mapped in strand Minus*| *Seq. mapped in strand Minus* |
|                       |           AF116603           |          BF020318             |
|                       |                              |          BE200180             |
|                       |                              |          AY263368             |
|                       |                              |          BX516492             |
|                       |                              |          BE137141             |
|                       |                              |          BE690088             |
|                       |                              |          CK346211             |
|                       |                              |          BB633809             |
|                       |                              |          BX517674             |
|                       |                              |          AI956320             |
|                       |                              |          AI851789             |
|                       |                              |          AI842370             |
+-----------------------+------------------------------+-------------------------------+
|      #   48           | *Seq. mapped in strand Plus* | *Seq. mapped in strand Plus*  |
|                       |           AV685990           |          BY282322             |
|                       |                              |          BY329249             |
|                       |                              |          BY333152             |
|                       |                              |          BY277213             |
|                       |                              |          BY273311             |
|                       |                              |          BU918196             |
|                       |                              |          BB609885             |
|                       |                              |          CD551144             |
|                       |                              |          BB844142             |
|                       |                              |          AK039280             |
|                       |                              |          BY016427             |
|                       |                              |          BB633164             |
|                       |                              |          AK042070             |
|                       |                              |          BY298920             |
|                       |                              |          BB637474             |
|                       |                              |          BY230817             |
|                       |                              |          BY011317             |
|                       |                              |          BB614599             |
|                       |                              |          BB629003             |
|                       |                              |          BB616766             |
|                       |                              |          BY747239             |
|                       |                              |          BB619799             |
|                       |                              |          AK029533             |
|                       |                              |          AK030843             |
|                       |                              |          AK036210             |
|                       |                              |          AK088922             |
|                       |                              |          AK018217             |
|                       |                              |          BB638859             |
|                       |                              |          CA890108             |
|                       |                              |          CA890118             |
|                       |                              |          CA886398             |
|                       |                              |          BI155289             |
|                       |                              |          BI731649             |
|                       |                              |          CF727259             |
|                       |                              |          AI592851             |
|                       |                              |          CD349729             |
|                       |                              |          CB526580             |
|                       |                              |          BC056390             |
|                       |                              |          BC059017             |
|                       |                              |          CN704703             |
|                       |                              |          BY129889             |
|                       |                              |          BY278865             |
|                       |                              |          CD563085             |
|                       |                              |          BY250221             |
|                       |                              |          CN677920             |
|                       |                              |          BY336000             |
|                       |                              |          CD551618             |
|                       |                              |          BE380220             |
|                       |                              |          CD552220             |
|                       |                              |          BX512729             |
|                       |                              |          AW320421             |
|                       |                              |          AK030862             |
|                       |                              |          CA564186             |
|                       |                              |          BU709056             |
|                       |                              |          CA891299             |
|                       |                              |          CA893054             |
|                       |                              |          BM942691             |
|                       |                              |          CK336045             |
|                       |                              |          CF164250             |
|                       |                              |          BU581864             |
|                       |                              |          BU055574             |
|                       |                              |          BQ265525             |
|                       |                              |          CN725258             |
|                       |                              |          BI081717             |
|                       |                              |          BQ715889             |
|                       |                              |          AI060353             |
|                       |                              |          CB574640             |
|                       |                              |          BE628882             |
|                       | *Seq. mapped in strand Minus*| *Seq. mapped in strand Minus* |
|                       |           BC022510           |          BY742683             |
|                       |           AI973246           |                               |
|                       |           AI679928           |                               |
|                       |           AA428579           |                               |
|                       |           AF230397           |                               |
|                       |           AF230398           |                               |
|                       |           AF230399           |                               |
|                       |             L04510           |                               |
|                       |             H19413           |                               |
|                       |           AW304804           |                               |
|                       |           AA689423           |                               |
|                       |           AA489014           |                               |
|                       |           BM677710           |                               |
|                       |             R60064           |                               |
|                       |             T65376           |                               |
|                       |           AU137144           |                               |
|                       |           BM452300           |                               |
|                       |           BE701432           |                               |
|                       |           AL121038           |                               |
|                       |             H78121           |                               |
|                       |             T08981           |                               |
|                       |           BM547031           |                               |
|                       |           BE813356           |                               |
|                       |           BX369783           |                               |
|                       |             T65453           |                               |
|                       |             F11746           |                               |
|                       |           CN409230           |                               |
|                       |           AA429534           |                               |
|                       |           AL527955           |                               |
|                       |           AW993847           |                               |
|                       |           BE813305           |                               |
|                       |           AV758494           |                               |
|                       |           CN409228           |                               |
|                       |           CN409231           |                               |
|                       |           BI463842           |                               |
|                       |           BU662797           |                               |
|                       |           BQ369121           |                               |
|                       |           CV022958           |                               |
|                       |           BG699131           |                               |
|                       |           BI462708           |                               |
|                       |           CD691700           |                               |
|                       |           AL133900           |                               |
|                       |           BI548719           |                               |
|                       |           BI859501           |                               |
+-----------------------+------------------------------+-------------------------------+
|      #   49           | *Seq. mapped in strand Plus* | *Seq. mapped in strand Plus*  |
|                       |           BP356683           |          BC020320             |
|                       |           BP315154           |                               |
|                       |           AU140918           |                               |
|                       |           BC069633           |                               |
|                       |           BP313140           |                               |
|                       |           BX950928           |                               |
|                       |           BI057280           |                               |
|                       |           BI057282           |                               |
|                       |           BX505717           |                               |
|                       |           AI872702           |                               |
|                       |           BF370440           |                               |
|                       |           AW473832           |                               |
|                       |           AI918478           |                               |
|                       |           AU137827           |                               |
|                       |           AK002034           |                               |
|                       | *Seq. mapped in strand Minus*| *Seq. mapped in strand Minus* |
|                       |           BQ012533           |          BY365279             |
|                       |           AI769212           |          BB728889             |
|                       |           BM979613           |          BY388278             |
|                       |           BX105796           |                               |
+-----------------------+------------------------------+-------------------------------+
|      #   50           | *Seq. mapped in strand Plus* | *Seq. mapped in strand Plus*  |
|                       |           BC039406           |          BQ031775             |
|                       | *Seq. mapped in strand Minus*| *Seq. mapped in strand Minus* |
|                       |             U43195           |          AK077297             |
+-----------------------+------------------------------+-------------------------------+
|      #   51           | *Seq. mapped in strand Plus* | *Seq. mapped in strand Plus*  |
|                       |           AY604867           |          BY020171             |
|                       |                              |          BY290792             |
|                       |                              |          BY198377             |
|                       |                              |          BY103168             |
|                       |                              |          BY280315             |
|                       |                              |          BY086991             |
|                       |                              |          BY102386             |
|                       |                              |          BB634466             |
|                       |                              |          BB654175             |
|                       |                              |          CN665274             |
|                       |                              |          BB651760             |
|                       |                              |          AK049193             |
|                       |                              |          AK047334             |
|                       |                              |          BB646892             |
|                       |                              |          BY353896             |
|                       |                              |          CF426507             |
|                       |                              |          BC068119             |
|                       |                              |          CB520271             |
|                       |                              |          BF580311             |
|                       |                              |          CN667792             |
|                       |                              |          CB244096             |
|                       |                              |          CN459838             |
|                       |                              |          BY761630             |
|                       |                              |          CB056684             |
|                       |                              |          CD774833             |
|                       |                              |          BB435452             |
|                       |                              |            W40746             |
|                       |                              |          AI391178             |
|                       |                              |          BB399017             |
|                       |                              |          BG968708             |
|                       |                              |          BY460695             |
|                       |                              |          AI060548             |
|                       |                              |          BY452767             |
|                       |                              |          AI413242             |
|                       |                              |          BB825874             |
|                       |                              |          BY434825             |
|                       |                              |          BB809846             |
|                       |                              |          BX640157             |
|                       |                              |          BB606588             |
|                       |                              |          AK053525             |
|                       |                              |          BE287136             |
|                       |                              |          BY439468             |
|                       |                              |          AI606068             |
|                       |                              |          BB446415             |
|                       |                              |          BY416464             |
|                       |                              |          BB399673             |
|                       |                              |          BU605022             |
|                       | *Seq. mapped in strand Minus*| *Seq. mapped in strand Minus* |
|                       |           AK056200           |          AI391294             |
|                       |           AY146652           |            W16406             |
|                       |           BC015368           |                               |
|                       |           CR596230           |                               |
|                       |           BC043437           |                               |
|                       |           CR620785           |                               |
+-----------------------+------------------------------+-------------------------------+
|      #   52           | *Seq. mapped in strand Plus* | *Seq. mapped in strand Plus*  |
|                       |           CR621421           |          BY255030             |
|                       |           BC003694           |          BY250565             |
|                       |           CR608286           |          CA751661             |
|                       |           BC001643           |          BY283268             |
|                       |           BC001949           |          BF116571             |
|                       |           CR590463           |          BY341223             |
|                       |           CR621837           |          BF178624             |
|                       |             M80783           |          CK628432             |
|                       |           CR456819           |          BY227677             |
|                       |           CR615941           |          BG294344             |
|                       |                              |          BY270753             |
|                       |                              |          BY351671             |
|                       |                              |          BB868447             |
|                       |                              |          BY261523             |
|                       |                              |          BY311359             |
|                       |                              |          BY249746             |
|                       |                              |          BY241007             |
|                       |                              |          BY102858             |
|                       |                              |          BY103722             |
|                       |                              |          BY294524             |
|                       |                              |          BY782803             |
|                       |                              |          BY139491             |
|                       |                              |          AA919637             |
|                       |                              |          BB851856             |
|                       |                              |          BF538319             |
|                       |                              |          BY143491             |
|                       |                              |          BY301287             |
|                       |                              |          BY077218             |
|                       |                              |          BY323157             |
|                       |                              |          BY344395             |
|                       |                              |          BY293178             |
|                       |                              |          BY072630             |
|                       |                              |          BY313224             |
|                       |                              |          BY041453             |
|                       |                              |          BY256684             |
|                       |                              |          BY326240             |
|                       |                              |          BY336352             |
|                       |                              |          BY326405             |
|                       |                              |          BY131282             |
|                       |                              |          BY084177             |
|                       |                              |          BY322877             |
|                       |                              |          BY053034             |
|                       |                              |          BY046397             |
|                       |                              |          BY072458             |
|                       |                              |          BY087550             |
|                       |                              |          BY087742             |
|                       |                              |          BY240587             |
|                       |                              |          BY158648             |
|                       |                              |          BY152625             |
|                       |                              |          BB866998             |
|                       |                              |          BF300130             |
|                       |                              |          BY098651             |
|                       |                              |          BY326997             |
|                       |                              |          BY325396             |
|                       |                              |          BB610033             |
|                       |                              |          BB845444             |
|                       |                              |          BC012879             |
|                       |                              |          BF300652             |
|                       |                              |          BF532116             |
|                       |                              |          BQ890835             |
|                       |                              |          BY791606             |
|                       |                              |          BY771318             |
|                       |                              |          BY783269             |
|                       |                              |          BY281789             |
|                       |                              |          CA541822             |
|                       |                              |          BF178719             |
|                       |                              |          BG172849             |
|                       |                              |          BI558106             |
|                       |                              |          BC006833             |
|                       |                              |          CK789816             |
|                       |                              |          CA567338             |
|                       |                              |          BY342904             |
|                       |                              |          BY343494             |
|                       |                              |          BY335281             |
|                       |                              |          BY034940             |
|                       |                              |          BQ887801             |
|                       |                              |          CB565977             |
|                       |                              |          BI079539             |
|                       |                              |          BU925073             |
|                       |                              |          BG276726             |
|                       |                              |          BB856172             |
|                       |                              |          BI219000             |
|                       |                              |          BI221412             |
|                       |                              |          BG865005             |
|                       |                              |          CB954844             |
|                       |                              |          BE380445             |
|                       |                              |          AA273359             |
|                       |                              |          AA462739             |
|                       |                              |            W97950             |
|                       |                              |          BF022161             |
|                       |                              |          BQ571105             |
|                       |                              |          BY703962             |
|                       |                              |          AK003702             |
|                       |                              |          BQ961073             |
|                       |                              |          BG247747             |
|                       |                              |          BI466169             |
|                       |                              |          BQ931681             |
|                       |                              |          CB055942             |
|                       |                              |          AV027622             |
|                       |                              |          BU918094             |
|                       |                              |          BY384927             |
|                       |                              |            W36403             |
|                       |                              |          BI106392             |
|                       |                              |          AW988994             |
|                       |                              |            W85559             |
|                       |                              |            W62468             |
|                       |                              |          BF166098             |
|                       |                              |          AA415877             |
|                       |                              |          CF618271             |
|                       |                              |          BG243474             |
|                       |                              |          BB637899             |
|                       |                              |          CF895567             |
|                       |                              |          BI249728             |
|                       |                              |          BE309414             |
|                       |                              |          BF123062             |
|                       |                              |          BI465886             |
|                       |                              |          AA208051             |
|                       |                              |          BM946342             |
|                       |                              |            W18445             |
|                       |                              |          BE380428             |
|                       |                              |          AA122723             |
|                       |                              |          BX639388             |
|                       |                              |          BY688961             |
|                       |                              |          BY079326             |
|                       |                              |          BB759802             |
|                       |                              |          BE448613             |
|                       |                              |            W62166             |
|                       |                              |          AA231311             |
|                       |                              |          BF720351             |
|                       |                              |          CB321126             |
|                       |                              |            W10066             |
|                       |                              |          AW988401             |
|                       |                              |          BE627383             |
|                       |                              |          BY635286             |
|                       | *Seq. mapped in strand Minus*| *Seq. mapped in strand Minus* |
|                       |           CR613502           |          AI848033             |
|                       |           BC000655           |          BF462104             |
|                       |           AL110156           |          AA914299             |
|                       |           AF077203           |                               |
|                       |           CR598987           |                               |
|                       |           BC018864           |                               |
|                       |           BC009265           |                               |
|                       |           CR599702           |                               |
|                       |           CR607559           |                               |
|                       |           AF179891           |                               |
|                       |           CR597690           |                               |
|                       |           CR618590           |                               |
|                       |           CR597774           |                               |
+-----------------------+------------------------------+-------------------------------+
|      #   53           | *Seq. mapped in strand Plus* | *Seq. mapped in strand Plus*  |
|                       |           BX102205           |          AK030221             |
|                       |                              |          AK039754             |
|                       | *Seq. mapped in strand Minus*| *Seq. mapped in strand Minus* |
|                       |           AK127150           |          AK016451             |
|                       |           BI462300           |                               |
+-----------------------+------------------------------+-------------------------------+
|      #   54           | *Seq. mapped in strand Plus* | *Seq. mapped in strand Plus*  |
|                       |           AK057062           |          AK012404             |
|                       |           AK092321           |                               |
|                       |           AK026026           |                               |
|                       | *Seq. mapped in strand Minus*| *Seq. mapped in strand Minus* |
|                       |           BC036364           |          AK051076             |
|                       |           AK094795           |          AK045731             |
|                       |           BC009197           |          AK052356             |
|                       |           CR611157           |          AK077657             |
|                       |           CR600376           |          BC002031             |
|                       |           CR612150           |            U75506             |
|                       |           AF042083           |                               |
|                       |           AF370380           |                               |
|                       |           BC033634           |                               |
|                       |           AF087891           |                               |
|                       |           BC022072           |                               |
|                       |           AF250233           |                               |
|                       |           AY005151           |                               |
|                       |           CR456389           |                               |
|                       |           CR407603           |                               |
+-----------------------+------------------------------+-------------------------------+
|      #   55           | *Seq. mapped in strand Plus* | *Seq. mapped in strand Plus*  |
|                       |           BP327709           |          BB647297             |
|                       |           BG703101           |                               |
|                       |           BI116528           |                               |
|                       |           BG399094           |                               |
|                       |           BG702442           |                               |
|                       |           BG772187           |                               |
|                       |           BG545423           |                               |
|                       |           AK093560           |                               |
|                       |             L08488           |                               |
|                       |           BI547788           |                               |
|                       |           BG703835           |                               |
|                       |           AI278797           |                               |
|                       |           AI244189           |                               |
|                       |           AI634687           |                               |
|                       |           AI874356           |                               |
|                       |           BI758004           |                               |
|                       |           AW166431           |                               |
|                       |           BG149501           |                               |
|                       |           BE858116           |                               |
|                       |           BM789889           |                               |
|                       |           CF597171           |                               |
|                       |           AA731999           |                               |
|                       |           BF724171           |                               |
|                       |           BI913848           |                               |
|                       |           AA961497           |                               |
|                       |             R85219           |                               |
|                       |             H52141           |                               |
|                       |           AI864884           |                               |
|                       |           AA808737           |                               |
|                       |           AA280804           |                               |
|                       |           BF590222           |                               |
|                       |           BI754802           |                               |
|                       |           AL528108           |                               |
|                       |           CR600162           |                               |
|                       |           BG253592           |                               |
|                       |           BI669081           |                               |
|                       |             C04168           |                               |
|                       |           BQ109559           |                               |
|                       |           AA343360           |                               |
|                       |           BI821499           |                               |
|                       |           BC015496           |                               |
|                       |           BE839132           |                               |
|                       |           BI766008           |                               |
|                       |           BE617167           |                               |
|                       |           BU935243           |                               |
|                       |           AA315676           |                               |
|                       |           BP215320           |                               |
|                       |           CV023510           |                               |
|                       |           CR541707           |                               |
|                       |           CR456840           |                               |
|                       |           BI762814           |                               |
|                       |             T29356           |                               |
|                       |           CB159529           |                               |
|                       |           AA338777           |                               |
|                       |           AW950550           |                               |
|                       |           BG035107           |                               |
|                       |           BG742368           |                               |
|                       |           BF676669           |                               |
|                       |             H86224           |                               |
|                       |           AA013093           |                               |
|                       |           CK001646           |                               |
|                       |           BF575080           |                               |
|                       |           BQ311461           |                               |
|                       |           AA149555           |                               |
|                       |           AA052905           |                               |
|                       |             N48484           |                               |
|                       |             N46538           |                               |
|                       |             T97208           |                               |
|                       |           AW363727           |                               |
|                       |           AW364760           |                               |
|                       |           BM827910           |                               |
|                       |           AA280854           |                               |
|                       |           BP223671           |                               |
|                       |           BP223697           |                               |
|                       |           AL563062           |                               |
|                       |           AA115192           |                               |
|                       |           AA102821           |                               |
|                       |           BE617655           |                               |
|                       |           BX327085           |                               |
|                       |           CK430388           |                               |
|                       |           BM512560           |                               |
|                       | *Seq. mapped in strand Minus*| *Seq. mapped in strand Minus* |
|                       |           AI024373           |          BC025072             |
+-----------------------+------------------------------+-------------------------------+
|      #   56           | *Seq. mapped in strand Plus* | *Seq. mapped in strand Plus*  |
|                       |           CR591498           |          BE981018             |
|                       | *Seq. mapped in strand Minus*| *Seq. mapped in strand Minus* |
|                       |           AK023594           |          CO041454             |
+-----------------------+------------------------------+-------------------------------+
|      #   57           | *Seq. mapped in strand Plus* | *Seq. mapped in strand Plus*  |
|                       |           BF507796           |          BY083822             |
|                       |             Y08302           |          BY094715             |
|                       |           AU138205           |          BY067924             |
|                       |           CB996050           |          BY070928             |
|                       |           CB995169           |          BE854699             |
|                       |           BC060837           |          BY729888             |
|                       |           AW235800           |          BY106180             |
|                       |           AI473721           |          BY009481             |
|                       |           BE326643           |          BY033039             |
|                       |           AU117302           |          BY076669             |
|                       |           BF507922           |          BY012655             |
|                       |           AW024965           |          BY015097             |
|                       |           AI434619           |          BY075541             |
|                       |           AI640337           |          BY014164             |
|                       |           AI806537           |          BY094659             |
|                       |           AI219065           |          BY035901             |
|                       |           AW028488           |          BY774156             |
|                       |           AW271700           |          BY095159             |
|                       |           BF197039           |          BY095477             |
|                       |           AI133339           |          BY083380             |
|                       |           BX464823           |          BY739893             |
|                       |           BX439704           |          BY090211             |
|                       |           CR622905           |          BY092762             |
|                       |           BG006548           |          BU514280             |
|                       |           BM552680           |          CD553300             |
|                       |           BM910756           |          CA543907             |
|                       |           BM910652           |          BQ266507             |
|                       |           CN312620           |          AY316312             |
|                       |           BP277658           |          CK032774             |
|                       |             R51175           |          BY070073             |
|                       |             U87167           |          BY072707             |
|                       |                              |          BE573694             |
|                       |                              |          BE306688             |
|                       |                              |          BQ941390             |
|                       |                              |          AA014742             |
|                       |                              |          BB618835             |
|                       | *Seq. mapped in strand Minus*| *Seq. mapped in strand Minus* |
|                       |           AI434422           |          CF617629             |
+-----------------------+------------------------------+-------------------------------+
|      #   58           | *Seq. mapped in strand Plus* | *Seq. mapped in strand Plus*  |
|                       |           CA437760           |          BU923748             |
|                       |           AI283551           |                               |
|                       | *Seq. mapped in strand Minus*| *Seq. mapped in strand Minus* |
|                       |           BC036820           |          BI794320             |
|                       |           AK000926           |          CB933927             |
|                       |           AI698407           |          AI843298             |
|                       |           AA120944           |          BC055067             |
|                       |           AA195077           |          AK129431             |
|                       |           AI659948           |          BQ287499             |
|                       |           AA731724           |          BQ287681             |
|                       |           BC025977           |          AI853285             |
|                       |           BC022237           |          BF462885             |
|                       |           BC051868           |          AI839584             |
|                       |           AL832432           |          AI850426             |
|                       |           BU740780           |          CF734677             |
|                       |           AI926347           |          AI853532             |
|                       |           AK024657           |          BE989810             |
|                       |           AI699570           |          BC051108             |
|                       |           BQ027611           |          AK045398             |
|                       |           AI918003           |          BC023329             |
|                       |           BU859314           |          AI849856             |
|                       |           BU741156           |          BC039233             |
|                       |           BM682413           |          BC059193             |
|                       |           BX117113           |          BM948118             |
|                       |           CK300625           |          BF163419             |
|                       |           BQ437129           |          AA414093             |
|                       |           AI761115           |          BI731710             |
|                       |           AW675737           |          CN721768             |
|                       |           AA938177           |            W34557             |
|                       |           AW152157           |          CA553429             |
|                       |           BC004525           |          CF156720             |
|                       |           AK022940           |          CK339835             |
|                       |           AK025999           |          CK388549             |
|                       |           AB051521           |          CN683326             |
|                       |           BF206958           |          BY125350             |
|                       |           BI195368           |          BQ894971             |
|                       |           BE903831           |          BU610649             |
|                       |           BE260664           |          BM950220             |
|                       |           BI195735           |          CN695683             |
|                       |           BE312889           |          BM945083             |
|                       |           BE261205           |          BG869264             |
|                       |           BE313854           |          BI732172             |
|                       |           BE743205           |          CN672219             |
|                       |           BI197005           |          CF537020             |
|                       |           BF797573           |          BM963787             |
|                       |           BI195299           |          AA445870             |
|                       |           BE261633           |            U20780             |
|                       |           BE313739           |          BY356988             |
|                       |           BG329301           |          CF732859             |
|                       |           BF982377           |          CF732940             |
|                       |           AU133538           |          CF172672             |
|                       |           BP344685           |          BB838836             |
|                       |           BE903131           |          CF174032             |
|                       |           AA195176           |          BY122875             |
|                       |           BF751473           |          CB524400             |
|                       |           BQ949601           |          CF533138             |
|                       |           BG763111           |          CD579058             |
|                       |           BC007822           |          BM963402             |
|                       |           BE251320           |          CB249951             |
|                       |           BI196976           |          CF900979             |
|                       |           BQ278145           |          BU605464             |
|                       |           BG323519           |          CN694228             |
|                       |           BE272597           |          BU611757             |
|                       |           BE314084           |          CN695974             |
|                       |           BE313513           |          BI665897             |
|                       |           BI199910           |          CN677091             |
|                       |           BP218824           |          CF734164             |
|                       |           BF946697           |          CN673813             |
|                       |           BF946843           |          CF896675             |
|                       |           BP287496           |          AW741017             |
|                       |           BP244663           |          CF356549             |
|                       |           BF333196           |                               |
|                       |           BU167334           |                               |
|                       |           CN334477           |                               |
|                       |           BP254352           |                               |
|                       |           BQ217875           |                               |
|                       |           BU431783           |                               |
|                       |           BF946780           |                               |
|                       |           BG896481           |                               |
|                       |           CN334476           |                               |
|                       |           CN334474           |                               |
|                       |           AW863006           |                               |
|                       |           BX504863           |                               |
|                       |           BG826925           |                               |
|                       |           BU948231           |                               |
|                       |           BF528378           |                               |
|                       |           BM313772           |                               |
|                       |           BQ369299           |                               |
|                       |           AI198113           |                               |
|                       |           BU431356           |                               |
|                       |           BQ307828           |                               |
|                       |           BF377246           |                               |
|                       |             R58558           |                               |
|                       |           BU948499           |                               |
|                       |           CD580295           |                               |
|                       |           CK905180           |                               |
|                       |           BU430811           |                               |
|                       |           BF900824           |                               |
|                       |           BM314047           |                               |
|                       |           BF726224           |                               |
|                       |           CK000406           |                               |
|                       |           AA341325           |                               |
|                       |           CB960973           |                               |
+-----------------------+------------------------------+-------------------------------+
|      #   59           | *Seq. mapped in strand Plus* | *Seq. mapped in strand Plus*  |
|                       |           CR592336           |          AK027956             |
|                       |           BC080193           |                               |
|                       |             X03363           |                               |
|                       |             M11730           |                               |
|                       |           AF177761           |                               |
|                       |             S57296           |                               |
|                       |           CR591377           |                               |
|                       | *Seq. mapped in strand Minus*| *Seq. mapped in strand Minus* |
|                       |           BC063395           |          AK041314             |
|                       |           BC006006           |          BC021589             |
|                       |           CR613484           |          AK009922             |
|                       |           AY508814           |          AK007795             |
|                       |           AF490253           |          AK002959             |
+-----------------------+------------------------------+-------------------------------+
|      #   60           | *Seq. mapped in strand Plus* | *Seq. mapped in strand Plus*  |
|                       |             U75586           |          AK086903             |
|                       | *Seq. mapped in strand Minus*| *Seq. mapped in strand Minus* |
|                       |           BX648465           |          BC019633             |
+-----------------------+------------------------------+-------------------------------+
|      #   61           | *Seq. mapped in strand Plus* | *Seq. mapped in strand Plus*  |
|                       |           BU191740           |          BX515175             |
|                       |           BX363351           |          AI117064             |
|                       |           AU132423           |                               |
|                       |           AF054996           |                               |
|                       |           BQ689108           |                               |
|                       |           CD673192           |                               |
|                       |           AW405608           |                               |
|                       |           BM808781           |                               |
|                       |           CB142892           |                               |
|                       |           BE397486           |                               |
|                       |           BE562415           |                               |
|                       |           BE397405           |                               |
|                       |           BI227388           |                               |
|                       |           BU597553           |                               |
|                       |           BP325651           |                               |
|                       |           BE397547           |                               |
|                       |           BE397741           |                               |
|                       |           BG342008           |                               |
|                       |           BG912768           |                               |
|                       |           BG755681           |                               |
|                       |           BG754365           |                               |
|                       |           BG342185           |                               |
|                       |           BG340516           |                               |
|                       |           BG759316           |                               |
|                       |           BG753952           |                               |
|                       |           BG718039           |                               |
|                       |           CN344968           |                               |
|                       |           BG720143           |                               |
|                       |           BM747882           |                               |
|                       |           BM758126           |                               |
|                       |           BM758629           |                               |
|                       |           BM739604           |                               |
|                       |           BG390654           |                               |
|                       |           BM479134           |                               |
|                       |           CB994401           |                               |
|                       |           CB991102           |                               |
|                       |           BG674604           |                               |
|                       |           AL560484           |                               |
|                       |           BG035475           |                               |
|                       |           BF129163           |                               |
|                       |           CD673515           |                               |
|                       |           BE791672           |                               |
|                       |           BU595409           |                               |
|                       |           BX335021           |                               |
|                       |           CR593468           |                               |
|                       |           CN344964           |                               |
|                       |           BE278488           |                               |
|                       |           BI113766           |                               |
|                       |           BF309685           |                               |
|                       |           BF348003           |                               |
|                       |           BP373430           |                               |
|                       |           BG027095           |                               |
|                       |           BG757064           |                               |
|                       |           BG757680           |                               |
|                       |           BG392665           |                               |
|                       |           BI830473           |                               |
|                       |           BX406830           |                               |
|                       |           BU147396           |                               |
|                       |           BM834494           |                               |
|                       |           BP247421           |                               |
|                       |           BP247486           |                               |
|                       |           BM787472           |                               |
|                       |           CB267787           |                               |
|                       |           BE548923           |                               |
|                       |           CN344970           |                               |
|                       |           BG403641           |                               |
|                       |           BG422156           |                               |
|                       |           CA488164           |                               |
|                       |           AL701546           |                               |
|                       |           BQ651813           |                               |
|                       |           BG401371           |                               |
|                       |           CN344965           |                               |
|                       |           BF314766           |                               |
|                       |           BE241759           |                               |
|                       |           BE258636           |                               |
|                       |           BX479063           |                               |
|                       |           BE253791           |                               |
|                       |           BF984021           |                               |
|                       |           BM688862           |                               |
|                       |           BM835077           |                               |
|                       |           BG755174           |                               |
|                       |           BE251788           |                               |
|                       |           BF337951           |                               |
|                       |           BI857539           |                               |
|                       |           BE252819           |                               |
|                       |           BF525851           |                               |
|                       |           BI600709           |                               |
|                       |           BG708732           |                               |
|                       |           CN344966           |                               |
|                       |           BX483786           |                               |
|                       |           BQ219314           |                               |
|                       |           CN344969           |                               |
|                       |           BM809178           |                               |
|                       |           BM809304           |                               |
|                       |           BP348032           |                               |
|                       |           BX280090           |                               |
|                       |           AA132814           |                               |
|                       |           BM469249           |                               |
|                       |           BM927429           |                               |
|                       |           BC010042           |                               |
|                       |           BE257588           |                               |
|                       |           BX341446           |                               |
|                       |           BP311376           |                               |
|                       |           BP198934           |                               |
|                       |           BP208149           |                               |
|                       |           BG436654           |                               |
|                       |           BM714597           |                               |
|                       |           BG479929           |                               |
|                       |           BG438060           |                               |
|                       |           BG774267           |                               |
|                       |           BM804238           |                               |
|                       |           BI832858           |                               |
|                       |           BQ689899           |                               |
|                       |           BQ888500           |                               |
|                       |           BU145963           |                               |
|                       |           BI761694           |                               |
|                       |           BM853479           |                               |
|                       |           BQ440045           |                               |
|                       |           CB156743           |                               |
|                       |           BG818229           |                               |
|                       |           BG471111           |                               |
|                       |           BU150523           |                               |
|                       |           BG281614           |                               |
|                       |           BG767879           |                               |
|                       |           BU860161           |                               |
|                       |           BU846227           |                               |
|                       |           BG165747           |                               |
|                       |           BU854939           |                               |
|                       |           BQ052779           |                               |
|                       |           BG764996           |                               |
|                       |           BG424638           |                               |
|                       |           BG419908           |                               |
|                       |           BI115134           |                               |
|                       |           BM702393           |                               |
|                       |           BM048674           |                               |
|                       |           BG679217           |                               |
|                       |           BX444433           |                               |
|                       |           CR619192           |                               |
|                       |           BU541061           |                               |
|                       |           CN344962           |                               |
|                       |           BM826038           |                               |
|                       |           AA232604           |                               |
|                       |           BG031396           |                               |
|                       |           BF304787           |                               |
|                       |           BG419085           |                               |
|                       |           BU501998           |                               |
|                       |           AW407811           |                               |
|                       |           BE259883           |                               |
|                       |           BE296014           |                               |
|                       |           BQ889461           |                               |
|                       |           BQ957386           |                               |
|                       |           BQ706523           |                               |
|                       |           BE271537           |                               |
|                       |           BI194578           |                               |
|                       |           BE409847           |                               |
|                       |           BE275343           |                               |
|                       |           CF128524           |                               |
|                       |           BQ889555           |                               |
|                       |           AL525049           |                               |
|                       |           BG832008           |                               |
|                       |           BQ422049           |                               |
|                       |           BG479246           |                               |
|                       |           AU125480           |                               |
|                       |           BX335020           |                               |
|                       |           BE792269           |                               |
|                       |           BE258591           |                               |
|                       |           BE396959           |                               |
|                       |           BE265937           |                               |
|                       |           BE791828           |                               |
|                       |           BE254936           |                               |
|                       |           AI205177           |                               |
|                       |           BE271250           |                               |
|                       |           BF212671           |                               |
|                       |           BE271263           |                               |
|                       |           BG421498           |                               |
|                       |           BI825647           |                               |
|                       |           BX453752           |                               |
|                       |           CN344971           |                               |
|                       |           AA459148           |                               |
|                       |             W44466           |                               |
|                       |           BI546367           |                               |
|                       |           BI546366           |                               |
|                       |           AA282150           |                               |
|                       |           AA207136           |                               |
|                       |           BG823474           |                               |
|                       |           BM044766           |                               |
|                       |           BM913611           |                               |
|                       |             H61261           |                               |
|                       |           BI859783           |                               |
|                       |           BI046062           |                               |
|                       |           BE173810           |                               |
|                       |           BI045799           |                               |
|                       |           BI046348           |                               |
|                       |           AA338109           |                               |
|                       |           BQ707565           |                               |
|                       |           BQ708702           |                               |
|                       |           BG955063           |                               |
|                       |           AW374430           |                               |
|                       |           BG121034           |                               |
|                       |           BG468428           |                               |
|                       |           BG823852           |                               |
|                       |           BQ945890           |                               |
|                       |           AA307895           |                               |
|                       |           CN398887           |                               |
|                       |           BP378627           |                               |
|                       |           BM675361           |                               |
|                       |           BU629484           |                               |
|                       |           BU631175           |                               |
|                       |           BM975099           |                               |
|                       |           BE908885           |                               |
|                       |           BF529134           |                               |
|                       |           BG112236           |                               |
|                       |           CF264883           |                               |
|                       |           CF272534           |                               |
|                       |           BG392569           |                               |
|                       |           AW971441           |                               |
|                       |             W01368           |                               |
|                       |           AI345947           |                               |
|                       |           CD370186           |                               |
|                       |           AI149522           |                               |
|                       |           AW605059           |                               |
|                       |           AW580608           |                               |
|                       |           CF552770           |                               |
|                       |           BE819649           |                               |
|                       |           BE819651           |                               |
|                       |           BE819653           |                               |
|                       |           BE018754           |                               |
|                       |           BU683153           |                               |
|                       |           BM742331           |                               |
|                       |           BM742800           |                               |
|                       |           BM744454           |                               |
|                       |           BM746675           |                               |
|                       |           BU597707           |                               |
|                       |           AW834735           |                               |
|                       |           BQ021802           |                               |
|                       |           BX385862           |                               |
|                       |           BX396042           |                               |
|                       |           CR621399           |                               |
|                       |           BU729172           |                               |
|                       |           BG677213           |                               |
|                       |           BE833048           |                               |
|                       |           BP213447           |                               |
|                       |           AW575367           |                               |
|                       |           AW362883           |                               |
|                       |           AA777461           |                               |
|                       |           AW362879           |                               |
|                       |           AI566310           |                               |
|                       |           AI284212           |                               |
|                       |           BG120719           |                               |
|                       |           AA458932           |                               |
|                       |           BG747960           |                               |
|                       |           BG254937           |                               |
|                       |           BU941409           |                               |
|                       |           AI708753           |                               |
|                       |           BU161645           |                               |
|                       |           BM802623           |                               |
|                       |           AA995863           |                               |
|                       |           BU727329           |                               |
|                       |           AW630966           |                               |
|                       |           AI523406           |                               |
|                       |           AW468726           |                               |
|                       |           AA149891           |                               |
|                       |             H61265           |                               |
|                       |             W44427           |                               |
|                       |             N78623           |                               |
|                       |           AA857236           |                               |
|                       |           BM794988           |                               |
|                       |           AA132703           |                               |
|                       |           AI339634           |                               |
|                       |           BM452129           |                               |
|                       |           BM452149           |                               |
|                       |             F36688           |                               |
|                       |           AA995162           |                               |
|                       |           AA282151           |                               |
|                       |           AA743533           |                               |
|                       |           AA767301           |                               |
|                       |           AA632328           |                               |
|                       |           BG170568           |                               |
|                       |           BG180074           |                               |
|                       |             F35800           |                               |
|                       |           BI013029           |                               |
|                       |             F26803           |                               |
|                       | *Seq. mapped in strand Minus*| *Seq. mapped in strand Minus* |
|                       |           AA831631           |          AI853654             |
|                       |           BQ575029           |          BQ031615             |
|                       |           BE048574           |          BG230083             |
|                       |           AI083990           |          AK010002             |
|                       |           CK301040           |          BC019430             |
|                       |           AA148512           |          BG243363             |
|                       |           CB529756           |          BE863324             |
|                       |           BC006429           |          AA718297             |
|                       |           AK054693           |          BI112530             |
|                       |           CA420264           |          BG518616             |
|                       |           CA748482           |          BE284125             |
|                       |           AW243192           |                               |
|                       |           AA838429           |                               |
|                       |           BM678257           |                               |
|                       |           BQ772465           |                               |
|                       |           CD365943           |                               |
|                       |           CD368555           |                               |
|                       |           AK092196           |                               |
|                       |           AK124405           |                               |
|                       |           BM998748           |                               |
|                       |           CR591728           |                               |
|                       |           BX382040           |                               |
|                       |           CR626420           |                               |
|                       |           CR618993           |                               |
|                       |           BU622818           |                               |
|                       |           BU727025           |                               |
|                       |           AW207675           |                               |
|                       |           AW162612           |                               |
|                       |           CB852646           |                               |
|                       |           BM973036           |                               |
|                       |           AW583996           |                               |
|                       |           AI926821           |                               |
|                       |           AI624659           |                               |
|                       |           AW190370           |                               |
|                       |           AI431528           |                               |
|                       |           AI934360           |                               |
|                       |           AL571677           |                               |
|                       |           CR603465           |                               |
|                       |           BX379485           |                               |
|                       |           CR621252           |                               |
|                       |           BG743358           |                               |
|                       |           BG685360           |                               |
|                       |           CR600871           |                               |
|                       |           BM015598           |                               |
|                       |           BX382041           |                               |
|                       |           BI603080           |                               |
|                       |           BG117899           |                               |
|                       |           BM906263           |                               |
|                       |           BM547699           |                               |
|                       |           AL546280           |                               |
|                       |           BE385697           |                               |
|                       |           BG473304           |                               |
|                       |           BG329299           |                               |
|                       |           BM451734           |                               |
|                       |           BE736069           |                               |
|                       |           BM550253           |                               |
|                       |           BQ065852           |                               |
|                       |           BQ060845           |                               |
|                       |           BQ063054           |                               |
|                       |           BQ070633           |                               |
|                       |           BG329419           |                               |
|                       |           BM542307           |                               |
|                       |           BG332706           |                               |
|                       |           BM017754           |                               |
|                       |           BI855842           |                               |
|                       |           BQ064393           |                               |
|                       |           BI521276           |                               |
|                       |           BU183165           |                               |
|                       |           BI909636           |                               |
|                       |           BU180767           |                               |
|                       |           BU557724           |                               |
|                       |           AL557449           |                               |
|                       |           BG472993           |                               |
|                       |           BQ056584           |                               |
|                       |           BI114214           |                               |
|                       |           BE737014           |                               |
|                       |           BU526984           |                               |
|                       |           BI915411           |                               |
|                       |           BQ653552           |                               |
|                       |           BG539139           |                               |
|                       |           BQ678148           |                               |
|                       |           BF974284           |                               |
|                       |           BG697222           |                               |
|                       |           BQ887087           |                               |
|                       |           BM920060           |                               |
|                       |           BX379486           |                               |
|                       |           BQ059524           |                               |
|                       |           BG740756           |                               |
|                       |           BG437306           |                               |
|                       |           BQ059150           |                               |
|                       |           BF967521           |                               |
|                       |           BI910807           |                               |
|                       |           BG823638           |                               |
|                       |           BQ641918           |                               |
|                       |           BG329121           |                               |
|                       |           BQ928504           |                               |
|                       |           BQ692021           |                               |
|                       |           BE894629           |                               |
|                       |           BX338896           |                               |
|                       |           BE251985           |                               |
|                       |           BG700889           |                               |
|                       |           BG179982           |                               |
|                       |           BI553860           |                               |
|                       |           BI823189           |                               |
|                       |           BQ684268           |                               |
|                       |           BG401574           |                               |
|                       |           CN261635           |                               |
|                       |           BI668694           |                               |
|                       |           BI907738           |                               |
|                       |           AL558958           |                               |
|                       |           BG429563           |                               |
|                       |           CN261637           |                               |
|                       |           BE895053           |                               |
|                       |           BE255874           |                               |
|                       |           CB989952           |                               |
|                       |           BI770977           |                               |
|                       |           BU149458           |                               |
|                       |           BG398513           |                               |
|                       |           BI766381           |                               |
|                       |           BE255658           |                               |
|                       |           BI912234           |                               |
|                       |           BE735533           |                               |
|                       |           BF212966           |                               |
|                       |           BQ897148           |                               |
|                       |           BP308526           |                               |
|                       |           BG035636           |                               |
|                       |           AW160427           |                               |
|                       |           BX386575           |                               |
|                       |           BP309078           |                               |
|                       |           BQ639247           |                               |
|                       |           CN261636           |                               |
|                       |           BI837379           |                               |
|                       |           BE254306           |                               |
|                       |           BP297818           |                               |
|                       |           BP340651           |                               |
|                       |           BP297331           |                               |
|                       |           BP301843           |                               |
|                       |           BP371572           |                               |
|                       |           BP299468           |                               |
|                       |           BP297541           |                               |
|                       |             H10707           |                               |
|                       |           CV026934           |                               |
|                       |           BP213735           |                               |
|                       |           BI765748           |                               |
|                       |           BI914506           |                               |
|                       |           AI879351           |                               |
|                       |             W38853           |                               |
|                       |           CD674957           |                               |
|                       |           BI600053           |                               |
|                       |             Z44536           |                               |
|                       |           BE827371           |                               |
|                       |           BI919206           |                               |
|                       |           BM352938           |                               |
|                       |           BP212225           |                               |
|                       |           AW161045           |                               |
|                       |           AW582996           |                               |
|                       |           BI913214           |                               |
|                       |           CB134484           |                               |
|                       |           BI910277           |                               |
|                       |           BX473378           |                               |
|                       |           BP366642           |                               |
|                       |           BF882590           |                               |
|                       |           BF897735           |                               |
|                       |           AW630237           |                               |
|                       |           BF928500           |                               |
|                       |           BI915687           |                               |
|                       |           AL702513           |                               |
|                       |           CB270212           |                               |
|                       |           BM688639           |                               |
|                       |             R34406           |                               |
|                       |           BQ676446           |                               |
|                       |           BU558162           |                               |
|                       |           BQ640293           |                               |
|                       |             Z44243           |                               |
|                       |           AA323634           |                               |
|                       |           AA341658           |                               |
|                       |           AL702680           |                               |
+-----------------------+------------------------------+-------------------------------+
|      #   62           | *Seq. mapped in strand Plus* | *Seq. mapped in strand Plus*  |
|                       |           BC040641           |          AK044599             |
|                       |           AK074703           |          AB055781             |
|                       |           AY358497           |          AK003020             |
|                       |           AK074855           |          BC034655             |
|                       |           AK074947           |          BC023052             |
|                       |           AF173889           |                               |
|                       |           BX640934           |                               |
|                       |           BC008326           |                               |
|                       |           AK074750           |                               |
|                       | *Seq. mapped in strand Minus*| *Seq. mapped in strand Minus* |
|                       |           AK055470           |          BC038479             |
|                       |                              |          AK033400             |
|                       |                              |                               |
|                       |                              |                               |
|                       |                              |                               |
+-----------------------+------------------------------+-------------------------------+
|      #   63           | *Seq. mapped in strand Plus* | *Seq. mapped in strand Plus*  |
|                       |           AY358410           |          BF455654             |
|                       |           AK124200           |          BE996464             |
|                       |           AB046821           |                               |
|                       |           AF100750           |                               |
|                       |           AF304450           |                               |
|                       |           CR627321           |                               |
|                       |           AL834538           |                               |
|                       |           AK022561           |                               |
|                       | *Seq. mapped in strand Minus*| *Seq. mapped in strand Minus* |
|                       |           AF147413           |          BE689716             |
|                       |                              |          BC021457             |
+-----------------------+------------------------------+-------------------------------+
|      #   64           | *Seq. mapped in strand Plus* | *Seq. mapped in strand Plus*  |
|                       |           AK124594           |          BC033910             |
|                       |           AK126970           |                               |
|                       |             C18930           |                               |
|                       |             C18305           |                               |
|                       |           BX504123           |                               |
|                       |           BX648433           |                               |
|                       |           BE903553           |                               |
|                       |           BG773579           |                               |
|                       |           BG473772           |                               |
|                       |           BP237209           |                               |
|                       |           BP320684           |                               |
|                       |           BE019549           |                               |
|                       |           BE902342           |                               |
|                       |           BE900933           |                               |
|                       |           BE278475           |                               |
|                       |           BF667689           |                               |
|                       |           BU849628           |                               |
|                       |           BM458840           |                               |
|                       |           BU930968           |                               |
|                       |           BM737575           |                               |
|                       |             U51477           |                               |
|                       |           BG699111           |                               |
|                       |           BC041770           |                               |
|                       |           BQ948670           |                               |
|                       |           CR735602           |                               |
|                       |           AL043657           |                               |
|                       |           BQ056710           |                               |
|                       |           BQ058483           |                               |
|                       |           BE531112           |                               |
|                       |           CN311930           |                               |
|                       |             U94905           |                               |
|                       |           CN311929           |                               |
|                       |           CN311921           |                               |
|                       |           CN311924           |                               |
|                       |           BM757364           |                               |
|                       |           BM738152           |                               |
|                       |           BM772925           |                               |
|                       |           BM737925           |                               |
|                       |           BM737843           |                               |
|                       |           BM737892           |                               |
|                       |           BM738217           |                               |
|                       |           BQ427900           |                               |
|                       |           BM738200           |                               |
|                       |           CD617334           |                               |
|                       |           BM742137           |                               |
|                       |           BG480767           |                               |
|                       |           BU502675           |                               |
|                       |           BQ936294           |                               |
|                       |           BU148562           |                               |
|                       |           BU956171           |                               |
|                       |           BP220264           |                               |
|                       |           AU131298           |                               |
|                       |           BM832578           |                               |
|                       |           BP222585           |                               |
|                       |           BI911771           |                               |
|                       |           BU181498           |                               |
|                       |           CB152739           |                               |
|                       |           AA297129           |                               |
|                       |           BP199619           |                               |
|                       |           CN311928           |                               |
|                       |           BP206134           |                               |
|                       |           BP341481           |                               |
|                       |           BP203329           |                               |
|                       |           BF568625           |                               |
|                       |           BI194193           |                               |
|                       |           BI160861           |                               |
|                       |           BX456311           |                               |
|                       |           BE018998           |                               |
|                       |           CO247560           |                               |
|                       |           BF762323           |                               |
|                       |           BC008018           |                               |
|                       |             M79109           |                               |
|                       |           BF955282           |                               |
|                       |           AL134638           |                               |
|                       |           CD617333           |                               |
|                       |           CB045838           |                               |
|                       |           BX400019           |                               |
|                       |           BM796623           |                               |
|                       |           AW504084           |                               |
|                       |           BM799032           |                               |
|                       |           BM854496           |                               |
|                       |           CD579371           |                               |
|                       |           BX115617           |                               |
|                       |           AA326664           |                               |
|                       |           BQ962617           |                               |
|                       |           BM019243           |                               |
|                       |           BU956842           |                               |
|                       |           BG253428           |                               |
|                       |           BI090439           |                               |
|                       |           BQ898217           |                               |
|                       |           AA262204           |                               |
|                       |           BU521182           |                               |
|                       |           BE207200           |                               |
|                       |           BI092718           |                               |
|                       |           CN311931           |                               |
|                       |           BF845281           |                               |
|                       |           BG010019           |                               |
|                       |           AA252418           |                               |
|                       |           BM722648           |                               |
|                       |           BG008633           |                               |
|                       |           BU931802           |                               |
|                       |           BU844966           |                               |
|                       |           CN311925           |                               |
|                       |           CN311920           |                               |
|                       |           BM010631           |                               |
|                       |           AL529227           |                               |
|                       |           AL529033           |                               |
|                       |           AL529034           |                               |
|                       |           BM708849           |                               |
|                       |           BX370257           |                               |
|                       |           BU730879           |                               |
|                       |           CA424647           |                               |
|                       |           BM679137           |                               |
|                       |           BU741843           |                               |
|                       |           AI862452           |                               |
|                       |           AA742392           |                               |
|                       |           AA252502           |                               |
|                       |           AI768521           |                               |
|                       |           AI000903           |                               |
|                       |           BM193507           |                               |
|                       |           BE672475           |                               |
|                       |           BM987433           |                               |
|                       |           AW615638           |                               |
|                       |           AA977068           |                               |
|                       | *Seq. mapped in strand Minus*| *Seq. mapped in strand Minus* |
|                       |           AA814439           |          BC014860             |
+-----------------------+------------------------------+-------------------------------+
|      #   65           | *Seq. mapped in strand Plus* | *Seq. mapped in strand Plus*  |
|                       |           BP228536           |          BB852612             |
|                       |           AK075382           |          BY792270             |
|                       |           CF993870           |          BY784908             |
|                       |           BP227463           |          BY788757             |
|                       |           BI669398           |          BB849305             |
|                       |           BI550728           |          BY289559             |
|                       |           BM146497           |          BY264610             |
|                       |           CB996629           |          CB231320             |
|                       |           CB996881           |          BY042997             |
|                       |           CB991979           |          BB586499             |
|                       |           BG529889           |          BY110415             |
|                       |           BP380008           |          BY792499             |
|                       |           BP212507           |          CB233275             |
|                       |           BI598550           |          AU050986             |
|                       |           BG701716           |          AA815658             |
|                       |           BM788036           |          BE948392             |
|                       |           BM172186           |          CA888322             |
|                       |           BG714144           |          CB236484             |
|                       |           BG709010           |          CB232195             |
|                       |           BG703581           |          CB231925             |
|                       |           BG748332           |          BB842995             |
|                       |           BP237793           |          AW912002             |
|                       |           BP378864           |          CB236372             |
|                       |           BX956245           |          AI316897             |
|                       |           BG757745           |          BY309990             |
|                       |           BG759989           |          BY146497             |
|                       |           BG756910           |          BY334251             |
|                       |           BI602693           |          BY786542             |
|                       |           BI597260           |          BY081472             |
|                       |           BI553110           |          BY312663             |
|                       |           BG426572           |          BY033349             |
|                       |           BM146142           |          BY095738             |
|                       |           BI670104           |          BY097797             |
|                       |           BG714762           |          BY266883             |
|                       |           CB961051           |          BY156446             |
|                       |           CB988816           |          BY279095             |
|                       |           BG548534           |          BY290451             |
|                       |           BP231424           |          BY260179             |
|                       |           BM839658           |          BY286334             |
|                       |           BG714399           |          BY268473             |
|                       |           BG702354           |          BY031642             |
|                       |           BG717413           |          BY738328             |
|                       |           AA194554           |          AA823046             |
|                       |           AF248966           |          AU035395             |
|                       |           AY038990           |          AA822963             |
|                       |           BP334987           |          AU080227             |
|                       |           BP354526           |          BG976388             |
|                       |           BI553543           |          AU079816             |
|                       |           BG720064           |          AW107107             |
|                       |           BG705507           |          BY274469             |
|                       |           BG702177           |          BI645981             |
|                       |           BI551171           |          BG863776             |
|                       |           BI547816           |          BY287015             |
|                       |           BI667962           |          CB596677             |
|                       |           BI552612           |          CK793277             |
|                       |           BI544956           |          BY790259             |
|                       |           BG719929           |          BY141846             |
|                       |           BG699243           |          BY787345             |
|                       |           BI461369           |          BY784257             |
|                       |           BI561588           |          AI876886             |
|                       |           BI545593           |          CB236843             |
|                       |           BF185484           |          BG298560             |
|                       |           BF666506           |          BY131103             |
|                       |           CB994132           |          BY302228             |
|                       |           BP276620           |          CB234478             |
|                       |           BP241040           |          CA545323             |
|                       |           BP227840           |          CB209302             |
|                       |           AU280668           |          BG916154             |
|                       |           BP276763           |          BU571357             |
|                       |           BP213329           |          AU080111             |
|                       |           BP276132           |          BU503818             |
|                       |           BP297536           |          AA755546             |
|                       |           BP210367           |          AK076495             |
|                       |           CB996364           |          BI733996             |
|                       |           BI667550           |          BI665810             |
|                       |           BI597465           |          BY320674             |
|                       |           BP280503           |          BB614185             |
|                       |           CB990533           |          BY351002             |
|                       |           CB996406           |          BB847462             |
|                       |           BI667418           |          CB237147             |
|                       |           CD385906           |          CF168973             |
|                       |           CN291360           |          BY123495             |
|                       |           BE312617           |          CB231825             |
|                       |           BM193854           |          CN708065             |
|                       |           BG703676           |            C88814             |
|                       |           AF291814           |          BU706946             |
|                       |           BI669246           |          BY160985             |
|                       |           BI549117           |          BI732052             |
|                       |           BG713982           |          BF784590             |
|                       |           BG714337           |          BI657661             |
|                       |           BG708218           |          BB859528             |
|                       |           BG716215           |          BB864663             |
|                       |           BI599716           |          BB864169             |
|                       |           BI554007           |          BB860377             |
|                       |           BI596873           |          BF178365             |
|                       |           BI551951           |          CF905294             |
|                       |           BI600190           |          CF904358             |
|                       |           BI600429           |          CF906049             |
|                       |           BI551798           |          CK617883             |
|                       |           BI596508           |          BY717274             |
|                       |           BP227620           |          CB234293             |
|                       |           BP227167           |          AK017482             |
|                       |           BP290790           |          BY126470             |
|                       |           BP372630           |          BY129000             |
|                       |           BP196483           |          BY123138             |
|                       |           BP270704           |          BY316835             |
|                       |           BP341537           |          BY320053             |
|                       |           BM751198           |          BY352323             |
|                       |           BG759126           |          BB841857             |
|                       |           BI552663           |          BY317415             |
|                       |           BU543595           |          BY260465             |
|                       |           BI457903           |          BI078312             |
|                       |           BI598368           |          BY173837             |
|                       |           BG705052           |          BY171700             |
|                       |           BM839240           |          BY080585             |
|                       |           BI667259           |          BY161959             |
|                       |           BI547777           |          BB630870             |
|                       |           BP211693           |          BY277374             |
|                       |           BI547699           |          CA493165             |
|                       |           BG699888           |          AU080392             |
|                       |           BI667797           |          AI226594             |
|                       |           BI553732           |          AA867880             |
|                       |           BI560770           |          AI931504             |
|                       |           BQ956599           |          AI527733             |
|                       |           BU556894           |          AW106795             |
|                       |           BI599385           |          BI685790             |
|                       |           BG703191           |          BE573270             |
|                       |           CB960730           |          BE533490             |
|                       |           CB960864           |          BE569483             |
|                       |           AA297471           |          AW988918             |
|                       |           AA298119           |          AU035148             |
|                       |           BI603333           |          CB237754             |
|                       |           BG773211           |          BY250731             |
|                       |           BF697773           |          AA645356             |
|                       |           BG705983           |          AW318610             |
|                       |           BI545697           |          AW318828             |
|                       |           BP353046           |          BF143123             |
|                       |           BP292300           |          BI250326             |
|                       |           AI092581           |          BF123569             |
|                       |           AI333667           |          AA277184             |
|                       |           BI755821           |          CB234637             |
|                       |           BP267890           |          BG172247             |
|                       |           AI880032           |          BG294880             |
|                       |           AI349539           |          BI329731             |
|                       |           BF431079           |          BQ715584             |
|                       |           AI366997           |          AW989004             |
|                       |           AI040265           |          BQ551095             |
|                       |           AW392222           |          BE853800             |
|                       |           AW003884           |          BI729740             |
|                       |           AY429341           |          AW259031             |
|                       |           AI589478           |          AW260017             |
|                       |           AI567231           |          BY267460             |
|                       |           AI682889           |          BY269941             |
|                       |           CB066889           |          BG966028             |
|                       |           AW438835           |          BG965853             |
|                       |           AA584287           |          CB233258             |
|                       |           CA388902           |          BE687794             |
|                       |           BP328025           |          BE851264             |
|                       |           BI468447           |          BG972466             |
|                       |           CA773546           |          BG519469             |
|                       |           BI764649           |          BQ928017             |
|                       |           BI603997           |          BF101430             |
|                       |             H07998           |          BI988188             |
|                       |           AA782669           |          BE569816             |
|                       |           AI804330           |          BG965935             |
|                       |           AI244079           |          BF719294             |
|                       |           AI244083           |          CD805868             |
|                       |           AI349540           |          BF011672             |
|                       |           AA025751           |          CF911760             |
|                       |           AI244926           |          BF468315             |
|                       |           AI084246           |          BU707267             |
|                       |             H86728           |          BY122811             |
|                       |           AA053862           |          BE303448             |
|                       |           AI352243           |          AK029405             |
|                       |           AI948455           |          BF119697             |
|                       |           AI349546           |          AA171248             |
|                       |           AI214761           |          AW741947             |
|                       |           AI034047           |          CD771692             |
|                       |           AA854083           |          CN539891             |
|                       |             R63080           |          BI078606             |
|                       |           BF058045           |          AA839653             |
|                       |           AA489232           |          BG173896             |
|                       |           AA878389           |          BU920947             |
|                       |             H12551           |          BE851218             |
|                       |             R44801           |          AI789021             |
|                       |             R66609           |          AI930319             |
|                       |           AA890540           |          BM943075             |
|                       |             W15286           |          BC014706             |
|                       |             H96859           |          BU709540             |
|                       |             R63665           |          BF100333             |
|                       |           AA846586           |          BE291891             |
|                       |           AI095352           |          AW413298             |
|                       |           AI022504           |          CK619748             |
|                       |           AA251015           |          AW744313             |
|                       |           AA844543           |          CA947798             |
|                       |             R79321           |          CF352899             |
|                       |           AI128976           |          CF908663             |
|                       |             N24900           |          BF786430             |
|                       |             N27425           |          AW985491             |
|                       |             R79812           |          BI104299             |
|                       |           AI366775           |          AA647140             |
|                       |           AI159809           |          CK624500             |
|                       |           AI075460           |          CF749485             |
|                       |           AA903999           |          BG298183             |
|                       |           AI242587           |          BE309388             |
|                       |           AI023956           |          CD773738             |
|                       |           BF732377           |          BG919050             |
|                       |           AA947478           |          CN707920             |
|                       |           AA988744           |          BB176707             |
|                       |           AA713478           |          BF149989             |
|                       |             W37530           |          BE374581             |
|                       |           AW166105           |          AW908431             |
|                       |           BF196461           |          BF166848             |
|                       |           AI817629           |          BI145899             |
|                       |           AA397898           |          BG244389             |
|                       |           AW024459           |          BE198768             |
|                       |           BF060652           |          BF020733             |
|                       |           AI493440           |          BY579357             |
|                       |           AA528178           |          CF105663             |
|                       |             R34849           |          BF788565             |
|                       |           AA890442           |          AI173768             |
|                       |           AI571215           |          AI789946             |
|                       |           AI028502           |          BQ177855             |
|                       |           AA298829           |          BG101010             |
|                       |           AA313317           |          BF100402             |
|                       |             W52608           |          AA763415             |
|                       |           AL049929           |                               |
|                       |           AA025912           |                               |
|                       |           BP341864           |                               |
|                       |           AA255919           |                               |
|                       |             W37670           |                               |
|                       |           CN291362           |                               |
|                       |             R17981           |                               |
|                       |             H65558           |                               |
|                       |             H10236           |                               |
|                       |           AA636009           |                               |
|                       |           BQ777336           |                               |
|                       |             N76218           |                               |
|                       |           AA393599           |                               |
|                       |           BP265486           |                               |
|                       |           BF087152           |                               |
|                       |           AV702380           |                               |
|                       |           BG530418           |                               |
|                       |           AA298825           |                               |
|                       |           AW958900           |                               |
|                       |             R77590           |                               |
|                       |           BE927639           |                               |
|                       |           CK301135           |                               |
|                       |             H59176           |                               |
|                       |           AV721438           |                               |
|                       |             H56745           |                               |
|                       |           CD251988           |                               |
|                       |           CB998477           |                               |
|                       |           BP368053           |                               |
|                       |           BG696765           |                               |
|                       |           BG403007           |                               |
|                       |           BP289916           |                               |
|                       |           BF214591           |                               |
|                       |           BP294801           |                               |
|                       |           AA416560           |                               |
|                       |             N41633           |                               |
|                       |           AV726680           |                               |
|                       |             F12487           |                               |
|                       |             R51426           |                               |
|                       |           BE967755           |                               |
|                       |             T74223           |                               |
|                       |           CN291365           |                               |
|                       |           BP367103           |                               |
|                       |           AA313617           |                               |
|                       |           AW951698           |                               |
|                       |           BX118820           |                               |
|                       |           CB123145           |                               |
|                       |           BC010395           |                               |
|                       |           AA084217           |                               |
|                       |           BI850231           |                               |
|                       |           BF665336           |                               |
|                       |           CN291363           |                               |
|                       |           BG288867           |                               |
|                       |             N35735           |                               |
|                       |           AA298831           |                               |
|                       |           BQ219111           |                               |
|                       |             H85240           |                               |
|                       |           BP228514           |                               |
|                       |           BE897106           |                               |
|                       |           BU679094           |                               |
|                       |           CB994486           |                               |
|                       |           BI334714           |                               |
|                       |             N48016           |                               |
|                       |           BI906526           |                               |
|                       |           BQ335419           |                               |
|                       |           CA432635           |                               |
|                       |           BU193939           |                               |
|                       |           CK904572           |                               |
|                       |           BG501143           |                               |
|                       |           AA298846           |                               |
|                       |             Y17975           |                               |
|                       |           BU607997           |                               |
|                       |           BM069679           |                               |
|                       |           BM054885           |                               |
|                       |           CR739577           |                               |
|                       |             N30541           |                               |
|                       |           CA776340           |                               |
|                       |           CA773328           |                               |
|                       |             N57460           |                               |
|                       |           BI468446           |                               |
|                       |           BG679828           |                               |
|                       |           AA164656           |                               |
|                       |           AA437350           |                               |
|                       |           BE709243           |                               |
|                       |           BG109874           |                               |
|                       |           AA298870           |                               |
|                       |           BG622445           |                               |
|                       |           BF570757           |                               |
|                       |           CK904571           |                               |
|                       |           BE707078           |                               |
|                       |           BE169352           |                               |
|                       |           BE707045           |                               |
|                       |           BQ788022           |                               |
|                       |           BG778430           |                               |
|                       |             W58241           |                               |
|                       |           BG714109           |                               |
|                       |           BG538574           |                               |
|                       |           AW392267           |                               |
|                       |             W42526           |                               |
|                       |           BF212134           |                               |
|                       |             N41531           |                               |
|                       |           BM054691           |                               |
|                       |           AA576267           |                               |
|                       |           AA255918           |                               |
|                       |           BM069457           |                               |
|                       |           AA417296           |                               |
|                       |           CD678490           |                               |
|                       |             N27239           |                               |
|                       |           BE827568           |                               |
|                       |             H96444           |                               |
|                       |           AA928947           |                               |
|                       |           BG104855           |                               |
|                       |           AA148013           |                               |
|                       |           AA298852           |                               |
|                       |           CV030058           |                               |
|                       |             W94541           |                               |
|                       |             R78826           |                               |
|                       |           BE068801           |                               |
|                       |           BP382958           |                               |
|                       |             T61972           |                               |
|                       |           AA773339           |                               |
|                       |           CA393493           |                               |
|                       |           AI983274           |                               |
|                       |           BI869290           |                               |
|                       |           AI797520           |                               |
|                       |           BE538275           |                               |
|                       |           AA825307           |                               |
|                       |           AA281406           |                               |
|                       |           AI200275           |                               |
|                       |           BG219537           |                               |
|                       |             N73409           |                               |
|                       |             R34946           |                               |
|                       | *Seq. mapped in strand Minus*| *Seq. mapped in strand Minus* |
|                       |           BU685665           |          AI844668             |
+-----------------------+------------------------------+-------------------------------+
|      #   66           | *Seq. mapped in strand Plus* | *Seq. mapped in strand Plus*  |
|                       |           BM997890           |          AK033276             |
|                       |                              |          BC072584             |
|                       |                              |          AK011557             |
|                       |                              |          BC028444             |
|                       | *Seq. mapped in strand Minus*| *Seq. mapped in strand Minus* |
|                       |           AY378097           |          AK037147             |
|                       |           AW135830           |          AK003910             |
|                       |           AW001657           |                               |
|                       |           AW005184           |                               |
|                       |           AK000408           |                               |
|                       |           BC021860           |                               |
|                       |           BC028614           |                               |
|                       |           AI241453           |                               |
|                       |           BX389914           |                               |
|                       |           CR608210           |                               |
|                       |           BM993355           |                               |
|                       |           AI830353           |                               |
|                       |           AL578602           |                               |
|                       |           AA082573           |                               |
|                       |           BX089325           |                               |
|                       |           AW969501           |                               |
|                       |           AA487002           |                               |
|                       |           AW369661           |                               |
|                       |             T87441           |                               |
|                       |           BM906443           |                               |
|                       |           BI334056           |                               |
|                       |             R80760           |                               |
|                       |           AL556313           |                               |
|                       |             R25478           |                               |
|                       |           BP271696           |                               |
|                       |           BF516466           |                               |
|                       |           AI478756           |                               |
|                       |           BE222437           |                               |
|                       |           AW615408           |                               |
|                       |           BE301708           |                               |
|                       |           BF058506           |                               |
|                       |           BC042111           |                               |
|                       |           BM694838           |                               |
|                       |           BM922235           |                               |
|                       |           BI822309           |                               |
|                       |           CB177560           |                               |
|                       |           BI819925           |                               |
+-----------------------+------------------------------+-------------------------------+
|      #   67           | *Seq. mapped in strand Plus* | *Seq. mapped in strand Plus*  |
|                       |           BX113702           |          BF455520             |
|                       |                              |          AJ006821             |
|                       |                              |          AK033437             |
|                       |                              |          BC003933             |
|                       |                              |          BE956092             |
|                       |                              |          BE980741             |
|                       |                              |          AI504365             |
|                       |                              |          BX519726             |
|                       |                              |          BE990728             |
|                       | *Seq. mapped in strand Minus*| *Seq. mapped in strand Minus* |
|                       |           BI712556           |          AW060953             |
|                       |           BU076089           |                               |
|                       |             R44702           |                               |
|                       |             R44703           |                               |
|                       |           AI033762           |                               |
|                       |           AW103783           |                               |
|                       |           CA435514           |                               |
|                       |           AW074400           |                               |
|                       |           AF113224           |                               |
|                       |           BE349039           |                               |
|                       |           CB120276           |                               |
|                       |           AA825596           |                               |
|                       |           AI805379           |                               |
|                       |           BF446754           |                               |
|                       |           AI371803           |                               |
|                       |           AJ711912           |                               |
|                       |           BG199318           |                               |
|                       |           AI423223           |                               |
|                       |           BG207610           |                               |
|                       |           BG207609           |                               |
|                       |           BG211810           |                               |
|                       |           BG187511           |                               |
|                       |           BG212863           |                               |
|                       |           CD629240           |                               |
|                       |           AF208863           |                               |
|                       |           AW771347           |                               |
|                       |           BM996358           |                               |
|                       |           AI159832           |                               |
|                       |           BQ287809           |                               |
|                       |           BG217153           |                               |
|                       |           BG217154           |                               |
|                       |           BG206599           |                               |
|                       |           BG183266           |                               |
|                       |           BG194797           |                               |
|                       |           BG212331           |                               |
|                       |           BG189594           |                               |
|                       |           BG204988           |                               |
|                       |           AF211480           |                               |
|                       |           BX647737           |                               |
|                       |           BM129279           |                               |
|                       |           BG219704           |                               |
|                       |           CD678164           |                               |
|                       |           BG181712           |                               |
|                       |           BG215008           |                               |
|                       |           BG191634           |                               |
|                       |           AA838762           |                               |
|                       |           BG183267           |                               |
|                       |           BG191636           |                               |
|                       |           BG211275           |                               |
|                       |           BU947386           |                               |
|                       |           AA627345           |                               |
|                       |           BG190143           |                               |
|                       |           BG199319           |                               |
|                       |           AA446322           |                               |
|                       |           BU950686           |                               |
|                       |           AA721753           |                               |
|                       |           BU950383           |                               |
|                       |           AA043190           |                               |
|                       |           AL551352           |                               |
|                       |           CR612672           |                               |
|                       |           AA641529           |                               |
|                       |           BQ286780           |                               |
|                       |           AI766639           |                               |
|                       |           CA943518           |                               |
|                       |           AI886950           |                               |
|                       |           AU157616           |                               |
|                       |           BI711177           |                               |
|                       |           BG213382           |                               |
|                       |           BG206598           |                               |
|                       |           BG188510           |                               |
|                       |           BG198131           |                               |
|                       |           BG191635           |                               |
|                       |           BG190998           |                               |
|                       |           BG197255           |                               |
|                       |           BG209244           |                               |
|                       |           BM969548           |                               |
|                       |           BQ003134           |                               |
|                       |           BM975765           |                               |
|                       |           AK002102           |                               |
|                       |           BC047073           |                               |
|                       |           BG215009           |                               |
|                       |           BG212865           |                               |
|                       |           AW971689           |                               |
|                       |           BC010360           |                               |
|                       |           BX329881           |                               |
|                       |           AI147871           |                               |
|                       |           BF677334           |                               |
|                       |           BU568687           |                               |
|                       |           BI490782           |                               |
|                       |           CR595980           |                               |
|                       |           BM142293           |                               |
|                       |           CR618396           |                               |
|                       |           BX406877           |                               |
|                       |           AL550888           |                               |
|                       |           AA446503           |                               |
|                       |           BM354027           |                               |
|                       |           BE173585           |                               |
|                       |           BE173409           |                               |
|                       |           CD629241           |                               |
|                       |           AL551308           |                               |
|                       |           BE173481           |                               |
|                       |           BE173471           |                               |
|                       |           BF102702           |                               |
|                       |             C20624           |                               |
|                       |           BG402269           |                               |
|                       |           AA043189           |                               |
|                       |           BE173590           |                               |
|                       |           BG191633           |                               |
|                       |           BG212864           |                               |
|                       |           AA369579           |                               |
|                       |           BU790301           |                               |
|                       |           BG190643           |                               |
|                       |           BQ417266           |                               |
|                       |           AW827132           |                               |
|                       |           AA369411           |                               |
|                       |           CB157000           |                               |
|                       |           BM352255           |                               |
|                       |           BG211811           |                               |
|                       |           BG220190           |                               |
|                       |           BG565225           |                               |
|                       |             N57339           |                               |
|                       |           BI859816           |                               |
|                       |           BF218355           |                               |
|                       |           BF691477           |                               |
|                       |             H92698           |                               |
|                       |           AA326713           |                               |
|                       |           BX452524           |                               |
|                       |           BF576897           |                               |
|                       |           CB998672           |                               |
|                       |           AA349280           |                               |
|                       |           BQ439774           |                               |
|                       |           BQ950910           |                               |
|                       |           AA256744           |                               |
|                       |           AA376823           |                               |
|                       |           AU136625           |                               |
|                       |           BX411451           |                               |
|                       |           AL560287           |                               |
|                       |           CV030059           |                               |
|                       |           AA046581           |                               |
|                       |             W31760           |                               |
|                       |           AA385915           |                               |
|                       |             T61007           |                               |
|                       |           BF695259           |                               |
|                       |           CN427022           |                               |
|                       |           AA459236           |                               |
|                       |           AA127618           |                               |
|                       |           BE502920           |                               |
|                       |           AW452739           |                               |
|                       |           AA022820           |                               |
|                       |           BF208141           |                               |
|                       |           BM802342           |                               |
|                       |           BM761422           |                               |
|                       |           BP239884           |                               |
|                       |           BG314772           |                               |
|                       |           BQ721814           |                               |
|                       |           BX421453           |                               |
|                       |           AV758108           |                               |
|                       |           CN427020           |                               |
|                       |           AW361626           |                               |
|                       |           CB991666           |                               |
|                       |           BQ072725           |                               |
|                       |           BX346030           |                               |
|                       |           AI566027           |                               |
|                       |           BQ214397           |                               |
|                       |           BE150505           |                               |
|                       |           BF951257           |                               |
|                       |           BI489830           |                               |
|                       |           BG542873           |                               |
|                       |           CN427018           |                               |
|                       |           BI597530           |                               |
|                       |           BG548486           |                               |
|                       |           BG716772           |                               |
|                       |           AW813810           |                               |
|                       |           BM504911           |                               |
|                       |           CF265153           |                               |
|                       |           BI460488           |                               |
|                       |           AU139755           |                               |
|                       |           BI552305           |                               |
|                       |           BG542622           |                               |
|                       |           BG400199           |                               |
|                       |           BG720009           |                               |
|                       |           BF352288           |                               |
|                       |           CB266777           |                               |
|                       |           BE866130           |                               |
|                       |           BI550519           |                               |
|                       |           CN427019           |                               |
|                       |           BP270148           |                               |
|                       |           AU138444           |                               |
|                       |           BP274019           |                               |
|                       |           BI553614           |                               |
|                       |           BI712797           |                               |
|                       |           BP197997           |                               |
|                       |           BP198344           |                               |
|                       |           BX489558           |                               |
|                       |           CA388735           |                               |
|                       |           BP197679           |                               |
|                       |           CA867502           |                               |
|                       |           BP220037           |                               |
|                       |           BI561534           |                               |
|                       |           BU072102           |                               |
|                       |           BI715429           |                               |
|                       |           BF966206           |                               |
|                       |           BQ270120           |                               |
|                       |           BP342304           |                               |
|                       |           BG900983           |                               |
|                       |           BP341775           |                               |
|                       |           AL703217           |                               |
|                       |           BX395894           |                               |
|                       |           BP259652           |                               |
|                       |           AU280545           |                               |
|                       |           BP274346           |                               |
|                       |           CD699244           |                               |
|                       |           CN427021           |                               |
|                       |           BX471084           |                               |
|                       |           CD692913           |                               |
|                       |           BP344168           |                               |
|                       |           BI551220           |                               |
|                       |           BP274670           |                               |
|                       |           AW753423           |                               |
|                       |           BP259160           |                               |
|                       |           CB270820           |                               |
|                       |           BX489574           |                               |
|                       |           BI710859           |                               |
|                       |           CN427017           |                               |
+-----------------------+------------------------------+-------------------------------+
|      #   68           | *Seq. mapped in strand Plus* | *Seq. mapped in strand Plus*  |
|                       |           BC014050           |          BY347589             |
|                       |           AK023738           |          BY350928             |
|                       |           AK023448           |          BY349089             |
|                       |           BC068997           |          BY351462             |
|                       |           AK056465           |          BY621586             |
|                       |           AK054897           |          BU563770             |
|                       |                              |          CK381357             |
|                       |                              |          BU534238             |
|                       |                              |          BY338876             |
|                       |                              |          BU558499             |
|                       |                              |          BU851122             |
|                       |                              |          BY097460             |
|                       |                              |          CA469297             |
|                       |                              |          BY285531             |
|                       |                              |          BY460016             |
|                       |                              |          BY261759             |
|                       |                              |          AA048225             |
|                       |                              |          BU534523             |
|                       |                              |          BX514067             |
|                       |                              |          BY348295             |
|                       |                              |          BY352600             |
|                       |                              |            W82854             |
|                       |                              |          BY056697             |
|                       |                              |          BY349388             |
|                       |                              |          CF351582             |
|                       |                              |          CF929075             |
|                       |                              |          CF968641             |
|                       |                              |          CF379958             |
|                       |                              |          CK381927             |
|                       |                              |          CF969986             |
|                       |                              |          BM122700             |
|                       |                              |          BQ553963             |
|                       |                              |          BY449533             |
|                       |                              |          BF456459             |
|                       |                              |          BY600981             |
|                       |                              |          BY238864             |
|                       |                              |          BY094717             |
|                       |                              |          BY108214             |
|                       |                              |          BY082612             |
|                       |                              |          BY245889             |
|                       |                              |          BX526162             |
|                       |                              |          BE624249             |
|                       |                              |            W82213             |
|                       |                              |            W91392             |
|                       |                              |            W20580             |
|                       |                              |            W74885             |
|                       |                              |            W78599             |
|                       |                              |          AW114002             |
|                       |                              |          BY514033             |
|                       |                              |          AA253944             |
|                       |                              |          CB317658             |
|                       |                              |          BE626632             |
|                       |                              |          BC028526             |
|                       |                              |          CA466469             |
|                       |                              |          AI429768             |
|                       |                              |          CA463146             |
|                       |                              |          AA869307             |
|                       |                              |          BY674153             |
|                       |                              |          AA492958             |
|                       |                              |          CF423565             |
|                       |                              |          AA423483             |
|                       |                              |          BU961836             |
|                       |                              |          AA242198             |
|                       |                              |          BI715575             |
|                       |                              |          AK007332             |
|                       |                              |          BY412226             |
|                       |                              |          AA199385             |
|                       |                              |          BI715458             |
|                       |                              |          BI789628             |
|                       |                              |            W64661             |
|                       |                              |          BI789897             |
|                       |                              |          BI714870             |
|                       |                              |          BI714957             |
|                       |                              |          AA521570             |
|                       |                              |          AA268962             |
|                       |                              |          BE631928             |
|                       |                              |          CF356861             |
|                       |                              |          AI118854             |
|                       |                              |          AV070090             |
|                       |                              |          AV056118             |
|                       |                              |          CA586786             |
|                       |                              |          AV072164             |
|                       |                              |          AV064558             |
|                       |                              |          AV053267             |
|                       |                              |          AV080217             |
|                       | *Seq. mapped in strand Minus*| *Seq. mapped in strand Minus* |
|                       |           AK026550           |          BQ832714             |
|                       |           AK001285           |          AV253561             |
|                       |           BC005398           |          BQ556366             |
|                       |           AL050051           |          BU961454             |
|                       |           BX537696           |          AK076549             |
|                       |           CR601244           |          BU962998             |
|                       |           AF086169           |          CA465871             |
|                       |                              |          AI790495             |
|                       |                              |          BY442983             |
|                       |                              |          CO043629             |
|                       |                              |          BC048718             |
|                       |                              |          AA138615             |
|                       |                              |          AW107703             |
|                       |                              |          BM119519             |
|                       |                              |          BM120062             |
|                       |                              |          BM119877             |
|                       |                              |          BU962961             |
|                       |                              |          BU937276             |
|                       |                              |          CN832973             |
|                       |                              |          CB273035             |
|                       |                              |          AI449683             |
|                       |                              |          BQ885434             |
|                       |                              |          BQ922498             |
|                       |                              |          AA675707             |
|                       |                              |          CB271195             |
|                       |                              |          BQ042681             |
|                       |                              |          CB272704             |
|                       |                              |          BX518721             |
|                       |                              |          BB009578             |
|                       |                              |          AA275867             |
|                       |                              |          AA119790             |
|                       |                              |          BU935774             |
|                       |                              |          BU937409             |
|                       |                              |          BF150382             |
|                       |                              |          CN835513             |
|                       |                              |          BE646863             |
|                       |                              |          CA979660             |
|                       |                              |          BU515854             |
|                       |                              |          CN539375             |
|                       |                              |          CA773020             |
|                       |                              |          BY747287             |
|                       |                              |          BF731488             |
|                       |                              |          CN702854             |
|                       |                              |          CF730334             |
|                       |                              |          BB609050             |
|                       |                              |          CF894572             |
|                       |                              |          BU936412             |
|                       |                              |          CN698006             |
|                       |                              |          BB572459             |
|                       |                              |          BG800206             |
|                       |                              |          BE985269             |
|                       |                              |          AW106743             |
|                       |                              |          CD560827             |
|                       |                              |          AI606414             |
|                       |                              |          AA138938             |
|                       |                              |          CK388577             |
|                       |                              |          CD552653             |
|                       |                              |          AI746656             |
|                       |                              |          CN681430             |
|                       |                              |          AW046078             |
|                       |                              |          BQ556367             |
|                       |                              |          BY270782             |
|                       |                              |          AA879953             |
|                       |                              |          BY334253             |
|                       |                              |          BG062414             |
|                       |                              |          BF464025             |
|                       |                              |                               |
|                       |                              |                               |
|                       |                              |                               |
|                       |                              |                               |
|                       |                              |                               |
|                       |                              |                               |
|                       |                              |                               |
|                       |                              |                               |
|                       |                              |                               |
|                       |                              |                               |
|                       |                              |                               |
|                       |                              |                               |
|                       |                              |                               |
|                       |                              |                               |
|                       |                              |                               |
|                       |                              |                               |
|                       |                              |                               |
|                       |                              |                               |
|                       |                              |                               |
+-----------------------+------------------------------+-------------------------------+
|      #   69           | *Seq. mapped in strand Plus* | *Seq. mapped in strand Plus*  |
|                       |           AK125247           |          AW495629             |
|                       |                              |          BE947385             |
|                       | *Seq. mapped in strand Minus*| *Seq. mapped in strand Minus* |
|                       |           BC021046           |          CF379827             |
|                       |           AL136702           |          AW214186             |
+-----------------------+------------------------------+-------------------------------+
|      #   70           | *Seq. mapped in strand Plus* | *Seq. mapped in strand Plus*  |
|                       |           BU570504           |          CA526466             |
|                       |           BC056685           |          BY336038             |
|                       |           BP363682           |          BY331503             |
|                       |           BP192859           |          AF132726             |
|                       |           BC012574           |          CN701342             |
|                       |           BC027850           |          CF166688             |
|                       |           CN356213           |          CF585404             |
|                       |           BP201466           |          AK011818             |
|                       |           CN356212           |          AI639914             |
|                       |           CD652327           |          AA675670             |
|                       |           AL603075           |          AU015347             |
|                       |           AF165161           |          CF585403             |
|                       |           BE675187           |          BG073027             |
|                       |           CN356211           |          BM200858             |
|                       |           CN356215           |                               |
|                       |           BM970205           |                               |
|                       |           BI917496           |                               |
|                       |           AA737658           |                               |
|                       |           AV723228           |                               |
|                       | *Seq. mapped in strand Minus*| *Seq. mapped in strand Minus* |
|                       |           CA312234           |          CK374321             |
+-----------------------+------------------------------+-------------------------------+
|      #   71           | *Seq. mapped in strand Plus* | *Seq. mapped in strand Plus*  |
|                       |           AI831837           |          BF148719             |
|                       | *Seq. mapped in strand Minus*| *Seq. mapped in strand Minus* |
|                       |           AI391505           |          BF658916             |
|                       |           AW449397           |          AW411575             |
|                       |           BG057197           |          BE996262             |
|                       |           CA443458           |          CF354869             |
|                       |             U94831           |          CF382344             |
|                       |             R44778           |          CF423821             |
|                       |             N93753           |          CF805118             |
|                       |           BQ001853           |          CF925892             |
|                       |           AW296244           |          CK385095             |
|                       |           CA424205           |          AI893436             |
|                       |           AI690830           |          AK004754             |
|                       |           AI167659           |          CF423902             |
|                       |           BE503565           |          BG920687             |
|                       |           AW589479           |          CF354532             |
|                       |           BG057709           |          CF805059             |
|                       |           BQ001680           |          CF927031             |
|                       |           BP330035           |          BE691068             |
|                       |           AA861929           |          AW488028             |
|                       |           BU683191           |          AI851160             |
|                       |           AI982533           |          BC007187             |
|                       |           BU631911           |          AF269152             |
|                       |           BU630261           |          BC017617             |
|                       |           CA449038           |          BG917179             |
|                       |           CA446452           |          BY755221             |
|                       |           CB241575           |          AK090065             |
|                       |           AI609706           |          BE571685             |
|                       |           BC010856           |          CB193028             |
|                       |           BM682525           |          BU846871             |
|                       |           CR604869           |          CK793465             |
|                       |           BX448280           |          BE532519             |
|                       |           CN407358           |          AI322383             |
|                       |           BX381585           |          BX518851             |
|                       |           CR606704           |          AA940158             |
|                       |           CR618221           |            W83187             |
|                       |           CR614922           |          BE862236             |
|                       |           BX161390           |          BE289524             |
|                       |           BF529529           |          BG976961             |
|                       |           BF984481           |          CN704207             |
|                       |           BX347118           |          CN704616             |
|                       |           BX377557           |          CB571089             |
|                       |           AA127785           |          CB203660             |
|                       |           BF946032           |          CB947583             |
|                       |           BM727764           |          BU148726             |
|                       |           BG777562           |          BE288335             |
|                       |           BE881525           |          BI661984             |
|                       |             R70430           |          CB587568             |
|                       |           BX450618           |          BF532894             |
|                       |           BX367828           |          BU058972             |
|                       |             N26442           |          CF741632             |
|                       |           BF679313           |          BF015623             |
|                       |           CB988286           |          BE914042             |
|                       |           CB990147           |          BI653871             |
|                       |             W44908           |          CB231738             |
|                       |           BX347058           |          BF101257             |
|                       |           BM887877           |          BE285738             |
|                       |           BE003856           |          BE285478             |
|                       |           BP431963           |          CB237491             |
|                       |           BE786730           |          BF607178             |
|                       |           CA489196           |          BQ936109             |
|                       |           BM556175           |          BI078592             |
|                       |           BX326236           |          BB610966             |
|                       |           BX450245           |          BI555458             |
|                       |           BE717116           |          BF714768             |
|                       |           BM717185           |          CA533707             |
|                       |           BM726091           |          CA894425             |
|                       |           BX429470           |          BY745059             |
|                       |           BX368291           |          BY745108             |
|                       |           AA303616           |          BF658615             |
|                       |           CF596293           |          CA893371             |
|                       |           CB216297           |          BG807685             |
|                       |           BX373120           |          BI328070             |
|                       |           BX352487           |          CD563005             |
|                       |           CR621520           |          BU922823             |
|                       |           BX346656           |          BY316465             |
|                       |           BX161494           |          BY249827             |
|                       |           BX362269           |          AA268737             |
|                       |           AW001226           |          BY704952             |
|                       |           BX347271           |          AI607769             |
|                       |           BX347395           |          BY166567             |
|                       |           BX388657           |          BY171413             |
|                       |           BX450617           |          BY159647             |
|                       |           BX326211           |          BY165532             |
|                       |           BX368249           |          BY074153             |
|                       |           CB989490           |          BY160722             |
|                       |           BX347006           |                               |
|                       |           CD243164           |                               |
|                       |           BX347049           |                               |
|                       |           BX390767           |                               |
|                       |           BX347250           |                               |
|                       |           BX368102           |                               |
|                       |           BX329303           |                               |
|                       |           BX408040           |                               |
|                       |           BG925497           |                               |
|                       |           BF094556           |                               |
|                       |           BF094552           |                               |
|                       |           BP261939           |                               |
|                       |           BP262267           |                               |
|                       |           BM701393           |                               |
|                       |           BX347165           |                               |
|                       |           BX417101           |                               |
|                       |             W52643           |                               |
|                       |           BX368124           |                               |
|                       |           BE872435           |                               |
|                       |           BF945858           |                               |
|                       |           CB147545           |                               |
|                       |           AI601220           |                               |
|                       |           BX331391           |                               |
|                       |           CR614007           |                               |
|                       |           BU527086           |                               |
|                       |           AI086839           |                               |
|                       |           BM353671           |                               |
|                       |           BG398091           |                               |
|                       |           BX331392           |                               |
|                       |           BF316720           |                               |
|                       |           BQ897875           |                               |
|                       |           BX335531           |                               |
|                       |           AU118420           |                               |
|                       |           BQ221987           |                               |
|                       |           BG179330           |                               |
|                       |           BP300148           |                               |
|                       |           BX367924           |                               |
|                       |           BX429145           |                               |
|                       |           CF138781           |                               |
|                       |           BX367971           |                               |
|                       |           BX347480           |                               |
|                       |           BX347479           |                               |
|                       |           BI823379           |                               |
|                       |           AI752329           |                               |
|                       |           AI752330           |                               |
|                       |           BF742191           |                               |
|                       |           BU162304           |                               |
|                       |           BF765542           |                               |
|                       |           BQ436677           |                               |
|                       |           BG824193           |                               |
|                       |           CN484799           |                               |
|                       |           BX377558           |                               |
|                       |           AA436865           |                               |
|                       |           AI638805           |                               |
|                       |           BX339718           |                               |
|                       |           BQ682946           |                               |
|                       |           BG427372           |                               |
|                       |           BX446577           |                               |
|                       |           BX393958           |                               |
|                       |           BX346657           |                               |
|                       |           BX396160           |                               |
|                       |           BX381586           |                               |
|                       |           AW673452           |                               |
|                       |           BX448281           |                               |
|                       |           BX388485           |                               |
|                       |           BX332624           |                               |
|                       |           CO247342           |                               |
|                       |           BE899402           |                               |
|                       |           BP292570           |                               |
|                       |           BX362270           |                               |
|                       |           CA487757           |                               |
|                       |           BP292566           |                               |
|                       |           BI916120           |                               |
|                       |           BI826650           |                               |
|                       |           AI766919           |                               |
|                       |           AA857204           |                               |
|                       |           AI264423           |                               |
|                       |           AI288175           |                               |
|                       |           AI262410           |                               |
|                       |           AI346254           |                               |
|                       |           BE669959           |                               |
|                       |           AW205944           |                               |
|                       |           AI281278           |                               |
|                       |           AI167155           |                               |
|                       |           AI861789           |                               |
|                       |           CK299561           |                               |
|                       |           AI401405           |                               |
|                       |           BU624905           |                               |
|                       |           CD368817           |                               |
|                       |           AI765810           |                               |
|                       |           BX395173           |                               |
|                       |           AU140435           |                               |
|                       |           BE898318           |                               |
|                       |           BQ189428           |                               |
|                       |           BU195363           |                               |
|                       |           BI517770           |                               |
|                       |           BI561945           |                               |
|                       |           BE907977           |                               |
|                       |           BX398908           |                               |
|                       |           BM887909           |                               |
|                       |           CF122418           |                               |
|                       |           BI770689           |                               |
|                       |           BM353954           |                               |
|                       |           BI518795           |                               |
|                       |           BM048425           |                               |
|                       |           BU164600           |                               |
|                       |           CN407359           |                               |
|                       |           BM827837           |                               |
|                       |           AI084548           |                               |
|                       |           BI601991           |                               |
|                       |           BQ439592           |                               |
|                       |           CB988769           |                               |
|                       |           BP237944           |                               |
|                       |           CB960778           |                               |
|                       |           AI557365           |                               |
|                       |           CV023228           |                               |
|                       |           BQ437298           |                               |
|                       |           CN407363           |                               |
|                       |           BE091331           |                               |
|                       |           CN407364           |                               |
|                       |           CF141100           |                               |
|                       |           BI820652           |                               |
|                       |           BI765299           |                               |
|                       |           BP335584           |                               |
|                       |           CN407361           |                               |
|                       |           BM046168           |                               |
|                       |           BP213450           |                               |
|                       |           BM922952           |                               |
|                       |             T10746           |                               |
|                       |           BX103990           |                               |
|                       |           BI457830           |                               |
|                       |             H72494           |                               |
|                       |           BP197795           |                               |
|                       |           BP257067           |                               |
|                       |           BI769800           |                               |
|                       |           BP256682           |                               |
|                       |           BP243067           |                               |
|                       |           BM919828           |                               |
|                       |           CN407360           |                               |
|                       |           BP343056           |                               |
|                       |           BM926944           |                               |
|                       |           CB164645           |                               |
|                       |           CB997961           |                               |
|                       |           CB128737           |                               |
|                       |           CB960948           |                               |
|                       |           BG327948           |                               |
|                       |           BI916668           |                               |
|                       |           CD109375           |                               |
|                       |           CB164732           |                               |
|                       |           BP265173           |                               |
|                       |           BI829089           |                               |
|                       |           BG913854           |                               |
|                       |           BP253539           |                               |
|                       |           BM755434           |                               |
|                       |           AL037237           |                               |
|                       |           CF121149           |                               |
|                       |           AU098818           |                               |
|                       |           BP326003           |                               |
|                       |           CN407362           |                               |
|                       |           AA442622           |                               |
|                       |             T75428           |                               |
|                       |           AU076647           |                               |
+-----------------------+------------------------------+-------------------------------+
|      #   72           | *Seq. mapped in strand Plus* | *Seq. mapped in strand Plus*  |
|                       |           AA564986           |          BE989497             |
|                       |                              |          BE989829             |
|                       | *Seq. mapped in strand Minus*| *Seq. mapped in strand Minus* |
|                       |           AB014517           |          AI839569             |
|                       |           AY007151           |          AK034695             |
|                       |           BQ429163           |          BE686325             |
|                       |           BF592976           |          BF461241             |
|                       |           AW960235           |          AK003885             |
|                       |           AI174906           |          BY704142             |
|                       |           BF196381           |          CF913427             |
|                       |           AA534658           |          BQ417575             |
|                       |           CA445364           |          BE632006             |
|                       |           AK098531           |          BB692497             |
|                       |           CA422856           |          BQ747397             |
|                       |           AA731127           |          CD741519             |
|                       |           AI250643           |          BC027304             |
|                       |           AA507155           |          BI652902             |
|                       |             R46484           |          AI840051             |
|                       |           AA748309           |          BM115472             |
|                       |           AA757051           |          AW546541             |
|                       |           CR748051           |          BM248658             |
|                       |           AA115733           |          BG072702             |
|                       |             N45996           |          BE631146             |
|                       |           BM679251           |          BE851872             |
|                       |             U58089           |          CF617016             |
|                       |           AF064087           |          CK790513             |
|                       |           AF062537           |          AW457180             |
|                       |           AI952996           |          CK343468             |
|                       |           AI341854           |          BG094393             |
|                       |           BM670800           |          CF950022             |
|                       |           CN369215           |          BM243075             |
|                       |           BQ575175           |          BE634167             |
|                       |           AA627747           |          BM240976             |
|                       |           AW771558           |          AI505175             |
|                       |           AW469402           |          BM243462             |
|                       |           BU686707           |          BM243230             |
|                       |           AW051740           |          BM242339             |
|                       |           BQ004545           |          BF464034             |
|                       |           BQ004777           |          CK330192             |
|                       |           CA447802           |          CK332441             |
|                       |           CA434348           |          AA611975             |
|                       |           BM977573           |          CK625228             |
|                       |           AI884610           |          CB193024             |
|                       |           CB851530           |          AA270452             |
|                       |           BF196314           |            C88780             |
|                       |           BC031844           |          AA221204             |
|                       |           BC039598           |          CK620821             |
|                       |           BG025344           |          BB033401             |
|                       |           AW295789           |          AA174992             |
|                       |           AI084250           |          AA423064             |
|                       |           AI767733           |          CB274637             |
|                       |           AI126155           |          BI080985             |
|                       |             W44397           |          AA596386             |
|                       |           AU151319           |          BQ892027             |
|                       |           BQ019764           |          BU938992             |
|                       |           AI813721           |          AA419847             |
|                       |           AI471456           |          AA674802             |
|                       |           BM696813           |          BM937294             |
|                       |           BQ017762           |          BX521019             |
|                       |           BQ017779           |          AA467481             |
|                       |           CA311477           |          CA879332             |
|                       |           BU687479           |          BG694945             |
|                       |           BM677168           |          BF161002             |
|                       |           BM668625           |          AA212173             |
|                       |           CA441445           |          AA162800             |
|                       |             R38742           |          AA183871             |
|                       |           AW051763           |          AF129738             |
|                       |           AF052147           |          AA798217             |
|                       |           AA620633           |          AI893030             |
|                       |           AI916202           |          CA870779             |
|                       |           AA824296           |          AL022857             |
|                       |           AW051762           |          AL022856             |
|                       |           AA778737           |          BE850451             |
|                       |           AI143499           |          CA537634             |
|                       |           AI129514           |          CA566386             |
|                       |           BF062712           |          BG147446             |
|                       |           AU144109           |          BG655363             |
|                       |           BE463531           |          CB271094             |
|                       |           AA994964           |          CN720317             |
|                       |           AY337761           |          BE447562             |
|                       |           AW296753           |          BI105498             |
|                       |           AA489370           |          CK346418             |
|                       |             W38413           |          CF911093             |
|                       |           AI089963           |          BG148226             |
|                       |           AI422363           |          CF913370             |
|                       |           AI245820           |          CF910545             |
|                       |           AI761831           |          CF910384             |
|                       |           AW976334           |          CN666703             |
|                       |           BG943566           |          BG144969             |
|                       |           AI276317           |          CN723154             |
|                       |           AA831437           |          CN703992             |
|                       |           AW014555           |          CN715547             |
|                       |           BM671004           |          BG085494             |
|                       |           BF197061           |          CN696730             |
|                       |           AA489058           |          CN697997             |
|                       |           CA311723           |          AA672183             |
|                       |           BU619091           |          CA880439             |
|                       |           CB054678           |          CD565727             |
|                       |           CB306448           |          AA717568             |
|                       |           CA314171           |          CA892986             |
|                       |           BG204233           |          CK390528             |
|                       |           BG182500           |          CA560538             |
|                       |           AL041557           |          CA561147             |
|                       |           BF000412           |          CA538709             |
|                       |           BU902905           |          AA636441             |
|                       |           BM690764           |          AA413636             |
|                       |           AA058931           |          CA575165             |
|                       |           BG291918           |          CA576209             |
|                       |           BG991455           |          CA576595             |
|                       |           AW867343           |          CA577008             |
|                       |           CN369211           |          CA574902             |
|                       |           BF839206           |          CA574088             |
|                       |           BF839306           |          CN710729             |
|                       |           BI826746           |          CN666765             |
|                       |           BI764821           |          CN723949             |
|                       |           BI754796           |          AI789885             |
|                       |             D61657           |          CF897967             |
|                       |           BG896207           |          BE627157             |
|                       |             N42403           |          CB947091             |
|                       |           CB054679           |          CA575328             |
|                       |           BM693654           |          CA877161             |
|                       |             N25141           |          CN692878             |
|                       |           AI693466           |          CA576358             |
|                       |           AI697959           |          CA464061             |
|                       |           AI457835           |          BE336147             |
|                       |           AA379690           |          BE305997             |
|                       |           BE669555           |          CN723040             |
|                       |           AU126709           |          BB835705             |
|                       |           AI739635           |          CF912300             |
|                       |           BP371077           |          CF907133             |
|                       |           AV748396           |          CN710007             |
|                       |           AW499872           |          BF144562             |
|                       |           AW867410           |          BI154580             |
|                       |           AA449502           |          CF896816             |
|                       |           AA380036           |          CB572551             |
|                       |           BI761457           |          CA575266             |
|                       |           AV721057           |          CF911198             |
|                       |           BG430660           |          CA575436             |
|                       |             H27081           |          CB272618             |
|                       |           BG118455           |          CF911846             |
|                       |           CD512186           |          CN675230             |
|                       |           BE717377           |          AA122934             |
|                       |           AL041781           |          AA183682             |
|                       |             W33007           |          BF583684             |
|                       |           BE717309           |          CA319607             |
|                       |           CB164739           |          BE631727             |
|                       |           BE717394           |          AA863500             |
|                       |           AU117213           |          BE851177             |
|                       |           BU198361           |          CK621811             |
|                       |             W44396           |          AA072897             |
|                       |           CR736523           |          CB201913             |
|                       |           CB957009           |          AA543345             |
|                       |           AA488807           |          CF911634             |
|                       |           BE717406           |          AA672645             |
|                       |           BG432138           |          AA792104             |
|                       |           BG006111           |          BF165390             |
|                       |           BE939574           |          BI852975             |
|                       |           BU177908           |          AA414800             |
|                       |           BF671734           |          AA547444             |
|                       |           BF107199           |          BQ958150             |
|                       |           BE935094           |          CB235934             |
|                       |           BF791057           |          BB574741             |
|                       |           BE885607           |          AL362572             |
|                       |           BU570953           |          CB209409             |
|                       |           BM721541           |          BE847840             |
|                       |           AA133493           |          AA959077             |
|                       |           AA282670           |          CB272355             |
|                       |           BP303980           |          AI503727             |
|                       |           BG026053           |          CF750034             |
|                       |           BQ224557           |          CK788427             |
|                       |           AW859310           |          BE859888             |
|                       |           BG506176           |          AA547180             |
|                       |           BE717356           |          CF724608             |
|                       |           CN369212           |          CF748867             |
|                       |           BM855146           |          BG142562             |
|                       |             C04032           |          BG142540             |
|                       |           BF240258           |          CB519172             |
|                       |           BG170796           |          BG142547             |
|                       |           BF700553           |          AL364148             |
|                       |           AL036762           |          CF731253             |
|                       |           BE537208           |          CD807151             |
|                       |           BM693350           |          CF104719             |
|                       |           CN369216           |          BF300989             |
|                       |           BE074161           |          CK621587             |
|                       |           BM563477           |          CD349436             |
|                       |           BE973777           |          BQ178932             |
|                       |           AW602749           |          BE291364             |
|                       |           BE714610           |          BF163307             |
|                       |           BF239946           |          CF948699             |
|                       |           AA282431           |          CK634421             |
|                       |           BM722606           |          BE571875             |
|                       |           AL042684           |          CK637878             |
|                       |           CR603582           |          BE573775             |
|                       |             H04569           |          BF119871             |
|                       |           BF673217           |          CK634506             |
|                       |           CD641443           |          CN526442             |
|                       |           BU602857           |          BF165583             |
|                       |           BQ687143           |          BP428083             |
|                       |           BM550409           |          AA755255             |
|                       |           BF038331           |          BQ923074             |
|                       |           BP370649           |          BG245833             |
|                       |           BP370491           |          BE626675             |
|                       |           BU189364           |          BI987845             |
|                       |           AW351938           |          AI593662             |
|                       |             W32045           |          BB322218             |
|                       |           AW351916           |          CK621054             |
|                       |           AW965561           |          BF101596             |
|                       |           CD171621           |          BQ477623             |
|                       |           AA057844           |          BI989489             |
|                       |           CB157780           |                               |
|                       |           BP369549           |                               |
|                       |           BG289031           |                               |
|                       |           BX451916           |                               |
|                       |           BP206672           |                               |
|                       |             R07145           |                               |
|                       |             R07277           |                               |
|                       |             R05322           |                               |
|                       |             H05204           |                               |
|                       |           BF680857           |                               |
|                       |           BU570075           |                               |
|                       |           BX451917           |                               |
|                       |           AU136604           |                               |
|                       |           BF990803           |                               |
|                       |           CB144138           |                               |
|                       |           CB111647           |                               |
|                       |             T80008           |                               |
|                       |             T08366           |                               |
|                       |           BU191284           |                               |
|                       |           CF595487           |                               |
|                       |           BP260082           |                               |
|                       |             R12612           |                               |
|                       |           BQ688440           |                               |
|                       |           BE622173           |                               |
|                       |           BP380109           |                               |
|                       |           AU138230           |                               |
|                       |           AW406889           |                               |
|                       |             Z42544           |                               |
|                       |           CB107465           |                               |
|                       |           BM785578           |                               |
|                       |           BM741691           |                               |
|                       |           CN369214           |                               |
|                       |           BG258285           |                               |
|                       |           BP243118           |                               |
|                       |           BM475745           |                               |
|                       |           CF144478           |                               |
|                       |           AW401889           |                               |
|                       |           BX955688           |                               |
|                       |             R21388           |                               |
|                       |           AA010024           |                               |
|                       |           AW965336           |                               |
|                       |           BX955683           |                               |
|                       |           BF326556           |                               |
|                       |           BM837767           |                               |
|                       |           BI333322           |                               |
|                       |           BE078456           |                               |
|                       |             W40351           |                               |
|                       |           CD244625           |                               |
|                       |           BF693365           |                               |
|                       |           BE545050           |                               |
|                       |           AU129139           |                               |
|                       |           AW296301           |                               |
|                       |             X85578           |                               |
|                       |             Z21352           |                               |
|                       |           BP239003           |                               |
|                       |           BP239480           |                               |
|                       |             R94796           |                               |
|                       |           CB154719           |                               |
|                       |           BP207691           |                               |
|                       |           BP297196           |                               |
|                       |           AA333182           |                               |
|                       |           BF984683           |                               |
|                       |           AU133105           |                               |
|                       |           BX440746           |                               |
|                       |           BP236695           |                               |
|                       |           BP348476           |                               |
|                       |           AL041556           |                               |
|                       |           AV656115           |                               |
|                       |           BM452307           |                               |
|                       |           BP222471           |                               |
|                       |           BU665270           |                               |
|                       |           BP351700           |                               |
|                       |           CF551863           |                               |
|                       |           BX956353           |                               |
|                       |           BP355008           |                               |
|                       |           AL519157           |                               |
|                       |           BU189717           |                               |
|                       |           AA489429           |                               |
|                       |           AA307541           |                               |
|                       |           AW327363           |                               |
+-----------------------+------------------------------+-------------------------------+
|      #   73           | *Seq. mapped in strand Plus* | *Seq. mapped in strand Plus*  |
|                       |           AB033023           |          BU055428             |
|                       |           AK023370           |          BY320647             |
|                       |           BC043393           |          BY063894             |
|                       |           BC082270           |          BB859376             |
|                       |           BC032368           |          BY734119             |
|                       |           AK022903           |          BU056288             |
|                       |           CR605431           |          BU058104             |
|                       |                              |          BY780850             |
|                       |                              |          BB615513             |
|                       |                              |          CA751431             |
|                       |                              |          BY713303             |
|                       |                              |          BB857521             |
|                       |                              |          BB853680             |
|                       |                              |          CN716826             |
|                       |                              |          CN715185             |
|                       |                              |          CN716825             |
|                       |                              |          CN720540             |
|                       |                              |          CN725438             |
|                       |                              |          CN723808             |
|                       |                              |          CN720641             |
|                       |                              |          CN664078             |
|                       |                              |          CK781300             |
|                       |                              |          CA328328             |
|                       |                              |          BC057045             |
|                       |                              |          CN530723             |
|                       |                              |          BU055609             |
|                       |                              |          BU704237             |
|                       |                              |          CF747124             |
|                       |                              |          CK636094             |
|                       |                              |          BI658633             |
|                       |                              |          CN454768             |
|                       |                              |          CK635760             |
|                       |                              |          BF320677             |
|                       |                              |          CF538542             |
|                       |                              |          BQ180376             |
|                       |                              |          CA750981             |
|                       |                              |          CF533954             |
|                       |                              |          CF539058             |
|                       |                              |          CN669787             |
|                       |                              |          CF746827             |
|                       |                              |          CB589448             |
|                       |                              |          CN533090             |
|                       |                              |          CA566661             |
|                       |                              |          CF535012             |
|                       |                              |          CF535024             |
|                       |                              |          CF536383             |
|                       |                              |          CA527536             |
|                       |                              |          CF905528             |
|                       |                              |          CN535604             |
|                       |                              |          BU706394             |
|                       |                              |          CD540255             |
|                       |                              |          CK633846             |
|                       |                              |          CN667245             |
|                       |                              |          CN535845             |
|                       |                              |          CN536783             |
|                       |                              |          CB527452             |
|                       |                              |          CN680390             |
|                       |                              |          BQ714659             |
|                       |                              |          CN533363             |
|                       |                              |          CF734340             |
|                       |                              |          CF534151             |
|                       |                              |          CK639143             |
|                       |                              |          CF533281             |
|                       |                              |          CN461226             |
|                       |                              |          CB246490             |
|                       |                              |          CA324850             |
|                       |                              |          CN535253             |
|                       |                              |          CN526821             |
|                       |                              |          CF164426             |
|                       |                              |          CB519429             |
|                       |                              |          BE376973             |
|                       |                              |          CF727830             |
|                       |                              |          CK637130             |
|                       |                              |          CD543521             |
|                       |                              |          CB249038             |
|                       |                              |          CN461534             |
|                       |                              |          AW911206             |
|                       |                              |          CN528558             |
|                       |                              |          CN718509             |
|                       |                              |          BC042768             |
|                       |                              |          BE290969             |
|                       |                              |          CD550173             |
|                       |                              |          BF169010             |
|                       |                              |          AI616218             |
|                       |                              |          AA692675             |
|                       |                              |          BM454169             |
|                       |                              |          CD553049             |
|                       |                              |          CF948550             |
|                       |                              |          CB205307             |
|                       |                              |          CD804903             |
|                       |                              |          AW258486             |
|                       |                              |          BX522690             |
|                       |                              |          CF749470             |
|                       |                              |          BY220631             |
|                       |                              |          BY211042             |
|                       |                              |          CN696770             |
|                       |                              |          BU694873             |
|                       |                              |          CK333118             |
|                       |                              |          CA534901             |
|                       |                              |          CA543115             |
|                       | *Seq. mapped in strand Minus*| *Seq. mapped in strand Minus* |
|                       |           AK090720           |          BQ174174             |
|                       |                              |          CF578937             |
|                       |                              |                               |
|                       |                              |                               |
|                       |                              |                               |
|                       |                              |                               |
|                       |                              |                               |
|                       |                              |                               |
|                       |                              |                               |
|                       |                              |                               |
|                       |                              |                               |
|                       |                              |                               |
|                       |                              |                               |
|                       |                              |                               |
|                       |                              |                               |
|                       |                              |                               |
|                       |                              |                               |
|                       |                              |                               |
|                       |                              |                               |
|                       |                              |                               |
|                       |                              |                               |
|                       |                              |                               |
|                       |                              |                               |
|                       |                              |                               |
|                       |                              |                               |
|                       |                              |                               |
|                       |                              |                               |
|                       |                              |                               |
|                       |                              |                               |
|                       |                              |                               |
|                       |                              |                               |
|                       |                              |                               |
|                       |                              |                               |
|                       |                              |                               |
|                       |                              |                               |
|                       |                              |                               |
|                       |                              |                               |
|                       |                              |                               |
|                       |                              |                               |
|                       |                              |                               |
|                       |                              |                               |
|                       |                              |                               |
|                       |                              |                               |
|                       |                              |                               |
|                       |                              |                               |
|                       |                              |                               |
|                       |                              |                               |
|                       |                              |                               |
|                       |                              |                               |
|                       |                              |                               |
|                       |                              |                               |
|                       |                              |                               |
|                       |                              |                               |
|                       |                              |                               |
|                       |                              |                               |
|                       |                              |                               |
|                       |                              |                               |
|                       |                              |                               |
|                       |                              |                               |
|                       |                              |                               |
|                       |                              |                               |
|                       |                              |                               |
|                       |                              |                               |
|                       |                              |                               |
|                       |                              |                               |
|                       |                              |                               |
|                       |                              |                               |
|                       |                              |                               |
|                       |                              |                               |
|                       |                              |                               |
|                       |                              |                               |
|                       |                              |                               |
|                       |                              |                               |
|                       |                              |                               |
|                       |                              |                               |
|                       |                              |                               |
|                       |                              |                               |
|                       |                              |                               |
|                       |                              |                               |
|                       |                              |                               |
|                       |                              |                               |
|                       |                              |                               |
|                       |                              |                               |
|                       |                              |                               |
|                       |                              |                               |
|                       |                              |                               |
|                       |                              |                               |
|                       |                              |                               |
|                       |                              |                               |
|                       |                              |                               |
|                       |                              |                               |
|                       |                              |                               |
|                       |                              |                               |
|                       |                              |                               |
|                       |                              |                               |
|                       |                              |                               |
|                       |                              |                               |
|                       |                              |                               |
|                       |                              |                               |
|                       |                              |                               |
+-----------------------+------------------------------+-------------------------------+
|      #   74           | *Seq. mapped in strand Plus* | *Seq. mapped in strand Plus*  |
|                       |           AK126579           |          AV322197             |
|                       |           AL833031           |                               |
|                       | *Seq. mapped in strand Minus*| *Seq. mapped in strand Minus* |
|                       |           CR590857           |          BE691681             |
+-----------------------+------------------------------+-------------------------------+
|      #   75           | *Seq. mapped in strand Plus* | *Seq. mapped in strand Plus*  |
|                       |           BX647755           |          AY282793             |
|                       |           BC035031           |            M74515             |
|                       |             D13318           |          BC052448             |
|                       |             U13044           |          AY282794             |
|                       |                              |          AY282795             |
|                       |                              |          BC013562             |
|                       |                              |          AY282796             |
|                       |                              |          AY282797             |
|                       |                              |          AY282798             |
|                       |                              |          AY282799             |
|                       | *Seq. mapped in strand Minus*| *Seq. mapped in strand Minus* |
|                       |             M73031           |            U77128             |
|                       |             M37104           |          BC010766             |
|                       |           AL110183           |          AK078484             |
|                       |           BC066310           |                               |
|                       |           BC001178           |                               |
|                       |           CR456950           |                               |
|                       |           BT007244           |                               |
+-----------------------+------------------------------+-------------------------------+
|      #   76           | *Seq. mapped in strand Plus* | *Seq. mapped in strand Plus*  |
|                       |           BC020541           |          BE981002             |
|                       |           BE999934           |                               |
|                       |           AA742189           |                               |
|                       |           BM976847           |                               |
|                       |           CK819823           |                               |
|                       |           BE043949           |                               |
|                       |           BE222094           |                               |
|                       |           BX098064           |                               |
|                       |           AW675817           |                               |
|                       |           BM141730           |                               |
|                       |           BE049548           |                               |
|                       |           BG370205           |                               |
|                       |           AI681849           |                               |
|                       |           AI824563           |                               |
|                       | *Seq. mapped in strand Minus*| *Seq. mapped in strand Minus* |
|                       |           BG182298           |          AK033427             |
+-----------------------+------------------------------+-------------------------------+
|      #   77           | *Seq. mapped in strand Plus* | *Seq. mapped in strand Plus*  |
|                       |           BC030011           |          AK005235             |
|                       |           AF052188           |          BY705414             |
|                       |           AB018278           |          BB627751             |
|                       |           CR457086           |          AK035406             |
|                       | *Seq. mapped in strand Minus*| *Seq. mapped in strand Minus* |
|                       |           AL110286           |          AK035927             |
|                       |                              |          BI735056             |
|                       |                              |          BI737217             |
|                       |                              |          BB641657             |
+-----------------------+------------------------------+-------------------------------+
|      #   78           | *Seq. mapped in strand Plus* | *Seq. mapped in strand Plus*  |
|                       |           AK127284           |          AK011114             |
|                       |           BC038439           |          BY018506             |
|                       |                              |          AK042884             |
|                       |                              |          BB640108             |
|                       |                              |          BY238248             |
|                       |                              |          BY715671             |
|                       |                              |          AK016073             |
|                       |                              |          BI985607             |
|                       |                              |          BY293087             |
|                       |                              |          BU938944             |
|                       |                              |          BB572606             |
|                       |                              |          CA328451             |
|                       |                              |          BB249737             |
|                       |                              |          AA183671             |
|                       |                              |          AI643091             |
|                       |                              |          BB823587             |
|                       |                              |          AV108785             |
|                       | *Seq. mapped in strand Minus*| *Seq. mapped in strand Minus* |
|                       |           AY358949           |          BI554642             |
|                       |           BC071616           |          BI100630             |
|                       |           BC064948           |          BE648009             |
|                       |           CR623218           |          BF786957             |
|                       |           CR615959           |          CK788466             |
|                       |                              |          AI607349             |
|                       |                              |          BE953790             |
|                       |                              |          AA065945             |
|                       |                              |          BI331369             |
|                       |                              |          CA468336             |
|                       |                              |          CB949107             |
|                       |                              |          BF533815             |
|                       |                              |          CF755020             |
|                       |                              |          AI835706             |
|                       |                              |          BF455504             |
|                       |                              |          AA717610             |
|                       |                              |          BF232678             |
+-----------------------+------------------------------+-------------------------------+
|      #   79           | *Seq. mapped in strand Plus* | *Seq. mapped in strand Plus*  |
|                       |           BC041961           |          BB649073             |
|                       |           AK094650           |          AK081622             |
|                       |           AL834454           |          AK034391             |
|                       |           AB056652           |          BB623023             |
|                       |           AK094191           |          BB641342             |
|                       |                              |          AK043673             |
|                       |                              |          AK039555             |
|                       |                              |          BB190361             |
|                       |                              |          BB633558             |
|                       |                              |          BB628810             |
|                       |                              |          BY731699             |
|                       |                              |          BY238389             |
|                       |                              |          BF471454             |
|                       |                              |          BY080299             |
|                       |                              |          BY728140             |
|                       |                              |          AK081222             |
|                       | *Seq. mapped in strand Minus*| *Seq. mapped in strand Minus* |
|                       |           AK092549           |          BB641842             |
|                       |           BC041480           |          BB580431             |
+-----------------------+------------------------------+-------------------------------+
|      #   80           | *Seq. mapped in strand Plus* | *Seq. mapped in strand Plus*  |
|                       |           BX648515           |          BC069971             |
|                       |           BC063117           |                               |
|                       |           BX647785           |                               |
|                       |           AL137456           |                               |
|                       |           AB002380           |                               |
|                       | *Seq. mapped in strand Minus*| *Seq. mapped in strand Minus* |
|                       |           AL833074           |          AF467766             |
+-----------------------+------------------------------+-------------------------------+
|      #   81           | *Seq. mapped in strand Plus* | *Seq. mapped in strand Plus*  |
|                       |           BM683553           |          AV309996             |
|                       |                              |          BY340960             |
|                       |                              |          BY093147             |
|                       |                              |          BY339914             |
|                       |                              |          BY726224             |
|                       |                              |          BB855856             |
|                       |                              |          AK017655             |
|                       |                              |          BY717436             |
|                       |                              |          CF743587             |
|                       |                              |          AA104693             |
|                       |                              |          CF532238             |
|                       |                              |          CA480920             |
|                       |                              |          BY263691             |
|                       |                              |          AK007781             |
|                       |                              |          AK010539             |
|                       |                              |          BY707791             |
|                       |                              |          BY709955             |
|                       |                              |          BM938577             |
|                       |                              |          BY104563             |
|                       |                              |          BY097311             |
|                       |                              |          CB570836             |
|                       |                              |          BE986604             |
|                       |                              |          CF750685             |
|                       |                              |          BG861827             |
|                       |                              |          BY781199             |
|                       |                              |          BY781288             |
|                       |                              |          BY735193             |
|                       |                              |          AA087517             |
|                       |                              |          AA919737             |
|                       |                              |          BQ256961             |
|                       |                              |          BQ918276             |
|                       |                              |          BG142991             |
|                       |                              |          BU510286             |
|                       |                              |          BU841064             |
|                       |                              |          BU704217             |
|                       |                              |          CD561921             |
|                       |                              |          BC055875             |
|                       |                              |          CF182197             |
|                       |                              |          BC019194             |
|                       |                              |          BY775093             |
|                       |                              |          BU514740             |
|                       |                              |          BE911270             |
|                       |                              |          BQ714929             |
|                       |                              |          BQ714915             |
|                       |                              |          CF616827             |
|                       |                              |          CN539415             |
|                       |                              |          BI986341             |
|                       |                              |          BI077528             |
|                       |                              |          BC034135             |
|                       |                              |          BG804364             |
|                       |                              |          BF121706             |
|                       |                              |          BU056730             |
|                       |                              |          CD805400             |
|                       |                              |          BQ944292             |
|                       |                              |          BM963718             |
|                       |                              |          CF617694             |
|                       |                              |          CN525057             |
|                       |                              |          CN682349             |
|                       |                              |          CB249933             |
|                       |                              |          BQ961031             |
|                       |                              |          BQ572015             |
|                       |                              |          BF181134             |
|                       |                              |          CN683298             |
|                       |                              |          BY648790             |
|                       |                              |          BB682918             |
|                       |                              |          AI450249             |
|                       |                              |          BB684973             |
|                       |                              |          CN688699             |
|                       |                              |          AW761910             |
|                       |                              |          CN688707             |
|                       |                              |          CF534469             |
|                       |                              |          CF532785             |
|                       |                              |          CA328541             |
|                       |                              |          CD355186             |
|                       |                              |          BB459310             |
|                       |                              |          CF951283             |
|                       |                              |          AV326197             |
|                       |                              |          BE306762             |
|                       |                              |          CB521451             |
|                       |                              |          BI249891             |
|                       |                              |          BQ840440             |
|                       |                              |          CF540108             |
|                       |                              |          BG975423             |
|                       |                              |          CF532628             |
|                       |                              |          CF540021             |
|                       |                              |          CF532485             |
|                       |                              |          BF470510             |
|                       |                              |          BF470303             |
|                       |                              |          AI153366             |
|                       |                              |          BU611574             |
|                       |                              |          BM570550             |
|                       |                              |          BB675981             |
|                       |                              |          BY623457             |
|                       |                              |          AW495576             |
|                       |                              |          BM570794             |
|                       | *Seq. mapped in strand Minus*| *Seq. mapped in strand Minus* |
|                       |           BC065198           |          CF354038             |
|                       |           AW193381           |                               |
|                       |             N29757           |                               |
|                       |           AI858366           |                               |
|                       |           AU146348           |                               |
|                       |           AJ404326           |                               |
|                       |           AK001093           |                               |
|                       |           AY005111           |                               |
|                       |           AI334115           |                               |
|                       |           AI695078           |                               |
|                       |           AW129550           |                               |
|                       |           AI912668           |                               |
|                       |           CR617017           |                               |
|                       |           AI049518           |                               |
|                       |           AW304450           |                               |
|                       |           BU728025           |                               |
|                       |           AW001661           |                               |
|                       |           AI858321           |                               |
|                       |           AI694936           |                               |
|                       |           AW050490           |                               |
|                       |           CD678499           |                               |
|                       |           BQ002571           |                               |
|                       |           BM978424           |                               |
|                       |           CA446711           |                               |
|                       |           BM977168           |                               |
|                       |           BX647161           |                               |
|                       |           AI570098           |                               |
|                       |           AA621718           |                               |
|                       |           BX455019           |                               |
|                       |           CR610923           |                               |
|                       |           AW027308           |                               |
|                       |           BM671561           |                               |
|                       |           BE138732           |                               |
|                       |           AI077454           |                               |
|                       |           AL521879           |                               |
|                       |           AA843214           |                               |
|                       |           CR622525           |                               |
|                       |           CR590928           |                               |
|                       |           AW162941           |                               |
|                       |           BX423310           |                               |
|                       |           BX406354           |                               |
|                       |           AL578205           |                               |
|                       |           AL528488           |                               |
|                       |           CR601233           |                               |
|                       |           BM710136           |                               |
|                       |           AL521249           |                               |
|                       |           BF768957           |                               |
|                       |           BP376650           |                               |
|                       |           BQ315163           |                               |
|                       |           AI241828           |                               |
|                       |           AA613804           |                               |
|                       |           AI810340           |                               |
|                       |           AK097974           |                               |
|                       |           BM686305           |                               |
|                       |           AW780317           |                               |
|                       |           AW386240           |                               |
|                       |           BQ361044           |                               |
|                       |           BG959292           |                               |
|                       |           BX333093           |                               |
|                       |           BF878173           |                               |
|                       |           AA317465           |                               |
|                       |           AA143197           |                               |
|                       |           BF969645           |                               |
|                       |           BF768780           |                               |
|                       |           BF768970           |                               |
|                       |           BF768915           |                               |
|                       |           BF836601           |                               |
|                       |           BG959395           |                               |
|                       |           BM686667           |                               |
|                       |           AW731862           |                               |
|                       |           BU730481           |                               |
|                       |           AV743568           |                               |
|                       |           AI244540           |                               |
|                       |           BE301744           |                               |
|                       |           BQ028177           |                               |
|                       |           BM666366           |                               |
|                       |           BU620144           |                               |
|                       |           BC003194           |                               |
|                       |           AL565161           |                               |
|                       |           CR590109           |                               |
|                       |           CK429311           |                               |
|                       |           BM695189           |                               |
|                       |           BM704846           |                               |
|                       |           BE018266           |                               |
|                       |           CA488581           |                               |
|                       |           BI763612           |                               |
|                       |           BE179035           |                               |
|                       |           AL528519           |                               |
|                       |           AL533556           |                               |
|                       |           BE179217           |                               |
|                       |           CN386977           |                               |
|                       |           AA143213           |                               |
|                       |           CD628888           |                               |
|                       |           CD628890           |                               |
|                       |           CD628891           |                               |
|                       |           CD628892           |                               |
|                       |           CD628889           |                               |
|                       |           BM468114           |                               |
|                       |           CD628887           |                               |
|                       |           BE277263           |                               |
|                       |           BF339523           |                               |
|                       |           AL583486           |                               |
|                       |           BI760587           |                               |
|                       |           BF341655           |                               |
|                       |           CN337831           |                               |
|                       |           BG281482           |                               |
|                       |           BG109686           |                               |
|                       |           CB123105           |                               |
|                       |           BE796292           |                               |
|                       |           CN386978           |                               |
|                       |           AW373527           |                               |
|                       |           BE384607           |                               |
|                       |           BE793205           |                               |
|                       |           BI055908           |                               |
|                       |           BQ230333           |                               |
|                       |           BE799022           |                               |
|                       |           BF125290           |                               |
|                       |           BM545792           |                               |
|                       |           BX423353           |                               |
|                       |           BX460349           |                               |
|                       |           AL555791           |                               |
|                       |           AL521880           |                               |
|                       |           BG701003           |                               |
|                       |           CB267106           |                               |
|                       |           BE793076           |                               |
|                       |           BI522545           |                               |
|                       |           AU120222           |                               |
|                       |           BF686701           |                               |
|                       |           BE792421           |                               |
|                       |           BM016243           |                               |
|                       |           CN386979           |                               |
|                       |           CN386981           |                               |
|                       |           BE142633           |                               |
|                       |           BU152950           |                               |
|                       |           CV025882           |                               |
|                       |           CA943235           |                               |
|                       |           BE254123           |                               |
|                       |           BE255283           |                               |
|                       |           BF686773           |                               |
|                       |           BE266357           |                               |
|                       |           BP329866           |                               |
|                       |           BP271862           |                               |
|                       |           BM016330           |                               |
|                       |           CD514016           |                               |
|                       |           CD252044           |                               |
|                       |           BP194581           |                               |
|                       |           BE383983           |                               |
|                       |           BE384664           |                               |
|                       |           BF224277           |                               |
|                       |           BE263438           |                               |
|                       |           BG832051           |                               |
|                       |           AI744735           |                               |
|                       |           AW449867           |                               |
|                       |           BQ637652           |                               |
|                       |           CB128807           |                               |
|                       |           BF568296           |                               |
|                       |           BP247317           |                               |
|                       |           BP219381           |                               |
|                       |           BP247318           |                               |
|                       |           BP248512           |                               |
|                       |           BG330114           |                               |
|                       |           BP193095           |                               |
|                       |           BG337132           |                               |
|                       |           CA407834           |                               |
|                       |           BP263972           |                               |
|                       |           BM006229           |                               |
|                       |           CB156392           |                               |
|                       |           BP247352           |                               |
|                       |           CB116893           |                               |
|                       |           AW813330           |                               |
|                       |           BF375783           |                               |
|                       |           BM750408           |                               |
|                       |           BF343123           |                               |
|                       |           BF339027           |                               |
|                       |           AW898149           |                               |
|                       |           BP247353           |                               |
|                       |           AA730774           |                               |
|                       |           CN337832           |                               |
|                       |           CN386980           |                               |
|                       |           BQ072433           |                               |
|                       |           AW749444           |                               |
|                       |           AW890360           |                               |
|                       |           CN302315           |                               |
|                       |           BM714908           |                               |
|                       |           BF086451           |                               |
|                       |           AA352322           |                               |
|                       |           BF882409           |                               |
|                       |             H24668           |                               |
|                       |             Z84716           |                               |
|                       |           BI462499           |                               |
|                       |           BF516065           |                               |
|                       |           BF514039           |                               |
|                       |           CK430073           |                               |
|                       |           BM822085           |                               |
|                       |           BE143941           |                               |
|                       |           BE143995           |                               |
+-----------------------+------------------------------+-------------------------------+
|      #   82           | *Seq. mapped in strand Plus* | *Seq. mapped in strand Plus*  |
|                       |             Z26653           |          AK016323             |
|                       |             M59832           |                               |
|                       |           CR610868           |                               |
|                       |           AK125980           |                               |
|                       |           AK026829           |                               |
|                       | *Seq. mapped in strand Minus*| *Seq. mapped in strand Minus* |
|                       |           BC035400           |          AK076479             |
+-----------------------+------------------------------+-------------------------------+
|      #   83           | *Seq. mapped in strand Plus* | *Seq. mapped in strand Plus*  |
|                       |           BF364718           |          AY348866             |
|                       |                              |          BC059811             |
|                       |                              |          AK032023             |
|                       |                              |          AK049830             |
|                       | *Seq. mapped in strand Minus*| *Seq. mapped in strand Minus* |
|                       |           AK122849           |          AK016607             |
|                       |           CK821547           |                               |
|                       |           BM353955           |                               |
|                       |           AK093115           |                               |
|                       |           BC032954           |                               |
|                       |           AF086924           |                               |
|                       |           AI417254           |                               |
|                       |           AF145027           |                               |
|                       |           CA843584           |                               |
|                       |           BM353672           |                               |
|                       |           AI857659           |                               |
|                       |           BM707436           |                               |
|                       |           BE467683           |                               |
|                       |           BE703666           |                               |
|                       |           BE703621           |                               |
|                       |           BE703750           |                               |
|                       |           AA971842           |                               |
|                       |           BE703713           |                               |
|                       |           BE703545           |                               |
|                       |           BE703743           |                               |
|                       |           BE703712           |                               |
|                       |           BE670585           |                               |
|                       |           AI869282           |                               |
|                       |           BE703825           |                               |
|                       |           AI123878           |                               |
|                       |           AI199739           |                               |
|                       |           AI912114           |                               |
|                       |           AI869291           |                               |
|                       |           BE703824           |                               |
|                       |           BF196095           |                               |
|                       |             R84971           |                               |
|                       |             H50243           |                               |
|                       |             H50276           |                               |
|                       |           CK821548           |                               |
|                       |           AW002102           |                               |
|                       |           AI498989           |                               |
|                       |             H51599           |                               |
|                       |           BE857530           |                               |
|                       |           BF941030           |                               |
|                       |           AI306573           |                               |
|                       |           AA733098           |                               |
|                       |           AA985171           |                               |
|                       |           AL118589           |                               |
|                       |           BE703710           |                               |
|                       |           BE703858           |                               |
|                       |           BE703784           |                               |
|                       |           BE703725           |                               |
|                       |           BE703775           |                               |
|                       |           BF364717           |                               |
|                       |           BE703547           |                               |
|                       |           BE703555           |                               |
|                       |           BE703556           |                               |
|                       |           BE703559           |                               |
|                       |           BE703593           |                               |
|                       |           BE703628           |                               |
|                       |           BE703630           |                               |
|                       |           BE703631           |                               |
|                       |           BE703632           |                               |
|                       |           BE703657           |                               |
|                       |           BE703667           |                               |
|                       |           BE703669           |                               |
|                       |           BE703671           |                               |
|                       |           BE703700           |                               |
|                       |           BE703708           |                               |
|                       |           BE703711           |                               |
|                       |           BE703724           |                               |
|                       |           BE703738           |                               |
|                       |           BE703741           |                               |
|                       |           BE703744           |                               |
|                       |           BE703749           |                               |
|                       |           BE703751           |                               |
|                       |           BE703756           |                               |
|                       |           BE703761           |                               |
|                       |           BE703764           |                               |
|                       |           BE703778           |                               |
|                       |           BE703779           |                               |
|                       |           BE703783           |                               |
|                       |           BE703785           |                               |
|                       |           BE703790           |                               |
|                       |           BE703791           |                               |
|                       |           BE703795           |                               |
|                       |           BE703860           |                               |
|                       |           BE703867           |                               |
|                       |           BE703869           |                               |
|                       |           BE703870           |                               |
|                       |           BE703881           |                               |
|                       |           BF364724           |                               |
|                       |           BE703746           |                               |
|                       |           BF224166           |                               |
|                       |           BE818769           |                               |
|                       |           BF364726           |                               |
|                       |           BE703622           |                               |
|                       |           AV724369           |                               |
|                       |           BE703788           |                               |
|                       |           AW973006           |                               |
|                       |           CF454884           |                               |
|                       |           BI828080           |                               |
|                       |             H45299           |                               |
|                       |           AI078291           |                               |
|                       |           AI351312           |                               |
|                       |           AI932273           |                               |
|                       |           AA911147           |                               |
|                       |           AA570652           |                               |
|                       |           AI272056           |                               |
|                       |           AI523680           |                               |
|                       |             N40919           |                               |
|                       |           AW043652           |                               |
|                       |           AW070378           |                               |
|                       |           AW166283           |                               |
|                       |             H50302           |                               |
|                       |           AI220464           |                               |
|                       |           BI462012           |                               |
|                       |           CV024432           |                               |
|                       |           BI547024           |                               |
|                       |           AA101298           |                               |
|                       |           BX097255           |                               |
|                       |           BM689787           |                               |
|                       |             M78063           |                               |
|                       |           BG724433           |                               |
|                       |           CN431219           |                               |
|                       |           AI560292           |                               |
|                       |           AA340759           |                               |
|                       |           AA340760           |                               |
+-----------------------+------------------------------+-------------------------------+
|      #   84           | *Seq. mapped in strand Plus* | *Seq. mapped in strand Plus*  |
|                       |           BC068292           |          AK048242             |
|                       |           AB004066           |          AK090032             |
|                       |           BC082238           |          BC010720             |
|                       |           CR456921           |          AF010305             |
|                       |                              |          AF364051             |
|                       |                              |            Y07836             |
|                       | *Seq. mapped in strand Minus*| *Seq. mapped in strand Minus* |
|                       |           AK056892           |          AK039720             |
|                       |                              |          AK002866             |
|                       |                              |                               |
|                       |                              |                               |
|                       |                              |                               |
|                       |                              |                               |
+-----------------------+------------------------------+-------------------------------+
|      #   85           | *Seq. mapped in strand Plus* | *Seq. mapped in strand Plus*  |
|                       |           AL831946           |          BC010835             |
|                       |                              |          AF176814             |
|                       |                              |          AK005255             |
|                       | *Seq. mapped in strand Minus*| *Seq. mapped in strand Minus* |
|                       |           BC007298           |          AK029840             |
|                       |           BC009396           |          BC050817             |
|                       |           AF007151           |          AK028317             |
|                       |           AK025496           |          AK011754             |
|                       |           BC002692           |          AK030619             |
|                       |           AK056244           |          BC018614             |
|                       |           BC006575           |          AF319949             |
|                       |           AK056581           |          AK036983             |
|                       |           AK027710           |                               |
|                       |           BC033101           |                               |
|                       |           BC080532           |                               |
|                       |           AF357881           |                               |
|                       |           AF319947           |                               |
|                       |           CR615346           |                               |
|                       |           AJ306408           |                               |
+-----------------------+------------------------------+-------------------------------+
|      #   86           | *Seq. mapped in strand Plus* | *Seq. mapped in strand Plus*  |
|                       |           BF515241           |          CF736206             |
|                       | *Seq. mapped in strand Minus*| *Seq. mapped in strand Minus* |
|                       |           AK131221           |          AK033328             |
+-----------------------+------------------------------+-------------------------------+
|      #   87           | *Seq. mapped in strand Plus* | *Seq. mapped in strand Plus*  |
|                       |           AF009745           |          BY777853             |
|                       |           AF071106           |          BY718197             |
|                       |           AF071107           |          AK018077             |
|                       |           AF071108           |          BB665534             |
|                       |           CR749366           |            U58993             |
|                       |           CR749473           |          AF063006             |
|                       |           AF071109           |          CN680262             |
|                       |           AF071110           |          BY781589             |
|                       |           BC009682           |          BY132441             |
|                       |             U73825           |          BY131678             |
|                       |           AF010601           |          BY725725             |
|                       |             U59913           |          CD351593             |
|                       |           AF009678           |          BQ957892             |
|                       |           CR457424           |          CN669482             |
|                       |                              |            U77638             |
|                       |                              |          CD351828             |
|                       |                              |          BY228670             |
|                       |                              |          AK082997             |
|                       |                              |          BY729538             |
|                       |                              |          AW258989             |
|                       |                              |          BC048233             |
|                       |                              |          BC050001             |
|                       |                              |          BU919274             |
|                       |                              |          CN532942             |
|                       |                              |          BI695413             |
|                       |                              |          AW260284             |
|                       |                              |          CB246662             |
|                       |                              |          BU704014             |
|                       |                              |          CF745173             |
|                       |                              |          BY584514             |
|                       |                              |          CD803563             |
|                       |                              |          BU704204             |
|                       |                              |          CD298517             |
|                       |                              |          AI326624             |
|                       |                              |          AA088132             |
|                       |                              |          AI573395             |
|                       |                              |          BE532435             |
|                       |                              |          BU055381             |
|                       |                              |          BC050130             |
|                       |                              |          BC057286             |
|                       |                              |          AW488600             |
|                       |                              |          AW487938             |
|                       |                              |          BE852925             |
|                       | *Seq. mapped in strand Minus*| *Seq. mapped in strand Minus* |
|                       |           AF086556           |          CF744983             |
|                       |           AF071111           |                               |
+-----------------------+------------------------------+-------------------------------+
|      #   88           | *Seq. mapped in strand Plus* | *Seq. mapped in strand Plus*  |
|                       |           BM681969           |          BE983506             |
|                       | *Seq. mapped in strand Minus*| *Seq. mapped in strand Minus* |
|                       |           AI912528           |          AK003440             |
|                       |           AI493314           |          AK088946             |
|                       |           AI367889           |          AK014483             |
|                       |           BM545914           |          AK032586             |
|                       |           BC050742           |          BC005728             |
|                       |           BE328018           |          BG920124             |
|                       |           BQ016172           |          CK019690             |
|                       |           AW573055           |          AK018514             |
|                       |           BF940801           |          BG518536             |
|                       |           BM789727           |          BG518704             |
|                       |           AI282951           |          CB246842             |
|                       |           BE886323           |          AV299459             |
|                       |           AI753612           |          AK077604             |
|                       |           BU624797           |          BE988160             |
|                       |             U04209           |          CK030556             |
|                       |           BC023557           |          BE137857             |
|                       |           BG057047           |          AA866870             |
|                       |           CA311063           |          BM949971             |
|                       |           BQ013684           |          BI736939             |
|                       |           BM662222           |          CA576599             |
|                       |           CR603002           |          CA573638             |
|                       |           BG200436           |          CA548094             |
|                       |           AL514367           |          AA914773             |
|                       |           BM992417           |          AW913504             |
|                       |           BI962792           |          CF157681             |
|                       |           AI612906           |          CF159703             |
|                       |           AI583016           |          CD808215             |
|                       |           AI215563           |          AI527607             |
|                       |           BU687875           |          CF157459             |
|                       |           AI370295           |          CF159575             |
|                       |           AL565099           |          CA548900             |
|                       |           CR600815           |          CB845280             |
|                       |           BX420434           |          CA549380             |
|                       |           CB130041           |          BY081462             |
|                       |           AW575886           |          CF157426             |
|                       |           BX091435           |          CA553737             |
|                       |           AU134683           |          CD805426             |
|                       |           BM716875           |          CN672340             |
|                       |           AI916292           |          BG245529             |
|                       |           AA932419           |          CA750788             |
|                       |           AI304957           |          CK638493             |
|                       |           BM550744           |          CF174008             |
|                       |           BG106341           |          AL364227             |
|                       |             R01323           |          CF730219             |
|                       |           BM691576           |          BU505810             |
|                       |           AA436797           |          BF169049             |
|                       |           BP208769           |          BG972821             |
|                       |           BQ638299           |          BB619035             |
|                       |           BM853677           |          CF750677             |
|                       |           BM807546           |          BI688369             |
|                       |           AA318042           |          BI660892             |
|                       |           BX421919           |          CK796091             |
|                       |           BQ055022           |          BY117856             |
|                       |           CD688862           |          BI143780             |
|                       |           BU554087           |          BI143997             |
|                       |           BX433450           |          CK023496             |
|                       |           BQ641941           |          AI115205             |
|                       |           AL514368           |          AU080085             |
|                       |           BQ065228           |          BG862791             |
|                       |           BU161359           |          CF725568             |
|                       |           BM083453           |          CF906068             |
|                       |           BQ943643           |          CF894788             |
|                       |           AI828674           |          CB273803             |
|                       |           BM825239           |          CN683362             |
|                       |           BP203587           |          CA875215             |
|                       |           BE385535           |          CK624559             |
|                       |           BF095536           |          BY747001             |
|                       |           BX420435           |          BB654194             |
|                       |           BQ431998           |          BY747284             |
|                       |           BQ056767           |          CN685130             |
|                       |           CD050994           |          CB952238             |
|                       |           BF350485           |          BY720687             |
|                       |           AW382531           |          BI647054             |
|                       |           BQ944227           |          BB622985             |
|                       |           BQ649064           |          CN701799             |
|                       |           AL533163           |          CD547870             |
|                       |           AW865301           |          CF900472             |
|                       |           AU121948           |          CF162569             |
|                       |           BE937045           |          CN529201             |
|                       |           AW865372           |          BU562171             |
|                       |           AW865170           |          BB610005             |
|                       |           BI089933           |          CA569740             |
|                       |           CN294724           |          CN701657             |
|                       |           CB961765           |          CN672517             |
|                       |           BX424528           |          BB698494             |
|                       |           CB991265           |          BY711809             |
|                       |           BE294696           |          CB601163             |
|                       |           BM048331           |          AK012688             |
|                       |           BE897526           |          CD562440             |
|                       |           BP248550           |          CA563706             |
|                       |           CN294721           |          CD563091             |
|                       |           BG425282           |          CN666438             |
|                       |           CN294719           |          BE534192             |
|                       |           BE926213           |          BX522535             |
|                       |           BE700899           |          CF909231             |
|                       |           BE818920           |          CK335829             |
|                       |           BE818946           |          BB839321             |
|                       |           AW889835           |          CF905169             |
|                       |           BE818949           |          BY007090             |
|                       |           BE818947           |          BY140696             |
|                       |           CN294722           |                               |
|                       |           BF981568           |                               |
|                       |           BX283092           |                               |
|                       |           BG682880           |                               |
|                       |           BU942377           |                               |
|                       |           CN294723           |                               |
|                       |           BP195324           |                               |
|                       |           BP343645           |                               |
|                       |           BP332326           |                               |
|                       |           BP366759           |                               |
|                       |           BP276711           |                               |
|                       |           BP225326           |                               |
|                       |           BP211873           |                               |
|                       |           BP349583           |                               |
|                       |           BP214823           |                               |
|                       |           BP372999           |                               |
|                       |           BP209401           |                               |
|                       |           BP221611           |                               |
|                       |           CN294718           |                               |
|                       |           BG686931           |                               |
|                       |           AU135607           |                               |
|                       |           BM748778           |                               |
|                       |           AU279939           |                               |
|                       |           AW578807           |                               |
|                       |           AW855775           |                               |
|                       |           AW855776           |                               |
|                       |           AW855783           |                               |
|                       |           AW855756           |                               |
|                       |           BP292320           |                               |
|                       |           BF746634           |                               |
|                       |           BP238751           |                               |
|                       |           BE066517           |                               |
|                       |           BG424079           |                               |
|                       |           BF747348           |                               |
|                       |           BE693434           |                               |
|                       |           BU928071           |                               |
|                       |           BF001102           |                               |
|                       |           BP236398           |                               |
|                       |           BM754487           |                               |
|                       |           AA315627           |                               |
|                       |           CD694012           |                               |
|                       |           BP197538           |                               |
|                       |           AL047535           |                               |
|                       |           BM848006           |                               |
|                       |           AL543064           |                               |
|                       |           AW821182           |                               |
|                       |           CD357762           |                               |
|                       |           CN294720           |                               |
|                       |           AA317586           |                               |
|                       |           CB138012           |                               |
|                       |           CB139434           |                               |
|                       |           CB992472           |                               |
|                       |           AU100079           |                               |
+-----------------------+------------------------------+-------------------------------+
|      #   89           | *Seq. mapped in strand Plus* | *Seq. mapped in strand Plus*  |
|                       |           AL523561           |          AW825230             |
|                       |           BU181986           |                               |
|                       |           BC037963           |                               |
|                       |           BX421464           |                               |
|                       |           BX410970           |                               |
|                       |           BX420655           |                               |
|                       |           AL561477           |                               |
|                       |           BX443130           |                               |
|                       |           AL524902           |                               |
|                       |           AL529353           |                               |
|                       |           CR616194           |                               |
|                       |           CB998119           |                               |
|                       |             X61157           |                               |
|                       |             M80776           |                               |
|                       |           BI256612           |                               |
|                       |           CV030879           |                               |
|                       |           AL558411           |                               |
|                       |           BG395964           |                               |
|                       |           BG826670           |                               |
|                       |           BF305443           |                               |
|                       |           BE251954           |                               |
|                       |           BE298717           |                               |
|                       |           BM465092           |                               |
|                       |           CN410166           |                               |
|                       |           AW793930           |                               |
|                       |           BG391819           |                               |
|                       |           BM147496           |                               |
|                       |           BM149345           |                               |
|                       |           BP327293           |                               |
|                       |           BP327321           |                               |
|                       |           BG829196           |                               |
|                       |           BE300440           |                               |
|                       |           BQ687285           |                               |
|                       |           BU180078           |                               |
|                       |           BU196732           |                               |
|                       |           BQ689062           |                               |
|                       |           AW843896           |                               |
|                       |           CA454809           |                               |
|                       |           BQ711601           |                               |
|                       |           BE712864           |                               |
|                       |           BE712991           |                               |
|                       |           BE764733           |                               |
|                       |           CF125051           |                               |
|                       |           BE816334           |                               |
|                       |           CB269245           |                               |
|                       |           BE712968           |                               |
|                       |           BE713031           |                               |
|                       |           BE712907           |                               |
|                       |           BE712908           |                               |
|                       |           BE712893           |                               |
|                       |           BE713046           |                               |
|                       |           BE712905           |                               |
|                       |           BE712951           |                               |
|                       |           BE712898           |                               |
|                       |           BE712954           |                               |
|                       |           BE712910           |                               |
|                       |           BE712914           |                               |
|                       |           BE712939           |                               |
|                       |           BE712925           |                               |
|                       |           BE712995           |                               |
|                       |           BE712872           |                               |
|                       |           BE712980           |                               |
|                       |           BE712989           |                               |
|                       |           BI117782           |                               |
|                       |           BE793722           |                               |
|                       |           BE713048           |                               |
|                       |           BE712933           |                               |
|                       |           BE713021           |                               |
|                       |           BE900875           |                               |
|                       |           BE712957           |                               |
|                       |           BE764762           |                               |
|                       |           BE836278           |                               |
|                       |           BF346000           |                               |
|                       |           BE764756           |                               |
|                       |           BF806263           |                               |
|                       |           BE713029           |                               |
|                       |           BM762539           |                               |
|                       |           BM761213           |                               |
|                       |           BE836306           |                               |
|                       |           AI424305           |                               |
|                       |           BE613761           |                               |
|                       |           BF767291           |                               |
|                       |           BF091964           |                               |
|                       |           AW967945           |                               |
|                       |           AA255647           |                               |
|                       |           BE939301           |                               |
|                       |           BE939316           |                               |
|                       |           BE711115           |                               |
|                       |           BE713039           |                               |
|                       |           BE712941           |                               |
|                       |           BE713045           |                               |
|                       |           AA324644           |                               |
|                       |           CN410162           |                               |
|                       |           BQ226093           |                               |
|                       |           BU429891           |                               |
|                       |           BQ052934           |                               |
|                       |           BG910358           |                               |
|                       |           BQ070009           |                               |
|                       |           BU430860           |                               |
|                       |           BF899554           |                               |
|                       |           AK131506           |                               |
|                       |           BF975373           |                               |
|                       |           BF974670           |                               |
|                       |           BE273242           |                               |
|                       |           BG393348           |                               |
|                       |           BE737079           |                               |
|                       |           BU957332           |                               |
|                       |           BG392959           |                               |
|                       |           BF315660           |                               |
|                       |           BG954415           |                               |
|                       |           BI771458           |                               |
|                       |           BE247329           |                               |
|                       |           BE392195           |                               |
|                       |           BQ223251           |                               |
|                       |           AL119235           |                               |
|                       |           BX430700           |                               |
|                       |           BQ229765           |                               |
|                       |           BG490691           |                               |
|                       |           CA454172           |                               |
|                       |           BF993734           |                               |
|                       |           BM548874           |                               |
|                       |           BE397849           |                               |
|                       |           BI224041           |                               |
|                       |           CB997965           |                               |
|                       |           BE396873           |                               |
|                       |           BE513542           |                               |
|                       |           BE559742           |                               |
|                       |           BE560182           |                               |
|                       |           BE297884           |                               |
|                       |           BI224838           |                               |
|                       |           BI224947           |                               |
|                       |           BI224925           |                               |
|                       |           BU150921           |                               |
|                       |           BI025032           |                               |
|                       |           BE794605           |                               |
|                       |           AW732675           |                               |
|                       |           BE396870           |                               |
|                       |           BE513285           |                               |
|                       |           BE396794           |                               |
|                       |           BE561418           |                               |
|                       |           BE560298           |                               |
|                       |           BM922905           |                               |
|                       |           AW408770           |                               |
|                       |           AW674368           |                               |
|                       |           CV417842           |                               |
|                       |           BI917956           |                               |
|                       |           BQ689379           |                               |
|                       |           BG036335           |                               |
|                       |           BG397697           |                               |
|                       |           BF975060           |                               |
|                       |           CV329388           |                               |
|                       |           BQ421533           |                               |
|                       |           BF663330           |                               |
|                       |           BG684395           |                               |
|                       |           BG685583           |                               |
|                       |             R88246           |                               |
|                       |           BE939814           |                               |
|                       |           BQ723485           |                               |
|                       |           BG768423           |                               |
|                       |           CF140313           |                               |
|                       |             T28088           |                               |
|                       |           AW966962           |                               |
|                       |           BG468575           |                               |
|                       |           BG328693           |                               |
|                       |           CD671377           |                               |
|                       |           BU191678           |                               |
|                       |           CN410167           |                               |
|                       |           BI056580           |                               |
|                       |           BF935628           |                               |
|                       |           BG574720           |                               |
|                       |           AW245699           |                               |
|                       |           CN410164           |                               |
|                       |           BF873692           |                               |
|                       |           BG755515           |                               |
|                       |           AW246049           |                               |
|                       |           BM784361           |                               |
|                       |           BI026421           |                               |
|                       |           BU527750           |                               |
|                       |           BQ931998           |                               |
|                       |           BG389396           |                               |
|                       |           CB122718           |                               |
|                       |           AW881797           |                               |
|                       |           AW856222           |                               |
|                       |           BM920472           |                               |
|                       |           BQ066093           |                               |
|                       |           AA291247           |                               |
|                       |           BF751657           |                               |
|                       |           BG744038           |                               |
|                       |           BG256728           |                               |
|                       |           BE246015           |                               |
|                       |           BM144440           |                               |
|                       |           BG118817           |                               |
|                       |           BE179119           |                               |
|                       |           BQ694523           |                               |
|                       |           BE140531           |                               |
|                       |           AW806763           |                               |
|                       |           AW806789           |                               |
|                       |           AW806794           |                               |
|                       |           AW806795           |                               |
|                       |           AW845203           |                               |
|                       |           BG177376           |                               |
|                       |           BM466254           |                               |
|                       |           BM148362           |                               |
|                       |           BE140522           |                               |
|                       |           BG389356           |                               |
|                       |           BE140518           |                               |
|                       |           BQ955636           |                               |
|                       |           BI912037           |                               |
|                       |           BI031312           |                               |
|                       |           BM917489           |                               |
|                       |           BE243364           |                               |
|                       |           BI524012           |                               |
|                       |           BE646518           |                               |
|                       |           BE927968           |                               |
|                       |           BE512997           |                               |
|                       |           BG008256           |                               |
|                       |           CF121377           |                               |
|                       |           BM144986           |                               |
|                       |           BX436573           |                               |
|                       |           BQ053956           |                               |
|                       |           BE872032           |                               |
|                       |           BG177875           |                               |
|                       |             R84723           |                               |
|                       |           BI258270           |                               |
|                       |           BF812644           |                               |
|                       |           BM450415           |                               |
|                       |           BQ329101           |                               |
|                       |           AW502609           |                               |
|                       |           CF140302           |                               |
|                       |           BI909359           |                               |
|                       |           BQ946928           |                               |
|                       |           AA161236           |                               |
|                       |           BF958358           |                               |
|                       |           BI031348           |                               |
|                       |           BI020536           |                               |
|                       |             T09391           |                               |
|                       |           BG744465           |                               |
|                       |           AA985290           |                               |
|                       |           BI193081           |                               |
|                       |           BU739935           |                               |
|                       |           BM554400           |                               |
|                       |           BM010001           |                               |
|                       |           BM010214           |                               |
|                       |           BQ335435           |                               |
|                       |           BE561545           |                               |
|                       |           CN410165           |                               |
|                       |           BE838468           |                               |
|                       |           BI226698           |                               |
|                       |           BF821710           |                               |
|                       |           BI000372           |                               |
|                       |           CF145775           |                               |
|                       |           BG283538           |                               |
|                       |           CF126902           |                               |
|                       |           BE513745           |                               |
|                       |           BE559825           |                               |
|                       |           CK430495           |                               |
|                       |           BF514634           |                               |
|                       |           CR620940           |                               |
|                       |           BU621764           |                               |
|                       |           CA426376           |                               |
|                       |           BU621331           |                               |
|                       |           BM977642           |                               |
|                       |           CD742800           |                               |
|                       |           BM975379           |                               |
|                       |           BU689828           |                               |
|                       |           BQ009053           |                               |
|                       |           BQ448312           |                               |
|                       |           CA417882           |                               |
|                       |           CD368523           |                               |
|                       |           BU689404           |                               |
|                       |           BQ446125           |                               |
|                       |           BM974173           |                               |
|                       |           BQ186824           |                               |
|                       |           CK299931           |                               |
|                       |           BM720075           |                               |
|                       |           BM970815           |                               |
|                       |           BU681701           |                               |
|                       |           BE465461           |                               |
|                       |           AI074715           |                               |
|                       |           AI185748           |                               |
|                       |           CA310458           |                               |
|                       |           AW294038           |                               |
|                       |           BE327961           |                               |
|                       |           AI623998           |                               |
|                       |           AI888604           |                               |
|                       |           AW439175           |                               |
|                       |           AA830116           |                               |
|                       |           AI435873           |                               |
|                       |           AW204462           |                               |
|                       |           AA489013           |                               |
|                       |           BM663241           |                               |
|                       |           AI690611           |                               |
|                       |           AW196377           |                               |
|                       |           AI689045           |                               |
|                       |           AW245343           |                               |
|                       |           BQ026485           |                               |
|                       |           AA459417           |                               |
|                       |           AI916070           |                               |
|                       |           BF941906           |                               |
|                       |           AA595312           |                               |
|                       |           AA780981           |                               |
|                       |           AA740925           |                               |
|                       |           BQ027431           |                               |
|                       |           AI276436           |                               |
|                       |           AW298240           |                               |
|                       |           AA872212           |                               |
|                       |           AA743246           |                               |
|                       |           AW674536           |                               |
|                       |           AA504173           |                               |
|                       |           AI473561           |                               |
|                       | *Seq. mapped in strand Minus*| *Seq. mapped in strand Minus* |
|                       |           BM998797           |          AW125212             |
+-----------------------+------------------------------+-------------------------------+
|      #   90           | *Seq. mapped in strand Plus* | *Seq. mapped in strand Plus*  |
|                       |           AW292907           |          BE944250             |
|                       | *Seq. mapped in strand Minus*| *Seq. mapped in strand Minus* |
|                       |           AA629830           |          BE625179             |
+-----------------------+------------------------------+-------------------------------+
|      #   91           | *Seq. mapped in strand Plus* | *Seq. mapped in strand Plus*  |
|                       |           AK026208           |          AK079116             |
|                       |           BC017959           |          AK007831             |
|                       |           CR622948           |          BC071241             |
|                       |           CR591130           |          BC024945             |
|                       |           AL832964           |                               |
|                       | *Seq. mapped in strand Minus*| *Seq. mapped in strand Minus* |
|                       |           BX647677           |          BC058590             |
|                       |           AK095272           |          BC023868             |
|                       |           BC025758           |                               |
|                       |           BC063502           |                               |
|                       |           BX648265           |                               |
+-----------------------+------------------------------+-------------------------------+
|      #   92           | *Seq. mapped in strand Plus* | *Seq. mapped in strand Plus*  |
|                       |           AK126009           |          AF144629             |
|                       | *Seq. mapped in strand Minus*| *Seq. mapped in strand Minus* |
|                       |           AF086552           |          AK090173             |
|                       |           AB017169           |                               |
|                       |           AY358884           |                               |
|                       |           AF075240           |                               |
|                       |           AL122074           |                               |
+-----------------------+------------------------------+-------------------------------+
|      #   93           | *Seq. mapped in strand Plus* | *Seq. mapped in strand Plus*  |
|                       |           AF542512           |          BY297240             |
|                       |                              |          BQ943353             |
|                       |                              |          BY234834             |
|                       |                              |          BY101972             |
|                       |                              |          CK389092             |
|                       | *Seq. mapped in strand Minus*| *Seq. mapped in strand Minus* |
|                       |           BC047944           |          AK087798             |
|                       |                              |          BB666583             |
|                       |                              |                               |
|                       |                              |                               |
|                       |                              |                               |
+-----------------------+------------------------------+-------------------------------+
|      #   94           | *Seq. mapped in strand Plus* | *Seq. mapped in strand Plus*  |
|                       |           AL537191           |          AU015808             |
|                       |           BG772735           |          BG070381             |
|                       |           BX463064           |                               |
|                       |           AL520863           |                               |
|                       |           CR613478           |                               |
|                       |           CR600658           |                               |
|                       |           AV751402           |                               |
|                       |           BC022428           |                               |
|                       |           BX404388           |                               |
|                       |           BC004465           |                               |
|                       |           BC005841           |                               |
|                       |           AL538108           |                               |
|                       |           CR612877           |                               |
|                       |           BX396946           |                               |
|                       |           BC016739           |                               |
|                       |           BC078171           |                               |
|                       |           AL535049           |                               |
|                       |           AF289599           |                               |
|                       |           BX444969           |                               |
|                       |           BX432250           |                               |
|                       |           AL536914           |                               |
|                       |           BX406959           |                               |
|                       |           BX440103           |                               |
|                       |           AV752255           |                               |
|                       |           AL537342           |                               |
|                       |           BX440102           |                               |
|                       |           AL540465           |                               |
|                       |           CR626430           |                               |
|                       |           BG758709           |                               |
|                       |           BQ420330           |                               |
|                       |           BI547571           |                               |
|                       |           BE796367           |                               |
|                       |           AL538722           |                               |
|                       |           BX404427           |                               |
|                       |           BX432511           |                               |
|                       |           BX441563           |                               |
|                       |           BI760619           |                               |
|                       |           BX397127           |                               |
|                       |           BE559856           |                               |
|                       |           AF262988           |                               |
|                       |           CR601111           |                               |
|                       |           BU181249           |                               |
|                       |           CB851838           |                               |
|                       |           BX453659           |                               |
|                       |           BX409479           |                               |
|                       |           CD513010           |                               |
|                       |           BI222768           |                               |
|                       |           CR605991           |                               |
|                       |           BQ636288           |                               |
|                       |           BX450996           |                               |
|                       |           AV750035           |                               |
|                       |           BX418265           |                               |
|                       |           CA841398           |                               |
|                       |           BP265451           |                               |
|                       |           BX450985           |                               |
|                       |           BX451004           |                               |
|                       |           AW138787           |                               |
|                       |           BM467744           |                               |
|                       |             W43028           |                               |
|                       |           BG751060           |                               |
|                       |           BM707412           |                               |
|                       |           CD513089           |                               |
|                       |             T08673           |                               |
|                       |           BE207963           |                               |
|                       |           BP209471           |                               |
|                       |           CF140581           |                               |
|                       |           CF140508           |                               |
|                       |           AW408494           |                               |
|                       |           BQ695129           |                               |
|                       |           AW236918           |                               |
|                       |           BI334706           |                               |
|                       |           BI334411           |                               |
|                       |           BF149399           |                               |
|                       |             T26592           |                               |
|                       |           BX347862           |                               |
|                       |             H08241           |                               |
|                       |           AV702853           |                               |
|                       |           AA382949           |                               |
|                       |           AA307828           |                               |
|                       |           AV654392           |                               |
|                       |           CD251204           |                               |
|                       |           BI258901           |                               |
|                       |           BG502340           |                               |
|                       |           BG533789           |                               |
|                       |           BP347617           |                               |
|                       |           BF102490           |                               |
|                       |           BM970480           |                               |
|                       |           BX326534           |                               |
|                       |           BX451035           |                               |
|                       |           CK003507           |                               |
|                       |           BG532395           |                               |
|                       |           AW802710           |                               |
|                       |           AA325554           |                               |
|                       |           BG500901           |                               |
|                       |             R88485           |                               |
|                       |           CB140916           |                               |
|                       |           BF672861           |                               |
|                       |           BP290681           |                               |
|                       |           BP193159           |                               |
|                       |           BM665871           |                               |
|                       |           BX409477           |                               |
|                       |           AW965938           |                               |
|                       |           BX431849           |                               |
|                       |           BF691503           |                               |
|                       |           BF934182           |                               |
|                       |           BU677033           |                               |
|                       |           BM979375           |                               |
|                       |             R38880           |                               |
|                       |           AI174694           |                               |
|                       |           BU567248           |                               |
|                       |           CA448061           |                               |
|                       |           CA502758           |                               |
|                       |           BU687403           |                               |
|                       |           CA944421           |                               |
|                       |           BU682884           |                               |
|                       |           BU675995           |                               |
|                       |           BU682518           |                               |
|                       |           BQ001025           |                               |
|                       |           BQ017033           |                               |
|                       |           CA422241           |                               |
|                       |           CA449024           |                               |
|                       |           BU728687           |                               |
|                       |           AA865293           |                               |
|                       |           AW292103           |                               |
|                       |           BQ270650           |                               |
|                       |           AI051988           |                               |
|                       |           BU676550           |                               |
|                       |           AA834539           |                               |
|                       |           AI921412           |                               |
|                       |           AA807452           |                               |
|                       |           AA447396           |                               |
|                       |           AI359355           |                               |
|                       |           AI538504           |                               |
|                       |           AI086663           |                               |
|                       |           AI679349           |                               |
|                       |           AW248587           |                               |
|                       |           BU734959           |                               |
|                       |           AA744908           |                               |
|                       |           CN428477           |                               |
|                       |           AA814511           |                               |
|                       |           AI862204           |                               |
|                       |           AW007885           |                               |
|                       |             T23061           |                               |
|                       |           AA769291           |                               |
|                       |           AI672152           |                               |
|                       |           AA604991           |                               |
|                       |           CA841378           |                               |
|                       |           AI473152           |                               |
|                       |           CD723051           |                               |
|                       |           CD723086           |                               |
|                       | *Seq. mapped in strand Minus*| *Seq. mapped in strand Minus* |
|                       |           BX329434           |            W77297             |
|                       |           BG180718           |          BF227280             |
|                       |           AA970759           |          BF719400             |
|                       |           AI039385           |          BM210140             |
|                       |           BI861026           |          BG094341             |
|                       |           BI870350           |          BB801499             |
|                       |             T85095           |          BE628478             |
|                       |           BF732658           |          CK331006             |
|                       |           AA759087           |          CK331009             |
|                       |           AI185717           |          BE630881             |
|                       |           BM313059           |          BI713808             |
|                       |           AI291211           |          BI713188             |
|                       |           AA443856           |          BB734742             |
|                       |           BF439075           |          BM206729             |
|                       |           BE646109           |          BM218635             |
|                       |           BG202381           |          BY574283             |
|                       |           AI494629           |          BY467510             |
|                       |           BG939801           |          CA465221             |
|                       |           AA621215           |          BE335350             |
|                       |           AI081745           |          AI536437             |
|                       |           CD243221           |          BI793820             |
|                       |           BG108257           |          CB842878             |
|                       |           BG179024           |          CB196005             |
|                       |           AI927264           |          CF579123             |
|                       |           BQ672333           |          BM224682             |
|                       |           CR604139           |          CA885530             |
|                       |           BI092683           |          BM221833             |
|                       |           AI239926           |          BY507381             |
|                       |           AA425858           |          AU045983             |
|                       |           AI568980           |          BB809718             |
|                       |           AI971978           |          BB798559             |
|                       |           BE048358           |          AA407939             |
|                       |           CD742935           |          BB837016             |
|                       |             H05831           |          BY603897             |
|                       |           AI150764           |          BY518277             |
|                       |           AI565855           |          BY621697             |
|                       |           AI302477           |          BB737760             |
|                       |           BM996669           |          BY620954             |
|                       |           AA570143           |          BY644763             |
|                       |             R43186           |          AA221158             |
|                       |             R43187           |          BM899251             |
|                       |             H05288           |          BQ031970             |
|                       |             R43913           |          BY653099             |
|                       |           BM668020           |          BY653149             |
|                       |           BF213389           |          AI837220             |
|                       |           CB045163           |          BF226619             |
|                       |           AI369212           |          BY510675             |
|                       |             N70800           |          BY653034             |
|                       |           AI346692           |          BY516125             |
|                       |           BU680547           |          BY624747             |
|                       |           BM993728           |          BY619319             |
|                       |           CA443742           |          BY627654             |
|                       |           AA446904           |          AI847834             |
|                       |           BM974653           |          BY647374             |
|                       |             W93690           |          BB781793             |
|                       |           CA419324           |          BF462581             |
|                       |           AW263557           |          BY389102             |
|                       |           BM669131           |          BY389869             |
|                       |           BM678272           |          BY474190             |
|                       |           BU618314           |          BY513544             |
|                       |           AI093591           |          BY622014             |
|                       |           CA446512           |          AW240425             |
|                       |           CD370187           |          BY512092             |
|                       |           CA309294           |          BG066271             |
|                       |           BM984833           |          BU057044             |
|                       |           BU632607           |          BB779223             |
|                       |           CA431473           |          BB783732             |
|                       |           BU615513           |          AW049538             |
|                       |           CA414816           |          BY644943             |
|                       |           CA449000           |          BB773988             |
|                       |           BX332297           |          CB588123             |
|                       |           BM970982           |          BY498515             |
|                       |           BG187955           |          AI462504             |
|                       |           AA431976           |          BB745670             |
|                       |           AI268801           |          CF739537             |
|                       |           AI094363           |          CF739584             |
|                       |           AW675748           |          BM293436             |
|                       |           AI041024           |          BY471093             |
|                       |           BX455745           |          BY466337             |
|                       |           BM548997           |          BM223166             |
|                       |           AW071209           |          BM222913             |
|                       |             H62843           |          BM224534             |
|                       |           AA758504           |          BM209773             |
|                       |           AI378252           |          CA318721             |
|                       |           AA568883           |          BM222334             |
|                       |           AW149354           |          BM207671             |
|                       |           AA714876           |          AI591534             |
|                       |           AW009742           |          BI438005             |
|                       |           AA025134           |          BI987937             |
|                       |           AA025163           |          BQ128992             |
|                       |             N54535           |          BG866959             |
|                       |           AI122674           |          BF227349             |
|                       |           AI867301           |          BC027356             |
|                       |           AW328170           |          BC035324             |
|                       |             T28841           |          AK089053             |
|                       |           AI720514           |          BC036289             |
|                       |           AA704403           |          BB800777             |
|                       |           AA639540           |          BB695630             |
|                       |           AA665448           |            C78670             |
|                       |           AI361505           |          BY384997             |
|                       |             N55561           |          BB695629             |
|                       |           AI347811           |          BB752100             |
|                       |           AI934833           |          CA326865             |
|                       |           AI096598           |          AK090203             |
|                       |             N50552           |          BB755118             |
|                       |           AI127844           |          BB756148             |
|                       |           BG389581           |          AA689746             |
|                       |           BF679500           |          AA727543             |
|                       |           CA445516           |            W08610             |
|                       |           AA662444           |          AW011945             |
|                       |           BU684321           |          BF722696             |
|                       |           CB044330           |          BF023445             |
|                       |           AI280874           |          BI407536             |
|                       |           AW051813           |          AA606664             |
|                       |           AW131838           |          AA815798             |
|                       |           BM973347           |          AA645501             |
|                       |           AA573283           |          BM210819             |
|                       |           CA422876           |          AA475557             |
|                       |           BM978172           |          AA606653             |
|                       |           AI401599           |          AA422603             |
|                       |           CA422993           |          AA692131             |
|                       |           CA415965           |          AI120893             |
|                       |           CA431850           |          AA982821             |
|                       |           BQ006238           |          BM199985             |
|                       |           AW170618           |          BM235434             |
|                       |           CD365889           |          BG079487             |
|                       |           AI762539           |          AA209876             |
|                       |           BU687902           |          BF660903             |
|                       |           CB306617           |          BG147742             |
|                       |           CA447522           |          BF021088             |
|                       |           BC004132           |          AI155677             |
|                       |           AK126584           |          AL034818             |
|                       |             H91283           |          AA120029             |
|                       |           BE300746           |          CA534336             |
|                       |           CB044272           |          CF908256             |
|                       |           AI651426           |            W13581             |
|                       |           AI097035           |          AA615776             |
|                       |           AA582748           |          BQ884532             |
|                       |           AA583419           |          BE572324             |
|                       |           AI682990           |          BF023131             |
|                       |             D32053           |          BE647539             |
|                       |           AI674443           |          CF352600             |
|                       |             F02808           |          BF159882             |
|                       |             T15632           |          AL033367             |
|                       |           AI475211           |          CA547836             |
|                       |           AA736396           |            W83709             |
|                       |           AW170709           |          AA920403             |
|                       |           BU956814           |          BF151993             |
|                       |           BM970284           |          BI112452             |
|                       |           BU681465           |          CF951761             |
|                       |           AA780600           |          BI990918             |
|                       |           CB243792           |          AA734265             |
|                       |           BQ676595           |          AA119635             |
|                       |           BU157816           |          AA896080             |
|                       |             D31890           |          CA533828             |
|                       |           AA614334           |          CA533507             |
|                       |           BG391437           |          CA533525             |
|                       |           BF971248           |          CA528416             |
|                       |           BX405221           |          AA589550             |
|                       |           BM672274           |          AI046971             |
|                       |           BX428014           |          AA144513             |
|                       |             T69945           |          AA646272             |
|                       |           BQ576311           |          AA619873             |
|                       |           CA416084           |          AA647728             |
|                       |           AL581487           |          AA562920             |
|                       |           BC013246           |          BI156680             |
|                       |           CR611117           |          AA467662             |
|                       |           AA225489           |          BF011961             |
|                       |           AI859099           |          CB952291             |
|                       |           BF033254           |          BY052945             |
|                       |           AL581565           |          AW908546             |
|                       |           CR593300           |          AA120517             |
|                       |           AA076288           |          AL024205             |
|                       |           AA486220           |          BI412178             |
|                       |           AI864725           |          BU516714             |
|                       |           AA219436           |            W30629             |
|                       |           AA729526           |          AL024334             |
|                       |           BE139506           |          AA067716             |
|                       |           AW244063           |          BI407979             |
|                       |           AI499978           |          BI554375             |
|                       |           AW193613           |          BI646172             |
|                       |           AA765674           |          BQ964450             |
|                       |           BG057186           |          BG145178             |
|                       |           AA810122           |          CF579122             |
|                       |           AA826636           |          BI219330             |
|                       |             R01261           |          BI654924             |
|                       |           AI886692           |          AA547662             |
|                       |           AA157319           |          BF138169             |
|                       |           AA187940           |          BI152123             |
|                       |           AI242405           |          BU706166             |
|                       |           AI952695           |          AA060341             |
|                       |           AI572983           |          BI159619             |
|                       |           AA099697           |          BG872192             |
|                       |           AA574018           |          CF730037             |
|                       |           AA977448           |          CA751747             |
|                       |           AA136638           |          BQ928166             |
|                       |           AA588070           |          BU709638             |
|                       |           AA593700           |          CA751896             |
|                       |           AA954213           |          CA945392             |
|                       |           AA622340           |          BI453597             |
|                       |           AA489715           |          BI153393             |
|                       |           AA490053           |          BU571200             |
|                       |           AW304794           |          CA749228             |
|                       |           AA151845           |          BQ178719             |
|                       |           AA599834           |          BI686476             |
|                       |           AA704099           |            W78644             |
|                       |           AA521031           |          BF160059             |
|                       |           AA599314           |            W14553             |
|                       |           AA602650           |          BY710288             |
|                       |           AW664616           |          AA138593             |
|                       |           AI247089           |          BE381973             |
|                       |           AA630704           |          BI646207             |
|                       |           AA614337           |          AA073787             |
|                       |           AA057672           |          BF023413             |
|                       |           AW305003           |          BG246133             |
|                       |           AW087940           |          CF950105             |
|                       |           BE221543           |          BU513838             |
|                       |           AW173723           |          BF162438             |
|                       |           AI801335           |          BI697179             |
|                       |           AI609397           |          BI081929             |
|                       |           AI608907           |          CD807407             |
|                       |           BG824214           |          BE289351             |
|                       |             H83409           |          CA983783             |
|                       |           AA703991           |          BY747461             |
|                       |           AL536062           |          CN539394             |
|                       |           CR601961           |          BY737500             |
|                       |           AV706651           |          BI986804             |
|                       |           BF971382           |          BF151926             |
|                       |           BX463770           |          BI989205             |
|                       |           AA086271           |          BG808653             |
|                       |           AA985162           |          BI437757             |
|                       |           AL534229           |          BU703073             |
|                       |           AA446647           |          BF471592             |
|                       |           CN292264           |          AI664371             |
|                       |           AL515660           |          AW044798             |
|                       |           CR622561           |          CB601239             |
|                       |           CN292265           |          AU051422             |
|                       |           AL571511           |          BI794141             |
|                       |           AL574355           |          BQ566758             |
|                       |           CR608423           |          CA752057             |
|                       |           BQ787349           |          CF730317             |
|                       |           AL567684           |            L26772             |
|                       |           BP429488           |          BB872426             |
|                       |           AL568115           |          CB232439             |
|                       |           CR617122           |          AW412934             |
|                       |           BQ311501           |          BI652585             |
|                       |           BX372730           |          CK337974             |
|                       |           BF838060           |          BY259788             |
|                       |           BQ371012           |          BB861876             |
|                       |           BU183314           |          BY260832             |
|                       |           AL580896           |          BU697556             |
|                       |           BQ338318           |          BG920105             |
|                       |           BQ338350           |          BY258996             |
|                       |           BQ338360           |          AA711947             |
|                       |           BE703823           |          BY259071             |
|                       |           BQ338351           |          BF719947             |
|                       |           BM796004           |          BY291434             |
|                       |           BQ338277           |          BY251042             |
|                       |           BE818761           |          BF467294             |
|                       |           BQ338278           |          AW912174             |
|                       |           BE834170           |          BY261866             |
|                       |           BQ338425           |          BY262351             |
|                       |           BQ338448           |          BI987482             |
|                       |           BM817567           |          CK337909             |
|                       |           BQ351423           |          AA657124             |
|                       |           BM794146           |          CK337907             |
|                       |           AA076287           |          BB847538             |
|                       |           BE703772           |          BY279924             |
|                       |           BQ311823           |          BY031470             |
|                       |           BQ338368           |          BY032269             |
|                       |           BQ338329           |          BY265018             |
|                       |           BQ338330           |          BY292478             |
|                       |           BQ338359           |          BY774186             |
|                       |           BQ338367           |          BY165133             |
|                       |           BQ338381           |          BB873420             |
|                       |           BQ338316           |          BY267491             |
|                       |           BQ338326           |          AA407940             |
|                       |           BQ338327           |          BB869897             |
|                       |           BQ338449           |          BY173783             |
|                       |           BQ338317           |          BB871833             |
|                       |           BQ338328           |          BU583490             |
|                       |           BI490581           |          BY147430             |
|                       |           AL535672           |          CK336585             |
|                       |           BG576997           |          BY104155             |
|                       |           BQ363339           |          BB869795             |
|                       |           AA460614           |          BQ417814             |
|                       |           AA410502           |          BY343447             |
|                       |           BM696989           |          BY331495             |
|                       |           BM782398           |          BY792549             |
|                       |           AA425264           |          BB859383             |
|                       |           AW362556           |          AW681788             |
|                       |           CB157784           |          BY026574             |
|                       |           BM832865           |          BY174108             |
|                       |           AW242168           |          BQ572652             |
|                       |           BU622687           |          AW258396             |
|                       |           BF509567           |          AI661225             |
|                       |           AI002294           |          BY000947             |
|                       |           AA037797           |          BY251150             |
|                       |           BM747754           |            W53766             |
|                       |           BQ347770           |          BY767376             |
|                       |             W22544           |          BM875961             |
|                       |           BM740195           |          BY291502             |
|                       |           AI739662           |          BY134491             |
|                       |           AF285758           |          AA692242             |
|                       |             W93742           |          BY137381             |
|                       |           BM700929           |          AA184397             |
|                       |           BQ360504           |          BY145101             |
|                       |           AI094401           |          BY290141             |
|                       |           AI802253           |          BY771035             |
|                       |           AA976735           |          BY768818             |
|                       |           AA974496           |          BF457428             |
|                       |           AA826269           |          BB598409             |
|                       |           AW614943           |          BY299047             |
|                       |           AW340864           |          BY779978             |
|                       |             F19177           |            R75144             |
|                       |           BQ227210           |                               |
|                       |           BQ316402           |                               |
|                       |           BQ377012           |                               |
|                       |           BQ377234           |                               |
|                       |           CN292263           |                               |
|                       |           BQ328289           |                               |
|                       |           CV387594           |                               |
|                       |           CF126114           |                               |
|                       |           BM696790           |                               |
|                       |           BM854508           |                               |
|                       |           BM313337           |                               |
|                       |           BM789308           |                               |
|                       |           AA486374           |                               |
|                       |           BG338250           |                               |
|                       |           BE266434           |                               |
|                       |           BQ328300           |                               |
|                       |           CB163454           |                               |
|                       |           BG574951           |                               |
|                       |           BG121381           |                               |
|                       |             H54256           |                               |
|                       |           BE409524           |                               |
|                       |           BQ352033           |                               |
|                       |           CN292274           |                               |
|                       |             H83513           |                               |
|                       |           BM836314           |                               |
|                       |           BM831438           |                               |
|                       |           BM823467           |                               |
|                       |           BQ053397           |                               |
|                       |             N50606           |                               |
|                       |           CN292272           |                               |
|                       |           BQ302155           |                               |
|                       |           BU176615           |                               |
|                       |           BQ314843           |                               |
|                       |           AA622761           |                               |
|                       |           BI857829           |                               |
|                       |             H62963           |                               |
|                       |           BM825248           |                               |
|                       |             H62254           |                               |
|                       |           BM797055           |                               |
|                       |           BI859970           |                               |
|                       |           BE545362           |                               |
|                       |           BI161121           |                               |
|                       |           BE727803           |                               |
|                       |           AA489898           |                               |
|                       |           BE746049           |                               |
|                       |             N40221           |                               |
|                       |           BM855099           |                               |
|                       |           BQ314661           |                               |
|                       |           BQ878141           |                               |
|                       |           AA368367           |                               |
|                       |           AA368373           |                               |
|                       |           BM831284           |                               |
|                       |           BM831333           |                               |
|                       |           BQ083732           |                               |
|                       |           CB124486           |                               |
|                       |           BX327446           |                               |
|                       |           BE797509           |                               |
|                       |             R01375           |                               |
|                       |           BQ215534           |                               |
|                       |           BE019105           |                               |
|                       |           BX327445           |                               |
|                       |           AW820405           |                               |
|                       |           BG682242           |                               |
|                       |           AW838484           |                               |
|                       |           BE174728           |                               |
|                       |           BE174731           |                               |
|                       |           BE265771           |                               |
|                       |           AA188575           |                               |
|                       |           BQ314660           |                               |
|                       |           BG166640           |                               |
|                       |           AI494630           |                               |
|                       |             N76946           |                               |
|                       |           AW328169           |                               |
|                       |           BX098680           |                               |
|                       |             T70020           |                               |
|                       |           BG260770           |                               |
|                       |           BE562429           |                               |
|                       |           BU193322           |                               |
|                       |           AI886185           |                               |
|                       |           AW406887           |                               |
|                       |           BG033295           |                               |
|                       |           AW362554           |                               |
|                       |           CN292270           |                               |
|                       |           BU172471           |                               |
|                       |             C16307           |                               |
|                       |           CN292266           |                               |
|                       |             N25508           |                               |
|                       |           BG403838           |                               |
|                       |           BG117607           |                               |
|                       |           BM696388           |                               |
|                       |             T99605           |                               |
|                       |           CF146596           |                               |
|                       |           BG819263           |                               |
|                       |           BQ951556           |                               |
|                       |           BM551208           |                               |
|                       |           BE266973           |                               |
|                       |           BG327116           |                               |
|                       |           BI868411           |                               |
|                       |           BE869374           |                               |
|                       |           CF139488           |                               |
|                       |           BG677463           |                               |
|                       |           BM792009           |                               |
|                       |           BQ375674           |                               |
|                       |           BX350332           |                               |
|                       |           AA157418           |                               |
|                       |           BQ347767           |                               |
|                       |           CD515079           |                               |
|                       |           BM792953           |                               |
|                       |           AW673397           |                               |
|                       |           BE385564           |                               |
|                       |           BG751434           |                               |
|                       |           BQ366335           |                               |
|                       |           BM764865           |                               |
|                       |           BM764905           |                               |
|                       |           BM741759           |                               |
|                       |           BM741740           |                               |
|                       |           BQ360479           |                               |
|                       |           BQ360475           |                               |
|                       |           BQ360699           |                               |
|                       |           BQ360477           |                               |
|                       |           BQ337812           |                               |
|                       |           BQ337813           |                               |
|                       |           BE538713           |                               |
|                       |           BM738236           |                               |
|                       |           AA307709           |                               |
|                       |           BG119400           |                               |
|                       |           BM741726           |                               |
|                       |           BM770603           |                               |
|                       |           CF140783           |                               |
|                       |           BE877352           |                               |
|                       |           BM743590           |                               |
|                       |           BM770256           |                               |
|                       |           BM741171           |                               |
|                       |           BG897994           |                               |
|                       |           BM744044           |                               |
|                       |           BM741445           |                               |
|                       |           BM756050           |                               |
|                       |           CN292261           |                               |
|                       |           AA099694           |                               |
|                       |           BM770340           |                               |
|                       |             H90374           |                               |
|                       |           BM737754           |                               |
|                       |           BE796823           |                               |
|                       |           AA447024           |                               |
|                       |           BM741750           |                               |
|                       |           BQ924003           |                               |
|                       |           BU155131           |                               |
|                       |           BM770911           |                               |
|                       |           BM756583           |                               |
|                       |           BM770526           |                               |
|                       |           BM770782           |                               |
|                       |           BM770811           |                               |
|                       |           BM770886           |                               |
|                       |           BM771518           |                               |
|                       |           BM737922           |                               |
|                       |           BM747139           |                               |
|                       |           CB159286           |                               |
|                       |           BE273278           |                               |
|                       |           BM766885           |                               |
|                       |           BX446330           |                               |
|                       |           BM843858           |                               |
|                       |           BQ427747           |                               |
|                       |           BX463771           |                               |
|                       |           CB116329           |                               |
|                       |           AW949599           |                               |
|                       |           AA348232           |                               |
|                       |           BM463167           |                               |
|                       |           AL515661           |                               |
|                       |           BF036969           |                               |
|                       |           BP429606           |                               |
|                       |             C03300           |                               |
|                       |           BM745154           |                               |
|                       |           AL559908           |                               |
|                       |           AL546307           |                               |
|                       |           BX461432           |                               |
|                       |           CF122894           |                               |
|                       |           BE383722           |                               |
|                       |           AL526819           |                               |
|                       |           BX443581           |                               |
|                       |           AL560056           |                               |
|                       |           BM475905           |                               |
|                       |           BM738029           |                               |
|                       |           BQ339038           |                               |
|                       |           BP429691           |                               |
|                       |           AL539917           |                               |
|                       |           BX464607           |                               |
|                       |           BX428015           |                               |
|                       |           AL560288           |                               |
|                       |           AL550320           |                               |
|                       |           CV363443           |                               |
|                       |           AA676507           |                               |
|                       |           BG032928           |                               |
|                       |           BM771085           |                               |
|                       |             H06123           |                               |
|                       |           BG034316           |                               |
|                       |           AL534230           |                               |
|                       |           AA321235           |                               |
|                       |           BM793003           |                               |
|                       |             R18803           |                               |
|                       |           BM468296           |                               |
|                       |           BE882596           |                               |
|                       |           BI871731           |                               |
|                       |           BG164634           |                               |
|                       |           BG166759           |                               |
|                       |           BQ949735           |                               |
|                       |           BM800241           |                               |
|                       |           BM554720           |                               |
|                       |           AL547633           |                               |
|                       |           AL559964           |                               |
|                       |           BM457695           |                               |
|                       |           BM464250           |                               |
|                       |           BM463353           |                               |
|                       |           BM459037           |                               |
|                       |           BG576983           |                               |
|                       |           BQ894405           |                               |
|                       |           BE538817           |                               |
|                       |           BQ936732           |                               |
|                       |           BM766789           |                               |
|                       |           AL514978           |                               |
|                       |           AL536063           |                               |
|                       |           CN292268           |                               |
|                       |           BM766757           |                               |
|                       |           BM743887           |                               |
|                       |             H05337           |                               |
|                       |           BQ898279           |                               |
|                       |           BQ437399           |                               |
|                       |           AA136845           |                               |
|                       |           BM451994           |                               |
|                       |           BX414922           |                               |
|                       |           BG746552           |                               |
|                       |           BM454229           |                               |
|                       |           BX442936           |                               |
|                       |           BM806865           |                               |
|                       |           BU165024           |                               |
|                       |           BM456205           |                               |
|                       |           BU178160           |                               |
|                       |           BQ431045           |                               |
|                       |           CV324282           |                               |
|                       |           AI914403           |                               |
|                       |           BG876542           |                               |
|                       |           CN292279           |                               |
|                       |           BI092646           |                               |
|                       |           BI489728           |                               |
|                       |           CV334473           |                               |
|                       |           BE391317           |                               |
|                       |           BI256422           |                               |
|                       |           AL516675           |                               |
|                       |           BM665327           |                               |
|                       |           BU733958           |                               |
|                       |           BU167163           |                               |
|                       |           BQ953992           |                               |
|                       |           BU175828           |                               |
|                       |           BM450346           |                               |
|                       |           BI256751           |                               |
|                       |           AL037575           |                               |
|                       |           BU527840           |                               |
|                       |           BG248620           |                               |
|                       |           BI114490           |                               |
|                       |           BE742199           |                               |
|                       |           BI755905           |                               |
|                       |           BU161961           |                               |
|                       |           BQ068785           |                               |
|                       |           BQ230018           |                               |
|                       |           BX403912           |                               |
|                       |           BM909391           |                               |
|                       |           BU163907           |                               |
|                       |           BI599492           |                               |
|                       |           CF619405           |                               |
|                       |           AW629971           |                               |
|                       |           BI463225           |                               |
|                       |           BI091759           |                               |
|                       |           BE734854           |                               |
|                       |           BI464078           |                               |
|                       |           CF141691           |                               |
|                       |           BI553601           |                               |
|                       |           AA295192           |                               |
|                       |           CF144532           |                               |
|                       |           BQ723634           |                               |
|                       |           BQ437255           |                               |
|                       |           BE933531           |                               |
|                       |           BE894538           |                               |
|                       |           BM725663           |                               |
|                       |           BM476603           |                               |
|                       |           BQ431237           |                               |
|                       |           BI596644           |                               |
|                       |           BG258082           |                               |
|                       |           AL539123           |                               |
|                       |           BX445718           |                               |
|                       |           BG519615           |                               |
|                       |           BU159373           |                               |
|                       |           BI559963           |                               |
|                       |           BX458674           |                               |
|                       |           BF795702           |                               |
|                       |           AL559241           |                               |
|                       |           BF311276           |                               |
|                       |           CD519495           |                               |
|                       |           CN292273           |                               |
|                       |           BG772203           |                               |
|                       |           BM771006           |                               |
|                       |           CV027752           |                               |
|                       |           CN292260           |                               |
|                       |           BE539823           |                               |
|                       |           BI094412           |                               |
|                       |           BM927632           |                               |
|                       |           CF131256           |                               |
|                       |           BE902888           |                               |
|                       |           BX425763           |                               |
|                       |           BQ062460           |                               |
|                       |           BQ212535           |                               |
|                       |           BU165955           |                               |
|                       |           CD358324           |                               |
|                       |           BX441770           |                               |
|                       |           BM473482           |                               |
|                       |           BG685623           |                               |
|                       |           CN292259           |                               |
|                       |           CD556439           |                               |
|                       |           BI459074           |                               |
|                       |           BQ439144           |                               |
|                       |           BU070349           |                               |
|                       |           BE787601           |                               |
|                       |           BG773021           |                               |
|                       |           CB045164           |                               |
|                       |             R20128           |                               |
|                       |           BE266025           |                               |
|                       |           BI602100           |                               |
|                       |           AW601686           |                               |
|                       |           BI599414           |                               |
|                       |           BM564262           |                               |
|                       |           BG115713           |                               |
|                       |           BG747981           |                               |
|                       |           BU151116           |                               |
|                       |             H75655           |                               |
|                       |           BE267194           |                               |
|                       |           CD579839           |                               |
|                       |           BI094009           |                               |
|                       |           CD248285           |                               |
|                       |             F06539           |                               |
|                       |           BG396393           |                               |
|                       |           AA316451           |                               |
|                       |           BQ893159           |                               |
|                       |           BQ879721           |                               |
|                       |           BI764598           |                               |
|                       |           CN292276           |                               |
|                       |           BG951941           |                               |
|                       |           CB044273           |                               |
|                       |           BG758110           |                               |
|                       |           CB044331           |                               |
|                       |           BM842977           |                               |
|                       |           BP281469           |                               |
|                       |           BP281470           |                               |
|                       |           BG106188           |                               |
|                       |           BG703745           |                               |
|                       |           AA025133           |                               |
|                       |           BE252631           |                               |
|                       |           BM147247           |                               |
|                       |           BG575333           |                               |
|                       |           BG329952           |                               |
|                       |           CN292280           |                               |
|                       |           BG723953           |                               |
|                       |           BG719376           |                               |
|                       |           AW937995           |                               |
|                       |           BG171241           |                               |
|                       |           CF132509           |                               |
|                       |             D31364           |                               |
|                       |           BP359923           |                               |
|                       |           BE278503           |                               |
|                       |           BG822744           |                               |
|                       |           CF144706           |                               |
|                       |             W40409           |                               |
|                       |           BQ694258           |                               |
|                       |           CN292269           |                               |
|                       |           BM781594           |                               |
|                       |           CN292278           |                               |
|                       |           BE258223           |                               |
|                       |           BG704067           |                               |
|                       |           BM194166           |                               |
|                       |           CN292277           |                               |
|                       |           AW452638           |                               |
|                       |           AV695394           |                               |
|                       |           BQ643297           |                               |
|                       |           CN292281           |                               |
|                       |           AL535673           |                               |
|                       |           CN481659           |                               |
|                       |           AA025162           |                               |
|                       |           BF312696           |                               |
|                       |           AW402454           |                               |
|                       |           BE259322           |                               |
|                       |           CN292262           |                               |
|                       |           CN292267           |                               |
|                       |           AA355985           |                               |
|                       |           BM465651           |                               |
|                       |           BE931209           |                               |
|                       |           BE018832           |                               |
|                       |           AA187871           |                               |
|                       |           AA311010           |                               |
|                       |           BE263778           |                               |
|                       |           BG986046           |                               |
|                       |           BM844389           |                               |
|                       |           BI547432           |                               |
|                       |           AA322826           |                               |
|                       |           AA356156           |                               |
|                       |           AU099501           |                               |
+-----------------------+------------------------------+-------------------------------+
|      #   95           | *Seq. mapped in strand Plus* | *Seq. mapped in strand Plus*  |
|                       |           AJ131245           |          AK047352             |
|                       |           BC040137           |                               |
|                       |           CR605949           |                               |
|                       | *Seq. mapped in strand Minus*| *Seq. mapped in strand Minus* |
|                       |           BC009800           |          AK089573             |
+-----------------------+------------------------------+-------------------------------+
|      #   96           | *Seq. mapped in strand Plus* | *Seq. mapped in strand Plus*  |
|                       |           BX109020           |          AW120647             |
|                       |                              |          AK078299             |
|                       |                              |          AK076775             |
|                       |                              |          BG092696             |
|                       |                              |          AI662230             |
|                       |                              |          BG229946             |
|                       | *Seq. mapped in strand Minus*| *Seq. mapped in strand Minus* |
|                       |             N66802           |          AW490392             |
|                       |           BI496461           |                               |
|                       |             R39111           |                               |
|                       |           CA435467           |                               |
|                       |           CA449499           |                               |
|                       |           AV645754           |                               |
|                       |             X63741           |                               |
|                       |             S40832           |                               |
|                       |           AW014038           |                               |
|                       |           AI379641           |                               |
|                       |           AI872362           |                               |
|                       |           CA443276           |                               |
|                       |           BP310746           |                               |
+-----------------------+------------------------------+-------------------------------+
|      #   97           | *Seq. mapped in strand Plus* | *Seq. mapped in strand Plus*  |
|                       |           AK057317           |          AK017430             |
|                       | *Seq. mapped in strand Minus*| *Seq. mapped in strand Minus* |
|                       |           AK127748           |          AF348465             |
|                       |           AL834182           |            Y14634             |
|                       |             U57352           |            U57353             |
|                       |           BC075042           |                               |
|                       |           BC075043           |                               |
|                       |             U50352           |                               |
|                       |             U53212           |                               |
+-----------------------+------------------------------+-------------------------------+
|      #   98           | *Seq. mapped in strand Plus* | *Seq. mapped in strand Plus*  |
|                       |           AK097693           |            X74266             |
|                       |           CD013948           |            U28244             |
|                       |           CD013949           |          BC045156             |
|                       |           CD013951           |            U32359             |
|                       |           CR607562           |                               |
|                       |           BX345779           |                               |
|                       |           AA293827           |                               |
|                       |           AV709457           |                               |
|                       |           BX389673           |                               |
|                       |           BC036792           |                               |
|                       |             U03090           |                               |
|                       |           CK003597           |                               |
|                       |           BM685655           |                               |
|                       |           AI349447           |                               |
|                       |           BX396083           |                               |
|                       |           BM688463           |                               |
|                       |           BM688506           |                               |
|                       |           AJ711621           |                               |
|                       |           AI701153           |                               |
|                       | *Seq. mapped in strand Minus*| *Seq. mapped in strand Minus* |
|                       |           AJ710468           |          AK090021             |
|                       |                              |          BC030899             |
|                       |                              |          AF162713             |
|                       |                              |            U66873             |
+-----------------------+------------------------------+-------------------------------+
|      #   99           | *Seq. mapped in strand Plus* | *Seq. mapped in strand Plus*  |
|                       |           BF514472           |          BY542401             |
|                       | *Seq. mapped in strand Minus*| *Seq. mapped in strand Minus* |
|                       |           AI991048           |          BB804429             |
|                       |           BU617774           |          BY687254             |
|                       |           AW452849           |          BY536891             |
|                       |           AI369891           |          BY535814             |
|                       |           AI559726           |          BY535034             |
|                       |           AI453685           |          BY535200             |
|                       |           BM903533           |          BB754715             |
|                       |           AI653850           |          BF450083             |
|                       |           BC023537           |          BX523711             |
|                       |           AW075769           |          BM212579             |
|                       |           BF475727           |          BM222850             |
|                       |           AI798483           |          CB590215             |
|                       |           AA293665           |          AW538723             |
|                       |           AA573879           |          BG063690             |
|                       |           AI983691           |          BM224536             |
|                       |           CR624236           |          AW541734             |
|                       |           BF197241           |          AW214375             |
|                       |           AI951251           |          BC064775             |
|                       |           BU624491           |          BF462640             |
|                       |           BC014979           |          BF462046             |
|                       |           CR745354           |          BY687369             |
|                       |           CA433893           |          AI463704             |
|                       |           AW262156           |          BI663935             |
|                       |           AI183550           |          BY517834             |
|                       |           BU727619           |          BB736563             |
|                       |             W67809           |          BB736674             |
|                       |           BM681065           |          BB812350             |
|                       |           AA836079           |          BY593419             |
|                       |           AA514399           |          BY442702             |
|                       |           BU684941           |          BB836653             |
|                       |           AI619837           |          BY534772             |
|                       |           AW083739           |          BY528309             |
|                       |           CA310745           |          BY686381             |
|                       |           AA837066           |          BY660942             |
|                       |           AW449424           |          BY513541             |
|                       |             H28711           |          BY602686             |
|                       |           AI089559           |          BY698103             |
|                       |           BI856786           |          BB731823             |
|                       |           AI824515           |          BB731737             |
|                       |           BQ440129           |          BY521023             |
|                       |           BX096027           |          BB826006             |
|                       |           CA426698           |          BY384579             |
|                       |           BQ011049           |          BM116803             |
|                       |           CD369430           |          BB779842             |
|                       |           AI740872           |          BY590699             |
|                       |           CF529253           |          BB832968             |
|                       |           BM982158           |          BY503825             |
|                       |           BC001502           |          BB763087             |
|                       |             L11669           |          BQ032173             |
|                       |           BX330993           |          BM221394             |
|                       |           CR622686           |          BQ939195             |
|                       |           BX460146           |          AK003763             |
|                       |           BP294793           |          BC023012             |
|                       |           BX454175           |          AK018736             |
|                       |           CR626767           |          AK089055             |
|                       |           AL523017           |          BB817683             |
|                       |           CR615910           |          AW125842             |
|                       |           BG763870           |          BB763664             |
|                       |           CR594805           |          BY534504             |
|                       |           AL562092           |          BY534097             |
|                       |           CR617179           |          BB784326             |
|                       |           CR595403           |          BY546053             |
|                       |           BX356809           |          BY553022             |
|                       |           CR600857           |          BB774301             |
|                       |           CR607704           |          BY535571             |
|                       |           BX401013           |          BY539340             |
|                       |           CR599208           |          BY548325             |
|                       |           CR592738           |          BY551372             |
|                       |           CR624960           |          BY535094             |
|                       |           BX351105           |          BY543886             |
|                       |           BX335965           |          BY555689             |
|                       |           BX333730           |          BB775630             |
|                       |           CR617483           |          BY531172             |
|                       |           CR599486           |          BB765635             |
|                       |           CR610651           |          BY763525             |
|                       |           BX359189           |          BB764552             |
|                       |           CR601259           |          BI412400             |
|                       |           CR607301           |          AA068372             |
|                       |           BX414102           |          BG973920             |
|                       |           BF570459           |          BG976148             |
|                       |           BX380002           |          BI686251             |
|                       |           CR617484           |          BG977021             |
|                       |           BX427418           |          BI109479             |
|                       |           CR609779           |          AA734790             |
|                       |           BF569956           |          BY704024             |
|                       |           BG437969           |          AA387954             |
|                       |           BX397762           |          AA822288             |
|                       |           AL577886           |          BE382248             |
|                       |           AL523191           |          BM934678             |
|                       |           BX365848           |          AA267919             |
|                       |           BX386665           |          BQ936092             |
|                       |           AL560663           |          CA325759             |
|                       |           CR614036           |          AA104143             |
|                       |           AL546527           |          BF160109             |
|                       |           AJ712760           |          AI121730             |
|                       |           BI160505           |          BX520348             |
|                       |             W67864           |          AA873967             |
|                       |           CA488150           |            W65060             |
|                       |           BG830572           |          BI156801             |
|                       |           BF569730           |          BE572102             |
|                       |           CF128881           |          BG914636             |
|                       |           BF569602           |          AA608295             |
|                       |           BX370587           |          BG920164             |
|                       |           BM907012           |          AA874113             |
|                       |           AI348333           |          CA319819             |
|                       |           BG761524           |          CN689885             |
|                       |           BM699657           |          AL117994             |
|                       |           BQ926489           |          BQ714422             |
|                       |           BG288807           |          BG077074             |
|                       |           BM687270           |          BI691257             |
|                       |           BU845278           |          BQ885016             |
|                       |           CN300884           |          CB193315             |
|                       |           BI770101           |          BI327747             |
|                       |           BF529072           |          BY702959             |
|                       |           BM727630           |          BU613070             |
|                       |           AA341303           |          BQ945116             |
|                       |           CD519512           |          CD545110             |
|                       |           AA292232           |          BB862255             |
|                       |           AA291605           |          BY007350             |
|                       |           BI192689           |          BI694560             |
|                       |           AW795057           |          BG973888             |
|                       |             W06831           |          BY311629             |
|                       |           BM808379           |          BY233656             |
|                       |           BE781891           |          CK789972             |
|                       |           AI962916           |          CK128856             |
|                       |           BU931617           |          BE531248             |
|                       |           BG747364           |          BQ892168             |
|                       |           BG821148           |          BY747463             |
|                       |           BG822862           |          BQ179691             |
|                       |           BM044712           |          BB567232             |
|                       |           BI014981           |          BG293533             |
|                       |           BE909068           |          BI662524             |
|                       |           BQ961657           |          CA549858             |
|                       |           BQ636769           |          BY205723             |
|                       |           BQ718463           |          AA166021             |
|                       |           BX380003           |          CA555274             |
|                       |           BI918864           |          BY299581             |
|                       |           BE743576           |          CA546200             |
|                       |           CD672686           |          CF158426             |
|                       |           CN300885           |          BF449704             |
|                       |           BQ877214           |          BY299207             |
|                       |           BE739885           |          AA103305             |
|                       |           BX386666           |          BY188905             |
|                       |           BE314035           |          BB854432             |
|                       |             Z20576           |          BY183246             |
|                       |           AL523192           |          BB839864             |
|                       |           BM010414           |          BB843099             |
|                       |           BX383307           |          CA545986             |
|                       |           BX397763           |          BY031042             |
|                       |           AA359361           |          BY170591             |
|                       |           BI013595           |          CF156230             |
|                       |           BE872409           |          BY320392             |
|                       |           BE273404           |          BY168572             |
|                       |           AL557981           |          CD559950             |
|                       |           BX356810           |          BY183745             |
|                       |           BX366056           |          BY189203             |
|                       |           BX356906           |          CF159309             |
|                       |           BI917343           |          CA543727             |
|                       |           BX365849           |          BY201387             |
|                       |           BM050730           |          BB859694             |
|                       |           BX335966           |          BY175948             |
|                       |           BF038091           |          BY201774             |
|                       |           AL520979           |          BY185568             |
|                       |           BX354070           |          BY027190             |
|                       |           BI915203           |          BY176727             |
|                       |           AL522372           |          BB872105             |
|                       |           AL560572           |          BY230351             |
|                       |           BX401014           |          CA544778             |
|                       |           AL520316           |          BY204073             |
|                       |           AL555360           |          BY226168             |
|                       |           BG323741           |          BY020313             |
|                       |           BI761794           |          BY024722             |
|                       |           BG469948           |          BY783515             |
|                       |           BI770376           |          BY188466             |
|                       |           BG470082           |          BY192699             |
|                       |           BI761658           |          BY231771             |
|                       |           BE908115           |          BY189140             |
|                       |           BX384091           |          BY351240             |
|                       |           BX421558           |          BY174914             |
|                       |           AL527457           |          BY209448             |
|                       |           BI764108           |          BY202621             |
|                       |           BX354418           |          BY182528             |
|                       |           BI760819           |          BY193773             |
|                       |           BX358116           |          BY196627             |
|                       |           BI523996           |          BY197040             |
|                       |           BX359190           |          BY134667             |
|                       |           BI912452           |          BY776492             |
|                       |           BI838763           |          BY189665             |
|                       |           BI761384           |          BY310920             |
|                       |           BI836578           |          BY189312             |
|                       |           BI769657           |          BY209755             |
|                       |           BI913808           |          BY134916             |
|                       |           BI762298           |          BY322588             |
|                       |           CN484357           |          BY207182             |
|                       |           BI764110           |          BY151632             |
|                       |           BI918966           |          BY793877             |
|                       |             R83587           |          BY352153             |
|                       |           BI757954           |          BY771241             |
|                       |           BI836823           |          BY009007             |
|                       |           BI818107           |          BY009578             |
|                       |             R70268           |          BB843014             |
|                       |           BG751901           |          BY188192             |
|                       |           BI915688           |          BY190920             |
|                       |           AL523018           |          BY350684             |
|                       |             R35372           |          BY138040             |
|                       |           BX336018           |          BY350711             |
|                       |           BI916585           |          BY196146             |
|                       |           BG757205           |          BY235696             |
|                       |           BI908479           |          BY008554             |
|                       |           CA454021           |          BY013652             |
|                       |           BQ421963           |          BY197727             |
|                       |           AA339235           |          BY145843             |
|                       |           BI770068           |          BY350384             |
|                       |           BI769671           |          BY189899             |
|                       |           BI764749           |          BY200540             |
|                       |           BI760602           |                               |
|                       |           BI916553           |                               |
|                       |           BG479143           |                               |
|                       |           BI762799           |                               |
|                       |           BM918354           |                               |
|                       |           BE311885           |                               |
|                       |           BE876378           |                               |
|                       |           BI765998           |                               |
|                       |           BX374626           |                               |
|                       |           BE879994           |                               |
|                       |           BF975662           |                               |
|                       |           BG755349           |                               |
|                       |           BG326480           |                               |
|                       |           BM552134           |                               |
|                       |           CA455021           |                               |
|                       |           BI839052           |                               |
|                       |           BI764742           |                               |
|                       |           BI818977           |                               |
|                       |           BF949231           |                               |
|                       |           CA406853           |                               |
|                       |             H28841           |                               |
|                       |           BI770924           |                               |
|                       |           BG746445           |                               |
|                       |           BX403090           |                               |
|                       |           BI908686           |                               |
|                       |           BI916104           |                               |
|                       |           CD516273           |                               |
|                       |           BI771627           |                               |
|                       |           BI771742           |                               |
|                       |           BG420086           |                               |
|                       |           BE261271           |                               |
|                       |           BE279028           |                               |
|                       |             R20994           |                               |
|                       |           BI909762           |                               |
|                       |           BE292804           |                               |
|                       |           BQ314942           |                               |
|                       |           BQ937108           |                               |
|                       |           BF949229           |                               |
|                       |           BE278575           |                               |
|                       |           CB851774           |                               |
|                       |           BQ935242           |                               |
|                       |           BU850208           |                               |
|                       |           BF206523           |                               |
|                       |           CD514680           |                               |
|                       |           CB852374           |                               |
|                       |           BE264386           |                               |
|                       |           BQ427677           |                               |
|                       |           BI819886           |                               |
|                       |           CV027574           |                               |
|                       |           BI769550           |                               |
|                       |           BI772139           |                               |
|                       |           BF304430           |                               |
|                       |           BM842823           |                               |
|                       |           BI909662           |                               |
|                       |           AL138160           |                               |
|                       |           BI911491           |                               |
|                       |             H14473           |                               |
|                       |           BG419734           |                               |
|                       |           BX424582           |                               |
|                       |           CB851475           |                               |
|                       |           CN300887           |                               |
|                       |           BM842517           |                               |
|                       |           AW732621           |                               |
|                       |           BM844033           |                               |
|                       |           CN422014           |                               |
+-----------------------+------------------------------+-------------------------------+
|      #  100           | *Seq. mapped in strand Plus* | *Seq. mapped in strand Plus*  |
|                       |           BC059399           |          AK040923             |
|                       |           BC013149           |          BB636945             |
|                       |           AK131101           |          CA894332             |
|                       |                              |          CK622587             |
|                       |                              |          BY242905             |
|                       |                              |          BC028937             |
|                       |                              |          BY358163             |
|                       |                              |          AW012678             |
|                       |                              |          BY342849             |
|                       |                              |          BU840502             |
|                       |                              |          CN704272             |
|                       |                              |          BY153834             |
|                       |                              |          BY036415             |
|                       |                              |          BY785310             |
|                       |                              |          BY790754             |
|                       |                              |          BY780680             |
|                       |                              |          BY768233             |
|                       |                              |          BY771643             |
|                       |                              |          BY175874             |
|                       |                              |          BY258822             |
|                       |                              |          BY246874             |
|                       |                              |          BY254084             |
|                       |                              |          BM950667             |
|                       |                              |          BY231372             |
|                       |                              |          BM947017             |
|                       |                              |          BY197930             |
|                       |                              |          BB849308             |
|                       |                              |          BY187457             |
|                       |                              |          BY177656             |
|                       |                              |            U15976             |
|                       |                              |          BG866381             |
|                       |                              |          CA882263             |
|                       |                              |          BF581973             |
|                       |                              |          CA568210             |
|                       |                              |          CN680913             |
|                       |                              |          BU148823             |
|                       |                              |          AA717985             |
|                       |                              |          BY092637             |
|                       |                              |          BY162243             |
|                       |                              |          AI323090             |
|                       |                              |          BM947614             |
|                       |                              |          CF909605             |
|                       |                              |          CA532543             |
|                       |                              |          CD803559             |
|                       | *Seq. mapped in strand Minus*| *Seq. mapped in strand Minus* |
|                       |           AK125289           |          BF467261             |
+-----------------------+------------------------------+-------------------------------+
|      #  101           | *Seq. mapped in strand Plus* | *Seq. mapped in strand Plus*  |
|                       |           BC014592           |          BC059910             |
|                       |           CR606005           |          AK004898             |
|                       |           CR597542           |                               |
|                       |           AF302786           |                               |
|                       |           AY203933           |                               |
|                       |           CR612240           |                               |
|                       |           AK126110           |                               |
|                       |           CR618970           |                               |
|                       | *Seq. mapped in strand Minus*| *Seq. mapped in strand Minus* |
|                       |           AK124977           |          AK078230             |
|                       |           AK024842           |          BC055872             |
|                       |           AK022685           |          AK044021             |
|                       |           BC000150           |          BC023869             |
|                       |           AK027013           |          AY465529             |
|                       |           BC011924           |                               |
+-----------------------+------------------------------+-------------------------------+
|      #  102           | *Seq. mapped in strand Plus* | *Seq. mapped in strand Plus*  |
|                       |           AV752295           |          AK036251             |
|                       |           BG202613           |                               |
|                       |           BC069298           |                               |
|                       |           AV745295           |                               |
|                       | *Seq. mapped in strand Minus*| *Seq. mapped in strand Minus* |
|                       |           CK822742           |            J00644             |
+-----------------------+------------------------------+-------------------------------+
|      #  103           | *Seq. mapped in strand Plus* | *Seq. mapped in strand Plus*  |
|                       |           AK127073           |          AK046260             |
|                       |           BC040547           |          AK046222             |
|                       |           AK054846           |          AK045995             |
|                       |           CR601310           |          AK040216             |
|                       |           AK098068           |          BC050757             |
|                       |           BC016615           |          AK039716             |
|                       |           AK057069           |          AK050483             |
|                       |                              |          AF233582             |
|                       | *Seq. mapped in strand Minus*| *Seq. mapped in strand Minus* |
|                       |           AF251706           |          BC057864             |
|                       |           AY358545           |          AF251703             |
|                       |           AK092757           |          AY214460             |
|                       |           BC028199           |          AY457047             |
|                       |           AF375480           |          AK089313             |
|                       |           AF375481           |          AK089465             |
|                       |           AY303545           |                               |
+-----------------------+------------------------------+-------------------------------+
|      #  104           | *Seq. mapped in strand Plus* | *Seq. mapped in strand Plus*  |
|                       |           AK125895           |          AK086699             |
|                       |                              |          BC009153             |
|                       |                              |          AK050860             |
|                       | *Seq. mapped in strand Minus*| *Seq. mapped in strand Minus* |
|                       |           BC007852           |          BC029875             |
|                       |           BC015793           |          BC052913             |
|                       |             D63780           |          AF004934             |
|                       |           AK092161           |          BC071218             |
|                       |           CR623111           |                               |
|                       |           CR618964           |                               |
|                       |             X99325           |                               |
|                       |           CR614826           |                               |
|                       |           CR621330           |                               |
+-----------------------+------------------------------+-------------------------------+
|      #  105           | *Seq. mapped in strand Plus* | *Seq. mapped in strand Plus*  |
|                       |           BU607934           |          BY785690             |
|                       |                              |          BY143144             |
|                       |                              |          BB849971             |
|                       |                              |          BY773818             |
|                       |                              |          BY016931             |
|                       |                              |          BY016309             |
|                       |                              |          BY067829             |
|                       |                              |          BF537939             |
|                       |                              |          BC015076             |
|                       |                              |          BB623257             |
|                       |                              |          AK032788             |
|                       |                              |          AI509200             |
|                       |                              |          AA288427             |
|                       |                              |          AF030454             |
|                       |                              |          BB651985             |
|                       |                              |          BY784205             |
|                       |                              |          BY736694             |
|                       |                              |          BY035747             |
|                       |                              |          BY021718             |
|                       |                              |          BY036637             |
|                       |                              |          BB610612             |
|                       |                              |          AA914653             |
|                       |                              |          BB864596             |
|                       |                              |          BY149357             |
|                       |                              |          BY031538             |
|                       |                              |          CA947825             |
|                       |                              |          BB605128             |
|                       |                              |          BY734968             |
|                       |                              |          BY018475             |
|                       |                              |          CA558928             |
|                       |                              |          CB194576             |
|                       |                              |          BY145124             |
|                       |                              |          BY791860             |
|                       |                              |          BY766876             |
|                       |                              |          BY016647             |
|                       |                              |          BY020603             |
|                       |                              |          BY017742             |
|                       |                              |          CA977300             |
|                       |                              |          CD540485             |
|                       |                              |          CN682166             |
|                       |                              |          CN684752             |
|                       |                              |          BF161001             |
|                       | *Seq. mapped in strand Minus*| *Seq. mapped in strand Minus* |
|                       |           AW473150           |          CD741741             |
|                       |           AW073724           |                               |
|                       |           AI623931           |                               |
|                       |           AI003314           |                               |
|                       |           CB243616           |                               |
|                       |           AI961901           |                               |
|                       |           BE049289           |                               |
|                       |           AF275945           |                               |
|                       |           CB243013           |                               |
|                       |           AW629661           |                               |
|                       |           BM978947           |                               |
|                       |           BU608143           |                               |
|                       |           BQ788092           |                               |
|                       |           AI500549           |                               |
|                       |           BM983281           |                               |
|                       |           CA314504           |                               |
|                       |           CA313145           |                               |
|                       |           BQ272389           |                               |
|                       |           BQ006436           |                               |
|                       |           CA448496           |                               |
|                       |           CB854240           |                               |
|                       |           AA860685           |                               |
|                       |           AI458449           |                               |
|                       |           AF304447           |                               |
|                       |           CR626217           |                               |
|                       |           AA768426           |                               |
|                       |           BG291993           |                               |
|                       |           BI334115           |                               |
|                       |           AY359061           |                               |
|                       |           AF030455           |                               |
|                       |           BP327230           |                               |
|                       |           BX503380           |                               |
|                       |           AI220250           |                               |
|                       |           BG260457           |                               |
|                       |           BU687681           |                               |
|                       |           CB250687           |                               |
|                       |           BF340135           |                               |
|                       |           BU942623           |                               |
|                       |           AA781956           |                               |
|                       |           BF437750           |                               |
|                       |           AW517161           |                               |
|                       |           BC017774           |                               |
|                       |           BM975702           |                               |
|                       |           CB306409           |                               |
|                       |           AW170355           |                               |
|                       |           BI494985           |                               |
|                       |           BI494986           |                               |
|                       |           BI762480           |                               |
|                       |           BU192146           |                               |
|                       |           BI908570           |                               |
|                       |           BX100668           |                               |
|                       |           CN359444           |                               |
|                       |           CR590568           |                               |
|                       |           AL557855           |                               |
|                       |           AA304933           |                               |
|                       |           BM353656           |                               |
|                       |           BG402955           |                               |
|                       |           CD637192           |                               |
|                       |           CD637193           |                               |
|                       |           CD637190           |                               |
|                       |           CD637191           |                               |
|                       |           CD694940           |                               |
|                       |           BG539357           |                               |
|                       |           BI761456           |                               |
|                       |           BE004003           |                               |
|                       |           AL602381           |                               |
|                       |           BM353936           |                               |
|                       |           AA088807           |                               |
|                       |           BQ719162           |                               |
|                       |           BM686862           |                               |
|                       |           CD702563           |                               |
|                       |             H48606           |                               |
|                       |           AL602357           |                               |
|                       |           BG535766           |                               |
|                       |           AL708643           |                               |
|                       |           AL602532           |                               |
|                       |           AL700734           |                               |
|                       |           CD704203           |                               |
|                       |             W61215           |                               |
|                       |           BX339461           |                               |
|                       |             H77296           |                               |
|                       |           AL710571           |                               |
|                       |           BX470878           |                               |
|                       |           BQ777419           |                               |
|                       |           AL709590           |                               |
|                       |           BM763627           |                               |
|                       |           AA336903           |                               |
|                       |           BE140648           |                               |
|                       |           AU100149           |                               |
|                       |           AL602699           |                               |
+-----------------------+------------------------------+-------------------------------+
|      #  106           | *Seq. mapped in strand Plus* | *Seq. mapped in strand Plus*  |
|                       |           BC021693           |          BQ747061             |
|                       |                              |          CA319038             |
|                       |                              |          BQ570407             |
|                       |                              |          CA318825             |
|                       |                              |          BQ044275             |
|                       | *Seq. mapped in strand Minus*| *Seq. mapped in strand Minus* |
|                       |           BC033674           |          AF272859             |
|                       |           AK058099           |          AK016403             |
|                       |           AK093453           |          BY716055             |
|                       |           AF272858           |          AK016125             |
|                       |           AK098824           |          BY715726             |
+-----------------------+------------------------------+-------------------------------+
|      #  107           | *Seq. mapped in strand Plus* | *Seq. mapped in strand Plus*  |
|                       |           AK056562           |          BY324791             |
|                       |                              |          BY091588             |
|                       |                              |          CB600570             |
|                       |                              |          BM943908             |
|                       |                              |          BC054723             |
|                       |                              |          BI734167             |
|                       |                              |          BY320991             |
|                       |                              |          AI616271             |
|                       |                              |          BY717704             |
|                       |                              |          BY299135             |
|                       |                              |          BI736308             |
|                       |                              |          BY707550             |
|                       |                              |          AK007491             |
|                       |                              |          AA445775             |
|                       |                              |          AA794486             |
|                       |                              |          BY048388             |
|                       |                              |          BY793955             |
|                       |                              |          BY788916             |
|                       |                              |          BY054214             |
|                       |                              |          BY040506             |
|                       |                              |          CA492780             |
|                       |                              |          AL360607             |
|                       |                              |          AL360613             |
|                       |                              |          BY037194             |
|                       |                              |          BY033778             |
|                       |                              |          BY154829             |
|                       |                              |          BY021963             |
|                       |                              |          BY057597             |
|                       |                              |          BY044883             |
|                       |                              |          BY038866             |
|                       |                              |          BY225027             |
|                       |                              |          BY017247             |
|                       |                              |          AA546563             |
|                       |                              |          BY718583             |
|                       |                              |          BY736402             |
|                       |                              |          BF608159             |
|                       |                              |          CD772633             |
|                       |                              |          BY056870             |
|                       |                              |          BY055610             |
|                       |                              |          BY318805             |
|                       |                              |          AI892408             |
|                       |                              |          AI325719             |
|                       |                              |          BY003352             |
|                       |                              |          BF531936             |
|                       |                              |          CK030755             |
|                       |                              |          CB723753             |
|                       |                              |          CA568065             |
|                       |                              |          BB656508             |
|                       |                              |          BY074678             |
|                       |                              |          BY066956             |
|                       |                              |          BY311549             |
|                       |                              |          BY067660             |
|                       |                              |          BY025483             |
|                       |                              |          BY059161             |
|                       |                              |          BY061660             |
|                       |                              |          BY042515             |
|                       |                              |          BY047587             |
|                       |                              |          BY097463             |
|                       |                              |          BY048252             |
|                       |                              |          BY072265             |
|                       |                              |          CA562369             |
|                       |                              |          CB055871             |
|                       |                              |          CA319859             |
|                       |                              |          CF897505             |
|                       |                              |          BF163141             |
|                       |                              |          BY040468             |
|                       |                              |          CF900070             |
|                       |                              |          CF951604             |
|                       |                              |          BY014509             |
|                       |                              |          CF900308             |
|                       |                              |          AW229275             |
|                       |                              |          BG862575             |
|                       |                              |          BX515391             |
|                       |                              |          BG245896             |
|                       |                              |          BG175770             |
|                       |                              |            D77075             |
|                       |                              |          AA172989             |
|                       |                              |          CA451582             |
|                       |                              |          BY312494             |
|                       |                              |          BY047059             |
|                       |                              |          CA978677             |
|                       |                              |          CB196331             |
|                       |                              |          AA240378             |
|                       |                              |          BM951971             |
|                       |                              |          BC062876             |
|                       |                              |          BB859443             |
|                       |                              |          BF386008             |
|                       |                              |          BE380192             |
|                       |                              |          BF120634             |
|                       |                              |          BG803023             |
|                       |                              |          BY050882             |
|                       |                              |          AA109203             |
|                       |                              |          CF617447             |
|                       |                              |          BY048461             |
|                       |                              |          BY045985             |
|                       |                              |          BY045519             |
|                       |                              |          BY048134             |
|                       |                              |          BY053926             |
|                       |                              |          BY045518             |
|                       |                              |          BY059178             |
|                       |                              |          BY043974             |
|                       |                              |          BY054283             |
|                       |                              |          BY044998             |
|                       |                              |          BG078525             |
|                       |                              |          BY738566             |
|                       |                              |          CN692478             |
|                       |                              |          BI217367             |
|                       |                              |          BI665007             |
|                       |                              |          BQ108981             |
|                       |                              |          BI736007             |
|                       |                              |          BG795758             |
|                       |                              |          AA032967             |
|                       |                              |          AA921002             |
|                       |                              |            W47978             |
|                       |                              |          BM937875             |
|                       |                              |          BG144097             |
|                       |                              |          BI905472             |
|                       |                              |          BI408978             |
|                       |                              |          BI408678             |
|                       |                              |          CF950468             |
|                       |                              |          CF750309             |
|                       |                              |          CA316500             |
|                       |                              |          BE376718             |
|                       |                              |          BQ964447             |
|                       |                              |          BI411080             |
|                       |                              |          BI415855             |
|                       |                              |          BI414650             |
|                       |                              |          AI876379             |
|                       |                              |          BI526115             |
|                       |                              |          BB619672             |
|                       |                              |          BI409390             |
|                       |                              |          AA675618             |
|                       |                              |          BG277477             |
|                       |                              |          CD540647             |
|                       |                              |          CD541748             |
|                       |                              |          CD540943             |
|                       |                              |          CK031363             |
|                       |                              |          BI525930             |
|                       |                              |          BF011616             |
|                       |                              |          BG148826             |
|                       |                              |          BB859775             |
|                       |                              |          AA110818             |
|                       |                              |          BG863383             |
|                       |                              |          CA540685             |
|                       |                              |          CA540725             |
|                       |                              |          AA666756             |
|                       |                              |          CB249243             |
|                       |                              |          CN843155             |
|                       |                              |          BU936428             |
|                       |                              |          AI595018             |
|                       |                              |          BM055809             |
|                       |                              |          AA537058             |
|                       |                              |          BY756776             |
|                       |                              |          BI408917             |
|                       |                              |          AA067801             |
|                       |                              |          BG065237             |
|                       |                              |          BG094906             |
|                       |                              |          AW537050             |
|                       |                              |          BY758872             |
|                       |                              |          BF147479             |
|                       |                              |          AI845934             |
|                       |                              |          BU756596             |
|                       |                              |          BM230733             |
|                       |                              |          CD774118             |
|                       |                              |          AW539448             |
|                       |                              |            C81306             |
|                       |                              |          AV264985             |
|                       |                              |          BM055526             |
|                       |                              |          CK333342             |
|                       |                              |          CA539514             |
|                       |                              |          BI790013             |
|                       |                              |          BM206247             |
|                       |                              |          BM206289             |
|                       |                              |          AI429134             |
|                       |                              |          AW537089             |
|                       |                              |          BI989389             |
|                       |                              |          BB702298             |
|                       |                              |          AW544892             |
|                       |                              |          BG243274             |
|                       |                              |          BY412394             |
|                       |                              |          CD540646             |
|                       |                              |          BY382944             |
|                       |                              |          BY413990             |
|                       |                              |          CD541050             |
|                       |                              |          BY413979             |
|                       |                              |          BY388682             |
|                       |                              |          BB834477             |
|                       |                              |          BB774045             |
|                       |                              |          BG089554             |
|                       |                              |          BY483101             |
|                       |                              |          BY460018             |
|                       |                              |          BB818875             |
|                       |                              |          BB828009             |
|                       |                              |          BY443494             |
|                       |                              |          BB774404             |
|                       |                              |          BE954238             |
|                       |                              |          BY574240             |
|                       |                              |          BY394475             |
|                       |                              |          BY698020             |
|                       |                              |          AU015663             |
|                       |                              |          BY412817             |
|                       |                              |          AI644970             |
|                       |                              |          AA655934             |
|                       |                              |          BE953176             |
|                       |                              |          BY577438             |
|                       |                              |          BY574831             |
|                       |                              |          BY410867             |
|                       |                              |          BY687134             |
|                       |                              |          BY587940             |
|                       |                              |          BY696113             |
|                       |                              |          BY404268             |
|                       |                              |          BY401962             |
|                       |                              |          BY401963             |
|                       |                              |          BY688284             |
|                       |                              |          AI585825             |
|                       |                              |          BB782529             |
|                       |                              |          BY499068             |
|                       |                              |          BY404424             |
|                       |                              |          BY575292             |
|                       |                              |          BY406747             |
|                       |                              |          BY575716             |
|                       |                              |          BY400781             |
|                       |                              |          BB791937             |
|                       |                              |          BY665900             |
|                       |                              |          BY586135             |
|                       |                              |          BY405782             |
|                       |                              |          BY404651             |
|                       |                              |          BY483542             |
|                       |                              |          BY404577             |
|                       |                              |          BY403948             |
|                       |                              |          BY664648             |
|                       |                              |          AW554474             |
|                       |                              |          BY414181             |
|                       |                              |          BY683083             |
|                       |                              |          AW540056             |
|                       |                              |          BY408020             |
|                       |                              |          BY397664             |
|                       |                              |          BY409286             |
|                       |                              |          BB813427             |
|                       |                              |          BY401268             |
|                       |                              |          BY396057             |
|                       |                              |          BY605465             |
|                       |                              |          CD541747             |
|                       |                              |          BY425933             |
|                       |                              |          BY400517             |
|                       |                              |          BY396679             |
|                       |                              |          BY397886             |
|                       |                              |          BY409648             |
|                       |                              |          BY416329             |
|                       |                              |          BY409738             |
|                       |                              |          BY399091             |
|                       |                              |          BY409651             |
|                       |                              |          BY403821             |
|                       |                              |          BY398718             |
|                       |                              |          BY407166             |
|                       |                              |          BU899015             |
|                       |                              |          AV093791             |
|                       |                              |          AV056736             |
|                       |                              |          AV121186             |
|                       |                              |          AV079291             |
|                       |                              |          AV142964             |
|                       |                              |          BB717832             |
|                       |                              |            C80358             |
|                       |                              |          AV378319             |
|                       |                              |          AV249608             |
|                       |                              |          AV295615             |
|                       |                              |          AV112783             |
|                       |                              |          AV110496             |
|                       |                              |          AV284262             |
|                       |                              |          AV158102             |
|                       |                              |          AV243697             |
|                       |                              |          CF353234             |
|                       |                              |          CF424433             |
|                       |                              |          CK377336             |
|                       |                              |          CK428752             |
|                       |                              |          CF355734             |
|                       |                              |          CF424267             |
|                       | *Seq. mapped in strand Minus*| *Seq. mapped in strand Minus* |
|                       |             U86602           |          AI452178             |
|                       |           CR605280           |          AA123023             |
|                       |           BC009175           |                               |
|                       |           CR613349           |                               |
|                       |           BC073924           |                               |
|                       |           CR616272           |                               |
|                       |           CR613176           |                               |
|                       |           CR605987           |                               |
|                       |           CR590575           |                               |
|                       |           CR614881           |                               |
|                       |           CR456975           |                               |
|                       |           BT006920           |                               |
+-----------------------+------------------------------+-------------------------------+
|      #  108           | *Seq. mapped in strand Plus* | *Seq. mapped in strand Plus*  |
|                       |           BQ184645           |          BG145062             |
|                       | *Seq. mapped in strand Minus*| *Seq. mapped in strand Minus* |
|                       |           AI050023           |            W29611             |
|                       |           AW022189           |          AB000803             |
|                       |           AW075850           |            D87747             |
|                       |           AI582682           |          BC031665             |
|                       |             T29035           |            X99582             |
|                       |             H29001           |            U59760             |
|                       |           AW193726           |            Z80112             |
|                       |           AW594481           |          BU523025             |
|                       |           AI683322           |          BG173867             |
|                       |           BQ025781           |          BF101953             |
|                       |           AI358446           |          BG915636             |
|                       |           AI675038           |          BG174412             |
|                       |           AI866909           |          AW227957             |
|                       |           AI920946           |            Z80111             |
|                       |           CA436565           |          BX513842             |
|                       |           BQ024573           |          BY041153             |
|                       |           AI300807           |          BB872868             |
|                       |           AI344724           |          BQ265435             |
|                       |           AA426566           |            W88094             |
|                       |           AA426644           |                               |
|                       |           AI159856           |                               |
|                       |           AA760743           |                               |
|                       |           AI583169           |                               |
|                       |           AI434652           |                               |
|                       |           AI249788           |                               |
|                       |           AA824607           |                               |
|                       |           AA804282           |                               |
|                       |           AA731086           |                               |
|                       |           AA743645           |                               |
|                       |           AA747545           |                               |
|                       |           AA411265           |                               |
|                       |           BU682354           |                               |
|                       |           BM971140           |                               |
|                       |           BM978372           |                               |
|                       |           BQ446320           |                               |
|                       |           CA436903           |                               |
|                       |           BQ003324           |                               |
|                       |           AI493618           |                               |
|                       |           CR598681           |                               |
|                       |           CR604071           |                               |
|                       |             D10924           |                               |
|                       |           AA284569           |                               |
|                       |           AF147204           |                               |
|                       |           BC020968           |                               |
|                       |             X71635           |                               |
|                       |             L06797           |                               |
|                       |           AK129916           |                               |
|                       |           CR614663           |                               |
|                       |           AW594595           |                               |
|                       |           CR596547           |                               |
|                       |           CR619476           |                               |
|                       |           CR605131           |                               |
|                       |           CR594428           |                               |
|                       |           CR623838           |                               |
|                       |             M99293           |                               |
|                       |           CR610268           |                               |
|                       |           CR598713           |                               |
|                       |           CR614594           |                               |
|                       |           CR614199           |                               |
|                       |           CR594588           |                               |
|                       |           CR601301           |                               |
|                       |             L01639           |                               |
|                       |           AF025375           |                               |
|                       |           AY242129           |                               |
|                       |           AF348491           |                               |
|                       |           BT006660           |                               |
|                       |           BX396365           |                               |
|                       |           BM545259           |                               |
|                       |           BX397685           |                               |
|                       |           BX418530           |                               |
|                       |           BX421511           |                               |
|                       |           BX462627           |                               |
|                       |           BX353253           |                               |
|                       |           BX421287           |                               |
|                       |           BX358346           |                               |
|                       |           BM920800           |                               |
|                       |           BX462182           |                               |
|                       |           BI824663           |                               |
|                       |           BI754094           |                               |
|                       |           BM051973           |                               |
|                       |           BI762229           |                               |
|                       |           BQ718617           |                               |
|                       |           CD389217           |                               |
|                       |           BX362477           |                               |
|                       |           CA454255           |                               |
|                       |           BI756157           |                               |
|                       |           BP260405           |                               |
|                       |           BI819797           |                               |
|                       |           BX443258           |                               |
|                       |           BX443334           |                               |
|                       |           BX443228           |                               |
|                       |           BI761664           |                               |
|                       |           BU146456           |                               |
|                       |           BI771103           |                               |
|                       |           BU945383           |                               |
|                       |           BX424593           |                               |
|                       |           BG685901           |                               |
|                       |           CO960366           |                               |
|                       |           BI917014           |                               |
|                       |           CB988971           |                               |
|                       |           BI597875           |                               |
|                       |           BI546999           |                               |
|                       |           BF797340           |                               |
|                       |           AU117058           |                               |
|                       |           CF595894           |                               |
|                       |           BQ109515           |                               |
|                       |           BI761118           |                               |
|                       |           BE617402           |                               |
|                       |           BI765768           |                               |
|                       |           BX421959           |                               |
|                       |           CB987769           |                               |
|                       |           CD640668           |                               |
|                       |           BI835125           |                               |
|                       |           CB215245           |                               |
|                       |           BG025793           |                               |
|                       |           CB122606           |                               |
|                       |           BF591285           |                               |
|                       |           CD251240           |                               |
|                       |           BP300248           |                               |
|                       |           BP224898           |                               |
|                       |           BP236598           |                               |
|                       |           BP338360           |                               |
|                       |           BP197054           |                               |
|                       |           BP198585           |                               |
|                       |           BP225804           |                               |
|                       |           BI668010           |                               |
|                       |           BP197755           |                               |
|                       |           BP197789           |                               |
|                       |           BP198110           |                               |
|                       |           BP208818           |                               |
|                       |           BP225182           |                               |
|                       |           BP338386           |                               |
|                       |           BP367587           |                               |
|                       |           BP331003           |                               |
|                       |           BP215948           |                               |
|                       |           BP339598           |                               |
|                       |           BP366593           |                               |
|                       |           BP224524           |                               |
|                       |           BP264409           |                               |
|                       |           BP304515           |                               |
|                       |           AW403941           |                               |
|                       |           AA354393           |                               |
|                       |           CD695230           |                               |
|                       |           BE245301           |                               |
|                       |           AA248174           |                               |
|                       |           AA354040           |                               |
|                       |           BE244758           |                               |
|                       |           BM700824           |                               |
|                       |           AA354044           |                               |
|                       |           BF792200           |                               |
|                       |           BP260206           |                               |
|                       |           AA357257           |                               |
|                       |           AA378777           |                               |
|                       |           AU099377           |                               |
|                       |           AA339027           |                               |
|                       |           AA091989           |                               |
|                       |           AA356857           |                               |
|                       |           BF173003           |                               |
|                       |           BF175266           |                               |
|                       |           AA355098           |                               |
|                       |           AA357215           |                               |
+-----------------------+------------------------------+-------------------------------+
|      #  109           | *Seq. mapped in strand Plus* | *Seq. mapped in strand Plus*  |
|                       |           AA568271           |          BY091433             |
|                       |                              |          BY141880             |
|                       |                              |          BB852317             |
|                       |                              |          BB851402             |
|                       |                              |          BB853963             |
|                       |                              |          BY235649             |
|                       |                              |          BY303091             |
|                       |                              |          BY234822             |
|                       |                              |          BB847980             |
|                       |                              |          BY234888             |
|                       |                              |          BY237091             |
|                       |                              |          BY021958             |
|                       |                              |          BY185164             |
|                       |                              |          BY202141             |
|                       |                              |          BB850911             |
|                       |                              |          BY749487             |
|                       |                              |          BY198093             |
|                       |                              |          BY210266             |
|                       |                              |          BY200132             |
|                       |                              |          BY748654             |
|                       |                              |          BY342642             |
|                       |                              |          BY342479             |
|                       |                              |          BY185782             |
|                       |                              |          BY187982             |
|                       |                              |          BY189709             |
|                       |                              |          BY182014             |
|                       |                              |          BY179734             |
|                       |                              |          BY202619             |
|                       |                              |          BY198926             |
|                       |                              |          BY188555             |
|                       |                              |          BY189368             |
|                       |                              |          BY005969             |
|                       |                              |          AK040894             |
|                       |                              |          CK791297             |
|                       |                              |          BY195850             |
|                       |                              |          BY184383             |
|                       |                              |          BY194564             |
|                       |                              |          BY210753             |
|                       |                              |          BY196594             |
|                       |                              |          BY205041             |
|                       |                              |          BE981239             |
|                       |                              |          BB636882             |
|                       |                              |          BY736438             |
|                       |                              |          AA013529             |
|                       |                              |          AA014165             |
|                       |                              |          BB597965             |
|                       |                              |          AK049868             |
|                       |                              |          BB645898             |
|                       |                              |          BF467400             |
|                       |                              |          BE646889             |
|                       |                              |          AA691717             |
|                       |                              |          BE983536             |
|                       |                              |          BF461047             |
|                       |                              |          BF464819             |
|                       |                              |          AW046007             |
|                       |                              |          AW049925             |
|                       |                              |          CA316471             |
|                       |                              |          AW047247             |
|                       |                              |          BF462067             |
|                       | *Seq. mapped in strand Minus*| *Seq. mapped in strand Minus* |
|                       |           BF594889           |          BY551648             |
|                       |           BQ011880           |          AK003948             |
|                       |           BE046203           |          BE988851             |
|                       |           AA768632           |          BY596166             |
|                       |           BX112508           |          AI854291             |
|                       |             R54356           |          BB752799             |
|                       |           CA432551           |          AK009262             |
|                       |           CK902581           |          AK020083             |
|                       |           BU729155           |          BC011315             |
|                       |           BQ278641           |          BB387859             |
|                       |           CN479692           |          AK082469             |
|                       |           BU579653           |          CK129497             |
|                       |           CD366564           |          AI156187             |
|                       |           BM972823           |          BI415492             |
|                       |           BQ045343           |          BY709059             |
|                       |           BQ576347           |          BU962921             |
|                       |           BQ015202           |          BE687616             |
|                       |           CF541107           |          CK796409             |
|                       |           CD369992           |          BI730889             |
|                       |           BG206320           |          CK619797             |
|                       |           AY632082           |          AI194461             |
|                       |           BM907645           |          BU513511             |
|                       |           AL050269           |          BU936970             |
|                       |           BC004195           |          AI046566             |
|                       |           AK123115           |          CB954827             |
|                       |           CR606498           |          BY103431             |
|                       |           BC004225           |          BY120225             |
|                       |           CR617253           |                               |
|                       |           CR614580           |                               |
|                       |           CR598253           |                               |
|                       |           CR619487           |                               |
|                       |           CR622351           |                               |
|                       |           CR590647           |                               |
|                       |           BG827318           |                               |
|                       |           BU956489           |                               |
|                       |           BG765619           |                               |
|                       |           BX331474           |                               |
|                       |           BP302318           |                               |
|                       |           BX340730           |                               |
|                       |           AL524302           |                               |
|                       |           BX333611           |                               |
|                       |           BX333236           |                               |
|                       |           CN306384           |                               |
|                       |           BU157878           |                               |
|                       |           BM464121           |                               |
|                       |           BX362998           |                               |
|                       |           BQ068039           |                               |
|                       |           BX332703           |                               |
|                       |           BQ058149           |                               |
|                       |           BM563544           |                               |
|                       |           AL558294           |                               |
|                       |           BM909550           |                               |
|                       |           BQ057846           |                               |
|                       |           BQ058372           |                               |
|                       |           BX377962           |                               |
|                       |           CB130416           |                               |
|                       |           BG702300           |                               |
|                       |           BU159218           |                               |
|                       |           AL558792           |                               |
|                       |           BE796550           |                               |
|                       |           CN306385           |                               |
|                       |           CB123661           |                               |
|                       |           BI544459           |                               |
|                       |           BI914046           |                               |
|                       |           BP324844           |                               |
|                       |           BI759170           |                               |
|                       |           BQ422278           |                               |
|                       |           BG769633           |                               |
|                       |           BM470228           |                               |
|                       |           BI085611           |                               |
|                       |           BQ669143           |                               |
|                       |           BX379416           |                               |
|                       |           BI559514           |                               |
|                       |           BX357154           |                               |
|                       |           BI834661           |                               |
|                       |           BE885598           |                               |
|                       |           BI523670           |                               |
|                       |           BM811551           |                               |
|                       |           CB995674           |                               |
|                       |           AL537925           |                               |
|                       |           CB997593           |                               |
|                       |           CB152598           |                               |
|                       |           BE782695           |                               |
|                       |           BF309125           |                               |
|                       |           BG116888           |                               |
|                       |           CB123550           |                               |
|                       |           CB997943           |                               |
|                       |           BQ375660           |                               |
|                       |           BX358640           |                               |
|                       |           BE871454           |                               |
|                       |           BI821280           |                               |
|                       |           BM923904           |                               |
|                       |           BE868656           |                               |
|                       |           CN306386           |                               |
|                       |           BG761603           |                               |
|                       |           BP198259           |                               |
|                       |           BP197633           |                               |
|                       |           CN306387           |                               |
|                       |           CV026173           |                               |
|                       |           BP375143           |                               |
|                       |           BE294493           |                               |
|                       |           BP321829           |                               |
|                       |           BP204122           |                               |
|                       |           BP345315           |                               |
|                       |           BI768160           |                               |
|                       |           BG421012           |                               |
|                       |           BP198136           |                               |
|                       |           BP377550           |                               |
|                       |           BP231776           |                               |
|                       |           BP356674           |                               |
|                       |           CB997391           |                               |
|                       |           CB157105           |                               |
|                       |           CN306391           |                               |
|                       |           BI914273           |                               |
|                       |           BQ653371           |                               |
|                       |           BI825162           |                               |
|                       |           AA311209           |                               |
|                       |           BI828300           |                               |
|                       |           BI910445           |                               |
|                       |           BP262078           |                               |
|                       |           CN306388           |                               |
|                       |           CD514092           |                               |
|                       |           CD627175           |                               |
|                       |           CD627176           |                               |
|                       |           CD627177           |                               |
|                       |           CD627178           |                               |
|                       |           BE264523           |                               |
|                       |             R54355           |                               |
|                       |           BI912862           |                               |
|                       |           CN306390           |                               |
|                       |           CB998104           |                               |
|                       |           BI907688           |                               |
|                       |           AL534343           |                               |
|                       |           BM918357           |                               |
|                       |           BM544236           |                               |
|                       |           AA357633           |                               |
|                       |           BX379351           |                               |
|                       |           AU099475           |                               |
|                       |           BQ432493           |                               |
|                       |           BG772533           |                               |
|                       |           BP365727           |                               |
|                       |           AA471387           |                               |
|                       |           CD358006           |                               |
+-----------------------+------------------------------+-------------------------------+
|      #  110           | *Seq. mapped in strand Plus* | *Seq. mapped in strand Plus*  |
|                       |           BG436212           |          BY779694             |
|                       |                              |          BY099428             |
|                       |                              |          BB870447             |
|                       |                              |          BB868782             |
|                       |                              |          BB872448             |
|                       |                              |          BY151357             |
|                       |                              |          CB951793             |
|                       |                              |          BY104828             |
|                       |                              |          BY145490             |
|                       |                              |          BY121129             |
|                       |                              |          BY777240             |
|                       |                              |          BY324306             |
|                       |                              |          BY119025             |
|                       |                              |          BY118490             |
|                       |                              |          BY119355             |
|                       |                              |          BY117258             |
|                       |                              |          BY788798             |
|                       |                              |          BY136987             |
|                       |                              |          BY096564             |
|                       |                              |          BY087767             |
|                       |                              |          BY094127             |
|                       |                              |          BY032831             |
|                       |                              |          BY737363             |
|                       |                              |          BY344873             |
|                       |                              |          BY783719             |
|                       |                              |          BY084043             |
|                       |                              |          BY033269             |
|                       |                              |          AI529997             |
|                       |                              |          BY109539             |
|                       |                              |          BF149960             |
|                       |                              |          AI117748             |
|                       |                              |          BF151384             |
|                       |                              |          AV118319             |
|                       |                              |          BI082165             |
|                       |                              |          BY355552             |
|                       |                              |          BY355942             |
|                       |                              |            U44389             |
|                       |                              |          BC021157             |
|                       |                              |          BF579493             |
|                       |                              |          BY110668             |
|                       |                              |          BY109410             |
|                       |                              |          BY109250             |
|                       |                              |          AA389057             |
|                       |                              |          BY036372             |
|                       |                              |          BY000968             |
|                       |                              |          BM878087             |
|                       |                              |          BY702995             |
|                       |                              |          BI693227             |
|                       |                              |          BQ928613             |
|                       |                              |          BI697712             |
|                       |                              |          BI852540             |
|                       |                              |          CA977267             |
|                       |                              |            W34959             |
|                       |                              |          BI153684             |
|                       |                              |          AI153528             |
|                       |                              |          BQ563830             |
|                       |                              |          AA244818             |
|                       |                              |          BE652588             |
|                       |                              |          AA867091             |
|                       |                              |          CB950117             |
|                       |                              |          AA255058             |
|                       |                              |          BU698831             |
|                       |                              |          BU694987             |
|                       |                              |          BU696301             |
|                       |                              |          BU698466             |
|                       |                              |          BI902025             |
|                       |                              |          BI901953             |
|                       |                              |          AA920743             |
|                       |                              |          CK030632             |
|                       |                              |          AA103686             |
|                       |                              |          AI386331             |
|                       |                              |          AI876216             |
|                       |                              |          AI876097             |
|                       |                              |          AW988035             |
|                       | *Seq. mapped in strand Minus*| *Seq. mapped in strand Minus* |
|                       |           AI435471           |          BE949360             |
|                       |           BE856527           |                               |
|                       |           AI192457           |                               |
|                       |           BC018986           |                               |
|                       |           AI784647           |                               |
|                       |           AI867986           |                               |
|                       |           AW276303           |                               |
|                       |           BQ022045           |                               |
|                       |           AI292168           |                               |
|                       |           AW271789           |                               |
|                       |           AK058013           |                               |
|                       |           CB250661           |                               |
|                       |             L76465           |                               |
|                       |           BM843261           |                               |
|                       |             J05594           |                               |
|                       |           CR603356           |                               |
|                       |           CA312296           |                               |
|                       |           AA150966           |                               |
|                       |           CR603849           |                               |
|                       |           CR606077           |                               |
|                       |           CR602985           |                               |
|                       |           CR608377           |                               |
|                       |           BU754245           |                               |
|                       |             R31230           |                               |
|                       |           AW444767           |                               |
|                       |           CA306054           |                               |
|                       |           AI636394           |                               |
|                       |           CR598443           |                               |
|                       |           CR594801           |                               |
|                       |           CR618581           |                               |
|                       |           BG288929           |                               |
|                       |           BG739949           |                               |
|                       |           BG191206           |                               |
|                       |           BG570571           |                               |
|                       |           BG288945           |                               |
|                       |           BG483148           |                               |
|                       |           BX370134           |                               |
|                       |           BG193809           |                               |
|                       |           BP378916           |                               |
|                       |           BP380197           |                               |
|                       |           AI269053           |                               |
|                       |           BG286949           |                               |
|                       |           BE566425           |                               |
|                       |           AU155744           |                               |
|                       |           BP378517           |                               |
|                       |           BU929551           |                               |
|                       |           CB164778           |                               |
|                       |           CB163779           |                               |
|                       |           AL542647           |                               |
|                       |           AL541940           |                               |
|                       |           CR590250           |                               |
|                       |           CR604844           |                               |
|                       |           BM983714           |                               |
|                       |           BX397652           |                               |
|                       |           AL542740           |                               |
|                       |             R78155           |                               |
|                       |           AU139547           |                               |
|                       |           BX395563           |                               |
|                       |           BX336772           |                               |
|                       |           BX337426           |                               |
|                       |           BX337354           |                               |
|                       |           AU139437           |                               |
|                       |           BX378923           |                               |
|                       |           BX417164           |                               |
|                       |           BE866442           |                               |
|                       |           BX335060           |                               |
|                       |           BG536590           |                               |
|                       |             T56149           |                               |
|                       |           BX379313           |                               |
|                       |           BI760394           |                               |
|                       |           BP261182           |                               |
|                       |           BX335048           |                               |
|                       |             U63296           |                               |
|                       |             X82460           |                               |
|                       |           BE567871           |                               |
|                       |           BE737107           |                               |
|                       |           BP277223           |                               |
|                       |           AU136714           |                               |
|                       |           BF725997           |                               |
|                       |           BP432758           |                               |
|                       |           AU136004           |                               |
|                       |           AL553299           |                               |
|                       |           AU137292           |                               |
|                       |           BG548168           |                               |
|                       |           AU139778           |                               |
|                       |           AU138277           |                               |
|                       |           CB996742           |                               |
|                       |           CB998121           |                               |
|                       |           AU139132           |                               |
|                       |           AU136156           |                               |
|                       |           CB988194           |                               |
|                       |           BP261612           |                               |
|                       |           BP262137           |                               |
|                       |           BE380009           |                               |
|                       |           AU134972           |                               |
|                       |           BP223405           |                               |
|                       |           AU134733           |                               |
|                       |           AU134572           |                               |
|                       |           AU137555           |                               |
|                       |           CB961799           |                               |
|                       |           AL709858           |                               |
|                       |           AU135812           |                               |
|                       |           CB961045           |                               |
|                       |           AU135578           |                               |
|                       |           AU140008           |                               |
|                       |           CB960416           |                               |
|                       |           BE565909           |                               |
|                       |           AU135353           |                               |
|                       |           AU136933           |                               |
|                       |           CF994776           |                               |
|                       |           BE727073           |                               |
|                       |           BX382225           |                               |
|                       |           CF994777           |                               |
|                       |           AI373671           |                               |
|                       |           BQ186338           |                               |
|                       |           AI985526           |                               |
|                       |           BE729004           |                               |
|                       |           BC007691           |                               |
|                       |           BP194926           |                               |
|                       |             R76385           |                               |
|                       |             R35787           |                               |
|                       |             R31834           |                               |
|                       |           BF801619           |                               |
|                       |           BQ323828           |                               |
|                       |             R77255           |                               |
|                       |           BQ323848           |                               |
|                       |           BP320434           |                               |
|                       |           BQ323919           |                               |
|                       |           BF027420           |                               |
|                       |           BP320563           |                               |
|                       |           BP320925           |                               |
|                       |           BP260794           |                               |
|                       |           BP379368           |                               |
|                       |           BP376637           |                               |
|                       |           BP270404           |                               |
|                       |           BP274771           |                               |
|                       |           BP343086           |                               |
|                       |           BP320051           |                               |
|                       |           BP293928           |                               |
|                       |           BP320398           |                               |
|                       |           BP320425           |                               |
|                       |           BP321032           |                               |
|                       |           BP342394           |                               |
|                       |           BP261030           |                               |
|                       |           BP375674           |                               |
|                       |           BP320258           |                               |
|                       |           BP341064           |                               |
|                       |           BP377061           |                               |
|                       |           BP377206           |                               |
|                       |           BP220483           |                               |
|                       |           BP320227           |                               |
|                       |           BP320354           |                               |
|                       |           BP326834           |                               |
|                       |           BP379130           |                               |
|                       |           BP326876           |                               |
|                       |           BP378459           |                               |
|                       |           BP380348           |                               |
|                       |           BP274448           |                               |
|                       |           BP378079           |                               |
|                       |           BP378410           |                               |
|                       |           BP375961           |                               |
|                       |           BP379897           |                               |
|                       |           BM791047           |                               |
|                       |           BP375394           |                               |
|                       |           BF194909           |                               |
|                       |           BP261830           |                               |
|                       |           BQ323779           |                               |
|                       |           BP293781           |                               |
|                       |           BP227995           |                               |
|                       |           BP375510           |                               |
|                       |           BP376746           |                               |
|                       |           BX395647           |                               |
|                       |           CR619023           |                               |
|                       |           BX395646           |                               |
|                       |           AI738616           |                               |
|                       |           CB988538           |                               |
|                       |           AU139944           |                               |
|                       |           BP296739           |                               |
|                       |           BP378890           |                               |
|                       |           BP378975           |                               |
|                       |           BP275827           |                               |
|                       |           BP274651           |                               |
|                       |           AU137184           |                               |
|                       |           AU136051           |                               |
|                       |           BQ324619           |                               |
|                       |           CD109937           |                               |
|                       |           AI468604           |                               |
|                       |           BU752406           |                               |
|                       |           AW439262           |                               |
|                       |           CR605509           |                               |
|                       |           CR600709           |                               |
|                       |           BX396257           |                               |
|                       |           BX417542           |                               |
|                       |           BX336857           |                               |
|                       |           CB988871           |                               |
|                       |           CB994814           |                               |
|                       |           AI190482           |                               |
|                       |           BP379984           |                               |
|                       |           AW044661           |                               |
|                       |           BP261189           |                               |
|                       |           BP260785           |                               |
|                       |           AW444847           |                               |
|                       |           CB988773           |                               |
|                       |             R77256           |                               |
|                       |           BP260924           |                               |
|                       |           BP261538           |                               |
|                       |             R76724           |                               |
|                       |           BF736330           |                               |
|                       |           BX396460           |                               |
|                       |           BG008641           |                               |
|                       |           AV653116           |                               |
|                       |           AI769038           |                               |
|                       |             H02560           |                               |
|                       |           CB990810           |                               |
|                       |           CD688746           |                               |
|                       |           AI202552           |                               |
|                       |           AW242594           |                               |
|                       |           BP263938           |                               |
|                       |           BP263849           |                               |
|                       |             R35676           |                               |
|                       |           AU136817           |                               |
+-----------------------+------------------------------+-------------------------------+
|      #  111           | *Seq. mapped in strand Plus* | *Seq. mapped in strand Plus*  |
|                       |           AK055128           |          BY324928             |
|                       |           CR617325           |          BY322045             |
|                       |           BC066336           |          BY026520             |
|                       |           CR595585           |          BY346489             |
|                       |           CR606475           |          BY162536             |
|                       |           CR615752           |          BY162550             |
|                       |             U86782           |          BY161945             |
|                       |           BC009524           |          BY164814             |
|                       |           AK091095           |          BY743966             |
|                       |                              |          BY787277             |
|                       |                              |          CB055691             |
|                       |                              |          BY127746             |
|                       |                              |          CF900391             |
|                       |                              |          BY049339             |
|                       |                              |          BQ256519             |
|                       |                              |          BB859818             |
|                       |                              |          CF901938             |
|                       |                              |          AI122442             |
|                       |                              |          BF151374             |
|                       |                              |          BY058509             |
|                       |                              |          BY095520             |
|                       |                              |          BY746765             |
|                       |                              |          BY130768             |
|                       |                              |          BY171684             |
|                       |                              |          BB622239             |
|                       |                              |          AK078134             |
|                       |                              |          BB599258             |
|                       |                              |          BB841656             |
|                       |                              |          BY782261             |
|                       |                              |          BY165193             |
|                       |                              |          BY047357             |
|                       |                              |          BY058576             |
|                       |                              |          BY166711             |
|                       |                              |          BB859595             |
|                       |                              |          BU696329             |
|                       |                              |          AK011974             |
|                       |                              |          BY145441             |
|                       |                              |          BY124905             |
|                       |                              |          BB851727             |
|                       |                              |          CF893797             |
|                       |                              |          CF894872             |
|                       |                              |          CD772028             |
|                       |                              |          BU846893             |
|                       |                              |          CB952052             |
|                       |                              |          CB574232             |
|                       |                              |          BY222738             |
|                       |                              |          AK031863             |
|                       |                              |          BY111171             |
|                       |                              |          BY098151             |
|                       |                              |          BY038772             |
|                       |                              |          BY071114             |
|                       |                              |          BY027078             |
|                       |                              |          BY743143             |
|                       |                              |          AI116415             |
|                       |                              |          CF584372             |
|                       |                              |          BY770523             |
|                       |                              |          BY164076             |
|                       |                              |          BY173345             |
|                       |                              |          BY013670             |
|                       |                              |          BY771020             |
|                       |                              |          BY025715             |
|                       |                              |          BY034028             |
|                       |                              |          BY167516             |
|                       |                              |          BQ085292             |
|                       |                              |          BI794407             |
|                       |                              |          BF136607             |
|                       |                              |          BQ101687             |
|                       |                              |          BQ086715             |
|                       |                              |          BF179696             |
|                       |                              |          AK012013             |
|                       |                              |          CF902223             |
|                       |                              |          CN694732             |
|                       |                              |          BY125318             |
|                       |                              |          BY143165             |
|                       |                              |          BF150359             |
|                       |                              |          BY711210             |
|                       |                              |          BY162084             |
|                       |                              |          BY172548             |
|                       |                              |          CA887005             |
|                       |                              |          BY151678             |
|                       |                              |          BY147017             |
|                       |                              |          CN682261             |
|                       |                              |            Y13071             |
|                       |                              |          CF618226             |
|                       |                              |          BF116835             |
|                       |                              |          CK622519             |
|                       |                              |          BM894650             |
|                       |                              |          CN686720             |
|                       |                              |          BX521816             |
|                       |                              |          CN685596             |
|                       |                              |          CA553040             |
|                       |                              |          BF383898             |
|                       |                              |          BY051028             |
|                       |                              |          BY307558             |
|                       |                              |          BI655478             |
|                       |                              |          CA979409             |
|                       |                              |          CF155374             |
|                       |                              |          CF155850             |
|                       |                              |          CB237759             |
|                       |                              |          CB233459             |
|                       |                              |          BQ899400             |
|                       |                              |          BY019529             |
|                       |                              |          BY145764             |
|                       |                              |          BY786082             |
|                       |                              |          BY122478             |
|                       |                              |          CA949217             |
|                       |                              |          BY018080             |
|                       |                              |            C89194             |
|                       |                              |          AA967727             |
|                       |                              |          BI440460             |
|                       |                              |          AA139447             |
|                       |                              |          AI892880             |
|                       |                              |          BQ108986             |
|                       |                              |          BI656415             |
|                       |                              |          CF951564             |
|                       |                              |          BY333404             |
|                       |                              |          BE287222             |
|                       |                              |          BF123256             |
|                       |                              |          CN671541             |
|                       |                              |          CA545345             |
|                       |                              |          CA569391             |
|                       |                              |          BQ257939             |
|                       |                              |          BM946403             |
|                       |                              |          BF608637             |
|                       |                              |          BC003742             |
|                       |                              |          CK392022             |
|                       |                              |          AW210332             |
|                       |                              |          CN697510             |
|                       |                              |          CD355641             |
|                       |                              |          AI790014             |
|                       |                              |          CA573395             |
|                       |                              |          CF913007             |
|                       |                              |          CA322785             |
|                       |                              |          BE368842             |
|                       |                              |          BQ102039             |
|                       |                              |          BU151910             |
|                       |                              |          BQ895386             |
|                       |                              |          AA986216             |
|                       |                              |          CA567969             |
|                       |                              |          BB872673             |
|                       |                              |          BG976726             |
|                       |                              |          BG976653             |
|                       |                              |          CF153759             |
|                       |                              |          CF155525             |
|                       |                              |          BE380364             |
|                       |                              |          BQ102111             |
|                       |                              |          BY173240             |
|                       |                              |          CD561798             |
|                       |                              |          BF658225             |
|                       |                              |          AI316302             |
|                       |                              |          CA551336             |
|                       |                              |          CA549959             |
|                       |                              |          CA551790             |
|                       |                              |          CA551068             |
|                       |                              |          BE571137             |
|                       |                              |          CF901393             |
|                       |                              |          CF902019             |
|                       |                              |          BE137732             |
|                       |                              |          AW226877             |
|                       |                              |          CK335461             |
|                       |                              |          CB597453             |
|                       |                              |          BI248292             |
|                       |                              |          CK331897             |
|                       |                              |          AI046392             |
|                       |                              |          CN702568             |
|                       |                              |          CA322808             |
|                       |                              |          BI660221             |
|                       |                              |          CD363559             |
|                       |                              |          BF162803             |
|                       |                              |          CB202081             |
|                       |                              |          CA569376             |
|                       |                              |          CB319869             |
|                       |                              |          CN456503             |
|                       |                              |          CN457472             |
|                       |                              |          BG090247             |
|                       |                              |          BF159100             |
|                       |                              |          AA684366             |
|                       |                              |          AV327678             |
|                       |                              |          BU571458             |
|                       |                              |          AA271684             |
|                       |                              |          CA451468             |
|                       |                              |          BU559940             |
|                       |                              |          BE860283             |
|                       |                              |          BY497648             |
|                       |                              |          AA114758             |
|                       |                              |          AA692278             |
|                       |                              |          AI386374             |
|                       |                              |          AA117037             |
|                       |                              |          CN715014             |
|                       |                              |          BI158255             |
|                       |                              |          AI121099             |
|                       |                              |          CB318151             |
|                       |                              |          AV119358             |
|                       |                              |          AA473137             |
|                       |                              |          BI988135             |
|                       |                              |          AI327277             |
|                       |                              |          BI646847             |
|                       |                              |          BY609619             |
|                       |                              |          CN832412             |
|                       |                              |          BY634894             |
|                       |                              |          BY762763             |
|                       |                              |          BY657638             |
|                       |                              |          CD742286             |
|                       |                              |          AV333918             |
|                       |                              |          BM213810             |
|                       |                              |          BF226745             |
|                       |                              |          BF658827             |
|                       |                              |          BM247658             |
|                       |                              |          BF607585             |
|                       |                              |          BM235566             |
|                       |                              |            C77549             |
|                       |                              |          BY413636             |
|                       |                              |          CA563728             |
|                       |                              |          AA617529             |
|                       |                              |          BM876987             |
|                       |                              |          BQ101404             |
|                       |                              |          CK329709             |
|                       |                              |          BQ086442             |
|                       |                              |          BQ086507             |
|                       |                              |          CA949044             |
|                       |                              |          BM215485             |
|                       |                              |          BI437949             |
|                       |                              |          BM214303             |
|                       |                              |          BM221902             |
|                       |                              |          AW538992             |
|                       |                              |          BI440038             |
|                       |                              |          BM876715             |
|                       |                              |          BQ101841             |
|                       |                              |          BM232014             |
|                       |                              |          AW542067             |
|                       |                              |          BF318541             |
|                       |                              |          AW557503             |
|                       |                              |          BE955735             |
|                       |                              |          AI159636             |
|                       |                              |          BM249863             |
|                       |                              |          AI448878             |
|                       |                              |          CA887004             |
|                       |                              |          AW542043             |
|                       |                              |          AW210737             |
|                       |                              |          AV229030             |
|                       |                              |          AA866653             |
|                       |                              |          BB824940             |
|                       |                              |          BB785446             |
|                       |                              |          BQ044103             |
|                       |                              |          BM201967             |
|                       |                              |          BB834807             |
|                       |                              |          BB821718             |
|                       |                              |          AI845836             |
|                       |                              |          BB774177             |
|                       |                              |          BI660246             |
|                       |                              |          BM202038             |
|                       |                              |          BB732870             |
|                       |                              |          BB823176             |
|                       |                              |          BB732834             |
|                       |                              |          AA274539             |
|                       |                              |          BY515584             |
|                       |                              |          BQ086121             |
|                       |                              |          BB773603             |
|                       |                              |          BM212659             |
|                       |                              |          BB774464             |
|                       |                              |          BB781189             |
|                       |                              |          AI839033             |
|                       |                              |          BF459182             |
|                       |                              |          AI844639             |
|                       |                              |          AI846218             |
|                       |                              |          BU788625             |
|                       | *Seq. mapped in strand Minus*| *Seq. mapped in strand Minus* |
|                       |           BC059951           |          AW122112             |
|                       |           CR610996           |          AI849538             |
|                       |                              |          BE979428             |
|                       |                              |                               |
|                       |                              |                               |
|                       |                              |                               |
|                       |                              |                               |
|                       |                              |                               |
|                       |                              |                               |
|                       |                              |                               |
|                       |                              |                               |
|                       |                              |                               |
|                       |                              |                               |
|                       |                              |                               |
|                       |                              |                               |
|                       |                              |                               |
|                       |                              |                               |
|                       |                              |                               |
|                       |                              |                               |
|                       |                              |                               |
|                       |                              |                               |
|                       |                              |                               |
|                       |                              |                               |
|                       |                              |                               |
|                       |                              |                               |
|                       |                              |                               |
|                       |                              |                               |
|                       |                              |                               |
|                       |                              |                               |
|                       |                              |                               |
|                       |                              |                               |
|                       |                              |                               |
|                       |                              |                               |
|                       |                              |                               |
|                       |                              |                               |
|                       |                              |                               |
|                       |                              |                               |
|                       |                              |                               |
|                       |                              |                               |
|                       |                              |                               |
|                       |                              |                               |
|                       |                              |                               |
|                       |                              |                               |
|                       |                              |                               |
|                       |                              |                               |
|                       |                              |                               |
|                       |                              |                               |
|                       |                              |                               |
|                       |                              |                               |
|                       |                              |                               |
|                       |                              |                               |
|                       |                              |                               |
|                       |                              |                               |
|                       |                              |                               |
|                       |                              |                               |
|                       |                              |                               |
|                       |                              |                               |
|                       |                              |                               |
|                       |                              |                               |
|                       |                              |                               |
|                       |                              |                               |
|                       |                              |                               |
|                       |                              |                               |
|                       |                              |                               |
|                       |                              |                               |
|                       |                              |                               |
|                       |                              |                               |
|                       |                              |                               |
|                       |                              |                               |
|                       |                              |                               |
|                       |                              |                               |
|                       |                              |                               |
|                       |                              |                               |
|                       |                              |                               |
|                       |                              |                               |
|                       |                              |                               |
|                       |                              |                               |
|                       |                              |                               |
|                       |                              |                               |
|                       |                              |                               |
|                       |                              |                               |
|                       |                              |                               |
|                       |                              |                               |
|                       |                              |                               |
|                       |                              |                               |
|                       |                              |                               |
|                       |                              |                               |
|                       |                              |                               |
|                       |                              |                               |
|                       |                              |                               |
|                       |                              |                               |
|                       |                              |                               |
|                       |                              |                               |
|                       |                              |                               |
|                       |                              |                               |
|                       |                              |                               |
|                       |                              |                               |
|                       |                              |                               |
|                       |                              |                               |
|                       |                              |                               |
|                       |                              |                               |
|                       |                              |                               |
|                       |                              |                               |
|                       |                              |                               |
|                       |                              |                               |
|                       |                              |                               |
|                       |                              |                               |
|                       |                              |                               |
|                       |                              |                               |
|                       |                              |                               |
|                       |                              |                               |
|                       |                              |                               |
|                       |                              |                               |
|                       |                              |                               |
|                       |                              |                               |
|                       |                              |                               |
|                       |                              |                               |
|                       |                              |                               |
|                       |                              |                               |
|                       |                              |                               |
|                       |                              |                               |
|                       |                              |                               |
|                       |                              |                               |
|                       |                              |                               |
|                       |                              |                               |
|                       |                              |                               |
|                       |                              |                               |
|                       |                              |                               |
|                       |                              |                               |
|                       |                              |                               |
|                       |                              |                               |
|                       |                              |                               |
|                       |                              |                               |
|                       |                              |                               |
|                       |                              |                               |
|                       |                              |                               |
|                       |                              |                               |
|                       |                              |                               |
|                       |                              |                               |
|                       |                              |                               |
|                       |                              |                               |
|                       |                              |                               |
|                       |                              |                               |
|                       |                              |                               |
|                       |                              |                               |
|                       |                              |                               |
|                       |                              |                               |
|                       |                              |                               |
|                       |                              |                               |
|                       |                              |                               |
|                       |                              |                               |
|                       |                              |                               |
|                       |                              |                               |
|                       |                              |                               |
|                       |                              |                               |
|                       |                              |                               |
|                       |                              |                               |
|                       |                              |                               |
|                       |                              |                               |
|                       |                              |                               |
|                       |                              |                               |
|                       |                              |                               |
|                       |                              |                               |
|                       |                              |                               |
|                       |                              |                               |
|                       |                              |                               |
|                       |                              |                               |
|                       |                              |                               |
|                       |                              |                               |
|                       |                              |                               |
|                       |                              |                               |
|                       |                              |                               |
|                       |                              |                               |
|                       |                              |                               |
|                       |                              |                               |
|                       |                              |                               |
|                       |                              |                               |
|                       |                              |                               |
|                       |                              |                               |
|                       |                              |                               |
|                       |                              |                               |
|                       |                              |                               |
|                       |                              |                               |
|                       |                              |                               |
|                       |                              |                               |
|                       |                              |                               |
|                       |                              |                               |
|                       |                              |                               |
|                       |                              |                               |
|                       |                              |                               |
|                       |                              |                               |
|                       |                              |                               |
|                       |                              |                               |
|                       |                              |                               |
|                       |                              |                               |
|                       |                              |                               |
|                       |                              |                               |
|                       |                              |                               |
|                       |                              |                               |
|                       |                              |                               |
|                       |                              |                               |
|                       |                              |                               |
|                       |                              |                               |
|                       |                              |                               |
|                       |                              |                               |
|                       |                              |                               |
|                       |                              |                               |
|                       |                              |                               |
|                       |                              |                               |
|                       |                              |                               |
|                       |                              |                               |
|                       |                              |                               |
|                       |                              |                               |
|                       |                              |                               |
|                       |                              |                               |
|                       |                              |                               |
|                       |                              |                               |
|                       |                              |                               |
|                       |                              |                               |
|                       |                              |                               |
|                       |                              |                               |
|                       |                              |                               |
|                       |                              |                               |
|                       |                              |                               |
|                       |                              |                               |
|                       |                              |                               |
|                       |                              |                               |
|                       |                              |                               |
|                       |                              |                               |
|                       |                              |                               |
|                       |                              |                               |
|                       |                              |                               |
|                       |                              |                               |
|                       |                              |                               |
|                       |                              |                               |
|                       |                              |                               |
|                       |                              |                               |
|                       |                              |                               |
|                       |                              |                               |
|                       |                              |                               |
|                       |                              |                               |
|                       |                              |                               |
|                       |                              |                               |
|                       |                              |                               |
|                       |                              |                               |
|                       |                              |                               |
|                       |                              |                               |
|                       |                              |                               |
|                       |                              |                               |
|                       |                              |                               |
|                       |                              |                               |
|                       |                              |                               |
|                       |                              |                               |
|                       |                              |                               |
|                       |                              |                               |
|                       |                              |                               |
|                       |                              |                               |
|                       |                              |                               |
|                       |                              |                               |
|                       |                              |                               |
|                       |                              |                               |
|                       |                              |                               |
|                       |                              |                               |
|                       |                              |                               |
|                       |                              |                               |
|                       |                              |                               |
+-----------------------+------------------------------+-------------------------------+
|      #  112           | *Seq. mapped in strand Plus* | *Seq. mapped in strand Plus*  |
|                       |           BI094807           |          BQ960306             |
|                       | *Seq. mapped in strand Minus*| *Seq. mapped in strand Minus* |
|                       |           AA889055           |          BG080336             |
|                       |           AW293447           |          AK081081             |
|                       |           CR737952           |          BB350934             |
|                       |           AK127129           |          BB647276             |
|                       |           AA017079           |                               |
|                       |           BG007439           |                               |
+-----------------------+------------------------------+-------------------------------+
|      #  113           | *Seq. mapped in strand Plus* | *Seq. mapped in strand Plus*  |
|                       |           AK057292           |          AW106672             |
|                       |           BE242628           |          BY260462             |
|                       |           BG718324           |          BY302253             |
|                       |           AU120927           |          BY736762             |
|                       |           BC028360           |          BY208362             |
|                       |           AL600038           |          BY034113             |
|                       |           AU131156           |          AK004748             |
|                       |           CN341914           |          BY169148             |
|                       |           AL597394           |          BY164131             |
|                       |           BG897828           |          BY170128             |
|                       |           BE244460           |          BY056963             |
|                       |           AA428612           |          BY704947             |
|                       |           AI393458           |          BY718069             |
|                       |           CN341917           |          BG974010             |
|                       |           BX366830           |          BI661675             |
|                       |           AL553469           |          BI558285             |
|                       |           AA333255           |          BY194339             |
|                       |           BX445621           |          BE572633             |
|                       |           BM908032           |          BI104582             |
|                       |           BX458455           |          BY131716             |
|                       |           CN341906           |          BY322122             |
|                       |           CD708866           |          BY271223             |
|                       |           BM451832           |          AW488837             |
|                       |           BC039729           |          BI645599             |
|                       |           CN341909           |          BY195347             |
|                       |           CN341913           |          BF452420             |
|                       |           AL530306           |          AA068141             |
|                       |           CR610942           |          BY330207             |
|                       |           AI150371           |          BY335740             |
|                       |           BI598478           |          BY073744             |
|                       |           BG899657           |          BY005960             |
|                       |           CF137087           |          CF904848             |
|                       |           AW503817           |          BX525172             |
|                       |           BX343079           |          AA389597             |
|                       |           BU187701           |          AW230802             |
|                       |           BU431664           |          CK137519             |
|                       |           CN341910           |          BF100945             |
|                       |           CN341908           |          BC033271             |
|                       |           CN341916           |          BF119923             |
|                       |           BQ890222           |          BF142467             |
|                       |           CF136753           |          BB610206             |
|                       |           AF013591           |          CK020064             |
|                       |           AA768203           |          CF894010             |
|                       |           AA382317           |          BF021692             |
|                       |           AW956648           |          BF021693             |
|                       |             H59207           |          CB238139             |
|                       |           BX106080           |          BF168617             |
|                       |           BU186422           |          BG100485             |
|                       |           BE711223           |          CF728994             |
|                       |           BQ641320           |          BF134913             |
|                       |           AA113047           |          CA566458             |
|                       |           CN341911           |          CA321196             |
|                       |           AW382155           |          BE308968             |
|                       |           AA452706           |          BI154515             |
|                       |           BE544171           |          AW985640             |
|                       |           BF094220           |          CA980650             |
|                       |           BG026767           |          CN530858             |
|                       |           BG254303           |          BM935786             |
|                       |           CA312082           |          BC002255             |
|                       |           BX472653           |          BE305657             |
|                       |           BM806861           |          BG967282             |
|                       |             W56214           |          BM944762             |
|                       |           BX370698           |          BI156659             |
|                       |           BQ311197           |          AA981298             |
|                       |           BQ072738           |          AF013127             |
|                       |           BG574572           |          BE862394             |
|                       |           BE621277           |          BG277022             |
|                       |           BF984852           |          BU058941             |
|                       |           AU127529           |          BI105774             |
|                       |           BU429098           |          CA318553             |
|                       |           BX366829           |          BX519519             |
|                       |           AL600885           |          BE198424             |
|                       |           BG290313           |          BY710468             |
|                       |           AU137473           |          AK011110             |
|                       |             T54240           |          AA896325             |
|                       |           BI826444           |          AA061258             |
|                       |           AW006290           |          BF787288             |
|                       |           AW243341           |          CB193097             |
|                       |           BG150316           |          CD805627             |
|                       |           AA346970           |          CN539884             |
|                       |           BI261309           |          BI732984             |
|                       |           AI650817           |          AA982953             |
|                       |           AU141416           |          AI385938             |
|                       |           BG547664           |          AA137576             |
|                       |           BX479920           |          AW105746             |
|                       |           CB853831           |          CN539844             |
|                       |           AV717319           |          BM944130             |
|                       |           AI918917           |            L26671             |
|                       |           AI743057           |          AV032933             |
|                       |             W47066           |          AA795994             |
|                       |           CB135939           |          AA030787             |
|                       |           AU119284           |          BX515899             |
|                       |           AU128169           |          AW541719             |
|                       |           BX496793           |          BM240798             |
|                       |           AA432342           |          BQ562706             |
|                       |           BG223578           |          AA208502             |
|                       |           AU142572           |          AW545100             |
|                       |           BU673968           |          CF173922             |
|                       |           CD251540           |          BQ265740             |
|                       |           AA936902           |          BY412480             |
|                       |           BX494464           |          BF533377             |
|                       |           CD102601           |          BF451812             |
|                       |           BX640929           |          BQ714638             |
|                       |           BG504052           |          AA623460             |
|                       |           AA397584           |          CN710646             |
|                       |           AA749128           |          CN712143             |
|                       |           BG499831           |          BB690188             |
|                       |           BF383033           |          BP427073             |
|                       |           AA720601           |          BB726355             |
|                       |           BE709902           |          AI429540             |
|                       |           CB242096           |          BY483164             |
|                       |             W46966           |          BB685807             |
|                       |           AA732512           |          BY575621             |
|                       |                              |          BB710181             |
|                       |                              |          CF622741             |
|                       |                              |          BB740666             |
|                       |                              |          BY438620             |
|                       |                              |          AL361629             |
|                       |                              |          AA467232             |
|                       |                              |          AI156961             |
|                       |                              |          AA967967             |
|                       | *Seq. mapped in strand Minus*| *Seq. mapped in strand Minus* |
|                       |           CB250569           |          CO040044             |
|                       |                              |          AW048134             |
|                       |                              |          BB629579             |
|                       |                              |                               |
|                       |                              |                               |
|                       |                              |                               |
|                       |                              |                               |
|                       |                              |                               |
|                       |                              |                               |
|                       |                              |                               |
|                       |                              |                               |
|                       |                              |                               |
|                       |                              |                               |
|                       |                              |                               |
|                       |                              |                               |
|                       |                              |                               |
|                       |                              |                               |
|                       |                              |                               |
|                       |                              |                               |
|                       |                              |                               |
|                       |                              |                               |
|                       |                              |                               |
|                       |                              |                               |
|                       |                              |                               |
|                       |                              |                               |
|                       |                              |                               |
|                       |                              |                               |
|                       |                              |                               |
|                       |                              |                               |
|                       |                              |                               |
|                       |                              |                               |
|                       |                              |                               |
|                       |                              |                               |
|                       |                              |                               |
|                       |                              |                               |
|                       |                              |                               |
|                       |                              |                               |
|                       |                              |                               |
|                       |                              |                               |
|                       |                              |                               |
|                       |                              |                               |
|                       |                              |                               |
|                       |                              |                               |
|                       |                              |                               |
|                       |                              |                               |
|                       |                              |                               |
|                       |                              |                               |
|                       |                              |                               |
|                       |                              |                               |
|                       |                              |                               |
|                       |                              |                               |
|                       |                              |                               |
|                       |                              |                               |
|                       |                              |                               |
|                       |                              |                               |
|                       |                              |                               |
|                       |                              |                               |
|                       |                              |                               |
|                       |                              |                               |
|                       |                              |                               |
|                       |                              |                               |
|                       |                              |                               |
|                       |                              |                               |
|                       |                              |                               |
|                       |                              |                               |
|                       |                              |                               |
|                       |                              |                               |
|                       |                              |                               |
|                       |                              |                               |
|                       |                              |                               |
|                       |                              |                               |
|                       |                              |                               |
|                       |                              |                               |
|                       |                              |                               |
|                       |                              |                               |
|                       |                              |                               |
|                       |                              |                               |
|                       |                              |                               |
|                       |                              |                               |
|                       |                              |                               |
|                       |                              |                               |
|                       |                              |                               |
|                       |                              |                               |
|                       |                              |                               |
|                       |                              |                               |
|                       |                              |                               |
|                       |                              |                               |
|                       |                              |                               |
|                       |                              |                               |
|                       |                              |                               |
|                       |                              |                               |
|                       |                              |                               |
|                       |                              |                               |
|                       |                              |                               |
|                       |                              |                               |
|                       |                              |                               |
|                       |                              |                               |
|                       |                              |                               |
|                       |                              |                               |
|                       |                              |                               |
|                       |                              |                               |
|                       |                              |                               |
|                       |                              |                               |
|                       |                              |                               |
|                       |                              |                               |
|                       |                              |                               |
|                       |                              |                               |
|                       |                              |                               |
|                       |                              |                               |
|                       |                              |                               |
|                       |                              |                               |
|                       |                              |                               |
|                       |                              |                               |
|                       |                              |                               |
|                       |                              |                               |
|                       |                              |                               |
|                       |                              |                               |
|                       |                              |                               |
|                       |                              |                               |
|                       |                              |                               |
|                       |                              |                               |
+-----------------------+------------------------------+-------------------------------+
|      #  114           | *Seq. mapped in strand Plus* | *Seq. mapped in strand Plus*  |
|                       |           BU679828           |          AW489286             |
|                       |           CB321850           |                               |
|                       | *Seq. mapped in strand Minus*| *Seq. mapped in strand Minus* |
|                       |           CA945213           |          BX524069             |
|                       |           AI493922           |          BQ126956             |
|                       |           AI762862           |          BC003871             |
|                       |           BQ020351           |          AK011419             |
|                       |           CN478575           |          BE457013             |
|                       |           AA935054           |          AK078870             |
|                       |           AI675905           |          AA560576             |
|                       |           AA885762           |                               |
|                       |           AA765542           |                               |
|                       |           AA992608           |                               |
|                       |           CA443518           |                               |
|                       |           AA858281           |                               |
|                       |           AI753792           |                               |
|                       |           BU689170           |                               |
|                       |           CA447719           |                               |
|                       |           CA421530           |                               |
|                       |           BU608451           |                               |
|                       |           AW044230           |                               |
|                       |             W37784           |                               |
|                       |           AA668860           |                               |
|                       |           AA811274           |                               |
|                       |           CR592913           |                               |
|                       |           CA423401           |                               |
|                       |           BU146192           |                               |
|                       |           BU738618           |                               |
|                       |           BG178994           |                               |
|                       |           BX348899           |                               |
|                       |           AW006619           |                               |
|                       |           AI128139           |                               |
|                       |           AI683305           |                               |
|                       |           BQ029973           |                               |
|                       |           BQ023701           |                               |
|                       |           AA481252           |                               |
|                       |           BU619680           |                               |
|                       |           AI473341           |                               |
|                       |           AA812118           |                               |
|                       |           BC013106           |                               |
|                       |           BX348900           |                               |
|                       |           BE885133           |                               |
|                       |           BQ951616           |                               |
|                       |           BM717141           |                               |
|                       |           CB241779           |                               |
|                       |           CF891535           |                               |
|                       |           AA713672           |                               |
|                       |           AA713673           |                               |
|                       |           BU624975           |                               |
|                       |           AW949870           |                               |
|                       |           AU135514           |                               |
|                       |           AV721723           |                               |
|                       |             N62810           |                               |
|                       |           BG168858           |                               |
|                       |           AW363088           |                               |
|                       |           BG193741           |                               |
|                       |           AA434587           |                               |
|                       |           BG028932           |                               |
|                       |           BG110348           |                               |
|                       |             T27659           |                               |
|                       |           BM701373           |                               |
|                       |             R21415           |                               |
|                       |           AW582106           |                               |
|                       |           BE710207           |                               |
|                       |           BM987856           |                               |
|                       |           BM817829           |                               |
|                       |           BX324335           |                               |
|                       |             H44468           |                               |
|                       |           BG110274           |                               |
|                       |           AA459547           |                               |
|                       |           BG428983           |                               |
|                       |           BI761398           |                               |
|                       |           CD642522           |                               |
|                       |             R79880           |                               |
|                       |           AA234396           |                               |
|                       |           AA293148           |                               |
|                       |           AA253280           |                               |
|                       |           AF493924           |                               |
|                       |             M31468           |                               |
|                       |           AI207768           |                               |
|                       |             W01322           |                               |
|                       |           CV023925           |                               |
|                       |           BX503352           |                               |
|                       |           BX345953           |                               |
|                       |           CN422538           |                               |
|                       |           BE925586           |                               |
|                       |           BF365674           |                               |
|                       |           BG028482           |                               |
|                       |           AL575330           |                               |
|                       |           BU507954           |                               |
|                       |           CN422539           |                               |
|                       |           BI861438           |                               |
|                       |           BG468294           |                               |
|                       |           BE873011           |                               |
|                       |           CF242914           |                               |
|                       |           BU179081           |                               |
|                       |           CF994267           |                               |
|                       |           BE887570           |                               |
|                       |           AA248735           |                               |
|                       |           CB961515           |                               |
|                       |           BG721590           |                               |
|                       |           BP276073           |                               |
|                       |           BP206021           |                               |
|                       |           BP206589           |                               |
|                       |           BP276244           |                               |
|                       |           AA305138           |                               |
|                       |           CD109544           |                               |
+-----------------------+------------------------------+-------------------------------+
|      #  115           | *Seq. mapped in strand Plus* | *Seq. mapped in strand Plus*  |
|                       |           BG771854           |            X80420             |
|                       |           BI461680           |                               |
|                       |           BI547951           |                               |
|                       |           BG723453           |                               |
|                       |           BP197503           |                               |
|                       |           BQ430013           |                               |
|                       |           BQ897793           |                               |
|                       |           BP210512           |                               |
|                       |           BI560090           |                               |
|                       |           BI549852           |                               |
|                       |           BP341423           |                               |
|                       |           BM838879           |                               |
|                       |           AK098485           |                               |
|                       |           CB127836           |                               |
|                       |           BU542973           |                               |
|                       |           BP210706           |                               |
|                       |           BQ432033           |                               |
|                       |           AF218011           |                               |
|                       |           BG686769           |                               |
|                       |           CA943211           |                               |
|                       |           AL540350           |                               |
|                       |           CR616387           |                               |
|                       |           AW583589           |                               |
|                       |           BG896279           |                               |
|                       |           BI600294           |                               |
|                       |           BU179044           |                               |
|                       |           CB266060           |                               |
|                       |           AU098536           |                               |
|                       |           CN418078           |                               |
|                       |           AF100615           |                               |
|                       |           AY148481           |                               |
|                       |           CN335645           |                               |
|                       |           BM509671           |                               |
|                       |           BG823234           |                               |
|                       |           BG494302           |                               |
|                       |           BC002936           |                               |
|                       |           BC022845           |                               |
|                       |           BP341579           |                               |
|                       |           BE788373           |                               |
|                       |           BP378845           |                               |
|                       |           CN335649           |                               |
|                       |           BM752593           |                               |
|                       |           BM742684           |                               |
|                       |           BM742435           |                               |
|                       |           BM785730           |                               |
|                       |           CN335618           |                               |
|                       |           BI858689           |                               |
|                       |           BG720635           |                               |
|                       |           BI223631           |                               |
|                       |           BG477438           |                               |
|                       |           BU190524           |                               |
|                       |           BM781892           |                               |
|                       |           AA307885           |                               |
|                       |           BE383902           |                               |
|                       |           CN335643           |                               |
|                       |           BE296980           |                               |
|                       |           BE795703           |                               |
|                       |           BE563762           |                               |
|                       |           BI259842           |                               |
|                       |           BM049356           |                               |
|                       |           BU542880           |                               |
|                       |           BU502682           |                               |
|                       |           BU542457           |                               |
|                       |           BX378591           |                               |
|                       |           BX459260           |                               |
|                       |           BM172412           |                               |
|                       |           BI601432           |                               |
|                       |           BF217957           |                               |
|                       |           BU189454           |                               |
|                       |           BM763267           |                               |
|                       |           CD244855           |                               |
|                       |           BI602080           |                               |
|                       |           BI159936           |                               |
|                       |           BQ439878           |                               |
|                       |           BI086947           |                               |
|                       |           BG703124           |                               |
|                       |           BI546293           |                               |
|                       |           BM852891           |                               |
|                       |           BM555020           |                               |
|                       |           BX337248           |                               |
|                       |           CR600778           |                               |
|                       |           CN335667           |                               |
|                       |           CN335639           |                               |
|                       |           BI088874           |                               |
|                       |           BG333207           |                               |
|                       |           BE891429           |                               |
|                       |           BM555855           |                               |
|                       |           AL530068           |                               |
|                       |           BP316434           |                               |
|                       |           CN481890           |                               |
|                       |           BG572962           |                               |
|                       |           CN335634           |                               |
|                       |           BG925631           |                               |
|                       |           BG109662           |                               |
|                       |           BF183280           |                               |
|                       |           BM016334           |                               |
|                       |           BQ881712           |                               |
|                       |           BQ707527           |                               |
|                       |           BQ225797           |                               |
|                       |           CD243861           |                               |
|                       |           BQ228560           |                               |
|                       |           BG714391           |                               |
|                       |           CB148415           |                               |
|                       |           BP193433           |                               |
|                       |           BF967536           |                               |
|                       |           BG702060           |                               |
|                       |           BU187949           |                               |
|                       |           BQ230147           |                               |
|                       |           AW673584           |                               |
|                       |           BE895474           |                               |
|                       |           CN335612           |                               |
|                       |           BE077956           |                               |
|                       |           BI553196           |                               |
|                       |           BM460777           |                               |
|                       |           BG258452           |                               |
|                       |           BI598681           |                               |
|                       |           BQ636122           |                               |
|                       |           BI598192           |                               |
|                       |           BI596779           |                               |
|                       |           CD558607           |                               |
|                       |           CD693108           |                               |
|                       |           CD687936           |                               |
|                       |           CD702814           |                               |
|                       |           BQ721312           |                               |
|                       |           BX378329           |                               |
|                       |           CR613628           |                               |
|                       |           CF597416           |                               |
|                       |           BG715238           |                               |
|                       |           BE398061           |                               |
|                       |           BE298372           |                               |
|                       |           BU077393           |                               |
|                       |           AL537569           |                               |
|                       |           CR613862           |                               |
|                       |           CN335674           |                               |
|                       |           BX457878           |                               |
|                       |           CR605109           |                               |
|                       |           CR605106           |                               |
|                       |           BE539153           |                               |
|                       |           BU945255           |                               |
|                       |           CD514031           |                               |
|                       |           BU165128           |                               |
|                       |           CD359955           |                               |
|                       |           BG501248           |                               |
|                       |           BG531825           |                               |
|                       |           BG425745           |                               |
|                       |           BG429213           |                               |
|                       |           BG427547           |                               |
|                       |           BP314654           |                               |
|                       |           BP356962           |                               |
|                       |           BP207695           |                               |
|                       |           CB143000           |                               |
|                       |           BM789552           |                               |
|                       |           BM854560           |                               |
|                       |           BI551660           |                               |
|                       |           AV738509           |                               |
|                       |           BU598931           |                               |
|                       |           BE549114           |                               |
|                       |           AF070664           |                               |
|                       |           BE882366           |                               |
|                       |           BQ935454           |                               |
|                       |           CD703310           |                               |
|                       |             T31705           |                               |
|                       |           BU963858           |                               |
|                       |           BE786107           |                               |
|                       |           CD683950           |                               |
|                       |           CN335619           |                               |
|                       |           BG705156           |                               |
|                       |           BG707305           |                               |
|                       |           BU183052           |                               |
|                       |           AL538484           |                               |
|                       |           AL538499           |                               |
|                       |           CD511485           |                               |
|                       |           BM051280           |                               |
|                       |           CD107964           |                               |
|                       |           BU149441           |                               |
|                       |           AF167173           |                               |
|                       |           CB839888           |                               |
|                       |           AA232150           |                               |
|                       |           BU940432           |                               |
|                       |           BG715292           |                               |
|                       |           BG941315           |                               |
|                       |           CN335656           |                               |
|                       |           BX641828           |                               |
|                       |           CN335668           |                               |
|                       |           BG675349           |                               |
|                       |           BM559188           |                               |
|                       |           CD708850           |                               |
|                       |           BI439060           |                               |
|                       |           BI598293           |                               |
|                       |           BG539029           |                               |
|                       |           CN335609           |                               |
|                       |           BG706966           |                               |
|                       |           BG708665           |                               |
|                       |           CN335681           |                               |
|                       |           BG483792           |                               |
|                       |           BG708396           |                               |
|                       |           BG715134           |                               |
|                       |           CD521612           |                               |
|                       |           BG506298           |                               |
|                       |           BI668036           |                               |
|                       |           BI667441           |                               |
|                       |           BG615702           |                               |
|                       |           BQ423707           |                               |
|                       |           CN335608           |                               |
|                       |           BG683017           |                               |
|                       |           CN335636           |                               |
|                       |           BF218557           |                               |
|                       |           BF692181           |                               |
|                       |           BG399997           |                               |
|                       |           BG548673           |                               |
|                       |           CD686747           |                               |
|                       |           CD684345           |                               |
|                       |           BG707407           |                               |
|                       |           BI544650           |                               |
|                       |           BX420399           |                               |
|                       |           CR608590           |                               |
|                       |           AV729112           |                               |
|                       |           CD298912           |                               |
|                       |           BF210273           |                               |
|                       |           BG168852           |                               |
|                       |           CN335625           |                               |
|                       |           BG249779           |                               |
|                       |           CD558685           |                               |
|                       |           BQ215413           |                               |
|                       |           AL539598           |                               |
|                       |           CR599199           |                               |
|                       |           AL137697           |                               |
|                       |           AA101563           |                               |
|                       |           AV759334           |                               |
|                       |           CN335635           |                               |
|                       |           BF573761           |                               |
|                       |           BU167966           |                               |
|                       |           BG533122           |                               |
|                       |           CD518907           |                               |
|                       |           AL534204           |                               |
|                       |           AL560001           |                               |
|                       |           CR621917           |                               |
|                       |           CR590569           |                               |
|                       |           BG943637           |                               |
|                       |           AV740112           |                               |
|                       |           CB215353           |                               |
|                       |           BG769577           |                               |
|                       |           BG760431           |                               |
|                       |           BQ421243           |                               |
|                       |           BG761796           |                               |
|                       |           BQ217282           |                               |
|                       |           BQ066347           |                               |
|                       |           BU159225           |                               |
|                       |           BM479592           |                               |
|                       |           AV743546           |                               |
|                       |           BI545453           |                               |
|                       |           CB993162           |                               |
|                       |           CB996749           |                               |
|                       |           BG394946           |                               |
|                       |           AL047191           |                               |
|                       |           BQ681437           |                               |
|                       |           CN335669           |                               |
|                       |           BG259562           |                               |
|                       |           BM460566           |                               |
|                       |           BQ218621           |                               |
|                       |           BE885718           |                               |
|                       |           BM464465           |                               |
|                       |           BF105104           |                               |
|                       |           BE294604           |                               |
|                       |           BF307811           |                               |
|                       |           BU542982           |                               |
|                       |           CD690271           |                               |
|                       |           BM795897           |                               |
|                       |           CD689965           |                               |
|                       |           CN335629           |                               |
|                       |           BI223309           |                               |
|                       |           BG575797           |                               |
|                       |           BU160337           |                               |
|                       |           BQ929779           |                               |
|                       |           AL518718           |                               |
|                       |           CR614825           |                               |
|                       |           CN335611           |                               |
|                       |           CN335604           |                               |
|                       |           BQ898495           |                               |
|                       |           CN335682           |                               |
|                       |           BM923800           |                               |
|                       |           AF161546           |                               |
|                       |           CB149986           |                               |
|                       |           BG944594           |                               |
|                       |           AI929671           |                               |
|                       |           BM009065           |                               |
|                       |           CN335633           |                               |
|                       |           BG534150           |                               |
|                       |           BQ425133           |                               |
|                       |           BX441118           |                               |
|                       |           AV691402           |                               |
|                       |           CN335617           |                               |
|                       |           AJ707127           |                               |
|                       |           BG720477           |                               |
|                       |           BI462638           |                               |
|                       |           CD697210           |                               |
|                       |           BX488792           |                               |
|                       |           CA397153           |                               |
|                       |           CN335627           |                               |
|                       |           BF685571           |                               |
|                       |           BG114533           |                               |
|                       |           CN335653           |                               |
|                       |           BM453349           |                               |
|                       |             T19226           |                               |
|                       |           BF208335           |                               |
|                       |             T72366           |                               |
|                       |           AA081719           |                               |
|                       |           BI257451           |                               |
|                       |           BI086781           |                               |
|                       |           BG616023           |                               |
|                       |           BQ420310           |                               |
|                       |           BQ722924           |                               |
|                       |           BI090043           |                               |
|                       |           BQ048911           |                               |
|                       |           CD171617           |                               |
|                       |           CD558055           |                               |
|                       |           BX419158           |                               |
|                       |           CN335648           |                               |
|                       |           CD521946           |                               |
|                       |           BG118447           |                               |
|                       |           CN335640           |                               |
|                       |           BQ878397           |                               |
|                       |           BU166623           |                               |
|                       |           BE397964           |                               |
|                       |           CB148686           |                               |
|                       |           BG942359           |                               |
|                       |           BE734372           |                               |
|                       |           BG431993           |                               |
|                       |           BX418197           |                               |
|                       |           BX418198           |                               |
|                       |           CR599453           |                               |
|                       |           CN335665           |                               |
|                       |           BE260790           |                               |
|                       |           BC067826           |                               |
|                       |           AV763662           |                               |
|                       |           BP308187           |                               |
|                       |           AV714252           |                               |
|                       |           CR617357           |                               |
|                       |           AA333031           |                               |
|                       |           BG178676           |                               |
|                       |           AW965327           |                               |
|                       |           BG763299           |                               |
|                       |           BM804107           |                               |
|                       |           BQ436879           |                               |
|                       |           AA207181           |                               |
|                       |           CN335650           |                               |
|                       |           BE393753           |                               |
|                       |           CB118015           |                               |
|                       |           BI548559           |                               |
|                       |           CB123557           |                               |
|                       |           BQ420323           |                               |
|                       |           CN335678           |                               |
|                       |           CN335661           |                               |
|                       |           AA128658           |                               |
|                       |           AL564525           |                               |
|                       |           BE734651           |                               |
|                       |           BE734738           |                               |
|                       |           AA070785           |                               |
|                       |           CN335658           |                               |
|                       |           CN335670           |                               |
|                       |           BF346672           |                               |
|                       |           BE513480           |                               |
|                       |           BM799905           |                               |
|                       |             T32080           |                               |
|                       |           CK818361           |                               |
|                       |           CV026478           |                               |
|                       |           CN335630           |                               |
|                       |           BE894419           |                               |
|                       |           CN335605           |                               |
|                       |           BF032726           |                               |
|                       |           BE543589           |                               |
|                       |           CN335662           |                               |
|                       |           CN335679           |                               |
|                       |           CN335666           |                               |
|                       |           BQ050738           |                               |
|                       |           AL534534           |                               |
|                       |           CR601080           |                               |
|                       |           BX442099           |                               |
|                       |           BX444904           |                               |
|                       |           BQ217718           |                               |
|                       |           BX460613           |                               |
|                       |           CR622863           |                               |
|                       |           AA248491           |                               |
|                       |           AA312401           |                               |
|                       |           BG168638           |                               |
|                       |           BE907020           |                               |
|                       |           CF147408           |                               |
|                       |           CF455322           |                               |
|                       |           BX336155           |                               |
|                       |           BX364920           |                               |
|                       |           BG943311           |                               |
|                       |           BF127816           |                               |
|                       |           CN335671           |                               |
|                       |           BX420365           |                               |
|                       |           BG675142           |                               |
|                       |           BM974278           |                               |
|                       |           AA214280           |                               |
|                       |           BQ130442           |                               |
|                       |           BM508075           |                               |
|                       |           BG437705           |                               |
|                       |           CK431121           |                               |
|                       |           BE888601           |                               |
|                       |           CD579946           |                               |
|                       |           CK431103           |                               |
|                       |           CN335654           |                               |
|                       |           AW605905           |                               |
|                       |           CN335628           |                               |
|                       |           BU787865           |                               |
|                       |           BG287759           |                               |
|                       |           BQ549495           |                               |
|                       |           BG760155           |                               |
|                       |           BI861433           |                               |
|                       |           CF136802           |                               |
|                       |           AW408255           |                               |
|                       |           BE255297           |                               |
|                       |           BI092575           |                               |
|                       |           CR613253           |                               |
|                       |           CR606633           |                               |
|                       |           CA407607           |                               |
|                       |           BU186874           |                               |
|                       |           BU508870           |                               |
|                       |           CN335646           |                               |
|                       |           CN335631           |                               |
|                       |           BQ643814           |                               |
|                       |           BM478156           |                               |
|                       |             T31642           |                               |
|                       |           CN421234           |                               |
|                       |           BG829855           |                               |
|                       |           BG331082           |                               |
|                       |             T36138           |                               |
|                       |             T36139           |                               |
|                       |           CN335675           |                               |
|                       |           BM550693           |                               |
|                       |           AL534658           |                               |
|                       |           CB146700           |                               |
|                       |           BP194597           |                               |
|                       |           CN335657           |                               |
|                       |           BE568638           |                               |
|                       |           CN335642           |                               |
|                       |           BE256302           |                               |
|                       |           BE253128           |                               |
|                       |           BE888817           |                               |
|                       |           BP218312           |                               |
|                       |           BX382045           |                               |
|                       |           BE394299           |                               |
|                       |           BE378815           |                               |
|                       |           BF791160           |                               |
|                       |           BG718314           |                               |
|                       |           BM565616           |                               |
|                       |           BU517053           |                               |
|                       |           BG282798           |                               |
|                       |           BP369110           |                               |
|                       |           BF033927           |                               |
|                       |           BE786410           |                               |
|                       |           CN335606           |                               |
|                       |           CN335672           |                               |
|                       |           CD242222           |                               |
|                       |           AA081760           |                               |
|                       |           AA191406           |                               |
|                       |           BU790454           |                               |
|                       |           CN335638           |                               |
|                       |           CN335664           |                               |
|                       |           BG704614           |                               |
|                       |             T31093           |                               |
|                       |           AA223417           |                               |
|                       |           BF820152           |                               |
|                       |           AA312300           |                               |
|                       |           BI713130           |                               |
|                       |           BE889989           |                               |
|                       |           BM801335           |                               |
|                       |           BU507891           |                               |
|                       |           CR606364           |                               |
|                       |             T36132           |                               |
|                       |           CN335652           |                               |
|                       |           CD358598           |                               |
|                       |           CN335651           |                               |
|                       |           AV704993           |                               |
|                       |           BG536491           |                               |
|                       |             R30767           |                               |
|                       |           CN335677           |                               |
|                       |           CN335610           |                               |
|                       |           BG983924           |                               |
|                       |           BF172652           |                               |
|                       |           AV749043           |                               |
|                       |           BE378572           |                               |
|                       |           CN418077           |                               |
|                       |           BE908588           |                               |
|                       |           CN335632           |                               |
|                       |           AW372979           |                               |
|                       |           BF895957           |                               |
|                       |           BU790313           |                               |
|                       |           BE546679           |                               |
|                       |           BM738694           |                               |
|                       |           BM557465           |                               |
|                       |           BM049282           |                               |
|                       |           AA115682           |                               |
|                       |           BQ069274           |                               |
|                       |           BG764901           |                               |
|                       |           CN335663           |                               |
|                       |           BG978908           |                               |
|                       |           BG572549           |                               |
|                       |           AW238568           |                               |
|                       |             T08274           |                               |
|                       |           BE883425           |                               |
|                       |           CN335659           |                               |
|                       |           BM696407           |                               |
|                       |           CB146107           |                               |
|                       |           BE872941           |                               |
|                       |           CD243142           |                               |
|                       |           BF341069           |                               |
|                       |           AA984650           |                               |
|                       |           CN418079           |                               |
|                       |           BG108068           |                               |
|                       |           BE910526           |                               |
|                       |           BG705166           |                               |
|                       |           BE940649           |                               |
|                       |           CB114154           |                               |
|                       |           BE180657           |                               |
|                       |           AW402338           |                               |
|                       |           CN335616           |                               |
|                       |           BG944831           |                               |
|                       |           BG575515           |                               |
|                       |           BM754480           |                               |
|                       |           BE163141           |                               |
|                       |           CF140935           |                               |
|                       |           AL046936           |                               |
|                       |           AF131847           |                               |
|                       |           BE172777           |                               |
|                       |           CF144717           |                               |
|                       |           CF122369           |                               |
|                       |           BG716579           |                               |
|                       |           BG035439           |                               |
|                       |           CN335644           |                               |
|                       |           CD356359           |                               |
|                       |           BG286960           |                               |
|                       |           BF691527           |                               |
|                       |           BG180655           |                               |
|                       |           BG432885           |                               |
|                       |           CD000300           |                               |
|                       |           BU932394           |                               |
|                       |           BG752528           |                               |
|                       |           CN335613           |                               |
|                       |           CB215208           |                               |
|                       |           BE542128           |                               |
|                       |           BE617152           |                               |
|                       |           CN335647           |                               |
|                       |           BG028289           |                               |
|                       |           BE888139           |                               |
|                       |           BG171815           |                               |
|                       |           BG994286           |                               |
|                       |           AI929039           |                               |
|                       |           BU930437           |                               |
|                       |           BG686855           |                               |
|                       |           BM810014           |                               |
|                       |           CF140871           |                               |
|                       |           CB853123           |                               |
|                       |           BI335555           |                               |
|                       |           BE926984           |                               |
|                       |           BG572087           |                               |
|                       |           BG987765           |                               |
|                       |           AI366471           |                               |
|                       |           AI216973           |                               |
|                       |           BI004875           |                               |
|                       |           BM556610           |                               |
|                       |           BG025114           |                               |
|                       |           BQ354025           |                               |
|                       |           BF790555           |                               |
|                       |           CF141392           |                               |
|                       |           BM263012           |                               |
|                       |           BE894315           |                               |
|                       |           BE894318           |                               |
|                       |           AA337149           |                               |
|                       |           BF968884           |                               |
|                       |           BF968891           |                               |
|                       |           BI033487           |                               |
|                       |           BF575156           |                               |
|                       |           BM821707           |                               |
|                       |           CB135423           |                               |
|                       |           BM706375           |                               |
|                       |           BU621490           |                               |
|                       |           BU618589           |                               |
|                       |           CB529808           |                               |
|                       |           CF141973           |                               |
|                       |           AL517479           |                               |
|                       |           CR625082           |                               |
|                       |           AL517478           |                               |
|                       |           CA412864           |                               |
|                       |           BU663171           |                               |
|                       |           BU620130           |                               |
|                       |           AA383000           |                               |
|                       |           BE972486           |                               |
|                       |           BE936398           |                               |
|                       |           BM831238           |                               |
|                       |             R25697           |                               |
|                       |             R34386           |                               |
|                       |             R58618           |                               |
|                       |           BE887951           |                               |
|                       |           AW998136           |                               |
|                       |           BE788367           |                               |
|                       |           BE735297           |                               |
|                       |             R14235           |                               |
|                       |             R98792           |                               |
|                       |           AV705396           |                               |
|                       |           BF971030           |                               |
|                       |           BM669150           |                               |
|                       |           AW994485           |                               |
|                       |           AW994490           |                               |
|                       |           BE694094           |                               |
|                       |           BE694104           |                               |
|                       |             W03005           |                               |
|                       |           BM702665           |                               |
|                       |           CB163049           |                               |
|                       |           CD678477           |                               |
|                       |           BU634339           |                               |
|                       |           BG120821           |                               |
|                       |           BM142588           |                               |
|                       |             T99994           |                               |
|                       |           BE709824           |                               |
|                       |           AI215823           |                               |
|                       |           CB164492           |                               |
|                       |           CB164327           |                               |
|                       |           BG430314           |                               |
|                       |           AW992848           |                               |
|                       |           BX378328           |                               |
|                       |           AL518717           |                               |
|                       |           AV716502           |                               |
|                       |             N44654           |                               |
|                       |           AA328617           |                               |
|                       |           AW962400           |                               |
|                       |           AW962406           |                               |
|                       |           BU621736           |                               |
|                       |           CA424296           |                               |
|                       |             W78205           |                               |
|                       |           BU627751           |                               |
|                       |           AV715823           |                               |
|                       |           AL581520           |                               |
|                       |           BF340288           |                               |
|                       |           BE327864           |                               |
|                       |           CN335622           |                               |
|                       |           BX364919           |                               |
|                       |           BU600838           |                               |
|                       |           BG570854           |                               |
|                       |           AL565419           |                               |
|                       |           BM762217           |                               |
|                       |           CN335673           |                               |
|                       |           CA411735           |                               |
|                       |           AW999320           |                               |
|                       |           AA385479           |                               |
|                       |           BM823486           |                               |
|                       |           AV727914           |                               |
|                       |           BG292181           |                               |
|                       |           BG741158           |                               |
|                       |           AA173584           |                               |
|                       |           BE868027           |                               |
|                       |           BU076720           |                               |
|                       |           BQ228284           |                               |
|                       |           CA422255           |                               |
|                       |           BF033113           |                               |
|                       |           BG676814           |                               |
|                       |           BQ671132           |                               |
|                       |           AI453357           |                               |
|                       |             R93548           |                               |
|                       |           AA757134           |                               |
|                       |           BE885168           |                               |
|                       |           CB142337           |                               |
|                       |           AL566872           |                               |
|                       |           BQ030047           |                               |
|                       |             T32700           |                               |
|                       |           BE047748           |                               |
|                       |           CB162882           |                               |
|                       |           BF213050           |                               |
|                       |           BE178427           |                               |
|                       |           BE178115           |                               |
|                       |           BE177939           |                               |
|                       |           BG619015           |                               |
|                       |             Z20674           |                               |
|                       |           BM693969           |                               |
|                       |           BM663966           |                               |
|                       |           BM668446           |                               |
|                       |           AA977812           |                               |
|                       |           BG770962           |                               |
|                       |           CD641404           |                               |
|                       |           AL567339           |                               |
|                       |           BM797316           |                               |
|                       |           AA527347           |                               |
|                       |           AL568361           |                               |
|                       |           CF552900           |                               |
|                       |           CR737335           |                               |
|                       |           AW020060           |                               |
|                       |           AA165020           |                               |
|                       |           AL567332           |                               |
|                       |           CN335626           |                               |
|                       |           BF791656           |                               |
|                       |           BE935095           |                               |
|                       |           BX383236           |                               |
|                       |           CR624868           |                               |
|                       |           BQ631868           |                               |
|                       |           BF030785           |                               |
|                       |           AI634263           |                               |
|                       |           AI026926           |                               |
|                       |           BG033229           |                               |
|                       |           CD357284           |                               |
|                       |           BE542018           |                               |
|                       |           BE172102           |                               |
|                       |           AA190463           |                               |
|                       |           CB240944           |                               |
|                       |             N42881           |                               |
|                       |           BX281371           |                               |
|                       |             W73434           |                               |
|                       |             N42856           |                               |
|                       |           AL534533           |                               |
|                       |           AA128721           |                               |
|                       |           BU579530           |                               |
|                       |           BE710100           |                               |
|                       |           BU074268           |                               |
|                       |           BE000411           |                               |
|                       |           AW013984           |                               |
|                       |           BU620653           |                               |
|                       |           BI023688           |                               |
|                       |             R80750           |                               |
|                       |           BX378590           |                               |
|                       |           AW602384           |                               |
|                       |           AA136153           |                               |
|                       |           BG008407           |                               |
|                       |           CR740621           |                               |
|                       |             N36285           |                               |
|                       |           AV715094           |                               |
|                       |           BG283833           |                               |
|                       |           BF668549           |                               |
|                       |             F30926           |                               |
|                       |             F36529           |                               |
|                       |           BE163914           |                               |
|                       |           BE163593           |                               |
|                       |           BF246516           |                               |
|                       |           AA047677           |                               |
|                       |           AA047710           |                               |
|                       |             W51928           |                               |
|                       |           BF699554           |                               |
|                       |           BF772560           |                               |
|                       |           BI049017           |                               |
|                       |           BE907652           |                               |
|                       |             N83719           |                               |
|                       |           BU075413           |                               |
|                       |           CK005109           |                               |
|                       |           BX372898           |                               |
|                       |             W93180           |                               |
|                       |             R58005           |                               |
|                       |           BU679610           |                               |
|                       |           AI609237           |                               |
|                       |           BU532810           |                               |
|                       |           BM311150           |                               |
|                       |           CF529468           |                               |
|                       |             W49825           |                               |
|                       |           BI060619           |                               |
|                       |             C16372           |                               |
|                       |           BG572483           |                               |
|                       |           BM786700           |                               |
|                       |           AI052705           |                               |
|                       |           BQ775740           |                               |
|                       |           BM999555           |                               |
|                       |           BQ008134           |                               |
|                       |           CN335637           |                               |
|                       |           BU684522           |                               |
|                       |           BM999896           |                               |
|                       |           BU684061           |                               |
|                       |           CB306040           |                               |
|                       |           BU617027           |                               |
|                       |           BM843895           |                               |
|                       |           BM995374           |                               |
|                       |           BQ574081           |                               |
|                       |           AW778937           |                               |
|                       |           BQ017956           |                               |
|                       |           BM995044           |                               |
|                       |           BU683666           |                               |
|                       |           BI491086           |                               |
|                       |           BM970198           |                               |
|                       |           BM973626           |                               |
|                       |           BU618910           |                               |
|                       |           BU678508           |                               |
|                       |           BU627912           |                               |
|                       |           AV721981           |                               |
|                       |           BU787590           |                               |
|                       |           BU077572           |                               |
|                       |           BM979455           |                               |
|                       |           BM969996           |                               |
|                       |           BQ267880           |                               |
|                       |           BM995325           |                               |
|                       |           BP255293           |                               |
|                       |           CB136406           |                               |
|                       |           BQ632167           |                               |
|                       |           AI928872           |                               |
|                       |           AI041829           |                               |
|                       |           CA415294           |                               |
|                       |           BU729799           |                               |
|                       |           BM995234           |                               |
|                       |           BM995016           |                               |
|                       |           CA440721           |                               |
|                       |           BU674101           |                               |
|                       |           AI057100           |                               |
|                       |           BM996530           |                               |
|                       |           AA809076           |                               |
|                       |           BQ632069           |                               |
|                       |           AI753765           |                               |
|                       |           CN335614           |                               |
|                       |           BM971260           |                               |
|                       |           AW130490           |                               |
|                       |           AA778087           |                               |
|                       |           BQ029381           |                               |
|                       |           AI583649           |                               |
|                       |           AI583931           |                               |
|                       |           AI937304           |                               |
|                       |           AI299238           |                               |
|                       |           AI190949           |                               |
|                       |             W48673           |                               |
|                       |           BI496123           |                               |
|                       |           AA602670           |                               |
|                       |           AI096355           |                               |
|                       |           AI753332           |                               |
|                       |           AA703919           |                               |
|                       |           BI495028           |                               |
|                       |           BI495046           |                               |
|                       |           AL047743           |                               |
|                       |           AI866531           |                               |
|                       |           BM668831           |                               |
|                       |           AI929829           |                               |
|                       |           AA134177           |                               |
|                       |           AI263698           |                               |
|                       |           BE350903           |                               |
|                       |           AW069880           |                               |
|                       |           AA854828           |                               |
|                       |           CA946829           |                               |
|                       |           BM768330           |                               |
|                       |           AI264873           |                               |
|                       |           AI311564           |                               |
|                       |           BM840085           |                               |
|                       |           AI524369           |                               |
|                       |           BU733752           |                               |
|                       |           AW192189           |                               |
|                       |           AV737315           |                               |
|                       |           AI559401           |                               |
|                       |           BU732315           |                               |
|                       |           AA876050           |                               |
|                       |           CD678572           |                               |
|                       |           AI041220           |                               |
|                       |           AI923294           |                               |
|                       |           AI184059           |                               |
|                       |           BM664244           |                               |
|                       |           AW473188           |                               |
|                       |           AI868769           |                               |
|                       |           BM728572           |                               |
|                       |           CA449931           |                               |
|                       |           AW205905           |                               |
|                       |           CA310705           |                               |
|                       |           CB144139           |                               |
|                       |           CB242356           |                               |
|                       |           BM993953           |                               |
|                       |           CA435691           |                               |
|                       |             H17332           |                               |
|                       |             R37171           |                               |
|                       |           AA470143           |                               |
|                       |           CB852160           |                               |
|                       |           CN335655           |                               |
|                       |           AV740284           |                               |
|                       |           BQ029377           |                               |
|                       | *Seq. mapped in strand Minus*| *Seq. mapped in strand Minus* |
|                       |           BU690065           |          BF450967             |
+-----------------------+------------------------------+-------------------------------+
|      #  116           | *Seq. mapped in strand Plus* | *Seq. mapped in strand Plus*  |
|                       |           AA806776           |          AK040429             |
|                       |           AA826101           |                               |
|                       | *Seq. mapped in strand Minus*| *Seq. mapped in strand Minus* |
|                       |           AY008294           |          AK047925             |
|                       |           AF130863           |          AF044257             |
|                       |           BC028936           |          AK044851             |
|                       |           AY587062           |          BC055378             |
|                       |           AY587063           |          BC050049             |
|                       |           AY587064           |                               |
|                       |           AY587065           |                               |
|                       |           AY587066           |                               |
|                       |           AY587067           |                               |
|                       |           AY587068           |                               |
|                       |           AY587069           |                               |
|                       |           AY587070           |                               |
|                       |           AY587071           |                               |
|                       |           AY587072           |                               |
|                       |           AY587073           |                               |
|                       |           BG393422           |                               |
|                       |           BP381096           |                               |
|                       |           CD707416           |                               |
+-----------------------+------------------------------+-------------------------------+
|      #  117           | *Seq. mapped in strand Plus* | *Seq. mapped in strand Plus*  |
|                       |           AK125403           |          BB374260             |
|                       |           BI552152           |                               |
|                       |           BC036067           |                               |
|                       |           AK091937           |                               |
|                       |           BP239419           |                               |
|                       |           AK056749           |                               |
|                       |           AU122709           |                               |
|                       |           AK024208           |                               |
|                       |           CB996449           |                               |
|                       |           BP382077           |                               |
|                       |           CB156547           |                               |
|                       |           BX371176           |                               |
|                       |           AI800764           |                               |
|                       |           BF508119           |                               |
|                       |           AI313418           |                               |
|                       |           AI800741           |                               |
|                       |           AI761941           |                               |
|                       |           AI766415           |                               |
|                       |           AA918416           |                               |
|                       |           AI805167           |                               |
|                       |           AW299838           |                               |
|                       |           BX326367           |                               |
|                       |           BF439587           |                               |
|                       |           AI807037           |                               |
|                       |           AA744567           |                               |
|                       |           BM007490           |                               |
|                       |           BQ710303           |                               |
|                       |           BQ711722           |                               |
|                       |           BQ706533           |                               |
|                       |           BX338054           |                               |
|                       |           CR598857           |                               |
|                       | *Seq. mapped in strand Minus*| *Seq. mapped in strand Minus* |
|                       |           BM674130           |          AK084264             |
+-----------------------+------------------------------+-------------------------------+
|      #  118           | *Seq. mapped in strand Plus* | *Seq. mapped in strand Plus*  |
|                       |           CR743993           |          AK016164             |
|                       | *Seq. mapped in strand Minus*| *Seq. mapped in strand Minus* |
|                       |           AY136549           |          AY135562             |
|                       |           AF044578           |          BC027393             |
|                       |           AL122048           |          BC023882             |
|                       |           CA421871           |          BC025497             |
|                       |           AI674093           |          AK049414             |
|                       |           AK131239           |                               |
|                       |           AA436996           |                               |
|                       |           BX105243           |                               |
|                       |           BX509164           |                               |
+-----------------------+------------------------------+-------------------------------+
|      #  119           | *Seq. mapped in strand Plus* | *Seq. mapped in strand Plus*  |
|                       |           BI667392           |          AK087258             |
|                       |           BG715413           |          AK036571             |
|                       |           AK056210           |          AK081002             |
|                       |           BU844674           |          AK036282             |
|                       |           BC068992           |          AK081605             |
|                       |           BF206152           |          BC030861             |
|                       |           BF689824           |          BC069851             |
|                       |           BC007639           |                               |
|                       |           BI559616           |                               |
|                       |           BM006622           |                               |
|                       |           BQ897806           |                               |
|                       |           AI830881           |                               |
|                       |           AW024569           |                               |
|                       |           AW024282           |                               |
|                       |           AW170307           |                               |
|                       |           AW192605           |                               |
|                       |           AJ312332           |                               |
|                       |           AI971639           |                               |
|                       |           BE737036           |                               |
|                       |           BM560604           |                               |
|                       |           BE790704           |                               |
|                       |           BI117023           |                               |
|                       |           BE392066           |                               |
|                       |           CA489840           |                               |
|                       |           BQ674063           |                               |
|                       |           BQ879986           |                               |
|                       |           BQ674330           |                               |
|                       |           BC016845           |                               |
|                       |           BE791979           |                               |
|                       |           BE887885           |                               |
|                       |           BM710114           |                               |
|                       |           BQ638019           |                               |
|                       |           BE563369           |                               |
|                       |           BX283408           |                               |
|                       |           BG762094           |                               |
|                       |           BQ893956           |                               |
|                       |           BM017835           |                               |
|                       |           BM019867           |                               |
|                       |           AA416624           |                               |
|                       |           BM719083           |                               |
|                       |           BQ691888           |                               |
|                       |           BQ685882           |                               |
|                       |           BU157350           |                               |
|                       |           BQ897613           |                               |
|                       |           BQ685568           |                               |
|                       |           BQ687308           |                               |
|                       |           BQ691009           |                               |
|                       |           BQ880521           |                               |
|                       |           AW246095           |                               |
|                       |           BM695036           |                               |
|                       |           BU553580           |                               |
|                       |           BM713152           |                               |
|                       |           CV029724           |                               |
|                       |           CB161138           |                               |
|                       |           BF690210           |                               |
|                       |           AW183194           |                               |
|                       |           BM675021           |                               |
|                       |           BM691966           |                               |
|                       |           BU681506           |                               |
|                       |           BM045055           |                               |
|                       |             W25662           |                               |
|                       |           CF132111           |                               |
|                       |           BF791801           |                               |
|                       |           BF791732           |                               |
|                       |           BM666820           |                               |
|                       |           BM683053           |                               |
|                       |           BU728461           |                               |
|                       |           BU615868           |                               |
|                       |           BF515667           |                               |
|                       |           CB851811           |                               |
|                       |           BM671285           |                               |
|                       |           AI824949           |                               |
|                       |           AW249556           |                               |
|                       |             F30370           |                               |
|                       |           CN366752           |                               |
|                       |             R43862           |                               |
|                       |           AI271998           |                               |
|                       |           BF593091           |                               |
|                       |           AI554573           |                               |
|                       | *Seq. mapped in strand Minus*| *Seq. mapped in strand Minus* |
|                       |           AA764729           |          BC021438             |
|                       |           BU739852           |          AK051997             |
|                       |           AW016135           |                               |
|                       |           BM676050           |                               |
|                       |           BM969710           |                               |
|                       |           AW469972           |                               |
|                       |           BU736470           |                               |
|                       |             W31199           |                               |
|                       |           BF513460           |                               |
|                       |           BF514423           |                               |
|                       |           BQ636190           |                               |
|                       |           BQ028941           |                               |
|                       |           BU608739           |                               |
|                       |           BC009876           |                               |
|                       |           BM683858           |                               |
|                       |           BU155562           |                               |
|                       |           BU157452           |                               |
|                       |           BU161157           |                               |
|                       |           BU634208           |                               |
|                       |           BQ050538           |                               |
|                       |           CD242745           |                               |
|                       |           AK025907           |                               |
|                       |           BC002890           |                               |
|                       |           AK023011           |                               |
|                       |           BG685960           |                               |
|                       |           BG755033           |                               |
|                       |           BG697987           |                               |
|                       |           CB269500           |                               |
|                       |           BG757872           |                               |
|                       |           BQ051780           |                               |
|                       |           BG481293           |                               |
|                       |           BG482167           |                               |
|                       |           BE258947           |                               |
|                       |           CF128264           |                               |
|                       |           BE281057           |                               |
|                       |           BE279266           |                               |
|                       |           BE278993           |                               |
|                       |           BG005126           |                               |
|                       |           CB123919           |                               |
|                       |           BG025766           |                               |
|                       |           BQ957365           |                               |
|                       |           BE792136           |                               |
|                       |           BM021009           |                               |
|                       |           BF878933           |                               |
|                       |           BM044638           |                               |
|                       |           BX283794           |                               |
|                       |           BF818349           |                               |
|                       |           BM044704           |                               |
|                       |           CN371991           |                               |
|                       |           BG324431           |                               |
|                       |           BU194530           |                               |
|                       |           CN371990           |                               |
|                       |           AA421738           |                               |
|                       |           BQ695143           |                               |
|                       |           BP253095           |                               |
|                       |           BG982331           |                               |
|                       |           BM008237           |                               |
|                       |           BQ683166           |                               |
|                       |           BU168623           |                               |
|                       |           BQ052530           |                               |
|                       |           BE747480           |                               |
|                       |           BP376425           |                               |
|                       |           BG680140           |                               |
|                       |           CO247791           |                               |
|                       |           CD631718           |                               |
|                       |           BE743915           |                               |
|                       |           CN371986           |                               |
|                       |           BG748605           |                               |
|                       |           BE799910           |                               |
|                       |           AA320472           |                               |
|                       |           BG489002           |                               |
|                       |           BG766777           |                               |
|                       |           BG770104           |                               |
|                       |           BG766386           |                               |
|                       |           AU124084           |                               |
|                       |           BG768148           |                               |
|                       |           BE378414           |                               |
|                       |           BQ950163           |                               |
|                       |           BQ918981           |                               |
|                       |           BX343824           |                               |
|                       |           BU528569           |                               |
|                       |           BU538709           |                               |
|                       |           BM738605           |                               |
|                       |           BI258525           |                               |
|                       |           BG764225           |                               |
|                       |           BU557995           |                               |
|                       |           BG403277           |                               |
|                       |           BU944168           |                               |
|                       |           BM452480           |                               |
|                       |           BE408531           |                               |
|                       |           BQ922121           |                               |
|                       |           CN371992           |                               |
|                       |           CN371988           |                               |
|                       |           AA355671           |                               |
|                       |           BF892166           |                               |
|                       |           BM557090           |                               |
|                       |           AL546178           |                               |
|                       |           BG761239           |                               |
|                       |           BM470633           |                               |
|                       |           BE262225           |                               |
|                       |           BE206823           |                               |
|                       |           CN371993           |                               |
|                       |           AU129335           |                               |
|                       |           BG761502           |                               |
|                       |           CN371994           |                               |
|                       |           BE276512           |                               |
|                       |           BE894766           |                               |
|                       |           BE541125           |                               |
|                       |           BP357691           |                               |
|                       |           BF336729           |                               |
|                       |           BF326261           |                               |
|                       |           CD631706           |                               |
|                       |           CD631709           |                               |
|                       |           CD631710           |                               |
|                       |           CD631707           |                               |
|                       |           CD631711           |                               |
|                       |           BM150288           |                               |
|                       |           BM747925           |                               |
|                       |           BG468603           |                               |
|                       |           CD631708           |                               |
|                       |           CO248962           |                               |
|                       |           BM833746           |                               |
|                       |           CN371989           |                               |
|                       |           BP372346           |                               |
|                       |           BP281551           |                               |
|                       |           BI116870           |                               |
|                       |           BM149791           |                               |
|                       |           AL039569           |                               |
|                       |           AW672783           |                               |
|                       |           BF352657           |                               |
|                       |           BG489997           |                               |
|                       |           AU280109           |                               |
|                       |           AW859926           |                               |
|                       |           BG765393           |                               |
|                       |           BQ318244           |                               |
|                       |           BM834915           |                               |
|                       |           CD631716           |                               |
|                       |           CD631713           |                               |
|                       |           CD631714           |                               |
|                       |           CD631712           |                               |
|                       |           CD631715           |                               |
|                       |           CN371987           |                               |
+-----------------------+------------------------------+-------------------------------+
|      #  120           | *Seq. mapped in strand Plus* | *Seq. mapped in strand Plus*  |
|                       |           BM475351           |          BY014621             |
|                       |                              |          BY198423             |
|                       |                              |          AK052295             |
|                       |                              |          BY011310             |
|                       |                              |          BB659111             |
|                       |                              |          BB867692             |
|                       |                              |          BU709204             |
|                       |                              |          BU609264             |
|                       |                              |          BC055719             |
|                       |                              |          BU614586             |
|                       |                              |          CA751637             |
|                       |                              |          BQ443180             |
|                       |                              |            X83587             |
|                       |                              |          BU057733             |
|                       |                              |          AW321202             |
|                       |                              |          CK782017             |
|                       |                              |          AW244507             |
|                       |                              |          BQ442586             |
|                       |                              |          BQ955395             |
|                       |                              |          CA320273             |
|                       |                              |          BQ570966             |
|                       |                              |          AL362036             |
|                       |                              |          BG277843             |
|                       |                              |          CA979084             |
|                       |                              |          CB202531             |
|                       |                              |          BQ748930             |
|                       |                              |          BU918286             |
|                       | *Seq. mapped in strand Minus*| *Seq. mapped in strand Minus* |
|                       |           BM678625           |          BU519690             |
|                       |           AI962660           |                               |
|                       |           BG056912           |                               |
|                       |           BG222933           |                               |
|                       |           BF528119           |                               |
|                       |           BM682108           |                               |
|                       |           BM683018           |                               |
|                       |           BG056049           |                               |
|                       |           BC010608           |                               |
|                       |           AW444628           |                               |
|                       |           BC023587           |                               |
|                       |           BQ880948           |                               |
|                       |           CN368257           |                               |
|                       |           CN368254           |                               |
|                       |           CN368258           |                               |
|                       |           BU856438           |                               |
|                       |           CN368255           |                               |
|                       |           BM719037           |                               |
|                       |           BI914592           |                               |
|                       |           CN368256           |                               |
|                       |           BF530276           |                               |
|                       |           BM724284           |                               |
|                       |           BF944639           |                               |
|                       |           BG913127           |                               |
|                       |           BG958612           |                               |
|                       |           AI903098           |                               |
|                       |           BF944683           |                               |
|                       |           BG683007           |                               |
+-----------------------+------------------------------+-------------------------------+
|      #  121           | *Seq. mapped in strand Plus* | *Seq. mapped in strand Plus*  |
|                       |           BG701336           |          AK005725             |
|                       |           BG717416           |          BC049579             |
|                       |           BG718091           |          AK005749             |
|                       |           BC016742           |                               |
|                       |           BP369015           |                               |
|                       |           BI829610           |                               |
|                       |           BM554447           |                               |
|                       |           CR739356           |                               |
|                       |           AF368276           |                               |
|                       |           AL133660           |                               |
|                       |           CV024294           |                               |
|                       |           AI126166           |                               |
|                       |           BX336643           |                               |
|                       |           BX336644           |                               |
|                       |           CR607613           |                               |
|                       |           AI223433           |                               |
|                       |           BF115620           |                               |
|                       |           AA812978           |                               |
|                       |           BU627018           |                               |
|                       |           AW235250           |                               |
|                       | *Seq. mapped in strand Minus*| *Seq. mapped in strand Minus* |
|                       |           BQ447977           |          AK006881             |
+-----------------------+------------------------------+-------------------------------+
|      #  122           | *Seq. mapped in strand Plus* | *Seq. mapped in strand Plus*  |
|                       |           CR607695           |            U28807             |
|                       |           CR601429           |            D85612             |
|                       |                              |          BC021835             |
|                       |                              |          AK088536             |
|                       |                              |          BC049877             |
|                       | *Seq. mapped in strand Minus*| *Seq. mapped in strand Minus* |
|                       |           AK025571           |          AK088755             |
|                       |           AF289577           |          AK020578             |
|                       |           BC052309           |          BC031444             |
|                       |           BC030146           |                               |
|                       |           BC069241           |                               |
|                       |           AK025901           |                               |
|                       |           CR592167           |                               |
+-----------------------+------------------------------+-------------------------------+
|      #  123           | *Seq. mapped in strand Plus* | *Seq. mapped in strand Plus*  |
|                       |           BM559979           |          AK048878             |
|                       |           BP308677           |                               |
|                       |           BP347334           |                               |
|                       |           BP370285           |                               |
|                       |           BM822247           |                               |
|                       |           BP307236           |                               |
|                       |           BM554407           |                               |
|                       |           AK091894           |                               |
|                       |           AK090908           |                               |
|                       |           BG715284           |                               |
|                       |           BQ067936           |                               |
|                       |           CN301657           |                               |
|                       |           BP200945           |                               |
|                       |           BG497654           |                               |
|                       |           BP370642           |                               |
|                       |           BP369050           |                               |
|                       |           BE266927           |                               |
|                       |           AL517153           |                               |
|                       |           BU932118           |                               |
|                       |           AI815694           |                               |
|                       |           AI929609           |                               |
|                       |           AI929167           |                               |
|                       |           CN301656           |                               |
|                       |           BM704370           |                               |
|                       |           BM465903           |                               |
|                       |           BX394682           |                               |
|                       |           BC064963           |                               |
|                       |           BQ425447           |                               |
|                       |           CN301659           |                               |
|                       |           BU169415           |                               |
|                       |           CD105092           |                               |
|                       |           BP231758           |                               |
|                       |           BE896604           |                               |
|                       |           BM923692           |                               |
|                       |           BX471119           |                               |
|                       |           BI760606           |                               |
|                       |           BI764769           |                               |
|                       |           BE780781           |                               |
|                       |           AF411076           |                               |
|                       |           BQ639206           |                               |
|                       |           BU850168           |                               |
|                       |           BE799259           |                               |
|                       |           AL556649           |                               |
|                       |           CR617167           |                               |
|                       |           BE798094           |                               |
|                       |           BX336607           |                               |
|                       |           CB116680           |                               |
|                       |           BE783412           |                               |
|                       |           BI820781           |                               |
|                       |           BC021295           |                               |
|                       |           AA351116           |                               |
|                       |           BU156650           |                               |
|                       |           BM686124           |                               |
|                       |           BP349730           |                               |
|                       |           BQ440019           |                               |
|                       |           BI256302           |                               |
|                       |           AI962178           |                               |
|                       |           AW301662           |                               |
|                       |           BE777997           |                               |
|                       |           CN301658           |                               |
|                       |           BI031063           |                               |
|                       |           AA206437           |                               |
|                       |           AA906865           |                               |
|                       |           BU183811           |                               |
|                       |           AI655904           |                               |
|                       |           BF056302           |                               |
|                       |           CD632435           |                               |
|                       |           BX329263           |                               |
|                       |           BC006267           |                               |
|                       |           BE899106           |                               |
|                       |           BU633667           |                               |
|                       |           BU730531           |                               |
|                       |           BQ343733           |                               |
|                       |           BM930627           |                               |
|                       |           BX640832           |                               |
|                       |           AW131805           |                               |
|                       |           AI879995           |                               |
|                       |           AI928914           |                               |
|                       |           AI978667           |                               |
|                       |           AW058236           |                               |
|                       |           CD632436           |                               |
|                       |           AK091403           |                               |
|                       |           BM662965           |                               |
|                       |           BU743279           |                               |
|                       |           BG757114           |                               |
|                       |           BG759764           |                               |
|                       |           BF663368           |                               |
|                       |           BF975745           |                               |
|                       |           BF975953           |                               |
|                       |           BE938790           |                               |
|                       |           CB215455           |                               |
|                       |           BX370440           |                               |
|                       |           BI033660           |                               |
|                       |           AI561010           |                               |
|                       |           AA865374           |                               |
|                       |           CR620676           |                               |
|                       |           BQ646012           |                               |
|                       |           BU685745           |                               |
|                       |           BU688025           |                               |
|                       |           BM674704           |                               |
|                       |           AI928342           |                               |
|                       |           AW450906           |                               |
|                       |           AI810254           |                               |
|                       |           AI401797           |                               |
|                       |           AW612512           |                               |
|                       |           AI628890           |                               |
|                       |           AI656166           |                               |
|                       |           AI418296           |                               |
|                       |           AI355271           |                               |
|                       |           AI628902           |                               |
|                       |           AW516418           |                               |
|                       |           AI990291           |                               |
|                       |           AW291921           |                               |
|                       |           AW206730           |                               |
|                       | *Seq. mapped in strand Minus*| *Seq. mapped in strand Minus* |
|                       |           AI628653           |          AK030209             |
|                       |           AI628631           |          BC016106             |
|                       |           AW243454           |          AK010381             |
|                       |           BF434744           |          AK006147             |
+-----------------------+------------------------------+-------------------------------+
|      #  124           | *Seq. mapped in strand Plus* | *Seq. mapped in strand Plus*  |
|                       |           AW303071           |          BE995465             |
|                       |                              |          BF721761             |
|                       | *Seq. mapped in strand Minus*| *Seq. mapped in strand Minus* |
|                       |           AA433921           |          CO044467             |
|                       |           AI682131           |          BU700744             |
+-----------------------+------------------------------+-------------------------------+
|      #  125           | *Seq. mapped in strand Plus* | *Seq. mapped in strand Plus*  |
|                       |           AF412034           |          BY240737             |
|                       |           BC001780           |          BY291883             |
|                       |           AF420248           |          BY287672             |
|                       |           BC011696           |          BY289537             |
|                       |           AK091162           |          BY286144             |
|                       |           CR623572           |          BY298378             |
|                       |           BC000169           |          BY287951             |
|                       |           CR615237           |          BY272343             |
|                       |           CR602260           |          BY259095             |
|                       |           CR603690           |          BY027248             |
|                       |           AF218007           |          BY283011             |
|                       |           CR623973           |          BY279140             |
|                       |           AK126224           |          BU848304             |
|                       |                              |          BY267629             |
|                       |                              |          BY295867             |
|                       |                              |          BY253046             |
|                       |                              |          BY256961             |
|                       |                              |          BY243837             |
|                       |                              |          BY245097             |
|                       |                              |          BY297323             |
|                       |                              |          BY266822             |
|                       |                              |          BY278820             |
|                       |                              |          BY275921             |
|                       |                              |          BY286583             |
|                       |                              |          BY277176             |
|                       |                              |          BY243068             |
|                       |                              |          BY255815             |
|                       |                              |          BI655470             |
|                       |                              |          BY257047             |
|                       |                              |          BY241525             |
|                       |                              |          BY241710             |
|                       |                              |          BY011017             |
|                       |                              |          BC057892             |
|                       |                              |          BG974180             |
|                       |                              |          BY269317             |
|                       |                              |          BY267035             |
|                       |                              |          BY725109             |
|                       |                              |          BY252887             |
|                       |                              |          BY259570             |
|                       |                              |          BY275415             |
|                       |                              |          BY259037             |
|                       |                              |          BY254453             |
|                       |                              |          BY271952             |
|                       |                              |          BY126109             |
|                       |                              |          BY289752             |
|                       |                              |          BY293140             |
|                       |                              |          BY289038             |
|                       |                              |          BY260853             |
|                       |                              |          BY260632             |
|                       |                              |          BY244209             |
|                       |                              |          BY248224             |
|                       |                              |          BB648021             |
|                       |                              |          BY291867             |
|                       |                              |          BY258600             |
|                       |                              |          BQ715840             |
|                       |                              |          BU054848             |
|                       |                              |          BY263250             |
|                       |                              |          BC047133             |
|                       |                              |            D45208             |
|                       |                              |          BU146391             |
|                       |                              |          BU707435             |
|                       |                              |          BY095753             |
|                       |                              |          CN709169             |
|                       |                              |          BU057334             |
|                       |                              |          BY278244             |
|                       |                              |          BM949361             |
|                       |                              |          CD364178             |
|                       |                              |          BF118947             |
|                       |                              |          BF118920             |
|                       |                              |          CK794836             |
|                       |                              |          BE226499             |
|                       |                              |          BI109765             |
|                       |                              |          BC023379             |
|                       |                              |          BE944979             |
|                       |                              |          BE989548             |
|                       |                              |          AI837875             |
|                       |                              |          AI844347             |
|                       |                              |          BE946649             |
|                       |                              |          AW049639             |
|                       | *Seq. mapped in strand Minus*| *Seq. mapped in strand Minus* |
|                       |           CR621226           |          BE986802             |
|                       |             D37932           |                               |
|                       |           BC064644           |                               |
|                       |           BC000444           |                               |
|                       |           BC003011           |                               |
|                       |             L37792           |                               |
|                       |           CR601836           |                               |
|                       |             U12918           |                               |
|                       |           CR457018           |                               |
|                       |           AB086954           |                               |
+-----------------------+------------------------------+-------------------------------+
|      #  126           | *Seq. mapped in strand Plus* | *Seq. mapped in strand Plus*  |
|                       |           AF075045           |          AW045143             |
|                       |                              |          BY732316             |
|                       |                              |          BY274419             |
|                       |                              |          BY067795             |
|                       |                              |          BY278130             |
|                       |                              |          BY281120             |
|                       |                              |          BY251334             |
|                       |                              |          BY270110             |
|                       |                              |          BY244132             |
|                       |                              |          BB846311             |
|                       |                              |          AU080391             |
|                       |                              |          AU080201             |
|                       |                              |          AU080380             |
|                       |                              |          AI317499             |
|                       |                              |          BG922687             |
|                       |                              |          BY244630             |
|                       |                              |          BY256796             |
|                       |                              |          BY345659             |
|                       |                              |          BY786664             |
|                       |                              |          BY321633             |
|                       |                              |          BY345157             |
|                       |                              |          BY244786             |
|                       |                              |          BY255434             |
|                       |                              |          BY105512             |
|                       |                              |          BF781215             |
|                       |                              |          BY073266             |
|                       |                              |          BY316482             |
|                       |                              |          BY163592             |
|                       |                              |          BG962155             |
|                       |                              |          BI663647             |
|                       |                              |          BY268323             |
|                       |                              |          AI929978             |
|                       |                              |          BY324456             |
|                       |                              |          BY295170             |
|                       |                              |          CA461908             |
|                       |                              |          BY317792             |
|                       |                              |          BY101587             |
|                       |                              |          AA014394             |
|                       |                              |          BY703365             |
|                       |                              |          AB019212             |
|                       |                              |          AK002867             |
|                       |                              |          BY326276             |
|                       |                              |          CA890898             |
|                       |                              |          AI787931             |
|                       |                              |          CK331754             |
|                       |                              |          BY333131             |
|                       |                              |          BY736031             |
|                       |                              |          BB871865             |
|                       |                              |          AW610979             |
|                       |                              |          BY061616             |
|                       |                              |          AA619423             |
|                       |                              |          BI109004             |
|                       |                              |          BY327097             |
|                       |                              |          BY318332             |
|                       |                              |          BY069098             |
|                       |                              |          BY034295             |
|                       |                              |          BY250692             |
|                       |                              |          BY250123             |
|                       |                              |          BF140240             |
|                       |                              |          AW045162             |
|                       |                              |          AI036632             |
|                       |                              |          CD553403             |
|                       |                              |          BX518753             |
|                       |                              |          BY210938             |
|                       |                              |          BY120648             |
|                       |                              |          BY211930             |
|                       |                              |          BY222382             |
|                       |                              |          BY316581             |
|                       |                              |          BY276694             |
|                       |                              |          BY275829             |
|                       |                              |          BY218215             |
|                       |                              |          BY263297             |
|                       |                              |          BY206750             |
|                       |                              |          BY153235             |
|                       |                              |          AI527811             |
|                       |                              |          AI226684             |
|                       |                              |          BM053799             |
|                       |                              |          AI226664             |
|                       |                              |          BE571612             |
|                       |                              |          BE308823             |
|                       |                              |          BQ887678             |
|                       |                              |          AK082927             |
|                       |                              |          CB238166             |
|                       |                              |          CA534856             |
|                       |                              |          BF181353             |
|                       |                              |          AW318468             |
|                       |                              |          AW259741             |
|                       |                              |          AA118833             |
|                       |                              |          BB652469             |
|                       |                              |          BG296121             |
|                       |                              |          BU696388             |
|                       |                              |          CK790677             |
|                       |                              |          BY341571             |
|                       |                              |          BG970506             |
|                       |                              |          CA533803             |
|                       |                              |          CF157658             |
|                       |                              |          CF155429             |
|                       |                              |          CA549202             |
|                       |                              |          CF154345             |
|                       |                              |          AW106589             |
|                       |                              |          BI666323             |
|                       |                              |          BI687942             |
|                       |                              |          BU922882             |
|                       |                              |          AI157922             |
|                       |                              |          BF452341             |
|                       |                              |          BB842523             |
|                       |                              |          CB587631             |
|                       |                              |          CN673214             |
|                       |                              |          AW323907             |
|                       |                              |          AI529025             |
|                       |                              |          CA569383             |
|                       |                              |          BE629126             |
|                       |                              |          BI688008             |
|                       |                              |          BQ952649             |
|                       |                              |          CN712400             |
|                       |                              |          CN714735             |
|                       |                              |          BG915940             |
|                       |                              |          BG087754             |
|                       |                              |          BE308262             |
|                       |                              |          BB851379             |
|                       |                              |          AI527774             |
|                       |                              |          BG261751             |
|                       |                              |          AA498762             |
|                       |                              |          AF056323             |
|                       |                              |          AK013869             |
|                       |                              |          AI929977             |
|                       |                              |          AI181254             |
|                       |                              |          AW743596             |
|                       |                              |          CB954114             |
|                       |                              |          BG873191             |
|                       |                              |            W97604             |
|                       |                              |            W82671             |
|                       |                              |          CA534702             |
|                       |                              |          CA534767             |
|                       |                              |          BU057337             |
|                       |                              |          CD564292             |
|                       |                              |          BU053872             |
|                       |                              |          BI466692             |
|                       |                              |          AA404148             |
|                       |                              |          AI466880             |
|                       |                              |          BQ926228             |
|                       |                              |          BF783037             |
|                       |                              |          BE915820             |
|                       |                              |          BI557261             |
|                       |                              |          CK619924             |
|                       |                              |          CA323455             |
|                       |                              |          BY763155             |
|                       |                              |          BF016896             |
|                       |                              |          BQ935020             |
|                       |                              |          AW211057             |
|                       |                              |          BF536799             |
|                       |                              |          CA322332             |
|                       |                              |          BY756407             |
|                       |                              |          BU517715             |
|                       |                              |          AA111166             |
|                       | *Seq. mapped in strand Minus*| *Seq. mapped in strand Minus* |
|                       |           AK001367           |          BE987421             |
|                       |           AJ420529           |          BE987418             |
|                       |           AF131808           |          BE946700             |
|                       |           CR618907           |                               |
|                       |           CR596095           |                               |
|                       |             U77942           |                               |
|                       |           BC011975           |                               |
|                       |           CR615646           |                               |
|                       |           CR607128           |                               |
+-----------------------+------------------------------+-------------------------------+
|      #  127           | *Seq. mapped in strand Plus* | *Seq. mapped in strand Plus*  |
|                       |           BC040176           |          AK009282             |
|                       |           CN415210           |                               |
|                       |           BQ056975           |                               |
|                       |           BX093451           |                               |
|                       |           AI003843           |                               |
|                       |           AA428854           |                               |
|                       |           AA291377           |                               |
|                       |           AF435957           |                               |
|                       |           CN359801           |                               |
|                       |           CN359800           |                               |
|                       |           CN359802           |                               |
|                       |           BF038269           |                               |
|                       |           BG201372           |                               |
|                       |           BG186587           |                               |
|                       |           BG188632           |                               |
|                       |           BG210403           |                               |
|                       |           BG193365           |                               |
|                       |           BG215727           |                               |
|                       |           AW361411           |                               |
|                       |           BG212073           |                               |
|                       |           BG194988           |                               |
|                       |           BE089872           |                               |
|                       |           BG207251           |                               |
|                       |           BG260366           |                               |
|                       |             W16535           |                               |
|                       |           BX113570           |                               |
|                       |           AA446081           |                               |
|                       |           AA446064           |                               |
|                       |           BM726904           |                               |
|                       |           AA477085           |                               |
|                       |           BE719681           |                               |
|                       |           BE719721           |                               |
|                       |           BE719719           |                               |
|                       |             W40362           |                               |
|                       |           BE719697           |                               |
|                       |             R73714           |                               |
|                       |           BE719674           |                               |
|                       | *Seq. mapped in strand Minus*| *Seq. mapped in strand Minus* |
|                       |           AA774572           |          AK044108             |
|                       |           BX088751           |                               |
+-----------------------+------------------------------+-------------------------------+
|      #  128           | *Seq. mapped in strand Plus* | *Seq. mapped in strand Plus*  |
|                       |           AI760611           |          AF119498             |
|                       |           BF590750           |          AK086863             |
|                       |                              |          BC019440             |
|                       |                              |          BF319542             |
|                       |                              |          BM899327             |
|                       |                              |          BE948832             |
|                       |                              |          BU700764             |
|                       |                              |          AW226503             |
|                       |                              |          BG101161             |
|                       |                              |          AI430865             |
|                       | *Seq. mapped in strand Minus*| *Seq. mapped in strand Minus* |
|                       |           CA951052           |          CN840442             |
|                       |           BI966055           |                               |
|                       |           CK904088           |                               |
|                       |           BM996547           |                               |
|                       |           CA420937           |                               |
|                       |           BX107431           |                               |
|                       |           BU607817           |                               |
|                       |           CB852304           |                               |
|                       |           AF119497           |                               |
|                       |           BI966249           |                               |
|                       |           AK027081           |                               |
|                       |           BC052240           |                               |
|                       |           BC057799           |                               |
|                       |           BP340883           |                               |
|                       |           BC029825           |                               |
|                       |           BC038089           |                               |
+-----------------------+------------------------------+-------------------------------+
|      #  129           | *Seq. mapped in strand Plus* | *Seq. mapped in strand Plus*  |
|                       |           CR617045           |          BC050838             |
|                       |           BC053894           |            X55318             |
|                       |           CR600750           |          AK078758             |
|                       |           AK000445           |          AK012173             |
|                       |           BC032769           |                               |
|                       | *Seq. mapped in strand Minus*| *Seq. mapped in strand Minus* |
|                       |           BC044251           |          BC034904             |
+-----------------------+------------------------------+-------------------------------+
|      #  130           | *Seq. mapped in strand Plus* | *Seq. mapped in strand Plus*  |
|                       |           BC070217           |          CB598420             |
|                       |           BC070218           |          BY004534             |
|                       |           CR603137           |          BB611101             |
|                       |           BC000354           |          BY703131             |
|                       |           BC018810           |          BY066802             |
|                       |             L05091           |          AA879929             |
|                       |           CR606185           |          BC061055             |
|                       |             U58682           |          AK002623             |
|                       |           BC021239           |          BC037741             |
|                       |           CR457055           |          CN671136             |
|                       |                              |          BE653510             |
|                       |                              |          CN842590             |
|                       |                              |          AI595114             |
|                       |                              |          AA062096             |
|                       |                              |          BG970100             |
|                       |                              |          BG969956             |
|                       |                              |          CK021066             |
|                       |                              |          BQ829629             |
|                       |                              |          CA531804             |
|                       |                              |          BU604427             |
|                       |                              |          AA137792             |
|                       |                              |          BM943646             |
|                       |                              |          AA023356             |
|                       |                              |          AA914302             |
|                       |                              |          BX512813             |
|                       |                              |          AW989480             |
|                       |                              |          AI152556             |
|                       |                              |          CK128529             |
|                       |                              |          AA930684             |
|                       |                              |            W29230             |
|                       |                              |          CD775069             |
|                       |                              |          AW987582             |
|                       |                              |          AA832771             |
|                       |                              |          AA444543             |
|                       |                              |          CA465586             |
|                       |                              |          BM069051             |
|                       |                              |          AU021557             |
|                       |                              |            C79906             |
|                       |                              |          AV133960             |
|                       |                              |          AA200187             |
|                       |                              |          BF465699             |
|                       |                              |          AI172760             |
|                       |                              |          CK796367             |
|                       |                              |          BY420333             |
|                       |                              |          BX634925             |
|                       |                              |          BF181734             |
|                       |                              |          AW557792             |
|                       |                              |          AI847524             |
|                       | *Seq. mapped in strand Minus*| *Seq. mapped in strand Minus* |
|                       |           AK128815           |          BU698484             |
|                       |           BC066124           |                               |
|                       |           BC066775           |                               |
|                       |           BC035849           |                               |
+-----------------------+------------------------------+-------------------------------+
|      #  131           | *Seq. mapped in strand Plus* | *Seq. mapped in strand Plus*  |
|                       |             J03464           |          BY139890             |
|                       |           BP364653           |          BY148436             |
|                       |           BP286359           |          BY344156             |
|                       |           BP285732           |          BY778462             |
|                       |           BP374933           |          BY785874             |
|                       |           BP321621           |          CA325264             |
|                       |             Z74616           |          BY308222             |
|                       |           CD607474           |          CD348331             |
|                       |           CD607475           |          BY138414             |
|                       |           CN390634           |          BY316578             |
|                       |           CN390371           |          BY353095             |
|                       |           CK000431           |          CF734767             |
|                       |           CB045027           |          BY338082             |
|                       |           CK000631           |          BY719375             |
|                       |           CK000782           |          BY319926             |
|                       |           CN390425           |          BY315181             |
|                       |           CN390306           |          BY320298             |
|                       |           CN390304           |          BY320089             |
|                       |           CN390427           |          BY145889             |
|                       |           CN390395           |          BY774847             |
|                       |           CA392685           |          BY149600             |
|                       |           CN390671           |          BY146801             |
|                       |           CN390301           |          BY149916             |
|                       |           CK000331           |          BY784724             |
|                       |           CN390607           |          BY149189             |
|                       |           CN390344           |          BY781635             |
|                       |           AI582550           |          BY781658             |
|                       |           CN390592           |          BY781864             |
|                       |           BI459605           |          BY778298             |
|                       |           CD607449           |          BY782909             |
|                       |           CN484424           |          BY147177             |
|                       |           CD607505           |          BY782051             |
|                       |           CN390402           |          BY151608             |
|                       |           CD607451           |          BY141639             |
|                       |           CN390493           |          BY144179             |
|                       |           CD607445           |          BY145190             |
|                       |           CN390633           |          BY134944             |
|                       |           CD607443           |          BY310423             |
|                       |           CD607447           |          BY792431             |
|                       |           CD607453           |          BY138702             |
|                       |           CD607495           |          BY774977             |
|                       |           CN390538           |          BY131792             |
|                       |           CD655883           |          BY771271             |
|                       |           CN390313           |          BY319532             |
|                       |           CN390309           |          BY131658             |
|                       |           CN390621           |          BY777658             |
|                       |           CN390678           |          BY133590             |
|                       |           CN390463           |          BY137953             |
|                       |           CN390555           |          BY767629             |
|                       |           CN390551           |          BY135916             |
|                       |           CN390308           |          BY313744             |
|                       |           BX439797           |          BY783248             |
|                       |           CN481215           |          BY790746             |
|                       |           CN483661           |          BY145411             |
|                       |           CN483609           |          BY138139             |
|                       |           CN482526           |          BY142806             |
|                       |           CN390481           |          BY777112             |
|                       |           CN390315           |          BY146390             |
|                       |           CN390696           |          BY132130             |
|                       |           CN390342           |          BY769349             |
|                       |           CN390366           |          BB611602             |
|                       |           CN390614           |          BY140800             |
|                       |           BX417864           |          BY141923             |
|                       |           BX459180           |          BY311573             |
|                       |           CN390368           |          BY144601             |
|                       |           CN390659           |          BY789335             |
|                       |           CN481447           |          BY136180             |
|                       |           CN482242           |          BY131688             |
|                       |           CN390530           |          BY133192             |
|                       |           CN390675           |          BY135128             |
|                       |           CN390456           |          BY133810             |
|                       |           CN390458           |          BY339193             |
|                       |           CN390296           |          BY776549             |
|                       |           CN390684           |          BY142248             |
|                       |           CN390504           |          BY788094             |
|                       |           CN390648           |          BY336576             |
|                       |           CN390556           |          BY143436             |
|                       |           CN390389           |          BY310064             |
|                       |           CN390688           |          BY148535             |
|                       |           CN390321           |          BY790454             |
|                       |           CN390330           |          BY149900             |
|                       |           CN390705           |          BY140759             |
|                       |           CN390627           |          BY149195             |
|                       |           CN390441           |          BY139616             |
|                       |           BU529026           |          BY139573             |
|                       |           CN390597           |          BY149650             |
|                       |           CN390685           |          BY786883             |
|                       |           CN390656           |          BY141092             |
|                       |           CN390405           |          BY146067             |
|                       |           CN390478           |          BY151657             |
|                       |           CN484362           |          BY789418             |
|                       |           CB043921           |          BY792670             |
|                       |           AL042517           |          BY778164             |
|                       |           CN390631           |          BY780581             |
|                       |           CN483483           |          BY786756             |
|                       |           CN481295           |          BY790498             |
|                       |           CN483263           |          BY147755             |
|                       |           CB215548           |          BY147824             |
|                       |           CN482239           |          BY778223             |
|                       |           CN483384           |          BY140850             |
|                       |           BG697134           |          BY148159             |
|                       |           BC042586           |          BY147822             |
|                       |           BQ723216           |          BY151280             |
|                       |           BM546697           |          BY787329             |
|                       |           CN390628           |          BY146252             |
|                       |           CD607440           |          BY788016             |
|                       |           CN481387           |          BY141318             |
|                       |           CN482318           |          BY143648             |
|                       |           CD607438           |          BY145330             |
|                       |           CN483114           |          BY785981             |
|                       |           CN390509           |          BY790927             |
|                       |           CN390435           |          BY108761             |
|                       |           CN481276           |          BY138977             |
|                       |           CN483636           |          BY146697             |
|                       |           CN390526           |          BY109835             |
|                       |           CN390579           |          BY132824             |
|                       |           CN390623           |          BY768567             |
|                       |           CN390539           |          BY349272             |
|                       |           CN390604           |          BY768592             |
|                       |           CN390345           |          BY149530             |
|                       |           CN390294           |          BY785913             |
|                       |           CN390311           |          BY793367             |
|                       |           AI751872           |          BY133925             |
|                       |           CN390483           |          BY145659             |
|                       |           BC054498           |          BY151419             |
|                       |           BQ949088           |          BY786396             |
|                       |           CN482411           |          BY151155             |
|                       |           CN390613           |          BY309776             |
|                       |           CN390590           |          BY783099             |
|                       |           CN483403           |          BY133564             |
|                       |           CN390598           |          BY145668             |
|                       |           AI751480           |          BY140481             |
|                       |           CN390700           |          BY146165             |
|                       |           AI750663           |          BY782369             |
|                       |           CN390608           |          BY784323             |
|                       |           CN390516           |          BY789531             |
|                       |           BM708418           |          BY150638             |
|                       |           BM932203           |          BY770422             |
|                       |           BX427589           |          BY132897             |
|                       |           CN390358           |          BY150486             |
|                       |           CN390521           |          BY316121             |
|                       |           CN390448           |          BY144109             |
|                       |           CN390660           |          BY771273             |
|                       |           CN390363           |          BY134055             |
|                       |           CN390403           |          BY146934             |
|                       |           CN390498           |          BY133574             |
|                       |           CN390644           |          BY147359             |
|                       |           CN390329           |          BY308436             |
|                       |           CN390695           |          BY777533             |
|                       |           CN390694           |          BY134480             |
|                       |           CN390433           |          BY776477             |
|                       |           CN390398           |          BY133570             |
|                       |           CN390602           |          BY144815             |
|                       |           CN390578           |          BY775603             |
|                       |           CN390511           |          BY779249             |
|                       |           CN390339           |          BY135458             |
|                       |           CN390365           |          BY143334             |
|                       |           CN390670           |          BY314752             |
|                       |           CN390291           |          BY136814             |
|                       |           CN390535           |          BY136968             |
|                       |           CN390697           |          BY316110             |
|                       |           CN390611           |          BY774391             |
|                       |           CN390386           |          BY782434             |
|                       |           CN390328           |          BY775410             |
|                       |           CN390502           |          BY774414             |
|                       |           CD607478           |          BY766834             |
|                       |           CD607479           |          BY143349             |
|                       |           CN390310           |          BY137513             |
|                       |           CN390559           |          BY131926             |
|                       |           CN390534           |          BY143944             |
|                       |           CN390332           |          BY773275             |
|                       |           CN390563           |          BY143519             |
|                       |           CN390618           |          BY753460             |
|                       |           CN390347           |          BY767040             |
|                       |           CN390353           |          BY766548             |
|                       |           CN390567           |          BY135133             |
|                       |           CN390571           |          BY770166             |
|                       |           CN390638           |          BY133652             |
|                       |           CN390547           |          BY770724             |
|                       |           CN390676           |          BY767641             |
|                       |           CN390666           |          BY770230             |
|                       |           CN390689           |          BY135371             |
|                       |           CN390399           |          BY776951             |
|                       |           CN390382           |          BY133025             |
|                       |           CN390480           |          BY307914             |
|                       |           CN390450           |          BY136470             |
|                       |           CN390476           |          BY138793             |
|                       |           CN390317           |          BY776960             |
|                       |           CN390575           |          BY312153             |
|                       |           CN390505           |          BY312209             |
|                       |           CN390392           |          BY771686             |
|                       |           CN390649           |          BY137927             |
|                       |           CN390378           |          BY136201             |
|                       |           CN390549           |          BY308115             |
|                       |           CN390698           |          BY313897             |
|                       |           CN390706           |          BY773146             |
|                       |           CN390580           |          BY768329             |
|                       |             Y00724           |          BY001727             |
|                       |           CN390418           |          BY754611             |
|                       |           CN390461           |          BY713385             |
|                       |           CN390523           |          BY141627             |
|                       |           CN390346           |          BY149688             |
|                       |           CD656012           |          BY142052             |
|                       |           CN390439           |          BY141687             |
|                       |           BM721267           |          BY786060             |
|                       |           CN390307           |          BQ563102             |
|                       |           CN390503           |          BY770853             |
|                       |           CN390647           |          BY793985             |
|                       |           CN390356           |          BY149999             |
|                       |           CN390318           |          BY138239             |
|                       |           CN390525           |          BY788938             |
|                       |           CN390577           |          BY343646             |
|                       |           CN390587           |          BY139302             |
|                       |           CN390350           |          BY774416             |
|                       |           CN390413           |          BY137499             |
|                       |           CN390496           |          BY793209             |
|                       |           CN390619           |          BY330455             |
|                       |           CN390632           |          BY767661             |
|                       |           CN390487           |          BY769255             |
|                       |           CN390514           |          BY777759             |
|                       |           CN390599           |          BY308179             |
|                       |           AI750827           |          BY135227             |
|                       |           AI366082           |          BM232805             |
|                       |           BF515530           |          BY784676             |
|                       |           CN390357           |          BY784657             |
|                       |           CN390360           |          BY775209             |
|                       |           CN390479           |          BY145109             |
|                       |           CN390490           |          BY133371             |
|                       |           CN390473           |          BY342666             |
|                       |           CN390665           |          BY776559             |
|                       |           CD607494           |          BY142835             |
|                       |           CN390430           |          BY769232             |
|                       |           CN390391           |          BY133151             |
|                       |           AL041040           |          BY132097             |
|                       |           CN481871           |          BY773351             |
|                       |           CN390426           |          BY136923             |
|                       |           CN390407           |          BY142040             |
|                       |           CN390326           |          BY301470             |
|                       |           CN390550           |          BY337258             |
|                       |           CD607476           |          BY147854             |
|                       |           CD607477           |          BY138640             |
|                       |           CN390661           |          BY144817             |
|                       |           CN390562           |          BY001822             |
|                       |           AA853300           |          BY788181             |
|                       |           CN390586           |          BY330782             |
|                       |           CN390341           |          BY148541             |
|                       |           AI750885           |          BY778011             |
|                       |           CA407324           |          BY778301             |
|                       |           CN390469           |          BY145769             |
|                       |           AA853279           |          BY151582             |
|                       |           AI752314           |          BY132848             |
|                       |           AW068625           |          BY149257             |
|                       |           CN390335           |          BY784795             |
|                       |           CN390636           |          BY308487             |
|                       |           CN390414           |          BY350051             |
|                       |           CN390334           |          BY768614             |
|                       |           CN390462           |          BY310418             |
|                       |           CN390566           |          BY310498             |
|                       |           CN390486           |          BY135816             |
|                       |           CK000867           |          BY776063             |
|                       |           CN390682           |          BY776989             |
|                       |           BG875560           |          BY784764             |
|                       |           CN390610           |          BY786534             |
|                       |           BF940510           |          BY340462             |
|                       |           AL042358           |          BY785917             |
|                       |           BQ883011           |          BY353262             |
|                       |           BQ349911           |          BY310721             |
|                       |           CD607509           |          BY781409             |
|                       |           CD607498           |          BY147267             |
|                       |           CA395861           |          BY311122             |
|                       |           AI752512           |          BY312635             |
|                       |           BQ366427           |          BY344535             |
|                       |           CN390510           |          BY776196             |
|                       |           BM721182           |          BY310269             |
|                       |           AI750302           |          BY780670             |
|                       |           BU625674           |          BY772585             |
|                       |           CN390606           |          BY136809             |
|                       |           CN390673           |          BY311055             |
|                       |           CN390442           |          BY312680             |
|                       |           CN390495           |          BY315076             |
|                       |           AI752770           |          BY343701             |
|                       |           CN390558           |          BY770461             |
|                       |           CN390572           |          BY139511             |
|                       |           BG939159           |          BY320062             |
|                       |           CN390385           |          BY330602             |
|                       |           CN390375           |          BY766435             |
|                       |           CD607490           |          BY769909             |
|                       |           CD607488           |          BY769838             |
|                       |           CN390471           |          BY309415             |
|                       |           CD607504           |          BY298912             |
|                       |           CN390513           |          BY307830             |
|                       |           CD644307           |          BY308056             |
|                       |           CK000887           |          BY311689             |
|                       |           CK000996           |          BY313016             |
|                       |           BQ328574           |          BY769746             |
|                       |           CN390527           |          BY753960             |
|                       |           AI752813           |          BY753760             |
|                       |           CF995668           |          BY788109             |
|                       |           CN390536           |          BY334011             |
|                       |           CD607471           |          BY783762             |
|                       |           CD607460           |          BY791170             |
|                       |           CD607462           |          BY145053             |
|                       |           CD607464           |          BY790424             |
|                       |           CD607466           |          BY778633             |
|                       |           CD607468           |          BY346410             |
|                       |           CD607461           |          BY139926             |
|                       |           CD607463           |          BY346984             |
|                       |           CD607465           |          BY778869             |
|                       |           CD607467           |          BY141523             |
|                       |           CD607469           |          BY147069             |
|                       |           CD607473           |          BY780350             |
|                       |           CD607472           |          BY150289             |
|                       |           CD607470           |          BY786837             |
|                       |           CN390663           |          BY147102             |
|                       |           CN390704           |          BY148273             |
|                       |           CB266512           |          BY345312             |
|                       |           AI459523           |          BY141120             |
|                       |           BQ365964           |          BY336814             |
|                       |           CN481912           |          BY779117             |
|                       |           AI750704           |          BY342324             |
|                       |           CN390362           |          BY788521             |
|                       |           BQ365967           |          BY792662             |
|                       |           BF989586           |          BY142027             |
|                       |           BF999356           |          BY347429             |
|                       |           BQ365853           |          BY778958             |
|                       |           BQ365852           |          BY140365             |
|                       |           BQ366096           |          BY346128             |
|                       |           BQ365966           |          BY791304             |
|                       |           CV394983           |          BY142479             |
|                       |           BQ366352           |          BY147024             |
|                       |           BQ366939           |          BY147481             |
|                       |           BQ365965           |          BY341348             |
|                       |           BQ365854           |          BY787351             |
|                       |           CN390485           |          BY107778             |
|                       |           CD607491           |          BY341321             |
|                       |           BE879766           |          BY349884             |
|                       |           AI752075           |          BY778476             |
|                       |           CN390542           |          BY352733             |
|                       |           CD607489           |          BY110022             |
|                       |           CN390603           |          BY779981             |
|                       |           CD607458           |          BY786435             |
|                       |           CD607508           |          BY352253             |
|                       |           CN390436           |          BY791066             |
|                       |           CO246568           |          BY149804             |
|                       |           CD607496           |          BY779758             |
|                       |           CN390554           |          BY109623             |
|                       |           CN390467           |          BY111246             |
|                       |           AA853463           |          BY785686             |
|                       |           CD557321           |          BY138499             |
|                       |           BX431599           |          BY785803             |
|                       |           CD607441           |          BY788026             |
|                       |           AI750858           |          BY132796             |
|                       |           CD607499           |          BY149481             |
|                       |           CN390620           |          BY771337             |
|                       |           CF125258           |          BY337504             |
|                       |           CD607439           |          BY768562             |
|                       |           BG897006           |          BY794843             |
|                       |           CK000169           |          BY793527             |
|                       |           AI751606           |          BY335147             |
|                       |           AI752118           |          BY311158             |
|                       |           CD607444           |          BY337343             |
|                       |           CD607442           |          BY349188             |
|                       |           CD607454           |          BY144492             |
|                       |           CN390336           |          BY337232             |
|                       |           CD607450           |          BY784400             |
|                       |           CN390637           |          BY784622             |
|                       |           CO246147           |          BY134894             |
|                       |           CN390423           |          BY310808             |
|                       |           CN390297           |          BY793094             |
|                       |           CN390464           |          BY149992             |
|                       |           CN390693           |          BY775239             |
|                       |           CN390370           |          BY792458             |
|                       |           CN390589           |          BY145598             |
|                       |           CN390445           |          BY340431             |
|                       |           CD607452           |          BY777387             |
|                       |           CD607492           |          BY779339             |
|                       |           CD607486           |          BY783109             |
|                       |           CD607487           |          BY794348             |
|                       |           CD607493           |          BY150624             |
|                       |           CN390591           |          BY311621             |
|                       |           CD607448           |          BY774693             |
|                       |           CN483277           |          BY146685             |
|                       |           CD607446           |          BY308549             |
|                       |           AI751854           |          BY771261             |
|                       |           CN481678           |          BY137117             |
|                       |           CD607485           |          BY140415             |
|                       |           CD607484           |          BY788885             |
|                       |           AI751471           |          BY136292             |
|                       |           CO248549           |          BY143531             |
|                       |           AW067967           |          BY779297             |
|                       |           CN390553           |          BY147230             |
|                       |           CN390522           |          BY308509             |
|                       |           CN390643           |          BY311033             |
|                       |           AA852498           |          BY315886             |
|                       |           CN390494           |          BY339832             |
|                       |           AI751985           |          BY339865             |
|                       |           CN390367           |          BY774033             |
|                       |           CN390406           |          BY134393             |
|                       |           CF125442           |          BY310335             |
|                       |           AI751499           |          BY339103             |
|                       |           CN390650           |          BY775576             |
|                       |           CN390285           |          BY134367             |
|                       |           CD607497           |          BY339803             |
|                       |           AI752440           |          BY311473             |
|                       |           BF742531           |          BY319551             |
|                       |           BG946477           |          BY311056             |
|                       |           AA853449           |          BY311343             |
|                       |           CN390651           |          BY341257             |
|                       |           CN390379           |          BY344656             |
|                       |           CN390506           |          BY347767             |
|                       |           BQ945700           |          BY347898             |
|                       |           CN390528           |          BY770129             |
|                       |           CN485181           |          BY770591             |
|                       |           CO246404           |          BY775752             |
|                       |           CN390325           |          BY769486             |
|                       |           CF124956           |          BY134258             |
|                       |           AA853793           |          BY136801             |
|                       |           CO249805           |          BY774412             |
|                       |           CN390422           |          BY135703             |
|                       |           CA406983           |          BY308689             |
|                       |           CN390314           |          BY309042             |
|                       |           BQ350078           |          BY318755             |
|                       |           CN390460           |          BY769519             |
|                       |           AA853815           |          BY767063             |
|                       |           CN390434           |          BY769636             |
|                       |           CN390302           |          BY787834             |
|                       |           CN390452           |          BY135987             |
|                       |           AA852900           |          BY131777             |
|                       |           AA852977           |          BY131845             |
|                       |           CN390683           |          BY136087             |
|                       |           AW068526           |          BY143920             |
|                       |           CN390512           |          BY317547             |
|                       |           CN390373           |          BY769302             |
|                       |           CN390612           |          BY767237             |
|                       |           CN390390           |          BY771057             |
|                       |           CN390630           |          BY772181             |
|                       |           CD656983           |          BY768747             |
|                       |           CN390687           |          BY133896             |
|                       |           CF995815           |          BY341802             |
|                       |           CN390658           |          BY771104             |
|                       |           CD244031           |          BY316485             |
|                       |           CO249600           |          BY771640             |
|                       |           CN390652           |          BY131495             |
|                       |           CN390412           |          BY135920             |
|                       |           CN390560           |          BY772959             |
|                       |           CN390529           |          BY777808             |
|                       |           CN390449           |          BY787485             |
|                       |           BQ708926           |          BY137983             |
|                       |           CN390520           |          BY776968             |
|                       |           CN390654           |          BY133061             |
|                       |           CN390702           |          BY133188             |
|                       |           CN482293           |          BY137989             |
|                       |           CK000789           |          BY138835             |
|                       |           AW068162           |          BY307850             |
|                       |           AI751490           |          BY308951             |
|                       |           CN390492           |          BY311719             |
|                       |           CN390640           |          BY312175             |
|                       |           CN390374           |          BY313255             |
|                       |           AL047384           |          BY314707             |
|                       |           AL039674           |          BY336553             |
|                       |           CN390393           |          BY773030             |
|                       |           CO404996           |          BY773576             |
|                       |           CN390625           |          BY773893             |
|                       |           CD607483           |          BY135249             |
|                       |           CF786090           |          BY312171             |
|                       |           CD607482           |          BY773572             |
|                       |           CN390383           |          BY308005             |
|                       |           CD358838           |          BY139225             |
|                       |           CN390470           |          BY001817             |
|                       |           CO397145           |          BY001790             |
|                       |           CN390585           |          BY060221             |
|                       |           CO245970           |          BB621138             |
|                       |           CN390645           |          BY783795             |
|                       |           AA852718           |          BY149111             |
|                       |           CN390300           |          BY324313             |
|                       |           CN390352           |          BY148755             |
|                       |           CO393789           |          BY785945             |
|                       |           CN390417           |          BY312434             |
|                       |           AA852684           |          BY142799             |
|                       |           CN390354           |          BY340679             |
|                       |           CN483828           |          CN460694             |
|                       |           CN390409           |          CF734142             |
|                       |           AI752045           |          BB863035             |
|                       |           CN390544           |          BY146836             |
|                       |           CN390569           |          BY790096             |
|                       |           BQ366644           |          BY318015             |
|                       |           CO246330           |          BY792258             |
|                       |           BQ349824           |          BY110466             |
|                       |           CN390605           |          BY140325             |
|                       |           CF125446           |          BY141072             |
|                       |           CF125365           |          BY141413             |
|                       |           CF125721           |          BY141441             |
|                       |           CN390692           |          BY780404             |
|                       |           CO246716           |          BY141659             |
|                       |           CN390616           |          BY793991             |
|                       |           AI752529           |          BY780544             |
|                       |           CA394074           |          BY349266             |
|                       |           CN390565           |          BY138112             |
|                       |           CN483095           |          BY352186             |
|                       |           AW067794           |          BY138059             |
|                       |           AI906078           |          BY776652             |
|                       |           CN390662           |          BY134629             |
|                       |           CN390361           |          BY134949             |
|                       |           AA852139           |          BY309750             |
|                       |           CN390664           |          BY340252             |
|                       |           CO246920           |          BY774665             |
|                       |           BQ353276           |          BY151171             |
|                       |           AA348185           |          BY150631             |
|                       |           CN390305           |          BY784276             |
|                       |           CO249480           |          BY134495             |
|                       |           CN390679           |          BY768225             |
|                       |           CN390667           |          BY132969             |
|                       |           BI754651           |          BY133442             |
|                       |           CK000693           |          BY307889             |
|                       |           AA852289           |          BY767605             |
|                       |           AA852589           |          BY135063             |
|                       |           BQ363401           |          BY713401             |
|                       |           CN390376           |          BY713286             |
|                       |           AA852245           |          BY713271             |
|                       |           CN390340           |          BY713296             |
|                       |           CB268235           |          CN460501             |
|                       |           BQ363409           |          BY713288             |
|                       |           AA852237           |          BY133167             |
|                       |           CF995615           |          CF726737             |
|                       |           BQ004985           |          CN525196             |
|                       |           AA853339           |          CN526727             |
|                       |           BQ302233           |          CF742340             |
|                       |           CN390491           |          CN460781             |
|                       |           CN390531           |          CF728308             |
|                       |           CO247920           |          CF726735             |
|                       |           CD358295           |          CF734835             |
|                       |           CN390701           |          CN525490             |
|                       |           AI751969           |          BU702240             |
|                       |           CO246221           |          CF728373             |
|                       |           CN483908           |          CK639934             |
|                       |           CO397655           |          CF734757             |
|                       |           CO248976           |          CF735110             |
|                       |           BX479866           |          CA327959             |
|                       |           CN390465           |            W63955             |
|                       |           AW068034           |          CB723209             |
|                       |           CN390424           |          CF734588             |
|                       |           CN390419           |          CB523344             |
|                       |           CN390429           |          CF742975             |
|                       |           CB268341           |          CF906270             |
|                       |           CN390601           |          CF622833             |
|                       |           CF995616           |          AA122695             |
|                       |           CV409710           |          CB247679             |
|                       |           CN390576           |          BY307846             |
|                       |           CN390451           |          BY304501             |
|                       |           CN390545           |          BF150339             |
|                       |           AI752214           |          CF538971             |
|                       |           BX479711           |          CN526928             |
|                       |           AI751397           |          CF531651             |
|                       |           BF355946           |          BI455711             |
|                       |           CD607521           |          AA760197             |
|                       |           CN390507           |          CN459910             |
|                       |           BQ349912           |          CN459609             |
|                       |           AA340690           |          CA563081             |
|                       |           AA340666           |          AA103298             |
|                       |           CN390609           |          CN459608             |
|                       |           CN390444           |          CF728372             |
|                       |           CN390489           |          BC007158             |
|                       |           CO246852           |          BY343449             |
|                       |           AL047715           |          BY308952             |
|                       |           BF527113           |          CF537143             |
|                       |           CN390288           |          CF726548             |
|                       |           CN390669           |          CF734087             |
|                       |           CN390404           |          CN458712             |
|                       |           CF125967           |          CN460000             |
|                       |           CN390564           |          BQ917580             |
|                       |           CN390500           |          BY311834             |
|                       |           CN390583           |          BY303642             |
|                       |           CN390457           |          BI656522             |
|                       |           CN390415           |          BC042503             |
|                       |           CV417840           |          CK628441             |
|                       |           CN390349           |          CK788042             |
|                       |           AA341131           |          CB521364             |
|                       |           CO402790           |          AA117109             |
|                       |           BX489506           |          BQ713075             |
|                       |           CN484931           |          CF742827             |
|                       |           AI751184           |          BY069918             |
|                       |           CF125809           |          BU611274             |
|                       |           CN482504           |          BY792346             |
|                       |           CN390518           |          BY790456             |
|                       |           CN390348           |          BY785798             |
|                       |           CN390691           |          BY140749             |
|                       |           BQ901019           |          BY345767             |
|                       |           BQ900971           |          BY146709             |
|                       |           BQ303908           |          BY150234             |
|                       |           CN390303           |          BY773966             |
|                       |           AI752594           |          BY786089             |
|                       |           AW068026           |          BY794477             |
|                       |           BG938877           |          BY133719             |
|                       |           BG938807           |          BY310446             |
|                       |           CN390343           |          BY769936             |
|                       |           BQ949891           |          BY775961             |
|                       |           BU150626           |          BY794156             |
|                       |           BG938844           |          BY140532             |
|                       |           CN390388           |          BY150020             |
|                       |           AU117263           |          BY143981             |
|                       |           AI750530           |          BY150015             |
|                       |           AL043494           |          BY145984             |
|                       |           CO405969           |          BY789318             |
|                       |           CN280116           |          BY133312             |
|                       |           CN390428           |          BY137473             |
|                       |             V00503           |          BY772057             |
|                       |           CV361881           |          BY769124             |
|                       |           CN390298           |          BY769407             |
|                       |           BQ218746           |          BY316468             |
|                       |           CV361895           |          BY771033             |
|                       |             S96821           |          BY308242             |
|                       |           CN482785           |          BY139428             |
|                       |           BU150058           |          BY135907             |
|                       |           AI751992           |          BY138858             |
|                       |           AA348074           |          BY771572             |
|                       |           BQ328287           |          AW323930             |
|                       |           CO246157           |          BU520751             |
|                       |           BX480077           |          CF534448             |
|                       |           BQ694201           |          CB518527             |
|                       |           CO247547           |          CF736637             |
|                       |           AA331827           |          CF735173             |
|                       |           CN390387           |          BQ715475             |
|                       |           BG938840           |          CF732977             |
|                       |           CN390668           |          CN458688             |
|                       |           CF125870           |          CB522352             |
|                       |           CF126039           |          CF734407             |
|                       |           CN390515           |          CN460846             |
|                       |           CN484107           |          CF727330             |
|                       |           BX480130           |          BY349917             |
|                       |           CN390453           |          BY312716             |
|                       |           AW068308           |          BY309202             |
|                       |           BM721191           |          BY133748             |
|                       |           CN390454           |          BQ770953             |
|                       |           BM721072           |          BY308241             |
|                       |           BQ312583           |          CN693927             |
|                       |           BQ312588           |          BM503081             |
|                       |           CO248655           |          CB723077             |
|                       |             L47668           |          CB722973             |
|                       |           AI751600           |          CF726691             |
|                       |           BM722647           |          CN669637             |
|                       |           AL047370           |          CF734522             |
|                       |           CV373572           |          CF727658             |
|                       |           CN390482           |          CN460402             |
|                       |           CN481242           |          CN460976             |
|                       |           CB963005           |          CF537019             |
|                       |           AA970206           |          BE308706             |
|                       |           CN390421           |          CB520811             |
|                       |           CN482701           |          CB521681             |
|                       |           CB215874           |          CF742973             |
|                       |           CN390570           |          CN526086             |
|                       |           BQ877113           |          CF742323             |
|                       |           BQ930125           |          CB526065             |
|                       |           CN390519           |          CB518816             |
|                       |           AI752647           |          CN461013             |
|                       |           BP365037           |          CB058062             |
|                       |           AW068105           |          BQ180488             |
|                       |           AA347392           |          CF734071             |
|                       |           AW068663           |          CN526501             |
|                       |           BX479768           |          CF726898             |
|                       |           CO406156           |            X58251             |
|                       |           CN390543           |          CB248977             |
|                       |           AI752313           |          BY147284             |
|                       |           BQ328444           |          CK331127             |
|                       |           CD607507           |          BY307954             |
|                       |           CN390488           |          AI427842             |
|                       |           BQ334461           |          CD349762             |
|                       |           CN390287           |          BY310424             |
|                       |           CN390532           |          BY768206             |
|                       |           CN280133           |          BY316497             |
|                       |           AL043259           |          CB522444             |
|                       |           CV371026           |          BY108308             |
|                       |           BE880147           |          CN527243             |
|                       |           BM721999           |          AW411724             |
|                       |           AA954943           |          CF532045             |
|                       |           CD607500           |          CF726379             |
|                       |           BE886039           |          CB722867             |
|                       |           AA340686           |          BQ921848             |
|                       |           CD607502           |          CA568278             |
|                       |           BQ893369           |          CB527325             |
|                       |             T59123           |          BY311777             |
|                       |           AW068442           |          CN458723             |
|                       |           CO249541           |          BY139800             |
|                       |           AA348072           |          BI652651             |
|                       |           BM720653           |          BU611890             |
|                       |           BG877247           |          BY312819             |
|                       |           CN390377           |          CA326808             |
|                       |           CN280142           |          BF100234             |
|                       |           CD607503           |          BY151156             |
|                       |           BQ718532           |          BY148296             |
|                       |           AW068343           |          BY780456             |
|                       |           CN390401           |          BY793708             |
|                       |           CN280131           |          BY145514             |
|                       |           CD607522           |          BY307911             |
|                       |           CN280145           |          BF181193             |
|                       |           CB265781           |          CN526832             |
|                       |           CB268601           |          BG172617             |
|                       |           CF132161           |          BY309965             |
|                       |           CN482474           |          BF100131             |
|                       |           AA853792           |          BY312479             |
|                       |           CN280128           |          CB246941             |
|                       |           CN280121           |          CF741630             |
|                       |           CN280135           |          AV145195             |
|                       |           CB269181           |          BY136467             |
|                       |           CN280144           |          BY713406             |
|                       |           CN481791           |          BU510959             |
|                       |           AA334545           |          BY778009             |
|                       |           CV379499           |          BY357049             |
|                       |           BG938549           |          BY358292             |
|                       |           BQ723631           |          BY310830             |
|                       |           CN280143           |          BQ715016             |
|                       |           CN280118           |          BY085073             |
|                       |           CN484471           |          BB859546             |
|                       |           CN280117           |          BY768124             |
|                       |           AI906037           |          CK622596             |
|                       |           BG938550           |          CN461084             |
|                       |           BG954444           |          BQ714094             |
|                       |           CV322925           |          BQ770404             |
|                       |           CN483579           |          BY310591             |
|                       |           CN481555           |          BM963018             |
|                       |           CN280119           |          BQ442073             |
|                       |           CN483356           |          CN458923             |
|                       |           CN280139           |          CN695778             |
|                       |           AU120016           |          BY309276             |
|                       |           CN484089           |          BF582795             |
|                       |           CV325234           |          CB520735             |
|                       |           BI038807           |          CF727480             |
|                       |           CD607506           |          BF178199             |
|                       |           CD358647           |          CN527339             |
|                       |           CV424206           |          CF729253             |
|                       |           CN482211           |          BI696465             |
|                       |           CN481861           |          CF734158             |
|                       |           CN280140           |          BY311715             |
|                       |           AA852102           |          BY308255             |
|                       |             C03386           |          BY307989             |
|                       |           CN280132           |          BF117659             |
|                       |           BU147517           |          BY354909             |
|                       |           AL038163           |          AV140023             |
|                       |           AI751245           |          BI662690             |
|                       |           BQ366428           |          CN696314             |
|                       |           AA179691           |          CK333809             |
|                       |           BU150240           |          BQ563103             |
|                       |           AA599205           |          BY786719             |
|                       |           BQ349463           |          CN664482             |
|                       |           BQ349544           |          CA327625             |
|                       |           BE877233           |          CN665367             |
|                       |           AA852101           |          CN670740             |
|                       |           CN280124           |          BE288138             |
|                       |           AW241362           |          AW230145             |
|                       |           CN280115           |          CN691134             |
|                       |           BM723777           |          BE307888             |
|                       |           CN280120           |          BE377917             |
|                       |           BQ722216           |          CN526674             |
|                       |           CV325222           |          CN460531             |
|                       |           BG675890           |          CF741964             |
|                       |             F05547           |          BF119719             |
|                       |           CD517399           |          BE534082             |
|                       |           CN280134           |          BF179002             |
|                       |           CD607501           |          CA564633             |
|                       |           CN280113           |          AW215308             |
|                       |           BQ694530           |          BF179735             |
|                       |           CO247417           |          BI690439             |
|                       |           BQ328626           |          BY789896             |
|                       |           BQ349221           |          BQ928331             |
|                       |           AA367432           |          CN691333             |
|                       |           CN280122           |          CF735360             |
|                       |           AW630478           |          BY139357             |
|                       |           BQ876830           |          CN696976             |
|                       |           AW068279           |          AW323444             |
|                       |           CN280136           |          CF727835             |
|                       |           AL045656           |          AI425223             |
|                       |           AW630577           |          AA756823             |
|                       |           BQ347628           |          BG321795             |
|                       |           AW069835           |          BY730182             |
|                       |           AI366501           |          BM454167             |
|                       |           AI754078           |          BQ265245             |
|                       |           CN280129           |          BE380410             |
|                       |           AA386035           |          CF742422             |
|                       |           BG178711           |          BE285792             |
|                       |           AL042099           |          BG242137             |
|                       |           AI751628           |          CN459713             |
|                       |           CV373673           |          AA760030             |
|                       |           CN482361           |          BM963502             |
|                       |           AA339001           |          BI645562             |
|                       |           BQ899323           |          CK624528             |
|                       |           CN280141           |          BY312453             |
|                       |           CN280127           |          BG975217             |
|                       |           AI751204           |          CN456811             |
|                       |           AA331673           |          AA967366             |
|                       |           AA375677           |          AA080007             |
|                       |           CN280112           |          CN531864             |
|                       |           CN280114           |          BF582537             |
|                       |           CN280130           |          BQ571664             |
|                       |           AW068774           |          BY074567             |
|                       |           BQ889122           |          BY067498             |
|                       |           CN482814           |          BY075247             |
|                       |           CN280137           |          BB609955             |
|                       |           AA324702           |          AK075707             |
|                       |           CN280126           |          AW229244             |
|                       |           BQ328292           |          AA073705             |
|                       |           AW238944           |          BY355671             |
|                       |           CB215847           |          CF904060             |
|                       |           BG877008           |          CN527210             |
|                       |           AL046706           |          CF904684             |
|                       |           AA347581           |          CA565545             |
|                       |           BQ354652           |          CN668563             |
|                       |           CN390319           |          CF905929             |
|                       |           AA853048           |          CF903796             |
|                       |           CN390596           |          CF905299             |
|                       |           CN390595           |          CF905084             |
|                       |           BQ328354           |          CF906208             |
|                       |           BQ717231           |          CA563260             |
|                       |           CN280138           |          CN694484             |
|                       |           BX416975           |          CN691941             |
|                       |           CD358280           |          CF906575             |
|                       |           CV316128           |          CK342295             |
|                       |           CD557554           |          CA564421             |
|                       |           CN280125           |          CA563525             |
|                       |           BM721146           |          CA566829             |
|                       |           CN390443           |          CA562509             |
|                       |           CD358372           |          CA562829             |
|                       |           BM722645           |          CA564296             |
|                       |           BF059275           |          CA564783             |
|                       |           AA853894           |          CA566279             |
|                       |           BQ328618           |          CD553976             |
|                       |           BQ328539           |          CA565353             |
|                       |           CN390372           |          CA563937             |
|                       |           BQ311379           |          CA568901             |
|                       |           BQ311380           |          CA569312             |
|                       |           BQ328348           |          CA563569             |
|                       |           BQ315169           |          CN700939             |
|                       |           BG877828           |          CA569058             |
|                       |           BM722065           |          CA568070             |
|                       |           BG939197           |          CA563076             |
|                       |           CV373679           |          CA564202             |
|                       |           BI752003           |          CA567833             |
|                       |           CN390432           |          CK341745             |
|                       |           AA331340           |          CA562576             |
|                       |           BU077147           |          CA564180             |
|                       |           BG938809           |          CA564644             |
|                       |           BG938841           |          CA566594             |
|                       |           CV314475           |          CA568003             |
|                       |             X55525           |          CA562375             |
|                       |           AW631245           |          CA562800             |
|                       |           CN280123           |          CA562839             |
|                       |           BM722324           |          CA564437             |
|                       |             J00114           |          CA564870             |
|                       |           BQ349110           |          CF906030             |
|                       |           AA295602           |          CA564667             |
|                       |           AA375177           |          CA562949             |
|                       |           CD558681           |          CA563014             |
|                       |           BQ349464           |          CA569878             |
|                       |           BQ349545           |          CA563876             |
|                       |           CD515508           |          CA564543             |
|                       |           BM721215           |          CA562672             |
|                       |           BQ328382           |          CA564476             |
|                       |           AA346925           |          CA565287             |
|                       |           CN390703           |          CA563457             |
|                       |           BQ722388           |          CA564356             |
|                       |           BQ924305           |          CA565737             |
|                       |           BM723740           |          CN669809             |
|                       |           BM512261           |          CA564833             |
|                       |           AA545750           |          BQ561241             |
|                       |           BM686254           |          CD554917             |
|                       |           AA346689           |          CN700043             |
|                       |             N30461           |          CA563617             |
|                       |           BM697582           |          CA565559             |
|                       |           BQ354376           |          CA565567             |
|                       |           CV318825           |          CA564470             |
|                       |           CD607510           |          CA562699             |
|                       |           AL043214           |          CN666013             |
|                       |           BQ347463           |          CA565304             |
|                       |           CD607511           |          CA562908             |
|                       |           BQ328248           |          CA564899             |
|                       |           BG938808           |          CA564940             |
|                       |           BQ328249           |          CA566000             |
|                       |           CN484247           |          CA563740             |
|                       |           CD245867           |          CD552139             |
|                       |           CN390410           |          CA566122             |
|                       |           BG938878           |          CA565509             |
|                       |           CD245127           |          CA566416             |
|                       |           BF339024           |          CA567009             |
|                       |           CN390617           |          CN670756             |
|                       |           CN390327           |          CA562508             |
|                       |           CB215784           |          CA563091             |
|                       |           AW577408           |          CA565286             |
|                       |           BG938635           |          CN665817             |
|                       |           BQ302637           |          CN667337             |
|                       |           BQ363172           |          CN670765             |
|                       |           AW582358           |          CN666442             |
|                       |           BQ302282           |          CN670227             |
|                       |           AA377504           |          CA563498             |
|                       |           AI751991           |          CN667753             |
|                       |           AA377993           |          CA567569             |
|                       |           CD245032           |          CA564560             |
|                       |           BQ302419           |          CN666181             |
|                       |           CN390594           |          CN667271             |
|                       |           BQ354381           |          CN690895             |
|                       |           CV318830           |          CN691368             |
|                       |             D58586           |          CN668859             |
|                       |           CN390548           |          CN670539             |
|                       |           CN483411           |          CN667443             |
|                       |           BQ368466           |          CA565958             |
|                       |           BQ328307           |          CN667524             |
|                       |           BQ128005           |          CA562701             |
|                       |           BM507209           |          CA564874             |
|                       |           BQ314636           |          CA565043             |
|                       |           CB159621           |          CA565266             |
|                       |           AA365687           |          CA565321             |
|                       |           AA581781           |          CA565721             |
|                       |           AA581764           |          CA565915             |
|                       |           CV359419           |          CD550950             |
|                       |           AA378973           |          CF903133             |
|                       |           BM698384           |          CA567150             |
|                       |           AA331429           |          CA565471             |
|                       |             T51666           |          CA563229             |
|                       |           BQ328353           |          CA565174             |
|                       |             T70424           |          CA565210             |
|                       |           BQ364692           |          CA565230             |
|                       |           BQ362933           |          CA567604             |
|                       |           AA347541           |          CD551138             |
|                       |           BI038125           |          CA563323             |
|                       |           CV322899           |          CA564757             |
|                       |           CV322923           |          CA565051             |
|                       |           BG876222           |          CA565075             |
|                       |           BG876225           |          CA565984             |
|                       |           AI905291           |          CA568846             |
|                       |           CV425183           |          CD540432             |
|                       |           BI038806           |          CN670321             |
|                       |           BM313102           |          CN670331             |
|                       |           CV322919           |          CA565196             |
|                       |           BI038119           |          CN700263             |
|                       |           CV322909           |          CA564008             |
|                       |             N89323           |          CA565991             |
|                       |           BQ292172           |          CF906811             |
|                       |           BQ366258           |          CA565347             |
|                       |           BQ371519           |          CN669501             |
|                       |           AW069066           |          CN699462             |
|                       |           BM847062           |          CA563183             |
|                       |           BG876485           |          CA569600             |
|                       |             C02602           |          CN665773             |
|                       |           BI038122           |          CN704099             |
|                       |           BQ315128           |          CF905066             |
|                       |           CN390438           |          CN665326             |
|                       |           CN390499           |          CN698530             |
|                       |           CN390646           |          CN705252             |
|                       |           CN390351           |          CF903686             |
|                       |           CN390455           |          CF903798             |
|                       |           BQ326150           |          CF903840             |
|                       |           BQ326153           |          CF903958             |
|                       |           BQ311606           |          CF904224             |
|                       |           BQ371521           |          CF905555             |
|                       |           CN390517           |          CF906539             |
|                       |           CV322914           |          CF906570             |
|                       |           BQ316393           |          CF906688             |
|                       |           BQ952311           |          CN663463             |
|                       |           AA545775           |          CN666862             |
|                       |           AA343308           |          CN666933             |
|                       |           BQ374552           |          CN667760             |
|                       |           AA347737           |          CN695990             |
|                       |           AA359707           |          CN699058             |
|                       |           CV322917           |          CN699302             |
|                       |           CN390653           |          CF169682             |
|                       |           BQ346241           |          CF906547             |
|                       |           BF733723           |          CF906550             |
|                       |           AI752353           |          CF906842             |
|                       |           AA367058           |          CN668454             |
|                       |           CN390396           |          CF906463             |
|                       |           AA361109           |          CF907381             |
|                       |           AA341111           |          CF905015             |
|                       |           AA375995           |          CN668342             |
|                       |           CN390681           |          CN704276             |
|                       |           AA366354           |          CF907363             |
|                       |           AA373098           |          CN702722             |
|                       |           AA446505           |          CN703413             |
|                       |           AA376501           |          CF906156             |
|                       |           BM721970           |          CN704700             |
|                       |           AA347616           |          CN701060             |
|                       |           BI000306           |          CF908962             |
|                       |           AA375322           |          CF903192             |
|                       |           AW838242           |          CF903672             |
|                       |           AW748220           |          CF904005             |
|                       |           BM724476           |          CF904857             |
|                       |           BF229196           |          CF910383             |
|                       |           AW860525           |          CF904065             |
|                       |           AW841503           |          CF904452             |
|                       |           BG676041           |          CF904947             |
|                       |           BF229201           |          CF905511             |
|                       |           AA347375           |          CF906081             |
|                       |           BF871454           |          CF906114             |
|                       |           AI750674           |          CF906300             |
|                       |           BF229224           |          CN663472             |
|                       |           BF326171           |          CN664409             |
|                       |           BF088485           |          CN666394             |
|                       |           BF081497           |          CN705271             |
|                       |           CN390497           |          CF904332             |
|                       |           AA599855           |          CF905486             |
|                       |           AW860523           |          CN698684             |
|                       |           BG939071           |          CF903944             |
|                       |           AA343307           |          CN698058             |
|                       |           BU787919           |          CF906127             |
|                       |           BM722005           |          CA568285             |
|                       |           AW580717           |          CF906646             |
|                       |           AW875259           |          CK334944             |
|                       |           CD514195           |          CN666060             |
|                       |           CD556720           |          CN667066             |
|                       |           CN390397           |          CF905119             |
|                       |           AW368677           |          CF906057             |
|                       |           BG938659           |          CF903730             |
|                       |           AA369703           |          CN669272             |
|                       |           AU118150           |          CF904551             |
|                       |           CN390641           |          CF906283             |
|                       |           AA788711           |          CF909722             |
|                       |           BP231314           |          CF904735             |
|                       |           AA295556           |          CF905150             |
|                       |           CD607513           |          CF905240             |
|                       |           CD607516           |          CF905248             |
|                       |           CD607519           |          CF906279             |
|                       |           AA985513           |          CF906332             |
|                       |           AA789043           |          CF906359             |
|                       |           AA317813           |          CF903860             |
|                       |           BQ300797           |          CN666286             |
|                       |           BM722716           |          CN698789             |
|                       |           AA601917           |          CA565628             |
|                       |           CN390384           |          CN702206             |
|                       |           BE825969           |          CN702238             |
|                       |           AA331822           |          BG244026             |
|                       |           BF935479           |          BG061335             |
|                       |           AA503319           |          BG061659             |
|                       |           BG988473           |          CF904159             |
|                       |           AA330659           |          CF905748             |
|                       |           AA330542           |          CN664048             |
|                       |           AL040210           |          CF904147             |
|                       |           BQ320663           |          CF903290             |
|                       |           AW580719           |          CK341785             |
|                       |           CN390459           |          CA564513             |
|                       |           AU118362           |          CA563033             |
|                       |           BE844006           |          CN668216             |
|                       |           BP207943           |          CF903239             |
|                       |           BP232001           |          CF905733             |
|                       |           BQ300052           |          CF906483             |
|                       |           BP232024           |          CF910198             |
|                       |           AA341640           |          CF910248             |
|                       |           BE839233           |          CF906455             |
|                       |           AW608072           |          CF910417             |
|                       |           AI752297           |          CF906429             |
|                       |           AA334816           |          CF910048             |
|                       |           BU073902           |          CF906440             |
|                       |             R36360           |          CF908827             |
|                       |           AA296655           |          BY310790             |
|                       |             W03033           |          CF908423             |
|                       |           BM698132           |          CN696080             |
|                       |             H66499           |          CF904783             |
|                       |           BM697604           |          BI656489             |
|                       |           AW022698           |          CF904766             |
|                       |           AI800093           |          CF903226             |
|                       |           AA334082           |          BF584466             |
|                       |           CF995776           |          BY779591             |
|                       |           AA330752           |          CF905877             |
|                       |           AI198930           |          CF905804             |
|                       |           BE839043           |          CF904081             |
|                       |           BE881455           |          CA565113             |
|                       |           CN481291           |          CA565152             |
|                       |           AA377791           |          AW258376             |
|                       |           BM724551           |          CA563193             |
|                       |           BE938396           |          AW821941             |
|                       |           CN390574           |          CF735605             |
|                       |           CN390380           |          CF906985             |
|                       |           AA334820           |          CA565680             |
|                       |           BE835530           |          CN699250             |
|                       |           AA330421           |          CF733126             |
|                       |           AI567880           |          CF738664             |
|                       |           CN390557           |          BI111462             |
|                       |           AW959329           |          CN527350             |
|                       |           AW084906           |          CF910249             |
|                       |           AU120047           |          CF905950             |
|                       |           CA395887           |          BE376292             |
|                       |           AA386142           |          CN705334             |
|                       |           AA317773           |          CA563413             |
|                       |           AW839512           |          CF727812             |
|                       |           AW382235           |          CF727743             |
|                       |           AW382471           |          CA568241             |
|                       |           BF677585           |          CN664548             |
|                       |           AU117772           |          CF904697             |
|                       |           BU075422           |          CN705144             |
|                       |           AW382223           |          CA565415             |
|                       |           AA456211           |          BF100870             |
|                       |           CN484779           |          BU516004             |
|                       |           BG991301           |          CF904642             |
|                       |           BF357921           |          CN700879             |
|                       |           CN390615           |          CK342451             |
|                       |           BM697689           |          AA396953             |
|                       |           BM722109           |          AA396954             |
|                       |           AI954910           |          CF905835             |
|                       |           BF737899           |          BB569100             |
|                       |           BE711064           |          BI113489             |
|                       |           AA137088           |          CF905190             |
|                       |           AI986217           |          CA562515             |
|                       |             W16644           |          CA562340             |
|                       |             W16646           |          CF907913             |
|                       |           AA359508           |          CN703366             |
|                       |           AI284667           |          CA565848             |
|                       |           CN390672           |          CF904088             |
|                       |           BU147519           |          BF100860             |
|                       |           AA339978           |          BQ569971             |
|                       |           AA339871           |          CF904873             |
|                       |           BM310109           |          CF904108             |
|                       |           CK905296           |          CA569073             |
|                       |           BU782066           |          BF180370             |
|                       |           AI679313           |          BI250911             |
|                       |           AI922760           |          BF151616             |
|                       |           BE815948           |          BQ219970             |
|                       |             W16772           |          CF905661             |
|                       |           BQ008068           |          CK388208             |
|                       |           BF351028           |          CF903790             |
|                       |           AI685128           |          BY774239             |
|                       |           AA983202           |          BG917535             |
|                       |           AA070710           |          AW647467             |
|                       |           BF081721           |          CN461831             |
|                       |           AA340139           |          BI454298             |
|                       |           AI357874           |          CA567167             |
|                       |           BM679603           |          CF738745             |
|                       |           AI814147           |          BQ714867             |
|                       |           BF666013           |          BQ715341             |
|                       |           BM722043           |          CA563157             |
|                       |           CD000234           |          BI112012             |
|                       |           BM721977           |          BG965428             |
|                       |           AW337944           |          CF904151             |
|                       |             R91972           |          BE374937             |
|                       |           BG028880           |          BI077558             |
|                       |             N89200           |          BE289863             |
|                       |           CN390289           |          CA564816             |
|                       |           AW078822           |          CA565451             |
|                       |           CN390381           |          BF152219             |
|                       |           BM679967           |          CF906576             |
|                       |           BM997477           |          CB590402             |
|                       |           BQ183729           |          BY308878             |
|                       |           BQ448250           |          CN529897             |
|                       |           BQ575922           |          BU522317             |
|                       |           BQ576235           |          CN693698             |
|                       |           CA427114           |          CA567749             |
|                       |           CA447248           |          CN699646             |
|                       |           CN479749           |          CF904266             |
|                       |           BM679661           |          BI077826             |
|                       |           BQ446702           |          CA563493             |
|                       |           BQ447091           |          CF749892             |
|                       |           CA415346           |          CN526638             |
|                       |           BM679705           |          CF908309             |
|                       |           BU628327           |          CF907106             |
|                       |           AA346530           |          BE308020             |
|                       |           AA346709           |          CF907455             |
|                       |           AA347934           |          BF582090             |
|                       |           BQ006800           |          BB839901             |
|                       |           BQ573783           |          CA567931             |
|                       |           BQ574870           |          CA563585             |
|                       |           CA414419           |          BE911005             |
|                       |           CA445321           |          CF729368             |
|                       |           CA448517           |          BG963024             |
|                       |           AL047787           |          CF742698             |
|                       |           BQ000235           |          BG962948             |
|                       |           BQ950031           |          CN696125             |
|                       |           AA169743           |          AA168196             |
|                       |           AW167791           |          CN693215             |
|                       |           BG619078           |          BF181150             |
|                       |           BM990278           |          CF907535             |
|                       |           BM997532           |          CF910100             |
|                       |           AA334500           |          BI455921             |
|                       |           BM721110           |          CF733453             |
|                       |           BM697843           |          BI249230             |
|                       |             W23947           |          BE307401             |
|                       |           AJ707146           |          BF152154             |
|                       |           AA376192           |          AA109059             |
|                       |           BM722133           |          CB246586             |
|                       |           AW304259           |          BF582644             |
|                       |           BM992045           |          CF906284             |
|                       |           AW367442           |          CA562711             |
|                       |           BM697718           |          AW323311             |
|                       |           AI445851           |          CF742311             |
|                       |           BM698510           |          BI113379             |
|                       |           BF878005           |          CF905577             |
|                       |           AU117726           |          CF905170             |
|                       |           AW385827           |          CF904970             |
|                       |           AW792947           |          BE287370             |
|                       |           AW792948           |          BF122498             |
|                       |           BU623983           |          CF906125             |
|                       |           BF378301           |          BQ715461             |
|                       |           AI678028           |          BQ442585             |
|                       |           BX445650           |          CF725246             |
|                       |           AU116820           |          BE307930             |
|                       |           BM720125           |          CA565576             |
|                       |           AW839421           |          BE554295             |
|                       |           BF378299           |          CA565902             |
|                       |           AW471452           |          CN693093             |
|                       |           CB143666           |          CA565989             |
|                       |           AA083901           |          CF743866             |
|                       |           BE349936           |          CB587821             |
|                       |           BQ299803           |          CA565108             |
|                       |           BQ019403           |          BI660443             |
|                       |           AA346600           |          BQ950461             |
|                       |           CB998373           |          CF906313             |
|                       |           AW068664           |          CA565787             |
|                       |           BI005401           |          CA563904             |
|                       |           CN390292           |          CA563923             |
|                       |           AI692257           |          BF119591             |
|                       |           BF844251           |          CF903788             |
|                       |           AA666129           |          BF116648             |
|                       |           AW276533           |          AA624907             |
|                       |           BG938876           |          BB840490             |
|                       |           AI589314           |          CF906422             |
|                       |             N58636           |          CF171437             |
|                       |           BM721610           |          BF123158             |
|                       |           AI750840           |          CK624629             |
|                       |           BF475405           |          CF729398             |
|                       |           AI636349           |          BE308405             |
|                       |           BQ005376           |          BY312327             |
|                       |           AW338943           |          BY308285             |
|                       |           AI446284           |          CF903223             |
|                       |           AW778910           |          BQ572916             |
|                       |           BM312815           |          BF181561             |
|                       |           AW378443           |          CF170991             |
|                       |           AI750217           |          AA561969             |
|                       |           CN390524           |          CF904631             |
|                       |           AW068527           |          CF904486             |
|                       |           AA578201           |          CF906759             |
|                       |           BG572294           |          CN698154             |
|                       |           CN390324           |          CN698147             |
|                       |           AA386187           |          CN703107             |
|                       |           AW839337           |          CF735649             |
|                       |           AI805473           |          CA565298             |
|                       |           BF736869           |          CA565953             |
|                       |           BM721722           |          BI697760             |
|                       |           AW190417           |          CA569917             |
|                       |           BM685598           |          BF179740             |
|                       |           BG573357           |          CA569016             |
|                       |           BP373187           |          BI657212             |
|                       |           CN390541           |          AA763289             |
|                       |           BF994659           |          CD807470             |
|                       |           AW262681           |          CF905963             |
|                       |           BM663064           |          CF904045             |
|                       |           AW608425           |          CN666691             |
|                       |           BF665732           |          BY336696             |
|                       |           AW470135           |          BE307916             |
|                       |           BQ010365           |          BE917545             |
|                       |           BQ366129           |          BF581955             |
|                       |           BP231234           |          CF904925             |
|                       |           BI038510           |          BQ567746             |
|                       |           BP233178           |          CF171215             |
|                       |           AA295053           |          CF907518             |
|                       |           BQ183969           |          BE912599             |
|                       |           BU854033           |          BI455317             |
|                       |           BF734613           |          BF581151             |
|                       |           AA343289           |          CD553268             |
|                       |           BF759072           |          CF168783             |
|                       |           AW578615           |          CF903831             |
|                       |           AW578600           |          CA569804             |
|                       |           AW069274           |          BI251832             |
|                       |           CN390299           |          BC012438             |
|                       |           AW023022           |          BG518865             |
|                       |             R94369           |          BE287204             |
|                       |           AW023611           |          BG245433             |
|                       |           AW068626           |          BI853720             |
|                       |           BP233262           |          BI854056             |
|                       |           CN390690           |          BI696973             |
|                       |           AA599810           |          BI694658             |
|                       |           AW513214           |          CF903122             |
|                       |           BM720624           |          CF905140             |
|                       |           CN390420           |          CB247004             |
|                       |           AA454538           |          BI082265             |
|                       |           BF088292           |          BG861937             |
|                       |           AW663239           |          BF121889             |
|                       |           BM724264           |          BI251621             |
|                       |           BP233190           |          BQ939482             |
|                       |           BI493976           |          CN702498             |
|                       |           AW022832           |          BE913853             |
|                       |           BI493975           |          CF736223             |
|                       |             T60754           |          CF531681             |
|                       |           AA374260           |          CF910456             |
|                       |           AA366137           |          AW412178             |
|                       |           AA366229           |          BG172135             |
|                       |           AI921434           |          BF181951             |
|                       |           BM722019           |          CF906408             |
|                       |           AA331002           |          CN665172             |
|                       |           AA600098           |          CN694192             |
|                       |           AA332245           |          CF903421             |
|                       |           AW662646           |          CF905992             |
|                       |           CD697957           |          CF738892             |
|                       |           BM697508           |          CB173614             |
|                       |           AW608364           |          BQ827799             |
|                       |           AW168316           |          BQ715604             |
|                       |           CA448755           |          BI108998             |
|                       |           AW385972           |          BF178030             |
|                       |             W05512           |          CA977109             |
|                       |           BM698021           |          BF102356             |
|                       |           AI857694           |          BY147975             |
|                       |           AW385949           |          BY771442             |
|                       |           AW385971           |          BY794857             |
|                       |           AW608374           |          BY137032             |
|                       |           AW608390           |          BY773433             |
|                       |           AW608435           |          BI456883             |
|                       |           AW069037           |          BG175626             |
|                       |           BM724327           |          BQ569166             |
|                       |           AI824490           |          BY315471             |
|                       |           AW069727           |          BY318672             |
|                       |           AA167493           |          BY753766             |
|                       |           AI750664           |          BY753301             |
|                       |           CD299894           |          AA563158             |
|                       |           AA827344           |          BG244039             |
|                       |           AA836845           |          CF723610             |
|                       |           AW385981           |          BQ922433             |
|                       |           AA296975           |          AA000924             |
|                       |           BF358025           |          CA565854             |
|                       |           AW608373           |          BE916587             |
|                       |           AW608378           |          CF168647             |
|                       |           AW608382           |          BF101307             |
|                       |           AW608395           |          CN460486             |
|                       |           AW608436           |          CB173922             |
|                       |           AW385973           |          CA565749             |
|                       |           AW608347           |          CF906253             |
|                       |           AW608375           |          CK622554             |
|                       |           AA663444           |          BF139678             |
|                       |             H73868           |          BI454784             |
|                       |           AW021817           |          CB589771             |
|                       |             T85405           |          CA978528             |
|                       |           AA366496           |          CF737607             |
|                       |           AI814183           |          CN694034             |
|                       |           BQ574659           |            W90967             |
|                       |           AW027670           |          CD545017             |
|                       |             H22295           |          CB842839             |
|                       |           AA298493           |          AW229150             |
|                       |           AA112896           |          BE286443             |
|                       |           BQ181291           |          CN526986             |
|                       |             H89269           |          CF737424             |
|                       |           AA340355           |          BE652727             |
|                       |           AW838650           |          AW742176             |
|                       |             R07418           |          AW647312             |
|                       |           BP231423           |          CB844400             |
|                       |           AI751185           |          BI692282             |
|                       |           AI752600           |          AW701647             |
|                       |           AI754833           |          BQ830537             |
|                       |           AI446484           |          BG173239             |
|                       |           CA426769           |          BI411475             |
|                       |             H27955           |          CF735971             |
|                       |           BQ180907           |          AA855594             |
|                       |           BM698164           |          CK792544             |
|                       |           BU929137           |          BI113601             |
|                       |           AW069840           |          CB055563             |
|                       |             H91600           |          BQ953751             |
|                       |           BQ026918           |          BI415868             |
|                       |           AI818084           |          AA500003             |
|                       |           AI751855           |          BI413550             |
|                       |           AI125410           |          CF742921             |
|                       |           AA295199           |          CN461411             |
|                       |           AW020819           |          CD773000             |
|                       |           AW020828           |          CF727753             |
|                       |           BQ004853           |          CF747403             |
|                       |           AW384935           |          BF150496             |
|                       |           BE160827           |          CA565696             |
|                       |           AW020927           |          CB842118             |
|                       |           AW023936           |          BQ566874             |
|                       |           CA436359           |          AW744346             |
|                       |             H58853           |          BQ714701             |
|                       |           AI561235           |          AA165892             |
|                       |           CA411911           |          AA544035             |
|                       |           BF436538           |          CB575731             |
|                       |           AW608118           |          CB587422             |
|                       |           AW608163           |          CA566091             |
|                       |           AW067869           |          BI452490             |
|                       |           AA167494           |          CB204575             |
|                       |           AI752215           |          AA615782             |
|                       |           AA774026           |          BB563421             |
|                       |           AI815217           |          BI412522             |
|                       |           AW582702           |          AA015224             |
|                       |           BQ181974           |          BI904495             |
|                       |           AW608391           |          BE381693             |
|                       |             N75863           |          BI249151             |
|                       |           AW023053           |          CF729341             |
|                       |             D57764           |          CF729381             |
|                       |           BF698894           |          BM569756             |
|                       |           AL047716           |          CF903821             |
|                       |             T19005           |          CB317715             |
|                       |           AW020228           |          AA673286             |
|                       |             R83067           |          BI109193             |
|                       |           AI671020           |          AA245589             |
|                       |           BE885848           |          AA097340             |
|                       |           BI497135           |          AA168260             |
|                       |           BE927568           |          BI250873             |
|                       |             R09884           |          BI694524             |
|                       |           BE693668           |          CA567124             |
|                       |           BM720214           |          BF179595             |
|                       |           AA328846           |          BI647854             |
|                       |             W05459           |            W30231             |
|                       |           AA169251           |          AA016538             |
|                       |           CA428854           |          BQ266135             |
|                       |           BF197758           |          BQ713372             |
|                       |           AW169749           |          AA222809             |
|                       |           AW081651           |          BQ924461             |
|                       |           CN390477           |          BG695240             |
|                       |           BU627724           |          AW742200             |
|                       |           BG620819           |          BI412901             |
|                       |           CN390674           |          BI453267             |
|                       |           AA361771           |          BE290262             |
|                       |           CN390635           |          BG966559             |
|                       |           CA448829           |          AA607751             |
|                       |           AI185128           |          AA276342             |
|                       |           BI493181           |          BQ887787             |
|                       |           CN390584           |          BU524321             |
|                       |           AI127091           |          BY357234             |
|                       |           BQ009722           |          AA034734             |
|                       |           BU627127           |          AA794538             |
|                       |           BQ182136           |          BE285355             |
|                       |           AI569297           |          AA869245             |
|                       |           AW367202           |          BI695766             |
|                       |           AW068288           |          BF164215             |
|                       |           BU617726           |          BI905102             |
|                       |           AW888702           |          BI407900             |
|                       |           AI750468           |          BI407375             |
|                       |           AI491744           |          CF906894             |
|                       |           AI751986           |          BQ900571             |
|                       |           AI560658           |          AW647373             |
|                       |           AW338948           |          BU523284             |
|                       |           AW019917           |          BQ567635             |
|                       |           AW192861           |          BQ569289             |
|                       |           CA439612           |          BQ919715             |
|                       |           AI925401           |          BG087142             |
|                       |           AW026678           |          BQ265114             |
|                       |           CA445315           |          BU551787             |
|                       |           AI922732           |          AA763114             |
|                       |           BQ002076           |          AA560990             |
|                       |           BM678513           |          AI615700             |
|                       |           BQ004114           |          AA674379             |
|                       |           BQ004178           |          AA870958             |
|                       |           AI086608           |          BQ920774             |
|                       |           AI304912           |          AA798999             |
|                       |           AI750846           |          CB571558             |
|                       |           AI754939           |          BF179874             |
|                       |           BU782951           |          CA566156             |
|                       |           AW192794           |          BI455704             |
|                       |           AW068192           |          AA792545             |
|                       |           BF758707           |          BI905724             |
|                       |           AI752814           |          AW743444             |
|                       |           AW272444           |          BF582047             |
|                       |           BP228226           |          BI416060             |
|                       |           BP316318           |          BI407404             |
|                       |           CA748863           |          BE372492             |
|                       |           AI921273           |          CA564750             |
|                       |           BI493180           |          CA565803             |
|                       |           AW021834           |          BE292091             |
|                       |           BP373016           |          CF909184             |
|                       |           BM672045           |          BU952357             |
|                       |           AW888703           |          AA793412             |
|                       |             N34183           |          CF731392             |
|                       |           BM720686           |          CA979278             |
|                       |           BM722524           |          AW324007             |
|                       |           BM722432           |          BE309173             |
|                       |           BM661919           |          BI453458             |
|                       |           BI496902           |          BI455339             |
|                       |           AI569198           |          BE287505             |
|                       |           AW058436           |            W59061             |
|                       |           AA329543           |          CB317226             |
|                       |           AI446722           |          CB846744             |
|                       |           CF995389           |          AA726740             |
|                       |           AI571517           |          AA980444             |
|                       |             T70339           |          BQ565970             |
|                       |           AI752648           |          CF535005             |
|                       |           AI956042           |          BQ829439             |
|                       |           AI862323           |          AW551318             |
|                       |           BI494718           |          AA059753             |
|                       |             N55585           |          BF119928             |
|                       |             N68324           |          BQ964595             |
|                       |             N81153           |          AA008109             |
|                       |           AA339892           |          AA003622             |
|                       |           BE810481           |          CB318460             |
|                       |           CA449156           |          BI111441             |
|                       |           BM721184           |          BM875179             |
|                       |           CN390284           |          AI325291             |
|                       |           CN390408           |          BF119004             |
|                       |           AI888437           |          BQ713611             |
|                       |           AW023673           |          AI156911             |
|                       |           BI491535           |          BF152465             |
|                       |           AW020523           |          BQ831408             |
|                       |           CA446005           |          CB237172             |
|                       |           BF941312           |          BG100220             |
|                       |           BF941405           |          AA615221             |
|                       |           AW149012           |          CB235369             |
|                       |           AW023521           |          BQ085820             |
|                       |           BI492042           |            W62594             |
|                       |           BI493192           |          BQ830961             |
|                       |           AW021095           |          BE309342             |
|                       |           AW021054           |          CA566158             |
|                       |           AI814881           |          CA566176             |
|                       |           AI754578           |          BF118838             |
|                       |           AW838187           |          CB319905             |
|                       |           AI921908           |          BF124432             |
|                       |           AI446086           |          AA796109             |
|                       |           AW023799           |          BI112087             |
|                       |           AW021792           |          CB589227             |
|                       |           BI492722           |          BY345127             |
|                       |           AW608330           |          AA002525             |
|                       |           BU075119           |          BQ832164             |
|                       |           CA418758           |          BI250684             |
|                       |             N22070           |          CA978732             |
|                       |           BM991445           |            W97673             |
|                       |           AW023287           |          BQ126875             |
|                       |             W46174           |          CA978733             |
|                       |           AL038164           |          CB588069             |
|                       |           AW080814           |          BI697008             |
|                       |           AI570911           |          AA798451             |
|                       |           AA384479           |          BI454005             |
|                       |           AI983519           |          AA142691             |
|                       |           BM720648           |            W84209             |
|                       |           AI674858           |          AA032405             |
|                       |           AI751873           |          BQ832518             |
|                       |           AI624395           |          BQ830341             |
|                       |           BI497189           |            W83518             |
|                       |           BQ009731           |            W61883             |
|                       |           AA300707           |          AW212528             |
|                       |           AI074790           |            W70367             |
|                       |           BI493903           |          CA567159             |
|                       |           CN390323           |          AW743913             |
|                       |           BI493902           |          AI385406             |
|                       |           AI203839           |            W62737             |
|                       |             T84559           |          AA059754             |
|                       |           AI985941           |          BF140757             |
|                       |           AA961771           |            W97716             |
|                       |           AA853462           |          BE625685             |
|                       |           AA669922           |          BE308845             |
|                       |             N67436           |            W13280             |
|                       |           BF448005           |          AA869354             |
|                       |           BQ005661           |          BM570594             |
|                       |           AW438906           |          BM231214             |
|                       |           AI983019           |          BF099852             |
|                       |           CN483520           |          BU744143             |
|                       |           BU078424           |            W36771             |
|                       |           BU741715           |          BF227507             |
|                       |           BI492764           |          BE289358             |
|                       |           BQ026816           |          BM232452             |
|                       |             H25533           |          BM233619             |
|                       |           BM310369           |          BM232699             |
|                       |           CD679503           |          BM234393             |
|                       |           AA853210           |          BI249058             |
|                       |           AW150333           |          BM233041             |
|                       |           AI696857           |          BM231013             |
|                       |           AI887438           |          BM231124             |
|                       |           CA420532           |          AA763255             |
|                       |           BG057528           |          AW647179             |
|                       |           BG939196           |          AW647349             |
|                       |           AA852683           |          BM231814             |
|                       |           AI865892           |          AW412501             |
|                       |           BM687836           |          AA763435             |
|                       |           BM698404           |          BQ563316             |
|                       |           BF893018           |          BE382078             |
|                       |           BF893010           |          BF227630             |
|                       |           AW363376           |          AA450432             |
|                       |           AW069336           |          AI646051             |
|                       |           BG056780           |          AA762857             |
|                       |           AW883607           |          BM232450             |
|                       |           BM971530           |          BM232984             |
|                       |           AI818319           |          BM233018             |
|                       |           BQ004937           |          BM233401             |
|                       |           BU627405           |          BG869722             |
|                       |           BU628477           |          AW701417             |
|                       |           AI589415           |          BM235537             |
|                       |           AW262838           |          BM233339             |
|                       |           AA653308           |          AW553502             |
|                       |           CA426071           |          BM231357             |
|                       |           AA330541           |          BM233650             |
|                       |           BU580995           |          BF116750             |
|                       |           AI627597           |          CK342075             |
|                       |           AA852138           |          BM231037             |
|                       |           BF089050           |          BM231476             |
|                       |           AA853450           |          BM232187             |
|                       |           BU618124           |          BM232574             |
|                       |           BQ181661           |          BM233225             |
|                       |           BQ775354           |          BM233631             |
|                       |           CA426003           |          BM233965             |
|                       |           AI679598           |          BM234218             |
|                       |           BQ182559           |          BM234923             |
|                       |           CA428123           |          BM232833             |
|                       |           AI751599           |          BM233709             |
|                       |           AA022859           |          BM233771             |
|                       |           AL046996           |          BM233924             |
|                       |           CN480058           |          BM234791             |
|                       |           BM995741           |          BM235280             |
|                       |           AW263005           |          BM231532             |
|                       |           BQ020272           |          BM232944             |
|                       |           AW392438           |          BM233392             |
|                       |           AW861589           |          BM234010             |
|                       |           BF085531           |            W89727             |
|                       |           AA669889           |          BM233790             |
|                       |           CA440508           |          AA589565             |
|                       |           BQ010009           |          BM237033             |
|                       |           BQ017660           |            W62413             |
|                       |           BQ183632           |            W35727             |
|                       |           CA749001           |          BQ958719             |
|                       |           BQ005627           |          AW743474             |
|                       |           BU628377           |          BM233640             |
|                       |           BU678039           |          BE553656             |
|                       |           CA422003           |          BM231858             |
|                       |           BQ005758           |          BM233542             |
|                       |           BQ005783           |          BM231156             |
|                       |           AW580622           |          BM234047             |
|                       |           CA419408           |          BE447889             |
|                       |           BQ009980           |          BE553664             |
|                       |           CA440605           |          AW411858             |
|                       |           BM679536           |          BM233422             |
|                       |           BU737116           |          AW411834             |
|                       |           CN479652           |          BF151033             |
|                       |           BQ005012           |          AA561257             |
|                       |           BQ007951           |          BM233457             |
|                       |           BQ019347           |          BM233586             |
|                       |           BU627610           |          BM232169             |
|                       |           BU737113           |          AA646836             |
|                       |           CA421525           |          BM232969             |
|                       |           CA427544           |          BQ833462             |
|                       |           CA427718           |          BM506167             |
|                       |           CA441012           |          BB696607             |
|                       |           AW189708           |          AI604364             |
|                       |           AA037222           |          BQ944911             |
|                       |           BM991153           |          CF622834             |
|                       |           AW471276           |            W30566             |
|                       |           BU741604           |          AA500512             |
|                       |           CA434919           |          AA038896             |
|                       |           AW470129           |          AA624570             |
|                       |           BQ004674           |          AA615189             |
|                       |           AW305093           |            W10921             |
|                       |           CA428312           |          BM232433             |
|                       |             W17283           |          BE848928             |
|                       |           BU628039           |            W17969             |
|                       |           BI492004           |          AW554237             |
|                       |           BQ004472           |          BQ265113             |
|                       |           BU623738           |          BQ565551             |
|                       |           CA427914           |          AA959729             |
|                       |           CA428034           |          BF227857             |
|                       |           CA447188           |            W16148             |
|                       |           BG939070           |          CK627536             |
|                       |           BI493526           |          BM232200             |
|                       |           BM679942           |          BE447017             |
|                       |           BM997190           |          AA792447             |
|                       |           CA419206           |            W75787             |
|                       |           BM678518           |            W75794             |
|                       |           BM994909           |          BF583297             |
|                       |           BQ004579           |          BE199175             |
|                       |           BQ007849           |          BI251712             |
|                       |           BU616505           |          BE307131             |
|                       |           CA412872           |            W61839             |
|                       |           CA427777           |          AA219959             |
|                       |           CA446042           |          BB732187             |
|                       |           AW007256           |          BM232668             |
|                       |           BU627890           |          BM232674             |
|                       |           AA853301           |          BM235472             |
|                       |           AA341076           |          BM220559             |
|                       |           BE049613           |          BB678143             |
|                       |           AW029598           |          BM235036             |
|                       |           AI828428           |          BB692491             |
|                       |           AW084626           |          AA011810             |
|                       |           AW837969           |          CB844824             |
|                       |           AA298249           |          AI882450             |
|                       |           CN390546           |          AA466398             |
|                       |           AW020978           |          BQ265244             |
|                       |           BF790233           |          AA038922             |
|                       |           BI491937           |          BB694096             |
|                       |           BE815149           |          BB759524             |
|                       |           BE815157           |          BM217779             |
|                       |           AA332891           |          CD741746             |
|                       |           AA853801           |          BB691361             |
|                       |             N31613           |            W66963             |
|                       |           AI082277           |          BE290098             |
|                       |           AW069735           |          BG074512             |
|                       |           AA970852           |          BM233173             |
|                       |           AW024032           |          BM233219             |
|                       |           AW051420           |          BM236533             |
|                       |           BI493756           |          BM236627             |
|                       |           AA852214           |          BM233860             |
|                       |           AW291886           |          BM232916             |
|                       |           BM679570           |          AA799209             |
|                       |           AW513278           |          AA762663             |
|                       |           AW304162           |          AA789927             |
|                       |           CA448357           |          BQ566594             |
|                       |           BM090776           |          BM233510             |
|                       |           BG057935           |          BB692908             |
|                       |           BM680403           |          AW411772             |
|                       |           AA853809           |          BE310901             |
|                       |           AW190620           |          BF099857             |
|                       |           AA551240           |          BB681379             |
|                       |           BQ004735           |          BM236363             |
|                       |           AW007952           |          BM234054             |
|                       |           AW020008           |          CB575147             |
|                       |           AI254720           |          BM236407             |
|                       |           BQ573853           |          CD772651             |
|                       |           BI491884           |          BF161521             |
|                       |           AL037529           |          BB697772             |
|                       |           BI491032           |          BE623302             |
|                       |           AW608428           |            W11675             |
|                       |           AW608437           |          BM231503             |
|                       |             H89540           |          BM234016             |
|                       |           AW511792           |          AA500545             |
|                       |           AW087359           |          BB670660             |
|                       |           BM722388           |          BB670030             |
|                       |           AI445471           |          BE285264             |
|                       |           AW612962           |          BM231905             |
|                       |           AA852103           |          BM233391             |
|                       |           CN390540           |          BQ947610             |
|                       |           AW770436           |          BM235007             |
|                       |           AA853655           |          BB761431             |
|                       |           AW023787           |          AA759986             |
|                       |           AI274651           |          BY754758             |
|                       |           BM679136           |          CF736062             |
|                       |           AA359572           |          BI152823             |
|                       |           AA359288           |          BB670344             |
|                       |           AU144156           |          CF726364             |
|                       |           BM999089           |          AA286523             |
|                       |           AI720522           |            W82139             |
|                       |           AI950737           |          BM233284             |
|                       |           AA340377           |          AA097381             |
|                       |           BQ772296           |          AA960264             |
|                       |           BQ029357           |          BB670012             |
|                       |           AI283965           |          BB678916             |
|                       |             T55483           |          AA036233             |
|                       |           AI681959           |          BB681255             |
|                       |           BP231337           |          BI646428             |
|                       |           BF357914           |          BY358950             |
|                       |           BE815164           |          CN527316             |
|                       |           AA852424           |          BB669350             |
|                       |           BM994162           |          BB678203             |
|                       |           BQ004628           |          BB670107             |
|                       |           BQ020136           |          AA008565             |
|                       |           BQ772317           |          BM233309             |
|                       |           BQ774586           |          BB670038             |
|                       |           BQ775179           |          BB678247             |
|                       |           BQ776276           |          BB749233             |
|                       |           BU615329           |          AA727373             |
|                       |           CA415000           |          BM218253             |
|                       |           CA420293           |          BB677501             |
|                       |           BE858596           |          BB669789             |
|                       |           BQ004441           |          BB670437             |
|                       |           BQ017884           |          BY686571             |
|                       |           BI497012           |          BE308807             |
|                       |           BQ019422           |          BB693767             |
|                       |           CA427800           |          BM234332             |
|                       |           BM990688           |          BB676327             |
|                       |           AA599889           |          BB678594             |
|                       |           AA582588           |          BB670375             |
|                       |           CF596176           |          BB670428             |
|                       |           AW069125           |          BY697929             |
|                       |           BI496315           |          BB678752             |
|                       |           AI524656           |          AA388366             |
|                       |           AW021749           |          BB669331             |
|                       |           AW078763           |          BB670112             |
|                       |           AW780308           |          BB674317             |
|                       |           BI492688           |          BE686073             |
|                       |           BF357972           |          BB672038             |
|                       |           BE149458           |          BB670067             |
|                       |           CD511312           |          BB670088             |
|                       |           AA037077           |          BB676330             |
|                       |           AW023923           |          BB748804             |
|                       |           AI597938           |          BB676401             |
|                       |           AI696981           |          BB669996             |
|                       |           AA477045           |          BB670049             |
|                       |             W00666           |          BB674291             |
|                       |           AI707837           |          BB670094             |
|                       |           BQ182352           |          BB678707             |
|                       |           AI140288           |          BB732498             |
|                       |             H88723           |          CB572974             |
|                       |           CA448297           |          AL364660             |
|                       |             R66843           |          BB670064             |
|                       |           AI598262           |          BM218315             |
|                       |             R50240           |          CD772736             |
|                       |           AW874429           |          BM219522             |
|                       |             R77329           |          BB670098             |
|                       |             N75208           |          BB670656             |
|                       |           CB157492           |          BB670019             |
|                       |           BE932431           |          BB669848             |
|                       |           AI692249           |          BB674588             |
|                       |           AW513323           |          BB670031             |
|                       |             W68316           |          BB670373             |
|                       |             H89143           |          BB675391             |
|                       |           AI751244           |          BY696056             |
|                       |           AI696526           |          BB669984             |
|                       |             W68318           |          BB669964             |
|                       |           AA599454           |          BB691638             |
|                       |           AA669925           |          BB674097             |
|                       |           AA287227           |          BB675241             |
|                       |           AI754401           |          BB670297             |
|                       |           AW029333           |          BB670779             |
|                       |           BF698462           |          BB732496             |
|                       |           BU627523           |          BB671844             |
|                       |           CA433800           |          AW240485             |
|                       |           AI754466           |          BB678936             |
|                       |           CA412668           |          BF450145             |
|                       |           BM672676           |          BY360079             |
|                       |           AA295387           |          BB684442             |
|                       |           AA295392           |          BB670369             |
|                       |           AA295393           |          BY358896             |
|                       |           AA295174           |          BB669998             |
|                       |           AI752298           |          BB670024             |
|                       |           BU615588           |          BB682991             |
|                       |           AA284498           |          BB670078             |
|                       |           BE710665           |          BB670085             |
|                       |             R71565           |          BB672984             |
|                       |             H89074           |          BB669233             |
|                       |           AA481909           |          BB676565             |
|                       |           BI493344           |          BY690947             |
|                       |           CA415100           |          BY694850             |
|                       |           CN480145           |          BB669759             |
|                       |           AW192479           |          BB686277             |
|                       |           AW337813           |          BB670060             |
|                       |             H88474           |          BB674518             |
|                       |           CF129168           |          BQ569729             |
|                       |           AL039496           |          CB204566             |
|                       |           AA599824           |          BM237262             |
|                       |           AI493212           |          BB678445             |
|                       |           AW022026           |          BY697305             |
|                       |           BM995800           |          BB670169             |
|                       |           BI492978           |          BB674943             |
|                       |           CR740714           |          BB677627             |
|                       |           AW513253           |          BB670008             |
|                       |           BI494983           |          BY693785             |
|                       |           BI494984           |          AA821525             |
|                       |           BU930231           |          BB674118             |
|                       |           AA386036           |          BB678506             |
|                       |           AI751481           |          BB673347             |
|                       |           AA599358           |          BB674232             |
|                       |             W47112           |          BB678322             |
|                       |           BU617512           |          BB678362             |
|                       |           BX095546           |          BB678408             |
|                       |           AW088926           |            W11726             |
|                       |           AA599828           |          BB669777             |
|                       |           AI753340           |          BB670140             |
|                       |           BM667187           |          BB673331             |
|                       |           BF436746           |          BB675938             |
|                       |           AI751605           |          BB678238             |
|                       |             F01818           |          BY691554             |
|                       |           BI494719           |          BB678396             |
|                       |           CA441828           |          BB678233             |
|                       |           AI444953           |          BB670165             |
|                       |           BU735787           |          BB670213             |
|                       |           AI750675           |          BB673812             |
|                       |           AW069615           |          BB678297             |
|                       |           AI355313           |          BB678345             |
|                       |           AA582261           |          BB678435             |
|                       |             H68446           |          BQ565897             |
|                       |           BM667505           |            W13779             |
|                       |           BQ017667           |          BB674480             |
|                       |           BM667297           |          BB678195             |
|                       |             N66753           |          CF554208             |
|                       |           BI495309           |          BB669066             |
|                       |           AW023242           |          BB678251             |
|                       |           BI495310           |          BB678318             |
|                       |             W03720           |          BB678227             |
|                       |             N44517           |          BB678294             |
|                       |           AI752592           |          BB670208             |
|                       |           CB214999           |          BB670240             |
|                       |           BI496010           |          BB670543             |
|                       |           CB045026           |          BB732641             |
|                       |           AI751498           |          CF908202             |
|                       |           AI751398           |          BB677151             |
|                       |           AI926321           |          BB679980             |
|                       |           AA480055           |          BB678232             |
|                       |             R07367           |          BB670640             |
|                       |             N66884           |          BB675975             |
|                       |           AA662234           |          BB678527             |
|                       |             H27123           |          BB678557             |
|                       |           BM698068           |          BB678576             |
|                       |           AI366489           |            W78542             |
|                       |           AI754388           |          CB318629             |
|                       |           AI446477           |          BQ565408             |
|                       |           BU151718           |          BB669340             |
|                       |           BU740163           |          BB677587             |
|                       |           BI493666           |          BB678657             |
|                       |           BI493665           |          BB670193             |
|                       |           BI497329           |          BB676696             |
|                       |           BQ004832           |          BB678191             |
|                       |           BI496019           |          BB678317             |
|                       |           BI496020           |          BB679170             |
|                       |             N68071           |          BB678574             |
|                       |           AA995864           |          BY481177             |
|                       |           BI497368           |          BB669598             |
|                       |           BG938636           |          BE287125             |
|                       |           BQ008283           |          BB670214             |
|                       |           CA440715           |          BB674741             |
|                       |           AI801495           |          BB686107             |
|                       |           BM677309           |          BB678209             |
|                       |           AA642513           |          BB673068             |
|                       |           BF437406           |          BB678570             |
|                       |           AI584088           |          BB679135             |
|                       |           AW020573           |          BB669294             |
|                       |           BU624779           |          BB669310             |
|                       |           AW873128           |          BB669349             |
|                       |           BF436652           |          BB669596             |
|                       |           AI752046           |          BB678633             |
|                       |           BF436444           |          BB670621             |
|                       |           AI752117           |          BB678524             |
|                       |           BM729244           |          BB678642             |
|                       |           AA376033           |          BB731829             |
|                       |             N53237           |          BB678467             |
|                       |           BF436499           |          BB678654             |
|                       |           AI080597           |          BB669274             |
|                       |           BM683229           |          BB673759             |
|                       |           BX489097           |          BB676200             |
|                       |           AL040456           |          BB677454             |
|                       |           BQ181136           |          BB678622             |
|                       |           AI754031           |          BY358800             |
|                       |           AI754910           |          BY359698             |
|                       |           AI753260           |          BB669269             |
|                       |           AI754413           |          BB669248             |
|                       |           AI754815           |          BB678534             |
|                       |           CA436822           |          BB678628             |
|                       |           AI080554           |          BB672864             |
|                       |           CA439860           |          BB675576             |
|                       |           CA445830           |          BB677423             |
|                       |           CA445916           |          BB677557             |
|                       |           AW069318           |          BB678186             |
|                       |           AI753499           |          BB678255             |
|                       |           AW069638           |          BB678335             |
|                       |           AI753064           |          BB678363             |
|                       |           AW069317           |          CB842481             |
|                       |           AI754655           |          CF733842             |
|                       |           AI753296           |          BY683986             |
|                       |           AI754174           |          BY361045             |
|                       |           AI754836           |          BB678604             |
|                       |           CA414403           |          BB673584             |
|                       |           BI491900           |          BB678426             |
|                       |           BI497214           |          BB731822             |
|                       |           BM673977           |          CB587655             |
|                       |           BM715512           |          BY699399             |
|                       |           AI752957           |          BB690878             |
|                       |           BI493027           |          BB678583             |
|                       |           AL048681           |          BB669196             |
|                       |           BI494575           |          BB677086             |
|                       |           AI042258           |          BB678264             |
|                       |           BU736137           |          BM235024             |
|                       |           AI127326           |          BY683605             |
|                       |           BQ027906           |          BY683475             |
|                       |           BI494009           |          BB669300             |
|                       |           AI952322           |          BB669307             |
|                       |           AA931996           |          BB670171             |
|                       |           CA445901           |          BB670190             |
|                       |           BM993163           |          BB678377             |
|                       |           AI753925           |          BB669312             |
|                       |           AW021305           |          BB669941             |
|                       |           BI492337           |          BB676092             |
|                       |             N67590           |          BB678184             |
|                       |           AW069703           |          BB670199             |
|                       |           BI493410           |          BB670200             |
|                       |           AI631420           |          BB678334             |
|                       |           AI753362           |          BB678404             |
|                       |             N64255           |          BB669311             |
|                       |             N68120           |          BB669788             |
|                       |           BM677220           |          BB669938             |
|                       |           BU615279           |          BB670198             |
|                       |           AW020051           |          BB677876             |
|                       |           BI491077           |          BB677915             |
|                       |           BQ028969           |          BB678206             |
|                       |           AW021165           |          BB678215             |
|                       |           BI492112           |          BB678319             |
|                       |           AI250152           |          BB678493             |
|                       |           AI250657           |          BB669885             |
|                       |           AI696770           |          BB678172             |
|                       |           BI494560           |          BB669914             |
|                       |           AW019977           |          BB669944             |
|                       |           BI491002           |          BB670439             |
|                       |           AI755044           |          BB670136             |
|                       |           AI288099           |          BB669203             |
|                       |           BI494279           |          BB669282             |
|                       |           BM667722           |          BB669309             |
|                       |           AI752929           |          BB669687             |
|                       |             N67522           |          BB678262             |
|                       |           AW023030           |          BB678554             |
|                       |           AW020804           |          BB678585             |
|                       |           AI753439           |          BB678667             |
|                       |           BU735766           |          AA855983             |
|                       |           CN482069           |          BB669954             |
|                       |           AW024043           |          BB673675             |
|                       |           CA414367           |          BB675558             |
|                       |           BI496886           |          BB677192             |
|                       |           AW276371           |          BB678308             |
|                       |           BU735704           |          BB678441             |
|                       |           BQ027051           |          BB678669             |
|                       |           AA522641           |          BY686924             |
|                       |           BU735615           |          BB669039             |
|                       |           BU853826           |          BB675360             |
|                       |           AW020879           |          BB677199             |
|                       |           BG026362           |          BB677290             |
|                       |           CA447245           |          BB669247             |
|                       |           BQ026982           |          BB669667             |
|                       |           CA414651           |          BB674042             |
|                       |           BQ027038           |          BB675217             |
|                       |           CA445352           |          BB676398             |
|                       |           BQ010068           |          BB678196             |
|                       |           BM679202           |          BB678273             |
|                       |           BQ007686           |          BB678502             |
|                       |           AI559274           |          BY687897             |
|                       |           BM671972           |            W17523             |
|                       |           CA447157           |          BB675305             |
|                       |           BM678965           |          BB675306             |
|                       |           BM678492           |          BB678540             |
|                       |           BM679079           |          BB676366             |
|                       |           BU628524           |          BB676949             |
|                       |           CA428871           |          BB678610             |
|                       |           CA447745           |          BB669357             |
|                       |           BQ008709           |          BB672790             |
|                       |           AI983991           |          BB677091             |
|                       |           BM678955           |          BY686873             |
|                       |           BU615549           |          BB669091             |
|                       |           BM678919           |          BB669215             |
|                       |           BX501227           |          BB669006             |
|                       |           CA449164           |          BB669262             |
|                       |           BM995116           |          BB669314             |
|                       |           BM972492           |          BB669906             |
|                       |           BQ007833           |          CD773028             |
|                       |           BQ028802           |          AA518868             |
|                       |           CA447660           |          BB669835             |
|                       |           AI571507           |          BB669900             |
|                       |           CA420792           |          BB678182             |
|                       |           BM995696           |          BB669182             |
|                       |           AW021189           |          BB669058             |
|                       |           CA445347           |          BB669063             |
|                       |           BM679605           |          BB669930             |
|                       |           BQ008058           |          BB672815             |
|                       |           CA414317           |          BB678649             |
|                       |           BM678531           |          BB668996             |
|                       |           AI991743           |          BB669067             |
|                       |           BQ019374           |          BB669159             |
|                       |           CA420559           |          BY687005             |
|                       |           AA928581           |          BF121342             |
|                       |           BQ017778           |          BB669181             |
|                       |           CA414701           |          BB668998             |
|                       |           BI494110           |          BB676487             |
|                       |           BI494111           |          AW493948             |
|                       |           CA440919           |          BB669065             |
|                       |           BI493932           |          BB669893             |
|                       |           CA448969           |          BB669973             |
|                       |           AW023921           |          BB669993             |
|                       |           BQ182840           |          BB676445             |
|                       |           BQ004935           |          BB680354             |
|                       |           CA446038           |          BY698323             |
|                       |           BQ020221           |          BB669145             |
|                       |           BQ005038           |          BB669147             |
|                       |           BQ005690           |          BB669093             |
|                       |           AW069296           |          BB669117             |
|                       |           BG939476           |          BB669981             |
|                       |           BI491812           |          BB689979             |
|                       |           CA421600           |          BB676508             |
|                       |           BQ773502           |          BB669119             |
|                       |           AW337248           |          BB669151             |
|                       |           BM970332           |          BB669222             |
|                       |           BM679072           |          BB675408             |
|                       |           AI755125           |          BB676475             |
|                       |           BQ010070           |          BB669854             |
|                       |           BU737094           |          BB669169             |
|                       |           BU681546           |          BY358873             |
|                       |           BM679677           |          BB669020             |
|                       |           BU616342           |          BB676571             |
|                       |           BU627764           |          BB686918             |
|                       |           CA412455           |          BB669149             |
|                       |           BM678691           |            W98082             |
|                       |           BM679032           |          AA086991             |
|                       |           CA411825           |          AA123411             |
|                       |           CA420604           |          BB669002             |
|                       |           CA421760           |          BB669048             |
|                       |           BQ008056           |          AA572204             |
|                       |           CA414943           |          BB669027             |
|                       |           BM997191           |          BB669133             |
|                       |           BQ004179           |          BB678090             |
|                       |           CA414934           |          BB669075             |
|                       |           CA440990           |          BB669112             |
|                       |           BM679124           |          BF150495             |
|                       |           BQ007957           |          BF455498             |
|                       |           BQ026908           |          BM236519             |
|                       |           BQ026941           |          BB669141             |
|                       |           BQ026943           |          BY685943             |
|                       |           BU627093           |          BB669015             |
|                       |           CA412359           |          AI836775             |
|                       |           CA413709           |          AA050788             |
|                       |           CA414622           |          BB669188             |
|                       |           CA414731           |          BB669102             |
|                       |           CA420325           |          BB669108             |
|                       |           CA445300           |          BB669161             |
|                       |           CA445410           |          AA288886             |
|                       |           CA447228           |          BY700333             |
|                       |           CA447516           |          CN529560             |
|                       |           BQ005626           |            W11136             |
|                       |           CA413583           |          AI841855             |
|                       |           CA428862           |          AI841886             |
|                       |           CA420972           |            W59416             |
|                       |           CA448541           |          AA041962             |
|                       |           BQ773397           |          BM231325             |
|                       |           BE221675           |          BB147321             |
|                       |           BQ004725           |          AA051535             |
|                       |           BM678485           |          BM233266             |
|                       |           BM997181           |          BB669028             |
|                       |           BQ045028           |          AA051178             |
|                       |           BQ183408           |            W12309             |
|                       |           BU627931           |          CB587569             |
|                       |           CA426930           |          AA798297             |
|                       |           CA446050           |          BM219264             |
|                       |           CA414081           |          BB678043             |
|                       |           BU627490           |          BE289840             |
|                       |           BU681569           |          BE290093             |
|                       |           BQ004393           |          BB678015             |
|                       |           BM512020           |          AA220080             |
|                       |           BI495057           |          BM235824             |
|                       |           BQ020352           |          BB122053             |
|                       |           CA412006           |          BY689565             |
|                       |           AW069175           |          BI454884             |
|                       |           BM679283           |          BB144283             |
|                       |           AW874262           |          BM119534             |
|                       |           BM996929           |          BB464977             |
|                       |           AW069261           |          CB588567             |
|                       |           BI491791           |          BB692962             |
|                       |           AI754113           |          BB144092             |
|                       |           AI801341           |          AI119883             |
|                       |           BM996450           |          AI324073             |
|                       |           BQ019912           |          BB678120             |
|                       |           BQ573709           |          BB669418             |
|                       |           BM667231           |          BB678089             |
|                       |           BM995052           |          BY690065             |
|                       |           BM995294           |          CN527019             |
|                       |           BM996904           |          BB233010             |
|                       |           BU687841           |          BB678105             |
|                       |           AI754485           |          BB669437             |
|                       |           AW069257           |          BB678110             |
|                       |           CA441925           |          BB678057             |
|                       |           BE328000           |            W78431             |
|                       |           AW190939           |          BX512479             |
|                       |           AI457138           |          BU788463             |
|                       |           AI754375           |            W29395             |
|                       |           CA419910           |          BB669457             |
|                       |           BU615318           |          BB669507             |
|                       |           AI829344           |          BB678084             |
|                       |           AA599432           |          BB669470             |
|                       |           CA440908           |          AI181311             |
|                       |           BU618115           |          BB678067             |
|                       |           BI496009           |          BY688983             |
|                       |           AI829353           |          AI462070             |
|                       |           AA599421           |          BB669372             |
|                       |           AI754743           |          BB669540             |
|                       |           AW591877           |          BB678139             |
|                       |           BI496431           |          BB669521             |
|                       |           CA412674           |          BB678056             |
|                       |           BI494391           |          BM875263             |
|                       |           BM667401           |          BB678136             |
|                       |           BI494011           |          AV165652             |
|                       |           AA446324           |          BB678134             |
|                       |           BI492905           |          BB669410             |
|                       |           BI492641           |          BB669417             |
|                       |           BQ772805           |          BB678159             |
|                       |           BU621499           |          AA733545             |
|                       |           BQ019762           |          CK341814             |
|                       |           CA416727           |          BQ963692             |
|                       |           BU740025           |          BB375972             |
|                       |           AI445464           |          BG975084             |
|                       |           AI582548           |          BB669375             |
|                       |           BQ004175           |          BB099208             |
|                       |           AI537166           |          BB678129             |
|                       |           AI077912           |          BB678144             |
|                       |           BI495207           |          AA022319             |
|                       |           BI495208           |          BI454863             |
|                       |           AA603177           |          BB669558             |
|                       |           BF448535           |          BB678112             |
|                       |           BU736186           |          AA039007             |
|                       |           AI672201           |          BB678097             |
|                       |           BM679611           |          BB669533             |
|                       |           BQ017693           |          BB669549             |
|                       |           AI955867           |          BB678091             |
|                       |           AA860263           |          CK625772             |
|                       |           AI520725           |          BB140282             |
|                       |           AI096348           |          BM220344             |
|                       |           AI435232           |          AA033271             |
|                       |           CA411846           |          AA049236             |
|                       |           AI955544           |          BB669390             |
|                       |           BQ004553           |          BB669398             |
|                       |           BM679332           |          AA512692             |
|                       |           AI584068           |          BB669525             |
|                       |           AW069076           |          BB372494             |
|                       |           AI755165           |          CK341849             |
|                       |           BF448563           |          BB669536             |
|                       |           AI753501           |          BB678167             |
|                       |           AI755233           |          BB669495             |
|                       |           AI753481           |          BB678116             |
|                       |           BU736208           |          BB678131             |
|                       |           AI754766           |          AV235150             |
|                       |           AI755189           |            W80269             |
|                       |           AI922779           |          BB669552             |
|                       |           BM679387           |          BB669555             |
|                       |           AI801453           |          BB669453             |
|                       |           AW069409           |          BB678009             |
|                       |           AI074234           |          BM231035             |
|                       |           AI636393           |          CK341874             |
|                       |           AI376912           |          BB678135             |
|                       |           BI491069           |          BM234485             |
|                       |           BM667638           |          CK342057             |
|                       |           AW104448           |          CK342113             |
|                       |           AI610692           |          BM570821             |
|                       |           BM667678           |          CK342084             |
|                       |           BM679355           |          BB376092             |
|                       |           BM679353           |            W14051             |
|                       |           CA413558           |          BF227756             |
|                       |             N67468           |          BB669377             |
|                       |           AI753632           |          BQ126567             |
|                       |           AI754595           |            W99021             |
|                       |           BU736183           |          AA265475             |
|                       |           AI755130           |          CK342360             |
|                       |             N64585           |          CD742064             |
|                       |           AI042248           |          CK341996             |
|                       |           AI452935           |          CK341869             |
|                       |           AI961907           |          CK342450             |
|                       |           BM667342           |          CD741245             |
|                       |           AI753881           |          CK341643             |
|                       |             N22402           |          BB722570             |
|                       |           AW025810           |          BM232642             |
|                       |             N66359           |          BG228062             |
|                       |           AI752505           |          AV242866             |
|                       |             R42997           |          BB696155             |
|                       |           AI753110           |          AA562907             |
|                       |           AI754915           |          BG276156             |
|                       |           AI352594           |          AI841907             |
|                       |           AW137810           |          AI841679             |
|                       |           AI754101           |          BB732702             |
|                       |           AW069776           |          BI455714             |
|                       |           AW068979           |          AW412568             |
|                       |           AW069206           |          BU524233             |
|                       |           AW069522           |          CK342292             |
|                       |           AI754272           |          BB669249             |
|                       |           AI753154           |          BB669476             |
|                       |             N68114           |          BM234209             |
|                       |             H06981           |          BE554361             |
|                       |             N94363           |          AV227070             |
|                       |           AI356602           |          BE553730             |
|                       |           CA445941           |          CK342093             |
|                       |           BU736184           |          AV242223             |
|                       |           AW069707           |          CK342114             |
|                       |           AI753708           |          AW048443             |
|                       |           AI569311           |          BI248712             |
|                       |           CR745323           |          AA028329             |
|                       |           BI493119           |            W33702             |
|                       |             N66305           |          AA241178             |
|                       |           AW021243           |          CK342630             |
|                       |           BI492183           |          BE289331             |
|                       |           BI492247           |          CD741518             |
|                       |           BI495286           |          AK003150             |
|                       |           AW019953           |          BC023203             |
|                       |           AW020556           |          BQ563601             |
|                       |           AW022612           |          BQ563602             |
|                       |           AW022689           |          BG090712             |
|                       |           AW022783           |          BF149080             |
|                       |           AW022806           |          BG092448             |
|                       |           AW022881           |          AI838329             |
|                       |           BI490985           |          AI838652             |
|                       |           BI492957           |          AI838618             |
|                       |           BI496996           |          AI841672             |
|                       |           BM710911           |          CD740914             |
|                       |           BI494032           |          CD741297             |
|                       |           BI494033           |          CD741962             |
|                       |           AW020994           |          BQ567641             |
|                       |           BG938668           |          BQ833474             |
|                       |           AI962828           |          AA175431             |
|                       |           AI659779           |          AI845840             |
|                       |                              |          CD741752             |
|                       |                              |          AI849281             |
|                       |                              |          CD741663             |
|                       |                              |          BQ569527             |
|                       |                              |          BI083318             |
|                       | *Seq. mapped in strand Minus*| *Seq. mapped in strand Minus* |
|                       |           BU687304           |          BY734807             |
|                       |           BI491775           |          CK021731             |
|                       |           AI913204           |          BY020288             |
|                       |           AI473965           |          BY333189             |
|                       |           BM699523           |                               |
+-----------------------+------------------------------+-------------------------------+
|      #  132           | *Seq. mapped in strand Plus* | *Seq. mapped in strand Plus*  |
|                       |           BC021264           |          AK039030             |
|                       | *Seq. mapped in strand Minus*| *Seq. mapped in strand Minus* |
|                       |           AY014284           |          AK081029             |
+-----------------------+------------------------------+-------------------------------+
|      #  133           | *Seq. mapped in strand Plus* | *Seq. mapped in strand Plus*  |
|                       |           CN260703           |          BY357678             |
|                       |           BM720349           |                               |
|                       |           CB992122           |                               |
|                       |           CB994074           |                               |
|                       |           BQ069051           |                               |
|                       |           BG471823           |                               |
|                       |           BM746779           |                               |
|                       |           BM788256           |                               |
|                       |           BM740785           |                               |
|                       |           BF307960           |                               |
|                       |           BF527484           |                               |
|                       |           BG036304           |                               |
|                       |           BX342590           |                               |
|                       |           CR613137           |                               |
|                       |           BX436590           |                               |
|                       |           BX397181           |                               |
|                       |           CR625948           |                               |
|                       |           BX442219           |                               |
|                       |           BE018721           |                               |
|                       |           BI756898           |                               |
|                       |           BC039617           |                               |
|                       |           BG325819           |                               |
|                       |           AL539312           |                               |
|                       |           BI554056           |                               |
|                       |           CR600070           |                               |
|                       |           BG910821           |                               |
|                       |           CA487389           |                               |
|                       |           BF345422           |                               |
|                       |           BM921627           |                               |
|                       |           BG899955           |                               |
|                       |           BE258700           |                               |
|                       |           BM470922           |                               |
|                       |           BM924338           |                               |
|                       |           BC064425           |                               |
|                       |           BM699864           |                               |
|                       |           AB054538           |                               |
|                       |           BI256785           |                               |
|                       |           CN260706           |                               |
|                       |           BU521224           |                               |
|                       |           BI819623           |                               |
|                       |           BU172255           |                               |
|                       |           BX456279           |                               |
|                       |           BC050666           |                               |
|                       |           BE379104           |                               |
|                       |           CN260707           |                               |
|                       |           BM475948           |                               |
|                       |           BG912987           |                               |
|                       |           BE886053           |                               |
|                       |           BI824438           |                               |
|                       |           BI915788           |                               |
|                       |           BF684703           |                               |
|                       |           BF347425           |                               |
|                       |           CD675378           |                               |
|                       |           BG393647           |                               |
|                       |           BG393724           |                               |
|                       |           BQ878487           |                               |
|                       |           CN260704           |                               |
|                       |           CD673701           |                               |
|                       |           BE885035           |                               |
|                       |           BF876388           |                               |
|                       |           BM696101           |                               |
|                       |           BM692017           |                               |
|                       |           BG830285           |                               |
|                       |           AI452939           |                               |
|                       |           BF871296           |                               |
|                       |           BX436589           |                               |
|                       |           BM976004           |                               |
|                       |             Z43866           |                               |
|                       |           CN260705           |                               |
|                       |           BF806686           |                               |
|                       |           AW207170           |                               |
|                       |           AI380875           |                               |
|                       |           BM727532           |                               |
|                       |             W44366           |                               |
|                       |           CA311215           |                               |
|                       |           BQ574774           |                               |
|                       |           CB853228           |                               |
|                       |           BU620401           |                               |
|                       |           BU634402           |                               |
|                       |           CA310187           |                               |
|                       |           CA503148           |                               |
|                       |           CA312871           |                               |
|                       |           CD367009           |                               |
|                       |           BU689693           |                               |
|                       |           CB305805           |                               |
|                       |           BG489553           |                               |
|                       |           BQ002820           |                               |
|                       |           BF972866           |                               |
|                       |           BU526738           |                               |
|                       |           BM977033           |                               |
|                       |           BM664386           |                               |
|                       |           BG757152           |                               |
|                       |           CB851813           |                               |
|                       |           BM681745           |                               |
|                       |           BU621437           |                               |
|                       |           BX342589           |                               |
|                       |           BF514616           |                               |
|                       |           BM678747           |                               |
|                       |           AI928233           |                               |
|                       |           AI609368           |                               |
|                       |           BU625900           |                               |
|                       |           BU733172           |                               |
|                       |           AI636558           |                               |
|                       |           BM987529           |                               |
|                       |           AI971476           |                               |
|                       |           BU738974           |                               |
|                       |           BQ010502           |                               |
|                       |           AI366670           |                               |
|                       |           BM670581           |                               |
|                       |           CA433506           |                               |
|                       |           CA425723           |                               |
|                       |           BU680875           |                               |
|                       |           CN403795           |                               |
|                       |           CN403791           |                               |
|                       |           BE548803           |                               |
|                       |           BI458557           |                               |
|                       |           BM547532           |                               |
|                       |           BI859610           |                               |
|                       |           BI600693           |                               |
|                       |           BQ420282           |                               |
|                       |           BF663962           |                               |
|                       |           BF976430           |                               |
|                       |           BG912152           |                               |
|                       |             W24575           |                               |
|                       |           BC010012           |                               |
|                       |           BF726607           |                               |
|                       |           BG032936           |                               |
|                       |           BC071626           |                               |
|                       |           BI596577           |                               |
|                       |           BG701669           |                               |
|                       |           BG716152           |                               |
|                       |           CB997070           |                               |
|                       |           BM695947           |                               |
|                       |           BM810403           |                               |
|                       |           CA395821           |                               |
|                       |           BQ720263           |                               |
|                       |           CR619748           |                               |
|                       |           CN403790           |                               |
|                       |           BM549199           |                               |
|                       |           BU169524           |                               |
|                       |           BF971439           |                               |
|                       |           CN403797           |                               |
|                       |           CK430508           |                               |
|                       |           BF309824           |                               |
|                       |           BG111586           |                               |
|                       |           BI964042           |                               |
|                       |           BG288937           |                               |
|                       |           AL529660           |                               |
|                       |           AL533175           |                               |
|                       |           CR618691           |                               |
|                       |           BM686810           |                               |
|                       |           BE906705           |                               |
|                       |           BQ719848           |                               |
|                       |           BQ901208           |                               |
|                       |           CB998291           |                               |
|                       |           CD110176           |                               |
|                       |           BM547885           |                               |
|                       |           BX345362           |                               |
|                       |           CB993855           |                               |
|                       |           CB996965           |                               |
|                       |           CR626166           |                               |
|                       |           BC071850           |                               |
|                       |           BP308345           |                               |
|                       |           CB113639           |                               |
|                       |           BM799744           |                               |
|                       |           BE314426           |                               |
|                       |           CR607952           |                               |
|                       |           AL528013           |                               |
|                       |           BM718348           |                               |
|                       |           BM707932           |                               |
|                       |           BG120898           |                               |
|                       |           BQ420303           |                               |
|                       |           CD675568           |                               |
|                       |           CN403787           |                               |
|                       |           BG674390           |                               |
|                       |           BM805624           |                               |
|                       |           BI832568           |                               |
|                       |           BQ639897           |                               |
|                       |           BE905601           |                               |
|                       |           CN403801           |                               |
|                       |           BU738377           |                               |
|                       |           BI754906           |                               |
|                       |           CF140269           |                               |
|                       |           BE910075           |                               |
|                       |           BM699673           |                               |
|                       |           BM712142           |                               |
|                       |           BI045728           |                               |
|                       |           CN403796           |                               |
|                       |           AI133427           |                               |
|                       |           CN403793           |                               |
|                       |             Z42333           |                               |
|                       |           BM729101           |                               |
|                       |           BM917896           |                               |
|                       |           BG822849           |                               |
|                       |           BG750613           |                               |
|                       |           BQ361194           |                               |
|                       |           BM687156           |                               |
|                       |           BG820526           |                               |
|                       |           CN403802           |                               |
|                       |           BM672983           |                               |
|                       |           BE255716           |                               |
|                       |           BC064482           |                               |
|                       |           BE735736           |                               |
|                       |           BG472267           |                               |
|                       |           BM424034           |                               |
|                       |           AA350360           |                               |
|                       |           AF087888           |                               |
|                       |           BQ336064           |                               |
|                       |           AW407842           |                               |
|                       |           AI372759           |                               |
|                       |           CN403788           |                               |
|                       |           BE394389           |                               |
|                       |           BF846202           |                               |
|                       |           BI549863           |                               |
|                       |           CN403794           |                               |
|                       |             W23910           |                               |
|                       |           BF573284           |                               |
|                       |           BQ422505           |                               |
|                       |           BQ331945           |                               |
|                       |           AI192912           |                               |
|                       |           BI225753           |                               |
|                       |           AA742707           |                               |
|                       |           CN403792           |                               |
|                       |           CK430963           |                               |
|                       |           AI336808           |                               |
|                       |             R60434           |                               |
|                       |             R18809           |                               |
|                       |           BF093554           |                               |
|                       |           CA867553           |                               |
|                       |           BX397180           |                               |
|                       |           AW578878           |                               |
|                       |           AI669449           |                               |
|                       |             T85919           |                               |
|                       |           AL563035           |                               |
|                       |           BG032361           |                               |
|                       |           AA223799           |                               |
|                       |           CB268436           |                               |
|                       |           BF871342           |                               |
|                       |           BF871214           |                               |
|                       |           CB995923           |                               |
|                       |           CB993846           |                               |
|                       |             R88070           |                               |
|                       |           BF932320           |                               |
|                       |           BI481315           |                               |
|                       |           BE837574           |                               |
|                       |           BX361783           |                               |
|                       |           AW973158           |                               |
|                       |           AA536032           |                               |
|                       |           AW973135           |                               |
|                       |           AA328758           |                               |
|                       |           BM804710           |                               |
|                       |           BX345361           |                               |
|                       |           AA083263           |                               |
|                       |           CN403798           |                               |
|                       |           BM825657           |                               |
|                       |           BQ083829           |                               |
|                       |           BF914316           |                               |
|                       |           CA418903           |                               |
|                       |           CB854866           |                               |
|                       |           CA422365           |                               |
|                       |           BM670206           |                               |
|                       |           CA417304           |                               |
|                       |           BM976374           |                               |
|                       |           BQ448315           |                               |
|                       |           CA420564           |                               |
|                       |           BX098979           |                               |
|                       |           CA306622           |                               |
|                       |           BU729193           |                               |
|                       |           BM682031           |                               |
|                       |           AI745517           |                               |
|                       |             R43919           |                               |
|                       |           AA526699           |                               |
|                       |           BU737495           |                               |
|                       |           AI291429           |                               |
|                       |           AI760672           |                               |
|                       |           AI810976           |                               |
|                       |           AI291350           |                               |
|                       |           AI382209           |                               |
|                       |           AI681964           |                               |
|                       |           AI828575           |                               |
|                       |           BM973607           |                               |
|                       |           CN480311           |                               |
|                       |           BU630035           |                               |
|                       |           CA449445           |                               |
|                       |           CA431235           |                               |
|                       |           CB306223           |                               |
|                       |           AA635803           |                               |
|                       |           AA224332           |                               |
|                       |           AA635837           |                               |
|                       |           AA652370           |                               |
|                       |           AA618584           |                               |
|                       |           AJ573163           |                               |
|                       |           CB853675           |                               |
|                       |           BU735649           |                               |
|                       |           AI277302           |                               |
|                       |           AI925482           |                               |
|                       |           CN368425           |                               |
|                       | *Seq. mapped in strand Minus*| *Seq. mapped in strand Minus* |
|                       |           AA443363           |          BI646461             |
+-----------------------+------------------------------+-------------------------------+
|      #  134           | *Seq. mapped in strand Plus* | *Seq. mapped in strand Plus*  |
|                       |           CB113383           |          AA674362             |
|                       |             X02750           |                               |
|                       |           BI762861           |                               |
|                       |           BC034377           |                               |
|                       |           AL531077           |                               |
|                       |           CR606412           |                               |
|                       |           AV655828           |                               |
|                       |           CV029637           |                               |
|                       |           AV689626           |                               |
|                       |           AV698460           |                               |
|                       |             K02059           |                               |
|                       |           BI764225           |                               |
|                       |           AV684946           |                               |
|                       |             S72338           |                               |
|                       |           CB145040           |                               |
|                       |           CB145461           |                               |
|                       |           AL531076           |                               |
|                       |           CB128895           |                               |
|                       |           BF237847           |                               |
|                       |           BU607676           |                               |
|                       |           BX107014           |                               |
|                       |           BI252272           |                               |
|                       |           BQ646591           |                               |
|                       |           BM975043           |                               |
|                       |             S50739           |                               |
|                       | *Seq. mapped in strand Minus*| *Seq. mapped in strand Minus* |
|                       |           AI950004           |          AI893881             |
+-----------------------+------------------------------+-------------------------------+
|      #  135           | *Seq. mapped in strand Plus* | *Seq. mapped in strand Plus*  |
|                       |             Z22533           |            L48015             |
|                       |           AI400112           |          BY353394             |
|                       |           AI277722           |          BY135642             |
|                       |           AI660167           |          BY135627             |
|                       |           AI149062           |          BB869400             |
|                       |           AI740840           |          BB869983             |
|                       |           AI017380           |          BB873532             |
|                       |           BP302249           |          BC014291             |
|                       |           CR599504           |          BC015083             |
|                       |             L17075           |          BQ878861             |
|                       |           AL525277           |          BY342422             |
|                       |           BI822412           |          BI414304             |
|                       |           BI822484           |          BY339734             |
|                       |           BC042637           |          BB851123             |
|                       |             H43049           |          BY309317             |
|                       |             R72888           |          BY096360             |
|                       |           BI770269           |          BY311731             |
|                       |           CD607151           |          CK128491             |
|                       |           CD607152           |          BG175250             |
|                       |           CA406164           |          BF102257             |
|                       |             R73364           |          BY333729             |
|                       |           AW951319           |            Z31664             |
|                       |           CD367285           |          BY235445             |
|                       |             W24356           |          BY163632             |
|                       |           BX105121           |          BB868224             |
|                       |           AI799601           |          BY188719             |
|                       |           BU633341           |          BY718554             |
|                       |             T29378           |          AA124163             |
|                       |           CD367529           |          AA981801             |
|                       |           BU624496           |          AK018754             |
|                       |           BM971727           |          BE847168             |
|                       |           BU633072           |          AA518256             |
|                       |           BI523915           |          BX525966             |
|                       |           BU739570           |          BU517701             |
|                       |           BU621321           |          BE852128             |
|                       |           BM713543           |          BY466626             |
|                       |           BE467710           |          AW741856             |
|                       |           BM712769           |          BB759975             |
|                       |           BM726875           |          BY530131             |
|                       |           BM676112           |          BY373113             |
|                       |           BU741163           |          BY534607             |
|                       |           AW205372           |          BB798238             |
|                       |           AI871562           |          BB802574             |
|                       |           AI247327           |          BB806857             |
|                       |           BP346241           |          BB803177             |
|                       |           AI973111           |          BB806155             |
|                       |           AI420822           |          BX636620             |
|                       |           AI474440           |          BY511214             |
|                       |           AI685899           |          BB805289             |
|                       |           BF222616           |          BU755363             |
|                       |           BF510452           |                               |
|                       |           AI669541           |                               |
|                       |           AI659369           |                               |
|                       |           AI799845           |                               |
|                       |           AI804689           |                               |
|                       |           AI971112           |                               |
|                       |           AW292024           |                               |
|                       |           BF447241           |                               |
|                       |           BF447319           |                               |
|                       |           AW449875           |                               |
|                       |             N95334           |                               |
|                       |           AI686659           |                               |
|                       | *Seq. mapped in strand Minus*| *Seq. mapped in strand Minus* |
|                       |             H21874           |          AI586021             |
|                       |                              |          AA717195             |
|                       |                              |                               |
|                       |                              |                               |
|                       |                              |                               |
|                       |                              |                               |
|                       |                              |                               |
|                       |                              |                               |
|                       |                              |                               |
|                       |                              |                               |
|                       |                              |                               |
|                       |                              |                               |
|                       |                              |                               |
|                       |                              |                               |
|                       |                              |                               |
|                       |                              |                               |
|                       |                              |                               |
|                       |                              |                               |
|                       |                              |                               |
|                       |                              |                               |
|                       |                              |                               |
|                       |                              |                               |
|                       |                              |                               |
|                       |                              |                               |
|                       |                              |                               |
|                       |                              |                               |
|                       |                              |                               |
|                       |                              |                               |
|                       |                              |                               |
|                       |                              |                               |
|                       |                              |                               |
|                       |                              |                               |
|                       |                              |                               |
|                       |                              |                               |
|                       |                              |                               |
|                       |                              |                               |
|                       |                              |                               |
|                       |                              |                               |
|                       |                              |                               |
|                       |                              |                               |
|                       |                              |                               |
|                       |                              |                               |
|                       |                              |                               |
|                       |                              |                               |
|                       |                              |                               |
|                       |                              |                               |
|                       |                              |                               |
|                       |                              |                               |
|                       |                              |                               |
|                       |                              |                               |
+-----------------------+------------------------------+-------------------------------+
|      #  136           | *Seq. mapped in strand Plus* | *Seq. mapped in strand Plus*  |
|                       |           AA831980           |          AA119328             |
|                       |           AA687407           |          CF729059             |
|                       |                              |          AA756466             |
|                       |                              |          BF017323             |
|                       |                              |          BF783539             |
|                       |                              |          CA878877             |
|                       |                              |          AW457814             |
|                       |                              |          BB619152             |
|                       |                              |          BM963206             |
|                       |                              |          CN531634             |
|                       |                              |          AW321269             |
|                       |                              |          BE647686             |
|                       |                              |          AW214892             |
|                       |                              |          CA880290             |
|                       |                              |          CB236488             |
|                       |                              |          BC027013             |
|                       |                              |          BI696933             |
|                       |                              |          CA872155             |
|                       |                              |          BE137262             |
|                       |                              |          BG923172             |
|                       |                              |          BF720391             |
|                       |                              |          CN527857             |
|                       |                              |          BG083487             |
|                       |                              |          BP428307             |
|                       |                              |          AL363670             |
|                       |                              |          BF471424             |
|                       |                              |          BG295942             |
|                       |                              |          BU514811             |
|                       |                              |          BB835681             |
|                       | *Seq. mapped in strand Minus*| *Seq. mapped in strand Minus* |
|                       |           AB028996           |          BB452665             |
|                       |           AI354370           |          BQ030910             |
|                       |           AA938916           |          BB747628             |
|                       |             R46367           |          BB760072             |
|                       |           AI880015           |          BY589688             |
|                       |           CA416208           |          BB833049             |
|                       |           BX647997           |                               |
|                       |           AA830351           |                               |
|                       |           CR744896           |                               |
|                       |           AI762425           |                               |
|                       |           BQ012764           |                               |
|                       |           BU683583           |                               |
|                       |           BU739506           |                               |
|                       |           AW206479           |                               |
|                       |           BC012086           |                               |
|                       |           AI950612           |                               |
|                       |           AI080098           |                               |
|                       |           AA706542           |                               |
|                       |           BG034498           |                               |
|                       |           AI935013           |                               |
|                       |           CA439299           |                               |
|                       |           BF109923           |                               |
|                       |           AK027038           |                               |
|                       |           BC040432           |                               |
|                       |           BC034474           |                               |
|                       |           CB306764           |                               |
|                       |           BG164863           |                               |
|                       |           BE535354           |                               |
|                       |             U58033           |                               |
|                       |           BI223918           |                               |
|                       |           AW138456           |                               |
|                       |           AI634730           |                               |
|                       |           CA446130           |                               |
|                       |           CD365842           |                               |
|                       |           BG034702           |                               |
|                       |           BM753519           |                               |
|                       |           AW950451           |                               |
|                       |           AL527507           |                               |
|                       |           BM463164           |                               |
|                       |             R66174           |                               |
|                       |           BC052990           |                               |
|                       |           BM676202           |                               |
|                       |           BG190335           |                               |
|                       |           BG166656           |                               |
|                       |           CD106964           |                               |
|                       |           BM770601           |                               |
|                       |           BQ229241           |                               |
|                       |           BP206785           |                               |
|                       |           BM770252           |                               |
|                       |           BM741892           |                               |
|                       |           BM770502           |                               |
|                       |           CD106875           |                               |
|                       |           BM771076           |                               |
|                       |           BE795554           |                               |
|                       |           BM750739           |                               |
|                       |           BM766907           |                               |
|                       |           AA329879           |                               |
|                       |           BM751797           |                               |
|                       |           AA471351           |                               |
|                       |             F11352           |                               |
|                       |           BE869818           |                               |
|                       |           BM750750           |                               |
|                       |           AA569789           |                               |
|                       |           BE047636           |                               |
|                       |           AL601200           |                               |
|                       |           AA551423           |                               |
|                       |           CD512947           |                               |
|                       |           BF088335           |                               |
|                       |           BF155534           |                               |
|                       |           AL601189           |                               |
|                       |           BE926085           |                               |
|                       |           BG573442           |                               |
|                       |           BM713476           |                               |
|                       |           BM713008           |                               |
|                       |           BX328973           |                               |
|                       |           BP383376           |                               |
|                       |           BE785597           |                               |
|                       |           AV757506           |                               |
|                       |           AW505463           |                               |
|                       |           BP240667           |                               |
|                       |           BF665231           |                               |
|                       |           BG507100           |                               |
|                       |           BI861147           |                               |
|                       |           BM806457           |                               |
|                       |           BE958459           |                               |
|                       |           AW605839           |                               |
|                       |           BE768089           |                               |
|                       |           BF371857           |                               |
|                       |           BE148525           |                               |
|                       |           BE148529           |                               |
|                       |           BE148663           |                               |
|                       |           AW605841           |                               |
|                       |           CB122513           |                               |
|                       |           BX348246           |                               |
|                       |           BG911681           |                               |
|                       |           BP337893           |                               |
|                       |           BP337940           |                               |
|                       |           BP241556           |                               |
|                       |           BP194184           |                               |
|                       |           CD518351           |                               |
|                       |           BU507705           |                               |
|                       |           BM478924           |                               |
|                       |           BG574577           |                               |
|                       |           AI815715           |                               |
|                       |           CN362821           |                               |
|                       |           BM475570           |                               |
|                       |           CF994319           |                               |
|                       |           BP349296           |                               |
|                       |           BI560486           |                               |
|                       |           BE784072           |                               |
|                       |           BI462183           |                               |
|                       |           BG164822           |                               |
|                       |           CK000761           |                               |
|                       |           BG392303           |                               |
|                       |           BE545482           |                               |
|                       |           BP352495           |                               |
|                       |           CD109429           |                               |
|                       |           BP234455           |                               |
|                       |           CN362823           |                               |
|                       |           BP356992           |                               |
|                       |           AL527508           |                               |
|                       |           BP219665           |                               |
|                       |           BP219684           |                               |
|                       |           BG722215           |                               |
|                       |           AL601605           |                               |
|                       |           AL705005           |                               |
|                       |           BG720186           |                               |
|                       |           BP291064           |                               |
|                       |           BU193225           |                               |
|                       |           BG723167           |                               |
|                       |           BP315445           |                               |
|                       |           BG337752           |                               |
|                       |           CN362820           |                               |
|                       |           CF137994           |                               |
|                       |           BP358035           |                               |
|                       |           BP238160           |                               |
|                       |           BP361172           |                               |
+-----------------------+------------------------------+-------------------------------+
|      #  137           | *Seq. mapped in strand Plus* | *Seq. mapped in strand Plus*  |
|                       |           AK127449           |          AK042429             |
|                       |           AB045116           |                               |
|                       |           AB045117           |                               |
|                       |           AA358111           |                               |
|                       |           AA453573           |                               |
|                       |           BX095122           |                               |
|                       |           BQ635937           |                               |
|                       |           BM925940           |                               |
|                       |           BI759211           |                               |
|                       |           BX642957           |                               |
|                       |           BM700622           |                               |
|                       |           BF889218           |                               |
|                       |           AF028701           |                               |
|                       |           BX463326           |                               |
|                       |           BI824471           |                               |
|                       | *Seq. mapped in strand Minus*| *Seq. mapped in strand Minus* |
|                       |           BU680978           |          AK035653             |
+-----------------------+------------------------------+-------------------------------+
|      #  138           | *Seq. mapped in strand Plus* | *Seq. mapped in strand Plus*  |
|                       |           BC063294           |          AK077896             |
|                       |             M93718           |            U53142             |
|                       |             M95296           |          BC052636             |
|                       |           BC069465           |                               |
|                       |           AF400594           |                               |
|                       |             S80791           |                               |
|                       |           CR596138           |                               |
|                       | *Seq. mapped in strand Minus*| *Seq. mapped in strand Minus* |
|                       |           AY316116           |          AK010032             |
|                       |           AY515311           |                               |
|                       |           AK027791           |                               |
|                       |           AK096734           |                               |
|                       |           CR607530           |                               |
|                       |           CR613491           |                               |
+-----------------------+------------------------------+-------------------------------+
|      #  139           | *Seq. mapped in strand Plus* | *Seq. mapped in strand Plus*  |
|                       |           AA669689           |          AK039418             |
|                       |                              |          AK046134             |
|                       | *Seq. mapped in strand Minus*| *Seq. mapped in strand Minus* |
|                       |           CR591849           |            M26756             |
|                       |           AJ420574           |          AK077968             |
|                       |           CA445613           |            J02652             |
|                       |             R39955           |          AK004980             |
|                       |           BG191640           |          BC003287             |
|                       |           BG192549           |          BC011081             |
|                       |           BG185237           |          AK006387             |
|                       |           BG218033           |                               |
|                       |           BG212740           |                               |
|                       |           BG209639           |                               |
|                       |           BG211654           |                               |
|                       |           BG202845           |                               |
|                       |             H06037           |                               |
|                       |             R60023           |                               |
|                       |           BG213275           |                               |
|                       |           BG193608           |                               |
|                       |           BG200860           |                               |
|                       |           BG201243           |                               |
|                       |           BG204336           |                               |
|                       |           BG186296           |                               |
|                       |           BG197690           |                               |
|                       |           BG214901           |                               |
|                       |           BG203326           |                               |
|                       |           BG190008           |                               |
|                       |           BG184584           |                               |
|                       |           BG196138           |                               |
|                       |           BG211657           |                               |
|                       |           BG216360           |                               |
|                       |           BG182613           |                               |
|                       |           BG210035           |                               |
|                       |           BG183117           |                               |
|                       |           BG193073           |                               |
|                       |           BG206500           |                               |
|                       |           BG187593           |                               |
|                       |           BG193072           |                               |
|                       |           BG205483           |                               |
|                       |           BG209641           |                               |
|                       |           BG211158           |                               |
|                       |           BG212244           |                               |
|                       |           BG217795           |                               |
|                       |           BG218951           |                               |
|                       |           BG184169           |                               |
|                       |           AI978721           |                               |
|                       |           BU616849           |                               |
|                       |           BC017403           |                               |
|                       |             U43944           |                               |
|                       |           BQ631261           |                               |
|                       |           BG188381           |                               |
|                       |           BG201140           |                               |
|                       |           BG192044           |                               |
|                       |           BG201272           |                               |
|                       |           BG203358           |                               |
|                       |           BG210106           |                               |
|                       |           BQ631537           |                               |
|                       |           AI366847           |                               |
|                       |           AI339685           |                               |
|                       |           BG203799           |                               |
|                       |           BG196641           |                               |
|                       |           CD108416           |                               |
|                       |           BG194601           |                               |
|                       |           BG204869           |                               |
|                       |           BG188380           |                               |
|                       |           BG210560           |                               |
|                       |           BM676971           |                               |
|                       |           BG192548           |                               |
|                       |           BG035312           |                               |
|                       |           BG199214           |                               |
|                       |           BG209640           |                               |
|                       |           BG221514           |                               |
|                       |           BG207513           |                               |
|                       |           BG184583           |                               |
|                       |           BG193074           |                               |
|                       |           BQ128254           |                               |
|                       |           BG184170           |                               |
|                       |             X77244           |                               |
|                       |           BG215401           |                               |
|                       |           BG192093           |                               |
|                       |           BG195143           |                               |
|                       |           BG215980           |                               |
|                       |           BG217049           |                               |
|                       |           BG200255           |                               |
|                       |           BG196173           |                               |
|                       |           BG196642           |                               |
|                       |           BG205482           |                               |
|                       |           BG211656           |                               |
|                       |           BG221513           |                               |
|                       |           BG110590           |                               |
|                       |           CR601714           |                               |
|                       |           AL528588           |                               |
|                       |           BG220059           |                               |
|                       |           BF967984           |                               |
|                       |           BG213771           |                               |
|                       |           BG215400           |                               |
|                       |           BP225648           |                               |
|                       |           BG206499           |                               |
|                       |           BG186820           |                               |
|                       |           AI217952           |                               |
|                       |           BG193610           |                               |
|                       |           BG198243           |                               |
|                       |           BG189447           |                               |
|                       |           BG215402           |                               |
|                       |           AI739394           |                               |
|                       |           BG187327           |                               |
|                       |           BG211655           |                               |
|                       |           CA441611           |                               |
|                       |           BC025246           |                               |
|                       |           AI971510           |                               |
|                       |           CA420693           |                               |
|                       |           CB157765           |                               |
|                       |           BG185747           |                               |
|                       |           BG199701           |                               |
|                       |           BG221018           |                               |
|                       |           CK005353           |                               |
|                       |           BG213770           |                               |
|                       |           BG698479           |                               |
|                       |           BP432726           |                               |
|                       |           BG193609           |                               |
|                       |           BG204868           |                               |
|                       |           AV682650           |                               |
|                       |           BG199739           |                               |
|                       |           BF577185           |                               |
|                       |           BG196643           |                               |
|                       |           BG219572           |                               |
|                       |           BG186936           |                               |
|                       |           BF695089           |                               |
|                       |           CB160273           |                               |
|                       |           BU181299           |                               |
|                       |           BG291677           |                               |
|                       |           BG215570           |                               |
|                       |           AI798687           |                               |
|                       |           BX348738           |                               |
|                       |           CB143689           |                               |
|                       |           CD106324           |                               |
|                       |           CA867156           |                               |
|                       |           BF130406           |                               |
|                       |           BG185939           |                               |
|                       |           AA855071           |                               |
|                       |           CB148779           |                               |
|                       |           CD107900           |                               |
|                       |           BF208881           |                               |
|                       |           CB120022           |                               |
|                       |           BX371460           |                               |
|                       |           BX371459           |                               |
|                       |           CB163735           |                               |
|                       |           BG696063           |                               |
|                       |           BX488025           |                               |
|                       |           BG743725           |                               |
|                       |           BI914712           |                               |
|                       |             F05866           |                               |
|                       |           AL528589           |                               |
|                       |           AA443222           |                               |
|                       |           BX376125           |                               |
|                       |             D56166           |                               |
|                       |           BF568564           |                               |
|                       |           BP278155           |                               |
|                       |           BI192700           |                               |
|                       |           BG764703           |                               |
|                       |           BF967261           |                               |
|                       |           AI857682           |                               |
|                       |           CN266511           |                               |
|                       |           BP195390           |                               |
|                       |           BG767364           |                               |
|                       |           BP212653           |                               |
|                       |           BI458819           |                               |
|                       |           CN266512           |                               |
|                       |           BI668963           |                               |
|                       |           BQ214597           |                               |
|                       |           CD557494           |                               |
|                       |           CB118929           |                               |
|                       |           BP210420           |                               |
|                       |           BE206224           |                               |
|                       |           CA942451           |                               |
|                       |           BI544786           |                               |
|                       |           BP221078           |                               |
|                       |           BP282072           |                               |
|                       |           BP252365           |                               |
|                       |           BP259314           |                               |
|                       |           BP219581           |                               |
|                       |           AW662146           |                               |
|                       |           AU279729           |                               |
|                       |           BP203271           |                               |
|                       |           AW002307           |                               |
|                       |           BQ127991           |                               |
|                       |           AL041987           |                               |
+-----------------------+------------------------------+-------------------------------+
|      #  140           | *Seq. mapped in strand Plus* | *Seq. mapped in strand Plus*  |
|                       |             U97276           |          BC054066             |
|                       |           AY358941           |                               |
|                       |           BC017692           |                               |
|                       |           AF361868           |                               |
|                       |           CR613263           |                               |
|                       |           CR619667           |                               |
|                       |           CR601148           |                               |
|                       |           CR597598           |                               |
|                       |           AL832927           |                               |
|                       |           AK074447           |                               |
|                       | *Seq. mapped in strand Minus*| *Seq. mapped in strand Minus* |
|                       |           BC067355           |          AK009444             |
+-----------------------+------------------------------+-------------------------------+
|      #  141           | *Seq. mapped in strand Plus* | *Seq. mapped in strand Plus*  |
|                       |           AB028967           |          AK032268             |
|                       |           AJ010969           |          AB045326             |
|                       |           BF792906           |          AB093280             |
|                       |           BG196870           |          AF107780             |
|                       |           BG205115           |          BB623231             |
|                       |           CN428603           |          AK032772             |
|                       |                              |          AV340249             |
|                       |                              |          AV329720             |
|                       |                              |          CB235756             |
|                       |                              |          AW493525             |
|                       |                              |          BQ176762             |
|                       |                              |          AW045320             |
|                       |                              |          AW493035             |
|                       | *Seq. mapped in strand Minus*| *Seq. mapped in strand Minus* |
|                       |           BG206619           |          BE944629             |
|                       |           AI732822           |                               |
|                       |           BG201891           |                               |
|                       |           AA485445           |                               |
+-----------------------+------------------------------+-------------------------------+
|      #  142           | *Seq. mapped in strand Plus* | *Seq. mapped in strand Plus*  |
|                       |           BX116401           |          AK035433             |
|                       | *Seq. mapped in strand Minus*| *Seq. mapped in strand Minus* |
|                       |           AF038852           |          AK035905             |
|                       |           AY054985           |          AK046400             |
|                       |             U95020           |          AK014145             |
|                       |           BC075049           |                               |
|                       |           AW134825           |                               |
|                       |           BP219438           |                               |
|                       |           BP229182           |                               |
+-----------------------+------------------------------+-------------------------------+
|      #  143           | *Seq. mapped in strand Plus* | *Seq. mapped in strand Plus*  |
|                       |           BE835389           |          AK085566             |
|                       |                              |          AB093278             |
|                       |                              |          AK046017             |
|                       |                              |          BC035276             |
|                       |                              |          AK009255             |
|                       |                              |          AK031470             |
|                       |                              |          BC030360             |
|                       |                              |          AK028083             |
|                       | *Seq. mapped in strand Minus*| *Seq. mapped in strand Minus* |
|                       |           AI280556           |            M32745             |
|                       |           BM972030           |          BC014690             |
|                       |           AA778162           |          BC005513             |
|                       |           BM972711           |                               |
|                       |           BM677003           |                               |
|                       |           BX370149           |                               |
|                       |           AW439570           |                               |
|                       |           BM674666           |                               |
|                       |           BF940264           |                               |
|                       |           BQ006716           |                               |
|                       |           AA993573           |                               |
|                       |           AK122902           |                               |
|                       |             X14149           |                               |
|                       |           AA557172           |                               |
|                       |           BQ130448           |                               |
|                       |             W92741           |                               |
|                       |           AI973075           |                               |
|                       |           BM981373           |                               |
|                       |           AW015841           |                               |
|                       |             R80106           |                               |
|                       |           BQ030450           |                               |
|                       |           BQ002666           |                               |
|                       |           CA413302           |                               |
|                       |             J03241           |                               |
|                       |           BU623981           |                               |
|                       |           CR611616           |                               |
|                       |           CA454327           |                               |
|                       |           CD671877           |                               |
|                       |           AL532438           |                               |
|                       |           BX645476           |                               |
|                       |           BI043391           |                               |
|                       |           CA392865           |                               |
|                       |           BE157967           |                               |
|                       |           BC018503           |                               |
|                       |           BT007287           |                               |
|                       |           BX336094           |                               |
|                       |           BI057300           |                               |
|                       |           BQ130704           |                               |
|                       |           BU151315           |                               |
|                       |           AA579057           |                               |
|                       |             F08314           |                               |
|                       |           BG254479           |                               |
|                       |           BQ926104           |                               |
|                       |           BM804677           |                               |
|                       |           BI771985           |                               |
+-----------------------+------------------------------+-------------------------------+
|      #  144           | *Seq. mapped in strand Plus* | *Seq. mapped in strand Plus*  |
|                       |           BC000986           |            L42115             |
|                       |                              |            D85044             |
|                       |                              |          BC037462             |
|                       |                              |          BC029873             |
|                       |                              |          BI645711             |
|                       |                              |          AF246130             |
|                       |                              |          BI659726             |
|                       |                              |          BI904837             |
|                       |                              |          BY235338             |
|                       |                              |          BI078731             |
|                       |                              |          AA423214             |
|                       |                              |          CA563614             |
|                       |                              |          CA562554             |
|                       |                              |          BE286953             |
|                       |                              |          BG867465             |
|                       |                              |          BG866824             |
|                       |                              |          BG864955             |
|                       |                              |          BG864868             |
|                       |                              |          BM947262             |
|                       |                              |          BE289069             |
|                       |                              |          BF580715             |
|                       |                              |          CO045878             |
|                       |                              |          AA727697             |
|                       |                              |          BF535591             |
|                       |                              |          BF139342             |
|                       |                              |          AA738833             |
|                       |                              |          CK625888             |
|                       |                              |          AA759728             |
|                       |                              |          BM937843             |
|                       |                              |          AA450735             |
|                       |                              |          BE692569             |
|                       |                              |          BI986226             |
|                       |                              |          CB321357             |
|                       |                              |          BF536958             |
|                       |                              |          CF982742             |
|                       |                              |          BY104914             |
|                       |                              |          CB195152             |
|                       |                              |          BY054599             |
|                       |                              |          BY041328             |
|                       |                              |          BY053054             |
|                       |                              |          BY051648             |
|                       |                              |          BY044968             |
|                       |                              |          BY042976             |
|                       |                              |          BY050246             |
|                       |                              |          AI036834             |
|                       |                              |          AA163783             |
|                       |                              |          AA563426             |
|                       |                              |          BG868164             |
|                       |                              |          CF585639             |
|                       |                              |          BI792519             |
|                       |                              |          AA739280             |
|                       |                              |          AI021389             |
|                       |                              |          AA182368             |
|                       | *Seq. mapped in strand Minus*| *Seq. mapped in strand Minus* |
|                       |           AK055630           |          AI315946             |
|                       |           AF334818           |                               |
|                       |           AF102826           |                               |
|                       |           BC000062           |                               |
|                       |           AK125222           |                               |
|                       |           AF105423           |                               |
|                       |             U53347           |                               |
|                       |           CR618074           |                               |
|                       |           AF105230           |                               |
+-----------------------+------------------------------+-------------------------------+
|      #  145           | *Seq. mapped in strand Plus* | *Seq. mapped in strand Plus*  |
|                       |           AF230336           |          BC040776             |
|                       |           AF340183           |          BC042619             |
|                       |           BC038666           |          BC042870             |
|                       |           BC013582           |                               |
|                       |           AK022613           |                               |
|                       |           AL832427           |                               |
|                       |           AK074406           |                               |
|                       | *Seq. mapped in strand Minus*| *Seq. mapped in strand Minus* |
|                       |           BC028122           |          AK016461             |
|                       |           AF230334           |          BC049660             |
|                       |           AY248899           |          BC042706             |
+-----------------------+------------------------------+-------------------------------+
|      #  146           | *Seq. mapped in strand Plus* | *Seq. mapped in strand Plus*  |
|                       |           BC004988           |          AK129000             |
|                       |           AK074232           |          AK034412             |
|                       |           AY356352           |          BC054382             |
|                       |           AJ311359           |          AK080978             |
|                       |           AL365408           |          BC009161             |
|                       |           CR624347           |          AK005041             |
|                       |           AL359589           |          AK009354             |
|                       |           CR749732           |          AK075860             |
|                       |           CR590695           |          AW046536             |
|                       |           CA308500           |          AA444403             |
|                       |           BQ014723           |          BF226364             |
|                       |           CA313072           |          BF227157             |
|                       |           BM982236           |          AW050265             |
|                       |           CA430502           |          AI836048             |
|                       |           BU619053           |          BE994178             |
|                       |           BQ774161           |                               |
|                       |           BU617995           |                               |
|                       |           BQ776328           |                               |
|                       |           BQ775035           |                               |
|                       |           BM968171           |                               |
|                       |           BM981659           |                               |
|                       |           BM980798           |                               |
|                       |           CA422658           |                               |
|                       |           BM671075           |                               |
|                       |           BU616772           |                               |
|                       |           BM680750           |                               |
|                       |           BU681419           |                               |
|                       |           BU742631           |                               |
|                       |           CB305389           |                               |
|                       |           BM665215           |                               |
|                       |           BM992082           |                               |
|                       |           AI972907           |                               |
|                       |           AW006190           |                               |
|                       |           AI937074           |                               |
|                       |           AA854164           |                               |
|                       |           AI564706           |                               |
|                       |           CF529953           |                               |
|                       |           BE302342           |                               |
|                       |           AW263020           |                               |
|                       |             W49528           |                               |
|                       |           AA603668           |                               |
|                       |             H06279           |                               |
|                       |             R59631           |                               |
|                       |           AA668787           |                               |
|                       |           AI076244           |                               |
|                       |           AI081111           |                               |
|                       |           AA603080           |                               |
|                       |           AA782406           |                               |
|                       |           BU677539           |                               |
|                       |           AI473591           |                               |
|                       |           BU741759           |                               |
|                       | *Seq. mapped in strand Minus*| *Seq. mapped in strand Minus* |
|                       |           CA311661           |          BF455796             |
|                       |           AA731842           |                               |
+-----------------------+------------------------------+-------------------------------+
|      #  147           | *Seq. mapped in strand Plus* | *Seq. mapped in strand Plus*  |
|                       |           CD700089           |          BI133906             |
|                       |           AU130529           |                               |
|                       |           AU100282           |                               |
|                       |           BM834479           |                               |
|                       |           BP362226           |                               |
|                       |           BP282300           |                               |
|                       |           BP196568           |                               |
|                       |           BP380955           |                               |
|                       |           CF780800           |                               |
|                       |           BG332681           |                               |
|                       |           BI253747           |                               |
|                       |           CN255494           |                               |
|                       |           BQ232139           |                               |
|                       |           BM472821           |                               |
|                       |           CF141384           |                               |
|                       |           BC065245           |                               |
|                       |           CK431064           |                               |
|                       |           BI837425           |                               |
|                       |           BU176732           |                               |
|                       |           BF314188           |                               |
|                       |           BI197931           |                               |
|                       |           BM854073           |                               |
|                       |             M94065           |                               |
|                       |           CB121224           |                               |
|                       |           BF434819           |                               |
|                       |           BQ070891           |                               |
|                       |           BQ216447           |                               |
|                       |           BM465205           |                               |
|                       |           AA173122           |                               |
|                       |           BU150925           |                               |
|                       |           BM793484           |                               |
|                       |           BG024665           |                               |
|                       |           AL547598           |                               |
|                       |           CR593960           |                               |
|                       |           BI830262           |                               |
|                       |           BQ919039           |                               |
|                       |           BE312892           |                               |
|                       |           BU553197           |                               |
|                       |           BU845147           |                               |
|                       |           BU859158           |                               |
|                       |           CN255497           |                               |
|                       |           CN255495           |                               |
|                       |           CN255496           |                               |
|                       |           BM795077           |                               |
|                       |           AW006026           |                               |
|                       |           BG479315           |                               |
|                       |           CA314529           |                               |
|                       |           BE902847           |                               |
|                       |           AK097300           |                               |
|                       |             T28623           |                               |
|                       |           AL572486           |                               |
|                       |           CN255498           |                               |
|                       |           BQ880089           |                               |
|                       |           AL525420           |                               |
|                       |           AI491891           |                               |
|                       |           BM770899           |                               |
|                       |           BM756358           |                               |
|                       |           BU069561           |                               |
|                       | *Seq. mapped in strand Minus*| *Seq. mapped in strand Minus* |
|                       |           AI553824           |          AK030798             |
+-----------------------+------------------------------+-------------------------------+
|      #  148           | *Seq. mapped in strand Plus* | *Seq. mapped in strand Plus*  |
|                       |           CR622317           |          BC035549             |
|                       |             L07633           |          AK019390             |
|                       |           BC000352           |            D87909             |
|                       |           BC007503           |          AK008514             |
|                       |           CR594470           |            U60328             |
|                       |           CR593193           |                               |
|                       |           CR541658           |                               |
|                       |           CR456780           |                               |
|                       |           BX248293           |                               |
|                       |           CR614004           |                               |
|                       | *Seq. mapped in strand Minus*| *Seq. mapped in strand Minus* |
|                       |           AF151870           |          AB054001             |
|                       |           BC002491           |          BC024704             |
|                       |           AY368908           |          AK005154             |
+-----------------------+------------------------------+-------------------------------+
|      #  149           | *Seq. mapped in strand Plus* | *Seq. mapped in strand Plus*  |
|                       |           AW292992           |          BC035278             |
|                       |                              |          CN699770             |
|                       |                              |          CN691780             |
|                       |                              |          BQ771276             |
|                       |                              |          CA557860             |
|                       |                              |          CF892938             |
|                       |                              |          CD542633             |
|                       |                              |          BU055417             |
|                       |                              |          AA990290             |
|                       |                              |          BB627471             |
|                       |                              |          AK035188             |
|                       |                              |          AW909835             |
|                       |                              |          BX512215             |
|                       |                              |          AA967153             |
|                       |                              |          BG082581             |
|                       | *Seq. mapped in strand Minus*| *Seq. mapped in strand Minus* |
|                       |           AJ708523           |          BE987760             |
|                       |           BM661564           |          AW124363             |
|                       |           AA769310           |          BE956541             |
|                       |           BM971315           |                               |
|                       |           CA449582           |                               |
|                       |           BM459496           |                               |
|                       |           AY007102           |                               |
|                       |           AK023226           |                               |
|                       |           AF392454           |                               |
|                       |           BC010422           |                               |
|                       |           BC065213           |                               |
|                       |           CR613218           |                               |
|                       |           AA169886           |                               |
|                       |           AA286844           |                               |
|                       |           BG216892           |                               |
|                       |           AK023074           |                               |
|                       |           BG181457           |                               |
|                       |           BG208087           |                               |
|                       |           CA425413           |                               |
|                       |           BG997599           |                               |
|                       |           BP293758           |                               |
|                       |           CB164284           |                               |
|                       |           BQ217555           |                               |
|                       |           AA026021           |                               |
|                       |           BF081591           |                               |
|                       |           BF208277           |                               |
|                       |           BM729033           |                               |
|                       |           BG621460           |                               |
|                       |           CA866364           |                               |
|                       |           BQ416966           |                               |
|                       |           AA286843           |                               |
|                       |           AA251918           |                               |
|                       |           BP216644           |                               |
|                       |           BG197710           |                               |
|                       |           BG288063           |                               |
|                       |           BF736545           |                               |
|                       |           BE170545           |                               |
|                       |           CN345607           |                               |
|                       |           AW795854           |                               |
|                       |           BX501055           |                               |
|                       |           CA842219           |                               |
|                       |           AA169573           |                               |
|                       |           CD631588           |                               |
|                       |           CD631589           |                               |
|                       |           CV030075           |                               |
|                       |           AU130298           |                               |
|                       |           BM680399           |                               |
|                       |           AU132028           |                               |
|                       |           BF900889           |                               |
|                       |           CN345608           |                               |
|                       |           BF904822           |                               |
|                       |           BE299160           |                               |
|                       |           CB990530           |                               |
|                       |           CN345609           |                               |
|                       |           BX280695           |                               |
|                       |           BF892050           |                               |
|                       |           AF346292           |                               |
|                       |           BU429574           |                               |
|                       |           CD643358           |                               |
|                       |           AW500582           |                               |
|                       |           BF326880           |                               |
|                       |           BI023717           |                               |
|                       |           CN345611           |                               |
|                       |           AL709163           |                               |
|                       |           CA307380           |                               |
|                       |           BE095224           |                               |
|                       |           BP336382           |                               |
|                       |           BE622358           |                               |
|                       |           BM724261           |                               |
|                       |           BP229887           |                               |
|                       |           CN345610           |                               |
|                       |           BX506747           |                               |
|                       |           BU521850           |                               |
+-----------------------+------------------------------+-------------------------------+
|      #  150           | *Seq. mapped in strand Plus* | *Seq. mapped in strand Plus*  |
|                       |           AJ222801           |          AK053968             |
|                       |           BC000038           |                               |
|                       |           CR591613           |                               |
|                       | *Seq. mapped in strand Minus*| *Seq. mapped in strand Minus* |
|                       |           BC026359           |          BC010978             |
+-----------------------+------------------------------+-------------------------------+
|      #  151           | *Seq. mapped in strand Plus* | *Seq. mapped in strand Plus*  |
|                       |           AL133111           |          AB028640             |
|                       |                              |          AJ012475             |
|                       | *Seq. mapped in strand Minus*| *Seq. mapped in strand Minus* |
|                       |           BX641159           |          AK043375             |
|                       |           BC023195           |          BC053741             |
|                       |           AB005047           |          BC018237             |
|                       |           AL117422           |          AB016835             |
|                       |           CR601004           |                               |
|                       |           CR592833           |                               |
|                       |           BC010123           |                               |
|                       |           AK090524           |                               |
|                       |           BC006169           |                               |
+-----------------------+------------------------------+-------------------------------+
|      #  152           | *Seq. mapped in strand Plus* | *Seq. mapped in strand Plus*  |
|                       |           AJ002511           |          AK043675             |
|                       |           CD610385           |                               |
|                       |             Z30126           |                               |
|                       |             Z32832           |                               |
|                       |           BX644992           |                               |
|                       |           AL600994           |                               |
|                       |             T94696           |                               |
|                       |             F05904           |                               |
|                       |             F12039           |                               |
|                       |             F05905           |                               |
|                       |             T65667           |                               |
|                       |             N44993           |                               |
|                       | *Seq. mapped in strand Minus*| *Seq. mapped in strand Minus* |
|                       |           AI004147           |          AF295105             |
+-----------------------+------------------------------+-------------------------------+
|      #  153           | *Seq. mapped in strand Plus* | *Seq. mapped in strand Plus*  |
|                       |           AB024964           |          AK052449             |
|                       |           AB026048           |            Y10926             |
|                       |           AB027710           |                               |
|                       | *Seq. mapped in strand Minus*| *Seq. mapped in strand Minus* |
|                       |           AK027323           |          AK041073             |
|                       |           AK074595           |          BC036558             |
|                       |           AK074597           |          AJ006140             |
|                       |           AK074495           |                               |
|                       |           AK074593           |                               |
|                       |           AJ007581           |                               |
+-----------------------+------------------------------+-------------------------------+
|      #  154           | *Seq. mapped in strand Plus* | *Seq. mapped in strand Plus*  |
|                       |             M74715           |          AK031414             |
|                       |           AK125223           |          AK045330             |
|                       |           BC029959           |          BC057935             |
|                       |           AK098805           |            L34111             |
|                       | *Seq. mapped in strand Minus*| *Seq. mapped in strand Minus* |
|                       |           BC015517           |          AY093420             |
|                       |           AY124771           |          AK128888             |
|                       |           AF297659           |          BC025824             |
|                       |                              |          BC022130             |
+-----------------------+------------------------------+-------------------------------+
|      #  155           | *Seq. mapped in strand Plus* | *Seq. mapped in strand Plus*  |
|                       |           BC026187           |          AK006754             |
|                       |           AK097456           |          AK049116             |
|                       |           AL833052           |          BC006866             |
|                       |                              |          AK007245             |
|                       |                              |          AK014884             |
|                       | *Seq. mapped in strand Minus*| *Seq. mapped in strand Minus* |
|                       |           BC010276           |          BC005770             |
|                       |           BX647788           |          AK083800             |
|                       |           AF139131           |          AK050329             |
|                       |             L38932           |          AF077302             |
|                       |           AK130396           |          BC027738             |
|                       |           AF077301           |                               |
+-----------------------+------------------------------+-------------------------------+
|      #  156           | *Seq. mapped in strand Plus* | *Seq. mapped in strand Plus*  |
|                       |           CA314493           |          AK009156             |
|                       |                              |          BC027639             |
|                       | *Seq. mapped in strand Minus*| *Seq. mapped in strand Minus* |
|                       |           AA878857           |          AK128941             |
|                       |           CK300225           |          BC056448             |
|                       |           CK300004           |          AF006688             |
|                       |             W44745           |          BC054727             |
|                       |           AW024365           |          AB034914             |
|                       |           CK299942           |                               |
|                       |           BU619300           |                               |
|                       |           BX100495           |                               |
|                       |           BU616842           |                               |
|                       |           AK095501           |                               |
|                       |           CR595778           |                               |
+-----------------------+------------------------------+-------------------------------+
|      #  157           | *Seq. mapped in strand Plus* | *Seq. mapped in strand Plus*  |
|                       |           BI548110           |          AF513714             |
|                       |           BI462536           |          AK129292             |
|                       |           BP308393           |          BC006917             |
|                       |           CO250170           |          AK009313             |
|                       |           BC029138           |                               |
|                       |           BG719283           |                               |
|                       |           BG708229           |                               |
|                       |           BG720435           |                               |
|                       |           BI562347           |                               |
|                       |           BI460520           |                               |
|                       |           BI463758           |                               |
|                       |           BI907246           |                               |
|                       |           BG708030           |                               |
|                       |           BU151624           |                               |
|                       |           BI832423           |                               |
|                       |           CR593800           |                               |
|                       |           BG705291           |                               |
|                       |           BG752552           |                               |
|                       |           CD050064           |                               |
|                       |           BG700579           |                               |
|                       |           BU931106           |                               |
|                       |           BG714584           |                               |
|                       |           BI561714           |                               |
|                       |           BG724240           |                               |
|                       |           BG721136           |                               |
|                       |           BI597953           |                               |
|                       |           BG708336           |                               |
|                       |           BI561520           |                               |
|                       |           BG715656           |                               |
|                       |           BG721085           |                               |
|                       |           BG773306           |                               |
|                       |           BG714731           |                               |
|                       |           BI560938           |                               |
|                       |           BI561275           |                               |
|                       |           BG721346           |                               |
|                       |           BG706276           |                               |
|                       |           BG714198           |                               |
|                       |           BI598254           |                               |
|                       |           BQ926946           |                               |
|                       |           CB992293           |                               |
|                       |           CB995518           |                               |
|                       |           BG766945           |                               |
|                       |           BM194282           |                               |
|                       |           BM148125           |                               |
|                       |           BP280377           |                               |
|                       |           BP201176           |                               |
|                       |           BP283588           |                               |
|                       |           BP357729           |                               |
|                       |           BP229757           |                               |
|                       |           BP358650           |                               |
|                       |           BP359391           |                               |
|                       |           BP364002           |                               |
|                       |           BP365220           |                               |
|                       |           BP358622           |                               |
|                       |           BP284042           |                               |
|                       |           BP363644           |                               |
|                       |           BP280237           |                               |
|                       |           BP199100           |                               |
|                       |           BI465071           |                               |
|                       |           BM806170           |                               |
|                       |           BU186674           |                               |
|                       |           BQ955164           |                               |
|                       |           BG753444           |                               |
|                       |           BF979755           |                               |
|                       |           BI545915           |                               |
|                       |           BG714615           |                               |
|                       |           BG900567           |                               |
|                       |           BG821404           |                               |
|                       |           CN309308           |                               |
|                       |           BM564406           |                               |
|                       |           BU501164           |                               |
|                       |           BU500967           |                               |
|                       |           AL555801           |                               |
|                       |           CA495288           |                               |
|                       |           BF340862           |                               |
|                       |           BI919069           |                               |
|                       |           CN309299           |                               |
|                       |           CN309312           |                               |
|                       |           BG326426           |                               |
|                       |           BC002536           |                               |
|                       |           CD359496           |                               |
|                       |           AL540434           |                               |
|                       |           BG753338           |                               |
|                       |           BG420033           |                               |
|                       |           BG422463           |                               |
|                       |           BG424785           |                               |
|                       |           BG425082           |                               |
|                       |           AL521752           |                               |
|                       |           AL558150           |                               |
|                       |           BX424263           |                               |
|                       |           AL546949           |                               |
|                       |           AL545263           |                               |
|                       |           BX443584           |                               |
|                       |             D25328           |                               |
|                       |           AA088354           |                               |
|                       |           BG419437           |                               |
|                       |           BE734796           |                               |
|                       |           CN309307           |                               |
|                       |           BM472000           |                               |
|                       |           BI911475           |                               |
|                       |           BG748649           |                               |
|                       |           BG751346           |                               |
|                       |           BI087928           |                               |
|                       |           BU857549           |                               |
|                       |           CA454854           |                               |
|                       |           BQ933378           |                               |
|                       |           BQ063259           |                               |
|                       |           BQ065839           |                               |
|                       |           BU183331           |                               |
|                       |           BG331643           |                               |
|                       |           BI914291           |                               |
|                       |           CN309310           |                               |
|                       |           BI820088           |                               |
|                       |           BG762752           |                               |
|                       |           BI824407           |                               |
|                       |           BQ431020           |                               |
|                       |           BE735199           |                               |
|                       |           BI823643           |                               |
|                       |           BU153838           |                               |
|                       |           BG773693           |                               |
|                       |           BI823603           |                               |
|                       |           AL514714           |                               |
|                       |           AL534637           |                               |
|                       |           BI461857           |                               |
|                       |           CD615227           |                               |
|                       |           CD615228           |                               |
|                       |           BE789984           |                               |
|                       |           BE514674           |                               |
|                       |           BE272668           |                               |
|                       |           BE780864           |                               |
|                       |           BQ953187           |                               |
|                       |           BE276672           |                               |
|                       |           BE275809           |                               |
|                       |           BE386379           |                               |
|                       |           BI755325           |                               |
|                       |           AW239137           |                               |
|                       |           BE279911           |                               |
|                       |           BE279913           |                               |
|                       |           CN309311           |                               |
|                       |           CV029603           |                               |
|                       |           CV029221           |                               |
|                       |           BI823952           |                               |
|                       |           BG424782           |                               |
|                       |           AL551718           |                               |
|                       |           BG773118           |                               |
|                       |           BG120904           |                               |
|                       |           CN309313           |                               |
|                       |           CA406916           |                               |
|                       |           BE843185           |                               |
|                       |           AK092597           |                               |
|                       |           AU280030           |                               |
|                       |           AU142902           |                               |
|                       |           CB143095           |                               |
|                       |           BE909372           |                               |
|                       |           BE253891           |                               |
|                       |           BQ213543           |                               |
|                       |           BG752629           |                               |
|                       |           BQ068811           |                               |
|                       |           BE867488           |                               |
|                       |           BG328195           |                               |
|                       |           BE747649           |                               |
|                       |           BQ898451           |                               |
|                       |           BQ669209           |                               |
|                       |           BE514654           |                               |
|                       |           BE258708           |                               |
|                       |           BE745431           |                               |
|                       |           BM016809           |                               |
|                       |           BM020961           |                               |
|                       |           BQ935569           |                               |
|                       |           BM551242           |                               |
|                       |           BG471900           |                               |
|                       |           BG473325           |                               |
|                       |           BG472973           |                               |
|                       |           BG472639           |                               |
|                       |           BG472819           |                               |
|                       |           BG471767           |                               |
|                       |           BM553398           |                               |
|                       |           BM553367           |                               |
|                       |           BQ919945           |                               |
|                       |           BM913070           |                               |
|                       |           BE258299           |                               |
|                       |           BE252119           |                               |
|                       |           CO248984           |                               |
|                       |           BQ226532           |                               |
|                       |           BM019681           |                               |
|                       |           BM925658           |                               |
|                       |           BQ232769           |                               |
|                       |           BM559814           |                               |
|                       |           BM467513           |                               |
|                       |           BM710951           |                               |
|                       |           BG117502           |                               |
|                       |           BQ311500           |                               |
|                       |           CB132182           |                               |
|                       |           BG770610           |                               |
|                       |           CF131934           |                               |
|                       |           BQ691739           |                               |
|                       |           BQ689545           |                               |
|                       |           BG391496           |                               |
|                       |           BG896335           |                               |
|                       |           BG897115           |                               |
|                       |             H30695           |                               |
|                       |             H30836           |                               |
|                       |             H30835           |                               |
|                       |           AW674901           |                               |
|                       |           BE867899           |                               |
|                       |             H39126           |                               |
|                       |           CB144607           |                               |
|                       |           CV365243           |                               |
|                       |           CF131994           |                               |
|                       |           BE561618           |                               |
|                       |           BQ669956           |                               |
|                       |           BU856282           |                               |
|                       |           BE396790           |                               |
|                       |           BE255919           |                               |
|                       |           AA311651           |                               |
|                       |           BG767957           |                               |
|                       |           CN309309           |                               |
|                       |           BP245909           |                               |
|                       |           AA297725           |                               |
|                       |           CV344871           |                               |
|                       |           CN309302           |                               |
|                       |           BF845028           |                               |
|                       |           BQ318031           |                               |
|                       |           BQ318189           |                               |
|                       |           BG324649           |                               |
|                       |           CN309304           |                               |
|                       |           BP266676           |                               |
|                       |           BP267080           |                               |
|                       |           BG750384           |                               |
|                       |           BG481341           |                               |
|                       |           AL120833           |                               |
|                       |           BE869083           |                               |
|                       |           BQ882284           |                               |
|                       |           CV387443           |                               |
|                       |           BG323511           |                               |
|                       |           BQ721400           |                               |
|                       |           BF972360           |                               |
|                       |           CD655490           |                               |
|                       |           BI552246           |                               |
|                       |           BP193634           |                               |
|                       |           BM707550           |                               |
|                       |           BI009309           |                               |
|                       |           BP194453           |                               |
|                       |           BP346781           |                               |
|                       |             R88153           |                               |
|                       |           BI824634           |                               |
|                       |           BI009891           |                               |
|                       |           CB154000           |                               |
|                       |           BE379229           |                               |
|                       |           BI822711           |                               |
|                       |           BX951577           |                               |
|                       |           CB154395           |                               |
|                       |           BU509580           |                               |
|                       |           AA121990           |                               |
|                       |           BG421616           |                               |
|                       |           BE311456           |                               |
|                       |           BG251321           |                               |
|                       |           BI087516           |                               |
|                       |           BQ441704           |                               |
|                       |           BG319605           |                               |
|                       |           BU165380           |                               |
|                       |           BG751291           |                               |
|                       |           BQ877549           |                               |
|                       |           CV344902           |                               |
|                       |           BG753646           |                               |
|                       |           BG753729           |                               |
|                       |           BG478899           |                               |
|                       |           AA297040           |                               |
|                       |           BE535741           |                               |
|                       |           BX350375           |                               |
|                       |           BE387317           |                               |
|                       |           AL527877           |                               |
|                       |           CR624196           |                               |
|                       |           AA853405           |                               |
|                       |           BE274057           |                               |
|                       |           CB140100           |                               |
|                       |           BE272547           |                               |
|                       |           BP265747           |                               |
|                       |           AA143274           |                               |
|                       |           BP204470           |                               |
|                       |           AL701980           |                               |
|                       |           BP268574           |                               |
|                       |           BP268571           |                               |
|                       |           AA324224           |                               |
|                       |           BX383282           |                               |
|                       |           AA102159           |                               |
|                       |           BQ058296           |                               |
|                       |           BQ637834           |                               |
|                       |           BP255237           |                               |
|                       |           BE410931           |                               |
|                       |           BI870807           |                               |
|                       |           AA156346           |                               |
|                       |           BF982853           |                               |
|                       |           BU956096           |                               |
|                       |           BM046976           |                               |
|                       |           BU857152           |                               |
|                       |           BU857850           |                               |
|                       |           BU174460           |                               |
|                       |           BU538386           |                               |
|                       |           BG752388           |                               |
|                       |           BQ217462           |                               |
|                       |           CN309305           |                               |
|                       |           BP269385           |                               |
|                       |             N88262           |                               |
|                       |           AI460372           |                               |
|                       |           CF142247           |                               |
|                       |           BE544990           |                               |
|                       |           AA143737           |                               |
|                       |           BE741702           |                               |
|                       |           BU857893           |                               |
|                       |           BF880399           |                               |
|                       |             T12211           |                               |
|                       |           BG117053           |                               |
|                       |           CN309301           |                               |
|                       |           CF140848           |                               |
|                       |           BG336041           |                               |
|                       |           BG480138           |                               |
|                       |           BC021698           |                               |
|                       |           BQ918954           |                               |
|                       |           BF701320           |                               |
|                       |           BI005552           |                               |
|                       |           AW178985           |                               |
|                       |           AW374560           |                               |
|                       |           BQ923789           |                               |
|                       |           BE730970           |                               |
|                       |           AA326635           |                               |
|                       |           BI554036           |                               |
|                       |           BE906807           |                               |
|                       |           BG760487           |                               |
|                       |           BF314044           |                               |
|                       |           BF477244           |                               |
|                       |           BF592371           |                               |
|                       |           CA488984           |                               |
|                       |           BG680103           |                               |
|                       |           BE938003           |                               |
|                       |           BE938009           |                               |
|                       |           BI259291           |                               |
|                       |             D21863           |                               |
|                       |             H19449           |                               |
|                       |           BM771266           |                               |
|                       |           CB154856           |                               |
|                       |           BQ311716           |                               |
|                       |           BG113929           |                               |
|                       |           CB148921           |                               |
|                       |           BG753025           |                               |
|                       |           BG752849           |                               |
|                       |           BG753620           |                               |
|                       |             R47796           |                               |
|                       |             R47802           |                               |
|                       |           BQ932878           |                               |
|                       |           BF081274           |                               |
|                       |           AL121190           |                               |
|                       |             R12860           |                               |
|                       |           BG422312           |                               |
|                       |           BG574903           |                               |
|                       |           BU618220           |                               |
|                       |           AW249362           |                               |
|                       |           AW675029           |                               |
|                       |           BM747437           |                               |
|                       |           BU169209           |                               |
|                       |           BQ056022           |                               |
|                       |           BE378739           |                               |
|                       |           BI869741           |                               |
|                       |           AL043512           |                               |
|                       |           CA489664           |                               |
|                       |           BG334209           |                               |
|                       |           BG282133           |                               |
|                       |           BG336936           |                               |
|                       |           BG248968           |                               |
|                       |           BG675926           |                               |
|                       |           BE408786           |                               |
|                       |           BG818344           |                               |
|                       |           BM737999           |                               |
|                       |           AA081837           |                               |
|                       |           BP256002           |                               |
|                       |           BQ896627           |                               |
|                       |           BG183704           |                               |
|                       |           AA374051           |                               |
|                       |           BI754858           |                               |
|                       |           BE311615           |                               |
|                       |           BQ324518           |                               |
|                       |           BF247173           |                               |
|                       |           BG257599           |                               |
|                       |           BI760552           |                               |
|                       |           BQ638920           |                               |
|                       |           AA315645           |                               |
|                       |           CN309306           |                               |
|                       |           BG030647           |                               |
|                       |           BQ302157           |                               |
|                       |           BM823941           |                               |
|                       |           BM809144           |                               |
|                       |             M64784           |                               |
|                       |           CB109036           |                               |
|                       |           AL578215           |                               |
|                       |           CV334481           |                               |
|                       |           BX119542           |                               |
|                       |           BG252150           |                               |
|                       |           BQ052870           |                               |
|                       |           CV328992           |                               |
|                       |           BQ638612           |                               |
|                       |           BM806484           |                               |
|                       |           AL571061           |                               |
|                       |           AL527876           |                               |
|                       |           BM799223           |                               |
|                       |           AL575210           |                               |
|                       |           BG875780           |                               |
|                       |           BG875789           |                               |
|                       |           CN309300           |                               |
|                       |           AL568405           |                               |
|                       |           AA316113           |                               |
|                       |           AA374646           |                               |
|                       |           AL565600           |                               |
|                       |           AL572093           |                               |
|                       |           BE620189           |                               |
|                       |           BG393090           |                               |
|                       |           CR598026           |                               |
|                       |           AA324565           |                               |
|                       |           BI084052           |                               |
|                       |           AL540265           |                               |
|                       |           AL540266           |                               |
|                       |           BI711653           |                               |
|                       |           BQ550024           |                               |
|                       |             W16562           |                               |
|                       |           AK126153           |                               |
|                       |             R53255           |                               |
|                       |           BC043156           |                               |
|                       |           BG236261           |                               |
|                       |           AK097692           |                               |
|                       |           AA280175           |                               |
|                       |           BF529170           |                               |
|                       |           AL521751           |                               |
|                       |           BI084854           |                               |
|                       |           BI913035           |                               |
|                       |           BF966628           |                               |
|                       |           BF980057           |                               |
|                       |           AA308362           |                               |
|                       |           AW951725           |                               |
|                       |           BQ352894           |                               |
|                       |           BI520873           |                               |
|                       |           AL514713           |                               |
|                       |           CB145316           |                               |
|                       |           AW805242           |                               |
|                       |           BG722389           |                               |
|                       |           BI258288           |                               |
|                       |           BG722311           |                               |
|                       |           AL521452           |                               |
|                       |           AL521453           |                               |
|                       |           CR621705           |                               |
|                       |           BF815303           |                               |
|                       |           AA608558           |                               |
|                       |           BF902958           |                               |
|                       |             H14937           |                               |
|                       |           AA384565           |                               |
|                       |           BQ269045           |                               |
|                       |           BF902973           |                               |
|                       |           BF688848           |                               |
|                       |           CB159322           |                               |
|                       |           CB161153           |                               |
|                       |           BP205389           |                               |
|                       |           BF217075           |                               |
|                       |             U91917           |                               |
|                       |             H30766           |                               |
|                       |             H43768           |                               |
|                       |           AW891548           |                               |
|                       |           BQ773394           |                               |
|                       |           BF340773           |                               |
|                       |           AW994518           |                               |
|                       |           BE694108           |                               |
|                       |           AI813652           |                               |
|                       |           AA169712           |                               |
|                       |           BM689215           |                               |
|                       |           CD365858           |                               |
|                       |           AW868192           |                               |
|                       |           BG323345           |                               |
|                       |           BG163386           |                               |
|                       |           BM666050           |                               |
|                       |           AI565573           |                               |
|                       |           BM792060           |                               |
|                       |           BM989461           |                               |
|                       |           BQ002798           |                               |
|                       |           BM995200           |                               |
|                       |           CA438114           |                               |
|                       |           BX394747           |                               |
|                       |           CR603216           |                               |
|                       |           BQ182450           |                               |
|                       |             W07368           |                               |
|                       |           AI187907           |                               |
|                       |           AW248944           |                               |
|                       |           BU622250           |                               |
|                       |           CD365447           |                               |
|                       |           BQ006408           |                               |
|                       |           CA418856           |                               |
|                       |           CA430152           |                               |
|                       |           BQ772166           |                               |
|                       |           CA416780           |                               |
|                       |           CA419772           |                               |
|                       |           BM824878           |                               |
|                       |           AI479642           |                               |
|                       |           AJ712030           |                               |
|                       |           BQ446288           |                               |
|                       |           BM996146           |                               |
|                       |           CA312032           |                               |
|                       |           BE300944           |                               |
|                       |           AA617959           |                               |
|                       |           CA866932           |                               |
|                       |           BQ614180           |                               |
|                       |           AA725570           |                               |
|                       |           AA247371           |                               |
|                       |           BF834589           |                               |
|                       |           BU737488           |                               |
|                       |           BQ268776           |                               |
|                       |           CB131090           |                               |
|                       |           AA603074           |                               |
|                       |           BQ006936           |                               |
|                       |           AW864812           |                               |
|                       |           CK825640           |                               |
|                       |           BE935086           |                               |
|                       |           AW613145           |                               |
|                       |           BF911874           |                               |
|                       |           AW300823           |                               |
|                       |           AA227722           |                               |
|                       |           BG122298           |                               |
|                       |           BF038705           |                               |
|                       |           BG474225           |                               |
|                       |           AW338365           |                               |
|                       |           AA946886           |                               |
|                       |           AI570920           |                               |
|                       |           AW769140           |                               |
|                       |           AI498239           |                               |
|                       |           BG057796           |                               |
|                       |           AA570091           |                               |
|                       |           AA634394           |                               |
|                       |           BX351097           |                               |
|                       |           AA622467           |                               |
|                       |           AI865816           |                               |
|                       |           AW571827           |                               |
|                       |           BI711249           |                               |
|                       |           BF769071           |                               |
|                       |           AW517726           |                               |
|                       |           BF769777           |                               |
|                       |           AI653786           |                               |
|                       |           AI861994           |                               |
|                       |           BF769781           |                               |
|                       |           AI568954           |                               |
|                       |             W35243           |                               |
|                       |           AI312567           |                               |
|                       |           CA398111           |                               |
|                       |           AA565609           |                               |
|                       |           AI913029           |                               |
|                       |           AW674259           |                               |
|                       |           AL045356           |                               |
|                       |           AU160096           |                               |
|                       |           AA827484           |                               |
|                       |           AI199670           |                               |
|                       |           CA422414           |                               |
|                       |           AI201785           |                               |
|                       |           BU607298           |                               |
|                       |           BE536802           |                               |
|                       |           AA503834           |                               |
|                       |           AA878031           |                               |
|                       |           AI183668           |                               |
|                       |           BI006943           |                               |
|                       |           BU608282           |                               |
|                       |           AW770004           |                               |
|                       |           BM669825           |                               |
|                       |           AW248638           |                               |
|                       |           AA847671           |                               |
|                       |           CA310663           |                               |
|                       |           CB242092           |                               |
|                       |           AA633506           |                               |
|                       |           AI634941           |                               |
|                       |           AA406495           |                               |
|                       |           AA383687           |                               |
|                       |           AA383688           |                               |
|                       |           AI932686           |                               |
|                       |           BX104332           |                               |
|                       |           AA121991           |                               |
|                       |           CN479779           |                               |
|                       |             H53527           |                               |
|                       |           AA617688           |                               |
|                       |           AA406517           |                               |
|                       |           AA984558           |                               |
|                       |           AA406340           |                               |
|                       |           AA406382           |                               |
|                       |           AI192072           |                               |
|                       |           AI653882           |                               |
|                       |           AI869874           |                               |
|                       |           AI929834           |                               |
|                       |           AW166540           |                               |
|                       |           AW469070           |                               |
|                       |           AA554183           |                               |
|                       |           AI476052           |                               |
|                       |           CB242235           |                               |
|                       |           CN309303           |                               |
|                       |           AA769419           |                               |
|                       |           AA579837           |                               |
|                       |           AI032219           |                               |
|                       |           AA622586           |                               |
|                       |             N80241           |                               |
|                       |             T28549           |                               |
|                       |             N90878           |                               |
|                       |           BM145904           |                               |
|                       |           AA826472           |                               |
|                       |           AI870799           |                               |
|                       |           AW069643           |                               |
|                       |           AI969266           |                               |
|                       |           AW103808           |                               |
|                       |           BQ000992           |                               |
|                       |           CB110734           |                               |
|                       |           AI332993           |                               |
|                       |           AA156311           |                               |
|                       |             R87480           |                               |
|                       |           AA223736           |                               |
|                       |           BM145892           |                               |
|                       |           AA228101           |                               |
|                       |           BP328982           |                               |
|                       |           CB155223           |                               |
|                       |           BE925392           |                               |
|                       |           BF229295           |                               |
|                       |             W60432           |                               |
|                       |           AW474675           |                               |
|                       |           AA079349           |                               |
|                       |             R49952           |                               |
|                       |           BM673385           |                               |
|                       |           AA303532           |                               |
|                       |           BX373727           |                               |
|                       |           BX339431           |                               |
|                       |           CR622143           |                               |
|                       |             H28157           |                               |
|                       |           BM799158           |                               |
|                       |             H28156           |                               |
|                       |           AI926938           |                               |
|                       |             H43690           |                               |
|                       |           BU733928           |                               |
|                       |           CA434957           |                               |
|                       |           BG056046           |                               |
|                       |           AA834352           |                               |
|                       |           AA609613           |                               |
|                       |           CA438046           |                               |
|                       |           BM929549           |                               |
|                       |           AI274501           |                               |
|                       |           AI309947           |                               |
|                       |           BM150042           |                               |
|                       |           AI367848           |                               |
|                       |           BM145954           |                               |
|                       |           AA865053           |                               |
|                       |           CD742553           |                               |
|                       |           BM980148           |                               |
|                       |           AI674256           |                               |
|                       |           BG993674           |                               |
|                       | *Seq. mapped in strand Minus*| *Seq. mapped in strand Minus* |
|                       |           AI218450           |          AK047303             |
|                       |                              |          AK049841             |
|                       |                              |          AK098162             |
|                       |                              |          AK004790             |
+-----------------------+------------------------------+-------------------------------+
|      #  158           | *Seq. mapped in strand Plus* | *Seq. mapped in strand Plus*  |
|                       |             D49677           |          BB631919             |
|                       |           BC065719           |                               |
|                       |           BC050451           |                               |
|                       |           AK090845           |                               |
|                       | *Seq. mapped in strand Minus*| *Seq. mapped in strand Minus* |
|                       |           BC039434           |            D45205             |
+-----------------------+------------------------------+-------------------------------+
|      #  159           | *Seq. mapped in strand Plus* | *Seq. mapped in strand Plus*  |
|                       |           BG331189           |          BB630417             |
|                       |           BM013439           |                               |
|                       |           BI045648           |                               |
|                       |           BF507909           |                               |
|                       |           BM013440           |                               |
|                       |           AA381547           |                               |
|                       | *Seq. mapped in strand Minus*| *Seq. mapped in strand Minus* |
|                       |           AI418291           |          BU705617             |
|                       |             U47741           |          CF182283             |
|                       |             R23928           |          BU056491             |
|                       |           AI051629           |          BX514254             |
|                       |           BC036486           |          AI153503             |
|                       |           BM676880           |          CB248782             |
|                       |             U89355           |          BB839506             |
|                       |           BQ029925           |          AV312780             |
|                       |           BM129508           |          BF017160             |
|                       |           BM129360           |          BB174319             |
|                       |           AA916844           |          BG066859             |
|                       |           CK819722           |          BB727918             |
|                       |           BI492072           |          CO044016             |
|                       |           CD679501           |          BC072594             |
|                       |           BU754011           |          AV344996             |
|                       |           AW078578           |          BQ031132             |
|                       |           BU687259           |          CN665068             |
|                       |             U89354           |          BB703374             |
|                       |             U85962           |          BB698972             |
|                       |           CF143643           |          BE853454             |
|                       |           BM905201           |          AI507501             |
|                       |           BI058853           |          AI644480             |
|                       |           AW502055           |          BM237740             |
|                       |           BF870477           |          BM239308             |
|                       |           BF926303           |          BM238543             |
|                       |           BF814065           |            C87889             |
|                       |           AI478359           |          AU019006             |
|                       |           AI953646           |          CN663127             |
|                       |           BU685551           |          AU016337             |
|                       |           CA415220           |          BM239120             |
|                       |           BF514806           |          BM219533             |
|                       |           AI498147           |          BM238078             |
|                       |           AK124320           |          BM238266             |
|                       |           BE646643           |          BM239582             |
|                       |             T81980           |          BM220224             |
|                       |           BU954372           |          BM238274             |
|                       |           AW589253           |          BM239969             |
|                       |           CN281972           |          AW543312             |
|                       |           BI962925           |          BM239728             |
|                       |           BF743594           |          BM238982             |
|                       |             T81558           |          BG069466             |
|                       |           BP247980           |          BB451123             |
|                       |           CK299361           |          BB728004             |
|                       |           BF999489           |          BB373385             |
|                       |           BE813991           |          CK386491             |
|                       |           BG428469           |          BY777952             |
|                       |           BI062731           |          BI407694             |
|                       |           BI005431           |          AI615847             |
|                       |           BE765160           |          AA208496             |
|                       |           BE933140           |          BG080014             |
|                       |           BP361121           |          AA572296             |
|                       |           BP217635           |          BM237379             |
|                       |           AW502109           |          CD578470             |
|                       |           CD703653           |          BF134213             |
|                       |           AW502645           |          BY146407             |
|                       |           AW502494           |          CK780359             |
|                       |           BP327287           |          CB245015             |
|                       |           BF828376           |          CB522426             |
|                       |           BF750958           |          CF161724             |
|                       |           BQ948066           |          BI655732             |
|                       |           AW384757           |          CF743638             |
|                       |           BI032961           |          CD803887             |
|                       |           BI032885           |          BF122259             |
|                       |           AW499946           |          BF301690             |
|                       |           AW835332           |          BE848026             |
|                       |           BQ340094           |          CN663007             |
|                       |           AW387730           |          CN685934             |
|                       |           AW378272           |          CN696780             |
|                       |           AW387732           |          AA867413             |
|                       |           AW378283           |          CB179118             |
|                       |           AW370361           |          BE631719             |
|                       |           AW378291           |          AI597117             |
|                       |           AW370297           |          CN661227             |
|                       |           AW378337           |          CN719812             |
|                       |           BF349322           |          CN663014             |
|                       |           AW370379           |          BG800545             |
|                       |           AW370324           |          CA571020             |
|                       |           AW366264           |          BU922423             |
|                       |           AW378261           |          CA526617             |
|                       |           AW370363           |          CN668492             |
|                       |           AW370367           |          CN721226             |
|                       |           AW370376           |          CA529843             |
|                       |           AW370351           |          BG082395             |
|                       |           AW378310           |          AA066593             |
|                       |           AW387735           |          BI408062             |
|                       |           AW387733           |          CK620288             |
|                       |           AW581885           |          CN724864             |
|                       |           AW370353           |          CN660277             |
|                       |           BF349831           |          CN661360             |
|                       |           AW370309           |          CA571929             |
|                       |           AW370314           |          CN660896             |
|                       |           AW581882           |          CN716481             |
|                       |           AW366263           |          CN660508             |
|                       |           AW366262           |          CN661943             |
|                       |           BQ708976           |          CN661900             |
|                       |           AW366272           |          CA571367             |
|                       |           AW366269           |          CN661544             |
|                       |           AW366270           |          CN660053             |
|                       |           AW366271           |          CN663393             |
|                       |           AW366273           |          CA572709             |
|                       |           BE925907           |          CA570776             |
|                       |           BE925909           |          CA570423             |
|                       |           BE925901           |          CA571035             |
|                       |           CD618634           |          CA572271             |
|                       |           BF081912           |          CA572224             |
|                       |           CD618633           |          CA570019             |
|                       |           BE901879           |          BY719995             |
|                       |           CN281973           |          CA573291             |
|                       |           BE903065           |          CB178985             |
|                       |           BE902874           |          CA572091             |
|                       |           CF143186           |          BB376788             |
|                       |           BQ226286           |          BC010297             |
|                       |           BP363852           |          CF910302             |
|                       |           BP251370           |          CA572541             |
|                       |           CF143080           |          CF910468             |
|                       |           AI902500           |          CF891998             |
|                       |           AI902526           |          CF892924             |
|                       |           BF881055           |          BY012440             |
|                       |           BI023128           |          CN662548             |
|                       |           CF143586           |          CD349855             |
|                       |           BP333132           |          BI985484             |
|                       |           AA723653           |          BG808377             |
|                       |           AA928265           |          CF165619             |
|                       |           AW236046           |          BI854240             |
|                       |           AW242963           |          BU704205             |
|                       |           AI015481           |          AW762866             |
|                       |           AI425092           |          CD554567             |
|                       |           AA994887           |          CN703149             |
|                       |           AA609754           |          CN696234             |
|                       |           AW591995           |                               |
|                       |           AA912637           |                               |
|                       |           AW779831           |                               |
|                       |           AI934977           |                               |
|                       |           BM464147           |                               |
|                       |           BF511449           |                               |
|                       |           AI902536           |                               |
|                       |           BF881071           |                               |
|                       |           BG387701           |                               |
|                       |           AI902527           |                               |
|                       |           AW499646           |                               |
|                       |           AW382816           |                               |
|                       |           BE706623           |                               |
|                       |           BE154040           |                               |
|                       |           AW501746           |                               |
|                       |           BI013191           |                               |
|                       |           BU175243           |                               |
|                       |           BG013960           |                               |
|                       |           BE710451           |                               |
|                       |           BE836750           |                               |
|                       |           BM013519           |                               |
|                       |           BE156640           |                               |
|                       |           BP251758           |                               |
|                       |           BE694422           |                               |
|                       |           BE932404           |                               |
|                       |           BF855593           |                               |
|                       |           CF137651           |                               |
|                       |           BG913806           |                               |
|                       |           BP383653           |                               |
|                       |           BG955716           |                               |
|                       |           BG980203           |                               |
|                       |           BG955729           |                               |
|                       |           AW369380           |                               |
|                       |           AW369376           |                               |
+-----------------------+------------------------------+-------------------------------+
|      #  160           | *Seq. mapped in strand Plus* | *Seq. mapped in strand Plus*  |
|                       |           AB035898           |          BY086720             |
|                       |           AL832908           |          BB659442             |
|                       |           AK098533           |          AK052362             |
|                       |           AF308294           |          CN687726             |
|                       |           AK027816           |          BB569835             |
|                       |                              |          AK014461             |
|                       |                              |          CN536462             |
|                       |                              |          CN535951             |
|                       |                              |          CN534621             |
|                       |                              |          AB001432             |
|                       |                              |          CN538991             |
|                       |                              |          CN533772             |
|                       |                              |          CO045535             |
|                       |                              |          BQ561081             |
|                       |                              |          CF726821             |
|                       |                              |          CK781013             |
|                       |                              |          CK637857             |
|                       |                              |          CK392337             |
|                       |                              |          BB440582             |
|                       |                              |          CN530651             |
|                       |                              |          CN681468             |
|                       |                              |          CN683768             |
|                       |                              |          CN459053             |
|                       |                              |          CA568408             |
|                       |                              |          AA386442             |
|                       |                              |          BY731621             |
|                       |                              |          CF729422             |
|                       |                              |          BB616630             |
|                       |                              |          BX529224             |
|                       |                              |          AW320147             |
|                       |                              |          CK635186             |
|                       |                              |          BB411437             |
|                       |                              |          BB536905             |
|                       |                              |          CF909341             |
|                       |                              |          CN675345             |
|                       |                              |          CF727421             |
|                       |                              |          CD543027             |
|                       |                              |          CD544068             |
|                       |                              |          CK635716             |
|                       |                              |          CN695657             |
|                       |                              |          CF531253             |
|                       |                              |          CN459719             |
|                       |                              |          BU743745             |
|                       |                              |          CK635825             |
|                       |                              |          CA872349             |
|                       |                              |          CD552215             |
|                       |                              |          CK638934             |
|                       |                              |          BB479493             |
|                       |                              |          BQ956226             |
|                       |                              |          CA542650             |
|                       |                              |          BY378180             |
|                       |                              |          AW320970             |
|                       |                              |          CN673919             |
|                       |                              |          CA569108             |
|                       |                              |          BQ886190             |
|                       |                              |          AI603840             |
|                       |                              |          AA144566             |
|                       |                              |          BY745450             |
|                       |                              |          BM250113             |
|                       |                              |          CD544067             |
|                       |                              |          CF899663             |
|                       |                              |          BM569898             |
|                       |                              |          CD543026             |
|                       |                              |          BU939632             |
|                       |                              |          AV275273             |
|                       |                              |          BY360469             |
|                       |                              |          BU939536             |
|                       |                              |          CK782338             |
|                       |                              |          AA396604             |
|                       | *Seq. mapped in strand Minus*| *Seq. mapped in strand Minus* |
|                       |           AF147440           |          AK044385             |
+-----------------------+------------------------------+-------------------------------+
|      #  161           | *Seq. mapped in strand Plus* | *Seq. mapped in strand Plus*  |
|                       |           AY358165           |          BE987206             |
|                       |           AB058760           |          BE987492             |
|                       |           CA393159           |                               |
|                       |           BU159259           |                               |
|                       |           BX281558           |                               |
|                       |           BC013770           |                               |
|                       |           BF791911           |                               |
|                       |           AF131842           |                               |
|                       |           BI031833           |                               |
|                       |           BI030875           |                               |
|                       |           BM930930           |                               |
|                       |           BQ894522           |                               |
|                       | *Seq. mapped in strand Minus*| *Seq. mapped in strand Minus* |
|                       |           BM674233           |          BE956726             |
|                       |                              |          BM934422             |
+-----------------------+------------------------------+-------------------------------+
|      #  162           | *Seq. mapped in strand Plus* | *Seq. mapped in strand Plus*  |
|                       |           BI545457           |          AK044319             |
|                       |           BM835384           |                               |
|                       |           BP251824           |                               |
|                       |           BP282570           |                               |
|                       |           CA336158           |                               |
|                       |           BM828958           |                               |
|                       |           BP291066           |                               |
|                       |           BP307603           |                               |
|                       |           BX403404           |                               |
|                       |           BX362695           |                               |
|                       |           CR615990           |                               |
|                       |           AW149562           |                               |
|                       |           AW444598           |                               |
|                       |           BF513563           |                               |
|                       |           AW451076           |                               |
|                       |           AW293980           |                               |
|                       |           BI522512           |                               |
|                       |           CN308678           |                               |
|                       |           AL699236           |                               |
|                       |           BP208974           |                               |
|                       |           BP280858           |                               |
|                       |           AK055032           |                               |
|                       |           BI912741           |                               |
|                       |           BM807479           |                               |
|                       |           BI917912           |                               |
|                       |           BX395351           |                               |
|                       |           BX385110           |                               |
|                       |           BM799940           |                               |
|                       |           BC001816           |                               |
|                       |           BC001851           |                               |
|                       |           BX458184           |                               |
|                       |           BG422831           |                               |
|                       |           BX478685           |                               |
|                       |           BX446603           |                               |
|                       |           AL135523           |                               |
|                       |           AI830810           |                               |
|                       |           BG419298           |                               |
|                       |           BX333832           |                               |
|                       |           BX093701           |                               |
|                       |           BX460694           |                               |
|                       |           CD683563           |                               |
|                       |           BE897208           |                               |
|                       |           BI821872           |                               |
|                       |           AA234764           |                               |
|                       |           BM413681           |                               |
|                       |           BP312927           |                               |
|                       |           BX420929           |                               |
|                       |           BE621967           |                               |
|                       |           AA760746           |                               |
|                       |           BP218457           |                               |
|                       |           BP235389           |                               |
|                       |           AW821184           |                               |
|                       |           CV026622           |                               |
|                       |           BT006837           |                               |
|                       |           AY376900           |                               |
|                       |           AW582705           |                               |
|                       |           BI048001           |                               |
|                       |           AF215923           |                               |
|                       |           AF237413           |                               |
|                       |             X63465           |                               |
|                       |           BX388214           |                               |
|                       |           AA090686           |                               |
|                       |           BU902525           |                               |
|                       |           BP254111           |                               |
|                       |           AI084630           |                               |
|                       |           AI336300           |                               |
|                       |           BP291260           |                               |
|                       |           BU927599           |                               |
|                       |           BM928237           |                               |
|                       |           BG943988           |                               |
|                       |           BU942154           |                               |
|                       |           BP331765           |                               |
|                       |             F07939           |                               |
|                       |           BG696741           |                               |
|                       |           AA349424           |                               |
|                       |           BG026762           |                               |
|                       |           BP275697           |                               |
|                       |             T08323           |                               |
|                       |           BM450132           |                               |
|                       |           BX349221           |                               |
|                       |           BE089929           |                               |
|                       |           CA454928           |                               |
|                       |           BP254832           |                               |
|                       |           BG121196           |                               |
|                       |           BF244934           |                               |
|                       |           BQ953292           |                               |
|                       |             R51677           |                               |
|                       |           CB123119           |                               |
|                       |             T27797           |                               |
|                       |           BF217372           |                               |
|                       |           BP203529           |                               |
|                       |           CB136170           |                               |
|                       |           AA780404           |                               |
|                       |           CN308679           |                               |
|                       |             F05663           |                               |
|                       |           BP330552           |                               |
|                       |           BF802754           |                               |
|                       |           BF803194           |                               |
|                       |           BF818032           |                               |
|                       |           BP331155           |                               |
|                       |             T95719           |                               |
|                       |             T81875           |                               |
|                       |             T95162           |                               |
|                       |             T95687           |                               |
|                       |             T99265           |                               |
|                       |             R54161           |                               |
|                       |           BM793886           |                               |
|                       |           BX388213           |                               |
|                       |           BI223701           |                               |
|                       |           CF145489           |                               |
|                       |           CF134539           |                               |
|                       |           BX282852           |                               |
|                       |           CR736764           |                               |
|                       |           CB164488           |                               |
|                       |           CD107090           |                               |
|                       |           CD236984           |                               |
|                       |           BQ325094           |                               |
|                       |           AW195934           |                               |
|                       |           AI609628           |                               |
|                       |             W28750           |                               |
|                       |             W26956           |                               |
|                       |           BP218283           |                               |
|                       |           BI090748           |                               |
|                       |           CB159040           |                               |
|                       |           AA429759           |                               |
|                       |           BI765360           |                               |
|                       |           BQ772239           |                               |
|                       |           AI741574           |                               |
|                       |           AW118760           |                               |
|                       |           BQ018689           |                               |
|                       |           AW197796           |                               |
|                       |           BM988681           |                               |
|                       |           AA770511           |                               |
|                       |           BQ014546           |                               |
|                       |           BG217113           |                               |
|                       |           BG183742           |                               |
|                       |           BG216156           |                               |
|                       |           CA430602           |                               |
|                       |           BG185849           |                               |
|                       |           BG201843           |                               |
|                       |           BM991718           |                               |
|                       |           BQ021595           |                               |
|                       |           BU733880           |                               |
|                       |           AI361836           |                               |
|                       |             R51808           |                               |
|                       |           AI039349           |                               |
|                       |           AI693920           |                               |
|                       |             R54106           |                               |
|                       |           AW028577           |                               |
|                       |           AW169435           |                               |
|                       |           AA846463           |                               |
|                       |           AW169571           |                               |
|                       |           AI243599           |                               |
|                       | *Seq. mapped in strand Minus*| *Seq. mapped in strand Minus* |
|                       |             N27496           |          BC011279             |
+-----------------------+------------------------------+-------------------------------+
|      #  163           | *Seq. mapped in strand Plus* | *Seq. mapped in strand Plus*  |
|                       |           AA534912           |          AK019740             |
|                       |           BM683955           |                               |
|                       |           BQ016903           |                               |
|                       | *Seq. mapped in strand Minus*| *Seq. mapped in strand Minus* |
|                       |           AI362988           |          AK122537             |
|                       |           BU632817           |          AF177026             |
|                       |           AI015699           |          AK078476             |
|                       |           BQ020959           |          BC011163             |
|                       |           CB241205           |          AK049766             |
|                       |           BQ447126           |                               |
|                       |           AW139211           |                               |
|                       |           AA496040           |                               |
|                       |           AI264563           |                               |
|                       |           AI244897           |                               |
|                       |           BU857661           |                               |
|                       |           AI928198           |                               |
|                       |             H15152           |                               |
|                       |           AI033761           |                               |
|                       |           AI142835           |                               |
|                       |           AI096639           |                               |
|                       |           AI422105           |                               |
|                       |           BE669754           |                               |
|                       |           BF740226           |                               |
|                       |           AA761918           |                               |
|                       |           BQ185645           |                               |
|                       |           BU680305           |                               |
|                       |           AA694240           |                               |
|                       |           AA878742           |                               |
|                       |           AI916263           |                               |
|                       |           BI255558           |                               |
|                       |           CA448220           |                               |
|                       |           BX112857           |                               |
|                       |           BQ184014           |                               |
|                       |           BQ772595           |                               |
|                       |           CA419943           |                               |
|                       |           BM980082           |                               |
|                       |           CA314610           |                               |
|                       |           BQ000883           |                               |
|                       |           AL133080           |                               |
|                       |           BC038957           |                               |
|                       |           BM549040           |                               |
|                       |           AI354493           |                               |
|                       |           BQ183347           |                               |
|                       |           BG111948           |                               |
|                       |           BM977338           |                               |
|                       |           CA423471           |                               |
|                       |           CA443013           |                               |
|                       |           AW575611           |                               |
|                       |           AI246751           |                               |
|                       |           AB051450           |                               |
|                       |           AW139676           |                               |
|                       |           BM150856           |                               |
|                       |           CR601882           |                               |
|                       |           AW295638           |                               |
|                       |           BQ014012           |                               |
|                       |           AI080270           |                               |
|                       |           AA541454           |                               |
|                       |           BM673033           |                               |
|                       |           BM671672           |                               |
|                       |           CD366042           |                               |
|                       |           AA749031           |                               |
|                       |           CR456594           |                               |
|                       |           CR541684           |                               |
|                       |           BC028919           |                               |
|                       |           BU521126           |                               |
|                       |           BX340231           |                               |
|                       |           BM704525           |                               |
|                       |           BQ218543           |                               |
|                       |           BM803647           |                               |
|                       |           CB959922           |                               |
|                       |           CN260207           |                               |
|                       |           CD676094           |                               |
|                       |           CN260209           |                               |
|                       |           CA392427           |                               |
|                       |           BM689377           |                               |
|                       |           CN260213           |                               |
|                       |           BM453650           |                               |
|                       |           BM795385           |                               |
|                       |           BG911484           |                               |
|                       |           CR746436           |                               |
|                       |           BM152841           |                               |
+-----------------------+------------------------------+-------------------------------+
|      #  164           | *Seq. mapped in strand Plus* | *Seq. mapped in strand Plus*  |
|                       |           BX425403           |          BQ714450             |
|                       |           BX375575           |          BC003197             |
|                       |           BP382193           |          BB863636             |
|                       |           CB148781           |          BY735802             |
|                       |           AU141328           |          AK031366             |
|                       |           BP252461           |          BY032530             |
|                       |           BM845877           |          BY296621             |
|                       |           BP253738           |          BY746262             |
|                       |           BP271955           |          BB859660             |
|                       |           AU126854           |          BB620752             |
|                       |           AU125544           |          BY735868             |
|                       |           CB131159           |          AW011970             |
|                       |           CB113650           |          AI882201             |
|                       |           BM847998           |          AW226661             |
|                       |           AU129615           |          BQ256863             |
|                       |           BP253216           |          BQ256935             |
|                       |           BP275844           |          BQ961208             |
|                       |           BP311938           |          BU514454             |
|                       |           BP275721           |          BB866666             |
|                       |           BP281509           |          BY288940             |
|                       |           AU126224           |          BE374012             |
|                       |           AU133009           |          BB663204             |
|                       |           AK054611           |          BQ900413             |
|                       |           AK094206           |          CA562770             |
|                       |           BU155742           |          BI651963             |
|                       |           CD516909           |          AA471756             |
|                       |           BX424635           |          BI556649             |
|                       |           BX354912           |          BY736324             |
|                       |           BX419660           |          CK341410             |
|                       |           CN413266           |          AA465877             |
|                       |           CN413270           |          BY097906             |
|                       |           AL535208           |          BY087744             |
|                       |           BX378899           |          BY094643             |
|                       |           BM453610           |          CA481882             |
|                       |           BX397727           |          BY737340             |
|                       |           BX397579           |          BB851865             |
|                       |           BX386113           |          BB854699             |
|                       |           BM547078           |          BU512592             |
|                       |           BG324033           |          AU066571             |
|                       |           BU541887           |          CF903824             |
|                       |           BX424524           |          BB647121             |
|                       |           BQ222718           |          AI530069             |
|                       |           BX423112           |          CB193399             |
|                       |           BX403918           |          CB193396             |
|                       |           AL537979           |          BI555892             |
|                       |           AL560135           |          BY032418             |
|                       |           AL513748           |          BY278778             |
|                       |           CR611998           |          AA638213             |
|                       |           AL519202           |          BI218149             |
|                       |           AL559033           |          BU152066             |
|                       |           AL556969           |          CF899799             |
|                       |           BC012302           |          CF899636             |
|                       |           BU171647           |          BG962973             |
|                       |           BM479994           |          BG975889             |
|                       |           BM449980           |          BY007052             |
|                       |           BP272689           |          CD550392             |
|                       |           BM547630           |          CK389336             |
|                       |           BG706569           |          CF903507             |
|                       |             D67025           |          CF905067             |
|                       |           CB960404           |          BY732946             |
|                       |           CN413262           |          BM948155             |
|                       |           CD109732           |          CD555247             |
|                       |           AI339734           |          BQ572990             |
|                       |           AI379272           |          BG864048             |
|                       |           CN413260           |          BQ886682             |
|                       |           BQ687860           |          AK089933             |
|                       |           BQ685956           |          BF161984             |
|                       |           BQ709802           |          BI153227             |
|                       |           BM473579           |          BI646877             |
|                       |           BG419686           |          AA771107             |
|                       |           BG769733           |          BU839846             |
|                       |           BU186728           |          BI664171             |
|                       |           BG766931           |          BY736722             |
|                       |           BI117451           |          AL365022             |
|                       |           BP340612           |          CK625791             |
|                       |           CB153933           |          AK077252             |
|                       |           AU131085           |          BI650861             |
|                       |           BP357370           |          BY772442             |
|                       |           BE397516           |          BY260711             |
|                       |           CN413267           |          BB617438             |
|                       |           AU125637           |          CA469420             |
|                       |           BE386006           |          BB864764             |
|                       |           BP300291           |          BE290818             |
|                       |           BP233108           |          BF123326             |
|                       |           BP254262           |          BE569524             |
|                       |           AU122193           |          BB867307             |
|                       |           AU122445           |          BE309634             |
|                       |           BF026169           |          CA751578             |
|                       |           BM558096           |          BI657956             |
|                       |           BM464711           |          BI152887             |
|                       |           BM479552           |          CN664724             |
|                       |           AK055799           |          BG964266             |
|                       |           BU192690           |          BY263744             |
|                       |           CN413269           |          BF101519             |
|                       |           BG386419           |          BE569217             |
|                       |           BG679860           |          BI555406             |
|                       |           BU165001           |          BY732919             |
|                       |           BM475593           |          BI656529             |
|                       |           BM918348           |          AW318443             |
|                       |           BG333986           |          BY098086             |
|                       |           BG740606           |          CF173940             |
|                       |           BQ227121           |          CF173304             |
|                       |           BM905132           |          BQ930386             |
|                       |           BQ711572           |          BI854107             |
|                       |           AL559889           |          CA751946             |
|                       |           BG256903           |          CA562637             |
|                       |           BM847851           |          BI331525             |
|                       |           BQ919169           |          CK332500             |
|                       |           BU502751           |          CF894531             |
|                       |           BX367053           |          BI692523             |
|                       |           BX404529           |          CF904933             |
|                       |           BQ063367           |          BF022473             |
|                       |           AL514982           |          BG172707             |
|                       |           CD580389           |          BI662403             |
|                       |           CF131393           |          BI155324             |
|                       |           BX444429           |          BE371660             |
|                       |           AW162713           |          BI986200             |
|                       |           BQ707153           |          CA534984             |
|                       |           AW162822           |          CA554411             |
|                       |           BM827336           |          BQ887167             |
|                       |           AU124516           |          CF734941             |
|                       |           CN413273           |          BI083255             |
|                       |           BM786210           |          BF178874             |
|                       |           BF312059           |          BF468069             |
|                       |           BF316866           |          CA563087             |
|                       |           BG750727           |          BF168126             |
|                       |           BM916130           |          BY110769             |
|                       |           BM552498           |          AA028215             |
|                       |           BE297951           |          CA977040             |
|                       |           BM913522           |          CB195470             |
|                       |           AL536306           |          AI314431             |
|                       |           BE796397           |          BI692212             |
|                       |           BQ428328           |          AA475855             |
|                       |           BG396711           |          BF781754             |
|                       |           BG396862           |          AV004957             |
|                       |           AU121655           |          CA751152             |
|                       |           BX357623           |          BQ714356             |
|                       |           AK022896           |          BE571183             |
|                       |           BE299327           |          CN721778             |
|                       |           BG339343           |          CA551457             |
|                       |           BP361133           |          BF142403             |
|                       |           BE277110           |          BG918600             |
|                       |           BP196876           |          BF123289             |
|                       |           BF306707           |          BG871943             |
|                       |           BF307021           |          BF100777             |
|                       |           BU161735           |          BF579748             |
|                       |           BU192280           |          BQ571004             |
|                       |             W61041           |          BE227228             |
|                       |           CB125363           |          AA118473             |
|                       |           BG830388           |          BI655239             |
|                       |           BI088307           |          AA612172             |
|                       |           CN413272           |          AA473949             |
|                       |           BI225241           |          BG804252             |
|                       |           BI226046           |          BQ256775             |
|                       |           BI200158           |          CK640295             |
|                       |           BC000074           |          BY354004             |
|                       |           AW732940           |          BF169227             |
|                       |           AL542819           |          BF160614             |
|                       |           CF243136           |          BE863859             |
|                       |           BQ070822           |          BB565530             |
|                       |           BC020518           |          AA543460             |
|                       |           BC025686           |          AA138006             |
|                       |           BG468591           |          BF452573             |
|                       |           BQ068081           |          CD772539             |
|                       |           BQ328495           |          AA762792             |
|                       |           CF131402           |          AA389544             |
|                       |           BM471658           |          BE853913             |
|                       |           BI200192           |          BF116751             |
|                       |           BX358448           |          BF141837             |
|                       |           BE299750           |          CD564860             |
|                       |           BE296547           |          BI652557             |
|                       |           BF309945           |          BG245076             |
|                       |           BE250280           |          CB192790             |
|                       |           BG422727           |          CF731818             |
|                       |           BG258575           |          BU604198             |
|                       |           AI929784           |          CA322081             |
|                       |           BU159530           |          BF449416             |
|                       |           BX443328           |          AA073854             |
|                       |           BE336738           |          BQ043041             |
|                       |           BQ231543           |          BF119959             |
|                       |           AI479891           |          BI853757             |
|                       |           BE397158           |          BI526088             |
|                       |           BE407657           |          AA760555             |
|                       |           BE270797           |          AI021223             |
|                       |           BE280452           |          AA588950             |
|                       |           BI821028           |          CF902663             |
|                       |           BE731022           |            W62195             |
|                       |           BF316791           |          BI158590             |
|                       |           BF311521           |          AA681541             |
|                       |           BI196268           |          BI689275             |
|                       |           BQ943831           |          AA003441             |
|                       |           BF983572           |          AA260148             |
|                       |           BC004859           |          AA008861             |
|                       |           BE897843           |          AA821329             |
|                       |           CD359013           |          AA066957             |
|                       |           BG744087           |          BI155618             |
|                       |           CD558068           |          AA575021             |
|                       |           BG771983           |          BI694749             |
|                       |           BI459346           |          AI255837             |
|                       |           CV026424           |          BI157667             |
|                       |           CV025337           |          AA103339             |
|                       |           BT007217           |          AA718098             |
|                       |           CR456772           |          BF164974             |
|                       |           CD244621           |          BI652109             |
|                       |           AI750985           |          BI159190             |
|                       |           BQ053670           |          AI196981             |
|                       |           BU849450           |          CB522864             |
|                       |           BX356162           |          BI692447             |
|                       |           CR603532           |          AW740621             |
|                       |           BE833204           |          BE534571             |
|                       |           BG328661           |          BI656853             |
|                       |           BG468493           |          BI150120             |
|                       |           BI089813           |          BI648300             |
|                       |           BP265661           |          AI099222             |
|                       |           BE175349           |          BI156968             |
|                       |           BU557831           |          BE623352             |
|                       |           BQ320532           |          AA155234             |
|                       |           BG982744           |          BE952250             |
|                       |           BG983472           |          BY757093             |
|                       |           BG982918           |          CF949336             |
|                       |           BG480307           |            W20792             |
|                       |           AU125880           |          AI152344             |
|                       |           CN413259           |          CA980066             |
|                       |           BM018171           |          CB194593             |
|                       |           BE278142           |          BF449285             |
|                       |           BE280877           |          AI155275             |
|                       |           BG982733           |          BY756242             |
|                       |           BG419142           |          BY756179             |
|                       |           BG696784           |          AA545608             |
|                       |           BU179102           |          BY755116             |
|                       |           BM799890           |          BY756701             |
|                       |           AW732853           |          CA558672             |
|                       |           CF144967           |          AA217961             |
|                       |           CF145500           |          BU613045             |
|                       |           AA305756           |          AA612166             |
|                       |           BF748139           |          CF900074             |
|                       |           BG768236           |          AW990462             |
|                       |           BG675049           |          BY762168             |
|                       |           BQ212928           |          AV286645             |
|                       |           BF110190           |          BY757683             |
|                       |           BG289449           |          CA451985             |
|                       |           BE545296           |          BE854147             |
|                       |           BG252344           |          BM230979             |
|                       |           BP268927           |          BM213918             |
|                       |           BM854320           |          AW214592             |
|                       |           BP256086           |          AW557356             |
|                       |           AA380663           |          BM231101             |
|                       |           BP326393           |          BM233682             |
|                       |           BG739986           |          BM231395             |
|                       |           BP329882           |          AA624206             |
|                       |           BM821377           |          AA170409             |
|                       |           BU184065           |          AI322896             |
|                       |           BM554908           |          BE291876             |
|                       |           BE873532           |          BQ101259             |
|                       |           BP269706           |          AW413485             |
|                       |           BM550985           |          CK330228             |
|                       |           BP255287           |          BQ126878             |
|                       |           BU147155           |          AW763857             |
|                       |           BU187664           |          CF738317             |
|                       |           BM472723           |          AI874730             |
|                       |           BG118935           |          BF449871             |
|                       |           BG425008           |          AW260342             |
|                       |           CF145417           |          AI117820             |
|                       |           CF145457           |          BB771511             |
|                       |           CF145745           |          AW537427             |
|                       |           CF145681           |          BB767233             |
|                       |           BM466339           |          BM232394             |
|                       |           BQ673504           |          BB821332             |
|                       |           BI869092           |          BB767928             |
|                       |           AA452795           |          BB283450             |
|                       |           BG750190           |          BB764090             |
|                       |           BU501224           |          BM231367             |
|                       |           BG775627           |          AI607387             |
|                       |           CB119793           |          BB771132             |
|                       |           CN413268           |          BB763226             |
|                       |           CN413261           |          BM199573             |
|                       |           CB145503           |          BB822830             |
|                       |           BP371271           |          AA413008             |
|                       |           BG119856           |          AA413009             |
|                       |           BX371821           |          AW541882             |
|                       |           BX371822           |          AW555222             |
|                       |           BG750473           |          BE533561             |
|                       |           BG910303           |          BM116042             |
|                       |           BI116020           |          BE137364             |
|                       |           CA395743           |          BX517882             |
|                       |           BG392516           |          BQ031880             |
|                       |           CF122869           |          BE945840             |
|                       |           CF146574           |          BF464475             |
|                       |           BG674705           |          BE945016             |
|                       |           BG752835           |          BQ126571             |
|                       |           BF797183           |          AI851124             |
|                       |           CB149343           |          BU755193             |
|                       |           AA076334           |          BF451162             |
|                       |           BE904215           |          AW046260             |
|                       |           BI870839           |          BE990932             |
|                       |           CF122302           |          BE944963             |
|                       |           BM728712           |          AW259163             |
|                       |           CF122354           |          BQ033991             |
|                       |           BI252155           |          BE990691             |
|                       |           BG764336           |          BE946476             |
|                       |           BI199026           |          BI219224             |
|                       |           BQ684786           |                               |
|                       |           BQ897846           |                               |
|                       |           BQ064755           |                               |
|                       |           BE547639           |                               |
|                       |           BQ278901           |                               |
|                       |           BM749492           |                               |
|                       |           BM742061           |                               |
|                       |           AU121647           |                               |
|                       |           BM737617           |                               |
|                       |           BX410263           |                               |
|                       |           BG012277           |                               |
|                       |           CF130955           |                               |
|                       |           BG675058           |                               |
|                       |           AA311695           |                               |
|                       |           AW958617           |                               |
|                       |           BG005431           |                               |
|                       |           BG114668           |                               |
|                       |           AW250106           |                               |
|                       |           BM011732           |                               |
|                       |           CN413265           |                               |
|                       |             N49496           |                               |
|                       |           AW372919           |                               |
|                       |           BE266818           |                               |
|                       |           BQ216044           |                               |
|                       |           BM455963           |                               |
|                       |           AW406205           |                               |
|                       |           AV724142           |                               |
|                       |           BF792199           |                               |
|                       |           BE882160           |                               |
|                       |           BE562287           |                               |
|                       |           AA076333           |                               |
|                       |           BE561718           |                               |
|                       |           BM738203           |                               |
|                       |           CN413274           |                               |
|                       |           BM847335           |                               |
|                       |           BM761329           |                               |
|                       |           BM846504           |                               |
|                       |           BM846513           |                               |
|                       |           AL037990           |                               |
|                       |           CD615810           |                               |
|                       |           CD615811           |                               |
|                       |           BF734154           |                               |
|                       |           AW372915           |                               |
|                       |           BM843616           |                               |
|                       |           AA357018           |                               |
|                       |           BE268212           |                               |
|                       |           BE268119           |                               |
|                       |           BF033291           |                               |
|                       |           BQ278152           |                               |
|                       |           BE548368           |                               |
|                       |           BI055905           |                               |
|                       |           BG315173           |                               |
|                       |           BQ689133           |                               |
|                       |           BE931302           |                               |
|                       |           CN413263           |                               |
|                       |           BQ233895           |                               |
|                       |           BM563791           |                               |
|                       |           AW996275           |                               |
|                       |           AL581636           |                               |
|                       |           CN413257           |                               |
|                       |           BF796492           |                               |
|                       |             T18515           |                               |
|                       |           BE393634           |                               |
|                       |           BE379199           |                               |
|                       |           BU178182           |                               |
|                       |           CA489616           |                               |
|                       |           AA143753           |                               |
|                       |           BU154423           |                               |
|                       |           BG032057           |                               |
|                       |           BG005426           |                               |
|                       |           BQ637938           |                               |
|                       |           AL567085           |                               |
|                       |           BX386112           |                               |
|                       |           BX397578           |                               |
|                       |           BX356161           |                               |
|                       |           AL581416           |                               |
|                       |           AW602109           |                               |
|                       |           BX423111           |                               |
|                       |           AA579218           |                               |
|                       |           BE336915           |                               |
|                       |           BM801596           |                               |
|                       |           BU955934           |                               |
|                       |           BU860317           |                               |
|                       |           BU539574           |                               |
|                       |           BX375574           |                               |
|                       |           AA588378           |                               |
|                       |           BU594319           |                               |
|                       |           BF306452           |                               |
|                       |           AW069864           |                               |
|                       |           AA357019           |                               |
|                       |           AA455773           |                               |
|                       |           AA290885           |                               |
|                       |           AL569640           |                               |
|                       |           AA465599           |                               |
|                       |           AA446778           |                               |
|                       |           CN413258           |                               |
|                       |           BX461886           |                               |
|                       |           BX357622           |                               |
|                       |           BX378898           |                               |
|                       |             N45048           |                               |
|                       |           AF091075           |                               |
|                       |           BX413804           |                               |
|                       |           AA447066           |                               |
|                       |           AL513747           |                               |
|                       |           AW996253           |                               |
|                       |           AA255870           |                               |
|                       |           AL519201           |                               |
|                       |             H26223           |                               |
|                       |           AW886797           |                               |
|                       |           AA363395           |                               |
|                       |           CB122661           |                               |
|                       |           AL514981           |                               |
|                       |           BE908811           |                               |
|                       |           AL045927           |                               |
|                       |           AW796807           |                               |
|                       |           AW796899           |                               |
|                       |           BI040106           |                               |
|                       |           AL580740           |                               |
|                       |           BE787959           |                               |
|                       |             W19773           |                               |
|                       |           BM974293           |                               |
|                       |           BU742640           |                               |
|                       |           BF745475           |                               |
|                       |           BX435932           |                               |
|                       |           BM982074           |                               |
|                       |           BQ004850           |                               |
|                       |           CN413255           |                               |
|                       |           BE887524           |                               |
|                       |           BM977427           |                               |
|                       |           BM710240           |                               |
|                       |           AW603044           |                               |
|                       |           BU629849           |                               |
|                       |           BF342589           |                               |
|                       |           BG001521           |                               |
|                       |           BM979991           |                               |
|                       |           BM975397           |                               |
|                       |           AW190582           |                               |
|                       |           BX454032           |                               |
|                       |           BM746650           |                               |
|                       |           BM687364           |                               |
|                       |           BM747504           |                               |
|                       |           BX367052           |                               |
|                       |           BF740099           |                               |
|                       |           BM674077           |                               |
|                       |           CB528910           |                               |
|                       |           CF541127           |                               |
|                       |           BQ009833           |                               |
|                       |           BM830916           |                               |
|                       |           BQ006351           |                               |
|                       |           CD367269           |                               |
|                       |           CA945088           |                               |
|                       |           AI590164           |                               |
|                       |           AI184067           |                               |
|                       |           AA126531           |                               |
|                       |           BU628857           |                               |
|                       |           BF094650           |                               |
|                       |           BF094661           |                               |
|                       |           BF380193           |                               |
|                       |           BF380228           |                               |
|                       |           BF380238           |                               |
|                       |           BF094566           |                               |
|                       |           BF094817           |                               |
|                       |           BF092527           |                               |
|                       |           BF094659           |                               |
|                       |           BF380218           |                               |
|                       |           BF094539           |                               |
|                       |           BF908127           |                               |
|                       |           BF094535           |                               |
|                       |           BF094541           |                               |
|                       |           BF094794           |                               |
|                       |           BF094897           |                               |
|                       |           BF912608           |                               |
|                       |           BQ379646           |                               |
|                       |           BF094534           |                               |
|                       |           AI762491           |                               |
|                       |           BM765587           |                               |
|                       |           BF094531           |                               |
|                       |           BF094727           |                               |
|                       |           BF094797           |                               |
|                       |           BF094901           |                               |
|                       |           BF908126           |                               |
|                       |           BF908134           |                               |
|                       |           BF094820           |                               |
|                       |           BF094732           |                               |
|                       |           AA056624           |                               |
|                       |           BF094893           |                               |
|                       |           BF094731           |                               |
|                       |           CN479388           |                               |
|                       |           AI423058           |                               |
|                       |           BE767814           |                               |
|                       |           BF092707           |                               |
|                       |           BE934210           |                               |
|                       |           BE934207           |                               |
|                       |           BF094603           |                               |
|                       |           BF092535           |                               |
|                       |           BF094529           |                               |
|                       |           BF094691           |                               |
|                       |           BF094709           |                               |
|                       |           BF094853           |                               |
|                       |           BF094865           |                               |
|                       |           BF094567           |                               |
|                       |           BF094573           |                               |
|                       |           BF094608           |                               |
|                       |           BF094697           |                               |
|                       |           BF094714           |                               |
|                       |           BF094718           |                               |
|                       |           BF094722           |                               |
|                       |           BF094726           |                               |
|                       |           BF094789           |                               |
|                       |           BF094793           |                               |
|                       |           BF094816           |                               |
|                       |           BF094819           |                               |
|                       |           BF094866           |                               |
|                       |           BF912598           |                               |
|                       |           BF912602           |                               |
|                       |           BF912604           |                               |
|                       |           BF912610           |                               |
|                       |           BF912611           |                               |
|                       |           BF912612           |                               |
|                       |           BQ379644           |                               |
|                       |           BG001902           |                               |
|                       |           BF092372           |                               |
|                       |           BF094663           |                               |
|                       |           BF229313           |                               |
|                       |           BF229990           |                               |
|                       |           BF380232           |                               |
|                       |           BF094525           |                               |
|                       |           BF094648           |                               |
|                       |           BF094729           |                               |
|                       |           BF094898           |                               |
|                       |           BF380188           |                               |
|                       |           BG001899           |                               |
|                       |           BF094565           |                               |
|                       |           BF092396           |                               |
|                       |           BF092402           |                               |
|                       |           BF094546           |                               |
|                       |           BF094547           |                               |
|                       |           BF094614           |                               |
|                       |           BF094720           |                               |
|                       |           BF094735           |                               |
|                       |           BF094764           |                               |
|                       |           BF094788           |                               |
|                       |           BF094790           |                               |
|                       |           BF094864           |                               |
|                       |           BF380237           |                               |
|                       |           BF094728           |                               |
|                       |             W74542           |                               |
|                       |           AA150991           |                               |
|                       |           AI857951           |                               |
|                       |           BE934206           |                               |
|                       |           BM841153           |                               |
|                       |           BM841034           |                               |
|                       |           BM838657           |                               |
|                       |           AA026843           |                               |
|                       |           BE934209           |                               |
|                       |           BU630515           |                               |
|                       |           BX330316           |                               |
|                       |           BE934213           |                               |
|                       |           AW005756           |                               |
|                       |           BM824297           |                               |
|                       |           BM793734           |                               |
|                       |           AA485051           |                               |
|                       |           CN413256           |                               |
|                       |           BU741429           |                               |
|                       |           BU738644           |                               |
|                       |           AA706889           |                               |
|                       |           AA236988           |                               |
|                       |           BQ014964           |                               |
|                       |           BM724791           |                               |
|                       |           AU154428           |                               |
|                       |           AI151175           |                               |
|                       |           BF083887           |                               |
|                       |           AA620304           |                               |
|                       |           BG744754           |                               |
|                       |           BM701386           |                               |
|                       |           BM717173           |                               |
|                       |           BF439136           |                               |
|                       |           AA341224           |                               |
|                       |           AI889976           |                               |
|                       |           AA767036           |                               |
|                       |           AU149250           |                               |
|                       |           AW250860           |                               |
|                       |           BU689089           |                               |
|                       |           BU682504           |                               |
|                       |           CA445555           |                               |
|                       |           BE208802           |                               |
|                       |           AW575981           |                               |
|                       |           AI470019           |                               |
|                       |           CD369683           |                               |
|                       |           AI122856           |                               |
|                       |           AI680397           |                               |
|                       |           BX453979           |                               |
|                       |           AU148022           |                               |
|                       |           AU151706           |                               |
|                       |           BE464687           |                               |
|                       |           AI016300           |                               |
|                       |           AI471444           |                               |
|                       |           AI857891           |                               |
|                       |           BF094606           |                               |
|                       |           AI690684           |                               |
|                       |           BI225512           |                               |
|                       |           AA443679           |                               |
|                       |           AA432092           |                               |
|                       |           AI376193           |                               |
|                       |           AA760772           |                               |
|                       |           BE206650           |                               |
|                       |           BQ012701           |                               |
|                       |           BM672502           |                               |
|                       |           AI139610           |                               |
|                       |           BE513773           |                               |
|                       |           AA594607           |                               |
|                       |           BE312667           |                               |
|                       |           AI339706           |                               |
|                       |           AA465243           |                               |
|                       |           CB161356           |                               |
|                       |           AA648751           |                               |
|                       |           AA688110           |                               |
|                       |           AA830750           |                               |
|                       |           AI299163           |                               |
|                       |           CR745143           |                               |
|                       |           BU730294           |                               |
|                       |           BM664193           |                               |
|                       |           CF529065           |                               |
|                       |           BU729905           |                               |
|                       |           BM674945           |                               |
|                       |           AI678336           |                               |
|                       |           AW292534           |                               |
|                       |           AI623346           |                               |
|                       |           BF111407           |                               |
|                       |           CA434642           |                               |
|                       |           AI827272           |                               |
|                       |           BM149677           |                               |
|                       |           AA127249           |                               |
|                       |           AW088948           |                               |
|                       |           AI079594           |                               |
|                       |           AI925569           |                               |
|                       |           AA653252           |                               |
|                       |           AA476313           |                               |
|                       |           AI312638           |                               |
|                       |           AV743009           |                               |
|                       |           AA485052           |                               |
|                       |           AI784011           |                               |
|                       |           AA665587           |                               |
|                       | *Seq. mapped in strand Minus*| *Seq. mapped in strand Minus* |
|                       |           BG165103           |          BB843535             |
+-----------------------+------------------------------+-------------------------------+
|      #  165           | *Seq. mapped in strand Plus* | *Seq. mapped in strand Plus*  |
|                       |             J04513           |          AK031663             |
|                       |             M27968           |                               |
|                       |             M17599           |                               |
|                       | *Seq. mapped in strand Minus*| *Seq. mapped in strand Minus* |
|                       |           AF019633           |          BC027267             |
|                       |           AF019632           |          AF453428             |
|                       |           BC009842           |          AF453427             |
+-----------------------+------------------------------+-------------------------------+
|      #  166           | *Seq. mapped in strand Plus* | *Seq. mapped in strand Plus*  |
|                       |           BC054478           |          AK005721             |
|                       |           AK127982           |                               |
|                       |           BC029655           |                               |
|                       | *Seq. mapped in strand Minus*| *Seq. mapped in strand Minus* |
|                       |           AJ621583           |          AB059554             |
+-----------------------+------------------------------+-------------------------------+
|      #  167           | *Seq. mapped in strand Plus* | *Seq. mapped in strand Plus*  |
|                       |           CB992788           |          CA466397             |
|                       | *Seq. mapped in strand Minus*| *Seq. mapped in strand Minus* |
|                       |           AI469319           |          AB079067             |
|                       |           BX108457           |          BQ443082             |
|                       |           AA911990           |          BB668026             |
|                       |           CN426213           |          BE533775             |
|                       |           BU689070           |          BG861555             |
|                       |           BF175632           |          BF323091             |
|                       |           AI702322           |          CF540050             |
|                       |           AI932585           |          BQ442234             |
|                       |           AW575682           |          AI414417             |
|                       |           AA598921           |          AI536375             |
|                       |           AI022492           |          BM246922             |
|                       |           BU735548           |          AK042077             |
|                       |             N32871           |          BB235873             |
|                       |           AI131493           |            W42088             |
|                       |           AA808169           |          BB794348             |
|                       |           BM667590           |          CB246749             |
|                       |             N32016           |          CA578972             |
|                       |           AA522448           |          CF535441             |
|                       |           AW574563           |          CA579952             |
|                       |           CN480210           |          BY754042             |
|                       |           CA443049           |            R74736             |
|                       |           CR597559           |          BB638867             |
|                       |           CR609594           |          BY739924             |
|                       |           BC008382           |          BI733300             |
|                       |           AL137600           |          BY282741             |
|                       |           AK026892           |          BY271095             |
|                       |           AL832794           |          BB851537             |
|                       |           BC004278           |          BY231107             |
|                       |           AB051433           |          BB658968             |
|                       |           BU737963           |                               |
|                       |           BM983832           |                               |
|                       |           AK025083           |                               |
|                       |           CA424018           |                               |
|                       |           AB079066           |                               |
|                       |           CA771881           |                               |
|                       |           AV651636           |                               |
|                       |           CR457365           |                               |
|                       |           BU187541           |                               |
|                       |           BQ888835           |                               |
|                       |           CN296310           |                               |
|                       |           AJ457828           |                               |
|                       |           BE387095           |                               |
|                       |           CN296313           |                               |
|                       |           BC067255           |                               |
|                       |           BQ710170           |                               |
|                       |           CN296309           |                               |
|                       |           CR456404           |                               |
|                       |           CF135528           |                               |
|                       |           BX952302           |                               |
|                       |           CK000525           |                               |
|                       |           CD655311           |                               |
|                       |           CN296312           |                               |
|                       |           BU428707           |                               |
|                       |           CF138634           |                               |
|                       |           BM479389           |                               |
|                       |           BQ057191           |                               |
|                       |           CK000755           |                               |
|                       |           BQ054406           |                               |
|                       |           CN296311           |                               |
|                       |           BP224560           |                               |
|                       |             H55443           |                               |
|                       |           BU430866           |                               |
|                       |           BU430459           |                               |
|                       |           BQ063738           |                               |
|                       |           AW503999           |                               |
|                       |           CF138275           |                               |
|                       |           BP309990           |                               |
|                       |           BP310011           |                               |
|                       |           BU428926           |                               |
|                       |           AA355581           |                               |
|                       |           CD631566           |                               |
|                       |           CD631568           |                               |
|                       |           CD631567           |                               |
|                       |           CD631569           |                               |
|                       |           BQ879739           |                               |
|                       |           AW503483           |                               |
|                       |           BP274786           |                               |
|                       |           AW499858           |                               |
|                       |           AI738574           |                               |
|                       |           CD631564           |                               |
|                       |           CD631565           |                               |
+-----------------------+------------------------------+-------------------------------+
|      #  168           | *Seq. mapped in strand Plus* | *Seq. mapped in strand Plus*  |
|                       |           CA439589           |          BB642214             |
|                       |                              |          AK044290             |
|                       |                              |          AK041280             |
|                       |                              |          BB636440             |
|                       |                              |          AK079416             |
|                       |                              |          BC039225             |
|                       |                              |          BG175491             |
|                       |                              |          BY722803             |
|                       |                              |          BY063601             |
|                       |                              |          BB636956             |
|                       |                              |          AK040927             |
|                       |                              |          BI441143             |
|                       |                              |          CA315560             |
|                       |                              |          CA316634             |
|                       |                              |          CA320332             |
|                       |                              |          BI456527             |
|                       |                              |          BB203495             |
|                       |                              |          CN457208             |
|                       |                              |          BB226364             |
|                       |                              |          AA239050             |
|                       |                              |          BU610459             |
|                       |                              |          AA185109             |
|                       |                              |          AA185135             |
|                       |                              |          BQ567976             |
|                       | *Seq. mapped in strand Minus*| *Seq. mapped in strand Minus* |
|                       |           BU677165           |          BF461623             |
|                       |           AI865427           |                               |
|                       |             N39246           |                               |
|                       |           BQ029962           |                               |
|                       |           BM672985           |                               |
|                       |           BQ448557           |                               |
|                       |           BQ021139           |                               |
|                       |           AK057734           |                               |
|                       |           AL833188           |                               |
|                       |           AK056653           |                               |
|                       |           BX102842           |                               |
|                       |           AW878312           |                               |
|                       |           AA775514           |                               |
|                       |           CN479139           |                               |
|                       |           AI094304           |                               |
|                       |           CA436963           |                               |
|                       |           BC035827           |                               |
|                       |           BU736642           |                               |
|                       |           BQ181731           |                               |
|                       |           AW955745           |                               |
|                       |           AA040207           |                               |
|                       |           BC069346           |                               |
|                       |           BC069811           |                               |
|                       |           BC074887           |                               |
|                       |           CB127224           |                               |
|                       |           BF349456           |                               |
|                       |           AA366219           |                               |
|                       |           BI255004           |                               |
|                       |           CN270887           |                               |
|                       |           BF172505           |                               |
|                       |           CF272335           |                               |
|                       |           AV747450           |                               |
|                       |           CD637741           |                               |
|                       |           CD637742           |                               |
|                       |           CD637737           |                               |
|                       |           CD637740           |                               |
|                       |           CD637739           |                               |
|                       |           BP208326           |                               |
|                       |           CD637738           |                               |
|                       |           BP332114           |                               |
|                       |           AV704739           |                               |
|                       |           BP348778           |                               |
|                       |           BX500769           |                               |
|                       |           CD637733           |                               |
|                       |           CD637731           |                               |
|                       |           CD637729           |                               |
|                       |           CD637732           |                               |
|                       |           CD637730           |                               |
|                       |           CD637734           |                               |
|                       |           CD637736           |                               |
|                       |           CD703147           |                               |
|                       |           AA876450           |                               |
|                       |           AI024594           |                               |
+-----------------------+------------------------------+-------------------------------+
|      #  169           | *Seq. mapped in strand Plus* | *Seq. mapped in strand Plus*  |
|                       |           CF886989           |          BF462691             |
|                       | *Seq. mapped in strand Minus*| *Seq. mapped in strand Minus* |
|                       |             N91843           |          BB623646             |
|                       |           AA732565           |          AI844440             |
|                       |           AW006525           |          BF461437             |
|                       |           AI400647           |          AI852185             |
|                       |           AI300197           |          CF383551             |
|                       |           AI682368           |          CK381148             |
|                       |           AA714021           |          CB523723             |
|                       |           AI805961           |          CB181495             |
|                       |           AI400950           |          BU514233             |
|                       |           CA414580           |          BG228780             |
|                       |           AI086725           |          BM210174             |
|                       |           AI623538           |          BY758306             |
|                       |           CF528533           |          BY758272             |
|                       |           BU624680           |          CA451015             |
|                       |             W46144           |          BU516985             |
|                       |           BU681885           |          BU840969             |
|                       |           AI742008           |          BC025161             |
|                       |           CA432412           |          CA323622             |
|                       |           CN479772           |          BB667235             |
|                       |           BM970246           |          AF479288             |
|                       |           BM907469           |          BY703781             |
|                       |           BU753882           |          BI251703             |
|                       |           BC075808           |          AK003520             |
|                       |           BC007321           |          BF179121             |
|                       |           BU634276           |          CK789109             |
|                       |           CB529674           |          BQ445703             |
|                       |           CD364679           |          BF788782             |
|                       |           CD369410           |          BF607316             |
|                       |           BU688397           |          AA518619             |
|                       |           CR617199           |          BU935890             |
|                       |           CA307831           |          CN718625             |
|                       |           CR594092           |          CK020183             |
|                       |           BX376656           |          CB598375             |
|                       |           BX346441           |          BX511534             |
|                       |           BG109121           |          BG964642             |
|                       |           BG756882           |          BI525967             |
|                       |           BU506802           |          BE916154             |
|                       |           BQ067929           |          BI853918             |
|                       |           BQ231215           |          BF607097             |
|                       |           BX410370           |          AA050803             |
|                       |           CF455680           |          BQ746209             |
|                       |           BG337214           |          BY732366             |
|                       |           BQ049017           |          CA322438             |
|                       |           BQ674411           |          BG794510             |
|                       |           BQ428248           |          BY737990             |
|                       |           BI856796           |          BF234868             |
|                       |           BG476616           |          BG796484             |
|                       |           BX447077           |          BF781292             |
|                       |           CR604088           |          BY737953             |
|                       |           BE562371           |          CA459373             |
|                       |           BI227008           |          BI988777             |
|                       |           BE560850           |          BE282809             |
|                       |           BE545816           |          AA422646             |
|                       |           BI822004           |          AA624607             |
|                       |           BX410371           |          BE863148             |
|                       |           BI196556           |            W66691             |
|                       |           BF025656           |          BG277793             |
|                       |           BE791076           |          AW742248             |
|                       |           BM048560           |          BG077119             |
|                       |           CN366985           |          BE859743             |
|                       |           BG574647           |          AA111408             |
|                       |           BG254242           |          BX528036             |
|                       |           BE783296           |          BX521272             |
|                       |           BI858084           |          AW989326             |
|                       |           BE538318           |          AA239939             |
|                       |           CN366977           |            W82548             |
|                       |           BF125922           |          BY039315             |
|                       |           BQ218211           |          AA982991             |
|                       |           BG478794           |          AA499710             |
|                       |           CF455683           |          AA153170             |
|                       |           BE397848           |          BB862032             |
|                       |           BE893710           |            C89301             |
|                       |           BE386688           |          AA198216             |
|                       |           BI669844           |            W75345             |
|                       |           BE277828           |          BY039918             |
|                       |           BE397803           |          AA049886             |
|                       |           AI022637           |          CD564349             |
|                       |           BQ888155           |          BY059224             |
|                       |           BE277717           |          BY094880             |
|                       |           BI091894           |          BY132995             |
|                       |           CN366979           |          BY049923             |
|                       |           CN366978           |          AA646866             |
|                       |           BE267577           |          BY075804             |
|                       |           AA461220           |          BY047668             |
|                       |           BI196671           |          BY132369             |
|                       |           BE784809           |          BY051961             |
|                       |           AW363176           |          BY050382             |
|                       |           BE276444           |          BY099114             |
|                       |           AA149073           |          BY770159             |
|                       |           BE890924           |          BY076049             |
|                       |           BI822769           |          BE192680             |
|                       |           BE275850           |          BY338402             |
|                       |           CN366982           |          BY338403             |
|                       |           CA868090           |          BY131595             |
|                       |           AW958719           |          BY042519             |
|                       |             W22295           |          BE192794             |
|                       |           AI038508           |          BY087873             |
|                       |           CN366980           |          BY104731             |
|                       |           AV761654           |          BY058292             |
|                       |           CN366988           |          BY052007             |
|                       |           BM887946           |          BY780852             |
|                       |           CR456398           |          BY771960             |
|                       |           AA483830           |          BY055430             |
|                       |           BF025931           |          BY048849             |
|                       |           BG778074           |          BY111883             |
|                       |           CR746423           |          BY794490             |
|                       |           CB128691           |          BY045250             |
|                       |           BU584712           |          BY098382             |
|                       |           AW406517           |          BY149788             |
|                       |           CN366984           |          BY197534             |
|                       |             H82161           |          BY794653             |
|                       |             W49612           |          BY005711             |
|                       |           BQ335742           |          BY791244             |
|                       |           AA316936           |          AA170378             |
|                       |           AA306274           |          BB591954             |
|                       |           BF246086           |          BB567820             |
|                       |           CN366987           |          AA646841             |
|                       |             W17305           |          BY069688             |
|                       |             N64216           |          AI119888             |
|                       |           AA397638           |          AA597014             |
|                       |           AA424395           |                               |
|                       |           BP234469           |                               |
|                       |             W31137           |                               |
|                       |           AA447051           |                               |
|                       |           BM840844           |                               |
|                       |           CV026526           |                               |
|                       |           CN366989           |                               |
|                       |           CN366986           |                               |
|                       |           AA359309           |                               |
|                       |             W00540           |                               |
+-----------------------+------------------------------+-------------------------------+
|      #  170           | *Seq. mapped in strand Plus* | *Seq. mapped in strand Plus*  |
|                       |           AI089632           |          AY367765             |
|                       |                              |          BC037365             |
|                       | *Seq. mapped in strand Minus*| *Seq. mapped in strand Minus* |
|                       |           AL524234           |          BC062952             |
|                       |           CR620209           |          BC026474             |
|                       |           AA953034           |          BC037692             |
|                       |             R37580           |          BC066091             |
|                       |           AI203420           |          BC065169             |
|                       |           BX119613           |          AK035344             |
|                       |           BM998629           |          AK053628             |
|                       |           CA445164           |                               |
|                       |           BM981851           |                               |
|                       |           CB241326           |                               |
|                       |           CB306591           |                               |
|                       |           BC033636           |                               |
|                       |           AF070590           |                               |
|                       |           BC065278           |                               |
|                       |           BC053623           |                               |
|                       |           AF283512           |                               |
|                       |           AW206127           |                               |
|                       |           BM968606           |                               |
|                       |           BM970559           |                               |
|                       |           BQ070690           |                               |
|                       |           BM478308           |                               |
|                       |           BF818037           |                               |
|                       |           AW959906           |                               |
|                       |           BC039270           |                               |
|                       |             T02839           |                               |
|                       |           BM835974           |                               |
|                       |             T50337           |                               |
|                       |           BM466914           |                               |
|                       |           BM904771           |                               |
|                       |           AA326870           |                               |
|                       |           CN395596           |                               |
|                       |           CB138854           |                               |
|                       |           BE882416           |                               |
|                       |           BG118639           |                               |
|                       |           BX280394           |                               |
|                       |           BP269444           |                               |
|                       |           AW964377           |                               |
|                       |           CB139330           |                               |
|                       |           CB137907           |                               |
|                       |           AA318182           |                               |
|                       |           BF962786           |                               |
|                       |             T79997           |                               |
|                       |           BQ423120           |                               |
|                       |           BI090988           |                               |
|                       |           CN395595           |                               |
|                       |             F07775           |                               |
|                       |           AW965067           |                               |
|                       |           AA082691           |                               |
|                       |           BF970974           |                               |
|                       |           BQ962616           |                               |
|                       |           BG763759           |                               |
|                       |           BF763305           |                               |
|                       |           AI168790           |                               |
|                       |           BU189567           |                               |
|                       |           AA343694           |                               |
|                       |           AA383125           |                               |
|                       |           BU172622           |                               |
|                       |           AA307838           |                               |
|                       |           CD652962           |                               |
|                       |           BC060778           |                               |
|                       |           BQ719095           |                               |
|                       |             T61977           |                               |
|                       |           CN395594           |                               |
|                       |           AK057423           |                               |
|                       |           BM806259           |                               |
|                       |           AV750322           |                               |
|                       |           BC012454           |                               |
|                       |           BF036925           |                               |
|                       |           BI560128           |                               |
|                       |           BF333201           |                               |
|                       |           AA431613           |                               |
|                       |           CB996140           |                               |
|                       |           BF761043           |                               |
|                       |           BP229245           |                               |
+-----------------------+------------------------------+-------------------------------+
|      #  171           | *Seq. mapped in strand Plus* | *Seq. mapped in strand Plus*  |
|                       |           AF007835           |          BY005853             |
|                       |                              |          AK053179             |
|                       |                              |          BY232980             |
|                       |                              |          BY233091             |
|                       |                              |          BB664091             |
|                       |                              |          AK028957             |
|                       |                              |          BB613349             |
|                       |                              |          BY003616             |
|                       |                              |          BY781329             |
|                       |                              |          BB849771             |
|                       |                              |          BY770622             |
|                       |                              |          BY768055             |
|                       |                              |          BB603904             |
|                       |                              |          AK086121             |
|                       |                              |          BY081288             |
|                       |                              |          BX527039             |
|                       |                              |          AI664601             |
|                       |                              |          BY229683             |
|                       |                              |          BY118307             |
|                       |                              |          AI006603             |
|                       |                              |          CK620811             |
|                       |                              |          AK085225             |
|                       |                              |          AA986860             |
|                       | *Seq. mapped in strand Minus*| *Seq. mapped in strand Minus* |
|                       |           BC001943           |          BE632321             |
|                       |           BC002325           |                               |
|                       |           AK096741           |                               |
|                       |           AL832940           |                               |
|                       |           BC018959           |                               |
|                       |           BC000765           |                               |
|                       |           AK093826           |                               |
+-----------------------+------------------------------+-------------------------------+
|      #  172           | *Seq. mapped in strand Plus* | *Seq. mapped in strand Plus*  |
|                       |           BC029360           |          AK086575             |
|                       |           CR607132           |                               |
|                       |           CR600204           |                               |
|                       |           AK027067           |                               |
|                       |           BC007754           |                               |
|                       |           CR590158           |                               |
|                       |           CR602124           |                               |
|                       |           CR457372           |                               |
|                       |           AL834488           |                               |
|                       |           CR615952           |                               |
|                       | *Seq. mapped in strand Minus*| *Seq. mapped in strand Minus* |
|                       |           BC022254           |          BY206461             |
|                       |           BC000863           |                               |
|                       |           AK093757           |                               |
|                       |           AK022922           |                               |
|                       |           AF395747           |                               |
|                       |           AF395748           |                               |
|                       |           AF395749           |                               |
|                       |           AF395750           |                               |
|                       |           AF395751           |                               |
|                       |           AF395752           |                               |
|                       |           AJ296101           |                               |
|                       |           BC009185           |                               |
|                       |           BX648858           |                               |
|                       |           AF088055           |                               |
+-----------------------+------------------------------+-------------------------------+
|      #  173           | *Seq. mapped in strand Plus* | *Seq. mapped in strand Plus*  |
|                       |           BU743183           |          BB869126             |
|                       |                              |          BY709768             |
|                       |                              |          AK010301             |
|                       |                              |          CA977390             |
|                       |                              |          BY083658             |
|                       |                              |          BY012507             |
|                       |                              |          BB843476             |
|                       |                              |          BY011245             |
|                       |                              |          BY011471             |
|                       |                              |          BY338426             |
|                       |                              |          BY342724             |
|                       |                              |          BY340467             |
|                       |                              |          BY343718             |
|                       |                              |          BY233730             |
|                       |                              |          BB853103             |
|                       |                              |          BY337247             |
|                       |                              |          BY339252             |
|                       |                              |          BU522870             |
|                       |                              |          BG970293             |
|                       |                              |          CA490927             |
|                       |                              |          BY231557             |
|                       |                              |          CN684957             |
|                       |                              |          BY010324             |
|                       |                              |          BY231917             |
|                       |                              |          BY077721             |
|                       |                              |          BC021788             |
|                       |                              |          BF785167             |
|                       |                              |          BI101640             |
|                       |                              |          BY011322             |
|                       |                              |          CN679056             |
|                       |                              |          CF916169             |
|                       |                              |          CN678800             |
|                       |                              |          CN678810             |
|                       |                              |          AW319335             |
|                       |                              |          CF913548             |
|                       |                              |          CF915946             |
|                       |                              |          CF174622             |
|                       |                              |          CN681321             |
|                       |                              |          BG964335             |
|                       |                              |          BI144896             |
|                       |                              |          BY264097             |
|                       |                              |          BY257534             |
|                       |                              |          CD554319             |
|                       |                              |          BY084949             |
|                       |                              |          BY105475             |
|                       |                              |          BQ891315             |
|                       |                              |          CA480001             |
|                       |                              |          AI226720             |
|                       |                              |          AI225658             |
|                       |                              |          AW318751             |
|                       |                              |          CN680171             |
|                       |                              |          AI931432             |
|                       |                              |          CA559966             |
|                       |                              |          CA561815             |
|                       |                              |          AI597368             |
|                       |                              |          AA106388             |
|                       |                              |          CF160705             |
|                       |                              |          CF160753             |
|                       |                              |          BG078821             |
|                       |                              |          CF160698             |
|                       |                              |          CF895588             |
|                       |                              |          CK337726             |
|                       |                              |          BF538750             |
|                       |                              |          CB947908             |
|                       |                              |          BF608521             |
|                       |                              |          CK787939             |
|                       |                              |          BG972324             |
|                       |                              |          CA527798             |
|                       |                              |          CB320509             |
|                       | *Seq. mapped in strand Minus*| *Seq. mapped in strand Minus* |
|                       |           AW293233           |          AA517482             |
|                       |           BE671403           |                               |
|                       |           BM677360           |                               |
|                       |           AI274305           |                               |
|                       |           BQ773376           |                               |
|                       |           AI745402           |                               |
|                       |           AK127873           |                               |
|                       |           AK124123           |                               |
|                       |           AY369207           |                               |
|                       |           BQ447479           |                               |
|                       |           CD360030           |                               |
|                       |           AA317906           |                               |
|                       |           CN267052           |                               |
|                       |           BU508435           |                               |
+-----------------------+------------------------------+-------------------------------+
|      #  174           | *Seq. mapped in strand Plus* | *Seq. mapped in strand Plus*  |
|                       |           CB241199           |          BF719079             |
|                       | *Seq. mapped in strand Minus*| *Seq. mapped in strand Minus* |
|                       |           AL038649           |          AW990489             |
|                       |           AV703135           |          CK790582             |
|                       |           AA001201           |          CA457127             |
|                       |           BU627112           |          CK022197             |
|                       |           CA447817           |          AI842138             |
|                       |           AL520912           |          AW124114             |
|                       |           CR620250           |          AI839866             |
|                       |           CB985503           |          AI847405             |
|                       |           BF791869           |          BF017888             |
|                       |           BG112309           |          BF121384             |
|                       |           BM918358           |          BE630251             |
|                       |           AL520913           |          CF615939             |
|                       |           BG432151           |          BY412197             |
|                       |           BU154614           |          BI656258             |
|                       |           BG702910           |          AW536328             |
|                       |           BI859018           |          BU756616             |
|                       |           BE787437           |          AI841423             |
|                       |           BE546755           |          BE955831             |
|                       |           CB963588           |          BE980570             |
|                       |           BU541181           |          BF463151             |
|                       |           AW005301           |          AI841486             |
|                       |           BE560160           |          BE135871             |
|                       |           BQ683253           |          AI461987             |
|                       |           BQ073247           |          BF449904             |
|                       |           BQ896798           |          BI107614             |
|                       |           BG751113           |          CK792483             |
|                       |           BM051339           |          BE690663             |
|                       |           BG761243           |          AI838971             |
|                       |           BQ441715           |          AI322803             |
|                       |           BG341128           |          AV000445             |
|                       |           CD386006           |          CB845771             |
|                       |           BU190896           |          BY763173             |
|                       |           BU537954           |          BM206091             |
|                       |           BU944063           |          BE981341             |
|                       |           BF317293           |          CD774307             |
|                       |           BG472551           |          AW123183             |
|                       |           BG340024           |          CD776089             |
|                       |           BI598415           |          BY758068             |
|                       |           AI309610           |          CK794901             |
|                       |           BG419116           |          BE628709             |
|                       |           BG755221           |          BF681869             |
|                       |           BG420314           |          BY758645             |
|                       |           BF686088           |          CB192753             |
|                       |           BM013520           |            M29264             |
|                       |           CA432802           |          CB575163             |
|                       |           AI077744           |          CB589135             |
|                       |           AA844150           |          BQ921692             |
|                       |           AI077733           |          BQ939209             |
|                       |           AI587305           |          BC021537             |
|                       |             N27151           |          CA468426             |
|                       |           BM971822           |          CF982411             |
|                       |           BU674400           |          AW913777             |
|                       |           AW245426           |          AI226314             |
|                       |           BE904202           |          CA463426             |
|                       |           BM850033           |          CA463790             |
|                       |           BF686793           |          AK019310             |
|                       |           BF662854           |          BU946105             |
|                       |           BG758243           |          AA832979             |
|                       |           BG766361           |          BF119648             |
|                       |           BG758791           |          AA672489             |
|                       |           BI857355           |          BU524691             |
|                       |           AI274341           |          CK023149             |
|                       |           BU630786           |          BU847951             |
|                       |           CD368834           |          BI689070             |
|                       |           BE612959           |          AW825628             |
|                       |           BI160380           |          CB596455             |
|                       |           BQ923654           |          BE689675             |
|                       |           BF528604           |          BG242280             |
|                       |           BQ957833           |          BU937366             |
|                       |           BU148532           |          CF901187             |
|                       |           BG031356           |          AA016699             |
|                       |           BI116045           |          BY737755             |
|                       |           CB107637           |          BU604185             |
|                       |           CA313130           |          AA543657             |
|                       |           BM768467           |          CF106388             |
|                       |           AI040855           |          AI528806             |
|                       |           BU070324           |          BF020912             |
|                       |           AI917562           |          CA463706             |
|                       |           AI147608           |          BF682496             |
|                       |           AI193745           |          BE690245             |
|                       |           AA491683           |          BY751711             |
|                       |           AI369611           |          AA611117             |
|                       |           AA541408           |          CK623606             |
|                       |           BM988347           |          BF164619             |
|                       |             W72285           |          AA244512             |
|                       |             W94937           |          AI120303             |
|                       |           AU280103           |            W97240             |
|                       |           BU727789           |          AI020954             |
|                       |           AI992049           |          CK622618             |
|                       |           AI077892           |            W77137             |
|                       |             N26010           |          AW519425             |
|                       |           AI148664           |          CK623617             |
|                       |           AI347747           |          BY738337             |
|                       |           CA437235           |          CA325680             |
|                       |           BU677182           |          BE198701             |
|                       |           BU727663           |          CA478985             |
|                       |           CN273360           |          CF903035             |
|                       |           AV725233           |          AI746546             |
|                       |           AW162046           |          CA458207             |
|                       |           AI161360           |          CK627810             |
|                       |           BE273634           |          AA212401             |
|                       |           AW162225           |          BY712477             |
|                       |           BE729198           |          AA237653             |
|                       |           BQ787327           |          AA920757             |
|                       |           BF971840           |          AA061582             |
|                       |           BM041871           |            W75524             |
|                       |           BX437455           |          AA105057             |
|                       |           CR610559           |          BU559307             |
|                       |           BE260608           |          AA030934             |
|                       |           BI825894           |          CK625556             |
|                       |           BG757148           |          BI990434             |
|                       |           BG701309           |          AA268075             |
|                       |           BF975839           |          AA521517             |
|                       |           BG759195           |          AA014935             |
|                       |           BU170660           |            W82068             |
|                       |           BG031267           |          CA464262             |
|                       |           BI871133           |          AA238042             |
|                       |           CN273374           |          CA493480             |
|                       |             U07857           |          CB951994             |
|                       |           BF127680           |          AA871334             |
|                       |           AL551966           |            W65645             |
|                       |           CR610442           |          AA016571             |
|                       |           AI174841           |          BU743940             |
|                       |           BG774914           |          BE625367             |
|                       |           BF342280           |          AA241807             |
|                       |           BM450447           |          AA153982             |
|                       |           CN273368           |          BU921908             |
|                       |           BI603524           |          CF106625             |
|                       |           BU163498           |          AA268672             |
|                       |           BG418976           |          CA528604             |
|                       |           BI093226           |          AA572428             |
|                       |           BX446345           |          CF982712             |
|                       |           BX415890           |          AA691161             |
|                       |           CR610388           |          BG802340             |
|                       |           BG768973           |          BE626491             |
|                       |           BF971505           |          CN839156             |
|                       |           BG333782           |          BQ567403             |
|                       |           BG708363           |          AA693168             |
|                       |           BI544677           |          CN834938             |
|                       |           BI545058           |          BQ129812             |
|                       |           BI603853           |          CK137114             |
|                       |           BG707886           |            W67086             |
|                       |           BU941326           |          AA546679             |
|                       |           BG772029           |            W34264             |
|                       |           CR614758           |          BE625940             |
|                       |           AV715878           |          AL361794             |
|                       |           BG703732           |          AI047186             |
|                       |           BM795107           |          BU551660             |
|                       |           AV738069           |          BE654318             |
|                       |           BM857546           |          BU847729             |
|                       |           BQ015130           |          BX522794             |
|                       |           BU733082           |          AI746862             |
|                       |           CA440223           |          AW987750             |
|                       |           BP378584           |          AW743039             |
|                       |           BF435019           |          AA073037             |
|                       |           BU681028           |            W13138             |
|                       |           BQ674070           |          AA014062             |
|                       |           BQ879121           |            W30500             |
|                       |           BU150165           |          AI048487             |
|                       |           BQ028669           |          BG101417             |
|                       |           BU675712           |          AA821674             |
|                       |           CF129893           |          CN673879             |
|                       |           BU628704           |          CA540538             |
|                       |           CA414067           |            C89235             |
|                       |           CB528736           |          BG100384             |
|                       |           CB854003           |          AA870817             |
|                       |           BQ614165           |          AI892742             |
|                       |           BF115676           |          AI154240             |
|                       |           BU787580           |          BF117020             |
|                       |           CD367261           |          BF018585             |
|                       |           CD742803           |          BY248339             |
|                       |           BF115286           |            W98667             |
|                       |           CA425152           |          BE849497             |
|                       |           BU730624           |          BE134941             |
|                       |           BU688111           |          BE854120             |
|                       |           CA412807           |          BY039239             |
|                       |           CA428143           |          BY243315             |
|                       |           AA706314           |            W61914             |
|                       |           BG340005           |          AW210259             |
|                       |           BM969224           |          BY259703             |
|                       |           BM974039           |          BY271695             |
|                       |           BQ180979           |          BY274912             |
|                       |           BQ424484           |          BY224503             |
|                       |           BQ681656           |          BY291327             |
|                       |           BQ892686           |          BY218558             |
|                       |           BU174568           |            W35790             |
|                       |           BU625375           |          BY245923             |
|                       |           BU688091           |          BY224413             |
|                       |           CA411802           |          BY256497             |
|                       |           CD367801           |          BY268952             |
|                       |           BQ888362           |          BY269917             |
|                       |           BQ671182           |          BY297832             |
|                       |           BM423899           |          BF017723             |
|                       |           BQ675729           |            W11403             |
|                       |           BM463038           |          BY225926             |
|                       |           BQ673295           |          AL361941             |
|                       |           BQ233173           |          BY171374             |
|                       |           AI114452           |          BY157230             |
|                       |           BQ960041           |          BY295882             |
|                       |           BQ061193           |          BY260220             |
|                       |           BQ066569           |          AA958999             |
|                       |           CB528637           |          BY291961             |
|                       |             X73459           |          BY089735             |
|                       |           BQ938275           |          BY050034             |
|                       |           BU194397           |          BY133038             |
|                       |           BU616352           |          BY348781             |
|                       |           BC071716           |          BY056092             |
|                       |           BQ953178           |          BY036305             |
|                       |           BU160830           |          BY044803             |
|                       |           BU180337           |          BU552311             |
|                       |           BP372552           |          CF356763             |
|                       |           BQ672877           |          BY230668             |
|                       |           BM452349           |          BF449475             |
|                       |           BM542808           |          BY042355             |
|                       |           CD107753           |          BY279902             |
|                       |           CB529001           |          BB848168             |
|                       |           BQ070502           |          BY054280             |
|                       |           BU943870           |          BY054125             |
|                       |           BE973595           |          BY344835             |
|                       |           BC035495           |          BY112177             |
|                       |           BU156777           |          BY114709             |
|                       |           BU176588           |          BY113110             |
|                       |           BU596006           |          BF147143             |
|                       |           BC051006           |          BY324880             |
|                       |           BU596837           |          BY057299             |
|                       |           BU601239           |          BY327007             |
|                       |           BG339783           |          BY344477             |
|                       |           BQ052046           |          BY184883             |
|                       |           BU934990           |          BY140523             |
|                       |           AB061546           |          BY292804             |
|                       |           AV741783           |          BY127207             |
|                       |           AI824106           |          BY127442             |
|                       |           BE049433           |          BY328495             |
|                       |           BE253827           |          BY334823             |
|                       |           BU940842           |          BY055710             |
|                       |           AI870421           |          BY056672             |
|                       |           BX398601           |          BY345299             |
|                       |           BQ278407           |          BY345300             |
|                       |           AV741038           |          BY127356             |
|                       |           BE562852           |          BY109599             |
|                       |           BU534135           |          BY148515             |
|                       |           BU943679           |          CK794284             |
|                       |           BU954005           |          BB598713             |
|                       |           AI925060           |          BY122449             |
|                       |           BE547755           |          CN704308             |
|                       |           BU932876           |          BY281867             |
|                       |           BU932940           |          BY295525             |
|                       |           BE898412           |          BY000881             |
|                       |           BE898935           |          BY000137             |
|                       |           BU928233           |          BY217292             |
|                       |           BU954918           |          BY053579             |
|                       |           CB985439           |          BY035945             |
|                       |           BF036651           |            W18077             |
|                       |           BI252509           |          CB274434             |
|                       |           BG717586           |          BY333865             |
|                       |           BU845165           |                               |
|                       |           BE794686           |                               |
|                       |           CN273351           |                               |
|                       |           BU943115           |                               |
|                       |           BF038961           |                               |
|                       |           BI084449           |                               |
|                       |           BE895058           |                               |
|                       |           BG166611           |                               |
|                       |           BG420306           |                               |
|                       |           CN273353           |                               |
|                       |           BM791908           |                               |
|                       |           BG685565           |                               |
|                       |           CN273355           |                               |
|                       |           BI599620           |                               |
|                       |           CN273376           |                               |
|                       |           BU954840           |                               |
|                       |           BF679296           |                               |
|                       |           AW160722           |                               |
|                       |           BX437454           |                               |
|                       |           BX351316           |                               |
|                       |           BE787217           |                               |
|                       |           BU174898           |                               |
|                       |           BQ219256           |                               |
|                       |           BG284327           |                               |
|                       |           BX415889           |                               |
|                       |           AL551939           |                               |
|                       |           BM914172           |                               |
|                       |           BI547538           |                               |
|                       |           AV762660           |                               |
|                       |           AF063571           |                               |
|                       |           AI110696           |                               |
|                       |           CK001436           |                               |
|                       |           CF128301           |                               |
|                       |           BU859948           |                               |
|                       |           AL710812           |                               |
|                       |           BF568204           |                               |
|                       |           CD678216           |                               |
|                       |           BQ306175           |                               |
|                       |           BE278872           |                               |
|                       |           AA195929           |                               |
|                       |           BI035445           |                               |
|                       |           BG484211           |                               |
|                       |           BG620877           |                               |
|                       |           AV733443           |                               |
|                       |           BE252156           |                               |
|                       |           AV758764           |                               |
|                       |           BU927755           |                               |
|                       |           BU532742           |                               |
|                       |           BM825315           |                               |
|                       |           BM761915           |                               |
|                       |           BG775377           |                               |
|                       |           BF029286           |                               |
|                       |           CN273347           |                               |
|                       |           BE618632           |                               |
|                       |           BI254908           |                               |
|                       |           BF679526           |                               |
|                       |           BE887662           |                               |
|                       |           BG482688           |                               |
|                       |           BE277451           |                               |
|                       |           BF035557           |                               |
|                       |           BG438190           |                               |
|                       |           AL553660           |                               |
|                       |           CR596116           |                               |
|                       |           CR745883           |                               |
|                       |           BE613424           |                               |
|                       |           BM019141           |                               |
|                       |           CD639641           |                               |
|                       |           BG420227           |                               |
|                       |           AV734022           |                               |
|                       |           BG759813           |                               |
|                       |           BG570387           |                               |
|                       |           CN273361           |                               |
|                       |           BE895512           |                               |
|                       |           BE278028           |                               |
|                       |           CD678478           |                               |
|                       |           CD105098           |                               |
|                       |           BI252939           |                               |
|                       |           BF683912           |                               |
|                       |           BM042606           |                               |
|                       |           BE876314           |                               |
|                       |           AW860704           |                               |
|                       |           AI444600           |                               |
|                       |           BU662333           |                               |
|                       |           BG751118           |                               |
|                       |           BF038032           |                               |
|                       |           BI545133           |                               |
|                       |           BX104554           |                               |
|                       |           BQ720879           |                               |
|                       |           BG333113           |                               |
|                       |           CB984511           |                               |
|                       |           BM687512           |                               |
|                       |           BP316556           |                               |
|                       |           BX433497           |                               |
|                       |           BM687576           |                               |
|                       |           AI061625           |                               |
|                       |           BU533719           |                               |
|                       |           BI085054           |                               |
|                       |           BQ639305           |                               |
|                       |           CK003560           |                               |
|                       |           BQ550008           |                               |
|                       |           BF678680           |                               |
|                       |           CN273362           |                               |
|                       |           BP347309           |                               |
|                       |           BE251217           |                               |
|                       |           BP297890           |                               |
|                       |           AV747397           |                               |
|                       |           AL703328           |                               |
|                       |           CN273365           |                               |
|                       |           BU787856           |                               |
|                       |           AA196093           |                               |
|                       |           BM912598           |                               |
|                       |           BM739518           |                               |
|                       |           BE258081           |                               |
|                       |           CA338134           |                               |
|                       |           BM821939           |                               |
|                       |             U92988           |                               |
|                       |           BG471331           |                               |
|                       |           BP316597           |                               |
|                       |           CK002334           |                               |
|                       |           CN273348           |                               |
|                       |           BP319072           |                               |
|                       |           AL709837           |                               |
|                       |           BU662749           |                               |
|                       |           BP329975           |                               |
|                       |           BM694212           |                               |
|                       |           BF973530           |                               |
|                       |           BG766298           |                               |
|                       |           BI598938           |                               |
|                       |           BU662761           |                               |
|                       |           BP227710           |                               |
|                       |           BG433316           |                               |
|                       |           BX471679           |                               |
|                       |           BU953410           |                               |
|                       |           BG616374           |                               |
|                       |           CB957159           |                               |
|                       |           BP228302           |                               |
|                       |           BP341450           |                               |
|                       |           AW160430           |                               |
|                       |           BP341040           |                               |
|                       |           BE279233           |                               |
|                       |           AL712397           |                               |
|                       |           AV762521           |                               |
|                       |           AA225874           |                               |
|                       |           BI195644           |                               |
|                       |           BI160042           |                               |
|                       |           CN273349           |                               |
|                       |           BP347381           |                               |
|                       |           BP317878           |                               |
|                       |           BU942265           |                               |
|                       |           BP198030           |                               |
|                       |           BP211893           |                               |
|                       |           CB110441           |                               |
|                       |           BF243071           |                               |
|                       |           BP374223           |                               |
|                       |             W31049           |                               |
|                       |           BP341291           |                               |
|                       |           BP374224           |                               |
|                       |           AL712500           |                               |
|                       |           AV759805           |                               |
|                       |           AV744981           |                               |
|                       |           AV740536           |                               |
|                       |           BP360472           |                               |
|                       |           BP319376           |                               |
|                       |           BP262305           |                               |
|                       |           CN273373           |                               |
|                       |           BE000890           |                               |
|                       |           BP213586           |                               |
|                       |           BG943922           |                               |
|                       |           BP330502           |                               |
|                       |           BM694394           |                               |
|                       |           BP229525           |                               |
|                       |           BP227914           |                               |
|                       |           BP342589           |                               |
|                       |           CN273370           |                               |
|                       |           BU537619           |                               |
|                       |           BP213641           |                               |
|                       |             W07197           |                               |
|                       |           BP264471           |                               |
|                       |           AW385238           |                               |
|                       |           AL038648           |                               |
|                       |           CB160758           |                               |
|                       |           BM772291           |                               |
|                       |           BQ308560           |                               |
|                       |           BF975804           |                               |
|                       |           AW580505           |                               |
|                       |           AL698014           |                               |
|                       |           BG426453           |                               |
|                       |           AW245323           |                               |
|                       |           AV711799           |                               |
|                       |           CD685346           |                               |
|                       |           CD700430           |                               |
|                       |           CN273369           |                               |
|                       |           BX498452           |                               |
|                       |           BM740383           |                               |
|                       |           BX642005           |                               |
|                       |           BE208540           |                               |
|                       |           BM773275           |                               |
|                       |           CB138839           |                               |
|                       |           CB157804           |                               |
|                       |           CD684640           |                               |
|                       |           CD694555           |                               |
|                       |           AA187197           |                               |
|                       |           BP248842           |                               |
|                       |           CD701963           |                               |
|                       |             W92228           |                               |
|                       |           BF093487           |                               |
|                       |           BU180031           |                               |
|                       |             W44851           |                               |
|                       |           AA312700           |                               |
|                       |           BE818133           |                               |
|                       |           BF880358           |                               |
|                       |           AW370437           |                               |
|                       |           CD694623           |                               |
|                       |           AW239417           |                               |
|                       |           BG107491           |                               |
|                       |           CN273377           |                               |
|                       |           BM826943           |                               |
|                       |             W00442           |                               |
|                       |           AV758588           |                               |
|                       |           BP232183           |                               |
|                       |           CD694772           |                               |
|                       |             H52485           |                               |
|                       |           BF734461           |                               |
|                       |           CD706593           |                               |
|                       |             N40089           |                               |
|                       |           AV759221           |                               |
|                       |           BG254630           |                               |
|                       |           AA308597           |                               |
|                       |           AA211231           |                               |
|                       |           AW842629           |                               |
|                       |           AW842640           |                               |
|                       |             T68649           |                               |
|                       |           AW866869           |                               |
|                       |           AW068521           |                               |
|                       |           BM850985           |                               |
|                       |           AW607668           |                               |
|                       |             N35939           |                               |
|                       |           AW161600           |                               |
|                       |           CD696984           |                               |
|                       |           AW879683           |                               |
|                       |             H02472           |                               |
|                       |           CB108900           |                               |
|                       |           AA602161           |                               |
|                       |           BM755375           |                               |
|                       |             R24911           |                               |
|                       |           AA197258           |                               |
|                       |           AA092717           |                               |
|                       |           BM705487           |                               |
|                       |           AA384671           |                               |
|                       |             T18962           |                               |
|                       |           AV759679           |                               |
|                       |           CN273358           |                               |
|                       |           CA388857           |                               |
|                       |             W69564           |                               |
|                       |           AV754013           |                               |
|                       |           AV753960           |                               |
|                       |           AA525321           |                               |
|                       |           AA095367           |                               |
|                       |           AA082073           |                               |
|                       |           AJ714535           |                               |
|                       |             N36642           |                               |
|                       |           AJ714149           |                               |
|                       |           AA837219           |                               |
|                       |           BE769479           |                               |
|                       |           AA570366           |                               |
|                       |           AA996261           |                               |
|                       |             R76926           |                               |
|                       |           BG472826           |                               |
|                       |           CA388854           |                               |
|                       |           CB120878           |                               |
|                       |           CD709434           |                               |
|                       |             N85778           |                               |
|                       |           BF084969           |                               |
|                       |           AW604043           |                               |
|                       |           AV734084           |                               |
|                       |           BU536658           |                               |
|                       |           CN273372           |                               |
|                       |             D55732           |                               |
|                       |           AV744790           |                               |
|                       |           AA090055           |                               |
|                       |           CA337670           |                               |
|                       |             T71260           |                               |
|                       |           AA526288           |                               |
|                       |           CA337683           |                               |
|                       |             H46807           |                               |
|                       |           BF915340           |                               |
|                       |           BF825211           |                               |
|                       |           BF913640           |                               |
|                       |           BU565502           |                               |
|                       |             N99036           |                               |
|                       |           AA526714           |                               |
|                       |           AI174271           |                               |
|                       |           BM676328           |                               |
|                       |             R57446           |                               |
|                       |           CB106486           |                               |
|                       |           AA852650           |                               |
|                       |           CN273345           |                               |
|                       |           BF913210           |                               |
|                       |           BF915343           |                               |
|                       |           BQ379689           |                               |
|                       |           BF913636           |                               |
|                       |           AA359889           |                               |
|                       |           AA090825           |                               |
|                       |           AA385573           |                               |
|                       |           BF915318           |                               |
|                       |           CD707903           |                               |
|                       |           CD709818           |                               |
|                       |           BP213755           |                               |
|                       |             T71393           |                               |
|                       |             R57678           |                               |
|                       |           AU099486           |                               |
|                       |             N86022           |                               |
|                       |           CN273367           |                               |
|                       |           CB107325           |                               |
|                       |           BF326382           |                               |
|                       |           AA378107           |                               |
|                       |           AA148272           |                               |
|                       |           BI823349           |                               |
|                       |             N43746           |                               |
|                       |           BF949324           |                               |
|                       |           CD686873           |                               |
|                       |             H93221           |                               |
|                       |           CN273357           |                               |
|                       |             N47041           |                               |
|                       |           AA385875           |                               |
|                       |           CN273352           |                               |
|                       |           CN273378           |                               |
|                       |           BF174171           |                               |
|                       |           BF919735           |                               |
|                       |           AA001200           |                               |
|                       |           AW995155           |                               |
|                       |           AA319274           |                               |
|                       |             N85197           |                               |
|                       |           AW407303           |                               |
|                       |           AA089922           |                               |
|                       |           BU662734           |                               |
|                       |           CA946542           |                               |
+-----------------------+------------------------------+-------------------------------+
|      #  175           | *Seq. mapped in strand Plus* | *Seq. mapped in strand Plus*  |
|                       |           AK000780           |          BC013093             |
|                       |           AF367998           |          BC031772             |
|                       |           AF309777           |          AF315511             |
|                       |           BC049210           |          AK018125             |
|                       |           CR617202           |                               |
|                       |           AK092293           |                               |
|                       |           CR623235           |                               |
|                       | *Seq. mapped in strand Minus*| *Seq. mapped in strand Minus* |
|                       |           AF282594           |            D88769             |
|                       |           BC008438           |          AK032170             |
|                       |           AF087851           |                               |
|                       |           AF282626           |                               |
|                       |           BC005346           |                               |
|                       |           CR605027           |                               |
|                       |           CR622283           |                               |
+-----------------------+------------------------------+-------------------------------+
|      #  176           | *Seq. mapped in strand Plus* | *Seq. mapped in strand Plus*  |
|                       |           BF966277           |          AK080343             |
|                       |           BP362709           |                               |
|                       |           CD615599           |                               |
|                       |           AL583212           |                               |
|                       |           BM147695           |                               |
|                       |           BP364126           |                               |
|                       |           BM787479           |                               |
|                       |           BP235826           |                               |
|                       |           CB144776           |                               |
|                       |           AU124083           |                               |
|                       |           BI601483           |                               |
|                       |           AU131634           |                               |
|                       |           BP350747           |                               |
|                       |           AU131902           |                               |
|                       |           BM800801           |                               |
|                       |           BI463731           |                               |
|                       |           BG721106           |                               |
|                       |           BI464318           |                               |
|                       |           BE890882           |                               |
|                       |           CD699592           |                               |
|                       |           BU660693           |                               |
|                       |           BG721577           |                               |
|                       |           BG721978           |                               |
|                       |           BG721578           |                               |
|                       |           CB961776           |                               |
|                       |           BM754896           |                               |
|                       |           BX956340           |                               |
|                       |           BI599237           |                               |
|                       |           BX442986           |                               |
|                       |           BU661050           |                               |
|                       |           AL706010           |                               |
|                       |           BX956466           |                               |
|                       |           BG720313           |                               |
|                       |           BI462834           |                               |
|                       |           BI464213           |                               |
|                       |           BG716956           |                               |
|                       |           BF979289           |                               |
|                       |           BI562680           |                               |
|                       |           BC040483           |                               |
|                       |           BM787136           |                               |
|                       |           BM788286           |                               |
|                       |           AU131233           |                               |
|                       |           BC030019           |                               |
|                       |           BM560319           |                               |
|                       |           BQ937780           |                               |
|                       |           AL039291           |                               |
|                       |           BX421944           |                               |
|                       |           BI829341           |                               |
|                       |           AL560163           |                               |
|                       |           BM800223           |                               |
|                       |           BX460766           |                               |
|                       |           BM803798           |                               |
|                       |             Y00971           |                               |
|                       |           BQ229580           |                               |
|                       |           CB215654           |                               |
|                       |           BE884559           |                               |
|                       |           BE789151           |                               |
|                       |           BI523978           |                               |
|                       |           BI828222           |                               |
|                       |           CD359297           |                               |
|                       |           BQ422609           |                               |
|                       |           BM563647           |                               |
|                       |           CA431113           |                               |
|                       |           AL703105           |                               |
|                       |           BU658900           |                               |
|                       |           CD615598           |                               |
|                       |           AL045818           |                               |
|                       |           BF541825           |                               |
|                       |           BX349370           |                               |
|                       |           BG875493           |                               |
|                       |           BG165372           |                               |
|                       |           CF265099           |                               |
|                       |           BX487480           |                               |
|                       |             D55177           |                               |
|                       |           BI752392           |                               |
|                       |           BX358581           |                               |
|                       |             H11974           |                               |
|                       |             H15341           |                               |
|                       |           BF351561           |                               |
|                       |           BE170081           |                               |
|                       |           BF819172           |                               |
|                       |           BQ435745           |                               |
|                       |           BG771367           |                               |
|                       |           BM794626           |                               |
|                       |             F05348           |                               |
|                       |           BE169497           |                               |
|                       |           BP431404           |                               |
|                       |           BF028322           |                               |
|                       |             H17691           |                               |
|                       | *Seq. mapped in strand Minus*| *Seq. mapped in strand Minus* |
|                       |           AW014095           |          AK045007             |
+-----------------------+------------------------------+-------------------------------+
|      #  177           | *Seq. mapped in strand Plus* | *Seq. mapped in strand Plus*  |
|                       |           BC025318           |          AK082761             |
|                       |           BC062998           |          BC034517             |
|                       |           BC073841           |            U97572             |
|                       |           AK092481           |          AK004652             |
|                       | *Seq. mapped in strand Minus*| *Seq. mapped in strand Minus* |
|                       |           CR613691           |          BC017147             |
|                       |                              |          BC020422             |
|                       |                              |                               |
|                       |                              |                               |
+-----------------------+------------------------------+-------------------------------+
|      #  178           | *Seq. mapped in strand Plus* | *Seq. mapped in strand Plus*  |
|                       |           BP327286           |          BY784918             |
|                       |           CN421404           |          BY084138             |
|                       |           BX397193           |          BY124150             |
|                       |           BX324334           |          BY007407             |
|                       |           AK095420           |          BY332446             |
|                       |           BP259769           |          BI150337             |
|                       |           BI460190           |          BB856910             |
|                       |           BG703014           |          BU560399             |
|                       |           BC022550           |          BY328583             |
|                       |           BE244043           |          BY088122             |
|                       |           AL597465           |          BY094336             |
|                       |           CN421402           |          BY311953             |
|                       |           BI459429           |          BY062469             |
|                       |           BP326309           |          BY227938             |
|                       |           CD694878           |          BY739044             |
|                       |           BC036683           |          BY103687             |
|                       |           CN421401           |          BY256718             |
|                       |           BM453911           |          CA556003             |
|                       |           BX332394           |          CA560653             |
|                       |           AA356209           |          CA561117             |
|                       |           BP291021           |          CF915648             |
|                       |           BF326358           |          CF915638             |
|                       |           CA307674           |          CF915114             |
|                       |           BX092480           |          CF915585             |
|                       |           CA429722           |          CF172787             |
|                       |           AF086322           |          BB856739             |
|                       |           BQ026247           |          BI665825             |
|                       |           BU753218           |          CA566772             |
|                       |           BU624755           |          CN836610             |
|                       |           AI340149           |          BY234608             |
|                       |           BU753820           |          BC018498             |
|                       |                              |          BY334670             |
|                       |                              |          BY328480             |
|                       |                              |          BY051330             |
|                       |                              |          BQ565776             |
|                       |                              |          BF721318             |
|                       |                              |          BE283724             |
|                       |                              |          BI661152             |
|                       |                              |          BY092821             |
|                       |                              |          BB585364             |
|                       |                              |          BB863866             |
|                       |                              |          AA762209             |
|                       |                              |          BE332556             |
|                       |                              |          BB855880             |
|                       |                              |          BY716929             |
|                       |                              |          AK017198             |
|                       |                              |          CF724619             |
|                       |                              |          CK796372             |
|                       |                              |          BY118448             |
|                       |                              |          BY034147             |
|                       |                              |          CB573516             |
|                       |                              |          BU918342             |
|                       |                              |          BE334304             |
|                       |                              |          BU513247             |
|                       |                              |          BI410901             |
|                       |                              |          AA874564             |
|                       |                              |          CF910267             |
|                       |                              |          BG148622             |
|                       |                              |          AW763351             |
|                       |                              |          BE863992             |
|                       |                              |          CF540815             |
|                       |                              |          BG175596             |
|                       |                              |          AI121634             |
|                       |                              |          AW049726             |
|                       |                              |          BQ031979             |
|                       |                              |          BU756944             |
|                       |                              |          BE948308             |
|                       |                              |          BQ033754             |
|                       |                              |          BQ174352             |
|                       |                              |          CO039094             |
|                       |                              |          AI848231             |
|                       |                              |          BE945882             |
|                       |                              |          BE955748             |
|                       |                              |          AW457435             |
|                       | *Seq. mapped in strand Minus*| *Seq. mapped in strand Minus* |
|                       |           BX332393           |          BB657504             |
|                       |           CA437548           |                               |
+-----------------------+------------------------------+-------------------------------+
|      #  179           | *Seq. mapped in strand Plus* | *Seq. mapped in strand Plus*  |
|                       |           BX103104           |          AK049068             |
|                       |           CB123723           |                               |
|                       |           AA931161           |                               |
|                       |           AI655304           |                               |
|                       |           BF511073           |                               |
|                       |           BE501771           |                               |
|                       |           AI004551           |                               |
|                       |           AI001824           |                               |
|                       |           AW779246           |                               |
|                       | *Seq. mapped in strand Minus*| *Seq. mapped in strand Minus* |
|                       |             N44027           |          AK053974             |
|                       |           BF447504           |          AF003866             |
|                       |           BU623753           |            U78315             |
|                       |             U65676           |          BC058332             |
|                       |           AA737268           |            U97149             |
|                       |           AA767705           |          BC046329             |
|                       |           CR592714           |                               |
|                       |           BX358685           |                               |
|                       |           AA700609           |                               |
|                       |           BQ029507           |                               |
|                       |           BM150584           |                               |
|                       |           AW196158           |                               |
|                       |           AW129246           |                               |
|                       |           BU729991           |                               |
|                       |           AI073349           |                               |
|                       |           AW148629           |                               |
|                       |           BQ446227           |                               |
|                       |           BC002514           |                               |
|                       |           AW294258           |                               |
|                       |           AA639743           |                               |
|                       |           AW451861           |                               |
|                       |           BE908935           |                               |
|                       |           CN479187           |                               |
|                       |           CA306870           |                               |
|                       |           BU740837           |                               |
|                       |           BU743314           |                               |
|                       |           BM974137           |                               |
|                       |           AL833734           |                               |
|                       |           BU174982           |                               |
|                       |           AI185798           |                               |
|                       |             N58067           |                               |
|                       |           AA034915           |                               |
|                       |           AI375030           |                               |
|                       |           BQ015727           |                               |
|                       |           CD367578           |                               |
|                       |           CR593191           |                               |
|                       |           BQ182498           |                               |
|                       |           AL544436           |                               |
|                       |           CR625584           |                               |
|                       |           CR609635           |                               |
|                       |           BX358992           |                               |
|                       |           CR598347           |                               |
|                       |           BX379930           |                               |
|                       |           CR593063           |                               |
|                       |           AL573400           |                               |
|                       |           CR600212           |                               |
|                       |           BI762575           |                               |
|                       |           BQ672991           |                               |
|                       |           BG397595           |                               |
|                       |           BU172907           |                               |
|                       |           BI758599           |                               |
|                       |           BX092521           |                               |
|                       |           BM909452           |                               |
|                       |           BE906179           |                               |
|                       |           CB123960           |                               |
|                       |           BG912958           |                               |
|                       |             W45188           |                               |
|                       |             W78736           |                               |
|                       |           BF902096           |                               |
|                       |           AA418773           |                               |
|                       |           CB050924           |                               |
|                       |           AA452196           |                               |
|                       |           CN264692           |                               |
|                       |           AA322777           |                               |
|                       |             T57028           |                               |
|                       |             T57027           |                               |
|                       |           BQ066148           |                               |
|                       |           BQ062574           |                               |
|                       |           AA034982           |                               |
|                       |           BX326881           |                               |
|                       |           BM917882           |                               |
|                       |           BQ898616           |                               |
|                       |           BQ066490           |                               |
|                       |           BQ278880           |                               |
|                       |           AA658197           |                               |
|                       |           BU164814           |                               |
|                       |           BQ672450           |                               |
|                       |           CN264689           |                               |
|                       |           CD607779           |                               |
|                       |           CD607780           |                               |
|                       |           CA407004           |                               |
|                       |           BQ672912           |                               |
|                       |           CA488203           |                               |
|                       |           BX409133           |                               |
|                       |           BX368869           |                               |
|                       |           BX358993           |                               |
|                       |           BX449397           |                               |
|                       |           BX397712           |                               |
|                       |           CD607794           |                               |
|                       |           AW403101           |                               |
|                       |           BG386626           |                               |
|                       |           BX347853           |                               |
|                       |           BQ671543           |                               |
|                       |           CD671991           |                               |
|                       |           AW403103           |                               |
|                       |           AL046031           |                               |
|                       |           BM857040           |                               |
|                       |           BG674099           |                               |
|                       |           BX326884           |                               |
|                       |           BG388727           |                               |
|                       |           CF131265           |                               |
|                       |           BI518436           |                               |
|                       |           CA488995           |                               |
|                       |           CF122169           |                               |
|                       |           BQ100915           |                               |
|                       |           BI966698           |                               |
|                       |           BU620583           |                               |
|                       |           BI962849           |                               |
|                       |           BX647296           |                               |
|                       |           BC000175           |                               |
|                       |           AL578713           |                               |
|                       |           BX447384           |                               |
|                       |           BU622715           |                               |
|                       |           CR596924           |                               |
|                       |             U96721           |                               |
|                       |           BX430563           |                               |
|                       |           AW135768           |                               |
|                       |           AA969804           |                               |
|                       |           BU632705           |                               |
|                       |           AI624016           |                               |
|                       |           BX454349           |                               |
|                       |           AI280162           |                               |
|                       |           AW469521           |                               |
|                       |           BU629693           |                               |
|                       |           BQ229013           |                               |
|                       |           AA868926           |                               |
|                       |           BX368868           |                               |
|                       |           BQ706048           |                               |
|                       |           BQ101226           |                               |
|                       |           BX358686           |                               |
|                       |           BQ307313           |                               |
|                       |           BU196145           |                               |
|                       |           CF144630           |                               |
|                       |           BX406979           |                               |
|                       |           BQ063865           |                               |
|                       |           AL544466           |                               |
|                       |           BM824942           |                               |
|                       |           BX424906           |                               |
|                       |           CV029676           |                               |
|                       |           BX439137           |                               |
|                       |           BG479953           |                               |
|                       |           AL549061           |                               |
|                       |           BQ063615           |                               |
|                       |           BG120489           |                               |
|                       |           BI757867           |                               |
|                       |           BM128017           |                               |
|                       |           BQ072743           |                               |
|                       |           BM128530           |                               |
|                       |           CN264688           |                               |
|                       |           CK819290           |                               |
|                       |           BI771946           |                               |
|                       |           BI839009           |                               |
|                       |           CN264691           |                               |
|                       |           BQ212862           |                               |
|                       |           CB991418           |                               |
|                       |           BE905685           |                               |
|                       |           BG745677           |                               |
|                       |           BP202450           |                               |
|                       |           BP288150           |                               |
|                       |           BP379893           |                               |
|                       |           BI819303           |                               |
|                       |           BP213699           |                               |
|                       |           BQ223278           |                               |
|                       |           BM907992           |                               |
|                       |           BI014831           |                               |
|                       |           BI908168           |                               |
|                       |           BQ064846           |                               |
|                       |           BF667830           |                               |
|                       |           BI832079           |                               |
|                       |           BI465342           |                               |
|                       |           BM928379           |                               |
|                       |           BM761071           |                               |
|                       |           BM791684           |                               |
|                       |           BP202649           |                               |
|                       |           BP284302           |                               |
|                       |           AA625544           |                               |
|                       |           BE279240           |                               |
|                       |           BP295616           |                               |
|                       |           BP300658           |                               |
|                       |           CB143226           |                               |
|                       |           AL702912           |                               |
|                       |           BG714748           |                               |
|                       |           BM752871           |                               |
|                       |           AW675059           |                               |
|                       |           CD607781           |                               |
|                       |           CD607783           |                               |
|                       |           CD607785           |                               |
|                       |           CD607787           |                               |
|                       |           CD607789           |                               |
|                       |           BM821414           |                               |
|                       |           CD607786           |                               |
|                       |           CD607784           |                               |
|                       |           CD607791           |                               |
|                       |           CD607792           |                               |
|                       |           CD607782           |                               |
|                       |           CD607788           |                               |
|                       |           CD607790           |                               |
|                       |           BM845677           |                               |
|                       |           BX451587           |                               |
|                       |           AA176990           |                               |
|                       |           CR620028           |                               |
|                       |           AA056025           |                               |
|                       |           CN264690           |                               |
|                       |           BG719278           |                               |
|                       |           BP225340           |                               |
|                       |           AA309931           |                               |
|                       |           BM840138           |                               |
|                       |           BP316187           |                               |
|                       |           BG717697           |                               |
|                       |           BG478044           |                               |
|                       |           BE247540           |                               |
|                       |           CA390049           |                               |
|                       |           AU099789           |                               |
|                       |             T48994           |                               |
|                       |           CB992853           |                               |
|                       |           CD607795           |                               |
|                       |           CD607797           |                               |
|                       |           CD607798           |                               |
+-----------------------+------------------------------+-------------------------------+
|      #  180           | *Seq. mapped in strand Plus* | *Seq. mapped in strand Plus*  |
|                       |           BM553921           |          AI852668             |
|                       |           BC047306           |                               |
|                       |           AK091588           |                               |
|                       |           BM546217           |                               |
|                       |           BM728736           |                               |
|                       |           BX956124           |                               |
|                       |           BG563388           |                               |
|                       |           AW328507           |                               |
|                       |           BM547483           |                               |
|                       |           AY358082           |                               |
|                       |           BP258176           |                               |
|                       |           BI755824           |                               |
|                       |           BI756732           |                               |
|                       |           BF694943           |                               |
|                       |           BP371674           |                               |
|                       |           AL514580           |                               |
|                       |           BF692909           |                               |
|                       |           BE908546           |                               |
|                       |           BP352159           |                               |
|                       |           BC019704           |                               |
|                       |           BG032081           |                               |
|                       |           BG699269           |                               |
|                       |           AA334258           |                               |
|                       |           BG956088           |                               |
|                       |           CF146182           |                               |
|                       |           CF146495           |                               |
|                       |           CF146156           |                               |
|                       |           BU602750           |                               |
|                       |           BI763242           |                               |
|                       |           BU601649           |                               |
|                       |           BM718038           |                               |
|                       |             W92392           |                               |
|                       |             R08496           |                               |
|                       |           BU607794           |                               |
|                       |           BM923994           |                               |
|                       |           AK127177           |                               |
|                       |           BX109249           |                               |
|                       |             H67840           |                               |
|                       |           BM727187           |                               |
|                       |           CD370426           |                               |
|                       |           CD742895           |                               |
|                       |           BM693301           |                               |
|                       |           BX343263           |                               |
|                       |           BX403055           |                               |
|                       |           BX403056           |                               |
|                       |           CR606315           |                               |
|                       |           CR736754           |                               |
|                       |           AW137703           |                               |
|                       |           AI217148           |                               |
|                       |           CB242250           |                               |
|                       |           BQ185338           |                               |
|                       |           CA418978           |                               |
|                       |           CB851091           |                               |
|                       |           BI711150           |                               |
|                       |           BM681658           |                               |
|                       |             W56565           |                               |
|                       |           BM680490           |                               |
|                       |           BG218080           |                               |
|                       |           AI217725           |                               |
|                       |           AW380070           |                               |
|                       |           CK820460           |                               |
|                       |           AW905043           |                               |
|                       |           AI655972           |                               |
|                       |           CD250933           |                               |
|                       |           BF590059           |                               |
|                       |           AA523409           |                               |
|                       |           AA523391           |                               |
|                       |           AI809237           |                               |
|                       |           CD677519           |                               |
|                       |           BX343262           |                               |
|                       |           AI377032           |                               |
|                       |           AI872211           |                               |
|                       |           AI142666           |                               |
|                       |           BG231563           |                               |
|                       |           AI248517           |                               |
|                       |           AI381901           |                               |
|                       |           AW328508           |                               |
|                       |           BI710820           |                               |
|                       |           AI150552           |                               |
|                       |           AW769415           |                               |
|                       |           CR737126           |                               |
|                       |           AI241274           |                               |
|                       |           AW613735           |                               |
|                       |           BF509506           |                               |
|                       |             W92335           |                               |
|                       |           BX331455           |                               |
|                       |           BX331456           |                               |
|                       |           CR621786           |                               |
|                       |           AA609120           |                               |
|                       |           AA580479           |                               |
|                       |           AW194863           |                               |
|                       |             Z25384           |                               |
|                       |           AI912966           |                               |
|                       |           AW243314           |                               |
|                       |           BM682761           |                               |
|                       |           BM726305           |                               |
|                       |           BM719425           |                               |
|                       |           BQ183713           |                               |
|                       |           AL833556           |                               |
|                       |           BG196189           |                               |
|                       |           BG204383           |                               |
|                       |           BG182677           |                               |
|                       |           BG199237           |                               |
|                       |           CR593703           |                               |
|                       |           CA429441           |                               |
|                       |           AI953718           |                               |
|                       |           CA945120           |                               |
|                       |           BX489198           |                               |
|                       | *Seq. mapped in strand Minus*| *Seq. mapped in strand Minus* |
|                       |           BF362893           |          AI646616             |
+-----------------------+------------------------------+-------------------------------+
|      #  181           | *Seq. mapped in strand Plus* | *Seq. mapped in strand Plus*  |
|                       |           AI791595           |          BE988727             |
|                       |           BX095690           |                               |
|                       |           AI733099           |                               |
|                       |           AA902617           |                               |
|                       | *Seq. mapped in strand Minus*| *Seq. mapped in strand Minus* |
|                       |           AW451798           |          BI133747             |
|                       |           BU737631           |          CA321355             |
|                       |           CA748609           |          CO042057             |
|                       |           AW574536           |          BC053930             |
|                       |           BX115214           |          BQ899628             |
|                       |           BC039059           |          BF471923             |
|                       |           BC046180           |          CK635475             |
|                       |           BC012989           |          BY353709             |
|                       |           AK094089           |          CK335246             |
|                       |           AA278764           |          CA979033             |
|                       |           CA439424           |          CK332513             |
|                       |           AB040918           |          CF748958             |
|                       |           AB167740           |          CF749583             |
|                       |           BC070097           |          BQ832250             |
|                       |           AB167738           |          CF729322             |
|                       |           AB167739           |          BQ892127             |
|                       |           AL832066           |          CA327223             |
|                       |           AA809888           |          BU708741             |
|                       |           BX363590           |          BQ180631             |
|                       |           CR609716           |          CN666794             |
|                       |           BC025423           |          BY257018             |
|                       |           BE252691           |          BB653259             |
|                       |           BG474092           |                               |
|                       |           BE252356           |                               |
|                       |           BX363591           |                               |
|                       |           BQ070028           |                               |
|                       |           BE256208           |                               |
|                       |           BE074616           |                               |
|                       |           BQ935752           |                               |
|                       |           BG013005           |                               |
|                       |           BI862133           |                               |
|                       |           BG006882           |                               |
|                       |           BG006884           |                               |
|                       |           BG006876           |                               |
|                       |           BF809399           |                               |
|                       |           BQ944931           |                               |
|                       |           BG006888           |                               |
|                       |           BU542906           |                               |
|                       |           BG012612           |                               |
|                       |           BG012514           |                               |
|                       |           BI089601           |                               |
|                       |           BG012926           |                               |
|                       |           BG007266           |                               |
|                       |           BG014652           |                               |
|                       |           BG011110           |                               |
|                       |           BM796909           |                               |
|                       |           AL543479           |                               |
|                       |           BU196908           |                               |
|                       |           BU838137           |                               |
|                       |           BX397012           |                               |
|                       |           BE747464           |                               |
|                       |           BX400645           |                               |
|                       |           BG756925           |                               |
|                       |           AA278868           |                               |
|                       |           BQ361490           |                               |
|                       |           BM699892           |                               |
|                       |           BF771219           |                               |
|                       |           BM921816           |                               |
|                       |           BI092585           |                               |
|                       |           BG115028           |                               |
|                       |           AW401623           |                               |
|                       |           BG468749           |                               |
|                       |           BG469446           |                               |
|                       |           AB167741           |                               |
|                       |           CN429218           |                               |
|                       |           BU166933           |                               |
|                       |           BG768759           |                               |
|                       |           BX380499           |                               |
|                       |           CB993280           |                               |
|                       |           CB988209           |                               |
|                       |           BX646058           |                               |
|                       |           BX492766           |                               |
|                       |           BX492754           |                               |
|                       |           BX955023           |                               |
|                       |           BX646073           |                               |
|                       |           BE735035           |                               |
+-----------------------+------------------------------+-------------------------------+
|      #  182           | *Seq. mapped in strand Plus* | *Seq. mapped in strand Plus*  |
|                       |           AW298802           |          BF461085             |
|                       | *Seq. mapped in strand Minus*| *Seq. mapped in strand Minus* |
|                       |           AI189901           |          CO042008             |
|                       |           AA588758           |          AW825485             |
|                       |           CA424604           |          BC051539             |
|                       |           BQ448529           |          AK032829             |
|                       |           BM681356           |          CA879117             |
|                       |           BQ021241           |          BG077729             |
|                       |           CK299216           |          BG064390             |
|                       |           BQ020743           |                               |
|                       |           BU617420           |                               |
|                       |           CA313048           |                               |
|                       |           CA447966           |                               |
|                       |           BU742742           |                               |
|                       |           AI819280           |                               |
|                       |           AI186611           |                               |
|                       |           BF593030           |                               |
|                       |           BC013792           |                               |
|                       |           BC005101           |                               |
|                       |           BC020243           |                               |
|                       |           AK025581           |                               |
|                       |           BQ010301           |                               |
|                       |           BQ772656           |                               |
|                       |           AB037719           |                               |
|                       |           BC062341           |                               |
|                       |           AB072355           |                               |
|                       |           BG473376           |                               |
|                       |           BG472875           |                               |
|                       |           BE256978           |                               |
|                       |           BM908891           |                               |
|                       |           BG397630           |                               |
|                       |           BG758554           |                               |
|                       |           AB072356           |                               |
|                       |           AK095421           |                               |
|                       |           CR623842           |                               |
|                       |           BM047545           |                               |
|                       |           CN358049           |                               |
|                       |           BG741959           |                               |
|                       |           BG757943           |                               |
|                       |           BG686304           |                               |
|                       |           BG759105           |                               |
|                       |           AL121363           |                               |
|                       |           CK000114           |                               |
|                       |           BE244833           |                               |
|                       |           BX391546           |                               |
|                       |           BU195033           |                               |
|                       |           BM470627           |                               |
|                       |           BM560223           |                               |
|                       |           BG479594           |                               |
|                       |           BX354557           |                               |
|                       |           CN481327           |                               |
|                       |           BF950864           |                               |
|                       |           BX283273           |                               |
|                       |           BP222598           |                               |
|                       |           BP363810           |                               |
|                       |           BP278987           |                               |
|                       |           BQ900726           |                               |
|                       |           BE244945           |                               |
+-----------------------+------------------------------+-------------------------------+
|      #  183           | *Seq. mapped in strand Plus* | *Seq. mapped in strand Plus*  |
|                       |           AI290486           |          AK087276             |
|                       |           BM678290           |                               |
|                       |           AI039516           |                               |
|                       |           AA831650           |                               |
|                       | *Seq. mapped in strand Minus*| *Seq. mapped in strand Minus* |
|                       |           BM923340           |            U01135             |
|                       |           BG501711           |          BC053390             |
|                       |             H16656           |          AK002643             |
|                       |             H24232           |          AK028563             |
|                       |           BG106521           |          BC032929             |
|                       |           BI259679           |          AJ532593             |
|                       |           BM551247           |          AK051122             |
|                       |           AI800252           |                               |
|                       |           AI634050           |                               |
|                       |           BF594453           |                               |
|                       |           AW072321           |                               |
|                       |           AI081336           |                               |
|                       |           BQ647182           |                               |
|                       |           BF436803           |                               |
|                       |           BF222135           |                               |
|                       |           AA636036           |                               |
|                       |           AA180276           |                               |
|                       |           BM978772           |                               |
|                       |           CA443390           |                               |
|                       |           AI887829           |                               |
|                       |           AL562868           |                               |
|                       |             H38934           |                               |
|                       |           AA704153           |                               |
|                       |           AA114153           |                               |
|                       |           AW250185           |                               |
|                       |           AI273306           |                               |
|                       |           AA485982           |                               |
|                       |           BF593375           |                               |
|                       |           BE207556           |                               |
|                       |           BE301347           |                               |
|                       |           BC000335           |                               |
|                       |           AI696554           |                               |
|                       |             H18009           |                               |
|                       |           AW515880           |                               |
|                       |           AW249987           |                               |
|                       |           CN343662           |                               |
|                       |           AI798281           |                               |
|                       |           AA442083           |                               |
|                       |           AW572847           |                               |
|                       |           AA666298           |                               |
|                       |           AI347486           |                               |
|                       |           BI856221           |                               |
|                       |           CA437743           |                               |
|                       |           AA677672           |                               |
|                       |           AI589180           |                               |
|                       |           BF062758           |                               |
|                       |           AW467434           |                               |
|                       |           BU621198           |                               |
|                       |           BQ182096           |                               |
|                       |           BG686048           |                               |
|                       |           AA868783           |                               |
|                       |             Y08612           |                               |
|                       |           CN278172           |                               |
|                       |           AI719598           |                               |
|                       |           BG190846           |                               |
|                       |           AA167003           |                               |
|                       |           BG194807           |                               |
|                       |             R16304           |                               |
|                       |           BE622874           |                               |
|                       |           BU940824           |                               |
|                       |           AL525959           |                               |
|                       |           AL120841           |                               |
|                       |             F05600           |                               |
|                       |           BG759661           |                               |
|                       |           AA650020           |                               |
|                       |           BF220297           |                               |
|                       |           BF980198           |                               |
|                       |           AA102809           |                               |
|                       |           BE148088           |                               |
|                       |           BG219184           |                               |
|                       |           AW361982           |                               |
|                       |           BE706251           |                               |
|                       |           BE147910           |                               |
|                       |           AW378982           |                               |
|                       |           BF002843           |                               |
|                       |           BF223234           |                               |
|                       |           AI566728           |                               |
|                       |           BI493555           |                               |
|                       |           AA166725           |                               |
|                       |           BG686994           |                               |
|                       |           AI702442           |                               |
|                       |           BG398117           |                               |
|                       |           BG187429           |                               |
|                       |           BE245541           |                               |
|                       |           BG342097           |                               |
|                       |           BF336950           |                               |
|                       |           BF336951           |                               |
|                       |           BX349939           |                               |
|                       |           BG961073           |                               |
|                       |           BX349940           |                               |
|                       |           AA814846           |                               |
|                       |           BG193709           |                               |
|                       |           BF834068           |                               |
|                       |           BE813962           |                               |
|                       |           BG205550           |                               |
|                       |           BG198100           |                               |
|                       |           BG170446           |                               |
|                       |           BF840297           |                               |
|                       |           BE243358           |                               |
|                       |           CK002324           |                               |
|                       |           BQ953718           |                               |
|                       |           AA488609           |                               |
|                       |           BX283742           |                               |
|                       |           BG613917           |                               |
|                       |           BE719627           |                               |
|                       |           CB158519           |                               |
|                       |           AA442188           |                               |
|                       |           BG030562           |                               |
|                       |           AA749357           |                               |
|                       |           AA827198           |                               |
|                       |           AA731010           |                               |
|                       |           AW080542           |                               |
|                       |           AI088437           |                               |
|                       |           BG990567           |                               |
|                       |           BU608997           |                               |
|                       |           AI417569           |                               |
|                       |           AI436235           |                               |
|                       |           AA453480           |                               |
|                       |           BU539430           |                               |
|                       |           BP265895           |                               |
|                       |           BP266190           |                               |
|                       |           BU858009           |                               |
|                       |           BE089342           |                               |
|                       |           BE004610           |                               |
|                       |           CF122933           |                               |
|                       |           BX504755           |                               |
|                       |           BU570849           |                               |
|                       |           BE004563           |                               |
|                       |           AW804089           |                               |
|                       |           AW804121           |                               |
|                       |           BE814987           |                               |
|                       |           BM783250           |                               |
|                       |           AW881168           |                               |
|                       |           BE089357           |                               |
|                       |           BQ315096           |                               |
|                       |           BM831876           |                               |
|                       |           CF122955           |                               |
|                       |           BM145554           |                               |
|                       |           BM272699           |                               |
|                       |           AJ705801           |                               |
|                       |           AW969628           |                               |
|                       |           AA983323           |                               |
|                       |           BG109737           |                               |
|                       |             H18008           |                               |
|                       |           BE396953           |                               |
|                       |           BU558277           |                               |
|                       |           BP354655           |                               |
|                       |           BF768401           |                               |
|                       |           BG258303           |                               |
|                       |           BU857481           |                               |
|                       |           BE869539           |                               |
|                       |           CF139531           |                               |
|                       |           CD689916           |                               |
|                       |           BM011264           |                               |
|                       |           AA491017           |                               |
|                       |           BE154041           |                               |
|                       |             R08497           |                               |
|                       |           AW371965           |                               |
|                       |           BF330760           |                               |
|                       |           BE696204           |                               |
|                       |           AW856760           |                               |
|                       |           AW753893           |                               |
|                       |           BE155230           |                               |
|                       |           AW371979           |                               |
|                       |           AW371986           |                               |
|                       |           BE706772           |                               |
|                       |           AW371990           |                               |
|                       |           BF330759           |                               |
|                       |           BI055055           |                               |
|                       |           BF857989           |                               |
|                       |           AA312496           |                               |
|                       |           BE254121           |                               |
|                       |           AL526005           |                               |
|                       |           BG395584           |                               |
|                       |           BE302764           |                               |
|                       |           BU193560           |                               |
|                       |           BE696198           |                               |
|                       |           BM146688           |                               |
|                       |           BF237772           |                               |
|                       |           BE706599           |                               |
|                       |           BE706601           |                               |
|                       |           AW382855           |                               |
|                       |           BE384580           |                               |
|                       |           AW748980           |                               |
|                       |           BF350699           |                               |
|                       |           BG177007           |                               |
|                       |           BE153819           |                               |
|                       |           AW855851           |                               |
|                       |           BE927326           |                               |
|                       |           AW579939           |                               |
|                       |           AL527261           |                               |
|                       |           AW382839           |                               |
|                       |           BE153590           |                               |
|                       |           CD359338           |                               |
|                       |           BF334526           |                               |
|                       |           AA380443           |                               |
|                       |           BI755466           |                               |
|                       |           AW748964           |                               |
|                       |           BU526916           |                               |
|                       |           BE153869           |                               |
|                       |           BP265520           |                               |
|                       |           BF932334           |                               |
|                       |           AW579940           |                               |
|                       |           AA114136           |                               |
|                       |           BE706622           |                               |
|                       |           BE902870           |                               |
|                       |           BI461310           |                               |
|                       |           AW382795           |                               |
|                       |           CD557705           |                               |
|                       |           BI552947           |                               |
|                       |           BI819253           |                               |
|                       |           BI549458           |                               |
|                       |           BI598624           |                               |
|                       |           BE706615           |                               |
|                       |           AA102808           |                               |
|                       |           AI240699           |                               |
|                       |           BE869785           |                               |
|                       |           BE901738           |                               |
|                       |           BI550338           |                               |
|                       |           CB997327           |                               |
|                       |           BF990117           |                               |
|                       |           BI458058           |                               |
|                       |           BE902062           |                               |
|                       |           BI461277           |                               |
|                       |           BE901843           |                               |
|                       |           CB990577           |                               |
|                       |           BE901464           |                               |
|                       |           BE512925           |                               |
|                       |           BE264933           |                               |
|                       |           BG722421           |                               |
|                       |           BG718589           |                               |
|                       |           BI463264           |                               |
|                       |           BF334520           |                               |
|                       |           BF976895           |                               |
|                       |           BI459146           |                               |
|                       |           BG720209           |                               |
|                       |           BM272945           |                               |
|                       |           BE967188           |                               |
|                       |             W56134           |                               |
|                       |           BF979473           |                               |
|                       |           BI459101           |                               |
|                       |           AA725080           |                               |
|                       |           BG719270           |                               |
|                       |           BG335266           |                               |
|                       |           BG756715           |                               |
|                       |           BG481123           |                               |
|                       |           BG700894           |                               |
|                       |           BG723346           |                               |
|                       |           BE153818           |                               |
|                       |           BI560056           |                               |
|                       |           AA320162           |                               |
|                       |           BE280459           |                               |
|                       |           BM837935           |                               |
|                       |           BG724219           |                               |
|                       |           BE278326           |                               |
|                       |           AI692907           |                               |
|                       |           AA456249           |                               |
|                       |           AA772299           |                               |
|                       |           AA453481           |                               |
|                       |           BE902758           |                               |
|                       |           BI463891           |                               |
|                       |           BG717684           |                               |
|                       |             H46058           |                               |
|                       |           BI560112           |                               |
|                       |           AI220284           |                               |
|                       |           CD691763           |                               |
|                       |           CN343663           |                               |
|                       |           BU528874           |                               |
|                       |           AW243534           |                               |
|                       |           BM834425           |                               |
|                       |           BP272580           |                               |
|                       |           BP334700           |                               |
|                       |           BQ642360           |                               |
|                       |           BP347760           |                               |
|                       |           BP279845           |                               |
|                       |           BP289296           |                               |
|                       |           BP360013           |                               |
|                       |           BP371916           |                               |
|                       |           AI384089           |                               |
|                       |           BP314003           |                               |
|                       |           BP380268           |                               |
|                       |           BP226444           |                               |
|                       |           BP301127           |                               |
|                       |           BP254138           |                               |
|                       |           BI460784           |                               |
|                       |           AI419815           |                               |
|                       |           BF219747           |                               |
|                       |           AU280596           |                               |
|                       |           BP333360           |                               |
|                       |           AL704431           |                               |
|                       |           AA834598           |                               |
|                       |           AW611740           |                               |
|                       |           AW246983           |                               |
|                       |           AA194922           |                               |
|                       |           AA778592           |                               |
|                       |           AA838418           |                               |
|                       |           BP272101           |                               |
|                       |           AI263447           |                               |
|                       |           AI378503           |                               |
|                       |           AW082770           |                               |
|                       |           AI022888           |                               |
|                       |             N25904           |                               |
|                       |           AA885224           |                               |
|                       |           AA994884           |                               |
|                       |           BP258714           |                               |
|                       |           AI034324           |                               |
|                       |             H50498           |                               |
|                       |           AL710973           |                               |
|                       |           BG773929           |                               |
|                       |           AL698377           |                               |
|                       |           BP333464           |                               |
|                       |           AW297981           |                               |
|                       |           AI808252           |                               |
|                       |           BG489699           |                               |
|                       |           BG773629           |                               |
|                       |           BG717293           |                               |
|                       |           BP326872           |                               |
|                       |           AL702561           |                               |
|                       |           CD696462           |                               |
|                       |           AA741362           |                               |
|                       |           AA776503           |                               |
|                       |           BP871029           |                               |
+-----------------------+------------------------------+-------------------------------+
|      #  184           | *Seq. mapped in strand Plus* | *Seq. mapped in strand Plus*  |
|                       |           AK095485           |          BC046508             |
|                       |           AK127048           |                               |
|                       | *Seq. mapped in strand Minus*| *Seq. mapped in strand Minus* |
|                       |           AF083811           |          AF083812             |
|                       |           AK090959           |          AF083813             |
|                       |             U33822           |          BC010702             |
|                       |           BC018870           |          AK040246             |
|                       |           BC009964           |                               |
|                       |           AF123318           |                               |
|                       |           CR600439           |                               |
+-----------------------+------------------------------+-------------------------------+
|      #  185           | *Seq. mapped in strand Plus* | *Seq. mapped in strand Plus*  |
|                       |           BC029519           |          CD241773             |
|                       |           CR591492           |          CN832040             |
|                       |                              |          CB599677             |
|                       |                              |          BY320099             |
|                       |                              |          BY319333             |
|                       |                              |          AA511173             |
|                       |                              |          BU937194             |
|                       |                              |          AB049953             |
|                       |                              |          BB660306             |
|                       |                              |          AK028226             |
|                       |                              |          BY104582             |
|                       |                              |          BY175001             |
|                       |                              |          BY089421             |
|                       |                              |          BY025138             |
|                       |                              |          BY025551             |
|                       |                              |          BY027189             |
|                       |                              |          BY100099             |
|                       |                              |          BU519225             |
|                       |                              |          BY703701             |
|                       |                              |          AK003444             |
|                       |                              |          CA490453             |
|                       |                              |          CN836534             |
|                       |                              |          CB599583             |
|                       |                              |          BY712165             |
|                       |                              |          CB595907             |
|                       |                              |          BU842007             |
|                       |                              |          CN841867             |
|                       |                              |          BB848482             |
|                       |                              |          BB850247             |
|                       |                              |          AA277689             |
|                       |                              |          BY217496             |
|                       |                              |          BY013810             |
|                       |                              |          BY256271             |
|                       |                              |          CA321319             |
|                       |                              |          BC058959             |
|                       |                              |          CF550875             |
|                       |                              |          CF982090             |
|                       |                              |          CK130117             |
|                       |                              |          AW230209             |
|                       |                              |          CB194843             |
|                       |                              |          AI154717             |
|                       |                              |          BE197254             |
|                       |                              |          CK619365             |
|                       |                              |          CK021884             |
|                       |                              |          AA230733             |
|                       |                              |          AI159133             |
|                       |                              |          AA832708             |
|                       |                              |          BG079766             |
|                       |                              |          BG966245             |
|                       |                              |          BI414205             |
|                       |                              |          AW989886             |
|                       |                              |          BQ932813             |
|                       |                              |          BI904515             |
|                       |                              |          BG276241             |
|                       |                              |          BG147954             |
|                       |                              |          AA268256             |
|                       |                              |          BG148381             |
|                       |                              |          BU524978             |
|                       |                              |          AA623284             |
|                       |                              |          CD776408             |
|                       |                              |            W18847             |
|                       |                              |          AI225348             |
|                       |                              |          BI904089             |
|                       |                              |          BC025456             |
|                       |                              |          BI690169             |
|                       |                              |          BE954502             |
|                       |                              |          CB248655             |
|                       |                              |          AA473458             |
|                       |                              |          CB194366             |
|                       |                              |          AA116869             |
|                       |                              |          AA415899             |
|                       |                              |          BC041151             |
|                       |                              |          BG976362             |
|                       |                              |          CD776393             |
|                       |                              |          AW476003             |
|                       |                              |          AI877026             |
|                       |                              |          AU042995             |
|                       |                              |          CD741192             |
|                       |                              |          BG066679             |
|                       |                              |          BE308274             |
|                       |                              |          BU560812             |
|                       |                              |            C79712             |
|                       |                              |          BU700183             |
|                       |                              |          BY468445             |
|                       |                              |          AA415986             |
|                       |                              |          BY597460             |
|                       |                              |          BG149006             |
|                       |                              |          BB697836             |
|                       |                              |          AA396371             |
|                       |                              |          BY588607             |
|                       |                              |          BB834500             |
|                       |                              |          BB834126             |
|                       |                              |          BY520401             |
|                       |                              |          BY696533             |
|                       |                              |          BY700680             |
|                       |                              |          BF459248             |
|                       |                              |          AW060921             |
|                       | *Seq. mapped in strand Minus*| *Seq. mapped in strand Minus* |
|                       |           AK027195           |          AW490434             |
|                       |                              |          AU042692             |
|                       |                              |          AU042685             |
|                       |                              |          BB809126             |
|                       |                              |          AI842339             |
|                       |                              |          BI134852             |
|                       |                              |          BE948918             |
|                       |                              |          BU743660             |
|                       |                              |          BB797872             |
|                       |                              |          BE949457             |
|                       |                              |          BY755578             |
|                       |                              |          BC019423             |
|                       |                              |          BY586893             |
|                       |                              |          BY586906             |
|                       |                              |          AK006440             |
|                       |                              |          AA120726             |
|                       |                              |          BY733872             |
|                       |                              |          BG072440             |
|                       |                              |          BU936345             |
|                       |                              |          BY706489             |
|                       |                              |          BE860903             |
|                       |                              |          BG085258             |
|                       |                              |          CN833605             |
|                       |                              |          BU583863             |
|                       |                              |            W11713             |
|                       |                              |          BX529619             |
|                       |                              |          BB868833             |
|                       |                              |          CF539559             |
|                       |                              |          BY229451             |
|                       |                              |          BG870584             |
|                       |                              |          BY328023             |
|                       |                              |          BY351286             |
|                       |                              |          BY350986             |
|                       |                              |          BY349166             |
|                       |                              |          BY349792             |
|                       |                              |          BY349168             |
|                       |                              |          BY349382             |
|                       |                              |                               |
|                       |                              |                               |
|                       |                              |                               |
|                       |                              |                               |
|                       |                              |                               |
|                       |                              |                               |
|                       |                              |                               |
|                       |                              |                               |
|                       |                              |                               |
|                       |                              |                               |
|                       |                              |                               |
|                       |                              |                               |
|                       |                              |                               |
|                       |                              |                               |
|                       |                              |                               |
|                       |                              |                               |
|                       |                              |                               |
|                       |                              |                               |
|                       |                              |                               |
|                       |                              |                               |
|                       |                              |                               |
|                       |                              |                               |
|                       |                              |                               |
|                       |                              |                               |
|                       |                              |                               |
|                       |                              |                               |
|                       |                              |                               |
|                       |                              |                               |
|                       |                              |                               |
|                       |                              |                               |
|                       |                              |                               |
|                       |                              |                               |
|                       |                              |                               |
|                       |                              |                               |
|                       |                              |                               |
|                       |                              |                               |
|                       |                              |                               |
|                       |                              |                               |
|                       |                              |                               |
|                       |                              |                               |
|                       |                              |                               |
|                       |                              |                               |
|                       |                              |                               |
|                       |                              |                               |
|                       |                              |                               |
|                       |                              |                               |
|                       |                              |                               |
|                       |                              |                               |
|                       |                              |                               |
|                       |                              |                               |
|                       |                              |                               |
|                       |                              |                               |
|                       |                              |                               |
|                       |                              |                               |
|                       |                              |                               |
|                       |                              |                               |
|                       |                              |                               |
|                       |                              |                               |
|                       |                              |                               |
|                       |                              |                               |
+-----------------------+------------------------------+-------------------------------+
|      #  186           | *Seq. mapped in strand Plus* | *Seq. mapped in strand Plus*  |
|                       |           AU100220           |          BB802209             |
|                       |           BP380586           |          BB797268             |
|                       |           BP377701           |                               |
|                       |           CN368973           |                               |
|                       |           CN369026           |                               |
|                       |           BP286063           |                               |
|                       |           BP285518           |                               |
|                       |           BP286710           |                               |
|                       |           BP286781           |                               |
|                       |           AA256585           |                               |
|                       |           CN369027           |                               |
|                       |           BP288512           |                               |
|                       |           BP232661           |                               |
|                       |           BP287694           |                               |
|                       |           BP373939           |                               |
|                       |           BP288271           |                               |
|                       |           BP233027           |                               |
|                       |           BP288389           |                               |
|                       |           BP288423           |                               |
|                       |           BP285904           |                               |
|                       |           BC052575           |                               |
|                       |           AU280526           |                               |
|                       |           BP374090           |                               |
|                       |           BP285922           |                               |
|                       |           BP286824           |                               |
|                       |           BP288357           |                               |
|                       |           BP340941           |                               |
|                       |           BP351981           |                               |
|                       |           BP285299           |                               |
|                       |           BP287497           |                               |
|                       |           BP372229           |                               |
|                       |           BP374060           |                               |
|                       |           BP287396           |                               |
|                       |           BP288701           |                               |
|                       |           BP321173           |                               |
|                       |           BP288197           |                               |
[truncated: 5,931,765 more chars]
